# Exploring the diversity of promoter and 5′UTR sequences in ancestral, historic and modern wheat

**DOI:** 10.1111/pbi.13672

**Published:** 2021-09-16

**Authors:** Michael C.U. Hammond‐Kosack, Robert King, Kostya Kanyuka, Kim E. Hammond‐Kosack

**Affiliations:** ^1^ Department of Biointeractions and Crop Protection Rothamsted Research Harpenden UK; ^2^ Department of Computational and Analytical Sciences Rothamsted Research Harpenden UK

**Keywords:** promoter capture, *Triticum aestivum*, *Triticum monococcum*, Watkins landraces, agronomic traits, sequence variation, haplotypes, transposable elements (TE), repetitive elements (RE), transcription factor binding sites (TFBS)

## Abstract

A data set of promoter and 5′UTR sequences of homoeo‐alleles of 459 wheat genes that contribute to agriculturally important traits in 95 ancestral and commercial wheat cultivars is presented here. The high‐stringency myBaits technology used made individual capture of homoeo‐allele promoters possible, which is reported here for the first time. Promoters of most genes are remarkably conserved across the 83 hexaploid cultivars used with <7 haplotypes per promoter and 21% being identical to the reference Chinese Spring. InDels and many high‐confidence SNPs are located within predicted plant transcription factor binding sites, potentially changing gene expression. Most haplotypes found in the Watkins landraces and a few haplotypes found in *Triticum monococcum*, germplasms hitherto not thought to have been used in modern wheat breeding, are already found in many commercial hexaploid wheats. The full data set which is useful for genomic and gene function studies and wheat breeding is available at https://rrescloud.rothamsted.ac.uk/index.php/s/DMCFDu5iAGTl50u/authenticate.

## Introduction

Wheat provides about one fifth of the calories consumed by humans globally and contributes the greatest source of proteins to the human diet (FAOSTAT, 2017a,b). Therefore, a sustainable and resilient wheat crop that can meet the nutritional demands of the ever‐growing human population is essential for global food security. Plant breeders strive continually to improve varieties by manipulating genetically complex yield and end‐user quality traits while maintaining yield stability, improving nutrient use efficiencies and providing regional adaptation to specific abiotic and biotic stresses, for example, an ever‐increasing number of pathogen and pest threats (Atlin *et al*., 2017; Bonjean and Angus, 2001; Fisher *et al*., 2012).

A fully annotated, high‐quality sequence assembly of the large and complex hexaploid wheat genome (2n = 6x = 42; AABBDD), IWGSCrefseq_v1.0 was used (The IWGSC *et al*., 2018). The 14.5‐Gbp genome of the wheat landrace Chinese Spring (CS) contains nearly 270 000 genes, of which 107 891 were predicted with high‐confidence. Development of a gene expression atlas representing all stages of wheat development together with the accurate genome assembly has enabled the discovery of tissue‐ and developmental stage‐related gene co‐expression networks (The IWGSC *et al*., 2018) and an exploration of the relative expression levels of the homoeo‐alleles of each predicted gene on the A, B and D sub‐genomes (Allen *et al*., 2017; Arora *et al*., 2019; Ramírez‐González *et al*., 2018; Winfield *et al*., 2018).

Phenotypic variation of a trait is thought to occur due to variations of the coding DNA sequences (CDS) within the genes underlying the trait, as well as the environmental factors and gene‐by‐environment interactions. However, accumulating evidence suggests that mutations within regulatory regions may be equally important in generation of significant phenotypic differences (Li *et al*., 2012; Wray, 2007). Therefore, polymorphisms in sequences regulating gene expression may be important in shaping the natural trait variation in wheat and other plant species.

Here we investigated the variation in the sequences (spanning 5′UTRs and potential promoters and for simplicity hereafter referred to as ‘promoters’) located within 1700 nucleotides upstream of the CDS of 459 wheat genes, associated with agriculturally important traits, in ancestral, synthetic, historic and modern wheat genotypes (Allen *et al*., 2017; Winfield *et al*., 2018). The main practical objective was to determine whether the current target capture sequencing technology, which has so far been mostly used for analysing variation in exons and gene‐specific marker discovery (Arora *et al*., 2019), could also be used to effectively capture and sequence promoters of homoeologous wheat genes. The main scientific aims were to (i) compare the promoter variation (haplotypes) present in different wheat genotypes, and assess levels of polymorphism between wheat species with different ploidy levels, (ii) assess promoter sequence variation in ancestral wheat and commercial wheat cultivars, (iii) determine whether any of the identified polymorphisms may be located at recognized regulatory motifs (transcription factor binding sites, TFBS), (iv) determine whether large deletions are associated with insertion/deletion of repetitive elements and (v) explore whether ancient species may have already contributed to modern wheat breeding.

## Results

### Gene and germplasm selection

For this study, ten commercial traits for wheat improvement were selected and known or candidate genes underlying these traits were collated by dedicated trait coordinators (see Acknowledgements). 459 wheat genes of interest with a total of 1273 unique homoeo‐allele sequences were chosen for sequence capture and detailed analyses (Table 1 and Data S1). The distribution of the selected genes across the Chinese Spring (CS) chromosomes (IWGSC_refseq_v1.0) are shown in Figure S1. For the germplasm to be analysed, 69 historic and modern commercial hexaploid wheat (*Triticum aestivum*) cultivars including CS, 15 wheat landraces (*T. aestivum*) from the A. E. Watkins collection (Winfield et al., 2018; Wingen et al., 2014), eight *T*. *monococcum* (2n = 2x = 14; A^m^A^m^) accessions (Jing *et al*., 2007; Li *et al*., 2018; McMillan *et al*., 2014; Simons *et al*., 2021) and single accessions for *T. durum* (2n = 4x = 28; AABB), *Aegilops tauschii* (2n = 2x = 14; DD), *Ae. speltoides* (ASP) (2n = 2x = 14; SS) and the wild species *Ae. peregrina* (APG) (2n = 4x = 28; S^v^S^v^UU) (Table S1, Data S2) were chosen collaboratively by the UK wheat community (see Acknowledgments).

**Table 1:** The 10 trait categories, numbers of nominated and unique genes, total number of homoeologues and genetic composition of genes per trait

Trait	Category	Nominated Genes	Unique genes	Homoeologues	ABD	AB	AD	BD	A	B	D	Others*
T1	Yield Resilience	28	28	82	18	3	1					1[Un,BD], 2[B,AD], 1[ABD, Un], 1[A,AD] 1[ABDD]
T2	Grain Composition	59	59	154	40	4	2	5	1	2	3	1[BBD], 1[A,AD]
T3	Grain Development	44	19	52	11	2	1	3				1[AAB,A], 1[ A,AD]
T4	Biotic Stress (fungi & insects)	59	59	164	40	3	4	1		1		3[A,AD], 1[A,BD], 1[AB,D], 1[AABBD], 1[A,D], 1[A,B, Un], 1[A,B], 1[AB, Un]
T5	Abiotic Stress (drought, temperature)	30	30	81	20		1	2			4	1[A,B, Un], 1[B,D], 1[AABBDD]
T6	Nutrient Use Efficiency	69	67	199	49	1	3	3				1[A,D], 2[A,BD], 1[AABBD], 1[AAB], 1[AA,B, Un], 1[A,AAD], 1[D,ABD], 1[Un,BD], 1[ADD], 1[ABBD]
T7	Canopy Development/Plant Architecture	58	56	161	47	2	1				2	1[A,BD], 1[AD, Un], 1[AABD], 1[B, Un]
T8	Flower Biology	26	23	66	20		1	2				
T9	Root Architecture	76	72	200	55	3	7	3	1			1[A,BD], 1[Un,B], 1[B,B,D]
T10	Recombination	46	46	114	26	3	3	5	5	1		1[BD, Un], 1[D, Un], 1[AB,D]
Total		495	459	1273	326	21	24	24	7	4	9	44

\x90* These combinations depict situations where:
one of the homoeologues resides on an unassigned chromosome Chr Un (e.g. [Un, BD]), BLAST search found two genes with high identity either of which could be the true homoeologue (e.g. [ABDD]), while normally the 3 homoeologues would be expected to reside on the same chromosome group, that is Chr 7A, 7B and 7D, in some cases only two of the three homoeologues reside on the same chromosome group, for example Chr 7A and 7B, but the third homoeologue resides on Chr 4D (denoted as [AB,D]) homoeologues were only found in two of the sub‐genomes, but one of these sub‐genomes contains two homoeologues on different chromosome groups (e.g. [A, AD]) ‐ 3 genes from T4 (T4‐18, T4‐19 and T4‐20), involved in fructan synthesis serve to explain this combination of homoeologues: these genes are found in close proximity on chromosomes 7A, 7D and also 4A. Whereas the two chromosome group 7 homoeologues reside close to the telomere of the short arms, the chromosome 4A homoeologues of all 3 genes are still in close but inverted proximity and are located close to the telomere of the long arm of chromosome 4A. The reciprocal translocation T(4AL; 7BS) and the 4AL paracentric inversion are well documented for bread wheat (e.g. Dvorak *et al*., 2018).

### Analysis of the captured sequence data – homoeologue specificity

**Figure 1:**
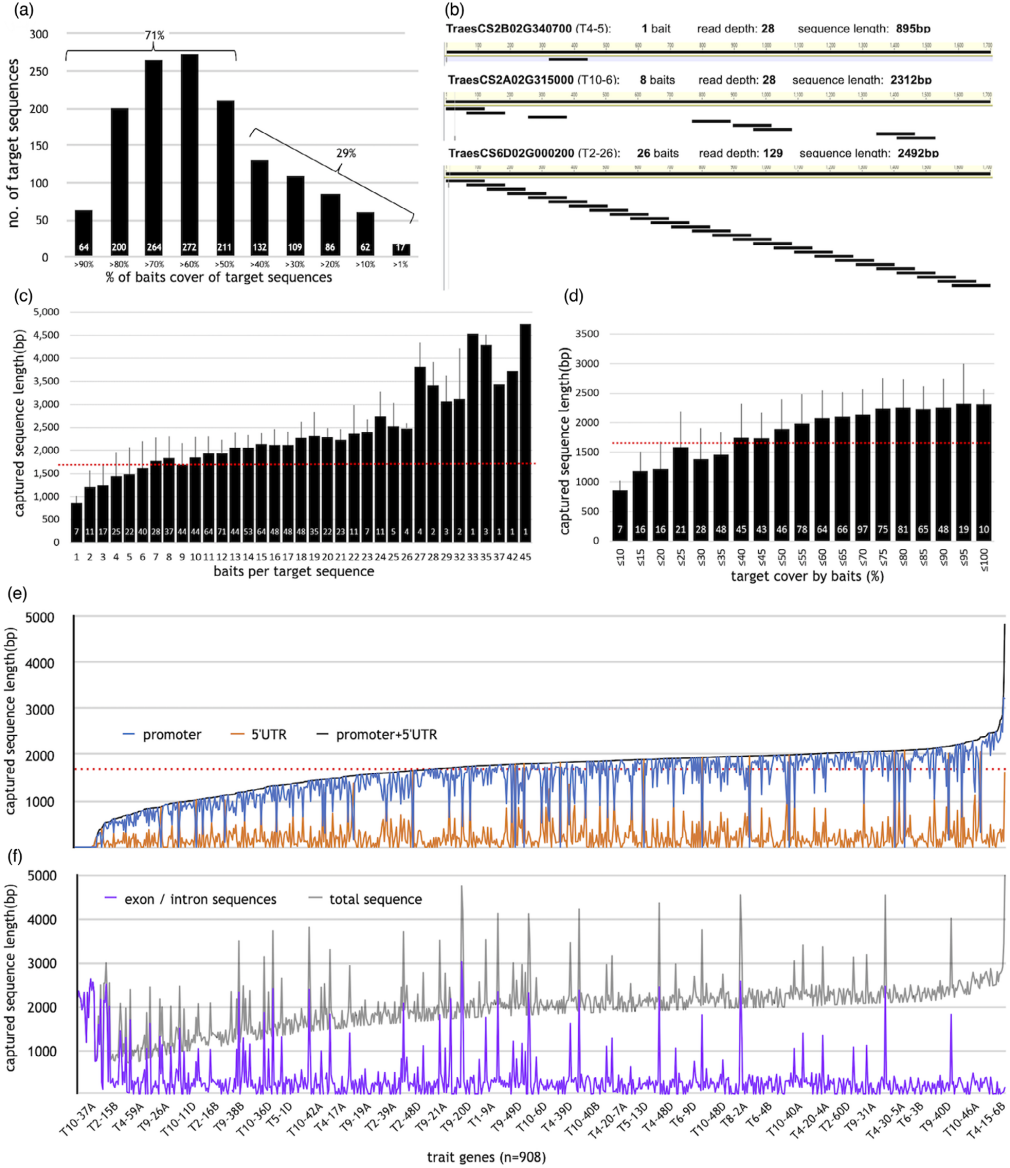
High‐specificity baits cover and sequence lengths obtained. (a) Percentage (%) of 1700 bp promoter (& 5′UTR) covered by high‐specificity baits (white numbers inside columns = number of genes with this %). (b) Three examples of lowest number of baits (1), medium (but evenly spaced) numbers (8) and highest number possible (26) and the resulting sequencing depths and lengths obtained. (c and d) Sequence length obtained in relation to numbers of baits per target sequence (c) and percentage of target sequence covered by baits (d). The white numbers show the numbers of genes. The desired target length of 1700 bp (red dotted line) was in many cases reached with just 4 baits and less than 25% baits cover of the target sequences, provided the baits were evenly spaced and not clustered. (e and f) Lengths of sequences captured for 908 trait genes. Genes are ordered by increasing size of combined promoter and 5′UTR length (black line), blue = promoter sequence, orange = 5′UTR. There are rare cases where the blue and orange line meet, only because the captured sequence lengths for 5′UTR and promoter are almost identical. Also, even rarer are genes with extremely long 5′UTR, that is only 5′UTR sequence was captured. In these cases, the orange line meets the black line and the blue line drops to zero (e). Additional sequence obtained for exon/intron sequences (purple) and total length of sequence captured for each gene (grey) (f). The x‐axis in (e and f) contains all 908 genes analysed but only a few tags can be shown for visibility's sake. (e and f) have been aligned, and hence, the labels are shown only in (f). All genes with total sequence above 3000 bp had either an enlarged target sequence and/or two sets of baits to cover alternate start sites (details in Data S1).

A myBaits (hereafter referred to as baits) capture technology developed by Daicel Arbor Biosciences was utilized to retrieve and sequence the specific promoter sequences of interest. To ensure the highly specific capture of promoters of individual homoeo‐alleles in wheat, a proprietary stringent workflow using RNA baits was chosen. In total, 17  745 unique baits were designed and manufactured to target 1700 bp of sequences located upstream of the annotated start codon of each of the 1273 homoeo‐alleles. For 71% of the promoters, there was >50% cover with highest stringency baits (Figure 1a). This extent of cover would be expected to allow capturing the entire target sequences, because the average length of DNA fragments prepared for capture by shearing genomic DNA was ˜500 bp. For the remainder, we decided to accept potentially less target sequence capture in order to allow high‐confidence mapping of captured sequences to the A, B and D homoeologues. The exact number of baits, their locations, sequences and percentage cover of the target sequences by baits are included in Data S1.

In total, 3.15 Mbp of genome aligned sequencing data (collapsed to 1× coverage) was generated from the captured CS sequences. Captured sequences for individual cultivars ranged from 1.46 Mbp (cv. Crusoe) to 9.81 Mbp (the diploid *T. monococcum* accession MDR308), except for Watkins 239 which for unknown reason(s) failed through the capture procedure. Total number of SNPs and InDels (≤20 bp) for each cultivar, ranging from 3536 – 242 384 SNPs and 381 – 15 116 InDels across the 95 accessions, are shown in Table S2. These numbers drop to ˜50% when filtering for homozygous polymorphisms. The homozygous polymorphism frequency for each cultivar was calculated, ranging from 0.6 kbp^−1^ for CS (which ideally should be zero; see below) to 15.1 kbp^−1^ for the tetraploid grass ASP. The slight variation in polymorphism frequency between individual cultivars is shown in Figure S2. Only the *T. monococcum* accessions (average 14.1 ± 0.9 kbp^−1^), ASP (15.1 kbp^−1^) and APG (12.0 kbp^−1^) have significantly higher polymorphism frequencies (which is confirmed by our visual analyses as described below) reflecting their distant relatedness/similarity to hexaploid wheat. The average frequency for hexaploid cultivars (including Watkins landraces) was found to be 1.9 ± 0.4 kbp^−1^, and only Sears Synthetic stands out with a ˜2x higher frequency of 4.7 kbp^−1^. However, this is again as expected due to the synthetic origin including foreign DNA introgression into this cultivar. These calculated values agree very well with our other analyses described below.

For the promoters of the 95 genotypes, for which sequencing data were obtained successfully, the maximum read depth (number of sequencing reads available for each nucleotide of the obtained sequence) ranged from 10‐ to 1115‐fold for the three diploid species, from 10‐ to 233‐fold for the two tetraploid species, and from 10‐ to 119‐fold for the hexaploid wheat CS (averages shown in Table 2, individual values for the analysed genes in Data S3), depending on the actual number of baits used for each promoter. The relationship between the number of baits per promoter and the overall sequence length and read depth obtained was analysed and this revealed that generally the capture and sequencing had been far more efficient than anticipated. Overall, the high efficiency of the RNA based baits capture technology is clearly demonstrated by the fact that the desired target length of 1700 bp is in many cases already achieved with only four baits providing less than 25% baits coverage of the target sequences, as long as the baits were evenly spaced and not clustered (Figure 1b–d). To illustrate this point, three examples for lowest, medium and highest baits cover are described. For the promoter of the gene TraesCS2B02G340700/ T4‐5 (Trait 4, gene 5) for which only a single high‐specificity bait could be designed, 895 bp of sequence with 28‐fold maximum read depth were obtained. For the promoter of gene TraesCS2A02G315000/ T10‐6 for which eight evenly spaced baits were available, a considerably longer sequence of 2312 bp (well in excess of the target length of 1700 bp) also with 28‐fold maximum read depth was obtained. For the promoter of gene TraesCS6D02G000200/ T2‐26) with overlapping baits covering 100% of the target sequence with 2‐fold bait coverage as in the original experimental design, the maximum read depth rose sharply to 129‐fold, while the overall sequence length obtained was similar to promoters represented by only 8–11 well‐spaced baits (Figure 1b).

**Table 2:** Average sequence lengths captured (a) and average sequencing depths separated by ploidy (b). (a) Average sequence lengths captured for the 908 fully analysed genes for wheat CS (Data S3). The additional retrieval of exon/intron sequences is an added benefit, resulting from baits close to the ATG start codon and/or additional downstream baits to cover alternate transcriptional start sites and thus substantially longer target sequences (details of individual bait positions in Data S1). (b) Maximum sequence depths were filtered before averaging (details in Data S3). The *n* numbers show how many genes were averaged for each cultivar. This includes all 908 analysed genes for CS (as all should be present and captured), but only varying numbers for the expected relevant sub‐genomes (as well as unexpected sub‐genome captures above the filter values) for the tetraploid and diploid species. [ratio] = diploid/tetraploid coverage depth divided by hexaploid (CS). Under ideal conditions, using the same amount of chromosomal DNA for all cultivars, the maximum theoretical coverage depth should be 3x higher for the diploid species and 1.5x higher for the tetraploids.

(a)	Promoter	5′UTR	Target sequence (promoter + 5′UTR)	Exons/Introns	Total sequence
Average Length (bp) (*n* = 908)	1416	235	1650	342	1993
±Stdev (bp)	575	327	536	496	568
±SEM (bp)	19	11	18	16	19

(b)	hexaploid	tetraploid		diploid		
cultivar	CS	KR	APG	M031	ASP	ENT
*n*	908	585	386	311	267	306
Average of maximum depth [ratio]	50 [1]	60 [1.2]	65 [1.3]	180 [3.6]	119 [2.4]	130 [2.6]
±Stdev	20	25	37	109	88	53
±SEM	0.65	1.03	1.88	6.17	5.39	3.03

For a subset of the trait gene homoeologues (*n* = 908), the total sequencing length obtained and the proportions of captured promoter and 5′UTR (the target sequence) as well as any exon and intron sequences were then determined. While the target sequence was usually 1700 bp, for 63 genes the target sequence was enlarged to take account of alternate transcriptional start sites. The total sequence lengths recovered from CS ranged from 629 bp for gene TraesCS3D02G113600/ T2‐14 (1 bait, 7.1% target coverage) to 4980 bp for TraesCS3D02G043500/ T2‐9 (19 baits, 90.1% target coverage), with a median value of 1993 ± 568 bp (Figure 1e,f, Table 2). Additionally, parts or complete first exon and first intron sequences were also captured for most genes in all cultivars. All data are included in Data S3.

One of the main aims of this study was to determine whether the baits capture technology could specifically capture promoters of the homoeologous A, B and D trait genes present in the allopolyploid wheat genome. Homoeologue‐specific capture of wheat promoters had not previously been reported. Among the cohort of 459 trait genes (1273 homoeologues), 326 genes had the complete homoeologue set (ABD), 69 genes had two homoeologues (AB, AD or BD) and 20 were singletons present only in one sub‐genome (Table 1). Another 44 genes had various other combinations of homoeologues, including 12 genes on ChrUn (the concatenated pseudo‐chromosome containing the unassigned genes and genomic sequences in the IWGSC refseq_v1.0).

**Figure 2:**
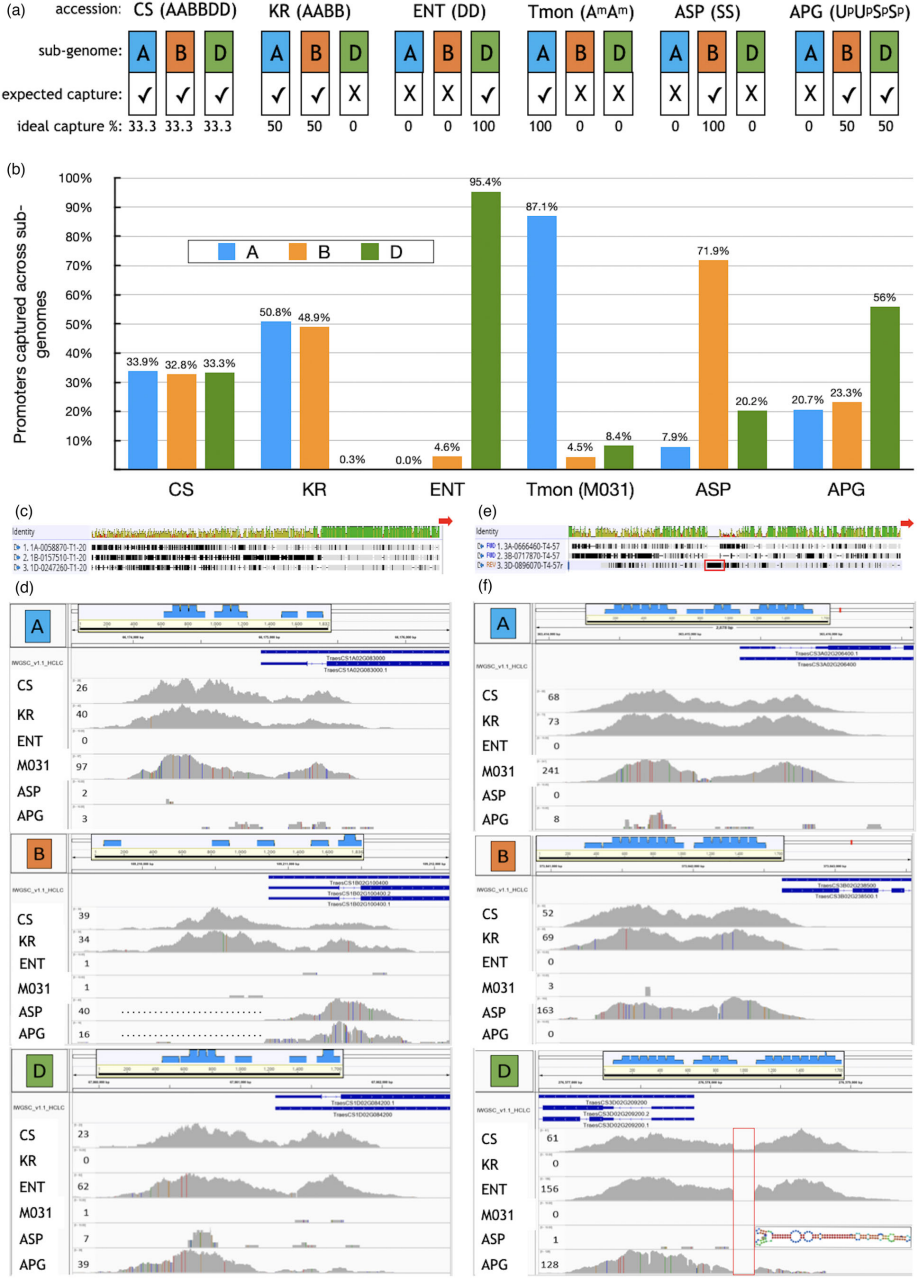
Homoeologue‐specific capture of promoters and 5′UTRs. (a and b) Expected (a) and observed (b) promoter capture for the three hexaploid wheat sub‐genomes (A, B, D). a, capture is homoeologue specific only if these coverage patterns are observed. However, *T. monococcum*, ASP and APG are only distally related to the wheat CS sub‐genomes, so a less strict specificity was expected. KR = *T. durum* cv. Kronos, ENT = *Ae. tauschii*, Tmon = *T. monococcum*, b, observed coverage patterns. vertical bars = percentage of homoeologues captured across the A, B and D sub‐genomes. Please note that only M031 is shown, but all 8 Tmon species showed the same distribution. For CS, the capture is extremely close to the ideal distribution, and capture was 100% successful for all analysed genes. For KR whose AABB genome has the highest similarity to CS, the distribution is very close to the ideal one, very close to 50% for both the A and B sub‐genome. For ENT, whose DD genome is very similar to CS, the vast majority of captured homoeologues map to the D sub‐genome, but it is interesting that 4.6% have been captured for the B sub‐genome suggesting some cross‐hybridization of B sub‐genome specific baits, whereas there is no cross‐hybridization for A sub‐genome specific baits. The other three species have reduced similarity to the CS genome and hence also the baits. But for both M031 (A^m^A^m^) and ASP (SS), which have A and B related genomes, the vast majority of captured homoeologues reside on the A or B sub‐genome, respectively. Only for APG (S^v^S^v^UU; B and D related genome), the result is unexpected. While the D sub‐genome has near 50%, both the A and B sub‐genomes have near 25% distribution of all captured homoeologues, suggesting that the reported S^v^ sub‐genome for APG has equal similarity to the A and B sub‐genomes of hexaploid wheat. (c and e) Identity between the three homoeologue promoters and 5′UTRs for two genes, T1‐20 (TraesCS1A02G083000, TraesCS1B02G100400, TraesCS1D02G084200) (c) and T4‐57 (TraesCS3A02G206400, TraesCS3B02G238500, TraesCS3D02G209200) (e) (green = all three homoeologues identical, yellow = two homoeologues, red = none), red arrow = ATG and gene orientation. (d and f) Coverage patterns observed for the A, B and D sub‐genomes of T1‐20 (d) and T4‐57 (f). Dark blue bars = location of the genes (thick = exons, thin = 5′UTR, thin line = introns), grey graphs = coverage (depth) – these graphs are NOT normalized, and hence, the numbers left of graphs show the maximum coverage depth, coloured lines within the coverage graphs = homozygous SNPs (allele frequency 1.0) compared to the reference sequence (IWGSCrefseq_v1.0). Boxed insets = location of target sequence (black bar) and number and position of baits (light blue bars, 120 nt each). The dotted lines inside the ASP and APG tracks in (e) show the lack of promoter sequence captured. Red box in (e) & (f) = 151 bp sequence – an insertion in the D homoeologue target sequence (e) which is partially absent in the CS used in this experiment (suggesting that CS is heterozygous for this MITE) or fully absent from ENT and APG as well as all hexaploid cultivars in this capture experiment (f). The inset in the D homoeologue capture shows the predicted hairpin structure.

To determine the extent of homoeologue‐specific sequence capture, captured data were compared from the included control species (described above). The data presented in Figure 2 indicate that homoeologue‐specific sequence capture was the predominant outcome. For CS, captured sequences mapped almost equally to the three sub‐genomes (33.9% (A), 32.8% (B) and 33.3% (D)). The very minor difference to the ideal ⅓ distribution reflects the fact that not all genes have homoeologue triplets (see Table 1). Homoeologue‐specific sequence capture can be determined by the absence of sequence capture for one (tetraploid species) or two (diploid species) of the three sub‐genomes. Baits that are specific for the A sub‐genome would be expected to mostly capture sequences from durum wheat cv. Kronos (AABB) and *T. monococcum* (A^m^A^m^) accessions but not from *Ae. tauschii* (DD), ASP or APG (Figure 2a), and this is exactly what was observed (Figure 2b). For cv. Kronos, 50.8% and 48.9% of all captured sequences map to the A and B sub‐genome, respectively, whereas only 0.3% mapped to the D sub‐genome, demonstrating the very low level of cross‐hybridization. Also, 95.4% of the *Ae. tauschii* sequences captured mapped to the D sub‐genome while the remainder mapped only to the B sub‐genome while zero cross‐hybridization with A sub‐genome sequences was observed. Similarly, for *T. monococcum*, 87.1% of captured sequences reside in the A sub‐genome, while 4.5% and 8.4% reside in the B and D sub‐genomes, respectively. This larger deviation from the ideal distribution was, however, not unexpected, because the A^m^ genome of *T. monococcum* is known to be closely related but not completely homologous to the A sub‐genome of hexaploid wheat, which originates from *T*. *urartu*, and the captured sequences consistently contained a large number of SNPs (as also indicated by the calculated polymorphism frequencies) which could contribute to cross‐hybridization (Table S2, Figure S2). It is interesting to note that despite the higher SNP frequency in *T. monococcum* promoters, the coverage depth observed was still on average ˜3x higher than for hexaploid wheat. This strongly suggests that the 120 nt length of the RNA baits and the strong DNA‐RNA hybridization employed overcome these mismatches. This is also true for the S genome of the diploid ASP where the majority of captured sequences map to the B sub‐genome (71.9%) with however more frequent capture for the A and D‐subgenome (7.9% and 20.2%, respectively) corresponding to reduced similarity to the CS genome (Figure 2a,b). It is also worth mentioning that frequently for this distantly related species (as well as APG) only parts of the CDS and 5′UTR were captured, with no capture for the predicted promoters as shown in Figure 2d for the B homoeologue of T1‐20 (TraesCS1B02G100400). This strongly suggests that the corresponding genes are present in these grass species, but that the promoter sequences are totally different from those in hexaploid wheat. Interestingly, for APG, the largest number of sequences mapped to the D sub‐genome which shows that the U sub‐genome of APG is more closely related to the wheat D sub‐genome. This is supported by the fact that the U genome originates from *Ae. umbellulata* which has been shown by phylogenetic analysis to be closely related to the D genome of *Ae. tauschii* (Petersen *et al*., 2006). However, the unanticipated almost equal capture of A and B homoeologues (20.7% and 23.3%) indicates that this ancient tetraploid species has a more complex origin than hitherto assumed, suggesting that the S^v^ genome of APG has near equal similarity to the A and B sub‐genomes of CS. Examples of sequences captured with the baits designed for the homoeo‐alleles of two CS genes, T1‐20 (TraesCS1A02G083000, TraesCS1B02G100400, TraesCS1D02G084200) and T4‐57 (TraesCS3A02G206400, TraesCS3B02G238500, TraesCS3D02G209200) are shown in Figure 2d,f for the homoeologue‐specificity control cultivars. All data regarding homoeologue‐specific capture are included in Data S3.

Alignments of promoter sequences (prior to the capture experiment) of the homoeologous genes in CS wheat in some cases clearly revealed insertions within one or more of the homoeologue promoters. For example, the alignment of the promoters of the three homoeo‐alleles of the gene T4‐57 revealed a 151 bp insertion in the promoter of the D sub‐genome located homoeologue (Figure 2e). This sequence is predicted to adopt a stable hairpin structure suggesting that it could be a miniature inverted‐repeat transposable element (MITE). This is further supported by the capture data (Figure 2f) which shows partial presence of this MITE in the D sub‐genome homoeologue of T4‐57 in CS, strongly suggesting that the CS used in this experiment is heterozygous for this potential MITE. It is even possible that this sequence was heterozygous in the IWGSC_refseq1.0. Alternatively, it is formally possible that the MITE was ‘caught in the act’ of excision in the single CS plant used for leaf sampling and DNA extraction. However, this sequence was fully absent in the D, S or U sub‐genomes in all other *Triticum* sp. and *Aegilops* sp. accessions included, strongly suggesting that this is a transposable element albeit with very limited mobility because this sequence was found in only 29 other locations in the CS genome, and on only 16 of the 21 chromosomes. However, the low copy number per se does not rule this sequence out as a MITE, because even single copy number MITEs have been reported in plants (Ye *et al*., 2016).

### Haplotype frequencies and evidence for ancestral introgression

To accelerate wheat improvement through breeding, haplotype mapping is frequently used for investigating genetic pedigrees and to identify blocks of linked alleles that are likely to be inherited together in genetic diversity panels as well as to identify genomic regions that contain novel sequence segments derived from other wheat genotypes and / or acquired through wider introgression breeding (Przewieslik‐Allen *et al*., 2021). Here, we analysed the homozygous SNPs in the promoters and 5′UTRs of 908 gene homoeologues (contributing to different traits) across the 95 *Triticum* sp. and *Aegilops* sp. genotypes.

The results from analysis of the promoter capture data include (i) the lengths and depths of captured sequences for promoters and CDSs (Data S3), (ii) the identification of shared and unique haplotypes among hexaploid cultivars (Data S4), (iii) shared haplotypes between diploid/ tetraploid and hexaploid cultivars (Data S5) and (iv) small and large InDels including identification of TEs and TFBSs (Data S6).

**Figure 3:**
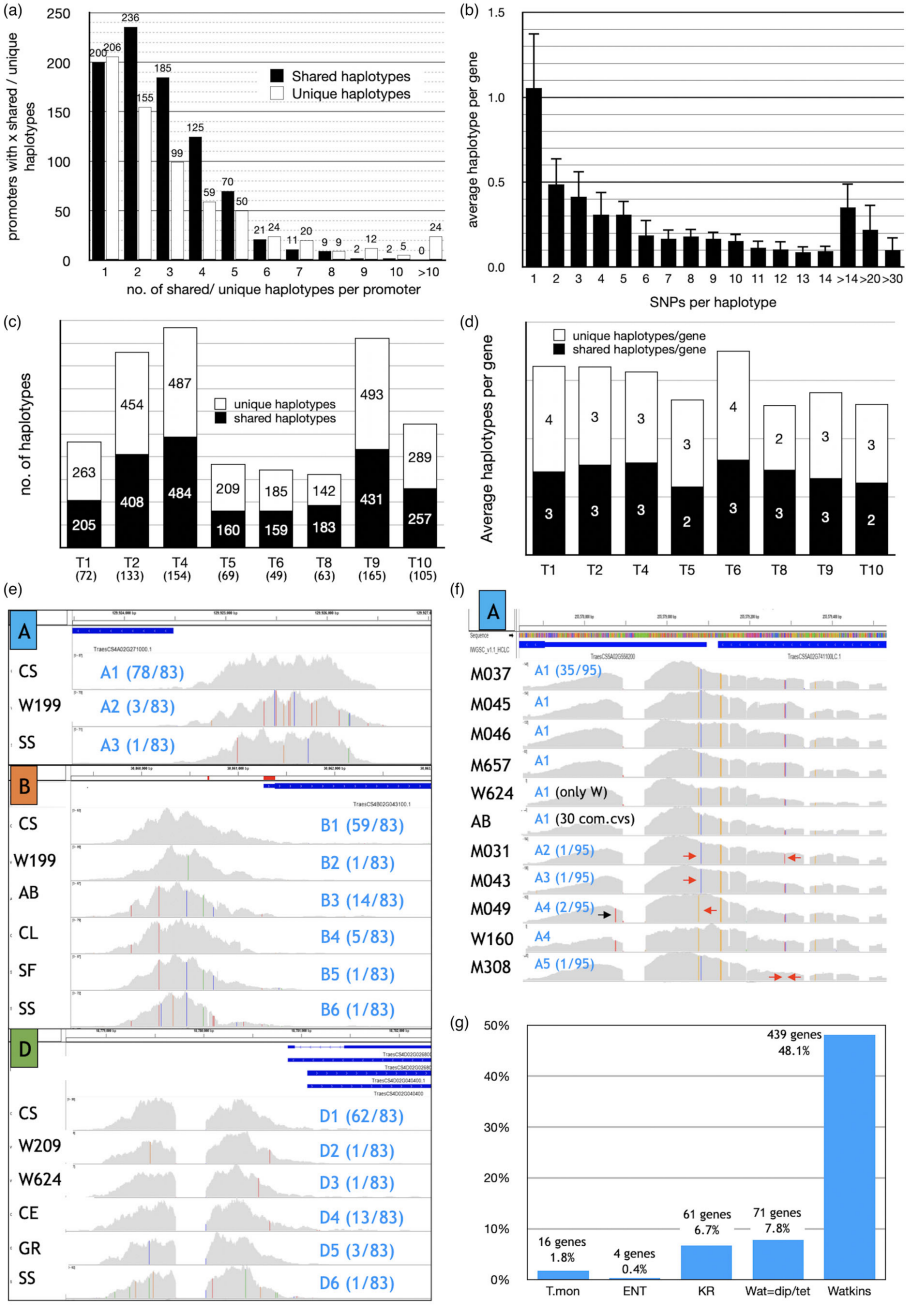
Haplotypes in hexaploid wheat cultivars and ancestral introgression. (a) Occurrence of x number of shared (black) and unique (white) haplotypes among all 83 hexaploid cultivars. A haplotype number of 1 indicates that for 200 genes all cultivars have just 1 shared haplotype, that is the same sequence as in CS for their promoters. This shows the very high number of promoters (200) with zero SNPs across all cultivars. Similarly, 206 genes have just 1 unique haplotype per promoter. Complete details for each gene are given in Data S4. (b) Haplotype diversity across all analysed traits. Total haplotypes per trait category with a specific number of SNPs (shown separately for 1–14 SNPs, combined from 15 SNPs upwards) were divided by the total genes within each trait category and averaged. The error bars reflect differences between traits. The graph shows that on average, every promoter had a haplotype with 1 SNP, and every other gene had a haplotype with 2 SNPs, etc. The average of 0.1 for haplotypes with 12 SNPs indicates that 1 in 10 genes contained this haplotype. (c and d) comparison of shared (black columns) and unique haplotypes (white columns) for each trait category. (c) Total numbers of shared *vs* unique haplotypes. The bracketed numbers indicate the numbers of genes for each category. (d) shared and unique haplotypes per gene. This allows direct comparison between the trait categories. (e) An example for the three homoeologues of *Rht1* (T9‐23: TraesCS4A02G271000, TraesCS4B02G043100, TraesCS4D02G040400). Representative cultivars for each haplotype observed are shown on the left with Watkins landraces indicated by W### and commercial cultivars by 2 letters (Table S1). Three haplotypes were observed for the A homoeologue and six haplotypes for both the B and D homoeologues, although only three of these are shared, the others being unique to the cultivars shown. The individual SNPs are indicated by coloured bars within the grey coverage graphs (blue = C, green = A, red = T, orange = G). The blue numbers indicate the name and frequency of each haplotype. The gap observed for all cultivars for T9‐23D is a long stretch of unidentified nucleotides in IWGSC_refseq_v1.0. (f) Coverage patterns and haplotypes for the A homoeologue of T5‐10 (TraesCS5A02G558200) on Chr 5A. Please note that haplotypes shown here are only the five observed in *T. monococcum* (Tmon). Haplotype A1 (M037) containing 6 SNPs and 6 InDels also occurred in three other Tmon varieties (M045, M046 and M657), one wheat landrace (W624) and 30 commercial wheat cultivars (AB, AM, BR, CH, CL, CO, CG, DI, EI, FL, GL, HF, HW, HU, IQ, KSA, KSI, MA, MH, ME, NA, RE, RV, RB, SA, SC, SP, SU and ZE) of which only one (AB) is shown. The arrows show the single additional SNP (black) and the few missing SNPs (red) in the other four Tmon accessions (M031, M043, M049 and M308) showing the close relatedness between the eight Tmon accessions included in this study. The observed gap is a deletion [AGCTGCTCGCGCGCACCCTCTTGCaagaagaagaagaagaagaagaa] found in CS, all Tmon, 5 Watkins landraces and 72 commercial wheat cultivars, but the sequence is present in KR, 9 Watkins lines and 10 cultivars (BW, CE, CP, IS, SS, SF, SO, TA, AP and UK). (g) Frequency of occurrence of diploid (Tmon and ENT), tetraploid (KR) and hexaploid Watkins landrace haplotypes shared by commercial cultivars in 908 analysed genes. Wat = dip(loid)/tet(raploid) indicates where any of the 14 Watkins lines share the same haplotype with Tmon (diploid), ENT (diploid) or KR (tetraploid) (see Data S5 for details).

The comparisons between the 83 hexaploid genotypes revealed only a small number of haplotypes (including both homozygous SNPs and InDels) for most of the 908 investigated promoter sequences. Haplotypes are grouped as ‘shared’ if at least two hexaploid cultivars show the same haplotype, the rest are referred to as ‘unique’ (singletons) within this set of cultivars (see Figure S3 for an example). These data are summarized for each analysed gene in Data S4 (columns D & E). In total, 52% of promoters had only 1 to 2 shared haplotypes of which 22% were identical to CS, while only 3.5% had 6 or more shared haplotypes across all trait genes (Figure 3a). The high identity with CS is however not overly surprising because pedigree analysis revealed that 32 of the commercial cultivars investigated here have CS as a (very) distant ancestor (Table S1, Figure S4b,c). Alternatively, this may just illustrate the relatively low sequence polymorphism in wheat and the relatively narrow selection of commercial cultivars included in this analysis, because this study focussed on cultivars grown in the UK. The haplotype diversity analysis (Figure 3b) for all homozygous SNPs shows that most haplotypes include only a small number of SNPs. On average, across the eight analysed traits, every promoter contains a haplotype with 1 SNP (average = 1.06), 50% of promoters contains a haplotype with 2 SNPs (average = 0.49), while haplotypes with for example 14 SNPs occur only in every 10th promoter (average = 0.095). Haplotypes with >14 SNPs are present but rare. As the average target sequence length captured was 1650 bp (Table 2a), 14 SNPs would only equate to 1 SNP every 118 bp, which clearly emphasizes the low number of SNPs in these promoter sequences. These results agree well with the SNP frequencies calculated from the homozygous polymorphisms per cultivar (Table S2, Figure S2). However, SNPs mostly clustered in a few regions of the promoter and were generally not evenly distributed. Regarding shared and unique haplotypes, individual traits differed only slightly from the overall pattern (Figure 3c,d) and this is also true for SNP diversity (Figure 3b). Surprisingly, the biggest difference between trait categories appears to be their chromosome distribution (Figure S1) rather than any differences in polymorphism frequency. For most promoters analysed, not only are many of the shared haplotype groups clearly related to mostly identical SNPs/InDels and only a few missing and/or additional SNPs, but this is also the case for a lot of the haplotypes called unique (Figure 3e,f, Figure S3). Overall, Sears Synthetic (SS) had by far the most unique haplotypes (625, 69% of genes) for the 908 analysed genes with examples included for *Rht1* (T9‐23) where haplotypes A3 (TraesCS4A02G271000), B6 (TraesCS4B02G043100) and D6 (TraesCS4D02G040400) are unique to SS (Figure 3e). Whereas for 200 promoters (22% of analysed genes) their sequence is identical to CS while the remainder is shared with other cultivars.

**Figure 4:**
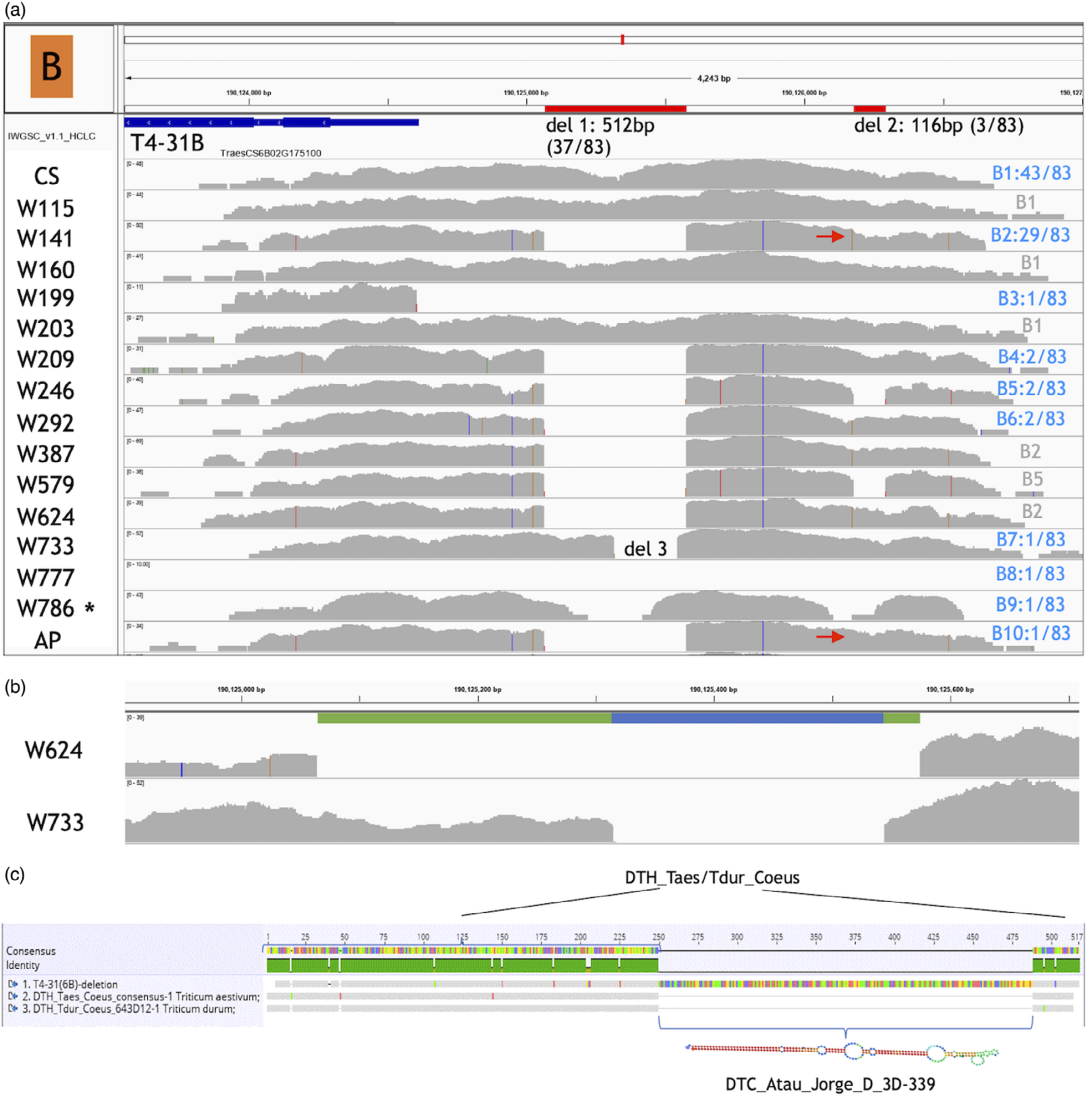
Large deletion found in the promoters of the B homoeologue of a WRKY transcription factor gene TraesCS6B02G175100/ T4‐31B. (a) Haplotypes, including deletions, observed in promoter capture data for the B homoeologue of this gene. Homoeologue haplotypes are notated as in Figure 3. Although there are 10 haplotypes, the occurrence of all but B1 and B2 is very rare or unique. Note two deletions (red horizontal bars): del1 is large and occurs in 37/83 cultivars while del2 is considerably smaller and occurs in only two landraces (W246 & W579), the synthetic wheat Sears Synthetic and the tetraploid cv. Kronos (KR) (data not shown) but in none of the commercial hexaploid cultivars. All the Watkins landraces included in this study are shown here, and while haplotype B2 occurs in 3 landraces and 26 commercial cultivars, the other Watkins haplotypes are either unique or shared with just one commercial cultivar. Del1 occurs in the diploid ASP, tetraploid APG and KR, the Watkins landraces W141, W209, W246, W292, W387, W579, W624 and commercial cultivars AB, AM, AV, BW, BR, BU, CE, CH, DI, FL, GT, GL, IS, IQ, KSI, KSL, MW, ME, PA, PI, RL, SS, SF, SO, TA, UK, AP, VA, VE, YU. *Note: W786 consistently had a slightly different coverage depth pattern (grey areas) to most other accessions for most analysed genes and this is not unique to the gene shown here. (b) Enlarged view of W624 with the complete del1 and W733 with only a partial del1 (del3). The ‘blue in green’ bars indicate two transposable elements (described in (c)). (c) Sequence alignment shows that del1 is a chimera consisting of known transposable elements Taes_Coeus with Atau_Jorge (from *Ae*. *tauschii*) integrated within the 3′ part of Taes_Coeus. The predicted stable hairpin secondary structure of the Atau_Jorge sequence is shown confirming this as a MITE. Note that the sequence alignment is exactly reflected in the W733 coverage pattern (haplotype B7).

Haplotypes observed in the Watkins landraces were also often present in several commercial hexaploid cultivars, but additionally some landraces exhibited unique haplotypes not observed in any of the commercial cultivars (details in Data S4). Both scenarios are illustrated here for the semi‐dwarfing gene *Rht1* (Hedden, 2003) (Figure 3e). For the A homoeologue of *Rht1*, the haplotype A2 (16 SNPs) found in the Watkins landrace W199 was also present in two commercial cultivars, Bobwhite and Apogee, while haplotypes B2, D2 and D3 were unique to individual Watkins landraces W199, W209 and W624, respectively. Interestingly, for most analysed genes the different haplotypes found in Watkins landraces are clearly related with a core of identical SNPs plus/ minus a few others (e.g. for the gene TraesCS6B02G175100/ T4‐31B, Figure 4a; Figure S3). Many haplotypes found in cultivars (e.g. *Rht1* haplotypes A3, B3‐B6 and D4‐D6) were not present in the Watkins landraces (for details see Data S4). Overall, 48% of analysed promoters have at least one haplotype shared between landraces and vastly differing numbers of commercial cultivars ranging from just 1 to over 60 (Figure 3g). This can clearly be discerned for every gene in Data S4 by the identical colour coding (identical haplotypes) of individual Watkins and commercial wheats and emphasizes that most commercial cultivars historically originate from landraces (Bonjean and Angus, 2001).

Our haplotype analysis also includes (i) identity with the CS IWGSC_refseq_v1.0 genome (0 SNPs) as a haplotype, as well as (ii) missing genes where neither promoter nor CDS sequences were captured from individual cultivars. Details of which cultivars have which gene missing are included in Data S4. The cultivar Hobbit has by far the greatest number of missing genes (45 genes). In total, for all cultivars, 59 genes are missing from only a single cultivar of which 34 are only absent from cv. Hobbit. Incidences where a large number of cultivars (ranging from 33 to 72) have a gene missing are only observed for single genes (Figure S5a).

Of the 45 missing genes in cv. Hobbit, 34 genes reside on chromosome arm 7BS in the CS genome. In fact, these 34 genes comprise all genes included in this project residing on 7BS and these are spread evenly across the entire chromosome arm, while all genes residing on 7BL are also present in cv. Hobbit (Figure S5b). This strongly suggests that the short arm of chromosome 7 is missing or has been substituted in the seed stock of cv. Hobbit acquired for this study. Another, albeit considerably smaller cluster of 6 missing genes in cv. Hobbit resides on 5BS, and again these are all the genes from 5BS included in this project, suggesting a very similar scenario for 5BS as for 7BS. These data strongly suggest the complete loss of 7BS and 5BS in this Hobbit line. Previously, a 5BS‐7BS translocation line has been reported for Hobbit sib (Arraiano *et al*., 2007). The translocation results in a very small fused chromosome consisting of 5BS‐7BS and a very large fused chromosome consisting of 5BL‐7BL. Our data suggest that cv. Hobbit used here is nullisomic for the fused chromosome 5BS‐7BS while retaining 5BL‐7BL. The same translocation has been reported for several other wheat cultivars, including ArinaLrFor and SY Mattis (Walkowiak *et al*., 2020) and Berseem, Cappelle‐Desprez, Vilmorin 27 and Carbo (Law, 1982).

By exploring the haplotypes further, evidence was also found for potential ancestral introgression events from *T. monococcum*, *Ae*. *tauschii* and *T*. *durum* (1.8%, 0.8% and 7%, respectively, of all analysed genes) based on the presence of identical haplotypes in these species and hexaploid cultivars (Figure 3). *T. monococcum* is of particular interest, because most accessions of this species harbour resistance to many agriculturally important pathogens and pests (Jing *et al*., 2007). *T. durum* introgressions, with significantly higher frequencies, are more likely ancestral and probably originating from emmer wheat (*T*. *turgidum* ssp. *dicoccoides*, AABB) (Maccaferri *et al*., 2015; Peng *et al*., 2011). An example of potential *T. monococcum* introgression is shown in Figure 3f for the A homoeologue of an abiotic stress gene TraesCS5A02G558200/ T5‐10. The exact haplotype A1 with 6 SNPs and 6 InDels as found in M037 (as well as M045, M046 and M657) was also present in only one of the Watkins landraces (W624) but intriguingly in 30 commercial cultivars. While this at first glance appears to be an unusually high occurrence of any potential ancestral introgression from diploid species, the fact that the M037 haplotype A1 is shared with the Watkins landrace W624 suggests that the original introgression occurred in the wild between *T. monococcum* and *T. aestivum* landraces or more likely via the tetraploid *T*. *timopheevii* (A^m^A^m^GG) and subsequently entered into commercial cultivars. Furthermore, among the 30 commercial cultivars sharing this haplotype, it is noteworthy that 27 of these are related by pedigree and only 3 cultivars show no relationship to any of the other 27 (Figure S4a). Interestingly, the other *T. monococcum* haplotypes (A2–A5) can be distinguished from A1 only by the presence/absence of just 1 or 2 SNPs (Figure 3f), yet another example of the overarching high similarity of individual haplotypes in wheat gene promoters. In total, for 16 promoters, identical haplotypes were found in *T. monococcum* and *T. aestivum* cultivars. These genes are not randomly distributed throughout the CS genome, instead twelve of these genes cluster in just three locations in the A sub‐genome on 5AL (2 genes), 6AS (5 genes) and 7AS (5 genes), in all three cases very close to the telomeric end of these chromosome arms. Foreign introgression events are more likely to have occurred towards the telomeres (Przewieslik‐Allen *et al*., 2021; Ribeiro‐Carvalho *et al*., 1997). While the occurrence of these *T. monococcum* haplotypes varies considerably in hexaploid cultivars, it is noteworthy that those found in the promoters of three fructan biosynthesis genes on 7AS are shared by the exact same group of 35 cultivars (Figure S6). However, of the 23 cultivars available for introgression analysis in the CerealsDB Putative Introgression Plotter (https://www.cerealsdb.uk.net/), only 12 showed evidence for ancestral introgression from *T*. *urartu*, *T*. *timopheevii* and/or *T*. *macha* whose A genomes are related to *T*. *monococcum*. Detailed description of all homoeologues with potential introgression events can be found in Data S5. This also emphasizes that this data resource could be used for rapid germplasm development if and when traits of interest are found in wild relatives/ancestral progenitor species.

CS itself showed 133 homoeologue target sequences out of 908 analysed (15%) where unexpectedly SNPs occurred compared to the IWGSC refseq_v1.0 genome assembly. However, 21% of these genes only have a single SNP in the promoter while 62% of promoters contained less than 5 SNPs across the whole target sequences and haplotypes with more than 10 SNPs were rare (Data S4 ‘CS SNPs’, Figure S7). In total, 814 SNPs were found in 133 promoters, but across all analysed promoters (*n* = 908) this only equates to 0.9 SNPs per promoter (polymorphism frequency of 0.6 kbp^−1^) which matches completely with the calculated homozygous polymorphism frequency of 0.6 kbp^−1^ (Table S2). This confirms the notion that there are more than one genetically slightly different CS accessions circulating among the wheat genetic community, probably as a result of different selection from the same Sichuan landrace. Interestingly, for some of these homoeologues, where CS SNPs were found, several Watkins landraces and commercial cultivars had no SNPs and thus were identical to the sequences in IWGSC CS_refseq_v1.0 (Data S4).

### The detection of homoeologue‐specific transposable elements, MITEs and other types of repeat sequences

The large wheat genome harbours a very high percentage of transposable elements (TEs), miniature inverted‐repeat transposable elements (MITEs) and other types of repeated sequences (The IWGSC *et al*., 2018). The capture data were explored visually in the Integrative Genomics Viewer (IGV) (https://software.broadinstitute.org/software/igv/) for evidence of homoeologue‐specific sequences of these types, by identifying cliff‐edge gaps in the sequence coverage. All deletions observed in various cultivars are listed in Data S6. A total of 326 small (<100 bp) and 257 large InDels were found across the 95 cultivars for the 908 analysed target sequences, typically just present in a single homoeologue promoter for each gene. Most small deletions either mapped only to their expected genome location (1 hit) or occasionally also to one or both of the corresponding homoeologues (2‐3 hits). All of the larger insertions/deletions (>100 bp) with increased BLAST hits (19 to >8800) mapped to the Wheat Transposon database and also to the CLARITE_CLARIrepeatwheat database. Surprisingly, of the larger insertions, 72 either only map to the promoter where first observed or also to the homoeologue promoters. These analyses can be viewed in Data S6.

For biotic stress (trait 4) genes, all 17 large deletions (compared to IWGSC_refseq_v1.0) were identified as (part of) named TEs (Figure S8). Five of these known TEs are only absent in a single cultivar, while the other 11 TEs are absent from several cultivars, ranging from 8 to 83, one even being absent from the CS stock used in this study. Some TEs were also absent from individual Watkins landraces, showing evidence for both historic and more recent excision of these TEs (Table S3).

Details of the promoter capture of the WRKY transcription factor gene TraesCS6B02G175100/ T4‐31B are shown in Figure 4. While for CS the whole target sequence was captured as expected, two deletions are apparent in many cultivars. Deletion 1 (del1, 512 bp) was identified in 7 landraces and 30 commercial hexaploid wheat cultivars (Figure 4a). The much smaller deletion 2 (del2, 116 bp) was found only in the two Watkins landraces W246 and W579 as well as the synthetic wheat Sears Synthetic and *T. durum* cv. Kronos, but not in any commercial hexaploid wheat cultivars. Accession W733 shows a unique pattern, in that it contains a smaller deletion (del3, 228 bp) within the region spanned by del1 (haplotype B7) (Figure 4b). Subsequent analysis of the CS sequences corresponding to regions spanned by del1 and del3 identified two recognized and named TEs, with an intact copy of the DTC_Atau_Jorge_D _3D‐339 element (del3) inserted inside the DTH_Taes/Tdur_Coeus element (Figure 4c). This shows that both TEs are potentially independently mobile, although independent excision of DTC_Atau_Jorge was only observed once in this dataset in W733 (Figure 4a). We did not observe any cultivars where DTC_Atau_Jorge remained inside this promoter, while DTH_Taes/Tdur_Coeus excised independently. However, this is not surprising because the 3′ end of Coeus resides downstream of Jorge, and therefore, whenever Coeus wants to travel, Jorge would be a (possibly unwilling) passenger. BLAST analysis revealed that even though the sequence corresponding to del1 maps to 8799 locations across all wheat chromosomes, there was only 1 full length hit for del1, inside the T4‐31B promoter. The remainder of the BLAST hits either mapped only to full or partial del3 sequences (*n* = 102 full length) or to the full or partial sequence in del1 upstream of del3 (*n* = 187 full length) in the T4‐31B promoter and elsewhere in the genome, reinforcing the chimeric nature of the del1 sequence. The sequence corresponding to del2 only maps to the three homoeologues of this gene. Most haplotypes found in Watkins landraces share many identical SNPs with just one or two additional or missing ones, but this is also true for the unique haplotype B10 for USU‐Apogee (AP) which has only one missing SNP compared to the haplotype B2 in Watkins landrace W141 (red arrow). The complete absence of captured sequence for W777 shows that this gene is missing in this Watkins landrace (haplotype B8) while the unique absence of promoter sequence in W199 (haplotype B3) suggests either a long deletion or complete replacement with a different sequence, most likely another transposable element.

### SNPs and InDels that remove or add potential transcription factor binding sites

**Figure 5:**
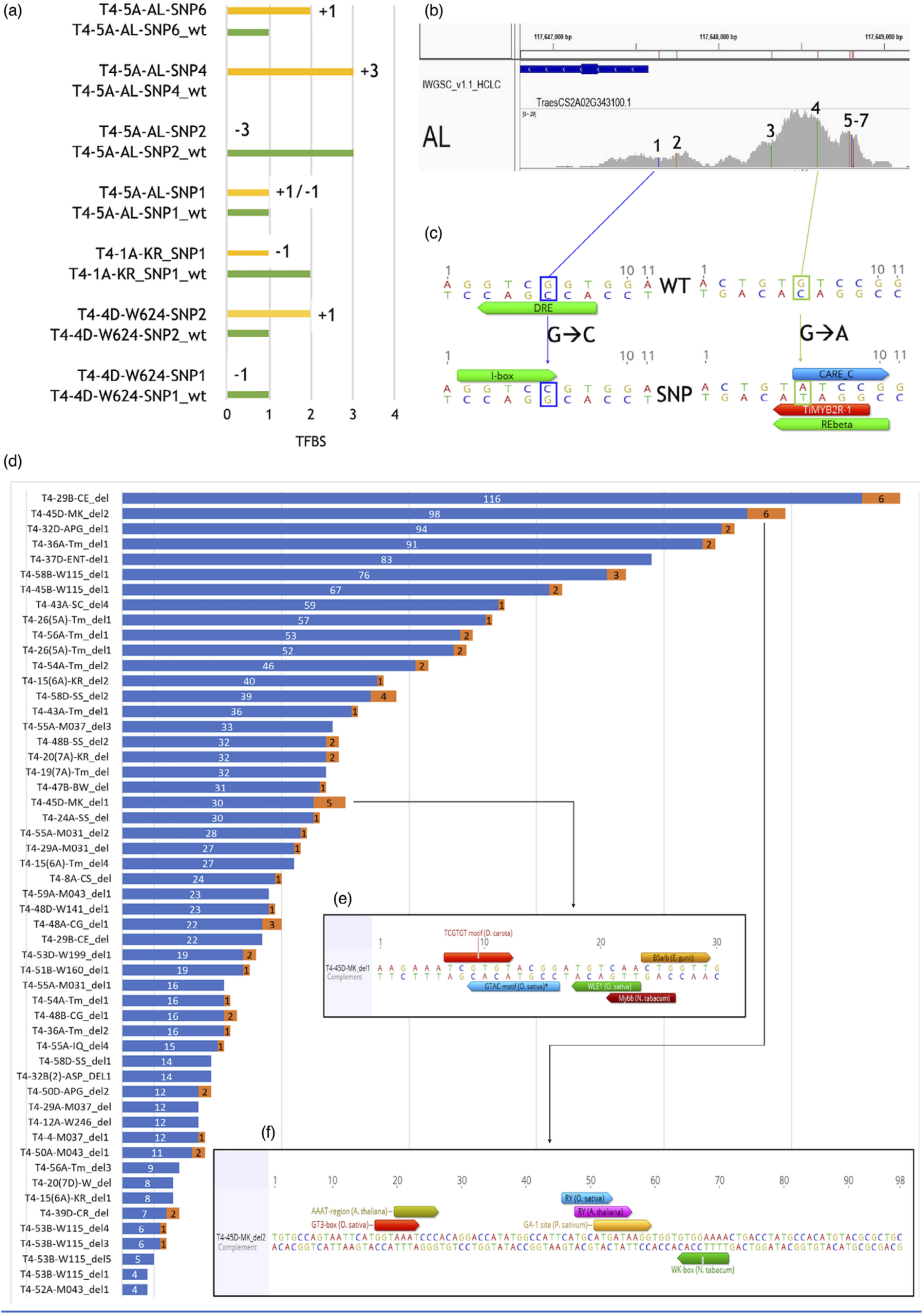
Loss or gain of Transcription Factor Binding Sites (TFBS) caused by individual SNPs and small deletions in all biotic stress gene promoters. (a) Shown are seven examples of TFBS in three Trait 4 (Biotic Stress) gene promoters (T4‐5A (TraesCS2A02G343100), T4‐1A (TraesCS7A02G264400) and T4‐4D (TraesCS3D02G049300)) across single SNPs in two commercial cultivars (AL = Alcedo, KR = Kronos) and 1 landrace from the Watkins collection (W624). Sequences were selected ±5 bp around the SNP position, and each 11 bp fragment was analysed for TFBSs without (WT) (green bars) and with the SNP (yellow bars). The numbers next to the yellow bars indicate the potential gain or loss of TFBS compared to WT. The number of TFBS found was filtered to include only TFBS with 100% match and without species duplications. (b) For the gene T4‐5A, the positions of the seven cv. Alcedo SNPs are shown. This exact SNP pattern also occurs in one landrace, W246, and 18 commercial cultivars (BR, BU, CL, CG, CR, EI, HF, IS, IQ, JB, KSA, MH, OA, RE, RV, RO, SC and ST (Data S4)). (c) Details of TFBS found across two of the T4‐5A cv. Alcedo SNPs (SNP1 (blue) & SNP4 (green)). For T4‐5A‐AL‐SNP1, the mutation results in the loss of the DRE binding site (Binding Factor TaDREB2, *T. durum*) but a gain of an I‐box motif, whereas for SNP4 there are no recognized plant TFBS in the WT sequence but the mutation results in three potential TFBS, including one from *T. aestivum* (TiMYB2R‐1). (d) Summary of all small deletions observed in any of the promoter sequences of the 171 Trait 4 genes. All deletions are labelled as follows: [trait category (T4)]‐[gene number and homoeologue (e.g. 45D)]‐[cultivar (e.g. MK = Marksman)]_deletion#. Deletions are ordered by size from 116 bp (T4‐29B‐CE_del) to 4 bp (T4‐52A‐M043_del1). Blue bars = deletion length (bp), orange bars = number of potential TFBS (100% match, no species duplications) found within the corresponding sequence in CS (IWGSCrefseq_v1.0). Of the 53 observed deletions, 17 (32%) contain no recognized TFBS. Exact details for each small deletion (for all traits) including sequence and position relative to the ATG start codon for all analysed promoters are given in Data S6. (e and f) details of the positions of TFBSs found for two deletions occurring in MK for del1 (e) and del2 (f). Please note that the MK haplotype including these 2 deletions also includes 14 SNPs and that this haplotype (del1, del2 and 14 SNPs) is shared by three other commercial cultivars (Piko, Revelation and Skyfall) (Data S4).

We investigated whether any of the identified SNPs resided within recognized plant transcription factor binding sites (TFBS), and if the small InDels contained or corresponded to TFBS. For individual SNPs, this could result in the gain or loss of potential TFBS, whereas cultivars containing the small deletions would have lost any TFBS contained within. This in turn may lead to changes in homoeologue‐specific gene expression. Typical examples for both scenarios in biotic stress genes are shown in Figure 5. The commercial cultivar Alcedo (AL) contains seven SNPs in the promoter of the gene TraesCS2A02G343100/ T4‐5A, which are identical in 18 other wheat cultivars and one landrace from the Watkins collection. Of these seven SNPs, three did not reside within any predicted TFBS. However, the other four SNPs resulted in the gain or loss of predicted TFBS (Figure 5a–c). The analysis of all small deletions in the promoters of the biotic stress genes is shown in Figure 5d, which also provides details for the two deletions identified in the promoter of TraesCS7D02G524300/ T4‐45 in cv. Marksman shown in Figure 5e,f. Importantly, of the 53 observed deletions, 36 spanned recognized TFBS. The polymorphisms (SNPs and InDels) identified in the predicted TFBS may be associated with phenotypic variation in traits, and this needs to be determined in future studies. Overall, this detailed analysis shows that the number of predicted TFBSs is not simply proportional to the length of sequence and not all sequences corresponding to deletions contain TFBS. These potential TFBS would of course have to be confirmed experimentally, but these predicted sites may prove a good starting point for studying regulation of gene expression of any of the genes included in this study. Details for all deletions are included in Data S6.

### Analysis of the promoter of *Stb6*, a novel disease resistance gene

The *Stb6* locus, residing on chromosome 3A, confers resistance to one of Europe's most important fungal pathogens, *Zymoseptoria tritici*, which causes Septoria tritici leaf blotch disease. Homoeologues of *Stb6* are not present on the B or D sub‐genomes (Saintenac *et al*., 2018).

**Figure 6:**
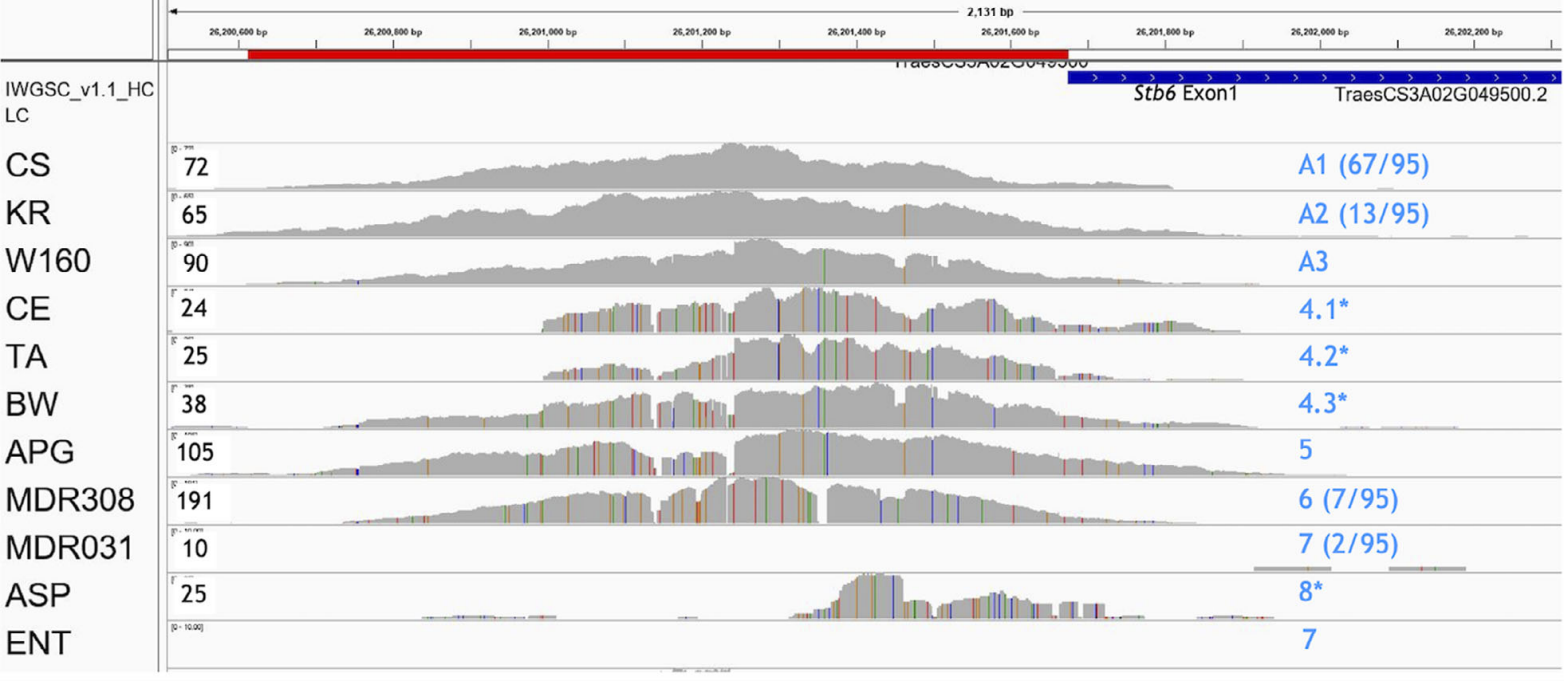
Sequence coverage and haplotypes for the promoter of the *Stb6* resistance gene and homologous sequences captured from genotypes not known to contain *Stb6*. Coverage patterns (grey) observed for *Stb6* on chromosome 3A (TraesCS3A02G049500, T4‐4) from hexaploid wheat genotypes and tetraploid or diploid species with genomes related to wheat. Only A1 to A3 are *Stb6* promoter haplotypes. The other six captured sequences correspond to promoters of genes homologous to *Stb6*. Black numbers show the maximum read depth for each cultivar. Red bar = promoter (target sequence), blue bar = exon 1. The observed haplotypes and their frequencies are shown on the right (blue text). *For the unique but very similar homologous sequences 4.1–4.3 (CE, TA, BW) and 8 (ASP), there is low coverage depth (˜25) compared to the *Stb6* haplotypes.

The promoter of this cloned wall‐associated receptor kinase‐like disease resistance gene, TraesCS3A02G049500/ T4‐4, was included in this study. A generally very low level of polymorphism in the *Stb6* promoter sequence was observed in line with most genes in this study (see above, Figure 3) and only three haplotypes have been identified. Sixty‐six hexaploid cultivars have the identical sequence (haplotype A1) to the CS reference (Figure 6). Twelve hexaploid bread cultivars and the tetraploid durum wheat KR contain a single SNP in the proximal promoter (haplotype A2, position [−143]). This SNP lies within a predicted TFBS, the ‘TTGATC motif’, which is lost, but a different TFBS, ‘W‐box’ potentially is created by this SNP. One unique haplotype carrying 5 SNPs was identified in Watkins160 landrace (haplotype A3). Interestingly, the first SNP (closest to the CDS) is identical to that in durum wheat KR. Moreover, the sequences captured from the wheat genotypes Cellule (CE), Taichung 29 (TA) and Bobwhite (BW) contained an unusually high level of SNPs and InDels suggesting that these likely represent unknown genes homologous to *Stb6* while the *Stb6* gene is missing in these genotypes. This fits well with our previously published study (Saintenac *et al*., 2018) in which we failed to amplify the *Stb6* CDS from these same three cultivars. These variants are very similar but not identical (see Figure 6 for comparison). While CE and TA both appear to have a large deletion from [−611] because the distal part of the promoter was not captured and have an almost identical SNP pattern, for cv. Bobwhite the distal promoter was captured (A4.3). Sequences similar to the *Stb6* promoter were captured from 7 out 8 analysed *T*. *monococcum* (A^m^A^m^) genotypes and the *Ae. peregrina* (S^v^S^v^UU) genome. The expected and observed absence of coverage for *Ae. tauschii* reconfirms the specificity of the baits used, because *Stb6* is present on 3A and no homoeologues are present in either the D or B sub‐genomes (Saintenac *et al*., 2018). No sequences similar to *Stb6* appear to be present in the *T. monococcum* accession MDR031 or as expected in genotypes with the S (related to B) or D genomes, *Ae*. *speltoides* (ASP) and *Ae*. *tauschii* accession ENT‐228 (ENT), respectively (Figure 6).

The low level of polymorphism of the *Stb6* promoter was confirmed through the subsequent BLAST analysis of 13 recently sequenced wheat genomes including Cadenza (CA), Kronos (KR), Svevo, Zavitan and *T*. *spelta* (Figure S9a). Moreover, through the BLAST analysis of the raw Illumina sequence reads archive (NCBI accession SRX4474698) originating from the whole genome re‐sequencing of a *T. monococcum* accession KU104‐1 at RIKEN, Japan we obtained the *Stb6* gene‐related sequence (Figure S9b) that is identical to the one we identified in this study in the seven *T. monococcum* accessions including M308 (aka DV92). Importantly, these data confirm the accuracy of the promoter sequence capture analysis pipeline employed in this study.

### Identity of promoter sequences for targeted genes between IWGSC_refseq_v1.0 and v2.0

During completion of this study, the updated Chinese Spring reference genome, CS_refseq_v2.0, was released by IWGSC. We have therefore subsequently compared both the target sequence similarity as well as the relative positions of all genes included in this project residing on one chromosome, Chr3A, between refseq_v1.0 used for this study and refseq_v2.0. This showed that 54 of the 57 genes (95%) have identical target sequences upstream of the ATG start site in both reference genomes. Of the remaining three genes, two have 99% homology (a single  nucleotide deletion (TraesCS3A02G105500) and a 9 bp insertion (TraesCS3A02G129000) in refseq_v2.0) while the third is still 93% identical (Identities = 1617/1748, Gaps = 77/1748) and is the only gene to contain a significant number of changes. Furthermore, the relative location of virtually all included genes on Chr3A has changed only slightly, with the exception of TraesCS3A02G311100 (T1‐4) which resides on 3AS in refseq_v2.0 compared to 3AL in refseq_v1.0, but the target sequence of this gene is again identical in both reference genomes (all data in Data S7). Additionally, all 133 target sequences where SNPs were found for CS in refseq_v1.0 on all chromosomes (see above, Figure S7) are also identical in refseq_v2.0.

The complete data set (fastq files for all cultivars) is available within the ENA BioProject PRJEB45647.

## Discussion

The high‐quality data set presented here allows for the first time detailed analysis of individual homoeologue promoters of wheat genes across the three sub‐genomes. The high‐stringency capture used permitted high‐confidence SNPs and InDels to be analysed within these individual homoeologue promoters. This should contribute directly to greater insight into the variance of homoeologue‐specific gene expression both within one species and across a wide variety of wheats and related species. In addition, these data are already being employed by UK wheat breeders and wheat researchers to generate high‐confidence KASP markers for a wide range of trait genes.

In this study, at a modest cost, a highly flexible experimental approach, hitherto only applied to exome analysis, was devised which now provides a wealth of comparative promoter and 5′UTR polymorphism data for a large cohort of UK elite hexaploid cultivars as well as a range of wheat accessions and species important for wheat improvement (e.g. Watkins and *T. monococcum* lines). These data can be used to provide new insights in numerous fundamental research projects and to enhance the knowledge associated with emerging wheat genetic resources (e.g. TILLING lines for cvs. Cadenza and Kronos (King *et al*., 2015), a tiling path population for the Avalon x Cadenza introgressions, that is ‘individual cv. Cadenza segment introgression into a cv. Avalon background and individual cv. Avalon segment introgression into a cv. Cadenza background’, https://designingfuturewheat.org.uk/resources/, http://www.wgin.org.uk/). The high specificity of the baits capture, which considerably simplified the subsequent data handling and analyses, was only achieved because a highest stringency approach was taken for the design and use of all the baits. This made individual capture of homoeologue promoter and 5′UTR sequences at high sequencing depths routinely possible. Also, we found that complete capture of the target sequences could be achieved with only a few well‐spaced baits, reducing the design and costs of similar capture experiments.

From this study, eight highlights are particularly noteworthy and these provide greater insights into wheat genomes and how analyses can be further refined:

1. The upstream regulatory regions of most genes were found to be remarkably conserved with <7 haplotypes per target sequence identified across the diverse set of 83 hexaploid cultivars used. Most of these haplotypes consist of only 5 or fewer SNPs and most of the identified haplotypes are very similar with a core of identical SNPs and a few either added or missing. This result was completely unexpected and strongly suggests that wheat promoters have been conserved during modern wheat breeding. Whereas prior to this study, the generally accepted view was that the promoter sequences were likely to be less conserved than the coding sequences.
2. A surprisingly high 48% of analysed promoters share identical haplotypes between Watkins landraces and commercial cultivars, suggesting that these specific haplotypes are fairly common in diverse wheat germplasm.
3. There is strong evidence for ancestral introgression either directly from *T. monococcum* or more likely indirectly via *T. timopheevii* to the A sub‐genome in many hexaploid wheats.
4. Many of the SNPs identified map to potential plant transcription factor binding sites either creating, changing or obliterating TFBSs. These SNPs may lead to changes in triad gene expression patterns and as a result altered trait phenotypes.
5. Individual trait categories differed only slightly from the overall pattern regarding shared and unique haplotypes and SNP diversity. Whereas the biggest difference between trait categories appears to be their non‐random chromosome distribution. We had anticipated promoter polymorphism differences between trait categories that need to respond to a wide range of environmental stimuli [e.g. biotic stress (Moore *et al*., 2011)], compared to those which primarily respond to internal stimuli [e.g. grain composition (Pfeifer *et al*., 2014)] or are involved in fundamental cellular processes [e.g. recombination]. Instead, these new findings indicate that there is a need for similar levels of promoter conservation for both cell type and stage‐dependent gene expression.
6. Missing transposable elements are very easy to identify in the comparative IGV displays because they appear as gaps in the sequencing coverage of individual cultivars with sharply defined ‘cliff edges’.
7. For *Ae. peregrina*, the data set clearly indicates that this ancient species has a more complex origin than hitherto suspected.
8. Our alignment of recently sequenced wheat cultivars to the *Stb6* gene and promoter as well as reverse alignments to a recently sequenced *T. monococcum* accession confirm the validity and high confidence of the SNPs reported in this study.

In other temperate inbreeding crop plant species, SNP frequencies present in coding and non‐coding regions of the genome have been calculated. Although no comparative databases currently exist to directly compare frequencies across plant species, two studies are of relevance to this promoter study. For commercial large fruited tomato cultivars, SNP frequencies are very low within the range ˜2 to 4 SNPs/1 kbp in the non‐coding regions even though >95% of SNPs occur in non‐coding regions (Causse *et al*., 2013). In comparison, a study of 433 barley accessions, including 344 wild and 89 domesticated barley genotypes, revealed SNP frequencies to be 29 SNPs/1 kbp in coding regions and 41 SNPs/1 kbp in non‐coding regions (Pankin *et al*., 2018). Whereas in the wheat promoter study reported here, homozygous SNP+InDel frequencies of 1.9 ± 0.4 kbp^−1^ were observed in the 69 commercial varieties, 1.9 ± 0.3 kbp^−1^ in the 14 Watkins landraces and a markedly increased 14.1 ± 0.9 kbp^−1^ in the eight *T. monococcum* lines. The near identical polymorphism frequencies between commercial wheat cultivars and Watkins landraces was surprising, but serves again to highlight the generally low polymorphism in different wheat genotypes including landraces. Although these different studies are not directly comparable, it is still surprising that the frequencies reported here appear to be tenfold less than reported for barley, but very close to tomato.

We report here, for the first time, highly specific individual capture and detailed analysis of homoeo‐allele promoters for a great diversity of functional wheat genes. This success was only possible because of the high‐stringency and high masking approach used when designing the baits (performed by Dr. J.Enk, Daicel Arbor Biosciences). This strategy also significantly reduces the time required to complete the bioinformatic alignment of the captured sequences to the CS reference genome and allows the calling of high‐confidence homozygous SNPs. Surprisingly, this level of bait stringency did not compromise our ability to capture sequences at a high read depth even from the non *T. aestivum* species. It is also noteworthy that although the design of a comprehensive bait set across the entire sequence of interest is recommended, this was not actually required for the acquisition of high‐quality data sets from either *T. aestivum* or non *T. aestivum* species. Our analysis of captured sequences revealed that even with just seven well‐spaced high‐stringency baits more than 1700 bp of target sequence can be captured with high specificity and good read depth. This more limited bait cover would permit researchers to investigate a far greater number (˜4 times greater) of genes of interest or considerably longer sequences within a single capture experiment for the same cost. Finally, the technical approach used in this study also successfully permitted the calling of absent sequences within the promoters and absent genes in individual cultivars, even to the point that a nullisomic cultivar (Hobbit) could be identified. Likewise, entire promoters with large numbers of polymorphisms for individual homoeologues from non *T. aestivum* species were captured and sequenced to high depth. These important observations and reported findings would allow researchers to explore very diverse germplasm collections using the same experimental approach with a high level of confidence.

In another wheat study, a different array‐based approach was used to capture gene and promoter sequences across the entire wheat genome for CS and eight other *T. aestivum* lines from the CIMMYT breeding programme (Gardiner *et al*., 2019). Both a reduced bait cover and sample multiplexing were used. Using this approach, capture sequences for the target genes and putative promoter target regions ranged between 62 and 73%. However, no detailed analysis of the polymorphisms present in either the exon or promoter sequences obtained was reported, nor was the specificity of capture of the homoeologues from the three sub‐genomes explored. Furthermore, the target read depths were considerably lower, most likely due to the DNA‐DNA hybridization used in that study compared to the stronger RNA‐DNA baits hybridization employed in our study. We therefore would strongly recommend RNA‐DNA hybridization methodology as used in this study to be used for similar capture experiments.

Overall, an unanticipated low number of haplotypes were identified in the germplasm explored. This can be partially explained because wheat is an inbreeding species, modern wheat breeding is only ˜120 years old and most commercial germplasm is related by pedigree. However, the finding that a lot of haplotypes found in the Watkins landraces and some haplotypes found in *T. monococcum*, both germplasms having diverse origins and ploidy levels and not having been previously extensively used in modern wheat breeding, were already present in many modern commercial wheats would not have been anticipated. This provides evidence for either direct or indirect ancestral introgression events and merits further investigation. This new knowledge will immediately speed up the exploitation of variant promoter sequences in modern wheat breeding.

Over the next few years and at considerable cost, the genomes of many additional wheat lines will be sequenced, of different read depths, fully or partially assembled and then annotated (e.g. the 10+ Wheat Genomes Project; http://www.10wheatgenomes.com) (Adamski *et al*., 2020). In the meantime, our highly flexible and cost‐effective way of reducing the complexity of the hexaploid wheat genome could be adopted to obtain comparative sequence information for any part of the CDS of interest, for any gene type, any large or small gene family and/ or different wheat germplasm. Using the current promoter and 5′UTR data sets, either KASP markers to individual SNPs can be designed or targeted genotyping by sequencing could be done to provide SeqSNPs, both of which could then be used by wheat breeders to immediately exploit this hitherto unknown promoter variation. In addition, the capture of homoeologue‐specific 5′ exon/intron sequence data for the different wheat genotypes is likely to be exceptionally useful when linking the promoter and 5′ UTR sequences to other projects which have generated cultivar specific transcriptome data sets. Finally, wheat GWAS studies to link phenotypes to genotypes by field phenotyping many traits within large cohorts of diverse germplasm could be greatly improved by capturing promoter data sets in order to identify potentially causal polymorphisms in TFBSs.

The identity in the reference genomes IWGSC CS refseq_v1.0 (used in this study) and refseq_v2.0 (released subsequently) for 54 of the 57 Chr3A genes included in this study demonstrates again the extremely high quality of the IWGSC CS refseq_v1.0 genome and strongly suggests that similar identities would be found on the other wheat chromosomes. Therefore, the analyses and results reported here using CS refseq_v1.0 would be expected to be either very close or identical in refseq_v2.0.

The freely available complete data set generated here will allow researchers to examine specific genes of interest directly and should in particular contribute to gene regulation studies because the low number of SNPs and InDels in the promoters should accelerate confirmation and / or discovery of TFBSs.

## Methods

### Germplasm selection, seed acquisition and seed stock retention

A collaborative approach was taken for the selection of the 96 wheat genotypes (Table S1). In total, 68 of the 96 selected genotypes were commercial historic and modern hexaploid wheat cultivars. A further 15 were hexaploid wheat landraces selected from the A. E. Watkins collection (Winfield *et al*., 2018; Wingen *et al*., 2014). Also included were eight accessions of the diploid species *T*. *monococcum* (2n = 2x = 14; A^m^A^m^), whose genome is related but not identical to the A sub‐genome of durum and bread wheat, and which possess desirable new traits for wheat improvement (Jing *et al*., 2007; Li *et al*., 2018; McMillan *et al*., 2014; Simons *et al*., 2021). Further controls included were the hexaploid bread wheat landrace CS for which a fully annotated reference genome is available; the tetraploid durum wheat cv. Kronos (2n = 4x = 28; AABB); the ancestral species *Ae. tauschii* (2n = 2x = 14; DD) that contributed the D sub‐genome of hexaploid wheat, *Ae*. *speltoides* (2n = 2x = 14; SS) whose diploid genome is related to the B sub‐genome of hexaploid wheat and the tetraploid wild species *Ae. peregrina* (2n = 4x = 28; S^v^S^v^UU). These controls were included to be able to determine the specificity of the technology used in capturing homoeo‐alleles, and in the case of the reference CS genome to determine the overall accuracy of the sequencing methodology – ideally no SNPs should appear in the captured sequences of CS relative to the CS reference to which all reads were mapped.

Seed stocks for the majority of the accessions were obtained from the Genetics Resources Unit (GRU) at the John Innes Centre (https://www.jic.ac.uk/research‐impact/germplasm‐resource‐unit; https://www.seedstor.ac.uk). Seed stocks for most of the *T. monococcum* genotypes originally came from The Vavilov Institute, St Petersburg, Russia (Jing *et al*., 2007). Whereas seeds for MDR308 and MDR657 came from Professor Jorge Dubcovsky, University of California at Davis and the Max Planck Institute, Cologne, Germany, respectively (Jing *et al*., 2009). Each plant used for sampling was grown to maturity and seed from the first spike was collected for future reference. Additional information on each genotype is given in Data S2.

### Plant growth and DNA preparation

Seeds were pre‐germinated on moist filter paper for 3 days at room temperature and then transferred to Levington F2S seed & modular compost in P40 trays. Leaf tip samples (5 cm in length) were taken at the 2‐leaf stage from each seedling for DNA preparation. Only a single plant for each of the 96 genotypes was selected for DNA extraction. Genomic DNA was extracted from young leaf material with NorGen Plant / Fungus DNA Isolation kits (https://norgenbiotek.com/product/plantfungi‐dna‐isolation‐kit) and DNA integrity and concentrations confirmed by 0.8% agarose gel electrophoresis and Qubit fluorescent dye measurements. All seedlings of the winter wheat accessions selected for DNA extraction were then transferred into vernalization conditions for 8 weeks. Either post‐vernalization or when the seedlings of the spring wheat varieties were at the 3‐leaf stage each plant was transferred singly into a 1.5 L pot containing Rothamsted prescription mix compost with fertilizers added when required. Each plant was individually bagged prior to anthesis until full grain maturation. All mature ears were photographed before storage.

### Gene selection

Following discussions with UK academics and wheat breeders, ten traits for wheat improvement were selected and known or candidate genes underlying these traits were collated. For each of the ten traits shown in Table 1, trait coordinators were chosen who provided the gene IDs linked to each trait. Approximately 10% of candidate genes originated from other crop species, and therefore, for these a BLAST search against IWGSC_refseq_v1.0 was done to identify the likely wheat orthologues.

### Bait design, bait selection, promoter capture and DNA sequencing

A myBaits capture technology service provided by Daicel Arbor Biosciences was utilized to retrieve the specific promoter sequences of interest. To ensure the highly specific capture of promoters of individual homoeo‐alleles in wheat, a high‐stringency workflow was followed for the baits design. The original target FASTA file comprised roughly 2.4 Mbp sequence space. This was first soft‐masked using the cross_match algorithm and the Triticum repeat library available at RepeatMasker.org. These targets were then tiled with 120 nt probe candidates every 60 nt (i.e. with 50% probe‐probe overlap) and then screened against the IWGSC RefSeq_v1.0 for specificity. Probes with multiple strong predicted hybridization sites and/or that were 25% or more soft‐masked were then removed. This reduced the original probe candidate list by more than 50%, leaving a final 17745 surviving probe sequences that were subsequently synthesized as part of a myBaits‐1 kit with Daicel Arbor Biosciences. These 17745 high‐stringency baits were targeting 1700 bp of sequences located upstream of the annotated start codon of each of the 1273 homoeo‐alleles. For 63 genes, the target sequence was enlarged to take into account alternate transcriptional start sites (up to a maximum of 4376 bp target length for the gene TraesCS2A02G122200/ T2‐22 from the most downstream alternate translation start site). For 34 genes, only 5′UTR sequence baits were designed because these genes have very large predicted 5′UTRs (up to 5 kbp). Furthermore, for 33 genes the 1700 bp target sequence had to be reduced because of large stretches of unidentified nucleotides (Ns) upstream in the reference sequence (down to a minimum of 854 bp for gene TraesCS5B02G175800/ T2‐39). Short stretches of Ns within the target sequence were randomly assigned nucleotides using the standard proprietary Daicel Arbor Biosciences algorithms. These nucleotides are shown as small letters in the bait sequences (Data S1).

The myReads team at Daicel Arbor Biosciences first sonicated the DNA extracts using a QSonica Q800R sonicator and subsequently size‐selected the sheared material to 400–600 bp lengths. Then, they converted up to 80% of the size‐selected material (between 18 and 500 ng) to dual‐indexed TruSeq‐style Illumina sequencing libraries, each with unique combinations of dual 8 bp indexes, using 6 cycles of indexing amplification. Then, 500 ng of each library (with one exception: 81 ng of library for sample ‘Watkins 239’) was enriched with the custom myBaits‐1 kit following manual version 4.01, with 10 cycles of post‐capture amplification. They then constructed two pools of 48 enriched libraries with equal mass contribution per library and submitted these for sequencing on a HiSeq 2500 instrument using PE100 chemistry at a third party provider. FASTQs were post‐processed and demultiplexed by both index sequences and subsequently taken to analysis.

### Galaxy workflow

No trimming of reads took place. The captured sequences were mapped to the CS genome reference (IWGSC_refseq_v1.0). Within Galaxy (Giardine, 2005), BWA mem (v0.7.17) was used to map the raw reads, with samTools (v1.3.1) to convert and sort to bam, followed by picard tools (v2.14) for marking duplicate reads. The resulting bam files were left aligned to amalgamate tandem repeat indels. Polymorphisms (variants) were called using Freebayes, using a minimum quality of bases and read mapped of 10. SnpSift (v4.0.0) (Cingolani *et al*., 2012) was used to filter with a minimum coverage of 10 total reads and a quality score of 30.

### Visualization of mapped reads

Binary Alignment Map (BAM) and Variant Call Format (VCF) files were downloaded from Galaxy and used for subsequent visualization and analysis using the IGV (Integrative Genome Viewer) software, initially. All BAM/VCF files generated for this project will be made available upon full publication of the manuscript together with the full genome (161010_Chinese_Spring_v1.0_pseudomolecules_parts.fasta) and the second version (1.1) of the gene annotation file used (IWGSC_v1.1_HCLC_parts_genome.gff3). The latest version of IGV can be downloaded from https://software.broadinstitute.org/software/igv/download.

### Pedigree and introgression visualization

Pedigrees were viewed using the Helium software (Fradgley *et al*., 2019) normally to a pedigree depth of eight to gauge the relationships between cultivars. For the few cultivars where no relationship to any of the other 83 hexaploid wheat cultivars at this pedigree depth was found, all available data were investigated (https://github.com/cardinalb/helium‐docs/wiki).

For comparison of the potential introgression events on chromosome arms 5AL, 6AS and 7AS as found in this study, available cultivars were checked using the CerealDB Putative Introgression Plotter (https://www.cerealsdb.uk.net/cerealgenomics/CerealsDB/search_introgressions.ph).

### Bespoke bioinformatics analyses

For the TFBS analyses, all small deletions and some individual SNPs were searched for containing or being part of TFBS using the NSite‐PL (Recognition of PLANT Regulatory motifs with statistics) software online (http://www.softberry.com/berry.phtml?topic=nsitep&group=programs&subgroup=promoter). Concerning individual SNPs, the sequence was selected in IGV ±5 bp surrounding the SNP and both the 11 bp sequence for the wild type and SNP version were searched. For this analysis, the search results were filtered to include only 100% matches of recognized plant TFBS (Shahmuradov and Solovyev, 2015; Solovyev *et al*., 2010).

The Geneious bioinformatics platform was used for the comparison of homoeologue sequences using various alignment tools (https://www.geneious.com/ *)*. Specifically for the *Stb6* analyses, multiple sequences alignment was carried out using ClustalW in Geneious.

To search for transposable elements, all the large deletions were compared using BLASTN against the TREP (https://botserv2.uzh.ch/kelldata/trep‐db/index.html) and CLARITE_CLARIrepeatwheat databases.

## Conflict of Interest

The authors declare no competing interests.

## Author contributions

KK and KHK conceived the project. MHK, KK and KHK designed the experiment. MHK performed the experiment, that is generate the FASTA file for all target sequences and isolated genomic DNA for all cultivars. RK and MHK handled the raw data and performed the sequence alignments and SNP calling. MHK analysed the experimental data. KK analysed the *Stb6* experimental data. RK performed the polymorphism analyses for all cultivars. MHK, KK and KHK wrote the article with technical contributions from RK. All authors read, revised and approved the final manuscript.

## Supporting information


**Figure S1** Distribution of trait genes across the Chinese Spring wheat chromosomes.

**Figure S2** Polymorphism frequency per cultivar.

**Figure S3** The concept of core, shared and unique haplotypes.

**Figure S4** Relationships between (a) commercial varieties sharing the *T. monococcum* MDR037 haplotype A1 for gene TraesCS5A02G558200 (T5‐10) and (b) pedigrees of cultivars used in relation to Chinese Spring.

**Figure S5** Cultivars with missing genes.

**Figure S6** Physical locations of genes with potential *T. monococcum* introgression.

**Figure S7** SNP diversity and occurrence observed in the control Chinese Spring accession.

**Figure S8** Larger deletions observed in Biotic Stress (Trait 4) gene promoters.

**Figure S9** Alignments of recently fully sequenced wheat genomes for Stb6.

**Table S1** The 96 wheat cultivars/accessions included in this study.
**Table S2** Total numbers of mapped sequences, SNPs, InDels and homozygous polymorphisms frequency for each cultivar.
**Table S3** Cultivar distribution of Trait 4 promoter large deletions.

**Data S1** List of all 1273 homoeologues including IWGSCrefseq1.1 gene IDs, complete target sequences and individual details for all baits used.

**Data S2** Details for all cultivars.

**Data S3** Sequencing lengths, depths and homeologue specificity.

**Data S4** Hexaploid haplotypes.

**Data S5** Shared haplotypes between “ancestral” and hexaploids.

**Data S6** Deletions, TFBS.

**Data S7** Genes on Chr3A refseq1.0 vs 2.0.

## Data Availability

All the data files used for the analyses reported here are available from OwnCloud https://rrescloud.rothamsted.ac.uk/index.php/s/DMCFDu5iAGTl50u/authenticate. Raw sequencing reads have been deposited in the ENA database under BioProject PRJEB45647.
