# Supplementary figures and images for: Exploring the diversity of promoter and 5′UTR sequences in ancestral, historic and modern wheat
Source: Plant Biotechnol J. 2021 Sep 16;19(12):2469–87. doi: 10.1111/pbi.13672 (PMC8633512; doi:10.1111/pbi.13672)

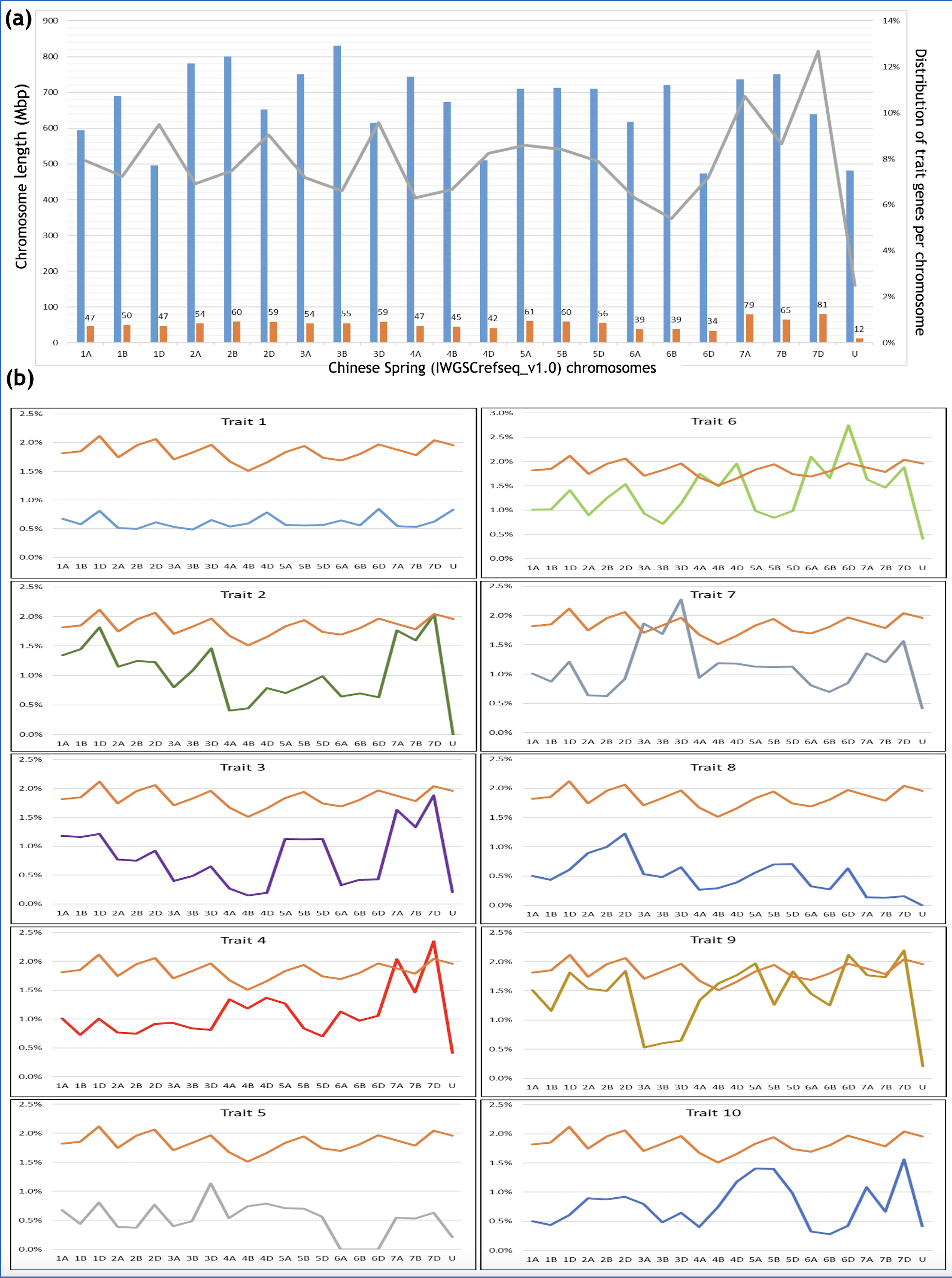

Supplement: Supplementary file 1 — Figure S1 Distribution of trait genes across the Chinese Spring wheat chromosomes. [file PBI-19-2469-s007.png]

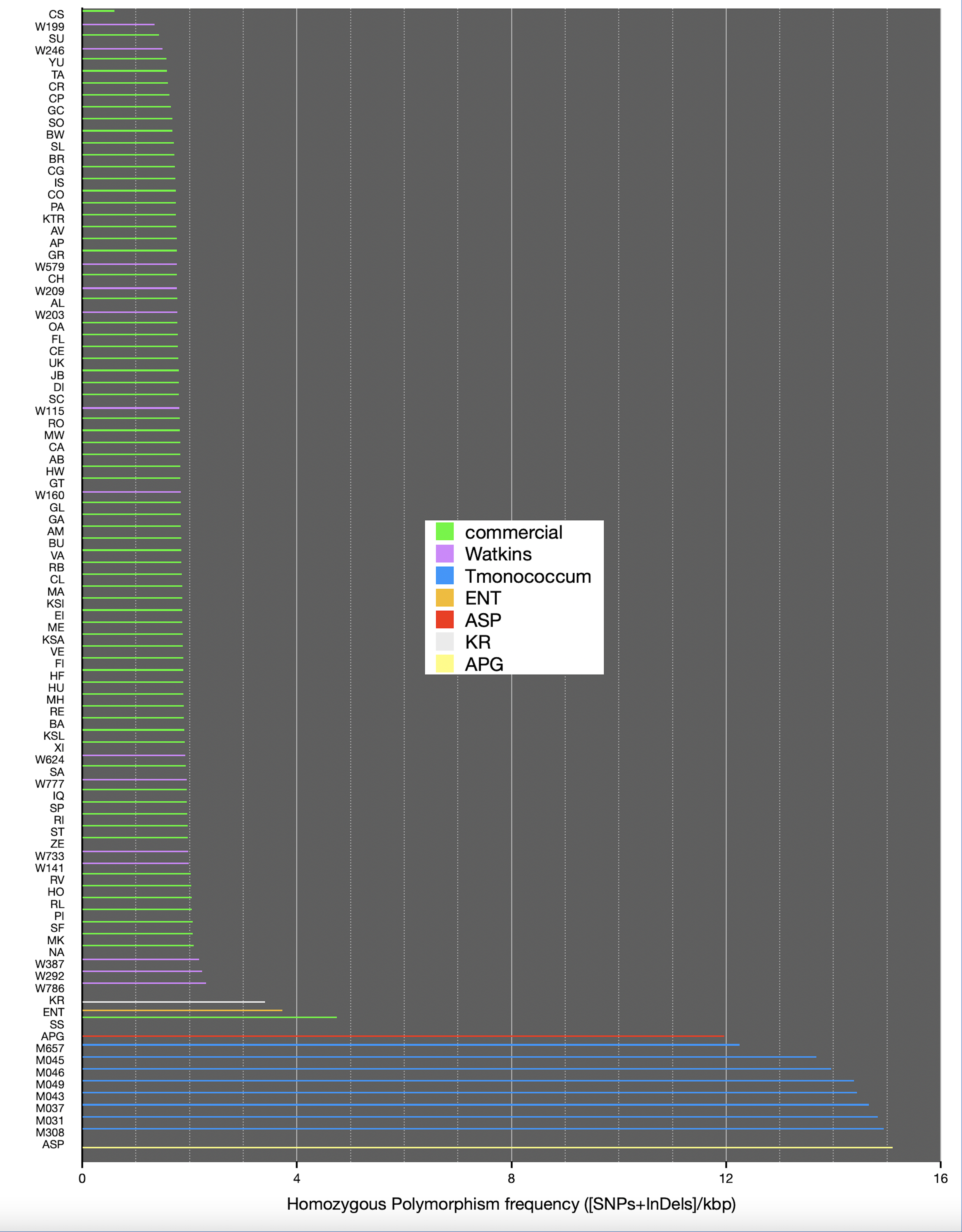

Supplement: Supplementary file 2 — Figure S2 Polymorphism frequency per cultivar. [file PBI-19-2469-s003.png]

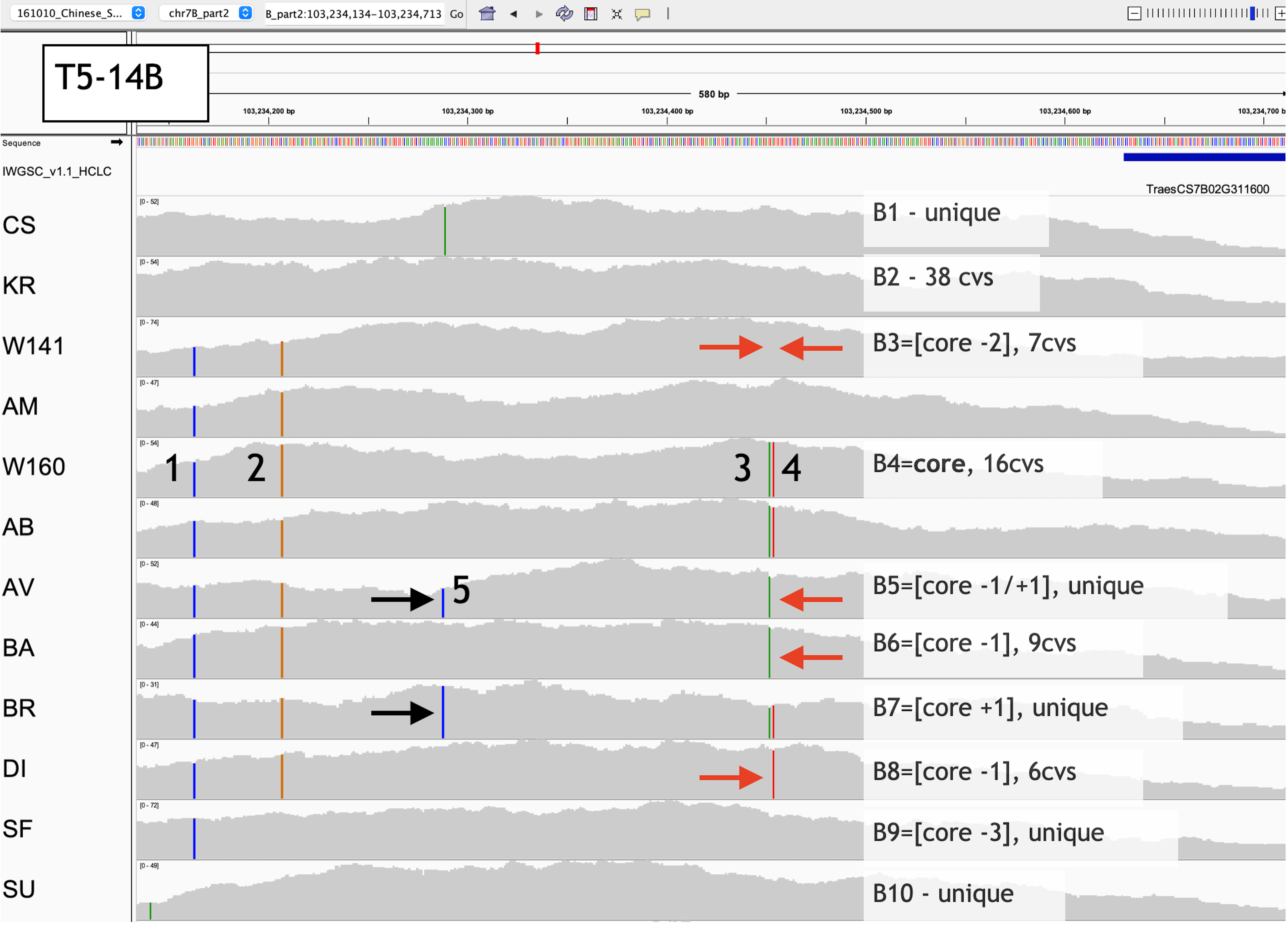

Supplement: Supplementary file 3 — Figure S3 The concept of core, shared and unique haplotypes. [file PBI-19-2469-s005.png]

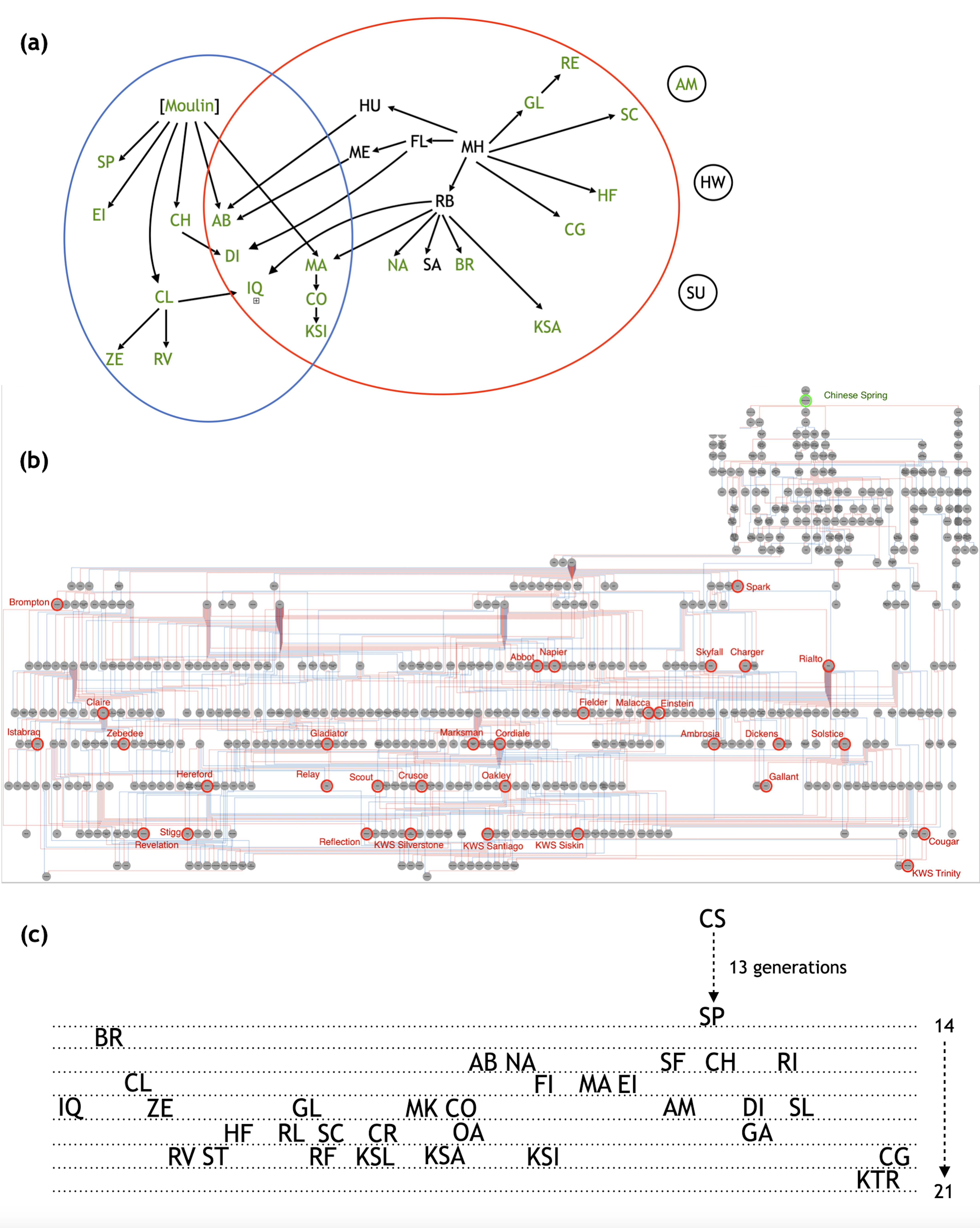

Supplement: Supplementary file 4 — Figure S4 Relationships between (a) commercial varieties sharing the T. monococcum MDR037 haplotype A1 for gene TraesCS5A02G558200 (T5‐10) and (b) pedigrees of cultivars used in relation to Chinese Spring. [file PBI-19-2469-s013.png]

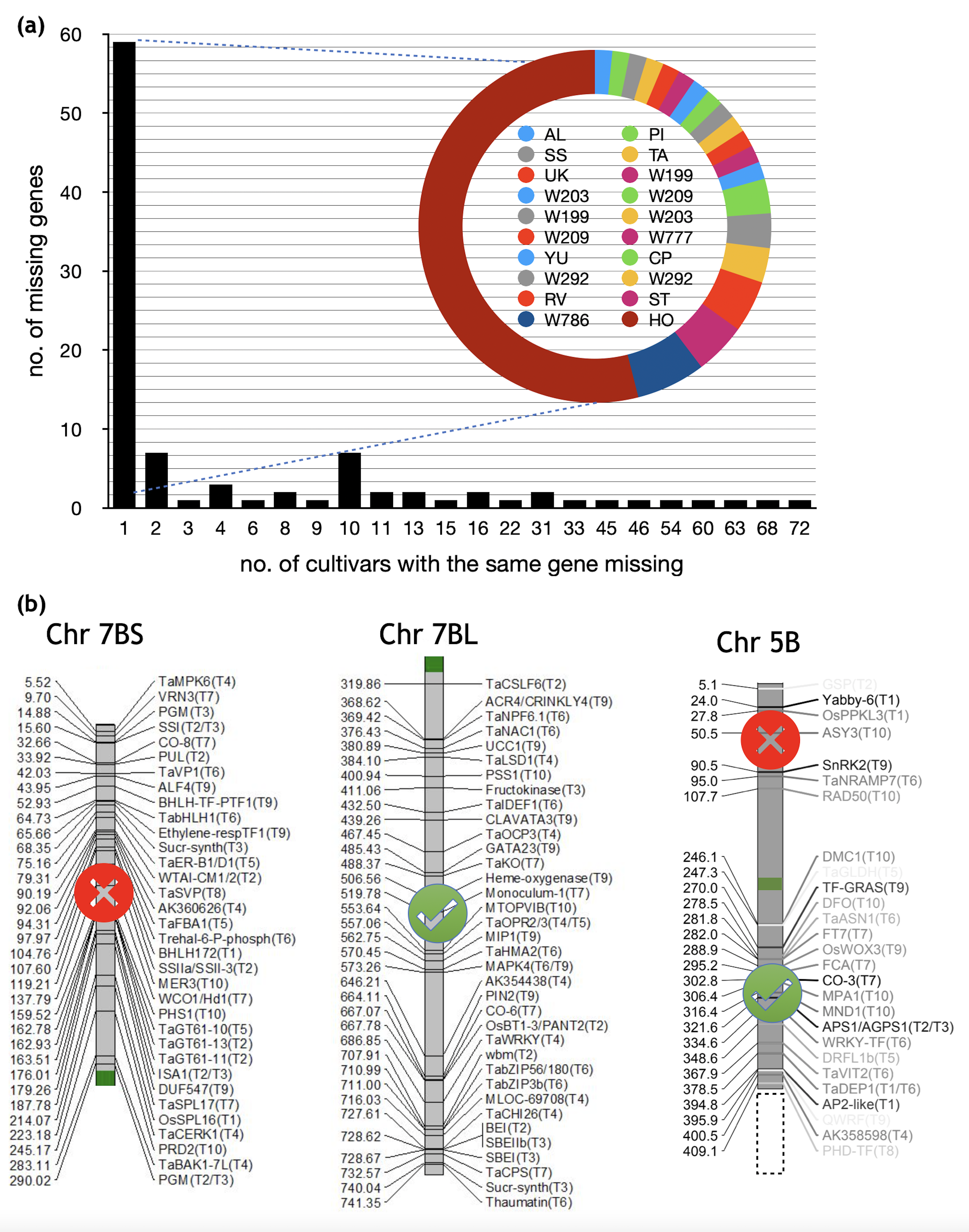

Supplement: Supplementary file 5 — Figure S5 Cultivars with missing genes. [file PBI-19-2469-s012.png]

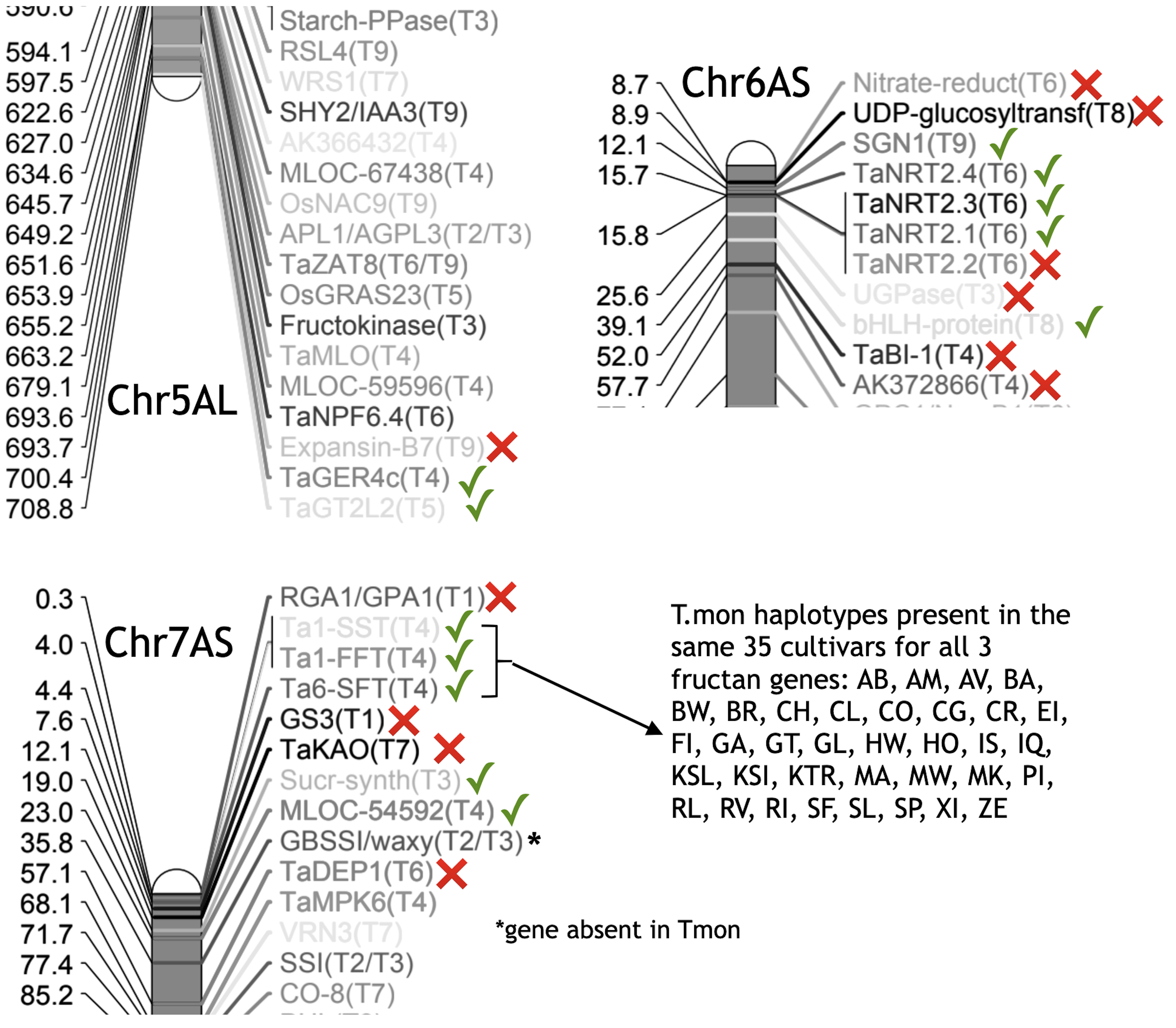

Supplement: Supplementary file 6 — Figure S6 Physical locations of genes with potential T. monococcum introgression. [file PBI-19-2469-s014.png]

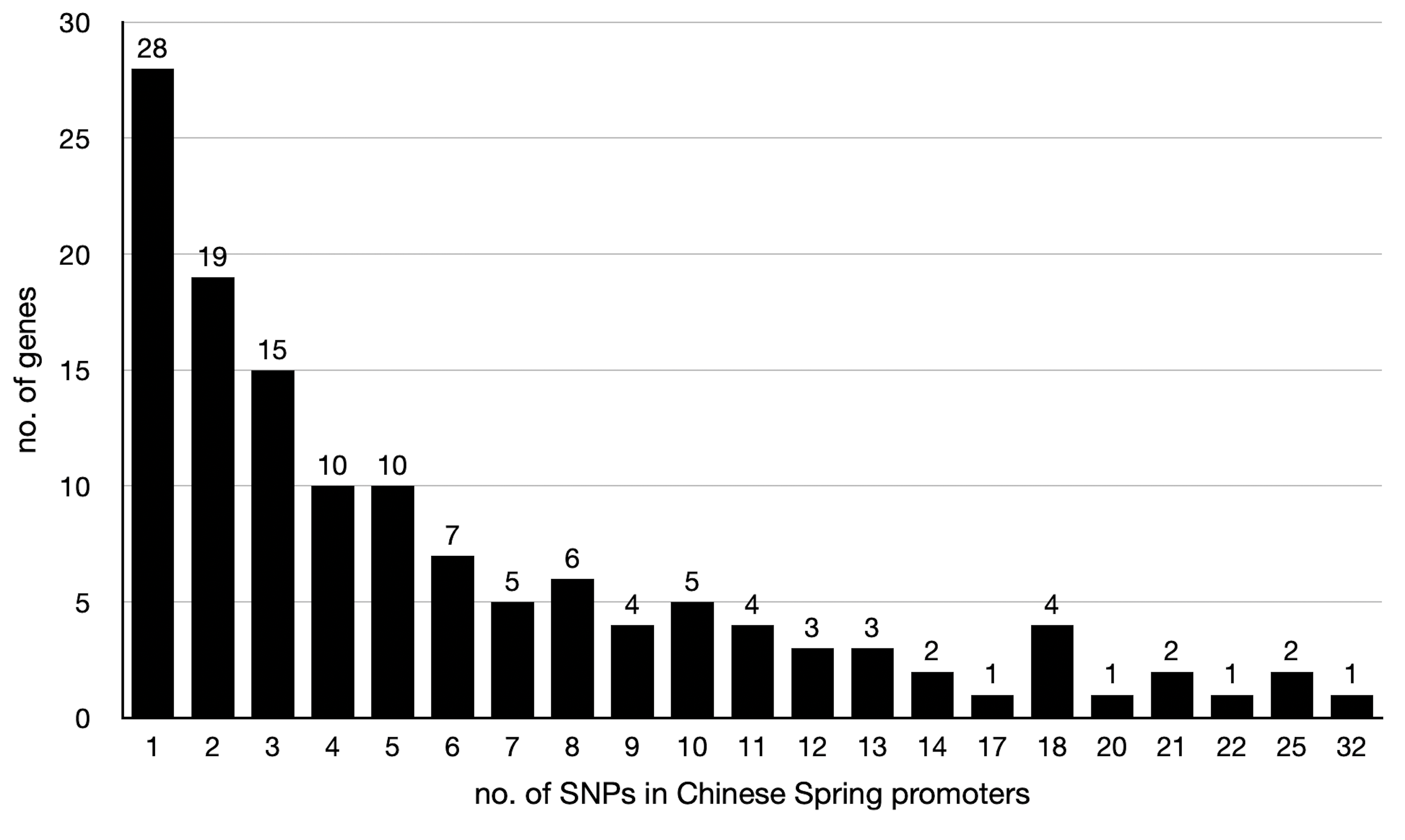

Supplement: Supplementary file 7 — Figure S7 SNP diversity and occurrence observed in the control Chinese Spring accession. [file PBI-19-2469-s010.png]

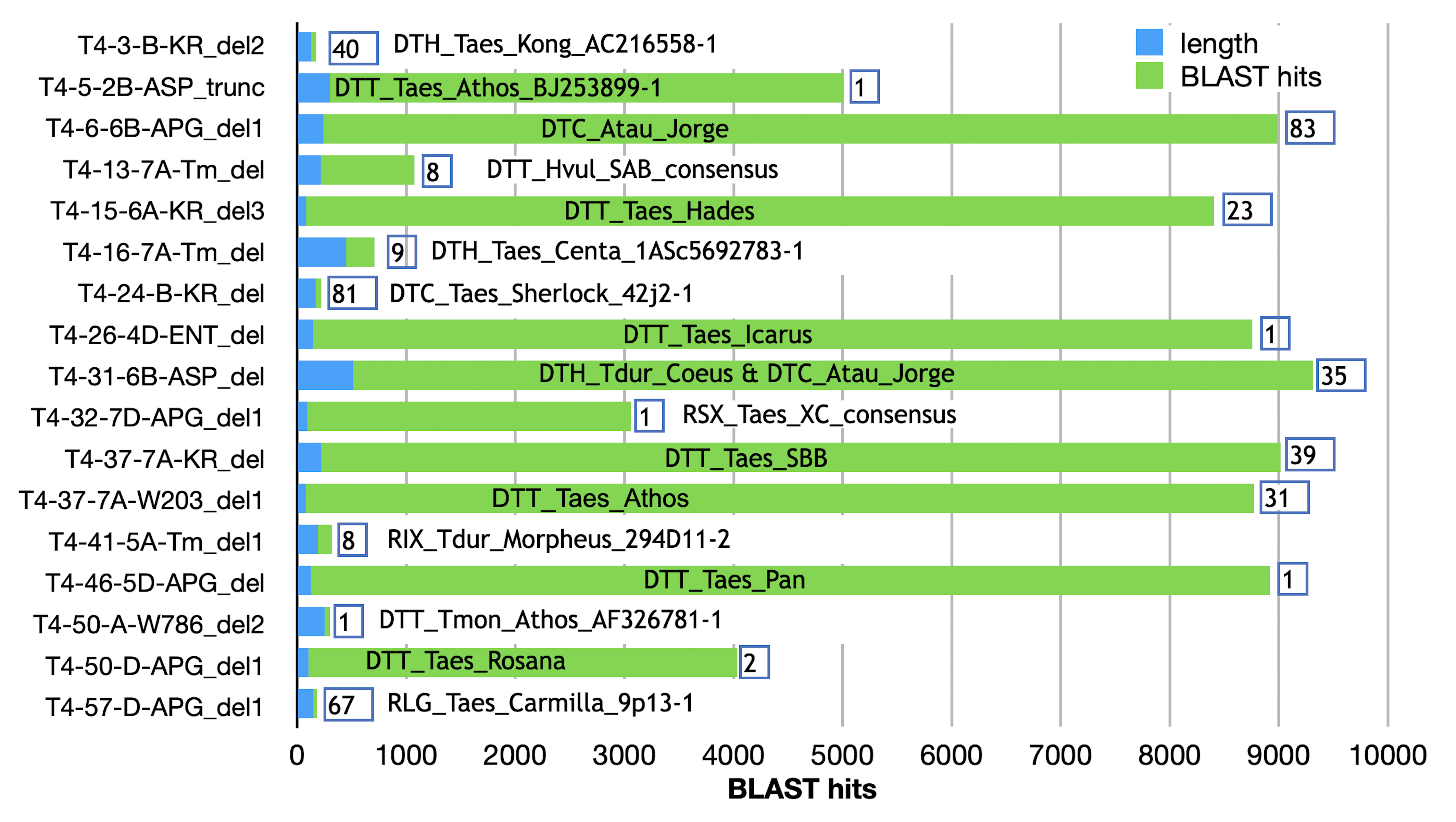

Supplement: Supplementary file 8 — Figure S8 Larger deletions observed in Biotic Stress (Trait 4) gene promoters. [file PBI-19-2469-s009.png]

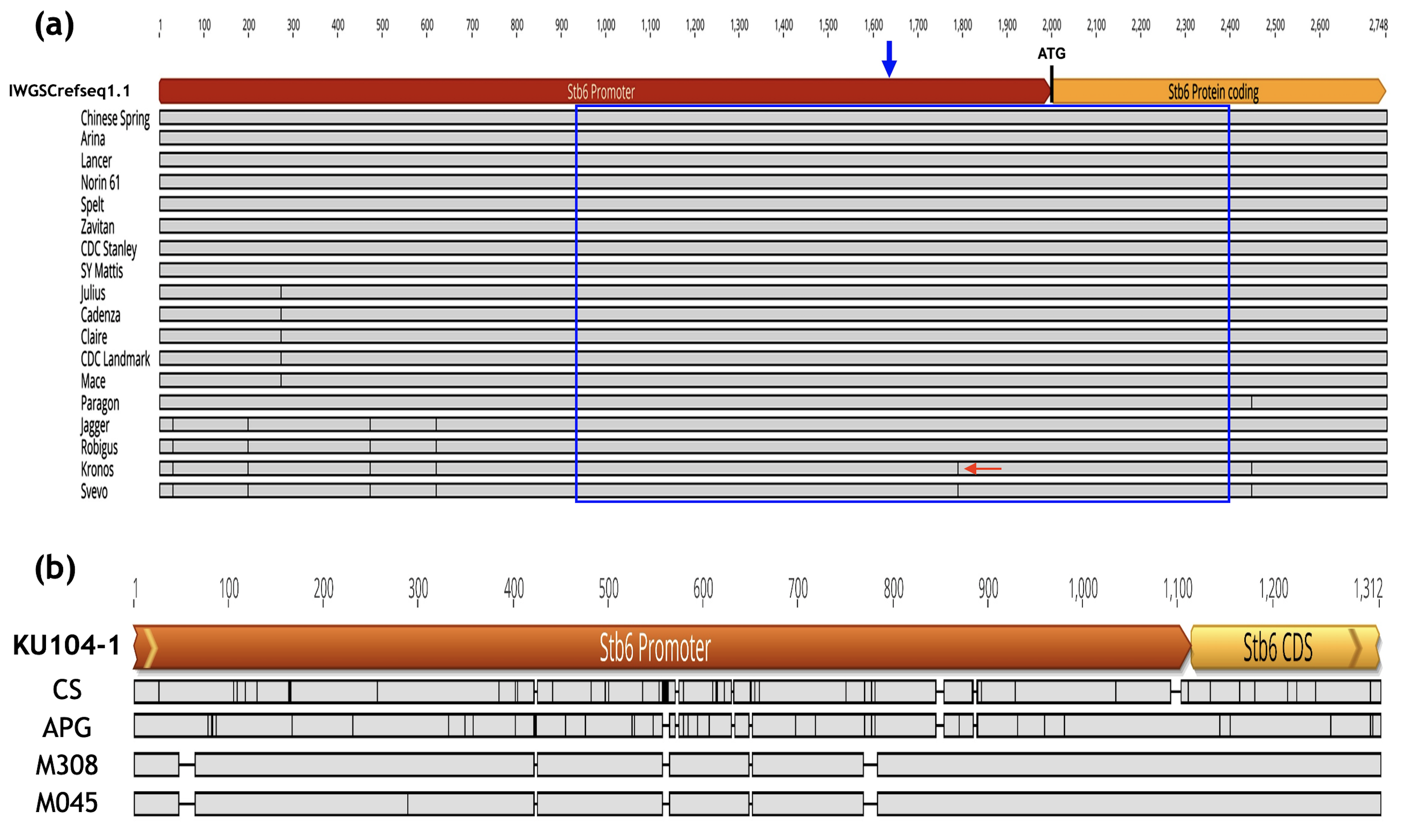

Supplement: Supplementary file 9 — Figure S9 Alignments of recently fully sequenced wheat genomes for Stb6. [file PBI-19-2469-s017.png]
