# Supplementary material for: Exploring the diversity of promoter and 5′UTR sequences in ancestral, historic and modern wheat
Source: Plant Biotechnol J. 2021 Sep 16;19(12):2469–87. doi: 10.1111/pbi.13672 (PMC8633512; doi:10.1111/pbi.13672)
Supplement: Supplementary file 10 — Table S1 The 96 wheat cultivars/accessions included in this study. Table S2 Total numbers of mapped sequences, SNPs, InDels and homozygous polymorphisms frequency for each cultivar. Table S3 Cultivar distribution of Trait 4 promoter large deletions. [file PBI-19-2469-s001.docx]

**Supplementary Figure 1: Distribution of trait genes across the Chinese Spring wheat chromosomes. a,** Distribution of all WGIN trait genes on individual wheat chromosomes. Shown are the lengths of chromosomes (blue bars), no. of WGIN trait genes on each chromosome (orange) and the percentage of WGIN trait genes per chromosomes (grey line). **b**, percentage of genes in individual WGIN trait categories per chromosome compared to all wheat genes (IWGSCrefseq_v1.0) (orange).

**Supplementary figure 2: Polymorphisms frequency per cultivar.** Homozygous polymorphism frequency is shown as homozygous [SNPs+InDels]/kbp (1000bp of captured sequence) from lowest (CS) to highest (ASP). Colour coding differentiates between commercial cultivars (green), 14 Watkins landraces (purple), the diploid 8 *T. monococcum* accessions (blue), *Ae. speltoides* (ASP, yellow) and tetraploid *T. durum* Kronos (KR, grey) and *Ae. peregrina* (APG, red). The vast majority of commercial cultivars have ≤2 polymorphisms/kpb, and this is also true for the Watkins landraces. Notably of the hexaploid cultivars, Sear Synthetic (SS) has a higher frequency (4.7) as anticipated. Considerably higher frequencies were also found for the distantly A genome related *T. monococcum* accessions (M031-M657) and the Aegilops species ASP and APG.

**Supplementary figure 3: The concept of core, shared and unique haplotypes.** This example shows the promoter of T5-14B (TraesCS7B02G311600, TaOPR2). 38 cultivars have NO_SNPs (B2, one shared haplotype). CS has a single unique SNP (hence a unique haplotype, B1) and this is also true for SU (another unique haplotype, B10). For this promoter, the SNP pattern of W160 (4 SNPs) has been identified as the core haplotype B4 (shared by another 15 cultivars, for which AB is shown as an example), from which the remaining other haplotypes can be generated through simple addition or subtraction. The missing and added SNPs are indicated by red and black arrows, respectively. The W141 haplotype B3 shared by 6 other cultivars (of which AM is shown as an example) is denoted as [core -2] because SNPs3&4 are absent, while the unique haplotype B5 for AV is shown as [core -1/+1] because it has lost SNP4 but has gained SNP5. Therefore, in total for this promoter there are 5 shared haplotypes (B2, B3, B4, B6, B8) and 5 unique haplotypes (B1, B5, B7, B9, B10). Note that all SNPs have the same nucleotide change in all haplotypes. If, as occurred in some promoters, two cultivars have a different nucleotide SNP at the same position, these would be called separate haplotypes.

**Supplementary Figure 4: Relationships between (a) commercial varieties sharing the *T. monococcum* MDR037 haplotype A1 for gene TraesCS5A02G558200 (T5-10) and (b) pedigrees of cultivars in relation to Chinese Spring. a)** generated from the Helium software pedigree displays (https://github.com/cardinalb/helium-docs/wiki) for the cultivars sharing haplotype A1 (with a pedigree depth of 8). The various circled groups show the relationship of these 30 cultivars, with 20 cultivars sharing the parent Maris-Huntsman (MH) and 12 sharing Moulin as a parent. Only 3 varieties, Ambrosia, Hereward and Sumai3 share no pedigree with any of the other 27. Green cultivars also have Chinese Spring (CS) as a distant ancestor. **b)** The pedigree of all cultivars included in this Promotome project originating from CS(Supplementary Table 1). **c)** Simplified version of panel (b) emphasising the generations.

**Supplementary Figure 5: Cultivars with missing genes. a,** The number of genes where any number of cultivars between 1 and 72 have the same gene missing. The doughnut insert shows the percentages of individual cultivars with missing genes where only 1 cultivar has the gene missing (all indicated inside the doughnut). Hobbit (HO) has 34 genes missing (details in Supplementary Data4 ‘NO gene’ tab). **b)** all these 34 genes reside on chromosome 7BS, but also these are ALL the genes included in this experiment residing in this location in the IWGSCrefseq1.1 Chinese Spring chromosomes. While all the genes from Chr7BS are missing in Hobbit, ALL genes residing on Chr7BL are present in this cultivar, as well as all other 82 hexaploid cultivars. This is also true for Chr5B, where all genes included in this study residing on Chr5BS in CS are missing from Hobbit, while genes residing on Chr5BL are present. Please note only the top half of Chr5BL (up to 409Mbp) are shown. Centromere location = green bar.

**Supplementary figure 6: Physical locations of genes with potential *T. monococcum* introgression.** Of the 16 promoters with potential *T. monococcum* introgression found in this analysis (details in Supplementary Data 5), 12 cluster in 3 locations on chromosomes 5AL, 6AS and 7AS, in all cases very close to the telomere end of the chromosome arms. Green ticks show genes with *T. monococcum* haplotypes found in hexaploid cultivars while red crosses show that these genes do not share *T. monococcum* haplotypes with *T. aestivum* cultivars. Particularly noteworthy is the cluster of the 3 fructan biosynthesis genes on Chr7AS, because the *T. monococcum* haplotypes found in these genes’ promoters are shared by exactly the same group of 35 commercial cultivars. Gene locations are shown in Mbp on left of chromosomes, functional gene names with trait category in brackets on right.

**Supplementary Figure 7: SNP diversity and occurrence observed in the control Chinese Spring accession.** Shown are the SNP diversity of Chinese Spring promoters and the number of promoters where these occur, eg a single SNP was observed in 28 promoters, whereas occurrences of more than 20 SNPs per promoter where confined to 1 or 2 genes, and the maximum no. of SNPs was 1 incidence with 32 SNPs. These SNPs (814 in total) were found across 133 homoeologue promoters compared to CSrefseq1.0 (used in this study) as well as CSrefseq2.0 (released subsequently) while the remaining 775 promoters (n=908) had zero SNPs.

**Supplementary Figure 8: Larger deletions observed in Biotic Stress (Trait 4) gene promoters.** The labels at the left show each large deletion in Biotic Stress gene promoters (Trait 4) marked as T4-[gene_number]-[homoeologue]-[cultivar]_del[number]. The named cultivar is the first observed with this deletion in the IGV analysis where cultivars were sorted by diploid, tetraploid, hexaploid landraces and hexaploid commercial cultivars in alphabetical order. blue bars = length of each observed deletion for the specified genes. green bars = number of BLAST hits against all IWGSCrefseq_v1.0 chromosomes. Each sequence was also searched against the CLARIrepeatwheat database (Urgi) as well as the TREP database. Text inside or next to the green bars shows identification of this sequence as (part of) known transposable elements (Supplementary Data 6). Furthermore, the boxed numbers outside the green bars show how many of the 95 cultivars included in this study have this deletion. Details about individual cultivars having these deletions are shown in Supplementary Table 3.

**Supplementary Figure 9: Alignments of more recently fully sequenced wheat genomes for Stb6. a,** Alignment of Stb6 putative promoter and partial protein coding sequence identified in the recently sequenced genomes of wheat: Spelt, *Triticum spelta* accession; Zavitan, *Triticum turgidum* ssp. *dicoccoides* genotype; Kronos and Svevo, *T. durum* cultivars. All others are *T. aestivum* cultivars. Blue arrow = predicted transcriptional start site, blue box = portion of the Stb6 gene and promoter captured in this study. The single Kronos SNP in the proximal Stb6 promoter found in this study (Figure 6) is confirmed by this subsequent analysis (red arrow). **b,** Alignment of Stb6 promoter and partial protein coding sequence with the homologous sequences identified in species related to wheat. Chinese Spring (CS), *T. aestivum* reference genome; APG, *Ae. peregrina*, MDR308 (=DV92), MDR045 and KU104-1 are *T. monococcum* accessions. Six out of eight *T. monococcum* accessions investigated using sequence capture in this study and one accession KU104-1, for which the whole genome re-sequencing data is available at NCBI, have the sequences identical to that in MDR308, while only a single SNP is present in *T. monococcum* MDR045. This reciprocal analysis (using *T. monococcum* as the reference genome) confirms the large number of SNPs found in MDR308 and APG in this study, but also confirms that most APG SNPs are distinct from *T. monococcum*.

**Supplementary Table 1: The 96 wheat cultivars/accessions included in this study.** Please note that all *T. aestivum* cultivars denoted with an asterisk are descendants of Chinese Spring, albeit remote ones (see Supplementary Figure 2b&c for details). For the *T. monococcum* accessions and the Watkins landraces the geographical origins are shown. T.=Triticum, Ae.=Aegilops. More details about these cultivars, including their source, are included in Supplementary Data 2.

| **species** | **cultivar [origin]** | **abbreviation** | **genome** | **species** | **cultivar [origin]** | **abbreviation** | **genome** |
| --- | --- | --- | --- | --- | --- | --- | --- |
| *Ae.peregrina* | *Not known* | **APG** | U^p^U^p^S^p^S^p^ | *T. aestivum* | KWS Trinity* | **KTR** | AABBDD |
| *Ae.speltoides* | *Not known* | **ASP** | SS | *T. aestivum* | Malacca* | **MA** | AABBDD |
| *Ae.tauschii* | ENT-228 | **ENT** | DD | *T. aestivum* | Maris Huntsman | **MH** | AABBDD |
| *T. durum* | Kronos | **KR** | AABB | *T. aestivum* | Maris Widgeon | **MW** | AABBDD |
| *T.monococcum* | MDR031 [Turkey] | **M031** | A^m^A^m^ | *T. aestivum* | Marksman* | **MK** | AABBDD |
| *T.monococcum* | MDR037 [Armenia] | **M037** | A^m^A^m^ | *T. aestivum* | Mercia | **ME** | AABBDD |
| *T.monococcum* | MDR043 [Greece] | **M043** | A^m^A^m^ | *T. aestivum* | Napier | **NA** | AABBDD |
| *T.monococcum* | MDR045 [Denmark] | **M045** | A^m^A^m^ | *T. aestivum* | Oakley | **OA** | AABBDD |
| *T.monococcum* | MDR046 [Romania] | M046 | A^m^A^m^ | *T. aestivum* | Paragon | **PA** | AABBDD |
| *T.monococcum* | MDR049 [Iran] | **M049** | A^m^A^m^ | *T. aestivum* | Piko | **PI** | AABBDD |
| *T.monococcum* | MDR308 (= DV92) [Italy] | **M308** | A^m^A^m^ | *T. aestivum* | Reflection | **RF** | AABBDD |
| *T.monococcum* | MDR657 [Turkey] | **M657** | A^m^A^m^ | *T. aestivum* | Relay | **RL** | AABBDD |
| *T. aestivum* | Abbot* | **AB** | AABBDD | *T. aestivum* | Revelation | **RV** | AABBDD |
| *T. aestivum* | Alcedo | **AL** | AABBDD | *T. aestivum* | Rialto | **RI** | AABBDD |
| *T. aestivum* | Ambrosia* | **AM** | AABBDD | *T. aestivum* | Riband | **RB** | AABBDD |
| *T. aestivum* | Avalon | **AV** | AABBDD | *T. aestivum* | Robigus | **RO** | AABBDD |
| *T. aestivum* | Badger | **BA** | AABBDD | *T. aestivum* | Savannah | **SA** | AABBDD |
| *T. aestivum* | Bobwhite | **BW** | AABBDD | *T. aestivum* | Scout | **SC** | AABBDD |
| *T. aestivum* | Brompton* | **BR** | AABBDD | *T. aestivum* | Sear Synthetic | **SS** | AABBDD |
| *T. aestivum* | Buster | **BU** | AABBDD | *T. aestivum* | Skyfall | **SF** | AABBDD |
| *T. aestivum* | Cadenza | **CA** | AABBDD | *T. aestivum* | Soisson | **SO** | AABBDD |
| *T. aestivum* | Cellule | **CE** | AABBDD | *T. aestivum* | Solstice | **SL** | AABBDD |
| *T. aestivum* | Charger* | **CH** | AABBDD | *T. aestivum* | Spark | **SP** | AABBDD |
| *T. aestivum* | Chinese Spring | **CS** | AABBDD | *T. aestivum* | Stigg | **ST** | AABBDD |
| *T. aestivum* | Claire* | **CL** | AABBDD | *T. aestivum* | Sumai 3 | SU | AABBDD |
| *T. aestivum* | Coppadra | **CP** | AABBDD | *T. aestivum* | Taichung 29 | TA | AABBDD |
| *T. aestivum* | Cordiale* | **CO** | AABBDD | *T. aestivum* | Ukrainka | UK | AABBDD |
| *T. aestivum* | Cougar* | **CG** | AABBDD | *T. aestivum* | USU-Apogee | **AP** | AABBDD |
| *T. aestivum* | Crusoe* | **CR** | AABBDD | *T. aestivum* | Valoris | VA | AABBDD |
| *T. aestivum* | Dickens* | **DI** | AABBDD | *T. aestivum* | Veranopolis | VE | AABBDD |
| *T. aestivum* | Einstein* | **EI** | AABBDD | *T. aestivum* | Watkins 115 | W115 | AABBDD |
| *T. aestivum* | Fielder* | **FI** | AABBDD | *T. aestivum* | Watkins 141 | W141 | AABBDD |
| *T. aestivum* | Flanders | **FL** | AABBDD | *T. aestivum* | Watkins 160 | W160 | AABBDD |
| *T. aestivum* | Gallant* | **GA** | AABBDD | *T. aestivum* | Watkins 199 | W199 | AABBDD |
| *T. aestivum* | Garcia | **GC** | AABBDD | *T. aestivum* | Watkins 203 | W203 | AABBDD |
| *T. aestivum* | Gatsby | **GT** | AABBDD | *T. aestivum* | Watkins 209 | W209 | AABBDD |
| *T. aestivum* | Gladiator* | **GL** | AABBDD | *T. aestivum* | Watkins 239 | W239 | AABBDD |
| *T. aestivum* | Graham | **GR** | AABBDD | *T. aestivum* | Watkins 246 | W246 | AABBDD |
| *T. aestivum* | Hereford* | **HF** | AABBDD | *T. aestivum* | Watkins 292 | W292 | AABBDD |
| *T. aestivum* | Hereward | **HW** | AABBDD | *T. aestivum* | Watkins 387 | W387 | AABBDD |
| *T. aestivum* | Hobbit | **HO** | AABBDD | *T. aestivum* | Watkins 579 | W579 | AABBDD |
| *T. aestivum* | Hustler | **HU** | AABBDD | *T. aestivum* | Watkins 624 | W624 | AABBDD |
| *T. aestivum* | Isengrain | **IS** | AABBDD | *T. aestivum* | Watkins 733 | W733 | AABBDD |
| *T. aestivum* | Istabraq* | **IQ** | AABBDD | *T. aestivum* | Watkins 777 | W777 | AABBDD |
| *T. aestivum* | JB Diego | **JB** | AABBDD | *T. aestivum* | Watkins 786 | W786 | AABBDD |
| *T. aestivum* | KWS Santiago* | **KSA** | AABBDD | *T. aestivum* | Xi19 | XI | AABBDD |
| *T. aestivum* | KWS Silverstone* | **KSL** | AABBDD | *T. aestivum* | Yumai 34 | YU | AABBDD |
| *T. aestivum* | KWS Siskin* | **KSI** | AABBDD | *T. aestivum* | Zebedee | ZE | AABBDD |
|  |  |  |  |  |  |  |  |

**Supplementary Table 2: Total numbers of mapped sequences, SNPs, InDels and polymorphisms frequency for each cultivar.** Accessions are listed in alphabetical order for ease of locating individual cultivars (abbreviations see Supplementary Table 1). cvs=cultivar, *sequ=total mapped sequence captured in Mbp with mapping quality 10 filter, total polymorphisms=all heterozygous and homozygous SNPs and InDels. Hom=Polymorphisms (SNPs+InDels) filtered for homozygosity, frequ= no. of homozygous SNPs+InDels/kbp

|  |  | **Total polymorphisms** | | **Homozygous polymorphisms** | |  |  | **Total polymorphisms** | | **Homozygous polymorphisms** | |
| --- | --- | --- | --- | --- | --- | --- | --- | --- | --- | --- | --- |
| **cvs** | **Sequ* (Mbp)** | **SNPs** | **InDels** | **Hom** | **Frequ** | **cvs** | **Sequ* (Mbp)** | **SNPs** | **InDels** | **Hom** | **Frequ** |
| **AB** | 2.93 | 9,896 | 933 | 5,351 | 1.8 | **M308** | 9.81 | 248,884 | 15,116 | 146,610 | 14.9 |
| **AL** | 2.85 | 8,755 | 847 | 5,040 | 1.8 | **M657** | 4.20 | 91,375 | 5,838 | 51,503 | 12.3 |
| **AM** | 2.90 | 9,923 | 937 | 5,336 | 1.8 | **MA** | 3.23 | 11,329 | 1,026 | 6,010 | 1.9 |
| **AP** | 3.28 | 10,428 | 969 | 5,772 | 1.8 | **ME** | 2.89 | 9,977 | 978 | 5,393 | 1.9 |
| **APG** | 2.43 | 67,902 | 5,802 | 29,045 | 12.0 | **MH** | 3.05 | 10,789 | 1,002 | 5,755 | 1.9 |
| **ASP** | 4.46 | 122,571 | 9,758 | 67,317 | 15.1 | **MK** | 3.99 | 16,809 | 1,377 | 8,216 | 2.1 |
| **AV** | 2.98 | 9,292 | 892 | 5,225 | 1.8 | **MW** | 3.59 | 13,071 | 1,115 | 6,537 | 1.8 |
| **BA** | 3.47 | 12,131 | 1,081 | 6,570 | 1.9 | **NA** | 3.87 | 17,688 | 1,462 | 8,053 | 2.1 |
| **BR** | 2.28 | 7,364 | 669 | 3,913 | 1.7 | **OA** | 2.88 | 9,061 | 895 | 5,089 | 1.8 |
| **BU** | 3.29 | 11,741 | 1,063 | 6,072 | 1.8 | **PA** | 3.68 | 12,288 | 1,104 | 6,428 | 1.7 |
| **BW** | 2.34 | 6,917 | 651 | 3,941 | 1.7 | **PI** | 3.95 | 15,741 | 1,349 | 8,068 | 2.0 |
| **CA** | 3.05 | 10,107 | 949 | 5,558 | 1.8 | **RB** | 3.77 | 13,395 | 1,149 | 6,980 | 1.8 |
| **CE** | 2.46 | 7,257 | 742 | 4,386 | 1.8 | **RE** | 4.41 | 16,854 | 1,366 | 8,342 | 1.9 |
| **CG** | 2.45 | 7,404 | 748 | 4,225 | 1.7 | **RI** | 5.01 | 20,632 | 1,624 | 9,814 | 2.0 |
| **CH** | 2.85 | 9,100 | 863 | 5,028 | 1.8 | **RL** | 3.54 | 13,007 | 1,207 | 7,213 | 2.0 |
| **CL** | 3.62 | 12,760 | 1,163 | 6,734 | 1.9 | **RO** | 3.65 | 12,695 | 1,142 | 6,641 | 1.8 |
| **CO** | 2.65 | 7,913 | 787 | 4,618 | 1.7 | **RV** | 3.79 | 14,841 | 1,230 | 7,659 | 2.0 |
| **CP** | 3.02 | 9,304 | 865 | 4,900 | 1.6 | **SA** | 3.27 | 12,054 | 1,064 | 6,306 | 1.9 |
| **CR** | 1.46 | 3,917 | 381 | 2,331 | 1.6 | **SC** | 2.71 | 8,538 | 810 | 4,876 | 1.8 |
| **CS** | 3.15 | 6,022 | 555 | 1,894 | 0.6 | **SF** | 4.43 | 17,655 | 1,447 | 9,115 | 2.1 |
| **DI** | 3.07 | 10,564 | 941 | 5,514 | 1.8 | **SL** | 2.67 | 7,218 | 755 | 4,553 | 1.7 |
| **EI** | 3.10 | 10,724 | 981 | 5,782 | 1.9 | **SO** | 3.15 | 9,029 | 821 | 5,295 | 1.7 |
| **ENT** | 4.44 | 24,002 | 2,413 | 16,527 | 3.7 | **SP** | 4.31 | 16,058 | 1,377 | 8,382 | 1.9 |
| **FI** | 3.24 | 12,199 | 1,109 | 6,098 | 1.9 | **SS** | 3.65 | 27,905 | 2,680 | 17,323 | 4.7 |
| **FL** | 2.86 | 10,277 | 882 | 5,101 | 1.8 | **ST** | 4.61 | 18,609 | 1,473 | 9,049 | 2.0 |
| **GA** | 2.72 | 9,124 | 904 | 4,996 | 1.8 | **SU** | 2.85 | 7,535 | 717 | 4,076 | 1.4 |
| **GC** | 2.21 | 5,884 | 638 | 3,655 | 1.7 | **TA** | 3.36 | 9,873 | 898 | 5,304 | 1.6 |
| **GL** | 2.57 | 9,261 | 856 | 4,725 | 1.8 | **UK** | 3.01 | 11,019 | 862 | 5,403 | 1.8 |
| **GR** | 2.66 | 8,717 | 836 | 4,677 | 1.8 | **VA** | 3.17 | 11,137 | 1,012 | 5,854 | 1.8 |
| **GT** | 2.78 | 9,825 | 911 | 5,075 | 1.8 | **VE** | 3.60 | 12,538 | 1,165 | 6,746 | 1.9 |
| **HF** | 2.81 | 10,029 | 962 | 5,288 | 1.9 | **W115** | 3.25 | 10,502 | 952 | 5,887 | 1.8 |
| **HO** | 5.34 | 23,466 | 1,744 | 10,845 | 2.0 | **W141** | 4.83 | 19,466 | 1,529 | 9,593 | 2.0 |
| **HU** | 3.38 | 13,053 | 1,156 | 6,368 | 1.9 | **W160** | 2.84 | 8,883 | 864 | 5,205 | 1.8 |
| **HW** | 3.55 | 13,331 | 1,161 | 6,487 | 1.8 | **W199** | 2.87 | 6,540 | 689 | 3,867 | 1.3 |
| **IQ** | 3.70 | 14,469 | 1,236 | 7,200 | 1.9 | **W203** | 2.65 | 7,394 | 733 | 4,688 | 1.8 |
| **IS** | 3.83 | 13,153 | 1,153 | 6,645 | 1.7 | **W209** | 3.35 | 10,667 | 982 | 5,910 | 1.8 |
| **JB** | 2.86 | 9,283 | 866 | 5,142 | 1.8 | **W246** | 3.59 | 10,177 | 913 | 5,374 | 1.5 |
| **KR** | 2.58 | 13,783 | 1,262 | 8,804 | 3.4 | **W292** | 5.73 | 26,504 | 1,968 | 12,815 | 2.2 |
| **KSA** | 3.49 | 13,282 | 1,166 | 6,531 | 1.9 | **W387** | 6.90 | 30,389 | 2,136 | 15,052 | 2.2 |
| **KSI** | 3.08 | 10,996 | 1,051 | 5,741 | 1.9 | **W579** | 4.14 | 15,086 | 1,273 | 7,286 | 1.8 |
| **KSL** | 3.44 | 12,952 | 1,114 | 6,524 | 1.9 | **W624** | 4.12 | 15,375 | 1,255 | 7,909 | 1.9 |
| **KTR** | 2.83 | 9,161 | 850 | 4,943 | 1.7 | **W733** | 3.81 | 14,602 | 1,308 | 7,529 | 2.0 |
| **M031** | 8.63 | 216,835 | 13,200 | 127,922 | 14.8 | **W777** | 3.74 | 13,558 | 1,137 | 7,277 | 1.9 |
| **M037** | 7.58 | 191,506 | 11,687 | 111,202 | 14.7 | **W786** | 9.31 | 47,466 | 2,433 | 21,453 | 2.3 |
| **M043** | 7.01 | 174,665 | 10,844 | 101,290 | 14.4 | **XI** | 3.75 | 12,575 | 1,118 | 7,154 | 1.9 |
| **M045** | 3.65 | 82,236 | 5,621 | 49,895 | 13.7 | **YU** | 2.82 | 7,158 | 718 | 4,413 | 1.6 |
| **M046** | 6.47 | 154,470 | 9,768 | 90,351 | 14.0 | **ZE** | 4.15 | 15,538 | 1,301 | 8,163 | 2.0 |
| **M049** | 9.03 | 224,696 | 13,456 | 129,843 | 14.4 |  |  |  |  |  |  |

**Supplementary Table 3: Cultivar distribution of Trait 4 promoter large deletions.** Please refer to Supplementary Table 1 for cultivar abbreviations. Blank cells show that none of the diploid, tetraploid or hexaploid cultivars have these deletions.

| **Gene ID [deletion]** | **transposable element** | **diploid** | **tetraploid** | **hexaploid** |
| --- | --- | --- | --- | --- |
| TraesCS4B02G329500 [T4-3-B-KR_del2] | DTH_Taes_Kong_AC216558-1 |  | KR | 39 (W141, W160, W209, W292, W387, W624, W733, W786, AB, AL, AM, BW, BR, BU, CL, FI, GT, GL, HF, HO, HU, IQ, KSL, KSI, ME, PA, PI, RE, RL, RI, SS, SO, SL, ST, SU, UK, AP, VA, ZE) |
| TraesCS2B02G340700 [T4-5-2B-ASP_del] | DTT_Taes_Athos_BJ253899-1 | ASP |  | only found in ASP |
| TraesCS6B02G223700 [T4-6-6B-APG_del1] | DTC_Atau_Jorge_D_3D-339 |  | APG | 82 (all but CS) |
| TraesCS7A02G412400 [T4-13-7A-Tm_del] | DTT_Hvul_SAB_consensus-1 | all Tmon |  | only found in T.mon |
| TraesCS6A02G083200 [T4-15-6A-KR_del3] | DTT_Taes_Hades_BQ243447-1 |  | KR | 22 (W141, W160, W203, W579, W624, W777, AM, AV, BA, CR, GL, IQ, MW, MK, NA, RL, RB, SA, SF, TA, UK, ZE) |
| TraesCS7A02G310500 [T4-16-7A-Tm_del] | DTH_Taes_Centa_1ASc5692783-1 | all Tmon | APG |  |
| TraesCS4B02G207100 [T4-24-B-KR_del] | DTC_Taes_Sherlock_42j2-1 |  | KR | 80 (all but CS & ST) |
| TraesCS4D02G319100 [T4-26-4D-ENT_del] | DTT_Taes_Icarus_BE591362-1 | ENT |  | only found in ENT |
| TraesCS6B02G175100 [T4-31-6B-ASP_del] | DTH_Tdur_Coeus_643D12-1, DTC_Atau_Jorge_D_3D-339  (see Figure 4) | ASP | KR | 33 (W141, W209, W292, W397, W579, W624, W733, AB, AM, AV, BW, BU, CE, CH, DI, FL, GT, GL, IS, IQ, KSL, KSI, MW, ME, PA, PI, RL, SF, SO, TA, UK, AP, YU) |
| TraesCS7D02G497400 [T4-32-7D-APG_del1] | RSX_Taes_XC_consensus-1 |  | APG | only found in APG |
| TraesCS7A02G049400 [T4-37-7A-KR_del] | DTT_Taes_SBB_42j2-2 |  | KR | 38 (W160, W579, AL, BU, CL, DI, EI, FL, GC, GT, GL, GR, HF, HO, HU, IQ, JB, KSA, KSL, KSI, KTR, MA, MH, ME, NA, OA, PI, RV, RB, RO, SA, SO, SL, ST, SU, TA, VA, VE) |
| TraesCS7A02G049400 [T4-37-7A-W203_del1] | DTT_Taes_Athos_42j2-5 |  |  | 31 (W203, W209, W387, W624, W777, AB, AM, AV, BA, BR, CA, CE, CH, CG, FI, GA, HW, IS, MW, MK, RE, RL, RI, SS, SF, SP, UK, AP, XI, YU, ZE) |
| TraesCS5A02G515600 [T4-41-5A-Tm_del1] | RIX_Tdur_Morpheus_294D11-2 | all Tmon |  | only found in T.mon |
| TraesCS5D02G233700 [T4-46-5D-APG_del] | DTT_Taes_Pan_42j2-5 |  | APG | only found in APG |
| TraesCS5A02G261400 [T4-50-A-W786_del2] | DTT_Tmon_Athos_AF326781-1 |  |  | W786,  only found in this landrace |
| TraesCS5D02G268800 [T4-50-D-APG_del1] | DTT_Taes_Rosana_4dss00551-1 |  | APG | W292,  only found in this landrace |
| TraesCS3D02G209200 [T4-57-D-APG_del1] | RLG_Taes_Carmilla_9p13-1 | ENT | APG | 83 (all)  found in ALL hexaploid including CS |

**Supplementary Data Files**

nb: explanations for each are on sheet 1 of each excel data file.

**Supplementary Data 1:** List of all 1273 homoeologues including IWGSCrefseq1.1 gene IDs, complete target sequences and individual details for all baits used.

**Supplementary Data 2:** Details for all cultivars

**Supplementary Data 3:** Sequencing lengths, depths and homeologue specificity

**Supplementary Data 4:** Hexaploid haplotypes

**Supplementary Data 5:** Shared haplotypes between “ancestral” and hexaploids

**Supplementary Data 6:** Deletions, TFBS, TEs

**Supplementary Data 7:** Genes on Chr3A refseq1.0vs2.0
