# Supplementary material for: Exploring the diversity of promoter and 5′UTR sequences in ancestral, historic and modern wheat
Source: Plant Biotechnol J. 2021 Sep 16;19(12):2469–87. doi: 10.1111/pbi.13672 (PMC8633512; doi:10.1111/pbi.13672)
Supplement: Supplementary file 12 — Data S2 Details for all cultivars. [file PBI-19-2469-s004.pdf]

species	cultivar [origin]	abbreviation	genome	Source	Source code/contact	Country of Origin	Synonyms	BREEDER	PEDIGREE
Ae.peregrina	*Ae.peregrina (variabilis)*	APG	$U^pU^pS^pS^p$	NIAB	Kay Trafford			wild grass	n/a
Ae.speltoides	*Not known*	ASP	SS	RRes	2140022			wild grass - diploid (S) related to hexaploid wh	n/a
Ae.tauschii	ENT-228	ENT	DD	RRes	Lesley Smart			wild grass - diploid D genome contributor to h	n/a
T. durum	Kronos	KR	AABB	GRU	W10282	USA	Control (WT) line to W10281.	Arizona Plant Breeders	APS MSFS POP Se1 (D03-21)
T.monococcum	MDR031	M031	A^mA^m	VIR	MDR031	Turkey		originally landraces collected by VIR, genetical	n/a
T.monococcum	MDR037	M037	A^mA^m	VIR	MDR037	Armenia		originally landraces collected by VIR, genetical	n/a
T.monococcum	MDR043	M043	A^mA^m	VIR	MDR043	Greece		originally landraces collected by VIR, genetical	n/a
T.monococcum	MDR045	M045	A^mA^m	VIR	MDR045	Denmark		originally landraces collected by VIR, genetical	n/a
T.monococcum	MDR046	M046	A^mA^m	VIR	MDR046	Romania		originally landraces collected by VIR, genetical	n/a
T.monococcum	MDR049	M049	A^mA^m	VIR	MDR049	Iran		originally landraces collected by VIR, genetical	n/a
T.monococcum	MDR308 (= DV92)	M308	A^mA^m	Dubcovsky Lab, UC Davis, CA	MDR308	Italy	DV92	Landrace	n/a
T.monococcum	MDR657	M657	A^mA^m	MPIZ	MDR657	Turkey		Landrace	n/a
T. aestivum	Abbot*	AB	AABBDD	GRU	W9471	GBE	CWW 93/12	PBI Cambridge Ltd	(Avalon*Brimstone)*Torfrida 'sib'
T. aestivum	Alcedo	AL	AABBDD	GRU	WBCDB0001	DEU	W2192	Bohnshaven-Lanstein	(Rekord*Poros)*Carsten 8
T. aestivum	Ambrosia*	AM	AABBDD	GRU	W10056	GBR	PBI-00-52	PBI Cambridge Ltd	(Cantata-sib*Genesis)*Pinder
T. aestivum	Avalon	AV	AABBDD	GRU	WBCDB0004	GBR	W2564	PBI Cambridge Ltd	TJB 30/148*TL 365a/34/5
T. aestivum	Badger	BA	AABBDD	GRU	WBCDB0006	GBR		Syngenta Seeds UK	Stephens3*/SF4//Stephens/3/ORATORIO
T. aestivum	Bobwhite	BW	AABBDD	GRU	WBCDB0010	MEX		CIMMYT	AVRORA//KALYANSONA/BLUEBIRD/3/(SIB)WOODPECKER
T. aestivum	Brompton*	BR	AABBDD	GRU	WBCDB0011	GBR	W10059	Elsoms Seeds Ltd	CWW 92.1*Caxton
T. aestivum	Buster	BU	AABBDD	GRU	WBCDB0012	GBR	W9367	Nickerson RPB Ltd	Brimstone*Parade
T. aestivum	Cadenza	CA	AABBDD	GRU	W9368		CPB.W1	Cambridge Plant Breeders	Axona*Tonic
T. aestivum	Cellule	CE	AABBDD	Desprez (FRA)		FRA		Desprez	Nogal x Almirante.
T. aestivum	Charger*	CH	AABBDD	GRU	WBCDB0016	GBR	W9474	PBI Cambridge Ltd	Fresco 'sib'*Mandate
T. aestivum	Chinese Spring	CS	AABBDD	GRU	W9493	CHN		Landrace	A Chinese landrace
T. aestivum	Claire*	CL	AABBDD	GRU	WBCDB0019	GBR	W9914	NSL Woolpit	Wasp*Flame
T. aestivum	Coppadra	CP	AABBDD	INRA-BRC	DPG_316	TUR			
T. aestivum	Cordiale*	CO	AABBDD	GRU	W10003	GBE	CPBT W83	CPB Twyfords	(Reaper*Cadenza)*Malacca
T. aestivum	Cougar*	CG	AABBDD	GRU	W10085	GBR	RW 40967	RAGT Seeds Ltd - Essex	Tuscan*Robigus
T. aestivum	Crusoe*	CR	AABBDD	GRU	W10080	GBR	NAWW25	Nickerson (UK) Ltd.	Cordiale*Gulliver
T. aestivum	Dickens*	DI	AABBDD	GRU	W10092	GBR	SC WW 071195	Secobra	(Equinox x Charger) x Defender
T. aestivum	Einstein*	EI	AABBDD	GRU	WBCDB0020	GBR	W10027	Nickerson (UK) Ltd.	(NHC 49*UK Yield Bulk)*(Haven*(Moulin x Galahad)
T. aestivum	Fielder*	FI	AABBDD	GRU	W10146	USA	CWW 99/19, ID-0044	Dr. Sunderman, University of Idaho	
T. aestivum	Flanders	FL	AABBDD	GRU	W2128	FRA	FD 6412/200	Elsoms Seeds Ltd	Champlein*FD 2816-348
T. aestivum	Gallant*	GA	AABBDD	GRU	W10042	GBR	NFC 10563	New Farm Crops (Syngenta)	(Malacca*Charger)*Xi-19
T. aestivum	Garcia	GC	AABBDD	Clare Lister (JIC)	R12P2_Gar#178	SPAIN		Secobra	
T. aestivum	Gatsby	GT	AABBDD	GRU	WBCDB0024	GBR	W10063	Zeneca Seeds Ltd	Nelson*Wasmo
T. aestivum	Gladiator*	GL	AABBDD	GRU	W10017	GBR	CWW 00/33 1/442	RAGT Seeds Ltd - Essex	Falstaff*Shannon
T. aestivum	Graham	GR	AABBDD	Syngenta UK ltd.	H 2015			Syngenta Seeds UK	Expert x Premio
T. aestivum	Hereford*	HF	AABBDD	GRU	WBCDB0027	GBR		Syngenta Seeds UK	Solist x Deben
T. aestivum	Hereward	HW	AABBDD	GRU	WBCDB0028	GBR	W9448	PBI Cambridge Ltd	Norman "Sib"*Disponent
T. aestivum	Hobbit	HO	AABBDD	GRU	WBCDB0029	GBR	W1578	PBI Cambridge Ltd	((CI 12633*(Cappelle)5)*(Cappelle*H 110)*Prof Marchal)*((Marne*VG 9144)*Nord D.)
T. aestivum	Hustler	HU	AABBDD	GRU	W1568	GBE	TJB 368/268/1024/6592	PBI Cambridge Ltd	Maris Huntsman*TL 365a/25(=Durin)
T. aestivum	Isengrain	IS	AABBDD	GRU	W10008	GBR		Desprez	Apollo*Soissons
T. aestivum	Istabraq*	IQ	AABBDD	Limagrain	Ed Flatman	GBR		Limagrain	Consort*Claire
T. aestivum	JB Diego	JB	AABBDD	RAGT	Ruth Bryant	GBR		RAGT Seeds Ltd - Essex	STR-2374*3351-B-2
T. aestivum	KWS Santiago*	KSA	AABBDD	KWS-UK	GRU	GBR	W10338	KWS	Sherborne x Oakley
T. aestivum	KWS Silverstone*	KSL	AABBDD	KWS-UK	Jacob Lage	GBR		KWS	KWS Sterling x JB Diego
T. aestivum	KWS Siskin*	KSI	AABBDD	KWS-UK	Jacob Lage	GBR		KWS	KWS Sterling x CPBT W134
T. aestivum	KWS Trinity*	KTR	AABBDD	KWS-UK	Jacob Lage	GBR		KWS	(Grafton x Einstein) x CPBT W134
T. aestivum	Malacca*	MA	AABBDD	GRU	WBCDB0035	GBR	W9481	CPB Twyfords	Riband*(Rendezvous)*Apostle
T. aestivum	Maris Huntsman	MH	AABBDD	GRU	W0730	GBE	TJB 32/218B	PBI Cambridge Ltd	((CI 12633*(Cappelle Desprez)5)*Hybrid 46)*Professeur Marchal
T. aestivum	Maris Widgeon	MW	AABBDD	GRU	W4114	GBE	Recommended list in 1971.<br>TB 106/40	PBI Cambridge Ltd	Holdfast*Cappelle Desprez
T. aestivum	Marksman*	MK	AABBDD	GRU	W10010	GBR	PBI-40447	VEB Berlin	98ST08*Aardvark
T. aestivum	Mercia	ME	AABBDD	GRU	W9333	GBE	D28-7	PBI Cambridge Ltd	(Talent*Virtue)*Flanders
T. aestivum	Napier	NA	AABBDD	GRU	W9906	GBE	CWW 96-17	PBI Cambridge Ltd	Hussar*Lynx
T. aestivum	Oakley	OA	AABBDD	GRU	WBCDB0037	GBR	W10000	CPB Twyfords	(Aardvark(sib)*Robigus)*Access
T. aestivum	Paragon	PA	AABBDD	GRU	WBCDB0040	GBR	W10074	AAFC-Brandon, Cananda	Axona*Tonic
T. aestivum	Piko	PI	AABBDD	GRU	W10025	GER			
T. aestivum	Reflection	RF	AABBDD	GRU	W10279	GBE		Syngenta Seeds UK	Denman x Oakley
T. aestivum	Relay	RL	AABBDD	GRU	W10278	GBE		RAGT Seeds Ltd - Essex	Gladiator x Vector
T. aestivum	Revelation	RV	AABBDD	GRU	W10190	GBE	NAWW39	Limagrain UK Ltd	(Alchemy x Claire) x Shepherd
T. aestivum	Rialto	RI	AABBDD	GRU	WBCDB0044	GBR	W9430	PBI Cambridge Ltd	Haven'S'*Fresco'S'
T. aestivum	Riband	RB	AABBDD	GRU	WBCDB0045	GBR	W5552	PBI Cambridge Ltd	Norman*(Maris Huntsman*TW 161)
T. aestivum	Robigus	RO	AABBDD	GRU	WBCDB0046	GBR	W9999	CPB Twyfords	2836*1366
T. aestivum	Savannah	SA	AABBDD	GRU	WBCDB0047	GBR	W9485	Zeneca Seeds Ltd	Riband*Brigadier
T. aestivum	Scout	SC	AABBDD	GRU	W10043	GBR	SWWC 503	SW Seeds Ltd - Great Abingdon	Z435*Deben
T. aestivum	Sears Synthetic	SS	AABBDD	JIC	Clare Lister			Dr. E Sears	synthetic wheat
T. aestivum	Skyfall	SF	AABBDD	GRU	W10198	GBE	S.J3326	RAGT Seeds Ltd - Essex	C4148 x Hurricane
T. aestivum	Soisson	SO	AABBDD	GRU	WBCDB0050	FRA	W9543	Desprez	Jena*HN35
T. aestivum	Solstice	SL	AABBDD	GRU	W10012	GBR	A18-98	Zeneca Seeds Ltd	Vivant*Rialto
T. aestivum	Spark	SP	AABBDD	GRU	WBCDB0052	GBR	W9442	Nickerson RPB Ltd	Moulin*Tonic
T. aestivum	Stigg	ST	AABBDD	GRU	W10052	GBR	NAWW19	Nickerson (UK) Ltd.	(Biscay*Septoria resistant line)*Tanker
T. aestivum	Sumai 3	SU	AABBDD	RAGT, now GRU	Ruth Bryant, now W9545	CHN		China	Funo x Taiwanxiaomai
T. aestivum	Taichung 29	TA	AABBDD	GRIN	PI 228069 (GRIN)	Taiwan			selection from landrace Chingtao Shantung
T. aestivum	Ukrainka	UK	AABBDD	GRU	W0473	SUN		Vavilov Inst., Russia	Banatka 1915 Selection
T. aestivum	USU-Apogee	AP	AABBDD	GRU	W10285	USA		Utah State University of Agriculture and Applie	Parula*Super Dwarf
T. aestivum	Valoris	VA	AABBDD	GRU	W10007	FRA		INRA-Clermont	(VM-173*OF-1851)*(c-1616*Revan)
T. aestivum	Veranopolis	VE	AABBDD	GRU	W5645	BRA		Roseworthy Agri Col	Trintecinco*B 2017-37
T. aestivum	Watkins 115	W115	AABBDD	JIC	Watkins 1190115	Yugoslavia		n/a	Landrace
T. aestivum	Watkins 141	W141	AABBDD	JIC	Watkins 1190141	France		n/a	Landrace
T. aestivum	Watkins 160	W160	AABBDD	JIC	Watkins 1190160	Spain		n/a	Landrace
T. aestivum	Watkins 199	W199	AABBDD	JIC	Watkins 1190199	India		n/a	Landrace
T. aestivum	Watkins 203	W203	AABBDD	JIC	Watkins 1190203	India		n/a	Landrace
T. aestivum	Watkins 209	W209	AABBDD	JIC	Watkins 1190209	Iran		n/a	Landrace
T. aestivum	Watkins 239	W239	AABBDD	JIC	Watkins 1190239			n/a	Landrace
T. aestivum	Watkins 246	W246	AABBDD	JIC	Watkins 1190246	India		n/a	Landrace
T. aestivum	Watkins 292	W292	AABBDD	JIC	Watkins 1190292	Cyprus		n/a	Landrace
T. aestivum	Watkins 387	W387	AABBDD	JIC	Watkins 1190387	Spain		n/a	Landrace
T. aestivum	Watkins 579	W579	AABBDD	JIC	Watkins 1190579	Iran		n/a	Landrace
T. aestivum	Watkins 624	W624	AABBDD	JIC	Watkins 1190624	Bulgaria		n/a	Landrace
T. aestivum	Watkins 733	W733	AABBDD	JIC	Watkins 1190733	Iran		n/a	Landrace
T. aestivum	Watkins 777	W777	AABBDD	JIC	Watkins 1190777	Finland		n/a	Landrace
T. aestivum	Watkins 786	W786	AABBDD	JIC	Watkins 1190786	USSR		n/a	Landrace
T. aestivum	Xi19	XI	AABBDD	GRU	W10072	GBR	A19-98	Limagrain UK Ltd	(Cadenza*Rialto)*Cadenza
T. aestivum	Yumai 34	YU	AABBDD	RRes	Alison Lovegrove	China			AIFENG-3//MENGXIAN-201/NEUZUCHT/3/YUMAI-2
T. aestivum	Zebedee	ZE	AABBDD	GRU	W10073	GBR	A45-02	Zeneca Seeds Ltd	Claire*Nelson

VIR - Vavilov Research Institute of Plant Science, St.Petersburg, Russia
JIC - John Innes Centre, Norwich, GBR
GRU - Germplasm Resource Unit, John Innes Centre, Norwich, GBR
RRes - Rothamsted Research, Harpenden, GBR
GRIN - Germplasm Resources Information Network https://www.ars-grin.gov/
MPIZ - Max Plank Institut fuer Zuechtungsforschung, Germany
INRA-BRC - Institut National de la Recherche Agronomique - Biological Resources Center, France
