# Supplementary material for: Exploring the diversity of promoter and 5′UTR sequences in ancestral, historic and modern wheat
Source: Plant Biotechnol J. 2021 Sep 16;19(12):2469–87. doi: 10.1111/pbi.13672 (PMC8633512; doi:10.1111/pbi.13672)
Supplement: Supplementary file 15 — Data S5 Shared haplotypes between “ancestral” and hexaploids. [file PBI-19-2469-s015.pdf]

included here are all genes for which at least 1 haplotype observed in diploid or tetraploid cultivars is also present in Watkins landraces and/or commercial cultivars	
**sheet "all dip&tet"**	
column A	**Gene ID**: trait category, gene number and homoeologue
column B	**IWGSCrefseq1.1 gene ID**
column C	**diploid/tetraploid species**: 8 *Triticum monoccoccum* accessions (M031-M657), *Aegilops tauschii* ENT), *Triticum durum* Kronos (KR)
column D	**landraces**: details which of the 14 Watkins lines have this haplotype
column E	**commercials**: details which of the 69 lines have this haplotype, abbreviations as in Supplementary Table 1
**sheet "Tmon only"**	please note: genes are sorted here by chromosome location
column A	**Gene ID**: trait category, gene number and homoeologue
column B	**description**: short name of gene
column C	**IWGSCrefseq1.1 gene ID**
column D	**T.mon accessions**: details of which Tmon accessions have a haplotype shared with commercials. For some genes there are more than 1 shared Tmon haplotype
column E	**landraces**: details which of the 14 Watkins lines has this haplotype
column F	**commercials**: details which of the 69 lines has this haplotype, abbreviations as in Supplementary Table 1
column G	**comments**: anything noteworthy

Gene ID	IWGSCrefseq1.1	diploid/ tetraploid species	Landraces	Commercial	
**T1-20A**	TraesCS1A02G083000	KR	W115, W209, W292, W387, W777	0	
**T1-24B**	TraesCS6B02G266200	KR	W160, W777	45	AB, AL, AM, AV, BA, CA, CE, CH, CO, CG, CR, DI, FL, GA, GR, HF, HO, HU, IS, IQ, JB, KSA, KSL, KSI, KTR, MA, MH, MW, MK, ME, NA, OA, P1, RL, RV, RB, RO, SA, SC, SO, SP, ST, VA, VE, XI
**T1-25A**	TraesCS1A02G102400	KR	W115, W199, W209, W292, W387	2	XI, YU
**T1-28A**	TraesCS3A02G021900	KR		13	BW, CE, CO, CR, GR, KSA, KSI, MA, MH, RB, SA, ST, UK
**T1-28B**	TraesCS2B02G566500	KR	W115, W141, W160, W209	4	BW, CP, VE, YU
**T2-2A**	TraesCS5A02G247200	M037, M045, M657,	W292, W624	9	AL, BW, GC, HU, RB, SA, SS, TA, UK
**T2-12A**	TraesCS7A02G441400	all 8 T.mon	W387	6	CG, OA, PA, RE, RO, YU
**T2-13A**	TraesCS2A02G220400	KR	W115, W209, W292, W624, W733	29	AM, CL, CG, EI, GA, GR, HF, HO, IQ, MH, MW, MK, ME, OA, PI, RL, RV, RI, SF, SP, ST, SU, AP, VA, XI, YU, ZE
**T2-17B**	TraesCS3B02G262600	KR	**all apart from** W141, W246, W387, W786	72	**all apart from** AM, BR, CG, CR, FL, GA, OA, RB, SS, UK
**T2-20A**	TraesCS3A02G494800	all 8 T.mon	W141, W209, W246, W292, W387, W786	3	CP, SS, TA
**T2-43B**	TraesCS7B02G018600	KR	W115, W160, W209, W292, W733	22	AB, AL, AM, CO, FI, FL, GA, HU, IS, KSA, ME, NA, OA, PA, PI, RE, RV, SS, UK, VA, YU, ZE
**T2-46B**	TraesCS7B02G746900LC	KR	W141, W209, W387	7	CE, GC, GR, HW, MW, RE, YU
**T2-61B**	TraesCS6B02G210000	KR	W160, W199, W203, W209, W387, W579, W777	18	BR, CL, EI, FI, GT, HF, HW, PA, RE, RL, RI, SF, SL, UK, VA, VE, YU, ZE
**T3-3(7A)**	TraesCS7A02G040900	M037	W115, W199, W246, W579, W777	36	AL, BW, BU, CL, CO, CR, DI, EI, FL, GC, GT, GL, GR, HF, HO, HU, IQ, JB, KSL, KSI, KTR, MA, MH, ME, NA, PI, RV, RB, SA, SS, SO, SL, SU, AP, VA, VE
**T4-1A**	TraesCS7A02G264400	KR	W115	6	CO, GA, HW, HO, KSI, YU
**T4-10B**	TraesCS4B02G197800	KR	W203, W292, W579	37	AL, AM, AV, CA, DI, FI, GA, GC, HF, HO, HU, KSA, KSL, KSI, MA, MH, MW, MK, ME, NA, OA, PI, RL, RI, RB, RO, SA, SC, SF, SL, SP, ST, SU, UK, VE, XI, YU
**T4-18(7A)**	TraesCS7A02G009800	M043, M657, KR	none	33	AB, AM, AV, BA, BW, BR, CH, CL, CO, CG, CR, EI, GA, GT, GL, HW, HO, IS, IQ, KSL, KSI, KTR, MA, MW, MK, RL, RV, RI, SF, SL, SP, XI, ZE
**T4-19(7A)**	TraesCS7A02G009100	M031	none	2	GA, SL
**T4-19(7A)**	TraesCS7A02G009100	M043	none	32	AB, AM, AV, BA, BW, BR, CH, CL, CO, CG, EI, FI, GT, GL, HW, HO, IS, IQ, KSL, KSI, KTR, MA, MW, MK, PI, RL, RV, RI, SF, SP, XI, ZE
**T4-19(7A)**	TraesCS7A02G009100	M046, M657	none	1	CR
**T4-20(7A)**	TraesCS7A02G009200	MDR043	none	35	AB, AV, BA, BW ,BR, CH, CL, CO, CG, CR, EI, FI, GA, GT, GL, HW, HO, IS, IQ, KSL, KSI, KTR, MA, MW, MK, PI, RL, RV, RI, SF, SL, SP, XI, ZE
**T4-25A**	TraesCS4A02G097500	KR	W141, W209, W246, W292	1	CP
**T4-30A**	TraesCS5A02G545100	MDR031, M037, M049	none	1	SU
**T4-37**	TraesCS7A02G049400	M031, M037, M043, M045, M046, M049, M657	W115, W292	1	CO
**T4-37**	TraesCS7A02G049400	M308	W199, W246	1	BW
**T4-38B**	TraesCS3B02G144800	KR	**all apart from** W199, W292, W777	68	**all apart from** AV, BW, CL, GT, MA, MW, RE, RV, SS, ST, SU, AP, VE, ZE
**T4-47B**	TraesCS3B02G148100	KR	W141, W160, W203, W292, W387, W777	49	AB, AL, AM, AV, BR, BU, CE, CH, CL, CO, CG, CR, DI, EI, FI, FL, GA, GT, GL GR, HF, HO, HU, IS, JB, KSA, KSL, KTR, MA, MH, MW, ME, NA, OA, PI, RE, RL, RV, RB, RO, SA, SC, SS, SO, ST, TA, VA, YU, ZE
**T4-49A**	TraesCS1A02G338700	KR	W141, W160, W209, W387, W579, W786	1	AP
**T4-57D**	TraesCS3D02G209200	ENT	**all Watkins**	65	**all apart from** AB, CP, CO, DI, EI, FL, GA, HU, IS, IQ, JB, KSL, MA, RB, SA, SS, YU
**T5-2B**	TraesCS3B02G115400	KR	W115, W141, W160, W203, W209, W292, W387, W777, W786	62	**all apart from** BA, CA, CP, GC, HW, IS, IQ, KSI, MK, PA, RV, RI, SF, SO, SL, SP, SU, UK, AP, XI
**T5-10A**	TraesCS5A02G558200	M037, M045, M046, M657	W624	49	AB, AM, AV, BR, CH, CL, CO, CG, DI, EI, FL, GC, GL, GR, HF, HW, HU, IQ, KSA, KSI, MA, MH, MW, ME, NA, RL, RV, RB, SA, SC, SP, SU, ZE
**T5-10A**	TraesCS5A02G558200		W203, W777, W786	18	AL, BA, BU, CA, FI, GA, GT, JB, KSL, MK, OA, PA, PI, RE, RI, RO, SL, XI
**T5-14A**	TraesCS7A02G412400	KR	W115, W141, W160, W777	3	AL, PA, UK,
**T5-23A**	TraesCS5A02G384100	KR	W115, W141, W160, W203, W209, W292, W387, W579, W624	62	AB, AL, BU, CH, CO, CR, DI, GA, GT, HW, JB, KSA, KSL, KSI, MA, MH, NA, PI, SS, ZE
**T5-23B**	TraesCS5A02G384100	KR	W733	3	TA, AP, VE
**T5-29B**	TraesCS3B02G139100	KR	**all apart from** W199, W733	76	**all apart from** IS, SS, SO, SU, AP, VE
**T6-1A**	TraesCS5A02G477600	KR	**all apart from** W624, w786	81	**all apart from** SS
**T6-3A**	TraesCS7A02G422500	KR	W199	5	GA, GT, RE, SF, SU
**T6-3B**	TraesCS7B02G322900	KR	W203	4	BR, IS, MW, VA
**T6-13A**	TraesCS6A02G298100	KR	W141, W160	4	GC, NA, OA, ZE
**T6-13B**	TraesCS6B02G327500	KR	W115, W141, W160, W203	2	RL, UK
**T6-15A**	TraesCS6A02G031100	M045, M657	NONE	2	HF, AL
**T6-16B**	TraesCS6B02G044000	KR	W624, W777	0	
**T6-17A**	TraesCS6A02G031000	all 8 T.mon	W579	2	AL, HF
**T6-18A**	TraesCS6A02G030700	M037, M043	W203, W292	55	AB, AM, AV, BA, BU, CA, CH, CL, CP, CO, DI, EI, FI, GA, GC, GT, GL, GR, HW, HO, HU, IQ, JB, KSL, KSI, KTR, MA, MH, MW, MK, ME, NA, PA, PI, RV, RI, RB, SA, SF, SL, SP, UK, VA, XI, ZE
**T6-18A**	TraesCS6A02G030700	M045, M049	W579	1	AL
**T8-1B**	TraesCS3B02G217100	KR	**all apart from** W199, W246, W579, W733	70	**all apart from** BW, GR, HO, JB, KSL, SC, SS, SU, TA, VE, XI, ZE
**T8-3A**	TraesCS5A02G233600	KR	W115, W203, W387, W579	60	AM, BA, BW, CA, CR, GA, GC, GT, GL, HU, JB, KSA, MK, NA, OA, PA, RB, SA, TA, UK, VE, XI
**T8-7A**	TraesCS6A02G071200	all 8 T.mon		5	BW, EI, SC, SP, VE
**T8-8B**	TraesCS5B02G396700	KR	**all apart from** W115, W203, W733	77	**all apart from** GC, GR, SS, SF, TA
**T8-11A**	TraesCS1A02G199600	KR	W115, W199, W203, W209, W292, W387	75	**all apart from** CP, GT, PA, SS, TA, UK, YU
**T8-12A**	TraesCS2A02G443100	all 8 T.mon		3	IS, SO, AP
**T8-12A**	TraesCS2A02G443100	KR	**all apart from** W199, W387, W579,	27	AL, AV, BA, BR, BU, CA, CE, DI, EI, FI, GT, GR, HU, JB, MH, MW, ME, PA, RI, SS, SF, SL, SP, VE, XI, YU, ZE
**T8-12D**	TraesCS2D02G442200	ENT	all Watkins	80	**all apart from** SS, UK
**T8-19A**	TraesCS3A02G093200	KR	**all apart from** W246, W786	73	**all apart from** BA, BW, CA, KSA, PA, SP, UK, XI
**T8-19B**	TraesCS3B02G108500	KR	W624, W777	9	BA, CA, CP, PA, SS, SP, SU, TA, YU
**T8-23B**	TraesCS6B02G286400	KR	W387, W579, W624	8	BW, CP, MA, MK, NA, RV, AP, VE
**T9-4A**	TraesCS1A02G113400	KR	W115, W209, W292, W387, W733	7	AB, AV, FL, MW, ME, RB, SS,
**T9-10A**	TraesCS7A02G492400	KR	W115, W141, W160, W209, W786	35	AB, AM, BR, BU, CL, CP, CR, DI, EI, GT, GL, HF, HW, HU, IS, IQ, JB, KSA, KTR, MA, MK, NA, OA, RE, RO, SA, SC, SO, SL, ST, SU, TA, AP, VA, YU
**T9-15A**	TraesCS7A02G415300	KR	W115, W141, W777	3	AL, PA, UK
**T9-15B**	TraesCS7B02G315200	KR	W115, W199, W203, W209, W292, W387, W624, W777, W786	59	AL, BU, CA, CO, CG, GA, GC, GT, IS, KSL, KSI, KTR, MA, PA, PI, RB, RO, SC, SO, SP, TA, UK, XI
**T9-15D**	TraesCS7D02G408500	ENT	**all apart from** W199, W246	81	**all apart from** SS
**T9-20A**	TraesCS2A02G192600	KR	W115, W141, W209, W292, W777	2	CP, UK
**T9-20B**	TraesCS2B02G214600	KR	**all apart from** W160, W199, W246, W579, W733, W786	46	AL, AM, BA, BW, BR, CA, CH, CL, CP, CO, CR, DI, EI, FL, GA, GC, GT, GL, HW, HO, IQ, KSA, KSL, KSI, KTR, MK, OA, PA, RE, RV, RI, RB, RO, SA, SC, SF, SO, SL, SP, ST, UK, AP, VA, VE, XI, ZE
**T9-32B**	TraesCS6B02G252000	KR	W141, W387, W777	0	
**T9-38A**	TraesCS6A02G024300	M043	NONE	5	CA, GA, TA, UK, AP
		M045, M046	W115, W160, W209, W292, W777	5	AL, BR, PA, SP, YU
**T9-40A**	TraesCS2A02G230100	KR	**all apart from** W199, W246	75	**all apart from** BA, CA, KTR, PA, RB, SA, VE
**T9-42B**	TraesCS2B02G489800	KR	W199, W246, W579, W786	3	CE, IS, SO
**T9-44B**	TraesCS3B02G314000	KR	W155	6	AV, GA, KSI, MA, MW, ME
**T9-46B**	TraesCS5B02G446100	KR	W579	35	AM, BA, BR, CA, CH, CL, CG, CR, DI, EI, FI, GL, HW, HO, HU, IS, IQ, JB, KSL, KSI, KTR, MH, MK, PA, RL, RV, RI, SC, SF, SL, SP, ST, VA, XI, ZE
**T9-48A**	TraesCS2A02G491000	KR	W115, W292, W387, W579, W777	13	AL, BR, CA, CP, CO, GA, KSA, KSL, KSI, MA, SU, YU, ZE
**T9-55B**	TraesCS7B02G084800LC	KR	W115, W141, W160, W209, W292, W579, W624, W777	6	BW, SS, SO, SU, VE, YU
**T9-56A**	TraesCS1A02G217900	KR	W115, W292, W387, W733	38	AM, CH, CL, CG, CR, DI, EI, FI, FL, GA, GC, GL, HF, HW, IS, IQ, KSA, KSL, KSI, KTR, MA, MK, ME, NA, OA, RE, RL, RV, RI, RO, SC, SS, SF, ST, AP, VA, VE, ZE
**T9-56B**	TraesCS1B02G231300	KR	W115, W160, W203, W292, W387, W624, W777	37	AB, AM, AV, BW, CE, CH, CR, DI, EI, FI, GC, GT, GL, HF, HW, HO, IS, IQ, KSL, KSI, MA, MW, MK, ME, NA, OA, PI, RE, RL, RV, RI, SA, SO, SL, SP, TA, ZE
**T9-57A**	TraesCS6A02G343300	KR	**all apart from** W733 (=KR +1SNP!)	79	**all apart from** KSI, SS, YU
**T9-57D**	TraesCS6D02G324200	ENT	**all apart from** W209 (=ENT +1SNP!)	77	**all apart from** CL, NA, SL, SU, YU
**T9-59B**	TraesCS7B02G062200	KR	W115, W160, W203, W579	49	AM, AV, BA, BR, BU, CE, CH, CG, CR, DI, EI, FI, FL, GA, GT, GL, GR, HF, HU, IS, IQ, JB, KSA, KSL, KTR, KSI, MA, MH, MW, MK, ME, NA, OA, PI, RE, RL, RV, RI, RB, RO, SA, SC, SS, SF, SO, SL, ST, VA, ZE
**T9-63A**	TraesCS5A02G477600	KR	**all apart from** W624, W786	81	**all apart from** SS
**T9-64A**	TraesCS7A02G422500	KR	W160	5	FL, GC, PI, SS, ST,
**T9-64B**	TraesCS7B02G322900	KR	W199	7	BA, CA, CG, OA, RE, RO, SP
**T10-7A**	TraesCS5A02G081400	KR	W115, W292, W387, W579, W733, W733, W786	67	**all apart from** AL, GC, GT, HW, IS, KSL, KSI, MW, PI, RE, RI, SF, SO, SU, UK,
**T10-9A**	TraesCS1A02G162200	KR	W115, W209, W292, W387, W733	1	SS,
**T10-10B**	TraesCS3B02G143800	KR	W141, W203, W292, W777	26	AB, AV, BA, CP, DI, EI, FL, GL, HF, HW, HO, HU, IS, KSA, MA, MK, NA, OA, PA, RE, RV, RB, RO, SC, SO, XI
**T10-32B**	TraesCS6B02G220900	KR	W199, W203, W209, W246, W387, W579, W733, W777, W786	13	BR, CL, CP, EI, FI, HF, HW, PA, RE, RI, SF, VA, YU

Gene	description	IWGSCrefseq1.1 ID	T.mon accessions	chromosome location	position (bp)	commercial cultivars	comments
**T8-12A**	LFY	TraesCS2A02G443100	all 8 T.mon	2A	693,231,203	IS, SO, AP	
**T2-20A**	TaGT61_6	TraesCS3A02G494800	all 8 T.mon	3A	721,085,862	CP, SS, TA	
**T2-2A**	PGI	TraesCS5A02G247200	M037, M045, M657,	5AL	461,487,242	AL, BW, GC, HU, RB, SA, SS, TA, UK	
**T4-30A**	TaGER4c	TraesCS5A02G545100	M031, M037, M649,	5AL	700,364,469	SU	
**T5-10A**	AK336250	TraesCS5A02G558200	M037, M045, M046, M657	5AL	708,809,223	AB, AM, AV, BR, CH, CL, CO, CG, DI, EI, FL, GC, GL, GR, HF, HW, HU, IQ, KSA, KSI, MA, MH, MW, ME, NA, RL, RV, RB, SA, SC, SP, SU, ZE	
**T9-38A**	SGN1	TraesCS6A02G024300	M043	6AS	12,119,995	CA, GA, TA, UK, AP	
	SGN1		M045, M046	6AS	12,119,995	AL, BR, PA, SP, YU	
**T6-17A**	TaNRT2.3	TraesCS6A02G031000	all 8 T.mon	6AS	15,756,654	AL, HF	
**T6-15A**	TaNRT2.1	TraesCS6A02G031100	M045, M657	6AS	15,765,983	AL, HF	
**T6-18A**	TaNRT2.4	TraesCS6A02G030700	M037, M043	6AS	15,727,844	AB, AM, AV, BA, BU, CA, CH, CL, CP, CO, DI, EI, FI, GA, GC, GT, GL, GR, HW, HO, HU, IQ, JB, KSL, KSI, KTR, MA, MH, MW, MK, ME, NA, PA, PI, RV, RI, RB, SA, SF, SL, SP, UK, VA, XI, ZE	2SNPs only
	TaNRT2.4	TraesCS6A02G030700	M045, M049	6AS	15,727,844	AL	1SNP only: M037 -1
**T8-7A**	bHLH protein	TraesCS6A02G071200	all 8 T.mon	6AS	39,049,342	BW, EI, SC, SP, VE	
**T2-12A**	TaGT43_1	TraesCS7A02G441400	all 8 T.mon	7AL	635,497,069	CG, OA, PA, RE, RO, YU	
**T3-3(7A)**	Sucrose synthase	TraesCS7A02G040900	M037	7AS	19,003,197	AL, BW, BU, CL, CO, CR, DI, EI, FL, GC, GT, GL, GR, HF, HO, HU, IQ, JB, KSL, KSI, KTR, MA, MH, ME, NA, PI, RV, RB, SA, SS, SO, SL, SU, AP, VA, VE	
**T4-37**	MLOC_54592	TraesCS7A02G049400	M031, M037, M043, M045, M046, M049, M657	7AS	22,976,922	CO	
			M308			BW	
**T4-18(7A)**	Ta6-SFT	TraesCS7A02G009800	M043, M657	7AS	4,433,162	AB, AM, AV, BA, BW, BR, CH, CL, CO, CG, CR, EI, FI, GA, GT, GL, HW, HO, IS, IQ, KSL, KSI, KTR, MA, MW, MK, RL, RV, RI, SF, SL, SP, XI, ZE	
**T4-19(7A)**	Ta1-SST	TraesCS7A02G009100	M031	7AS	3,986,563	GA, SL	all 3 T4-19A(7A) haplotypes very similar
			M046, M657	7AS	3,986,563	CR	
			M043	7AS	3,986,563	AB, AM, AV, BA, BW, BR, CH, CL, CO, CG, EI, FI, GT, GL, HW, HO, IS, IQ, KSL, KSI, KTR, MA, MW, MK, PI, RL, RV, RI, SF, SP, XI, ZE	
**T4-20(7A)**	Ta1-FFT	TraesCS7A02G009200	MDR049	7AS	4,017,160	AB, AM, AV, BA, BW ,BR, CH, CL, CO, CG, CR, EI, FI, GA, GT, GL, HW, HO, IS, IQ, KSL, KSI, KTR, MA, MW, MK, PI, RL, RV, RI, SF, SL, SP, XI, ZE	
