# Supplementary material for: Exploring the diversity of promoter and 5′UTR sequences in ancestral, historic and modern wheat
Source: Plant Biotechnol J. 2021 Sep 16;19(12):2469–87. doi: 10.1111/pbi.13672 (PMC8633512; doi:10.1111/pbi.13672)
Supplement: Supplementary file 17 — Data S7 Genes on Chr3A refseq1.0 vs 2.0. [file PBI-19-2469-s008.pdf]

**Trait gene**		**e.g. T5-28**	gene 28 of trait category 5 (Supplementary Data 1)
**Gene name**		**TaGT61-21**	
**IWGSC CSrefseq1.1 ID**		**TraesCS3A02G021800**	This gene ID can be directly searched in IGV and KnetMiner
**CSrefseq1.0 target sequence**	**Start**	**e.g. 12,436,992**	Start position of target sequence (promoter & 5'UTR) from top (short arm) of chromosome
	**End**	**e.g. 12,438,691**	End position of target sequence from top (short arm) of chromosome
**CSrefseq2.0 target sequence identity**	**IDENTITY to refseq1.0**	**e.g. 100%**	100%=complete identity of target sequence between refseq1.0 and 2.0, any divergent genes marked in orange
	**Start**	**e.g. 12,618,133**	Start position of target sequence (promoter & 5'UTR) from top (short arm) of chromosome
	**End**	**e.g. 12,619,832**	End position of target sequence from top (short arm) of chromosome
	**Comments**		Details of discrepancies
	**Refseq1.0 start/total length**	**e.g. 0.0165**	Relative position of each gene (gene position/chr3A length)
	**Refseq2.0 start/total length**	**e.g. 0.0165**	Relative position of each gene (gene position/chr3A length)
	**∆(K-L)**	**e.g. 0.0000**	**Change in gene location**: Difference in relative position of each gene; 0.0000=near identical position, >0.1=substantial relocation of gene
**Sheet2**	**All Chr3A genes(Chr position)**		All Chr3A residing genes listed by chromosome position (top of short arm to bottom of long arm)
**Sheet3**	**All Chr3A genes (by trait)**		All Chr3A residing genes listed by trait category

Trait gene	Gene name	IWGSC CSrefseq1.1 ID	CSrefseq1.0 target sequence		CSrefseq2.0 target sequence identity						
			Start	End	IDENTITY to refseq1.0	Start	End	Comments	Refseq1.0 start/total length	Refseq2.0 start/total length	∆(K-L)
T5-28	TaGT61-21	TraesCS3A02G021800	12,436,992	12,438,691	100%	12,618,133	12,619,832		0.0166	0.0165	0.0000
T1-28	Brassinosteroids	TraesCS3A02G021900	12,477,540	12,479,239	100%	12,658,166	12,659,865		0.0166	0.0166	0.0000
T2-9	pGlcT	TraesCS3A02G036600	20,014,671	20,016,370	100%	20,657,492	20,659,191		0.0267	0.0270	-0.0004
T4-4	STB6	TraesCS3A02G049500	26,199,975	26,201,674	100%	26,821,179	26,822,878		0.0349	0.0351	-0.0002
T7-21	GID2	TraesCS3A02G056000	32,253,038	32,254,737	100%	32,947,770	32,949,469		0.0430	0.0431	-0.0002
T6-25	TaASN2	TraesCS3A02G077100	47,847,084	47,847,919	100%	48,228,334	48,229,169		0.0637	0.0631	0.0006
T8-19	VRS4	TraesCS3A02G093200	59,530,466	59,532,165	93%	47,579,699	47,581,397	T8-19A on Chr3A_v2_IWGSC_refseq Length=754109222<br><br>Score = 2533 bits (2808), Expect = 0.0 Identities = 1617/1748 (93%), Gaps = 77/1748 (4%) Strand=Plus/Plus	0.0793	0.0623	0.0170
T5-2	DREB1	TraesCS3A02G099200	64,029,825	64,031,524	100%	64,411,885	64,413,584		0.0853	0.0843	0.0010
T7-57	BRI1-like protein 2	TraesCS3A02G099400	64,279,733	64,281,432	100%	64,662,717	64,664,416		0.0856	0.0847	0.0010
T4-14	TaNH1	TraesCS3A02G105400	69,289,051	69,290,750	100%	69,647,104	69,648,803		0.0923	0.0912	0.0011
T6-38	Myb_TF_APL	TraesCS3A02G105500	69,492,410	69,493,458	99%	69,849,496	69,850,543	Deletion of a single A at pos885 of target sequence	0.0926	0.0915	0.0011
T2-14	TaBAHD1	TraesCS3A02G111400	78,903,053	78,904,752	100%	79,284,479	79,286,178		0.1051	0.1038	0.0013
T7-35	TaGI	TraesCS3A02G116300	84,188,436	84,190,135	100%	84,573,271	84,574,970		0.1122	0.1107	0.0014
T5-29	TaBAHD2	TraesCS3A02G119500	93,042,874	93,044,573	100%	93,478,024	93,479,723		0.1240	0.1224	0.0016
T7-26	GA3ox2	TraesCS3A02G122600	97,971,305	97,973,004	100%	98,377,695	98,379,394		0.1305	0.1288	0.0017
T10-10	COM1	TraesCS3A02G124100	100,186,468	100,188,167	100%	100,584,025	100,585,724		0.1335	0.1317	0.0018
T4-38	MLOC_55516	TraesCS3A02G125200	100,882,828	100,884,527	100%	101,273,773	101,275,472		0.1344	0.1326	0.0018
T4-47	MLOC_37050	TraesCS3A02G129000	106,440,787	106,442,486	99%	106,809,124	106,810,832	Insertion of 9bp (AATGGGGAA) at position 233 of target sequence	0.1418	0.1399	0.0019
T7-24	GA2ox5	TraesCS3A02G133400	110,330,977	110,332,676	100%	126,803,359	126,801,660		0.1470	0.1660	-0.0190
T9-37	LRR kinase (M8C3Z3)	TraesCS3A02G135400	112,951,165	112,952,864	100%	124,196,336	124,194,637		0.1505	0.1626	-0.0121
T7-2	FT2	TraesCS3A02G143100	124,171,960	124,172,880	100%	135,529,084	135,530,004		0.1654	0.1775	-0.0120
T10-24	FIDGETIN	TraesCS3A02G151300	140,056,409	140,058,108	100%	151,551,057	151,552,756		0.1866	0.1984	-0.0119
T7-41	Shaggy-like kinase 5	TraesCS3A02G164200	168,839,113	168,841,733	100%	180,360,881	180,363,501		0.2249	0.2362	-0.0112
T9-39	LRR kinase (M8C3Z3)	TraesCS3A02G167100	172,628,085	172,629,784	100%	184,295,627	184,297,326		0.2300	0.2413	-0.0113
T1-2	TaGS5-3A	TraesCS3A02G212900LC	176,554,257	176,555,956	100%	188,193,473	188,195,172		0.2352	0.2464	-0.0112
T8-1	Myb-TF	TraesCS3A02G187800	225,895,117	225,896,816	100%	237,713,492	237,715,191		0.3009	0.3113	-0.0103
T1-15	SRS3/ OsKINESIN-13A	TraesCS3A02G194100	269,226,808	269,228,507	100%	280,906,651	280,908,350		0.3587	0.3678	-0.0091
T10-43	p31 COMET	TraesCS3A02G198600	314,622,724	314,624,423	100%	326,078,193	326,079,892		0.4192	0.4270	-0.0078
T4-57	AK366042	TraesCS3A02G206400	363,414,282	363,415,982	100%	374,604,500	374,606,200		0.4842	0.4905	-0.0064
T2-16	TaBAHD3	TraesCS3A02G220800	408,949,426	408,951,125	100%	420,294,092	420,295,791		0.5448	0.5503	-0.0055
T4-9	TaSGT1	TraesCS3A02G347800LC	429,327,072	429,328,771	100%	434,614,148	434,612,449		0.5720	0.5691	0.0029
T7-34	Bri1	TraesCS3A02G245000	458,679,784	458,681,483	100%	470,135,375	470,137,074		0.6111	0.6156	-0.0045
T8-4	Myb-TF	TraesCS3A02G251200	470,830,018	470,831,717	100%	482,278,397	482,280,096		0.6273	0.6315	-0.0042
T6-71	PSTOL1	TraesCS3A02G261800	484,638,493	484,640,192	100%	496,077,524	496,079,223		0.6457	0.6496	-0.0039
T6-63	TaHRZ	TraesCS3A02G262700	485,675,716	485,677,415	100%	497,112,417	497,114,116		0.6470	0.6509	-0.0039
T6-23	Glutamate synthase (NADH), plastidic	TraesCS3A02G266300	490,928,004	490,929,703	100%	502,346,437	502,348,136		0.6540	0.6578	-0.0037
T4-43	MLOC_64975	TraesCS3A02G270800	498,227,479	498,229,178	100%	509,600,344	509,602,043		0.6638	0.6673	-0.0035
T9-44	RBOHF	TraesCS3A02G280200	509,428,096	509,429,795	100%	520,741,121	520,742,820		0.6787	0.6819	-0.0032
T6-30 & T8-16	SnRK	TraesCS3A02G282800	511,078,384	511,080,083	100%	522,381,101	522,382,800		0.6809	0.6840	-0.0031
T7-55	ETTIN = ARF3	TraesCS3A02G292400	522,277,033	522,278,732	100%	533,567,745	533,569,444		0.6958	0.6987	-0.0029
T7-22	GA2ox3	TraesCS3A02G294000	526,865,601	526,867,300	100%	538,137,523	538,139,222		0.7019	0.7046	-0.0027
T3-18	PGM	TraesCS3A02G306900	545,150,228	545,151,927	100%	539,997,054	539,995,355		0.7263	0.7071	0.0192
T1-4	TaCKX2	TraesCS3A02G311100	550,050,564	550,052,263	100%	112,920,863	112,922,562		0.7328	0.1479	0.5849
T7-56	FZP = ERFL1	TraesCS3A02G328000	573,110,750	573,112,449	100%	573,509,458	573,511,157		0.7635	0.7510	0.0126
T6-35	alpha-expansin TaEXPA2	TraesCS3A02G344800	594,490,493	594,492,192	100%	594,867,549	594,869,248		0.7920	0.7789	0.0131
T7-48	LAX	TraesCS3A02G350600	599,324,345	599,326,044	100%	599,713,788	599,715,487		0.7984	0.7853	0.0132
T3-26	Starch PPase	TraesCS3A02G366300	615,280,623	615,282,322	100%	615,588,924	615,590,623		0.8197	0.8060	0.0136
T3-26	Starch PPase	TraesCS3A02G366400	615,705,717	615,707,416	100%	616,013,303	616,015,002		0.8203	0.8066	0.0137
T2-29	PHOH	TraesCS3A02G366400	615,705,718	615,707,417	100%	616,013,304	616,015,003		0.8203	0.8066	0.0137
T10-22	XRCC2	TraesCS3A02G381500	631,717,710	631,718,673	100%	631,984,250	631,985,213		0.8416	0.8275	0.0141
T7-28	GA20ox3	TraesCS3A02G399800	646,682,530	646,684,229	100%	646,894,696	646,896,395		0.8615	0.8470	0.0145
T10-34	ZIP4	TraesCS3A02G401700	647,482,860	647,484,559	100%	647,679,005	647,680,704		0.8626	0.8481	0.0145
T2-4		TraesCS3A02G440800	683,253,427	683,255,811	100%	683,382,831	683,385,215		0.9103	0.8948	0.0154
T2-20	TaGT61_6	TraesCS3A02G494800	721,084,162	721,085,861	100%	721,711,109	721,712,808		0.9607	0.9450	0.0156
T3-19	PGM	TraesCS3A02G495800	721,584,182	721,585,881	100%	722,224,505	722,226,204		0.9613	0.9457	0.0156
T10-30	MLH1	TraesCS3A02G500600	724,882,306	724,884,005	100%	725,614,593	725,616,292		0.9657	0.9501	0.0156
T7-47	FIZZY-RELATED 4	TraesCS3A02G527600	741,974,618	741,976,317	100%	744,107,440	744,109,139		0.9885	0.9743	0.0142
Chr3A length	Refseq1.0		750,620,385								
	Refseq2.0		763,711,468								

Trait gene	Gene name	IWGSC CSrefseq1.1 ID	CSrefseq1.0 target sequence		CSrefseq2.0 target sequence identity			
			Start	End	IDENTITY to refseq1.0	Start	End	Comments
**T1-15**	SRS3/ OsKINESIN-13A	TraesCS3A02G194100	269,226,808	269,228,507	100%	280,906,651	280,908,350	
**T1-2**	TaGS5-3A	TraesCS3A02G212900LC	176,554,257	176,555,956	100%	188,193,473	188,195,172	
**T1-28**	Brassinosteroids	TraesCS3A02G021900	12,477,540	12,479,239	100%	12,658,166	12,659,865	
**T1-4**	TaCKX2	TraesCS3A02G311100	550,050,564	550,052,263	100%	112,920,863	112,922,562	
**T2-14**	TaBAHD1	TraesCS3A02G111400	78,903,053	78,904,752	100%	79,284,479	79,286,178	
**T2-16**	TaBAHD3	TraesCS3A02G220800	408,949,426	408,951,125	100%	420,294,092	420,295,791	
**T2-20**	TaGT61_6	TraesCS3A02G494800	721,084,162	721,085,861	100%	721,711,109	721,712,808	
**T2-29**	PHOH	TraesCS3A02G366400	615,705,718	615,707,417	100%	616,013,304	616,015,003	
**T2-4**		TraesCS3A02G440800	683,253,427	683,255,811	100%	683,382,831	683,385,215	
**T2-9**	pGlcT	TraesCS3A02G036600	20,014,671	20,016,370	100%	20,657,492	20,659,191	
**T3-18**	PGM	TraesCS3A02G306900	545,150,228	545,151,927	100%	539,997,054	539,995,355	
**T3-19**	PGM	TraesCS3A02G495800	721,584,182	721,585,881	100%	722,224,505	722,226,204	
**T3-26**	Starch PPase	TraesCS3A02G366300	615,280,623	615,282,322	100%	615,588,924	615,590,623	
**T3-26**	Starch PPase	TraesCS3A02G366400	615,705,717	615,707,416	100%	616,013,303	616,015,002	
**T4-14**	TaNH1	TraesCS3A02G105400	69,289,051	69,290,750	100%	69,647,104	69,648,803	
**T4-38**	MLOC_55516	TraesCS3A02G125200	100,882,828	100,884,527	100%	101,273,773	101,275,472	
**T4-4**	STB6	TraesCS3A02G049500	26,199,975	26,201,674	100%	26,821,179	26,822,878	
**T4-43**	MLOC_64975	TraesCS3A02G270800	498,227,479	498,229,178	100%	509,600,344	509,602,043	
**T4-47**	MLOC_37050	TraesCS3A02G129000	106,440,787	106,442,486	99%	106,809,124	106,810,832	Insertion of 9bp (AATGGGGAA) at position 233 of target sequence
**T4-57**	AK366042	TraesCS3A02G206400	363,414,282	363,415,982	100%	374,604,500	374,606,200	
**T4-9**	TaSGT1	TraesCS3A02G347800LC	429,327,072	429,328,771	100%	434,614,148	434,612,449	
**T5-2**	DREB1	TraesCS3A02G099200	64,029,825	64,031,524	100%	64,411,885	64,413,584	
**T5-28**	TaGT61-21	TraesCS3A02G021800	12,436,992	12,438,691	100%	12,618,133	12,619,832	
**T5-29**	TaBAHD2	TraesCS3A02G119500	93,042,874	93,044,573	100%	93,478,024	93,479,723	
**T6-23**	Glutamate synthase (NADH), plastidic	TraesCS3A02G266300	490,928,004	490,929,703	100%	502,346,437	502,348,136	
**T6-25**	TaASN2	TraesCS3A02G077100	47,847,084	47,847,919	100%	48,228,334	48,229,169	
**T6-30 & T8-16**	SnRK	TraesCS3A02G282800	511,078,384	511,080,083	100%	522,381,101	522,382,800	
**T6-35**	alpha-expansin TaEXPA2	TraesCS3A02G344800	594,490,493	594,492,192	100%	594,867,549	594,869,248	
**T6-38**	Myb_TF_APL	TraesCS3A02G105500	69,492,410	69,493,458	99%	69,849,496	69,850,543	Deletion of a single A at pos885 of target sequence
**T6-63**	TaHRZ	TraesCS3A02G262700	485,675,716	485,677,415	100%	497,112,417	497,114,116	
**T6-71**	PSTOL1	TraesCS3A02G261800	484,638,493	484,640,192	100%	496,077,524	496,079,223	
**T7-2**	FT2	TraesCS3A02G143100	124,171,960	124,172,880	100%	135,529,084	135,530,004	
**T7-21**	GID2	TraesCS3A02G056000	32,253,038	32,254,737	100%	32,947,770	32,949,469	
**T7-22**	GA2ox3	TraesCS3A02G294000	526,865,601	526,867,300	100%	538,137,523	538,139,222	
**T7-24**	GA2ox5	TraesCS3A02G133400	110,330,977	110,332,676	100%	126,803,359	126,801,660	
**T7-26**	GA3ox2	TraesCS3A02G122600	97,971,305	97,973,004	100%	98,377,695	98,379,394	
**T7-28**	GA20ox3	TraesCS3A02G399800	646,682,530	646,684,229	100%	646,894,696	646,896,395	
**T7-34**	Bri1	TraesCS3A02G245000	458,679,784	458,681,483	100%	470,135,375	470,137,074	
**T7-35**	TaGI	TraesCS3A02G116300	84,188,436	84,190,135	100%	84,573,271	84,574,970	
**T7-41**	Shaggy-like kinase 5	TraesCS3A02G164200	168,839,113	168,841,733	100%	180,360,881	180,363,501	
**T7-47**	FIZZY-RELATED 4	TraesCS3A02G527600	741,974,618	741,976,317	100%	744,107,440	744,109,139	
**T7-48**	LAX	TraesCS3A02G350600	599,324,345	599,326,044	100%	599,713,788	599,715,487	
**T7-55**	ETTIN = ARF3	TraesCS3A02G292400	522,277,033	522,278,732	100%	533,567,745	533,569,444	
**T7-56**	FZP = ERFL1	TraesCS3A02G328000	573,110,750	573,112,449	100%	573,509,458	573,511,157	
**T7-57**	BRI1-like protein 2	TraesCS3A02G099400	64,279,733	64,281,432	100%	64,662,717	64,664,416	
**T8-1**	Myb-TF	TraesCS3A02G187800	225,895,117	225,896,816	100%	237,713,492	237,715,191	
**T8-19**	VRS4	TraesCS3A02G093200	59,530,466	59,532,165	93%	47,579,699	47,581,397	T8-19A on Chr3A_v2_IWGSC_refseq Length=754109222 Score = 2533 bits (2808), Expect = 0.0 Identities = 1617/1748 (93%), Gaps = 77/1748 (4%) Strand=Plus/Plus
**T8-4**	Myb-TF	TraesCS3A02G251200	470,830,018	470,831,717	100%	482,278,397	482,280,096	
**T9-37**	LRR kinase (M8C3Z3)	TraesCS3A02G135400	112,951,165	112,952,864	100%	124,196,336	124,194,637	
**T9-39**	LRR kinase (M8C3Z3)	TraesCS3A02G167100	172,628,085	172,629,784	100%	184,295,627	184,297,326	
**T9-44**	RBOHF	TraesCS3A02G280200	509,428,096	509,429,795	100%	520,741,121	520,742,820	
**T10-10**	COM1	TraesCS3A02G124100	100,186,468	100,188,167	100%	100,584,025	100,585,724	
**T10-22**	XRCC2	TraesCS3A02G381500	631,717,710	631,718,673	100%	631,984,250	631,985,213	
**T10-24**	FIDGETIN	TraesCS3A02G151300	140,056,409	140,058,108	100%	151,551,057	151,552,756	
**T10-30**	MLH1	TraesCS3A02G500600	724,882,306	724,884,005	100%	725,614,593	725,616,292	
**T10-34**	ZIP4	TraesCS3A02G401700	647,482,860	647,484,559	100%	647,679,005	647,680,704	
**T10-43**	p31 COMET	TraesCS3A02G198600	314,622,724	314,624,423	100%	326,078,193	326,079,892	
