# Supplementary material for: Exploring the diversity of promoter and 5′UTR sequences in ancestral, historic and modern wheat
Source: Plant Biotechnol J. 2021 Sep 16;19(12):2469–87. doi: 10.1111/pbi.13672 (PMC8633512; doi:10.1111/pbi.13672)
Supplement: Supplementary file 11 — Data S1 List of all 1273 homoeologues including IWGSCrefseq1.1 gene IDs, complete target sequences and individual details for all baits used. [file PBI-19-2469-s006.pdf]

Column	Header	Explanation
A	Trait&gene no.	T1-1: trait 1 (Yield resilience) gene 1
B	Gene	name of gene
C	Homoeologues	A, B, D or U; missing homoeologues shown in green text; homoeologues with the same chromosome number are shown as ABD, **BUT** if one of the homoeologues is on a differently numbered chromosome a comma is used: eg A,BD shows there are 3 homoeologues but A is on a differently numbered chromosome whereas B&D are on the same chromosome number in the B- and D-genome
D	IWGSC gene ID	refseq1.1 annotations ID. Further information and publications on each gene can be obtained by pasting the IWGSC gene ID into the wheat version of Knetminer and exploring the resulting knowledge graphs (Hassani-Pak et al. 2021. Plant Biotechnology Journal - https://doi.org/10.1111/pbi.13583) - https://knetminer.org
E	WGIN Promotome ID	shortened ID including reference to original Ensembl ID
F	Orientation	sense (forward) or anti-sense (reverse)
G	ATG	position of start codon on chromosome
H	Sequence	complete nucleotide target sequence upstream from ATG start codon
I	MYbaits	number of high-specificity MYbaits in target sequence
J	MYbaits coverage of target (%)	% of target sequence covered by MYbaits
K	MYbaits separator	*
L	MYbaits ID	each bait is tagged with the WGIN Promotome ID & location of first nucleotide in bait, eg '6A-1552750-T1-1_448' identifies the MYbait as belonging to the A homoeologue of trait 1, gene 1 and the bait location starts at position [-448] from the ATG of the target sequence
M	MYbaits sequence	the actual 120 nucleotide sequence of this particular bait
	***please note:** for ease of locating individual baits they are presented in a vertical arrangement for ease of scrolling down. To locate all baits belonging to a particular gene and homoeologue it is easiest to search (Ctrl F) column N with the respective WGIN Promotome ID (column F)	

Trait&gene no.	Gene	Homoeologues	IWGSC gene ID (refseq1.1)	WGIN Promotome ID	Orientation	ATG	Target Sequence	MYbaits	MYbaits coverage (%)
T1-1	TaGW2-REUL1	ABD	TraesCS6A02G189300	6A-1552750-T1-1	forward	237,734,835	GCATGTAGGAGCAACAAAGGGGGTTCTTTTCGTACCTTAGATGGATCATTCTGAGCCTTCTTGAAGAGCTAGGCGCCG	11	54.6
T1-1	TaGW2-REUL1		TraesCS6B02G215300	6B-1675340-T1-1	forward	291,761,397	CACGTTTTGACAAGCCTTCATTAGGAAATTGCTTTTTGAAGCATGTAGGAGCAGGAAAGGGGGTTCTGTTCATACCTT	11	54.6
T1-1	TaGW2-REUL1		TraesCS6D02G176900	6D-1728330-T1-1	forward	175,712,421	AATTGCTTTTTGAAGCATGTAGGAGCAGGAAAGGGGGTTCTGTTCGTACCTTAGATGGATCAGTCTGAGCCTTCTTGA	12	55.1
T1-10	GIF1/OsCIN2	ABD	TraesCS2A02G295400	2A-0318750-T1-10	forward	508,031,557	TATCAAGGACCTGGGTGCTCTCCATTATTTCCTTGGCATTGAGGTCGTTCGGCGTCCGGACGGTTTCTTTCTTCATCAA	16	47.6
T1-10	GIF1/OsCIN2		TraesCS2B02G311900	2B-0441180_T1-10	forward	446,080,294	CTGGCCTGCGCTTCGCTGCTCCCAGGAGGAGGCCACTTGAGGGCAGTGCTCAGACTCTTTCCGTCGGAAATCCATTA	9	43.8
T1-10	GIF1/OsCIN2		TraesCS2D02G293200	2D-0538230-T1-10	forward	375,592,753	ATCTGCTCTCCGGACCAAACCCTAGCCACTTCACTATTCCACTTAAGCTCCCGTCTGGCGATCTCCAGCATGGCGGGCA	18	57.7
T1-11	GL3.1/qGL3/qGL3-1/OsPPKL1	ABD	TraesCS5A02G373900	5A-1240140-T1-11	forward	571,780,262	GTGTCCAATCAAACTTGACACAACAAGGCATCTTCTTTGCTCGAGTAGGCATTTTGTCTCTTCATCACTTTCCTTTTTG	13	58.2
T1-11	GL3.1/qGL3/qGL3-1/OsPPKL1		TraesCS5B02G375800	5B-1328580-T1-11	forward	553,346,917	TCGTGTCCAATCAAGCTTGACACAACAAGACATCTTCTTTGCTTAAGTAGGCATTTTGTCTCTTCATCACTTTCCCTTTT	11	54.2
T1-11	GL3.1/qGL3/qGL3-1/OsPPKL1		TraesCS5D02G383300	5D-1427730-T1-11	forward	452,843,590	TCACTTTCTTTTTGAGTGCCTGAGGATTGCCCAACATCAGACAAAGGCGAATTTAATCATCATCTCCCAAATAGGA	12	52.4
T1-12	OsPPKL2/BSL1	ABD	TraesCS1A02G088000	1A-0065720-T1-12	forward	76,022,842	TCTAATTGATATTGTGTGAGTTTGCATATGAGTATATATTAAACTCACTTCTCTTGTTTTCTTAGAAAAGGAGAATCAACT	10	47.5
T1-12	OsPPKL2/BSL1		TraesCS1B02G107000	1B-0179720-T1-12	forward	119,040,466	CTGTGAGTTTGCATACGAGTATATATTAAACACCCTTCTCTTGTTTTCTTAGAAAAGGAGAATCAACTCATGGCCTCTGC	9	40
T1-12	OsPPKL2/BSL1		TraesCS1D02G089300	1D-0259360-T1-12	forward	75,531,904	GCCTACAAGCGGGGTTTTGAGCCCTGCCAAGTTCGAACCGAAAAAGCGTGGGGCGCTGAATTGAGGTGTTTAAGGA	11	54.6
T1-13	OsPPKL3/BSL2 homolog	AB	TraesCS5A02G030300	5A-1271590-T1-13	forward	26,440,467	TTCGTGACTTGCATTGTGTGTAAATAAAGGGTATACATGTACACTAGACCATATCAGTTGCAACAATTAAATCGGGGTAA	16	73.4
T1-13	OsPPKL3/BSL2 homolog		TraesCS5B02G029100	5B-1381490-T1-13	reverse	27,839,660	GGCCGCCGCCGTCGCACCTAGCTACTAGGGTTTCCGCGAGCTCGAGATCCCCGCGCGGTCACGGAAACTAAGACAG	13	57.6
T1-14	DEP2/EP2/SRS1	ABD	TraesCS2A02G168600	2A-0369330-T1-14	reverse	122,014,540	GGCAGCGCCGCCGTCGCCGGCGAGGTCGGATCTGGTGGATCGCGGACGAGACCCGCCCCCCGCCCCTCCGCCTCCG	16	72.2
T1-14	DEP2/EP2/SRS1		TraesCS2B02G194900	2B-0467450-T1-14	reverse	172,003,474	GGCGCCGCTGCGGCCGGCGAGAGGTCGGATCTGGTGGATCGCGGACGAGACCCGCCCCACGCCCCTCCGCCTCCGA	15	68.5
T1-14	DEP2/EP2/SRS1		TraesCS2D02G176200	2D-0600080-T1-14	reverse	119,667,936	GGCAGCGCCGCCGCGGCCGGCGAGGTCGGATCTGGTGGATCGCGGACGAGACCCGCCCCCCGCCCCTCCGCCTCCG	16	70.1
T1-15	SRS3/OsKINESIN-13A	ABD	TraesCS3A02G194100	3A-0680890-T1-15	reverse	269,226,807	CACCGCGTCCCCGAGTCAGTCGCGGCAGACGACGCCGCGGCAGCTGTGGCGGCGGCCGCGCCACCGCCCCCGCCC	4	24.9
T1-15	SRS3/OsKINESIN-13A		TraesCS3B02G223900	3B-0812340-T1-15	forward	296,182,260	AGAGTAAAACAGACTCATCATAGATAAAACATTCTTACTTAATTAACTTAGCATGAGTACTCACTTAGCATAGTAGGTCT	3	21.2
T1-15	SRS3/OsKINESIN-13A		TraesCS3D02G197700	3D-0906440-T1-15	forward	209,673,507	AGCGGCATATGCTGGAGTTCTCATGCCAATTAGGGACAGATCCCAATGGCCCAAGGTAAATCCTGGATTTGATATGATC	12	64.9
T1-16	SRS5/TID1/TUBA	ABD	TraesCS1A02G350200	1A-0026030-T1-16	forward	535,063,029	AGCACAACGTCGCGCCCGCTCGCCCGCTGCTCATCCTCTTCCGCACCACGCCTCATCGAGCAGTGTAACGCCTACTCCC	22	67.8
T1-16	SRS5/TID1/TUBA		TraesCS1B02G364500	1B-0139290-T1-16	forward	594,138,226	AATGATTATAAATATAAGAGAATGATGATTATGTTTTGTGCAGATTTCCCACTGATTTCTGTCCTCGAGGAAGTCATTATT	11	48
T1-16	SRS5/TID1/TUBA		TraesCS1D02G353100	1D-0189800-T1-16	forward	438,368,548	CGGGCCGGCCCGTGGCACGTTTAGCACGCTGGCCCATCTGATTTTTAGCCTGTTGGGCTGTATTTTAGGCCTATTAGG	17	49.7
T1-17	GL7/GW7/SLG7	ABD	TraesCS2A02G176000	2A-0370760-T1-17	forward	135,192,157	AGAGTTTAGCTTCATCCCAGGGGCCATTGATCTGAAACCGGCCAAAAGTCGGTGGCCAAAATTGCACATATGCTGCAC	6	35.8
T1-17	GL7/GW7/SLG7		TraesCS2B02G202300	2B-0485790-T1-17	reverse	182,015,179	TCGCCGTGGTCATGACAGTTAACCCTCCCGAGTCCCGGTTGATGGAGTCCTTGACGATGTCTCTGAAACCGACGTTCG	22	54.8
T1-17	GL7/GW7/SLG7		TraesCS2D02G183400	2D-0603290-T1-17	forward	128,394,940	CATCGCCAAACGGGGCTAGGCAGGAAGAACCCTAACGATTCAATCTACAGAGAGCCGTCGCGGCTTCGTCTATGAGC	24	62.6
T1-18	DEP3	B, BD	TraesCS6B02G416400	U-1257530-T1-18	forward	63,674,767	AACACTTTGGAGCAACATCCGGGCCAGATGTTGCCCTGGGCCTATTGTTTGTTTCGACTAGCCCTTGCTGATTAGTGG	14	69.2
T1-18	DEP3		TraesCS5B02G379300	5B-1294540-T1-18	forward	557,354,507	GCTTGGACTATCGTTCGTAGGTGGCCTAGCTAGCTAGCTAGGTCGCCGTGACAAGAATATCTAAACGTGATTATTTGTT	13	60.9
T1-18	DEP3		TraesCS5D02G385900	5D-1422290-T1-18	forward	455,614,202	GTTGTGATACTGCGGCGATCGGCTATTAGTTTATCAAGCGTTCAGATTTCTGATCTACGAGCATCAACTTGGCAAGAAC	11	53.4
T1-19	PGL1/ILI6	ABD	TraesCS4A02G016300	4A-1025700-T1-19	forward	10,938,614	TCATCAAGTAAAACTATGTTGCAAACTGTGTAAGCCTACATGTATTGATACATTCCCGAGAGTTTGTTGGGAGCTGAAA	15	69.6
T1-19	PGL1/ILI8		TraesCS4D02G286600	4B-1058590-T1-19-1	reverse	572,157,641	TATGGCAAATGTGAGTGATCGGATGTACTTAGGAATTGGATACTTGGCAGAGAGAGCCTAAGACAGTGAGGGTTTATG	14	67.9
T1-19	PGL1/ILI7		TraesCS4B02G287900	4D-1112190-T1-19	reverse	458,091,675	CACTGCAAATGTGAGTGATCGGATGTACTTAGGAATTGGATACTTGGCAGAGAGAGCCCAAGACAGTGCGGGTTTATG	11	61.2
T1-2	TaGS5-3A	ABD	TraesCS3A02G212900LC	3A-0675260-T1-2	forward	176,555,957	GGCCACGCACGCACGAATCTCTGGTCCCTGGGACCCACTTGCCAGTCACACGGTGGTGTCGAGCACTGCCGGTGTGA	8	42.1
T1-2	TaGS5-3A		TraesCS3B02G433000	3B-0735220-T1-2	forward	671,996,341	AGATATCAATATACAAGCCCAGAGTGGGCCGACTCCTAGTAGGCGGCCACCAGAGTGGCCGACCTCTAGCAGGCAGC	5	22.1
T1-2	TaGS5-3A		TraesCS3D02G172900	3D-0927310-T1-2	reverse	153,179,401	GAGAATGTGCAAATGTGAGAGTGAGTAGCTTGGCTGGGACAGCAAGAGCAGAGAGGCAGGGGCTGGGCGCAACTC	2	14.1
T1-20	APG/OsPIL16	ABD	TraesCS1A02G083000	1A-0058870-T1-20	forward	66,175,409	GCTCCCCAGGCGCGCGGGCGTACGCGGGTCCGCGGCTTGACGCCCACGCCGTCGCCATCGCCGTGTCCATCGCCACC	9	43.1
T1-20	APG/OsPIL16		TraesCS1B02G100400	1B-0157510-T1-20	forward	109,211,348	CCTACAATGACGGGGGGCCAACTGGTTGCACAGACTCCGCATCTTCTGCGTCTCCTTCGGCATCGGAAGAAGGCATCA	6	35
T1-20	APG/OsPIL16		TraesCS1D02G084200	1D-0247260-T1-20	forward	67,861,697	TATACCCATACCCTATCCATTTATACTAACATGTGAATTTTACCCGTGATTCACAAGTGTACCTATGTTAAAGTTGTGTAGT	9	49.2
T1-21	PGL2/ILI5/BHLH170	AD	TraesCS6A02G306200	6A-1524810-T1-21	forward	539,865,676	GCGTCGGCGACGTAGCAGGCGGGCGAGGATGTGAATCCAATATGCCACCGACCAGCGGGCCTGGTAAAAAAAGAG	10	47.5
T1-21	PGL2/ILI5/BHLH170		TraesCS6D02G285300	6D-1709060-T1-21	forward	393,299,428	GATCGACGACGGGGCCCTCGTGTCGTGCCGTCCAGCGGTGGAGAGGCTCGTGACATGACGGTCACGCGGCGGAGG	8	43.3
T1-22	BU1/LI4/BHLH172	ABD	TraesCS7A02G185300	7A-1832600-T1-22	forward	139,468,173	CGTCGACTGGACTCGGGAGCTCGCTGACGCGCTCGCTCGTCGAGTGTTCGCTGGAGATTCTCCAGTCGAGTGCGCTC	3	14.6
T1-22	BU1/LI4/BHLH172		TraesCS7B02G090500	7B-1941450-T1-22	forward	104,757,605	GGGGCGCTTGGGGAGCCGGCGGACCCAGGCCGAACCCGGCGCCCTGGGGGGCGCTTGGGGAGCCGGCACAAGC	2	10.8
T1-22	BU1/LI4/BHLH172		TraesCS7D02G187000	7D-2023210-T1-22	forward	139,828,859	CAAAAGGAGCCGGACCGCGGCCCAGGACGAGGCGTGAGCGTGGGGGTGCCCCCGCGCGACAGGCGCTGTGGCC	13	65.4
T1-23	AP2-like	ABD	TraesCS5A02G221600	5A-1216860-T1-23	reverse	437,916,588	TGTGCGTGCTGCTGCTGCGTGGGGGAGGGAGGGAGGGAGGGAGGGAGGTTGGGTGGCCAACTATGGTGGCACGG	16	74
T1-23	AP2-like		TraesCS5B02G220400	5B-1313490-T1-23	reverse	394,747,764	TGCGCTGCTGCCTGCTGCTGCGTGTGGGAGGGAGTGAGGGGGAAGGAGGGAGGTTGGGTGGCCAACTATGGTGG	16	74.6
T1-23	AP2-like		TraesCS5D02G229400	5D-1420620-T1-23	reverse	336,426,224	TGCACGTGCTGCTGCTGCTGCGTGTGGGGGGGGAGGAGTGAGGGGGAGGTTGGGTGGCCAACTATGGTGGCACC	11	50.1
T1-24	YABBY-4	AB	TraesCS6A02G237700	6A-1511990-T1-24	reverse	446,903,072	TATGAATGTATGTGGTAGCAGCCGCTATGCCCTGAGTCTGCTGATCGATCGGGGACGGCTAAGTATGTGTGTGGGTGG	14	74.6
T1-24	YABBY-4		TraesCS6B02G266200	6B-1591850-T1-24	forward	478,753,047	AGTTAGAGATGACAAAAAAAGATGTCTACAATGGGTCATTTCTTAGTCTTATCTTCAATAACTAGTTATTCCTAAAAACG	8	49.9
T1-25	Glabra-3	ABD	TraesCS1A02G102400	1A-0065150-T1-25	forward	98,584,808	CGGACATCTAAAACCATAGGCCTCAACCAATCTGCACTACGGCGGGTTGCGGGCACAAACCGGTCAAACGGACTCAC	9	53.6
T1-25	Glabra-3		TraesCS1B02G113100	1B-0173960-T1-25	forward	131,775,632	AGGAGGTGCACATGATTGGACTGCCTGCGGAGACCTCACACCAATGCCAAACATCTAAAACCAGAGGCCTCAACCAA	8	40
T1-25	Glabra-3		TraesCS1D02G094200	1D-0258100-T1-25	forward	79,937,738	TGTAGATTTCGACATTTTGAGAACTTCTCAGTGTATTCAATTTTGAGAGGGCTTGTTCTAGGAATCACTTGCATCATTAT	5	28.8
T1-26	Yabby-6	ABD	TraesCS5A02G025900	5A-1271170-T1-26	reverse	20,998,405	TCCTCCTGATTATCCCCTGGAGCCGCCGCCTCGCTGCCCCCCTAGCCGTTCAGACTACGGCGAGGTTCACCAGGCGGC	14	69.2
T1-26	Yabby-6		TraesCS5B02G025100	5B-1375620-T1-26	reverse	24,014,113	TCTTCCTTCTGATTATCCCCTGGCGCCGCCGCCTCACTGCCCCCCTAGCCGTTCAGACCACGGCGAGGTTCAACAGGC	13	68.7
T1-26	Yabby-6		TraesCS5D02G033700	5D-1483350-T1-26	reverse	32,299,899	ATGCTATCCAAGCAAAAAGACAAGTGCATGGGGGAGGAAACCAAAGATCAAGGTAGATTGGAAAAGGTCTGCGATT	16	72.2
T1-27	NAM-TF	AB	TraesCS6A02G378500	6A-1508490-T1-27	forward	599,259,537	ACACATGGGACTGGAGGGAGTGGATGCTGGGTGTATATGACGCGTATGCTCCAACAACCGTATACCATGATATGTACTG	12	67.1
T1-27	NAM-TF		TraesCS6B02G416400	6B-1606780-T1-27	reverse	688,666,287	GGAAATGCTTATTATCTCTATCTGTGAACTATCTTCTCTTTGAAACCTTCCCCTGCTCAGCTCAGCTCGACTCTATCTACA	10	57.4
T1-28	Brassinosteroids	B,AD	TraesCS2B02G566500	2B-0429050-T1-28	reverse	757,479,135	AGGCAGAGGCGAAGAGGAAGAAGGTGTGAGCGAGCTAGAGGTGCTGAGTTGATCAGCTGCTTGGCTGCAGCGACC	16	66.8
T1-28	Brassinosteroids		TraesCS3A02G021900	3A-0673330-T1-28	forward	12,479,240	GGGCTAGTTGGGGCTCAAACCAAACACACCCTAACACCCCCGGCCTACTAAAATTCCCCTCCCTGTCTCATTCTCTTCC	14	68
T1-28	Brassinosteroids		TraesCS3D02G024700	3D-0899560-T1-28	reverse	8,341,355	GGGCGGAGAGCTTCTCTTGCTGAGCTGATCCGCTCGCTCTGTGGTGTGGGTGTGGCTGCAGCAACAGAAACAATTAC	7	39.5
T1-3 & T6-41	TaDEP1	ABD	TraesCS5A02G215100	5A-1192740-T1-3	forward	430,488,527	CTACGGGCTGCTGCCGCTCCTCCTACTCCCACTCCCACTAGCCAGTAGCGAGCGCGCCCGTCTTATCCTGGGCCCCCGC	6	35.7
T1-3 & T6-41	TaDEP1		TraesCS5B02G208700	5B-1323430-T1-3	reverse	378,520,474	GTCTCTTTGTCTCTTACACGCCCGGAACTGGAGGGCTTAGGCTTAGCCACCGGCCGCATTAGGCGCCGGCGGCGGG	7	41.1
T1-3 & T6-41	TaDEP1		TraesCS5D02G216900	5D-1456550-T1-3	reverse	326,129,224	GTCTCTCTGTCTCTCACACGCCCGGACCCGGAGGGCTTAGGCTTAGCCACCGGCCGCATTAGGCGCCGGCGGCGGG	7	35.9
T1-4	TaCKX2	ABD	TraesCS3A02G311100	3A-0704020-T1-4	reverse	550,050,563	CTCCTGGGTGTCTTAGCTTGAGTGATCGATGGATCGATCTCTGTGTCTTTGTGGCTCTTCTCTTGCGGATGGTGGGCTAT	19	83.5
T1-4	TaCKX2		TraesCS3B02G161000	3B-0710040-T1-4	forward	157,660,306	ACAAAGGCCAAGGTGTCCGCCGTTGAGGGCTCTCTGGCGTCTGACGCTGCCTTCTATCGCTCCATCGTCGACGCTCTC	4	24.9
T1-4	TaCKX2		TraesCS3D02G143300	3D-0908010-T1-4	forward	105,891,412	CCGAATCCCCCGCGCCCGCTGGAGCGCGCGTAACCGCTCCACGCGTGCTAAAACATTTGTTTCCCCGTGCGCAATATAT	14	65.9
T1-4B	TaCKX2		TraesCS3D02G143500	3D-0907390-T1-4B	forward	106,736,628	GCCACGCGGGTGGCCTCGTGCGCGTTCTCGAACGTCTCGAGACCGAGACGTGCGCCACCGGAGCGTATCTCCGCGTA	11	50.8
T1-5	Vrs1/Hox1(AB478714)	ABD, U	TraesCS2A02G188500	2A-0323970B-T1-5	forward	153,452,292	AGCACGGCGATGTAACAAGTCAATCCACTGCTCGTTGTTGCGACGAAAGGTCGTTCGATCGCATTGGGCAGTTCAATT	10	47.5
T1-5	Vrs1/Hox1(AB478714)		TraesCS2B02G405700	2B-0439760-T1-5	forward	573,974,813	ATGTTGCCACCCATTCCTCTACCATGTCCGCCGCCCATCATGTTACTCCTAGCATGGCCTCCTTTGCCAACATTTCATTG	14	65.9
T1-5	Vrs1/Hox1(AB478714)		TraesCS2D02G385500	2D-0514530-T1-5	forward	490,118,000	GAGTACCCGGCGTCGTCCTTCTCTCGCCGCTGCAAAAGCTCCTAGATGTCGAGGCGCATGGACACGCTGTCCTCGTCG	2	14.1
T1-5	Vrs1/Hox1(AB478714)		TraesCS6A02G378500	U-0323970A-T1-5	forward	13,023,491	GCATGCCCTCCTTTGCCAACATTTCATCGCGACGAAGATCAACATGTATGCCTCTTGCTTCACGCCAAGACAACTTATGT	22	81.2
T1-6	RGA1/D1/GA1/GPA1	B,AD	TraesCS1B02G479100	1B-0088580-T1-6	reverse	687,092,412	TATTATTCTAGACGGGCGCGTGGTCTCATGCTTCCTTGCAAAGACAGACAGACAGACGCCTAGATCCTCCTAGTGCATT	16	68.3
T1-6	RGA1/D1/GA1/GPA1		TraesCS7A02G000200	7A-1810320-T1-6	reverse	245,459	CAGGACAGGAGAGGAGGAATTGAATGAAATGAAGAGGTCTAGTAAGTGGTATGGAAAGGATGAGCTTACTCACTCT	6	32.5
T1-6	RGA1/D1/GA1/GPA1		TraesCS7D02G000100	7D-2033470-T1-6	reverse	211,727	GGAGACTTATTATTATTATTATTCTAGAAGACAAGACGCGCGGTGGGTCTCATGCTTCCTTCCTTGCCAATGACCTGAA	9	43.8
T1-7	BG1	ABD	TraesCS4A02G012100	4A-1015830-T1-7	forward	6,922,238	GAATGGGGAGCCTGGGCTGTACTGCGTGGCGCGCTGCCATTCCCCGCGATTAAAGACACGGATCGAGGTCTTACAA	20	84
T1-7	BG1		TraesCS4B02G292300	4B-1038330-T1-7	reverse	577,698,631	TCCAGCAGCAACACACGGACACAGCAACCACACCAACAATGCGCTGTTCTAGTGCAGAAAGTGGCGCGAGGCAGAG	14	72.5
T1-7	BG1		TraesCS4D02G290900	4D-1125360-T1-7	reverse	461,813,588	TCCACGGACAATGCTCTGTTCTACAGCGGAAAGTGGCGCGAGGCAGAGGAACTCCGTGTACAGTGAGATGGTTCTCC	17	80.5
T1-8	GS3	A,AD	TraesCS4A02G474000	4A-0991420-T1-8	reverse	733,695,306	TGCCGGCCTCGGAGGAGCCTGGCTAGCTGGACGGTGGTGGATGGAGGGCTCGCTCGCGCCGGCTGGCTAGCTGGG	17	76
T1-8	GS3		TraesCS7A02G017700	7A-1848980-T1-8	forward	7,598,370	CAGTTCTCGAATTGTTTTCCATGCCGACATCCCCATTAATGTCACAACTATTCTGTTCAACTAATTATTTTAGCATGACAAT	16	76.7
T1-8	GS3		TraesCS7D02G015000	7D-2034360-T1-8	forward	6,483,394	ACACCATCTGCTTAATATTTACTCCGTGATCCAACATATTTGTCTACCTACTGTAAACAAAGTAGCTGATACTACTGTAGA	15	72.9
T1-9	GW8/qGW8/OsSPL16	ABD	TraesCS7A02G260500	7A-1835860-T1-9	forward	252,715,654	TACGTACCGCCATTGCAAAGCTAGGTAGTACTGACATCCAGATCACAAACCACGGTACGCAGACAGCATAGCCGGCCG	12	61.6
T1-9	GW8/qGW8/OsSPL16		TraesCS7B02G158500	7B-1957310-T1-9	forward	214,070,890	CGCGCCACCACGTCCACGTACGTACGTACGTACCGCCATTGCAAAGCTAAGTAGTACTGACATCCAGATCACAAACCAC	12	64.9
T1-9	GW8/qGW8/OsSPL16		TraesCS7D02G261500	7D-2014240-T1-9	forward	237,410,697	ATGCCGGTCGCGCCACCACGTACTACGTAGTACGTACGTACCGCCATTGCAAGGCTAAGTAGTACTGACATCCAGATCA	12	64.9

MYbaits IDs	MYbaits sequences
6A-1552750-T1-1_128	TTTCAGCAACCAAGACTAGATCCAGTTCAGTACAAAGAATGGAAGAACAAAAAAACATGACATGATTCGATCCTTGCGCTGATTTTCTTACCCGTCGAGGATGTGGGTGTGGTCACATGG
6A-1552750-T1-1_320	CATGCACATCATGAACGAGAAACACGAGTAAAGCACTACATGAACCCCTTATGAACGATTTAGCAATGAACACAAAATTCTAGTATTTAGACATGAGAATGCCTTGTACAGAATTTAAA
6A-1552750-T1-1_384	CAATGAACACAAAATTCTAGTATTTAGACATGAGAATGCCTTGTACAGAATTTAAAAGTCATGAAAAACGTTACCTCTGGTTTGGGTGTCGTGCAAATAAAACAGAGATAATAAATTCCT
6A-1552750-T1-1_448	AAAACGTTACCTCTGGTTTGGGTGTCGTGCAAATAAAACAGAGATAATAAATTCCTTATAGCCAATGTGGTGAACATAGCAAATTGATTCCCCCGGGTTTGATTCCATGTGCTCTAGCCA
6A-1552750-T1-1_512	ATGTGGTGAACATAGCAAATTGATTCCCCCGGGTTTGATTCCATGTGCTCTAGCCAAAATAGATCAAATCAGCAAGATATCTTATGCTATGAATGGTGATAGTGGTCGCTCATGTTCATC
6A-1552750-T1-1_960	AGCAGCTCGCAACAAACCCTAGCTCGCGCGAGAAGAGAGAGGGGATGTTCGGATCAAAGAGAGGACGAGAGAAAACCGGCGTGGTAAGAAAATCGATAAGGAAAGAACATCGTATGAGT
6A-1552750-T1-1_1024	ACGAGAGAAAACCGGCGTGGTAAGAAAATCGATAAGGAAAGAACATCGTATGAGTGGAGAAGGGTGAGACGAAAATAAATCGAACGAAAATAATCATAAAGTGAAAGCTACCAAGTCCT
6A-1552750-T1-1_1088	GTGAGACGAAAATAAATCGAACGAAAATAATCATAAAGTGAAAGCTACCAAGTCCTTCTTTAAAAGTAGAGATCACATATTCGCTTAGAGGAAAGATGAAGGGGTAGGTGATGCGCCCGC
6A-1552750-T1-1_1216	ACTCATCATGTCGCTTCCCATTTACGAAAGCATTACCTATGTTTATCAAGCGTTACATGGGATAGGGGATATACACAAACATGTTCCTAAATTAATTAAAAAAAACATGTTTCTAAATTG
6A-1552750-T1-1_1280	GGGGATATACACAAACATGTTCCTAAATTAATTAAAAAAAACATGTTTCTAAATTGTGACACAGATCGATGGTCCAGAATCTACGTGTCACAAAACTAATTGGGAAGATTTTCGAGAGGT
6A-1552750-T1-1_1344	ATCGATGGTCCAGAATCTACGTGTCACAAAACTAATTGGGAAGATTTTCGAGAGGTGTATCTCTTGCAGCGCAGCGGGCCAGCGGCAGAGAAGCCGAAGCCTAGTGAGCTGTGTTGTCGT
6B-1675340-T1-1_64	TCTGTTCATACCTTAAATGGATCAGTCTGAGCCTTCTTGAAGATCTAGGTGCCGAAGCTCCCAGTGATTGGTGATTGATCCCCGCCGGCAAGCAAGACCAGATCCAGTTCAGTACAAAGA
6B-1675340-T1-1_320	CATGTCCGGCCCGCGGCACCTGCACATCATGAACGAGAAACACGAGTAAAGCACTACATGAACCCCTTATGAACGATTTAGCAATGAACACAAAATTATAGTATTTAGACATGAGAATA
6B-1675340-T1-1_384	CCTTTATGAACGATTTAGCAATGAACACAAAATTATAGTATTTAGACATGAGAATACCTTGTATAGAATTTAAAAGTCATAAAAAACGTTACCTCTGGTTTGGGTATCCTGCAAATAAAA
6B-1675340-T1-1_448	AGAATTTAAAAGTCATAAAAAACGTTACCTCTGGTTTGGGTATCCTGCAAATAAAACAGAGATAATAAATTCCTTATAGCTAATGTGGTGAACATAGCAAATTGATTACATCGGGTTTGA
6B-1675340-T1-1_512	ATAAATTCCTTATAGCTAATGTGGTGAACATAGCAAATTGATTACATCGGGTTTGATTCCGTGTGCTCTAGCCAAAATAGATCAAATCAACAAGATATCTTATGCTATGAATGGTGATAG
6B-1675340-T1-1_576	GCTCTAGCCAAAATAGATCAAATCAACAAGATATCTTATGCTATGAATGGTGATAGTGGTCGCTCATGTTCATCTCGACTGCCGAAATCATGTGCCCTTAGCGGACGTTGTACTCCTCGG
6B-1675340-T1-1_960	GAGATCCCGTGCAGTAGCTCGCAACAAACCCTAGCTCACGCCAGGAGAGAGAAGGGATGTTCAGATCAAAGAGAGAACGAGAGAAAACCGGCGTGGTAAGAAAATCGATGAGGAAAGGA
6B-1675340-T1-1_1024	ATCAAAGAGAGAACGAGAGAAAACCGGCGTGGTAAGAAAATCGATGAGGAAAGGACATCGTACGAGTGGGAAAGGGTGAGACGAAAATAATTCGGACGAAAATAAACGTAAAGTGAAAG
6B-1675340-T1-1_1088	GAGTGGGAAAGGGTGAGACGAAAATAATTCGGACGAAAATAAACGTAAAGTGAAAGCTATCAAATCCTCCTTTAAAAGTAGGGATCACGTATTTGCTTGGATGAAAGATGAAAGGGTAGG
6B-1675340-T1-1_1280	TTACAGGGGATAGGGGATATACACAAACATGTTCCTAAATTAATAAAAAAACATGTTTCTAAATTGTGACACAGATCGATGGTCCAGAATCTACGTGTCACAAAAGTCCTTCACACAGGG
6B-1675340-T1-1_1344	TGTGACACAGATCGATGGTCCAGAATCTACGTGTCACAAAAGTCCTTCACACAGGGAAGATTTTCGAGAGGTGTATCTCTTGCAGCGCAGCGGGCCAGCAGCAGAGAGAGAAGCCGAAGC
6D-1728330-T1-1_128	GCTGGATCTTGCTGTTTCAGCAACCAAGACCAGATCCAGTTAAGTACAAAGAATGGAAGAAAAAAATTAGGGCATGATTCGATCCTTGCGCTGATTTTCTTACCCGTCGAGGATGTGTGT
6D-1728330-T1-1_320	GGGCCTGCGACACCTGCACATCACGAACGAGAAACACGAGTAAAGAACTACATGAACCCCTTATGAACGATTTAGCAATGAACACAAAATTCTAGTATTTAGACATGAGAATACCTTGT
6D-1728330-T1-1_384	TGAACGATTTAGCAATGAACACAAAATTCTAGTATTTAGACATGAGAATACCTTGTACAGAATTTAAAAGTCATTAAAAATGTTACCTCTGGTTTGGGTATCCTGCAAATAAAACAGAGA
6D-1728330-T1-1_448	TAAAAGTCATTAAAAATGTTACCTCTGGTTTGGGTATCCTGCAAATAAAACAGAGATAATAAATTCCTTATAGCTAATATGGTGAACATAGCAAATTGATTACCCCGGGTTTGATTCCAT
6D-1728330-T1-1_512	TCCTTATAGCTAATATGGTGAACATAGCAAATTGATTACCCCGGGTTTGATTCCATGCGCTCTAGCCAAAATAGATCAAATCAGCAAGATATCTTATGCTATGAATGGTGAAAGTGGTCG
6D-1728330-T1-1_576	GCCAAAATAGATCAAATCAGCAAGATATCTTATGCTATGAATGGTGAAAGTGGTCGCTCATGTTCATCTCGACTGCAGAAATCTGTGCCCTTAGCGGACGTTGTACTCCTCGGCAGTGG
6D-1728330-T1-1_960	CCCGTGCAGCAGCTCGCAACAAACCCTAGCTGGCGCGAGGAGAGAGATGGGATGTTCGGATCAAAGAGAGGACGAGAGAAAACCGGCGTGGTAAGAAAAATTGATGAGGAAAGGACATCA
6D-1728330-T1-1_1024	AGAGAGGACGAGAGAAAACCGGCGTGGTAAGAAAAATTGATGAGGAAAGGACATCATACAAGTGGGGAAGGATGAGACGCAAATAAATCGGACGGAAATAACCGTAAAGTGAAAGCTACC
6D-1728330-T1-1_1088	GGGAAGGATGAGACGCAAATAAATCGGACGGAAATAACCGTAAAGTGAAAGCTACCAAGTCCTCCTTTAAAAGTAGAGATCACGTATTCACTTGGAGGAAAGATGAAGGGGTAGGTGATG
6D-1728330-T1-1_1152	CTTTAAAAGTAGAGATCACGTATTCACTTGGAGGAAAGATGAAGGGGTAGGTGATGCGCCCGCGGTGATGCGCTCATCATGTTGCTTCCTATTTACGAAAACATTACCTATGTTTATCAA
6D-1728330-T1-1_1216	GTGATGCGCTCATCATGTTGCTTCCTATTTACGAAAACATTACCTATGTTTATCAAGCGTTACAGGGGATAGGGGATATGCACAAACATGTTCCTAAATTAATTAAAAAAACATGTTTCT
6D-1728330-T1-1_1280	GGGGATAGGGGATATGCACAAACATGTTCCTAAATTAATTAAAAAAACATGTTTCTAAATTGTGACACAGATCGATGGTCCAGAATCTACGTGTCACAAAACTCCTTCACACAGGGAAGA
3A-0675260-T1-2_128	TTAAGAGAAAGGCATATTCCGAATTGCAATCTTGGATAGAAAGATTTGCATACTTCTCTCAATTTTCTTGCTTTTTGTATAACTGCCTTTGTAGATGTGCAAGCAAAGACAAACAAAAAA
3A-0675260-T1-2_192	TTCTTGCTTTTTGTATAACTGCCTTTGTAGATGTGCAAGCAAAGACAAACAAAAAAAATGCATGCAGTACAATTGTATGGGACGGTATGGGAAGTCCCCTCCATCAACAGATCGGGTCCG
3A-0675260-T1-2_832	TGTATATTTTTCATTTTAATTTTGTCTTATATCATAGGGTTAGTTAGTGGCGGAGCCAACCTTTCAAAAAGGTCGGGCAAAGTCTATGCATGCATGGTGACGTTGCCAGTATTTGCACCA
3A-0675260-T1-2_896	CAAAAAGGTCGGGCAAAGTCTATGCATGCATGGTGACGTTGCCAGTATTTGCACCAGAGCATCAGCAATTAGCATTACTAACCATGTTCTGGTAATATTATTTAATAGAGACTGGCCGGA
3A-0675260-T1-2_1024	GGCAGCCGCCCCAGGTTGCCGGGAGGTAGGGTTAATGTCTATCAGATGGAGCTACTGTCGCGAGGCATTCAGCCTCGAATTTTAATTTTGCCTTTTTTTCGGGTCTGGTGTTCGGCTGCT
3A-0675260-T1-2_1088	GCATTCAGCCTCGAATTTTAATTTTGCCTTTTTTTCGGGTCTGGTGTTCGGCTGCTGATTTCTCATTTTTAATAATATATGGCTACGCGCATCAAGGCTTTCGAGAAAAAGAACATGTAG
3A-0675260-T1-2_1152	ATTTTTAATAATATATGGCTACGCGCATCAAGGCTTTCGAGAAAAAGAACATGTAGTACAATGGTACTGTTTTCGGTaaaaatgaaaaagaaagagagaaaacagaaactgcaaacGCCA
3A-0675260-T1-2_1216	TACTGTTTTCGGTaaaaatgaaaaagaaagagagaaaacagaaactgcaaacGCCAATCTCACTTGTCTCATGCACGAAGGTTTCTACTGTATGCTCGTCCAACTTGATGAATCGGTACG
3A-0675260-T1-2_1536	CTcacccccacccccacccccacccccCAGCTCTGAGCTCCCTCCAACTTCACATGATCTTCTAGTGTGATCTCACTCTCACACAGATTGACTTGCGCCCAGCCCCTGCCTCTCTGCTCT
3A-0675260-T1-2_1580	CAACTTCACATGATCTTCTAGTGTGATCTCACTCTCACACAGATTGACTTGCGCCCAGCCCCTGCCTCTCTGCTCTTGCTGTCCCAGCCAAGCCACTCACTCTCACATTTGCACATTCTC
3B-0735220-T1-2_1024	TTGCACCTACCACCCGACACGCGCCGAGAACCGATCGTGGCTCGGAGAAGGTTGGACAAAAATGTACGTAAAACCGAAGAAGAAATCTTGCATTATTTTTATTAAGTAGCAGTATCAGGA
3B-0735220-T1-2_1088	TACGTAAAACCGAAGAAGAAATCTTGCATTATTTTTATTAAGTAGCAGTATCAGGAGACACAACATCTTCTCCTTTATTTATTATGGCTTTTACTCAGCCTCTCTTATCCATAACAAGAA
3B-0735220-T1-2_1152	ATCTTCTCCTTTATTTATTATGGCTTTTACTCAGCCTCTCTTATCCATAACAAGAAGTTCAACTCTATTTAAGGATGAGGCTGTTCTTACCGCAAAAGAAGGCTGGGACTGTTCTCACA
3B-0735220-T1-2_1216	TCTATTTAAGGATGAGGCTGTTCTTACCGCAAAAGAAGGCTGGGACTGTTCTCACACCCCTTCTCCCTTATCCGTAAAAAACCCTATTTTTAGTGCTGGCCATTAGCCTAAAACATTTGT
3B-0735220-T1-2_1280	CCCTTATCCGTAAAAAACCCTATTTTTAGTGCTGGCCATTAGCCTAAAACATTTGTCTTGGAGAGAAATCCATATCCATCAGATTCAGTTCCAGGATAAATCTGATCCTATCCAAGTTT
3B-0735220-T1-2_1344	GAAATCCATATCCATCAGATTCAGTTCCAGGATAAATCTGATCCTATCCAAGTTTTGACTAGGCAAAAGCTAGCTACTACCCTAGTTAACGGGCCAAGATTACACCGCCGTGCTCAATC
3B-0735220-T1-2_1408	CAAAAGCTAGCTACTACCCTAGTTAACGGGCCAAGATTACACCGCCGTGCTCAATCAACGACCCTCGCCGCCGCAAAAACGCAGCCGCACAAGGCTCCGCATTTTCCCCCGTGATCCCCT
3B-0735220-T1-2_1472	TCGCCGCCGCAAAAACGCAGCCGCACAAGGCTCCGCATTTTCCCCCGTGATCCCCTCCCATCTCCCGATCTCCGGCCTCCTTCCCTCTATATAAGCGCCCGCCTCGCCACCTCACCAGAA
3D-0927310-T1-2_0	GAGAATGTGCAAATGTGAGAGTGAGTAGCTTGGCTGGGACAGCAAGAGCAGAGAGGCAGGGGCTGGGCGCAACTCAATGTGTgtgagagtgagatcacactagaagatcatgtgaagttg
3D-0927310-T1-2_384	TAGAAACCTTCGTGCATGAGACAAGTGAGATTGGCgtttgcagtttctgttttctctctttctttttcattttACCGAAAACAGTCCCCACCAGTTCAAACTCTGAGGTTTGGGTCCTT
3D-0927310-T1-2_576	TTTGCCACCCATTCCGACGGAGCCACTTGTCTTGTCGCCGAAGTGTGGACCAGCCTTGCCAGGAGTGGCGCGTGCCTCGACTCCGACTAGCAAGACAGCAGCTGGGCAGGAACACATGGC
3D-0927310-T1-2_1466	ACAGGTATGTCGGTTAATAAGCATTGCTCTATTCTGCATTTTTCTGAACATGAGATTATGAGTAGAGCCCATGACATAGGAATCCTCGCTAGGGGCAAATGGGGTCGAAATTGTTAATTCA
5A-1192740-T1-3_63	TATCCTGGGCCCCCGGGGCGGACGTGGACGCGTCAGTGTGCGCGCGCGGATACGTCGTCCGTGTGGTTGCGTCCCTGTCCGCGCAAGCAAGCCGAGGGCGCGCGGCCTCGTCGTTCTGT
5A-1192740-T1-3_126	GTGGTTGCGTCCCTGTCCGCGCAAGCAAGCCGAGGGCGCGCGGCCTCGTCGTTCTGTCCCGTTGCCCCGCGGATGGCTTGCTTTTCCGTACGCCCGGCTCGGGGGGCGCGCCACCCCGGC
5A-1192740-T1-3_504	GGCAAATTGTGGCTGAGAACCAAACAGCCCCgtactagtactagtacagtactactctagtagtaCGTGACGGATCGTTCAGGACGCCGTGCGCGCAATCAATGCGGCCGTCCGTGCGTG
5A-1192740-T1-3_567	taCGTGACGGATCGTTCAGGACGCCGTGCGCGCAATCAATGCGGCCGTCCGTGCGTGCGTGGCGCTACTACTGTTCCCCCACTCGTCGCTGGCAAATTCATCAATTGATCACCTCGCCCT
5A-1192740-T1-3_630	GCTACTACTGTTCCCCCACTCGTCGCTGGCAAATTCATCAATTGATCACCTCGCCCTGCGGCAGCCTTTTCCTCCATCTCAAACTCGGCACTGTTTGCAATTGATCGCTCCTGTTACCAA
5A-1192740-T1-3_693	GCCTTTTCCTCCATCTCAAACTCGGCACTGTTTGCAATTGATCGCTCCTGTTACCAAGCGTTTGAGCTCCATAATCATCGCTCCAACGTACGTAGCATTGGTTTCCTTGGCATTACGGTC
5A-1192740-T1-3_756	GAGCTCCATAATCATCGCTCCAACGTACGTAGCATTGGTTTCCTTGGCATTACGGTCCTGGCCGCCCAGGCCTGCCCCCATACCGGCCGGGCGGGCGGACCcaaggcacatcaccggcac
5A-1192740-T1-3_882	gcacggacgAGCGCAGAGGCTCATCACAAGTCACGAACCGGCCCGCACAGTCGCCtccgtccgtccgtccgtccgtccgcccTTGATTTTCTTTCTCTCTCATTAAACCCCACCCCACCC
5A-1192740-T1-3_945	tccgtccgtccgtccgcccTTGATTTTCTTTCTCTCTCATTAAACCCCACCCCACCCCTGCCCTGCGGCGGCActctctctctctctctAAACCCCACGCGCCCTGCTCCCTCCCTCG
5A-1192740-T1-3_1134	ACCTgctctgctctgctccgctccgctcctccccagcccacgcccccgcctcatccctcgcgtgcacgcacgcgctcgctgcgccgctcAGATTCCTCGCATCACCGCCGGCCAGC
5B-1323430-T1-3_315	CAAAGGGAGGGAGCAGGGCGCGTGGGGTTTAGACTTTAGAGAGAGAGTGCGAGTGCAGCCACAggggtggggtggggtggggtggggtTTAATGAGAGAGAAAGAAGATCAAGGGAGGAC
5B-1323430-T1-3_504	GCGATGTGCGATGGGTCCGCCCGCCCACCCGGTATGGGGGCTGGCCTGGGCGGCCATGACCGCGAGCCAGCCCAACAGGGAGCCGCCCGTAATGCCAAGGAAACCAATACCCCAGTAGTA
5B-1323430-T1-3_567	GAGCCAGCCCAACAGGGAGCCGCCCGTAATGCCAAGGAAACCAATACCCCAGTAGTAGTACGTTGGAGCGATGATTATCGAGCTCAAACACTTGGTAGCAGGAGTGATCAATTGCAAATA
5B-1323430-T1-3_630	TGGAGCGATGATTATCGAGCTCAAACACTTGGTAGCAGGAGTGATCAATTGCAAATAGTGTCGAGTTTGATGATGGAAAAGGCTGCTGCAGGGTGATCAATTGAATTTGCCAGCGACGAG
5B-1323430-T1-3_693	AGTTTGATGATGGAAAAGGCTGCTGCAGGGTGATCAATTGAATTTGCCAGCGACGAGTGGGGAATAGTAGCGCCACGCACGCCATCACGTACTAGCAGTACGTATACCTCACACGAGCGT
5B-1323430-T1-3_882	GAACGAGGCCCTCGATCGGGCTTGCTTGGACAGAGACGCAACCACACTGACACACACACTGACGCGTCCACGTCCGCCCGGGGGCCCAGGATAAGATGGGCGCGCTCGCTACTGGCTAGC
5B-1323430-T1-3_1071	GTTGCCCGATTTGAAACGGGCAGGCCGGATCAATCAATGCCTTCCCCCGctcgtccccacccacccgcgcctctcctcgccgcttcctcctctccGGCgcctgcctgcctgccgctctgg
5B-1323430-T1-3_1134	accgcgcctctcctcgccgcttcctcctctccGGCgcctgcctgcctgccgctctgggcctgctgcggcctgccGCACGAGGGAGGGGAGGGGACGAAGGAAGGGGTGGTTCCATTTCGG
5B-1323430-T1-3_1386	TTCTGGGGATGGGCGAGAGCAGGGGCTACGCTCTTCATCCCAGGGTCGTTCCGTTCCGAATGCAAGTGTGCTCTTCTGTGTTTCGTCTTCCCCAAGTAAGGCCAACTCCACCGCACGATC
5D-1456550-T1-3_122	TGGCCGGCGGTGATGTAATAAGGAATCTGAGCAGCGCAGGTGAgtgcgtgcgtgcgtgcgtgcgtgcgtgcgatgcgAggagatggaggcgggggacgggggcgggggcgggggcgtgggctgg
5D-1456550-T1-3_366	GGGTTTagagagagagagagagtgcgagTGCAGCCGCAGGGGTGGGGGGGGGTGGAGTGGGGTTTAATGAGAGAGAAAGAAGATCAAGGGAGGACGGAGGAGGCGACTGTGCGGGCCGGT
5D-1456550-T1-3_481	CCGGTTCGTGACTTGTGATGAGCCTCTGCGCTCGTCCGTGCCGGTGAATCGGTGATGTGCCATGGGTCCGCCCGCCCGCCCGGTATGGGGGCTGGGCAGCGAGCGCGGCCGTGACCGTGA
5D-1456550-T1-3_1201	GTGCCATGGGTCCGCCCGCCCGCCCGGTATGGGGGCTGGGCAGCGAGCGCGGCCGTGACCGTGAGCCAGCGCAACGGCCGCCCGTAGTGCCAAGGAAACAAATGCCACTCCAGAAGTATG
5D-1456550-T1-3_1265	GCCAGCGCAACGGCCGCCCGTAGTGCCAAGGAAACAAATGCCACTCCAGAAGTATGTTGGAGCGATGATTATAGAGTTCAAACACTTGGTAGTAGGAGTAGGAGCGATCAATTGCAAATA
5D-1456550-T1-3_1329	ATGATTATAGAGTTCAAACACTTGGTAGTAGGAGTAGGAGCGATCAATTGCAAATAGTGCCGAGTTTGATGGAGGAAAAGGCTGCTGCAGGGTGATGTGATGAATTGAGTTTGCCAGCGA
5D-1456550-T1-3_1393	TTTGATGGAGGAAAAGGCTGCTGCAGGGTGATGTGATGAATTGAGTTTGCCAGCGACGGGTGGGGAATAGTACCGCCACGCACGGACGGCCACATTGATTGCACGCACGGCGTCCTCACC
5D-1456550-T1-3_1457	GAATAGTACCGCCACGCACGGACGGCCACATTGATTGCACGCACGGCGTCCTCACCGATCCATCACGTACCAGGAGTACGTACGTGTAGCACCACAAGCGTGACGGTGGCCGGGGTGGCG
3A-0704020-T1-4_0	CTCCTGGGTGTCTTAGCTTGAGTGATCGATGGATCGATCTCTGTGTCTTTGTGGCTCTTCTCTTGCGGATGGTGGGCTATAAATACCGGCGCCGTGCCTATGGAGAGAGGCTGCGATCGA
3A-0704020-T1-4_128	CAGGCAGACAACTAGCTAGCTAGATGGCGCGACCGACGACTTGGGGTTGGGGGAGAGAGACTCAAAGATTCTTGTTTGATTTTGCCGTACGTGCATTATGATCATGGCAGTACGTACCT
3A-0704020-T1-4_192	AAAGATTCTTGTTTGATTTTGCCGTACGTGCATTATGATCATGGCAGTACGTACCTACAGTGAATGCACGAACTGTGGCAAAAGAACACGAGATACGTACGTGCAGAGATCTTGCAAATC
3A-0704020-T1-4_256	TGCACGAACTGTGGCAAAAGAACACGAGATACGTACGTGCAGAGATCTTGCAAATCTATGCTGAATGAATACCGTACAAACAGCGGCCCAGCGAGCTAGCAAGTTGCACCATGTAGCCGG
3A-0704020-T1-4_320	ATGAATACCGTACAAACAGCGGCCCAGCGAGCTAGCAAGTTGCACCATGTAGCCGGTCCATCCTGCTTGCGTAGGTACGTGTGATAGTACATTTTTTTTCTTTTCTTTTGAATGATCTC
[illegible]	[illegible]

Trait&gene no.	Gene	Homoeologues	IWGSC gene ID (refseq1.1)	WGIN Promotome ID	Orientation	ATG	Target Sequence	MYbaits	MYbaits coverage (%)	MYbaits ID	MYbaits sequence
T2-1	TaGT43_2	ABD	TraesCS4A02G107400	4A-1006230-T2-1	forward	124,205,364	GACCAAGACGAAACTTCTTACACTTTGGTGTGGTTGGGGAAGTGTGCCCGTTAGTGCGTCACTTGCCCGCA	18	74.4	4A-1006230-T2-1_128	CCCTCATGCTGCTTCCT
T2-1	TaGT43_2		TraesCS4B02G197000	4B-1071300-T2-1	reverse	424,289,845	GAAGCAGACCACCACCGACGCCTCCCTAGCCGCAGCAGACGACGATCTCCCTGGCGCTCTCGAGAGCCTC	17	70.6	4A-1006230-T2-1_192	ACCACTACCAAATTAAA
T2-1	TaGT43_2		TraesCS4D02G197300	4D-2073850-T2-1	reverse	342,090,931	GAAGCAGACCACCACCGACGCCTCCCTAGCCGCAGCAGACGACGATCTCCCTCGCGCTCTCGAGAGCCTC	17	70.6	4A-1006230-T2-1_256	CGCATGGAGTCAGTCA
T2-10	TaXAT2	ABD	TraesCS1A02G351900	1A-0015790-T2-10	reverse	536,355,695	AAAGCAGGAAGTTGAGCATCGTTTCAGGGAGGCCAAATCCGTTGCAATCTGGAGCCCGTGGACGGGATC	8	45.4	4A-1006230-T2-1_384	CGAGCATCCATAGCAT
T2-10	TaXAT2		TraesCS1B02G371300	1B-0119840-T2-10	forward	600,958,175	ACGTGCTTGGTTGTTGTTTAACCTCTGGCGAGCCGCGATGCACTCACGCACAGAAGGATGTCTCCCGTTTT	14	65.9	4A-1006230-T2-1_448	GCAAACGTAGACAAAA
T2-10			TraesCS1D02G358500	1D-0233520-T2-10	forward	442,331,841	TCCTCTGCTCAGTTATTAATTATAATAATAACCAACGAGCGTCGCTGTTGTTTAATCTCTCGCGAGCCGCGAC	18	56	4A-1006230-T2-1_512	GCCTCTTTGCTCAATT
T2-11	GPT1	A no BD	TraesCS7A02G287500	7A-1843160-T2-11	forward	342,806,611	ACCACCCCCCCTCTCCTGCAAGCCGCAGCCTCTGGCTTCTCTGTTCTATATTTCATAAATGGATGCGAAATCT	8	49.9	4A-1006230-T2-1_576	ATCTGTTTGATACTTTA
T2-12	TaGT43_1	AD	TraesCS7A02G441400	7A-1791360-T2-12	reverse	635,497,069	CGCCGCCGCGGCCGGATCTGGCGGACTCCCTCTGCCACCGCCGCCGCCGACGCCGACTCCGAGTCCCGTG	11	53.4	4A-1006230-T2-1_640	AATCCAAAAGATTTTG
T2-12	TaGT43_1	no B	TraesCS7D02G430700	7D-2009540-T2-12	reverse	550,098,989	CGCCGCCGCGGCCGGATCTGGTGGGCTCCCTCCGCCGCCACCGCCTCGCCCTGCGGATCAATCGGCCGCA	17	72.7	4A-1006230-T2-1_704	ACGTATCGTCTGAAAC
T2-13 & T3-25	GPT2	ABD	TraesCS2A02G220400	2A-0338910-T2-13	forward	208,109,358	TCAGTCTAGTGGATACGACAGCAAGACGTCACCCTAAACAATTTAATATCAGAATTATTTTAGGGGAACCA	19	84.7	4A-1006230-T2-1_768	GAAAATCTGAGGGATG
T2-13 & T3-25	GPT2		TraesCS2B02G245900	2B-0461810-T2-13	forward	250,065,630	GCGACGGCAGGAGGCAGAAACCCTAACTTTTTTGTGGAGGAAGGAGGAGGCGGCGGCTAGCTTTGGCTC	23	89.9	4A-1006230-T2-1_832	CTACTTTTTCGAATGAA
T2-13 & T3-25	GPT2		TraesCS2D02G226000	2D-0601440-T2-13	forward	194,187,709	GAAATGAACAAAGACACAATTTAACCTATTCAAATGGCTAAGTAGGCGAAAATGAGAACTCAGGCGAAAA	24	93.6	4A-1006230-T2-1_896	CTCTTCGTATCTACATA
T2-14	TaBAHD1	ABD	TraesCS3A02G111400	3A-0705780-T2-14	reverse	78,903,052	GGCCGCTCCCGCTCGGCTCTGCTCTGCTATGCTCTGGGCTTCTGTGAGGCGTGTGTGTGGAGTCTTTGGGA	15	69.6	4A-1006230-T2-1_960	TGGCTATAATCTTTGTT
T2-14	TaBAHD1		TraesCS3B02G132400	3B-0810200-T2-14	forward	114,877,779	ATAAGGTACACTCTAAAGCAATCAAGGTGCTCGTTACTCATTGGGCATCGACATGGGAAGGGAGAAAGTAC	16	70.1	4A-1006230-T2-1_1024	GACTTGGAGCTTATAG
T2-14	TaBAHD1		TraesCS3D02G113600	3D-0924320-T2-14	reverse	67,544,524	GGCCGCTCCCGCTCTCGCTCTGCTCTGGGCTTCTGTGAGGCGCGCGTGTGTGTGGAGTTTTTGGGACG	1	7.1	4A-1006230-T2-1_1088	ATTAGTAGTATTGTTTC
T2-15	MT	AB	TraesCS2A02G436100	2A-0288920-T2-15	reverse	688,244,559	GGCGGGCGCACCGGAGGTGGATTCAGGCGGGGCGAGGTGGGCGCTTCAGGAGGAACCTGCGCCGTGTG	6	29.2	4A-1006230-T2-1_1152	GGATATATGGTCCGGC
T2-15	MT	no D	TraesCS2B02G457100	2B-0440210-T2-15	forward	651,921,054	AATATAAACAACTTACTGATTGCAAAAGGAGGCCAAAATCATTTCAAGTTGGTGATAAGGTGATGGTCTACC	10	43.1	4A-1006230-T2-1_1216	GAGAGGGAGATGGTG
T2-16	TaBAHD3	ABD	TraesCS3A02G220800	3A-0642540-T2-16	reverse	408,949,425	GGTCTCGTGGGTTGGTGGGGGAGCGCGCTGGTTGTTTGCTTTGGCGCTTCCCCACTAGGAATGATGAATG	2	10.8	4A-1006230-T2-1_1280	CAACAATCCATTCATCA
T2-16	TaBAHD3		TraesCS3B02G251100	3B-0752390-T2-16	reverse	401,413,669	GGTCTCGTGGGTTGGTGGGGGAAATCAAAATGGGAGCGCGCTGGTTGCTTGCTTTGGCGCTTCCCCACTA	7	32.9	4B-1071300-T2-1_320	CCAACCACATCGAATCT
T2-16	TaBAHD3		TraesCS3D02G231700	3D-0855390-T2-16	forward	317,551,047	GTAAAATGCACCTCCGTTCCTTGGCAAGTTTGAGCTTCTCCATCTGAACCTCCAACCTAGCACTCTCTTCTAC	1	7.1	4B-1071300-T2-1_384	GACGGATTCAGTCTAG
T2-17	NTT1	BD	TraesCS3B02G262600	3B-0711520-T2-17	reverse	421,240,338	ATGAGGACGGGATCGGCCGTGGGTTTCGGGCCCGAGACGATCCTGGCCGTTGGTTCGGGCTCTGGGGTT	15	66.4	4B-1071300-T2-1_448	CAACATTAAATGTAGAT
T2-17	NTT1	no A	TraesCS3D02G219900	3D-0837950-T2-17	forward	299,906,753	GGTGGAAGGCAGCTCTAATGCCGAAGAGTGGCCGTCTTACTCTAGTGCAATCTGTGCTGTGTGCCATGCCT	2	14.1	4B-1071300-T2-1_512	ATATATACCCGAATGTA
T2-18	TaGT61_11	ABD	TraesCS7A02G236200	7A-1836660-T2-18	reverse	209,167,605	GCCGTCGAGGCGCTCGCGCAACGCCCGCCCGTCGTTTCTCTCCGCCCTTTTAATTTGTCTGTGTGCAGCAA	9	43.8	4B-1071300-T2-1_576	TACTAATGCTGGCTATT
T2-18	TaGT61_11		TraesCS7B02G134200	7B-1932650-T2-18	reverse	163,508,979	GCCGTCGAGGCGCTCGCGCAACGCCCTCCCGTCGTTTCTCTCTGCCTGCTTTTGTCTGTGTGCAAGGCTCG	12	64.9	4B-1071300-T2-1_640	AAAACAACATAGTCAA
T2-18	TaGT61_11		TraesCS7D02G236300	7D-2049830-T2-18	reverse	199,416,131	GCCGTCGAAGCGCTCGCGCAACGCCCTCCCGCTCTACCTTCTTTTGTCTGTGTGTAAGTTACAAGGCTCGG	7	42.8	4B-1071300-T2-1_704	ACAGATCCGAAATTAG
T2-19	NTT2	ABD	TraesCS6A02G178000	6A-1538560-T2-19	forward	199,806,953	TCATAGTAAAATAACACCATATGATACACATCAATCAACCCTCCAACGGTGACACGGGCCACGGCATTTTGG	10	47.5	4B-1071300-T2-1_768	TAAGCATACATATTTAC
T2-19	NTT2		TraesCS6B02G206800	6B-1667080-T2-19	reverse	260,551,471	GGGGACGGAGAAGGTGGGAAGGAAGATTCCCGGGATGGAGGATGGAGGAGATAAGGGTGAGGAGCTC	7	36.2	4B-1071300-T2-1_832	AAGGACAATTTTGCAA
T2-19	NTT2		TraesCS6D02G166400	6D-1731330-T2-19	reverse	147,885,932	GGGGGCGGAGAAGGTGGGAGAATTCCCGGGATGGAGGATGGAGGAGATAAGGGTGAGGGGCTCGGGC	11	51.3	4B-1071300-T2-1_896	ATCCAATGACCCCAAAT
T2-2 & T3-13	PGI	ABD	TraesCS5A02G247200	5A-1237760-T2-2	forward	461,487,242	TACTGAAGCGTTTAACACATCACCACAGTGCAGAGGAGGCAATCGCTTTCGGGTTTTTTTTTTTGAGACATA	18	80.9	4B-1071300-T2-1_960	CGAATGCCTTCGATGG
T2-2 & T3-13	PGI		TraesCS5B02G244300	5B-1362930-T2-2	forward	425,878,428	CTAGTAGTCTATCATAGTCGGAAGGGGGCAGTCGCTTTCCTAAATGGCCATTAGCGCTGAGCATGAGGCAG	14	59.3	4B-1071300-T2-1_1024	CGACTGCACACGAGAT
T2-2 & T3-13	PGI		TraesCS5D02G253700	5D-1440810-T2-2	forward	360,539,840	TGTTTGTTATTTTGTCCCTCGGCCAGCCAGGCTGTTGGTCGCCGCTGCAATGAGAATCGCCGCCCCCTCGGC	21	88.9	4B-1071300-T2-1_1088	TCTACCAAATTAATGTA
T2-20	TaGT61_6	ABD	TraesCS3A02G494800	3A-0655890-T2-20	forward	721,085,862	TATTTGTTATTCATGAATTTGCTACAAATGTACTCGCGATGATATGCTTTTTGTTGGATTTCATAAACTAACATA	21	95.5	4B-1071300-T2-1_1152	TCAGAGTGTTGCGTTG
T2-20	TaGT61_6		TraesCS3B02G556500	3B-0716390-T2-20	forward	790,521,291	AAGCGTTTAAAATCAAACTCATATGGAATTTTTGAACAATAGCATTGTTTCTCAGGGATCAACGAGAATTTTG	17	83.8	4B-1071300-T2-1_1344	TTGTTCGGTGATGGGT
T2-20	TaGT61_6		TraesCS3D02G501900	3D-0875400-T2-20	reverse	590,525,827	GCCGACGTTCGGTTGGAGAAATGGAAACGGGATGGGTGCAGCTTCGCGCGGTCGCCCTTGTCGCGTGCC	19	86.8	4B-1071300-T2-1_1408	GCACGCATGGACAAAG
T2-21	OsBT1-2/PANT1	ABD	TraesCS1A02G115500	1A-0063880-T2-21	forward	120,002,880	GTTCATGAAGCTGCAATGATGTGGGAATCAGAAACTTTGTGGCAGGCCATGACATGTTGTGTTATTTTGCAT	9	47.1	4B-1071300-T2-1_1472	GGTGGTGGGTTTTAAA
T2-21	OsBT1-2/PANT1		TraesCS1B02G135400	1B-0178860-T2-21	forward	172,378,739	CCCTCATTGATGACGAGGGCGTCGATGCGGGTGCGGCGCCCAAGTGGCGTCTCCTGCGTCTCCGGCTTGA	1	7.1	4D-2073850-T2-1_320	ACACTGCCCCAACTAC
T2-21	OsBT1-2/PANT1		TraesCS1D02G116900	1D-0263790-T2-21	forward	112,767,428	CAATAATCTTGTGGAGCATATGTGGACCAAAGAGCTAATGTTTGAGTTGTGCAATTAAAATAAATTTTATGTA	7	39.5	4D-2073850-T2-1_384	TGGATATCAGACGAAT
T2-22	TaGH10_1	ABD	TraesCS2A02G122200	2A-0343600-T2-22	forward	71,622,148	TCTTCTGGCAGCACGTGCATGATTCATTTCACGCCCAAAAGCACATTGTGCCATATGACATGCACATAATTCA	45	85.3	4D-2073850-T2-1_448	CACGCACTACCAAAAT
T2-22	TaGH10_1		TraesCS2B02G144300	2B-0470790-T2-22	forward	110,460,917	GTATGAGACGACTTAGAGATTTGCAAAGTTCAGGGGGAGAAATCTCCTGAAAATATAATCCTTTTAGTTATC	9	38.7	4D-2073850-T2-1_512	AATTTCTATCTTCAAGC
T2-22	TaGH10_1		TraesCS2D02G124900	2D-0598390-T2-22	forward	73,019,399	TGGCACACATGCCAACATACAAAAAAGATGACGCTCTCGACCATAGATCAACCATCTAGCTGAAGCCTCATC	21	70.7	4D-2073850-T2-1_576	TCATCATTATTTTGATGA
T2-23	OsBT1-3/PANT2	ABD	TraesCS7A02G495700	7A-1787960-T2-23	reverse	685,907,343	GGGTGGGTGAACGTCTTGGCCGGCCTCAGAGTGCAAACGCACAATCAGAGGTGGACGGCCGCCCTGCAC	15	68.5	4D-2073850-T2-1_640	CTGTTTTGAAACTTGG
T2-23	OsBT1-3/PANT2		TraesCS7B02G401600	7B-1901610-T2-23	forward	667,777,711	GCTAGCAAATTTATTTGCTGCAAGAGCGAACGTGCATATACAAATTCATCACATACCAAAGAACATTAACTAT	11	57.9	4D-2073850-T2-1_704	TTCTTCTTCCAAAGATC
T2-23	OsBT1-3/PANT2		TraesCS7D02G483000	7D-2005130-T2-23	reverse	593,178,720	GGGTGGGTAAACGTCTTGGCCGGCCTCAGACTGCAAACGCACAATCAGTGGTGGACGGCCGCCCTGCACT	10	52.9	4D-2073850-T2-1_768	CCGTTAAAAATATGTAA
T2-24	TaGT61_13	ABD	TraesCS7A02G235500	7A-1805710-T2-24	forward	207,547,425	GAGAGAAACTGCGTCCGGATCGCTCGGCGGACGGTTACATGCCCATATTGGATGACACAACACTTTGAGAC	13	65.4	4D-2073850-T2-1_832	GGGAGAGTAAGATGTG
T2-24	TaGT61_13		TraesCS7B02G133900	7B-1928540-T2-24	reverse	162,925,002	TCCCGAACCTCCTCGGCTCGCCCCTCGCAGAGCTCCGGGACTTCATCTCCTCCGGGAGTAGTGGTTCTTTGT	10	54.1	4D-2073850-T2-1_896	CCAACAATTCACTTTCT
T2-24	TaGT61_13		TraesCS7D02G235600	7D-1187590-T2-24	forward	198,500,393	TCCGCTGCCCATCATCGAGCCCAAGGCGGAGCCGGAGCCCATGCCGACGCGGAAGCGCTCACCGCCTCCA	7	42.8	4D-2073850-T2-1_960	GGTGCACTTGGTGCAC
T2-25	PUL	ABD	TraesCS7A02G133500	7A-1829510-T2-25	reverse	85,621,344	TTTGTATGCGAGGAAGGAAGGAAGAAGAGAGAGCCTTTTCTTCTTCTTCCCTTTGTGCTTTTGCGGCCAA	24	99.1	4D-2073850-T2-1_1024	TGATGCCTTTTGCTCTA
T2-25	PUL		TraesCS7B02G034600	7B-1922330-T2-25	reverse	33,915,610	TTGTATCCAAGGAAAGGAAGGAAGGAAGAAGAAGAGAAAGACTTGTTTTTTTTTCCTTTGTGCTTTTGG	23	99.1	4D-2073850-T2-1_1088	TTCACGCTGTCGTTCGT
T2-25	PUL		TraesCS7D02G133100	7D-2054220-T2-25	reverse	84,274,298	TTTGTATGCAAGGAAGGAAGGAAGGAAGGAGGAAGAGAGAGATTTTTTTTTCCCTTTGTGCTTTTGCGG	25	99.5	4D-2073850-T2-1_1152	CCGAGGGTCATATCTTC
T2-26	WMAI	BD	TraesCS6B02G008200	6B-1669490-T2-26	forward	5,282,957	GGGGATTCATTTTGCCGAACCTCTACAAGTTCAGAGTTTAAAATGCACCTTTTCTAAACAGAAGCCGTCAGT	23	92.4	4D-2073850-T2-1_1216	GCTATCGTGTGTTGTCG
T2-26	WMAI	no A	TraesCS6D02G000200	6D-1733450-T2-26	forward	52,514	ATTGCTTTTGTAGGGAAACAACATCCAATCAGGTTCCACTTTTGTCGAAATAGTTTCCAGGTCAAGAACATG	26	100	4D-2073850-T2-1_1408	AGTTGGTGCATGTCCA
T2-27 & T3-27	PHOL	ABD	TraesCS5A02G395200	5A-1199980-T2-27	reverse	590,605,208	TGGCGGCAGTGGTCGCGCTGACGGTGGTGTGCAGCGCGCGCGATGCGACGACGCGAGAGTCACGCGGC	19	83.5	4D-2073850-T2-1_1472	ACGCCATATTAGGATAA
T2-27 & T3-27	PHOL		TraesCS5B02G400000	5B-1299180-T2-27	reverse	577,306,200	CTGAACTGCAACTTTAGAGGGGAAGTCCTCCCAGTTGAGAGAATCACCAGCCCTCGACTCAAAACGAGAA	20	85.2	5A-1237760-T2-2_0	TACTGAAGCGTTTAAC
T2-27 & T3-27	PHOL		TraesCS5D02G404500	5D-1428360-T2-27	reverse	469,560,617	TGGTGGCAGTGGTCGCGCTGACGGTGGTGTGCAGCGCGCGCGATGCGACGACGCGAGAGTCACGCGGC	20	80.7	5A-1237760-T2-2_64	GAGACATAGGCAATGC
T2-28	WDAI-1	BD	TraesCS3B02G111100	3B-0717810-T2-28	forward	77,315,443	ATGCTCATTGGTTGATGCAGGTTAAGATGTAGCCACTCTTCTTTTGTCACCCTGCTTCTTCATACTCCGCTTG	17	85.9	5A-1237760-T2-2_128	CGCAGTCGGAAGGAG
T2-28	WDAI-1	no A	TraesCS3D02G096000	3D-0935160-T2-28	reverse	48,732,516	TCTTCCTCTATGCACAGACTTTCTGCCTCTAATTTAGTACTATCTTAATTAGTTGTGTATATGGATAACTACACT	11	65.3	5A-1237760-T2-2_192	AGTTGGGGTTTCTGAC
T2-29	PHOH	ABD	TraesCS3A02G366400	3A-0650140-T2-29	reverse	615,705,717	GGCTAAAGCGTTCAGAACTTCTCCACCGGCCCATTTCCGCCTATCACATGATAAAGCAAACAAACATATCAAT	11	61.4	5A-1237760-T2-2_256	GGCAAGAGAGGCGGA
T2-29 & T3-26	PHOH		TraesCS3B02G397900	3B-0753850-T2-29	reverse	628,122,398	CGTGGGGTGGGAGATGGCGGTGTGTGTGGGGGAGGTGGTGAGTTCGGGGCGGGCACCGAATGGGGAC	8	39.1	5A-1237760-T2-2_320	CGGCCATCGGCTTCTT
T2-29 & T3-26	PHOH		TraesCS3D02G359200	3D-0841320-T2-29	reverse	472,951,384	CGTGGGGTGGGGAATGGCGGTGTGTCTGGGAGGTGGTGAGTTCGGGGCGGGCACCGAATGGGAACGGT	6	39.1	5A-1237760-T2-2_384	GAACACAGAGGGGGC
T2-3 & T3-12	PGI	ABD	TraesCS1A02G040600	1A-0068020-T2-3	reverse	22,301,216	GGCCGGGCGGACCTGGCGGAATTGAGGCGGAGGATAAGCTGCCTTTAATCCCAGCAAAAAAATAGTAATAA	2	14.1	5A-1237760-T2-2_448	TCGGCCAGTGTTATCA
T2-3 & T3-12	PGI		TraesCS1B02G053600	1B-0166630-T2-3	reverse	33,990,120	GGCCGATGCGGCGGAGGGCCGGGCGGACCTGGCGGAATTGAGGCGGAGGATAAGCTCAGTTTAGTCCCA	15	68.5	5A-1237760-T2-2_704	AGACTAACCAAAATAG
T2-3 & T3-12	PGI		TraesCS1D02G041200	1D-0260080-T2-3	forward	20,043,946	GTCCTGTGTGTCAGGCACAACCTAGGAACAGAATGTTTTGCCACTTAAAAGGACTACGTGTTTAATACAATA	10	47.5	5A-1237760-T2-2_768	CTGTATCGTGTTTTTGT
T2-30	WDAI-2	D	TraesCS3D02G095700	3D-0932870-T2-30	forward	48,543,331	TTAGAGTAACCCTCTAACCCATTATTGCCATTCTTATAACTATAGTGTGTCTTGCTAATTAACTAGGCCCCAATC	16	75.5	5A-1237760-T2-2_896	CCGCCGCAGGTGCGA
T2-31	DPE1	ABD	TraesCS2A02G159300	2A-0343000-T2-31	reverse	106,810,108	GCGGCAGGGGGGCGGGGCGGGGTGGCGGAGCGGGGATGGAAGGGGGGAGGAGGAAGAAGGGAGAT	14	76.3	5A-1237760-T2-2_960	TACGACGGCCACGGCG
T2-31	DPE1		TraesCS2B02G184900	2B-0472380-T2-31	reverse	159,896,289	GCGGCAGGGGGGCGGGGCGGGGTGGCGGGGCGGGGGATGGAAGGGGGGGAGGAGGAAGAAGGGAC	13	67.5	5A-1237760-T2-2_1024	GAATTGAATCGCTTTCC
T2-31	DPE1		TraesCS2D02G166600	2D-0595670-T2-31	reverse	110,669,860	GCGGCAGGGGGGCGGGACGGGGTGGCGTAGCGGGGAGGGAATGGGGGAGGAGGAAGAAGGAAGAG	8	43.3	5A-1237760-T2-2_1088	AAAACAAACGTCGCCT
T2-32	WTAI-CM2 & WTAI-CM1	BD	TraesCS7B02G072000	7B-1935150-T2-32	reverse	79,309,947	ATTATTCTTTCTAGCTTGGTCCGGTTTGTTGGTGTAGTTTCTTGAGGTTGTGCACTGTGCTGGTGGCTGTAAG	17	71.4	5A-1237760-T2-2_1152	GAGAGCTGCAGGACC
T2-32	WTAI-CM2 & WTAI-CM1	no A	TraesCS7D02G168000	7D-2082710-T2-32	reverse	118,157,335	GTTATTCTTTCTAGCCTTGTCCGGTTTGTTGGTGTAGTTTCTTGAGTTGTGCACTGCACTAGTGGTTGTAAGC	12	55.9	5A-1237760-T2-2_1216	CGCGGACGCGGACGC
T2-33		ABD	TraesCS2A02G123800	2A-0344470-T2-33	reverse	73,165,916	CTCTTCAGAGCAGGCAGGCAGAAACATGAGCGCAGCAAGGTTAATTACGAGCTGAATGCATCAATGGCAA	15	73.7	5A-1237760-T2-2_1280	ACACTTCTGTCGCAGT
T2-33	DPE2		TraesCS2B02G145700	2B-0494910-T2-33	reverse	111,462,132	CTCTTCAGAACAGGCAGGCAGAAACACAGGCGCAGCAAGGTTAGGAGCTGAATGCATCAATGGCAAACA	11	54.6	5A-1237760-T2-2_1472	CCGACGCTGACCTGTG
T2-33	DPE2		TraesCS2D02G126600	2D-0577070-T2-33	reverse	73,578,184	CTCTTCAGAGCAGGCAGGCAGACACACAAGCGCAGCAAGGTCAGGAGCTGAATGCGTCAGTGGGAAAAA	11	63.8	5B-1362930-T2-2_128	GATGCGACCAGGCAG
T2-34	WTAI-CM3	BD	TraesCS4B02G328100	4B-1063570-T2-34	reverse	618,654,873	GGTATTCTAGCCAAGTCTGGTTCGCTGGTGTAGTTCCTTGAGTTTATGCTGGATGCGCCACACATGCTCGCAT	17	70.6	5B-1362930-T2-2_192	GGTACGGACAATAACA
T2-34	WTAI-CM3	no A	TraesCS4D02G325100	4D-1137420-T2-34	reverse	485,484,917	GGTATTCTAGCCAAGTCTGGTTCGCTGGTGTAGTTCCTTGAGTGTATGCTGGATGCACCACACAATACATGCT	19	81.4	5B-1362930-T2-2_256	ACGATACAAATTTTTTT
T2-35 & T3-23	APL1/AGPL3	ABD	TraesCS5A02G472000	5A-1225500-T2-35	forward	649,187,425	AAAGGTATTTTTTTAGAAAAATGACTTAAATATTATGTTCTTTTTGTTAATGAACCTATTGAACTGGCAGGTC	18	80.9	5B-1362930-T2-2_320	AAGATAGAGCAAGCAA
T2-35 7 T3-23	APL1/AGPL3		TraesCS5B02G484700	5B-1286990-T2-35	forward	656,228,474	TTTTTTATTTTATGCAACGTTCCCCTTCAGAAATTGTAGAGAACTTAAATGATGTCCTTTTCTTAGTGGTGTGA	19	81.4	5B-1362930-T2-2_384	ACTTCTCGAGATCATAA
T2-35 & T3-23	APL1/AGPL3		TraesCS5D02G484500	5D-1438030-T2-35	forward	520,719,360	CATGGCAAATTCACAAAAAAAAAACTGCCAATTAGATCATGGCAAATGAGAGTGTGCAACCCATCCCGTACA	9	47.1	5B-1362930-T2-2_448	TGTGGGGCAGCCTGCT
T2-36	GSP	B no AD	TraesCS5B02G003500	5B-1385620-T2-36	forward	5,057,454	CGAAGAGCGCCGGGGAGGGCGAGGAGGAGGGGAAGAAGGGGAGCGGGACGGTGGTGAGGAGGACG	17	76	5B-1362930-T2-2_512	TATCCCAAAAAAAGAA
T2-37 & T3-16	APL3/AGPL1	ABD	TraesCS1A02G419600	1A-0022300-T2-37	reverse	576,374,703	TCGCAGTCCACCTGCAACTGCAACAAGATGAGAAGTTCAGAACGATCAGCAACCATGGAACCTTCAGAAAT	11	57.9	5B-1362930-T2-2_576	CACACATGCCGTCAGA
T2-37&T3-16	APL3/AGPL1		TraesCS1B02G449700	1B-0119450-T2-37	reverse	668,132,472	GGGAATGTCGATGAGCCTGTAACATCCTCCAATAGGAACCTGAGCAATGCAACAAAGGGAAAGGAACAAG	23	72.2	5B-1362930-T2-2_640	ACAACGGAGAAGCAA
T2-37 & T3-16	APL3/AGPL1		TraesCS1D02G427400	1D-0227010-T2-37	reverse	479,892,869	TCGCAGTCCACCTGCAACTGCAACAAGATGAGAAGTTGAGAACGATCAGCAACCATGGAATCTTCAGAATC	15	76.2	5B-1362930-T2-2_704	TAGCTAGCTTTCTCCGT
T2-39 & T3-24	APS1/AGPS1	ABD	TraesCS5A02G292900LC	5A-1192890-T2-39	reverse	375,579,322	CGGGGGCAGAGTGTGACGGGTCGAGGCGGTGGCAGCGACGGCCGAGAGAGGGCGAGGGAGTGAGGTC	13	58.8	5B-1362930-T2-2_832	GCTTCCAGAAATGCAC
T2-39 & T3-24	APS1/AGPS1		TraesCS5B02G175800	5B-1354680-T2-39	reverse	321,624,817	CGGGGCCAGAGAGTGACGGGTCGAGGCGGTGGCAGCGACGGCCGAGAGAGACCGAGGGAGTGAGGTG	4	24.7	5B-1362930-T2-2_896	GGATTCCCGTTTCCAG
T2-39 & T3-24	APS1/AGPS1		TraesCS5D02G182600	5D-1448600-T2-39	reverse	283,493,724	CGGGGGCAGAGAGTGACGGGTCGAGGCGGTGGCAGCGACGGCCGAGAGAGGGCGAGGGAGTGAGGTC	6	29.2	5B-1362930-T2-2_960	GCTTCAATACTTTCTGT
T2-4		ABD	TraesCS3A02G440800	3A-0642360-T2-4	reverse	683,254,111	CTATGAGTGTCGCCCAAAAGCAGAGCAAAGAATATACCAAGCTTGATAACAGGATCACATAGCACTTTGATT	24	78.2	5B-1362930-T2-2_1024	CCCACCTGCCGTTGGC
T2-4			TraesCS3B02G474900	3B-0756260-T2-4	reverse	723,507,534	CTATGAGTGTTGCCCAAAAGCAGAGCAAAGAATATACCAAGCTTGATAACAGTATCACATAGCACTTTGATT	19	69.6	5D-1440810-T2-2_0	TGTTTGTTATTTTGTCC
T2-4			TraesCS3D02G433400	3D-0865620-T2-4	reverse	546,477,962	CTATGAGTGTTGCCCAAAAGCAGAGCAAAGAATATACCAAGCTTGATAACAGGGTCACATAGCACTTTGATT	24	80.7	5D-1440810-T2-2_64	CCCTCGGCAACAAATC
T2-40	HMW Glu-1y	AD	TraesCS1A02G466500LC	1A-2139570-T2-40	reverse	508,925,603	TTCAGTGGACTGTCAGTGAATTGATCTCTATTTTGTGGTGCTCGGTGTTGTGGGTGATGATGAGATTGTGGG	16	83	5D-1440810-T2-2_128	GGAAAACAGTTCCTTT
T2-40	HMW Glu-1y	no B	TraesCS1D02G435700LC	1D-0214200-T2-40	reverse	412,219,631	CTCGGTGGACTGTCAGTGAATTGATCTCTATTTTGTGGTGCTCGGTGTTGTGGGTGATGATGAAATTGTGGA	13	68.7	5D-1440810-T2-2_192	ACGGCGTGCATTCGTA
T2-42	pinA	D noAB	TraesCS5D02G004100	5D-pinA-T2-42	forward	3,591,520	GCGGCACCATGGCTGCTGGTCGCGACGGAAGGGAGCTTCACACGCCGGCCACGACAGAAGGGAGCTCCA	16	65.6	5D-1440810-T2-2_256	GGGCAGCTCAGTGTCA
T2-43 & T3-30	SSI[c]	ABD	TraesCS7A02G120300	7A-1826470-T2-43	reverse	77,441,923	CTGGAGGTGAGCCCGCAGAAAACAGGAGGCGGGCGGAGGTGCGGACGGAAGGTGGATCACCCCGCTCG	12	55.1	5D-1440810-T2-2_448	GGGGTTTTTGCCCTGT
T2-43 & T3-30	SSI[c]		TraesCS7B02G018600	7B-1942520-T2-43	reverse	15,602,178	GGGTGCGGGCCCGCGGAGCAGAGGAGGAGGTGCGGACGGGCGGACGGTGGATCGCCCCGCGCGCTCG	14	62.6	5D-1440810-T2-2_512	AAGGCGGAGGAGGGT
T2-43 & T3-30	SSI[c]		TraesCS7D02G117800	7D-2043770-T2-43	forward	72,938,824	TGCGTCGTCCGCGCGCGGCTCCGGCGCTTGGCGCGGGGCCGCTACGTCGCCGAGCTCAGCAGGGAGGGC	13	62.1	5D-1440810-T2-2_576	TGAGAAATGAGCCTAC
T2-44	pinB	D noAB	TraesCS5D02G004300	5D-pinB-T2-44	forward	3,609,672	CTCTCCGGGAGAAAGTATTTTCGAGAACCTAAAGTTAGATTTTTAGCAAAATTATTTAAACCGAATATAAGAC	11	43.5	5D-1440810-T2-2_640	TACTACCGGTCAAGATA
T2-45 & T3-31	SSIIa/SSII-3	ABD	TraesCS7A02G189000	7A-1809640-T2-45	forward	145,464,919	TGTCCAAGAAATGGGATGGAGAATAGATGCATGTACGTGTCCTGGATATGAAATTTTTTTGAGTATGAGAGA	14	69.2	5D-1440810-T2-2_704	TCATGGGACTTCTCGA
T2-45 & T3-31	SSIIa/SSII-3		TraesCS7B02G093800	7B-1945350-T2-45	forward	107,604,543	AAATATTTTGAAATGTTAATCAAGGATTTGAGAAATGCTAAACGTGTATAGAAAAAATGTAGACCATGTATTA	14	65.9	5D-1440810-T2-2_768	AGCAAAATAGGCTTGG
T2-45 & T3-31	SSIIa/SSII-3		TraesCS7D02G190100	7D-2059200-T2-45	reverse	144,137,287	GGATCGGGGCGGGAGATGGTACGGTTCGCGGGTTGGTCCGCCCAGAGCGCGGCGCAGGCGGAGGTGGT	10	59.5	5D-1440810-T2-2_832	CGTGTGTTTTGTGTTAG
T2-46	wbm	B no AD	TraesCS7B02G746900LC	7B-1877640-T2-46	forward	707,910,164	AGCTTGTCAAAAGAAAAAAAATAGGAAATTTTTTGGTTTGACATAACTAGATTGCCAAATTCTAGCTTGATA	19	88	5D-1440810-T2-2_896	AGGTTTGGATGAAGGA
T2-47 & T3-32	SSIIb/SSII-2	AB	TraesCS6A02G307800	6A-1512520-T2-47	forward	542,254,722	GTTAGCGACCGAGATTGACAGGGTGATCACTCAGGACCGCTCGGGCTCGATAAACCTTGACTCCTTGCTCC	13	56.2	5D-1440810-T2-2_960	CGAGACCCATCACAGA
T2-47 7 T3-32	SSIIb/SSII-2	no D	TraesCS6B02G336400	6B-1619110-T2-47	forward	591,576,717	ATTATTTTGCTTGGGCTTTTGAAACATTCGGTGTTTACTCTGTCAAATCAGCGTACCGTGCTCTCGTGAATCAT	11	57.9	5D-1440810-T2-2_1024	ACAGAGATGCAAAACC
T2-48	LMWG-1D1	BBD	TraesCS1B02G398500	1B-0112800-T2-48A	forward	629,652,723	GAAAAAGATTTTTTGATGGGTTTATAGAGTTTTTTTTTATCACGCTTCCTTGTGAATTAGTCATTAGTAATTAT	20	87.3	5D-1440810-T2-2_1088	AGAATCGCTCTCTCTCA
T2-48	LMWG-1D1	no A	TraesCS1B02G012400	1B-0153990-T2-48B	forward	6,060,472	CAAGCACTTGAATTGACAAGAAAGTGGGGGACAATTGATGTAGCTGATGCACTAGAGCTCCTCTCACCAGA	21	91.1	5D-1440810-T2-2_1152	CTTCCGTGGCAGAAAG
T2-48	LMWG-1D1		TraesCS1D02G000100	1D-0249790-T2-48	reverse	76,856	GCCACCCTGTTGAAGACAGACCTTCAGCCTTTCCCACTTATGGACCTCTCTGTTCCAGACATCATCCAATACA	18	73.2	5D-1440810-T2-2_1216	TTTCATCATCAGAGAA
T2-49	SSIIc/SSII-1	ABD	TraesCS1A02G137200	1A-0001960-T2-49	forward	221,695,822	GAAGAGAGCTTTATTGTTTTATCGTAAGAGCATGGTTAATAGTACAACCCACAATGGATATGTGAAGTTAAT	5	28.7	1A-0068020-T2-3_128	GGTAGGTGCGTACCCC
T2-49 & T3-33-2	SSIIc/SSII-1		TraesCS1B02G155700	1B-0101070-T2-49	forward	263,548,087	ATCGAAAAGAACAATTATGTGAACAGAAATGATCAACCAGCCTTGGGAAGAGAGTTTTATTCTTTATCGTAA	6	35.8	1A-0068020-T2-3_320	TAAATAAAAAGGGTTA
T2-49 & T3-33	SSIIc/SSII-1		TraesCS1D02G138100	1D-0205540-T2-49	forward	189,646,049	AGAGCATGGTTAATAGTATAACCCACAATGGGATATGTGAAGTTAATAGCCCGCTATTACAATAGTGAGTCAT	10	50.8	1B-0166630-T2-3_64	TCCCAACAAAAAAGAA
T2-5 & T3-17	PGM	ABD	TraesCS7A02G287400	7A-1821750-T2-5	forward	342,610,534	GTACGCCATTTTTGGGCGTGTTCTCGAAAAAATGATCCATGTGTGCTTTCTGTATGACTAAACTTGCTGCCAA	13	58.8	1B-0166630-T2-3_128	GAGCAATTGATCCAGA
T2-5 & T3-17	PGM		TraesCS7B02G183300	7B-1921060-T2-5	reverse	290,020,067	TGAGGTGGATGACGAGCTCGAGTCACGGGGCTCCCGTGGCGAGAGATCACGAGGGGGAAGGGAGAGAT	15	69.6	1B-0166630-T2-3_192	GGCTGCCGGAGAAGG
T2-5 & T3-17	PGM		TraesCS7D02G284900	7D-2042690-T2-5	reverse	305,834,137	TGCCGAGGGACAAACAATGAAAGTATATAGATATTGAGTGATGTGTGAGCAAACTGTTCTCTAATTTATAGG	9	37.2	1B-0166630-T2-3_256	gaagagaatagaagcggag
T2-50 & T3-34	SSIIIa/SSIII-2	AB	TraesCS1A02G091500	1A-0075220-T2-50	forward	84,002,707	GCACTTATTCGCAAAATTGCTTTTGTGTTCATATTTGACGTGAGGCTGGTCAAAAACAACCAAGTGTTAGCA	6	39.9	1B-0166630-T2-3_384	CCAGAGAACGGCTGG
T2-50 & T3-34	SSIIIa/SSIII-2	no D	TraesCS1B02G119300	1B-0177050-T2-50	forward	141,283,814	TGGAACCGCTTTAGAAAAACGATCGAGAGGTGACATCCTTTCCTAATCTCAACATCGGTGTCATCTTCTGGG	10	57.4	1B-0166630-T2-3_448	GAGAAGAGAGGCTGC
T2-51 & T3-35	SSIIIb/SSIII-1	ABD	TraesCS2A02G468800	2A-0288560-T2-51	reverse	712,581,222	TGCCCAGGGAAAAATCAAAACCCGACCCCCTCCGCTGATCCCTTCCCCCTCTCTCCCTCCCTCCCTCCTCCCT	18	86.4	1B-0166630-T2-3_512	AACCAAACCAGGGGC
T2-51 & T3-35	SSIIIb/SSIII-1		TraesCS2B02G491700	2B-0423210-T2-51	reverse	690,034,415	TGCCGAGGGAAAATCAAAACCCGACCCCCTCCGCTGATCTCTCCCCCCTCTCTCCCTCCCTCCCTCCACGAA	17	76	1B-0166630-T2-3_576	GTACCGGCTGCGTCGC
T2-51	SSIIIb/SSIII-1		TraesCS2D02G468900	2D-0540290-T2-51	reverse	574,113,211	TGCCGAGGGAAAATCAAAACCCGACCCCCTCCGCTGATCTCTCCCCCTTCTCTCCCTCCCTCCCTCCCTCCTC	37	85.4	1B-0166630-T2-3_640	GTCACCGTTGACGCCT
T2-52 & T3-36	SSIVa/SSIV-1	ABD	TraesCS1A02G353300	1A-0010710-T2-52	reverse	536,963,702	CGTATCTGCTTGGGCGGCTATAAACCTAGACTCCAGCTCCCTAAGGGAAGCATCTAAAAGAGCACGCTCTTT	21	88.9	1B-0166630-T2-3_768	CATGAAGCGAGCCGTA
T2-52 & T3-36	SSIVa/SSIV-1		TraesCS1B02G368500	1B-0108420-T2-52	forward	598,877,339	TTCTGTTGTCTTTGGCGACACCCGCGACAAAGCTAGTGCACATGGAGCAGTAGGTCGGCGACATGAAGCA	6	35.9	1B-0166630-T2-3_1216	GTTGCCGTCTCGCTTTC
T2-52-1 & T9-67	SSIVa/SSIV-1		TraesCS1D02G303700	1D-0221570-T2-52	reverse	401,671,728	CACCGACCGGAGCGCCGCGGCTGCCCCGGCAAAAACCCACACACAGCAAACAAGGTCAGCTCAGCTGGG	11	60.1	1B-0166630-T2-3_1280	TGAGGAGGCAGGCCG
T2-53 & T3-28	GBSSI/waxy	A,AD	TraesCS4A02G418200	4A-0093880-T2-53	reverse	688,100,234	CGCGCGCGCAGGGTGTGGCTACCTGCAGCGAGAGACGACTACACTGATTGTTAGTGAGTAGTGGGATAAA	21	91.1	1B-0166630-T2-3_1344	ATAGGAGTCAGACTGA
T2-53 & T3-28	GBSSI/waxy		TraesCS7A02G070100	7A-1817020-T2-53	forward	35,766,248	CAAGCAAAAGAGAAAAAGAAGGGGCGAAAGGTGATACGCCCGGCCGTGTCGTCGTGCTGCAGAGGAAG	15	69.6	1B-0166630-T2-3_1408	GACCGAACAAATTCGA
T2-53 & T3-28	GBSSI/waxy		TraesCS7D02G064300	7D-2037380-T2-53	forward	35,596,364	AATCAATGAAGCAAAAGAGAACAAGAAGAGGCGAAAGGTGACACGCCCGGCCGTGTGCGTCGTCGTGCT	21	91.1	1B-0166630-T2-3_1472	AGCCAGCTTTTCATTTG
T2-54 & T3-29	GBSSII	ABD	TraesCS2A02G373600	2A-0273760-T2-54	forward	616,979,816	ACGGCTCCCGTGCCGCCTCCCATCCCCACGCGAGGGCCCGGCGCTTCACCATCGCGATTCACGCTCCCGTC	21	81.2	1B-0166630-T2-3_1536	TCCACCATATATCAAAA
T2-54 & T3-29	GBSSII		TraesCS2B02G390700	2B-0429850_T2-54	forward	555,321,220	CGCTACCCGTGCGACACCTCCCCCTCCTCTTCCCATCCGCAGGATAATTCCTGCCGCTCACCGCCAGCCGAA	17	70.6	1B-0166630-T2-3_1580	ATAAACCATAATTCAAA
T2-54 & T3-29	GBSSII		TraesCS2D02G369800	2D-0502660-T2-54	forward	474,502,378	CCGAACCGAGTCCGACGCACGCAACGCGCCGGTGCCGGTACGGTGGCGGTGCCCCGCCCCGGTTTGTCG	26	85.4	1D-0260080-T2-3_0	GTCCTGTGTGTCAGGC
T2-55	BEI	ABD	TraesCS7A02G549300	7A-1766630-T2-55	reverse	723,293,490	CGTCTTCCACGGCACGGCGGGGCGCCGCGGAGCTGTCAGCGTCGGCGGAGAATCTCCGCCGGGCTCGTG	17	76	1D-0260080-T2-3_64	ATACAATAAACTACAAG
T2-55	BEI		TraesCS7B02G472300	7B-1913520-T2-55	forward	728,624,346	GATGATGTGCCCATATTTGGTTTTCTGGGTGTAGTGTTGTCTTCTGCAGGTTTGTCTTAGTCGTGGTGTCCTA	21	88.9	1D-0260080-T2-3_128	AGGACTGCTGGTTGAA
T2-55	BEI		TraesCS7D02G535600	7D-1976400-T2-55	reverse	627,358,921	CGTCGTCCACGGCACGGCGGGGGTGTCGGCGGAGCTCGTGGCCCGGCGTGCTTTCGAGCGGCCGGAGG	22	88.2	1D-0260080-T2-3_192	TGAGCTGGTAGAAGCA
T2-56 & T3-39	BEIIa	ABD	TraesCS2A02G293400	2A-0289500-T2-56	forward	504,335,305	GCTCGGCGTCGAACCTCGAGTTTAATCCCGTGTCAACAAGGAGAACTTGTGGACTACATATCGCTACGTGG	24	71.4	1D-0260080-T2-3_256	AGCCTCCAACGCGTGT
T2-56	BEIIa		TraesCS2B02G309500	2B-0425980-T2-56	reverse	442,525,037	AAACCACAATGATATGTAACGTAAAGGAGCATGGAGGACGTGTATGATATTAATTTCTTGTAAAAAATAATAT	9	54.2	1D-0260080-T2-3_320	TTCAACAAGCCCTCAA
T2-56 & T3-39	BEIIa		TraesCS2D02G290800	2D-0515330-T2-56	reverse	372,934,730	CCCCGCGTAGTGATTGAGTCAGCCAGTCGCCCAGATCTCACTCAACCCGCCGGCCGAAATGCAGCGCGGA	15	71.8	1D-0260080-T2-3_1024	AGAGGCCCTAGGCTTT
T2-57 & T3-40	BEIIb/Ae	ABD	TraesCS2A02G310300	2A-0314520-T2-57	forward	533,894,113	CCTTCATGAACGACAATTGTCAAGACCATCTCCTATCCAAGAGGTAACAAAATCACTTTGATGCAAGACAAA	12	64.9	1D-0260080-T2-3_1088	CGCAGCCGGTGCATGA
T2-57-1	BEIIb/Ae		TraesCS2B02G327300	2B-0440100-T2-57A	forward	468,660,103	GTGGTGCCATGGTGGTGGTGGTTGTTGGCGGTAGCGAAGTGCCTGGTGCACACGGCTAGGGTTTCGGCG	8	42.1	1D-0260080-T2-3_1152	CCGGTTTGGTTTCCTTC
T2-57 & T3-40	BEIIb/Ae		TraesCS2D02G308600	2D-0512220-T2-57	forward	395,603,079	AGCTCCTGCGTGCAATTATACCATCCATGGGCATGACTACAACATGGGGTACTGTCTTTGTGACAATATCTATC	12	64.9	1D-0260080-T2-3_1344	ACGGCACGGACACGC
T2-58 & T3-42	ISA1	ABD	TraesCS7A02G251400	7A-1824860-T2-58	forward	235,460,722	GCAATTATTTTCATGGTGACGTGGGGTCACTGGAATTAGTTCCTACTGAGGTGGCATGGTTTGTTGATGTGG	6	32.5	1D-0260080-T2-3_1536	GCACCTACCGCGATCT
T2-58 & T3-42	ISA1		TraesCS7B02G139700	7B-1931390-T2-58	reverse	176,007,199	GCCGGGAACCCCGAGCGATCGGTGATTGATTTTGGGGAGGAGCGGCGAGGCGGGGTTATTATCGGTGG	21	81.2	1D-0260080-T2-3_1580	cttcctctccaatccgtccatc
T2-58 & T3-42	ISA1		TraesCS7D02G249500	7D-2040790-T2-58	forward	220,970,552	CCGAGACTGATCGGGTCTTCCCGGGAGAAGGTTTATCCTTCGTTGACCGTGAGAGCTTATAATGGGCTAAG	2	10.8	3A-0642360-T2-4_0	CTATGAGTGTCGCCCA
T2-59 & T3-43	ISA2	ABD	TraesCS1A02G247100	1A-0053840-T2-59	reverse	438,368,309	GGGGGCCTGCGCGGCGCGATCAGCCGTCGGGGAATTGCGTCGAGCAGTGGGCGGGCATGGGGGACTGT	9	47.1	3A-0642360-T2-4_62	CACTTTGATTGAAAGG
T2-59 &T3-43	ISA2		TraesCS1B02G257700	1B-0112170-T2-59	reverse	453,515,532	GTGGGGCTAGCGCGGCGCGATCAGCCGTCGGGGAATTGCGTCGAGCAGTGGGCGGGCATGGGGGACTG	8	31.3	3A-0642360-T2-4_124	ATGGCATGAATCATGAT
T2-59 & T3-43	ISA2		TraesCS1D02G246300	1D-0203340-T2-59	reverse	338,943,720	GGGGCCGGCGCGGCGCGATCAGCCGTCGGGGAATTGCGTCGAGCAGTGGGCGGGCATGGGGGGCTGTG	2	10.8	3A-0642360-T2-4_186	AATAGCATTTGGATGTT
T2-6	TaCSLF6	ABD	TraesCS7A02G298600	7A-1751470-T2-6	forward	404,469,377	ATGAACCGGCCAGGGGACCAACGCATTCCTTCTCTTCTCGTGAAGCATTTGCTCGCCAAAGCCAAAGCCAA	10	52.9	3A-0642360-T2-4_248	TGGAGGTGGAACGGC
T2-6	TaCSLF6		TraesCS7B02G188400	7B-1876170-T2-6	reverse	319,858,729	GGCCGTGGTCCTCAATGCACGCACTCTCCTTTACGACAAGGAGAGCAGCAGCAAGAATGGAGGGAGGC	10	50.8	3A-0642360-T2-4_434	GAAACAGCTAGAGAA

T2-6	TaCSLF6		TraesCS7D02G294300	7D-2011180-T2-6	forward	366,451,430	TCTCATCGCCTTCACCCTGTTCGTGATCTGGCGTATTTCCCACAAGAACCCTGACGCCATGTGGCTGTGGGT	6	32.5	3A-0642360-T2-4_682	CATCTTCCCGATCCCCA
T2-60 & T3-44	ISA3	ABD	TraesCS5A02G248700	5A-1227100-T2-60	reverse	462,873,670	CCAAATGCGGGGGTGCTGGGACGGGGTGGCGGGAGGGATTTGGCTCGGGGCCGGGGCTTAGTTATAGG	2	10.8	3A-0642360-T2-4_868	GGCCTATCCTAGGCTT
T2-60 & T3-46	ISA3		TraesCS5B02G246400	5B-1334970-T2-60	reverse	427,939,462	TGCGCTTTCCCTTTCCTGACGGTGTTACAGAATGAACATGCACTTTAGCAAAAATAATAACATGGTACAGAA	15	52.4	3A-0642360-T2-4_992	ACGAGGAGGGAGGGA
T2-60 &T3-44	ISA3		TraesCS5D02G255800	5D-1467800-T2-60	reverse	362,066,416	CCAAATGCGGGGGTGGTGGGACGGGTAGCGGGAGGGATTTGGCTCGGGACCGGGGCTTAGTTATAGGG	17	72.7	3A-0642360-T2-4_1178	TTTCTTTCCTGCTCCCC
T2-61 & T3-22	OsBT1-1	AB	TraesCS6A02G175100	6A-1573650-T2-61	forward	189,920,981	TTCAAACAGGACTTGCACTACCATGTAGACTCCTAGTGAATTACATCTTGATACATCAGGAAAGATTCAGTTC	12	57.2	3A-0642360-T2-4_1240	CTTGAACATGTCAACTT
T2-61 7 T3-22	OsBT1-1	**no D**	TraesCS6B02G210000	6B-1646110-T2-61	reverse	276,746,931	CTGATTCAACTCGAACACATAAAGGATTATTATATGCAAGGGCGAAAGAGCTAAGTTGTACAGTGACTAGTG	9	43.8	3A-0642360-T2-4_1302	TAGTAGTAGTAGTGATT
T2-7	PGM	ABD	TraesCS4A02G267000	4A-0943460-T2-7	reverse	579,438,751	TCGGTTGCTCGCCTTCGCTCCTCCTTTTTTTCCTTATTTTTTTAGGGAAATTCGGTGGGTCGCGAGCGAGCT	1	7.1	3A-0642360-T2-4_1364	GGGTCCTCTGTCTCTTT
T2-7	PGM		TraesCS4B02G047300	4B-1095320-T2-7	forward	34,715,153	ATATGACATAGAGTACTGAATGCAATGCTGAATTCATCTGCAATAGCTTCGCACTAAGTATGTTGTGTCATCTA	11	54.6	3A-0642360-T2-4_1426	GATGGAAATTATACCA
T2-7	PGM		TraesCS4D02G047300	4D-1162870-T2-7	forward	22,938,812	CGCCAAGCCTCCGCTGCTCCATGCCGCCAAGAGCCGCCGCCAAGAATATATAGAAAGAAACACCGCTCCAC	8	36.7	3A-0642360-T2-4_1674	CGCGGCATTGGGAAG
T2-8	TaXAT1	ABD	TraesCS6A02G309400	6A-1513690-T2-8	forward	545,320,589	AGCTTGAGCCTGCCCAGGTGCGGCCTCACGTTCTGCTTCTCCAGGTACACCGTCTTGAGCGCCTCCCACCC	4	21.6	3A-0642360-T2-4_1736	GAAAAGACGCTTTCTC
T2-8	TaXAT1		TraesCS6B02G339100	6B-1621980-T2-8	forward	597,558,447	GAGCGCCTCCAGGAACGTGAGCTTGAGCCTGCCCAGGTGCGGCCTCACGTTCTGCTTCTCCAGGTACACC	4	18.4	3A-0642360-T2-4_1798	TATACCAGGTGACTACT
T2-8	TaXAT1		TraesCS6D02G288800	6D-1690440-T2-8	forward	399,220,562	GCCTCCAGGAAGGTGAGCTTGAGCCTGCCCAGGTGCGGCCTCACGTTCTGCTTCTCCAGGTACACCGTCTT	4	18.4	3A-0642360-T2-4_1984	GTTAAACCCATGTGGC
T2-9	pGlcT	ABD	TraesCS3A02G036600	3A-0684980-T2-9	forward	20,016,371	AAAAGAAAAAGAAAGAAAGAAATATATCTGAAAATTCAACCACTAAAGGGTTCTTTGACAAAGTATTTTGC	12	61.6	3A-0642360-T2-4_2046	GTAGTCAAAGTGTATCT
T2-9	pGlcT		TraesCS3B02G047300	3B-0801600-T2-9	reverse	23,962,669	CCACGCCACGAGAGAGAGAGAGAAGGTCTGCCGTGCTGGCAACGATGTGTGCATTGGATAAGGATTTTTGTA	16	72.2	3A-0642360-T2-4_2108	CTCCAATATACATTTGA
T2-9	pGlcT		TraesCS3D02G043500	3D-0926560-T2-9-1	reverse	16,520,396	CTTAACGGGGGGCAGAAGGGGGACCTTCCTGCGATCGCACACCACCGCAGGAACTTTCAGCTTCCGTTAC	19	90.1	3A-0642360-T2-4_2170	GGTTGATGTTTTTTGC
										3A-0642360-T2-4_2232	TAGTTACATTTTCTACG
										3A-0642360-T2-4_2265	TATTTACATGGCAAGCT
										3B-0756260-T2-4_0	CTATGAGTGTTGCCCA
										3B-0756260-T2-4_63	ACTTTGATTGAAAGGA
										3B-0756260-T2-4_126	TGAATCATGATTGCCCT
										3B-0756260-T2-4_189	GTTTGAATTGAACCAG
										3B-0756260-T2-4_252	CTGGGGAAGTCTAACC
										3B-0756260-T2-4_315	TGTCTTTAGCAGCTGCT
										3B-0756260-T2-4_378	GCACAAACTGACAAGA
										3B-0756260-T2-4_567	GGCAACCAAGACCCG
										3B-0756260-T2-4_756	GGCCTGTCCTACGCCG
										3B-0756260-T2-4_819	GAGGGAAGGGGAAG
										3B-0756260-T2-4_1071	ACTTGGACATTCGACC
										3B-0756260-T2-4_1134	TTTTTCTCCCATTCTTT
										3B-0756260-T2-4_1197	AATTGATATTGTATTTG
										3B-0756260-T2-4_1323	CCCGGTGGAAGGTAA
										3B-0756260-T2-4_1386	CGACGATCGGAGGGC
										3B-0756260-T2-4_1575	TTTTATACTGTTCATTA
										3B-0756260-T2-4_1638	CCTGTCTCCGCCACCAT
										3B-0756260-T2-4_1701	ATAGTAATCTGAATTGT
										3B-0756260-T2-4_1890	ACATGCAAGCAGGAGT
										3B-0756260-T2-4_1953	AAGAGATGGCCACTTA
										3D-0865620-T2-4_0	CTATGAGTGTTGCCCA
										3D-0865620-T2-4_62	CACTTTGATTGAAAGG
										3D-0865620-T2-4_124	CATGAATCATGATTGCC
										3D-0865620-T2-4_186	TCATCTGGATGTTCAAT
										3D-0865620-T2-4_248	GGAGGTGGAACGCCA
										3D-0865620-T2-4_434	AAACAGCTAGAGAAAT
										3D-0865620-T2-4_682	CCATCATCCTCTCGATC
										3D-0865620-T2-4_806	CCTTGCTTGTCCCACCA
										3D-0865620-T2-4_868	CCGAGGCCGAGCTTGA
										3D-0865620-T2-4_992	GGCTGATTCTTCCCTCC
										3D-0865620-T2-4_1178	GACATTCGACCTCTCA
										3D-0865620-T2-4_1240	TCTCTTTTTCCCTCATT
										3D-0865620-T2-4_1302	GGTAAGTGATTAATTG
										3D-0865620-T2-4_1488	TCAACTCATTTCTGCCG
										3D-0865620-T2-4_1550	GGTAGTTGAACCACGC
										3D-0865620-T2-4_1612	TCTGCGTCTACGAGAA
										3D-0865620-T2-4_1674	ACGGGATGAATGAAAA
										3D-0865620-T2-4_1860	AACTGAGTGGGCGGG
										3D-0865620-T2-4_1922	accatgatcattttctcggtca
										3D-0865620-T2-4_1984	TTCATTAGTGATATTTT
										3D-0865620-T2-4_2046	CATAATAATTGCTGCCA
										3D-0865620-T2-4_2108	TTGAGATGCTCTCCAG
										3D-0865620-T2-4_2170	TGTTTACATGGCAAGCT
										3D-0865620-T2-4_2232	AGCAATCAATGTTAAA
										3D-0865620-T2-4_2269	AATTTGCACATAAGATG
										7A-1821750-T2-5_0	GTACGCCATTTTTGGG
										7A-1821750-T2-5_64	TGCTGCCAATAAAGCG
										7A-1821750-T2-5_128	CTAATTTCGTCGCACAA
										7A-1821750-T2-5_192	GTGAAGAGCATATGAA
										7A-1821750-T2-5_256	ACAAAGATATCCATGTG
										7A-1821750-T2-5_320	CCTAGAAAGCCAAAAA
										7A-1821750-T2-5_384	ATGTGCGCCTTTTTATA
										7A-1821750-T2-5_576	TCATCGAAGCATGCAG
										7A-1821750-T2-5_640	ATTTCCATTTTCTATTCT
										7A-1821750-T2-5_704	GAAAGACTTCTTTAAG
										7A-1821750-T2-5_768	TTAAGCAGTATGTTATT
										7A-1821750-T2-5_960	TATATAAATCTATGAAA
										7A-1821750-T2-5_1024	AATCCAAGTGAGAAGA
										7A-1821750-T2-5_1472	ACGTATCAAGCACctct
										7B-1921060-T2-5_64	AGATACGCCTGACACC
										7B-1921060-T2-5_128	CTTTCCATTTTCAGTTT
										7B-1921060-T2-5_192	agagggagagaaagtgagT
										7B-1921060-T2-5_256	GACATGACCACGATGA
										7B-1921060-T2-5_640	AGGGAGTAAGATGGA
										7B-1921060-T2-5_704	ACAAGTGAGGTGCACA
										7B-1921060-T2-5_768	TCTTCTAATTATGCTCG
										7B-1921060-T2-5_832	ACTGCATATGAAACAC
										7B-1921060-T2-5_1024	GAGGGATAAGCAATGG
										7B-1921060-T2-5_1088	TAGGTGAAGGGAGTAT
										7B-1921060-T2-5_1152	GTTGTTATGTGACAATC
										7B-1921060-T2-5_1216	AGTTTAGCACATACAA
										7B-1921060-T2-5_1280	GACAAGCTGTAAAGTT
										7B-1921060-T2-5_1344	ACATTAAAAAGCGCAC
										7B-1921060-T2-5_1408	AGTTCTTGGCGTTCCA
										7B-1921060-T2-5_1472	CACATAGATCATTTTTT
										7D-2042690-T2-5_0	TGCCGAGGGACAAACA
										7D-2042690-T2-5_64	TTTATAGGTGAAGGAA
										7D-2042690-T2-5_128	ATTAGTTGTTATGTGAC
										7D-2042690-T2-5_192	CAGCAAGTTTAGCCAC
										7D-2042690-T2-5_256	GTGACAAGCTATAAAG
										7D-2042690-T2-5_320	CCACATAAAAAAGCAC
										7D-2042690-T2-5_384	AAAGTTCTTGGCGTTC
										7D-2042690-T2-5_448	CACACATAGATCATTTT
										7D-2042690-T2-5_512	ACAAAGATGCCCACCC
										7A-1751470-T2-6_64	aagccaaaaccaaaaccaa
										7A-1751470-T2-6_256	TCGCAGTTTGCTACTA
										7A-1751470-T2-6_448	TGCAGGATGCTACAGG
										7A-1751470-T2-6_512	GCGCAAGAAAAGAAT
										7A-1751470-T2-6_576	TTATTATTAATTAAGAA
										7A-1751470-T2-6_640	ATCATCGCCACTCATCG
										7A-1751470-T2-6_1024	GCCCTCAATCGGTGGA
										7A-1751470-T2-6_1088	TTAATTAATTAACAATG
										7A-1751470-T2-6_1344	CACACCGCTGAAACAC
										7A-1751470-T2-6_1536	TCATTCATTGCTTCCTT
										7A-1751470-T2-6_1580	TGCCCATTGCCTGAGC
										7B-1876170-T2-6_0	GGCCGTGGTCCTCAAT
										7B-1876170-T2-6_64	GAGGCAGGCAAGGTA
										7B-1876170-T2-6_256	TTCAAGCTTTGCTCTG
										7B-1876170-T2-6_512	CTGCTGTGCCATGTGAT
										7B-1876170-T2-6_576	TAGCATTTCCGATTTTT
										7B-1876170-T2-6_640	TTGATTGGTGCCGCAA
										7B-1876170-T2-6_704	ATGTAATACTAATACTA
										7B-1876170-T2-6_768	AAGGCACCGGGGCGA
										7B-1876170-T2-6_832	TCTTCTCTTTCTTAACTA
										7B-1876170-T2-6_1024	CCCAACGGCAAATGCG
										7B-1876170-T2-6_1216	CAGTGGTCGGAGTAAT
										7D-2011180-T2-6_704	GCGACCACGCCGGCAT
										7D-2011180-T2-6_768	TACTGTTTCTTTCCTAC
										7D-2011180-T2-6_960	CAAGCACACTTGTGGA
										7D-2011180-T2-6_1152	CTTAACTGCCTCAATCA
										7D-2011180-T2-6_1216	ATTAATTACTCCAACCA
										7D-2011180-T2-6_1280	CTTTTACTATGGAGCAG
										4A-0943460-T2-7_0	TCGGTTGCTCGCCTTC
										4A-0943460-T2-7_192	GGTTGCGCGAGGTGA
										4B-1095320-T2-7_64	GTGTCATCTAGTGAAG
										4B-1095320-T2-7_128	AAATAATCAATTCTTCA
										4B-1095320-T2-7_192	ATATACAATCACTTTTT
										4B-1095320-T2-7_256	caacaacaaaaacaaaac
										4B-1095320-T2-7_320	CACATCTTTAGGCTCCT
										4B-1095320-T2-7_384	ATTTTTTTCCTAGGGTG
										4B-1095320-T2-7_448	TACTTCACTTTAAATGG
										4B-1095320-T2-7_576	ACACGACACTCCATCCT
										4B-1095320-T2-7_640	ATGCCCATAATGGGAA
										4B-1095320-T2-7_704	TAATTTGTGAAGCCCA
										4B-1095320-T2-7_768	ATATTAAAGAGCCACT
										4B-1095320-T2-7_1344	AGATATAATTTTTATTAT
										4B-1095320-T2-7_1408	TAAAAAAAAGCCATTC
										4B-1095320-T2-7_1580	AGCAGCACACCGACTA
										4D-1162870-T2-7_704	cgggagggacCTCTTGAT
										4D-1162870-T2-7_768	ATTCTAGTAGTTTTGCA
										4D-1162870-T2-7_832	TGTTAACAAAAAAACCA
										4D-1162870-T2-7_896	TGAAATGCTCATTAATG
										4D-1162870-T2-7_1280	ATAAATGAAGATCTATA
										4D-1162870-T2-7_1344	TAGTAACATAGCATATA
										4D-1162870-T2-7_1408	CATTTGAATTATATGATT
										4D-1162870-T2-7_1472	TGCAAAACTACTACTG
										6A-1513690-T2-8_1216	cctcctcctccGCTGCAGA
										6A-1513690-T2-8_1280	CTCCATTGGATACCCAT
										6A-1513690-T2-8_1344	ATGCAACTCCGAATGG

6A-1513690-T2-8_1536	CGTCTTCTCTGCAGCA
6B-1621980-T2-8_1216	GGCGGAGTTtcttcctcct
6B-1621980-T2-8_1280	GATCTGGTCTGATCCC
6B-1621980-T2-8_1344	ACATCAAATTTCCTGCC
6B-1621980-T2-8_1408	GAACAAATTCATCACA
6D-1690440-T2-8_1216	GAGCTtcttcctcctcctcct
6D-1690440-T2-8_1280	TCTGATCCCCCACAAA
6D-1690440-T2-8_1344	AATTTCCTGCCGTACCA
6D-1690440-T2-8_1408	CATCACAGGAAGGAA
3A-0684980-T2-9_448	TAGACTTCCTTGAGAA
3A-0684980-T2-9_512	CCACTCTCCACTCGGC
3A-0684980-T2-9_576	CTAAAGCTTGAGGACT
3A-0684980-T2-9_640	AAGCCCACAATCTCGC
3A-0684980-T2-9_832	CGCAGGTGGCAGGTG
3A-0684980-T2-9_960	TGCAGCCTCCATTGGG
3A-0684980-T2-9_1024	GATGCCTCCATTGTACA
3A-0684980-T2-9_1088	AAAAAAATCGTATGTT
3A-0684980-T2-9_1216	ATCGTTGCCAGCACGG
3A-0684980-T2-9_1344	CTCCCTCGGAACTATCG
3A-0684980-T2-9_1408	CCCCCTTCTTCCCCATC
3A-0684980-T2-9_1472	CTCCAGAGGGCCGGC
3B-0801600-T2-9_64	TTTGTACGCAACCGAT
3B-0801600-T2-9_128	ACAAATCTCGATGGAA
3B-0801600-T2-9_576	TGCAGATTAGACGCAC
3B-0801600-T2-9_640	TGAGATCGGCCGTGTA
3B-0801600-T2-9_704	TCTGCATTCGAGTTTTT
3B-0801600-T2-9_768	CTTTAGTCTGCCGGAC
3B-0801600-T2-9_832	GAGTGGTTGTTGGCG
3B-0801600-T2-9_1024	TTTAAAACAAGTAATT
3B-0801600-T2-9_1152	GTCTTGTCGACTTCAAT
3B-0801600-T2-9_1216	GGTGGCTGTATCGAAA
3B-0801600-T2-9_1280	GAAATGATGGTTGAAC
3B-0801600-T2-9_1344	AAAAACAACAAATGCT
3B-0801600-T2-9_1408	CATTTTGCAGAGTTTA
3B-0801600-T2-9_1472	CACTATTTTATTTTATTT
3B-0801600-T2-9_1536	ACCGAAACGGTAAATT
3B-0801600-T2-9_1580	AGTGGGGGACTGAAA
3D-0926560-T2-9-1_0	CTTAACGGGGGGCAG
3D-0926560-T2-9-2_0	TCGATAAGGACTTTTG
3D-0926560-T2-9-1_64	GCGGGCCTTTTGTCAC
3D-0926560-T2-9-2_64	TACGTCGACTCAAAAA
3D-0926560-T2-9-2_1216	TTTTGTCAGCAACCCA
3D-0926560-T2-9-1_192	CCGCCCAAGAAGTTG
3D-0926560-T2-9-1_320	TAAAATTTAAAAACAA
3D-0926560-T2-9-1_512	CATTATTCATTTTTCAA
3D-0926560-T2-9-1_576	TTCTTTCTCATGATTCT
3D-0926560-T2-9-1_640	ATAACAAAACCGAAAC
3D-0926560-T2-9-1_704	TTCTTCTCGAGATCCGA
3D-0926560-T2-9-1_768	ATTCTTCCTCCTCCTCTT
3D-0926560-T2-9-1_832	CTGGCGCTCCTGGAGC
3D-0926560-T2-9-2_832	GTTTTCTCTTTTCTTTTT
3D-0926560-T2-9-1_896	TTCCCGAGATCCGATA
3D-0926560-T2-9-2_960	CGGGTGGATTGCGGCT
3D-0926560-T2-9-2_1024	GCGATGGATATATCTTC
3D-0926560-T2-9-1_1088	CACGAGGCTGCCCCGT
3D-0926560-T2-9-1_1152	AAAGAAAAAGAATGA
3D-0926560-T2-9-1_1280	CGTTACCAAAAAAAAT
3D-0926560-T2-9-1_1344	AAGGTCTTATATTACTT
3D-0926560-T2-9-1_1408	CCAACATCTAAAGATG
3D-0926560-T2-9-1_1472	CTTCATGAAGCGACCT
3D-0926560-T2-9-1_1536	GATCCAACCTAAGCTT
3D-0926560-T2-9-2_1536	ACCCGTGGGTAGAAA
3D-0926560-T2-9-1_1580	CCTTGTCAATAAGCTTA
3D-0926560-T2-9-2_1580	CCTTTTCTCTGCTTTAT
1A-0015790-T2-10_192	AGCGCGCTCTTCTCCT
1A-0015790-T2-10_256	TTCCAAGGTAAAATAG
1A-0015790-T2-10_512	AATGAGGAGGGGGCA
1A-0015790-T2-10_768	TTCGCCCGCTCCACCG
1A-0015790-T2-10_1024	GCTGCTCGCGCTCGTG
1A-0015790-T2-10_1216	CTCTCCTCTCCAAAAAT
1A-0015790-T2-10_1280	CCCCTGAACAACGGAA
1A-0015790-T2-10_1344	GCCTTGTTCCCTTCAGT
1A-0015790-T2-10_1536	GGTGCAGCGAAAACCT
1A-0015790-T2-10_1580	GGGCTGGACCGCAGT
1B-0119840-T2-10_128	TCGCCGTTCGTCGGAG
1B-0119840-T2-10_320	GACCGGTGTGGACGC
1B-0119840-T2-10_384	TTCGTGGTAAGGAACA
1B-0119840-T2-10_448	GAGTGGATGAACAGC
1B-0119840-T2-10_512	ATCGATCGATCAATCAA
1B-0119840-T2-10_576	ACGGTCTTATCACGAA
1B-0119840-T2-10_640	AACGGGGAAAAGTTT
1B-0119840-T2-10_704	GGCGCGGCAAAGTGC
1B-0119840-T2-10_768	CACCGCAGTCCAGCTA
1B-0119840-T2-10_832	CGGCGTGATCGGCGG
1B-0119840-T2-10_1024	CGGCACGCGGCCAGA
1B-0119840-T2-10_1088	GGAGTGACAGGGAGG
1B-0119840-T2-10_1152	GTCTGGTGGCCGCAAC
1B-0119840-T2-10_1344	GCGCTCCTTCCCCTCGT
1B-0119840-T2-10_1580	GCGCCCGCCTTCCTCTT
1D-0233520-T2-10_0	TCCTCTGCTCAGTTATT
1D-0233520-T2-10_122	ACAGGATGAACCGAAA
1D-0233520-T2-10_183	CTGGCTGGCCACTGCC
1D-0233520-T2-10_244	CAAGCCACCGACGCG
1D-0233520-T2-10_427	GAACAACAAATTCGTG
1D-0233520-T2-10_488	GAACAGCAGCAGAAG
1D-0233520-T2-10_549	TGATGATCAATCAATGG
1D-0233520-T2-10_610	CAGTTATACCACCGAC
1D-0233520-T2-10_793	GGCTCCACCGCAGTCC
1D-0233520-T2-10_854	CCAGAACAGAAGCGC
1D-0233520-T2-10_915	GCGAGCCGACAACCG
1D-0233520-T2-10_1098	TCGGCAGATGCTAACA
1D-0233520-T2-10_1159	GCGCCGGTGTGCTGGA
1D-0233520-T2-10_1220	AGCATAATAATCAATCA
1D-0233520-T2-10_1342	AACCAAAAGCGGCGG
1D-0233520-T2-10_1586	AGGAGGGGCGCCCGC
1D-0233520-T2-10_1830	CCCTGCTCTTCAACCGT
1D-0233520-T2-10_1891	tctttctttcctccttccattctt
1D-0233520-T2-10_2196	AAATCCTGGGGCCTAG
1D-0233520-T2-10_2440	CCATCCACGGGCTCCA
1D-0233520-T2-10_2623	CGACAATGCCAAGCCG
1D-0233520-T2-10_2684	GCCGTTGGTAATTAGG
7A-1843160-T2-11_0	ACCACCCCCCCTCTCCT
7A-1843160-T2-11_192	TTTGACCTTGCGTTTTA
7A-1843160-T2-11_448	TGGTGGGCCCTGCTGC
7A-1843160-T2-11_832	CCATTCATGACCGCCCT
7A-1843160-T2-11_896	AGTAGTATAGCAATGT
7A-1843160-T2-11_1280	TTTTGTCAGTACGGAT
7A-1843160-T2-11_1408	GGATCATGCTTGGATG
7A-1843160-T2-11_1472	TGGAGTAAATATTTTCA
7A-1791360-T2-12_0	cgccgccgcggccggatctg
7A-1791360-T2-12_320	AGGGGAAGCCGGGCG
7A-1791360-T2-12_384	TCTCTGTCTGCTTTGTG
7A-1791360-T2-12_448	TTTTGGCACCATGTTTT
7A-1791360-T2-12_512	TCTCCCGTACATTTTAA
7A-1791360-T2-12_576	TTTTTTTTGTTTCTACA
7A-1791360-T2-12_704	CACGACTCGTTTGCTG
7A-1791360-T2-12_768	GCTAGAGGCGAGCCTT
7A-1791360-T2-12_1024	ACCCCTGGCCTTAGCA
7A-1791360-T2-12_1472	TTCTTGACATTGGCCTC
7A-1791360-T2-12_1536	TTAGCTGCTCTCTCAGT
7A-1791360-T2-12_1580	CGACATGGAGGGCCAT
7D-2009540-T2-12_320	AGAGGAAgccgggcgga
7D-2009540-T2-12_384	AGCGTGTTCGGCGGG
7D-2009540-T2-12_448	TTCTCTCTCTTTCTTGT
7D-2009540-T2-12_512	CGCATGGGACATGTGG
7D-2009540-T2-12_704	CTTGCGAGCAGAAAG
7D-2009540-T2-12_832	TTGGTCGAAGTTCCCC
7D-2009540-T2-12_896	AGATCGACATGGAGCA
7D-2009540-T2-12_960	GCAGGACAATCATTCTT
7D-2009540-T2-12_1024	AGGCAGAGGAGAGAA
7D-2009540-T2-12_1088	CTTATCCTATGGTTCAA
7D-2009540-T2-12_1152	AAAACATGAAGCAGCA
7D-2009540-T2-12_1216	CATAAGCGAAGAAACA
7D-2009540-T2-12_1280	ACACCAAATTAATACCA
7D-2009540-T2-12_1344	TGGCAGACGACACACA
7D-2009540-T2-12_1408	ACAAGCATTCAAGCTG
7D-2009540-T2-12_1472	CACCATACCATATTCCG
7D-2009540-T2-12_1536	GAGCAATTGCTTCTGG
7D-2009540-T2-12_1580	GGTACAATATAGCAATA
2A-0338910-T2-13_0	TCAGTCTAGTGGATAC
2A-0338910-T2-13_64	GGGAACCACGCCTAGT
2A-0338910-T2-13_256	GAAAACCGTGGAATCG

2A-0338910-T2-13_384	TCCTGAAAGCTGAAAG
2A-0338910-T2-13_448	TCACGTTGCTACGAAA
2A-0338910-T2-13_512	GTTGCAGTGTTCGCAC
2A-0338910-T2-13_576	CAAAAAATGTAGTTGC
2A-0338910-T2-13_640	TATCTATTTATTCCGAG
2A-0338910-T2-13_704	CTGAAATGAACAAAAA
2A-0338910-T2-13_768	GGCGAAAAAATGTGC
2A-0338910-T2-13_832	AAAACAAGAAAAATCT
2A-0338910-T2-13_896	AAGTGTTGTACTATTCT
2A-0338910-T2-13_960	TTCCCATTTAACTGTCT
2A-0338910-T2-13_1024	CAATTCCAAGCGAGTA
2A-0338910-T2-13_1088	CAAAAATTTGACACAC
2A-0338910-T2-13_1152	AATTCAATTTGCAAGA
2A-0338910-T2-13_1216	CTATTTTTTTACAAAAA
2A-0338910-T2-13_1280	TTATATAACCACAACTT
2A-0338910-T2-13_1344	CTTTATTGATAAAAGA
2A-0338910-T2-13_1408	AGAATATGCACAAGAT
2A-0338910-T2-13_1472	TCTTTGCATGAACCAA
2B-0461810-T2-13_64	GGCTGCATGCATGGAT
2B-0461810-T2-13_128	GGTGCAGGTGCCGCA
2B-0461810-T2-13_192	AAATCCACGGTAGTAC
2B-0461810-T2-13_256	CTGGTATGCTGAAAGT
2B-0461810-T2-13_320	TCGAATAGAAACAAGT
2B-0461810-T2-13_384	AATACTGGCATGAGTG
2B-0461810-T2-13_448	ACACGCGAAGCAGGA
2B-0461810-T2-13_512	ACAAGACGGGACTAAT
2B-0461810-T2-13_576	CTCCTAAGTTGCGAAT
2B-0461810-T2-13_640	CCGTCTGTCCGCGAGA
2B-0461810-T2-13_704	ACCAACTACACTAGGA
2B-0461810-T2-13_768	TTCGATTTTTAAAGCTT
2B-0461810-T2-13_832	TTAAAGCTCCAACAGG
2B-0461810-T2-13_896	TTATTTTCTATTGTTTGT
2B-0461810-T2-13_960	AATTATTAGATATAAAA
2B-0461810-T2-13_1024	ATTTTTTTATAAATAAT
2B-0461810-T2-13_1088	ACAAAACCACAAATTT
2B-0461810-T2-13_1152	CTGCGTAGATCACGAG
2B-0461810-T2-13_1216	TATAAGCTCATAGGTTT
2B-0461810-T2-13_1280	CTTTGCTCACAGTGTG
2B-0461810-T2-13_1344	TGTCTATAATTTCATATT
2B-0461810-T2-13_1408	AATAAGAATATTCACA
2B-0461810-T2-13_1472	TAAACATTTTTTGCAT
2D-0601440-T2-13_0	GAAATGAACAAAGAC
2D-0601440-T2-13_64	CGAAAAAAATGTGCAT
2D-0601440-T2-13_128	AAACAAGAAAAATCTT
2D-0601440-T2-13_192	AGAGTTGTACTATTCTA
2D-0601440-T2-13_256	TTCCATTTAACCGTCAA
2D-0601440-T2-13_320	ATACTAACATTTCCAAG
2D-0601440-T2-13_384	TTTAATTCTAAAATTTG
2D-0601440-T2-13_448	ATAATGAAATTCAATTT
2D-0601440-T2-13_512	GGGGGGGGGGCAA
2D-0601440-T2-13_576	CTTGCGTATGAATATCT
2D-0601440-T2-13_640	GGTCACCTGAGAGGAT
2D-0601440-T2-13_704	TAAGAAGGAGGGACT
2D-0601440-T2-13_768	ACTGTTGGTTCACTGC
2D-0601440-T2-13_832	CTTAGTATATTTTTTATG
2D-0601440-T2-13_896	TGCTGGCTATTATTTCC
2D-0601440-T2-13_960	AAATATAATCATCTCTC
2D-0601440-T2-13_1024	TAAATTATACTGATGCA
2D-0601440-T2-13_1088	CTTCTTTGAGGCGGCG
2D-0601440-T2-13_1152	GACTTTTCATCTGCTAC
2D-0601440-T2-13_1216	GCCCAGAGTTGATAAC
2D-0601440-T2-13_1280	CAAATTTCTTCAAATGA
2D-0601440-T2-13_1344	GCCTTTGGGCCTTCTT
2D-0601440-T2-13_1408	AGATATTCTAGTAGTAC
2D-0601440-T2-13_1472	TTTTTGCATCGAAAAA
3A-0705780-T2-14_0	GGCCGCTCCCgctcggct
3A-0705780-T2-14_192	GGACACTTGGACTATT
3A-0705780-T2-14_256	GGGGTGTTTGACGTAC
3A-0705780-T2-14_320	TCTATAAGATCACTTTT
3A-0705780-T2-14_384	GGAATAGCATTTTGTTT
3A-0705780-T2-14_448	CTTAAATGGCGCGCCG
3A-0705780-T2-14_512	ATATGCTTATTACGATT
3A-0705780-T2-14_576	TTGCAGGTAGTTCGAT
3A-0705780-T2-14_640	CGGCAACGATATATTC
3A-0705780-T2-14_704	CTGTCCCTAATTAGTGT
3A-0705780-T2-14_768	ATGTTGTGTTGATCTAA
3A-0705780-T2-14_896	TCCCTGATCTCCTCGCC
3A-0705780-T2-14_960	AAAGTGACGATCCCCT
3A-0705780-T2-14_1152	CTGGGAGCTCGTCGTA
3A-0705780-T2-14_1216	GCTAAGCAAGAGAAAT
3B-0810200-T2-14_0	ATAAGGTACACTCTAA
3B-0810200-T2-14_64	AAAGTACACCAGAGA
3B-0810200-T2-14_128	CAGAGCGCTACCTGTC
3B-0810200-T2-14_192	GGTGACCCAACAAGG
3B-0810200-T2-14_704	ACAAGAGACGACTACT
3B-0810200-T2-14_768	TCCTTGAGCCGAGATA
3B-0810200-T2-14_832	CACCCTTAAAGCAACT
3B-0810200-T2-14_896	CCAAAGACAACTATCG
3B-0810200-T2-14_960	ACTCCTTGTACCTTCTT
3B-0810200-T2-14_1024	CTTAGTGTCGACAATC
3B-0810200-T2-14_1088	CTACGACGAGCCCACA
3B-0810200-T2-14_1152	AACGGCGAGTCAAGT
3B-0810200-T2-14_1216	CGCATGTCGGTGGACG
3B-0810200-T2-14_1280	GATTCAGGGGAGGAC
3B-0810200-T2-14_1344	AACTAGATTAGGAGAT
3B-0810200-T2-14_1580	CATCGAGCCACTGCCG
3D-0924320-T2-14_0	GGCCGCTCCCGCTCTC
2A-0288920-T2-15_0	GGCGGGCGCACCGGA
2A-0288920-T2-15_192	GGACCCGGGACCCCG
2A-0288920-T2-15_256	TGGGTCTTCACTGCCC
2A-0288920-T2-15_320	TCTTCGCTGCTCTCCAT
2A-0288920-T2-15_384	TTCACTGCTCTCGTAG
2A-0288920-T2-15_448	CAGTTTAGACAAGTTT
2A-0288920-T2-15_1216	ttcgtcatttgtgcatgcatgg
2B-0440210-T2-15_768	GCTGCTTCAGAAATCG
2B-0440210-T2-15_832	TTTTCGGTGATCAAAC
2B-0440210-T2-15_896	TCCAAAGCACTTCTAA
2B-0440210-T2-15_960	CGTGTATATTGATGGCC
2B-0440210-T2-15_1024	ATAGCCGTAGTTACAAT
2B-0440210-T2-15_1088	GATCCCCTTGGCGTAA
2B-0440210-T2-15_1152	ACTCGTGCTCGAGGTT
2B-0440210-T2-15_1216	TGTTTCATCAGAGCCC
2B-0440210-T2-15_1280	TAGAAGATCTTCAAGA
2B-0440210-T2-15_1344	GTTTCTCaaaaaagaaaa
2B-0440210-T2-15_1536	cggcgcgcgtcccccgcggc
2B-0440210-T2-15_1580	CCCGTCCTCACGTTCCC
3A-0642540-T2-16_448	TGGTTAACTCTTTTTTT
3A-0642540-T2-16_512	TGTACAAAAGTCCGAA
3B-0752390-T2-16_128	CACGACCTCACTTCTG
3B-0752390-T2-16_192	TGCTTTGGCTTCTTTTT
3B-0752390-T2-16_256	CTTTAGCAAGGTAATC
3B-0752390-T2-16_320	TTTCTTAACGCTGCGG
3B-0752390-T2-16_512	TCCCCTTTATTCATTTT
3B-0752390-T2-16_576	TACAACAAATCATCCA
3B-0752390-T2-16_640	CACATTGCCGCCCAAC
3D-0855390-T2-16_1472	AAAAAAATCTGATGGT
3B-0711520-T2-17_128	GCTCCGGGCTGCGGC
3B-0711520-T2-17_192	AGTTTTTCTTTCTACAA
3B-0711520-T2-17_256	GTGAAACGTGCAAGT
3B-0711520-T2-17_320	GGATTTTAGAAGAGCA
3B-0711520-T2-17_384	TTTTGACTCTATCACCA
3B-0711520-T2-17_448	TTGTCCTTCGCTGGAG
3B-0711520-T2-17_512	TGGCGATGGTGAGCTC
3B-0711520-T2-17_576	TGAAGAAATTGAATGC
3B-0711520-T2-17_640	ATGAGCTTCAACTGTC
3B-0711520-T2-17_704	ATCATGTTGTGTGTTCT
3B-0711520-T2-17_768	AGGTTTTCATAAAGGA
3B-0711520-T2-17_832	CCACATTGATGCTATTG
3B-0711520-T2-17_896	AGAGGTGAGGACATC
3B-0711520-T2-17_1024	CGTGTAGGAGTTACCC
3B-0711520-T2-17_1152	AGATTAAGATTAGAAT
3D-0837950-T2-17_1344	CTCCGTCGGTGGAGTC
3D-0837950-T2-17_1472	GCCCTTGAAGTTGAAT
7A-1836660-T2-18_128	CGCCGGGCAGAGTGG
7A-1836660-T2-18_1024	TTGTTCTTTATGATATG
7A-1836660-T2-18_1152	GGGGGTATCAAACCGT
7A-1836660-T2-18_1216	AGGATGAGGACATGTA
7A-1836660-T2-18_1280	GAAGGTTCATGTTGTC
7A-1836660-T2-18_1344	AGTTCTCTAAAAGGTT
7A-1836660-T2-18_1408	atatTCATAAACTTTTAA
7A-1836660-T2-18_1472	AGTAATATCAAAAAGG
7A-1836660-T2-18_1536	ATGGAAGAGAAAATG
7B-1932650-T2-18_128	AGTGGCGAGTGTTCCA
7B-1932650-T2-18_256	GAGGTAGCTCTCCCGG
7B-1932650-T2-18_384	CGGCGTAATAACAGTG
7B-1932650-T2-18_512	ATGCCCTTTTTTGTACG

7B-1932650-T2-18_576	GGCTCATGGTGAAAAT
7B-1932650-T2-18_640	TCTAACGTCCAGGATT
7B-1932650-T2-18_832	AACAAAGATTGTGCTA
7B-1932650-T2-18_896	TTGTGTTACGGATTTT
7B-1932650-T2-18_960	TCCCGCCACATCGGTT
7B-1932650-T2-18_1024	TTGAAACATCTATGTTT
7B-1932650-T2-18_1216	ACGCCAATCTAGGTAG
7B-1932650-T2-18_1280	AAGGAGAAAGGTAGC
7D-2049830-T2-18_128	CAGAGTGGTAGCGAG
7D-2049830-T2-18_256	CCGTTGGCTAGCGAG
7D-2049830-T2-18_960	TCTCGGACCATTGTCC
7D-2049830-T2-18_1024	GTTCGAAGCATTCTGG
7D-2049830-T2-18_1216	AATCAAGCAACTAGAC
7D-2049830-T2-18_1408	CAGGCAGAGGAAGAA
7D-2049830-T2-18_1472	GAGGTTGCCTCGGCG
6A-1538560-T2-19_0	TCATAGTAAAATAACA
6A-1538560-T2-19_64	CATTTTGGCGGGTGG
6A-1538560-T2-19_128	AAAAGAACTCTCCAGT
6A-1538560-T2-19_192	ATTTTGTAGTGGTTTTT
6A-1538560-T2-19_384	GATGGTTGCTTTATATT
6A-1538560-T2-19_448	TTGTGAGCAGCCTAAG
6A-1538560-T2-19_512	GGTCTTGCCGTGAAGG
6A-1538560-T2-19_1280	GCGGCATTCCGGCGG
6A-1538560-T2-19_1344	GGCAAAAAGAACTCTT
6A-1538560-T2-19_1408	TGTAATTCTGTAGTCGG
6B-1667080-T2-19_128	CTGCCACCGCTGGCGA
6B-1667080-T2-19_192	ACCTACCTTAATTCTGG
6B-1667080-T2-19_256	AAATTACCGCAAAAAA
6B-1667080-T2-19_768	CGCCGGAATGCCGCAG
6B-1667080-T2-19_832	GTCACTGACTGGCGGG
6B-1667080-T2-19_896	TAAATTTAGTGAAAGA
6B-1667080-T2-19_1024	GTGCGTCGAGCGCTTT
6D-1731330-T2-19_320	TGTAGCCAACTGAGTT
6D-1731330-T2-19_384	ACTAACAAAAAACAAG
6D-1731330-T2-19_448	TTTAATACGTGTACTCA
6D-1731330-T2-19_512	GTTGGGCTGTACTAAT
6D-1731330-T2-19_576	CCACGTAACAGACGCG
6D-1731330-T2-19_640	TTAATCTGCCGCATTGA
6D-1731330-T2-19_1024	TATTGCCCGACCTACCT
6D-1731330-T2-19_1088	GAATTATCGATGTTTAC
6D-1731330-T2-19_1152	AGGTTTACGGGAAGAT
6D-1731330-T2-19_1280	CAGCGGCCACCGCACC
6D-1731330-T2-19_1344	TTTTTATGTAATTAAAA
3A-0655890-T2-20_0	TATTTGTTATTCATGAAT
3A-0655890-T2-20_64	AAACTAACATACATCAA
3A-0655890-T2-20_128	CAATTTTAAGAATTAGA
3A-0655890-T2-20_192	AGCAAATCATTGTTTGT
3A-0655890-T2-20_256	ACTTCCGCACCGCGTT
3A-0655890-T2-20_384	GAGGGGGAGGGGTCT
3A-0655890-T2-20_448	TTTCACGATTCGTATTG
3A-0655890-T2-20_512	TAGAGGTTTTATGGGG
3A-0655890-T2-20_576	ACCGCAACCCCCAATC
3A-0655890-T2-20_640	TATAGGGAAAACTAAG
3A-0655890-T2-20_704	GATTCTAGGAATAAGC
3A-0655890-T2-20_768	CACTAGTAACCAAAAA
3A-0655890-T2-20_832	CAAATTAATGTTGCCCG
3A-0655890-T2-20_896	GAAGAAGGCGCAAAA
3A-0655890-T2-20_960	AACTCCCCAATTGGAC
3A-0655890-T2-20_1088	CGTATGCATACGTTCAC
3A-0655890-T2-20_1152	AAGCTTCCAATCTTGCT
3A-0655890-T2-20_1216	GCCTTTTATAAAAAGA
3A-0655890-T2-20_1280	TAGAATTGTTTATCTGA
3A-0655890-T2-20_1408	CTGGTCTCTGAAGACG
3A-0655890-T2-20_1580	TGCGACCCGACTCTCG
3B-0716390-T2-20_0	AAGCGTTTAAAATCAA
3B-0716390-T2-20_64	AGAATTTTGGTAACTG
3B-0716390-T2-20_128	CCCAACTCGTTCTATAC
3B-0716390-T2-20_192	AATTCCCTGCTGCACCT
3B-0716390-T2-20_256	TTCGGCAATAATTTTTT
3B-0716390-T2-20_448	ccttttctctttctttctttctttT
3B-0716390-T2-20_512	CTACTAAGGCCCTATAT
3B-0716390-T2-20_640	ATTGGGAAAAATGCAC
3B-0716390-T2-20_704	CACTAGAAGCAAACAT
3B-0716390-T2-20_768	TAAATCCCTGGAAACA
3B-0716390-T2-20_832	TCCAACTCCGAGCACC
3B-0716390-T2-20_1024	AAATCAATACGAATGC
3B-0716390-T2-20_1088	GATTTGACCACCGTAT
3B-0716390-T2-20_1152	AGTTAGCAACCTTCCA
3B-0716390-T2-20_1216	ATTAATTGCCGTATATA
3B-0716390-T2-20_1280	GTAGAATTTGTTTCTCT
3B-0716390-T2-20_1344	AAGATATATACCACgag
3B-0716390-T2-20_1408	AGGAAACTGGTCTCTG
3B-0716390-T2-20_1580	CGCGCAGGCGTGTGC
3D-0875400-T2-20_0	GCCGACGTTCGGTTGG
3D-0875400-T2-20_192	GCACGCTCGCGTCAGT
3D-0875400-T2-20_256	CCTGGATTCGGCACCT
3D-0875400-T2-20_384	GTGTGtatatatatatata
3D-0875400-T2-20_448	ATCTTTTAGATATTCAG
3D-0875400-T2-20_512	CTCCCAAAAGATATTCT
3D-0875400-T2-20_576	GTAGTATAGAGGAAGA
3D-0875400-T2-20_704	TGGGGTGGTAAGTGA
3D-0875400-T2-20_768	GGTTTTACGCGTTCTT
3D-0875400-T2-20_832	CGGACAACATTAATTT
3D-0875400-T2-20_896	TGTTACAGGTGTATTTC
3D-0875400-T2-20_960	TTCCTAGAATCATGTTT
3D-0875400-T2-20_1024	TCCTTATATCCTAGTGC
3D-0875400-T2-20_1088	TTGCGGTTTCACGGGG
3D-0875400-T2-20_1152	TATCCCCACCAATCCCC
3D-0875400-T2-20_1344	CAACTAGGGTGAGCCG
3D-0875400-T2-20_1408	AAGTAAAAAATCATTG
3D-0875400-T2-20_1472	GCTAGGATGCAGCTGA
3D-0875400-T2-20_1536	TATATATAGAACGAGTT
3D-0875400-T2-20_1580	ATGTCGGGATTTCCGG
1A-0063880-T2-21_128	ACTTGGAGAACATTGG
1A-0063880-T2-21_192	AAAATAGCCTGTGGGC
1A-0063880-T2-21_512	TTCGTTATTTATGATGC
1A-0063880-T2-21_576	TAAGCATGAAATATGA
1A-0063880-T2-21_640	CAACTGTAAACGCTCTT
1A-0063880-T2-21_704	TACTACAATCAAAGTCA
1A-0063880-T2-21_832	AATATAGATTTATCTATT
1A-0063880-T2-21_896	AATATTTAGACGGATG
1A-0063880-T2-21_1024	ACGTGTTATAGGGGCG
1B-0178860-T2-21_1024	ggcgtcaaaataggaccgaa
1D-0263790-T2-21_128	TGATGGACATGCATGG
1D-0263790-T2-21_192	ATGCGTCTGCGGACGT
1D-0263790-T2-21_256	CTCTTAGCACACCTTTT
1D-0263790-T2-21_448	ATCCATTTGTTTCGTAG
1D-0263790-T2-21_512	AGTGGATGGAGAGCA
1D-0263790-T2-21_640	AGGGGGGGGGATGTT
1D-0263790-T2-21_1024	CTGGAGATGCTCCAAG
2A-0343600-T2-22_183	CAACCGAGGGAATAGT
2A-0343600-T2-22_305	CCTCTATCCCGAGCTTC
2A-0343600-T2-22_366	CTTGATGCAAGCCTTC
2A-0343600-T2-22_427	GGTCATCATGCCTTGC
2A-0343600-T2-22_488	CGCTCCGTCGTTCGTG
2A-0343600-T2-22_549	GGCAAGGGGGGGGG
2A-0343600-T2-22_610	TGTGTGATGGACTAGT
2A-0343600-T2-22_671	CAACCCAAATTTATGTC
2A-0343600-T2-22_793	GTTGCCCTCGCTGACA
2A-0343600-T2-22_915	GATAGGGAAGTCAAG
2A-0343600-T2-22_976	GCGATAGAGATAGAGT
2A-0343600-T2-22_1037	CCAGCGATCTTTCTAG
2A-0343600-T2-22_1098	TCGATCTGGTCCATCG
2A-0343600-T2-22_1281	CAGAGCAGCCGTGGA
2A-0343600-T2-22_1342	CCACACGGCTGGGCG
2A-0343600-T2-22_1403	GAAGCAAGCGGACTC
2A-0343600-T2-22_1525	CTTCCCTTTCGCTTCAT
2A-0343600-T2-22_1769	ccgccgccCAACCCAGAT
2A-0343600-T2-22_1830	GTAACAGATTCTGCCT
2A-0343600-T2-22_1952	AAGATTCAGGATGATT
2A-0343600-T2-22_2013	ATCACGCCTCTGCTCTT
2A-0343600-T2-22_2074	ATTCCACATGCTCCTTG
2A-0343600-T2-22_2196	ATCTGACGCATGTGATG
2A-0343600-T2-22_2257	TCCAAAGCAAACAAAG

2A-0343600-T2-22_2318	GTGCAATCTTTTCCAGA
2A-0343600-T2-22_2379	TTCTTTTAATAAGTATT
2A-0343600-T2-22_2440	TTATAGCTCATGACATT
2A-0343600-T2-22_2562	GATTTTTCTGAAGATCT
2A-0343600-T2-22_2623	AAGGGTCTGGTTACCA
2A-0343600-T2-22_2806	AGGCCACGCTAAAACT
2A-0343600-T2-22_2867	CTTCAACTGGAAGGTG
2A-0343600-T2-22_3050	GTGTAGACTTGCTCATA
2A-0343600-T2-22_3111	ATATGTTTTGTTTTGCG
2A-0343600-T2-22_3233	TGGGACCACATTTCTC
2A-0343600-T2-22_3294	ATGAGACCCTACAACT
2A-0343600-T2-22_3355	GCCACCATAGTTTGTA
2A-0343600-T2-22_3416	ATTACCAATCGATTAGA
2A-0343600-T2-22_3477	AGCAGAGAGCAAGAC
2A-0343600-T2-22_3599	GGTCTGGTTTCCTTGA
2A-0343600-T2-22_3782	AAGCAACCCTGAGGTT
2A-0343600-T2-22_3965	TTCTTTTTCTTTGACAA
2A-0343600-T2-22_4087	TGAGTGCTTTTCTATGT
2A-0343600-T2-22_4148	TTGATAACCTGCTACCT
2A-0343600-T2-22_4209	GGCATTGAGGAGTTCT
2A-0343600-T2-22_4256	AAAAGATCGATTTTAC
2B-0470790-T2-22-2_1152	atattttttcccacaggatttt
2B-0470790-T2-22-2_1216	ctttcaaagatgaatcaaaa
2B-0470790-T2-22-2_1408	tgtataaatatgtgattctca
2B-0470790-T2-22-2_1472	AAGGTCCCCGGTCTGT
2B-0470790-T2-22-2_1536	GCGTGCGTAGAGACCT
2B-0470790-T2-22-2_1580	CCTAGCACTAGCCGTAT
2B-0470790-T2-22_256	atgagtttctttgaaaattttct
2B-0470790-T2-22_512	ttagcaacatatatctatacc
2B-0470790-T2-22_576	tcaagagatgaatgatagat
2B-0470790-T2-22_768	acatacgccttcacaatgcgt
2B-0470790-T2-22_832	ttactttttacaTGGATCTC
2B-0470790-T2-22_896	GTGTCCTCGTATTAATG
2B-0470790-T2-22_960	GTCTACTACTCCTAGCA
2B-0470790-T2-22_1088	ACATGACATTTGGTAG
2B-0470790-T2-22_1152	AGCACAGGTAACAACT
2B-0470790-T2-22_1344	GCCAAGATTGCTTATTT
2B-0470790-T2-22_1408	CATCCGATCCCTTCCAC
2B-0470790-T2-22_1472	TCCGCAAGCAAGCTGC
2D-0598390-T2-22_0	TGGCACACATGCCAAC
2D-0598390-T2-22_63	AGCCTCATCTTTTGCCT
2D-0598390-T2-22_441	TCATTTGCTCCTCTTTC
2D-0598390-T2-22_504	TTGTAAAGTTTTTTTTG
2D-0598390-T2-22_567	CTGTGGAGGCTGGAA
2D-0598390-T2-22_630	AGGCTTTTGAGAGAG
2D-0598390-T2-22_693	GCACAAACCGAACCCA
2D-0598390-T2-22_756	CTTGAGTGTGGTGGGT
2D-0598390-T2-22_819	CACCGGCCGTCGACAT
2D-0598390-T2-22_882	ATCGATCTGTACACATG
2D-0598390-T2-22_945	GCGCGAGTCCCCGTGG
2D-0598390-T2-22_1071	CTTTCTAgctccggctccg
2D-0598390-T2-22_1134	AGGTGCTCGATCTGGG
2D-0598390-T2-22_1323	AGAGGAGCCGTAGACT
2D-0598390-T2-22_1386	GTGTCCATTGGTGGCC
2D-0598390-T2-22_1512	CCCTTTCGCTTCCTGCC
2D-0598390-T2-22_1764	TCTTGCCGCCGCCCAA
2D-0598390-T2-22_1890	TTTAGCATTGTTTGACA
2D-0598390-T2-22_1953	AAAGATTCAGGATGCA
2D-0598390-T2-22_2016	AATCACGCCTCTGCTC
2D-0598390-T2-22_2079	TTCCACATGCTCCTTGC
2D-0598390-T2-22_2142	TTTCAAGGAAAATTGT
2D-0598390-T2-22_2205	AATGTGATGAACACTG
7A-1787960-T2-23_128	AACGCAAACTACCTAA
7A-1787960-T2-23_576	GGAGGACTCTGGTCG
7A-1787960-T2-23_640	AACGGCCAGACCGTTA
7A-1787960-T2-23_832	TCGCCGGCCGTGTGCT
7A-1787960-T2-23_960	CAGATCCGCATCCGCC
7A-1787960-T2-23_1024	ATGTTACAGACTTATGT
7A-1787960-T2-23_1088	TGTGTGAGGTGAGATG
7A-1787960-T2-23_1152	TGGCGTGGGAAAAAA
7A-1787960-T2-23_1216	ATTAGTGGGTGTAGAA
7A-1787960-T2-23_1280	CAAGAACATCAATTAA
7A-1787960-T2-23_1344	GTTCAGTGTTTCCATCA
7A-1787960-T2-23_1408	TCGTTGGCCTTTCTTTT
7A-1787960-T2-23_1472	TACATTCAGGATTTGG
7A-1787960-T2-23_1536	TCACCTGTATGTGCTAA
7A-1787960-T2-23_1580	CACCTTGTCTGATCTAC
7B-1901610-T2-23_0	GCTAGCAAATTTATTTG
7B-1901610-T2-23_64	ATTAACTATGTGGCTCA
7B-1901610-T2-23_192	CTAGATGTTAAGGATT
7B-1901610-T2-23_384	TGTGCTTAGACCAACA
7B-1901610-T2-23_448	CAGGCCATAGATTGTC
7B-1901610-T2-23_512	AATACAACAGTTCAGT
7B-1901610-T2-23_576	CAGATAGGACATCTCA
7B-1901610-T2-23_832	GGAGCACACGGCCGG
7B-1901610-T2-23_896	GCTCGTTTTTTCCCGA
7B-1901610-T2-23_960	TGCGCTTCCATGGGGT
7B-1901610-T2-23_1280	AGGCAGGTAGCCGCC
7B-1901610-T2-23_1472	AATTGGTGATCATTCTT
7D-2005130-T2-23_192	CAAGTAATCATCAAGA
7D-2005130-T2-23_640	CACCATGCGCGGCCCA
7D-2005130-T2-23_704	TGCAAAAACCACCCCA
7D-2005130-T2-23_960	CCGCCGACGCCGGAG
7D-2005130-T2-23_1024	GTCTTTTCACTGTGTAT
7D-2005130-T2-23_1088	TGTCCTATCTGATCCGA
7D-2005130-T2-23_1152	ACTGTTGTATTTGGCTT
7D-2005130-T2-23_1344	GCCCTGTCTGCTCTGTT
7D-2005130-T2-23_1536	TTGAAAGCATAGGACA
7D-2005130-T2-23_1580	CATGCCGTATTTTTGCT
7A-1805710-T2-24_64	TTGAGACCGCACGGTC
7A-1805710-T2-24_128	GGTGATCTGCTGGGTA
7A-1805710-T2-24_192	AACTAGCCTTAGAAAA
7A-1805710-T2-24_320	CTCCCGCTGCCCGCTG
7A-1805710-T2-24_384	ACCGACACTCAGATCA
7A-1805710-T2-24_448	GATTACTGGTACAGTG
7A-1805710-T2-24_576	TCGGTAGTACCAGTGG
7A-1805710-T2-24_768	TACCCCCCGCTGATTTA
7A-1805710-T2-24_1216	TGCCACGGCGGAGGC
7A-1805710-T2-24_1280	ACGGCGTGTCTATCGT
7A-1805710-T2-24_1344	GTGGTTGGGTGTCTAG
7A-1805710-T2-24_1408	TGCTGAGATTCAGCCT
7A-1805710-T2-24_1472	ACGGATTTAGCAGCAG
7B-1928540-T2-24_256	AAAGGCAGAGCTGCT
7B-1928540-T2-24_384	GCGCCGTAAAATAGCA
7B-1928540-T2-24_448	ATTGTTTTATGGTGAG
7B-1928540-T2-24_640	CGCTCAAAGGCAGAAT
7B-1928540-T2-24_704	CCCTTCGTATACGTTTT
7B-1928540-T2-24_768	GAGGTAGTAGTACCCA
7B-1928540-T2-24_832	CATATGTTTTGACCGTG
7B-1928540-T2-24_896	TCTCCGCCGAGCGATC
7B-1928540-T2-24_1024	CGCACGCGCTTTCCGT
7B-1928540-T2-24_1408	ACTCGCCACTCCAAGT
7D-1187590-T2-24_704	CATTTGGGTCGCCACG
7D-1187590-T2-24_960	TAAAATATAAAACGTTT
7D-1187590-T2-24_1152	AAATCTCCCAGCATTG
7D-1187590-T2-24_1216	TCAGTCTAGCGTGTCT
7D-1187590-T2-24_1280	AAAAGACAATCTCTAAT
7D-1187590-T2-24_1408	CACCGACCGACCACAG
7D-1187590-T2-24_1580	ATCCCCTGCTGATTTAC
7A-1829510-T2-25_0	TTTGTATGCGAGGAAG
7A-1829510-T2-25_64	CGGCCAACAGATTCTG
7A-1829510-T2-25_128	TTTTCTTAGGTTTCCAA
7A-1829510-T2-25_192	TTCATACTTTTTATACTT
7A-1829510-T2-25_256	GGACGGCCAACAAGT
7A-1829510-T2-25_320	ACATCACATGCTAGCTA
7A-1829510-T2-25_384	GCACATACACATCATTC
7A-1829510-T2-25_512	AAACGTCTCCTCTCACT
7A-1829510-T2-25_576	TGAAAATACCACTGTC
7A-1829510-T2-25_640	GATGCCAACATACCCG
7A-1829510-T2-25_704	TTCCATCTCTCTTTCAC
7A-1829510-T2-25_768	TATTGTTGCTTTCAACC
7A-1829510-T2-25_832	GGATATCAAAATATGA
7A-1829510-T2-25_896	AAAGAGAATTACATGT
7A-1829510-T2-25_960	AGTTCTATCCAGATGCA
7A-1829510-T2-25_1024	ACTCCACAAAGTTCTTT
7A-1829510-T2-25_1088	TCTATATATTTTTCTACA
7A-1829510-T2-25_1152	AAAGATACGAGTAAAT
7A-1829510-T2-25_1216	CAACGGTCTTCGTTGC
7A-1829510-T2-25_1280	TACTTATAAGCTCCATC
7A-1829510-T2-25_1344	AAATATTAGTACCAATG
7A-1829510-T2-25_1408	GTAGTAAATACCTGATA
7A-1829510-T2-25_1536	AAGGAAAAAAAATTCT
7A-1829510-T2-25_1580	CGATCGGAAGAAGGG
7B-1922330-T2-25_0	TTGTATCCaaggaaagga
7B-1922330-T2-25_64	TTTTGGGGCCAACGGA
7B-1922330-T2-25_128	TTCTTTTCTTATTTTTCC

7B-1922330-T2-25_320	CTAGCGACCAATTTTCT
7B-1922330-T2-25_384	GAGATACATCAAAACA
7B-1922330-T2-25_448	TCTCATCTCTCTTTCAT
7B-1922330-T2-25_512	TTTTCAGTGTGTTTCAT
7B-1922330-T2-25_576	AACTGACACTATCCATA
7B-1922330-T2-25_640	TTGTGCCTTTCTAGACA
7B-1922330-T2-25_704	CTTCTTCTTTCCCTTTCT
7B-1922330-T2-25_768	AAAGAATTTACTTTCTT
7B-1922330-T2-25_832	TGTTCAGTTAGAAGAG
7B-1922330-T2-25_896	TGCTCCACAAGTGTATA
7B-1922330-T2-25_960	TCGGCGATGGGAGTGA
7B-1922330-T2-25_1024	TGAAGACAAGTACTTG
7B-1922330-T2-25_1088	TACGTAGTAGTAGAGT
7B-1922330-T2-25_1152	AAGAAAGCAAGCAAT
7B-1922330-T2-25_1216	TTGTGTAACATGCTGA
7B-1922330-T2-25_1280	GGCGATGAGGGAGAC
7B-1922330-T2-25_1344	CTAATTTATGTTCCGTC
7B-1922330-T2-25_1408	TGAATACATTTTGACAA
7B-1922330-T2-25_1472	GTGAAATAATATTGTTC
7B-1922330-T2-25_1536	ATTATGATGCACCAAGT
7B-1922330-T2-25_1580	GACACAAAATTCGGCG
7D-2054220-T2-25_0	TTTGTATGCaaggaagga
7D-2054220-T2-25_64	TTGCGGCCAACAAATT
7D-2054220-T2-25_128	CTTTTCTTATTTTTCCA
7D-2054220-T2-25_256	AGGGGTGGCCAACAA
7D-2054220-T2-25_320	ATGATGCTAGCTACGA
7D-2054220-T2-25_384	TGGAACACATCACAAC
7D-2054220-T2-25_448	TCTCCCATCTCTCTTTC
7D-2054220-T2-25_512	ATCTTTGTTTTCAGTGT
7D-2054220-T2-25_576	CAGAAAAAGCGACACT
7D-2054220-T2-25_640	TTCCTTGTGCTTTTCTG
7D-2054220-T2-25_704	TTAGCTTGTTTTTTCCC
7D-2054220-T2-25_768	AAAGAATTTACTTTGTT
7D-2054220-T2-25_832	AATGAGTAACTGACAA
7D-2054220-T2-25_896	CAAAATATAACAACCCT
7D-2054220-T2-25_960	TCATTATTGGGTTGTCA
7D-2054220-T2-25_1024	ACCAATGATAATCTACT
7D-2054220-T2-25_1088	CCCGATAGTTGTTTGA
7D-2054220-T2-25_1152	TTTGGGTATTTACTACT
7D-2054220-T2-25_1216	GGTTGGAGAGAGCAT
7D-2054220-T2-25_1280	CTCTTCGAAGGCAGG
7D-2054220-T2-25_1344	CCTCAAGAAAAACTTT
7D-2054220-T2-25_1408	AGAGAGGAGGCAGAA
7D-2054220-T2-25_1472	ACCTGGGTTGGGCGC
7D-2054220-T2-25_1536	CAAATCTTCCTATGGCA
7D-2054220-T2-25_1580	TCTACACAAATAGATAA
6B-1669490-T2-26_0	GGGGATTCATTTTGCC
6B-1669490-T2-26_256	TTGGCGAAGAGCCTG
6B-1669490-T2-26_320	GAGCTAGCCATGTATG
6B-1669490-T2-26_384	ACCGAAATGTACTTAAT
6B-1669490-T2-26_448	ATAATATTTGATATGCA
6B-1669490-T2-26_512	GAAAGAAATTTGAGAT
6B-1669490-T2-26_576	GATGAATCATGCATTG
6B-1669490-T2-26_640	GGTTATACTCAACCACT
6B-1669490-T2-26_704	TTGGAAGAAAGTTAGT
6B-1669490-T2-26_768	CTCATGCAGATGGGAT
6B-1669490-T2-26_832	CCTTTGCTAATTTTGTC
6B-1669490-T2-26_896	CTATGGGTTGTAATCAT
6B-1669490-T2-26_960	GGAGGCGTGCAAGAA
6B-1669490-T2-26_1024	CGAACGTTTGGGACTT
6B-1669490-T2-26_1088	TTTCTGTTTCCCACCCT
6B-1669490-T2-26_1152	ATGTTTTTGTCACGCA
6B-1669490-T2-26_1216	TTTTGTTTGCGTGAATT
6B-1669490-T2-26_1280	AAAGCTTCCAATTATG
6B-1669490-T2-26_1344	ACTCATGCAGATGGGA
6B-1669490-T2-26_1408	TCCTGTGCTAATTTTGT
6B-1669490-T2-26_1472	CAAACTATGGGTCGTA
6B-1669490-T2-26_1536	GAATGGAGGCGTGCA
6B-1669490-T2-26_1580	GACCGATGGAGATCAT
6D-1733450-T2-26_0	ATTGCTTTTGTAGGGA
6D-1733450-T2-26_64	AGAACATGTATTTATCT
6D-1733450-T2-26_128	GTTTTCATTGGCACATT
6D-1733450-T2-26_192	TTTGACCTTGCATCTAT
6D-1733450-T2-26_256	TGACCTTACTACATACT
6D-1733450-T2-26_320	ATAAGTTATCTGTGCTA
6D-1733450-T2-26_384	ACTATACGTGGTAATTG
6D-1733450-T2-26_448	TGCTTACTAGTACTACG
6D-1733450-T2-26_512	TATTTTACTGCACCGTA
6D-1733450-T2-26_576	AATTAGTTTGTCAGGG
6D-1733450-T2-26_640	AGCGTCAGAGTGCTG
6D-1733450-T2-26_704	TTCATTACTATCATTTA
6D-1733450-T2-26_768	AGCAACCAGCGCGTA
6D-1733450-T2-26_832	GTGGGGAAAGAAATG
6D-1733450-T2-26_896	AAATAGGAGTTTTACG
6D-1733450-T2-26_960	TTTCCACCCCGTACCG
6D-1733450-T2-26_1024	CCTCACGCAAAGGTGT
6D-1733450-T2-26_1088	CGCATGAATTAACTTA
6D-1733450-T2-26_1152	AATTTTATGAACAGAT
6D-1733450-T2-26_1216	GCATGTATAATATATAC
6D-1733450-T2-26_1280	TTTATGTCCGTTCTTTG
6D-1733450-T2-26_1344	GGATGTAGCTAATTTTG
6D-1733450-T2-26_1408	AACTATGAGTTGTAAT
6D-1733450-T2-26_1472	ACGGAGGCGTGCAAG
6D-1733450-T2-26_1536	CTCTTTATCTTTCTCTC
6D-1733450-T2-26_1580	TTGTAGCTACCCATATA
5A-1199980-T2-27_64	CGGCCCGCTGCGCTCT
5A-1199980-T2-27_320	TTTGTCGTGTTTATTGG
5A-1199980-T2-27_384	CCTTGCAAAAGGCAAA
5A-1199980-T2-27_448	GATTAAAAATTACATCT
5A-1199980-T2-27_512	GCAGTGTGCCGTGAG
5A-1199980-T2-27_576	TAAGGAGTTTGTAGAA
5A-1199980-T2-27_640	CGATTTGTGTAACAGA
5A-1199980-T2-27_768	TCAACCTTCATCTGCCG
5A-1199980-T2-27_832	ACGTGACACTACCACT
5A-1199980-T2-27_896	GGACACACCACCCTCC
5A-1199980-T2-27_960	CGGCAAGGATTTATTC
5A-1199980-T2-27_1024	CCCCCTCCTCCCAAAG
5A-1199980-T2-27_1088	AACGTTTATCGAACCA
5A-1199980-T2-27_1152	TATAACTACCTTGGCCT
5A-1199980-T2-27_1216	ATCTTGAGCTATTTTGA
5A-1199980-T2-27_1280	TGGCGTGCATGACCCC
5A-1199980-T2-27_1344	CGGCAGAGTTGGAGT
5A-1199980-T2-27_1536	CAAGTTCTCCTGGTTA
5A-1199980-T2-27_1580	TCAAGATCCATTCAGC
5B-1299180-T2-27_128	TCCCCTCTGATGATTCG
5B-1299180-T2-27_192	TCACAAACTTAAGCTA
5B-1299180-T2-27_256	ACGTAACACACAGAGA
5B-1299180-T2-27_320	CGAGGAAAAGTCAACA
5B-1299180-T2-27_384	ACAAATCTGTCCAACC
5B-1299180-T2-27_448	GCTAGTTCAGAAAAAT
5B-1299180-T2-27_512	TTTGAACACCCATACTT
5B-1299180-T2-27_576	ATCATAGAGTAACGCT
5B-1299180-T2-27_640	ATTTTTTCTTGACACAG
5B-1299180-T2-27_704	TAAGAACACGCATAAA
5B-1299180-T2-27_768	AAACTCTGTTCATTTTT
5B-1299180-T2-27_832	CATATACAATAAGTTTT
5B-1299180-T2-27_896	GGTTTGTGTATATCAAA
5B-1299180-T2-27_960	AACTACTAGCAAAGCA
5B-1299180-T2-27_1024	CAAAATAAAGTAGACG
5B-1299180-T2-27_1088	ATCCCCAGCATTAAAG
5B-1299180-T2-27_1280	AGGATAAGAGACATCA
5B-1299180-T2-27_1344	TATAAATAGAAAAGAA
5B-1299180-T2-27_1408	TTCCAAACAGTAAACA
5B-1299180-T2-27_1580	AGACGGCCTAGACCAC
5D-1428360-T2-27_256	CCGTTGCAGCGCATTT
5D-1428360-T2-27_320	TTGGACGGCTGGGAAT
5D-1428360-T2-27_384	CAAAAGGCCACATATT
5D-1428360-T2-27_448	CATCTTCATAGTTTAGG
5D-1428360-T2-27_512	AGCAAAGCTGTTGCCG
5D-1428360-T2-27_576	TTGTAGAAGCGGCGGT
5D-1428360-T2-27_640	CCCGACGAGGCCGAA
5D-1428360-T2-27_704	ATAGTCGCGTCATCCG
5D-1428360-T2-27_768	AACCCAGATAACTAGA
5D-1428360-T2-27_832	AGCATCGAAACGAGG
5D-1428360-T2-27_896	GCCTTTTGCTCCCTCTC
5D-1428360-T2-27_960	ATTTTTTTCAAAGTGA
5D-1428360-T2-27_1024	GCACACACAAATACAT
5D-1428360-T2-27_1088	GATCGGAGATTCCATT
5D-1428360-T2-27_1152	ACACCTGGAATTCTGG
5D-1428360-T2-27_1216	GACAATGCGCTGACTG
5D-1428360-T2-27_1408	TGCACATCTGCCTGCC
5D-1428360-T2-27_1472	ATCCATTCTGTGCACCG

5D-1428360-T2-27_1536	TTCTGGCGAGCCCGAT
5D-1428360-T2-27_1580	CTTCCATTCTTGACACT
3B-0717810-T2-28_0	ATGCTCATTGGTTGATG
3B-0717810-T2-28_64	CTCCGCTTGCAAAATC
3B-0717810-T2-28_128	AGGAGAGTTTGGCCT
3B-0717810-T2-28_320	TTAGATTCATAGACCA
3B-0717810-T2-28_384	GGCATTCGGAAAACG
3B-0717810-T2-28_512	GCGGCCAGCGTCGCT
3B-0717810-T2-28_576	TTGTATTTGTTGTGTCC
3B-0717810-T2-28_640	TAGTTGTGTTGCATATG
3B-0717810-T2-28_704	ttacattatataattgcattgc
3B-0717810-T2-28_768	atataatctgaaatcacatac
3B-0717810-T2-28_832	GGTGCTTTTGGCACCC
3B-0717810-T2-28_896	ACTGCGTCTTTTGAAA
3B-0717810-T2-28_960	GTTGATATGTTCATTCT
3B-0717810-T2-28_1088	CATTCCTAGTTTTCTTT
3B-0717810-T2-28_1152	GGACTTGGAAAGCTG
3B-0717810-T2-28_1280	AGGGTTTTATGTCCGT
3B-0717810-T2-28_1344	GTAGCTAACTTTGTCG
3B-0717810-T2-28_1472	GCCCAAGCTATAGGCA
3B-0717810-T2-28_1536	CTTCCACCTTTTTAGCT
3B-0717810-T2-28_1580	ATCAAGAGTGTAGTTA
3D-0935160-T2-28_0	TCTTCCTCTATGCACAG
3D-0935160-T2-28_62	GGATAACTACACTCTTG
3D-0935160-T2-28_248	GTGATTACAACCCATA
3D-0935160-T2-28_434	ATTACGTTGACACATCA
3D-0935160-T2-28_496	TGCATAATTGTCAGCTT
3D-0935160-T2-28_620	GGAAATCATTAAATGG
3D-0935160-T2-28_682	GGAAAAGAAGAGAAT
3D-0935160-T2-28_806	ATTTGAACGTCTGCCG
3D-0935160-T2-28_868	caatatgatttcatatgtgatt
3D-0935160-T2-28_930	tatggtttcacatgcaatgca
3D-0935160-T2-28_992	ATTTCATATGCAACACG
3D-0935160-T2-28_1178	GTCGGCGCCATTAGTG
3D-0935160-T2-28_1364	TAGATCGAAAGTTTTC
3D-0935160-T2-28_1426	CAACACATATCATTCTT
3A-0650140-T2-29_64	CATATCAATTAGAGGG
3A-0650140-T2-29_128	GTCTGTGATAATGGAT
3A-0650140-T2-29_192	TTTTATCCCGTCTGATG
3A-0650140-T2-29_256	ACATTCATTCATACACG
3A-0650140-T2-29_320	CAAAGAAACATGGAG
3A-0650140-T2-29_384	TAAAGAAGCATCATAC
3A-0650140-T2-29_576	TCCCATGGGCTAAACT
3A-0650140-T2-29_896	GAGGGTCCAGATGTTT
3A-0650140-T2-29_960	CTTTTGGTAACAGATC
3A-0650140-T2-29_1024	CTTGCAGACCCTGGAT
3A-0650140-T2-29_1088	AGTGCACCTTCTACGC
3A-0650140-T2-29_1152	GCACTTTCTTTTTGTTT
3A-0650140-T2-29_1216	AACCGCTTCTACAAAA
3A-0650140-T2-29_1280	CAAACAAAGCACTAAT
3B-0753850-T2-29_64	GACGGTAAGAACTCTG
3B-0753850-T2-29_128	GAAAATTTTGGGCGA
3B-0753850-T2-29_192	TAAGTGGTAGAGGAC
3B-0753850-T2-29_256	TTTGATAGTTTCTTTGG
3B-0753850-T2-29_640	AAATGTTAAAATATGC
3B-0753850-T2-29_1024	AAACCATCATACTACTA
3B-0753850-T2-29_1152	CCCTCCAGAGGCCACC
3B-0753850-T2-29_1216	TAATACATACGTCATGT
3B-0753850-T2-29_1280	TATCTCTTTGCCTCTAA
3D-0841320-T2-29_128	AAATTTGGGAGAGAC
3D-0841320-T2-29_192	AAAAGCAGGAGAAAA
3D-0841320-T2-29_256	GCTCCTTGATTTGCGA
3D-0841320-T2-29_640	AATATGAATTTTGCAAA
3D-0841320-T2-29_1152	TATACTATATGAGACTG
3D-0841320-T2-29_1344	CCCGTGGTAGGCCTGC
3D-0841320-T2-29_1408	TTACCCAGGTTTAGGC
3D-0841320-T2-29_1472	TGTGTATGATCTTTTAT
3B-0717800-T2-30_64	GATCTGGACGATACTG
3B-0717800-T2-30_256	TCCTAGATCACAACAAT
3B-0717800-T2-30_320	CGTTCGGCTGAAAAG
3B-0717800-T2-30_448	AATAATAATAATTACATT
3B-0717800-T2-30_512	AGAGGCATTTGGAAA
3B-0717800-T2-30_576	TAAGGCTCACTAGTGG
3B-0717800-T2-30_704	TGTACTTATTGTATTCG
3B-0717800-T2-30_768	ataataataataataataata
3B-0717800-T2-30_832	TTGTATTGCTTATGAAA
3B-0717800-T2-30_896	cattGCCTATGAAACCA
3B-0717800-T2-30_960	GTGCCAAGCCTTTGGC
3B-0717800-T2-30_1024	TTGGACTGAGTCTTTTT
3B-0717800-T2-30_1088	CCTCATCGATATGTGCA
3B-0717800-T2-30_1152	TGCTTCTTGAGCACTC
3B-0717800-T2-30_1216	TGCTAATTTTCTTTGCG
3B-0717800-T2-30_1280	ATGCAAACAGATCCGC
3B-0717800-T2-30_1344	CATTTCACCTACCATAT
3B-0717800-T2-30_1408	AGCTTTATAGTAGTGG
3B-0717800-T2-30_1536	GCCACCCACGCAGATC
3B-0717800-T2-30_1580	CCTTTTTATCTTTATCTA
3D-0932870-T2-30_0	TTAGAGTAACCCTCTA
3D-0932870-T2-30_64	AGGCCCCAATCTTCGT
3D-0932870-T2-30_128	TATGTTGGTTGCCAAC
3D-0932870-T2-30_192	GGGTTGGGCTGAAAA
3D-0932870-T2-30_320	AAATAATAAAAATTATA
3D-0932870-T2-30_384	GTAGAGGCATCTAGAA
3D-0932870-T2-30_448	CATAAGGCTCACTAGT
3D-0932870-T2-30_512	GGCCAACGACACTGAT
3D-0932870-T2-30_576	TTGTTTTCGTGGTGTT
3D-0932870-T2-30_640	TGTGTTGTATATGAAGT
3D-0932870-T2-30_704	attatataatcgcattgcatat
3D-0932870-T2-30_768	acattacattatataatctgaa
3D-0932870-T2-30_896	TGAACTTTGTCTTGTTG
3D-0932870-T2-30_960	CTTTTCCACCAAACTC
3D-0932870-T2-30_1024	TGCATTGGCTATATTCT
3D-0932870-T2-30_1088	GACATATGGTGCCAAG
3D-0932870-T2-30_1152	TCTAACAAACTATGGG
3D-0932870-T2-30_1344	GCATTTCCCTTGCCATA
3D-0932870-T2-30_1536	AGCCACCCACGCAGAT
3D-0932870-T2-30_1580	ACCTTTTTAGCTTTATC
2A-0343000-T2-31_186	TTGACACGTGGTGGG
2A-0343000-T2-31_310	CTCGACACCATTTCTTC
2A-0343000-T2-31_496	tctaCCCCGGACATGCT
2A-0343000-T2-31_682	CTCCTCCAGCGTACCG
2A-0343000-T2-31_744	CTGGTTGAAGTCTCCA
2A-0343000-T2-31_806	TAAGTACTATGGTCGAT
2A-0343000-T2-31_992	CTACTAGTGATGCGCG
2A-0343000-T2-31_1054	ATGCCCTTTATTGGTTA
2A-0343000-T2-31_1116	TTAATGGGTATTTTGGG
2A-0343000-T2-31_1240	AGCATGCACGAGAGA
2A-0343000-T2-31_1302	AGAAAAATGGTTTGGC
2A-0343000-T2-31_1364	ATTGCAATAGGGGCAA
2A-0343000-T2-31_1426	ATTGAAATATGTTGAG
2A-0343000-T2-31_1550	TTTCTGGCTATTGAAG
2B-0472380-T2-31_192	CACGCGGCAGGCGTC
2B-0472380-T2-31_256	TTTTGTTACCGTACTCT
2B-0472380-T2-31_448	TCCTTCTCATTTTGTAT
2B-0472380-T2-31_512	ctctctctACCCCCGACA
2B-0472380-T2-31_640	ATTAAACACTCCACTAG
2B-0472380-T2-31_704	GGGGCACATATTACAA
2B-0472380-T2-31_768	AAACAAGGTCGGATCT
2B-0472380-T2-31_960	GGCGTGAACATGTCAC
2B-0472380-T2-31_1024	TGTCACTAGTACACTAA
2B-0472380-T2-31_1088	AACACTTAGCAGTGAT
2B-0472380-T2-31_1152	AAGTCATTTTGCAACG
2B-0472380-T2-31_1280	GGTTTTTTCGTTCAGAT
2B-0472380-T2-31_1536	TTTGCTTGCAAGGGAT
2B-0472380-T2-31_1580	TGATTGCTTTGCAAGG
2D-0595670-T2-31_198	TTGACACGCGGCGGG
2D-0595670-T2-31_264	CTTTTTCTTACCGTACG
2D-0595670-T2-31_462	TCTCATTTTGTATCAtct
2D-0595670-T2-31_528	CCCCGACACGCTCATA
2D-0595670-T2-31_726	CCCGGGCGCACATTAC
2D-0595670-T2-31_792	AAATAAGGTCGGATCT
2D-0595670-T2-31_858	TTTCATTGGCGATAGAT
2D-0595670-T2-31_990	GGTGGGGCGTGAACA
2D-0595670-T2-31_1049	ATGGGCCATTTGTTACT
7B-1935150-T2-32_0	ATTATTCTTTCTAGCTT
7B-1935150-T2-32_62	GTGGCTGTAAGCAATA
7B-1935150-T2-32_124	GAGGAGCTGCCAGCT
7B-1935150-T2-32_186	GCACTTAATTAGTTTGT
7B-1935150-T2-32_248	ATTGAGATATTCGTAG
7B-1935150-T2-32_310	TTTCTTTCGAACCATGT
7B-1935150-T2-32_372	GAAACTTTTAGCTAGT
7B-1935150-T2-32_428	TTTTTGCGCCATGTGA
7B-1935150-T2-32_907	TTATTTCCCCAAAAGCA
7B-1935150-T2-32_969	ACCTTGCCATCTTGTAC
7B-1935150-T2-32_1031	AAAAATTCACAATGTG
7B-1935150-T2-32_1279	TCTTATATTTGTGAATG

										7B-1935150-T2-32_1341	TTTTTTTTCTCTCACTC
										7B-1935150-T2-32_1403	AGTCTAGGAGCAACTT
										7B-1935150-T2-32_1465	TAAAAATATTTATCAAC
										7B-1935150-T2-32_1527	TATTTCTCTTCAATTTG
										7B-1935150-T2-32_1580	ATTTGTATATATGTGAC
										7D-2082710-T2-32_0	GTTATTCTTTCTAGCCT
										7D-2082710-T2-32_58	CTAGTGGTTGTAAGCA
										7D-2082710-T2-32_116	GAAAGCTCCAGCTATT
										7D-2082710-T2-32_174	GTGCACTTAATTAGTTT
										7D-2082710-T2-32_232	GGAAGCATTGAGATAT
										7D-2082710-T2-32_290	GGCATCCTTTTTTTTC
										7D-2082710-T2-32_348	CGGTGCTTAAAGTGGA
										7D-2082710-T2-32_929	AAAAACAGAGTCAGT
										7D-2082710-T2-32_995	ATTTTTCTTCCGGATAG
										7D-2082710-T2-32_1193	TGCTTACAGTCTTTCTG
										7D-2082710-T2-32_1523	CTAGATACATTCAACTT
										7D-2082710-T2-32_1580	GTAGTAAATATGCATGT
										2A-0344470-T2-33_0	CTCTTCAGAGCAGGCA
										2A-0344470-T2-33_62	AATGGCAAAAAATATA
										2A-0344470-T2-33_124	TCTCCCTCGGAGATGA
										2A-0344470-T2-33_186	TGACCCCCCACCCAC
										2A-0344470-T2-33_248	CACGGCTTTTCCGGTT
										2A-0344470-T2-33_310	CCCACGTTGGCACGTC
										2A-0344470-T2-33_372	AGACGGCAGCGGCCC
										2A-0344470-T2-33_558	AGGAGAGCGAGAGCA
										2A-0344470-T2-33_868	AGACGAACCGCAGTT
										2A-0344470-T2-33_930	CTGCAACTTGCGGACG
										2A-0344470-T2-33_1054	TCTGGGCTGTATCCGAT
										2A-0344470-T2-33_1178	GCTGATGGGATCTGGA
										2A-0344470-T2-33_1240	TCCCTCCACCCCAAAAT
										2A-0344470-T2-33_1550	CGATTACGATGGGCCA
										2A-0344470-T2-33_1607	TCGAGATCCCTGGACG
										2B-0494910-T2-33_0	CTCTTCAGAACAGGCA
										2B-0494910-T2-33_64	AAACAAAGAAAAGAA
										2B-0494910-T2-33_128	AATGAATCCGCCATTT
										2B-0494910-T2-33_192	CCCCGCACACTACTCAT
										2B-0494910-T2-33_256	AATATCTTATCAGCGTC
										2B-0494910-T2-33_448	AGAGCACCGCTCACTC
										2B-0494910-T2-33_576	GCCACGGACGGGGAG
										2B-0494910-T2-33_640	TGGATGGAGGCACGG
										2B-0494910-T2-33_960	GAGACGAACCGCAGT
										2B-0494910-T2-33_1024	TACGGCTGCAACTTGA
										2B-0494910-T2-33_1088	TGTCCATAGGGCAAGT
										2B-0494910-T2-33_1216	GGTCCCAAGATAAGTT
										2D-0577070-T2-33_0	CTCTTCAGAGCAGGCA
										2D-0577070-T2-33_64	AAAAAAAGATGGCTTA
										2D-0577070-T2-33_192	CTCCCCATTCCTCCGAA
										2D-0577070-T2-33_384	CAGCTCAGAGGCGAA
										2D-0577070-T2-33_576	GGAGGGAGGAGGCTC
										2D-0577070-T2-33_960	CGTCCGTCTCGATGCG
										2D-0577070-T2-33_1088	CATCTGGAAGGGATGA
										2D-0577070-T2-33_1152	GTAAAATTAAAGTGCG
										2D-0577070-T2-33_1216	TATTTTCTCGCCGTAAA
										2D-0577070-T2-33_1280	CTCCAATTGTTTACTGA
										2D-0577070-T2-33_1472	TGGGCTACCACTAGAT
										2D-0577070-T2-33_1580	GACTTCACATCTAGGC
										4B-1063570-T2-34_64	TGCTCGCATATATACCC
										4B-1063570-T2-34_256	TTTCTCGTCATGGTTGA
										4B-1063570-T2-34_320	GCGAGTTTATACATGC
										4B-1063570-T2-34_384	AACATGTGACCTACTC
										4B-1063570-T2-34_448	CCCAGCTCCTCGACGG
										4B-1063570-T2-34_512	ATGTAATAAAACATCTC
										4B-1063570-T2-34_576	GTGATCCATTTAATGCT
										4B-1063570-T2-34_640	TAGTGTTGTTGTGAGA
										4B-1063570-T2-34_704	TTATGATGAGGGGATA
										4B-1063570-T2-34_768	AGCCTAGTGATAGAAA
										4B-1063570-T2-34_832	CGGGTAAAGAGAGTG
										4B-1063570-T2-34_896	GTATGCCGAAGTTTTT
										4B-1063570-T2-34_960	ATCCAAGTGACTTGGC
										4B-1063570-T2-34_1024	ATTTTTATGACTATGTG
										4B-1063570-T2-34_1088	CCTTGTTTACGAAAGA
										4B-1063570-T2-34_1152	ACTCCAAAAATACTTTA
										4B-1063570-T2-34_1216	AAATCTAATGCAACCA
										4D-1137420-T2-34_0	GGTATTCTAGCCAAGT
										4D-1137420-T2-34_64	ATACATGCTTGCATATA
										4D-1137420-T2-34_256	CCGCTCGAACCTAGCT
										4D-1137420-T2-34_320	TTATCTCGTCAATTAAG
										4D-1137420-T2-34_384	GTTTTGCATGCAGTTC
										4D-1137420-T2-34_448	ATCATTTTTCGAGTTAA
										4D-1137420-T2-34_512	GATATTTTCTCACCTTC
										4D-1137420-T2-34_576	CATGATGTTAGCGAGC
										4D-1137420-T2-34_640	TTTAATTTTCAAAAGA
										4D-1137420-T2-34_704	ATTTGGGAGCTCTAAA
										4D-1137420-T2-34_768	AATCCTTAACTCGCAAT
										4D-1137420-T2-34_832	CAAAGTCGTCGCTTGC
										4D-1137420-T2-34_896	AGTTCCACATATCTCAT
										4D-1137420-T2-34_960	GTCGCGCCACGCTGAC
										4D-1137420-T2-34_1024	ACTATATAAAATGTACA
										4D-1137420-T2-34_1088	TCATTATGCTTCTTATTA
										4D-1137420-T2-34_1280	TCGATCCCATCTTCTCC
										4D-1137420-T2-34_1344	CAGTTTTTTTCGGGCC
										4D-1137420-T2-34_1408	CAGACACTTCCGTACA
										5A-1225500-T2-35_0	AAAGGTATTTTTTTAGA
										5A-1225500-T2-35_64	TGGCAGGTCGACAAGT
										5A-1225500-T2-35_128	AAAACTATGTTTTGGA
										5A-1225500-T2-35_192	AAGtgatgatgatgatgatg
										5A-1225500-T2-35_256	GTGTGAGAGTCTTATG
										5A-1225500-T2-35_320	TCTTCTACTCTTTTAAC
										5A-1225500-T2-35_384	TCTCATTGCGCATGTTT
										5A-1225500-T2-35_576	TTTATTGTGTTGGGGT
										5A-1225500-T2-35_640	AGCTCGATCCGCGGTT
										5A-1225500-T2-35_704	GGGGAGACAGAGTCC
										5A-1225500-T2-35_896	CTGAACCGATCTTCAA
										5A-1225500-T2-35_960	TGCGCCGCCAGCCAAT
										5A-1225500-T2-35_1024	TCCAAGGGCCCACCTT
										5A-1225500-T2-35_1152	GTTAACGGCAACCCCT
										5A-1225500-T2-35_1216	TTCTTAGTTTAAGGGA
										5A-1225500-T2-35_1280	AGGTTTACAAAAGTGC
										5A-1225500-T2-35_1344	AAATAGGAGGGGCAA
										5A-1225500-T2-35_1408	GTCGGTCCGTTAATTC
										5A-1225500-T2-35_1472	TTACTAAAATAAAAATG
										5B-1286990-T2-35_0	TTTTTTATTTTATGCAA
										5B-1286990-T2-35_64	AGTGGTGTGAGAGTCT
										5B-1286990-T2-35_128	GTTTCTCATGAATTAAC
										5B-1286990-T2-35_192	AGGCCACGACTTCAGA
										5B-1286990-T2-35_256	CAGAATGGCAAGGCC
										5B-1286990-T2-35_448	AGTGATTCTCAATGTCT
										5B-1286990-T2-35_512	AGGATACATCTTAGTC
										5B-1286990-T2-35_576	TGCACGCCAAGCCTTC
										5B-1286990-T2-35_640	ACAGAGGGCTACTAAA
										5B-1286990-T2-35_704	GGACACCATCAATAGC
										5B-1286990-T2-35_768	TCATCGTCTCCGGAGC
										5B-1286990-T2-35_832	CGCCGAGTTGCTTCCC
										5B-1286990-T2-35_896	GGCATGCACATCAGAA
										5B-1286990-T2-35_960	CACGAAGGTTTATCTG
										5B-1286990-T2-35_1152	CCCCTTGATCCCCGTG
										5B-1286990-T2-35_1216	AGGGAGTTGTTGGAG
										5B-1286990-T2-35_1280	AGTGCTAGAGTTGCTC
										5B-1286990-T2-35_1344	GCAACTCGAACTATGC
										5B-1286990-T2-35_1408	TCGTGTAGAAAAAAAA
										5B-1286990-T2-35_1472	GGCCCCTCCCTCATAA
										5D-1438030-T2-35_0	CATGGCAAATTCACAA
										5D-1438030-T2-35_64	CCCGTACATTTTACCCT
										5D-1438030-T2-35_320	CATATAAAGAACCCAA
										5D-1438030-T2-35_384	AGGAAGGAAACTCAA
										5D-1438030-T2-35_448	GTACAGTCGCCATGTA
										5D-1438030-T2-35_768	AAATCAAAGGAGGACT
										5D-1438030-T2-35_832	CCACGAACCCTCCACC
										5D-1438030-T2-35_1408	CCGCCGTCTCCCGTGC
										5D-1438030-T2-35_1472	ATTTTCGGAGGCAACT
										5B-1385620-T2-36_192	GGCAGGGGGAAAAAG
										5B-1385620-T2-36_256	ACCGGGTTTAATAATCT
										5B-1385620-T2-36_320	TGAGTTTTTTGTGAGC
										5B-1385620-T2-36_640	GAGATATCACTGTCTTC

										5B-1385620-T2-36_704	TCTAACATTTTGGGTAT
										5B-1385620-T2-36_768	CTAAAACATGACTTAGT
										5B-1385620-T2-36_832	TTTAGTCCTTGGTGCA
										5B-1385620-T2-36_896	CCTAATAACTTGCTACA
										5B-1385620-T2-36_1024	GAGTCATTGTTTATCGT
										5B-1385620-T2-36_1088	TCCTATGAAACAACCTA
										5B-1385620-T2-36_1152	TTAACCATATGTTTTTA
										5B-1385620-T2-36_1216	TACTCATTGTATGTTTC
										5B-1385620-T2-36_1280	ACACAATGGCTAATAC
										5B-1385620-T2-36_1344	TTATCATGTTCTATTAC
										5B-1385620-T2-36_1408	GACCCGCACAATTACA
										5B-1385620-T2-36_1536	TAAAATTATGTGTGCTT
										5B-1385620-T2-36_1580	ATTTGTAAGCATTCAAA
										1A-0022300-T2-37_64	CAGAAATCCGTTAACT
										1A-0022300-T2-37_128	AAAAAGAGCACGGAC
										1A-0022300-T2-37_192	TATACTTCAGAACAATG
										1A-0022300-T2-37_320	TTCTTCCTTTGCTGTTT
										1A-0022300-T2-37_512	CAGAGCCTAGAAGCCG
										1A-0022300-T2-37_640	ACAGGTAGAGATCGAG
										1A-0022300-T2-37_704	CCTTTTTACAGCGAGC
										1A-0022300-T2-37_768	CTAATTCAGACAAGAG
										1A-0022300-T2-37_832	AAACCGAGGGGCCGT
										1A-0022300-T2-37_896	GGCCGCTGGCCCACCA
										1A-0022300-T2-37_1088	GGCAGCGGCCCGCCA
										1A-0022300-T2-37_1216	GCGACCGGGCAAGCG
										1B-0119450-T2-37_183	AAACCACTGCTGCTAA
										1B-0119450-T2-37_244	AGCTGTACTTCAACTG
										1B-0119450-T2-37_305	ATTTAAATAAAGCTTCC
										1B-0119450-T2-37_366	TCCTTTAAGTAATAAAG
										1B-0119450-T2-37_427	CAGATTTTCTAAAATTC
										1B-0119450-T2-37_610	GACGGCCGCCACCTCG
										1B-0119450-T2-37_671	AGGTATTTTTATTTTTT
										1B-0119450-T2-37_976	ATTCGCAGTCCACCTG
										1B-0119450-T2-37_1037	ACCTTGAGAAATCCGT
										1B-0119450-T2-37_1098	GGGCAAAAAGAGCAC
										1B-0119450-T2-37_1159	AAGCTCAGTAGTAGTA
										1B-0119450-T2-37_1342	AGCGAGGCAGAATATC
										1B-0119450-T2-37_1403	GCGGTGCAGTGCAGT
										1B-0119450-T2-37_1464	TGTGGACTGTATTTTAA
										1B-0119450-T2-37_1525	GAAATACTTGAAGAAA
										1B-0119450-T2-37_1586	GTCGGGAAGGCAAAC
										1B-0119450-T2-37_1647	GGTCGCCGTCAGTTAA
										1B-0119450-T2-37_1769	TCCCCGGCGGCTGGCC
										1B-0119450-T2-37_1952	GGAGGAACAGGCAGC
										1B-0119450-T2-37_2135	AATCAAATGCAGGGAG
										1B-0119450-T2-37_2318	GAACGGACGGTGTGG
										1B-0119450-T2-37_2379	GCGGCATGGCCCGGA
										1B-0119450-T2-37_2558	GCCCGCCACGCTACCG
										1D-0227010-T2-37_64	CAGAATCCATTAACTG
										1D-0227010-T2-37_128	TGATGAATTTACAGGT
										1D-0227010-T2-37_192	CCTCTGTAAAGCTCAG
										1D-0227010-T2-37_256	GAAAGGTGACGAGGA
										1D-0227010-T2-37_320	GATCTTGTGGCTGGCG
										1D-0227010-T2-37_512	ATGAAACCGAGCTGGT
										1D-0227010-T2-37_640	ACTGGGCACAAGTAGA
										1D-0227010-T2-37_704	TCCCTTTTACAGCGAC
										1D-0227010-T2-37_768	TCAGACAGGAGGAAA
										1D-0227010-T2-37_832	ATCCTGAAATTAAGTTG
										1D-0227010-T2-37_896	TTTTGCCCCAACCACG
										1D-0227010-T2-37_1088	CCCGCCCGCCACCTTT
										1D-0227010-T2-37_1280	GCCCCGCTGATGTGGT
										1D-0227010-T2-37_1408	TGCACTGCAAGCGAAC
										1D-0227010-T2-37_1472	GTAGCGTGAGGCCGG
										5A-1192890-T2-39_64	GTGGGGCTGGCGGCA
										5A-1192890-T2-39_256	CACGCGTGTCCATATTT
										5A-1192890-T2-39_320	GGGCAACTCTGTTTTT
										5A-1192890-T2-39_384	CCCCTAGAATCGAAAT
										5A-1192890-T2-39_448	GGTATGCGGGGGCCG
										5A-1192890-T2-39_512	TAGGCGTACTCCTCTGT
										5A-1192890-T2-39_640	ACGATCCGGACGACCC
										5A-1192890-T2-39_704	CTTGCCCATCTTTTCGG
										5A-1192890-T2-39_832	CAGCGCTGCTTCAATG
										5A-1192890-T2-39_896	AAAAGCGTTAATCAGT
										5A-1192890-T2-39_960	CGAGTCGTGTCCTCCC
										5A-1192890-T2-39_1024	AGGGTTTTGGGTTGG
										5A-1192890-T2-39_1088	CCTAGATGTTTGCAAA
										5A-1192890-T2-39_1152	GGCACAAAGTGTGTG
										5A-1192890-T2-39_1216	ATGCCTTAATGATGGA
										5B-1354680-T2-39_60	tgaggtgaggtgaggtgggg
										5B-1354680-T2-39_120	TTCAGCGGCTGCGAG
										5B-1354680-T2-39_300	TATTTCACGTTTTCTTT
										5B-1354680-T2-39_1580	ACGGAATTGTCGAAGT
										5D-1448600-T2-39_128	CCGTTTCAGCGGCTGT
										5D-1448600-T2-39_320	TTTTTTATGACAGGAAT
										5D-1448600-T2-39_384	AGGTTCAGGCCAACTC
										5D-1448600-T2-39_448	AATTCGAAATTAGCATT
										5D-1448600-T2-39_512	GACGGCGAGGGCGGC
										5D-1448600-T2-39_576	ATGTCCGCACGGCCGC
										1A-2139570-T2-40_64	ATTGTGGGGATTGGTT
										1A-2139570-T2-40_128	CGGTGATGTTAGTTCA
										1A-2139570-T2-40_256	GTCTGCTAAAGCCACG
										1A-2139570-T2-40_320	GTAGACGTTTGTATATC
										1A-2139570-T2-40_384	CTTGCATGGATAAGGA
										1A-2139570-T2-40_448	CATGTAGTTGAATTTTT
										1A-2139570-T2-40_576	AACACCTTTCGATGAC
										1A-2139570-T2-40_640	TAGGATCTTGAGAGAA
										1A-2139570-T2-40_832	GTCTTTCCCTGGCCTTT
										1A-2139570-T2-40_960	TTTGAATGAAATTTGG
										1A-2139570-T2-40_1024	AAAACACATATGATTTA
										1A-2139570-T2-40_1152	CATGCTGAGGTGGCAT
										1A-2139570-T2-40_1216	TGTGTAACTGCAACTTT
										1A-2139570-T2-40_1280	ACTAAGCTTCATTATCT
										1A-2139570-T2-40_1472	TGCAACCCGTTCGAAC
										1A-2139570-T2-40_1536	TGGAGGCACCCGAGC
										1D-0214200-T2-40_64	ATTGTGGAGATTGGTT
										1D-0214200-T2-40_128	CGACGAGGTTAGTTCA
										1D-0214200-T2-40_320	AGTGTAGACGTTTGTA
										1D-0214200-T2-40_384	TTTGGGTGTCTGTTAG
										1D-0214200-T2-40_448	ACTAGTGGAAGGTAGC
										1D-0214200-T2-40_512	TGTTTTGTTTCTTGTCA
										1D-0214200-T2-40_640	TTGGACTTATGTAAACA
										1D-0214200-T2-40_704	TCTAAAATTAACATAGA
										1D-0214200-T2-40_768	GGGGATTTTTCCACTT
										1D-0214200-T2-40_960	CACTTGCAGGACATCA
										1D-0214200-T2-40_1216	TCCATGCATGCTACATG
										1D-0214200-T2-40_1280	AAAAGGTTGCGACTAT
										1D-0214200-T2-40_1472	ACAGGGCCCATGATTT
										5D-pinA-T2-42_512	TTTGAAACCCTAGCCT
										5D-pinA-T2-42_576	TCCTGTTTATCCTATGA
										5D-pinA-T2-42_640	TTTCCATATTGATTATTA
										5D-pinA-T2-42_704	ATAATTGTTTCTGGGG
										5D-pinA-T2-42_768	CGATAAACCAAGGGAG
										5D-pinA-T2-42_832	GTCAGAATCAAAACCC
										5D-pinA-T2-42_896	CTAACCCGTGTTGTTTG
										5D-pinA-T2-42_960	GTTAAAAAAAATTACT
										5D-pinA-T2-42_1024	ACATTTGACATGAACG
										5D-pinA-T2-42_1088	AAGATGATGTCTTGATA
										5D-pinA-T2-42_1152	GTCTCTAACAAATTTGG
										5D-pinA-T2-42_1216	AGTTTCGCAAAAGAAT
										5D-pinA-T2-42_1280	gagagagagagagagagag
										5D-pinA-T2-42_1344	CACAAAGTTGCACATA
										5D-pinA-T2-42_1408	GATACTGGACGAAAAA
										5D-pinA-T2-42_1472	CTACTACCTAGAAAAAT
										5D-pinA-T2-42_1536	CTACAAGTTCAGTTTC
										5D-pinA-T2-42_1580	CAACAACAACTTATGG
										7A-1826470-T2-43_0	CTGGAGGTGAGCCCG
										7A-1826470-T2-43_192	TCGGGGGGTTTGCCTG
										7A-1826470-T2-43_256	gagagagagagaggagtgg
										7A-1826470-T2-43_448	CGTACGCCGGCGAGA
										7A-1826470-T2-43_512	ATCCAGACGATTCTCG
										7A-1826470-T2-43_576	TTGTGAATAATTGCGTG
										7A-1826470-T2-43_640	AGTGAAACTCTTTTGT
										7A-1826470-T2-43_960	CGCTCAATTTTTACGG
										7A-1826470-T2-43_1024	CCAAAATGTCTAATTCC
										7A-1826470-T2-43_1152	TAAGTATATGAGGAGG
										7A-1826470-T2-43_1216	GATCTTCTATCATCAAT
										7A-1826470-T2-43_1280	TAAATCCTAGAATAGA
										7A-1826470-T2-43_1344	GAAGCTTCTAAAGTGT
										7A-1826470-T2-43_1408	TGGGGGAGCGAGTTT
										7A-1826470-T2-43_1472	CAACATCACCATGTTCA
										7B-1942520-T2-43_0	GGGTGCGGGCCCGCG
										7B-1942520-T2-43_195	GCCGAGGTGGTGATG
										7B-1942520-T2-43_325	GTGCGGCCCGTGTCTG
										7B-1942520-T2-43_520	GAGAGGCTGCCTCACA
										7B-1942520-T2-43_585	CAATTGCATGGGTTTTT
										7B-1942520-T2-43_650	CATTCCCGCAAAAAAA

7B-1942520-T2-43_715	AAAGAAAGATAATACT
7B-1942520-T2-43_780	GGAAAGTGCATTCTTT
7B-1942520-T2-43_845	TCCCATTTTTCAATAAA
7B-1942520-T2-43_910	CAACACCAACCGTAAC
7B-1942520-T2-43_975	ACATATCCGGAGACGT
7B-1942520-T2-43_1040	TGTGGCGTATTTAACA
7B-1942520-T2-43_1105	AGTGTTGTCGATAGTG
7B-1942520-T2-43_1170	TCTGATACGTGGGTGT
7B-1942520-T2-43_1224	CACGAGGGAAGCCAT
7D-2043770-T2-43_192	GGCGTGGGGAACTC
7D-2043770-T2-43_256	CACTCCGCGTCTTCGT
7D-2043770-T2-43_320	GTCGATTTCTTCCTGA
7D-2043770-T2-43_512	GGTGCACCGCCGATGT
7D-2043770-T2-43_576	TGGTATCGTAATTTACT
7D-2043770-T2-43_640	GTTGGGAACGTAGTTA
7D-2043770-T2-43_704	GGTGTTAGTCCCTGTA
7D-2043770-T2-43_960	agttgagtcatctattttggaa
7D-2043770-T2-43_1152	ACTTTGTGTTTGCTGT
7D-2043770-T2-43_1216	CAGGTTGCACCATTCT
7D-2043770-T2-43_1280	TGAGTATTAATTAATCA
7D-2043770-T2-43_1344	ATGAATATTTTCCATTT
7D-2043770-T2-43_1408	GCAAGTGAGCAGGATT
7D-2043770-T2-43_1580	TGGTTGTAATGCCAAG
5D-pinB-T2-44_960	TGTATCTGGAAGTTATC
5D-pinB-T2-44_1024	GCCTTTGCATCTGGGC
5D-pinB-T2-44_1088	AGCAACATGTAAGGAG
5D-pinB-T2-44_1152	AGAAATAAAAAGGTG
5D-pinB-T2-44_1216	TCTCTCTGTCCTGATAA
5D-pinB-T2-44_1280	CTAAATACAAAACAGA
5D-pinB-T2-44_1344	ACTAATACCTTGTTAAA
5D-pinB-T2-44_1408	ACACTACAAGGCCAGT
5D-pinB-T2-44_1472	ATCTTGAGAAAAGGG
5D-pinB-T2-44_1536	TGTTGGCTCGCAAAGT
5D-pinB-T2-44_1580	GTGAGCATTAGCCAAA
7A-1809640-T2-45_0	TGTCCAAGAAATGGGA
7A-1809640-T2-45_64	ATGAGAGAACAGCATA
7A-1809640-T2-45_128	GATGTTTAAGACGGTG
7A-1809640-T2-45_192	TGAGCTATGGAATCAG
7A-1809640-T2-45_320	TAATATTTCTTTGAGAT
7A-1809640-T2-45_384	ATCTAGGAGACTAGGA
7A-1809640-T2-45_448	TAGAGAAAATGTTTAT
7A-1809640-T2-45_512	TTAGCAAGTATTTAAAT
7A-1809640-T2-45_576	TGTATAGAAAAATGTTA
7A-1809640-T2-45_896	AAAAAATCTTAAACTT
7A-1809640-T2-45_960	GTAAAATAACAATGAA
7A-1809640-T2-45_1024	aaaaatgaaggaaaaaag
7A-1809640-T2-45_1088	aacaaacaaaagggaaaga
7A-1809640-T2-45_1152	gacaaacaaggaaaaaaT
7A-1809640-T2-45_1216	TTGGTAGCGCCCTACC
7A-1809640-T2-45_1280	GGAACGAGGAGATCC
7A-1809640-T2-45_1408	TTGAGATTCCAGGACC
7A-1809640-T2-45_1536	GAAAAGGCAAAGCAG
7B-1945350-T2-45_256	TAAACTTGTATTGAATC
7B-1945350-T2-45_320	AATGAGAACCCGAGG
7B-1945350-T2-45_384	CGCAAGAAAAAAAATT
7B-1945350-T2-45_448	AGAAAAAAACTAGTAA
7B-1945350-T2-45_512	TTTTAAAGGACAAGGC
7B-1945350-T2-45_576	TAACACGAACTTGACG
7B-1945350-T2-45_640	TCAAACCGTGCTCGTA
7B-1945350-T2-45_768	ATAATAAAACATTACAT
7B-1945350-T2-45_896	ACAACCTGGACAACAG
7B-1945350-T2-45_1088	CGAAACAGTGCAGTTG
7B-1945350-T2-45_1152	CGCGAGCCCCGTTCCG
7B-1945350-T2-45_1216	CTCCCTGGGAGCTCGC
7B-1945350-T2-45_1280	TGGCGGCACCGACAG
7B-1945350-T2-45_1344	CCGTTCGTTGTGCCGC
7D-2059200-T2-45_256	TGCGGTTTGCGGGCG
7D-2059200-T2-45_384	AGGCTCCTCACCAGCT
7D-2059200-T2-45_448	GAGAAGCGAACGGAC
7D-2059200-T2-45_640	TAGATAGAGCACTGCA
7D-2059200-T2-45_704	cggtgcggcacatgcgttcgt
7D-2059200-T2-45_832	CTTTGCTTCTTCTGCTG
7D-2059200-T2-45_1024	AATAGATTTGGATTATT
7D-2059200-T2-45_1216	TCGGCTCTCAGCCCGT
7D-2059200-T2-45_1280	TGAGCTTGTGACGTTG
7D-2059200-T2-45_1344	AGATAATAAGGGAACA
7D-2059200-T2-45_1408	tttatccattattcctatttaac
7D-2059200-T2-45_1472	ATATTTTGGGTGGGAG
7D-2059200-T2-45_1536	GGTTAATTTTTTTAAAT
7D-2059200-T2-45_1580	TATTTTGTGTTCCCGGT
7B-1877640-T2-46_0	AGCTTGTCAAAAGAAA
7B-1877640-T2-46_64	GCTTGATACCAAACAC
7B-1877640-T2-46_128	CTTTAGCCTGTATTTTT
7B-1877640-T2-46_192	GAGAGGTGGACTTGT
7B-1877640-T2-46_256	TGTGTTggggaggggggg
7B-1877640-T2-46_320	GGAATCTTTTGCCTAGT
7B-1877640-T2-46_512	CCTGGATGGCTCCTCG
7B-1877640-T2-46_576	GTGGTCACCGCCTCCG
7B-1877640-T2-46_640	TTACCGTATCCACGCCT
7B-1877640-T2-46_704	AAACGTGGAAGTTAAC
7B-1877640-T2-46_768	GGTGTGCAGCTATGTG
7B-1877640-T2-46_832	TCATTCAGCAATAGCAT
7B-1877640-T2-46_896	ATACTAAGGGAGCATA
7B-1877640-T2-46_960	GCCTCAAGTGTATAGG
7B-1877640-T2-46_1024	CCCGTCGATACCTGCT
7B-1877640-T2-46_1152	GCATATCCCTGTTAGCC
7B-1877640-T2-46_1344	AAGTACTCCCAACTAAT
7B-1877640-T2-46_1408	TTAACGGTAATCAAATG
7B-1877640-T2-46_1580	GACGCATATCCATATAC
6A-1512520-T2-47_130	TTCATCTGGTGGCAGA
6A-1512520-T2-47_195	TATGTCAATACAGGTTC
6A-1512520-T2-47_260	AGAGTGATCAGGTTTG
6A-1512520-T2-47_325	ATTGCGGATAATCTCTC
6A-1512520-T2-47_390	TGCAGCCTCTAAGCTA
6A-1512520-T2-47_455	TGGTGATAGAATCTGAT
6A-1512520-T2-47_520	CCAGCAGTGTCTATTAT
6A-1512520-T2-47_715	ATTTGATAAACAAAGTT
6A-1512520-T2-47_780	GACTCTGAAATATGTCC
6A-1512520-T2-47_845	ACCATGCGGACGAACT
6A-1512520-T2-47_910	CAAGCTTTTTAAACCA
6A-1512520-T2-47_975	ATCGGCCCATATTTCCA
6A-1512520-T2-47_1040	ACATGGGTACATAAAC
6B-1619110-T2-47_256	GTATGTCTGGCAAAAG
6B-1619110-T2-47_320	CCTCGGATGATGTCCA
6B-1619110-T2-47_384	AAGAGACATTCTCTGT
6B-1619110-T2-47_512	CTGTTCGACGTATTCG
6B-1619110-T2-47_576	TCCAGGTCAGGGTTGG
6B-1619110-T2-47_768	GGGAGTCCTATTCGCA
6B-1619110-T2-47_832	TCTGGAACGATTGCCA
6B-1619110-T2-47_1024	TCCTGATCACGAGCCT
6B-1619110-T2-47_1088	TTTCCACGAAAAAAAA
6B-1619110-T2-47_1216	CACGGCACATGGGTAC
6B-1619110-T2-47_1280	GCAGCAGCCACAACTA
1B-0112800-T2-48A_0	GAAAAAGATTTTTTGA
1B-0153990-T2-48B_0	CAAGCACTTGAATTGA
1B-0112800-T2-48A_64	TAGTAATTATGCAATTC
1B-0153990-T2-48B_64	CACCAGATTCTGAAAG
1B-0112800-T2-48A_128	GTCCTTTTTTCAAGCAT
1B-0153990-T2-48B_128	AGATGTCTGTGCTATG
1B-0112800-T2-48A_192	TATTTTAGTAGAAGTAG
1B-0153990-T2-48B_192	GGGTTTATAGAAATTTT
1B-0112800-T2-48A_256	GGAGACATTTACCTTT
1B-0153990-T2-48B_256	TTTGTTCAGGAGTTTCT
1B-0112800-T2-48A_320	TTGGGGAAATTTTTTG
1B-0153990-T2-48B_320	ATATTCAGTTTCCGCCA
1B-0112800-T2-48A_384	TCAGCTATACTGTTCAT
1B-0153990-T2-48B_384	GGGATACTCAGGTTTG
1B-0112800-T2-48A_448	TAGTGAACAATTTATGT
1B-0153990-T2-48B_448	TTAACTTGGTCAGATG
1B-0153990-T2-48B_512	TGATAGCGATCTCATAT
1B-0153990-T2-48B_576	TTCTTCATTGATAGTGA
1B-0112800-T2-48A_640	TGTAAGATAACCACGG
1B-0112800-T2-48A_704	AAAATGATTAATAAAA
1B-0153990-T2-48B_704	GAGCACGTCTCCTCCC
1B-0112800-T2-48A_768	AGCAAAATAAGACAAG
1B-0153990-T2-48B_768	CATTAGCAGCTATACTA
1B-0112800-T2-48A_832	TGCTAAAATAAAAGAA
1B-0153990-T2-48B_832	AAATAATTTCAAAAAAT
1B-0112800-T2-48A_896	TCTAGTTAGCAACCTG
1B-0153990-T2-48B_896	CGATCTAATTAATACTG
1B-0153990-T2-48B_960	AGAGAGTACTGACTAG
1B-0112800-T2-48A_1088	CATGTCTCCCTGAATTC

1B-0112800-T2-48A_1152	CCAACTTCCAAAAGCT
1B-0153990-T2-48B_1152	AGCTTAGCTGCTTTCC
1B-0153990-T2-48B_1216	ACACCCACATATCCGC
1B-0112800-T2-48A_1344	CGCTGTTGCTGCGTTG
1B-0112800-T2-48A_1408	AATATTTGATTTTGAGT
1B-0153990-T2-48B_1408	TCGTGAAGGTAACTATA
1B-0112800-T2-48A_1472	CACGTCTTCCTCACAA
1B-0153990-T2-48B_1472	AAGCACTCAAGCTATAT
1B-0112800-T2-48A_1536	TTTTCACCCCTGTAGAT
1B-0153990-T2-48B_1536	TTTCATTTTAGTTTTTT
1B-0112800-T2-48A_1580	ATTTCACAAATAGGTT
1B-0153990-T2-48B_1580	GAAAAATTGACGGAA
1D-0249790-T2-48_320	TTGCATTGCTAGCTTCA
1D-0249790-T2-48_576	AACGGTACACACACGG
1D-0249790-T2-48_640	TGTGAGCACAGGTGCA
1D-0249790-T2-48_704	CTGTTAACCAATCACAA
1D-0249790-T2-48_768	TTTTTGGGCATACCAC
1D-0249790-T2-48_832	CCAGACAACCAGAATA
1D-0249790-T2-48_896	GCAGTTTTTCAAAATTT
1D-0249790-T2-48_960	ATACAAAAAGTCGCAG
1D-0249790-T2-48_1024	CAAAAGACAATTCATC
1D-0249790-T2-48_1088	TGGTTGAATCTTGAAT
1D-0249790-T2-48_1152	GAGCTACAAAGGAAC
1D-0249790-T2-48_1216	TGGTATTTCTAGTTTCT
1D-0249790-T2-48_1280	AGCTGGTTGGTGCACT
1D-0249790-T2-48_1344	GTACATTTTGATATACT
1D-0249790-T2-48_1408	TAAGAACTCTGGACTT
1D-0249790-T2-48_1472	AGTCCTCATGGTGAGA
1D-0249790-T2-48_1536	GCACACTGCAGAACG
1D-0249790-T2-48_1580	CTTGGTTTGCGTGTGA
1A-0001960-T2-49_0	GAAGAGAGCTTTATTG
1A-0001960-T2-49_64	GAAGTTAATAGCCCGC
1A-0001960-T2-49_128	CCACCTTTTTCTCTGAC
1A-0001960-T2-49_192	GCTTTTTTCTCTTTCTT
1A-0001960-T2-49_576	ATATTGCATAATTGTTT
1A-0001960-T2-49_704	GAGACCGGCGGCCTC
1A-0001960-T2-49_1088	TTTTAAACTATGGCTCC
1A-0001960-T2-49_1344	aaaacattttattaattaaat
1A-0001960-T2-49_1472	ACTCAGCAGCTGCAAA
1B-0101070-T2-49_0	ATCGAAAAGAACAATT
1B-0101070-T2-49_64	TATCGTAAGAGCATGG
1B-0101070-T2-49_128	tacaatagtgattcatatttgt
1B-0101070-T2-49_192	aagttgttgaagcacgtgct
1B-0101070-T2-49_256	caaccaggtaaaaatctaat
1B-0101070-T2-49_640	TGTTTTCAGACTTTTTC
1B-0101070-T2-49_704	GAGCCTCGTCGCCAAA
1B-0101070-T2-49_1024	GCCAATAAACGAGGA
1B-0101070-T2-49_1408	GGGCCTGGGTGAGCC
1B-0101070-T2-49_1472	AGCAGCTGCGAATGCT
1D-0205540-T2-49_0	AGAGCATGGTTAATAG
1D-0205540-T2-49_64	GTGAGTCATATTTGTAT
1D-0205540-T2-49_128	TGAAACACATGCTACA
1D-0205540-T2-49_192	GTAAAAATCTAATATGG
1D-0205540-T2-49_256	TTTTGGAGGTCGAGCT
1D-0205540-T2-49_704	ATTTCAAAATTTTAAGA
1D-0205540-T2-49_960	GATGTATTAACATTTTAT
1D-0205540-T2-49_1024	ATAAAGGAGAAAGAA
1D-0205540-T2-49_1408	TTAAGAAAGACTTTTTT
1D-0205540-T2-49_1472	CAGCTGCGAATGCCAG
1A-0075220-T2-50_0	GCACTTATTCGCAAAAT
1A-0075220-T2-50_79	ACACAATTTCGACTTAG
1A-0075220-T2-50_630	TGTCTTGTGGTGAAAA
1A-0075220-T2-50_1078	AGCACCCAGGAGCTCT
1A-0075220-T2-50_1270	CCTCTGCTCTGTCTTCC
1A-0075220-T2-50_1462	AAACCAAACGAAAATA
1B-0177050-T2-50_0	TGGAACCGCTTTAGAA
1B-0177050-T2-50_128	TTTCTCTTCGGTAGTGA
1B-0177050-T2-50_192	GGATAACATCACATCA
1B-0177050-T2-50_256	CGACATTGGGGAGCA
1B-0177050-T2-50_320	GATTGTTGGCACACAA
1B-0177050-T2-50_512	TGTTGCATCAGCGACG
1B-0177050-T2-50_640	GGTGAAAACATGTGTA
1B-0177050-T2-50_1024	AGTTTTTTCGAATTTTC
1B-0177050-T2-50_1088	AGGAGCTCTGGTTTATT
1B-0177050-T2-50_1280	CTCTGTCATCCGCTTCT
2A-0288560-T2-51_0	TGCCCAGGGAAAAATC
2A-0288560-T2-51_64	ctcctccctccctGGTGCTG
2A-0288560-T2-51_192	AAGCGATAGTGTGCTT
2A-0288560-T2-51_384	CGAGGAGGGCAACGT
2A-0288560-T2-51_448	TCTCACCTCTGGAACAT
2A-0288560-T2-51_512	AACGATGGGCCGCATG
2A-0288560-T2-51_576	TAAGCTTTATTAATGGC
2A-0288560-T2-51_768	CCGCAGCAGACGTACC
2A-0288560-T2-51_896	TCACCTTTGTTCTCATC
2A-0288560-T2-51_960	CCGGGAGCTGTGGTC
2A-0288560-T2-51_1024	ATCCTCTCCAATTATTA
2A-0288560-T2-51_1152	GAGCCTTGCCCATCAG
2A-0288560-T2-51_1216	TGAACGAAACATACATT
2A-0288560-T2-51_1280	CACATCAAGTGCGCAA
2A-0288560-T2-51_1344	ATGTAAAGTGGATGCG
2A-0288560-T2-51_1408	AAGGGGGATGCAGGT
2A-0288560-T2-51_1472	GCGTCAACAATTAACG
2A-0288560-T2-51_1536	TTTGCTGATCCCATCTA
2A-0288560-T2-51_1580	ATATCAGCAAACAAATT
2B-0423210-T2-51_64	tccACGAAGCTGACCG
2B-0423210-T2-51_256	GCGGGAAATACATGTG
2B-0423210-T2-51_384	GTGGTCCAGGGATGG
2B-0423210-T2-51_448	GGAACATTCGGTGTTT
2B-0423210-T2-51_512	GGCCGCATGTGAAGA
2B-0423210-T2-51_576	TATTAATTGCAAACCCC
2B-0423210-T2-51_640	TCAGCAAGAAACCAAG
2B-0423210-T2-51_704	TGGATACACAGACGGC
2B-0423210-T2-51_768	CCTAGTTTTTTTTTTTT
2B-0423210-T2-51_832	AGTAGTTTGTAACATGT
2B-0423210-T2-51_896	CCCAAGGCCCGTCCGT
2B-0423210-T2-51_960	TCTAGTTATTAATAGTA
2B-0423210-T2-51_1024	AGAGCCTAGCTCACCG
2B-0423210-T2-51_1088	AAAAAACAGACTAAAA
2B-0423210-T2-51_1152	GTCAATGCTGAGTTGC
2B-0423210-T2-51_1536	GAAGTTGTCTTTAGGC
2B-0423210-T2-51_1580	GAGGATAACACCAGTT
2D-0540290-T2-51_0	TGCCGAGGGAAAATCA
2D-0540290-T2-51_61	ccctccctccTCGGTGCTG
2D-0540290-T2-51_366	AGTGTTGTGGACGGCT
2D-0540290-T2-51_427	ATGACTCGTGTAGCTT
2D-0540290-T2-51_488	CTTCTTTTCTGCCTGAA
2D-0540290-T2-51_549	GCAGAAATGACCATCT
2D-0540290-T2-51_610	CCACCTTTACTCCACCG
2D-0540290-T2-51_793	CAGACGTACCATCCAA
2D-0540290-T2-51_854	AGTGGCTTTAACATGT
2D-0540290-T2-51_915	GAGTAAACGTGTCTGA
2D-0540290-T2-51_976	TCCCAGGCCCGTCCGT
2D-0540290-T2-51_1037	GTTAACTGATTGGCCTT
2D-0540290-T2-51_1220	TCTGAACAGAAAAATG
2D-0540290-T2-51_1281	TGCAGTCAATGCTGAC
2D-0540290-T2-51_1464	CCAAACACACGCCCAC
2D-0540290-T2-51_1525	TCTCATTAGATGGTGCC
2D-0540290-T2-51_1586	CTACATGCAAACTGGA
2D-0540290-T2-51_1647	TTCTCGCTATCGTTAAC
2D-0540290-T2-51_1708	ACAGGGCATCAACAAA
2D-0540290-T2-51_1769	TAACAGGGCATCAACA
2D-0540290-T2-51_1830	TCATCAACAGGGCATC
2D-0540290-T2-51_1891	TTGATATACGAAACAA
2D-0540290-T2-51_1952	TATATAGGGTTGCAGG
2D-0540290-T2-51_2013	ATCAGCAAACAAATGG
2D-0540290-T2-51_2074	ATGCAAATTACATGCTA
2D-0540290-T2-51_2135	GATAAATTTGTATCAAG
2D-0540290-T2-51_2196	GATGGAGCAAGCACG
2D-0540290-T2-51_2257	AGCAAAATAAATGCAT
2D-0540290-T2-51_2318	TGAACAATATGCTTGA
2D-0540290-T2-51_2379	AACAACATCAGAACAA
2D-0540290-T2-51_2440	ATTTCGTGACATCCCTG
2D-0540290-T2-51_2501	TTGATATTTTCATATTG
2D-0540290-T2-51_2562	ACTCATAGTATGTGGCA
2D-0540290-T2-51_2623	GTAGATAGGCATGAAG
2D-0540290-T2-51_2684	TCACCTCAAAGAATGT
2D-0540290-T2-51_2745	AGCAGCAAGCGCCAA
2D-0540290-T2-51_2806	AGGTGCAGAAGTTTCT

2D-0540290-T2-51_2854	GAATGGTAAAACCAAT
1A-0010710-T2-52-2_320	CGGCTCGAGAGCAGA
1A-0010710-T2-52-2_512	CATGCTGCACAAGGCT
1A-0010710-T2-52-2_576	TGCTTGCGTGTGAATA
1A-0010710-T2-52-2_640	GCTGAGCATCTCCGCG
1A-0010710-T2-52_64	CGCTCTTTCTCCAATTT
1A-0010710-T2-52_128	AAAATTATATGTCCTCC
1A-0010710-T2-52_192	TGGAGAGTCACTGAAA
1A-0010710-T2-52_256	GTCTCTAAAATGTTTAT
1A-0010710-T2-52_320	CCATTGCCTGAAGACG
1A-0010710-T2-52_384	TGTTTGTTATGTGCATA
1A-0010710-T2-52_512	ATATGCCATCTCCATCG
1A-0010710-T2-52_576	AACAACAGTAACTAAT
1A-0010710-T2-52_640	GTTAGCTTTGTTTAACT
1A-0010710-T2-52_704	TTTAAAATATAGATGTC
1A-0010710-T2-52_768	ATAAAATGATGATGCAT
1A-0010710-T2-52_832	TCACACCAAGTAACCT
1A-0010710-T2-52_896	ACAATAGAACTATCTCA
1A-0010710-T2-52_960	CACTTACAGGATTTACT
1A-0010710-T2-52_1024	ATGTTATTTACATTTTTA
1A-0010710-T2-52_1088	CAATAGTAGTGTCCCA
1A-0010710-T2-52_1152	AATACAGCACTAATTAT
1A-0010710-T2-52_1216	TACCTTACGACAGCAA
1A-0010710-T2-52_1280	TAGCTGGCAATATAGCT
1A-0010710-T2-52_1344	TGGAAGCCAGTAGATG
1A-0010710-T2-52_1472	TTTGTGGAAAAGAAGA
1B-0108420-T2-52_0	TTCTGTTGTCTTTGGCG
1B-0108420-T2-52_130	TCACGCGGTGGTTTTG
1B-0108420-T2-52_195	GAGACGAGGGGCCGC
1B-0108420-T2-52_325	TGTCCATGGGGATCGG
1B-0108420-T2-52_390	CCACTGGGCAGCGGC
1B-0108420-T2-52_455	AAGTGCCCTATTTTCAT
1B-0108420-T2-52_520	GGAACATTTTAGGCCA
1B-0108420-T2-52_780	GAAGGTGCTCATAGGA
1D-0221570-T2-52_0	CACCGACCGGAGCGC
1D-0221570-T2-52_63	GCTGGGTGGGGTGGG
1D-0221570-T2-52_252	ACGGAGGACGGGGCA
1D-0221570-T2-52_378	CCTGCCTGGCTCCTCT
1D-0221570-T2-52_504	AGCAGCCTGCTTGCTT
1D-0221570-T2-52_693	GGAGCCGACCGCGCAT
1D-0221570-T2-52_756	CGAGCTGGAATATTCA
1D-0221570-T2-52_882	GCTGGTGGCCTGTAGC
1D-0221570-T2-52_1071	TGCGTCGTCTTCGGTT
1D-0221570-T2-52_1197	GCTGAATGGATCTATCT
1D-0221570-T2-52_1323	GGCTGATGGCGACGG
1D-0221570-T2-52_1386	AGTTGAGGCCGGGCA
1D-0221570-T2-52_1449	TATCATCACATCGCAGA
1D-0221570-T2-52_1575	CAGAGAATAACAGCCA
1D-0221570-T2-52_1764	GGCAAAATGGCGGCC
4A-0093880-T2-53_0	CGCGCGCGCAGGGTGT
4A-0093880-T2-53_64	GATAAACATAATTTTGC
4A-0093880-T2-53_128	GTATGAATTTGAAATAG
7A-1817020-T2-53_128	AGCACACATGGCCCCC
4A-0093880-T2-53_192	CGAATTAATTGCACGT
4A-0093880-T2-53_256	GTTACTTGTTTGATCCA
4A-0093880-T2-53_320	CTAATCCTTAACCATGA
7A-1817020-T2-53_320	CACGACGCCGCTGGA
4A-0093880-T2-53_384	CACGATTTAAATCTGA
7A-1817020-T2-53_384	TACCACTAGTCTCGTAC
4A-0093880-T2-53_448	AAAGAAAAAAAGGCA
4A-0093880-T2-53_512	ACTGACCTTGATTAGAT
4A-0093880-T2-53_576	GGGTGCGTACCTGGTC
4A-0093880-T2-53_640	CGGTCGCCGGCCGGG
4A-0093880-T2-53_704	tcctcctcctcctGGTTGTA
7A-1817020-T2-53_768	CGAGTACAAATAACCC
7A-1817020-T2-53_832	CGCCCGCCCGCCCCAC
4A-0093880-T2-53_896	GACGGGACGGGACCG
7A-1817020-T2-53_896	CTCACGAATGCCCGGC
4A-0093880-T2-53_960	CCGTGTGATGAAAGGC
7A-1817020-T2-53_960	agcagaagaagaagaagca
4A-0093880-T2-53_1024	ttgtttgtttCTGCGTGCGT
7A-1817020-T2-53_1024	CGGCCCAGTTCCCGGC
4A-0093880-T2-53_1088	TGCCGCGGAGTGGCT
7A-1817020-T2-53_1088	GTTGGTCTAGTAGTAG
7A-1817020-T2-53_1152	TTTTTACTTAAAGATCG
7A-1817020-T2-53_1216	TGTAAAAATTAAAAAA
4A-0093880-T2-53_1280	CCTTCTCGGTGCCGGA
7A-1817020-T2-53_1280	CGATCTTAGATCTGCG
4A-0093880-T2-53_1344	TGTGTGCTCTCGCGCT
7A-1817020-T2-53_1344	ACAGAGGCAGCATAAT
4A-0093880-T2-53_1408	GCGCGAGCTTGCTTGC
7A-1817020-T2-53_1408	AGGTCACGCACCAAG
7A-1817020-T2-53_1472	GAACAAATTCCATTTCT
4A-0093880-T2-53_1536	AACTGGCGCGTGAGG
7A-1817020-T2-53_1536	ACCAGTGCGGAGACA
4A-0093880-T2-53_1580	ACGTGCCCTTGCTTTT
7D-2037380-T2-53_0	AATCAATGAAGCAAAA
7D-2037380-T2-53_64	GTGCTGCGAGGAAGC
7D-2037380-T2-53_128	AGGCGAAAGCGAGAG
7D-2037380-T2-53_320	GCAAAGGCGCATCGG
7D-2037380-T2-53_384	CACGGTTCCGCCCCCG
7D-2037380-T2-53_448	ACTAGGTAGAAACAAA
7D-2037380-T2-53_640	GGGATCCAGCCACCG
7D-2037380-T2-53_704	GCCACGCACCGTTCGT
7D-2037380-T2-53_768	AGGAGCCTCTGCCAGT
7D-2037380-T2-53_832	GCGACTGTGAGTACGC
7D-2037380-T2-53_896	GGTACGCACGCACGCA
7D-2037380-T2-53_960	AGTCCAGTCTGATAAT
7D-2037380-T2-53_1024	TTGGCGCCCTGTTATAA
7D-2037380-T2-53_1088	AGTTCAGAGGATCTTG
7D-2037380-T2-53_1216	GGATTAAATCCCCTTCA
7D-2037380-T2-53_1280	CAGATCCAATGGATTC
7D-2037380-T2-53_1344	AACGCACAACCATTTCT
7D-2037380-T2-53_1408	TACGCACCAAGGCTTT
7D-2037380-T2-53_1472	AACAAAACAGCATCTC
7D-2037380-T2-53_1536	GGATGCAGACTCATGC
7D-2037380-T2-53_1580	AAATGAAATTTAAAAT
2A-0273760-T2-54_320	CAGTTCCCCACTGCCA
2A-0273760-T2-54_384	AGGTACGCACTGCGCT
2A-0273760-T2-54_448	CTTGGTTCTGCCATTG
2A-0273760-T2-54_512	ACCATGGAGTTTCCCT
2A-0273760-T2-54_576	CATTCTCACCCGTCTTT
2A-0273760-T2-54_640	CTCCAAAATGTGCACC
2A-0273760-T2-54_704	CCACTAGTGACCAGTC
2A-0273760-T2-54_768	GCAGTACAAGTTTTTG
2A-0273760-T2-54_832	GGATGTTGTGATGTACT
2A-0273760-T2-54_896	ACTGCTATTTGATTCCG
2A-0273760-T2-54_960	TTGTACCTGACTGAGG
2A-0273760-T2-54_1024	TTACATGGTTTGCGCA
2A-0273760-T2-54_1088	GGTTACTGCCTATGTG
2A-0273760-T2-54_1152	AACAGACTAGTGTCCT
2A-0273760-T2-54_1216	GACGGCTGGTTTGGGT
2A-0273760-T2-54_1280	ATTAAAAGCCTGTGCA
2A-0273760-T2-54_1344	AAAGCATGTACAATTAT
2A-0273760-T2-54_1408	TGCAATTGTAGCACTA
2A-0273760-T2-54_1472	CAAGCAGTTAGCACGT
2A-0273760-T2-54_1536	ATTTGTGGCATTATAAA
2A-0273760-T2-54_1580	TTTACATTCAGAATATA
2B-0429850-T2-54_384	TGCACGATTCTCCCTC
2B-0429850-T2-54_448	GGGTGTCCAAGTATTC
2B-0429850-T2-54_512	CTGTGCAGTAGCCAGT
2B-0429850-T2-54_576	GAAACCCTTGGCATGT
2B-0429850-T2-54_640	CACCTTAGTTTCTGATA
2B-0429850-T2-54_704	TGTACCATTGAGGGTG
2B-0429850-T2-54_768	TAGTACACGCTTTCCTT
2B-0429850-T2-54_832	GATGTACTGCTCTTCAC

2B-0429850-T2-54_896	CGGGGTGTTGGTGTTA
2B-0429850-T2-54_960	GCTAGGATACTGACAT
2B-0429850-T2-54_1024	TCATTTATATATCTATAT
2B-0429850-T2-54_1216	GTTCCTGGACAGTTGA
2B-0429850-T2-54_1280	GCATTGGTTTCTTTCG
2B-0429850-T2-54_1344	CTTTTTGCCTTTTAACC
2B-0429850-T2-54_1408	AATTGGTCTGTTCCTTT
2B-0429850-T2-54_1472	GGGTATGCAAGCAGTT
2B-0429850-T2-54_1536	CATATATATTTGTGGCA
2D-0502660-T2-54_183	CGCCGAACACTTCTGC
2D-0502660-T2-54_244	AGCCACTACGCCTGCT
2D-0502660-T2-54_305	CTATTTTCTCCCGCTCC
2D-0502660-T2-54_366	GGGCCGTTTGATTTTG
2D-0502660-T2-54_427	CCTGTGTTCCCAACCA
2D-0502660-T2-54_488	TGTTTCCTTACTGCATT
2D-0502660-T2-54_549	TTCACCGATCCGACAT
2D-0502660-T2-54_610	GCACCGTCTTCGTACG
2D-0502660-T2-54_671	ATGGGACGGAGGGAG
2D-0502660-T2-54_732	AGTATTAATGTTCTATT
2D-0502660-T2-54_793	GTTTTTTTTCATCTCA
2D-0502660-T2-54_854	GTGTTGTGATGTACTG
2D-0502660-T2-54_915	CACTGCTATTTGATTTT
2D-0502660-T2-54_976	AACATTGTACCTCAGG
2D-0502660-T2-54_1037	ACATTGCATGGGTTGC
2D-0502660-T2-54_1098	AATATGGTTGCTCCCTA
2D-0502660-T2-54_1220	ATACCAACGGCTGGTT
2D-0502660-T2-54_1281	ATAAGAAAAATAAAAG
2D-0502660-T2-54_1342	TTCGAAACAGCATGCA
2D-0502660-T2-54_1403	TTTTAACCTTGCAGTTG
2D-0502660-T2-54_1464	TCTGTTCAGGGTATGC
2D-0502660-T2-54_1525	TGAGAAAATGACATAT
2D-0502660-T2-54_1708	CATTCCTACTTATTGTT
2D-0502660-T2-54_1769	ATCCAATTCCAGCAAT
2D-0502660-T2-54_1952	TGCAAAACTGGAGGC
2D-0502660-T2-54_2011	TATTCCTTGTCACGTAC
7A-1766630-T2-55_0	CGTCTTCCACGGCACG
7A-1766630-T2-55_256	AGCCAGTGAAAAGGT
7A-1766630-T2-55_320	AGTTTGTCATGTTTTGA
7A-1766630-T2-55_384	AAATAGAAAATCTCCT
7A-1766630-T2-55_448	AGTGATTTTGCCTTTTC
7A-1766630-T2-55_832	CCTCTCCCACCTCGCC
7A-1766630-T2-55_896	CCTCGTAATACAAATG
7A-1766630-T2-55_960	GTCCCACAATATAAGAT
7A-1766630-T2-55_1024	GAGCTCGGGACCGAA
7A-1766630-T2-55_1088	CACTAGTGTCTGAAAA
7A-1766630-T2-55_1152	TAAGGCTAGGCTGCAC
7A-1766630-T2-55_1216	CTTCAATCTTTTCACTC
7A-1766630-T2-55_1280	AGATGTTTATTTATGGT
7A-1766630-T2-55_1344	AGGTTTGCTTTTAATAA
7A-1766630-T2-55_1408	AACTGTTTGTTTTCCCT
7A-1766630-T2-55_1536	TGTCCATCCTGTCTCCG
7A-1766630-T2-55_1580	GGATGGCAAAAAAAA
7B-1913520-T2-55_0	GATGATGTGCCCATATT
2B-0440100-T2-55-1_64	AAATTGTTGGATATATC
2B-0440100-T2-55-2_64	CGATCTGAAGGTAGTT
2B-0440100-T2-55-2_128	TTCGCTTGAAATACCTG
2B-0440100-T2-55-2_192	ACTGAATTCCTATAATT
2B-0440100-T2-55-2_256	TTAAGTTTCAGTTGTAT
2B-0440100-T2-55-1_320	CTGGAAAGATTAAATC
2B-0440100-T2-55-2_320	TTTAGTTTCAAGTGAA
2B-0440100-T2-55-2_384	TGAATTTCAGTAGTGG
2B-0440100-T2-55-1_448	GGAACCATTGTTCCCA
2B-0440100-T2-55-2_448	TATATACTAAAGATTTG
2B-0440100-T2-55-1_512	CCAATGGGTCATGGAA
2B-0440100-T2-55-2_512	GGAATGGTTCTTATTAT
2B-0440100-T2-55-1_576	GACTAAATTTCCAACC
2B-0440100-T2-55-2_576	CATATGCGAGGCCTAC
2B-0440100-T2-55-1_640	AGATTATGCAGGAATT
2B-0440100-T2-55-2_640	TTTGTCTTGCACATTGT
2B-0440100-T2-55-2_704	GAAGATAGTATTTGGT
2B-0440100-T2-55-1_832	TGAAGGTCCAGACTTC
2B-0440100-T2-55-1_896	TTGCATGGTGCCGAGT
2B-0440100-T2-55-2_896	GATTCGCTTCTATTGTG
2B-0440100-T2-55-1_960	GGGTCTGGTGTGTGG
2B-0440100-T2-55-2_960	TTGATGTATCACGAAAT
2B-0440100-T2-55-1_1024	AGTCGTCGATTCGTCT
2B-0440100-T2-55-2_1024	TTGGTAACCGAACAAA
2B-0440100-T2-55-1_1088	CGGCGGATGGACAGA
2B-0440100-T2-55-2_1088	TGTAATTGGGTAGACT
2B-0440100-T2-55-2_1152	TGCCAATTGAACAACA
2B-0440100-T2-55-2_1216	GATACGCATACTCATGC
2B-0440100-T2-55-1_1280	ttgtgtgttgtTGAGGTTT
2B-0440100-T2-55-2_1280	TTGCTTTTGTAAGGAT
2B-0440100-T2-55-1_1472	CTTGTGTATGAAAAGG
2B-0440100-T2-55-2_1472	GACTAAATAACTATGTA
2B-0440100-T2-55-1_1536	CTATTTTCTATCCATTTC
2B-0440100-T2-55-2_1536	CTGAAGGTATCACTTA
2B-0440100-T2-55-1_1580	CATGAAAGTCAGATGA
2B-0440100-T2-55-2_1580	TGTTCTTCTAACCATCT
7B-1913520-T2-55_64	GGTGTCCTACAGAGCG
7B-1913520-T2-55_128	GCAAGTTTGTATCAAT
7B-1913520-T2-55_192	AGTTGATCCAGTTTTCA
7B-1913520-T2-55_256	TTGTGTTCTGCAGGTC
7B-1913520-T2-55_320	CTCCAGGGCGGCGATT
7B-1913520-T2-55_384	CTTTCCTATGATGCTTC
7B-1913520-T2-55_448	CAATGATGGAAATGAG
7B-1913520-T2-55_512	GCACCCCCATGACTTG
7B-1913520-T2-55_576	CTTAATATATGGCATTT
7B-1913520-T2-55_640	TTTATTAACTGAATTAT
7B-1913520-T2-55_704	TTTAAAAGAAATACAT
7B-1913520-T2-55_768	CACACTAATCATCCCTC
7B-1913520-T2-55_896	GCTCCGATAGCGAGTT
7B-1913520-T2-55_960	ATATGTGTTCCTTTGTC
7B-1913520-T2-55_1024	TACTGGCTCCATCTTGG
7B-1913520-T2-55_1152	CATCGTCAATTTGAGT
7B-1913520-T2-55_1216	AGTGCCTGTGATGTGC
7B-1913520-T2-55_1280	GGGACATCCCCCAGTC
7B-1913520-T2-55_1344	ATTTTCTATTTTTGGAG
7B-1913520-T2-55_1408	GACGAAGGCTTGGGA
2B-0440100-T2-55-2_0	CGACGGTCCCGTTCAG
7D-1976400-T2-55_192	TCGGGGAGTGCCCGT
7D-1976400-T2-55_256	GCCTCTCTCCCAGCTT
7D-1976400-T2-55_320	GAGGTTTCTGAAGAA
7D-1976400-T2-55_384	TGCATAGTGACATTCC
7D-1976400-T2-55_448	GAAAATACGATTTGAA
7D-1976400-T2-55_512	CCACCCGAAAAAGTG
7D-1976400-T2-55_576	ATATGTATTGTTTTTTT
7D-1976400-T2-55_640	ATACAACACGCGCAAG
7D-1976400-T2-55_704	CTGAACATCCCTCCAA
7D-1976400-T2-55_768	GGCCCGTCGGTTGCG
7D-1976400-T2-55_832	TCTATCGTGGAACCGC
7D-1976400-T2-55_896	AGACCACATCCTAGTT
7D-1976400-T2-55_960	CCACCACCGGCTTACC
7D-1976400-T2-55_1024	ACATGGATCCCTCGAC
7D-1976400-T2-55_1088	ATTCCAACTGCAGACG
7D-1976400-T2-55_1216	GTGGCCTCGCCGACAG
7D-1976400-T2-55_1280	GAAAGGATACACTCAG
7D-1976400-T2-55_1344	ATCTGTATTGTTAGATA
7D-1976400-T2-55_1408	TTTTTGTTTTATCTTTCT
7D-1976400-T2-55_1472	TACTTTCATTTTGTTTT
7D-1976400-T2-55_1536	AGCACCCTTATAAAATT
7D-1976400-T2-55_1580	CCCAACTCTTCTTACTC

										2A-0289500-T2-56_0	AAACCACAATGATATGT
										2A-0289500-T2-56_63	AAAATAATATCATGGGT
										2A-0289500-T2-56_126	TGCCATCGTACATTAAA
										2A-0289500-T2-56_189	TCAACATCAAGTAGCA
										2A-0289500-T2-56_252	ACAATGGATGCATTTT
										2A-0289500-T2-56_315	GAAAGGGCTCATTCGG
										2A-0289500-T2-56_378	AATGCCGTCTCCACTC
										2A-0289500-T2-56_441	AAGGAATCTAGTACCT
										2A-0289500-T2-56_504	GGGTCAAAAATCTTAT
										2A-0289500-T2-56_567	AATTATAAAGGCATTTT
										2A-0289500-T2-56_630	TTCATAAAATTTTAATA
										2A-0289500-T2-56_693	ATTTCACCATTTTTAGA
										2A-0289500-T2-56_756	GTAAAAACTAGTGCCG
										2A-0289500-T2-56_819	TGACATTCTGCTTTATT
										2A-0289500-T2-56_882	GGAGAACTTGTGGACT
										2A-0289500-T2-56_945	GATTTAGTGCCAAGGT
										2A-0289500-T2-56_1008	GAGGTGGTAGCAAAG
										2A-0289500-T2-56_1071	GGATGACACTGAAAGT
										2A-0289500-T2-56_1197	CATGCGTGCAGATTGG
										2A-0289500-T2-56_1260	AATAGTACTTTTTTTGT
										2A-0289500-T2-56_1827	GGAAGAAGGACTCCT
										2A-0289500-T2-56_1953	TCAACGAGAGAGATAG
										2A-0289500-T2-56_2016	ATCACGGCTTTGATCAT
										2A-0289500-T2-56_2205	CCGGCGCAACCTGAAG
										2A-0289500-T2-56_2394	TGGGCCACTGAAAACT
										2A-0289500-T2-56_2423	ATTCTAGCAAGAACTT
										2B-0425980-T2-56_315	GAGGACGGGATCGCA
										2B-0425980-T2-56_378	TAGTACCCTCGTTTATA
										2B-0425980-T2-56_504	TGCGGGCCAGCTATGG
										2B-0425980-T2-56_567	GTAGGGTACATTATGTA
										2B-0425980-T2-56_630	CATCCTCTTCTCGACTC
										2B-0425980-T2-56_882	ACATGTCATGGTGATTT
										2B-0425980-T2-56_945	TGCTTCCATTAAAAAAA
										2B-0425980-T2-56_1134	GGGCTCCTGCCTCAGC
										2B-0425980-T2-56_1197	CCTGTTTGGGTGGTTG
										2B-0425980-T2-56_1323	TAATTTTGGGTCGGTT
										2B-0425980-T2-56_1386	TACAGACTTGTTCAATG
										2B-0425980-T2-56_1446	AGAATGAGTAATTCTC
										2D-0515330-T2-56_320	GCGTGCGGGAGGACG
										2D-0515330-T2-56_384	CGGCCTCTAGTACCCT
										2D-0515330-T2-56_512	ATGCGGGCCAGCTATG
										2D-0515330-T2-56_576	AATTGTAGGGTACATTA
										2D-0515330-T2-56_704	ATGGACTCGTCGGTGC
										2D-0515330-T2-56_768	GTTTGAAAATGCATGT
										2D-0515330-T2-56_832	CCAGAGGGGAAAAGG
										2D-0515330-T2-56_1024	GTTTTGGTGGTCTCGC
										2D-0515330-T2-56_1088	GCCTTTTGTAAAGCAG
										2D-0515330-T2-56_1152	CGACCCCACCGTCCGA
										2D-0515330-T2-56_1216	GCTGCAAAACGGGCAT
										2D-0515330-T2-56_1280	AGTCATGCTTATTTTAA
										2D-0515330-T2-56_1472	AGAAACAAACGAGAA
										2D-0515330-T2-56_1536	TCCCCTGTGACTTGATA
										2D-0515330-T2-56_1580	CGAAAGAAAAAAAAA
										2A-0314520-T2-57_192	tgatcatgcataaacataatt
										2A-0314520-T2-57_256	AAAACACGGGTGATTT
										2A-0314520-T2-57_448	TTTATTTAGTAATTATAT
										2A-0314520-T2-57_512	TTATCTTTGAGTTAGAA
										2A-0314520-T2-57_576	TGCGTGTGATTCATTAA
										2A-0314520-T2-57_640	ACATAGGGATAACCCA
										2A-0314520-T2-57_704	ACCAGCCTAAACTAGA
										2A-0314520-T2-57_768	TAAGACTACAGTCGAC
										2A-0314520-T2-57_896	GGGCTGGCTGCCACC
										2A-0314520-T2-57_1024	ACAGGGGAGGAGCGA
										2A-0314520-T2-57_1088	TTATTTTCAATATTCAA
										2A-0314520-T2-57_1216	ACGCGCTCGCCAACAA
										2A-0314520-T2-57_1536	CCCGTGCTAGTAGTATA
										2B-0440100-T2-57A_64	CGGCGGATGGACAGA
										2B-0440100-T2-57A_320	TGAAGGTCCAGACTTC
										2B-0440100-T2-57B_378	atttgaccctttttccactagt
										2B-0440100-T2-57B_441	CAAGACCAACAGATGA
										2B-0440100-T2-57A_448	TTGCATGGTGCCGAGT
										2B-0440100-T2-57B_504	GCAAGCTTTGTAGCCT
										2B-0440100-T2-57A_512	GGGTCTGGTGTGTGGA
										2B-0440100-T2-57B_567	TTTGCTCAAAAAAAAA
										2B-0440100-T2-57A_576	AGTCGTCGATTCGTCT
										2B-0440100-T2-57A_640	ttgtgtgttgtTGAGGTTT
										2B-0440100-T2-57A_832	CTTGTGTATGAAAAGG
										2B-0440100-T2-57A_896	CTATTTTCTATCCATTTC
										2B-0440100-T2-57A_960	CATGAAAGTCAGATGA
										2B-0440100-T2-57A_1024	AAATTGTTGGATATATC
										2B-0440100-T2-57A_1088	CTGGAAAGATTAAATC
										2B-0440100-T2-57A_1280	GGAACCATTGTTCCCA
										2B-0440100-T2-57B_1311	CCGTGCCTCCTCATTTC
										2B-0440100-T2-57A_1472	CCAATGGGTCATGGAA
										2B-0440100-T2-57B_1527	CTCTTCGGCCGCAACA
										2B-0440100-T2-57A_1536	GACTAAATTTCCAACC
										2B-0440100-T2-57A_1580	AGATTATGCAGGAATT
										2D-0512220-T2-57_128	CTTTGATGCAAGACAA
										2D-0512220-T2-57_320	AATCCACGACATTTGA
										2D-0512220-T2-57_448	CGAATTAATTGAAATCA
										2D-0512220-T2-57_512	TTATTTGAACTATTGTC
										2D-0512220-T2-57_576	TTGATTGTTTTCAAAC
										2D-0512220-T2-57_640	GCACAACCATTACAAA
										2D-0512220-T2-57_704	ACAATTAATAAACCAA
										2D-0512220-T2-57_768	AGGACAACATTTTCAG
										2D-0512220-T2-57_896	GCCACAAATCCACCAC
										2D-0512220-T2-57_960	GGCATAAACCAGAGCA
										2D-0512220-T2-57_1024	AACAGGGGAGGAGTG
										2D-0512220-T2-57_1088	TGATTATTTTCAATATT
										2D-0512220-T2-57_1216	TAGCCTTGCACGGGCT
										2D-0512220-T2-57_1536	CTCCGTGCTAGCAGTAT
										7A-1824860-T2-58_0	GCAATTATTTTCATGGT
										7A-1824860-T2-58_64	TGATGTGGATGGACTA
										7A-1824860-T2-58_192	GACATTGAAAGATATTA
										7A-1824860-T2-58_896	ATCTCCGAGAGAAAGA
										7A-1824860-T2-58_960	GAGGATGGTGACCCCA
										7A-1824860-T2-58_1024	TAAAACGTTTTTTTTAC
										7B-1931390-T2-58_320	GGGCGGGTCCGCTGG
										7B-1931390-T2-58_384	TAGAACTGGGAAGAA
										7B-1931390-T2-58_448	TGCCAAAAAATCGTTT
										7B-1931390-T2-58_512	ATATGTTGCTCACAGCT
										7B-1931390-T2-58_576	TGCCTCTAAGTGCTGC
										7B-1931390-T2-58_640	ATTCACGCTGCGGCGG
										7B-1931390-T2-58_704	CCAGATCCACGCGTCT
										7B-1931390-T2-58_768	ATCGACTGCCACCGCA
										7B-1931390-T2-58_832	CGTTGGAACCATGAAC
										7B-1931390-T2-58_896	CCATTCAGCCGCCACC
										7B-1931390-T2-58_960	ATCGGCTCGTGCGACC
										7B-1931390-T2-58_1024	CGTCGCGCCGCCGCCC
										7B-1931390-T2-58_1088	CTACACCGTCAAAATT
										7B-1931390-T2-58_1152	ATCGCCACCCATGGGT
										7B-1931390-T2-58_1216	TCCCCGATGTTCCGAC
										7B-1931390-T2-58_1280	AGGAATATCGTCTAGT
										7B-1931390-T2-58_1344	GTCGAGGCCAATCCGC
										7B-1931390-T2-58_1408	ACAAGAATGAGAGGA
										7B-1931390-T2-58_1472	TTGGACGACGGCATTG
										7B-1931390-T2-58_1536	TTACACCATTGTTTAAA
										7B-1931390-T2-58_1580	TTTTATCACTAAGGACT
										7D-2040790-T2-58_1152	AATCGAGGAGGCCGG
										7D-2040790-T2-58_1216	CCGCCATCTCACACCG
										1A-0053840-T2-59_64	CTGTGCGGGTTTACTT
										1A-0053840-T2-59_192	ATCGGACCGCCGTTGA
										1A-0053840-T2-59_320	TTGCGTGCGAGCAAGA
										1A-0053840-T2-59_384	CGTATGAAAAAGGTTG
										1A-0053840-T2-59_448	ACTAGGAGGTTTTGAC
										1A-0053840-T2-59_512	CAACTTCTACCTCACGT
										1A-0053840-T2-59_640	CCTCCCTCGCGCCGGC
										1A-0053840-T2-59_704	GACCCCTCCAGCCTGT
										1A-0053840-T2-59_768	CCGAATCGTTGGGCTC
										1A-0053840-T2-59_960	TTTGCTCGCCTTCGTCT
										1A-0053840-T2-59_1024	CAAGTGCTTTAGGGAA
										1A-0053840-T2-59_1088	GGGCTTGAAGGGCAC
										1A-0053840-T2-59_1280	GACACCATACCTGCGG
										1A-0053840-T2-59_1472	GCGCAATTGGATATCC
										1B-0112170-T2-59_128	GGTGGGGGCTggaaga
										1B-0112170-T2-59_192	ggcACGGGATCGGAAC
										1B-0112170-T2-59_256	CGCTCCCACACCGCCG
										1B-0112170-T2-59_320	ACTTGCGTGCTTTTGT
										1B-0112170-T2-59_1024	AGTGACATGTGAGCAA
										1B-0112170-T2-59_1088	CATCGTATGAAAAAGG
										1B-0112170-T2-59_1152	GTACAACAAACAGTAG
										1B-0112170-T2-59_1408	AAGGGTACGCGCACG
										1B-0112170-T2-59_1536	TTATATTTCTTTACAGA
										1B-0112170-T2-59_1580	TGAAGCTTTTGTAGAA
										1D-0203340-T2-59_192	gatcgggacggACGGCTC
										1D-0203340-T2-59_320	ACTGCTGCTGTGAACA

											1D-0203340-T2-59_384	CGATGAGTTTGTGCAC
											5A-1227100-T2-60_320	GTGGTGTCGTCTCGTC
											5A-1227100-T2-60_384	GGTCGAAATCACGGA
											5B-1334970-T2-60_0	TGCGCTTTCCCTTTCCT
											5B-1334970-T2-60_62	TGGTACAGAACGAGG
											5B-1334970-T2-60_124	CTTAGCGTGAGGTTTT
											5B-1334970-T2-60_186	ACTGGTAGCTAAATGG
											5B-1334970-T2-60_310	CTCGCCGTAGTACTCC
											5B-1334970-T2-60_496	GCCGCTACACTGCACG
											5B-1334970-T2-60_992	cgggggatgggaagtggac
											5B-1334970-T2-60_1054	ATGTGCTTTCCATATAC
											5B-1334970-T2-60_1116	GGACAGAACCTTGCTT
											5B-1334970-T2-60_1178	AACAAAATCTTCTCTAA
											5B-1334970-T2-60_1240	ATTAGACTCCCTAAATA
											5B-1334970-T2-60_1302	AGCAATTTTATTGAGT
											5B-1334970-T2-60_1426	ACTCCTACAATCACGTA
											5B-1334970-T2-60_1488	TAGCGCCTACTCAATT
											5B-1334970-T2-60_1674	CGTTTGTAGTTCAATT
											5D-1467800-T2-60_320	GTCATGTGCTTTCCATA
											5D-1467800-T2-60_384	AACTCACGGACAGAA
											5D-1467800-T2-60_448	AAAGCAAACCAAATCT
											5D-1467800-T2-60_512	ATTAGACTCACTAAATA
											5D-1467800-T2-60_704	CTCCACCTATTCAGGG
											5D-1467800-T2-60_896	GAGAAGAACGAAGAG
											5D-1467800-T2-60_960	TGAAGACCATGATCCA
											5D-1467800-T2-60_1024	GTCTCTTACGTTGACA
											5D-1467800-T2-60_1088	TCAACATCAGAACCAC
											5D-1467800-T2-60_1152	CTTGTATCCTCTAAATT
											5D-1467800-T2-60_1216	AAAATGAAGTAATTAC
											5D-1467800-T2-60_1280	AAAAAACGTCCTAAAA
											5D-1467800-T2-60_1344	AGAACATAGTAAATTA
											5D-1467800-T2-60_1408	AGTAAGTACACAGAAA
											5D-1467800-T2-60_1472	ACAAAATGCTTGAAAT
											5D-1467800-T2-60_1536	CTAGAGAAATACAGAG
											5D-1467800-T2-60_1580	TTGCACTGTAAGTACG
											6A-1573650-T2-61_0	TTCAAACAGGACTTGC
											6A-1573650-T2-61_576	GTGAGGGAGTGAAGG
											6A-1573650-T2-61_640	GAGGTATGCGTATAGC
											6A-1573650-T2-61_704	GGTCGGCTCAAAGAG
											6A-1573650-T2-61_768	AATTTTAATGCATAGAA
											6A-1573650-T2-61_832	GGTTCATATAGTTTTCA
											6A-1573650-T2-61_896	GTTTTCATCTAGCTTAG
											6A-1573650-T2-61_1280	TGGGCTGGATCGGCG
											6A-1573650-T2-61_1344	TATTGGATCTTGGTCG
											6A-1573650-T2-61_1408	CAGGCGATTGACGTGT
											6A-1573650-T2-61_1536	GTGTGTTTTGTGGCCT
											6A-1573650-T2-61_1580	TGATATATATGAATTGA
											6B-1646110-T2-61_0	CTGATTCAACTCGAAC
											6B-1646110-T2-61_192	TATGGTGATGGAATTA
											6B-1646110-T2-61_256	TAAACACTACTGAAAA
											6B-1646110-T2-61_320	GAAAACTGAAAACGT
											6B-1646110-T2-61_384	TATGAACCAGTACTATT
											6B-1646110-T2-61_448	TTAAAATTAACATGCTT
											6B-1646110-T2-61_512	AGCCGACCCCTAGTCA
											6B-1646110-T2-61_1088	GCCGGGTCACGCTGG
											6B-1646110-T2-61_1152	TATCAAGATGTAATTAA

Trait&gene no.	Gene	Homoeologues	IWGSC gene ID (refseq1.1)	WGIN Promotome ID	Orientation	ATG	Target Sequence	MYbaits	MYbaits coverage of target (%)
T3-1	Sucrose synthase	BD, B	TraesCS6B02G466600	6B-1509400B-T3-1	forward	716,618,145	GCGACAAGATGGGGAGCCGGGAGGAGATGCGGTGGCGCAGTTCCCCT	21	79.5
T3-1	Sucrose synthase		TraesCS6D02G403800	6D-1709240-T3-1	forward	471,917,775	TTTCTCGAAAAAAAAAAGTTCATTGTTGCTGTCGTGGGCCATGAGGCAC	18	77.2
T3-1	Sucrose synthase		TraesCS5B02G246400	U-1509400A-T3-1	reverse	74,821,551	GGCTGAGCTGAGGTGGAGGTGGTTTGGCTTGGTGGTGGTGTTATGTGA	15	65.9
T3-10	Fructokinase	ABB	TraesCS5A02G484000	5A-1219370-T3-10	reverse	655,189,292	GTGTCCGAACTCGTCGTCGCCAAACTGAAAAGAAAAACATCATCATCCG	10	50.4
T3-10	Fructokinase		TraesCS5B02G497400	5B-1363130-T3-10	reverse	664,417,472	CGGAACTGGTGCGGAAGGAATAGATCAAACTCAGGAGGATAGGTGCTC	7	25.9
T3-10	Fructokinase		TraesCS5B02G497500	5B-1366900-T3-10	reverse	664,475,891	CGGAACTGGTGCGGAAGGAATAGATCAAACTCAGGAGGATAGGTGCTC	19	83.1
T3-11	Fructokinase	ABD	TraesCS7A02G319000	7A-1775330-T3-11	reverse	459,211,266	TGGAAGCTTGGTCTAAACGCACGAAACGGAAACGCGAACGCGACGGA	19	83.1
T3-11	Fructokinase		TraesCS7B02G219800	7B-1855980-T3-11	reverse	411,059,957	GTGGCCGAACTCGTCGTCGCCAAACTGCAAAACAACATCGCCGTTCGAT	21	90.6
T3-11	Fructokinase		TraesCS7D02G315500	7D-2002120-T3-11	reverse	401,550,890	GTGGCCGAACTCGTCATCGCCAAACTGCAAAAAAACGTCACCGTTCGAT	20	84.7
T3-12	PGI	ABD	TraesCS1A02G040600	1A-0068020-T2-3	reverse	22,301,216	GGCCGGGCGGACCTGGCGGAATTGAGGCGGAGGATAAGCTGCCTTTA	17	80
T3-12	PGI		TraesCS1B02G053600	1B-0166630-T2-3	reverse	33,990,120	GGCCGATGCGGCGGAGGGCCGGGCGGACCTGGCGGAATTGAGGCGG	16	69.6
T3-12	PGI		TraesCS1D02G041200	1D-0260080-T2-3	forward	20,043,946	GTCCTGTGTGTCAGGCACAACCTAGGAACAGAATGTTTTGCCACTTAAA	14	62.1
T3-13	PGI	ABD	TraesCS5A02G247200	5A-1237760-T2-2	forward	461,487,242	TACTGAAGCGTTTAACACATCACCACAGTGCAGAGGAGGCAATCGCTTT	15	65.9
T3-13	PGI		TraesCS5B02G244300	5B-1362930-T2-2	forward	425,878,428	CTAGTAGTCTATCATAGTCGGAAGGGGGCAGTCGCTTTCCTAAATGGCC	11	46.2
T3-13	PGI		TraesCS5D02G253700	5D-1440810-T2-2	forward	360,539,840	TGTTTGTTATTTTGTCCCTCGGCCAGCCAGGCTGTTGGTCGCCGCTGCA	22	94.8
T3-14	UGPase	ABD	TraesCS5A02G353700	5A-1211020-T3-14-2	forward	556,679,270	GGAAACCGAAAAGCCTGGCACGTACATGCGGGGAAGTTTTGTAGAACC	21	87.3
T3-14	UGPase		TraesCS5B02G356300	5B-1316790-T3-14-1	forward	536,046,024	CCTATAGGAATTTCAACATCCACTATAACCTCTTTTTTACACTGATTCGCG	20	80.2
T3-14	UGPase		TraesCS5D02G361000	5D-1397780-T3-14	forward	440,642,438	CGACGCACGTTTTAAGTATAACCAGAACCTACCCTCAAAAGAAGTATAAC	17	70.5
T3-15	UGPase	AB	TraesCS6A02G050100	6A-1571840-T3-15	reverse	25,641,677	TGTCGTTGCGAGAGAGAGAGAGAGAGAGAGCTTGGCGCGTGTGCGGGGA	20	86.8
T3-15	UGPase	no D	TraesCS6B02G087600LC	6B-1637150-T3-15	reverse	44,110,480	TGTCGATGCGAGAGAGAGAGAGCGCTTGGCTTGGCGCGTGTGCGGGG	6	29.2
T3-16	APL3/AGPL1	ABD	TraesCS1A02G419600	1A-0022300-T2-37	reverse	576,374,703	TCGCAGTCCACCTGCAACTGCAACAAGATGAGAAGTTCAGAACGATCAC	17	77.2
T3-16	APL3/AGPL1		TraesCS1B02G449700	1B-0119450-T2-37	reverse	668,131,494	GGGAATGTCGATGAGCCTGTAACATCCTCCAATAGGAACCTGAGCAATG	11	44.2
T3-16	APL3/AGPL1		TraesCS1D02G427400	1D-0227010-T2-37	reverse	479,892,869	TCGCAGTCCACCTGCAACTGCAACAAGATGAGAAGTTGAGAACGATCA	12	57.9
T3-17	PGM	ABD	TraesCS7A02G287400	7A-1821750-T2-5	forward	342,610,534	GTACGCCATTTTTGGGCGTGTTCTCGAAAAAATGATCCATGTGTGCTTTC	14	65.4
T3-17	PGM		TraesCS7B02G183300	7B-1921060-T2-5	reverse	290,020,067	TGAGGTGGATGACGAGCTCGAGTCACGGGGCTCCCGTGGCGAGAGATC	9	83.4
T3-17	PGM		TraesCS7D02G284900	7D-2042690-T2-5	reverse	305,834,137	TGCCGAGGGACAAACAATGAAAGTATATAGATATTGAGTGATGTGTGAG	12	50.1
T3-18	PGM	ABD	TraesCS3A02G306900	3A-0625130-T3-18	forward	545,151,928	TATTGATATAAGTATCCCTAATAAGTCAGGATATCACATACACCTCACTACC	4	17.9
T3-18	PGM		TraesCS3B02G329400	3B-0725970-T3-18	reverse	532,346,258	GGATGAATCGCGGAGGAGACAAGGCGAGTCACGGAGGAGATTGGCGC	14	68.7
T3-18	PGM		TraesCS3D02G294900	3D-0869330-T3-18	reverse	406,404,790	GGATGAATCGCGGAGGAGACAAGGCGAGTCACGGAGGAGATTGGCGC	14	70.8
T3-19	PGM	ABD	TraesCS3A02G495800	3A-0631700-T3-19	reverse	721,584,181	TATGGACCGGGCCAAATCTGCGCCTGTCCTAGCTCGCTGCAAACGAACA	12	60
T3-19	PGM		TraesCS3B02G559400	3B-0756980-T3-19	forward	793,263,736	CTTCTGTCATTGCTGCTCCTCCCATCACCACAGCCGGTCCAAGTAAATTC	2	14.1
T3-19	PGM		TraesCS3D02G503300	3D-0849370-T3-19	reverse	591,254,259	CGATCTTTCTCCGTCGATCTTGTTTTCGTTTTGTGCGGTTCTCCGATCGAT	15	68.5
T3-2	Sucrose synthase	BD	TraesCS4B02G167500	4B-1042030-T3-2	reverse	364,682,450	GGCTGGACCCGTGTCACTGTGCTCCCGTCCCGCGATGTTGCGAGCGCTC	10	47.5
T3-2	Sucrose synthase	no A	TraesCS4D02G169800	4D-1153090-T3-2	forward	295,013,191	CGGCAGGAGCTGCTTCCCCCGGTGGTCGCGTAGGGGGCAGTTCGGGG	18	80.9
T3-20	PGM	ABD	TraesCS1A02G146600	1A-0047200-T3-20	reverse	252,828,691	TTCCAAGGACGGATGAGCTAAATGCAGCAAGAGACAAATTATTTGGGA	14	59.3
T3-20	PGM		TraesCS1B02G164900	1B-0104590-T3-20	forward	288,159,824	GTCCCCGACACAAACTTAGCCCAAATCTGGGAACAAATGGGGACGAAG	21	88.9
T3-20	PGM		TraesCS1D02G144800	1D-0233240-T3-20	reverse	199,927,763	TTCCAAGGACGGACGAGGTAAATGCAGCAAGAGACAAATTATTTGGGA	15	69.2
T3-21	PGM	BD	TraesCS7B02G017100	7B-1924090-T3-21	reverse	14,875,826	ATTCTCGCTTTCAACTAAAATAAGCTGCCCCTTTCGAAAGCTGTGAAGA	16	66.4
T3-21	PGM	no A	TraesCS7D02G118300	7D-2027430-T3-21	forward	73,128,644	GCAATTTTAGTTTTTTATGATGGCAATTCCAGTACTTTGACCATAAAAATA	21	81.9
T3-22	OsBT1-1	AB	TraesCS6A02G175100	6A-1573650-T2-61	forward	189,920,981	TTCAAACAGGACTTGCACTACCATGTAGACTCCTAGTGAATTACATCTTG	19	83.1
T3-22	OsBT1-1	no D	TraesCS6B02G210000	6B-1646110-T2-61	reverse	276,746,931	CTGATTCAACTCGAACACATAAAGGATTATTATATGCAAGGGCGAAAGA	16	68.2
T3-23	APL1/AGPL3	ABD	TraesCS5A02G472000	5A-1225500-T2-35	forward	649,187,425	AAAGGTATTTTTTTAGAAAAATGACTTAAATATTATGTTCTTTTTGTTAAT	11	57.9
T3-23	APL1/AGPL3		TraesCS5B02G484700	5B-1286990-T2-35	forward	656,228,474	TTTTTTATTTTATGCAACGTTCCCCTTCAGAAATTGTAGAGAACTTAAATG	23	72.2
T3-23	APL1/AGPL3		TraesCS5D02G484500	5D-1438030-T2-35	forward	520,719,360	CATGGCAAATTCACAAAAAAAAAACTGCCAATTAGATCATGGCAAATGA	15	76.2
T3-24	APS1/AGPS1	ABD	TraesCS5A02G292900LC	5A-1192890-T2-39	reverse	375,579,322	CGGGGGCAGAGTGTGACGGGTCGAGGCGGTGGCAGCGACGGCCGAG	13	58.8
T3-24	APS1/AGPS1		TraesCS5B02G175800	5B-1354680-T2-39	reverse	321,624,817	CGGGGCCAGAGAGTGACGGGTCGAGGCGGTGGCAGCGACGGCCGAG	15	69.6
T3-24	APS1/AGPS1		TraesCS5D02G182600	5D-1448600-T2-39	reverse	283,493,724	CGGGGGCAGAGAGTGACGGGTCGAGGCGGTGGCAGCGACGGCCGAG	9	37.2
T3-25	G6P-Pi translocator	ABD	TraesCS2A02G220400	2A-0338910-T2-13	forward	208,109,358	TCAGTCTAGTGGATACGACAGCAAGACGTCACCCTAAACAATTTAATATC	5	21.6
T3-25	G6P-Pi translocator		TraesCS2B02G245900	2B-0461810-T2-13	forward	250,065,630	GCGACGGCAGGAGGCAGAAACCCTAACTTTTTTGTGGAGGAAGGAGG	5	18.4
T3-25	G6P-Pi translocator		TraesCS2D02G226000	2D-0601440-T2-13	forward	194,187,709	GAAATGAACAAAGACACAATTTAACCTATTCAAATGGCTAAGTAGGCGA	8	32.9
T3-26	Starch PPase	AAB, A	TraesCS3A02G366300	3A-0614380-T3-26	reverse	615,280,622	TTTTGTTGTGAAATAGTGGGTTTTATTCGAGTGGGACGTTTTGGTGACC	25	95.8
T3-26	Starch PPase		TraesCS3A02G366400	3A-0650140-T3-26	reverse	615,705,716	AGGCTAAAGCGTTCAGAACTTCTCCACCGGCCCATTTCCGCCTATCACAT	19	79.8
T3-26	Starch PPase		TraesCS3B02G329400	3B-0753850-T2-29	reverse	532,347,958	CGTGGGGTGGGAGATGGCGGTGTGTGTGGGGGAGGTGGTGAGTTCGC	14	67.5
T3-26	Starch PPase		TraesCS7A02G287400	3D-0841320-T2-29	reverse	342,608,834	CGTGGGGTGGGGAATGGCGGTGTGTCTGGGAGGTGGTGAGTTCGGGC	9	34.9
T3-27	Starch PPase	ABD	TraesCS5A02G395200	5A-1199980-T2-27	reverse	590,605,208	TGGCGGCAGTGGTCGCGCTGACGGTGGTGTGCAGCGCGCGCGATGCG	12	46.8
T3-27	Starch PPase		TraesCS5B02G400000	5B-1299180-T2-27	reverse	577,306,200	CTGAACTGCAACTTTAGAGGGGAAGTCCTCCCAGTTGAGAGAATCACC	20	58.2
T3-27	Starch PPase		TraesCS5D02G404500	5D-1428360-T2-27	reverse	469,560,617	TGGTGGCAGTGGTCGCGCTGACGGTGGTGTGCAGCGCGCGCGATGCG	21	87.3
T3-28	GBSSI	A,AD	TraesCS4A02G418200	4A-0093880-T2-53	reverse	688,100,234	CGCGCGCGCAGGGTGTGGCTACCTGCAGCGAGAGACGACTACACTGAT	16	61.9
T3-28	GBSSI		TraesCS7A02G070100	7A-1817020-T2-53	forward	35,766,248	CAAGCAAAAGAGAAAAAGAAGGGGCGAAAGGTGATACGCCCGGCCGT	12	57.2
T3-28	GBSSI		TraesCS7D02G064300	7D-2037380-T2-53	forward	35,596,364	AATCAATGAAGCAAAAGAGAACAAGAAGAGGCGAAAGGTGACACGCC	9	43.8
T3-29	GBSSII	ABD	TraesCS2A02G373600	2A-0273760-T2-54	forward	616,979,816	ACGGCTCCCGTGCCGCCTCCCATCCCCACGCGAGGGCCCGGCGCTTCAC	18	80.9
T3-29	GBSSII		TraesCS2B02G390700	2B-0429850_T2-54	forward	555,321,220	CGCTACCCGTGCGACACCTCCCCCTCCTCTTCCCATCCGCAGGATAATTC	19	81.4
T3-29	GBSSII		TraesCS2D02G369800	2D-0502660_T2-54	forward	474,502,378	CCGAACCGAGTCCGACGCACGCAACGCGCCGGTGCCGGTACGGTGGC	9	47.1
T3-3	Sucrose synthase	A,AD	TraesCS4A02G446700	4A-0958320-T3-3	reverse	714,180,521	CGCTCAAGCCTAGTCTGCTGCAGAACAGGACAGGGTTATCAGTGGTGT	13	58.8
T3-3	Sucrose synthase	no B	TraesCS7A02G040900	7A-1808380-T3-3	forward	19,003,197	CCTCACCATCGACGACCGAGGTCGCCGGAGGACGGCGCTGGAAGATTC	4	24.7
T3-3	Sucrose synthase		TraesCS7D02G036600	7D-2043420-T3-3	forward	18,679,580	TTATTACAATTTTTTTTCTAGAATACATATATTAATATTGTATGTAACCATCTC	6	29.2
T3-30	SSI	ABD	TraesCS7A02G120300	7A-1826470-T2-43	reverse	77,441,923	CTGGAGGTGAGCCCGCAGAAAACAGGAGGCGGGCGGAGGTGCGGAC	19	84.7
T3-30	SSI		TraesCS7B02G018600	7B-1942520-T2-43	reverse	15,602,178	GGGTGCGGGCCCGCGGAGCAGAGGAGGAGGTGCGGACGGGCGGAC	23	89.9
T3-30	SSI		TraesCS7D02G117800	7D-2043770-T2-43	forward	72,938,824	TGCGTCGTCCGCGCGCGGCTCCGGCGCTTGGCGCGGGGCCGCTACGTC	24	93.6
T3-31	SSIIa	ABD	TraesCS7A02G189000	7A-1809640-T2-45	forward	145,464,919	TGTCCAAGAAATGGGATGGAGAATAGATGCATGTACGTGTCCTGGATATC	6	25.4
T3-31	SSIIa		TraesCS7B02G093800	7B-1945350-T2-45	forward	107,604,543	AAATATTTTGAAATGTTAATCAAGGATTTGAGAAATGCTAAACGTGTATAC	13	50.9
T3-31	SSIIa		TraesCS7D02G190100	7D-2059200-T2-45	reverse	144,137,287	GGATCGGGGCGGGAGATGGTACGGTTCGCGGGTTGGTCCGCCCAGAG	11	32.9
T3-32	SSIIb	D	TraesCS6D02G287000	6D-1692940-T3-32	reverse	395,694,101	AAGAAAAAAAGAAATTCAGGTTGCGACAAGTAGCGTGCATGCAGTGCC	10	39.5
T3-33	SSIIc	ABD	TraesCS1A02G137200	1A-0001960-T2-49	forward	221,695,822	GAAGAGAGCTTTATTGTTTTATCGTAAGAGCATGGTTAATAGTACAACCC	19	83.5
T3-33	SSIIc		TraesCS1B02G155700	1B-0101070-T2-49	forward	263,548,087	ATCGAAAAGAACAATTATGTGAACAGAAATGATCAACCAGCCTTGGGA	20	85.2
T3-33	SSIIc		TraesCS1D02G138100	1D-0205540-T2-49	forward	189,646,049	AGAGCATGGTTAATAGTATAACCCACAATGGGATATGTGAAGTTAATAGC	20	80.7
T3-34	SSIIIa	AB	TraesCS1A02G091500	1A-0075220-T2-50	forward	84,002,707	GCACTTATTCGCAAAATTGCTTTTGTGTTCATATTTGACGTGAGGCTGGT	21	91.1
T3-34	SSIIIa	no D	TraesCS1B02G119300	1B-0177050-T2-50	forward	141,283,814	TGGAACCGCTTTAGAAAAACGATCGAGAGGTGACATCCTTTCCTAATCT	15	69.6
T3-35	SSIIIb	ABD	TraesCS2A02G468800	2A-0288560-T2-51	reverse	712,581,222	TGCCCAGGGAAAAATCAAAACCCGACCCCCTCCGCTGATCCCTTCCCCC	21	91.1
T3-35	SSIIIb		TraesCS2B02G491700	2B-0423210-T2-51	reverse	690,034,415	TGCCGAGGGAAAATCAAAACCCGACCCCCTCCGCTGATCTCTCCCCCT	21	81.2
T3-35	SSIIIb		TraesCS2D02G468900	2D-0540290-T2-51	reverse	574,111,937	TGCCGAGGGAAAATCAAAACCCGACCCCCTCCGCTGATCTCTCCCCCTT	17	70.6
T3-36	SSIVb	ABD	TraesCS1A02G353300	1A-0010710-T3-36	reverse	536,965,650	GGGGAATAGGGAGAGGCAGGGCGACTGGTGGGGCGAGGGGAGCTTC	26	85.4
T3-36	SSIVb		TraesCS1B02G368500	1B-0108420-T3-36	forward	598,877,339	TTCTGTTGTCTTTGGCGACACCCGCGACAAAGCTAGTGCACATGGAGCA	12	55.1
T3-36	SSIVb		TraesCS1D02G356900	1D-0210070-T3-36	forward	441,067,296	CCCCTCCATCTAGAAAAAGAAGTAAAAAGGAAAAAAAACACTCATCAAC	14	62.6
T3-37	SBEI	ABD	TraesCS7A02G549100	7A-1766600-T3-37	reverse	723,265,225	CTTCGTCGATGGCGGCGGACGGGGATCCCATTGTCCGCGGGGCCCGTG	13	62.1
T3-37	SBEI		TraesCS7B02G472500	7B-1879200-T3-37	forward	728,691,955	TTAGTTGTTGTAAATCTGCCGCTCGCTCTAACTGTCGTTCGTTTATGGAAC	14	69.2
T3-37	SBEI		TraesCS7D02G535400	7D-1976420A-T3-37	forward	627,326,089	GGGAAATCCTGATTGACCAAACACCATAATTATCCTTTGTCATCCTGTGC	14	65.9
T3-38	SBEI	ABD	TraesCS7D02G535500	7A-1766610-T3-38	reverse	723,280,279	CGTCGTCAACGGCGCAACCGAGGGCGGTGGAGCTGTCAGCCTCGGTG	10	59.5
T3-38	SBEI		TraesCS7A02G549200	7B-1879190-T3-38	forward	728,665,334	GCCTCCACGGGCTCCTGTTCATCCAGCCTCCACCGCGCGCTACTCCACC	11	60.7
T3-38	SBEI		TraesCS7B02G472400	7D-1976410-T3-38	reverse	627,350,874	CGTCGTCGACGGCGCAACCGAGGGCGGTGGAGCTGTCAGCCTTGGTG	5	28.7
T3-39	SBEIIa	ABD	TraesCS2A02G293400	2A-0289500-T3-39	forward	504,334,462	AAACCACAATGATATGTAACGTAAAGGAGCATGGAGGACGTGTATGATA	6	35.8
T3-39	SBEIIa		TraesCS2B02G309500	2B-0425980-T3-39	reverse	442,525,037	CCCCGCGCAGCGAGTGAGTCGGTCAGTGGCCCAGATCTCACTCAACCC	10	50.8
T3-39	SBEIIa		TraesCS2D02G290800	2D-0515330-T3-39	forward	372,931,549	GATATATTTTCCAATAGACTTGGTCAAACTTAAAGAAGTTTGACTTAGGA	6	39.9
T3-4	Sucrose synthase	ABD	TraesCS2A02G406700	2A-0316300-T3-4	forward	662,338,619	ATCTGACAACCAGGATGGAGATTGAGAAAGTTGAAGGCAGCTCAAACT	10	57.4
T3-4	Sucrose synthase		TraesCS2B02G424300	2B-0388680-T3-4	forward	610,415,963	TGAGAAAGTTGAAGGCAGCTCAAACTATTACTTGTTGCATCTCTGCACTC	18	86.4
T3-4	Sucrose synthase		TraesCS2D02G403600	2D-0541730-T3-4	forward	518,265,430	TACCAAACACGACATGCATTGGTTAGTTCATTGCATGCATTGTTATTAATT	17	76
T3-40	SBEIIb	ABD	TraesCS7A02G549300	7A-1766630-T2-55	forward	723,293,490	CGTCTTCCACGGCACGGCGGGGCGCCGCGGAGCTGTCAGCTCGGCG	37	85.4
T3-40	SBEIIb		TraesCS7B02G472300	7B-1913520-T2-55	forward	728,624,346	GATGATGTGCCCATATTTGGTTTTCTGGGTGTAGTGTTGTCTTCTGCAGG	7	17.9
T3-40	SBEIIb		TraesCS7D02G535600	7D-1976400-T2-55	forward	627,358,921	CGTCGTCCACGGCACGGCGGGGGTGTCGGCGGAGCTCGTGGCCCGGC	8	28.8
T3-41	SBEIII	AD	TraesCS7A02G336400	7A-1773500-T3-41	forward	492,237,817	CAAGGGGTACCATGACTGACTGCCGCCGAATCGAGAATTAAGATGTACT	13	61.6
T3-41	SBEIII	no B	TraesCS7D02G344000	7D-1978090-T3-41	forward	440,559,949	TGTATATTCCCAAGAAACACACCTCAAATTTATATCGTATTTTTTCACCGC	20	76.9
T3-42	ISAI	ABD	TraesCS7A02G251400	7A-1824860-T2-58	forward	235,460,722	GCAATTATTTTCATGGTGACGTGGGGTCACTGGAATTAGTTCCTACTGAC	14	65.4
T3-42	ISAI		TraesCS7B02G139700	7B-1931390-T2-58	reverse	176,007,199	GCCGGGAACCCCGAGCGATCGGTGATTGATTTTGGGGAGGAGCGGCG	14	62.1
T3-42	ISAI		TraesCS7D02G249500	7D-2040790-T2-58	forward	220,970,552	CCGAGACTGATCGGGTCTTCCCGGGAGAAGGTTTATCCTTCGTTGACCG	15	69.2
T3-43	ISAII	ABD	TraesCS1A02G247100	1A-0053840-T2-59	reverse	438,368,309	GGGGGCCTGCGCGGCGCGATCAGCCGTCGGGGAATTGCGTCGAGCAG	7	39.1
T3-43	ISAII		TraesCS1B02G257700	1B-0112170-T2-59	reverse	453,515,532	GTGGGGCTAGCGCGGCGCGATCAGCCGTCGGGGAATTGCGTCGAGCAC	8	39.3
T3-43	ISAII		TraesCS1D02G246300	1D-0203340-T2-59	reverse	338,943,720	GGGGCCGGCGCGGCGCGATCAGCCGTCGGGGAATTGCGTCGAGCAGT	24	73.5
T3-44	ISAIII	ABD	TraesCS5A02G248700	5A-1227100-T2-60	reverse	462,873,670	CCAAATGCGGGGGTGCTGGGACGGGTGGCGGAGGGATTTGGCTCC	11	46.4
T3-44	ISAIII		TraesCS5B02G246400	5B-1334970-T2-60	reverse	427,939,462	TGCGCTTTCCCTTTCCTGACGGTGTTACAGAATGAACATGCACTTTAGCA	12	54.2
T3-44	ISAIII		TraesCS5D02G255800	5D-1467800-T2-60	reverse	362,066,416	CCAAATGCGGGGGTGGTGGGACGGGTAGCGGAGGGATTTGGCTCGC	12	64.9
T3-5	Sucrose synthase	ABD	TraesCS7A02G557600	7A-2116050-T3-5	reverse	730,429,753	GGTGGGAGACTACCAGGCTTGATAAACTGGGTGGAGAAGATCAAAGA	8	42.1
T3-5	Sucrose synthase		TraesCS7B02G482200	7B-1866170-T3-5	reverse	740,036,881	GTTGGAAGACTACCAGGCTTGTGAAACTGGGTGGTGAAGACCCAAGA	12	64.9
T3-5	Sucrose synthase		TraesCS7D02G552800	7D-2003310-T3-5	forward	635,585,023	TCTGGAGTGATACGGTTTAATATGGTTTAAGTACTTGCAGGAGTACTTGC	16	63.1
T3-6	Sucrose synthase	AB	TraesCS7A02G158900	7A-1839220-T3-6	reverse	115,208,145	GGCTCAGGACACAAAACTGCAGAGCAAGGTAAAGATCAGCATTCAGCC	21	88.5
T3-6	Sucrose synthase		TraesCS7B02G063400	7B-1922120-T3-6	reverse	68,348,404	GGCTCAGGTCACAAAACTGGAGAGCAGGGTAAAGATCAGCAGTCAGC	6	32.5
T3-7	Sucrose synthase	ABD	TraesCS2A02G168200	2A-0330380-T3-7	reverse	121,145,658	TGCTGTCCTCAAACCTGAACAGCGGAAACAGTCATGTAAATCTGAACAG	21	81.2
T3-7	Sucrose synthase		TraesCS2B02G194200	2B-0451230-T3-7	reverse	171,034,762	TGCTGTCCTCAAACCTGAACAGCGGAAACAGACATGTAAACCTGAACAC	2	10.8
T3-7	Sucrose synthase		TraesCS2D02G175600	2D-0577630-T3-7	reverse	119,070,977	TGCTATCCTCAAACCTGAACGGCAGAAACAGTCATGTAAATCTGAATAG	14	57.4
T3-8	Fructokinase	BD	TraesCS3B02G433000	3B-0799040-T3-8	reverse	671,996,341	AGATATCAATATACAAGCCCAGAGTGGGCCGACTCCTAGTAGGCGGCCA	11	36.2
T3-8	Fructokinase		TraesCS3D02G394200	3D-0840030-T3-8	forward	509,228,735	GTAGTAAGGATGCCATTAGTGATGAGAACACATTAATGGTTGAACAATG	5	17.9
T3-9	Fructokinase	ABD	TraesCS5A02G286200	5A-1205960-T3-9	reverse	493,866,887	CTCCGGGCACACGGAGGGAGACAGAGAGAGAAAGAGAGCGGGGGGC	2	10.8
T3-9	Fructokinase		TraesCS5B02G285500	5B-1315300-T3-9	reverse	470,978,978	TTCCGGGCACACGGATGGAGACAGAGAGAGAAAGAGAACTGGGGGG	15	52.4
T3-9	Fructokinase		TraesCS5D02G293800	5D-1435750-T3-9	reverse	390,573,147	TTCCGGGCACACGGACGGAGACAGGGAGAGAAAGAGAACGGGGGA	17	72.7

red=duplicate genes

MYbaits ID	MYbaits sequence
6B-1509400B-T3-1_0	GCGACAAGATGGGGAGCCGGGAGGAGATGCGGTGGCGCAGTTCCCCTCAGCCTGGCTGAGAGGGCTCTTTTGCTGCACAGGTGCCACATGCAAGCAGTCATCCAAATGCG
6B-1509400B-T3-1_64	CTCTTTTGCTGCACAGGTGCCACATGCAAGCAGTCATCCAAATGCGCCCTTGGTGTTGTTTGTTTGTCTGGGCTTGATTACTTCTTTTGTTGCTCCGGACCATCCCCCCAAAA
6B-1509400B-T3-1_128	TGTCTGGGCTTGATTACTTCTTTTGTTGCTCCGGACCATCCCCCCAAAAAAACTATCCACTTTTTTTCTTATTCTTTCCACTTTTGTTACCGGCAACTTTCATGCATAATTCTGTC
6B-1509400B-T3-1_192	TTTTTCTTATTCTTTCCACTTTTGTTACCGGCAACTTTCATGCATAATTCTGTCCATCGAAAATTAGGCTTCCTAAACACATTAAATATTCCTCATGGACTAATTACATTATATATTGA
6B-1509400B-T3-1_256	TAGGCTTCCTAAACACATTAAATATTCCTCATGGACTAATTACATTATATATTGAGAAAAACATATATTATTTTCTAGTCCAGAATCATGCAAGGGAAATACACCTTTTGGTGTTATG
6B-1509400B-T3-1_320	TATATTATTTTCTAGTCCAGAATCATGCAAGGGAAATACACCTTTTGGTGTTATGAACAGGTGAAAGTTCGCATGTGTTTAAATAATTCACGTGTCCTTCATTTTATTTCACGTCTTA
6B-1509400B-T3-1_384	AAGTTCGCATGTGTTTAAATAATTCACGTGTCCTTCATTTTATTTCACGTCTTAACACGCAGTCATATGTAAAAAGCTGTCAAATTTATGAAGCAAGGCGGTCTCATAAATTATCTT
6B-1509400B-T3-1_448	ATATGTAAAAAGCTGTCAAATTTATGAAGCAAGGCGGTCTCATAAATTATCTTTTCCAGTATTATATTTTCTAATTTCTAGCATGCTTTTTTGCTGGAAAAACATCTGATCTACTAC
6B-1509400B-T3-1_512	TATTTTCTAATTTCTAGCATGCTTTTTTGCTGGAAAAACATCTGATCTACTACTTATAATGTATCATGACAGTACAAAAAACACCAGAGGTAATAGGTCTATTGACCACCTAGTGAT
6B-1509400B-T3-1_576	TCATGACAGTACAAAAAACACCAGAGGTAATAGGTCTATTGACCACCTAGTGATGACTAGAAATACTGGAGCAAGCCGAAAGTCATCAAAGACGGGGCGGACCGGTGGCGGT
6B-1509400B-T3-1_640	ACTGGAGCAAGCCGAAAGTCATCAAAGACGGGGCGGACCGGTGGCGGTGCTAGAGCATGTAAATATAAATATGGGAAACGATCAAAGTTATATGTTAGACAGATACTCATCCT
6B-1509400B-T3-1_704	ATAAATATGGGAAACGATCAAAGTTATATGTTAGACAGATACTCATCCTACATCGAAAGCGATGTCTCTTTACGGATGGAAGGGGGGCTAAACATACATTATTATTTTTGTAAAAC
6B-1509400B-T3-1_768	TCTCTTTACGGATGGAAGGGGGGCTAAACATACATTATTATTTTTGTAAAACAATGCTAAACATACATTTAAAGATTCCAATATAAACTACATACAAAGCAAAATGAGTGAATCAA
6B-1509400B-T3-1_960	GGAGTAATTGATTTGTTTCCAGGAGTTTTTGCACATTTCTAATATAGTAACAATACAGATGCTAATGAATATATATATCGCACTACCGAAAAAACACGTAATACTATGATTTAGAAG
6B-1509400B-T3-1_1024	ATGAATATATATATCGCACTACCGAAAAAACACGTAATACTATGATTTAGAAGGAAAACAAATTCTAAACTTTTCACATGGTGAACTAGTAGTAGCCTAATAGGTGCAGCCTTAGT
6B-1509400B-T3-1_1088	CTAAACTTTTCACATGGTGAACTAGTAGTAGCCTAATAGGTGCAGCCTTAGTCCATAGAATACAAGACTCTTCTTCTGCCTGTAAAGGTAGAAACCCAAAAGGCTAAGCTAGGCT
6B-1509400B-T3-1_1152	AGACTCTTCTTCTGCCTGTAAAGGTAGAAACCCAAAAGGCTAAGCTAGGCTAGCCTAGGTAAAAAGGTAACCATGAATCTTAGCTAGCTTAGTTATCTATACCACCATGAGAGG
6B-1509400B-T3-1_1216	AGGTAACCATGAATCTTAGCTAGCTTAGTTATCTATACCACCATGAGAGGAACAAATCTCCTCCTCCTTAATTTACACCCAGACAAGATTAACCAAACGTGAATATTTACATCT
6B-1509400B-T3-1_1280	CTCCTTAATTTTACACCCAGACAAGATTAACCAAACGTGAATATTTACATCTTCCGACATGCAATTCGGTTGTGCATTTACAAGGGGACATGAATTAAATTGAATGTTTTTGTGC
6B-1509400B-T3-1_1344	ATTCGGTTGTGCATTTACAAGGGGACATGAATTAAATTGAATGTTTTTGTGCCAACGATTAATCGAATCTTAATTATCTTGCATGGCGTAACTATAACATGTAAACAAATTGATTAA
6B-1509400B-T3-1_1408	GAATCTTAATTATCTTGCATGGCGTAACTATAACATGTAAACAAATTGATTAATTAACAAGGGGAACAAAATAAATGCCTTGCTTAAAGTGTCACCCAtcctccttcttccttttgcttc
6B-1509400B-T3-1_1472	AACAAAATAAATGCCTTGCTTAAAGTGTCACCCAtcctccttcttcctttgcttcctctcttgcctttcatcttcctcctctcctctgaaagagacagacagcagagacagagacggag
6B-1509400B-T3-1_1536	cctttcatcttcctcctctcctctgaaagagacagacagcagagacagagacggagGAGAGCATAATTAGTTAACTTGCTTCCATCTTCCTCCCCTTGCCTATAAACTTTGCCTTGCCAT
6D-1709240-T3-1_0	TTTCTCGAAAAAAAAAAGTTCATTGTTGCTGTCGTGGGCCATGAGGCACGTGTCCGAGCTTGCTTGGTGGGTTATTGAGACGCTTCCTCCTTTGAACCCTTTTGCCTGCGTGTT
6D-1709240-T3-1_64	TGGTGGGTTATTGAGACGCTTCCTCCTTTGAACCCTTTTGCCTGCGTGTTTCTTTCAGGTCTAGGCTTGTTTGCTGCTTCGGTTTCCCGCTGTTGATCGGTCAGTCGTCCCTGCG
6D-1709240-T3-1_128	GCTTGTTTGCTGCTTCGGTTTCCCGCTGTTGATCGGTCAGTCGTCCCTGCGCTGCACTTGAGGTACGCACACTCATCATTTCTTTAGCAGGAGCTTTTTGTTAACAAGAAAAGA
6D-1709240-T3-1_192	ACGCACACTCATCATTTCTTTAGCAGGAGCTTTTTGTTAACAAGAAAAGAGCTGATTTTTTTTGAGAAAAACAAGAAAAGAGCTGATGCCTGCTTGATGTTATATCATGGCTT
6D-1709240-T3-1_256	GAGAAAAACAAGAAAAGAGCTGATGCCTGCTTGATGTTATATCATGGCTTGTAGGCTTGCTTGATAAACTGACCTGGTGCCTGCTAGTGGGCCATTCATTTGGGTCTTGTTTG
6D-1709240-T3-1_320	GATAAACTGACCTGGTGCCTGCTAGTGGGCCATTCATTTGGGTCTTGTTTGTTTGTCTGGGCTTGATTATTTCTTTTGCTGCTCTGGCCCATCGACGACAGATCTCTTCTTATTCT
6D-1709240-T3-1_384	GATTATTTCTTTTGCTGCTCTGGCCCATCGACGACAGATCTCTTCTTATTCTTTCTGCATTTGTTACCAGCAACTTTCACTCGGATAAAGCATAATTCTGACCGTTGAAAAATTGG
6D-1709240-T3-1_448	TTACCAGCAACTTTCACTCGGATAAAGCATAATTCTGACCGTTGAAAAATTGGCTTCCTAAAACATACTGTATGAAATTTTCCTCATGGACCACTTTGCATTATATACCGAAatatata
6D-1709240-T3-1_512	ATACTGTATGAAATTTTCCTCATGGACCACTTTGCATTATATACCGAAatatatatatatatatatatatatatatatatatatatatatatatatatatataAAATTCAGTTCAGAA
6D-1709240-T3-1_576	atatatatatatatatatatatatatatatatatataAAATTCAGTTCAGAATCATGCAAGGGTAATTCACCTTTTAATTTTGTCAACAGTTGAAAGTTCACATGTGGTGCAAACA
6D-1709240-T3-1_640	GGGTAATTCACCTTTTAATTTTGTCAACAGTTGAAAGTTCACATGTGGTGCAAACAAACCACATGTGTTTTCATTTTATTTCAGGTATAACACGCAGTCATATCTGGAAAGCGGT
6D-1709240-T3-1_704	GTGTTTTTCATTTTATTTCAGGTATAACACGCAGTCATATCTGGAAAGCGGTCAAATTTCTGAAGCGAGGCTGTTATAAATTATTGTTTCCGCTACTATATTTTCAACCCCGTCCGT
6D-1709240-T3-1_768	GCGAGGCTGTTATAAATTATTGTTTCCGCTACTATATTTTCAACCCCGTCCGTTCACAAATATAAGATGTTCTAATTCTTTGAGAATCAAATCTATACACACATTttttattttatttt
6D-1709240-T3-1_896	ttagttcatattGAAATATCCAAAACATCTTATATTCATAAACAATGAGAGTAGTTAATTACTGGAAGAGGGGAATAAAATTGCTTGATAAACATCTTACATTCATACTTACGGGAATGAG
6D-1709240-T3-1_960	GAAGAGGGGAATAAAATTGCTTGATAAACATCTTACATTCATACTTACGGGAATGAGGGATAACATGCGTTTTTTTTTTACAAACGATGCTAATTAATCTGTTCCAGGAGTTTGT
6D-1709240-T3-1_1024	TGCGTTTTTTTTTTTACAAACGATGCTAATTAATCTGTTCCAGGAGTTTGTAGACATTTTGAAGGTTTTGAAGGAAATATTTTCAGGACTCTTCAGATGGTGAACTTTTTCAGT
6D-1709240-T3-1_1088	AGGTTTTGAAGGAAATATTTTCAGGACTCTTCAGATGGTGAACTTTTTCAGTGCACGGGTGCATAGAATACAAGACTCTTCTGCATGTAAAGGTAGAAACCCAAAAGGCTAGG
6D-1709240-T3-1_1280	CTTCCATCTTAATTTTACACCCAGACAAGATTAACTGAGCATGAAAATTATCATCTTCTGACATGCAGTTCAGTTTTGCATTTACAGGCGGGCATGAATTAAACTGAATGTTTTTG
6D-1709240-T3-1_1344	TGCAGTTCAGTTTTGCATTTACAGGCGGGCATGAATTAAACTGAATGTTTTTGCACCAACGATTAATCGAATCTTATCTAACATATAAACAAATTGATTAACAAGAGGAACAAAAT
6D-1709240-T3-1_1408	AATCGAATCTTATCTAACATATAAACAAATTGATTAACAAGAGGAACAAAATAAATGCCTTGCTTAAAGTGCCACCCATCActcttctttcctttgcttcctctccttcctctcttgcct
6D-1709240-T3-1_1472	TAAAGTGCCACCCATCActcttctttcctttgcttcctctccttcctctcttgcctttcatcttcctcctctcctctGAAAGGGACAGACAGAGACAGAGACAGAGGAGAGCATAATTAG
U-1509400A-T3-1_128	ctctgtctgtctctttcAGAGGAGATGaggaagatgaaaggcaagagaggaaggaggaggaagcaaaggaaagaagaaggaTGGGCGACACTTTAAGCAAGGCATTTTATTCCCCTCGTTA
U-1509400A-T3-1_192	aaggaaagaagaaggaTGGGCGACACTTTAAGCAAGGCATTTTATTCCCCTCGTTAATCAATTCGTTACATGTTATAATTATGCCATGCAAGATTCGATTAATCCTTGGCACAAAAAC
U-1509400A-T3-1_256	GTTTACATGTTATAATTATGCCATGCAAGATTCGATTAATCCTTGGCACAAAAACATTCAATTTAATTCATGTCCTCTTGTTAATGCAGAACCAAATTGCATGTCGGTTAAATCTTATC
U-1509400A-T3-1_320	AATTCATGTCCTCTTGTTAATGCAGAACCAAATTGCATGTCGGTTAAATCTTATCTGGGTGTAAAATTAGGAAGAACGATTTGTTGGCTCTGAGGTTTGCTTTGGCGTGGTGGCA
U-1509400A-T3-1_512	TGTATTCTATGCACTCGTGCACATGTCTTAAAAAATGTCTACAAACTCGTGGAAACAGATTAATTAATTAGACGAGGAGTTCCAATATATTGAGTGAAGATGGACATTGCTGAAGC
U-1509400A-T3-1_640	GAGATAAAAGTCTTCATAGGTTTTATGCTTAGTGCAAGTTATAACAATCAACAATTTACCTTTTGTGTCTAGTGTTGATCTTTAGTTGACATGGAAAAAAGGTGCTTTTTTATTCGT
U-1509400A-T3-1_704	GTGTCTAGTGTTGATCTTTAGTTGACATGGAAAAAAGGTGCTTTTTTATTCGTGCTTGCATATAAGAGGGCCTCATCGATATTTTGCGCTAGGCCCCCTGACACGCCAGGACTGC
U-1509400A-T3-1_832	CATCCAGAGGTAGAGGTATGCATATACACACACAACTTGGAAAGATTTTGAATTTCATATGATTTCCGTACATCACGGTGTTGTACTAGAAGCATTCCACAAGCACAACCGTCCTA
U-1509400A-T3-1_896	TCCGTACATCACGGTGTTGTACTAGAAGCATTCCACAAGCACAACCGTCCTACAATTAGGACCTTCGTTGAGCCGACTTTTGAAAAGAATTAAGAAAATATCCATATGAAATGGG
U-1509400A-T3-1_960	TCGTTGAGCCGACTTTTGAAAAGAATTAAGAAAATATCCATATGAAATGGGTACACGTTTTTCACTCCTATATGGTTCCTTTCTTGTAGTTCTAACCTCACAGATTAGGACAAGAT
U-1509400A-T3-1_1024	CTCCTATATGGTTCCTTTCTTGTAGTTCTAACCTCACAGATTAGGACAAGATTTATGAAAGCCGAGTTATTAACAAAGAAATTCATTCTCTAAAACACGAGGATCGACATCGGGTG
U-1509400A-T3-1_1088	AGTTATTAACAAAGAAATTCATTCTCTAAAACACGAGGATCGACATCGGGTGTCAACGATAAAAAAAACCTCAGAAAAAAAATTTGAATCGAAGCTGGCAGTAAGGACTAAGG
U-1509400A-T3-1_1152	AAAACCTCAGAAAAAAAATTTGAATCGAAGCTGGCAGTAAGGACTAAGGAAGCAGAGAAAGGGCCGAGAACGGGCCGCGACGGGCCAGTGGGGCGGTGAGAGAGGGG
U-1509400A-T3-1_1280	CGATGGCCCGCCAACGGTTACTTAGCAGGACGGTTTCCTCTCTCTCCTTCCCGTTCCTTCCGCAGAAATTCAAAATCCAAATCCCACCCCCACCTCCTCCCCTCGCCGGCGGCGA
U-1509400A-T3-1_576	TAATTAGACGAGGAGTTCCAATATATTGAGTGAAGATGGACATTGCTGAAGCAAAATTCGAATTGAGATAAAAGTCTTCATAGGTTTTATGCTTAGTGCAAGTTATAACAATCAAC
4B-1042030-T3-2_64	GCCGAGTTGCCCAACTTTATAATGCAGCGCCTTGCTGACGCGTGGTGGAGAGCGTTGGGCAGTTAGCGAAAATGATTGTACAATGTAGAGTACTCCCTCCCTCCGTTCGGAATT
4B-1042030-T3-2_384	GAACGAAGGAAGTACCTAGAACATTTTTGTGGCGTATTCGTACGGAAGTATATGGCGATTTCGTTCATTTTAGGCTCTCAAGTATGGCGTGAATGAATGTGCCCGCTCAGTCTCT
4B-1042030-T3-2_448	TCATTTTAGGCTCTCAAGTATGGCGTGAATGAATGTGCCCGCTCAGTCTCTCAAAGGTATTGTCACAATGTGCGTGCATGCGTCGATAAGGGCGAGTAtatatgtatatgtatgagcgtc
4B-1042030-T3-2_512	ACAATGTGCGTGCATGCGTCGATAAGGGCGAGTAtatatgtatatgtatgagcgtctgcatctgtactatgtTAAAAAATACGAAGTATATATGGTTTGGATTGTTAGAAAGTTATTGAT
4B-1042030-T3-2_576	tactatgtTAAAAAATACGAAGTATATATGGTTTGGATTGTTAGAAAGTTATTGATGCGATCTAAAAAAAGAAATTGTTGGTGCCTTAACGCTTGCTGTCTAAATAATACTTCCTCCT
4B-1042030-T3-2_640	AAAAAAGAAATTGTTGGTGCCTTAACGCTTGCTGTCTAAATAATACTTCCTCCTGTTTTTGTTTTACTTCGCGAATTCGCGTACAATGTTTTTAAGTCAAACTACAAAAGTATAGC
4B-1042030-T3-2_704	TTACTTCGCGAATTCGCGTACAATGTTTTTAAGTCAAACTACAAAAGTATAGGAGCAATGTTTTCATCTATTACGATGTGTTTGCGGTGACTTGATTTTTAGACGAAAAGGAGT
4B-1042030-T3-2_768	TCATCTATTACGATGTGTTTGCGGTGACTTGATTTTTAGACGAAAAGGAGTTTCCCCTCACCCCAACTTTTATAAAAAGCGAAGGCAAAACAACCAGTCACGGACTGTTAAACA
4B-1042030-T3-2_1280	TTGATCAAAAGAGTTAGGAACATTTATCGCAAAGATATCAGCAATTAAGCTCCAGACAGATTCAACAACAACGCAGTCAAAAAACAGATGCTGAATAGATTCAGGTTCACAAC
4B-1042030-T3-2_1580	GTACTTGTTAGAAGAATCTAACATCCAGAGAGGTTGATCTGGAGAGTCAGACAAACAAACAGTCTTAACAATCTCAGTGAGTTCCTCCCATCTAACTAATGTATTTTCTGTGACA
4D-1153090-T3-2_1088	tgcattgtccagatgcagaggccagggggttctcctccatttttaaaagaaaaaTGCACTTCTACAATAATAGATGAAAACATTGCCCCCTATACTTTTGTAGTTTGATTTTAAACAAAAT
4D-1153090-T3-2_1152	AATAATAGATGAAAACATTGCCCCCTATACTTTTGTAGTTTGATTTTAAACAAAATTTATATATGAAATAAAACAAAAACAGGAGGAAGTATTATTTAGACACCAAAGCGTTAAGG
4D-1153090-T3-2_1216	GAAATAAAACAAAAACAGGAGGAAGTATTATTTAGACACCAAAGCGTTAAGGCACCAATAACTTTTTCTTCTGAAAGGATCGCACCAATAACTTTTTAACAGCCCAATCATTTTT
4D-1153090-T3-2_1280	TTTCTTCTGAAAGGATCGCACCAATAACTTTTTAACAGCCCAATCATTTTTTTAAAATAGTATAAATGCGGACATTTATACATACATATTTACACTCACCCCATTAACGCATGCACGC
4D-1153090-T3-2_1344	AATGCGGACATTTATACATACATATTTACACTCACCCCATTAACGCATGCACGCATATCGTGACATTACCTTCGAGAGACTGAGCCGGTACATTCACTCACGACATACTTCAAAGCC
4D-1153090-T3-2_1408	ATTACCTTCGAGAGACTGAGCCGGTACATTCACTCACGACATACTTCAAAGCGTAAAATGGACGAAATCGCCATATACTTCCGTATGAATACACCACAAAAATGTTCTACGTAGTA
4D-1153090-T3-2_1472	AAATCGCCATATACTTCCGTATGAATACACCACAAAAATGTTCTACGTAGTAGTACATTGTATACATTTTCGCTAACTGCCCAACGCTCTCCACCACGCGTCAGCAGGCGCTG
4A-0958320-T3-3_0	CGCTCAAGCCTAGTCTGCTGCAGAACAGGACAGGGTTATCAGTGGTGTGTTCTTCAGTTCAGTTCTGTTCAGATCCGGTGACATACAAGCAGACTGCAAGTAATCAAGAAGTC
4A-0958320-T3-3_64	CTGTTCAGATCCGGTGACATACAAGCAGACTGCAAGTAATCAAGAAGTCGAAGCATATATTGCTTTAGTCGAGAGCTTGCGTGAGCGCCAACAATGGCCGTCCGTTGGAAGCG
7A-1808380-T3-3_64	GCTAGGGTTAGAGCTGCCAGGGACGAGAGCGCTTCTATTATTGTAACCAAATTTCTCGATCATACTATCTCCGTCGGCGAAACCAGTGATTTATAACATTGTGTAAGCGGTGCAC
7A-1808380-T3-3_128	CTATCTCCGTCGGCGAAACCAGTGATTTATAACATTGTGTAAGCGGTGCACATTCTTTTCCCTATATGTACATTTTGTTTCATGACATAAGATTTGTTGACTCAAAACGTCACACCA
7A-1808380-T3-3_192	TATGTACATTTTGTTTCATGACATAAGATTTGTTGACTCAAAACGTCACACCAAGATGACACGAGCGAAAAAGGAGCTCATGCCACGTATGTGTGTATATGTGAGCATCAACGAT
4A-0958320-T3-3_256	TTCTTGCAGGCCGTGCCAAGGTGGTTAAGAAGACTAATGGCGGTAATTAGGCGTACTGATGAGAAATAATCTGGAGCTAATTAGCCATGGCTGCTAGCACAAGAAAGGAGAA
7A-1808380-T3-3_256	GCGAAAAAGGAGCTCATGCCACGTATGTGTGTATATGTGAGCATCAACGATTGTACCGTGTTCAAGAAAGAAAAAGCTCATACTCGGCCAGCCGTTGATAATAATACAATCTTTT
7A-1808380-T3-3_320	AGAAAGAAAAAGCTCATACTCGGCCAGCCGTTGATAATAATACAATCTTTTTAAATGCGTACACTAGGATAAAAAACCGAAGGCCAATCCTGAGAGCAATAGCTTTGGCACAG
7A-1808380-T3-3_384	CTAGGATAAAAAACCGAAGGCCAATCCTGAGAGCAATAGCTTTGGCACAGATTAGAATTATGACCCCGCGATCCATCCATTTGATCCATCCACAGAAACCCACGTACACATTG
4A-0958320-T3-3_448	GTGAGCAAGCTCACTAGATCTGACAACATCTAAGGTGCATCCACTTGGTGGGTTGGTGCTGAAACCTTAAACCCGGAGAAGAGAATGATGGCAACTTGGCAGGACCGCGGAT
7A-1808380-T3-3_448	ACCCCGCGATCCATCCATTTGATCCATCCACAGAAACCCACGTACACATTGATGGAAAGAAATCAACATACGGGTCAGATCACAATGCACTAATTGGATAAATTGTTTCGCAAGC
7A-1808380-T3-3_512	AACATACGGGTCAGATCACAATGCACTAATTGGATAAATTGTTTCGCAAGCAAAGAGATGTGCACAGTGAAGAAGGGGGCAGGGCAAAGATGAGGGTGCGTGCCACCTGCC
4A-0958320-T3-3_640	AAACCACTTTAACagcaaaacagagcagagcatagcagagcGCTACTACTGATCGAACCAGCACGCGTAGAAGAAACAAGAACACCGTTGGCAGCAGAGGAGGACGAGCTCACCTT
4A-0958320-T3-3_704	GCGTAGAAGAAACAAGAACACCGTTGGCAGCAGAGGAGGACGAGCTCACCTTGGCACCTTGCTCTTCTTCTCCGCGGGGTAGAAACGAAGGGAAGCAGACAGAGAGAGG
7A-1808380-T3-3_704	CTCTGCTTCCCTTGGTTCCTGCCTCGCCGTGGCGTTTCCCGGGGCTCTCCTCGCGGAGAAGGAGAGCAAGGTCAGGTGAGCTCGTCCTCCTGCCAATGCCAATGCCAATGGACT
7A-1808380-T3-3_768	GCAAGGTCAGGTGAGCTCGTCCTCCTGCCAATGCCAATGCCAATGGACTTCTTCTTTCTTCTGCGCGTGCTCTGCTATGCTCTGTTTCAGTGTCGTCGGTGCGGCTTCCTCTGTT
7A-1808380-T3-3_832	GCGTGCTCTGCTATGCTCTGTTTCAGTGTCGTCGGTGCGGCTTCCTCTGTTTTCTCTCGTCTTGCTGTCTAACTGTCTAGCTTCTGTCTTAATAAGAAAAAATGGTTTGCTGGAGTAGC
4A-0958320-T3-3_896	GGTGGCACGGACCGTCATCTTTGCACTGCCCCCTTCCTCCTTCACTGTGCAGCAGGATTCCTTGCTTGCGAAACAATTATCCAATTAGTGTATTCGATTCGCGGTGATCTGAC
4A-0958320-T3-3_960	GCTTGCGAAACAATTATCCAATTAGTGTATTCGATTCGCGGTGATCTGACCCGTATCTTGATTTCTTTCCATCCATCTGTACGTGGGTCTCTGTGGCGAGAAGATACGGCCGCTC
4A-0958320-T3-3_1024	TCTTTCCATCCATCTGTACGTGGGTCTCTGTGGCGAGAAGATACGGCCGCTCTCTTCTGCTCCAGCTAGAAAACCAGATGAGGAAAGGTTCTCCCTTCTGTTCTAGCGTTGGAAGGTT
7A-1808380-T3-3_1024	TAAGGTTTCAGCACCAACTCACCAAGTGGATGCATGGATGCTGTCAGATCTGGTGAGCTTCAGTCCGTGTCGACCAAGTCTGCGCCTCAGTCCGCGTAGGTGTCGAAACGCGCG
4A-0958320-T3-3_1088	CAGGCGGGAAAAGGTTTCTCCCTTTCCTGTTCTAGACGTTTGGGAAGGTTTGGGGAGCAAGCCATGAAACAAATGGATGGATCGCGGGGTCGTAAATAAATTCGTAATCTGT
7A-1808380-T3-3_1088	CCGTGTCGACCAAGTCTGCGCCTCAGTCCGCGTAGGTGTCGAAACGCGCGATCGAAGGTCTGTCGGTTCCGGTTCTGGTGCTAGCAGCCATGGTTAATTGTTTCTGCCA
4A-0958320-T3-3_1152	TGAAACAAATGGATGGATCGCGGGGTCGTAAATAAATTCGTAATCTGTGGCCAACACTATTGCTCAGGATTGGCCTCCAGTTTTTTTTATCTTAATTAATGTATGTACTTTTGAAAA
7A-1808380-T3-3_1152	CCTTTCTTGTGCTAGCAGCCATGGTTAATTAGCTCCAGATTATTTCTGCCATTTCCCGGTAACACGAAAAGCTCGACGCCTAATCACCACCATTAGTCTTAACCACCTTGGCATGG
4A-0958320-T3-3_1216	AGGATTGGCCTCCAGTTTTTTTATCTTAATTAATGTATGTACTTTTGAAAAGAAAAGAAAAGTTTATTATTATCAGAGGCCGGCCGAGTATGAGCTTATTCTTTTAATATAGTATCATC
7A-1808380-T3-3_1216	CGAAAAGCTCGACGCCTAATCACCACCATTAGTCTTAACCACCTTGGCATGGCCTGCAAGAAAGGAATCCCACGCTGATCTCGTGCTTCTAAGGTGCTCGCACGCGTCACTCT
4A-0958320-T3-3_1280	TATTATCAGAGGCCGGCCGAGTATGAGCTTATTCTTTTAATATAGTATCATCACAGATGCTCACACACACACATGCACAGCTCAGCAATATGAGCTTCTTTTTGGGCGTGTCA
4A-0958320-T3-3_1344	CACACACACACATGCACAGCTCAGCAATATGAGCTTCTTTTTGGGCGTGTCATCTGGTGTGACGTGTTTCGAGTCAACAAATCTCTTGTCATGGAACAAAATGTACATATACGGA
4A-0958320-T3-3_1408	CGTTTCGAGTCAACAAATCTCTTGTCATGGAACAAAATGTACATATACGGATATAAAATGCACACATATTATACAATGTTTATAAATCTCTGGTTTCGCCGACAATGATGATATCGTGA
7A-1808380-T3-3_1408	TCGGCTACTTGAACTTCTCGCCGGACAACACCTGGGCTACCTGTTGGTTTCTCACTTCAGATCCGTATAATTATAAACGCACACTCCACTGCTTTCTCCTTCTCGGTCCTCTGT
4A-0958320-T3-3_1472	TATTATACAATGTTATAAATCACTGGTTTCGCCGACAATGATGATATCGTGAATTCGTGTCAATGAGGACATTTATGCACGGTTTAAGGTTAGTTCAGCTAGAGTGAGAATTTGAGTT
4A-0958320-T3-3_1536	ACACGAAACGTGCATTGCTCAGCTAAGATGAATTTGGAGGCGAATTTAAGTTGATGCTACTTTCTGACTATGTTTATACGAAGCCAACAGCCGGTTTTGTGGTAATGGTAACA
7A-1808380-T3-3_1536	GTCTAGATTTTGATTCTTGTATGCCTCTCGATCCTCTAGTTTGCGCCGTCGAATGTTTTTTTCTTTGGTCTTCTCCAAATCTTGCTCAGTTCCTGCTCATCTGTTGGATCTCTGAACT
4A-0958320-T3-3_1580	TTTAAAGTTGATGCTACTTTCTGACTATGTTTATACGAAGCCAACAGCCGGTTTTGTGGTAATGGTAACACAACAAACTTGGGTGCTTAATCTACCTCGTTCTTCCATCGCCA
7A-1808380-T3-3_1580	GCCGTCGAATGTTTTTTTCTTTGGTCTTCTCCAAATCTTGCTCAGTTCCTGCTCATCTGTTGGATCTCTGAACTGAAGAACACACCGCTGATAATCTTGTTCTGCAGGCTAGCTTG
7D-2043420-T3-3_0	TTATTACAATTTTTTTTCTAGAATACATATATTAATATTGTATGTAACCATCTCGCATTGTATTATGTTCAAAAAAAAAGAAAAAAATGAGCTCATACTCAGCCGGCTATGATAATACGC
7D-2043420-T3-3_64	ATGTTCAAAAAAAAAGAAAAAAATGAGCTCATACTCAGCCGGCTATGATAATACGGTGTTTGCTAATCTCGGACTTTGTTATGTCTCAGTCCATGTAAATTCAGTCCAACTG
7D-2043420-T3-3_128	ATCTCGGACTTTGTTATGTCTCAGTCCATGTAAATTCAGTCCAACTGCATGCCGATAATAATACATTTTATCTCTCTTTTGCAAAAAATAAAATTCAATTTATCTCTAGAAATACATACTAC
7D-2043420-T3-3_192	AGCCACGCCCCCGATAATAATACATTTTATCTCTCTTTTGCAAAAAATAAATTCAATTTATCTCTAGAAATACATACTACATTAATCAAGATAAAAAAACGGGAGGCCAATCCTG
7D-2043420-T3-3_256	CTAGAAATACATACTACATTAATCAAGATAAAAAAACGGGAGGCCAATCCTGAGCAATAGCTTTTGGCACGGATTATGACCCCGCGATCCATCCATTTGATCCATTGGCTTCGGTT
7D-2043420-T3-3_320	TGGCACGGATTATGACCCCGCGATCCATCCATTTGATCCATTGGCTTCGGTTTGGCTTCCCCAAACGTCTCGAACAGGAAAGGAAGAAACCTTTTCCCGGCTGCACACAGACACGGC
7D-2043420-T3-3_384	CTCGAACAGGAAAGGAAGAAACCTTTTCCCGGCTGCACACAGACACGGCCGTATATCGCCACAGAGACCCACGTACACATGGAAAGAAATCAAACATACGGGTCAGATCACC
7D-2043420-T3-3_448	AGACCCACGTACACATGGAAAGAAATCAAACATACGGGTCAGATCACAATGCACTAATTGATCAGCTAAATTGTTTCGCAAGCAAACAAGTTCACGAGGTCGCACAGTGAAG
7D-2043420-T3-3_512	AATTGATCCAGCTAAATTGTTTCGCAAGCAAACAAGTTCGCACAGTGAAGAAGGGGCAGTGCAAAGATGACGGTGCGTGCCACCTGCCACCCCACCCCGGCCGTGCAGCAT
7D-2043420-T3-3_704	TCGCGTGGCGGTTCCTGGGGCTCTCCTCGCGGAGAAGAAGTGCAAGGTGAGCTCCTCCACATTCCAATGGAGTTCTTCTTTCTTCTGCGTGTGCTCTGCTATGCTCTGTTTCGG
7D-2043420-T3-3_768	CAATGGAGTTCTTCTTTCTTCTGCGTGTGCTCTGCTATGCTCTGTTTCGGTGTCGTCGGTGCAGCTTCCTCTGTTTTCTCTCGTTGCTTGCTGTTTAATAAGAAAAAATAAAGTGGTTTGCTG
7D-2043420-T3-3_832	CCTCCTCTGTTTTTGCTGTTAATAATTAAGAAAAAAATAAAGTGGTTTGCTGCACTAGCCACTAGGGGAGGGGACAGAGATGGCAATCAGCATCCACTTGCGGAGATGTGGT
7D-2043420-T3-3_960	ACCGGATCTGCGGCCCTGGCAAGTTGCCATCATTCTCTTCTGTAGACGGGTTTAAGGTTTCAGCACCAACCCACCACCAAGTGGATGCACCTAGATGTTGTAGATCTAGCGA
7D-2043420-T3-3_1024	ACCAACCCACCACCAAGTGGATGCACCTAGATGTTGTAGATCTAGCGAGCTTCAGTCCGTGTCGACCAAGGCTACTCACCTCATGGATCCTCGTCGGTTCCGGTTCTGGT
7D-2043420-T3-3_1088	GGATCCTCATCCCCTTCCCGCCGAAAAGTCTGCGCCTCAGTCAGTCCGTGTCGGAGCTCGCGCTGTCGGTCGGCTCTCCTTTCTGTGCTAGCAGCCATGGTTAATTAGCTCCA
7D-2043420-T3-3_1152	TGTCGGTCGGCTCTCCTTTCTGTGCTAGCAGCCATGGTTAATTAGCTCCAGATTATTTCTGCCATTTCCCGGTAACACGAAAAGCTCGACGCCTAATCACCACCATTAGCCTTAA
7D-2043420-T3-3_1216	ATTTCCCGGTAACACGAAAAGCTCGACGCCTAATCACCACCATTAGCCTTAACCACCTTGGCATGGCCTGCAAGAAAGGAATCCCACGCTGATCTCGTGCTTCTAAGGCGCTC
7D-2043420-T3-3_1408	CCGGCTTCTGTCGGCTACTTCAACTTCTCGCCGGACACCACCTGGGCTACCTCTAAACTATAATTATAAATGCGCACACTCCACTGCTTTCTCCTTCTCGGTCCTCTGTCTGAAAC
7D-2043420-T3-3_1536	TTTTGATTCTTGTATGCCTCTCGATCCTCTAGTTTGCGCCGTCGAATGTTTATTTTTCTTTGGTCTTCTCCAAATCTTGCTCGGTTCCTGCTCATCTGTTGGATCTCTGAACTGAAG
7D-2043420-T3-3_1580	CATTGTTTATTTTTCTTTGGTCTTCTCCAAATCTTGCTCGGTTCCTGCTCATCTGTTGGATCTCTGAACTGAAGAACAAGACACCGCTGATAATCTTGTTCTGCAGGCTAGGCTTG
[illegible]	[illegible]

Trait&gene no.	Gene	Homoeologues	IWGSC gene ID (refseq1.1)	WGIN Promotome ID	orientation	ATG	Target Sequence	MYbaits	MYbaits coverage (%)	MYbaits ID	MYbaits sequence
T4-1	TaCERK1	ABD	TraesCS7A02G264400	7A-1826280-T4-1	forward	263,714,673	CGATGGAGGAGTGGGGAACCTTGGTGCTCCGGTCCCGGCCTTTAGATAGGGCTTGGGTTTGTAGGTCTATCGGCGGCGCTTCGGTGGG	18	74.4	7A-1826280-T4-1_0	CGATGGAGGAGTGGGGAAC
T4-1	TaCERK1		TraesCS7B02G162500	7B-1934340-T4-1	forward	223,175,071	GGCGGAGCTATGTGTAAGTCTGGGGGTGCTGTGGCCCCCCCAACTTTGGACCAAACTATGAAGATTTTTTTTGAGGAAAGGGCTTAGAT	11	51.3	7A-1826280-T4-1_64	GGTCTATCGGCGGCGCTTCGG
T4-1	TaCERK1		TraesCS7D02G265400	7D-2045100-T4-1	forward	246,812,310	GCACACTTATCTCGCTGGACACGAATCCGAAAGCGGAAGTCAGCAACCACCACTTATGCCGAGGGACAACACTCTCTCCAGGCATCACCT	9	39.8	7A-1826280-T4-1_128	CTCCAACACATCGATGTTCTCG
T4-10	TaMPK3	ABD	TraesCS4A02G106400	4A-1008960-T4-10	reverse	120,607,334	TCTCAAGCACAGCCAAGAAACTCTCAAAACCCAATCACAGGAGCGAGGCAACACGCACGCACGCACGCGCAACGGGATGAGGCTGAGG	7	41.6	7A-1826280-T4-1_192	CTTTGCGGTGGAGGATCTGGA
T4-10	TaMPK3		TraesCS4B02G197800	4B-1033300-T4-10	reverse	426,767,166	GTGACCAAGGCATATAATTATAGACAATTTATGCTGAAATTATCCTTGTGTAATTTGTGGATTAGCGGCAAGATAAACCTTTAGGCTAGGAAA	14	65.2	7A-1826280-T4-1_256	GTTTGTTCGTTCGGTTGCGTT
T4-10	TaMPK3		TraesCS4D02G198600	4D-1148960-T4-10	reverse	344,943,318	TCTCAAACACAGCCAAGAAACTCTCAAAACCCAATCACAGGAGGGAGGCAACACGCACGCACGCACGCGCAACGGGATGAGGCTGAGC	12	58.4	7A-1826280-T4-1_320	GGGATTTGTACAGGAAATCAT
T4-11	TaMPK6	ABD	TraesCS7A02G111300	7A-1834630-T4-11	reverse	68,094,141	CTCCGCCCGGATCGCGCGCGCGGGGGTGCGGGGAGGGGAGGGGAATTCGCTCCCGAGATTTGGTCGCTTTCCGCCTCTTGTTTGTTTAG	4	21.6	7A-1826280-T4-1_384	CGAAGATCTACGACCAATCCG
T4-11	TaMPK6		TraesCS7B02G009200	7B-1954300-T4-11	reverse	5,520,920	CTCCGCCCGGATCGCGCGCGTGGGGGGTGCGGGGAGGGGAGGGGAGGGGAGAGCCCGTGCGGTGGATTTCGCTCGCGAGATTTGGTCGCTTT	5	25.4	7A-1826280-T4-1_448	GCAGCCCGACTCCGCCGTTGC
T4-11	TaMPK6		TraesCS7D02G106400	7D-2040040-T4-11	reverse	64,277,473	CTCCGCCCGGATCGCGCGCGTGGGGAGGGGAGGGGAAAGCCCGTGCGGTGGATTTGGCTCGCGAGATTTGGTCGCTTTCCGCCTCTTGT	6	32.5	7A-1826280-T4-1_512	GACTCTTCAAAGACCTAACTG
T4-12	TaOCP3	ABD	TraesCS7A02G373500	7A-1770880-T4-12	forward	546,631,662	TGGGAAGTCATCATACGGTCACCGGAATAATTCGGGGTTATCGGTATTGTACCGGGACCACCGGAGGGGTTCCGGGGGTCCACCGAGAG	13	52.2	7A-1826280-T4-1_576	GGGTAATAGACTGAGGCTTTT
T4-12	TaOCP3		TraesCS7B02G253500	7B-1867300-T4-12	reverse	467,446,534	TTTTTCCGGAGGAGGAGGAGAGGTACAAGCGCTTATTAGATTTTTTTTTTAGGCTAACACGCACTTTACTTTATTGCTCAAAAATATGTAGA	18	77.6	7A-1826280-T4-1_640	AAAAGTACATCAGGCTCTCGT
T4-12	TaOCP3		TraesCS7D02G349600	7D-2004100-T4-12	reverse	449,460,701	TTCCACGAGCTCGTTGCGGATATTTCGCCGTCTTCTAACCCTCTCCGTGGGTTTATTGTCTGAATCCAAACTTTTTTTTTTTGAGGTGAGTCT	22	91.5	7A-1826280-T4-1_704	AAGCTGTACCTAAAGCCGCAT
T4-13	TaOPR3	ABD	TraesCS7A02G412400	7A-1801960-T4-13	forward	603,291,733	GGCCATTGCAGCCCAGCTCGCGGATGCGGAAGCTCCAAGCATGCGCGTCATTCAGCAACCCGGGGGAGCATGACATCACTGGCATTGTG	8	43.3	7A-1826280-T4-1_768	GCAAGAAAAGAAGAGAAAAC
T4-13	TaOPR3		TraesCS7B02G311600	7B-1860490-T4-13	forward	557,057,439	CATATAATCAACTCACGGATACTCGTGGTGACATTGGAGCATATAGGTGACATTAGAGTTGGTTGATGTATACCATATGGTGTTATTTAGTAC	7	29.6	7A-1826280-T4-1_832	CACTTGAGTTTTATTTTGAAT
T4-13	TaOPR3		TraesCS7D02G405500	7D-2008410-T4-13	forward	523,045,580	CATTCTTACCAAGGATAAATTACTGAAGCGACGATGGGTCGGTAGTTCATGATGTTGCTTTTGTGTTCATGACGAAACAATACAACATCTATT	6	25.9	7A-1826280-T4-1_896	TTGATCAAACAACGGCACCAA
T4-14	TaNH1	ABD	TraesCS3A02G105400	3A-0688550-T4-14	forward	69,290,751	CAGGGTTACAACTTCACCCGCTGGTTCCCCTCGCCTCTAGGTGCCTTCCGTCGCCAGCGAGCGAGGTGGGGGAACCCCAGTTTGGGTTGT	14	65.9	7A-1826280-T4-1_1152	ACGGCCGGGGGAGGTTCCGG
T4-14	TaNH1		TraesCS3B02G158300LC	3B-0744050-T4-14	forward	96,833,828	CATTTAGGCCCCAAATAAATGTTCATCTTAACCAGCTTGATTTTAGAAGTCGGTGAAAATTCATTTCAGGATTTTTGGAGTCCGTTTAGTATT	10	43.1	7A-1826280-T4-1_1216	ATTCTAATACTAATCTAATCCAG
T4-14	TaNH1		TraesCS3D02G107500	3D-0914040-T4-14	forward	61,072,417	ACATATGGTTTTGACCGAATAAGCGCCTCACGCAAATACAACATGCTACCAGGACAACTGCCACCGCTGGTTCCCCTTGGCTCTAGGTGCCT	12	58.4	7A-1826280-T4-1_1280	TAGTACTATACTACAATATTGCC
T4-15	TaBI-1	AB, D	TraesCS1D02G018500	1D-0240360-T4-15	forward	8,014,222	GCAGGCGCTGCCCAGCTTCCACTTGCCTCAGCACCAGCATCACCACCGGAGAAGGGTGGACAGGGAGAGGGAGAAGGAGAGGACGGT	7	28.5	7B-1934340-T4-1_448	AGAGGATTCTAATGCGTAGAT
T4-15	TaBI-1		TraesCS6A02G083200	6A-1573990-T4-15	forward	51,947,903	GCATCCATTTCTACAAGGCGGAGGGAGTACTATTTTATGCTCTCTCTGTGTAAGGTGCTTGAAAACGATAGACCAGTCGATGGGAACATGC	15	76.2	7B-1934340-T4-1_832	TTTAGGTATATAGGATACGGAA
T4-15	TaBI-1		TraesCS6B02G113900	6B-1671980-T4-15	reverse	96,404,412	CCCTGCGCTGCGCCTGCGTCTGTCGAATCGGAGCGGCCTCGCCTCGCACGCAAGGTTGTTCGTCCCTGTCCTGCGCTCGCGCTTTCGCGTC	17	82.6	7B-1934340-T4-1_896	AGTGCTGCTATTCGAATTGCAT
T4-16	TaLSD1	ABD	TraesCS7A02G310500	7A-1798270-T4-16	forward	439,575,548	TAAAGTGTCACTTATCTTAAACTCCCATTCCCTGTCTTTAATTCCCCCCAACCAAACACGCTGTCAAGTAAAAAATTAACTCTAAAATATTTCT	6	36	7B-1934340-T4-1_1024	GTTGTAGAGTGATACTGGTGC
T4-16	TaLSD1		TraesCS7B02G381100LC	7B-1864900-T4-16	forward	384,095,292	AACTTCTCGTAAAATCGGCGCTATGCCTTGATGGGGGCGGAGCTCCGCCATGTACGCCTCATTCGTGGCCTCCGCCACGATGCGCTTCCGA	15	72.9	7B-1934340-T4-1_1088	GTAGTTCCAGCTATATTAATGT
T4-16	TaLSD1		TraesCS7D02G307000	7D-1974840-T4-16	forward	388,963,723	CCTCGCCGATGTCGTTACCGCCGCCGCCGTCTTCATTGNNNNNNNNNNNNNNNNNNNNNNNNNNNNNNNNNNNNNNNNNNNNNNNNNN	14	71.1	7B-1934340-T4-1_1152	TGACACCACATATTTCTTTCTT
T4-17	TaEDR1	AABBD	TraesCS1A02G131600	1A-0075270A-T4-17	forward	176,449,614	TGCTATGATGGCTTGGAGAGTTATTAAGAGTCCTAATTCTCTTGTTACGAAAGTTCTCAAAGCCAAATATTTTCATAACTCTTCTATTTGGAAA	6	35.8	7B-1934340-T4-1_1216	TGTTGCTGATCCTGTGTGTTGT
T4-17	TaEDR1		TraesCS1A02G131600	1A-0124980B-T4-17	forward	176,451,097	AAATCTGGATGTAAAGCTCAACAAGCTTTAAACTCTATCTCCTTTCCTTTTGGAAAGTTGATCTCTGTACACTGTTTGGGTTGCAAATAAATA	12	65.2	7B-1934340-T4-1_1280	GTTGCCAAGAATTGCATAGTTA
T4-17	TaEDR1		TraesCS1B02G154400	1B-0075270C-T4-17	forward	256,903,624	CGAGCTCGGTCCTGCTGCATCGTGCGCGAGACGAGCTCTGTCCTGCGGCACTGGGCGCGAGACGAGCTGTGGACCGCCGTTGGGAGAG	10	50.8	7B-1934340-T4-1_1344	CATGTGAAGTATTGAGTCAATA
T4-17	TaEDR1		TraesCS1B02G154400	1B-0124980A-T4-17	forward	256,905,120	CAAGATATGCTTCATCAATCCCACCCGTAGCCGCCGCTGCCGCTCAGCACTCACTAATCGACACCACTTTTCTTAAACCCCCCTCCCTTTCCG	12	60.5	7B-1934340-T4-1_1408	AATCGATCACTTGTTCCTGAAA
T4-17	TaEDR1		TraesCS1D02G136400	1D-0075270B-T4-17	forward	181,626,972	GTTAGTGTAGTTTGTTGAGATAGGCCTTGAAATCTAGCATAATTAGTTGCCAAAACTAATTAGCAAAAAGTCATGTAAACACTTAAAAAGTT	22	89.4	7B-1934340-T4-1_1472	CTACCTTCCTCTGGAATCACAA
T4-18	Ta6-SFT	A, AD	TraesCS4A02G485400	4A-0939290-T4-18	forward	739,064,972	AATACACATGTGAAATGTCTCTCAGACTGGATGTGATATATCTATGTGTTCCTTATGGGCAGATGAGATTAAAACTAGTCGATTGAGTGCATG	18	84.2	7D-2045100-T4-1_1024	GGTTTCATCTCCATATTGATATG
T4-18	Ta6-SFT		TraesCS7A02G009800	7A-1820410-T4-18	reverse	4,433,162	CGCAGCGCCGCTCGTGAACTCCAAGAGAGTTTGGTAGATTGTGGGTGCTGGATGGGTGTGGGTTCTGGAGCCGGCGAGCTAGCGCCATT	24	92.5	7D-2045100-T4-1_1088	CAATTCTGCATGTATGGTAACT
T4-18	Ta6-SFT		TraesCS7D02G009400	7D-2044770-T4-18	reverse	4,518,811	CGCAGCGCCGCTCGTGAACTCCAAGAGAGTTTGGTAGATTGTGGGTGCTCGATGGGTGTGGGTTCTGGAGCCGGCGAGCTAGCGCCATT	15	78.4	7D-2045100-T4-1_1152	TTTGTGTACTATTAATTTTGAG
T4-19	Ta1-SST	A, AD	TraesCS4A02G486000	4A-0991190-T4-19	forward	739,416,696	TGAATCGCAACCACGTACTAAAAGGAACCGCGGCGGGAGGATCTGCCGATCTTATGGGGCCATCTTGTTTGTAGATATTTTTTCCAAGTAA	4	28.2	7D-2045100-T4-1_1216	TGTGGTTACAATGTTGACATTC
T4-19	Ta1-SST		TraesCS7A02G009100	7A-1820640-T4-19	reverse	3,986,563	GGCGCCGCCGCTGCTGGCCCGATGATCCCGAGGTAACTCGGTAGATTCTTTTCTGACCGATGGATGGGTGGTGGCTTCAGTGGGGGAGC	3	17.9	7D-2045100-T4-1_1280	AGTTGCCATGAATTGCATAGTT
T4-19	Ta1-SST		TraesCS7D02G008700	7D-2014520-T4-19	reverse	4,200,800	GTCGCTGGCCTTGCGGCCCCCGCCGACGGCGTAGAGGTCGATGCACTCGTACTCGCCGGTGCCCTTGGGGCCGCGGTACATGTACCCCGC	8	38.8	7D-2045100-T4-1_1344	TCATGAGAAGTATTGAGTCAAT
T4-2	TaCERK2	ABD	TraesCS6A02G417900	6A-1523250-T4-2	forward	615,973,140	TACCCAAGACGATGATATACCTAAATATATAACTGTATATATGACTTCGTATTTTATTATGTAAAAAAGAAAGTAATGCATGAATAAGAGAGAA	19	90.6	7D-2045100-T4-1_1408	CAATTGATCACGTGTTCCTGAA
T4-2	TaCERK2		TraesCS6B02G471100	6B-1612290-T4-2	reverse	719,409,850	TAATATTGTACTGGCTGGAACAAAAAGAATAATAGTTCAGAAATAAGAAGGCACATCCTGTTGTGATTAATATCAGTCAAGGCCTGGTGAAC	20	81.9	7D-2045100-T4-1_1472	GCTACCTTCCTCTGAAATCACA
T4-2	TaCERK2		TraesCS6D02G403200	6D-1699670-T4-2	forward	471,251,096	GATGGTGCCACCGGGAGCGGCATCGTGTATATCCCTGCCAAAGGTACGCGAGGCTTCGATCGTAGCTCCTCTCTCTATGGTGCACCTCACA	16	82.6	7D-2045100-T4-1_1580	TAAGTATCCTCATCTTGTGCAG
T4-20	Ta1-FFT	A, AD	TraesCS4A02G485900	4A-0939360-T4-20	forward	739,310,325	GCCCAGTCAGCCAGCATGCGACGAGTGAACCACATGTGAGCTCCGTCGATTCCATTCCATGGCTGTGCTTCCTCTGCCGTATTGTGGCAGC	14	69.2	6A-1523250-T4-2_0	TACCCAAGACGATGATATACCT
T4-20	Ta1-FFT		TraesCS7A02G009200	7A-1820630-T4-20	reverse	4,017,160	GGCGCCGCCGGGCCGCTCGCCAGGAGAATCCCGTGGGAATTTGGTGGGTTCACTCTCACCGACGGATGGGTGGATGGTGGGTTCAGTG	18	79.8	6A-1523250-T4-2_128	AATGGACCATTAATTGGTGATT
T4-20	Ta1-FFT		TraesCS7D02G008800	7D-2019550-T4-20	reverse	4,270,668	GGCGCCGGCGCTCGCCACGAGAATCCCGTGGGAATTTGGTAGATATTCTTTCTCGCCGACGGATGGGTGCTGGGTTCAGTGGGGGGAGC	17	82.6	6A-1523250-T4-2_192	ATAGAATCAGTATTTGCTGGAA
T4-21	Ta4-CL1	ABD	TraesCS6A02G151700	6A-1557090-T4-21	reverse	135,562,668	CCGCCGGAGCCCCGCCGCCGCCTTCCGGGAGAGCGACTCCACCTCGGCGTAGGTGTAGGAGGCGCCCGTCTGGCCGTCGATGATGCAGC	14	69.2	6A-1523250-T4-2_384	TGATTTAAACCTATCTTATCTCT
T4-21	Ta4-CL1		TraesCS6B02G179900	6B-1667010-T4-21	forward	200,010,196	CGCACCACCGTGCCGCACGACCCGGACTTGACCTTGAACGGCTCCTTGGCGAACGCCAGGCACATGGCCAGCACCGGCCCGGCCTCCGT	5	28.7	6A-1523250-T4-2_448	ACTTTGTTTTATACTGGAAAGA
T4-21	Ta4-CL1		TraesCS6D02G141700	6D-1748090-T4-21	reverse	111,062,509	CTGAAAATCACCACCATTTCATCAGAACAAGAAAAAAAAAGGTTCTAGCGATCAGGGTGCAATTCGAAACCTCTGACTTTTGGCGCCAAA	9	50.4	6A-1523250-T4-2_512	GGATTAGGTTACGACCCTAATA
T4-22	TaDMR6	ABD	TraesCS2A02G395000	2A-0276380-T4-22	reverse	646,627,800	TTGGGGCCTAGCCGAGGCTCAGTCTCGCGCGGCGGTAGAAGAAGGGGGATGAGCGAGGGGGATCGCCATATATCGAGAGTACCCGAAG	14	65.9	6A-1523250-T4-2_576	ACGCATTGCTCACAAGTACATC
T4-22	TaDMR6		TraesCS2B02G413000	2B-0427630-T4-22	reverse	588,730,411	TTGGGGCCTAGCCGAGGCTCAGTCTCGCGCGGCGGTAGAAGAAGGGGGAGGAGCGAGGGGGATCGCCATATATCGAGTACCCTGTACC	12	61.6	6A-1523250-T4-2_640	ATTATAAATATCAATTTCAAGA
T4-22	TaDMR6		TraesCS2D02G392800	2D-0502570-T4-22	forward	500,687,830	CATATTAGGGATTCATGCCGCGCACGATCAGAAAGAACAACTCTGAGTAGATTTGCATGACCCTGTTACAAAATTAAAAAGAAATAGTCCTA	6	32.3	6A-1523250-T4-2_704	TACCATGTTGTTACAGTGGTCT
T4-23	PAD4	ABD	TraesCS4A02G216100	4A-0978150-T4-23	reverse	514,432,965	CGCGGCGGCGGCCGCGCACCGGTCCCAGGCGCGCGCCAGCAGCGGCGACGAGGCCAGCAGCGCGCCGAGGACATGGCTGGTCTCGAA	5	28.7	6A-1523250-T4-2_768	ATGTTGTGGAGAGTTGATCAC
T4-23	PAD4		TraesCS4B02G100100	4B-1105190-T4-23	forward	105,956,707	TAAATATGAACAAGAAAATAAATAATAATGTTTTGCTATTGCCTCTAAGTTCCAACAAGTTCAAGGTGGCTCTAATGCCAACTGTTGGCCGGA	11	48.8	6A-1523250-T4-2_832	CCCAATGGAAGTTACCTTCCTC
T4-23	PAD4		TraesCS4D02G096400	4D-1175370-T4-23-2	forward	72,908,328	GATTCCTCTCGTGTTTTCCCTCGTGTTTCCCCTCGTATTCTTCGTGTTCTTCGTAGGATCCCCTCCATTTCATGAAAGATCGGGCATCTAGGGT	6	35.8	6A-1523250-T4-2_896	TCTTATCCACTTAATTTTCTTGC
T4-24	TaBx1	ABD	TraesCS4A02G097400	4A-1004820-T4-24	forward	108,369,503	TATCCGTAAGGACCCAGACCTAGGTCTAGCGTCCAAGATATAGCTGGGTTCCTCCGTGTTACAGGCAAATCCTTTAGGACTGTGGTTTAACC	10	49.6	6A-1523250-T4-2_960	GTATCCCTCATCTTGTGCAGTA
T4-24	TaBx1		TraesCS4B02G207100	4B-1041080-T4-24	reverse	440,927,168	TTCGCTAGCTGACTGAGAAACCTTCCTGTGTAGAGATTTATTGTTTGTGAATGATGCATGAGTTGCGCGTCTGGACGCGTTTATAGTATCGG	10	53.6	6A-1523250-T4-2_1024	TTAAGGTTTTTTGGGGAAGAA
T4-24	TaBx1		TraesCS4D02G207900	4D-2109110-T4-24	reverse	357,531,376	TTCGCTAGCTGAGACAGAAACCTTCTTGTGTATTGGAGATTTATTGTTTGAGAACGATGCATGAGTTGCGGTCTGCACGCGTTTATAGTATC	4	17.8	6A-1523250-T4-2_1088	TTATTCATATTCAACTCTCTTCG
T4-25	TaBx2	ABD	TraesCS4A02G097500	4A-1004810-T4-25	forward	108,373,896	AACTATGGTAAAAATACAAAAACAAATACTGCATAGCCCTTTGTTGACACTGTTTGGAAGTCCACTTCTTACAGATAATTGCATCGGTGTGA	18	77.1	6A-1523250-T4-2_1216	AGGCGAAACAGAATACACTGC
T4-25	TaBx2		TraesCS4B02G207000	4B-1041090-T4-25	reverse	440,923,289	GGCAGAATGCTAAAAGAGAGCTAGCTATCGTTAGTTCTTACTGGCTGTGAGGCTTAGCTTCTCCATCGGTGCGTTCATTTATAGCCCAAGAT	13	57.6	6A-1523250-T4-2_1408	CCTATGAGCAACTATATTAGC
T4-25	TaBx2		TraesCS4D02G207800	4D-2109100A-T4-25	reverse	357,527,442	GGCAACAATGCTAGCTATCGTTCTTAGTTCTTACTGGCTGTGAGGCTTAGCTTCTCCATCGGTGCGTTCATTTATAGCCCAAGATCGATGCAC	18	74.4	6A-1523250-T4-2_1472	ATTATGAGTAATAGTTTGAGAG
T4-26	TaMLO	A, BD	TraesCS4B02G322700	4B-1056380-T4-26	reverse	613,160,737	CGGTCCCCGAGCGCAACCTCTTTCCTTGTCCGGAGACCCAGCCGCCGGTCGTCTGAGCCAACGAGGTACCTACCTGGCCGGGTATCTATTC	12	67.1	6A-1523250-T4-2_1580	TCCAATTTTCTTCTCTTTTTTGT
T4-26	TaMLO		TraesCS4D02G319100	4D-1124700-T4-26	reverse	483,068,315	GGCTCCCCGCCGCCCGTCCTGAGCACAACCTCTTTCCTTGGCCGGAGCGAACGGCGGCCACCTCAGCCGGACAGGCGCGCGCCCGAGCC	13	67.5	6B-1612290-T4-2_0	TAATATTGTACTGGCTGGAACA
T4-26	TaMLO		TraesCS5A02G494800	5A-1214150-T4-26	reverse	663,218,535	CCGCCCGTCTTTAGCGCAACCTCTTTCCTTGGCCGGAGCAGGGCAGGGCGCGCACCCAAGTCCAACCGCCGATCGTCTGAGCCAACGAG	13	68.7	6B-1612290-T4-2_64	GATTAATATCAGTCAAGGCCTC
T4-28	TaBLN5	B no AD	TraesCS5B02G544800	5B-1330970-T4-28	reverse	697,347,287	GTTGTGCTTCTTCAGTAAGCTGTGTGTTTTTTGTACTACTGATATGCTGCACTTATACAAAAAAGGAGAACGGGTGAGCTGCAAGCGCCTTC	22	89.4	6B-1612290-T4-2_128	TCAAGGTTATCTAACTTTCTCTA
T4-29	TaCHI26	ABD	TraesCS7A02G548000	7A-1797990-T4-29	forward	722,763,923	GCCAGGCCCAACATGTGAGGCGGCGACACTCTGCCAGCCATGTGTGTTCTCATACAAAAGTAATTGCAGATGCACCAAGTAGGAGAAAAT	18	77.6	6B-1612290-T4-2_192	GGTTATACTTGAAGTTTCTCAA
T4-29	TaCHI26		TraesCS7B02G471500	7B-1872660-T4-29	reverse	727,608,280	CGTGCGTGGCAACGAACTGCTATCTTGCGGTCCAAGTGTGGTGGTGGTGATGGTGGCGTGTGTGTGTGTGGCAGGAGCGGTGGGGAGTC	20	87.3	6B-1612290-T4-2_256	CCACAATATTTCCAGGCTAAAT
T4-29	TaCHI26		TraesCS7D02G534700	7D-1967790-T4-29	forward	627,196,069	TTAAATTTATAATGGGCTGGTAAGTGGTGCGGCCCTTTATCTCTTACTATTTTTACATTTATATATTTCTTTGTATGTGAAATAAAATCAAGACAT	14	61.1	6B-1612290-T4-2_320	AATCCACACATATCCTTCTAGC
T4-3	TaCEBiP	BD	TraesCS4B02G329500	4B-2088710-T4-3	forward	620,056,385	AGATTTTTTAACCCCTGAAGCTTATTGCCAGCTCCAGTAAAACAGTTCCAGACACGATCAGAACTAAAATCATGGTGAGCGGGTTGGATCT	15	76.2	6B-1612290-T4-2_384	GTGGTGAAATTAGGACACGCA
T4-3	TaCEBiP	no A	TraesCS4D02G326400	4D-1131020-T4-3	reverse	486,183,136	GGTGAGAGCGAGGGGGGAGTGGGAATGCGAGGCGTTTGACTGCGGCGTTGACGCGGGTGGAGGAGGATGAGATGGTGCTCGGCAGC	16	73.4	6B-1612290-T4-2_576	TTTTTTCCTGTACCGAAGAGTT
T4-30	TaGER4c	A, B, U	TraesCS4B02G378500	4B-1062830-T4-30	reverse	660,456,329	GTCTTATGAGTTATAACATACAAAAAAGTAGTACTAAAAAGAACAACTTAATTATCCACAAATTACATAAAGATGTAATGTAATCTTTGTTACA	15	76.2	6B-1612290-T4-2_640	TAGTATCCAGTTTTTCTTCCCCA
T4-30	TaGER4c		TraesCSU02G128900	U-1151330-T4-30A	reverse	110,339,313	GGCGGGTGGGTGGTGGCGGCGCCGGCCGGAGGGAAGGAGAAACCAAACCAAAAGCGGTGGTTTTGGAGAGTTTGGGCCGGGCCG	20	81.9	6B-1612290-T4-2_704	GAAGTATGGCTATATTGCACAA
T4-30	TaGER4c		TraesCS5A02G545100	5A-1246480-T4-30-2	forward	700,364,469	ACTTTCTGTTATTGTTTAACTTATACATTAATTAAAGGGTGCCAACATGTATCATAATGAGTACTCTCTCCGTCCCAAAATAAGTGTCTCAACTC	17	77.2	6B-1612290-T4-2_768	AACATGCATGACTAAGAAAAT
T4-31	TaWRKY	ABD	TraesCS6A02G146900	6A-1558070-T4-31	reverse	126,346,256	CTCGCCCAGGCGGCGGTTCTCCTCGCTCACACGCTGTAGCTCCGACTCAAGAACCGCAACCTATACACAGACACAAACATTCAGTCGCAG	14	69.2	6B-1612290-T4-2_832	CTGATGATTTAAGAGGACGGT
T4-31	TaWRKY		TraesCS6B02G175100	6B-1643690-T4-31	reverse	190,124,294	TGGTCGACGACGGATTGATCGTCTCTCAAACACCAAGCTCTACTGTAATGGAATGGACGCTGTTCCTTCCGGTTCGAGGCAACGCGCGCA	10	56.2	6B-1612290-T4-2_896	AATTAGGAAGATGTGATCAAC
T4-31	TaWRKY		TraesCS6D02G136200	6D-1735290-T4-31	reverse	104,455,189	CGGTCGAGGATGGATGTAGAGTTCGCTTCCGGTTCGAGGTAACGCGCGGGAGAGAAGAGAAGGGAGAAGAGAAGGTTGAGATGGAGA	12	64.2	6B-1612290-T4-2_960	GACATTCAAAAAGACCACTGT
T4-32	TaWRKY	ABD	TraesCS7A02G508800	7A-1779770-T4-32	reverse	696,623,467	GTCTTTCTTAAATTCGCAGTAGCTCAAGAATGAAAGAAGTCGAAGAAGAAGAAGACCGGGAAGCTAGATGGGCAGCTTGGTAGAGAAGT	7	39.5	6B-1612290-T4-2_1024	TTCAATTTGTTTCTTGAAATTG
T4-32	TaWRKY		TraesCS7B02G418400	7B-1893610A-T4-32	forward	686,849,911	AGACAATTTTATCAGTTGTTTGTTTAACTCAAGAAAAGCATAATTATACAAATAAAAGTGCATTTGCGACACATTTATGAAAATAATAAAGAT	18	77.6	6B-1612290-T4-2_1088	AGTATCTGGAACATGTACTTGT
T4-32	TaWRKY		TraesCS7B02G418500	7B-2140600B-T4-32	reverse	686,888,423	GTGTCTTCCTTGAATTCGCAGTAGCTCAAGAATGGGAAACGAATCTACGACGGCCGGGGAAGAAGTCGAGGAAGACCGGGAAGCTGGA	6	38.5	6B-1612290-T4-2_1152	ATGCAGGTCGCTATTAGGGTC
T4-32	TaWRKY		TraesCS7D02G497400	7D-2140600-T4-32	forward	603,982,571	GGTCCAGGCCCAGTGTATATCTCCTGGCAGAAATCGAAATCGTAGGTTTTGACAGGACATGTGTGGGAACCGAGCAGAAATAGCGTATTTC	9	53.6	6B-1612290-T4-2_1216	ATATCGGGTAGTCCTTCCAATA
T4-33	Lr34	A, D	TraesCS4A02G384800	4A-0968550-T4-33	forward	662,278,659	GTGTGTGGCGTTGTTCGGGTGCGTCGACGAGGAGGCAGGCAGGAGGTGGAGGCCGACGCATGCGCTATCGCCACGACCTCGCTCTACAT	20	77.4	6B-1612290-T4-2_1280	TTAAGCTTTCCATACTTAAATAC
T4-33	Lr34	no B	TraesCS7D02G080300	7D-2025300-T4-33	reverse	47,424,077	CTCAAGATGGGTGAGTTAAATCGACGCCCTTTGCTATTGCCTAGCCGTACTCTAAGCAGTCATTTCCTTCTCTTTAGAGTACATATATAAGGT	14	65.9	6B-1612290-T4-2_1344	AAAGTAATTTCATGCAGAAATA
T4-34	MLOC_39318	AD	TraesCS4A02G296300	4A-0979890-T4-34	forward	596,523,171	TTGCTTTATGTCCTATATGTTTCACCGGCGAAATATGTGTCGGTGAAACATATGGGAAGAAGGAAAATAGATGCAACCCGGGGAAAATATGTC	17	66.1	6D-1699670-T4-2_128	ATTGATTGATATTGATCTACGTT
T4-34	MLOC_39318	no B	TraesCS4D02G015800	4D-1174080-T4-34	forward	7,035,353	TAAGAGCATCTTCAACAGACGTCCAAAAAATGTCACACGCTAAAAACCATCATTTTTGTGCGCCGGAGACAAAAAACAACGCTCCGACAG	21	91.1	6D-1699670-T4-2_192	ATATATATCGTATTAACTTGG
T4-35	MLOC_51041	AD	TraesCS1A02G295900	1A-0031050-T4-35	forward	490,893,890	ACACTGTCCTCTCGAAGCCAGCCGAACGTTTAACAGAAACCATGACTTGTTTCTATGCTCCGATCATCATGTTCAGGCCTCTTCGTTCGTGTC	14	69.2	6D-1699670-T4-2_256	CAACTATATTCAAACTACTTGG
T4-35	MLOC_51041	no B	TraesCS1D02G296300	1D-0187950-T4-35	reverse	394,409,444	AGTAGGTTTCTTCCTTCCCTTCCCTTCACGAACTCTGCACGCACGCAAGCCTCGACGTGCACGAGCTTGGACTTCTTGGACTTATCAGCTAC	13	65.4	6D-1699670-T4-2_320	TATAATGTATTTGTGCATGGAA
T4-36	MLOC_53924	AB	TraesCS5A02G236700	5A-1186440-T4-36	forward	452,122,970	TATTTCTTTAAAAAATTCATGTTTCTTTTGGTTATGAACATTTTTAAACATCTAAGATTTCTACCAAGACACCAAGCTCCCTAGATTTGAAGCC	16	80.3	6D-1699670-T4-2_448	TTTGTTTTATACTGGAAAGACT
T4-36	MLOC_53924	no D	TraesCS5B02G236300	5B-1307890-T4-36	reverse	417,343,002	CGAACGATTCCCCCGCCGGCGGGTGCAAGTGTCACGATCAGTTTTGCTTGCTTTTTCGCCGGCAAGTTGCTTCTAGCCCTCCGTGGTTTCC	11	63.3	6D-1699670-T4-2_512	ATGGATTAGGTTACGACCCTA
T4-37	MLOC_54592	AD	TraesCS7A02G049400	7A-1837360-T4-37	reverse	22,976,922	TGCTGCTGCTGCTGCTGGTGTGGTGTCTATGAGCTGCTCAAGGTGGTCTCAGCTTTGCACGTAGTAGATGGCTTGTGTTTTGGTTCCTCAGT	15	75.1	6D-1699670-T4-2_576	TGACACATTGCTCACAAGTAC
T4-37	MLOC_54592	no B	TraesCS7D02G044500	7D-2064320-T4-37	reverse	22,624,779	AGCAGAGACTCTTCCGTTAGAACTTACTCTACAAGGCAAAATGTTTGGTGGGTTAATTCTACTTTCTACATTTGAACTGTCTACTTTACCGG	16	70.1	6D-1699670-T4-2_640	AAATTGTAAATATCAATTTCAA
T4-38	MLOC_55516	ABD	TraesCS3A02G125200	3A-0681650-T4-38	reverse	100,882,827	GGCTGATCGGCCGTGGCAGCAAAAGTGCGAGCGGAGTCTGTCAAGTTGGGAGGACTTGGAAGCCGTGCAATGGCAACCGTAATGTATAT	17	73.9	6D-1699670-T4-2_704	GATACCATGTTGTTACAGTGGT
T4-38	MLOC_55516		TraesCS3B02G144800	3B-0752330-T4-38	forward	133,200,250	GTGATGGGGGTTACTTTGGGAACATGATGAACTAGGGTTTCAGCAGCCTAACTTCTAAGGGGACGAGACGCTCCACGGTCATAGGTTCTC	14	69.2	6D-1699670-T4-2_832	ATCCCGATGGAAGTTACCGTC
T4-38	MLOC_55516		TraesCS3D02G126200	3D-0915560-T4-38	reverse	84,498,196	GGCTGATCGGCTATTGTAGCAGAAGCACTGGTGGTGGAGTCAGTCAAACTGGGAAGAACAGTGACACCGGCAAGATAGCACGATGTGTG	9	47.1	6D-1699670-T4-2_960	AAGTATCCCTCATCTTGTGCAG
T4-39-1	MLOC_57236	ABD	TraesCS2A02G259500	2A-0284830-T4-39-1	reverse	407,742,618	GGTTGGTCGGTCGGCAAGAACTCAGAGCACTACAATGATTAAGCAGGCCCAAAGCTGATGATCAAGGAGGTATGGGGTGTGGTGAGAAC	12	64.9	6D-1699670-T4-2_1024	CGTTAAGTTTTTTTGGGGAGG
T4-39-2	MLOC_57236		TraesCS2A02G259500	2A-0284830-T4-39-2	reverse	407,740,562	TCCAATGTTAGACCAAACACAGATTTCTTTCTTTTCTTTTATCATAATTTGAAAGCACAAACTAATAACATGACGCTTAATTAGATGTGCTTTA	15	76.2	6D-1699670-T4-2_1216	AGGCGAAACAGAATGCACTGC
T4-39-1	MLOC_57236		TraesCS2B02G282400	2B-0425860-T4-39-2	forward	390,923,551	AGAGCTCGGGCATAACACTTTTCTGAAGGTGACAGCCGCTTTGCGTATGTTGGCATACGGTATCCCGGCTGATCTAGTTGATGATCATTTGC	16	82.6	6D-1699670-T4-2_1408	CTATGAGCAACTATATTAGCC
T4-39-3	MLOC_57236		TraesCS2B02G282400	2B-0425860-T4-39	forward	390,923,316	TGCATATCAATAAAAAACCGAAGCACGGTGGTTCGGTTATGGGTTGGGAGAAAATTTGGAGAGATAGGATTGATGCCCATAACAGATTGAT	3	15.7	6D-1699670-T4-2_1472	TTATGAGTAATAGTTTGAGAG
T4-39	MLOC_57236		TraesCS2D02G264200	2D-0563460-T4-39	forward	323,074,705	GGAGTAGTTAAGCGGCAGGACAGCTTGCATAATGCATATGCTAAACAAACTAAGATGCATGTGAATGAAGGCCAGCAGTCTGCGGATTGC	12	68.2	6D-1699670-T4-2_1580	TCCAATTTTCTTCTCTTTTTTGT
T4-4	STB6	A	TraesCS3A02G049500	3A-0682540-T4-4	forward	26,201,675	ATAGCTGATTTGGAGGCCCGTTGCTATTTTCATAGCCCGCTATTAAAACATTGATTTTTTTAGAAGAGTCAAAGTCTTTTGGTCCTCCAAG	17	76	4B-2088710-T4-3_0	AGATTTTTTAACCCCTGAAGCT
T4-4	STB6	paralogue	TraesCS3B02G056100	3B-0742130-T4-4	forward	29,618,482	TTTTTGACCGTGGATGGGCACAGCAATTGTTAGGCCGTGTGAGACTAGTATTTTTTTTTTTTGTCTCAAGTCTTCCCTTGCCCCGTCAATTGC	17	77.2	4B-2088710-T4-3_256	GCCCGGCATGCCGCCTTCGTC
T4-4	STB6	paralogue	TraesCS3D02G049300	3D-0902020-T4-4	forward	18,849,889	CTCCTCCCTCTGCACGTAAGTTCACCCGCCGCAAATCATCTCTTTAATCCATTTCCTGGAATGCTAGCTAGGACGCTTGGAAACCAGGCAAT	22	89.4	4B-2088710-T4-3_384	GCAATCTGGCTTTCATTGGTTC
T4-40	MLOC_59473	ABD	TraesCS1A02G186800	1A-0049460-T4-40	reverse	337,776,922	TGTTGGTGTGGTGTCTGTCAACTCAAGGTTTCACTGAACTTTGCACGTACGTAGATTGTATTTTGGTGCCTCTGTTTTAAGACTCCGGAGGA	3	17.9	4B-2088710-T4-3_448	TCGCCTTCATGTTCTACTAGCA
T4-40	MLOC_59473		TraesCS1B02G194700	1B-0122280-T4-40	reverse	349,596,162	TGCTGGTGTCTTGATCCGTTCTTCTGTGGTTGGTGGCTTGGTGCTTGACTTGACTGTTAGAGATGCAGGGGAGGAGCGGTGTATATATGGC	4	20.9	4B-2088710-T4-3_512	cagacagacagacagacaAAGTAG
T4-40	MLOC_59473		TraesCS1D02G189800	1D-0191530-T4-40	forward	262,036,257	AATAAATGTGTCTTAACTCAATGAAATTAATTAGTGGCCGTTGCTGCAACTTGACATGAACATCATGCATGTCACTCATCTGATCATTCACTCA	10	50.1	4B-2088710-T4-3_576	GCTTGAAATCTGTTTGTGGTC
T4-41	MLOC_59596	A, B	TraesCS4B02G346900	4B-1038150-T4-41	forward	640,532,917	GAGATCTGCTGGAGATGCTCTTTGGCCTTATACAATGCTAAGAGAGATGCTTAAAAAATAAACCGGAATTTTTTAAAAGCATCGTGCATATT	7	46.1	4B-2088710-T4-3_768	ACCCTGCCGTATGATATGGTCT
T4-41	MLOC_59596	no D	TraesCS5A02G515600	5A-1203500-T4-41	forward	679,134,425	CCTCCTCGCCAGAGGAGATGGCGATCTCCTCCTTGCTGGAGGAGCCGGCCGCCGTGGACGCCGAGGGGGGCGGAGAGCGCGGGCGGC	12	61.6	4B-2088710-T4-3_832	GTTGACCTGTAGACTGTTAGC
T4-42	MLOC_61430	ABD	TraesCS4A02G017000	4A-1011820-T4-42	reverse	11,600,220	AAAAATCAAGCTTTGAATTTGTGTTTTGTATGTGTTAGCAGATGCACTTTTTTCAAAATAACCATGATGCAGTTTTCGGTATGTGTTCCAGTG	15	69.6	4B-2088710-T4-3_1024	ACTTGTGGGCGTGGAATTCCA
T4-42	MLOC_61430		TraesCS4B02G287100	4B-1070420-T4-42	reverse	570,348,347	GGAGGAGATAGCGCTTCCTTCCCTCGGTGCTCTGGTTCTTGGACTCCTCTGCTGCTTTGGACGCGTTGGTCTTGGGTGTGGGAGTGGGTT	12	64.9	4B-2088710-T4-3_1152	CTTGTACCTTCTCCGAATTGGC
T4-42	MLOC_61430		TraesCS4D02G285800	4D-1152920-T4-42	reverse	456,129,195	GGAGGAGATGGTGCTTCCTTTCCTCGGTGCTCCGGTTCTTGGACTCCTCTGCTGCTTTGGACGCGTTGGGTTTGGGCGTGGGAGTGGGTT	12	68.2	4B-2088710-T4-3_1216	AACTTACAAAATCCGTAGACTT
T4-43	MLOC_64975	ABD	TraesCS3A02G270800	3A-0651840-T4-43	forward	498,229,179	GTTGTGATAGGCTTGCTCCAAGCTCACCTACCCTTCTTCCATGTTGAACAACTTCGTTTTCTTTTCTTGAAACAGGCTCTTTTGACGAAACTT	9	50.4	4B-2088710-T4-3_1280	ATTAATAGGCTGGAACTCACAC
T4-43	MLOC_64975		TraesCS3B02G304600	3B-0722470-T4-43	forward	489,351,968	CGCTCTCGGGCGCGTGCTCGCCGTCGCCGGTGCAGGTGACCATCTTCTACGTCTCGCTCTACCTAGTGGCGCTGGCGGAGGCCGGGCACA	1	7.1	4B-2088710-T4-3_1344	ATTTTGAAAGAATTTCGGAGG
T4-43	MLOC_64975		TraesCS3D02G270500	3D-2135880-T4-43	forward	375,187,276	TTTCATTTAAAGTTGGGATCTCAGCTTTCACCTTGTATGTATCCCTTGCATATGTACGATTGGGTACTCTCCGATATGCATAGAGACTGGGTTC	4	24.9	4B-2088710-T4-3_1408	ATATAAGAAGATTTCTCGAGCT
T4-44	MLOC_67438	ABD	TraesCS5A02G453200	5A-1198810-T4-44	forward	634,622,472	AATCATCGACCGTTCAAATGTTCAGAGCTAAGGTTTGTATATTTTTCGCAAAAAGAGAGCCAAGTTTTCTTTTTCTGCAGCGAAAGAGAGG	19	88	4B-2088710-T4-3_1472	TAGTAACCCATGTCGGCTAAGC
T4-44	MLOC_67438		TraesCS5B02G462200	5B-1293440-T4-44	forward	637,482,724	CCGACGCGCCGGCCGCCGAGCTCCGCCGCGCGCCCGAAGAAGCAGCCCCCCGCGGCCCCTGTTCCAGGCGAGGTTAAACGAGATCCC	10	54.4	4D-1131020-T4-3_128	GTTGTGCGGGGCCCTGCCCCC
T4-44	MLOC_67438		TraesCS5D02G464000	5D-1450500-T4-44	forward	507,435,021	ACGCGTTTTCTGTTTTTTTTCCATGAGAGTCACGGTTTTGCTTCCACAAGAAGCACGGTTGTGATTTCGCGAGAGGCACAGGCGTGCCTCT	15	69.6	4D-1131020-T4-3_192	TGCTTAGCCGACATGTGATCC
T4-45	MLOC_69708	ABD	TraesCS7A02G537200	7A-0615060-T4-45	forward	714,660,625	CCGCACGTTCGCTTCGGCCGGCGTTCTTTGCGGCGTTGGACACCGGCAGCAACTTCTTCAGGGTGTCCTGCGAGTGTAAACAGTGTGCG	21	88.9	4D-1131020-T4-3_256	TGTAGGAGTACAGCTTAAACC
T4-45	MLOC_69708		TraesCS7B02G766300LC	7B-1870160-T4-45	reverse	716,025,080	GGCGGCGAGGAAGTCAAAGTCTTGTGTGGTTTACCCCGGCGCGACGTCAACGGTGGGTGGTGGATGGCCGGCGGGGGCAGGGGAGCC	9	40.5	4D-1131020-T4-3_320	TACGATGATACACCATGCCGCT
T4-45	MLOC_69708		TraesCS7D02G524300	7D-2008440-T4-45	reverse	620,258,425	GGTGGGCAGAGTAGGCGTGCGTGCAGGGACGGCAATTGGACGGAGGACGGTTGGTGCACGGGGCTTAAGAGGAAGAGGGGACGCGC	20	85.2	4D-1131020-T4-3_384	TTACGTTTATAAGAACACCTCT
T4-46	AK358598	ABD	TraesCS5A02G226400	5A-1215280-T4-46	forward	442,281,937	TATGCATGGTTCGGTGTTTCTGTATTCCGTGAAGGATTATGGATAACTGGTGTAGTCTTATACTCTGCAGTCTGCACTCTGCACCTTAGCTATC	17	82.6	4D-1131020-T4-3_448	CGGTGAATTCAGAAGGAAAA
T4-46	AK358598		TraesCS5B02G224800	5B-1286670-T4-46	forward	400,449,191	ACAAGAAGAGGAAGATGAGCTTCAGGAGGAGCTACCAGAGGGACAGCTATCACAAGAGGAAGATGAGCTTCAAGACGAGCTGCCAGA	14	68.6	4D-1131020-T4-3_512	CAGCTCAGTGAAAAACAAGA
T4-46	AK358598		TraesCS5D02G233700	5D-1403090-T4-46	forward	341,037,168	GATGGGAAATGGTACAATGTTCCAGCCAAGCCTCACACACTGGTGATCAACTTGGGAGACTGCCTGGAGGTAGGCACCTACACATACAAG	16	76.7	4D-1131020-T4-3_576	TTATCAGATGATTGCTCCGTTG
T4-47	MLOC_37050	ABD	TraesCS3A02G129000	3A-0689800-T4-47	reverse	106,440,786	GGACGGGCGCAGGCTGGGTGAGTGGAGCGCCGGAGCGGGCGGGCGGGGGTTATATAGGGGGCCGGGCGGCGACACGTGCGCGGGG	5	24.2	4D-1131020-T4-3_704	CCACAAGTCGCGTCACAAGGA
T4-47	MLOC_37050		TraesCS3B02G148100	3B-0762490-T4-47	reverse	139,414,086	GAGAAGAGGCCGACTCGGTCGGCGTCCACGCTGATGATGTTGATCATCTCGCCGCTGGTGCGGCTCTGTCTGGAGATGCTGGACAGAGA	20	47.8	4D-1131020-T4-3_896	CTCCACGCCGGACGCCACTGC
T4-47	MLOC_37050		TraesCS3D02G129900	3D-0930440-T4-47	reverse	89,903,871	GGGTGGGCGCAGGCTGGGTGAGTGGAGCGCCGGAGCGGGCGGGCGGGGGTTATATAGGGGAACGGGCGGGGGGCGGCTGCGGCGC	18	86.4	4D-1131020-T4-3_960	TTTCTACTACCTTTCTCCCTTCT
T4-48	MLOC_59770	ABD	TraesCS2A02G136800	2A-0343440-T4-48	reverse	82,354,771	CGGACGGTGGCGCTCCGGTTGGATCCGCGGACTGGGATTGAGCTGGGCTCAGCTGGGCGGGGGGGATGGCTGGGATTATTATAACAGC	16	76.7	4D-1131020-T4-3_1024	TACAGCAAATCCGACCACAAA
T4-48	MLOC_59770		TraesCS2B02G160700	2B-0458840-T4-48	reverse	134,095,355	CCGACATTCTTTTGAAGTGGGGTACAATTCGGATCGTGGGGATTGGCCAACTAAACGTGGCGTCCCGGACACAGAGTAAGAGAGTGCTTA	16	76.7	4D-1131020-T4-3_1088	TTATTTCGCCTAGTTTGTCTGT
T4-48	MLOC_59770		TraesCS2D02G139500	2D-0593120-T4-48	reverse	82,189,925	CGGCGGCGATCCGGTTGGATCCGCGGGCTGGGATTGAGCTCAGCCGGGCGGGGGAAATGGCTGAGATTATAACGAGTGGATTGAGC	19	86.8	4D-1131020-T4-3_1152	TAGAACATGAAAGCCAAAAAA
T4-49	MLOC_60622	AB	TraesCS1A02G338700	1A-0031460-T4-49	reverse	528,103,055	TGTTGGTTGGTTGGTCTCGGGGATGGATGGAGAAGAAGGAGGGGGGGAGGGCTGGGTTCAGGGACGGCGGAGGGAGTGGGAATAA	21	85.6	4D-1131020-T4-3_1216	GATTGCGCTTGCTGCTCTTTTC
T4-49	MLOC_60622	no D	TraesCS1B02G351200	1B-0148870-T4-49	reverse	581,596,299	TGTTGGTTGGTTGGTTTCGTCGGGGATTGATGGAGAAGAAGAGAGGGGGGGAAGGGGATGGGTTCAGGGACGGCAGAGGGAGTGGG	16	69.1	4D-1131020-T4-3_1472	GTATATGGTGAAACGCACAGG
T4-5	TaBAK1	ABD	TraesCS2A02G343100	2A-0289900-T4-5	reverse	580,023,445	CCGAGCGCAGGCGGCTGCCAGGTGGGCCCGTCTCAAAGCCGCAGCATACGCGCGCTCCCGGTCTCCGGCGCCCTTGTGAGCTGTCTACG	6	32.5	3A-0682540-T4-4_64	GAAGAGTCAAAGTCTTTTGGT
T4-5	TaBAK1		TraesCS2B02G340700	2B-2107660-T4-5	reverse	486,196,723	CCAAGCGCAGGCAGCCGCCAGGTGGGCCCGTCTCAAAGCCGCGGCATACGCGCGCTCCCGGTCTCCGGCGCCCTTCTGAGCTGCCTACG	1	7.1	3A-0682540-T4-4_128	AGGCTACGTAAACCATTCATTC
T4-5	TaBAK1		TraesCS2D02G321400	2D-0534230-T4-5	reverse	412,839,075	CCGAGCGCAGGTGGGCCCGCCTCAAAGCCGCAGCATACGCGCGCTCCCGGTCTCCGGCGCCCTTGTGAGCTGCCCACGACGGCCCCCGA	6	35.8	3A-0682540-T4-4_256	GTGTTTACCGCTGATGCTTGCC
T4-50	MLOC_67318	AD	TraesCS5A02G261400	5A-1199640-T4-50	forward	475,294,801	GACTGGCCAACAAATTGCTCTAACGAGCACAAATAGGAGCACCCTTGGTTTCCTCCCCTACTCTTTTTGTCTCCTCCTATGGGTACATCCTTG	16	83.3	3A-0682540-T4-4_448	TGTGCCCGGTAGCCCCGGCTT
T4-50	MLOC_67318	no B	TraesCS5D02G268800	5D-1414830-T4-50	forward	371,862,068	GTGTGTTATCTTCTTTTATGCAAATTTGACATTTAAGCGTGAATTTTAGTATGCTTTGAAAGACCTTATCCATTTGTGCTTTTGATTAAAATTTA	12	61.6	3A-0682540-T4-4_832	CCCGGCTGGCGAAACGAACG
T4-51	AK248278	ABD	TraesCS1A02G089500	1A-0069860-T4-51	reverse	78,964,408	GGTGAAGTTCTTGACGAAGGTGAGTGAGTAGCTAGGTAGGCTCGGATCTGATCTCGCTACTAAGTGATGAGATGGTTGTTATCTGCAGGTC	13	63.2	3A-0682540-T4-4_896	TGGAATGAACCTCCTTCCCCTC
T4-51	AK248278		TraesCS1B02G117400	1B-0172070-T4-51	reverse	137,454,006	GGTGAAGTTCTCGACGAAGGTGAGTGAGTAGCTAGGTAGGCTCTGATCTGATCTCTGTGCAATGAGATGGTTGTTATCTGCAGGTGATGGT	20	87.3	3A-0682540-T4-4_960	GCTAGCCGCAGTGCCTAATTTC
T4-51	AK248278		TraesCS1D02G098000	1D-0264230T4-51	reverse	84,777,728	GGTGAAGTTCTTGACGAAGATGAGTGAGTAGCTAGGTAGGCTCTGGTCTGATCTCTCTACAATGAGATGGTTGTTATCTGCAGGTGATGGC	12	55.1	3A-0682540-T4-4_1024	CTTAGTGCTCTCAGTAGGTTCA
T4-52	AK354438	AB	TraesCS7A02G478700	7A-1755470-T4-52	reverse	671,483,848	GGCAGCGTGTCAGCCGGCCGGAGGGGAGCGCTCTGCTCTGCTTTGCTTTGCTCAGCTCAGCTCTTCTTGCGTCTTTGGGAGCTACTAGGA	16	73.4	3A-0682540-T4-4_1088	AATTTCTATTTCTAAGTTACCT
T4-52	AK354438	no D	TraesCS7B02G381400	7B-1860310-T4-52	reverse	646,210,404	GTCAGTGGGTCAGCCGGCCGGAGAAGAGCTCTGTCTGCTTTGCTGTGCTCTGCTTGCTGCGTGGGAGCTAGGAAGAGCTCGAGACCGG	20	84	3A-0682540-T4-4_1152	ACGCAGTGGCTAACGCCTAAC
T4-53	AK354978	ABD	TraesCS5A02G321300	5A-1223530-T4-53	reverse	534,121,689	GGTTGGAATCACCGCAGCAACCTCTTTTTCCTTTCTCGGGGAGTCGCCGCAGCAACTACTGCTAGTAGCGTGGTGCAAGTGACAGATCCC	6	29	3A-0682540-T4-4_1216	AAGTGTATATTAGAGAGTATTT
T4-53	AK354978		TraesCS5B02G321800	5B-1314640-T4-53	reverse	506,793,932	GGCGGGAATCACCGCAGCAACCTCTTTTTCCTTTCCCGGGGAATCGCCGCAGCAACTACTACTAGTACCGTGGTGTAACTGACAGATCCCG	11	57.9	3A-0682540-T4-4_1280	CGTTAATTACATGCCAAGTATG
T4-53	AK354978		TraesCS5D02G328100	5D-1462650-T4-53	reverse	420,715,875	GGTGGGAATCACCGCAGCAACTACTAGCACCGTGGTATAACTGACAGACCCCGGATGAGATGAAATGAAAAAATAATGGCTAGTGTTATG	13	72	3A-0682540-T4-4_1344	GACAAGAACACAAACTAGCCC
T4-54	AK360626	ABD	TraesCS7A02G176700	7A-1831290-T4-54	reverse	130,092,104	CGTCGGCGCGTTTCTCGGTGCCGCGGGCGCGCGCGGGGGGTATAAAAACGAACTGGCGAGGGAAGAAAGCAAGGCGATGGAGCGAG	10	65.7	3A-0682540-T4-4_1408	ACGGGCTCACGGAGGTCATTC
T4-54	AK360626		TraesCS7B02G081700	7B-1921350-T4-54	reverse	92,063,611	CGTCGGCGCGTCTCTCGGTGCCGCGGGAGCGCGCTGGCGTATAAAAACGAACTTGGCGAGGGAAGCAAAGCTATAGAGCGAGGCGTGC	14	69.2	3A-0682540-T4-4_1472	TTACCGTCCAGACTCAGATCA

T4-54	AK360626		TraesCS7D02G178000	7D-2062950-T4-54	reverse	131,269,964	CGGCGCGTTTCTCGGTGCCGCGGGCGCGCGCGCTGGCGTATAAAAACGAACTGGCGAGGGAAGCAAGGCGATGTAGCGAGGCGTG	15	72.9		3A-0682540-T4-4_1536	CAGTCAAATCTTTCGGCCAAT
T4-55	AK362809	ABD	TraesCS2A02G583400	2A-0311710-T4-55	reverse	775,309,370	TTCTCGTGGGACTCATCTGACAAAGGGCAAAGATCAATGTCCAACGGAAAGAGCGACAACAGATCAATCAGGCCAACTTTAACCGATCCC	5	32		3A-0682540-T4-4_1580	TCAAGCTTCGACCACGCTGTT
T4-55	AK362809		TraesCS2B02G603100	2B-0425810-T4-55	reverse	785,230,189	CCTGGCGAGCTGGTGGTGGACGACGACGGCGGCGGCGGGGAGGACCTGAGGGGTGGTTCGGCTGGATGGGNNNNNNNNNNNN	12	61.2		3B-0742130-T4-4_0	TTTTTGACCGTGGATGGGCAC
T4-55	AK362809		TraesCS2D02G596300	2D-0518190-T4-55	reverse	649,377,298	CCCGTCGAGCTGGTGGTGGGCGGCGAGGAGGAGCTGAGGGAGATTTGACGGCGAGAGTGGGGAATTGGTGGCCTGGTGGATTGGATT	18	83.1		3B-0742130-T4-4_64	CTCAAGTCTTCCCTTGCCCCGT
T4-56	AK365503	ABD	TraesCS1A02G047000	1A-0078240-T4-56	reverse	28,762,374	CGCGGACCGCGCCGAGGAAAGGAGTCGCCTTTGACGGAAGGCCCCTCCGCCGGCGGCGATCTCAGCAATGCGAAATCGAGAGGCGG	9	47.1		3B-0742130-T4-4_128	GCTGGCAAGGACTCAACTACG
T4-56	AK365503		TraesCS1B02G060700	1B-0157010-T4-56	reverse	44,072,171	CGCGGACCGCGTCGAGGAAAGGAGTCGCCTTGGATGGAAGGCCCCTCCGCCGGCGGCGATTGTGTCAGCAATGCGAAATCGAGGGGC	4	24.9		3B-0742130-T4-4_192	TCTCTCTTGTATCGTATCGTATC
T4-56	AK365503		TraesCS1D02G047900	1D-0249290-T4-56	reverse	27,900,391	CGCGGACCGCGTCGAGGAAAGGAGTCGCCTTTGATTTGATGGAAGGCCCCTCCGCCGGCGGCGATCTCAGCAATGCGAAATCGAGGGG	16	65.6		3B-0742130-T4-4_448	TGGATTTCGAGGTCAATTGCA
T4-57	AK366042	ABD	TraesCS3A02G206400	3A-0666460-T4-57	forward	363,415,982	TGAGGTACGATGCAAATTAATTAGAATCTGAATAATGCAGAGCAACCGCTATGGGCAAGGCAGGGCCGCCCGGAAGGAGTAGTGCATGG	17	77.2		3B-0742130-T4-4_512	AAGTGGCTTCGCAATCAAGGA
T4-57	AK366042		TraesCS3B02G238500	3B-0717870-T4-57	forward	373,842,628	GAATTTGGCTCAAGGTGGAGATTGTTAAATTGTGATCCAAATTCTATCGTATTGGACTAGACGTAGTACATACGATGTGGGCCTTTGCGTTG	16	70.1		3B-0742130-T4-4_576	AAAGAGGAAGACTTTAACGT
T4-57	AK366042		TraesCS3D02G209200	3D-0896070-T4-57	reverse	276,577,234	CTCACCCCTTTTGTGGAGGAGACTTGTTGTCTCTGCAACCGCTGTGGCCCAGCCAGAGGGTCAACTTGATGTTTCTGCAAGGGCAAATCA	20	84		3B-0742130-T4-4_832	TTCTGGGCTCGCTGGGGAG
T4-58	AK366432	ABD	TraesCS5A02G447200	5A-1243870-T4-58	reverse	626,971,834	CCTGAAAAACGCCATGCATGTTAAAATACTTGCTTATTTGGCTTCAAAAGGTACAGTTGATCAGTCTACTCTTTTTTTTTTTACTTTTTCCGA	11	54.6		3B-0742130-T4-4_896	CCTGACATGGGATCCACCCCC
T4-58	AK366432		TraesCS5B02G572600	5B-1330040-T4-58	forward	712,965,220	CAGACATATTGATGTAGTAGTATTACTTTGTAGGGTATTTGTGAATAATGAATAAAATGGATGCATGCATCTTTGAGATGCAGAGGCCGGGG	27	67.5		3B-0742130-T4-4_960	CGTAGATAAGCTACTAGGACA
T4-58	AK366432		TraesCS5D02G564000	5D-1454170-T4-58	forward	562,719,951	GAAAATTCATGCAACCGCGACGGATTTTATATAATAAAACGGCTAAATTCGTTACATTTTAGACATTTTTTTAACTAAAATGATACCCAAGTA	15	79.5		3B-0742130-T4-4_1088	GCGGATGTTCGCTGCAGCTGG
T4-59	AK366590	ABD	TraesCS6A02G241000	6A-1508720-T4-59	reverse	452,279,712	TTCCCCGGAGAACGAAAGCTCTGCTACGATTTCAGTCTCGTGGTGGCCTTGTTTCCTCCATGCGCGTACGTGCGTGCCTCCACCTCGCTCG	5	25.1		3B-0742130-T4-4_1152	TCTCCGCATCACTCGTGGCTT
T4-59	AK366590		TraesCS6B02G283600	6B-1582820-T4-59	forward	512,186,753	TGGCTTGGTGAGACTCCCCTTGCACTTCAATATCCTACTCTGTACAACGTTGTTCAACGTAGAGAAGCTTATGTTGCAACCGCTCTACAATC	6	32.5		3B-0742130-T4-4_1216	TCCATGCATATTGGCCTTCTCG
T4-59	AK366590		TraesCS6D02G223300	6D-1703710-T4-59	reverse	315,364,753	TTCCCCGGAGAACGAAAGCTCTGCTACGATTTCAGCCTCGTGGCGGCCTTGTTTCCTCCATGCGCGTGCGTCCACCTCGCTCGGCAGCTCG	8	42.1		3B-0742130-T4-4_1280	TGCGCAGATTTTACAGTAAAG
T4-6	TaSERK2	ABD	TraesCS6A02G203600.3	6A-1519210-T4-6	forward	349,515,682	CTTGTCATGCAGATTAAGTGCCAACAGTACCATTCTGTTTCCTATTATTAGTTGTATCATTTGTTTCCTTGTATTTATAAGTCTTAACTGTTTTTT	12	71.5		3B-0742130-T4-4_1344	CGTTCCCTCGGACGGAAGTTA
T4-6	TaSERK2		TraesCS6B02G223700	6B-1582330-T4-6	reverse	337,239,922	TCCTGCTCTAGCGCCGACTGGGCCCTCACCGACGCATAGGCGGCCCCCACCGGTTCTCCCTTCCCCCAGATCCCCCGAGCAGTACGGATCT	10	57.4		3B-0742130-T4-4_1408	GGGTGTACTGTGTACAGACTA
T4-6	TaSERK2		TraesCS6D02G186900	6D-1680260-T4-6	reverse	249,456,708	GGCGCGCGGGACGTCTATCGCTGTCGCCAGAGTGGTCGGGGAAGGCGGCGCCTTGGGGCGTTGGTGGTTGCGCGGGATGGATCTGGG	8	42.1		3B-0742130-T4-4_1472	ACCATGGTCAACAAGTGCGTG
T4-60	AK372866	ABU	TraesCS6A02G089400	6A-1542800-T4-60	reverse	57,732,613	GATGGCCGGTCCTCGTCGTCGGAAGGGTCGTCGCCGGGAGAGTCGTCGTCGTCGTCGGGGGCGCGGGGGTTTGGGGGTGCGAGC	9	46.8		3D-0902020-T4-4_0	CTCCTCCCTCTGCACGTAAGTT
T4-60	AK366590		TraesCS6B02G111800	6B-1669980-T4-60	reverse	93,569,684	GGTGCCCGGTTCTCGTCGTGGCCGTACTCGTCGTCGGAAGGGTCGTCGTCGTCGTCGTGGTCGGGGGCGCGGGGGTTTGGGGGTGGGA	8	45.4		3D-0902020-T4-4_64	TAGCTAGGACGCTTGGAAAC
T4-60	AK366590		TraesCSU02G016900	U-2129720B-T4-60	forward	19,226,560	AGACCCTACGGGTTCGGGAGACACGCAGACATGACCGAGACGACTCTCCGGTCAATAACCAACAGCGGGATCTGGATACCCATGTTGGC	5	22.1		3D-0902020-T4-4_128	CGTGCCCAAATCATGGCGGCT
T4-7	TaBAK1-7L	ABD	TraesCS7A02G293300	7A-1775970-T4-7	forward	373,858,525	GTCACGAGGCTCTCCCCACCCGTGAGCAGCCCAAAAAGACCGGTACTCCACCGTCCAGTTCTCCAACCCATCGCGTGTCGTGAACCAGCC	11	51.3		3D-0902020-T4-4_192	CGCTGGTTGGTGCGATCCTTT
T4-7	TaBAK1-7L		TraesCS7B02G182300	7B-1946820-T4-7	reverse	283,107,913	CTCAACTTCTGTTTGAAATTGTAGCTCCCCACCAGGTGTCCTCTCCTCCTTCAGTCTCTTGACAGCTACTAGCGTACCATCTGTTAGTCTCCC	18	79.8		3D-0902020-T4-4_256	ATCTCGGGCTGACATTTCTTC
T4-7	TaBAK1-7L		TraesCS7D02G291200	7D-1988150-T4-7	reverse	348,196,064	TGCCGCCGCCCTAGATCCCACAGCAGCAGCCGCCGCCGCGCATTCCACTCCCCGCAGAGGCCAAGCCTTTTTGCGACAGAGGCGAAGAT	11	61.2		3D-0902020-T4-4_320	GCGGATTATGGTGTGTTTTGG
T4-8	TaRAR1	ABD	TraesCS2A02G414300	2A-0286350-T4-8	forward	671,336,191	ATATGTAATCAAATGGGCAAAGCATGTACAACTATGAACATGTACTATAGCACTACCATCTACATCTACAACTAAGAACATGTACTAACCTTTT	11	54.6		3D-0902020-T4-4_384	GTCTCCCTTCCTTCTTGGCTTC
T4-8-1	TaRAR1		TraesCS2B02G433400	2B-0421420-T4-8-1	forward	622,701,947	CACTACCACCCCTCCGTAAGCTCACTCCCCCTCCCCCACTCGAAAAAATCGCATCGTTTCGCTCCCTCGACCCCCTGTAGATCATGGAGGT	23	96.5		3D-0902020-T4-4_448	GATGAGTTGAGTGGTAGTGC
T4-8-2	TaRAR1		TraesCS2B02G433400	2B-0421420-T4-8-2	forward	622,700,133	CGAGATTGAACTAGGTATTGAGATACCGACGATCGAATCTCGGGCAAGTAACATACCGATGACAAAGGGAACAACGTATGTTGTTATGCGG	2	10.8		3D-0902020-T4-4_512	ACTACTTGCACGAAGCTTCGG
T4-8	TaRAR1		TraesCS2D02G411600	2D-0531290-T4-8	forward	526,373,265	GCTCAGCCATGGCCCTGGACCTCCTGACGACACACAGCTCCGACGGGGACATCTCCAGATCCGCCTGCACCTCGAGCCTCGGCTCCGCCC	3	17.9		3D-0902020-T4-4_576	CAGTTTAGATCAGTAGATCCC
T4-9	TaSGT1	ABD	TraesCS3A02G347800LC	3A-0648390-T4-9	reverse	429,327,071	CGAAGGATCAAGTTCAATTGCTTTGTTGGCATCAGCTACAGCCTCTGCAAGACAAAAATTACCACGCGCTTAGTACACTATATTGTGCAAGT	19	86.8		3D-0902020-T4-4_640	GGAAAGGAAGGCAATATACTA
T4-9	TaSGT1		TraesCS3B02G255300	3B-0818610-T4-9	forward	411,831,372	GCCAATCCGAACATTTGAGCTGTTTGATGCTGAGCTGTAAACCTAAGTATCTACATGGGAACTTCCCGATCTGCTAGGGCACCATGCCTCGG	15	76.2		3D-0902020-T4-4_704	AACCCTGAAGTCGGCACTAAA
T4-9	TaSGT1		TraesCS3D02G227500	3D-0835460-T4-9	reverse	309,666,194	GTCTGCGTCGAGGTGGAGAAGACCAGACGAAGACTTTGGGAGAGGTGAATAGAGATCGACGGTATTTGGTGGGGAGGGGATGGATGG	10	60.7		3D-0902020-T4-4_768	TGATTGTTTATATAGTTATTAGA
											3D-0902020-T4-4_832	GATGACAACGCGCTTGGTACC
											3D-0902020-T4-4_896	TTGGTGTCAACAGGTCCGTCA
											3D-0902020-T4-4_960	CCATGACCAGCATGGAGGTTT
											3D-0902020-T4-4_1024	TACCAGTCCTCGCACAAGTATA
											3D-0902020-T4-4_1216	GCAGTCAAACTTTCCGGCACA
											3D-0902020-T4-4_1280	CCTCCTCATATCACATATCCTCT
											3D-0902020-T4-4_1344	ACTATTCTCCTCTGTCCATTTG
											3D-0902020-T4-4_1408	GTCGTCCTCGATCGGCCGGAG
											3D-0902020-T4-4_1472	CTCCTCCTCTTGGCTGTCGTCG
											2A-0289900-T4-5_128	TGCCTCGACCCTCCCCTCTTCT
											2A-0289900-T4-5_1024	GTAGACGAGTCGTAGAGTGG
											2A-0289900-T4-5_1088	GACTGCGAGCCTGAAACGCC
											2A-0289900-T4-5_1152	TTTGTGTTTTTGATTGACGATC
											2A-0289900-T4-5_1216	GGCCAGAAGCACGCACGAAT
											2A-0289900-T4-5_1408	GATGGTCTAGGGCACGATTAG
											2B-2107660-T4-5_320	TCGCGTCGCTGTCAGGTGGG
											2D-0534230-T4-5_128	TCCCCTCTTCTCTGACGGAGC
											2D-0534230-T4-5_512	CACCAGATATCAATCAATCATT
											2D-0534230-T4-5_1088	CCTGCGATGCCACTGTTCGCG
											2D-0534230-T4-5_1152	TTGACATGCGATGATGAAGGA
											2D-0534230-T4-5_1216	AGTACGTACGAATACGATGGG
											2D-0534230-T4-5_1408	AGGGCACGATTAGCTGTTTCT
											2D-0534230-T4-5_1472	gcatgcagtaaggctgtggcctgtggc
											2D-0534230-T4-5_1536	TGCACACTCTGCGGTTCTAGG
											6A-1519210-T4-6_0	CTTGTCATGCAGATTAAGTGC
											6A-1519210-T4-6_64	TTCCTTGTATTTATAAGTCTTAA
											6A-1519210-T4-6_192	CTGTAAGTTGAACGTTGTGCT
											6A-1519210-T4-6_320	TGGCTCCTTTTCGCTCTTCACC
											6A-1519210-T4-6_512	TACAATCCTCCAACACCACCA
											6A-1519210-T4-6_704	TTCCTGCTATTGCATTTGCAAT
											6A-1519210-T4-6_768	TGGTCAGTTTCATATACTGATA
											6A-1519210-T4-6_896	CTTCAGCCGAGGAAGATCCAG
											6A-1519210-T4-6_960	TCAAGTTGCATCTGACAACTT
											6A-1519210-T4-6_1024	TACAAGGGGAGGCTTGCAGA
											6A-1519210-T4-6_1280	GAATGCGGCTTCCTCAGCCAA
											6A-1519210-T4-6_1472	CACTGTGATCCCAAGATCATC
											6B-1582330-T4-6_128	CCCTCTCTCTTCCCTGATGAAA
											6B-1582330-T4-6_320	GGTCACGGAGTTGAGGGCCC
											6B-1582330-T4-6_448	CCGGCTGCAAACGAATCCATG
											6B-1582330-T4-6_512	GCTACTAGATTGGCTTTACCTA
											6B-1582330-T4-6_832	AATGCATTAGGCCCTATATAGA
											6B-1582330-T4-6_960	tccacgtagggttcagattacgtggac
											6B-1582330-T4-6_1088	CGCGGTCCTTTTCGCGGTCGC
											6B-1582330-T4-6_1216	CGAGCCGCACGCGCGGGCGG
											6B-1582330-T4-6_1280	AATTCCCCGGCCGTCAAAGG
											6B-1582330-T4-6_1408	CGGAGGGGAGCATGGGAAG
											6B-1582330-T4-6_1472	CGTCAGCGTGGGAGCCGGCG
											6B-1582330-T4-6_1536	ATGCGGTGGTCGGGAGCGGC
											6D-1680260-T4-6_256	GCAGTGCTGGAGGAGGTTGG
											6D-1680260-T4-6_1024	GTCTTGTAATTCAACTTCTGCC
											6D-1680260-T4-6_1088	CCTTGTGAACCAAGTTTTTCTT
											6D-1680260-T4-6_1152	ACTTAAAAAAGGAGTAAGGTT
											6D-1680260-T4-6_1216	GCCTCCCTCACGATCGGTTTC
											6D-1680260-T4-6_1408	CTCCGCCGATGTTGCGTGTCT
											6D-1680260-T4-6_1536	GATCCATCGCAAGTTTCTCTCT
											6D-1680260-T4-6_1580	CATGGACTCCGACAAGCAAA
											7A-1775970-T4-7_0	GTCACGAGGCTCTCCCCACCC
											7A-1775970-T4-7_64	AACCCATCGCGTGTCGTGAAC
											7A-1775970-T4-7_128	GCGCTTATAAGGGTGGGTGTA
											7A-1775970-T4-7_192	GTCTCATACCGTAAGCAACCG
											7A-1775970-T4-7_448	ACAGAACGGGATCGATCGCAT
											7A-1775970-T4-7_512	GCGGGCGACACAGCTACAGG
											7A-1775970-T4-7_576	CTGATGAATATGTTCCTATCGC
											7A-1775970-T4-7_640	CGCGCTAGTCATGTGAGATCG
											7A-1775970-T4-7_704	AATTCATCAATACTTGCATGGT
											7A-1775970-T4-7_1280	TCTTTTGTTTCGCACAGGGTC
											7A-1775970-T4-7_1344	TTAGCCCACCAGAATTCAAGT
											7B-1946820-T4-7_64	GCTACTAGCGTACCATCTGTTA
											7B-1946820-T4-7_128	TGTTCTTATTGCTGAAGGTATC
											7B-1946820-T4-7_192	ACCAAGATGGACCTCTGGATC
											7B-1946820-T4-7_256	TTACAAGTAATTTTCCTCATGC
											7B-1946820-T4-7_448	AGAACAAAACCATCAATTCCA
											7B-1946820-T4-7_512	CAAGCAATGTTCCAGAATAGG
											7B-1946820-T4-7_704	TCTTGTATCATTGTTAAAAAAC
											7B-1946820-T4-7_768	ACATGAAGCACCAGATAATCT
											7B-1946820-T4-7_832	AATATCGACAACAGTATCGACT
											7B-1946820-T4-7_896	AGTCTCATCCCAGCTGGGATG
											7B-1946820-T4-7_1152	TAACTGCAAAATAAACAAGTG
											7B-1946820-T4-7_1216	CTAACATGCTAGCTTTACCTGA
											7B-1946820-T4-7_1280	CTGACAGCTTATTATTTGACAA
											7B-1946820-T4-7_1344	CGCTTCCTAGTTTATACCAAAG
											7B-1946820-T4-7_1408	TACAGAACTTGGAGAGCAGTA
											7B-1946820-T4-7_1472	TGTTAAGACGCCTATGCTCATA
											7B-1946820-T4-7_1536	TATAGGGAAAATTAACACAGT
											7B-1946820-T4-7_1580	TATTTTAATAACTTAATAACAA
											7D-1988150-T4-7_128	TCTCCTCGGCCCGAAGCCCCG
											7D-1988150-T4-7_448	TTGGATCTGCCCCTTTGGGTCT
											7D-1988150-T4-7_512	GCACAGTACCATCATATATACG
											7D-1988150-T4-7_704	AACGCTTGGTTGATCTAATGT
											7D-1988150-T4-7_768	TTCACGAGACAACCTCATCGG
											7D-1988150-T4-7_960	AGGGCACATAGGGGCTTACC
											7D-1988150-T4-7_1088	TGGTGATCACACACCAATTCT
											7D-1988150-T4-7_1152	CACAAAACTAGATCATTGGCT
											7D-1988150-T4-7_1344	ACATACCAGCGCCCAAGGAGT
											7D-1988150-T4-7_1408	TCCATGACTTTGTTGCATGGT
											7D-1988150-T4-7_1472	TGCCATCGAGACATTTGTCAAT
											2A-0286350-T4-8_0	ATATGTAATCAAATGGGCAAA
											2A-0286350-T4-8_384	TGCTTGGCGGCGGCGGCTAG
											2A-0286350-T4-8_704	AACATTGAGAGCAAGTTGTAG
											2A-0286350-T4-8_768	ATTGCGAAATTTCTGTTTTTGT
											2A-0286350-T4-8_832	AAATGGAATTTGTGTTGGGCC
											2A-0286350-T4-8_896	AATGTTTTCGTTTGGTTGTCTC
											2A-0286350-T4-8_960	AGTGAACCATTAGAGCCCCTC
											2A-0286350-T4-8_1024	CGATAACCAAATTAATACGAA
											2A-0286350-T4-8_1216	TCGAATGCACTAAGCTTATTTT
											2A-0286350-T4-8_1280	AAACACATACATGTGAATTGAT
											2A-0286350-T4-8_1408	ATAGCTTTAGTCTTAGCAAGT
											2A-0286350-T4-8_1472	TAATGCTAAAAAAAATGTTTCC
											2B-0421420-T4-8-1_128	GATCTCGCTTTGGTTAGACTC
											2B-0421420-T4-8-1_64	CCCTCGACCCCCTGTAGATCAT
											2B-0421420-T4-8-1_192	ATCCTCTACGTCCACGGAAAG
											2B-0421420-T4-8-1_256	CCAAATCTTGGAGACAGTCAA
											2B-0421420-T4-8-1_320	CAATCGATATGGCGGTAAATT
											2B-0421420-T4-8-1_448	GTTTTGTGGGTTGTAGACAGA
											2B-0421420-T4-8-1_512	GACGACCATGTTATATTATGTT
											2B-0421420-T4-8-1_576	GGACATAAAAACTTGGCACCC
											2B-0421420-T4-8-1_640	GTTTGATGTTATGATTGAAGTG
											2B-0421420-T4-8-1_704	AGTGAATTCCAGAAAGCTATA
											2B-0421420-T4-8-1_768	AATATACAACTACAGATTCGAG
											2B-0421420-T4-8-1_832	CTAAATCTGCAGTTCTTTGAA
											2B-0421420-T4-8-1_896	CTGTGTGTGAAGTTTCTTTAA
											2B-0421420-T4-8-1_960	AAAATAGCAATTTTAAACACTT
											2B-0421420-T4-8-1_1024	TGCATTAAGTAACTGGCGCCA
											2B-0421420-T4-8-1_1088	TTAGTTGTATTATACAATGGAT
											2B-0421420-T4-8-1_1152	TGATAGTTTCTACACTGAACAT
											2B-0421420-T4-8-1_1216	CACCTATTTCACTGATGCAGAT
											2B-0421420-T4-8-1_1280	CATATAATTCTGTTTTTGTCTTT
											2B-0421420-T4-8-1_1344	TAGGCAATCCCAAGTTCGATT
											2B-0421420-T4-8-2_1344	AAAAGAAAAGCCCCTCCCATC

2B-0421420-T4-8-1_1408	TTCAAAAGTACACTAATAAGA
2B-0421420-T4-8-2_1408	CCAAATTAATATGAAAAAAAA
2B-0421420-T4-8-1_1472	atattggaaaaataagttatgaATCA
2B-0421420-T4-8-1_1536	TTCAGAAAATTACAAATTTAC
2B-0421420-T4-8-1_0	CACTACCACCCCTCCGTAAGC
2D-0531290-T4-8_640	CCGCTCCCCATTGAACGAGGA
2D-0531290-T4-8_1088	TCCATTTTCTTTTGTTGGTAAC
2D-0531290-T4-8_1152	ATATTTCCGAATATACCAATATT
2D-0531290-T4-8_1580	CAGCCTGCCCCCGTCGTCTTT
3A-0648390-T4-9_64	CGCGCTTAGTACACTATATTGT
3A-0648390-T4-9_192	TGAAGGAATTAGCCTCAATGA
3A-0648390-T4-9_256	TTGATTTATGTGTTCTCTACTT
3A-0648390-T4-9_320	CCCAAGAAGAACACTTGAGA
3A-0648390-T4-9_384	ATGAATGTGAGAGGATAATGC
3A-0648390-T4-9_448	TTGAGCTTACATGATCTTTCAT
3A-0648390-T4-9_640	CAAAGAGCAGACCAAGGCTT
3A-0648390-T4-9_704	ACAGGGATTAGGTGCTAAGA
3A-0648390-T4-9_768	CTCCATGCACAACAATGTACCT
3A-0648390-T4-9_832	AATCTACAGATTTCACTATTAT
3A-0648390-T4-9_960	TCCAGACCTCACTATGCAGGG
3A-0648390-T4-9_1024	AAATAACCTCATACAGAGGAA
3A-0648390-T4-9_1088	TTCTAGCATATCAAGGAGTGG
3A-0648390-T4-9_1280	TAGGACACCGAATCTAACTTC
3A-0648390-T4-9_1344	AGGCAAAACAAAAGCACGCA
3A-0648390-T4-9_1408	GATGGCATTTTACTTTACATCA
3A-0648390-T4-9_1472	TGAACAAGTAATAAAACAAGA
3A-0648390-T4-9_1536	ATAACAAAATAACAATACTCCA
3A-0648390-T4-9_1580	TTATGGTGGTTAAGTGCCTCA
3B-0818610-T4-9_0	GCCAATCCGAACATTTGAGCT
3B-0818610-T4-9_128	ATGGCAAACGACTTAGCGTAG
3B-0818610-T4-9_192	CTAGAACAAATGACAAATCCG
3B-0818610-T4-9_256	GGACAGTCTTTGCGTGGCTCC
3B-0818610-T4-9_448	GGCCGTGGAAAGAAGTGTAA
3B-0818610-T4-9_512	ACCTCAAAGAGAAACGGCCA
3B-0818610-T4-9_576	ACGCATTTAGCTTTCCTCTTAT
3B-0818610-T4-9_640	GTATGCTGCGTCCTTTGATGA
3B-0818610-T4-9_832	TCCCGAAGCCGCGTCCATTAT
3B-0818610-T4-9_896	CACATTCCATGCTTTTTGAAAG
3B-0818610-T4-9_960	CTCCTGAAACACCAAGTCTTG
3B-0818610-T4-9_1024	ACCGTGTGCTCTCGAGCCGAG
3B-0818610-T4-9_1088	CCTTCCCCTTTTGATCAGTGAT
3B-0818610-T4-9_1280	CTCAGACACCAAGATCGAAAA
3B-0818610-T4-9_1344	GATTCGTGAAACTTCATGGGT
3B-0818610-T4-9_1408	aaggaaaggaaaggaaaggaaaaa
3B-0818610-T4-9_1472	CATGCACTGCAACTCCAATCC
3D-0835460-T4-9_0	GTCTGCGTCGAGGTGGAGAA
3D-0835460-T4-9_192	AGCCAGGCCGAGGGGGGGA
3D-0835460-T4-9_256	ttttcctcctctcttttcctttcctttcctt
3D-0835460-T4-9_384	CATCACCTAGTGCTGCCCCAA
3D-0835460-T4-9_448	TTCATTCAATTTAACCGATAGC
3D-0835460-T4-9_512	TCGCCATAGCACATGGACTTT
3D-0835460-T4-9_768	TATGGGGTTAAGGGGGTGAC
3D-0835460-T4-9_960	CTCAAGCAACATGGAGATGAG
3D-0835460-T4-9_1152	GAGGCCCGAATGCGACCGGA
3D-0835460-T4-9_1216	TCTGTCCCCATAAAAAACCCA
3D-0835460-T4-9_1408	GAGCTCCTCCTCGTCGGCAAT
3D-0835460-T4-9_1580	CCGGTGCCGGCACCACCCAG
4A-1008960-T4-10_64	ACGCGCAACGGGATGAGGCT
4A-1008960-T4-10_256	GGTCTCTGGCTCTTTTCTGTG
4A-1008960-T4-10_576	ACGCGTTATTGTTTTGCTTGG
4A-1008960-T4-10_704	ATCTGGAGACTGGGCAATCCT
4A-1008960-T4-10_768	ACTAGGTCCCATACATGAGAA
4A-1008960-T4-10_832	aaaaaagtcttacattatgggacggag
4A-1008960-T4-10_1408	agatgcagaggccgggggtcattctcc
4A-1008960-T4-10_1472	TATAATGTAGGCTACGAGGTT
4A-1008960-T4-10_1536	ATTATCCAAGTGACAACACTAT
4A-1008960-T4-10_1580	AAGCAATCTTCATTGTGTCAC
4B-1033300-T4-10_256	aggaacagagggagTGTATCGGG
4B-1033300-T4-10_320	AGGGGAGGGATTTTCTATTGA
4B-1033300-T4-10_384	TTCAATCTTGAACGCAACCAG
4B-1033300-T4-10_448	GGTTAACATTATTCATCTCACG
4B-1033300-T4-10_512	AGATTTGATCTCCTAAACTGA
4B-1033300-T4-10_576	ATCGAAATCTTTTAACTAGAA
4B-1033300-T4-10_640	ATAGTGTTGTCACTTGGATAAT
4B-1033300-T4-10_704	AAAACTTCGTAGCCTACTTTG
4B-1033300-T4-10_768	TTTAATAGCAAAAAAGAGAGT
4B-1033300-T4-10_832	CCTCAATCCCATAATGTAAGA
4B-1033300-T4-10_1024	ACGGTGCCGAAGAAAAGCCA
4B-1033300-T4-10_1408	TCCCCTCAGTTCCCGGGCGAT
4B-1033300-T4-10_1472	GCTGTGGTGTGCTCCCGCTCG
4B-1033300-T4-10_1580	TACAAGAATCGAGAGCCAGG
4D-1148960-T4-10_128	TGAGGAGGAGAGAATTATAA
4D-1148960-T4-10_256	GTCTCTCGCTCTTTTTTGTGCG
4D-1148960-T4-10_512	GGTGTTGAGGGCATGGAAAT
4D-1148960-T4-10_576	ACGCGTTATTGTTTTGCTTCG
4D-1148960-T4-10_704	ATCTAGAGTCGGGGCAATCCT
4D-1148960-T4-10_768	TCTCTTGGGGACGCGCACCGT
4D-1148960-T4-10_832	CAAGCGGGGCGGTCTGGCAG
4D-1148960-T4-10_896	TATGCTGTGGTAAGAAATCTT
4D-1148960-T4-10_960	TCTTCACAGCGTAAGCAATCT
4D-1148960-T4-10_1024	AAATCGTCGATGAATAGGTCC
4D-1148960-T4-10_1088	TGAGAGGAAGATAGAGATTAT
4D-1148960-T4-10_1152	AGGATTGGCTGTATGGAGGTT
4D-1148960-T4-10_1216	AACTGTCGAAATCGGATCTCT
4D-1148960-T4-10_1280	cacccaccctGCAAAAAGGTAG
7A-1834630-T4-11_64	CGCTTTCCGCCTCTTGTTTGTT
7A-1834630-T4-11_256	TTTTACTCCAAGTGGGAAAAT
7A-1834630-T4-11_320	TGCATCCCTAACCCTAGAAAA
7A-1834630-T4-11_512	ACCTTGTGTCCGTGCGTGGCC
7A-1834630-T4-11_576	GGTGCGCCTACGCTATTTATAG
7A-1834630-T4-11_832	TCTACAAGCACCTCGCGAAGT
7B-1954300-T4-11_192	gggaggagatggctggcagcgcgcgt
7B-1954300-T4-11_256	ACGGCTTTTTACTCCAAGTG
7B-1954300-T4-11_320	AAGCTCATCATGCCTACCTATG
7B-1954300-T4-11_512	TGTCGGCGCTGACTGGTGTGT
7B-1954300-T4-11_576	CGGATGCGTCTATACTATTTAT
7B-1954300-T4-11_640	GTGATTTGGGTTTCCCAACAC
7B-1954300-T4-11_704	AGTCTTTCTTGTCCACGCCCAT
7D-2040040-T4-11_64	GATTTGGTCGCTTTCCGCCTCT
7D-2040040-T4-11_256	GGGAGTGGCCGGCTGCTACG
7D-2040040-T4-11_320	CAACCTTCTCTGGTAGAGAAA
7D-2040040-T4-11_384	AAGAAAGCTACAGTCCATCCC
7D-2040040-T4-11_576	TGGCCGTGTAGGCGTCGTCGG
7D-2040040-T4-11_640	TTATAGGGGTAGGATGTCACG
7A-1770880-T4-12_704	tcttggtgatgcgtaaaaaaatttaaat
7A-1770880-T4-12_768	ACCCTCGTCTACGCCGAATAC
7A-1770880-T4-12_832	GATAGATGCAAGTTGGTTACA
7A-1770880-T4-12_896	ATCAAGATTCATATGATCTTGT
7A-1770880-T4-12_960	CCTTGATCCAACAACAACATT
7A-1770880-T4-12_1024	GATTAATACTAACGGTGCCAT
7A-1770880-T4-12_1088	TCCCACATGTCCTTCTTTGGG
7A-1770880-T4-12_1152	TGGAGCAATGGCCATGCGAG
7A-1770880-T4-12_1216	ATGGAAGTGGATTTTTTATAG
7A-1770880-T4-12_1280	TTATGACGCATATCTTAGATGA
7A-1770880-T4-12_1344	GCATGTCTCGAGAGATGCTAA
7A-1770880-T4-12_1408	TTTCAGAGTGTTGGCTCTAGG
7A-1770880-T4-12_1472	CGGGATTCTCTGAATCAATAA
7A-1770880-T4-12_1536	CCATGAAAGAATACAATGATC
7B-1867300-T4-12_0	TTTTTCCGGAGGAGGAGGAG
7B-1867300-T4-12_384	TGTCCATCTGTAATAATCCTAAT
7B-1867300-T4-12_448	GAGTTGTCGGGTCAGTGACA
7B-1867300-T4-12_512	GTCCCTGTGTGGCCGGCCGAT
7B-1867300-T4-12_576	CATTTATGTAGAAACTGTTTGG
7B-1867300-T4-12_640	TGTAAAAGTTATTCATGACTAA
7B-1867300-T4-12_704	TGTTTTAAAATTCAAGAAAAT
7B-1867300-T4-12_768	TAACAAAAATGTTTGGGAATT
7B-1867300-T4-12_832	AAAAAAACAAGTTTCTATAAA
7B-1867300-T4-12_896	GTATTTAAAAACGTGAATTTTA
7B-1867300-T4-12_960	AATACCTAAAAAAGTCCATGAT
7B-1867300-T4-12_1024	AAAAATGTTAATGAATTTTAAA
7B-1867300-T4-12_1088	GTTATCGTATTTCAGAAAAAGT
7B-1867300-T4-12_1152	TGGTTTTCAAAAATGTTCACA
7B-1867300-T4-12_1216	TTCAAAACAATTCATGAGTTTT
7B-1867300-T4-12_1280	GAAAAACGGAAAAACATAAA
7B-1867300-T4-12_1472	TGGAGTTCGACCTGAAGAGG
7B-1867300-T4-12_1536	GAAGCGAGAAAAAATTGTAA
7D-2004100-T4-12_64	TCCAAACTTTTTTTTTTGAGG
7D-2004100-T4-12_256	GCCGCGCGTGGCGGCACGTC
7D-2004100-T4-12_320	GAGGAGAGGTGCAAGGCTTA
7D-2004100-T4-12_384	AATATGTAGAGAAATACATTGA
7D-2004100-T4-12_448	AGAGTAAGTGCATACTTAGAA
7D-2004100-T4-12_512	AAATAACAAAATTATAAAACA
7D-2004100-T4-12_576	ATTGCCTTCTTTGACGTTTGG
7D-2004100-T4-12_704	TGACACCAACAAAGACCATCA
7D-2004100-T4-12_768	TCTTACCGAACCAACACTCGA
7D-2004100-T4-12_832	TAGGTGGAAGGGTCCATCTAG

7D-2004100-T4-12_896	GGTTTGCAAGAAGTGTCTCAG
7D-2004100-T4-12_960	AGGATGGAAACTCCTAATCGT
7D-2004100-T4-12_1024	CGCTTTAGGCTGGGCAGGCG
7D-2004100-T4-12_1088	TTTTCATGTCTGTAAAAAATGT
7D-2004100-T4-12_1152	ATATGGAAAAGTTATTCATGA
7D-2004100-T4-12_1216	AAATGTTCTAAAATTCAAAAA
7D-2004100-T4-12_1280	AGATAACAAAAATGTTTGGGA
7D-2004100-T4-12_1344	AAAAAAAAGTTTCAAGTTTCT
7D-2004100-T4-12_1408	ATTTCTGTATTTAAAAAACGTG
7D-2004100-T4-12_1472	TTTGGTAAATACCTAAAAAATT
7D-2004100-T4-12_1536	AAATTTTTAAAAATGTTAATGA
7D-2004100-T4-12_1580	TCAAAAAATGTTCTTGAATTT
7A-1801960-T4-13_0	GGCCATTGCAGCCCAGCTCG
7A-1801960-T4-13_768	ATGGTTAAAATGTATGCCGGA
7A-1801960-T4-13_832	TCCCTGTCCGTGACCAGATGC
7A-1801960-T4-13_960	ATTTTTGCAACCTTCTGTCCTT
7A-1801960-T4-13_1024	AGAATGCGAGATGATCAGAAA
7A-1801960-T4-13_1088	CAGCACGTGTTCATCTGAGAC
7A-1801960-T4-13_1344	CTTGTCTATTATGAAAATGCAT
7A-1801960-T4-13_1408	AAGGTTTTCACCTCGATGAGG
7B-1860490-T4-13_1024	ATGTCCGTGTCCATGGACGGA
7B-1860490-T4-13_1088	ACGTGGGTCGAGCTGTTCGA
7B-1860490-T4-13_1152	TCAACCTGATTTTTGCAAATT
7B-1860490-T4-13_1216	CCATGATCCGGTTCTACAGCT
7B-1860490-T4-13_1280	AATTGTTCGTCCTAACGAGAC
7B-1860490-T4-13_1344	CAATTATTATTTTTGCTCTTTA
7B-1860490-T4-13_1408	ACACATAGCTGAAGAAAAGG
7B-1860490-T4-13_1472	TGCATCCAAGTGTCAACACGC
7D-2008410-T4-13_1088	CGTGGACAGATGCGGAAGAG
7D-2008410-T4-13_1152	GCTCTTCGACGGTTATCTGAG
7D-2008410-T4-13_1216	AACCTTCTATCCTTGATCATGA
7D-2008410-T4-13_1280	AGATGATGAAAAAGCAAAATT
7D-2008410-T4-13_1344	TTCATCTCAGACCATTTCAATT
7D-2008410-T4-13_1408	AAGGTTTTCACCTCGATGAGG
3A-0688550-T4-14_128	GTTCAAGTGTCTGGCTGGTGG
3A-0688550-T4-14_192	CTTTCCTATCCTCATTTAGGCA
3A-0688550-T4-14_256	GGGCCAAATATTAGGTCTCGT
3A-0688550-T4-14_320	GGGGATGATGTTGTGGCGTTT
3A-0688550-T4-14_384	ATGGGACTGCATCGGGGAAC
3A-0688550-T4-14_448	TTATCTTCTGTCCCTTCTCGTC
3A-0688550-T4-14_512	CGACGTCTTCTAGTCTACATCG
3A-0688550-T4-14_576	CAAACTACTATGTCGAGAAGA
3A-0688550-T4-14_640	AAAAAAACTTGGACGGGCCA
3A-0688550-T4-14_1024	TCCGTTGCAATCGCACGGACA
3A-0688550-T4-14_1088	TCGTATGCCTCTCGGCCTTGTT
3A-0688550-T4-14_1216	ggatcatGTAGAAATATTTTAGT
3A-0688550-T4-14_1280	TTCGGATGGGGGAGTATTTTT
3A-0688550-T4-14_1472	TGCAAGATCGCCATGAATCCC
3B-0744050-T4-14_896	CGGCATCGGAGACGAGGACG
3B-0744050-T4-14_960	GGCCAAATGGAAGAGTAGGA
3B-0744050-T4-14_1024	TTCCGCGAGCCAGGCTGAGG
3B-0744050-T4-14_1088	TTTTTCTTCAAAAAACGAAAT
3B-0744050-T4-14_1152	TGTTTTCGCAATTTACTCGAA
3B-0744050-T4-14_1216	ACGGAACTGTTGACGAGCTCT
3B-0744050-T4-14_1280	GTGGAGATGCTCTTACGTAGT
3B-0744050-T4-14_1472	CGATACATCCCCCATGGTGTG
3B-0744050-T4-14_1536	AGCCGTGTGAATCCTCATGTG
3B-0744050-T4-14_1580	CGTTTAAACCTCTAATCAGATG
3D-0914040-T4-14_128	TTGTATGTAGTGTTTGGTCAC
3D-0914040-T4-14_192	GGTGAGATGTTGTTGTGATCG
3D-0914040-T4-14_256	TGTTGTTTGGCATCGGCGGG
3D-0914040-T4-14_320	TGGTCTTCGCTTTGTCTAGGT
3D-0914040-T4-14_384	TTTTTTCTTCGGTTCCTTCTTT
3D-0914040-T4-14_448	GCGAGGTTTGTGTCCTCTACC
3D-0914040-T4-14_512	GTCGACTTCTTCGGAACAACG
3D-0914040-T4-14_576	ATGGCTTTTCAGCCACTGCTT
3D-0914040-T4-14_640	AGGCGACAATGAGCTTTTCTA
3D-0914040-T4-14_1088	ACATTGCTAGTATTCTTCAATG
3D-0914040-T4-14_1152	TTATAGCTCCGCGAGTGCTATA
3D-0914040-T4-14_1216	TTAATTTCTtactcccttcgtccaga
3D-0914040-T4-14_1280	TCATTTTTATTTATTTTTGCGA
3D-0914040-T4-14_1472	GCAAGATTGCCATGAATCCCC
6A-1573990-T4-15_0	GCATCCATTTCTACAAGGCGG
6A-1573990-T4-15_64	ACGATAGACCAGTCGATGGGA
6A-1573990-T4-15_320	ttgggtcatctattttggaacggaggga
6A-1573990-T4-15_384	CCGGTAGAATCTGCTGTATATA
6A-1573990-T4-15_448	TATGTACCGCGTAGACACATA
6A-1573990-T4-15_512	TGCCGGGAGCACATTTCGCCT
6A-1573990-T4-15_576	AAAGAAACAAGGCTATAACCT
6A-1573990-T4-15_768	TGTTCGCGCAAGTTCGACGCC
6A-1573990-T4-15_832	GACCAGCCCTTCCCGCGCTCT
6A-1573990-T4-15_896	TTATTAGTTAAGATGTTAGCGT
6A-1573990-T4-15_1024	AGGCACAATCATTGTAACCGA
6A-1573990-T4-15_1216	GTCGCCGTCGGCCTCGGGATA
6A-1573990-T4-15_1344	GGGTCGGCCATTTCCTTCTCG
6A-1573990-T4-15_1408	GGATGGAGGAGCATGATTATT
6A-1573990-T4-15_1472	CATGTACCGAAGGTATATAGC
6A-1573990-T4-15_1536	CAGTATATACGTGCTTGCCTTG
6B-1671980-T4-15_64	CCTGTCCTGCGCTCGCGCTTT
6B-1671980-T4-15_256	GGTTGATTCGGTGGGAAGGA
6B-1671980-T4-15_384	CGCTGCACGCAGTTTTTTTTT
6B-1671980-T4-15_448	CTGCCTATGAACGCGCGAGG
6B-1671980-T4-15_512	GCGGCGTTTCACGTGAAATTT
6B-1671980-T4-15_576	atgtttttttatattaaaattaaaatGT
6B-1671980-T4-15_640	TTGGATCGGCCGGCTTTCGGA
6B-1671980-T4-15_704	CTTTGGTGGCCAACCAAACAA
6B-1671980-T4-15_768	AGGCCATTGGAGTGGCCGCC
6B-1671980-T4-15_896	GCATGTATGCTCTAGCCTTATG
6B-1671980-T4-15_960	AAGTCACCGCATGTGCATCAT
6B-1671980-T4-15_1024	AAAGCCTGAAATAAATCCAAA
6B-1671980-T4-15_1088	GAGGTGGTGGCAACAACATG
6B-1671980-T4-15_1344	TACATCCCTGCTATGTCGGCGA
6B-1671980-T4-15_1408	TCTGAAAAAAAATAGATACGT
6B-1671980-T4-15_1472	AAGCAGTTAGTTGGTAGTATG
6B-1671980-T4-15_1536	TGGTGGTAAAGGATTACAAGT
6B-1671980-T4-15_1580	TTAACCAATGTTCCTTCTGCGG
1D-0240360-T4-15_1216	CTCTCTGAGCCAGGCCCAGCA
1D-0240360-T4-15_1280	GTTATCATTCTGTGTGGATTGC
1D-0240360-T4-15_1344	CGTCCTGTGTGGGGAGGCGG
1D-0240360-T4-15_1408	GACTACAATTTTCTCAAGGAA
1D-0240360-T4-15_1472	GGACCGCGCAACCCAGGAAC
1D-0240360-T4-15_1536	ACTAGGAGCAATCACCGATTC
1D-0240360-T4-15_1580	CTAGGTTAGCTTCATTATATAT
7A-1798270-T4-16_0	TAAAGTGTCACTTATCTTAAAC
7A-1798270-T4-16_66	AGTAAAAAATTAACTCTAAAA
7A-1798270-T4-16_132	TTTTTTTTCTCTAACCGGTTTT
7A-1798270-T4-16_330	GAGATTAATAATTATGTAAGCT
7A-1798270-T4-16_1133	CATCCCTTCCACCATCGTCGC
7A-1798270-T4-16_1197	GCCAGCAAATCCATCCATCAC
7A-1798270-T4-16_1453	CTGTGGATCCTCCTAGATCGAT
7B-1864900-T4-16_128	CCCGCGGGTCGATGGGCTCC
7B-1864900-T4-16_192	GCCGAGCAGGCGGACGAACA
7B-1864900-T4-16_256	CAAGATTTTCTTGTAGGTGTG
7B-1864900-T4-16_320	TGCACACCGATGTACTGCCTC
7B-1864900-T4-16_384	GGCGTCCGCGGGAAGAAGA
7B-1864900-T4-16_576	AGAGTTGAATTTTAATCCGCA
7B-1864900-T4-16_640	AAATTAACTCCTCTAAAATATT
7B-1864900-T4-16_704	TTTTTTCTCTAACCGGTCGTAG
7B-1864900-T4-16_896	AGAACAAAGGATGGAGCATC
7B-1864900-T4-16_1024	GTCGCGTAGGAGTGAGAAAG
7B-1864900-T4-16_1088	CTCTCTCGAAGTATGGAATTG
7B-1864900-T4-16_1152	TCCATCCCTTCCACCATCGTCG
7B-1864900-T4-16_1216	TATCCAGCAAATCCATCCATCA
7B-1864900-T4-16_1408	GTTGGGTTCTTTGCTTGGTAC
7B-1864900-T4-16_1472	TGCATGTAGCTGCCAGGAAAT
7B-1864900-T4-16_1536	TGCGGCTCTTTTCTTGATGATG

7B-1864900-T4-16_1580	TGTACTGATCGAGCatgcattgc
7D-1974840-T4-16_273	CTCCGCCTTGACTTGCTCCGG
7D-1974840-T4-16_399	GGGTAGACGGGCTCCAGTTG
7D-1974840-T4-16_588	CTGGGATTGGGTGGAGGAGA
7D-1974840-T4-16_651	AATATAAGAGTTCGATGCCAC
7D-1974840-T4-16_714	GCTAGGTGTCGGTTAGAGGA
7D-1974840-T4-16_903	AAGACCAAAGGATGGAGCAT
7D-1974840-T4-16_1029	GAATTATTGGCGCGTCGCGTA
7D-1974840-T4-16_1092	CCAAAGCAATCTTTCTCTCTCG
7D-1974840-T4-16_1155	ATATCCGAAAGGCATCCATCC
7D-1974840-T4-16_1218	TCCGCGAATATATCCTAGCCAG
7D-1974840-T4-16_1407	GATCGGAGCAAGCCGCTTGT
7D-1974840-T4-16_1470	GATCGATATGATGTAGGCATG
7D-1974840-T4-16_1533	TTTATCTATAGCCTTCTGCGGC
7D-1974840-T4-16_1580	CATATATACTTGTACTGATCCA
1A-0075270A-T4-17_0	TGCTATGATGGCTTGGAGAGT
1A-0124980B-T4-17_64	CTGTACACTGTTTGGGTTGCA
1A-0075270A-T4-17_192	TACTTGTATTTGGACTAGTCCT
1A-0075270A-T4-17_256	AATCTAGGTATCCCTTGTCCTA
1A-0075270A-T4-17_320	ATGAGGATAAAATTAATATCTT
1A-0124980B-T4-17_576	AATCCTGTTAATTCCTTCAGTA
1A-0075270A-T4-17_704	TTACTTGCAATTTCACTAGAG
1A-0124980B-T4-17_704	CAGTATGTGAGATAAGCATTT
1A-0124980B-T4-17_768	ACACTTCCATCGATAAATTCCA
1A-0075270A-T4-17_832	GATACCAGTTTCACGTTGCTT
1A-0124980B-T4-17_832	tggtttgaaaaatgaacatagaaaTA
1A-0124980B-T4-17_896	TTGAGCAGACACTTGTAGTTG
1A-0124980B-T4-17_1088	CTCTCTCCCTTGCCATGCTTTT
1A-0124980B-T4-17_1152	AGAAAATCCTTTGGTCTTCATT
1A-0124980B-T4-17_1344	GGGTGTTGTTCACCTCTGATT
1A-0124980B-T4-17_1408	CTATAATTTCTATTTTGTTTAGG
1A-0124980B-T4-17_1536	AGCTTCAGATGAGCATTGGG
1A-0075270A-T4-17_1580	CAAGGGCACTCTTTGAGGGC
1A-0124980B-T4-17_1580	GTTGACTATAAATTTGATAATG
1B-0124980A-T4-17_64	CACTTTTCTTAAACCCCCCTCC
1B-0075270C-T4-17_256	ACCCATGCCACCGCTGTTATT
1B-0075270C-T4-17_320	AATTGTTTTTTTACTACCGCTG
1B-0075270C-T4-17_384	TGAGTGGTCTTACGTACGAAT
1B-0075270C-T4-17_512	AGAGAAATTTTAGGAAAACC
1B-0124980A-T4-17_576	ATCCTGTTAATTCCTTCAGTAG
1B-0124980A-T4-17_640	TCTCCTGGACGCATTAACACC
1B-0124980A-T4-17_704	AGTATGTGAGATAAGCACTTA
1B-0124980A-T4-17_768	CACTTCCATCGATAAATTCCAA
1B-0124980A-T4-17_832	TCTTTGAAAAATGAACATAGA
1B-0124980A-T4-17_896	TGAGCAGACACTTGTAGTTGC
1B-0075270C-T4-17_1088	TAACCACAACGCCGTCTCGTT
1B-0124980A-T4-17_1088	TCTCTTCCTTGCCATGCTTTTG
1B-0075270C-T4-17_1152	CATTGAGGACATGCATGCATA
1B-0124980A-T4-17_1152	GTAAATCCTTTGGTCTTCATTC
1B-0075270C-T4-17_1280	GTTTTTAGAAAAACGTCGATT
1B-0075270C-T4-17_1344	CAGTTTTGCATATACGCAAATT
1B-0124980A-T4-17_1344	GGGTGTTGTTCACCTCTGATT
1B-0075270C-T4-17_1408	CGGTTAACAAGATCTGGTTTT
1B-0075270C-T4-17_1472	AAGTAGAGATAATCACTACTC
1B-0124980A-T4-17_1536	AGCTTCAGATGAGCATTGGG
1B-0124980A-T4-17_1580	GTCGACTATAAATTTGATAATG
1D-0075270B-T4-17_0	GTTAGTGTAGTTTGTTGAGAT
1D-0075270B-T4-17_64	AAAAAGTCATGTAAACACTTA
1D-0075270B-T4-17_128	TGAACAATAGACATCCATGTG
1D-0075270B-T4-17_192	GAATTAGGCTTATCAACTTTAT
1D-0075270B-T4-17_256	TGGCTGAGTTAGGCTTCTTGA
1D-0075270B-T4-17_320	CCCTGACTCCTCTACAAAGTAT
1D-0075270B-T4-17_384	ACATTAGTATTACTGGTAAACC
1D-0075270B-T4-17_448	AACAACATTAGTATTTGGCGA
1D-0075270B-T4-17_512	GATGAAATATGTATCCAGCGG
1D-0075270B-T4-17_576	CCGAGGCCCAGATCCGCGTC
1D-0075270B-T4-17_640	AGAGTACATCCGCACGAGAG
1D-0075270B-T4-17_704	ATGCAGTCTAGTTCCCCTTAA
1D-0075270B-T4-17_768	AATTTGGATCGCCACTGTTTC
1D-0075270B-T4-17_832	TACAAGAAAATCTGCTCTCCC
1D-0075270B-T4-17_896	AAGGGGACGACGAGAGGTT
1D-0075270B-T4-17_960	GGGGAGAGATATATTGTTTGC
1D-0075270B-T4-17_1024	CGGGAGGAGGTGGGATTGG
1D-0075270B-T4-17_1088	CGCTCGGGTGAGATGATGCA
1D-0075270B-T4-17_1152	AAGGGAGGAGGGGAGCATG
1D-0075270B-T4-17_1344	GAGCGTGCGATGCTTTTCTCC
1D-0075270B-T4-17_1408	AAACCAACATGATGATAGGTG
1D-0075270B-T4-17_1472	TAACAACTGCTACTACATTAGA
4A-0939290-T4-18_0	AATACACATGTGAAATGTCTCT
4A-0939290-T4-18_64	GAGATTAAAACTAGTCGATTG
4A-0939290-T4-18_128	ATCAAATTTGTGGCAAAATTT
7A-1820410-T4-18_128	GTGGGGTTTTCACTGAGGTTT
4A-0939290-T4-18_192	ACACCTTTGAGATAGAACATA
7A-1820410-T4-18_192	AGGTCGATGCCAGTGTAGCCA
4A-0939290-T4-18_256	GCATAAGCTATAACATGATCAT
7A-1820410-T4-18_256	GACTGTAATTTACCATAAATAA
4A-0939290-T4-18_320	AACATCATGTAATCAGTCATCT
7A-1820410-T4-18_320	GTTGTGAGAAAGCTGTAGAG
7A-1820410-T4-18_384	ACCGTATGTTCAACACTATCCT
4A-0939290-T4-18_448	AGCTAGTCAGACACAACCTTA
7A-1820410-T4-18_448	GTGTTAGGTTCGGCTGTAATT
7A-1820410-T4-18_512	TGGGTTCTGCGTTGTGAGAAA
7A-1820410-T4-18_576	AGTTTGTAAAAACAGATGTTC
4A-0939290-T4-18_640	AGTAAATTACAGCTGAACCTA
7A-1820410-T4-18_640	GGCCAAGACTGTGTTAGGTT
4A-0939290-T4-18_704	CGAGAATAGTGACCAAAACC
7A-1820410-T4-18_704	AAATTAGTTATGGGATATGCGT
4A-0939290-T4-18_768	ACACATAGCTATTCTGTGTCGG
7A-1820410-T4-18_768	TGGCGTGTCACGTTTGTAAAA
4A-0939290-T4-18_832	AACATAGTCTTGGCATCCGTC
7A-1820410-T4-18_832	ATTGACGGATGCCAAGACTAC
4A-0939290-T4-18_896	CGTTTTACAATCGTGACACAC
7A-1820410-T4-18_896	TAGCCGACAAAGAATAGCTAT
4A-0939290-T4-18_960	AACTCACAACCAATAACTATTT
7A-1820410-T4-18_960	TAAAGATTTTGGCGTGTCACC
7A-1820410-T4-18_1024	TTAACCTTCATCAACAGAAGG
7A-1820410-T4-18_1088	GTAAGAAAGTTGTAAAGCCAT
4A-0939290-T4-18_1152	GCTTTCTCGCAACGCACAACC
7A-1820410-T4-18_1152	TGAAATGCCCCTGAAGAGCC
4A-0939290-T4-18_1216	TGAATTACAGCCGAACCTAAC
7A-1820410-T4-18_1216	AGGTTGATTTATTAGCTATAAA
7A-1820410-T4-18_1280	TCGAGTTGATTGGTGGGACTG
4A-0939290-T4-18_1344	CCTGTGCTGGCTCTACAGCTT
7A-1820410-T4-18_1344	CACCAAAAGACTGACATATAA
4A-0939290-T4-18_1408	CCCACTGTTGTTTCCTTATAAA
7A-1820410-T4-18_1408	AATTGTGCACATAAACATGCA
4A-0939290-T4-18_1472	GCGTAGTTTTGGCTACACTGG
7A-1820410-T4-18_1472	TAGATATATCACATCCAGTCTG
7A-1820410-T4-18_1536	GTGTAAAATACAAATCAAGAA
7A-1820410-T4-18_1580	ATGTAGAATACAAATCAAAAA
7D-2044770-T4-18_128	GTGGGGTTTTCACTGAGGTTT
7D-2044770-T4-18_192	CAGGTGGATGCCAGTGTAGC
7D-2044770-T4-18_320	CACGTCAGGGGTTTTGGCGC
7D-2044770-T4-18_384	GTAGCCAAAACTACACTTCAC
7D-2044770-T4-18_448	AGAAAACAGCTGTAGGAAAG
7D-2044770-T4-18_640	GTAATTCACCAGAAAACAACT
7D-2044770-T4-18_832	AGGTTTGGCTGTAATTCACCA
7D-2044770-T4-18_896	TATGAGTTGCAAGAAAGCTCT
7D-2044770-T4-18_1088	GTAAAGCTAACACAGTCGCGT
7D-2044770-T4-18_1152	ATTATCCCCATGTAGCCAAAAT
7D-2044770-T4-18_1280	GTGGCCGCGAGAGTAAATGT
7D-2044770-T4-18_1344	TAATGATAAGGATCTCATTGAA
7D-2044770-T4-18_1472	TGGCCTTTTCCATCTATTTCAC
7D-2044770-T4-18_1536	TACGAGTAATGATGGCATACA
7D-2044770-T4-18_1580	CAATCGACTAGTTTTAATCTCA
4A-0991190-T4-19_0	TGAATCGCAACCACGTACTAA
7A-1820640-T4-19_128	ATTTTTCTTGGGGTGGAACCA
4A-0991190-T4-19_192	GTGTGAGGTGTTTTTGTTTTG
7A-1820640-T4-19_256	CTCTTTGTTCTTGTGTTGATGG
7A-1820640-T4-19_320	AAAAATATCTAGAAACAAGGT
4A-0991190-T4-19_832	CCTGCCCCAGTTCATTCTCCG
4A-0991190-T4-19_1024	ATCCATGGATCGAATATTCTTA
7D-2014520-T4-19_512	TTTGGGGCTCCCACACGGAG
7D-2014520-T4-19_576	GTAAACCGGACCTGCAATGG
7D-2014520-T4-19_640	AAAAAGTTAGTACAATATATTC
7D-2014520-T4-19_832	CGGAAAAAAAGGAAAAGAA
7D-2014520-T4-19_1408	TATAGCTGGGGTTCGGGGGTA
7D-2014520-T4-19_1472	AGGGTTTTGGCATCTACCGTC
7D-2014520-T4-19_1536	GATGACACCTCACACTTTGTT
7D-2014520-T4-19_1580	TTATGGCAATGAGAAGGCATT
4A-0939360-T4-20_0	GCCCAGTCAGCCAGCATGCG
4A-0939360-T4-20_64	GTGCTTCCTCTGCCGTATTGTG

										7A-1820630-T4-20_64	GATGGGTGGATGGTGGGTTC
										4A-0939360-T4-20_128	GTGCTGCTCCACATGTGTGCT
										7A-1820630-T4-20_128	TTTATGACAAGAGGGAGGTT
										7A-1820630-T4-20_192	AACACCTCACGCTTAGATTAG
										4A-0939360-T4-20_256	CGACATGCATCGATGGCTACC
										7A-1820630-T4-20_256	GGATTGCTCTTGGGAATACTT
										4A-0939360-T4-20_320	AAAACTCTGTGCATTCGCTTT
										7A-1820630-T4-20_320	TTAGAAACAAGACGACGGCA
										7A-1820630-T4-20_384	ACCAACTTTTATGCCATGTGTT
										7A-1820630-T4-20_448	GCATTTCTTTTTTATTTTATTTT
										7A-1820630-T4-20_512	TCACCTGCCTTTTGCAGATTGT
										7A-1820630-T4-20_576	TGGTTGTTTGTAGAAGGGAG
										4A-0939360-T4-20_640	CGCGGGAGCACTACATTGTG
										4A-0939360-T4-20_704	AAATTTCAATGTGGTTGACCA
										7A-1820630-T4-20_704	CCGCGGGAGAATGGGTGGA
										7A-1820630-T4-20_768	TGTCGAAGAGGTTATGATCAA
										4A-0939360-T4-20_832	CAGCACTACGACCGCAGATCT
										7A-1820630-T4-20_832	ATCCCTTATATATTACAATCAAA
										4A-0939360-T4-20_896	TTGCATCTACACACTCCCTTCT
										4A-0939360-T4-20_960	CTCAGGGACATGCAATCTCTA
										7A-1820630-T4-20_1152	TGTACACGTTCACAGGAGTGA
										7A-1820630-T4-20_1216	AAAAACACACCAAATAAACAG
										4A-0939360-T4-20_1280	TGCTTTGTGCTACATGCGTGG
										7A-1820630-T4-20_1280	ACTTTTCGTACCCATCGGATA
										4A-0939360-T4-20_1344	ACACATGGCACAAAAGTTGGT
										7A-1820630-T4-20_1344	TGCGTGCGTGCTCCAATCACG
										4A-0939360-T4-20_1408	CCGCCGTCGTCTTGTTTCCAAT
										4A-0939360-T4-20_1472	GCCACCAGGCCACCGGCGGG
										7A-1820630-T4-20_1536	TCACAGCACACCGTACACGTG
										7A-1820630-T4-20_1580	GGCCGACGCTGAGTCGACTG
										7D-2019550-T4-20_0	GGCGCCGGCGCTCGCCACGA
										7D-2019550-T4-20_64	GGGTGCTGGGTTCAGTGGGG
										7D-2019550-T4-20_128	TGGTGGTAAAGATGAGAATTT
										7D-2019550-T4-20_192	ATTCTCTTTCTTCTTGTGTTAAT
										7D-2019550-T4-20_256	TTCAGAAAAAATAAATTTAGA
										7D-2019550-T4-20_320	GCAAGTTCTATGCTTTTAAAA
										7D-2019550-T4-20_384	TAAACGTAATATTTTTTTATAG
										7D-2019550-T4-20_448	AGATTATCGCCGAGTTTGTTA
										7D-2019550-T4-20_640	TTCATAGGATAAGTGTATGCG
										7D-2019550-T4-20_704	CCATATAAACGGAGTGTACAG
										7D-2019550-T4-20_768	ACCCATCGATGAATGCGACGG
										7D-2019550-T4-20_832	TCCGATCACGTagcagcagcagc
										7D-2019550-T4-20_896	ATCAGCAACAATAATGCAGCC
										7D-2019550-T4-20_1088	CGGCCGTGCATATCATCTCTGA
										7D-2019550-T4-20_1152	CAAAGTACTTAGTCGAACGTT
										7D-2019550-T4-20_1280	CATGCTACGCCCTATAGCACCT
										7D-2019550-T4-20_1472	GTCAATAGTACCCGCAGGCAT
										7D-2019550-T4-20_1536	CCCCTTATTGGTAGATAAAAGT
										7D-2019550-T4-20_1580	TCTTTTTTGGAATTTTGGAAC
										6A-1557090-T4-21_192	CGCGACCGACTCCTCCGGCA
										6A-1557090-T4-21_256	ACCGGCAAACGGAAGATGAT
										6A-1557090-T4-21_512	TCGTTGACTGTGCCATCGTTT
										6A-1557090-T4-21_704	ATGTGTCACGGCTGCATTTTA
										6A-1557090-T4-21_832	ACAGGCATGTTTCGCCTTTCA
										6A-1557090-T4-21_896	TGGAGGGGGGCTATAGTTGT
										6A-1557090-T4-21_960	TAACTTTCTTGCCATCGATGAT
										6A-1557090-T4-21_1024	TCTCTGGCTTGTGCCCACTCAT
										6A-1557090-T4-21_1088	TTGCACATCTAGCACGCCCCG
										6A-1557090-T4-21_1152	CGCGCAATTGGGAATTTTGG
										6A-1557090-T4-21_1280	GTCAGCAAAAACTGAAAAAC
										6A-1557090-T4-21_1344	TAAAAAAATGCTACATGCAAG
										6A-1557090-T4-21_1408	GGAAAGCTTAAGGGCAACTC
										6A-1557090-T4-21_1472	AATGGACAGACGACATGGCC
										6B-1667010-T4-21_512	CTTGCTGAAGTACAGGTTCGG
										6B-1667010-T4-21_704	GGCCCGCGTGCAGGGAACCG
										6B-1667010-T4-21_768	AAATAAAGCATGCAGCACGA
										6B-1667010-T4-21_832	TGAGCCAGCCGGCCAACTTG
										6B-1667010-T4-21_1024	AATTGTCGCAGGCTGAGAGCT
										6D-1748090-T4-21_0	CTGAAAATCACCACCATTTCAT
										6D-1748090-T4-21_128	CTCGCCCAGGCATGCACCTGG
										6D-1748090-T4-21_192	CTGTACAATTAAAATATACGCC
										6D-1748090-T4-21_256	AGCTCTGACCACGCGGAGGA
										6D-1748090-T4-21_448	CGTGGCGAATGATCGGAACG
										6D-1748090-T4-21_1152	GCCGCAACCGACTCCTCCGG
										6D-1748090-T4-21_1216	GAATCGACGAACGGAAGATA
										6D-1748090-T4-21_1280	AGGAGACGGCTGCTTTGTGC
										6D-1748090-T4-21_1472	GGCTGGGTTCGTTGACTGTG
										2A-0276380-T4-22_192	CCCACTGGCCCATGTACATAC
										2A-0276380-T4-22_256	TATTTGTCATGCAATGTACGTA
										2A-0276380-T4-22_448	CAAGAGTGGCGATCTATTGAT
										2A-0276380-T4-22_512	ATGGATACCCATGCGTTTACC
										2A-0276380-T4-22_576	ACGGGATGAACTCGACGATC
										2A-0276380-T4-22_768	TGTTGAAGTTCATCAATGTAA
										2A-0276380-T4-22_832	CTAGGATCCGTGTAGTGATGG
										2A-0276380-T4-22_896	AAGCATTTGCTACAGCTCACG
										2A-0276380-T4-22_960	TGTGATATTGAATATTTGAGCA
										2A-0276380-T4-22_1024	TAGAAAAGTGGTGTAAGTATT
										2A-0276380-T4-22_1152	TGGAAAAAGATGTAGGTGCA
										2A-0276380-T4-22_1216	GTCATCTTCCAGGAAGTCATC
										2A-0276380-T4-22_1280	TGCTCCCCAAAAACCCAATTG
										2A-0276380-T4-22_1344	TAAGGCAGCATGCACACGTTG
										2A-0276380-T4-22_1408	TGAGGCCAGGCTGACGGCGA
										2B-0427630-T4-22_192	CTGGCCTGGCCCACTGGCCC
										2B-0427630-T4-22_384	AACTTTTAGAAGGGAGACATA
										2B-0427630-T4-22_576	GATATCCATGCGATTACCACAT
										2B-0427630-T4-22_640	GGATGAACTCGACGATCAAAT
										2B-0427630-T4-22_704	ATCTTGTGGTTTTGCATGTCG
										2B-0427630-T4-22_768	GCAAATCTACTCAGAGTTGTT
										2B-0427630-T4-22_960	CATTTGCTACAGCTCATGCGA
										2B-0427630-T4-22_1024	ATATTGAATATTTGAGCAATTT
										2B-0427630-T4-22_1088	AAAGTGGTGTAAGTAGTGATG
										2B-0427630-T4-22_1280	CGTCCGGAAAGTCATCGTGCT
										2B-0427630-T4-22_1344	CCCAAAAAACCATTTGCCTCA
										2B-0427630-T4-22_1408	CAGCGCGCACACGTTGCATAG
										2D-0502570-T4-22_128	CAAAAACTCCAGCTTAGATGA
										2D-0502570-T4-22_192	CACTTAGATTAGATGTACAAAT
										2D-0502570-T4-22_256	AGTATGCCACAATAAGCTAGT
										2D-0502570-T4-22_448	CCCCCTTCGTTATATATGCGGC
										2D-0502570-T4-22_1408	GCGTGGCGTCATAGTGGTTTA
										4A-0978150-T4-23_128	CTCTCCCAAATTCCTCTCCTTT
										4A-0978150-T4-23_192	CCAAGCCTTTGTGCGTCGAGT
										4A-0978150-T4-23_448	TCTATCACCGCTCTGGTCAAAT
										4A-0978150-T4-23_512	AGTCCTCTCGTAGGGAAAATG
										4A-0978150-T4-23_960	AGTAATGATACAGTGGCAGTA
										4B-1105190-T4-23_0	TAAATatgaacaagaaaataaataa
										4B-1105190-T4-23-2_256	AGGTAAATTAGTCAATCAATTA
										4B-1105190-T4-23-2_448	TGTCCTTGTGTTCACACTTAAA
										4B-1105190-T4-23-2_512	CTTGATTACATTATGACAGTGT
										4B-1105190-T4-23-2_576	CTGGGACAATAAATTTAATAA
										4B-1105190-T4-23-2_640	TTAGAAAGTAAACCAAATCTA
										4B-1105190-T4-23-2_704	TGTACTGCCGTCTGAAATTGAT
										4B-1105190-T4-23-2_768	CTGTGTGATTTTTTTAGGATCA
										4B-1105190-T4-23-2_960	GCTATTTTGTTGTCACTTGCAT
										4B-1105190-T4-23-2_1024	ATTGAGATCAGCATGGAAGTA
										4B-1105190-T4-23-2_1216	GAAATGTCCGTGTTCTGGATG
										4B-1105190-T4-23-2_1280	GAATAATAGTGCATAGTTTACT
										4B-1105190-T4-23-2_1344	TGTGCGTTCCAGATGTAAACA
										4B-1105190-T4-23-2_1408	AGCAGGCATACGACCTTAATT
										4B-1105190-T4-23-2_1472	CTTCATCTGGTACTATAATTCT
										4B-1105190-T4-23-2_1536	CTTATTTATGAATCAATCTGGA
										4B-1105190-T4-23-2_1580	TCTCCTGAAAATAGTATTTGCT
										4B-1105190-T4-23_63	AGGTGGCTCTAATGCCAACTG
										4B-1105190-T4-23_126	AAAAAGCTCGCCGATGAGGT
										4B-1105190-T4-23_315	GTTTTCAGCAAAAATAGAAGT
										4B-1105190-T4-23_504	CACCACCATAAATTCACATATA
										4B-1105190-T4-23_1197	TTTATTGTTGCTGTATGTTATG
										4B-1105190-T4-23_1260	TACTTCCATACCTACGTTTTATT
										4B-1105190-T4-23_1323	GTGTTACTATATTATGCAATCTT
										4B-1105190-T4-23_1386	ACTTCTCTAATAGTAATAAATT
										4B-1105190-T4-23_1449	TTTTCTTTATAAAAAACATTGC
										4B-1105190-T4-23_1701	TGGAAGACGCAGAAGAAAAT
										4B-1105190-T4-23_1764	TCGCAGCATATATAAGTACTTC
										4D-1175370-T4-23-2_64	CCATTTCATGAAAGATCGGGC
										4D-1175370-T4-23-2_192	TGGTGCTGGTGGGCTCGACC
										4D-1175370-T4-23-1_320	TTCCAGGTAAATTAGTCAATCA
										4D-1175370-T4-23-1_384	ATCTCCTGGATTTATCAGCTTA
										4D-1175370-T4-23-1_512	GGTATGTCCTTGTGTTCACACT
										4D-1175370-T4-23-1_576	ACTCGATTATATTATGACAGTG
										4D-1175370-T4-23-1_640	ACTGGGACAATAAATTTAATAA
										4D-1175370-T4-23-1_704	GTTAGAAAGAAAACCAAATCT
										4D-1175370-T4-23-1_768	TATTGTAGTGCCGTCTGAAATT
										4D-1175370-T4-23-1_832	CTGTGTGATTTTTTTACGATCA
										4D-1175370-T4-23-2_832	TGGTGGCTTCACTCCCGGGTT
										4D-1175370-T4-23-1_1024	TGCTATTTTGTTGTCACTTGCA
										4D-1175370-T4-23-1_1088	GATTGAGAGCAGCATGGAAG
										4D-1175370-T4-23-1_1216	CCGTGCTCTGGATGAGCGCTG

4D-1175370-T4-23-1_1280	AGTGCATAGTTTACTGTCATCC
4D-1175370-T4-23-1_1344	GTCCAGATGTAAACAACTTATT
4D-1175370-T4-23-2_1344	TATATATAAAGCGGGGCGAAA
4D-1175370-T4-23-1_1408	CAGACATACGACCTTAATTGTT
4D-1175370-T4-23-2_1408	AAGCAAGTTATCTATTCAGGT
4D-1175370-T4-23-1_1472	TCATCTGGTACTATAATTCTCG
4D-1175370-T4-23-2_1472	TACGAGAGGACTAGGAAAAA
4D-1175370-T4-23-1_1536	TATTTATGAATCAATATGGATG
4D-1175370-T4-23-1_1580	TCCTGAAAATAGTATTTGCTAT
4A-1004820-T4-24_0	TATCCGTAAGGACCCAGACCT
4A-1004820-T4-24_64	GCAAATCCTTTAGGACTGTGG
4A-1004820-T4-24_128	CCCACACCTGAAATCAAGGAG
4A-1004820-T4-24_192	GGCTGATGTGGAGCAAATCAG
4A-1004820-T4-24_256	AGTTGATTTATGATTAGCGGG
4A-1004820-T4-24_384	ATTTGCAAAGGAAATATTCCT
4A-1004820-T4-24_1216	GAAAAAATCCGAAACTTTGTA
4A-1004820-T4-24_1472	CCTATAAAATTTCTATCCTATGG
4A-1004820-T4-24_1536	GATTAGAGGATGCACACATAC
4A-1004820-T4-24_1580	GGTGCTGCATCTAAGTTTACT
4B-1041080-T4-24_0	TTCGCTAGCTGACTGAGAAAC
4B-1041080-T4-24_80	TTATAGTATCGGAAATAGTAAA
4B-1041080-T4-24_620	CTTCTCTAAATTTCATTGTGGT
4B-1041080-T4-24_685	CCACCGTTGATCCTGAACCAA
4B-1041080-T4-24_1010	AGTATCATGTAAATGACTGTAA
4B-1041080-T4-24_1075	CTCAATCAATTCATCCGTTAAA
4B-1041080-T4-24_1140	ATATCTTGCACGCTAGACCTAG
4B-1041080-T4-24_1400	GTCGGCTGAAGTTTCATCGTG
4B-1041080-T4-24_1465	TCATATATGGACTATAAAAATG
4B-1041080-T4-24_1527	CCATATAATTCACGTACAAGAA
4D-2109110-T4-24_0	TTCGCTAGCTGAGACAGAAAC
4D-2109110-T4-24_61	TGAGTTGCGGTCTGCACGCGT
4D-2109110-T4-24_122	CCAGGCTGCACCTTGCGCACG
4D-2109110-T4-24_183	AATCCTAAATGTACGATTAACC
4A-1004810-T4-25_0	AACTATGGTAAAAATACAAAA
4A-1004810-T4-25_54	TGGAAGTCCACTTCTTACAGA
4A-1004810-T4-25_444	GGGGAAAGATTAATTGGCGTT
4A-1004810-T4-25_508	TATTGCAAATAAATTATCCGTG
4A-1004810-T4-25_572	TAGTGAGTGTCAACGTACATT
4A-1004810-T4-25_636	AAAACCATCATGTATAGTCACT
4A-1004810-T4-25_764	cttgagagtccactctcatctttttcatg
4A-1004810-T4-25_828	TAGGCTATAAACCTAATATTGT
4A-1004810-T4-25_892	TTATTTGAACTGCTTGTTCCAA
4A-1004810-T4-25_956	TTATAATAGTAGTACTTTCACA
4A-1004810-T4-25_1020	GGCTTGCATGATGTTTTCTCG
4A-1004810-T4-25_1084	TTTCATTATAGGCTCTTTCATA
4A-1004810-T4-25_1148	TAATTAAATACAAAGCATAATG
4A-1004810-T4-25_1212	TTAATCGGGTTCTTATACAATC
4A-1004810-T4-25_1276	GAAAACTCTGTAGATAGACAA
4A-1004810-T4-25_1340	AACAATATATAACATTGTGTGT
4A-1004810-T4-25_1404	TAAAAGCGTTTTTGCACTAGT
4A-1004810-T4-25_1468	CATGTCCATAGATCTTGGGCT
4A-1004810-T4-25_1532	GCCAATTATAATTGAAGGAAA
4B-1041090-T4-25_64	ATCGGTGCGTTCATTTATAGC
4B-1041090-T4-25_128	TATGATATTGTTTCCTTCAATTA
4B-1041090-T4-25_192	CTATATATATAGCATATATGAAC
4B-1041090-T4-25_320	gtcatctattttgaaacagagggagta
4B-1041090-T4-25_1024	ggagcagctggaggaTTCTTTTTT
4B-1041090-T4-25_1088	CAATTAACTATCCTTTAGCTTAT
4B-1041090-T4-25_1152	TGCTTTGTATTTTATTAGTGTT
4B-1041090-T4-25_1216	AATGCCCGTGTGTTGCAATGT
4B-1041090-T4-25_1280	CAGTTGTGAAAGTACTACTATT
4B-1041090-T4-25_1344	TCAAATCAAGAACATATCCTCA
4B-1041090-T4-25_1408	CAAATAAATATAATAGGCTTAT
4B-1041090-T4-25_1472	CATAAAAATAATGGGAGTGGA
4B-1041090-T4-25_1536	AGTCTAGGAAAGGAATTATTT
4B-1041090-T4-25_1580	CCTAATTTCGTCCTAAAAATCC
4D-2109100A-T4-25_64	CGTTCATTTATAGCCCAAGATC
4D-2109100A-T4-25_128	TCCTTCAATTATAATTGGCTGT
4D-2109100A-T4-25_192	CCCAAGACCTATGGACATGCA
4D-2109100A-T4-25_256	TCCTTCAATTATAGTTCCCATG
4D-2109100A-T4-25_320	CATGCTCAAGTGCTCCCGAAT
4D-2109100A-T4-25_384	TGGTAATATGCATTTTATTCATG
4D-2109100A-T4-25_448	TTAGTGCTACATTTTAAGTTAA
4D-2109100A-T4-25_512	CAAAAAAAGCATGTTGGGTCT
4D-2109100A-T4-25_576	GCAAGCAAAAAACACACTACA
4D-2109100A-T4-25_640	TTAAAATAGTTTTGGTAGCGA
4D-2109100A-T4-25_832	cagaggcaggggtcatccttcttttcaa
4D-2109100A-T4-25_960	ggagtaggctctcaagtaagagcctag
4D-2109100A-T4-25_1024	GAGAAAGAAAGAGAGAAGG
4D-2109100A-T4-25_1088	ACTATACATGATGGTTTTATAT
4D-2109100A-T4-25_1152	GCACGATGACACTCACTAAAG
4D-2109100A-T4-25_1216	GATAATTTATTTGCAATTATCG
4D-2109100A-T4-25_1280	CTAATTAATCTTTCCCCCAAAT
4D-2109100A-T4-25_1344	ATAATTGCCACCTAATTACAGT
4D-2109100A-T4-25_1408	CATTTATTTGTGGGGGAAAAA
4D-2109100A-T4-25_1472	TGAGAAGTGGACTTTCAAACA
5A-1214150-T4-26_128	GGCCGGCCGGAGCAAGAAA
5A-1214150-T4-26_320	CGGAGCCCGAGACCTACAGG
5A-1214150-T4-26_512	GTATTTTTTGACGGGGACGGT
5A-1214150-T4-26_576	AGGTAATCTGTAGTATTTTTCT
5A-1214150-T4-26_704	CCGACGCGTCCGGCTAGCTTG
5A-1214150-T4-26_768	GACTAATTAAAAGAAATTAGG
5A-1214150-T4-26_832	GCAAACCAAACCTTACACAGT
5A-1214150-T4-26_1152	TCTAAAAGCTCCTGAATGATC
5A-1214150-T4-26_1216	GAGATTTTTTTATATTTTTTTG
5A-1214150-T4-26_1280	GAAAACGGCAAAAGTGCTGC
5A-1214150-T4-26_1344	TTGGGTTGATTGATTTGGTGC
5A-1214150-T4-26_1408	ATGACGCGACGCATGTCTTGG
5A-1214150-T4-26_1580	GGATCAGGTCGCCGGCGCCC
4B-1056380-T4-26_64	GGTACCTACCTGGCCGGGTAT
4B-1056380-T4-26_320	TAGAGCTGATACGAATGTCGA
4B-1056380-T4-26_512	TTTTTTGACAGAGACGGTCAC
4B-1056380-T4-26_768	ATTTAGAAACGGAGGGAGTA
4B-1056380-T4-26_832	CTGGCCATAGTTTTGTCCCGC
4B-1056380-T4-26_1024	TTTCTTGGGGAATCGAATTGT
4B-1056380-T4-26_1088	CTCGATCGGAGACCCGTTCGC
4B-1056380-T4-26_1280	GGATCAGGTCGCCGGCGCCT
4B-1056380-T4-26_1408	GAGTTAGTTTTTCATACGGAG
4B-1056380-T4-26_1472	ATTGGACAAGCATAGAGTATT
4B-1056380-T4-26_1536	AATCTGTTGGGACGGACCAAA
4B-1056380-T4-26_1580	CCCGCCTTGCCTCTTCCCCGG
4D-1124700-T4-26_0	GGCTCCCCGCCGCCCGTCCTG
4D-1124700-T4-26_128	CCAGGTATCTATTCTACGTGGC
4D-1124700-T4-26_512	GGGACACCCGGTCGGTCCCG
4D-1124700-T4-26_704	GAATGGAGGGAGTATTATTCT
4D-1124700-T4-26_768	TATATCTGGCCATAGTTTTGTC
4D-1124700-T4-26_960	CCCGTTTCTTGGGGGATCGAA
4D-1124700-T4-26_1024	GCCGCTCGATCGGAGACCCG
4D-1124700-T4-26_1280	TGGCACCTTCGAGCGGGTCA
4D-1124700-T4-26_1344	GTGTACGATGGCTAGCCTGGT
4D-1124700-T4-26_1408	TCGCCCCCGGTTGACGTGATA
4D-1124700-T4-26_1472	CCTGTTTTGGACTTGATTAATG
4D-1124700-T4-26_1536	GCAAAGCTCTGTTTCTCTTGT
4D-1124700-T4-26_1580	AGGGATCGGGCGATCGAACT
4A-0989860-T4-27_0	AATTTTGTTTGTATTGTGGCCT
4A-0989860-T4-27_64	CTGCCCATGCTGGACTTTGAT
4A-0989860-T4-27_128	CTATGAGTATATATCGGTGTAT
4A-0989860-T4-27_192	GTTTCAGTAAGGAAGTATTTG
4A-0989860-T4-27_512	AGAAAGAAAAAGAAAATCCT
4A-0989860-T4-27_576	TTTTGGAGATTTCCTGAGGTA
4A-0989860-T4-27_640	TTTTTTTAAAGGAACCATTATT
4A-0989860-T4-27_704	TCACACAACCATATATTATTAA
4A-0989860-T4-27_768	AGAGGCCATCAGAAAAAGGA
5B-1330970-T4-28_0	GTTGTGCTTCTTCAGTAAGCT
5B-1330970-T4-28_64	GGAGAACGGGTGAGCTGCAA
5B-1330970-T4-28_128	TATCGACAAGTGGCCTGGGTG
5B-1330970-T4-28_192	CCACATCTGCACCTAACGTACT
5B-1330970-T4-28_256	TCCTAGTTTCAGACTACCTTTC
5B-1330970-T4-28_320	TTTTCTTTTGGACTAAGTTGAT
5B-1330970-T4-28_384	TGTACACTTGGCCACTTGGCC
5B-1330970-T4-28_448	TATACTCCCTGTGATCTAGAAT
5B-1330970-T4-28_512	TGGCACTTTGACTAGCAATCT
5B-1330970-T4-28_576	TAATATGTTATTCAAGTCTTTTT
5B-1330970-T4-28_640	TGGTATTCCTCTTCTTTTATTTA
5B-1330970-T4-28_704	TAATGACTTGTATATTCTCCGA
5B-1330970-T4-28_768	TACACTACATGATACTCATGCA
5B-1330970-T4-28_832	CTTCCCTTTCCATGGTGGCTA
5B-1330970-T4-28_896	CTGAAGCTGATGTAAGCATAT
5B-1330970-T4-28_960	CGCACGTGGCCACTTGGACA
5B-1330970-T4-28_1024	AGATGTCTTTTTTCTTTTCTTT
5B-1330970-T4-28_1088	TACTCCTTGGATGTATGGCTCA
5B-1330970-T4-28_1152	CTTAACCTCCGGTATACTTGAG

										5B-1330970-T4-28_1216	ttgatttattgatttatttatttgcttattt
										5B-1330970-T4-28_1280	GCATATTCTCCGTAGAACTCTG
										5B-1330970-T4-28_1344	CAGGTTTCCGTTGTTTGTTT
										5B-1330970-T4-28_1408	TGCACCAGATGTGCTACTCGA
										5B-1330970-T4-28_1472	ACAGCAGGTAGCAGCTTCCA
										7A-1797990-T4-29_0	GCCAGGCCCAACATGTGAGG
										7A-1797990-T4-29_64	TGCAGATGCACCAAGTAGGA
										7A-1797990-T4-29_128	GTTTATATTTTTAAATATTCTAA
										7A-1797990-T4-29_192	ATGTTCATCCCAGTTTAAAAAA
										7A-1797990-T4-29_256	TCACAGGGTCAAAAATTTGTT
										7A-1797990-T4-29_320	GCATAATAAAAAATAAATGTCT
										7A-1797990-T4-29_384	TAACATTCGAAATAAATGTTCC
										7A-1797990-T4-29_448	CAACGTGTATTAGAAAAGACA
										7A-1797990-T4-29_512	ATAAAACCGCCCCCCCCTAA
										7A-1797990-T4-29_576	CCACATGGAAGGTTCTAAAAC
										7A-1797990-T4-29_640	TTGCGCCAACCTATTTAGTGG
										7A-1797990-T4-29_832	AGGAGGCACTGTAGCATGCA
										7A-1797990-T4-29_960	AGCTTCCTACAATGATGTCAC
										7A-1797990-T4-29_1024	AGCCACAGCCTCCCCATCTTC
										7A-1797990-T4-29_1088	CTATTAAGCGGCCCAAGAATA
										7A-1797990-T4-29_1280	AGTCAAGGCAGTGCGAATGC
										7A-1797990-T4-29_1344	CTTCGAAAAGGAAAATTGGG
										7A-1797990-T4-29_1408	TCTATGGACTAAAATTCCACAT
										7A-1797990-T4-29_1472	AGCCGCTCACCGCCGTGCCTC
										7B-1872660-T4-29_0	CGTGCGTGGCAACGAACTGC
										7B-1872660-T4-29_64	tgtgtggcaggagcggtggggagtgC
										7B-1872660-T4-29_256	ATGCACGCATGCGGAATTTTT
										7B-1872660-T4-29_320	GAACGCAATTCCCAATTTTCTT
										7B-1872660-T4-29_384	AATCGTGCACGCATTCACACT
										7B-1872660-T4-29_448	GGAGTCTGGCGATACATAGCC
										7B-1872660-T4-29_512	ACTAAATCAGTGACACTTAATT
										7B-1872660-T4-29_576	TAATACTAGTTTTTTCGCACGT
										7B-1872660-T4-29_640	TCGCCAACGAAGACAAGCAAT
										7B-1872660-T4-29_832	CATCGAAGGCGGCTCGATGG
										7B-1872660-T4-29_896	GCACGGAATCAACTGACACA
										7B-1872660-T4-29_960	CCTCTAGTGCTTCACTAAACG
										7B-1872660-T4-29_1024	TCCATGGACCGGTTCGAGACG
										7B-1872660-T4-29_1088	GTGTTTTTATTGGTTTTCTTCTT
										7B-1872660-T4-29_1216	TGAACATTTTTCAAATATATGA
										7B-1872660-T4-29_1280	GTGATGAAACATTTTTTATTAT
										7B-1872660-T4-29_1344	AATAATAAAATTTTAGCACGG
										7B-1872660-T4-29_1408	GCGAACAATATTTTTACACTG
										7B-1872660-T4-29_1472	AACTAAATTAATTTTTTAAATG
										7B-1872660-T4-29_1536	GAATATATACATGGCTCCTATA
										7B-1872660-T4-29_1580	CGTGCACGACCTGTGTGAGC
										7D-1967790-T4-29_0	TTAAATTTATAATGGGCTGGTA
										7D-1967790-T4-29_62	ATATTTCTTTGTATGTGAAATA
										7D-1967790-T4-29_124	TATTGAAAAATAAAACATGTT
										7D-1967790-T4-29_186	ATGTTTTGAATATTTTAGCATA
										7D-1967790-T4-29_248	AGAGTATTTGCTTATCCATGTT
										7D-1967790-T4-29_310	TTTTTTAGAAAGATAAACAAA
										7D-1967790-T4-29_372	AGTAGAATGATAAAATAACAG
										7D-1967790-T4-29_434	AAAGAAGGATAATAATCCACG
										7D-1967790-T4-29_493	GCACACAACGGTAAGCCTATC
										7D-1967790-T4-29_1084	TGCGTGGAAATGGACTAATGC
										7D-1967790-T4-29_1146	TCCCCAGTGAAAAAAGGAGG
										7D-1967790-T4-29_1208	TTGCTCGAAAGGTAACCGACA
										7D-1967790-T4-29_1270	TTAGGTTCCAAATGCGGGTGG
										7D-1967790-T4-29_1456	CCTCTCCACCGGATCTAAACC
										5A-1246480-T4-30-1_64	AAAACTTGTACTGTACTTTTAA
										5A-1246480-T4-30-1_128	AATGTTCTAATGTCACAAGAC
										5A-1246480-T4-30-2_128	ttttgggacggagggagtaTTAAAC
										5A-1246480-T4-30-1_192	ATGATATTTGGCACAATACAAA
										5A-1246480-T4-30-2_192	TTACTGTGTACTCAATATATATA
										5A-1246480-T4-30-1_256	TTTCAGATGGTTTAATCTTTTG
										5A-1246480-T4-30-2_256	ATGCTTAAATGCTACATCTGCA
										5A-1246480-T4-30-2_320	CGTTACTATATCAAATGGGACT
										5A-1246480-T4-30-2_384	TTAGTTTGTTTTCAATAGTTTC
										5A-1246480-T4-30-2_576	GATACACATGACTTACCATGTG
										5A-1246480-T4-30-1_640	TATTTCAATAGACAAAGTTCA
										5A-1246480-T4-30-2_640	CTATAGCAGTCAATACTGCGG
										5A-1246480-T4-30-1_704	GAACCTTTTTTTAGGGAGCTT
										5A-1246480-T4-30-2_704	CCACTTTAATCCAAAGTATCG
										5A-1246480-T4-30-1_768	GAGATGGCAACATTTAACTGG
										5A-1246480-T4-30-1_832	GGACTAGTTGAGGAGAGTCC
										5A-1246480-T4-30-1_896	ATGTCTCGAGAATATTCTTTTA
										5A-1246480-T4-30-2_896	TCCACAATACCTCGAGAGTGT
										5A-1246480-T4-30-2_960	GTCTGCAGCATCTGCTTTGAA
										5A-1246480-T4-30-1_1024	GTCCATTCGTTCATATGGGTGA
										5A-1246480-T4-30-2_1024	ATTATCATCTATTCTTTGTTTCA
										5A-1246480-T4-30-2_1088	GAAATGGCTTCTTCAGAAAAA
										5A-1246480-T4-30-1_1216	GTAGTTTTTTCTTAAGCACCG
										5A-1246480-T4-30-1_1280	TAAACATTGGCTTAAGAAGAA
										5A-1246480-T4-30-2_1280	ACATCATTTCAAGCAAATATTT
										5A-1246480-T4-30-1_1344	TACTCACTCTGTTCCATATTGG
										5A-1246480-T4-30-2_1344	GCATACATTGCTATGGAGCAA
										5A-1246480-T4-30-1_1408	ATTTTTATTTGAAGAAAAGTA
										5A-1246480-T4-30-2_1408	CTGAATTGTATAGCTGGGCTCT
										5A-1246480-T4-30-1_1472	TGAGGCAAATTTAGGGAAATA
										5A-1246480-T4-30-2_1472	TGAAGTTATGTTTTTCTTGTGT
										5A-1246480-T4-30-2_1536	AGTTCCTGGAGAATTGTGGTA
										5A-1246480-T4-30-1_0	TAAATTTGGCTTGAGCAGGTT
										4B-1062830-T4-30_0	GTCTTATGAGTTATAACATACA
										4B-1062830-T4-30_64	ACATAAAGATGTAATGTAATCT
										4B-1062830-T4-30_256	CGAGAATCATATGAGGAGAA
										4B-1062830-T4-30_384	agcagcagcagcagcaAGTAGGC
										4B-1062830-T4-30_448	CTGTCAGGGCCTCCTCCATAG
										4B-1062830-T4-30_512	AAAGACAGTGACGTAATGCA
										4B-1062830-T4-30_704	ATTGCATCATCATCTGCAGCAA
										4B-1062830-T4-30_768	CAGAAAGAAAACTTGCCCCAT
										4B-1062830-T4-30_832	TCAAACAGTCCAACTTCCTAA
										4B-1062830-T4-30_896	AAATATTAGTACCACTGGGTG
										4B-1062830-T4-30_1088	TATAAGCCCCAATACAGACCT
										4B-1062830-T4-30_1152	TCAAGACTGCAAAGATTAAaa
										4B-1062830-T4-30_1216	ctcagaaaataaTGAAGTATTTG
										4B-1062830-T4-30_1280	GTTCAGACTTTAAACATAACA
										4B-1062830-T4-30_1344	TCCTTTAGATAACTTGGGTACT
										4B-1062830-T4-30_1472	TGGTTTTGAACATTTCCACAG
										4B-1062830-T4-30_1536	AGAAGCTGCAAAGGAATGTT
										U-1151330-T4-30A_128	CACCTCTTTCCCTAAATTTGCC
										U-1151330-T4-30A_192	AAATACTAAAGAAATATAATAA
										U-1151330-T4-30A_256	TTCTTTACAAAAGGAGTATTTT
										U-1151330-T4-30A_320	CCCCCGTCCCAAAATATAAGAT
										U-1151330-T4-30A_384	TGCTTAAAGAGATGTTTAATA
										U-1151330-T4-30A_512	CTCTAAACACCTTTTACACACT
										U-1151330-T4-30A_640	ACGGGAACATCGCCTCCCCTG
										U-1151330-T4-30A_704	GGTTTGATCCTCCTTAAGCATA
										U-1151330-T4-30A_768	CCACCACCACACACACATACT
										U-1151330-T4-30A_832	TGTACCAAAGCTCTCTAAAAA
										U-1151330-T4-30A_896	TGTATTGAACTTTGTCTTCTGA
										U-1151330-T4-30A_960	ATTTGCAAACAAACAAAAGAT
										U-1151330-T4-30A_1024	ACACCAATACACTTTTGTATTG
										U-1151330-T4-30A_1088	TCATGAAAGGTAAGAAGAAAT
										U-1151330-T4-30A_1152	TAtactccctccgtcccaaaataagtg
										U-1151330-T4-30A_1216	CATTTAAAAGTACAGTACAAG
										U-1151330-T4-30A_1280	CTCTAACCTGCTCAAGCCAAA
										U-1151330-T4-30A_1344	CATATTTGCCATTGGATTTGGA
										U-1151330-T4-30A_1408	TCTTGATTACACTACCAAATAA
										U-1151330-T4-30A_1472	TAGTTCACAAGAACTCCTCTT
										U-1151330-T4-30A_448	ACGATTCACGAGTGTCGAAG
										U-1151330-T4-30A_576	ACCTTCGAGAGACTAGTCAGT
										6A-1558070-T4-31_64	ACACAGACACAAACATTCAGT
										6A-1558070-T4-31_320	TCCATTGGTCGACGATGGACT
										6A-1558070-T4-31_448	CTATTTCCGAGTTCCGAGTGA
										6A-1558070-T4-31_512	CACGGGACTAGCGGTCTAGC
										6A-1558070-T4-31_768	GGCAGGCGAGGGGGGGAAG
										6A-1558070-T4-31_960	GTGGTGGTGATCGGGTCAAG
										6A-1558070-T4-31_1024	GAGAAAAAGGTTTCCCCTTTG
										6A-1558070-T4-31_1088	ACAAATCTCGTTCGGTGGTCA
										6A-1558070-T4-31_1152	CGGGCGCAAGTGGAAAGAG
										6A-1558070-T4-31_1216	tcttttctttTGGTGCCTTGCTAGG
										6A-1558070-T4-31_1280	ATTTGTTTTCTACTCCTCAGTT
										6A-1558070-T4-31_1344	CGAGGGAAACACTTCCCCGG
										6A-1558070-T4-31_1408	TTATTAGTGCGAGTTTGGGCT
										6A-1558070-T4-31_1472	CGATTTATCCTAGCTGGCGCG
										6B-1643690-T4-31_0	TGGTCGACGACGGATTGATCG
										6B-1643690-T4-31_128	GAACTATTTCCGAGTTCCGAG
										6B-1643690-T4-31_192	ACAGGACTAGCGGTCTAGCA
										6B-1643690-T4-31_576	GGATGTCGGCTGCCTCTGTCT
										6B-1643690-T4-31_640	GAAGTACACCACACAGACATG
										6B-1643690-T4-31_1216	AAAAACGCATTAGGCCCTATAT
										6B-1643690-T4-31_1280	TTATTACTCTAGGTTGATTGCG
										6B-1643690-T4-31_1408	CTAGGAAAACTTTGCTCTGTT
										6B-1643690-T4-31_1536	TTCCTTCCTTTTTGTTGGGAA
										6B-1643690-T4-31_1580	TTTGGCTGGTAATTAATTTTAC
										6D-1735290-T4-31_128	AGTCGTGTTATATAGCCAGCC
										6D-1735290-T4-31_192	AGGAAGGTCGGGTCGCAGG
										6D-1735290-T4-31_576	GAGTGTTTCGTGGTGGTGGT
										6D-1735290-T4-31_640	GGGGAAAGGAAAAATAGGTT
										6D-1735290-T4-31_704	TATTTAAAAAAAAAATCTCATT
										6D-1735290-T4-31_896	ATTTGTTTTCTACTTAGTTGAG
										6D-1735290-T4-31_960	GAAACACTTCCCCGATTCTTC
										6D-1735290-T4-31_1024	TATTAGTGCAAGTTTGGGCTT
										6D-1735290-T4-31_1088	GATTTATCCTACTAGCTGGCGT

6D-1735290-T4-31_1280	GTCGACGTTGGCTTCGCTTCT
6D-1735290-T4-31_1472	GGAGACTTGGTATTGCAAGA
6D-1735290-T4-31_1580	AGGAAGAATGGTTACACGTAG
7A-1779770-T4-32_320	TTTGTGGATGACACTGACAAT
7A-1779770-T4-32_512	CCAACGAAGAAAATTGCATGC
7A-1779770-T4-32_896	CACACCTCCACACACTCATCA
7A-1779770-T4-32_960	CCACACGTACCTGGAGCGATA
7A-1779770-T4-32_1024	TAATAATTACTTTTGCGAGAAA
7A-1779770-T4-32_1408	ACGCACCATTGGGAAGGGGC
7A-1779770-T4-32_1472	ACCTTGGGCCCAGACATGGC
7B-1893610A-T4-32_0	AGACAATTTTATCAGTTGTTTG
7B-2140600B-T4-32_0	GTGTCTTCCTTGAATTCGCAG
7B-1893610A-T4-32_64	TGCGACACATTTATGAAAATA
7B-1893610A-T4-32_128	AAACTCCAAATTTCACATGGA
7B-1893610A-T4-32_192	AACAAATTAGGAACTTGTGTT
7B-1893610A-T4-32_256	CGTATTGCATTCAAAATCAAA
7B-1893610A-T4-32_320	ACTCATCTTAGAACCTTACATG
7B-2140600B-T4-32_320	AAGAAGTAGTACTACTACGAT
7B-1893610A-T4-32_384	TCATCAAGTGGGCGGCACAAT
7B-1893610A-T4-32_448	AAACTAAACCAAAAATTAAAC
7B-1893610A-T4-32_512	ACCAGAGTTCTCCAAAATTCT
7B-2140600B-T4-32_512	GGGATGCTTTCATCCTTACATT
7B-1893610A-T4-32_576	AAACTTGTTCTAATCTCACCCC
7B-1893610A-T4-32_768	GGATGGCGGCATTTCTTCTTT
7B-1893610A-T4-32_832	GGGACAAATTTAACGGAGCT
7B-2140600B-T4-32_896	AGGCGAACTACGACCAGAGA
7B-2140600B-T4-32_960	GCGGGGCTAGGTGGGGAGA
7B-2140600B-T4-32_1024	GCCTCCACGCATGCAGTAGGC
7B-1893610A-T4-32_1152	TTGTACTTCTGTTTGACCTTTA
7B-1893610A-T4-32_1216	TCACGACTCAGATAAATAAAA
7B-1893610A-T4-32_1280	TTAAAGAAAATCAAGGCAGG
7B-2140600B-T4-32_1280	CGCCTCCCGGCTGGACACGC
7B-1893610A-T4-32_1344	AAGCATCCCCACGCGATGGCT
7B-1893610A-T4-32_1408	CTGTCCCACCCCACTCGACTA
7B-1893610A-T4-32_1472	ATGTGATCCACCGATTCCCTCA
7D-2140600-T4-32_0	GGTCCAGGCCCAGTGTATATC
7D-2140600-T4-32_64	GGGAACCGAGCAGAAATAGC
7D-2140600-T4-32_128	AGAAAAAATAGAATCGATAAA
7D-2140600-T4-32_320	AGCGGTGGGGTACCTTCTCCC
7D-2140600-T4-32_512	GCGGATGGATGGGCGACGTC
7D-2140600-T4-32_640	CTTCGCCGTGGTGGTCGTGGT
7D-2140600-T4-32_704	CCGAGTCGGCGATGCCGGAG
7D-2140600-T4-32_1088	CACCGCTGTAATTGAAAATGA
7D-2140600-T4-32_1280	CTGTCCCCACCCCACTCGACT
4A-0968550-T4-33_384	TCTCTGCAAACAAATTCTAGG
4A-0968550-T4-33_448	CAAGTCTTCGGGACTTCACTG
4A-0968550-T4-33_512	AACTTCTAAGGGTCTCACTTT
4A-0968550-T4-33_576	TATTGTCCATATCGCAGTTCCG
4A-0968550-T4-33_640	AATAAACTACGGTGCCATTTC
4A-0968550-T4-33_704	ATGTCGTCGGAGAGGCTCTTT
4A-0968550-T4-33_768	CCTGCCAGTAAAGAAGTTCA
4A-0968550-T4-33_832	CACACATCCAAGCAGAGCTCT
4A-0968550-T4-33_896	CAGCTGAGGGAGTATAGGGA
4A-0968550-T4-33_960	ACGACTTGTCACCCAGCCCCC
4A-0968550-T4-33_1024	CGTTTCGGATACTCCCCTTCTA
4A-0968550-T4-33_1088	GTCAAGGGCTGAATCTACCCG
4A-0968550-T4-33_1152	AAGTTCAAGGACAAGCACCA
4A-0968550-T4-33_1216	TTACCAAGTCCCGTTCTACAG
4A-0968550-T4-33_1280	GATAGTTTGCGGTTCTTTCCT
4A-0968550-T4-33_1344	CCAAAGTACCGTGGAGTCTTG
4A-0968550-T4-33_1408	GACCAGGGGAGTAGGGCAG
4A-0968550-T4-33_1472	ATCTTCCATGCGTGTTCCAAA
4A-0968550-T4-33_1536	ACTGGTCTTTCAAACTCTTCTG
4A-0968550-T4-33_1580	GGAAAGCATGTCTACGTACTC
7D-2025300-T4-33_0	CTCAAGATGGGTGAGTTAAAT
7D-2025300-T4-33_64	TCCTTCTCTTTAGAGTACATAT
7D-2025300-T4-33_128	GACATCATCAGTCAGAAGAGT
7D-2025300-T4-33_192	TGGAAACGCACATTCTGGAA
7D-2025300-T4-33_256	TCACAGTACTCCTGATTCGGA
7D-2025300-T4-33_320	AACCCCACCGCACTTTTTTTA
7D-2025300-T4-33_384	AAAAAGATTGAAGTGCAATTT
7D-2025300-T4-33_576	TCTGTGAGTAGCAACTTCGGT
7D-2025300-T4-33_768	AGACCTCTGCTTCGATGTGTG
7D-2025300-T4-33_832	TTGAACCTGGCAGGCACGGA
7D-2025300-T4-33_896	CTTCGACGACATTGACCACAG
7D-2025300-T4-33_960	TTGTTCGCGCTCTTGCGCGGA
7D-2025300-T4-33_1152	CAACTTAATGAAGTTTTAAAA
7D-2025300-T4-33_1216	TGTAATAGAAACATGAATATGG
4A-0979890-T4-34_576	ACGAAGCAAAGTGTCGCTCTT
4A-0979890-T4-34_640	TCAAATGTTTATGCAGTATTTG
4A-0979890-T4-34_704	TAATTGTCTTAGTGGAATTCAG
4A-0979890-T4-34_768	TTAAAAATTCTTTATGCAGTGT
4A-0979890-T4-34_832	AATAATGTTCACGCGCTAAAA
4A-0979890-T4-34_896	ATTTTTGTGAAATTCAAATAAA
4A-0979890-T4-34_960	AAGAAATATGTTCATAACATTT
4A-0979890-T4-34_1024	ATTTGTTTTATATTATGCTAAAA
4A-0979890-T4-34_1088	TGTTCAAAAAGATTCTGCACC
4A-0979890-T4-34_1152	ATCTCCTATATGTTTCACCGGG
4A-0979890-T4-34_1216	TGTGCGTCAACTTCGTCAATC
4A-0979890-T4-34_1280	TGCGCATGCATTCATAGTAGT
4A-0979890-T4-34_1344	AAAACAAAACAAAAGGCAAG
4A-0979890-T4-34_1408	AGACTAACCCACTTGTGAACA
4A-0979890-T4-34_1472	GCAAGCATGCGTAGCGTAGC
4A-0979890-T4-34_1536	GCTGTGGCCTAACCGCTTGCC
4A-0979890-T4-34_1580	TCGAGAAGCTGCAAAAAGTT
4D-1174080-T4-34_64	GGAGACAAAAAACAACGCTC
4D-1174080-T4-34_256	TCGTCGCACTCGCTCGCAAAA
4D-1174080-T4-34_320	GAAGCGCGCCTATTGGAGATG
4D-1174080-T4-34_384	AAAAAAATACTGAGCCCATTC
4D-1174080-T4-34_448	TAAACCAGCAATCTTCAACTT
4D-1174080-T4-34_512	GATACCGGGCTCGTTAAGGC
4D-1174080-T4-34_576	TTTTTAAAAAATTGCTAAGCT
4D-1174080-T4-34_640	GATAAGAGTGTGGGTGTAATT
4D-1174080-T4-34_704	TGAAGCCTCCTATCGGTTGCG
4D-1174080-T4-34_832	ATGAAACTCGATAGCTCATTC
4D-1174080-T4-34_896	ATGTTCACTAAAAAGCAAGTT
4D-1174080-T4-34_960	CAGCTACAAGCATACCAGGAT
4D-1174080-T4-34_1024	CATTTTAATTAATATCTGAAAT
4D-1174080-T4-34_1088	CTTAGATGGCTAGGTTCCTTAT
4D-1174080-T4-34_1152	CGGTGGTCGCAATTTTCTGAA
4D-1174080-T4-34_1216	TGCCTGTCGACTCTGAAACGT
4D-1174080-T4-34_1280	TGAAAGGTTCACACAGAGGT
4D-1174080-T4-34_1344	AAAGAAAAATCGGCACATCA
4D-1174080-T4-34_1408	ACACACAAAGAATAACCCACT
4D-1174080-T4-34_1536	TGTGGCCTAACCGCTTGCTCG
4D-1174080-T4-34_1580	GAGAAGCTGCAAAAGTTGAA
1A-0031050-T4-35_0	ACACTGTCCTCTCGAAGCCAG
1A-0031050-T4-35_128	TCTTTTATCAATGGAATGACA
1A-0031050-T4-35_192	GCCGGTACTTGTTCAAAAAAG
1A-0031050-T4-35_256	AAGAAAATTTTAATTTTACGA
1A-0031050-T4-35_320	TGCAACTGTCTGTTGCATCCT
1A-0031050-T4-35_448	TAGCCTACTCTCGCAGTATAAG
1A-0031050-T4-35_512	TCATTGCTAGGTAGTGTTGTTT
1A-0031050-T4-35_576	TTCATTGGATGGATCCAACAAT
1A-0031050-T4-35_640	ACACGAGGTGAACTCTTTTTG
1A-0031050-T4-35_704	TAACACACGTTTCCTGCATTTA
1A-0031050-T4-35_768	AAGATCAATGTGAGAGTGGA
1A-0031050-T4-35_960	CACCCTTGGCGATGTAACTGT
1A-0031050-T4-35_1088	TTGCTACAAGTCGTCCATCCC
1A-0031050-T4-35_1152	TCCCTGCAAATTAAGAAGAAG
1D-0187950-T4-35_384	GCAGAGGCGGAACTCGGAAG
1D-0187950-T4-35_448	ATCGAGCCGGCACGTACGGC
1D-0187950-T4-35_512	TGCGATATTCTCGGGTTCCGAT
1D-0187950-T4-35_640	GATTTTTCAGCGGCGAATGTT
1D-0187950-T4-35_832	TCGAGAGAATCTACAAGACTG
1D-0187950-T4-35_896	TTGCCCGCGGTCTTCGACGTG
1D-0187950-T4-35_960	TGTTGGTGGCAAGATTATGAA
1D-0187950-T4-35_1024	ACAAATTAAATTTATGCAAATG
1D-0187950-T4-35_1088	TTCTAGCCAGACTACCCACAA
1D-0187950-T4-35_1152	CAATGAAGATCAAAACTGAGG
1D-0187950-T4-35_1216	GTAAATTGGCACAAAAAACGT
1D-0187950-T4-35_1408	GACACGGCAAACTTTACCCTA
1D-0187950-T4-35_1580	TTTGAGCCCGAGATAAGCCCG
5A-1186440-T4-36_0	TATTTCTTTAAAAAATTCATGT
5A-1186440-T4-36_124	TGGGCCCCATCGCTAAGGGTG
5A-1186440-T4-36_186	TCTCTTCTTTATTTTTTAATTTT
5A-1186440-T4-36_248	ATTGTTTAAGTCCATGAGCTTT
5A-1186440-T4-36_310	ATTGAAATCCATGAATATTCAT
5A-1186440-T4-36_372	TTGGTTATGAACATTTTTAAGC
5A-1186440-T4-36_434	ATAAACATTTTCATAAAAAACA
5A-1186440-T4-36_496	ATGGCTAAGCAAGCGATTGAA
5A-1186440-T4-36_558	CCCATGTGAATGTTATAGAAA
5A-1186440-T4-36_620	ACTAGCTGACACTAAGGCCGC
5A-1186440-T4-36_682	ATTCTCTTTTTTTTGCGGTTAA
5A-1186440-T4-36_744	GCACCCAACACGATGTGCAAA
5A-1186440-T4-36_806	acgaagaaaaaatcaaaacaaaata
5A-1186440-T4-36_868	TCTCATGAGTAAACGACACGA
5A-1186440-T4-36_930	tagatgactcaacttgtactaGTAGG
5A-1186440-T4-36_992	AACTGACCAACGAAGCATCGC
5A-1186440-T4-36_1054	CGCAAATTGACGGCATATGGC
5A-1186440-T4-36_1178	TACAACGAAACGAAAGAAGC
5A-1186440-T4-36_1240	CTAGAGAGCTTTGATTTGGCA
5A-1186440-T4-36_1476	CTATCCTCTCGCTCCAAGCGG
5B-1307890-T4-36_0	CGAACGATTCCCCCGCCGGCG

											5B-1307890-T4-36_256	GAACTGCGTTTCATCGGTGGG
											5B-1307890-T4-36_448	AGGTTCAGGTTCAGGTGTTTT
											5B-1307890-T4-36_512	AATATATGCCATATGCCGTCAA
											5B-1307890-T4-36_704	CATTTACAGGAGACGATGCAA
											5B-1307890-T4-36_1088	AGACGCATCGCTTCATCATGT
											5B-1307890-T4-36_1152	CTCATGAGATTAAGTCTTTTTT
											5B-1307890-T4-36_1216	ttctttgtttgttaggttttaattatgttgt
											5B-1307890-T4-36_1280	GGTGCCGCTAGAGACGTTAAT
											5B-1307890-T4-36_1344	TCATACACATTGCGGCCTTAGT
											5B-1307890-T4-36_1472	TCCCTGCTTCACTCGGTTGCTT
											5B-1307890-T4-36_1536	TGGCTATTTTTTATACGAAAAT
											5B-1307890-T4-36_1580	CATGATTTTTTTTAAATCTTG
											7A-1837360-T4-37_0	tgctgctgctgctgctggtgTGGTGT
											7A-1837360-T4-37_64	TAGATGGCTTGTGTTTTGGTT
											7A-1837360-T4-37_192	GTAATCTAAACACTCTTATATTA
											7A-1837360-T4-37_320	ACGCTCTTATATTATGGGACGG
											7A-1837360-T4-37_384	AGCGCACAGATGTCCATCGCA
											7A-1837360-T4-37_448	ACAGATCTTGGCCTCTACTATG
											7A-1837360-T4-37_576	GACGCTGACGTTACCGACCAG
											7A-1837360-T4-37_640	ACAGCAACATAGATGAGGCTT
											7A-1837360-T4-37_704	AATTAAATAAAGAAAAAAAGA
											7A-1837360-T4-37_768	GAAGTTCAATTTTGGGTTAGT
											7A-1837360-T4-37_832	TTCTTTTTCTGGGCTGCTGGT
											7A-1837360-T4-37_1024	ACTTCCATTTTTCTACCGACCA
											7A-1837360-T4-37_1088	CCGACACAACCttcttctttcttttt
											7A-1837360-T4-37_1152	ttctttattacttgctttTTATTATCTC
											7A-1837360-T4-37_1472	TGCACAAAATGTGTGGCATAA
											7A-1837360-T4-37_1536	TGTCAATCGTCCGGTGCTACC
											7A-1837360-T4-37_1580	CTAACTTTCAGCCCATTTACAC
											7D-2064320-T4-37_0	AGCAGAGACTCTTCCGTTAGA
											7D-2064320-T4-37_64	TCTACATTTGAACTGTCTACTT
											7D-2064320-T4-37_128	GCACTAGCTATATTTAACATTC
											7D-2064320-T4-37_192	GATTCAAAGCTAGCCACGCCA
											7D-2064320-T4-37_320	GTTACCGACCAGGATCTAATG
											7D-2064320-T4-37_384	CGAGGAGAGAGATCCCCTGT
											7D-2064320-T4-37_448	TATGGCAAACATTTTTCTCCAA
											7D-2064320-T4-37_960	CTATGTTTGAGATGCTTTGTAC
											7D-2064320-T4-37_1024	AATGAGGAGCATTTGTGCCCG
											7D-2064320-T4-37_1088	TCTGAATGGGGGTCTAGCAAA
											7D-2064320-T4-37_1152	TTACTAAAAATGTGTAGACGT
											7D-2064320-T4-37_1216	GGGCCCTATACGCTTGCGGG
											7D-2064320-T4-37_1280	TCTCCCTGTCCACAACCGCAA
											7D-2064320-T4-37_1344	CGTAGATCAACATGGTAACGT
											7D-2064320-T4-37_1408	TAGTTCGTTGTCAGAGATAGT
											7D-2064320-T4-37_1472	AAATATATTAGCTAGACAGTTT
											3A-0681650-T4-38_0	GGCTGATCGGCCGTGGCAGC
											3A-0681650-T4-38_192	TGATGCATGCTCTCACGTCAG
											3A-0681650-T4-38_256	CTGAACCATCAGAAAGGGGG
											3A-0681650-T4-38_320	CCGCTACTGCCGAAAGAGGA
											3A-0681650-T4-38_384	CTGCCCGGATGGTCTTCAGGT
											3A-0681650-T4-38_448	AAAGCTGTGGAAGGTAGAGC
											3A-0681650-T4-38_512	GTCACCGTCAAAATTATTTTCT
											3A-0681650-T4-38_576	CTTGATCATATTACCTAGTCAA
											3A-0681650-T4-38_640	TTTGTAAGTGAGAAGCTTCTG
											3A-0681650-T4-38_704	TTTTGAGTGACGCCAATGGAG
											3A-0681650-T4-38_768	AGAGGTATTCCTTTTCCGTTTT
											3A-0681650-T4-38_832	ACAAAACCAAATAAACACACT
											3A-0681650-T4-38_896	CCTGTGGCGATTAGCAATGAA
											3A-0681650-T4-38_960	CTCTGGTGGAGCACGACCCG
											3A-0681650-T4-38_1024	TGAAGAAGAAAAATCAAAATA
											3A-0681650-T4-38_1344	ATTTAGGAACGGAGGGAGTA
											3A-0681650-T4-38_1408	ATACATCAAAGAAAAAGCACA
											3B-0752330-T4-38_0	GTGATGGGGGTTACTTTGGGA
											3B-0752330-T4-38_64	GAGACGCTCCACGGTCATAGG
											3B-0752330-T4-38_128	CCCAATTTTACTCACGTTTTTG
											3B-0752330-T4-38_192	AAGGTCAAGATGCAAATTGTG
											3B-0752330-T4-38_256	TTGTATCGAGGAGATCTTTTT
											3B-0752330-T4-38_320	ACTCCTATCGATTTGTTAAACA
											3B-0752330-T4-38_448	GAACGAGTCATGAAGTTTACT
											3B-0752330-T4-38_512	TAAAATACTACATGAAAGCATA
											3B-0752330-T4-38_704	CCATGCTCCACCAGACCAGAG
											3B-0752330-T4-38_768	ACAATAACAAACCTTGTTTCAT
											3B-0752330-T4-38_960	TCGTACCTCCTTGCGGCAGTA
											3B-0752330-T4-38_1024	TCGCGCCGAAAATTGACTTGT
											3B-0752330-T4-38_1088	ACTTCCAAAGGCCTGAGAGG
											3B-0752330-T4-38_1344	TTCAGTGCATCGAACGGCGTC
											3D-0915560-T4-38_256	GCCATGTGGATGAATCTTCTTA
											3D-0915560-T4-38_320	AGGGCTCAGATTAACCACCGC
											3D-0915560-T4-38_448	GGAAGGCCGAGCCAGCCGAT
											3D-0915560-T4-38_512	CAGTGTACTGTGACAGTGGGT
											3D-0915560-T4-38_704	TTCATGCACACAGACCTAATA
											3D-0915560-T4-38_960	AACCATCTAGAATAGCAGACT
											3D-0915560-T4-38_1024	GAGAGGATAATTGATATTCCC
											3D-0915560-T4-38_1088	CTGGTACAAAATTCTATATGAT
											3D-0915560-T4-38_1152	AAAAGCGAGCTTAGGTAATCT
											2A-0284830-T4-39-2_64	AATAACATGACGCTTAATTAGA
											2A-0284830-T4-39-2_128	GTCTGCATTGCTTTCAGTAGG
											2A-0284830-T4-39-1_192	TGCAAGCGGGTCAACGGCTG
											2A-0284830-T4-39-2_256	AAAATTCCAACTTGCGTATCTC
											2A-0284830-T4-39-1_320	GTCAAGTTGGTTGCTAGGTTT
											2A-0284830-T4-39-2_320	TGTGACAAATCGTACTAGAAG
											2A-0284830-T4-39-1_384	TCAATCACATGCATACATCATG
											2A-0284830-T4-39-2_384	TGCTCTTTTTTCGTACTGTACA
											2A-0284830-T4-39-1_448	TGCGCGAGTTGTGTTGATCGA
											2A-0284830-T4-39-2_512	TGCACGTGAATCCTCGGATGA
											2A-0284830-T4-39-2_576	GCACCAGCTGCTAAAATGAAT
											2A-0284830-T4-39-1_640	CTGTTTTAACCAGCGAGAAGA
											2A-0284830-T4-39-2_640	TCAATGGAAGATTGAATATCTA
											2A-0284830-T4-39-1_704	CAGCTTTATTTTCGTGGGAGA
											2A-0284830-T4-39-2_704	ATACATTTTTTTTAGGATAGTA
											2A-0284830-T4-39-1_896	ATCGATCGATCATGCTGTTTCT
											2A-0284830-T4-39-2_896	TAAGGGTGAGATACTAATTAAT
											2A-0284830-T4-39-1_960	GAAGCTTCCTTGTTTCGGATC
											2A-0284830-T4-39-2_1088	TTGTAACAGAAGAATCAGCAT
											2A-0284830-T4-39-1_1152	GCGCACATGCCTGACAACCAG
											2A-0284830-T4-39-2_1152	GAGGATGACATGTACTTGTAG
											2A-0284830-T4-39-1_1216	TGATTAACTGCACGAATCACG
											2A-0284830-T4-39-2_1216	CACTGTCTACTAAATTCGTGTA
											2A-0284830-T4-39-1_1280	AAGAGAAGACTCTCCGGCCG
											2A-0284830-T4-39-2_1408	GCCTTGACGTTAATTACAACG
											2A-0284830-T4-39-1_0	GGTTGGTCGGTCGGCAAGAA
											2A-0284830-T4-39-2_0	TCCAATGTTAGACCAAACACA
											2B-0425860-T4-39-2_64	TCCTTAGCTCCTTCATTTGTAC
											2B-0425860-T4-39-2_128	CTAACGTCAAGGCAGCCCATG
											2B-0425860-T4-39-2_192	TCAATCGTCGGTACATAACCTA
											2B-0425860-T4-39-2_256	GTTGATAAACTACTCCACAAA
											2B-0425860-T4-39-2_320	GTGGCAAGATGAAATGAATTT
											2B-0425860-T4-39-2_384	CCATGTCGAGCCATCTAATGA
											2B-0425860-T4-39-2_512	GCAAAGTCCAAGCCAGTAGG
											2B-0425860-T4-39-2_704	TATGTACCAGCTGCTAATTTTG
											2B-0425860-T4-39-2_768	TAAAACTAGCATTTCTACTACT
											2B-0425860-T4-39-2_960	AGTAGTGCATAGTATTCATTTT
											2B-0425860-T4-39-2_1088	GTAAACACCTGTTGATGCATG
											2B-0425860-T4-39-2_1152	GAAATATGCACAGTACGAAAA
											2B-0425860-T4-39-2_1344	GACTTTGACTCCGTTAGATCA
											2B-0425860-T4-39-2_1408	GGAACAAAGACGTACTGAAA
											2B-0425860-T4-39-2_1472	ACTGTAGCAATTTAATTTATTC
											2B-0425860-T4-39-2_1580	ATTTTAGTTCTTTCATTTTATAA
											2B-0425860-T4-39_1512	CGCGCGGCGCTTTAGCGCGG
											2B-0425860-T4-39_1575	AACAAAGTTCTATATAAGGGG
											2B-0425860-T4-39_1815	ATGACATCGGTATCTCAGGTCT
											2D-0563460-T4-39_64	ATGAAGGCCAGCAGTCTGCG
											2D-0563460-T4-39_128	ACGAAGCTAAGCTAGCTCGGT
											2D-0563460-T4-39_192	TAATCATCTATCTTTCTTTCTGT
											2D-0563460-T4-39_256	CGAGCAGCTTGGTTATTCTTC
											2D-0563460-T4-39_448	GTCTTCTCTTCCATCTCCATCA
											2D-0563460-T4-39_640	GAAGTATCTTTGAACTGAACA
											2D-0563460-T4-39_704	GCGGCCGACTTGCATGTCAAC
											2D-0563460-T4-39_768	ATTATATGCACCTCTGAAGAAA
											2D-0563460-T4-39_960	CCATCACGAGGGATCCGCCAA
											2D-0563460-T4-39_1152	ATCAATATGAATATGCATGATT
											2D-0563460-T4-39_1280	AGACGAACGAGTGTGTGTGT
											2D-0563460-T4-39_1472	ATCTAGTCCCAATTTTGACCAC
											1A-0049460-T4-40_128	GCGCAGCAGAGATTCTTCCGG
											1A-0049460-T4-40_192	AGTCTAGTTTTCCGGCTTACTT
											1A-0049460-T4-40_448	ACCAGACTAAGAGGATCTTGC
											1B-0122280-T4-40_0	TGCTGGTGTCTTGATCCGTTCT
											1B-0122280-T4-40_59	GAGATGCAGGGGAGGAGCG
											1B-0122280-T4-40_118	TTTTGTTGTCTGCGAGACTGT
											1B-0122280-T4-40_236	GAAGACTCGGACAATACGAG
											1D-0191530-T4-40_0	AATAAATGTGTCTTAACTCAAT
											1D-0191530-T4-40_64	ATGCATGTCACTCATCTGATCA
											1D-0191530-T4-40_192	CAAATGTTTCAAGAGCAAAAG
											1D-0191530-T4-40_256	GCTCCAAACGCTGTTCTACTTA
											1D-0191530-T4-40_320	AAGTTGATCATTTTTTCCTTTT
											1D-0191530-T4-40_384	AGGGATAACTCTACTTATCGG
											1D-0191530-T4-40_448	CAACAGGTTGTATTCATTAGA
											1D-0191530-T4-40_512	AACACATGAATCGAAGAGAGT
											1D-0191530-T4-40_1408	tgtgcaaacgcccacacgtgtgggcgt
											1D-0191530-T4-40_1472	ATATTCTCATATTGTCCGAGTCT

1D-0191530-T4-40_1580	AGACAACAAAACAAGTCGTG
5A-1203500-T4-41_128	CCTACCGACGCGGACCACCAT
5A-1203500-T4-41_192	AGGACAGGCTCGGAGAGGA
5A-1203500-T4-41_384	TCGACATCAATTCCGGTTGTT
5A-1203500-T4-41_576	AAAAGTACGTTTGGACCAGG
5A-1203500-T4-41_640	GTATGCGGATGTTTTGAGGGT
5A-1203500-T4-41_704	TTATATTTCTTTACTAACGAAG
5A-1203500-T4-41_768	TATTCTACATAAAATACGTCTG
5A-1203500-T4-41_832	AAAGAAGCTAAGAGCTTCTC
5A-1203500-T4-41_1216	AAATTTTAGCGTGGAGCAGTT
5A-1203500-T4-41_1344	GACACGCGGCCTTGACGCGC
5A-1203500-T4-41_1408	GCGGTCCGCCATCCATCCGTC
5A-1203500-T4-41_1472	TGGCTGTGGGTGACGACGCG
4B-1038150-T4-41_0	GAGATCTGCTGGAGATGCTCT
4B-1038150-T4-41_128	GCCGGTCGCGTCCTAGCCGTT
4B-1038150-T4-41_384	AAACAGCGGTGGTAGCAAAA
4B-1038150-T4-41_704	CAAAAACATATTACCGTGGTA
4B-1038150-T4-41_1216	CCGGCCGAGCGTTCGGTCGG
4B-1038150-T4-41_1280	ATAAGCATCGGTGCTTGAAAT
4B-1038150-T4-41_1472	AGCCGTCGCCAGGCCGATGA
4A-1011820-T4-42_0	AAAAATCAAGCTTTGAATTTG
4A-1011820-T4-42_64	TGATGCAGTTTTCGGTATGTG
4A-1011820-T4-42_128	TCGACTGTGGTTTAATGTAATT
4A-1011820-T4-42_192	GTGTAATTTATATTCTCGTTCA
4A-1011820-T4-42_256	TAGTGTCAGTGTTAGCTCATA
4A-1011820-T4-42_320	TTCTGGTTCAGTGTAATTTTGT
4A-1011820-T4-42_384	AATTTCTATTCCTGTTCACTGT
4A-1011820-T4-42_448	AATAGAACTTGCAGTGTAATT
4A-1011820-T4-42_640	TTGCAGGCTTTTTTTTGGCC
4A-1011820-T4-42_768	TTGGCCACCCGGTAAACGTGT
4A-1011820-T4-42_832	AAGACATACAACGCGGCAGG
4A-1011820-T4-42_896	GTCGGATGTTAAATAGACAGT
4A-1011820-T4-42_960	CAGCTTTTACCGCAAGAAAAT
4A-1011820-T4-42_1216	ACAATCGTCACAACCCACGGC
4A-1011820-T4-42_1280	CCCACCACTCCGTCCCAGCGC
4A-1011820-T4-42_1344	TGACGAGTTATCACGGTGAGC
4A-1011820-T4-42_1408	agaggaaaaaggaagaaggatagag
4B-1070420-T4-42_0	GGAGGAGATAGCGCTTCCTTC
4B-1070420-T4-42_192	CCGGGCCAGGCAGCGGAGGA
4B-1070420-T4-42_256	CGCTCCCCTGTCCGCCACTCG
4B-1070420-T4-42_320	ATTTCGTCTCTGACTTGGCTCC
4B-1070420-T4-42_512	TGCGTCGCCATCGCGCGCGCC
4B-1070420-T4-42_576	GCGGTAGAGGCAAAATCGAA
4B-1070420-T4-42_768	CGACGGCGTGGTCCGAGTTCT
4B-1070420-T4-42_960	AGAGCCCCGCCATGGCCTCCA
4B-1070420-T4-42_1024	TTTCTCCCACACACAAAAAGA
4B-1070420-T4-42_1088	CTACACACCTTGCGGTTCATGT
4B-1070420-T4-42_1280	TGAGCACCCCAACAACATCGG
4B-1070420-T4-42_1344	GTCGCGAGCTCCAACAACGC
4D-1152920-T4-42_256	CAGCTCCCTCTGTCCGCCACT
4D-1152920-T4-42_320	GATTTCGTCTCTGACTTGGCT
4D-1152920-T4-42_384	CCGAGATGATCCTAAAAAAAA
4D-1152920-T4-42_576	GTGCGCCCGAGCACGCGGGG
4D-1152920-T4-42_704	CATCTTCGAAAGACTGAGCCA
4D-1152920-T4-42_768	TTAAAATAAAGGTGTAGGGCA
4D-1152920-T4-42_960	TTGACAACCGCGACCATGTCT
4D-1152920-T4-42_1152	CTTCTTAAGAGCCCCGCCATG
4D-1152920-T4-42_1280	AACGGTCGCCACACACCTCGC
4D-1152920-T4-42_1344	GCGTGATTAATTGGGCGAAAA
4D-1152920-T4-42_1408	CCGTCCGTTGTCCGGCCAAAT
4D-1152920-T4-42_1580	GGAGAGGGAAAACGAGAGA
3A-0651840-T4-43_0	GTTGTGATAGGCTTGCTCCAA
3A-0651840-T4-43_640	AGCTAGAAGACTCTAAAAAAC
3A-0651840-T4-43_704	GAAGTATCTAGAGTAGCAAAC
3A-0651840-T4-43_896	AGAGGCGACGATGCACGGTG
3A-0651840-T4-43_1088	GTGCAAGATCGATAATGCAGG
3A-0651840-T4-43_1152	TTTGCTCCTTACGTGAGACAA
3A-0651840-T4-43_1216	GTTGCAGGGCAGCGCTAACA
3A-0651840-T4-43_1280	TCAATGGCTCACACATAATGC
3A-0651840-T4-43_1472	GGAAAAGGAAAGAAATGAAA
3B-0722470-T4-43_1472	CATACGGAAAAGGAAAGAAA
3D-2135880-T4-43_192	TAAGATCACCTACCCTTCTTCC
3D-2135880-T4-43_896	TTAAGAAAAAAACACTATATT
3D-2135880-T4-43_960	CTCATCTATTGTTCCTCTCAGG
3D-2135880-T4-43_1152	CATGTCGTGTTGTGATAGCGG
5A-1198810-T4-44_0	AATCATCGACCGTTCAAATGTT
5A-1198810-T4-44_64	TTTTCTTTTTCTGCAGCGAAA
5A-1198810-T4-44_128	TATTCAGCATGGACTGGAAAA
5A-1198810-T4-44_192	CTATCCAAACTTCGCATATTTG
5A-1198810-T4-44_256	GATAGTGTCTTAGACTAGACA
5A-1198810-T4-44_448	CTCGGGTAGAGTGCTTACATG
5A-1198810-T4-44_512	TTTGTACTATGTTATTAAAGAA
5A-1198810-T4-44_576	ACCGGCGGTCCGGTCGGTGA
5A-1198810-T4-44_640	GCATTCAGTTTCGTGCACTAAT
5A-1198810-T4-44_704	CAACGGTATGCTTGTGTTCCC
5A-1198810-T4-44_768	CACTTCATGACATGAGTGCGC
5A-1198810-T4-44_960	CCAAAGCACTAGGATTTGAAA
5A-1198810-T4-44_1088	TTCCGTGCTCCCGGTTGGACT
5A-1198810-T4-44_1152	ACTTTAAAATTTCAAAAAAAA
5A-1198810-T4-44_1216	TTAGTTCATGTTCTTCAACAAG
5A-1198810-T4-44_1280	GAATTTGTTATTTTTTCACGAG
5A-1198810-T4-44_1344	TGCAAAACTTTGTGTATTTTTT
5A-1198810-T4-44_1472	CGTCTCGGTGCGAAATGAAG
5A-1198810-T4-44_1536	TGTTCCCTCCCTTCATCCCAAC
5B-1293440-T4-44_618	GGGGCGACACAATGTGCAGC
5B-1293440-T4-44_748	TGGTGAGCATTGCATGACGAC
5B-1293440-T4-44_878	CTCACCCTAGCTCGCACGTCA
5B-1293440-T4-44_943	AGTCGCCAAAGCACTAGGATT
5B-1293440-T4-44_1138	TGTATTTTAAAATTTCAAAAAT
5B-1293440-T4-44_1203	TACGATGCACATACAGATTTG
5B-1293440-T4-44_1268	ATGGTGCCTGCTAGAAAAGCA
5B-1293440-T4-44_1333	TCATGTAATTGCACCAAACATA
5B-1293440-T4-44_1398	AAAGAAAAAAGCAAAAGCTG
5B-1293440-T4-44_1580	CATCCCAACTCTTCTCCGGGTT
5D-1450500-T4-44_384	ACGAGTACTCCTTAATTAGTGA
5D-1450500-T4-44_448	TAAAAAACCATCCCAATTTCCT
5D-1450500-T4-44_512	TACGTCTCATTTATGGTCTAAA
5D-1450500-T4-44_576	AAACCTGAACCGAGGACCTTA
5D-1450500-T4-44_640	ACTAATCAGTAGAACCAAAAA
5D-1450500-T4-44_704	TATATGCTTGTGTTCCCGCAAA
5D-1450500-T4-44_768	TTGCATGACATGTGTGCGCGT
5D-1450500-T4-44_960	AAAGCACTAGGATTTGAAAG
5D-1450500-T4-44_1088	CCGTGCTCCCGGTTGGACTTT
5D-1450500-T4-44_1152	ATTTCAAAAAAAAATCTTGAAA
5D-1450500-T4-44_1216	CAAATTTGCGTGTTAAAATGT
5D-1450500-T4-44_1280	TGCTAGAAAAGCAGTTTTTCT
5D-1450500-T4-44_1344	GATTGCACCAAACGTAAATCT
5D-1450500-T4-44_1408	AAAAAAAATGGGAATTTTCTT
5D-1450500-T4-44_1580	CATCCCAACTCTTCTCCGGGTT
7A-0615060-T4-45_0	CCGCACGTTCGCTTCGGCCGG
7A-0615060-T4-45_64	GTCCTGCGAGTGTAAACAGTG
7A-0615060-T4-45_192	ATATTATGAGAAAGAGGGAGT
7A-0615060-T4-45_256	TTTTCAGGGATCAAATCATGAT
7A-0615060-T4-45_320	AATGATGCTTTGGAAACTGAG
7A-0615060-T4-45_384	CTGTCTAATAAACCTCTTGATA
7A-0615060-T4-45_448	TTATGGTCACTCGATGGCTGA
7A-0615060-T4-45_512	TCAATTGGTATTCTATAGCCGG
7A-0615060-T4-45_576	CATGCATCGTTGTTTTCGTGCC
7A-0615060-T4-45_640	CCCCTAGAAAAGAAAACGCA
7A-0615060-T4-45_704	TGCTAATATGCACTACATTATGT
7A-0615060-T4-45_768	AGACTAGAGGGGGCTTCTTTT
7A-0615060-T4-45_896	CGATCAAGGAGCCATTCAACA
7A-0615060-T4-45_960	ACTGCACTATTTTGTTGGCTTG
7A-0615060-T4-45_1024	GTCTGTCTAGATTCGATCAAAT
7A-0615060-T4-45_1088	TCCTCTGTCCGTGCAACCTGTT
7A-0615060-T4-45_1152	ATCCTCTCCCATGGCTAGATTC
7A-0615060-T4-45_1216	CTGCGTTCCACGACGAGCTCA
7A-0615060-T4-45_1280	CTGCTACGTTTTTCTTTAAATT
7A-0615060-T4-45_1344	CAACCACGTACAGCTCTGAAC
7A-0615060-T4-45_1408	CCAAGCCACAAAGGAACAAA
7B-1870160-T4-45_128	TCCTCAAGTGTTCGACAGTGG
7B-1870160-T4-45_192	GGCCCACATCACCGCGTGTTG
7B-1870160-T4-45_256	TCCTTCCTGGTGCGCTGCTAG
7B-1870160-T4-45_512	TACTATGTAGTGGGCAGAGCG
7B-1870160-T4-45_576	TAGTGTACGAATGTCAACAGG
7B-1870160-T4-45_640	AAAATCATTACAATTGAAGAG
7B-1870160-T4-45_704	CCTGAAAATCATTCCAGCTGT
7B-1870160-T4-45_768	GGCCAGGGTACATGACTGGT
7B-1870160-T4-45_832	CGCAGCTTTGAGGCATTGGC
7D-2008440-T4-45_192	ACCGACCGACGTGGCCGAGT
7D-2008440-T4-45_256	ACTTGACTTTTGTGCCTTTGT
7D-2008440-T4-45_320	TCCATCCATTGTTGAACGCAAT
7D-2008440-T4-45_384	CATAAGAGGCAATGTAAATTT
7D-2008440-T4-45_448	TGTAATGGGCAGAGTGAAATC
7D-2008440-T4-45_576	TGATTTGATCGAATCTAGACTA
7D-2008440-T4-45_640	GTAGCAATCCAACAAAAATAG
7D-2008440-T4-45_704	CATACAGTATACAGCTGCTTGT
7D-2008440-T4-45_768	GCAGGATAATGGACATTCTCA
7D-2008440-T4-45_832	TAGTATGATCTCTGAAAAGATG
7D-2008440-T4-45_896	GTACTCTCTCCGTTGGATAATG

7D-2008440-T4-45_960	GGTAATAGTTGGGTTTTTTTC
7D-2008440-T4-45_1024	TCAAGAGGCACAAATCAACGA
7D-2008440-T4-45_1088	CCGGTTATAAAATGCCAATTGA
7D-2008440-T4-45_1152	AACTCCGTCTCTTCCTGGAGA
7D-2008440-T4-45_1216	GGTTGTTATTTGAGATGATAG
7D-2008440-T4-45_1280	AACTCTAAGTGTTGGAGAGCC
7D-2008440-T4-45_1344	CATCATTACAATTGAAGAGAA
7D-2008440-T4-45_1408	TATTATTCCAGCCGCTGCAGG
7D-2008440-T4-45_1536	CATGGTAAATCCCACAGGACC
5A-1215280-T4-46_128	GTTATTGCCTTGCTGGTGTTTT
5A-1215280-T4-46_192	TCTTGCAAATACTCTAGAATAC
5A-1215280-T4-46_256	TAGCAGTAGTATACTGATATTA
5A-1215280-T4-46_320	GTCCCTTTCTTTTTACTAATATT
5A-1215280-T4-46_384	GTGTTGAAGTCATAAGAAAAC
5A-1215280-T4-46_576	CGATCGTGTGGTACAAGATAG
5A-1215280-T4-46_640	GATAGGATAGTGGTAGCACCC
5A-1215280-T4-46_768	TGTGGAGTGTATCATGCATGA
5A-1215280-T4-46_960	AGGAGCCGCCGAGCTGGTGT
5A-1215280-T4-46_1024	ATAGTCTTGAAAACACTGTAG
5A-1215280-T4-46_1088	AACTTCATGTAGCAGTATTATA
5A-1215280-T4-46_1152	TACATTTTTCCTTGTCAATTGT
5A-1215280-T4-46_1216	CAGGCTTGAAGATTACTAAAT
5A-1215280-T4-46_1280	AATCAACTGGAATTGGGCCAG
5A-1215280-T4-46_1408	CAGCTCAGCCTGGCCAGTGG
5A-1215280-T4-46_1536	GAGTATGATACGATTGCGACG
5A-1215280-T4-46_1580	GCGTCATGCATGATGTCGCCC
5B-1286670-T4-46_189	GCTATCAGAAAAGGAAGATG
5B-1286670-T4-46_315	AGTAGAGGATTGCCTTGAGA
5B-1286670-T4-46_441	TACATCGAAGAGTGATCGACC
5B-1286670-T4-46_567	CGGGAGGACTTTGTCGACGT
5B-1286670-T4-46_630	ATGTCAAAGTCTAGCAACTGC
5B-1286670-T4-46_693	GATTATGGATAACTAGTGTAGT
5B-1286670-T4-46_756	CACTTTTTACTTCAGTGTCTAT
5B-1286670-T4-46_819	CTCGAGTCCAACAACTGAACA
5B-1286670-T4-46_882	ATTATCTTTGTACTCTTCAATCT
5B-1286670-T4-46_945	TTTTTGCAGCATTACTTTTTTC
5B-1286670-T4-46_1008	GTCGCTCCCTTTAGCTTGAAG
5B-1286670-T4-46_1071	TTAACAGAAATCAACTTGAAT
5B-1286670-T4-46_1134	TGACTTATCAGTCACTTTGAAT
5B-1286670-T4-46_1309	TGGCTATTAGCTCAGTCGCAA
5D-1403090-T4-46_64	TGGAGGTAGGCACCTACACAT
5D-1403090-T4-46_128	TCAGTGTTATATCCTATTTCCTG
5D-1403090-T4-46_384	TCTTTGAACATTTCCGTCAAG
5D-1403090-T4-46_448	TAGTTTATGGAGTTATAGCTGT
5D-1403090-T4-46_512	GAGCACCAGCGCCATGCCTCT
5D-1403090-T4-46_576	TTAAACTTTTACTCCCTCCGTT
5D-1403090-T4-46_640	GCTTGGCAAGATATGCAAGAT
5D-1403090-T4-46_704	ATCTTATACTAAGTATTATGCA
5D-1403090-T4-46_768	CTCTCTATTAATGTCATTGCCT
5D-1403090-T4-46_960	AGATAAATCTAAGACAAGAAT
5D-1403090-T4-46_1024	ATCTTCATGTAGCAGCATTATA
5D-1403090-T4-46_1088	TACGTTTTTCCTTATCAATTGC
5D-1403090-T4-46_1152	GGCTTGAAGATTAAATGATTAT
5D-1403090-T4-46_1344	AGCCTGGCCGGTGGCCACTG
5D-1403090-T4-46_1408	GTACTCACAGTCTGACTGGAG
5D-1403090-T4-46_1580	GAGGATTAAAACAAATTTTGA
3A-0689800-T4-47_64	GGCGGCGACACGTGCGCGGG
3A-0689800-T4-47_128	gcggGTCCTTGGGGTCCAAGA
3A-0689800-T4-47_384	GGCCCGGCACGGCCGGGGTT
3A-0689800-T4-47_448	GTATCTTCCACCGCACGGTTTA
3A-0689800-T4-47_512	GGTGGCTCTGTTGGGTTTTTT
3A-0689800-T4-47_1536	ACCAAGGGTGCAACATCAGC
3A-0689800-T4-47_1580	GTCCTTCCAAAATAATGTTGG
3B-0762490-T4-47_1364	CGACACGTGCGGGGAGAAGA
3B-0762490-T4-47_1488	CCTACCCACCCACGTCCCGCC
3B-0762490-T4-47_1674	CAGAATCCCGGCCCGGCCGG
3B-0762490-T4-47_1736	ATTAGTTAAGAGTATCTTCTAC
3B-0762490-T4-47_1798	GATAATAAAAGGTGGTGGCTC
3B-0762490-T4-47_1860	TGTTATTACCGTAGCCCAGATA
3B-0762490-T4-47_1922	TGGACTGTGCGTAATTTTTCCT
3B-0762490-T4-47_1984	TCAAGCCAGAGATAACAAGTC
3B-0762490-T4-47_2046	GCAACATCTTTGTCTCCACCTT
3B-0762490-T4-47_2170	CCCGATAATATCGCTGCCCAG
3B-0762490-T4-47_2232	ACAACAGGTTTTCGCTTGTTT
3B-0762490-T4-47_2294	GCTTTTTTTCTTTTCTTTTTTTG
3B-0762490-T4-47_2356	GCATGGGTGGCCTGGCCCGA
3B-0762490-T4-47_2418	GACGTTCAGGCCGGGCTCAG
3B-0762490-T4-47_2480	CGTGTTTTTTGATGCTTTTTCT
3B-0762490-T4-47_2604	TGAAGTCGGGCTTTTTGAAAC
3B-0762490-T4-47_2666	GGCTACAATCAGCTACTCCTA
3B-0762490-T4-47_2728	CCGGTGCATACCCCAACTCTG
3B-0762490-T4-47_2790	ACTAATTTTACGAGGGATAAA
3B-0762490-T4-47_2852	TGGCTATTCCTAGACCGAACA
3B-0762490-T4-47_2875	GAAATGCTAGTGAGAGCTTG
3D-0930440-T4-47_64	ggcggggggcggctgcggcgcggctc
3D-0930440-T4-47_128	TACCTACCCACCCGCGTCTCG
3D-0930440-T4-47_320	TTTTGACCACGCCTCCCTCAC
3D-0930440-T4-47_384	TTTAAAAATCCCCTTCGCAAA
3D-0930440-T4-47_448	TTCCGAAGGAAATGTGGTATT
3D-0930440-T4-47_576	TAGATCACTAAACTAGTGATCT
3D-0930440-T4-47_640	TATATATGTGCGGCTTCGATCA
3D-0930440-T4-47_704	GATAATAAGTCCAACAGCATC
3D-0930440-T4-47_768	TGGTGACTTGCACTTTTTTTTA
3D-0930440-T4-47_896	GCCTAGCTTTGTCGTTTGTGC
3D-0930440-T4-47_960	TTTTTGAAAATTTACTACTCCA
3D-0930440-T4-47_1024	TAGTACAAAGCTTGAAAAACA
3D-0930440-T4-47_1088	ATACCACGTCTAACTAATTCGT
3D-0930440-T4-47_1152	AAAAGCTTGAATCTCCAGGAA
3D-0930440-T4-47_1280	CGATCGTCGCAAGGTTAATGA
3D-0930440-T4-47_1344	TCAAACTGGAGCCTCAACCGC
3D-0930440-T4-47_1536	CTGCGACGGAGGATCCCTTTT
3D-0930440-T4-47_1580	AATACAATACGATGGGGGGA
2A-0343440-T4-48_0	CGGACGGTGGCGCTCCGGTT
2A-0343440-T4-48_192	GCGATGGAGACGACGCTCGG
2A-0343440-T4-48_512	GCCTCCGTGGGCTGCGCCGC
2A-0343440-T4-48_576	ATCGCAATCACCGGATAGTTTA
2A-0343440-T4-48_640	TGCCTCATTATTTTTCCTGGAT
2A-0343440-T4-48_704	AGCTTATCCACACCTTTTTTTT
2A-0343440-T4-48_896	GACACGCAACTAGGAGTGTG
2A-0343440-T4-48_960	AATGGAAGTACACTTAGACCG
2A-0343440-T4-48_1024	TCTTGCAACATACCATATGCCT
2A-0343440-T4-48_1088	ACATGATGACATCTTCTTCTCT
2A-0343440-T4-48_1152	TTATCTATTTATCTTGAGCGCA
2A-0343440-T4-48_1216	GAACCAGTTGGAAAACTAATC
2A-0343440-T4-48_1280	AATGAATAAGTGTCCCACTTG
2A-0343440-T4-48_1344	ATCCTTTATTCTCGCCACAACA
2A-0343440-T4-48_1408	CCATCTATTAGTGTCTCGTAGT
2A-0343440-T4-48_1580	CATCCCGAATGTGTTGAAGCA
2B-0458840-T4-48_64	CCGGACACAGAGTAAGAGAG
2B-0458840-T4-48_128	AAAAATAAAAATACATTGAAA
2B-0458840-T4-48_192	TGTCCCTGGTTTTCATTTCTAA
2B-0458840-T4-48_448	ACCCCTGCGGTGGTGCTTTTG
2B-0458840-T4-48_512	CGTTTCCTTCTTTCCGTCTCGT
2B-0458840-T4-48_576	GAGGAAAGTGGACTCTAGGT
2B-0458840-T4-48_640	AGAGCGTGACAGCCAATCTCA
2B-0458840-T4-48_704	ATTCTTAGCTCTTGGCTCCGTG
2B-0458840-T4-48_896	GCCTGGACAGTTGCCATAGGA
2B-0458840-T4-48_960	CCGATTTTTCTCCTCCACGTGC
2B-0458840-T4-48_1024	TGTTTTATGGTTGCCACAAGC
2B-0458840-T4-48_1152	ATCAATGGGTAAGAATGTTTG
2B-0458840-T4-48_1216	AAATCTGTCGCAAATCTCATG
2B-0458840-T4-48_1280	TGGGTATCCAGTTCTCTAGCC
2B-0458840-T4-48_1344	CTACTTTAGAACATCAACCCCT
2B-0458840-T4-48_1536	TTGAAACCCAGTCGACCCATA
2D-0593120-T4-48_0	CGGCGGCGATCCGGTTGGAT
2D-0593120-T4-48_192	TGGGGACGACGCTCGGGCGG
2D-0593120-T4-48_320	CGGGCCGTGTGGCGAACCCA
2D-0593120-T4-48_576	GGGCGGTGAGGTCGACCGGT
2D-0593120-T4-48_640	TTGAGTGGCCAGCCATTAACT
2D-0593120-T4-48_704	CATTGCCATACCACTCCTTGAC
2D-0593120-T4-48_768	TCTTTCTACCTTTGACATAGTA
2D-0593120-T4-48_832	TCTCGATTCAACTGCATCATGT
2D-0593120-T4-48_896	GAAGCACTTGGCTTTGTGTCT
2D-0593120-T4-48_960	TCTTTCTTCAGAGCCTGCTCG
2D-0593120-T4-48_1024	GAATCTAATTAAGGCATCTAGT
2D-0593120-T4-48_1088	ATGGCATCGACACAGAAATAG
2D-0593120-T4-48_1152	CATATGTCTGACCACAGAAAA
2D-0593120-T4-48_1216	ACCTGCCTTCAACATATCCATG
2D-0593120-T4-48_1344	CCCGGCCCCACCCGTCAATCT
2D-0593120-T4-48_1408	CTCTATTACTTTATCAGTAGGA
2D-0593120-T4-48_1472	TCTTCAACTGTGATGCCCTGTT
2D-0593120-T4-48_1536	TTGTTTTGTATCGCTACCGACC

										2D-0593120-T4-48_1580	AACATTTGAAGACAAATGAAT
										1A-0031460-T4-49_128	TTATTTATTTTTCTTCCTTTGAC
										1A-0031460-T4-49_192	TCCGTTCCGCGCTCCCGTGTTT
										1A-0031460-T4-49_256	AAGTCTTTTCATTTTCTTGGGA
										1A-0031460-T4-49_320	TGGAAAACTATTTAGTTTTTGG
										1A-0031460-T4-49_384	GACGCTACGCTCGACATTTGA
										1A-0031460-T4-49_448	TGAGAGATGTAAAGAAAAAA
										1A-0031460-T4-49_512	CTTTTCAAATCAATGTGAACCT
										1A-0031460-T4-49_576	TGGGAAACGTGTGGCCTTTTT
										1A-0031460-T4-49_768	GGTTGCAGTTATGATCAATTTA
										1A-0031460-T4-49_832	AGGAGAAGGCGGAGGGGTA
										1A-0031460-T4-49_896	CAAAAAGGGGGAAACACTAG
										1A-0031460-T4-49_960	TATGATCAAGTTGTCAGATCGG
										1A-0031460-T4-49_1024	GTGGAGAGACAGGGTAGATT
										1A-0031460-T4-49_1088	GAGAAGGCTAAAAGGTGGGG
										1A-0031460-T4-49_1152	AAGACGTTGGAGGTGTGATG
										1A-0031460-T4-49_1216	TTAGCGGATGGACAAAAAAG
										1A-0031460-T4-49_1280	GTAGACTACAGTTATGATCAA
										1A-0031460-T4-49_1344	AGGGAGAAGGAGGGTAGGG
										1A-0031460-T4-49_1408	AAACACCAGACGCATTGTGAA
										1A-0031460-T4-49_1472	TATTATGTCGATAAAAAAAGAA
										1A-0031460-T4-49_1536	TGTTGTTGTGATCAAGATATCA
										1B-0148870-T4-49_122	TATAGTGTTATTTATTTATTTTC
										1B-0148870-T4-49_183	GGGGCTTGACATGTCCGTTCC
										1B-0148870-T4-49_244	CTCTTTTCCTAAAAGGTCTTTT
										1B-0148870-T4-49_305	TATTGTTGTGCTTGTGCCATGG
										1B-0148870-T4-49_366	GGTGAAATTGTGAGGGTACCA
										1B-0148870-T4-49_427	TACTCTAGTACCATAAGGTGGA
										1B-0148870-T4-49_488	GGATAGGACTGCACTTTGATC
										1B-0148870-T4-49_940	AAGGGGAAACATTGGGGCAT
										1B-0148870-T4-49_1004	GTTATCATTTTGGCTGAATCAG
										1B-0148870-T4-49_1068	GGCTACATTACATTTATAGTCA
										1B-0148870-T4-49_1260	GAAGTTATCAGATGGACCAAA
										1B-0148870-T4-49_1324	GCGGGGTAGACTGCAGTTATG
										1B-0148870-T4-49_1388	GCGTGATGAAGGATAAAGCG
										1B-0148870-T4-49_1452	CCAAAAGGGGGGAACGCCA
										1B-0148870-T4-49_1516	TTATGATCAAGCTATTAGATCA
										1B-0148870-T4-49_1580	CGAAACAACGAGGTAGAGTG
										5A-1199640-T4-50_0	GACTGGCCAACAAATTGCTCT
										5A-1199640-T4-50_64	TTTTGTCTCCTCCTATGGGTAC
										5A-1199640-T4-50_256	GTATGGCAAATCACACATGGG
										5A-1199640-T4-50_320	CTTTGGTTTAAAGTATGATTTC
										5A-1199640-T4-50_384	GGATGATTGCTGAGTCACATA
										5A-1199640-T4-50_512	TGCAAATTTGACATTTAAACAT
										5A-1199640-T4-50_640	TTCCTATCAAATTCTATCCTACA
										5A-1199640-T4-50_704	CATAAATGTGGACTATAGTCGT
										5A-1199640-T4-50_768	GCTAAACAGGGAGGAGTTTT
										5A-1199640-T4-50_832	GCGACAGTACAGCTTGTCAAC
										5A-1199640-T4-50_960	GGCCATGCATTTTGTTGTTGAT
										5A-1199640-T4-50_1024	CTTTTTTTTTTGCGCATATGCA
										5A-1199640-T4-50_1088	TCCTTTAGCTCTGACTTTTGGA
										5A-1199640-T4-50_1280	CCGTCTAGACGCCCAGTAGGC
										5A-1199640-T4-50_1344	CCTGCCATGTAGGTTGAGAAC
										5A-1199640-T4-50_1472	GCGTTCGCCCGATTGACAGTC
										5D-1414830-T4-50_0	GTGTGTTATCTTCTTTTATGCA
										5D-1414830-T4-50_64	CTTATCCATTTGTGCTTTTGATT
										5D-1414830-T4-50_128	ATTTTTATGTCAGTTACATACA
										5D-1414830-T4-50_320	GCGACAGGTAATATGGATCGG
										5D-1414830-T4-50_704	AAATCAAAGGAGGCCCTACAT
										5D-1414830-T4-50_768	TCACAGGGAGAGAAAAATGT
										5D-1414830-T4-50_832	AAGAAGAAGAGGCATGTCAG
										5D-1414830-T4-50_1024	ACTGCAAACACAATCTCCTTC
										5D-1414830-T4-50_1088	TGACTTTTGGATGATGATTATT
										5D-1414830-T4-50_1280	GTAGGCCACGGCCGTGACAG
										5D-1414830-T4-50_1344	TCTGATACCAGCCATGAGAAC
										5D-1414830-T4-50_1408	AAAGCCGCGTCCAGTCTTCAG
										5D-1414830-T4-50_1472	CGCCCGATTAACAGTCGCCGG
										1A-0069860-T4-51_0	GGTGAAGTTCTTGACGAAGG
										1A-0069860-T4-51_130	ATAGGGCTCGGGATGCTCCTC
										1A-0069860-T4-51_325	TTGTCAAGGACTTCTCTCAGC
										1A-0069860-T4-51_390	GCAAAGTAGCCCACTACGGG
										1A-0069860-T4-51_455	AAAATAAAACAAACAATCAGA
										1A-0069860-T4-51_520	TGTTCTTCTGGCAGAAAAAAT
										1A-0069860-T4-51_585	GGCGGGCCGATCTTTTCCAAA
										1A-0069860-T4-51_650	GTTGCAATAAGACATTTACATT
										1A-0069860-T4-51_715	GTTGACGTCGCAATTGTAACG
										1A-0069860-T4-51_780	CCCTGTGTTGTGGTTGCAACA
										1A-0069860-T4-51_845	CTGGTTGCTTGCTGATGACAA
										1A-0069860-T4-51_1505	GTTGCAGGGATACCATTTTTAT
										1A-0069860-T4-51_1580	TATATTTATATTCTTACAACCCA
										1B-0172070-T4-51_0	GGTGAAGTTCTCGACGAAGG
										1B-0172070-T4-51_64	AGATGGTTGTTATCTGCAGGT
										1B-0172070-T4-51_128	GGGCTCGCGATGCTCCTCGAG
										1B-0172070-T4-51_320	TTGTCAAGGACTTCTCTCAGC
										1B-0172070-T4-51_384	CGTAAAGTAGCCTACTATGGG
										1B-0172070-T4-51_448	AAAAATAAAACAAACTATCAA
										1B-0172070-T4-51_512	ATGTTTTTCTTCTAGCAGAAAA
										1B-0172070-T4-51_576	CGGCCGGGTGATCTTGTCCAA
										1B-0172070-T4-51_640	CTCATTGCAATAAGACCATTAC
										1B-0172070-T4-51_704	GCAAAAGGCATCTCTAACTCG
										1B-0172070-T4-51_768	CATCTCCGGCCGTGCCCGAGT
										1B-0172070-T4-51_832	TAGAGTGCTGCTTGTTGTTGA
										1B-0172070-T4-51_1024	TTGCCTACTCCTCGCATGAGTT
										1B-0172070-T4-51_1216	TTGCAGGGATACCATTTTTATT
										1B-0172070-T4-51_1280	TATTACTTATATATTTTTACTCTT
										1B-0172070-T4-51_1344	AAAAAAATATGATGGCATTTG
										1B-0172070-T4-51_1408	ATCCACTTACAATACACCTTTT
										1B-0172070-T4-51_1472	AAAGATTGGAATCACATAGCT
										1B-0172070-T4-51_1536	CCCTCAGCAGCAGAGCTAGG
										1B-0172070-T4-51_1580	TCCGCTAGCCAGCTGGTGGAT
										1D-0264230-T4-51_128	GGGCTCGCGGTGCTGCTTGA
										1D-0264230-T4-51_320	TGTCAAGGACTTCTCTCAAGA
										1D-0264230-T4-51_384	GTAAAGTAGCCTACTATGGGG
										1D-0264230-T4-51_448	AAAAAAAAAATTTATCCGAGG
										1D-0264230-T4-51_512	GATCGGGAGGCTTGACCAAG
										1D-0264230-T4-51_1088	GGGGGCCACCGCGCCTCCAT
										1D-0264230-T4-51_1152	CGATAGGAGGGAGGGGACCA
										1D-0264230-T4-51_1216	AGAAAAGCGGTGTCGAGTCTT
										1D-0264230-T4-51_1280	AGGGATTACCTTTTTTGTTTTA
										1D-0264230-T4-51_1344	CTCTTAGAGCCCATAATGTTAA
										1D-0264230-T4-51_1408	GAGGCTTTTTTGGCTATATAA
										1D-0264230-T4-51_1472	CCAAATATAATGTAAGTAGGTA
										1D-0264230-T4-51_1536	GGATGGGAAAAAACTCTTTCC
										7A-1755470-T4-52_0	GGCAGCGTGTCAGCCGGCCG
										7A-1755470-T4-52_64	tcttgcgtctTTGGGAGCTACTAG
										7A-1755470-T4-52_128	GCAACGGAGCGCCACAGCGT
										7A-1755470-T4-52_192	ATCGTCCGGCGCGTCGCGGTC
										7A-1755470-T4-52_256	CTTCCGGGAAGCGTCGTTGAT
										7A-1755470-T4-52_320	CGTGCGCCACGTGCGCGCGT
										7A-1755470-T4-52_512	TTTGGGTGGAGGGAAGAGCC
										7A-1755470-T4-52_640	CAGCTATCTCAGAGAATGACC
										7A-1755470-T4-52_704	TAAAAAAAAAATAGTAACAACC
										7A-1755470-T4-52_768	TGGAGACAAAATTACCCCCAC
										7A-1755470-T4-52_832	CAGTTACACCCAAGACATGAT
										7A-1755470-T4-52_896	TGTTCTTATATTATGAGATAGA
										7A-1755470-T4-52_960	ACCTATTACATGCACATCACCC
										7A-1755470-T4-52_1024	CTCATGCATGTTTTGTCCATTG
										7A-1755470-T4-52_1088	CACTTGCACGCATCTGGTGGA
										7A-1755470-T4-52_1152	CCGTCCACAAAATAcacttacaa
										7A-1755470-T4-52_1216	ccctctatggtctattggagaacaata
										7A-1755470-T4-52_1280	gttggtcagactaccaaaagaaatgc
										7A-1755470-T4-52_1344	TCGTCATTACGACCCAACCAA
										7A-1755470-T4-52_1408	GACTTATCCACCCCCGCGAAG
										7A-1755470-T4-52_1472	TCGAGTTAAGGAGCATCACTC
										7B-1860310-T4-52_0	GTCAGTGGGTCAGCCGGCCG
										7B-1860310-T4-52_64	GAGCTAGGAAGAGCTCGAGA
										7B-1860310-T4-52_128	CGTCCACCAGCCGTTGGATCG
										7B-1860310-T4-52_192	GGTCTCGGAGGTGGCTGTCT
										7B-1860310-T4-52_256	GATCCGTCGTCCCTGCTAGGT
										7B-1860310-T4-52_320	GGCCCGGCTGGCCCCGGCCC
										7B-1860310-T4-52_512	ATGAATTTCGGCACGTGCGCC
										7B-1860310-T4-52_576	TTGGAGACCGTGGTCACATCG
										7B-1860310-T4-52_640	GGAGAATCATGTAAAAAACCC
										7B-1860310-T4-52_704	ACTACAAAATCACTCATCGTTT
										7B-1860310-T4-52_768	TATGATCTGACGCATATATATG
										7B-1860310-T4-52_832	ACATCTACGCACATCATCCAAA
										7B-1860310-T4-52_896	GTGTTTTGCCCATTTGTGCGTT
										7B-1860310-T4-52_1088	ttagtacaaagttgagtcatctatttag
										7B-1860310-T4-52_1152	ATCTTGAATGCCCAACCTTCTA
										7B-1860310-T4-52_1216	AGATACTCCATCTTTGATAGTC
										7B-1860310-T4-52_1280	CCGGTAAAAATCTTGTCTTGT
										7B-1860310-T4-52_1344	CACTGCTGCTACCCCTACAAG
										7B-1860310-T4-52_1408	GCCAAACAATGTGTGACATAC
										7B-1860310-T4-52_1472	TTCATTTCTGGAAATGTTAAAA
										7B-1860310-T4-52_1536	CCACGACCAGTTCTAGTACTA
										7B-1860310-T4-52_1580	TAAAATCTATTGATGCCTAGGT
										5A-1223530-T4-53_0	GGTTGGAATCACCGCAGCAA
										5A-1223530-T4-53_64	GTAGCGTGGTGCAAGTGACA
										5A-1223530-T4-53_128	GCTAACGTAATCGAGCCACTC
										5A-1223530-T4-53_192	GTAGCTGGGTGAGCGAATGA
										5A-1223530-T4-53_253	AAATACCGCTGGGAAGGCCA
										5A-1223530-T4-53_1522	TGTCAGGTAAAAGTTGAAACT
										5B-1314640-T4-53_0	GGCGGGAATCACCGCAGCAA
										5B-1314640-T4-53_64	GTACCGTGGTGTAACTGACAG
										5B-1314640-T4-53_128	GCTAACGTAATCGGATCACCC
										5B-1314640-T4-53_192	GTGTGTCTACTGGGCTGGGA
										5B-1314640-T4-53_320	CGAGGCCGCTGGCCTGGGTG
										5B-1314640-T4-53_768	TGCCGCTCGTCCTCTTGTCTTC

5B-1314640-T4-53_1024	CGCCAAGCAGCAGCCAGTAA
5B-1314640-T4-53_1088	TTATTCTCGGGACAACCACGT
5B-1314640-T4-53_1152	ATATAACACAAATTTATCATTA
5B-1314640-T4-53_1280	TTTTTTGAGCTAACATAGTCTG
5B-1314640-T4-53_1344	ACAAACGAATCAGATGTTTCT
5B-1314640-T4-53_1408	CTTCTTGCTTTCAACCACTTCT
5B-1314640-T4-53_1472	TCGACTCCTCTGTTTCTCTCAG
5D-1462650-T4-53_0	GGTGGGAATCACCGCAGCAA
5D-1462650-T4-53_64	ATGAAAAAATAATGGCTAGTG
5D-1462650-T4-53_128	ATCAGTAGTAGTAGTATCTACG
5D-1462650-T4-53_256	AGGAAGCACGCAAGGAAACG
5D-1462650-T4-53_576	TGCCGTGCGAGGCCGCACGC
5D-1462650-T4-53_768	CGCCTTTCTTCTTCCTCTTCAA
5D-1462650-T4-53_1024	CTCGCCCGCAGACAATGCCGA
5D-1462650-T4-53_1088	CAAACCATAGTATTCATGCAAA
5D-1462650-T4-53_1152	CAACAATTCAACAAAACAAAA
5D-1462650-T4-53_1280	ACGTTCTTATATTATGAGACAA
5D-1462650-T4-53_1408	AGTGTCCTGCAACATAATCCT
5D-1462650-T4-53_1472	TCTCAAGCACAATTCCTTGCAT
5D-1462650-T4-53_1536	CTTCAAGAGTCTCTCTCTCTCA
7A-1831290-T4-54_64	GAAAGCAAGGCGATGGAGCG
7A-1831290-T4-54_320	AGATAGGTCGACGAGGAGAA
7A-1831290-T4-54_384	CGACGTGGCCATGGCTCATGG
7A-1831290-T4-54_448	CAGATTTCAGATCAAATCTACG
7A-1831290-T4-54_640	GCCCGCCCGGCCGCCTCCGA
7A-1831290-T4-54_832	CACCTGTCCCCGTATTGGAAA
7A-1831290-T4-54_896	GCCAGGGACTAGAGCATATG
7A-1831290-T4-54_960	GAACTCCTGATAGCACATTTT
7A-1831290-T4-54_1216	TAACCAAGTCTCAGTCGGCTG
7A-1831290-T4-54_1279	ATAGGGTTCAATACATATTTTT
7B-1921350-T4-54_0	CGTCGGCGCGTCTCTCGGTG
7B-1921350-T4-54_448	TGCATGGGTTGGACTTTGGA
7B-1921350-T4-54_512	GATAGATCGCCGAGGGGCGC
7B-1921350-T4-54_640	ACTATGAGATTCAGATCAAAT
7B-1921350-T4-54_832	ACGGAAATGCTTTTTGTTCTT
7B-1921350-T4-54_960	TGGTGTGGTACCGTTGAGCT
7B-1921350-T4-54_1024	CGGTTGAAAAAAAAAACATG
7B-1921350-T4-54_1088	CTTCTTTTTGAATAGTTTGGAT
7B-1921350-T4-54_1152	CCTTCTTTTACGGTGCGAGGT
7B-1921350-T4-54_1216	ATAATACGTACACGTCCTTGAT
7B-1921350-T4-54_1280	CACAGTATAACACAAAGGCAT
7B-1921350-T4-54_1344	TATTTCTAGGAACCTAAGGAA
7B-1921350-T4-54_1408	AAAAACACAAGCGATTACAAA
7B-1921350-T4-54_1472	TGAATATGAACATAATTATCTAT
7D-2062950-T4-54_256	CTTTGGAGTTTGCGTCTGTGG
7D-2062950-T4-54_384	ACTGGCAAGCAAGCGACGTG
7D-2062950-T4-54_448	GACTATCAGATTCAGATCAAT
7D-2062950-T4-54_640	AAATGCTTTTTGTTCTTCATCA
7D-2062950-T4-54_832	GAAAAAAATGTGTGCTGGGT
7D-2062950-T4-54_896	GCCAGCAGTCCTGAACTTCAT
7D-2062950-T4-54_1024	TAATTAATCGACGAGGTAAAA
7D-2062950-T4-54_1088	TTTTTTCAAAAAAAAGAGGCA
7D-2062950-T4-54_1152	TACCCCGCTAAAATATATATGT
7D-2062950-T4-54_1216	CTAAAACACCATCCGTTTTGA
7D-2062950-T4-54_1280	TCTGGCTTTGGGCCTCGCCGG
7D-2062950-T4-54_1344	GCCCGGGCTGCCTATATTTGC
7D-2062950-T4-54_1408	TTTTTAGGGATCCAAGGCTAT
7D-2062950-T4-54_1472	TTACTTCTCCAAACGTGAAAA
7D-2062950-T4-54_1536	TTAACATATTATAGACTTTCTAA
2A-0311710-T4-55_0	TTCTCGTGGGACTCATCTGAC
2A-0311710-T4-55_128	TCTAATCGGCACCTAGTCGAT
2A-0311710-T4-55_192	ATCTGGCGCTTAAATTTATTCG
2A-0311710-T4-55_320	CTCATTGCCTTCCTTGGCCCT
2A-0311710-T4-55_448	CGGCCATCGCATCTTATGTTAC
2B-0425810-T4-55_399	GGGTTGGGTTGGGCAAGCAA
2B-0425810-T4-55_462	TGATTGCGCGTGGATTATCTT
2B-0425810-T4-55_525	TTTGTGGAAGGTTTTGGGTG
2B-0425810-T4-55_588	GGGGGTAGTGGTGGTGGGCG
2B-0425810-T4-55_651	TTAGTGGGGGAGGAGGTCAA
2B-0425810-T4-55_777	GTCCCGAGCTGTAATCATACG
2B-0425810-T4-55_840	CCAGATTTCATGCCAAATGCC
2B-0425810-T4-55_1155	CGGATGAACAAATAGGGGCT
2B-0425810-T4-55_1218	AGAGGTATGTGTGGATGAAG
2B-0425810-T4-55_1344	CAACTGTTAAAATGACGAGCG
2B-0425810-T4-55_1407	ACACAAATAATACTTTACTACC
2B-0425810-T4-55_1580	AAAGTTTTGCTTCCGGAGTCG
2D-0518190-T4-55_0	CCCGTCGAGCTGGTGGTGGG
2D-0518190-T4-55_64	TTGGTGGCCTGGTGGATTGG
2D-0518190-T4-55_128	GGGATTAATTGGGGGGACTG
2D-0518190-T4-55_192	GGGAGGCGACGACTCTCCCT
2D-0518190-T4-55_384	GTTGTAGCCGTTGTGGGCTTG
2D-0518190-T4-55_576	GGATTTCCTGCCAAATGTCGC
2D-0518190-T4-55_640	CAAGACCTAAAGGGTCTGATC
2D-0518190-T4-55_704	AAACTGCTAAAATGACGAACT
2D-0518190-T4-55_768	CCTAATATTCGCATGCAATTAA
2D-0518190-T4-55_832	CTTGGTCTGCCTATGTTGCCA
2D-0518190-T4-55_896	ACAATTTTGCTCTTCTCCTACG
2D-0518190-T4-55_960	GTGCCTACTCCCGGCTGCGGA
2D-0518190-T4-55_1024	CGGTGAACATTTCTAGCTAGT
2D-0518190-T4-55_1216	GCCTAGTCTCACATTTCAGCC
2D-0518190-T4-55_1280	GACCCAACAATCAACAACAAA
2D-0518190-T4-55_1472	CCTTTATATTCCGTCGAATGTT
2D-0518190-T4-55_1536	AGATGTCCTTTCTTTTGTGCG
2D-0518190-T4-55_1580	GAGAGCCGGCCATGCAATCAT
1A-0078240-T4-56_256	acacagaacccagacccaagcccaa
1A-0078240-T4-56_320	GCCGGAGTTCCCTGTCTCCGG
1A-0078240-T4-56_512	GCGAGGAAGGTGGCAGGAA
1A-0078240-T4-56_576	CTAGGTGCGTGCGGGTGTGAT
1A-0078240-T4-56_640	CCAAAAGCGGCACGAGGGGT
1A-0078240-T4-56_768	CAATGGCGGTATCCGCTTCCAT
1A-0078240-T4-56_832	CTCCTCGTGCCAAAGGAAATG
1A-0078240-T4-56_896	CCATCCACGTCGCTGATCTATC
1A-0078240-T4-56_960	CCGCCCCCTTTTGCGCACTGC
1A-0078240-T4-56_1088	CGTGAGCACAAAGGCGCCGC
1B-0157010-T4-56_192	TGTTAGCGTGCGGAGCCGGG
1B-0157010-T4-56_256	AGAACCCGAACCCAGACCCA
1B-0157010-T4-56_320	GCGCCGGAACTCAACCCCGG
1B-0157010-T4-56_384	GGAAAGGGGAgtggagggggtg
1B-0157010-T4-56_576	GGATCGGTGGTACTCCTACgtg
1B-0157010-T4-56_704	GCGCGGCGCGAGGGGTAGA
1B-0157010-T4-56_768	GGTACACTCTGCTCGGCTTCT
1B-0157010-T4-56_832	tcctcctcctcctctccctgcctccgcc
1D-0249290-T4-56_192	TAGCGTGCGGAGCCGGGATT
1D-0249290-T4-56_256	gacccaaacgcaaagccGGCACG
1D-0249290-T4-56_320	GCGCGGCCGGCAGTCACCGG
1D-0249290-T4-56_512	GAGGAAGGTGGCAGGAAGC
1D-0249290-T4-56_704	GCTCCTGTTTCACAGTGGTAG
1D-0249290-T4-56_768	GGCGGCACCTGCTCACCCGC
1D-0249290-T4-56_832	TCTTCCGAGCGTCCCTGCACA
1D-0249290-T4-56_896	TGATGACCGCGTGCTGTACGT
1D-0249290-T4-56_960	CTGGCGAGGAGTGCTATTTCA
1D-0249290-T4-56_1024	AATGTGCTGCTTGTAGATTTCT
1D-0249290-T4-56_1088	GTTGGAATTTCCTCATCTCTCG
1D-0249290-T4-56_1152	GGAGTCATCACTTAACAGAGA
1D-0249290-T4-56_1216	CGTCTGAAACAGGTGTCAGC
1D-0249290-T4-56_1280	GTAATCATGTCACAAATGTGG
1D-0249290-T4-56_1344	CTCATCACGTGTTAATGACAAT
1D-0249290-T4-56_1408	CGAGCATGAACTGTTCGACTC
1D-0249290-T4-56_1472	AACATCAAAATTGGCGTGAAC
1D-0249290-T4-56_1536	TTTAGGGCTTCTCCAACATTG
1D-0249290-T4-56_1580	CCGGACCCATTTCACCATCCA
3A-0666460-T4-57_128	TCCCTTGTCGTACTCTTCCTCG
3A-0666460-T4-57_192	GTATGGATACAGCCGATGCCG
3A-0666460-T4-57_256	TTTTTTTTAATGAAATATGTCG
3A-0666460-T4-57_320	GTATGCAATTTATCAAGTATAT
3A-0666460-T4-57_384	TTGCTTTTGGTATTGTGTAGCA
3A-0666460-T4-57_448	GTACCAGTACTTAATAATATGT
3A-0666460-T4-57_512	AGTGTACTTGTCTTCATAATGT
3A-0666460-T4-57_704	TACGTACGACGCAAATTAATTA
3A-0666460-T4-57_832	TACATCTATTTTCAAAATAAATA
3A-0666460-T4-57_896	TAAAACCAGAAGGATTCCGAA
3A-0666460-T4-57_960	CTCCAAGTTATTAAACGCGGC
3A-0666460-T4-57_1152	TAACTTGGACTTGTGCTCCCG
3A-0666460-T4-57_1216	TCCTCCTAGTATATAGCTGCGC
3A-0666460-T4-57_1280	CTCCACTTCCATCTGCTGCGC
3A-0666460-T4-57_1344	CTACTAGTTCTACTTCTACTTCT
3A-0666460-T4-57_1408	GGCCATGTTTCTTCATGGAGC
3A-0666460-T4-57_1472	TTCCTTCATCAGTCTTTTGGTT
3B-0717870-T4-57_320	CAAACTGTATATCCTGGTCATG
3B-0717870-T4-57_448	ATGCGAGCTCGTCCACCATAT
3B-0717870-T4-57_512	GTATGATCAACTTATTTGCTGT
3B-0717870-T4-57_576	AATGCAGTGCAATTGTGATCAT
3B-0717870-T4-57_640	ATTCTTGTACTATGTACATATTC
3B-0717870-T4-57_704	AGGGGTACGTGGTCAGCCAG
3B-0717870-T4-57_768	AGCAGGCGCTATGGGTGGGT
3B-0717870-T4-57_832	TACCTGCAACGCTGTGTGTCC
3B-0717870-T4-57_896	ACGAGAAGACGCTGTTTTCTT
3B-0717870-T4-57_1088	CAGCCACAGCCGGTCTTCCGG
3B-0717870-T4-57_1152	CAACCGCAACCCACGCCGCTT
3B-0717870-T4-57_1216	TAGTATATAGCTGCGCAACTCC

3B-0717870-T4-57_1280	TTCTCTGCTCCACTTCCATTTC
3B-0717870-T4-57_1344	TAGTCGATTGTTGCTCTTCTTC
3B-0717870-T4-57_1408	ATGGTCTAAGCCTGTTGTTATA
3B-0717870-T4-57_1472	CTCTGCTTACGATAAGGCTGT
3D-0896070-T4-57_64	CTTGATGTTTCTGCAAGGGCA
3D-0896070-T4-57_128	TGGTTTGGTAGGTGTTGGGCT
3D-0896070-T4-57_192	CCGAAAGACTGAAGGAAGAA
3D-0896070-T4-57_256	CATGAAGAAGCATGGCCAAG
3D-0896070-T4-57_320	ACTAGTAGATGGTGAACGTAC
3D-0896070-T4-57_384	AGAGAGCGGGAAGGTGGAA
3D-0896070-T4-57_448	CGCAGCTATATACTAGGAGTA
3D-0896070-T4-57_640	GTGCGTGCCGGACTTCCCACG
3D-0896070-T4-57_704	GATGGCCGCGTTCAATAACTT
3D-0896070-T4-57_768	CAGTCCAAGAAAACAGCGTC
3D-0896070-T4-57_832	TTTACCAGGACAGCGTTGCAG
3D-0896070-T4-57_1088	ATTGAGACGCTAATTAATTTG
3D-0896070-T4-57_1152	TCCTCCGTGTCGACATCCACG
3D-0896070-T4-57_1216	atataCAAGAATACTACTCGCA
3D-0896070-T4-57_1280	TGAAGACAAGTACACTGTTTT
3D-0896070-T4-57_1344	TTTAAGTATAGGTACATAATAC
3D-0896070-T4-57_1408	CAATACCAAAAGCAAATGATT
3D-0896070-T4-57_1472	GATAAATTTGCATGCAAACCA
3D-0896070-T4-57_1536	TCGACATATTTCATCAAACAAA
3D-0896070-T4-57_1580	TAAAACTATGGCCGCGCCCGC
5A-1243870-T4-58_0	CCTGAAAAACGCCATGCATGT
5A-1243870-T4-58_64	TCTACTCTTTTTTTTTTTACTT
5A-1243870-T4-58_128	GAACATTGTTGCTGATTCCGC
5A-1243870-T4-58_256	TATGTTGATGGTCCTCTTGTTC
5A-1243870-T4-58_320	TATCGTTGTGTAAGCAAATCAT
5A-1243870-T4-58_384	GGAGTAAATGAGAGAGACCA
5A-1243870-T4-58_448	CAGGGGAATGAggctggctggct
5A-1243870-T4-58_576	TGAGACTCGCCGCCGACCCG
5A-1243870-T4-58_640	CCAGGCTGAATTTTGGTCTCC
5A-1243870-T4-58_704	ACCAGACCAGAGTTGAACGC
5A-1243870-T4-58_896	AGCTAGGGGCCCCCACCCAG
5B-1330040-T4-58_62	GCATGCATCTTTGAGATGCAG
5B-1330040-T4-58_124	GGGAGGAGTAGCAGTAGCAG
5B-1330040-T4-58_186	gttgaattgagtttgagtACTAGTAC
5B-1330040-T4-58_248	GATCGTGAATGGATCGTGTAT
5B-1330040-T4-58_310	ATGACGTATGGTAAACCTTAAT
5B-1330040-T4-58_372	GGATCTTCTAGAAGGAGTAGT
5B-1330040-T4-58_434	CTTAAAGATATACGGACGAGA
5B-1330040-T4-58_496	AATTTCAATTCAACTCAATGG
5B-1330040-T4-58_558	ATTTAATAGGAGTATTTCTTGT
5B-1330040-T4-58_620	AAACAACCAATCAATCGTAAT
5B-1330040-T4-58_682	ATACTACTAGGTATATAGAGTA
5B-1330040-T4-58_744	TTTCAGGTGCGTGGGTGAGT
5B-1330040-T4-58_806	TTGTTAATTAACGATGGATATG
5B-1330040-T4-58_868	AGTTGGAAGCCGTAGCATATG
5B-1330040-T4-58_930	TTCTTCTTCCTCTAAAGGAGC
5B-1330040-T4-58_992	tcatctccatctccatctccattttcatct
5B-1330040-T4-58_1054	gtactaggactagtactattagtagtta
5B-1330040-T4-58_1116	CAACAGCATGCTGCGTTACTT
5B-1330040-T4-58_1178	ATGCAATGCTAAAAAATCCTTA
5B-1330040-T4-58_1302	TGAGGCAGGCTGCTGAGCAT
5B-1330040-T4-58_1364	TTCTTCTTATCGttcccttcccttcc
5B-1330040-T4-58_1426	TGTATCTCTCTCCTCTATATATG
5B-1330040-T4-58_1550	CCCATTCCCATTCCCATTCCCA
5B-1330040-T4-58_1612	TTCCTTTCCTCCACCACTCTCT
5B-1330040-T4-58_2232	TTCCATTCCATCCTGCTCTCCT
5B-1330040-T4-58_2294	GCTTGGTCTTTTCATTCCGATC
5B-1330040-T4-58_2356	TAGATTTTCATCAATTAAGTCA
5B-1330040-T4-58_2418	gatagatagatagatagataCTCTG
5B-1330040-T4-58_2480	AGAAGGGATATTTGTTTTTAC
5B-1330040-T4-58_2542	TGctcttcctctccatcctcccctcccc
5B-1330040-T4-58_2604	GCGGCGGCCAAAGGAAAGG
5B-1330040-T4-58_2666	CGACGACCACCAGGTCCagag
5B-1330040-T4-58_2728	GGACCAAATGTCTGTCTCTAA
5B-1330040-T4-58_2790	CACCAGACCAGAGACCACTtc
5B-1330040-T4-58_2852	TTGCCTTGCTGTATCAATCAAA
5B-1330040-T4-58_2914	ACTGCTTCTTTATGACTCtctgt
5B-1330040-T4-58_3100	ATACTCCGAAACGTCGACGGC
5B-1330040-T4-58_3134	CCAGGtttcttcttcctttccttttctttt
5D-1454170-T4-58_0	GAAAATTCATGCAACCGCGAC
5D-1454170-T4-58_64	ATTTTTTTAACTAAAATGATAC
5D-1454170-T4-58_192	TCATCAGACTACCGTGAGCCC
5D-1454170-T4-58_256	CATGTTCGGCAGTGTAGCTCA
5D-1454170-T4-58_448	CGCCGCCTCCTCGATCTCCAC
5D-1454170-T4-58_512	ACCACGCGTCGGCTTGGAGC
5D-1454170-T4-58_640	TTTATGATGGAGAACTCCGCT
5D-1454170-T4-58_832	GACGCGTGACCGGAGTAGGT
5D-1454170-T4-58_1024	CTCTAGTTTGTCATGGGCTTG
5D-1454170-T4-58_1088	GAAGCTAGCTACACTATGCCT
5D-1454170-T4-58_1152	TATTTAGTGAGCAAAAACAAG
5D-1454170-T4-58_1216	TCAATGTAGCTTTGCGACTGT
5D-1454170-T4-58_1280	AGCAATGGATGGATGGGATG
5D-1454170-T4-58_1344	CAAATTACCTTTGGTTGGTTG
5D-1454170-T4-58_1472	TTCTATATATATATATGTATGTAT
6A-1508720-T4-59_192	TGGCAGGCTGCATGGCGCCA
6A-1508720-T4-59_256	CTTAAGTTTCGGCTGTTGTTT
6A-1508720-T4-59_320	AAGAAGCAGAAAACGATGTG
6A-1508720-T4-59_512	GTGCTTTTGCTTTTCTATACGT
6A-1508720-T4-59_570	AGTACCCCAACTATGGTAGTT
6B-1582820-T4-59_960	gcatctctctgatgcagaggctggagt
6B-1582820-T4-59_1024	CCTTTTATAAACAATCTATACG
6B-1582820-T4-59_1088	TTAACTATCATAGTTGGGGTAC
6B-1582820-T4-59_1216	CTCCTCTCCCGTCGCCACCGG
6B-1582820-T4-59_1280	TGTAACTCCGTTCAGAAACTT
6B-1582820-T4-59_1344	GTTTTCTGCTTCTTCTCTTCCT
6B-1582820-T4-59_1536	TGGCGGCTTTGCGCCGCGCG
6D-1703710-T4-59_192	TGCATGGCGCCACCAAGGAG
6D-1703710-T4-59_256	GCTGTTGTTTCTGAACTTAAAT
6D-1703710-T4-59_320	AACGATGTGTGTGAAGACTAC
6D-1703710-T4-59_512	TGCTTTTCTATGCGCCAAACTA
6D-1703710-T4-59_576	TGGTAGTTAAATAATGTCTGCT
6D-1703710-T4-59_832	CATTATGAAACGATAACAAAA
6D-1703710-T4-59_1536	CCCTGCAAAAAAAAAATGTCC
6D-1703710-T4-59_1580	TACCTCCGTCCCAAATTAGTTG
6A-1542800-T4-60_648	AGggcggagcggaggaaggaggag
6A-1542800-T4-60_711	TGTCGCTGGTCAGTCAGTCAA
6A-1542800-T4-60_774	CCCCTCTTTCAGACCATTTCTT
6A-1542800-T4-60_837	GTAATGTAATTTTTCTGTTTAG
6A-1542800-T4-60_900	TTTCTGTGTTTCTGTTGTGTGT
6A-1542800-T4-60_1026	GTTTAAATAAGGCCGAATTTC
6A-1542800-T4-60_1215	TCTGGTGGCGGAGTCGTTGG
6A-1542800-T4-60_1404	GAGGGCGCCGGGCCTGGGA
6A-1542800-T4-60_1467	ACTTGGCCATCTCTCATCAAA
6A-1542800-T4-60_1530	GCGGTCTAGGGCCCGGGCGT
6B-1669980-T4-60_128	GGGAGGCGGAGGCGGAGGA
6B-1669980-T4-60_320	TGTCAACACACCTCCCTACCCT
6B-1669980-T4-60_384	ccttgtttctCCTGGTGTAACATAA
6B-1669980-T4-60_576	CTTATGTTTAGGAACGGGAGA
6B-1669980-T4-60_640	TAACTTGTAATTATCTGGTTAG
6B-1669980-T4-60_1344	CGTGCCGCCGTGCCCTCCCTC
6B-1669980-T4-60_1472	CCCCAGCCAACTTTAACGCCG
6B-1669980-T4-60_1536	AGAAAATGTCCTCTCCCTATTT
6B-1669980-T4-60_1580	ATTTGATGCTTCATGAATCGTC
U-2129720B-T4-60_1088	TCTGGTATCCATGTCTATGGAC
U-2129720B-T4-60_1152	GCGGGTCACCGTTGAAGATA
U-2129720B-T4-60_1216	CAAACAAAACGAATAATCAAA
U-2129720B-T4-60_1280	TCGCTGTAACTTAATAAAGCTA
U-2129720B-T4-60_1344	GAAGGGAAGAAATGGTCTGA
U-2129720B-T4-60_1408	GACGGTTTGACTGACTGGCC

Trait&gene no.	Gene	Homoeologues	IWGSC gene ID (refseq1.1)	WGIN Promotome ID	Orientation	ATG	Target Sequence	MYbaits	MYbaits coverage (%)	MYbaits ID	MYbaits sequence
T5-1	DREB2	ABD	TraesCS4A02G035400	4A-1019300-T5-1	reverse	391,260,120	CTTAGGATTGGGTCTTGTCCATCACATCATTCTCCTAATGATGTGATCCCGT	10	46.7	4A-1019300-T5-1_280	GAGATCTGCAAGCGGCGTGCCCAGCTCACGCTGGCACAGTGTCGTGACCCATCATACACAGAGGACTCCCCGAACTAGGATGTGTGGTTCACCAGCAAGCACGA
T5-1	DREB2		TraesCS4B02G268100	4B-1062650-T5-1	forward	541,910,294	TCGACAGTGGCCGATGAGGAGGGGAAGCTCCTCGCGGATGTCGTCCGTC	12	55.1	4A-1019300-T5-1_848	CGGGACGACGGCAACCGGCGCATGCAGTTAGAGCGGGAGGAGGAGCAGCGACGGCGTGGGCACGAGAGGAGGGGCGGTTTGCGAAAAAAACATGTTGT
T5-1	DREB2		TraesCS4D02G267500	4D-1144620-T5-1	forward	438,153,023	CGTTCGGTACTTGGATCGGTCGGATCGTGAAGACGCACGACTACATCAAC	11	56.7	4A-1019300-T5-1_910	ACGAGAGGAGGGGCGGTTTGCGAAAAAAACATGTTGTGTGCCTGTATAGGCTTTCAATCTTCTTAGTAAATTTTTTTGAAACAAATAGGAGTAGGTCCGATCT
T5-10	AK336250(TaGT2L2)	A, B, B	TraesCS4B02G396900	4B-1052320-T5-10	reverse	671,633,841	CTCGGACCGGATCTTGAGCAGCGCCAGCGTCTCCTGCCGCGGCCACCGG	14	67.5	4A-1019300-T5-1_972	CTTAGTAAATTTTTTTGAAACAAATAGGAGTAGGTCCGATCTCCGTGGCGAAAATTACACTCTCGTGTCTCTGCCGTATACAGTACATAGGGTATCACTGTCTGTAT
T5-10	AK336250(TaGT2L2)		TraesCS5A02G558200	5A-1188970-T5-10	reverse	708,809,223	GGCTCCGGCCCTCCCCTCACTCGCCGGCCACTGGTGAGGGAAATTGAGC	8	43.3	4A-1019300-T5-1_1034	CTCGTGTCTCTGCCGTATACAGTACATAGGGTATCACTGTCTGTATGTCCTGTTTCTGGAAGGAAAAAAGAAGACCATTGTTTG
T5-10	AK336250(TaGT2L2)		TraesCS1B02G138600	U-1129010-T5-10	forward	28,394,966	GAGTTATGTCCAGTGTTGAATAAATAAAATCTAAATTGACATGTTTACCTAC	21	98.1	4A-1019300-T5-1_1096	GAGTAAGGAACATCTTTCTGGAAGGAAAAAAGAAGACCATTGTTTGCCCACATTTCAATGGTACTGTAGGATAGTTCGCCACAAATTGCCCGTGTGTACCATCGCT
T5-11	AK333756(TaGT2L3)	AD	TraesCS1A02G176700	1A-0048840-T5-11	forward	317,811,200	ATATTTCTAAGATGCATTTCCATGTGAAAAGAAATGCCATCCTGCCCATACA	10	54.1	4A-1019300-T5-1_1220	CACCAAATCAAAGCGATGTACGGGCGGCCCTTGCATCGCCATCGTATCGTTACATCGACACCACTATTACCACCCCTCGATGTTGTATCGCCATACAATAGTACGTAC
T5-11	AK333756(TaGT2L3)	no B	TraesCS1D02G162400	1D-0190640-T5-11	reverse	231,467,241	GCTAGTACACACTGGGTGCACGTCGCCGGCAAAGCGTTACCAGAGGGGC	12	60.5	4A-1019300-T5-1_1282	ACTATTACCACCCCTCGATGTTGTATCGCCATACAATAGTACGTACAAAATTGGAAATGTACCATTTATAAGACAAACTACTGTGTATATACGTGCAATCTACGCTAGG
T5-12	KC775780	ABD	TraesCS1A02G020500	1A-0074210-T5-12	forward	10,067,169	ATGAGCTATCACGTCTTCCATGGGGATAGAGCGCTTGGACGGCCGCGGC	18	73.2	4A-1019300-T5-1_1344	CATTTATAAGACAAACTACTGTGTATATACGTGCAATCTACGCTAGGCGGCCTTGCAATCCCAGGAGGAAAAAACAAATCCGTGAGTGCTCGGAGACCGTAGAAA
T5-12	KC775780		TraesCS1B02G024500	1B-0156080-T5-12	forward	11,858,237	GAGGACAAGCGCGCGCGTCCGCCCGTCCGTTTCACCCCAAACCGAGCA	15	68.6	4A-1019300-T5-1_1406	AGGAGGAAAAAACAAATCCGTGAGTGCTCGGAGACCGTAGAAACCAGGCTGCCGCGTAGGTCAGTCACTCGACGACAGTCAAGTGACAGCTAGATCTGGACTT
T5-12	KC775780		TraesCS1D02G020000	1D-0258490-T5-12	forward	8,712,804	ACTAAAGGGTTAGGGCGTTAGTAACGACCCTTTAGTCCCGGTTCGAAAAC	18	76.5	4B-1062650-T5-1_0	TCGACAGTGGCCGATGAGGAGGGGAAGCTCCTCGCGGATGTCGTCCGTCGCTCCCTCGCTGGGAGAACGTCA
T5-13	TaFBA1	ABD	TraesCS7A02G178000	7A-1822340-T5-13	reverse	131,835,178	CCCTGATCCTTCCAGGACTGATCATGCAAGTTGTATGATCCACCTGTTTCAC	12	55.1	4B-1062650-T5-1_64	CAGAGACATACTCCCGTCGCCTCCGCTGGGAGAACGTCAAGGCACCCCAGCTCGGCATCAAGCAACCGGAGCACGTAACAAAGGAGAGGCGGCGGCGATGG
T5-13	TaFBA1		TraesCS7B02G083700	7B-1955490-T5-13	reverse	94,311,561	CCCTGATCCTTCCAGGACTGATCATGCAACTTGTATAATCCACCTGTTTCAC	16	78.8	4B-1062650-T5-1_192	CAGCAGCACCATGCCGACCAGAGGATGGCCGACTTCATCGACTCCTAGTTTGATGTCATCGATGACTCCTACTCAGGCTCCGACGCCCCTCCCGCCGCCGACGCCT
T5-13	TaFBA1		TraesCS7D02G179800	7D-2023910-T5-13	reverse	133,226,827	CCCTGACCCTTCCAGGACTGATCATGCAAGTTGTATGATCCACCTGTTTCA	13	62.1	4B-1062650-T5-1_256	ACTCCTACTCAGGCTCCGACGCCCCTCCCGCCGCCGACGCCTGCACTGAGGGCTACAGCCGCTCCGGTGATTGCGAGGGCAAGACCAATGAGAAAATGGTGAAT
T5-14	TaOPR2	ABD	TraesCS7A02G412400	7A-1801960-T5-14	forward	603,291,733	GGCCATTGCAGCCCAGCTCGCGGATGCGGAAGCTCCAAGCATGCGCGTC	5	32	4B-1062650-T5-1_320	CGGTGATTGCGAGGGCAAGACCAATGAGAAAATGGTGAATTCCTCGGCCTTCCATGTTTCTTTTAGCATATCTAGTCATTTTAGTATAAATCTATATCTGTTTGCTGG
T5-14	TaOPR2		TraesCS7B02G311600	7B-1860490-T5-14	forward	557,057,439	CATATAATCAACTCACGGATACTCGTGGTGACATTGGAGCATATAGGTGAC	4	24.9	4B-1062650-T5-1_384	AGCATATCTAGTCATTTTAGTATAAATCTATATCTGTTTGCTGGCTGAACTATGATGGTTAAACTGTGTATTTTAGTCTAGATTAACTGTGATGTTTTGCCCGCTTGTT
T5-14	TaOPR2		TraesCS7D02G405500	7D-2008410-T5-14	forward	523,047,722	GGATTTGTTTAAGCAGCGCTGCGTGCGCATTGCTATGATTAATATTCCTGC	19	87.3	4B-1062650-T5-1_448	TGTGTATTTTAGTCTAGATTAACTGTGATGTTTTGCCCGCTTGTTCTATGTTTCCGAAATCTCGTTGATGATTTGCATAGTTTGTTTACATGGCACGGATAGTATAG
T5-15	SAPK9	ABD	TraesCS4A02G235600	4A-0996560-T5-15	forward	544,198,527	CAAAACTGTGAGCTGAGTTGGGAGTAGTGCCATATGCATGAGGAAAGCA	16	73.4	4B-1062650-T5-1_512	GTTGATGATTTGCATAGTTTGTTTACATGGCACGGATAGTATAGATTTAGGGGGCGAATATGAGGTACACGACAGTGGGCGCGAGCGTTGACCATTGGCCGGTC
T5-15	SAPK9		TraesCS4B02G079300	4B-1083460-T5-15	reverse	76,819,160	CCCTCTTCCCTCCCCTCTCCCCTTTCCTTCCCCCACCGCCGGCGCCGCGGA	15	72.9	4B-1062650-T5-1_576	GGTACACGACAGTGGGCGCGAGCGTTGACCATTGGCCGGTCATTGTCCAGGCGCATCCGTGAATAGGAGAAGTTATGTATTTTTTTTTATGATAATACGTGTCT
T5-15	SAPK9		TraesCS4D02G078100	4D-1164930-T5-15	reverse	52,472,989	CCCCTCCCCTTCCCTGCCCTTCCCTTCCCCCACCGCCGGCGCCGCGGAGC	6	35.2	4B-1062650-T5-1_1216	GTGAAAGGACACAAGTCCACCACATGGTAGTTCCCAAATGCATTACCGATCATAGAGCTGATCGTCTTGTCGGTCGGAGAAGGAATTGAAGCTTCTTTGTTCAGC
T5-16	OsGRAS23	AABBDD	TraesCS5A02G481400	5A-1230270-T5-16	reverse	653,853,145	GGCCGGAGGCTGGTGGCGGGCTAGCTGGTCGCGAACTGCGGAGCGTAG	15	76.2	4B-1062650-T5-1_1280	TCTTGTCGGTCGGAGAAGGAATTGAAGCTTCTTTGTTCAGCGCGTCGACACCGAGATATGGAACGTCCCAAAACCTAGCCCACTAGATCCCTGAAACCTCTGCA
T5-16B	OsGRAS23		TraesCS5A02G6481300	5A-1230280-T5-16B	reverse	653,847,505	GGCTGCGGAGGCAAGACGCCGTCGCCGGCAGGAGATGGAGGTACAAG	10	60.7	4B-1062650-T5-1_1344	CGTCCCAAAACCTAGCCCACTAGATCCCTGAAACCTCTGCAGAAGAAACAAAAATGTTTTACCACCGAGCCGTGAGTGATCGTGAGTGCTCGGAGACCGTAGCA
T5-16A	OsGRAS23		TraesCS5B02G494600	5B-1361690-T5-16A	reverse	662,459,153	GGCCGGAGGGTGGTGGCGCGGTACTGCAGGCTAGCTGGCCGCGAACTG	15	76.2	4D-1144620-T5-1_192	TGGCACAGTGTTGTGACCCGGCGTACACGGAGGACTCCCCGAACTAGGACGTGTGGTTCACCACCGAGCACGATGCGCACCGCCACGGCGCCATCCAGATCGA
T5-16B	OsGRAS23		TraesCS5B02G494500	5B-1361700-T5-16B	forward	662,454,135	AAGTCATCATACGGTCACCGGAAATATTCGGGGGTATACCGGTATTGTATC	6	32.5	4D-1144620-T5-1_448	CGAGCTGTCAGTAGGGGGGCTGTGTCTACCCTCCACCACCACCGCCTCTCCTGGAACTACCCGAGCTCGAGCCTACGTTGGCACCACCCGCCACCCCGAGAGG
T5-16	OsGRAS23		TraesCS5D02G495000	5D-1434470-T5-16	reverse	526,692,302	GGCCGGAGGGTGGTGGCGCAGTACTGCAGGGTAGCTGGTCGCGAACTG	8	53.2	4D-1144620-T5-1_512	GCTCGAGCCTACGTTGGCACCACCCGCCACCCCCGAGAGGTACCAGTGGGAGGCGTGGTGAGGGAGTATGTGAGCGCGTCGGCCGCGTTGATGGGCGCGAC
T5-16B	OsGRAS23		TraesCS5D02G494900	5D-1434480-T5-16B	reverse	526,687,396	CCCATTCCTTGGGCTCGCCGGCGCTATTCGGCGTCAAGGCCGAGCCCGCC	7	36.8	4D-1144620-T5-1_704	GCGGGAGGAGGAGGAGCGCTGACAGCGGTAGGCACGAGAGAAGGGTCGGTTTGCGGAAAAAAAAAGCATGTTGTGTGTCCGTATAGGCTTTCAATCTTCTTAG
T5-17	Erd15	B,D	TraesCS3B02G554200	3B-0782020-T5-17	forward	788,140,093	GTCCCTGGCTCGGCTTGGATTGCCTGGAATATTCTGTCACAAAAGGCCCAT	19	83.5	4D-1144620-T5-1_768	AGCATGTTGTGTGTCCGTATAGGCTTTCAATCTTCTTAGTAATATTTTTTTGAAACAAATAAGAGTAGGTCCGATCTCCGTGGCTAAAATTACACTCTCGTGTCTCT
T5-17	Erd15	no A	TraesCS2D02G304800	2D-0520400-T5-17	reverse	390,594,737	CGTGATCTGGAGGTTTAGTAGGGAATTTTACAACCAGATGGACTGGAAAC	17	80.5	4D-1144620-T5-1_832	AGTAGGTCCGATCTCCGTGGCTAAAATTACACTCTCGTGTCTCTGCCGTATACAGTACATACGGTATCACTGTCTGCATGTCCTTGTTTCTGGAAGAGCAAGGAACA
T5-18	OsDREB2A	D no AB	TraesCS3D02G099500	3D-0899080-T5-18	reverse	51,103,277	GGAGCGAGAAGAGGGACGAGAGAGATCTCCTCTCGCCTCGGTCTCCCCG	11	60.5	4D-1144620-T5-1_896	TATCACTGTCTGCATGTCCTTGTTTCTGGAAGAGCAAGGAACATCTTTCTGGAAGGAAAATGAAGGCCATGGTGTGCTCACATTTTCCAATGGTACTATAGCATAAT
T5-19	OsLEA3	ABD	TraesCS2A02G432400	2A-0270740-T5-19	forward	685,410,655	TATATATATATTCAAAACAATATTTTTCCATAACCCATTTTTAGTTTAGTTTTTC	24	95.8	4D-1144620-T5-1_1088	CGCCATCGTATCGTTAAATCGACACCACTATTACCGGCCCTCGATGTTGTATTGCCATACAGTACAAAATTGGAAATGTACCATTTATATAAGACAAACAACAGTATAT
T5-19	OsLEA3		TraesCS2B02G453700	2B-0402570-T5-19	forward	647,539,053	GTCATTGTAGTATGTTTTCTTGGATATCCTATTTTCCAATGATACAATCTTGA	16	76.7	4D-1144620-T5-1_1152	CAAAATTGGAAATGTACCATTTATATAAGACAAACAACAGTATATACGTACGTACAATCTACACTAGGCGGCCTTGCAATCCCAGGAGGAAAAAACAAATCCGTGA
T5-19	OsLEA3		TraesCS2D02G430300	2D-0518390-T5-19	forward	542,710,413	ACACGGACTACGGTGTAGGTCTCGGCGACTTGTCCCTCGTATCAACTGATG	3	9.1	4D-1144620-T5-1_1536	GCAATGGCTGCTCTTGCTCACCAAATCAAGCGCTTCCTCCGCCGCTGCCCAACAGCTCGCACCAAGCTCCCATTCTCATAGCAAATCGTGCAGTTCATCCTAAATTA
T5-2	DREB1	ABD	TraesCS3A02G099200	3A-0701360-T5-2	reverse	64,029,824	GGAGCGAGAAGAGGGACGAGAGAGATCTCCTCTCGCTTCGGCTGCTCTG	17	72.7	4D-1144620-T5-1_1580	CTGCCCAACAGCTCGCACCAAGCTCCCATTCTCATAGCAAATCGTGCAGTTCATCCTAAATTACCAGCTCTCCTCGGCTTCTCTTCTCCAAATCGTCGGCGTTTCTT
T5-2	DREB1		TraesCS3B02G115400	3B-0762610-T5-2	reverse	82,054,648	CGAGCGAGAAGAGGGACGAGAGAGATCTCCTCTCGCTTCGGCTGCTCTG	12	57.6	3A-0701360-T5-2_384	CTCTCCTCCCGTGCCAGTATGCCACCACGGCCAGTAAAGGCAAGTCGTAACTTGCAGGCAGCCTTCCAAACGAACTCGATTCCCTCTGGATTCGCTGATATCGTATC
T5-2	DREB1		TraesCS3D02G099500	3D-0899080-T5-2	reverse	51,103,277	GGAGCGAGAAGAGGGACGAGAGAGATCTCCTCTCGCCTCGGTCTCCCCG	11	60	3A-0701360-T5-2_448	TCCAAACGAACTCGATTCCCTCTGGATTCGCTGATATCGTATCACCGTCGGGCGCCGATTTCAAATTTCGTTTCCGCCGTTTCCCCTTTTGATGGAAAGAGGGAGCG
T5-20	TaASR1	ABD	TraesCS4A02G208400	4A-0972660-T5-20	forward	501,468,566	TGAACGTGTCCAGATCGCGGAGGTGTCGTACCTTCGGTGCTAGGATCGGT	8	38.8	3A-0701360-T5-2_512	ATTTCGTTTCCGCCGTTCCCCTTTTGATGGAAAGAGGGAGCGGGAGAGGCTGAATCTCAACATTTTTCGTGTGATGTTGCCACTAAAGAGATGGATGTAGGGTTT
T5-20	TaASR1		TraesCS4B02G112000	4B-1084630-T5-20	forward	125,481,485	CTCTCCCACTTGAAATATCACCGTCGCATGCAGAACTAGGCGATCACACGC	15	78.4	3A-0701360-T5-2_576	TTTCGTGTGATGTTGCCACTAAAGAGATGGATGTAGGGTTTGTTTGGAATGCTGTCTCGAGCTGCCCTGCCAATTTTCGGCATCGGTGTGCACGTGGACCTCCGCC
T5-20	TaASR1		TraesCS4D02G109500	4D-1166720-T5-20	forward	88,700,622	GATAATAATAAGAAGATCTGAAAGATGAAGATACCTCTTAAGCATGAAATC	8	39.9	3A-0701360-T5-2_640	CCCTGCCAATTTTCGGCATCGGTGTGCACGTGGACCTCCGCCAACTTTCCGTAGTCGGTTCTGTTTTTACACCACACGTGTTGGCGAAACCGAGGATGTAAATTCC
T5-21	TabZIP28	ABD	TraesCS2A02G142800	2A-0356620-T5-21	reverse	88,013,066	GCGCGGCCGCGTGGGGGCGGTGGGGCCTAAGTCAGACGCCAGACGCGT	18	76.5	3A-0701360-T5-2_704	TTTTACACCACACGTGTTGGCGAAACCGAGGATGTAAATTCCCTCGTCAAATTATTGGCGCCCCTGAGTTTGGTGGGGTTGACGTGGGCGTCAATCCAAACAGAC
T5-21	TabZIP28		TraesCS2B02G167900	2B-0464450-T5-21	reverse	140,847,135	GGCGCGAGATCCGGTCACGGCGAGCGCATGCGCGACGGCGGGGCGGG	14	59.3	3A-0701360-T5-2_768	TGAGTTTGGTGGGGTTGACGTGGGCGTCAATCCAAACAGACACGTATGACCTTCCCGTCGTGAGGAGGCGGGCGGGAAAAGATGACGTTGGAGATGGACAAG
T5-21	TabZIP28		TraesCS2D02G146100	2D-0578420-T5-21	reverse	87,757,102	GGCGCGAGATCCGGTCACGGCGAGCGCATGCGCGACGGCGGGGCGGC	15	66.4	3A-0701360-T5-2_960	TTGAGATCTCATGAATATAGACATGAGAGTATGTGCCTCGCTCTTGTGAAGATTTGAGCTTCCCGGTAGACATAGGGCCTACTTGGTACATTGTCATTATTAGGGG
T5-22	HSP16.9	BD	TraesCS3B02G049800	3B-0739870-T5-22	reverse	25,122,173	TGTCGGTGTTGCTTGCTTTGGCTTGTAGCTTGGGGGGAAATTATGTATTTG	20	93.9	3A-0701360-T5-2_1024	GGTAGACATAGGGCCTACTTGGTACATTGTCATTATTAGGGGGGTTCTTCGATGCTTGGTCATTTAACCTGCAAGAGGATTTTTCTTTCTATGAAAAAAGACTAA
T5-22	HSP16.9	no A	TraesCS3D02G045700	3D-0901650-T5-22	reverse	17,413,909	CGTCGGTGTTGATTGCTTCGGCTTGTGCGCTCGGAAAATTCTGTAGATCGT	14	69.2	3A-0701360-T5-2_1088	TTAACCTGCAAGAGGATTTTTCTTTTCTATGAAAAAAGACTAACTGGCCCAATTCTCGGTAGGTACTCCCTTTTATAGTGATATGAACGCTCATATATTACTTTACATA
T5-23	TaGAAP	ABD	TraesCS5A02G384100	5A-1228210-T5-23	forward	581,926,267	AGGAAAAATAGGCCGAGATAGACACTTGAGGGCATTGGCAAAAAAAAAA	17	77.2	3A-0701360-T5-2_1216	GGGCCAACCACCGAATGAGCAACGATCTCTCGATCCCACTTGATTCCTTTCCTGGGGGATGTTTCCTATTTAAGTTCCTCGTCTCCTAGAAAGAGCCGCTAAATGAT
T5-23	TaGAAP		TraesCS5A02G384100	5B-1304490-T5-23	forward	567,873,934	TGAAATTGTGTTTCATATTTGTGTATCTTTGTCATTTTGTAAAAATAATGTTA	11	49.3	3A-0701360-T5-2_1280	CCTATTTAAGTTCCTCGTCTCCTAGAAAGAGCCGCTAAATGATCGAACCAGACAAGTGCACGTAGCTGATTGCATAGATATGGTATGTTCGGACGATCTATCTTGGCA
T5-23	TaGAAP		TraesCS5D02G393600	5D-1407710-T5-23	forward	461,912,557	AACCTGACTTAGCACAAAAAGCACAGCGTCACACGATGTTTACCGAAGAT	18	80.9	3A-0701360-T5-2_1344	GCTCGATTGCATAGATATAGTGTTCGGACGATCTATCTTGGCATGGCCAGTACCATGGTCAAAGTGGGCTTGTGACGAAATTAGGTCTCTCCACACCAATTTTTCGA
T5-24	TaRCA-L	ABD	TraesCS4A02G177600	4A-0980920-T5-24-1	forward	449,270,763	TGCAGTCAGCCCCAACCAGATTCATGTACGTTGGCTCATTTGGTTCAATAG	20	87.3	3A-0701360-T5-2_1408	TGGGCTTGTGACGAAATTAGGTCTCTCCACACCAATTTTTCGACATGGACTGGGAGCATGGGGCATGAAAGTTACAAACCTTGACATGCATCATATTGCATAGCAT
T5-24	TaRCA-L		TraesCS4B02G140200	4B-1077780-T5-24	forward	184,603,376	GAATTTTCTTTATCTTAAAAGCAGATTATGATTCAAAAAAATTCGTTGTCCT	35	76.1	3A-0701360-T5-2_1472	ATGAAAGTTACAAACCTTGACATGCATCATATTGCATAGCATATATAATATCTTACATAGTTCCAAGTGAACCATAATTGAGATGCACTGGATTGTAGCAACTTAGTT
T5-24	TaRCA-L		TraesCS4D02G134900	4D-1171230-T5-24	forward	121,811,802	GTGAGCAGATGTGATCTGTTCGTGGCGGTTAGGGTTAGGAGACACCGCA	11	53.4	3A-0701360-T5-2_1536	AAGTGAACCATAATTGAGATGCACTGGATTGTAGCAACTTAGTTGCACACTTAAACCTTAACATGCATATATGTATAGGATTTGAGTACAATTACATGTACGGCGA
T5-25	TaRof1	ABD	TraesCS2A02G277100	2A-0279760-T5-25	forward	458,051,107	CCTACTTCGTGAAGATCTACCCCGAGTGAGGCTAGTCCTTCATGGACGTA	8	43.3	3A-0701360-T5-2_1580	TGCACACTTAAACCTTAACATGCATATATGTATAGGATTTGAGTACAATTACATGTACGGCGAGGCACGAGCAACCATAAACACAGTGAAGAAGTTGCATCGACCA
T5-25	TaRof1		TraesCS2B02G294500	2B-0421050-T5-25	reverse	411,143,398	TGTTGATTAGGTTCGTGGACGCGAAAGGCTCTCTAGGTGGGATGGAAAG	16	78.8	3B-0762610-T5-2_633	CCACGGCCAGTAAAGGCAAGTCGTAACCTGCTTGCAGGCAGCCTTCCAAACGGACGGACACGCCGACTCGATCCCCCTGGATTCGCTGCGATATAGGAGTATC
T5-25	TaRof1		TraesCS2D02G276000	2D-0544770-T5-25	reverse	345,626,820	TGTTGGTTCGGTTCGTGGACGCGAAAGGCTCTCTAGGTGGAATGGAAAG	5	31.8	3B-0762610-T5-2_697	GACTCGATCCCCCTGGATTCGCTGCGATATAGGAGTATCACCGTCGGGCGCCGATTTCAAATTTCGTTTCCGCCGTTTTCCTTTTGATGAAAAGAGGGAGCGGG
T5-26	TaHSP16.8	D no AB	TraesCS3D02G046300	3D-0926370-T5-26	forward	17,556,213	TTTTTTTTTGAAAAGGAGGAAGACCCCCGGCCTCTGCATCTGGGTGATG	9	90.7	3B-0762610-T5-2_825	GTGTGATGTTGCCACTATGTAGGGTTTGTTTGGAATGCTGTCTGGAGCTGCCCTATCAATTTTCGGCATCGGTGCACGTGGACCTCCGCCGACTTTTCAGTAGTTG
T5-27	TaGT61-10	ABD	TraesCS7A02G235700	7A-1837250-T5-27	reverse	207,902,233	GGAAGATCACCCAGCCCAAAACAACACACGGTCACGACCGACGACCGAT	18	83.1	3B-0762610-T5-2_889	GGCATCGGTGCACGTGGACCTCCGCCGACTTTTCAGTAGTTGGTTCAGCTTTTACGCCACGTGTGTCGGTGAAACCGATGATGTAAATTCCTTCGTCAAATTATTG
T5-27	TaGT61-10		TraesCS7B02G133800	7B-1936690-T5-27	forward	162,778,805	ATTTGATTCTGGGAAGACATCTGGCTAGGCAATGCCAGTCTCAGAGAACA	5	32	3B-0762610-T5-2_953	GTCGGTGAAACCGATGATGTAAATTCCTTCGTCAAATTATTGGCGCGCCCGAGTTTGTGGGGTTGGCGTGGGCGCCGATCCAAATAGACACGTATCTTTCTTCCCG
T5-27	TaGT61-10		TraesCS7D02G235700	7D-2048000-T5-27	reverse	198,623,735	GGAAGATCACCCAACCCAAAACAACACACGGCCACACTCACGACCGATT	18	83.1	3B-0762610-T5-2_1017	GGCGTGGGCGCCGATCCAAATAGACACGTATCTTTCTTCCCGCCGAGGGAAGGCGGGCGGGAAAAGGTGACGTTGAAAATGGACAAGAGCATCGCTGGCAGT
T5-28	TaGT61-21	ABD	TraesCS3A02G021800	3A-0673300-T5-28	forward	12,438,692	TCTACTACGCTTTTTATAAAGCGGAATTTGGAACTACGTATGATGGATTTG	16	80	3B-0762610-T5-2_1145	CGGGGTTGCCGTTTGAGACCCCACGGTAGACGTCACACGTGGCTCTTGTTCGAGATCTCGTGATATAGACACGAGAGTATGTGCCTCGCTCTTGTGAGGATGTGA
T5-28	TaGT61-21		TraesCS3B02G024000	3B-0714960-T5-28	reverse	10,251,406	GCCGCCGTGGAGAACAACCTCTCGGAGCTCCTCTAGCTAGCTCGCCGGC	7	36.2	3B-0762610-T5-2_1209	ATAGACACGAGAGTATGTGCCTCGCTCTTGTGAGGATGTGAGCTCCCCGATAGATGTAGGGCCTACTCGGTACGTTGTCATTATCTAGATGATGTTTGATCATTAAA
T5-28	TaGT61-21		TraesCS3D02G025000	3D-0928520-T5-28	reverse	8,437,069	CATGGCTTTCTTATGAATAGGGTACACTAGAGTAAATTGCACAAAACCACC	8	49.9	3B-0762610-T5-2_1273	ACTCGGTACGTTGTCATTATCTAGATGATGTTTGATCATTAAACCTGGAAAGGGTATTTTTTTCTATGAAAATACTAACTGGCCCAATTCTGATAGGTACTCCCTTT
T5-29	TaBAHD2	ABD	TraesCS3A02G119500	3A-0686400-T5-29	reverse	93,042,873	GGGTGGGTCGGGGGCAGGAATGCTGCGATGGGGATCGGGGCTGGCGA	12	64.9	3B-0762610-T5-2_1337	CTATGAAAATACTAACTGGCCCAATTCTTGATAGGTACTCCCTTTTATAGTGATCTAAACGCTCTATATTACTTTGCAGAGGGATTACTTATTAGGGGGGGAGGGCA
T5-29	TaBAHD2		TraesCS3B02G139100	3B-0815030-T5-29	forward	125,778,975	TGTGTCAAGCGATTTGCAGTCAAAATTGTGCTAGTGTTTGGACTGGAGTA	4	24.9	3B-0762610-T5-2_1529	AAGAGCCACTGAACAATCGAACCAGACAAGTGCATGTAGCTCGACTGCATGGGTATAGTGTTCGGACGATCTATCTTGGCATGGCTGGTACCATGGTCAAAGTGG
T5-29	TaBAHD2		TraesCS3D02G121700	3D-0929780-T5-29	forward	79,428,067	ACCTAGACGGGAAGTAGCTCACAAGCTTAGTGCTTGTCGCGAGTTGATCT	12	64.9	3B-0762610-T5-2_1580	GGTATAGTGTTCGGACGATCTATCTTGGCATGGCTGGTACCATGGTCAAAGTGGGCTTGTGAAAATCAGGTCTCTCCACACCATTTTTTCAGCATGGACCGGGAGC
T5-3	DREB3	ABD	TraesCS1A02G221900	1A-0041900-T5-3-1	reverse	391,260,120	GGCCGGCCCGATCCGGATCGGGTCCAGATATCTAGCGTTCGCAATCTTGT	11	50.8	3D-0899080-T5-2_448	GCAGGCAGCCTTCCAAACGGACAGACACGCCGACTCGATTCCCTCTGGATTCGCTGAGATCGTATCACCGTCGAGCGCCGATTTCAAATTTCGTTTCCGCCTTTCC
T5-3	DREB3		TraesCS1B02G235100	1B-0119490-T5-3	reverse	421,100,357	GGCCGACCCGACCCGGATCGGGTCGAGATATCTAGCGTTCGCAATCTTGT	11	61.2	3D-0899080-T5-2_512	TCACCGTCGAGCGCCGATTTCAAATTTCGTTTCCGCCTTTCCAAAGAGGGAGCGGGAGAGGCCGAACTCAACAATTTTCGTGTGAGTGTTGCCGATTTCAATTTT
T5-3	DREB3		TraesCS1D02G223600	1D-0220160-T5-3-2	reverse	311,369,433	GGCCGACCCGATCCGGATCGGGTCCAGATATCTAGCGTTCGCAATCTTGT	7	36.2	3D-0899080-T5-2_576	AACTCAACAATTTTCGTGTGAGTGTTGCCGATTTCAATTTCGGTCTCCGCCTTGCCCAAATAGGTGTAGGGTTCGTTTAGAATGCTGCCTCGAGCTGCCCTGCCAATT
T5-30	TaBAHD4	ABD	TraesCS1A02G119100	1A-0080120-T5-30	reverse	129,004,387	GAACTGCGCCGCCCCGGGCCCGTCGGCCACCGCGTGGCTGAACCGGAAC	3	15.1	3D-0899080-T5-2_640	GTAGGGTTCGTTTAGAATGCTGCCTCGAGCTGCCCTGCCAATTTTTGCATCGGTGCACATGGACCTCCGCCGGCTTTTCGGTAGTCGGTTCAGTTTTTACGCCAC
T5-30	TaBAHD4		TraesCS1B02G138600	1B-0152360-T5-30	reverse	179,631,173	TTGGAACGGGGGAGGGCCGGAGGAGGACACACCTTCGGGTTGGTTGA	5	25.4	3D-0899080-T5-2_704	CCTCCGCCGGCTTTTCGGTAGTCGGTTCAGTTTTTACGCCACGCGTGCTGGCGAAACCGATGATGTAAATTCCTCGTCAAACTTTTGGCGCCCCGAGTTTGAGA
T5-30	TaBAHD4		TraesCS1D02G120000	1D-0249640-T5-30	reverse	117,400,835	TTGGAACGGGGGAGGGCCGGAGGAGGACACACCTTCGGGTTGGTTGA	5	25.4	3D-0899080-T5-2_768	GTAAATTTCCTCGTCAAACTTTTGGCGCCCCGAGTTTGAGAGGGGTGGCGTGGGCGTCAATCCAAACAGACACGTATGACCTTCCCGCCGTGAGGAGACGGGG
T5-5	TaER-D1/TaER-B1	ABD	TraesCS7A02G164000	7A-1838690-T5-5	reverse	120,583,429	AGCAGCATTAGCAGAAGAAAATGGCCGCAAAAACAGATTAAATGAAGAT	9	50.6	3D-0899080-T5-2_1024	GCCTCACTCTTGTGACGATTTGAGCTCCCCGATAGACGTAAGGCTTGCTCGGTATGTTGTCATTGTTTGAAAGGGTTATTCGATGCTTTATCATAAAACCTGAAAGG
T5-5	TaER-D1/TaER-B1		TraesCS7B02G069800	7B-1957790-T5-5	reverse	75,159,436	GGCCGGTGGTACGAAGCTGCGAAGAATCACCGTCCGGGCAGTGGCATTT	4	21.6	3D-0899080-T5-2_1088	GTTTGAAAGGGTTATTCGATGCTTTATCATAAAACCTGAAAGGGTTTTTTTTCTTACTTTACGGAGGGAGTACTAGTAGGGGGAGGGTCAAACACCGAATGAA
T5-5	TaER-D1/TaER-B1		TraesCS7D02G166100	7D-2056570-T5-5	reverse	116,477,479	GGCCGGTGGTACGAAGCTGCGAAGAATCACCGTCCGGGCAGTGGCATTT	19	76.9	3D-0899080-T5-2_1152	GAGGGAGTACTAGTAGGGGGAGGGTCAAACACCGAATGAACAATGATCTCTCGGCCCCAATTGATTCCTTTCCCAGGGGATGTTTATTTACATTCTGTCTCTTAG
T5-6	TaGLDH-A1/B1/D1	ABD	TraesCS5A02G132300	5A-1256040-T5-6	reverse	298,809,245	CGTGGGGAGCGGGAGTGGGTGCGCGGCGGCGGTCGAGTCGCGGCCGG	??		3D-0899080-T5-2_1280	CCAGACAAGTGCAGGTAGCTCGACTGCATAGGTATATTGTTCGGACGATATATATTGGCATGGTTGGTACCATGGTCAAAGTGGGTTTGGGACGAAATTAAGTCTC
T5-6	TaGLDH-A1/B1/D1		TraesCS5B02G132600	5B-1354790-T5-6	forward	247,251,434	GCATTGTTCAGACAAGATAAACAGAGTCATTTATGAAGGTTCAGTCAAGC	3	14.6	3D-0899080-T5-2_1344	TGGTACCATGGTCAAAGTGGGTTTGGGACGAAATTAAGTCTCTCCACACCAATTTTTCAGCATGGACGGGGAGGCATGGGCACACGTTTCAACCTTCGCCAATTT
T5-6	TaGLDH-A1/B1/D1		TraesCS5D02G140400	5D-1423370-T5-6	reverse	223,961,680	CGTGGGGAGCGGGAGTGGGAGCGCGGCGGCGGTCGAATCGCGGCCGG	4	17.2	3D-0899080-T5-2_1408	GACGGGGAGGCATGGGCACACGTTTCAACCTTCGCCAATTTCTCGATCCTCGATTCAATTTCTATGCATTGCGTATTGGCGAAACCAAGGATCTGAATTCCTCCTTC
T5-7	DRFL1b	BD	TraesCS5B02G193200	5B-1316100-T5-7	reverse	348,571,719	CTAAGGAACTAGGACGAACTTTTTTTTGGGGATTTTTGTTTTCTTAGAAAC	5	32	3D-0899080-T5-2_1472	TGCATTGCGTATTGGCGAAACCAAGGATCTGAATTCCTCCTTCAAATTATCAGCACATCATTTTTTTGCGGGGTTGTTGTCGATGTCATCCAAACAGACTTGTATCC
T5-7	DRFL1b	no A	TraesCS5D02G200900	5D-1418260-T5-7	reverse	304,163,300	CTGGGCCGGGCTGAGCTCGGTCTGGGCCTGTGCCTGCACCCCGTACGGG	9	39.3	3D-0899080-T5-2_1536	TTTTGCGGGGTTGTTGTCGATGTCATCCAAACAGACTTGTATCCTCTTCTTGTCGTGAGGAGACGGGCGAGAAAAGGTGACATTGGGAATGGAAAACAACATCG
T5-8	OsCML4	ABD	TraesCS5A02G382800	5A-1228470-T5-8	reverse	580,799,964	TCAGATAAGTTTATCGTCGTGTCATTTAGTGTTTAGTGTGCTCCTTCCAGA	18	84.2	3D-0899080-T5-2_1580	TCTTCTTGTCGTGAGGAGACGGGCGAGAAAAGGTGACATTGGGAATGGAAAACAACATCGGCAGCGGTTGTCTTGTTGCAAGAGATATGCACCGAGGCTCCC
T5-8	OsCML4		TraesCS5B02G387100	5B-1308890-T5-8	forward	566,497,118	GATATTTATATTCCAGTAGCAAACGTAAAATGCGGTGGAGTAGGGGCAT	12	58.9	1A-0041900-T5-3-1_0	GGCCGGCCCGATCCGGATCGGGTCCAGATATCTAGCGTTCGCAATCTTGTCGCCTGGTTTCTTCGCCGCGTCGACTTGGATAGAGAGTTGGGCAGAGGCGTGGA
T5-8	OsCML4		TraesCS5D02G387100	5D-1441690-T5-8	reverse	460,648,061	CCCTGATCAACAAGTCCTCCAAAGAGTGAACAGAAAAAATAGCAAAGGA	18	86.8	1A-0041900-T5-3-2_128	GTGATCTCCGAGGTCGCTTTTTCCTGTAGAACAAAGAGCAGAGCAGCAGTTAGTACCTTATATTCTCTTCAGATTTTTTTTCACCCAATATAAGCACAAGGTACAATC
T5-9	TaGT2L1D	D no AB	TraesCS2D02G387100	2D-0539740-T5-9	reverse	492,512,011	GGATGATGACACCACTGCACCACCGCCTTGAACGCGCGCTCTCAAATTGT	13	67.5	1A-0041900-T5-3-2_192	CTCTTCAGATTTTTTTCACCCAATATAAGCACAAGGTACAATCAATGAAAGAAACGCAAAACAATTGAGGCACCCAAACAGACCAGCTCCACATAAACATTTTGTTCA
										1A-0041900-T5-3-2_256	GAGGCACCCAAACAGACCAGCTCCACATAAACATTTTGTTCAATGACTAAACTATAAATAAATGGCACAAACAGACCATGCCCACAGGAACAGATGTACAGCAAC
										1A-0041900-T5-3-1_320	gtgcggggAAACTTCCCGAGACTTGGTGCCGAAGGAAACTCGGCTTCTCCTTCACGCTTTCGAGTGCTACCAGTGCTTTTCTTTCGGGGTGGATTGGACGTGCTGC
										1A-0041900-T5-3-2_320	GCACAAACAGACCATGCCCACAGGAACAGATGTACAGCAACAGGGACATATAAACTTCCTTGCGGAAGTACCATCTCCACAGAAAACAATACCATGATAAAATCGTA
										1A-0041900-T5-3-1_384	TGCTACCAGTGCTTTTCTTTCGGGGTGGATTGGACGTGCTGCTCTTGGGTGGGTTCCACGTGTCGGTCTACGTGTGGCCAGTGGCTGGGGGGCTGAGTCGATATA
										1A-0041900-T5-3-2_384	GAAGTACCATCTCCACAGAAAACAATACCATGATAAATCGTACTAATCGGCGCCACCCCTAAATGTGTTTAAGTAGCAAAATATGACAAAAAAGAACAGGATGAGA
										1A-0041900-T5-3-2_448	GTGTTTAAGTAGCAAAATATGACAAAAAAGAACAGGATGAGAACTGACTTCTATACTTGAAGACTGATTAATCATAAAGGACATTCAAAAAGGCAAAGTAGCCACTAG
										1A-0041900-T5-3-1_512	GATGACACGGTGGATCGTGAGACATCTTTTTTTGTTTTGTTGAGACGGTATGAGACGTCTTTGGTTGCATGGATCAGTGGTGGCCAGCGTGGTCTCCTTCTCCCGT
										1A-0041900-T5-3-2_512	TGATTAATCATAAAGGACATGGCAAAAATTAGCCACTACAAAACTGTTAACAACACCTAGGTCAGTTTAGGTGAGATGGCATCCAGATAGAGTGGTACATCAC
										1A-0041900-T5-3-1_576	TTGCATGGATCAGTGGTGGCCAGCGTGGTCTCCTTCTCCCGTCGTAATGGCAACTCCAACGCCCCGATGTATTTAATATGCCCGTGTTTGTTGGGTCAAAACGGAT
										1A-0041900-T5-3-2_576	AGTTTAGGTGAGATGGCATCCAGATAGAGTGGTACATCAACCAACAGCAAGACAATGGTTCAAAAACCAACTATTATAATGACAAATGCAAAACAGTCCAAATATT
										1A-0041900-T5-3-1_640	CGATGTATTTAATATGCCCGTGTTTGTTTGGGTCAAAACGGATGAAAACATTGGTCCAACGCTTCGATGCAAATTCAAATACCGTCCGATCGCTGACTGTCTAAACG
										1A-0041900-T5-3-2_640	AAACCAACTATTATAATGACAATGCAAAACAGTCCAAATATTGGCTGGACTCCCAGTACGGAACTGTACAACGTACAGTCAGTTTAAATGAGATCACATCCACAG
										1A-0041900-T5-3-1_704	CGATGCAAATTCAAATACCGTCCGATCGCTGACTGTCTAAACGGATTCTCGTCCGATCGGTCCGGTCCATATTTGCGTCCGATTGTGTCCACAGACGCCGTCCCATC
										1A-0041900-T5-3-2_704	CTGTACAACGTACAGTCAGTTTAAATGAGATCACATCCACAGAGAGTGATACATCAACCAACAGCAGGAAAATGGTTAAATAGCAGCCACTATGAAACCAACTGT
										1A-0041900-T5-3-2_768	GCAGGAAAATGGTTAAATAGCAGCCACTATGAAACCAACTGTTAGAATGATATGGCAAAACGGCCAAAAGGTTAGCTGGAACTCTTAGTCATAGCTCACATGCTC
										1A-0041900-T5-3-1_832	GACCCACCCACCTCGGGCCCACATGGCAGCCGCTGAAAGCGCTTGCAGTCCACGCTTATTTAAATGGCAAGCGTGCGGGAGGCAGCCTCACCCACTTCCCAC
										1A-0041900-T5-3-2_832	CAAAAGGTTAGCTGGAACTCTTAGTCATAGCTCACATGCTCTATAATACATAGAGCAGCCAGACATAGAACGGTTAAACACACCGCAGAATAGATGCCACTGGCAC
										1A-0041900-T5-3-1_896	ATGGCAAGCGTGCGGGAGGCAGCCTCACCCACTTCCCACTCCCGTCGCACATCTAGCTAAACTCCCGCTCCCTTGTCGGCGACAGGAAACCCTAGCCATGGTTG
										1A-0041900-T5-3-1_960	CCGCTCCCTTGTCGGCGACAGGAAACCCTAGCCATGGTTGGTCTTCTTTGGGAAGGGGAAGGGGAAGGGGAAGGACATGACGCCCAAGCCACCTCCTCACATGGG
										1A-0041900-T5-3-1_1024	AGGGGAAGCATGACGCCCAAGCCAACTCCTCACATGGGCTCACGAGGTGGGTGCTGGTGCCAGCGGCGGGGAACGCGTGTATATCTgatacgtccattttgcatcatgtt
										1A-0041900-T5-3-2_1024	TTTCTGCTGACTTCTCCTCAAACGCGAGGTACCATCTCAGTCATAGTTCATGCTTCTGTTTACAGGAGTGATCAAAGCAGCATACCTATTGGTTTGACGAGAACA
										[illegible]	[illegible]

Trait&gene no.	Gene	Homoeologues	IWGSC gene ID (refseq1.1)	WGIN Promotome ID	Orientation	ATG	Target Sequence	MYbaits	MYbaits coverage (%)	MYbaits ID	MYbaits sequence
T6-1 & T9-63	TaZAT8	AD	TraesCS5A02G477600	5A-1235830-T6-1	forward	651,635,549	TACGACTGTAAATGCACATTGACGATCGGTCGAGCGCGTGGTGCA	15	78.2	5A-1235830-T6-1_0	TACGACTGTAAATGCACATTGACGATCGG
T6-1 & T9-63	TaZAT8	no B	TraesCS5D02G491100	5D-1443700-T6-1	forward	525,250,314	TAGATTCATCATGCAGTTCATATGAAGAACTTGATGACAACTTTGA	12	61.6	5A-1235830-T6-1_64	TCCATTGCCGTGGCGCAGGGATTTAGATG
T6-10	Nitrate reductase	ABD	TraesCS6A02G017500	6A-1557980-T6-10	reverse	8,700,014	GCTGTGCTGTGTTTGTGTCGTGGGACAAGTGCTGGGTCGGTTCTG	9	44.1	5A-1235830-T6-1_128	GGAGAGCCAAAATCGGAAATGTGTAAAC
T6-10	Nitrate reductase		TraesCS6B02G024900	6B-1665930-T6-10	forward	15,128,399	CATTTATCGAATATGATCAAGAGTAGGAAGCTATGTTATACAACCTC	16	76.8	5A-1235830-T6-1_192	AGCCTGTCAGATCAACGTTCACGGGTGCA
T6-10	Nitrate reductase		TraesCS6D02G020700	6D-1744010-T6-10	reverse	8,155,798	GCTGTGTTTGTGTTTGTGTCGTGGGACAAGTGTTGGATCGGTTCT	12	58.5	5A-1235830-T6-1_256	TTTCTTTTTATTGCCCTCTGCGTAACACCG
T6-11	Nitrate reductase	A, D	TraesCS4A02G376700	4A-0966470-T6-11	forward	651366029	TTGACGACGTTAGCCGCTGGTCACGGGCGGGGTCGCCGGGTCCA	18	76.5	5A-1235830-T6-1_448	CGGGCGATCGCCCGCCGCCCGCCCAACT
T6-11	Nitrate reductase	no B	TraesCS7D02G073700	7D-2044470-T6-11	forward	43,210,073	TTCAATACCATGGGAAATTCATCTGTTTTGCTATCCCAATTCTGAAG	16	72.2	5A-1235830-T6-1_512	GCCCAACTGCCTTCAGTTGGCTTCCGTGC
T6-12	M8B3I4	BD	TraesCS2B02G528300	2B-0391860-T6-12	forward	722,630,407	AGTGGATGTCCTGGCTCAGACATCTCCCGCAGCAGCAGCACGGT	11	57.9	5A-1235830-T6-1_704	GAGGTCGGGTTACTGTAATGGCTTCCATG
T6-12	M8B3I4	no A	TraesCS2D02G500600	2D-0558280-T6-12	forward	595,161,982	TTTGTGCACATTGCTCTGCACTGCTGGTTGGAGGAGCAGGAACTT	15	71.8	5A-1235830-T6-1_896	tccgcctcccatacgctgctcctcctcttctctccgcct
T6-13	GS1	ABBD	TraesCS6A02G298100	6A-1499070-T6-13	reverse	531,398,090	ATGTACTCGGCGATGATCTTCTCCGTGGAGTCGGTGAGGTCGAGG	7	42.6	5A-1235830-T6-1_1088	cgccgtcccgattcggccgccgccCACTAACAAA
T6-13	GS1		TraesCS6B02G327500	6B-1608010-T6-13	reverse	577.186,677	GCCAGATCCGCCGATCCTGCAATAAATCCAAAATAAATACACGGTG	11	67.8	5A-1235830-T6-1_1152	TTTTTAATGATGTAATTTTTGATGTCTTCTG
T6-13	GS1		TraesCS6D02G383600LC	6D-1700820-T6-13	reverse	386,294,363	GGCCGGCTGACTGAGGGCGCGCAGAGTCGCGGAGGAAGGAAG	15	76.2	5A-1235830-T6-1_1280	TGGGGAGCAAGCTAACAATTGCTCTCAAC
T6-14	TaNFYA-B1	ABD	TraesCS6A02G335400	6A-1503230-T6-14	reverse	567,936,606	GGCTCCCACGCGCCTCCTTCTCCCTCCCGCCGCCCTCTCTCGGTGC	19	81.4	5A-1235830-T6-1_1472	CGCGCTTGGCTGCTACCACCTGCGAGTAC
T6-14	TaNFYA-B1		TraesCS6B02G366100	6B-1592660-T6-14	reverse	638,831,686	GGCTCCCACGCGCCTCCTCCCTCCCTCCCTCTCGCCGCCCTCTCTC	19	81.4	5A-1235830-T6-1_1536	TCCACGGAACACAACCACACATCCACGAG
T6-14	TaNFYA-B1		TraesCS6D02G315300	6D-1705140-T6-14	forward	423,749,688	ACTTTTTGTTGGTAGAATAGACCCTGGCTTGCCAGCGCGTGGCTC	18	84.2	5A-1235830-T6-1_1580	CTTCCGACTGACTGAATCCATCTCCCATCT
T6-15	TaNRT2.1	AB	TraesCS6A02G031100	6A-1548640-T6-15	forward	15,765,983	TGATATTGTGATAAATAGGAACCTAAGTCCAACATAAGTAACATCTG	19	81.4	5D-1443700-T6-1_384	AAATACTTTTACCGTTAAAACCTTATCATTT
T6-15	TaNRT2.1	no D	TraesCS6B02G044100	6B-1648900-T6-15	reverse	26,597,775	CTTTCTTGAGCTAGCCTAGTTGCACTCACAACTATGAGCAGCTAAC	12	68.2	5D-1443700-T6-1_704	TTTAAACCCACATATTTTCTCTTTTAAGAG
T6-16	TaNRT2.2	ABD	TraesCS6A02G031200	6A-1548650-T6-16	reverse	15,782,628	CCTCCTCGAGCTTGCTAGCCTAGCCGCACTGGCAACTAAGTGCGT	16	73.4	5D-1443700-T6-1_832	CGCCTTAGCCGTACCCAAAATCAAACGAT
T6-16	TaNRT2.2		TraesCS6B02G044000	6B-1648910-T6-16	forward	26,591,111	CCCCCCAAATTAATGAAAACGATGTGGATGTTATGTCGACTAGTGT	17	80.5	5D-1443700-T6-1_896	TCCCGTCATGGGTTGCCGTGTAAAAGAAC
T6-16	TaNRT2.2		TraesCS6D02G035600	6D-1727340-T6-16	forward	14,618,702	CATATATAGCTACCCCCCAAATTAATGAAAATGATGTGGATGTCATG	16	80	5D-1443700-T6-1_960	TCGAAAAAACAGACACGCGCATACGCGT
T6-17	TaNRT2.3	ABD	TraesCS6A02G031000	6A-1548630-T6-17	forward	15,756,654	GACTGTACATCTACTCTCTCATCAAAGTGAATTAGCCTCACTACCAT	12	61.6	5D-1443700-T6-1_1024	CCTGGTGCATGTACCTCCACAGAGAAATT
T6-17	TaNRT2.3		TraesCS6B02G044200	6B-1648890-T6-17	reverse	26,618,399	CTTTCCTGACAACTAAACGCAACTAATGGTGGTTGTTGCTGCTGCT	17	82.6	5D-1443700-T6-1_1088	CAAAGCCATTTTCGGACTAGAGAAGAGA
T6-17	TaNRT2.3		TraesCS6D02G035700	6D-1727350-T6-17	reverse	14,632,985	CTTTCCTTGACAACTAAAAGCAGCTAATGGTGGCTGCAGCTACTCT	13	65.8	5D-1443700-T6-1_1152	GTGAGTTCTTCTGACGCAAGACTCGTCGT
T6-18	TaNRT2.4	ABD	TraesCS6A02G030700	6A-1548600-T6-18	forward	15,727,844	GGCTAGCCATCCAATGTCTTGTGTGCAGCAACATCTTCTCAGAGTC	24	95.8	5D-1443700-T6-1_1216	ATTTCGGGGTCCCACAGCTCACCAACGCC
T6-18	TaNRT2.4		TraesCS6B02G044500	6B-1648870-T6-18	reverse	26,634,792	ATCTGCCTCTCCTGTCCTGACCGAGCTAGGCTTCTTCTCTGCTAGCT	16	70.1	5D-1443700-T6-1_1344	CACAGCCTTGCCTACCTGGGGAGCAAGC
T6-18	TaNRT2.4		TraesCS6D02G035900	6D-1727380-T6-18	reverse	14,680,775	ACCTACTGATCTAGCTTGATCGAGCTAGCTAGCCTTCTTCTTTGCTT	18	86.4	5D-1443700-T6-1_1408	AGAAGAGAAGGTTCGGCCTTTTCAAACA
T6-19	TaNar2.1	ABD	TraesCS6A02G210000	6A-1511800-T6-19	reverse	381,028,488	AGTCGCCAACTTGAGCACGGTGGCGCAGCCAGGGCGGAAGGTG	13	62.1	5D-1443700-T6-1_1580	TTCCGAGCTCTTCCGAGTCCCAGAAGCCA
T6-19	TaNar2.1		TraesCS6B02G238800	6B-1622420-T6-19	reverse	415,864,383	CGCCGCGATTCTAGCTTGGAACTTGGGCTCGAGCTCGCAGCTGCT	20	88.5	7A-1826920-T6-2_0	CTTTACACCCTTACAACTTGACTCCCCAAC
T6-19	TaNar2.1		TraesCS6D02G193200	6D-1685000-T6-19	reverse	267,515,418	CGCCGCGATTCTAGCTTGGAACTTGGGCTCGAACTCGCAGCTGCT	16	76.7	7A-1826920-T6-2_192	CTAGAACAAACCCTGTGCAACAAAGAATA
T6-2	TabHLH1	ABD	TraesCS7A02G157700	7A-1826920-T6-2	reverse	110,854,670	CTTTACACCCTTACAACTTGACTCCCCAACCTCTGCTCAGAAATATT	20	85.2	7A-1826920-T6-2_256	AAAAATGCATGCATGAACAAAATAATGTA
T6-2	TabHLH1		TraesCS7B02G062100	7B-1923970-T6-2	reverse	64,729,459	CTTTACACCCTTATAACTTGCCTCCCCAACCTCTGCTCAGAAATATT	8	40	7A-1826920-T6-2_320	AAGCTATATATTTTTTTGAAAGGTGTTATA
T6-2	TabHLH1		TraesCS7D02G158300	7D-2060360-T6-2	reverse	106,076,961	CTTTACACCCTTACAACTTGACTCCCCAACCTCTGCTCAGAAATATT	19	81.4	7A-1826920-T6-2_384	TGTTAACCACTTAAGTAAAATAGTATGTAC
T6-20	TaNar2.2	A, BD	TraesCS4A02G367300	4A-0994730-T6-20	forward	640232231	AGGAGCGACGGACGCTACCAGACCGAGCGTGAGAGTGTTCTTGA	5	24.7	7A-1826920-T6-2_448	ACCTATTGAAATGATTCTGCTTTTATCACA
T6-20	TaNar2.2		TraesCS5B02G719500LC	5B-1356990-T6-20	reverse	671,188,360	CGCCGTCGAACTTGGGCTCGATCGAACTCGGAGCTGCTGCTTGGT	9	53.6	7A-1826920-T6-2_512	ACAGTCAATTCAACAATTGGTTCTGTTAGA
T6-20	TaNar2.2		TraesCS5D02G506100	5D-1438330-T6-20	reverse	531,971,931	CGCCGTCGAACTTGGGCTCGATCGAACTCGCAGCTGCTGCTCGGT	15	75.1	7A-1826920-T6-2_576	TAGAACACGACAAGCAACGGATTATCTTC
T6-21	Nitrite reductase	ABD	TraesCS6A02G333900	6A-1504680-T6-21	forward	564,882,756	AAGTGATGGAAACGTTTCGTGGCCCCACGTGACACTTGGGTTATA	17	70.6	7A-1826920-T6-2_640	CATACACAATACAACCAGCTGGATATCTTC
T6-21	Nitrite reductase		TraesCS6B02G364600	6B-1596170-T6-21	forward	636,392,750	GATCGGCGACGCGAGGAGGAGATGCCCTGGGAGATCGACTACAC	17	70.6	7A-1826920-T6-2_704	CAACATACGCTTCCTCCAGTTGCTCACAA
T6-21	Nitrite reductase		TraesCS6D02G313100	6D-1679410-T6-21	forward	422,078,635	GGAGTTGGAAGCTGGAGAGGATCCGGACAGAGATAAAAAAAAC	21	85.6	7A-1826920-T6-2_768	TTGGCGGAGATATCTCCTTTATCTTTCACA
T6-22-1	Glutamate dehydrogenase	ABD	TraesCS2A02G389900	2A-0295460-T6-22-1	reverse	638,686,353	CAGCTGAGCAAGTGCATTTACTTCATCTGGATCAACCTGTCAAGCA	25	96.2	7A-1826920-T6-2_832	GAAATTGTACCATTGAACTGGCAATGAAA
T6-22-2	Glutamate dehydrogenase		TraesCS2A02G389900	2A-0295460-T6-22-2	reverse	638,688,747	GGCTGCGGCGGCGGCGCGGCGGGCTAACTTCTTCTTTTCTTCCCC	15	65.2	7A-1826920-T6-2_896	AGAAAATCAAAGGTGTGTATTAGAGATAG
T6-22	Glutamate dehydrogenase		TraesCS2B02G409300	2B-0393950-T6-22	forward	582,802,735	AACTAAGTGTAAGCAAAATGTTTATGTGCTTGGAAGAATGGACGT	13	57.6	7A-1826920-T6-2_960	AGGAGTAGCTTATAGATACAAGAAAAGTC
T6-22	Glutamate dehydrogenase		TraesCS2D02G388800	2D-0536210-T6-22	forward	495,095,682	AGATATCTATTTGATCTCGTGCATCTGGCTGGGGGTTGTTTGCTGT	13	56.8	7A-1826920-T6-2_1024	TGCCTAGTCTAGGCAGTTTTACCAGAAAA
T6-23	Glutamate synthase (NADI	AABBD	TraesCS3A02G266300	3A-0616020-T6-23	forward	490,929,704	CAAGTTGCCAGTGGACGATTTGGAGTTTCCAGCTATTATCTGACTA	23	89.4	7A-1826920-T6-2_1088	AATGTCCATCAAGTCGAGCTTTCACTGTC
T6-23	Glutamate synthase (NADH), plastidic		TraesCS3B02G299800	3B-0709800-T6-23	forward	481,603,610	ATCAAGCAAGTTGCCAGTGGACGATTTGGAGTTTCCAGCTATTAT	25	93.2	7A-1826920-T6-2_1152	TCCAGGAAAATTCCGTCACATAACTCATAA
T6-23A	Glutamate synthase (NADH), plastidic		TraesCS3D02G266400	3A-2150720A-T6-23	forward	490,924,742	TCGAGTTACCGCCACCAGGTGAGTATGCTGTTGGAATGGTCTTCCT	13	75.3	7A-1826920-T6-2_1216	CACTTTACACATTCGTGACTTGCGAGAAC
T6-23B	Glutamate synthase (NADH), plastidic		TraesCS3B02G299800	3B-2150720B-T6-23	forward	481,598,529	CACCAGGTGAGTATGCTGTTGGAATGGTCTTCCTGCCAACCGATG	14	72.5	7A-1826920-T6-2_1280	CCTTTTTTTCCAATCCATTCTGATCCCCCT
T6-23C	Glutamate synthase (NADH), plastidic		TraesCS3D02G266400	3D-2150720C-T6-23	forward	369,794,049	CGAGTTACCGCCACCAGGTGAGTATGCTGTTGGAATGGTCTTCCT	12	68.2	7A-1826920-T6-2_1580	AACCGCACAGGAAGGAAGCACAACGCG
T6-24	TaASN1	ABD	TraesCS5A02G153900	5A-1230940-T6-24	forward	331,399,779	TTGTGCCAATCAAGGAAAATAGTTGAGTAGCTCTTGGTGCGGCAA	14	62.6	7B-1923970-T6-2_0	CTTTACACCCTTATAACTTGCCTCCCCAAC
T6-24	TaASN1		TraesCS5B02G152600	5B-1310950-T6-24	forward	281,836,969	CCACAAAAGTATCACTAATCACTAATTAACACACTAAGCAAAGTTA	5	28.3	7B-1923970-T6-2_192	CTAGAACAAACCCTGTGCAACAAAGAATA
T6-24	TaASN1		TraesCS5D02G159100	5D-1468150-T6-24	forward	247,610,376	CTCCTCTCTCCCACCTGGCGACCTTGTCAGGGGCTGGCGAAGTGT	17	83.8	7B-1923970-T6-2_256	AAAAATGCATGCATGAACAAAATAATGTA
T6-25	TaASN2	AD	TraesCS3A02G077100	3A-0682630-T6-25-1	forward	47,847,920	GTCCCTATCCATCCCAAAATGTGCTACAGAAAACGAGATGCTGGA	9	47.4	7B-1923970-T6-2_320	AAGATTTTTTTAAGGTGTTATAAAGCTATC
T6-25	TaASN2		TraesCS3D02G077300	3D-0907290-T6-25	forward	37774353	CGCCGCCGCGGCATCTGACATGCCGTTGCCGCCGCCTGTTGCCTC	16	75.5	7B-1923970-T6-2_384	ACTTAAGTAAAATAGTATATACTACTTATTG
T6-26	Trehalose 6-phosphate ph	BD	TraesCS2B02G187200	2B-0453490-T6-26	reverse	162,448,779	GACGAGATCGCGGACCGACACAGGGAGGAAGACTGAAGAAGCG	10	54.1	7B-1923970-T6-2_1344	TATTGTGATTCCAAAAAAATGTATGGTGAT
T6-26	Trehalose 6-phosphate ph	no A	TraesCS2D02G168300	2D-0597240-T6-26	reverse	112,181,315	TTTGATGATTGGGAAATACCGGGAACAAAGAAGAAGAAATGAGA	5	28.7	7B-1923970-T6-2_1408	GATAGCTTTTACTCCAATTTGTTGTGCAAA
T6-27	Trehalose 6-phosphate ph	ABD	TraesCS6A02G248400	6A-1521780-T6-27	forward	461,145,380	TGGATGAAAAAAAAGAAAAGAAAGGGGTTTGAAATCGCGTGGG	17	80.5	7B-1923970-T6-2_1472	CACAGACTGTGTATTTTTTGTCATTTTTATT
T6-27	Trehalose 6-phosphate phosphatase		TraesCS6B02G276300	6B-1612430-T6-27	reverse	500,212,162	CGGGTCGGACGAAGAAAAAGTGTGGTCAGTACGGTGGTCGTCTC	16	80	7D-2060360-T6-2_0	CTTTACACCCTTACAACTTGACTCCCCAAC
T6-27	Trehalose 6-phosphate phosphatase		TraesCS6D02G230500	6D-1718540-T6-27	forward	323,713,668	GCTTCCCCGAGTGAACCTAGCAAATACATGGCGTAACAAAAACAT	14	72.5	7D-2060360-T6-2_192	CTAGAACAAACCCTGTGCAACAAAGAATA
T6-28	Trehalose 6-phosphate ph	ABD	TraesCS7A02G180800	7A-1828020-T6-28	reverse	135,008,564	GGCGCACGCAAACAACCACACGTGTGCTCACGACACGCGCCTCC	21	87.8	7D-2060360-T6-2_256	AAAAATGCATGCATGAACAAAAAAAAAAT
T6-28	Trehalose 6-phosphate phosphatase		TraesCS7B02G085800	7B-1924480-T6-28	reverse	97,974,911	GGCGCACGCAAACAACCACACGTGTGCTCACGACACGCGCCTCC	5	22.1	7D-2060360-T6-2_320	ATAAAGCTATATTTTCTTTGAAAGGTGTTA
T6-28	Trehalose 6-phosphate phosphatase		TraesCS7D02G182600	7D-2033460-T6-28	reverse	136,015,521	GGCGCACGCAAACAACCACACGTGTGCTCACGACACGCGCCTCC	21	84.5	7D-2060360-T6-2_384	TGTGTTAACCACTTAAGTAAAGTAGTATGT
T6-29 & T8-15			TraesCS1A02G350500	1A-0026620-T6-29	reverse	535,318,667	ATAATGATATTATGATAGAAGCAGACAAAAACAAAGTATCCACAGT	23	50.5	7D-2060360-T6-2_448	CATACAGAAAAATCTGGAGTGTAACTGCA
T6-29 & T8-15	TaSnRK1	ABD	TraesCS1B02G364800	1B-0108930-T6-29	reverse	595,184,659	CGTCGACCTAAAATAAATCACAGCCAAGCGATTCAGCGGCAAAGC	7	35.1	7D-2060360-T6-2_576	CTAGAAAGAGCGGTAACTGATTCAGACAT
T6-29-1 & T8-15	TaSnRK1		TraesCS1D02G353300	1D-0193790-T6-29-1	reverse	439,028,615	CGTCGGCCTAAATCACAGGGGCAAAAAGGAATTAAAAGCTATTAT	7	36.1	7D-2060360-T6-2_640	CTTGCATTTTTTAGATATCGGGAGCCAGA
T6-29-2 & T8-15	TaSnRK1		TraesCS1D02G353300	1D-0193790-T6-29-2	reverse	439,026,884	AATGATATTATGATAGAAGCAGACAAAAACGAGGTATCAACAGTG	19	84.7	7D-2060360-T6-2_704	TCTTCGTCTCATTAAAGCTAGACCGCGTAT
T6-3 & T9-64	MAPK4	ABD	TraesCS7A02G422500	7A-1792950-T6-3	reverse	612,993,956	GAGGCTCTCTGTCTATTTTTTATTTCGGGGAAGGGAGGGAGGGA	11	50.1	7D-2060360-T6-2_768	ACAAGCATGCAGCACTGTCATCTCCTTTAT
T6-3 & T9-64	MAPK4		TraesCS7B02G322900	7B-1859240-T6-3	reverse	573,263,406	AGAGTTACCAGTCTATTAATTGAGAACAAAGGAAGCATAACAAGG	9	56.2	7D-2060360-T6-2_832	TCACAACTAATTATCAAAAAATGCTCATAT
T6-3 & T9-64	MAPK4		TraesCS7D02G182600	7D-1974260-T6-3	reverse	532,901,470	ATTCTACCAAGAAGAAGGAAACACGGGTTAACATTCTATACTTGAT	6	39.6	7D-2060360-T6-2_896	GAAAAATAGTTCCAAAATATATAATTATATA
T6-30 & T8-16	SnRK	ABD	TraesCS3A02G282800	3A-0649780-T6-30	reverse	511,078,383	CTTCCACCTTAATCGTAATCACACCAAAGTACACTTCAGGAAACGA	6	32.5	7D-2060360-T6-2_960	ATAGTGTCCATATCTGAAACGTCAGGTATC
T6-30	SnRK		TraesCS3B02G316500	3B-0775050-T6-30	reverse	508,255,985	CTTCCACCTTAATCGCAATCACACCAAACTACACTTCAGGAAATGA	9	43.8	7D-2060360-T6-2_1024	AAGTCAGCACAATTCCAAATTTATAGTCA
T6-30 & T8-16	SnRK		TraesCS3D02G282800	3D-0889780-T6-30	reverse	390,669,972	CTTCCACCTTAATCGCAATCACACCAAACCACACTTCAGGAAACGA	7	46.1	7D-2060360-T6-2_1088	AAAAGTAATGGTCACACAGTAATTATTACC
T6-31	TaPht1.1	AAB	TraesCS4A02G416400	4A-0952320-T6-31	reverse	685,998,752	GGCCGCCGGCGATCTCTCAACTAAACTTCTGCCCCTACTGTACTTC	9	47.3	7D-2060360-T6-2_1152	TCACGGCATCTCTCGCGACACAACAACGC
T6-31	TaPht1.1		TraesCS4A02G416300	4A-2108990-T6-31	reverse	685,941,006	GGCCGCCGGCGATCTCTCAGCTAAACTCTTCCCCCTTCTATCTTTCT	25	99.5	7D-2060360-T6-2_1216	TAACTAAGCTGTCAAGAACAACGAAAAG
T6-31	TaPht1.1		TraesCS4B02G317200	4B-1034400-T6-31	reverse	606909149	GGCCGCCGGCGATCTCTCAACTAAACTTCTTCTGCTGCTCTCTTGT	21	92.2	7D-2060360-T6-2_1280	TGACTTGCGAGAACGAAATTCTCTTCTAG
T6-32	TaPht1.2	AD	TraesCS4A02G416400	4A-0989060-T6-32	reverse	685,998,242	GGCAAACACGGCGGCGATAAAGGTGCCGCGGGTCTTCTTGTTAG	9	44	7D-2060360-T6-2_1344	CAATTCCCCCTTGCAGCATCTAGGATTCAG
T6-32	TaPht1.2	no B	TraesCS4D02G313700	4D-1122450-T6-32	reverse	479,636,164	GGTGAATCCCATGAGCTGGATCCAAAACCTCCCAATAATGTCGATG	????		7A-1792950-T6-3_0	GAGGCTCTCTGTCTATTTTTTATTTCGGgg
T6-33	TaNAC1	ABD	TraesCS7A02G305200	7A-1759120-T6-33	forward	430,240,139	TGACAACGTGACATTCTTGTTCTATAGGCCGGCCGTACTTATGGTC	18	83.6	7A-1792950-T6-3_64	cgtacgcacgcacgcacgcgcgtagctccacggacT
T6-33	TaNAC1		TraesCS7B02G205600	7B-1884660-T6-33	forward	376,430,982	GGGTCTCGGCGATTGCAAGTCACCTTCCTTAGGATTCGGGTCATCT	16	76.7	7A-1792950-T6-3_576	CGCGTTGACCCGGCAGGCGCGTACGGTA
T6-33	TaNAC1		TraesCS7D02G302000	7D-1997730-T6-33	forward	382,255,410	TAACGAGCGACTAGGGCCGTTGATCTGCCTCGTCTCCTGTGGTCTT	20	85.2	7A-1792950-T6-3_640	GGGTTTCGCCGCGTGATGCGGGTTCGGC
T6-34	GPC1 / NamB1	ABD	TraesCS6A02G108300	6A-1564640-T6-34	reverse	77,100,127	CGGATCTTCTTCGTTCCCCCTAGCAAGCTAGCTACCAACAAACACA	14	58.1	7A-1792950-T6-3_704	TGATGATCACTCAGTGGTTACGCCTACGC
T6-34	GPC1 / NamB1		TraesCS6B02G207500LC	6B-1635270-T6-34	reverse	134,664,600	GGGTCGAACTTGTAGAGATCCACCTCGGCGATGATGTTGACGGG	5	28.7	7A-1792950-T6-3_832	CCTAATTCTGTTTGAACAACATTTTGAGTC
T6-34	GPC1 / NamB1		TraesCS6D02G096300	6D-1725630-T6-34	reverse	60,487,980	CGGATCCTCTTCCCTTAGCTAGCTAGCTACCAACAAACACACCGGA	13	57.6	7A-1792950-T6-3_896	TTTCGTGCTTGCATAAACTTTCCATATATG
T6-35	alpha-expansin TaEXPA2	ABD	TraesCS3A02G344800	3A-0638220-T6-35	forward	594,492,193	TGTGAAGGTGAAAAAAATGCTATAAGCTGAGTTTTAAGCACGAGG	12	64.9	7A-1792950-T6-3_1280	ACGCCATCTGACAATGTGAGACACATAGT
T6-35	alpha-expansin TaEXPA2		TraesCS3B02G376800	3B-0789720-T6-35	forward	593163568	TTCTGCTCGCGTAATTTTGTGTTACTTGGCCGTTTATTGTGAAGAA	9	50.4	7A-1792950-T6-3_1344	TCTTCAGGGAGCTGGCGCCGCCCTAAACA
T6-35	alpha-expansin TaEXPA2		TraesCS3D02G338600	3D-0873340-T6-35	forward	451,883,893	ATGCCACTCCAGTGAAGTCTTAAACTAGTTGCGCTATGTTTTCTTAG	19	84.7	7A-1792950-T6-3_1408	CGTTGGCAATTGGTCGGGATCTAACATGC
T6-36	Thaumatin (IPR001938)	AA, B, U	TraesCS4A02G498000	4A-0961700-T6-36	reverse	743,680,749	CGAACACGGCAAGGAAGAGGACCATGGAGGCAGCTGCTGCCAG	12	64.9	7A-1792950-T6-3_1472	TTGGCGATGATGCTAGCGGCGGGTGGAG
T6-36A	Thaumatin (IPR001938)		TraesCSU02G146600	U-0550820-T6-36A	forward	162,315,758	CGGCTCCAGCCGGCGAGGCCAACTGTCCGCTATAACCTCGTGTCG	2	14.1	7A-1792950-T6-3_1536	AAAAAAAAATTGTAGCGTGCCTTCTTAAC
T6-36B	Thaumatin (IPR001938)		TraesCS6B02G473800	6B-0550820-T6-36B	forward	720,805,007	GAACTTCCCCGTTGGTGCATCACCTTTAACCTTCCTTCCGCATGAC	2	14.1	7A-1792950-T6-3_1580	AGATTAACTAGGCAACAGCAAATACTCTG
T6-37	Thaumatin (IPR001938)	ABD	TraesCS7A02G558500	7A-1797770-T6-37	reverse	730,749,909	TGCTGGAAGCTCTAGTTAGCTTTAGTGCTCTTGCTGTGCTCGAGCT	7	32.9	7B-1859240-T6-3_0	AGAGTTACCAGTCTATTAATTGAGAACAA
T6-37	Thaumatin (IPR001938)		TraesCS7B02G483400	7B-1866300-T6-37	reverse	741,353,351	TGCTGCAAGCTCTAGTTAGCTTCAGTGCTCTTGCTGTGCTCGAGCT	10	50.8	7B-1859240-T6-3_128	ACCCCACACACAAGTCATCATCACCCTAA
T6-37	Thaumatin (IPR001938)		TraesCS7D02G551400	7D-1985660-T6-37	reverse	634,805,185	CAAGAACAACTGTGGCTTCACAATTTGGCCGGCGGGCATCCCGGT	14	69.2	7B-1859240-T6-3_256	CTACTAGCAGCATGTGTTTCCACCCCCACA
T6-38	Myb_TF_APL	ABD	TraesCS3A02G105500	3A-0690110-T6-38	forward	69,493,459	ATTTGTTAAAAGGAGCTAAAATTTGGTAGTTGCCTATTCACCCCCT	12	52.5	7B-1859240-T6-3_320	ACACTGTTTGTTTGACTGACCCAGTCCCA
T6-38	Myb_TF_APL		TraesCS3B02G124100	3B-0798870-T6-38	reverse	97,089,899	CTACAAGCACGTGCAATGCTCTGAGTCCACTAACAAAGTCCTCCCC	14	65.9	7B-1859240-T6-3_512	GGCGACCGGAGCCGGAAGCAAACCCAAT
T6-38	Myb_TF_APL		TraesCS3D02G107800	3D-0913190-T6-38	reverse	61,322,360	CTACAAGCACGTGCAATGCTCTGAGTCCACTAACAAAGTCCTCCCC	13	65.4	7B-1859240-T6-3_768	CGACCGAGCAGGAGGGCTCTCTGTCTATT
T6-39	Myb_TF_APL		TraesCS4A02G131700	4A-1025320-T6-39	forward	176,537,677	TTGTGTATTGAGAATGCAATTCAGTTTGCCACATGTGATAATCGTTC	25	53.6	7B-1859240-T6-3_1280	GGGTCGTTTTCCGGATCGGTGCACTAGTT
T6-39	Myb_TF_APL	ABD	TraesCS4B02G172900	4B-1064640-T6-39	reverse	377,801,728	CAAGAATTGTGGACAAGCGTTGCGTCTTTCCCATAAATTGATTTTG	13	55.5	7B-1859240-T6-3_1408	GCATGGTTTCGCCGCGTGATGCGGGTTCG
T6-39	Myb_TF_APL		TraesCS4D02G175000	4D-1137010-T6-39	reverse	303,081,456	CAAGAATTGTGGACAAGCGTTGCGTCTTTCCCATAAATTGATTTTG	16	70.1	7B-1859240-T6-3_1472	CATGATGATGAGTCAGTGGTTACGCTTAA
T6-4	T6-39	ABD	TraesCS7A02G301700	7A-1756350-T6-4	reverse	420,843,374	CGTTGCCGGCCGGCGGGGAGGATCTGTGGTGCGGGTTGGCCGGT	6	29.2	7B-1859240-T6-3_1580	TCAGCTGCCTAATTTTGTTTGGACATTTTG
T6-4	TaNPF6.1		TraesCS7B02G201900	7B-1862440-T6-4	reverse	369,417,090	AGTTGCCGGCCGGCGGGGAGGATCTGTGCGGGGGTGGCGGGCC	13	60.9	7D-1974260-T6-3_0	ATTCTACCAAGAAGAAGGAAACACGGGT
T6-4	TaNPF6.1		TraesCS7D02G297000	7D-1978630-T6-4	reverse	374,570,956	CTGCGACTTCTCGGTGCGGTCCGACTCATCGAACTGGTCCGACCC	12	61.6	7D-1974260-T6-3_64	ATGAAAACGTTTACTAGGTTAGGGCGCCA
T6-40	Alanin-aminotransferase	BD	TraesCS1B02G102700	1B-0153690-T6-40	forward	112,748,817	AGGATGGGCGGTGAGAGGCTGCCCCCCGGGTGCCGCGTGGCGG	10	50.8	7D-1974260-T6-3_128	TTCCCCATGGTACTAACAGTCTCTGTTTCC
T6-40	Alanin-aminotransferase	no A	TraesCS1D02G086700	1D-0253170-T6-40	forward	71,282,048	GTAAGCTACCTACAAAAATTTGCAATCAAAAGGATATGTGGTTTAT	16	83.3	7D-1974260-T6-3_256	CTTATGAGCTTGACCTTTTCCATGGCACCA
T6-41	*TaDEP1*	ABD	TraesCS5A02G215100	5A-1192740-T1-3	forward	430488527	CTACGGGCTGCTGCCGCTCCTCCTACTCCCACTCCCACTAGCCAGTAGCGAGCGCGCCCGTCTTATCCTGGGCCCCCG			7D-1974260-T6-3_320	TATCGTCAACCTAACACCAGTGTCTGTCTA
T6-41	*TaDEP1*		TraesCS5B02G208700	5B-1323430-T1-3	reverse	378,520,474	GTCTCTTTGTCTCTTACACGCCCGGAACTGGAGGGCTTAGGCTTAGCCACCGGCCCGCATTAGGCGCCGGCGGCGG			7D-1974260-T6-3_512	CTCCCGGCTGCCCCTAACACGAGCATCCC
T6-41	*TaDEP1*		TraesCS5D02G216900	5D-1456550-T1-3	reverse	326,129,224	GTCTCTCTGTCTCTCACACGCCCGGACCCGGAGGGCTTAGGCTTAGCCACCGGCCCGCATTAGGCGCCGGCGGCGG			7D-1974260-T6-3_576	CACCCGCATGCATAAGCTCttgctttgcttcgcc
T6-42	*TaDEP1*	A, AAD	TraesCS4A02G392600	4A-0942630-T6-42	forward	668,351,881	GTGTTCTAACTTCCGAGAAGGCTAAACTTCCAGAAGCCCCCCTCAT	3	21.2	7D-1974260-T6-3_768	GGAAGGGGATGGGGAGCGCACGCGGAG
T6-42	*TaDEP1*		TraesCS7A02G093600	7A-0801380-T6-42	forward	57,156,707	AACAGTCCTAACCATAGCACAAGTTCCAGCCACCTTACATTAAAGT	15	72.9	7D-1974260-T6-3_832	CGAGCAGGAGGGCACTCTGTCTATTTTTT
T6-42	*TaDEP1*		TraesCS7A02G093500	7A-1850170-T6-42	forward	57,096,000	ACGCGATCACCGCTCAACAAAAAGTATTGATGCCGCCTCGCCTATG	11	51.3	7A-1756350-T6-4_128	GGTATTCTGGGCAGAATCTCGTGCTTCGC
T6-42	*TaDEP1*		TraesCS7A02G093600	7D-2062490-T6-42	forward	53,711,008	TGACCAACTTTATAAAAAAAAATCAATATTCACACTTCGAAATCAAT	19	91.3	7A-1756350-T6-4_192	TGGGGACTCTCCTCTCCTCTCCTCTGGAC
T6-43	TaIRT1	ABD	TraesCS4A02G294300	4A-0982100-T6-43	reverse	595,663,903	TGCTCGATCCCGCCGTGCGTCTCCTCTTTACTTTTGGTGTGGAGCT	14	71.3	7A-1756350-T6-4_256	ATGATCTAATCTAAATTATCTGCGCTGAGAT
T6-43	TaIRT1		TraesCS4B02G019300	4B-1090980-T6-43	reverse	14002862	CTCGACTTGGATCTCCGCCCGGTGCTCAAGCGTGAGCATCTTATAA	14	62.6	7A-1756350-T6-4_320	TGGGAGGATAAGGGAAGGGCAATTGGTT
T6-43	TaIRT1		TraesCS4D02G017600	4D-1168460-T6-43	forward	7797210	CACTGGCGCATCATACGACTGCCCATACGAGCGGGCGCACCCGTA	17	82.6	7A-1756350-T6-4_512	GACCAACATAAGGTTCAAATTCAAATCAA
T6-44	TaZIP3	ABD	TraesCS2A02G424200	2A-0299310-T6-44	forward	678,666,985	CCCCACCGCAGCCGCCACAGCAGATACCTTCGGCTTGGCAGGAG	19	88	7A-1756350-T6-4_576	TAAATGATCACATCAACTAGCAGATTTATA
T6-44	TaZIP3		TraesCS2B02G443800	2B-0391920-T6-44	forward	636,771,165	CAGCTCCACTCCGCCGCATGCGTGCGTGGTGCTTGTGCAGCTGTT	16	76.7	7B-1862440-T6-4_0	AGTTGCCGGCCGGCGGGGAGGATCTGTG
T6-44	TaZIP3		TraesCS2D02G422000	2D-0546500-T6-44	forward	535,565,697	TATTTGGTGTCATATTCTTCAGATCGGTTCATGGGTTCAACATCAAC	15	72.9	7B-1862440-T6-4_192	GAAGAAGAAAGAGATGATTTTGTTAGCT
T6-45	TaZIP5	ABD	TraesCS4A02G025400	4A-1003790-T6-45	reverse	17,483,882	GGTTTTAGTCGGAGGGGTCGAAAGAATGGTGATCTACCGGAGAC	19	86.8	7B-1862440-T6-4_256	TAAGAATGATCTAATCTAAATTATCTGAGG
T6-45	TaZIP5		TraesCS4B02G278600	4B-1054990-T6-45	forward	561589164	ATATGATTCCACAAATGGCGACATTTTAGCTCATTCGAGTCTTGATG	13	64.2	7B-1862440-T6-4_320	TGGGAGGATAAGGGCAATTGGTTTTGTT
T6-45	TaZIP5		TraesCS4D02G277100	4D-1124750-T6-45	forward	449,201,671	TTGATGCTAAATGTGGTTCAGACAAGGATTTCCACTACCAACGGAT	17	82.6	7B-1862440-T6-4_384	GCTGGCAACCGGCCGCGGCTTCTCCACA
T6-46	TaZIP13	ABD	TraesCS2A02G505500	2A-0325680-T6-46	reverse	733,797,328	GTCTAGACTATCGTGATGCACTTGAGAGCGCGACAACGCGGGGG	12	63.8	7B-1862440-T6-4_704	GTTAGTCCTCACTATGACCAGCCTAAGTCA
T6-46	TaZIP13		TraesCS2B02G533800	2B-0397670-T6-46	reverse	730,082,970	GTCTAGACTATCGTGATGCACTTGAGATTGAGAGGGCGACGACG	11	61.2	7B-1862440-T6-4_768	ACCACTTGTTTCGCGATCTCTATTGGCGA
T6-46	TaZIP13		TraesCS2D02G506200	2D-0534750-T6-46	reverse	600,134,089	GGCTAGGCTGTCGTGATGCACGCGACTGGGTGAGCGATGAAGCG	8	36.7	7B-1862440-T6-4_832	CTATCAACACACGGTTTTTGGAGCGCGTA
T6-47	TaZIP6	ABD	TraesCS1A02G111000	1A-0073380-T6-47	reverse	111,153,817	GGCGGCGCGCGAGGTGCTCGGCGAAATATCGCGCCCGCGCACGC	4	24.9	7B-1862440-T6-4_1216	GCGGAATGTGTCCTACTCGTCCTGATCCG
T6-47	TaZIP6		TraesCS1B02G128800	1B-0167090-T6-47	forward	158,516,533	GTTAACACTCCGTGTTATTACAATTGAATGCAGACGATATTAACTCA	10	50.8	7B-1862440-T6-4_1344	GAGTAAGGATGGAGGACGGCGAGGGTC
T6-47	TaZIP6		TraesCS1D02G112500	1D-0263760-T6-47	reverse	108,262,701	GGCGGGGCGCGAGGTGCTCGGCGAAATATCGCGCCCGCGCACGC	3	17.9	7B-1862440-T6-4_1408	ATTTGTTGCAATTTTTGATGGGGTCGGGC
T6-48	TaZIP7	ABD	TraesCS1A02G125500	1A-0057560-T6-48	reverse	149,514,777	GGCCCGAGAATCCCGAGCGGTGCTAAATTCCTCGAGGACAAAAAT	13	60.9	7B-1862440-T6-4_1472	CTCGCCTTCACCCATAAGAAACAACACAC
T6-48	TaZIP7		TraesCS1B02G144500	1B-0150920-T6-48	reverse	196,465,019	GGCCTGAGAATCCCGAGCGGTGCTAAATTCCTCGAGGACAAAAA	6	27.5	7B-1862440-T6-4_1536	GTTGCCTGGTTTTTTAGTGGCGCCACGTT
T6-48	TaZIP7		TraesCS1D02G128000	1D-0264750-T6-48	forward	141,274,457	AAAAATACCACGGTTTTGAAAAAAAAAATGTCCATCATTCCAAAAAA	5	28.7	7B-1862440-T6-4_1580	ATGTCGTTCCTGATTGCACCCGGCTGCGC
T6-49	TabZIP56/180	ABD	TraesCS7A02G530300	7A-2089960-T6-49	forward	709,638,948	GCTTGTCTTTTTTTTATATGAAATTAGCTCGTCGGAATGCAGGCGC	20	84	7D-1978630-T6-4_192	cggcgccgccgTTGAGATCGTCAGTATCGTCA
T6-49	TabZIP56/180		TraesCS7B02G447900	7B-1878150-T6-49	forward	710,990,579	AGGCGTAGATTCCCGTCGTCTCATTGGGGAAGTGAGGTTAAGTTT	12	57.2	7D-1978630-T6-4_256	ATCTATCGCAACTTGTAAAACATTATTACT
T6-49	TabZIP56/180		TraesCS7D02G518100	7D-2002830-T6-49	forward	616,899,887	GAACATAGTGATGCCTTGTTTTTTCAGCGGGAGGGGAGGCCTAAA	15	65.2	7D-1978630-T6-4_320	AAAAAAGATAGTATGGGAAAAATTCAGAT
T6-5 & T9-58	TaNPF6.2	ABD	TraesCS1A02G210900	1B-0090310-T6-5	forward	403,541,257	ATTGCTTTTAATTTTGTTAACTATTTATACTGACAAGCGGTCGATCA	13	68.7	7D-1978630-T6-4_384	AGGATGAAGGAGATTTTAAGTAGTAGTCC
T6-5 & T9-58	TaNPF6.2		TraesCS1B02G224900	1A-0010190-T6-5	forward	373,766,720	TGAGCGCGCTTGAGAAAGAAACTCGAAGATGTAAACGAACAGTC	12	61.6	7D-1978630-T6-4_512	GGCCGTTCTTTTCACAGCAGTATCGAGTT
T6-5 & T9-58	TaNPF6.2		TraesCS1D02G214200	1D-0198100-T6-5	forward	299,576,089	AGTCAAAGCTCAGTTAGCTAAACCAAAAATGGGTGGAATTACAGA	10	60.7	7D-1978630-T6-4_576	AAAAGAACTAATTACAGATTGATATTTCTC
T6-50	TabZIP3b		TraesCS7A02G530400	7A-1763720-T6-50	forward	709,674,450	GTGGTTACGATGGAGATTTAGAAGGTACTAAGTAAACCACACCTA	11	51.3	7D-1978630-T6-4_640	CACCAAAAGAACGGGATCTTTTATTTAGG
T6-50	TabZIP3b	AAB	TraesCS7A02G558500	7A-1797770-T6-50	forward	730,749,836	CGTGCACATACGCGTGACAGGTCCTAGTTATTTGCTACTCCCTCCAT	14	62.6	7D-1978630-T6-4_1088	TGGGGGAGAAGGCCTACCATACTTCCCG
T6-50	TabZIP3b		TraesCS7B02G448000	7B-1878160-T6-50	forward	711,000,653	TACGTGGAGCCCCTTCTGTCCCGGTTCGTAAGCAAACCGGGACTA	17	77.2	7D-1978630-T6-4_1280	TGGGGAAGAAGAAAGAGATGATTTTATT
T6-51	TaVIT1	ABD	TraesCS2A02G336600	2A-0301300-T6-51	forward	570,192,913	ATCATTGCAGACTGTTCTGGAACTTGGGCCAACCATCCCAATATTC	17	77.2	7D-1978630-T6-4_1344	TTATATGCAATAATAAGAATGATCTAATCTA
T6-51	TaVIT1		TraesCS2B02G345300	2B-0389520-T6-51	reverse	492,148,298	CGCCGCCGCTCGCGAGGGCAACAGACAGAATTGAGCCGACAGG	14	63.1	7D-1978630-T6-4_1408	TCCTTCCCTTCCCTTCGCTTCCCTGGGAG
T6-51			TraesCS2D02G326300	2D-0533310-T6-51	reverse	419,783,650	GAGGGTAATTTGCAAGTTGCAGTTGAGGCTAAAGTAACGAACAG	23	70.6	7D-1978630-T6-4_1580	CGTCCGCCGGTCGCGTAAGGGTAGTAGA
T6-52	TaVIT2	ABD	TraesCS5A02G203400	5A-1191460-T6-52	forward	412,913,439	ATCCGATCTACCGATCTAGGGTTTCCCCGGGAGGTAACAGATAGA	9	60.2	1A-0010190-T6-5_64	AAAAATGGGTGGAATTACAGATATATCCA
T6-52	TaVIT2		TraesCS5B02G202100	5B-1342560-T6-52	forward	367,909,096	GCGTGGCGCGTGCGGCGGCGGTCGCTAGCGGTTCCTGCTTTTCT	11	60.1	1A-0010190-T6-5_128	ATGGAGTAGTAGTCAATTATTGTACAAAA
T6-52	TaVIT2		TraesCS5D02G209900	5D-1414720-T6-52	forward	318,061,312	CACATAGTACCTCGCGTGCCCAAATCCCAAGATTGCCCCGACTTCC	14	69.2	1A-0010190-T6-5_320	TAGCTTAGGTAATTAATTACGTCTAGCACA
T6-53	TaHMA2	ABD	TraesCS7A02G419500	7A-1769490-T6-53	forward	611,317,242	TTTACTGGAAGTACAAAGCACATCAAGCACCGGAGTTTGCCAGCA	16	73.4	1A-0010190-T6-5_448	GGTGAACCTTCTCCAAATCCCGCCTTAACT
T6-53	TaHMA2		TraesCS7B02G320100	7B-1907150-T6-53	forward	570,451,059	TTTCCGGCAATGTTCATTCACTAGGCGGAGATGTTTTCATCGACTAT	11	51.3	1A-0010190-T6-5_512	ACCCCACGAACGCCCTATATACTACTGGG
T6-53	TaHMA2		TraesCS7D02G412400	7D-1963900-T6-53	forward	530,909,177	ATGTAGCAAAGCCGCTGTCCGTGTCCTGCACCCCGCTCTGCCCGG	19	78.1	1A-0010190-T6-5_576	GAAGAAGAGGAAAAGGTAGACACCGGT
T6-54	TaHMA4	ABD	TraesCS6A02G158900	6A-1555330-T6-54	forward	150,966,906	ATGTCCGGGGCATGTCATGCGGTTCCTGGGTGGTGGCAGGATTGA	12	60.9	1A-0010190-T6-5_640	ATGCAAAAAAATTGACCGTGACACTGTAG
T6-54	TaHMA4		TraesCS6B02G192400	6B-1671990-T6-54	reverse	226,404,875	TTCCTGTTGCAGAATTGGTCAGAACAAGATCATAGCAGTAGCAGA	14	62.1	1A-0010190-T6-5_768	AGAAATAAAAAATTGCATCTATGTCCGTAG

T6-54	TaHMA4		TraesCS6D02G153600	6D-1735700-T6-54	reverse	127,386,335	CGCGAACAGGAACACAGGAACTGAAAAGAGGCAACTCCAAAGA	12	56.8	1A-0010190-T6-5_832	CCAGTAGCCAATTTTGACAGACAGTACAA
T6-55	TaNRAMP2		TraesCS4A02G050500	4A-1021690-T6-55	forward	41,112,168	CGGATAGGAAGGAGGCTATGACAATTTACCCACACTCCTCTTCAC	15	57.6	1A-0010190-T6-5_896	AAATGGCCGGACGTCAGTGAAATTACATG
T6-55	TaNRAMP2	ABD	TraesCS4B02G254300	4B-1050740-T6-55	forward	519606318	AATCATCACGTGCACATTCTCATATGCGCCCCTGTTCTTTTCCCGCA	13	67.5	1A-0010190-T6-5_960	CAGTCAACAACAGGTGAAATCAGAGGCA
T6-55	TaNRAMP2		TraesCS4D02G254100	4D-1144210-T6-55	forward	422,959,924	ACAAGAAGGCATGGCAAAAAAACATGTTCGGGTAAAAGAGAGA	8	46.6	1A-0010190-T6-5_1152	CAAAGATCCACCACCGTTCGGCTATAAAA
T6-56	TaNRAMP5	ABD	TraesCS4A02G004400	4A-1013050-T6-56	reverse	3,066,947	TGCCGCCGCCGAACTAGCTAGGCCGGCAAGGTTTCTGGAGAAGA	16	72.1	1B-0090310-T6-5_0	ATTGCTTTTAATTTTGTTAACTATTTATACT
T6-56	TaNRAMP5		TraesCS4B02G300600	4B-1069640-T6-56	forward	586,930,340	TTGAATATGAAAGAACTGCATGCCCAAAAGCCCTACAAGGGCAAT	12	64.9	1B-0090310-T6-5_64	CGTATCATATTCTGTCACCCATTTGCATTAC
T6-56	TaNRAMP5		TraesCS4D02G254100	4D-1144210-T6-56	forward	422959924	ACAAGAAGGCATGGCAAAAAAACATGTTCGGGTAAAAGAGAGA	12	63.1	1B-0090310-T6-5_256	TATCCAGGAGAAAAAACTCGAAGATGTGA
T6-57	TaNRAMP7	ABD	TraesCS5A02G072200	5A-1279000-T6-57	forward	82,982,111	TTCTACAACAAGCCACTTATGTGACTTGTTTGTTTTTAGAGTACCA	22	89.4	1B-0090310-T6-5_384	CCTGgcttgcatgcagcatgcagcatgcatggatCA
T6-57	TaNRAMP7		TraesCS5B02G078700	5B-1380480-T6-57	reverse	95,023,449	CGAGATGCGTTTCTGTGCGAGTTTACGCCGCGACGGTGAGGTGC	13	65.3	1B-0090310-T6-5_448	ATGTGGGACATATATATGCCTTGATCATCCA
T6-57	TaNRAMP7		TraesCS5D02G084900	5D-1479950-T6-57	forward	89,045,506	AATCTCTCTCTCACCTTCCTTTGTGTCGTCGGCGCCCTAGTCCCCAC	14	65.9	1B-0090310-T6-5_640	CCAGTATGCGCTCGTACTAACAGTACAGGT
T6-58	TaYSL2	D no AB	TraesCS6D02G223000	6D-1695080-T6-58	reverse	315,178,708	GGAGGCTGTCCGGCGGCGGCACCAGCGTGGCAGAAGGCTCTCA	5	25.4	1B-0090310-T6-5_704	AACCCAGTTCCCAGGTGAACCTTCTCCAA
T6-59	TaYSL9	ABD	TraesCS2A02G391000	2A-0299340-T6-59	forward	640,706,473	GATCTGCCCCTTTAGGTCTTTTTATATTGGTATAGATGTTTATTGTCC	15	70.3	1B-0090310-T6-5_768	AGGTGTAAGTACCCCACGAACACCCTATAT
T6-59	TaYSL9		TraesCS2B02G408000	2B-0399330-T6-59	reverse	579,691,172	TGCGGTGCCTTAAAGCACATGCCTTTGAATCACTGACATGTGGGC	23	76.1	1B-0090310-T6-5_832	TCCTTGAAAGCCGAAAAGAGGAGGAAAA
T6-59	TaYSL9		TraesCS2D02G387700	2D-0553420-T6-59	reverse	493,082,424	CGCGAGCTCGACGCCCGGCGACGGCGGCGGCGGCCTCGGG	9	40.1	1B-0090310-T6-5_1152	AATAATTCGAGCACCAGGACTTGAAACCT
T6-6	TaNPF6.4	A, BD	TraesCS4B02G375800	4B-1058840-T6-6	forward	659568996	GCAACGGTCTGTCATTAAGTAGCTTTAATAGTCTTCATGGCACTTTA	9	43.1	1B-0090310-T6-5_1216	TCACATCGCGAACCAACATGTTCGTCAAA
T6-6	TaNPF6.4		TraesCS4D02G361500	4D-1145530-T6-6	reverse	508271893	GGTGGATCTCTGGAGCAGTGGATTGGTGGTACGTACGTGTACGCG	14	61.4	1B-0090310-T6-5_1280	TGCATGCACTTTTTATCCTCCACTAAATTTG
T6-6	TaNPF6.4		TraesCS5A02G537100	5A-1186890-T6-6	reverse	693,642,362	CACGGATGTCTCGATGGATTGATCGGTGGTACGTACGCGCGCTAG	12	54	1B-0090310-T6-5_1344	TGAAATCAGAGGCACTTTGGTAGAGCAC
T6-60	TaYSL12	ABD	TraesCS2A02G377200	2A-0292820-T6-60	reverse	619,629,506	CGGGATCTTCTTCCTAACTCTGCGGATTAGCCTGACGGGATCTCCG	8	36.7	1B-0090310-T6-5_1536	CGTTCGGCTATAAAATGGCACGCCTGAGG
T6-60	TaYSL12		TraesCS2B02G394400	2B-0436110-T6-60	reverse	559,193,407	TGCCTTTGGAATGGACGCACCGCGTGCCCCTCCTCCTTGTCTCAG	15	72.9	1D-0198100-T6-5_0	AGTCAAAGCTCAGTTAGCTAAACCAAAAA
T6-60	TaYSL12		TraesCS2D02G373800	2D-0517040-T6-60	reverse	477,264,926	TGCCTTTTGGAATGGACGTACGCACCGCGTGCCCCTCCTCCTTGTT	15	69.6	1D-0198100-T6-5_64	TCGAATATGTGAACAAGCATAAGCATGTAC
T6-61		ABD	TraesCS1A02G071800	1A-0081220-T6-61	reverse	54,076,008	AATAGCTTCGTAGACAAGTATGCCAGCAAGGAGCCATATAAGCTG	19	76.8	1D-0198100-T6-5_192	ATGCATGGATCAGCCAACAGTTAATTTTTT
T6-61	TaMTP1		TraesCS1B02G090400	1B-0162340-T6-61	reverse	90,993,953	CAGAAAAGTCGCAAGCAGCACCTCTGCAAATTTTGTTCCCAGCTG	19	81.4	1D-0198100-T6-5_256	TCATCCATTAACCTAGCTTAGGTAATTAATT
T6-61	TaMTP1		TraesCS1D02G074400	1D-0248740-T6-61	reverse	55,670,390	CTGAAAACAACAACTGAGTCAATACACAAGTACAAGAATGATTCTA	18	87.5	1D-0198100-T6-5_576	TCTTTAAATCAATAAGTCCCTGGAAATAAA
T6-62	TaNAS9	ABD	TraesCS2A02G097500LC	2A-0366380-T6-62	reverse	49,222,238	ACATGCATGGTAATAATTGGTTTGTCCTCATGCATGGATGGATGGA	13	72	1D-0198100-T6-5_704	CATCAGGATCTCTTTTCTCCATGTGTAATTA
T6-62	TaNAS9		TraesCS2B02G111100	2B-0480120-T6-62	forward	72,895,158	CTATCAAAGCGGAGTTAATTTGATTCATTCCATCCCCCGACTGCTTC	13	75.3	1D-0198100-T6-5_768	TAGTGGGTAGCTGAGATCCTTCCTTGAAA
T6-62	TaNAS9		TraesCS2D02G094200	2D-0593010-T6-62	forward	45,799,198	CCATTGTAGAATCTCCTTGTATGTTGATGATGTTGGGATCTTTGTCA	13	64.9	1D-0198100-T6-5_1152	CCCTCAAACCATCGAACCACATGTTGGTT
T6-63	TaHRZ		TraesCS3A02G262700	3A-0646970-T6-63	forward	485677416	GCGGCGGAAAGGGCAAGGAACACATGAGCATGAGAAAGAGAGA	20	85.2	1D-0198100-T6-5_1216	AACATGTTCGTCTAAAATATGAACCTTAAA
T6-63-1	TaHRZ	ABD	TraesCS3B02G296100	3B-0712080-T6-63-1	forward	474,399,587	GTAATGTATAAAACTATTTTTTTACCAAAATAACACACCTCCTCATG	21	85.6	1D-0198100-T6-5_1280	ATCCTCCACTAAATTTGATAGACGGGCAC
T6-63-2	TaHRZ		TraesCS3B02G296100	3B-0712080-T6-63-2	forward	474,397,433	ATTAATTAAACATAGCAAGTACACAACATGACCGATAACTGTAAGG	24	99.5	1D-0198100-T6-5_1344	CTTTGGTAGAGCACGACGGAAGGAACTC
T6-63-1	TaHRZ		TraesCS3D02G262600	3D-0848520-T6-63-1	forward	364,253,801	GGAGAGAGAGAAAGAAACGGGGAGAATAGGAAGCATTGAAGTT	22	89.4	1D-0198100-T6-5_1536	ATGGCACGCCTGAGGacctccacagccaccaa
T6-64	TaTOM1	BD no A	TraesCS4B02G328500	4B-1039150-T6-64	forward	619443717	AGAAGAGTGGCATCCTTGGTAAGGCGTCCACGACTGCTTAATGGA	13	68.7	5A-1186890-T6-6_0	CACGGATGTCTCGATGGATTGATCGGTGG
T6-64	TaTOM1		TraesCS4D02G325600	4D-1125130-T6-64	forward	485,612,854	CGTCGTCTGTATATTATGGATCCTATTCAAACATCACAATCGTCGGA	9	47.1	5A-1186890-T6-6_59	CACTCACTTCATTTATATCCAGCCACTCAA
T6-65	TaSultr1/1a	ABD	TraesCS4A02G043400	4A-1010510-T6-65	reverse	36,339,903	TGTTGCCGACGTTGCTTGCACCTATGTGCAGATGGATACGTCTGTC	10	50.8	5A-1186890-T6-6_118	AGTGCACTTGTTCTTATGACACCAACTCTC
T6-65	TaSultr1/1a		TraesCS4B02G263900	4B-1052590-T6-65	reverse	534,738,513	TATCACCGACAAGGTTGCTTGTGCCTATGTGCAGATGGATACGTCT	12	54	5A-1186890-T6-6_177	AGTTGCGAATTGAACAGGACAAACAAAG
T6-65	TaSultr1/1a		TraesCS4D02G325600	4D-1125130-T6-65	forward	485612854	CGTCGTCTGTATATTATGGATCCTATTCAAACATCACAATCGTCGGA	9	43.8	5A-1186890-T6-6_233	AAGCTTGTTATTGGTTCCAGCGCTACACAT
T6-66	TaFRDL1	ABD	TraesCS4A02G066800	4A-1009640-T6-66	forward	64,142,451	TACCTTTAGTAAGATGATCTTTCACTGCACGGACATAATTTGTTACA	9	46.5	5A-1186890-T6-6_614	GAAAGTACTCCTACATTGCTACTCTTGTCG
T6-66	TaFRDL1		TraesCS4B02G244400	4B-1034040-T6-66	forward	506,139,247	GCATGCACTATCTCGTAACCATTTTTTTTACTATAATCCTGTACCAAA	14	69.2	5A-1186890-T6-6_679	TATGCCGTCCGTACACCTGGCTAGGACTTC
T6-66-1	TaFRDL1		TraesCS4D02G264000	4D-1115020-T6-66-1	reverse	434,839,420	TTTTGCCGACGTTGCTTGCACCTATGTGCAGATGGATACGTCTGTC	14	68	5A-1186890-T6-6_744	GATTTGGATGTATGGACGGATGGTCACGT
T6-66-2	TaFRDL1		TraesCS4D02G264000	4D-1115020-T6-66-2	reverse	434,832,117	CGCAGCGTATATCAGAGGTGGAACGAAGCTACTGTCTGCAAACGC	15	79.5	5A-1186890-T6-6_809	GAACCGGTCGTGTCGTCTTTTATTTCCTTC
T6-67	TaIDEF1	D, ABD	TraesCS4D02G245000	4D-1147220-T6-67	reverse	409,644,891	ACACAGTGGATGCTTGCCTGAGAGGTCGATCGATCAACCGATCGT	13	57.6	5A-1186890-T6-6_1004	AAACCCTGGAACATACACAAGGTATTCAG
T6-67	TaIDEF1		TraesCS7A02G329600	7A-1793720-T6-67	reverse	481,251,115	TCCCCTTGGCCTTTGGCAGACACCTCTACTCGCGCATCTACCAGCC	17	79.3	5A-1186890-T6-6_1069	TATTTACACTGTAAATTCAGTACAATGGCC
T6-67	TaIDEF1		TraesCS7B02G230100	7B-1880190-T6-67	reverse	432,504,504	TCCCCTTGGCCTTTGGCAGACACCGCTACTCGCGCATCTACCAGCC	13	62.1	5A-1186890-T6-6_1134	ACACTATACAAAGGCATGTATTTACAGTGT
T6-67	TaIDEF1		TraesCS7D02G326300	7D-1986040-T6-67	reverse	416,632,997	TCCCCTTGGCCTTTGGCAGACACCGCTACTCGCGCATCTACCAGCC	18	79.8	4B-1058840-T6-6_384	GCTTGCTCTTGAGCGGATCCATACCCGCG
T6-68	TaIDEF2	UBD	TraesCS2B02G616900	2B-0397780-T6-68	forward	793,797,057	GACAAAACGGTCGACGTTGTAGAAGACGGGGAGGGGATAAATTA	12	61.6	4B-1058840-T6-6_704	CTATGCTGAAGGCTTGCCACGATGACGCC
T6-68	TaIDEF2		TraesCS2D02G567000	2D-0521450-T6-68	forward	635,781,189	GTGACCCCCTGTCAAGACAGCACAACGGGGAAACTGGAACGACC	15	71.8	4B-1058840-T6-6_1207	AAACGCTCTTATATTTCTTTACAGATGGAG
T6-68	TaIDEF2		TraesCSU02G137200	U-0283710-T6-68	reverse	124,816,691	TAGGGGAAATTGATTGATATGTAGTAGAGTTGCCTTAACTATTTTTA	14	63.8	4B-1058840-T6-6_1270	CGACCAGTTCAGCCAACTAAAAGTTCAGC
T6-7	WRKY TF	ABD	TraesCS5A02G185700	5A-1229100-T6-7	forward	386162310	AGTAAGACTAAAGTCACCGTCTAAGTAACAATTTTATTAGTAAGGA	19	84.7	4B-1058840-T6-6_1333	ACGGACGGCATAGCAAGTACTAAGTACGT
T6-7	WRKY TF		TraesCS5B02G183800	5B-1334290-T6-7	forward	334,682,766	CTGCCATGCATATTTTCCCTGACTGATCTCGTTGAGATATCAGCCGC	12	61.2	4B-1058840-T6-6_1396	TAGCAGTGTAGGAGTACTATTTTTTGTCCT
T6-7	WRKY TF		TraesCS5D02G190800	5D-1437950-T6-7	forward	293,163,340	CGCTGGGAACAAATTCTCAACATCCCATCGAAATGTATATGTAATAC	15	79.5	4B-1058840-T6-6_1459	ATTAACTTTGATGTTCAATCTTGTAGTACTC
T6-71	PSTOL1	ABD	TraesCS3A02G261800	3A-PTO1-T6-71	reverse	484,638,492	TTTGTTTACTGCATCACAGTAGAGGCATGAAATCAGTGCAACTATA	20	88.5	4B-1058840-T6-6_1522	AAATAAATTGACAGTTAATTGAAATCGCAC
T6-71	PSTOL1		TraesCS3B02G295000	3B-PTO1-T6-71	reverse	473,695,792	TGAGCTGCCTGCTATTGTATCTGCATTTGGAGGGAGAAAGCCATCA	18	74.4	4B-1058840-T6-6_1580	TGATAAACCGGAGGAGTATACTACTTCTTC
T6-71	PSTOL1		TraesCS3D02G261800	3D-PTO1-T6-71	reverse	363,599,054	AAGTATGCCTGAGCTGCCTGCTATTGTATCTGCGTACAAATTTTGGA	22	91.5	4D-1145530-T6-6_0	GGTGGATCTCTGGAGCAGTGGATTGGTG
T6-72	TaVP1	ABD	TraesCS7A02G141300	7A-TaVP1A-T6-72	forward	92,888,489	TTAATATCTAATTTCTCCATAAGGCCTTATTCGTTAGAAGAGGTTTAC	18	84.2	4D-1145530-T6-6_64	TCACTCACTCACTTCATTTATATCCAGCCAC
T6-72	TaVP1		TraesCS7B02G042600	7B-TaVP1B-T6-72	forward	42,027,483	AAGGAATCACCGCCGGCCCCATGCCAACCCCTCCCCCTCCCCCTCC	17	77.2	4D-1145530-T6-6_128	TGGCGGGCAACTCGACCGGCTGATCAGTC
T6-72	TaVP1		TraesCS7D02G142600	7D-TaVP1D-T6-72	forward	90,848,761	ATGGGAATATCCCCATAGGGCCCAAATACCAAGGAATCACCGTCGC	19	88	4D-1145530-T6-6_192	ACTCCTCCGGCTCCAGCTAACTGTCAATTT
T6-73	TaNAS2	ADD	TraesCS6A02G163100	6A-TaNA-T6-73	reverse	158,317,829	CTTCTGGGCATCCATGTTATCGTTGCTTCTTGGTGACCGGAAGAAC	10	40.9	4D-1145530-T6-6_256	GCCAGTGCGATAGTTTGTTCTTTTCATCCC
T6-73	TaNAS2		TraesCS6D02G148200	6D-TaNA-T6-73A	reverse	121,226,571	CTGTCCTCAACTCGTCAGTCGCCTGCGATCGTGGTAAATTTCCTACT	7	42.8	4D-1145530-T6-6_320	GCACTACACGTACATACGACAGTGGTGTT
T6-73	TaNAS2		TraesCS6D02G148600	6D-TaNA-T6-73B	forward	121,579,447	AATTGGAGAGAGACGCTAAACCGGTTGTTGATCACCAACGACGGT	10	50.8	4D-1145530-T6-6_384	AAGTTAAATGGGGCGGTCACTCCAATACA
T6-8	WRKY TF	ABD	TraesCS5A02G185600	5A-1231240-T6-8	forward	385,955,408	GAATTTTTTCTTGAGGATTCGTCCTCGTCCGACGAATCGAACATAG	11	56.7	4D-1145530-T6-6_448	CATTGCTACTCTTGTCGAAACCAAGTATAT
T6-8	WRKY TF		TraesCS5B02G183700	5B-1326080-T6-8	forward	334,593,512	ACTATATGAACAACATTACACAACCTATTAGTGCCCACCTATATGAA	22	86.1	4D-1145530-T6-6_512	ACACCTGGCCCGGACTTGTGTTTGTCTTCT
T6-8	WRKY TF		TraesCS5D02G190700	5D-1447070-T6-8	forward	293,095,553	TACCATATCGTTAGTTGGCCCGACATCTGCAAGCCCCGGGAACAAC	3	17.9	4D-1145530-T6-6_576	atgtatggacggatggTCACATCGATCTTTGTT
T6-9	SUMO E3 Ligase	ABD	TraesCS1A02G065700	1A-0075430-T6-9	forward	48,577,215	ATGCACGGTGGTGAGCATCATGAAATTGGTGATGGAGGATGGTTG	15	46.9	4D-1145530-T6-6_1024	CCTCTTCGGGAAAATACCAACGCGGATAC
T6-9	SUMO E3 Ligase		TraesCS1B02G083900	1B-0253990-T6-9	forward	68,068,258	GAGGCGACCCCCTCGAATCGAGGCGGTAGGAGTCCGGCACCGCA	3	17.9	4D-1145530-T6-6_1088	GGGGCGGCGGAGCGGACGGAGCTTGCT
T6-9	SUMO E3 Ligase		TraesCS1D02G066700	1D-02416401-T6-9	forward	48,380,590	TTTGGGAGGAGCAGAGTGGGGAGAGATGGCGAGGAAGGAGGA	4	21.6	4D-1145530-T6-6_1152	AGCAGGCCCGTTCGGCTTCAGCTGGCGG
										4D-1145530-T6-6_1280	CGAGGAAGAAGATGAGTCGGGGTGGCG
										4D-1145530-T6-6_1408	ctttagtcccggctccaactaagaaccgggactaaag
										4D-1145530-T6-6_1472	gcgcggaagctgaaacctttagtcctggctggtggttg
										4D-1145530-T6-6_1536	ttaattttcattttgaagaaattattCGACAGATTCA
										4D-1145530-T6-6_1580	AGATTGAAATTAATTCTGTAAAATGCATAA
										5A-1229100-T6-7_0	AGTAAGACTAAAGTCACCGTCTAAGTAAC
										5A-1229100-T6-7_64	ATAAGACCAGTAATACTACAAGATCTTGTA
										5A-1229100-T6-7_128	TGTAGTGGGGTGCTGTCGAGGAGTCGTA
										5A-1229100-T6-7_192	TCCCAAAGGAAAGTGGATTTTACCTACCC
										5A-1229100-T6-7_256	GAAGATATACTATGTGGTATGTACTACCAT
										5A-1229100-T6-7_320	AAAGAGACAGAATATACTGCTGGAAGAA
										5A-1229100-T6-7_384	TCTTGTTGAGATATCAGCCGCATCATCGAC
										5A-1229100-T6-7_448	CGGGCTCACGGTCTCGCCACAAGATCACA
										5A-1229100-T6-7_512	TCGCCGAGCCTTCAAAAAAAAAGAAGAA
										5A-1229100-T6-7_576	TGGAACCAACCTtgcatgcatgcgtccatgccat
										5A-1229100-T6-7_640	GAACAAGACAAGGCCACCACGTCACATAT
										5A-1229100-T6-7_704	ATCACGGCGACTTACTTTGCATGTATGTGG
										5A-1229100-T6-7_896	GCTATTAAGAAGAAGAATCCAAACGCGTG
										5A-1229100-T6-7_960	CCATTCTTATTTCCTTTTGATGATGTCGTGG
										5A-1229100-T6-7_1024	AGCATGAATATAGCCCGTAAGATTCTATAT
										5A-1229100-T6-7_1152	CAGTGCATATAAATTTGGGGGATTACTCCT
										5A-1229100-T6-7_1216	CTCCCCCAGGAAAGATTCCAGTCATGAAT
										5A-1229100-T6-7_1280	GACTCTACTGTGAAAACAAAGCCAAAGG
										5A-1229100-T6-7_1472	AGCTCGCCGCGGTCACTGTCCTCTTGTTT
										5A-1229100-T6-7_1536	CACCTCCTCCCTTCTCCCCGATCACATCCT
										5A-1229100-T6-7_1580	TCCGCTCTCTCcacacacacacacgcacacactc
										5B-1334290-T6-7_0	CTGCCATGCATATTTTCCCTGACTGATCTC
										5B-1334290-T6-7_189	GCATGCGTCCATGCGCCACCATTCGGCCC
										5B-1334290-T6-7_252	GTCACATGCCTGCCTTGAGCGTGTACGGG
										5B-1334290-T6-7_315	CTTTATAGGAGTGGCAAAGGAAAGACAT
										5B-1334290-T6-7_441	GTCTTGTTTCGAAAATCCGGGCATGAACA
										5B-1334290-T6-7_504	ACGCGTGTATAGCCAACAGATCTCGATCG
										5B-1334290-T6-7_567	ATGGCTTTCATTTCTGCAAACAACTAAAG
										5B-1334290-T6-7_630	GTCGTGGCTTTGTGGTTTTTAGAACACTA
										5B-1334290-T6-7_693	TCTATATGAGAATTTCACAGAAACTTTAAG
										5B-1334290-T6-7_819	GATTACTCCTCTGTTATCCATAAGAAAATT
										5B-1334290-T6-7_882	AGGCATGAATTAAATTTCTCTGAAATTTCT
										5B-1334290-T6-7_1060	CGTCTATCTAGGAATCCATTAACTCCGACC
										5D-1437950-T6-7_0	CGCTGGGAACAAATTCTCAACATCCCATC
										5D-1437950-T6-7_64	ACTGCCAGAGATATCAGCTGCATGCATATT
										5D-1437950-T6-7_256	ACCTTCTTGCATGCGTCCATGCACCATTCG
										5D-1437950-T6-7_384	TGCATATATGTGGCCTCTAGGAGTGGCAA
										5D-1437950-T6-7_448	ATGATCCATCCATGTACAGTCATGTGACCG
										5D-1437950-T6-7_512	AGGCGCGCCGCCTTGTTCCGAAAAGCCG
										5D-1437950-T6-7_576	GAATCCAAGCGCGTATATAGCCAATAGCTA
										5D-1437950-T6-7_640	TCTCCTGTTGAGGATCGTTTTGATTTCTCC
										5D-1437950-T6-7_704	TGCGATGCCGGTGACGTGGCTTTGTGAAT
										5D-1437950-T6-7_896	GCATAGAAATTTGCGAGATTACTCTTCTAT
										5D-1437950-T6-7_960	CCCAGGAAAGATGTCATGCCTGAATTAAA
										5D-1437950-T6-7_1024	TGAAAACAAAGCGAACGGGGAATTTCTT
										5D-1437950-T6-7_1216	TGCCTCGAAATTCGAGGTTCTAAACTCAT
										5D-1437950-T6-7_1344	GCAGGGCCCGGGCTTTGCTAGCAACAAC
										5D-1437950-T6-7_1472	CTGCTCCCGGACGCGGTTCCTCCCCCCAT
										5A-1231240-T6-8_320	ACCCAAAGGAGAAACGCCGCGGGCTACT
										5A-1231240-T6-8_448	CTTTCTTAACTTTTTGAGACTGATATTGCC
										5A-1231240-T6-8_512	CATTAACTCATTGATGTTGCTAGCTTATTG
										5A-1231240-T6-8_832	gcgtcaacgccaatgcttcgattgcgccgggggaG
										5A-1231240-T6-8_960	ATGATATCTCTGTACATCTATATATTGGCAC
										5A-1231240-T6-8_1152	GGAGGGAGTAAGTCTCAAAGCGTGCACG
										5A-1231240-T6-8_1216	TTCACGCTCATAAGGCCGGCACACCATAT
										5A-1231240-T6-8_1280	CCGAAAAAAAGGCCCACTCAAGCTTGTT
										5A-1231240-T6-8_1344	GTATCACTTATGAGTCTGTCTTAAGCTGAA
										5A-1231240-T6-8_1408	GATCCGTTAGCTGAAACTTTGCAACCCGA
										5A-1231240-T6-8_1536	GCCCATTATATAAGACCGCCTCCTTTCGCT
										5A-1231240-T6-8_1580	TTCTCAACTCTCCGATCACATCTCGATCTT
										5B-1326080-T6-8_0	ACTATATGAACAACATTACACAACCTATTA
										5B-1326080-T6-8_64	GCGATGTTAGTTGAAACAACTAGAGTATT
										5B-1326080-T6-8_128	TAATTAAGAGGAGGAATGAGTTTGTTACA
										5B-1326080-T6-8_192	CAATTGTTCTTAGTTTTTGTTCGCGTCGGC
										5B-1326080-T6-8_256	TTTAGAAGGACCATTAGTTGCATGCTCTTG
										5B-1326080-T6-8_320	ACCAAATAGTCAAACCAATGAGCATTGTA
										5B-1326080-T6-8_384	GCTTCGGCCGGATGCTTGATCCACCACTC
										5B-1326080-T6-8_448	CAAAGCCATCGAGATGGAGCCTATCATTT
										5B-1326080-T6-8_512	AAAGAAGGGGTGCACACCTTATTCTTGCA
										5B-1326080-T6-8_576	CATCCGCGTTTGGCTAAATTATCAGCGGT
										5B-1326080-T6-8_640	CATGACCTCGGGAGGGGCCCAAGTGTGC
										5B-1326080-T6-8_704	GTGTAGACGGTAGCCACGGAGTAAGCAC
										5B-1326080-T6-8_768	CATGGGCGTCAAAAGTGGAAGTCGCAAA
										5B-1326080-T6-8_832	CTAAGGAGGAGTTAACGGTAATCTTTATT
										5B-1326080-T6-8_896	CACCTTCCAATTTTTTTTATTTTTTTTCTCG
										5B-1326080-T6-8_960	TTGGTTTCGGTTATTGATCCAAGGTGCCTA
										5B-1326080-T6-8_1024	ATGTTGGTGCTGGCATAGAAAAGGTCAAA
										5B-1326080-T6-8_1088	AAAATTTTGATGTTTCTTTCCATTCAAACT
										5B-1326080-T6-8_1152	TGCAGTGTTGAGCACCTGGATCCTCATGT

5B-1326080-T6-8_1216	GCATTTCATCCCGATAGTCTTGTCCGATTC
5B-1326080-T6-8_1280	TGCTAACCTTTTCATACTCCAGTTGTTTATA
5B-1326080-T6-8_1344	GATTATAAAAAGTGTCATAAGCTGAAGGT
5B-1326080-T6-8_1472	TAAGCCCGCCGGTCAAAAATACTGTTCAG
5B-1326080-T6-8_1536	TTATATAAGACCGCCTCCTTTCGCTtcactca
5B-1326080-T6-8_1580	aactctccgatcacatctcGATTTTTCCTCTTCTa
5D-1447070-T6-8_1344	GTCATAAGCTGAAGGTTCCTGCATGTAAG
5D-1447070-T6-8_1472	CAAAAATACTGTTCAGTTTGTTCACCTTAA
5D-1447070-T6-8_1536	GCCTCCTTTCGTTTCACTCACCATCTCCCC
1A-0075430-T6-9_496	AGACGTATCAAGTTGCAGTCTCTCTGGGT
1A-0075430-T6-9_558	GACAAACCCAAGGCCGGCAACCACCCGG
1A-0075430-T6-9_620	GATGCGACCTCACTGCGGATGGCAAGCT
1A-0075430-T6-9_868	GGGGTCCGCCCGTGTCGCCCTGGGGACG
1A-0075430-T6-9_930	AGCCCTTAAAATACACATCTACCCACAGAT
1A-0075430-T6-9_992	TACTAGAATTTTTTAGTTATAAGGCTCAAG
1A-0075430-T6-9_1798	GACGCGGGTGGGGGAATTCGAGCCCGTG
1A-0075430-T6-9_1860	TGCTGGTGGTAGCGATGGATGTGGAACT
1A-0075430-T6-9_1922	AGGAGGCAGTGGATTAGTTGGCGTCAGT
1A-0075430-T6-9_2108	TGGCATGTTTCTGTAATAATTTGGCTCCGT
1A-0075430-T6-9_2294	GGTTGGTCCAGTGTCCACAATCATATAGG
1A-0075430-T6-9_2356	GTCTTAAAGTTGACATGAGAAAATAGTTT
1A-0075430-T6-9_2418	CATAAGTAGGATTAGTTGTACTAGTGGGT
1A-0075430-T6-9_2480	GAGCGAGATTAGCTGTACTATGCTCAAAT
1A-0075430-T6-9_2597	ATCCTCCATGTCAGCTTGCCTGTAAAAATT
1B-0253990-T6-9_640	GTATGTGACACGGTTGGATGTCGTCCTTC
1B-0253990-T6-9_704	AACGGATGCGAAAGAAAATTTGCAGGTT
1B-0253990-T6-9_1024	CAGTCGGGAAGTCGTCGTACTGTAATTTT
1D-02416401-T6-9_0	TTTGGGAGGAGCAGAGTGGGGAGAGAT
1D-02416401-T6-9_832	GCGAGGGAGGGGGAGCCGGTGGCGGC
1D-02416401-T6-9_896	GGGGGCCGAGCGGGGTCAGACTGTCCGT
1D-02416401-T6-9_960	TCCATAGGCGGGTAAAATACATTTAATAGT
6A-1557980-T6-10_0	GCTGTGCTGTGTTTGTGTCGTGGGACAAG
6A-1557980-T6-10_183	TAGCAGCCCTATCCTTATAGTCGACTGGCC
6A-1557980-T6-10_242	TGCCTCGAGCAATTCAGTCAAAATCGAAA
6A-1557980-T6-10_854	CGCATGCATCCACCGTGTCGACCACCGAG
6A-1557980-T6-10_920	CTCAGGAGGTCGAGCAACATCACACCTAC
6A-1557980-T6-10_986	AGCACAAGGGCAGCCGTGCAATGTCGGC
6A-1557980-T6-10_1052	AACTATTCGGGGGACTCACTTAGGTTCTC
6A-1557980-T6-10_1118	ATAACTGTGTCATCCAGGTCCAAACCAGC
6A-1557980-T6-10_1184	TAAAGGCGACATCGGAAGAGGCGGCTTG
6B-1665930-T6-10_0	CATTTATCGAATATGATCAAGAGTAGGAA
6B-1665930-T6-10_65	GTGGATCTGGAAGAAATTCCCCCCTACCA
6B-1665930-T6-10_195	AGGTTGTTACTCTTGATCCTGATCCAACG
6B-1665930-T6-10_260	TACCACTATGGCCAAAATCAGCATCTCATT
6B-1665930-T6-10_325	GGGGGGGGGGCAACTTTGGACAAATCTT
6B-1665930-T6-10_455	TAACAGGAATTCTGGTGTAGCTCCGCCAC
6B-1665930-T6-10_520	CCGGACTCCATATATACATTTGTGTCTTTTT
6B-1665930-T6-10_585	GCCACCAAACCGCCTCTTCCAACGTGGCG
6B-1665930-T6-10_650	TTAGGGCTGGTTTGAACCTGAATGACACA
6B-1665930-T6-10_715	AAAGTAGAGAACCTAAGTGAGTACCTCG
6B-1665930-T6-10_780	GAGTCGAAGGCCAAGATTGCATGGTTGT
6B-1665930-T6-10_1271	TAATCGAAGATCTAAGTCGAGTATGTAGAT
6B-1665930-T6-10_1333	GCATTTCGGTTAGAGATGGTGCTAATTAA
6B-1665930-T6-10_1395	TTTCGATTTTGACTGAATTGATCGGGGCG
6B-1665930-T6-10_1519	TCCACTCGGGAGGTCGGCGTTTGCGTGT
6B-1665930-T6-10_1580	TTTCACTAATCCCGCGCCCCCTCCCGGCC
6D-1744010-T6-10_0	GCTGTGTTTGTGTTTGTGTCGTGGGACAA
6D-1744010-T6-10_53	CGCGGCAGATGAGAGAAGGAACCGGCA
6D-1744010-T6-10_788	CGCATGCGTCGACCGTGTCAACCATCGAG
6D-1744010-T6-10_854	CTCGGGAGATCGAGCAACAATCCCACCTA
6D-1744010-T6-10_920	GAGCATGAGGACACCCGTGCAATGTTAG
6D-1744010-T6-10_986	AAACTATTTCAAGGGACTCACTTAGGTTC
6D-1744010-T6-10_1052	CAATAAGTGTGTCATCCAGGTTGAAACCA
6D-1744010-T6-10_1118	GATCAAGGCCATGTCGGAAGAGGCTGTT
6D-1744010-T6-10_1250	GACAGTGGTGGCCATCAGTGGCGGAGCT
6D-1744010-T6-10_1316	TGAGGAGCCAAAATACCAATTTTCCTTTC
6D-1744010-T6-10_1448	TCGACTCGAGCAAGGTAGTGTGTAGTAGA
6D-1744010-T6-10_1514	GGTGTCGTTGATGGTGTTCTTTCCTCTGC
4A-0966470-T6-11_256	TTTCCGGTAGTGTTGTGCCCTTCTTAGAA
4A-0966470-T6-11_320	TCATCGATCACTTGATTAATCGTGTGGTCG
4A-0966470-T6-11_384	TAAACCCGCGCGCTCGTTACAAAGAACG
4A-0966470-T6-11_448	TCACTTGATTAATTGTCTGGTCGAGTTCTC
4A-0966470-T6-11_512	TCTCACAATATAATCTTGCCTAGTGGCCGT
4A-0966470-T6-11_576	GCTAGTTGTACAATGCTAGGGAATCTCTTT
4A-0966470-T6-11_640	ATCTAACTCTGTGAATGAATGAATGTATGA
4A-0966470-T6-11_896	tatttaaaacattgatGGATGCCCTTTCAAGCC
4A-0966470-T6-11_960	CTTTTTCTTATTATTATGCACACATCCTTAA
4A-0966470-T6-11_1024	CAACACACAGAATGGTGCATGCATGCATG
4A-0966470-T6-11_1088	ATTCCTCGCAAAAAAGAAAGGAAAAAAA
4A-0966470-T6-11_1216	ATAGACCGAGATCAAAGAGTTTGGTGTG
4A-0966470-T6-11_1280	TAGCTTTATCTATAAGTTCAGGGTTTACTT
4A-0966470-T6-11_1344	TACAAGTTATGCCATCATTAATAATTGTCA
4A-0966470-T6-11_1408	TATGAGCTGCACGGATCGTACATACAAAA
4A-0966470-T6-11_1472	TGAAACAGTTAATTAGCTAGCATCTATCGT
4A-0966470-T6-11_1536	TAATGTATTTTAATTAGGTTTTCATTAGGT
4A-0966470-T6-11_1580	AAACGCTTTTATATTATGGGACGGAGGGA
7D-2044470-T6-11_0	TTCAATACCATGGGAAATTCATCTGTTTTG
7D-2044470-T6-11_64	TTTTTTAAGGGACCGAAAACAAAAAAATT
7D-2044470-T6-11_128	AGAGTTTTATAATGGAATTGTCACATTCAT
7D-2044470-T6-11_192	CATACCATTATACTAGCGGGAGACAAACG
7D-2044470-T6-11_320	TGGCATCTATCGTCACTGATTTGGATGTTT
7D-2044470-T6-11_384	AGAAATCTGGACAGGCGTGCCTTGTTGCT
7D-2044470-T6-11_448	TATATTTGAGTGAGAATTTTTAACGATTAA
7D-2044470-T6-11_832	aagaatacaccttatataaaggaatggaaggTGTAT
7D-2044470-T6-11_896	TTAACACTTAACTTCTCTGGAAAAGTAGG
7D-2044470-T6-11_1088	CCGCTGCCGCCTTCCTCTAGCTTGGTGCT
7D-2044470-T6-11_1152	CCTCCTAGGGATCCTATAAGAGGTAGCAA
7D-2044470-T6-11_1216	TTCCCCATCCTTTACTCCTCTAAATTCTTCC
7D-2044470-T6-11_1280	CTTTTGCACACCTAGTCCTTCCCTTATAACT
7D-2044470-T6-11_1344	TTTCAACCACAAATTTAGACTATATTGATT
7D-2044470-T6-11_1408	CATGGCGTAACTAAGCACTAAACGAGCAT
7D-2044470-T6-11_1472	GAGCTAAAAGGACTAGAAGAAGGCGATC
7D-2044470-T6-11_1536	GCGCCGGAGACGTCGTGCTCATGGTCGT
7D-2044470-T6-11_1580	CTAGGGCATACCATCATTGTCCTTATCTTTG
2B-0391860-T6-12_0	AGTGGATGTCCTGGCTCAGACATCTCCCG
2B-0391860-T6-12_64	CGACGTCCTGGGACCCCTGGAAGAACTT
2B-0391860-T6-12_128	GCTGATTTCGCCGAGTTCAGGTCGAGGT
2B-0391860-T6-12_256	GCTGCACGGGAAGCATGGAGGCTAGTCT
2B-0391860-T6-12_320	TGGCCTTGACGTTTTGCAGCATCGGTCGT
2B-0391860-T6-12_384	TTTTGACATGCTACGATGTACTAGGATAAA
2B-0391860-T6-12_448	TGTACTAGGATAAAATCCCATGTGTTGTGC
2B-0391860-T6-12_896	GGCAAGTCACCGGATCTCAGTCCCCCCAT
2B-0391860-T6-12_1152	CTGGGCTGGGTATGGCGGGAGGCGGAC
2B-0391860-T6-12_1216	CCGGAGCCGGGGGAGGGAAGGAACAAG
2B-0391860-T6-12_1408	CAATCTAAATTACTTATACCACCGGCCTCC
2B-0391860-T6-12_1472	AAGGTCGTTccgcccccttccctccctcctctcct
2B-0391860-T6-12_1536	CCGGTGAGTACTGCGGACTCCTCGATGCA
2D-0558280-T6-12_0	TTTGTGCACATTGCTCTGCACTGCTGGTT
2D-0558280-T6-12_64	TCCAGTCCAAGCTGGAAGAACAAGCTCC
2D-0558280-T6-12_128	GCTCTCCAACAGCGATCCTAGCCTTCGAA
2D-0558280-T6-12_192	AGTAACTCTCTGAGAAGCAGCCATAGGTC
2D-0558280-T6-12_256	ACTGTGGCTTCGAGGATGCGCTGAGGTC
2D-0558280-T6-12_320	CTTCCAGTGGATGTCCTGGCTCCGGCATC
2D-0558280-T6-12_384	TGCGACGTCCTGGGGCCCTTGGAAGAGC
2D-0558280-T6-12_576	GCGTTGCACGGGAAGCATGGTGGGTGAC
2D-0558280-T6-12_640	TCCTGTACACACGCTGCGAACGCGGTGTC
2D-0558280-T6-12_704	AACCTGGAGTAGTTTGCGGTTTCCACTTT
2D-0558280-T6-12_896	GGACCAAGCTAGGGTGCCCCCGTGACAG
2D-0558280-T6-12_1152	TGGCGGGAGGCGGACGTGACAAGGAGA
2D-0558280-T6-12_1216	AACAAGGAACACATTGTAAACAACTCTAT
2D-0558280-T6-12_1408	TACGCTTCCTTCCTTCAAAACCACATAGAT
2D-0558280-T6-12_1472	GCCCCTTCcctccgtcccctcctccctcgtctcgtcc
2D-0558280-T6-12_1536	CTTCTCGATGCAATGCTTCCTCGATTCTCT
2D-0558280-T6-12_1580	CGGCGGTTGATCGGCGGCAATGGCCCTG
6A-1499070-T6-13_256	GCCGCTATACGTGCGGCGCTTTCTCTCTCT
6A-1499070-T6-13_320	CGTGGCGACGTCTCCTTCGCGGGATAAAA
6A-1499070-T6-13_576	TTTGTCGATGGTGTGGTGCTGCTGGGAGA
6A-1499070-T6-13_636	GTAAAGTAAAGTAGTAGtttttttttcttttttttat
6A-1499070-T6-13_1263	tgacttatacacccggacggagggagtaCTTATTA
6A-1499070-T6-13_1458	GCGGTGAATCGATCGCCGGTTCAGGAAA
6A-1499070-T6-13_1580	ATTGATGTGAATCTTGGTTGGCATGCCGG
6B-1608010-T6-13_0	GCCAGATCCGCCGATCCTGCAATAAATCCA
6B-1608010-T6-13_64	GTCCACAGAAACCAAACTCATGATCGCGC
6B-1608010-T6-13_320	GGCACATCCATCCTTGCGTTGCGCATATCC

										6B-1608010-T6-13_384	AAAACCTCTTCTCGTCGCGCTGCCATGCC
										6B-1608010-T6-13_512	GCCGCTCGCTGCACCCACGAGCGGATCG
										6B-1608010-T6-13_640	AAACCCAGCCCCCGAGCGATCCCTCCTC
										6B-1608010-T6-13_960	ATAATGGCGACGACCCAGCACCCGGCAG
										6B-1608010-T6-13_1088	TATCTCCGCGGACCTCCGCCGTGGCGACG
										6B-1608010-T6-13_1280	CGGGCCCGGCCGTCAGCTGTACGGTGCG
										6B-1608010-T6-13_1408	TGGGAGATTGATTATGAGTTCCGAGCATA
										6B-1608010-T6-13_1472	TTTTCGACGTTTTGCTTTGGAGACGAGTC
										6D-1700820-T6-13_128	CACGCACGCACCCGGCAGGAGCTACAGG
										6D-1700820-T6-13_256	GACTCCGCCGTGGCGACGTCTCCTTCGCG
										6D-1700820-T6-13_448	CCGTACGGTACGGAGTACGGAGTCTGAA
										6D-1700820-T6-13_576	TATCAGTTCCGAGCATAGGGGTAAAGTAA
										6D-1700820-T6-13_768	AGAGAATCTTCACCCGCCAGGGAATCTCG
										6D-1700820-T6-13_832	TTATTGAGGTGCAAAGAAGGAAGCGACT
										6D-1700820-T6-13_896	GTTTTTTTCCTTATATTACTCCGGAGAACC
										6D-1700820-T6-13_960	GTGTGCTTTTTGAAAAGCCCAAAATTACC
										6D-1700820-T6-13_1088	CGCCGGTTTAGGAAAAAAAAGCTGAGGT
										6D-1700820-T6-13_1152	TTGCGCATTCAGTGCCGAGGATCTTATCTT
										6D-1700820-T6-13_1216	GCCGGCTGCTCATTCGGTGTGGGTATCTG
										6D-1700820-T6-13_1280	AAAGGTTGCATTTGATATTATTATTCAGCT
										6D-1700820-T6-13_1344	GCCTCTCTTTTTTCCTTTGTTGTGGTGAAA
										6D-1700820-T6-13_1408	GCAAGAACGCTAAATTTGCCGAGGGTGC
										6D-1700820-T6-13_1472	GGTCCCGAAGAGGAGACCCCATCAGAGT
										6A-1503230-T6-14_128	GTCACTCCCTCCCTTTGCCCACTTGGGGC
										6A-1503230-T6-14_256	GCTGCCCAAGTGGGAGAGAACATTGGCA
										6A-1503230-T6-14_320	AAATTCCAAGTTTGAGCTGGGTTTTTTTT
										6A-1503230-T6-14_384	CCCACGGAGGAAACCGATGTGTTTCCTAG
										6A-1503230-T6-14_448	TCGCGAGGCCTGGTTTAACCGTGACAGA
										6A-1503230-T6-14_512	CATGATGTTGGTTTGCTCGAGGGAAAGTA
										6A-1503230-T6-14_576	TGACAACATAGGTGGCGTGGAGATTTTTA
										6A-1503230-T6-14_640	GTTATATTTTCTTATGGAGAAGAGCCCAAT
										6A-1503230-T6-14_704	TTGGTTATATAGAAATGTCATAGTTATTCGT
										6A-1503230-T6-14_768	TATCATGATGATGTATGATTTTATTAGTTGT
										6A-1503230-T6-14_832	TGACATGTAAATTGATAACTGTGCAAGTT
										6A-1503230-T6-14_896	TGATGTTTAGTGTACTTTTTGTTTACGAGA
										6A-1503230-T6-14_960	TCATTACCTGATTCAATCGATTGTGAAAAC
										6A-1503230-T6-14_1024	ACAGAAATTTGATACAACACAACTTGATG
										6A-1503230-T6-14_1088	TGTATTTTTTTCATGAGTGCAATGCTCGTA
										6A-1503230-T6-14_1152	GGACCATACATCATATAGAAAACACTTGA
										6A-1503230-T6-14_1216	CGTCGCAAATTAGAAAATGAAATGTGCAT
										6A-1503230-T6-14_1280	TACTACTTGCTTGACAAAATCATTTTGTCG
										6A-1503230-T6-14_1408	TACCCATAGCAAGAAGCATTGTGGATGTC
										6B-1592660-T6-14_128	CTCGACCGCGTCACTCCACTTGGGCTCCC
										6B-1592660-T6-14_256	TGCCCAAGTGGGAGAGAACATTAGCAAT
										6B-1592660-T6-14_320	ATTCCAAGTTTGCGAAGCTGGGTTTTTTT
										6B-1592660-T6-14_384	CCATGGAGGGAACCGATGTGTTTCCTAGT
										6B-1592660-T6-14_448	CGTGAGGCCTGGTTAAACCGTGATAGAG
										6B-1592660-T6-14_512	ATGTTGTTGGTTTGCTCGAGAGAAAGTAA
										6B-1592660-T6-14_576	GACAACATAGTGGCGTGGAGACTTTTACT
										6B-1592660-T6-14_640	TATATTTTCTTATGGAGAAGAGCCCAATGA
										6B-1592660-T6-14_704	GGTTATATAGAAATGTCATAGTTATTCGCG
										6B-1592660-T6-14_768	TCATGATGCTGTATGATTTTATTAGTTGTTG
										6B-1592660-T6-14_832	ACATGTAAATTGATAACTGTGCAAGTTGAT
										6B-1592660-T6-14_896	ATGTTTAGTGTACTTTTTGTTTACGAGGAC
										6B-1592660-T6-14_960	ATGACCATGCATGATTAAAACCAATATTTT
										6B-1592660-T6-14_1024	TTGATACAACAAAACTTGATGCAAGTGAA
										6B-1592660-T6-14_1088	TTTGTGAGTGCAATGCTCATAGTAGAGAC
										6B-1592660-T6-14_1152	ATCATATAAAAAACACTTGACACGATGTCA
										6B-1592660-T6-14_1216	ATAGAAAATGAAATGTGCATATCTTTACTT
										6B-1592660-T6-14_1280	ATCGACAAAATTATTTTGTCGAATATATATG
										6B-1592660-T6-14_1408	CAAGAAGCATTGTGGATGTCATGCATGCC
										6D-1705140-T6-14_0	ACTTTTTGTTGGTAGAATAGACCCTGGCT
										6D-1705140-T6-14_192	GCTTATTTAGTTTTTTTTTTTAGAAAGATC
										6D-1705140-T6-14_384	GGCTTGACGTTGCCAACAGGAATTTTTGG
										6D-1705140-T6-14_512	AATGCTTCTTGCTATGGGTACATGGGCTTG
										6D-1705140-T6-14_576	TTTAGATGGCTATACTGTTTCCTTGATGGT
										6D-1705140-T6-14_640	TAGTATGTGGTATGATTCAGACAGCCGTGT
										6D-1705140-T6-14_704	CAATGAATTAACTACACGAAATATAAAAG
										6D-1705140-T6-14_768	GGTCCATGGAGGAGAAGGTCTCACATGG
										6D-1705140-T6-14_832	ACACATTGTAAAACCCATTACGCTATTATG
										6D-1705140-T6-14_896	TACCTAATACTTCTTCAAACACAAAGTCAT
										6D-1705140-T6-14_1024	TTTACATGTCACTTTATTCTCGAGGAAACC
										6D-1705140-T6-14_1088	GATAATGTCTGTCTCTATCACCGTTTAATCT
										6D-1705140-T6-14_1152	ATACCCAAGAATTGGTCCCCTCCTTTGGG
										6D-1705140-T6-14_1216	AGATAACTCATACGTAACTACGGTAAAAA
										6D-1705140-T6-14_1280	GTTGTCACCAGCATCAACTTGCACAGTTA
										6D-1705140-T6-14_1344	CATCATGTTGGAACAACCATACATGGTCAT
										6D-1705140-T6-14_1408	CTCACGAATGACAATGATGTAGCATATGCC
										6D-1705140-T6-14_1472	CCATGGGTTAAACTATAAAACCACTCCCTT
										6A-1548640-T6-15_0	TGATATTGTGATAAATAGGAACCTAAGTCC
										6A-1548640-T6-15_64	ATGCCTCTCACCCAAGTGAATTAGGCTCG
										6A-1548640-T6-15_128	TTTAGTTCAAGAAGGAATGACCCCTCCCC
										6A-1548640-T6-15_192	ACTTTGATAGTGATGTGCAAATAAGCCAA
										6A-1548640-T6-15_256	ATAAATGAACGATAAACCAAGGGATTCTC
										6A-1548640-T6-15_320	TATCTCCCAAGACATCACATTCAAAACGA
										6A-1548640-T6-15_384	CATCATCGAAGAAACTATACCCTCAACAA
										6A-1548640-T6-15_448	AGTAACCAAACACCAGATTAAGGGTTTTT
										6A-1548640-T6-15_512	GTATACCGCCCCAGGATATACCAAGAGTG
										6A-1548640-T6-15_576	TGTAATAAGCGTGCGCAGGATTGCCGACG
										6A-1548640-T6-15_640	TATCGCACGTCAGCGTGAATTAGTCTGCC
										6A-1548640-T6-15_896	ACAAACTGAAACCGTTGCAGCCATGCTCC
										6A-1548640-T6-15_960	TTGGCTTTGAAGATTCTACACTAGTCCTAC
										6A-1548640-T6-15_1024	ACATTTTATTTATGTTGTCAGCCAAATATTT
										6A-1548640-T6-15_1088	CATGAAGGTTCCTTGTACCGCTAAGTAAT
										6A-1548640-T6-15_1152	CACATGTTTGTCACATCATGATCGTATGTG
										6A-1548640-T6-15_1344	TTACTTAGCTGTCCTGCTCATTTTCCTACCT
										6A-1548640-T6-15_1408	CCATGATTCCGTCAGGTGCAATAATACACA
										6A-1548640-T6-15_1472	CAGCCGGTTAAGAGATGAGAGTTGAGAG
										6B-1648900-T6-15_189	TGATTGAACGTCTCTCAACTCTCATCTCTT
										6B-1648900-T6-15_441	TGTCCAGAATCGAATTTCTACACGGTCGA
										6B-1648900-T6-15_504	GCCGCTAACAACATAAAATGTGTAAAAAG
										6B-1648900-T6-15_567	GAATTGGTGCCTTCTAATAGTACGTCATGA
										6B-1648900-T6-15_630	TGTAAGAAGATGACAGTTTGCGTTGTGGG
										6B-1648900-T6-15_882	CCTGGAAAGCATATGTCAAACATGACTCG
										6B-1648900-T6-15_1008	TGCTAATCAGTTTCAGGCAACTCAAAACA
										6B-1648900-T6-15_1071	CAAATTAGTCTCGAGATGCGGCAGAAACG
										6B-1648900-T6-15_1134	TCTACTCATGGTTTTGGGGGGAAAACCCT
										6B-1648900-T6-15_1197	CAATGCACATATATCATGATGTTGTTGAGG
										6B-1648900-T6-15_1260	TTTCGTTTATGTGCTTCCTCTTCGTTTTGA
										6B-1648900-T6-15_1323	CAAGGAGAATCCCTTGGTTTATCATTCATT
										6A-1548650-T6-16_128	TATTTATAACCGAGGCGCGCTCGCATGCA
										6A-1548650-T6-16_192	TTGGGATCCGATCTTACTCGATTCCTCAAG
										6A-1548650-T6-16_256	CAACCTGGCCTTTCGGCATTGTAGAAAAG
										6A-1548650-T6-16_320	ATAAACTACTCCTGCATTGCACCCTATGGA
										6A-1548650-T6-16_384	CTTAATCTGGCCCCCACCTTTGGAGAGCG
										6A-1548650-T6-16_640	CTTGCTGCTGTCAGCAGCTGAGAATCAAG
										6A-1548650-T6-16_704	GTGGGATGCTGTTCACATGGTTTGAAGTA
										6A-1548650-T6-16_768	TTTAGAAAATTCAGTTGGTAGCAAAATTT
										6A-1548650-T6-16_832	GGAAAAAAGGTGAATTCCGCCTGGCGGT
										6A-1548650-T6-16_896	AGGTAATATATTTGAATCATGATTGAGTGT
										6A-1548650-T6-16_1088	ATTTTAAGTTAATAAAAGTGCCCTTTAGCG
										6A-1548650-T6-16_1152	TTTGGAATGGTGGGAGTAATTTTTTTATG
										6A-1548650-T6-16_1216	AATCATGCATGTGAACCCTTTATCTTGGAG
										6A-1548650-T6-16_1280	TAGCCTGTGAGTCGTGGTGTCATATGTGA
										6A-1548650-T6-16_1344	TAGATGAAGAGTGTTTGTGAGGATGATGA
										6A-1548650-T6-16_1536	CAGCGACCGAATGGGCATGATGACCTTG
										6B-1648910-T6-16_0	CCCCCCAAATTAATGAAAACGATGTGGAT
										6B-1648910-T6-16_64	TGGCACAAGATGCAATGGTTGTGTGCAA
										6B-1648910-T6-16_128	AACAGTTTGTTGGGAAGATTCGGTTGATC
										6B-1648910-T6-16_320	ACCTCCAAGGAGCCCCAAGGGATGTGAC
										6B-1648910-T6-16_384	CAACTATTTACTTCATCATCCTCACAAAAA
										6B-1648910-T6-16_448	GCCGCATCTTGCTCAGAGATGACACCACA
										6B-1648910-T6-16_512	CGTTCGATTTCCTCCAAGATCAAGGGTTC
										6B-1648910-T6-16_576	AAATTGAAGATTAAAAAATTATTCCCAACG
										6B-1648910-T6-16_704	TTGGCCGTACATGTCCTTGAAGGGGTGAA
										6B-1648910-T6-16_768	TGATTCAAATATATAACCACCACATTCAAC
										6B-1648910-T6-16_832	CGGAATTCACCCTTTTTTACCGAAGCAAA
										6B-1648910-T6-16_896	TACTAACTGAATTTCGTAAAAATGTGCTTA
										6B-1648910-T6-16_960	TGAACAGTATCCTGCATTAATTCTGCCAAA
										6B-1648910-T6-16_1152	ACCACCCGTGCGGGGCCCCCAGAAGGCA
										6B-1648910-T6-16_1216	GAATCCCTTAACAATCGGTTAATTAACTTT
										6B-1648910-T6-16_1280	GGGCCAGATTAAGCCAAAACTGGAGCGA
										6B-1648910-T6-16_1472	AGCTATAGCTGTGAAACTGGCTAGAATCT
										6D-1727340-T6-16_0	CATATATAGCTACCCCCCAAATTAATGAAA
										6D-1727340-T6-16_64	GTTCACACTACTTGACATAATATGCAATGG
										6D-1727340-T6-16_128	TTCTTTTTCAAAAACAGTTTGTTGGAAAG
										6D-1727340-T6-16_320	TTTTCCCGACCCAACTCCAAGGAGCCCCA
										6D-1727340-T6-16_384	GGTTTCAACTATTTACTCCATCATCATCACA

6D-1727340-T6-16_512	GCGCGCTCGATTTCCTCCAAGATCAAGGG
6D-1727340-T6-16_576	ACCAAATTGAAATCTCATAAAAAAAAATT
6D-1727340-T6-16_704	ACCGAGGTAAGTATTTGGCCGTACATGTC
6D-1727340-T6-16_768	TGAACACTCAATCATGATTCAAATATATAA
6D-1727340-T6-16_832	ATTTTACCGCTAGGTGGAATTCACCTTTTT
6D-1727340-T6-16_896	ACTCAAATTTTGCTACTAACTGAATTTCGT
6D-1727340-T6-16_960	ACTTCAAACCATGTGAACAGTATCCCACAT
6D-1727340-T6-16_1216	GGATCTAGTCTAGAATCCCTTAACAATCGG
6D-1727340-T6-16_1280	CTCCAAAGGAGGGGGCCAGATTAAGCCA
6D-1727340-T6-16_1472	TGGTTAACCTATAGCTGTGAAACTTGCTAG
6D-1727340-T6-16_1536	GTCAACTCTGCCTGCCCCCCCTGCGAGCT
6A-1548630-T6-17_353	AATACCAAATGCATTAAGCTGAACAAAAC
6A-1548630-T6-17_548	CACTACCTCCCAAGACAATGCATTCAAGG
6A-1548630-T6-17_613	AAAACTTTCAGTTGAGTGGCCTGACACCA
6A-1548630-T6-17_808	AAGTGCACAATGGTTTTCCACCATATCTGA
6A-1548630-T6-17_873	ACGCAGATAGCCTCACTATCATCAGTGCA
6A-1548630-T6-17_938	AAAGATACACGAGGTCATTACTTGTCCCC
6A-1548630-T6-17_1133	TAAGAACAAGCTGTGGACCAGGCTGGCT
6A-1548630-T6-17_1198	GACGATACTGTATAGCCAACAAGTGAGCA
6A-1548630-T6-17_1393	GAGATTAGTGCGTTATTTAATCGCAGCTG
6A-1548630-T6-17_1458	CTTTCCTGGGTCGACAATGCTAATTGCAC
6A-1548630-T6-17_1523	TATCCAAGTGTCAACTCTGCTAATAGCATA
6A-1548630-T6-17_1580	ACCAAACCGATGCGAGATACAAAGCATCA
6B-1648890-T6-17_0	CTTTCCTGACAACTAAACGCAACTAATGG
6B-1648890-T6-17_64	GTAGTAATGGACTAATGGTGATGTGGTAG
6B-1648890-T6-17_128	ATTTATAGCGAGACGCGCTCACATTCATTT
6B-1648890-T6-17_192	CACACCACAACTCTTAGCTGTAGGTTAAG
6B-1648890-T6-17_384	CGATGCATGTCCAGAATCGAATTTCTTGT
6B-1648890-T6-17_448	AAAAGTTTTACCTTGTCCCAGTGTGCTAG
6B-1648890-T6-17_512	CTGGCTATACATAAAGTGAACAAAATACC
6B-1648890-T6-17_576	TTTAAAAAAAGTATCTAATTAAGGACAGT
6B-1648890-T6-17_768	ACTTGCAAATTTTGCAGCGATCCAACGGA
6B-1648890-T6-17_896	AGTGTATGTCAAACCTGACACTGACACCT
6B-1648890-T6-17_960	GTTAATTCTTGCCTACGTAGACTAGCTATA
6B-1648890-T6-17_1152	TTCAATTGCTCGATCAGACAACATCAACG
6B-1648890-T6-17_1216	TTCTTCACTGGTTGGTCCAACTAGTGTATG
6B-1648890-T6-17_1408	TTGTTTTGCAGGTTTTTTGGCTTTGAACT
6B-1648890-T6-17_1472	CATTTTGATAGTCATTATATTTTTATGTATA
6B-1648890-T6-17_1536	CACATTTTTAAAAATATTATATGCTTGAATC
6B-1648890-T6-17_1580	GGGCATCCGTTCATACTTATTATGTACATC
6D-1727350-T6-17_0	CTTTCCTTGACAACTAAAAGCAGCTAATG
6D-1727350-T6-17_66	ATAATGGTGATGTGGTAGTATGGCTTATGC
6D-1727350-T6-17_132	CGCGTTCACATTCATTTGCTACAGCAGAG
6D-1727350-T6-17_198	AGCTGTAGGTTAAGTGCAATTAGCATTGT
6D-1727350-T6-17_396	CTTGTGCACACTACAGAAAGGAAAGTTTT
6D-1727350-T6-17_462	TGTTTATTTGCTCACTTGTTGACTATACAG
6D-1727350-T6-17_522	GATCTTCAAAGCCAGCCAGCCTGGTCGG
6D-1727350-T6-17_1061	GTCAAACCTGACACTGACAGCATGCAAG
6D-1727350-T6-17_1126	TTCCCTACGCACAGACTAGCTAGATAATTT
6D-1727350-T6-17_1321	CCTTCAATTGCTCGATCAGACAACATCAA
6D-1727350-T6-17_1386	CTTTACTAGTTAATCCAACTAGTGTATGTT
6D-1727350-T6-17_1516	TGGTTGGGTTCATTGTTTAGCTCTAATTCA
6D-1727350-T6-17_1580	CTTCCACAATGTTGCTCTTCCCAAAGCAG
6A-1548600-T6-18_0	GGCTAGCCATCCAATGTCTTGTGTGCAGC
6A-1548600-T6-18_64	GTACTTAGCAACACCAACCACTGCCTTGT
6A-1548600-T6-18_128	AGAGAGTCAATGAGCGTCGTATAATGAAA
6A-1548600-T6-18_192	ATACAGCACATACATAATATAGCCATATAG
6A-1548600-T6-18_256	CAACCCTGATAAGTTCAAAGGACAGAC
6A-1548600-T6-18_320	GTCTGAATATATCTAAGTTAGACTTAGACC
6A-1548600-T6-18_384	CTGCATAAACATAACCAACCGGAAAACAA
6A-1548600-T6-18_448	TCGTGGAAATGGAAACGTTCAGGACATA
6A-1548600-T6-18_512	GCTATGCCTGAAATAGCTTATGGAAGCTC
6A-1548600-T6-18_576	CACCATACGACATAAGTCTAGACGATGAC
6A-1548600-T6-18_640	AGCCGGGACTCTTCCTTTTGTTACCTGTAT
6A-1548600-T6-18_704	TGTATGCTGTCAGTGTCAAGGTTGACATG
6A-1548600-T6-18_768	AGAGCATCGACCGCGTGCAATGTACACGT
6A-1548600-T6-18_832	GTGTGTACGTATGCGATCGCGGCCGTTGC
6A-1548600-T6-18_896	TTGAGCCATGCATGCTTCTGCTGGCCGAC
6A-1548600-T6-18_960	AGCAGGCAAGTATCAAACTATCGATTCAC
6A-1548600-T6-18_1024	ATAAATACTCCCTCTGTCCCATAATATAAGA
6A-1548600-T6-18_1088	GAGTAATAAGTACTAGGCTGCAGCTAAGC
6A-1548600-T6-18_1152	GACAGAGCACATGCACGGAATCGTGCAG
6A-1548600-T6-18_1216	GTGAGACCGCAAGAGAACAGAAGCTTGC
6A-1548600-T6-18_1408	CCTCTCTCGACTGCGTTGCGCGCCCGTTG
6A-1548600-T6-18_1472	CAGATGCAATAATAGTCTAATTCTTCTGTC
6A-1548600-T6-18_1536	GCAGCTTGATCCCAATGTCAACTCCTGCA
6A-1548600-T6-18_1580	CGATGGATCCCGGATCCAAAGCACCAAA
6B-1648870-T6-18_64	GCTACTTGCAGTGGCCGGCAACTAGGAG
6B-1648870-T6-18_128	GGATCGATTGGGTATTTATAGTAGGATACA
6B-1648870-T6-18_192	ATGCATCTCTCTTTTTCTTTTTAGGAGTGA
6B-1648870-T6-18_448	GCACTGCTCCGGCCGTACATGGGCCCCTG
6B-1648870-T6-18_512	CCTGCTGGCCACTACCCGACCTGCAGCTG
6B-1648870-T6-18_576	GTTTTGATCTCCTCTTAATTTCTGTAAGAT
6B-1648870-T6-18_640	TGTGCTGCACTGTGTTGCTAGCTTAGCTG
6B-1648870-T6-18_704	GTACTAGGCTAGCTACCTGCTAATGCAAA
6B-1648870-T6-18_768	CAGGGCACAGGTCGGCGATACGATTTCTT
6B-1648870-T6-18_832	GAACGGCCACGGCTACGACTATGGTGGC
6B-1648870-T6-18_896	AAATGGCGTGTACATTGCACACAGTCGAT
6B-1648870-T6-18_1024	TACATCTTTCAATTTCCCTTGTTGCATGCC
6B-1648870-T6-18_1088	TTTGATATTATTGATTGTGTTGGAAATAAC
6B-1648870-T6-18_1152	TCTCTATTGATTCATAAATGGTTCCCCGCA
6B-1648870-T6-18_1216	CAGCGGTGATGCATTCAGAAAGGAAAGG
6B-1648870-T6-18_1280	TAGCCAGTTCGTGAGTTTTCACCCAGAAG
6D-1727380-T6-18_0	ACCTACTGATCTAGCTTGATCGAGCTAGCT
6D-1727380-T6-18_64	CTTAATGACTTGCAGTGGTGAGGTGACTA
6D-1727380-T6-18_128	CCCGGATCGATCGGGTATTTATACaaggaag
6D-1727380-T6-18_192	TTGCATCTTCTCTCTTGATCAGCTGATGGG
6D-1727380-T6-18_384	AGGATCAGATCAGAATATGCACTGCCGCG
6D-1727380-T6-18_448	CCTCTGCTGGCGACTGCCCGACCTGCAG
6D-1727380-T6-18_512	ACTTTCAGTTCAGATCTCCTCTTAATTCGT
6D-1727380-T6-18_576	GATTCCGTGCATGTGCTCCGTTTGCTTCTG
6D-1727380-T6-18_640	GCTGCAGCTTAATTAATTTAACTGCAGTG
6D-1727380-T6-18_704	GACCACCAGGTCGGCCATAGTTACAAAG
6D-1727380-T6-18_832	TCTTCCTCCAACCCAAGGGACGTGTACGT
6D-1727380-T6-18_960	AGGTAGTGACCTCACGTACATCTTATCTTC
6D-1727380-T6-18_1216	TAACTTATTTTAAATTTACATTACTAAGATT
6D-1727380-T6-18_1280	AGAGAGACGATGTGGGTGTTGTACTTTTT
6D-1727380-T6-18_1344	TGCCACATCAAGGGGATACAAATGAACA
6D-1727380-T6-18_1408	TTAAAACGAGAATGACCCGTAGGAGTCA
6D-1727380-T6-18_1472	ACAACTACAACCATCGTTGACAACACCCT
6D-1727380-T6-18_1536	GAAACTGTGCATGAACGTCGTCGACATGG
6D-1727380-T6-18_1580	GACAAATGATGGGTTTTCACCCCGAAAG
6A-1511800-T6-19_64	CGGCACCGCGAGATGGATAAATGAGTCA
6A-1511800-T6-19_128	TGGTCTCATAGCATCTGATTTGGTGAGATT
6A-1511800-T6-19_192	TGGCAAGATTGGAAGAAAAAGATGCGCA
6A-1511800-T6-19_256	TTTGAagagagagagagagagagagagGCCTA
6A-1511800-T6-19_320	GGATTACAAATCAGACCTAATGCCTCAATT
6A-1511800-T6-19_448	AAAGGTGAATTTGGCACTGCTTTTGGCCT
6A-1511800-T6-19_512	tgagtctctcccttgttttgggagggagggagtaTTT
6A-1511800-T6-19_576	ACAATGCCATTGGACTAGTACAGAGGTGT
6A-1511800-T6-19_640	GTCGATACATAGTTTTATTTGAATTGGCCA
6A-1511800-T6-19_832	CATTTTATTCACTTCTGTCACTTTTTGCTGT
6A-1511800-T6-19_1152	AGCACATGCATGTACATCCCATAAATATCA
6A-1511800-T6-19_1216	TTGTACTTGTACTTACTTTGTACTACTGCTG
6A-1511800-T6-19_1280	ACATCTATTCTTCTGTTCTCACAAGTTCTTT
6A-1511800-T6-19_1344	AATTTCCTCCACTCGTGTACAACTTCCCAT
6A-1511800-T6-19_1408	TTTGCTTAATTATTTATTTAAACAAATAATA
6A-1511800-T6-19_1580	ctctttgtgtgtgtgtgatgtgatgtgtgtgttgtgtgCA
6B-1622420-T6-19_0	CGCCGCGATTCTAGCTTGGAACTTGGGCT
6B-1622420-T6-19_192	TTAACTAGTTAACTTATTTAGGTGGTCGAA
6B-1622420-T6-19_320	AAATTTAAGCAGCGGCAGGAGTAATTAA
6B-1622420-T6-19_384	TCTCTGTGCTTCCGTTCCAAACAAGATCTA
6B-1622420-T6-19_448	TCGACATTTCTACCCATTAGCCATCCAGAC
6B-1622420-T6-19_512	CGACTATGCAATTACGTAGCTTGCAAAGT
6B-1622420-T6-19_576	CAAAACAACATGCTGCAAATTTTATTGTG
6B-1622420-T6-19_640	ATATGTTTCTCCATGTTGAATTTGTTGACA
6B-1622420-T6-19_704	GTCGCTGCAGTACAACAAGAGGTCCGAG
6B-1622420-T6-19_768	AACCAAAGGCTACAAGGGTGCATTATTTG
6B-1622420-T6-19_832	GAGAAGCCATTTGACTTGTGGACAGCTA
6B-1622420-T6-19_896	CCGGTGCAGATGAATCATCAACCTAGCAC
6B-1622420-T6-19_960	TATTTAGGTTTCATATCTAGGCTCCCTTTG
6B-1622420-T6-19_1024	ATAAGTTTGTGCTGCAGACTGGTGAAGTG
6B-1622420-T6-19_1088	TTGAATTGCCCAATTTTATTCAAAACACAA
6B-1622420-T6-19_1152	AGTGTTGCCATCAAAATATATTCATTGAGA
6B-1622420-T6-19_1216	ATATGTACAGATAATGAGCACGTTACACAA
6B-1622420-T6-19_1280	ATCTATTTAATTATTTCTTCCATTCCATGGA
6B-1622420-T6-19_1344	CGACAAATGGAAGTCACGGCTCAAACATA
6B-1622420-T6-19_1536	CGTTTTGCATCACCAGATTCTGCTTGCACA

6D-1685000-T6-19_192	TGATTAACCTAATTACGATCGAAAGTCAG
6D-1685000-T6-19_320	GGCAGGAGTCATTAACTGAGCTAATTATT
6D-1685000-T6-19_512	AGCATTTCTAGTGCAGTTAGCCTTCCTTGT
6D-1685000-T6-19_576	AGCTGTTGCAAATTTTATCCCAAAAAGAA
6D-1685000-T6-19_704	AACATGCCGACATTGAAAACGGCAGTTA
6D-1685000-T6-19_768	ACATAGTTCACGTGACTCTTAGCCATAAC
6D-1685000-T6-19_832	GCTTTATCAATCAGAACGTAAGAGAAGC
6D-1685000-T6-19_896	GTGCAGATGAATCATCAACAACCTAGCAC
6D-1685000-T6-19_960	GTTTCATATCTAGGCTCCCTTTGGAACAAT
6D-1685000-T6-19_1024	ATTTGTAATAAGTTTAGACTGGTGAAGTG
6D-1685000-T6-19_1088	GAACTGCCCAATTTTATTCAAAATACAAAA
6D-1685000-T6-19_1152	TGTTGCCATCAAAATATATTCATTCAGAGA
6D-1685000-T6-19_1216	ATGTACAGATAATGAGCAGGTTACACAAG
6D-1685000-T6-19_1280	TAATTTAATTATTTCTTCCATTCCATGGAAG
6D-1685000-T6-19_1344	ACAAATGGAAGTCACGGCTCAAACATAGT
6D-1685000-T6-19_1536	GCAGAATGCTCAACGTTTTGCATCACCAG
4A-0994730-T6-20_1280	AGTACGTAGGTTAATTTCGTTTCTTTCTTT
4A-0994730-T6-20_1344	CGGTCACTACGCAGGCGTTTGAGGGGCC
4A-0994730-T6-20_1408	CAGGCAAATCCCGTCGTGCTCATGTGAAC
4A-0994730-T6-20_1472	GGCTCGACTGCCACTCAGGACGCGATGC
4A-0994730-T6-20_1580	CTCACTCGTCCGGCCGGACCCTTTGGAG
5B-1356990-T6-20_128	TGGAGATCGTTGACCATGAGCTGGAGCT
5B-1356990-T6-20_256	TCTTGAGGGTCTCCAAAGGGTCCGGCCG
5B-1356990-T6-20_576	ttttttatcaaccattgtacatgcccTAATTAACTGA
5B-1356990-T6-20_640	TTCGGCTGAGTTCACATGGTGCAACCGTT
5B-1356990-T6-20_704	TTAGTCGCCTAAACAGCTATTGCAAATGTT
5B-1356990-T6-20_896	TACACGGCTGCATTATTTGCTTGACTTGG
5B-1356990-T6-20_1024	CGATGCGAGCGACAAAGGTGTCGAGTTG
5B-1356990-T6-20_1088	GGTCGGGTTATGTAAGTGGGACGTCCTG
5B-1356990-T6-20_1216	TGACCGGTCTTAAAAATTCAACCCAGGCG
5B-1356990-T6-20_1472	ccggggctatagcccggttctgcactagtgATATGG
5B-1356990-T6-20_1536	CCTCGGTACACATCTGAGCTCTTTGTGTC
5D-1438330-T6-20_64	TTGATGGATGGGGCTGGGAGAGATGGG
5D-1438330-T6-20_320	GGAGTAATTAACTGAGCTAATTCTATGCG
5D-1438330-T6-20_384	GGGAGGGCATTTCGGCCGAGTTCACATG
5D-1438330-T6-20_448	ACAGCTGCAATTAGTTGCAAGGTGTTCGC
5D-1438330-T6-20_512	CAAGAAAATGCTGTTGCAAAATTGCAAG
5D-1438330-T6-20_640	CTCTTAGCCCTAACCAAAGGTTACACGGC
5D-1438330-T6-20_832	AATGAGAGTCGGCAAAGGAGGACGTCCT
5D-1438330-T6-20_896	TAAGAGCACCTACAGCTACAGCCGGGTG
5D-1438330-T6-20_1024	GCCGACCGCAACCCTCGTATCCAGCCCAA
5D-1438330-T6-20_1280	TCTGGTTCTATAATAACAAAACAGCTCCTA
5D-1438330-T6-20_1344	AATTAAAACTGAGGATAATTTAAACACAG
5D-1438330-T6-20_1408	TTTGTTTCTTTCTCCGATTTCTTCATTTTTC
5D-1438330-T6-20_1472	TTTTCTATAGAATCGGAATAGTTTATATACA
5D-1438330-T6-20_1536	TGTTTTGTACATGTTGAACCTTTTTCAATA
5D-1438330-T6-20_1580	TTGTCAGATATATTGTTTTGATGTATACTCC
6A-1504680-T6-21_0	AAGTGATGGAAACGTTTCGTGGCCCCAC
6A-1504680-T6-21_64	TCATAATTTAGTCCTTCTCGACTCAAAACT
6A-1504680-T6-21_128	GTAATTTGAAAAGCTTTCGACATAATACG
6A-1504680-T6-21_192	GAAACTATTCATCATGTTCATATCTTGCCCT
6A-1504680-T6-21_256	TTCCAATAACCGCGGCCAACCGGTGTTCG
6A-1504680-T6-21_320	CAACTTAGAGCTCAAATCCATGTGGGGCG
6A-1504680-T6-21_384	ATGATCATATTTGTGACAAACAAACGATTT
6A-1504680-T6-21_448	GACATGTGCAACCATTTGGAGGCAATAGC
6A-1504680-T6-21_512	ATACTACGGAATGTGAGACCTGAATTGTC
6A-1504680-T6-21_832	gtaaaaaaaaaacagagggagtaTGACTACGTA
6A-1504680-T6-21_896	CCAATACAGAAGAAACAGGCACCCTCCT
6A-1504680-T6-21_960	TTAAGAGGATTTAGCAAACCACTATCAATT
6A-1504680-T6-21_1024	TTGGTTATCTTTTTGATAATTTATTCAACCG
6A-1504680-T6-21_1088	TTTTAGTCTTCAGAAAAAAATAGCCAACTT
6A-1504680-T6-21_1152	ATTGTAGGTGCTTAAGGTTACAATGTCAG
6A-1504680-T6-21_1216	TAGTGATTATGATAATAATCTTATTCGTTTA
6A-1504680-T6-21_1280	ACTAACTTTTTTGTCGCAGCAAAATGTAG
6A-1504680-T6-21_1344	AATCTCAAAACTGCTGCCTGAGCCCTAGT
6B-1596170-T6-21_128	GTGTGCGATAGAAATTTTTATTTGGCGCT
6B-1596170-T6-21_192	ATAGGCTCGATGCCAAACCCTATAGGTGG
6B-1596170-T6-21_256	AATGTTTTGTGTATGTACCCCACGTGACAC
6B-1596170-T6-21_320	AATTTAATCCTGCTGGACTCAAAACTTCCT
6B-1596170-T6-21_384	CTTGAAAAGCTTTCAAATGTAATTTGGAA
6B-1596170-T6-21_448	TGCTTATGTAATTACGATGAAACTATTCAC
6B-1596170-T6-21_512	TGGCGGAATATGTATGTAACTTCCAACAA
6B-1596170-T6-21_576	CTATCTCACGGGGATTGATAGACAACTTA
6B-1596170-T6-21_640	GCACTATGTAGAAATTGTCCTAATAATCAT
6B-1596170-T6-21_704	tagattatcttcgtagatacttattggatctccacgtgg
6B-1596170-T6-21_768	agactctttgattggtgcaaccatttggagataatagc
6B-1596170-T6-21_832	gactgtcTAACACCATGGTTTGTGATCTCCAT
6B-1596170-T6-21_896	CTTTTTATTTATATGACTATGTATACTTTGC
6B-1596170-T6-21_960	AGTAGGTGGCACCCTCCTCTAAGGAAAA
6B-1596170-T6-21_1024	TGTAGCAAACCACTGTCAATTTATAAAATA
6B-1596170-T6-21_1088	TTTCAATTATTTATTCAGCCGCATACTTCAT
6B-1596170-T6-21_1152	TAAATAACACACTCGAAAACCAATTGAAG
6B-1596170-T6-21_1216	TCTAACTGCTCTTATATTTCTTTTTTTTTAA
6B-1596170-T6-21_1280	GCAAAATGTAGAATAAATTCAGCGCGACT
6B-1596170-T6-21_1344	AGCCCCAGTTTCGAATCGTTAACCATGAA
6D-1679410-T6-21_0	GGAGTTGGAAGCTGGAGAGGATCCGGA
6D-1679410-T6-21_128	ATCAGTAGGCTCGATGACAGAGCCTATAG
6D-1679410-T6-21_192	GAAGTGATGGAAACGTTTCATGTATCTGC
6D-1679410-T6-21_256	ATTCCAATCACAATTTAATCCTTCTCGACT
6D-1679410-T6-21_320	TTCGAATGTAATTTGAAAAGCTTTCAAATG
6D-1679410-T6-21_384	ACAGTAGATGTTGCTTATGTGATCACGATG
6D-1679410-T6-21_448	TGTGATTGCTATGGCGGAATATATGTAACT
6D-1679410-T6-21_512	GTTTGGGAACTATCTCACGGGACTGATAG
6D-1679410-T6-21_576	TTTATCTTGCACTATGTAGAAATTGTCCCA
6D-1679410-T6-21_640	GTTTGTTGTAGATTATCTTCGTGGATACTC
6D-1679410-T6-21_704	TTATATTCAACTAACTTGAATGACTGTCTA
6D-1679410-T6-21_768	TAATCTCCATGGAATTTGGCTACACCACTT
6D-1679410-T6-21_832	TTTGCCACAAAATGGTTGGGTGCACTATC
6D-1679410-T6-21_896	AAAACCCCAAACCGTCCAAATGGTGGCG
6D-1679410-T6-21_960	TATGGATGAGTTTATCACTGCATGAAATAT
6D-1679410-T6-21_1024	CCTCATTTTTGTTTGTAGAAGTGTTCTTTT
6D-1679410-T6-21_1088	AATTGAAGCGAGCTGTCCGAAATTACAAT
6D-1679410-T6-21_1152	AAGTCCTGCTTTAATATTGGTATTTAGAGA
6D-1679410-T6-21_1216	CACGATAATAATCTCATTTGTTTAACTAGTT
6D-1679410-T6-21_1280	TTGTCGCCGCAAAATGTAGAATAGATTCA
6D-1679410-T6-21_1344	CGCAGCCTGAGCCCTAGTTTCGAATTGTT
2A-0295460-T6-22-1_64	ATATGATAATCAGTCAACCCTTCAACTGAC
2A-0295460-T6-22-1_128	AACAACAACACATGGTGGAACAATTTCGA
2A-0295460-T6-22-1_192	AAACAACCGACAGTACATTAGCTAAGTTG
2A-0295460-T6-22-1_256	CAAACTTTCTAGGATCGCAAAATATCAGC
2A-0295460-T6-22-2_256	CCAAAGGCCCAGGGAGGAGGATGGTATT
2A-0295460-T6-22-1_320	ACGTTCTCTTGTCGTTATCAGCAACAGTTT
2A-0295460-T6-22-2_320	CCATTAACGTGCACCGGCCCTCCTCCTCC
2A-0295460-T6-22-1_384	AATTTCTAAATAAAATAGTGCTTACTCACA
2A-0295460-T6-22-2_384	AGCTGACTGGACCAGACGACGATTAGTTT
2A-0295460-T6-22-1_448	GCTAAGTTGGTTGTTTCCATAACTGAATTA
2A-0295460-T6-22-2_448	TGGCAGTTTAGAGCGTAATTACTCACGAT
2A-0295460-T6-22-1_512	AATATCACCAGAATATTGGATCAACGAATA
2A-0295460-T6-22-2_512	GGGCCCCGCGCGAGCCAGCCAGCCAGG
2A-0295460-T6-22-1_576	ATAACAGTCTATTTGCTGAGCGTTACACAA
2A-0295460-T6-22-1_640	AGATAGTGCTTACCCACAAAAGTAATTCA
2A-0295460-T6-22-1_704	GTTTCCTTAACTGAATTATCATTTAGGGTT
2A-0295460-T6-22-2_704	GCCCGAACTGAAAGTCGGAAGCGTCTGC
2A-0295460-T6-22-1_768	ATATTGGTTCAGCAAATAACAAGATCCGG
2A-0295460-T6-22-2_768	TACATCTTCTATCTTCTCCTCCGCCCCGGC
2A-0295460-T6-22-1_832	TTGCTGAGGTTACACAAAAACATCATGAC
2A-0295460-T6-22-1_896	GTAATTAGTCAAACCACCGACAATTCTTTA
2A-0295460-T6-22-1_960	TGAAGGATGAAGAACTTTCCAGGCTCGC
2A-0295460-T6-22-1_1024	GATCGGGCACATGTTCTTTTGTCGTTATCG
2A-0295460-T6-22-1_1088	CGTCTTGAAACAATTTCTAGATAAATCAGT
2A-0295460-T6-22-1_1152	CGACAAAATACTGGTTCAATGAATAATAA
2A-0295460-T6-22-2_1152	CAGTCTCACGTTTCTCTCCGTGTGTGTGG
2A-0295460-T6-22-1_1216	TAACAGATTATTTGTCGGGCGTTACAGAA
2A-0295460-T6-22-2_1216	GTGCTGCGCGTCGGCTGTAGGACTAGCC
2A-0295460-T6-22-1_1280	AAACAATTTCTAATAAAATATCTAACTAAA
2A-0295460-T6-22-2_1280	AGCAAGGAGTATCGCAACCTAAATTTGTC
2A-0295460-T6-22-1_1344	CTTCTTGAAACAATTTCTAATAAAATACTG
2A-0295460-T6-22-2_1344	TACCCGGTGGCACACCTCAAAACATCATT
2A-0295460-T6-22-1_1408	CAGTATGTTAGCTAAGTTGATTGTTTCCTT
2A-0295460-T6-22-2_1408	TCATACTATCTTTGTTATTTGAGATATTGGT
2A-0295460-T6-22-1_1472	AGGCTTTCAAAATATCAGCAGAATATTGG
2A-0295460-T6-22-2_1472	ATTAGGGATGCAAATTTGGAAAGTTAGCT
2A-0295460-T6-22-1_1536	TTTTGTCGTTATCGATAACAGATTATTTGT
2A-0295460-T6-22-2_1536	GATATCCTCAAGGTTCCAAATCCGCATCTA
2A-0295460-T6-22-1_1580	TCAACGACTTCGAAGATATGCCGGATTGT
2A-0295460-T6-22-2_1580	TGCTGGATATTTTGTGATTGAGAAAATGTA
2B-0393950-T6-22_0	AACTAAGTGTAAGCAAAATGTTTATGTGCT
2B-0393950-T6-22_64	GGTGAATGTGATGGGATGCAAGGGAGTT

2B-0393950-T6-22_128	TACGTTTTCTCGATCACAAAATATCCGGCA
2B-0393950-T6-22_192	TGAGGGTATCCTTCGAGGCTTCGACCCTT
2B-0393950-T6-22_256	CCAAATTTGCATCCCTCACGGAGAGAATT
2B-0393950-T6-22_320	CAAATAACAAAGATAGTAGTATGAGCCATA
2B-0393950-T6-22_384	AAATGATGTTTTGAGGTGTGCCACTGGGT
2B-0393950-T6-22_1152	CGTACTCTTGCAGGCAACGCAGCCAGGG
2B-0393950-T6-22_1216	ATCCAGCCGGGGCGGAGGAGGAGATAGA
2B-0393950-T6-22_1280	CGTTTCCGACTTTCAGTTGAGGCAGATGG
2B-0393950-T6-22_1472	CGCGTGGGGCCCCGATTTTCCTCGCTCTA
2B-0393950-T6-22_1536	CCAAAACTGCCAAGGCGTGCTGCAGGAT
2B-0393950-T6-22_1580	GTCGGCTCAACTCAACTCAGAAAAGAAA
2D-0536210-T6-22_0	AGATATCTATTTGATCTCGTGCATCTGGCT
2D-0536210-T6-22_63	ACTATGGGCCTCACCCGAAAGAAAGACTA
2D-0536210-T6-22_252	GACGGGCCTGCTACCAATATCTGAAATAA
2D-0536210-T6-22_315	ACCGTTTAGCCAGAAAATGATGTTTTGAG
2D-0536210-T6-22_378	ATTTAGATTGCGATACCCCTTACTAACTTT
2D-0536210-T6-22_756	CAGGGGGACGAGGCCAAGTTCGCCACC
2D-0536210-T6-22_819	GTAAAAACAAAAACGTGTTGGCTTTTGCA
2D-0536210-T6-22_1008	GCCCCGACTGACAGGTGGGCCACCAAGT
2D-0536210-T6-22_1197	ACCCTCCAAAACTGCCAAGGCATGCTGCA
2D-0536210-T6-22_1323	CATCGATCAGATGGAAGAGAAGAGGGCC
2D-0536210-T6-22_1827	tctctcctctcctctcctctcctcgccGCCGACCGG
2D-0536210-T6-22_2016	ACCACCCCGAGGTGACGGGATCTCTTCTC
2D-0536210-T6-22_2079	TGTCAATTGATTGCGATTTCTCCCGGAAG
3A-0616020-T6-23_0	CAAGTTGCCAGTGGACGATTTGGAGTTT
3A-0616020-T6-23_64	TAAAAATGGCTCAGGTACTTGACAATGTT
3A-2150720A-T6-23_64	TCGCGAGAGGAGCAAAACTGAGTTTACA
3A-0616020-T6-23_128	TAGAAAGCAACTGTATTCAATGTCATGTG
3A-2150720A-T6-23_128	GTGTGAGGCGAGTCTGCGATGATAAGCT
3A-0616020-T6-23_192	ATAGGGTGCTAAGCCTGGTGAGGGGGGT
3A-0616020-T6-23_256	GTTACCAGACATTCTACAGCTGGTGTTGG
3A-2150720A-T6-23_256	AGCTGCTCTCGACACTGAACCAGCGATTG
3A-0616020-T6-23_320	CTATCGAGGATCTTGCACAGCTTATCCACG
3A-2150720A-T6-23_320	GCCGACTTCGAACAGCAGGTCCTCCATGA
3A-0616020-T6-23_384	CCAAGGCATCTGCCTATTTATGTATGGTTG
3A-0616020-T6-23_448	TATCTAAAGCACTGATGGTCTAACCCTCTT
3A-0616020-T6-23_512	GGATCCTGCACATATGCATGTGTCTCCATT
3A-2150720A-T6-23_512	GGTCCTTACTTCTTCTGAATTGCATTTTCA
3A-0616020-T6-23_576	TGCAAATGTTACCTGCTGATGTAGTGATAT
3A-2150720A-T6-23_576	ATTAAGCTCCTGACTTTAATGGAATATTAA
3A-0616020-T6-23_640	TCAAGCTCGAATCAGCGTGAAGCTAGTGT
3A-2150720A-T6-23_640	CTATCTCTTAAACTTTAGAAATCTGTAATGT
3A-0616020-T6-23_704	GTAAAAGGACACGCAGACCATGTTCTTAT
3A-0616020-T6-23_768	GGACAGGTATCAAGAATGCTGGACTTCCA
3A-2150720A-T6-23_768	CCGTCTCAGCTTCAGGGGTACTACTATGC
3A-0616020-T6-23_832	AGTGGCAAATGGGCTTCGTGGTCGAGCT
3A-2150720A-T6-23_832	CTCTGGTAAATACGAAACCTTGATCCTCTT
3A-0616020-T6-23_896	GATGTTGCTGTGGCGTGCTTACTTGGTGC
3A-0616020-T6-23_960	CACTTGGCTGCATTATGATGCGGAAGTGT
3A-2150720A-T6-23_960	TGTCTATTCATATGCTATTTCAGTCCTGCTT
3A-0616020-T6-23_1024	AGATCCAGTGCTCCGAGAAAAATTTGCTG
3A-0616020-T6-23_1088	CTTGCTGAGGAGCTAAGAGAAATTATGG
3A-0616020-T6-23_1152	GACGCTCTGATATGCTTGAAGTTGATCCA
3A-2150720A-T6-23_1152	CTGTTTTGCATGAAATTATACGCAGCTGA
3A-0616020-T6-23_1216	TGATCTCTCATTGATCTTAAAACCAGCTGC
3A-0616020-T6-23_1280	GAAAAGCAAGACCATGGCCTTGACATGG
3A-0616020-T6-23_1344	CACTTGAAAAGGAAGTTCGTGTTTTCATT
3A-2150720A-T6-23_1344	TAGGCCACAATGGAGAGATCAATACTCTC
3A-0616020-T6-23_1408	CACTACACTTAGCCATGAAGTCACAAAAC
3A-0616020-T6-23_1472	CATGTGAAACTCACTGGAAGTGCTGGTCA
3A-2150720A-T6-23_1536	AATGTCAAAAATTCTTCCAATAGTGGATG
3B-0709800-T6-23_0	ATCAAGCAAGTTGCCAGTGGACGATTTG
3B-0709800-T6-23_64	TGCAGATAAAAATGGCTCAGGTACTTGAC
3B-2150720B-T6-23_64	CAAAACTGAGTTTACAAAGGTTGGTTGG
3B-0709800-T6-23_128	AGTTTATAGAAAGCAACTGTATTCAACGT
3B-0709800-T6-23_192	TATGTATAGGGTGCTAAGCCTGGTGAAGG
3B-0709800-T6-23_256	TTGCAGTTACCAGACATTCTACAGCTGGT
3B-2150720B-T6-23_256	TACTGAACCAGCCATTGAACAGGTTTTCC
3B-0709800-T6-23_320	ATATTCTATCGAGGATCTTGCACAGCTTAT
3B-2150720B-T6-23_320	CAGCAGGTCCTCCATGAGTTTAATTTTCT
3B-0709800-T6-23_384	TATAGCCAAGGCATCTGCCTATTTATGTAT
3B-0709800-T6-23_448	ATCTAAACGCTGTGATGGTCTAACCCTCCT
3B-0709800-T6-23_512	TGATCCTGCACATATGCATGTGTCTCCATT
3B-2150720B-T6-23_512	TTCTGAATTGCATTTTCACTACAAACTGAT
3B-0709800-T6-23_576	TGCAAATGTTACCTGCTGATGTAGTGATAT
3B-2150720B-T6-23_576	ACTTTAATGGAACATTAACAACACAAAAG
3B-0709800-T6-23_640	TCAAGCTCGAATCAGCGTGAAGCTAGTGT
3B-2150720B-T6-23_640	GCTATCTCTTAAGCATTAGAAATCTGTAAT
3B-0709800-T6-23_704	GTAAAAGGACACGCAGACCATGTTCTTAT
3B-2150720B-T6-23_704	GTAATTATTGTTAAATCTTCTATTGTGGTTT
3B-0709800-T6-23_768	GGACAGGTATCAAGAATGCTGGACTTCCA
3B-2150720B-T6-23_768	CCATCCCAGCTCCAAGGGTACTACTATGC
3B-0709800-T6-23_832	AGTGGCAAATGGGCTTCGTGGTCGAGCT
3B-2150720B-T6-23_832	CTCTGGTAAATACGAAACCTTGATACTCTT
3B-0709800-T6-23_896	GATGTTGCTGTGGCGTGCTTACTTGGTGC
3B-2150720B-T6-23_896	AATCAATAATAGCAAGCCAGTTGTAAGAT
3B-0709800-T6-23_960	CACTTGGCTGCATTATGATGCGGAAGTGC
3B-0709800-T6-23_1024	AGATCCAGTGCTCCGAGAAAAATTTGCTG
3B-0709800-T6-23_1088	CTTGCTGAGGAGCTAAGAGAAATTATGG
3B-0709800-T6-23_1152	GACGCTCTGATATGCTTGAAGTTGATCCA
3B-2150720B-T6-23_1152	CTGTTTTGCATGAAATTATACGCAGCTGA
3B-0709800-T6-23_1216	TGATCTCTCATTGATCTTAAAACCAGCTGC
3B-0709800-T6-23_1280	GAAAAGCAAGACCATGGCCTTGACATGG
3B-0709800-T6-23_1344	CACTTGAAAAGGAAGTTCGTGTTTTCATT
3B-2150720B-T6-23_1344	TAGGCCACAATGGAGAGATCAATACTCTC
3B-0709800-T6-23_1408	CACCACACTTAGCCATGAAGTCACAAAAC
3B-2150720B-T6-23_1408	CTTTTCCTCTTTGCTTTAACCTTTTCTTGG
3B-0709800-T6-23_1472	CATGTGAAACTCACTGGAAGTGCTGGTCA
3B-2150720B-T6-23_1580	CAGGTCCGCAGTCTAATATGCAGCATGAA
3D-2150720C-T6-23_64	CGCGAGAGGAGCAAAACTGAGTTTACAA
3D-2150720C-T6-23_256	GCTGCGCTCGACACTGAACCAGCGATTG
3D-2150720C-T6-23_320	CCGACTTCGAACAGCAGGTTCTCCATGAG
3D-2150720C-T6-23_512	GTCCTTAACTTCTTCTGAATTGCATTTTCA
3D-2150720C-T6-23_576	ATTAAGGTCCTGGCTGTAATGGAATATTAA
3D-2150720C-T6-23_640	CTATCTCTTAAACTTTAGAAATCTGTAATGT
3D-2150720C-T6-23_768	CCGTCTCAGCTTCAGGGGTACTACTATGC
3D-2150720C-T6-23_832	CTCTGGTAAATACGAAACCTTGATACTCTT
3D-2150720C-T6-23_896	AATCAATAATAGCAAGCCAGTTGTAAGAT
3D-2150720C-T6-23_1152	CTGTTTTGCATGAAATTATACGCAGCTGAT
3D-2150720C-T6-23_1344	TAGGCCACAATGGAGAGATCAATACTCTT
3D-2150720C-T6-23_1536	AATGTCAAAAATTCTTCCAATAGTGGATG
5A-1230940-T6-24_0	TTGTGCCAATCAAGGAAAATAGTTGAGTA
5A-1230940-T6-24_64	TTTGTTTTTGGGCATGGTATGATTTCTCTA
5A-1230940-T6-24_128	GTGTTGCGTCGGGTAATGTCCCAACCGTT
5A-1230940-T6-24_192	TGACTAATGCAGTCTTCAAAGCCTGGTAT
5A-1230940-T6-24_256	TGCGGTTGATGTACAATTGAAGAAAAAG
5A-1230940-T6-24_320	TCATTTTGTGCTTAGTCATCTTTCCATTTTT
5A-1230940-T6-24_384	ACTCCCACCGCCCGGAATTGATAGATATC
5A-1230940-T6-24_448	ATGGTAACCTTTCGGAGGTGCAACTCGAA
5A-1230940-T6-24_512	TGGATTTTCCTAGTAGATATATATGCCGAA
5A-1230940-T6-24_576	TTCACAAAACCAAAATGAACAAGAAGCA
5A-1230940-T6-24_768	CTCGTACCAATAATGCCGGGCGGAGGAC
5A-1230940-T6-24_1280	AGAAATTTCCTCATTTTGTATATACCACCA
5A-1230940-T6-24_1344	GAAAAACAATAATGCGTTGCACGATGCAA
5A-1230940-T6-24_1408	TCAATAATGCATGGAGATGGCAAGGCGGT
5B-1310950-T6-24_756	ACGCACATGAACCGCTCATGTCGACACTC
5B-1310950-T6-24_819	CTCCTGCTACGTAAGCGATGGGCAGCATA
5B-1310950-T6-24_882	AAGATGCACCCCTGACATACATATACCAAA
5B-1310950-T6-24_945	CAAATGCAGAGTAAGATAGATGAAGAAA
5B-1310950-T6-24_1008	AGGAACAGGAAGCAATAAACTAATCAATA
5D-1468150-T6-24_0	CTCCTCTCTCCCACCTGGCGACCTTGTCAG
5D-1468150-T6-24_64	TTCTTAGTTTTAATAGGTCCTTATTCTCCTT
5D-1468150-T6-24_192	GACGAAGGTCCTATGAGGGTAGGTCATG
5D-1468150-T6-24_384	CTCTCAACTGATGGTGAAGGATCGCAAGT
5D-1468150-T6-24_448	CTTTAACGCTTACTTGATCTTGTTTCCAAT
5D-1468150-T6-24_512	TGACATGTACATTTGCAGGTTTTCATTTCC
5D-1468150-T6-24_576	GCGATGATGTACAATCAAAGAAAAAAGAT
5D-1468150-T6-24_640	TCATTATGTGCTTAGTCATCTTTTCATTTTT
5D-1468150-T6-24_704	TTCCCTCCGTCCAGAATTACTTGTCTCTCA
5D-1468150-T6-24_768	TACACTAAAATACATCTAGATATAAGTAATT
5D-1468150-T6-24_832	GTCGAAGCCTTTTTCATTGATAAGCTAGC
5D-1468150-T6-24_896	AGTCGAAGCCTTTTTCATTGATAAGCTAG
5D-1468150-T6-24_960	AAGTCAGAAGCCTGTTCCGTGCTCTCTAG
5D-1468150-T6-24_1152	GCTATTGCTAGCCACAGTCACGCACATGA

5D-1468150-T6-24_1280	CAACAAGATGCATCCCTGACATACATATAC
5D-1468150-T6-24_1344	AACCAAATGCAGAGTAAGATACACGAAG
5D-1468150-T6-24_1580	GAGGGAGTAATCAATAATGCATGGAGATG
3A-0682630-T6-25-1_128	TGGAACCGGTGACTGCGTATGTTGGGAC
3A-0682630-T6-25-1_192	GCTACTACCGGTGAAAAAATTGCTACAAC
3A-0682630-T6-25-2_192	TCCATCGCCGTTGCCGTCAACGGGGAGG
3A-0682630-T6-25-2_320	CACCAGCCTTCTTCTTCTACTTGATATATAA
3A-0682630-T6-25-1_384	AAAAGCTACAACCAGCTGAACAAAATGC
3A-0682630-T6-25-2_384	CCGTAGCTTCTAGCATACAAAAAAAAGAT
3A-0682630-T6-25-1_447	TTCAGAAAAAAAAATGCAAGAAATAGATT
3A-0682630-T6-25-2_448	TAATTTCATATAGTAATCAGCGAAATTAAA
3A-0682630-T6-25-2_512	GATTAAAATTTTAATAAAAGGAAGATCTT
3A-0682630-T6-25-2_576	ACCTGAATGACAATTAAAATTTTAAAATAT
3A-0682630-T6-25-2_640	AAATATGATCCGGTGTTCTATATATATCCAC
3A-0682630-T6-25-2_704	GTACCGGGATAAAATGAAAAAAAACAGG
3A-0682630-T6-25-2_768	GCTTAACGACAAGAAAACTATAATCGGCT
3A-0682630-T6-25-2_832	CTCCAGTTACTCTGTGTTTTTTATGCAAGA
3A-0682630-T6-25-1_996	CATGGCTGCTGCGGGGGGAGAGAGCATG
3A-0682630-T6-25-2_1024	GTTAACTCCTCATTAAGCTTATTAAGTTTA
3A-0682630-T6-25-1_1062	TGGCGGCCACGTGATAGGATCCAGAGAC
3A-0682630-T6-25-2_1152	CCCTAACCACGGGTGTGATTTCTTGGTGG
3A-0682630-T6-25-2_1216	TTTTTTTTTGCTAATTTTCCTGATTTCTCTA
3A-0682630-T6-25-2_1280	TCCATTTTTAGATTCTTGCATGATTAAAAA
3A-0682630-T6-25-1_1326	GAGGATTGACTCGATACTGGTAGCTGCTA
3A-0682630-T6-25-2_1344	AGCTCTTAGTGGCATTCTCTTTGTTTAGCA
3A-0682630-T6-25-1_1392	CACGGACATGTAACAGGCAGAAACGCAT
3A-0682630-T6-25-1_1458	AACGAACCCATGGTCTTATGTGGTGCGGT
3A-0682630-T6-25-2_1536	TGGGGAATTGATGGTATAGTATCAAAAGT
3A-0682630-T6-25-2_1580	TCATGAAACAAAACTATCATTTGCTCTGTG
3D-0907290-T6-25_0	CGCCGCCGCGGCATCTGACATGCCGTTG
3D-0907290-T6-25_64	GATCGTCCGACCGCCAGCCAGACAGGAA
3D-0907290-T6-25_128	TTGGAACAAGCACAATTAATCTGGACTCT
3D-0907290-T6-25_256	CGTCGGGCGCTTGGCCTCTTTTTTCCGCA
3D-0907290-T6-25_320	GCCCTCCAAAATTCGCAGGGGCACGTAA
3D-0907290-T6-25_384	CTCAACGTGTCTCGCGATTTATGAAGTAG
3D-0907290-T6-25_704	TTTATTTTGTTTAAAAGATTTTTTTAAATCT
3D-0907290-T6-25_768	CACTTACTTGTCGTGAATTCTCGCCTTCCT
3D-0907290-T6-25_832	CATTTGTTTTATTTTATTTTTCAATAATAAG
3D-0907290-T6-25_896	ATCCATCCATCATCCATTTTTTTAATATAGT
3D-0907290-T6-25_1088	TATGTCCCCTCCCGCTGAACGCACGTCGC
3D-0907290-T6-25_1152	GCATTAATGTTAAGTTTAGAACTTAAACTC
3D-0907290-T6-25_1216	CCAACTATAGGTTGATTGGTTCTACCTTCC
3D-0907290-T6-25_1280	GGGGTGGGGGGCATAGTCGGAGCCTGA
3D-0907290-T6-25_1344	ACTAGCTGGTAATGGATGATGCGAGAAG
3D-0907290-T6-25_1536	CTCTCCGCCATCTCCGGCCAGGCATCTATC
3D-0907290-T6-25_1580	GCCAAGCCATTCCTGCACACCTCCATACA
2B-0453490-T6-26_128	GGAGCGGAGGAAGGAGAGGAAGTTGGT
2B-0453490-T6-26_192	GCGTGTAAGGGCTTGGCCCACCTCTTGG
2B-0453490-T6-26_320	CTAAAATATATGTAAATACATTCATTTCTTG
2B-0453490-T6-26_384	AAGAAGAAGAAATGGGATGTCCACGTAA
2B-0453490-T6-26_448	ACATGAATTACGTATACTCCGAGAGTTGC
2B-0453490-T6-26_576	ATATGGCGATGTCGCCTAGCGGTCGACGG
2B-0453490-T6-26_704	AGCCCGGCTTTGATTTAACAAAGCCATCA
2B-0453490-T6-26_768	AGAAAAGTAAGTACACGGCAAAAGGTAC
2B-0453490-T6-26_1024	CTCCTCGCCTCCAACACCCATCTTCAAAG
2B-0453490-T6-26_1088	CGAAGGAGGCCACAACTCGCCTGGATCA
2D-0597240-T6-26_256	GAGCACCTAAACTTAGGGGCTCCCGTAG
2D-0597240-T6-26_320	TTTGCGCTTCATGTTAATTGTGTTTCACAT
2D-0597240-T6-26_512	GTAAGTTACTATGCATATTCGACGAAAAA
2D-0597240-T6-26_832	AAAGGGAATCGATCGTGATTTGGAACCTC
2D-0597240-T6-26_896	AGAACGAAGAACAACATGAAACTAAGCC
6A-1521780-T6-27_0	TGGATGAAAAAAAAGAAAAGAAAGGGG
6A-1521780-T6-27_64	AATATACATGGCCTAACAAAAACATGTAG
6A-1521780-T6-27_128	TTCCTGCCTCCGCCTCTATAAATAGTCTTT
6A-1521780-T6-27_320	GTCAAATATTTGTTCTTGAGGAGTATTCTT
6A-1521780-T6-27_384	GCACAGCAAGGGCCAAAGCTGGACTGA
6A-1521780-T6-27_448	ATGAAATTTGCCAGACAACTGGTTGCAGT
6A-1521780-T6-27_512	CCCCTTCTCTTGAGTGATTTGCTAATCTCC
6A-1521780-T6-27_576	TATATGGGCGAGGTTCATTTAATTATTTCG
6A-1521780-T6-27_704	ACTAACCTGCTGTAACCTGACGTTCTCATG
6A-1521780-T6-27_768	TGGTATAGCGGCGCAATCGCTCTTCTCATA
6A-1521780-T6-27_960	GGCATATCATTGCAGATGTTTATATTCTTTC
6A-1521780-T6-27_1024	ATTTCTTTTGGCAATTGAGTAGTTTACCGT
6A-1521780-T6-27_1088	GCAAGTTTTTACTTTGACAGTTTCCCTTTT
6A-1521780-T6-27_1216	GTGCAAGTAACTAGTCTGCTATTTGAACT
6A-1521780-T6-27_1280	TTCGTGCAGTGACACCTTCTGAACAACGG
6A-1521780-T6-27_1344	TGCATCATTCTGAACATTGGAGTAAAACG
6A-1521780-T6-27_1408	CCACTCTTCTGATATAGATGTGTCATCTCT
6A-1521780-T6-27_1472	CCGAATTACCTTCTCTTTTCTCATGGAGCA
6B-1612430-T6-27_128	GAAGTTCATCAAAAGCAGAATACCTGTGA
6B-1612430-T6-27_192	GGCGTCTGCTCCATGAGAAAAGAGAAGG
6B-1612430-T6-27_256	GAGAGATGACACATCTATATCACAAGACT
6B-1612430-T6-27_320	TCCGTTTAACTCCAATGTTCAGAATGATGC
6B-1612430-T6-27_384	CTCCGATATTCAGAAGGTGTCACTGCACG
6B-1612430-T6-27_512	GCCATGTTAAATCTGTGTCGTTATCGTGAT
6B-1612430-T6-27_576	GGAAAGGGAAAATGTCAAAGTAAAAACT
6B-1612430-T6-27_640	TACGGTAAACTAATTGCCAAAAGAAATAT
6B-1612430-T6-27_768	GCCCAGGTGTTAATGCCTTGGTGAGTGTT
6B-1612430-T6-27_832	TAAACCTAACGCATCTAAATTCTGTACGCA
6B-1612430-T6-27_1024	GTTTCAAACATTTGACTTGACTATCAGTTC
6B-1612430-T6-27_1088	CAGCGGGAGATTTGTAAGAAACGAAATA
6B-1612430-T6-27_1216	GAGAAGGGGATTCAGTCCAATAGCAGGC
6B-1612430-T6-27_1280	AAATTTCATAGGGGGAGAGAGAAGGACG
6B-1612430-T6-27_1344	GCACTTGCTGTGCCTTTCGGCAGATGCAT
6B-1612430-T6-27_1536	GCgaggagaggaaggcgaggagagagggggaga
6B-1612430-T6-27_1580	GCCAAAGACTATTTATAGAGGCGCTGGGA
6D-1718540-T6-27_0	GCTTCCCCGAGTGAACCTAGCAAATACAT
6D-1718540-T6-27_64	TCAAGATTTTCCTCGCCCTTTTTTTCCCAG
6D-1718540-T6-27_256	TCGCATGCAATGCTCGGTTGGTCAAATATT
6D-1718540-T6-27_320	GTTGATACTTCTGCCGAAAGGCACAGCA
6D-1718540-T6-27_512	TTAGACTGATTTGCTAATGTCCTGCATATT
6D-1718540-T6-27_576	GCGAGGTTCTATTAATTATTTCGTTTCTTA
6D-1718540-T6-27_768	TACGCTTCGGTCATGGCTTATAGCGGTAC
6D-1718540-T6-27_960	ATCAGTGATTGGGTCGGCATATCATTGCA
6D-1718540-T6-27_1024	AAGGTTAAGCATCATATTTCTTTTGGCAAT
6D-1718540-T6-27_1088	CCCAGCTATCTGCAAGTTTTTACTTTGACA
6D-1718540-T6-27_1152	TTTTCTTCCTCTATCACGATAGAGACACAG
6D-1718540-T6-27_1216	TTGTCGACGTCATGGAAGTAACAGCCTGC
6D-1718540-T6-27_1280	TCTTCTTGATGCGTGCAGTGACACCTTCT
6D-1718540-T6-27_1408	GTGCCAATCCAGTCTTGTGATATAGATGTG
7A-1828020-T6-28_192	GTGGAACTACTGCGTTTTTTCTAACCGGA
7A-1828020-T6-28_256	GGTGCACAATTATATGAACAGTTAACATTC
7A-1828020-T6-28_320	GAGAATAACATTGTTGTTTTTGTCCTGCA
7A-1828020-T6-28_384	TATTCATATTTCGAGTTCAAATCAAAGTGG
7A-1828020-T6-28_448	ATTTTTGGATGAAATGTGTGGCCATTTCTC
7A-1828020-T6-28_512	AAAATTGACTCGAAGAATCAGAGGGACT
7A-1828020-T6-28_576	CATGCGGGAGCCGCGCACATCCAATAAA
7A-1828020-T6-28_640	AATAAGATCAAATACGGTCAAAGGAGAA
7A-1828020-T6-28_704	ACATGTATTATAGATGGACCAAGACTATGT
7A-1828020-T6-28_832	GGACCACGCGCAGCCTCCTCCCCCAACT
7A-1828020-T6-28_896	ACCAGCCTCCAAACCGCATTCCCCCATGC
7A-1828020-T6-28_960	GGTAAGGGATCCAGAATCTATGACCGGTG
7A-1828020-T6-28_1088	ATAGATATGCTCTCCTACGCCATGGCTCAA
7A-1828020-T6-28_1152	ATTAAGCTCTAGCAAGTGGGAGGCTGCC
7A-1828020-T6-28_1216	CCTAGTTTTTTTCCTGAGAAACAATTTCCT
7A-1828020-T6-28_1280	TGTTGAATTCAGTAAATAGTTGCATCCCAA
7A-1828020-T6-28_1344	CTGAAATTTTATGAATGTTGATGAGCTAAA
7A-1828020-T6-28_1408	TGTTAATGTTCAATTCAGCAATTTGCATCC
7A-1828020-T6-28_1472	TCAATTTTCTATATGTTGATGTTGAATACCA
7A-1828020-T6-28_1536	TTATGATTCAGGAAAGCGCAAAAACCCAA
7A-1828020-T6-28_1580	CCAGAAAACATTTTTGTCTCCCTTCTCTAA
7B-1924480-T6-28_192	AGTGGAACTACCGCGTTTTTCTAACCGGA
7B-1924480-T6-28_256	GGTGCACAATTATATGAACAGTTAACATTC
7B-1924480-T6-28_320	AAGAATAACATTGTTGTTTTTGTCCTGCA
7B-1924480-T6-28_384	TATTCATATAGAAGTTCAAATCAAAGTGGT
7B-1924480-T6-28_448	TCTTGGATGAAATGTGGGGCCATTTCTCG
7B-1924480-T6-28_1472	CTCAGAGGGACTCTGGTGGAAGAAGATC
7D-2033460-T6-28_192	TAGCCAGTGGAACTACTGCGTTTTTTCTA
7D-2033460-T6-28_256	ATTGTAGGGTGCACAATTATACGAACAGT
7D-2033460-T6-28_320	TTCAAGAATAACATTCTCGTAAAAAGAAA
7D-2033460-T6-28_384	TTTCAAATTATACACCCTTTTACTATTTGTA
7D-2033460-T6-28_448	TGGAGGCTGTGGAAATTCAAATATTCTTT
7D-2033460-T6-28_512	GTCACATATAGCCCTCTGTTGCAAGGGAG
7D-2033460-T6-28_576	ACGAAGATCCAATAAACCTAATGTCGAGA
7D-2033460-T6-28_640	TGGGGAGAGAGAAGCATAGTCCAACATG
7D-2033460-T6-28_704	CAAGTCTCCGGATCGAGATAGACTATGTT
7D-2033460-T6-28_896	TTGCAACGCAGCAACTAGGCTCCAAACTG
7D-2033460-T6-28_960	CCCCTTTTGCCCTGCTGTAAGGGATCCAG

7D-2033460-T6-28_1024	CCACCACTGCAACAATGGCACCCATAGTA
7D-2033460-T6-28_1088	GCACACCATATCGTCAGAGATATGCTCTC
7D-2033460-T6-28_1152	TTCCCCGGTAAAAGAATGAAGCACTAGC
7D-2033460-T6-28_1216	GTTGGTTTTGTAGAACTAGTTTTTCTGA
7D-2033460-T6-28_1280	TATGAATTTACTGAATGTTCATGTTGAATT
7D-2033460-T6-28_1344	GAATTCAGTAGGTTGCAAGACTTGAATTT
7D-2033460-T6-28_1408	ATATTGGTGAGCCGAATTAGCTGAATGTT
7D-2033460-T6-28_1472	TTGAATTCATCGAAAGTTACAAAGTTCAA
7D-2033460-T6-28_1536	GGGGACTCCCATTCAAATTTATGTGTTGC
7D-2033460-T6-28_1580	GAAAACCCGATTCAAAAAGATGCATCCAG
1A-0026620-T6-29_0	ATAATGATATTATGATAGAAGCAGACAAAA
1A-0026620-T6-29_63	AACATGAGGTGTACTTTATATTAGCTTATG
1A-0026620-T6-29_252	TCCAGATTTAACATACTCCATAACAACATA
1A-0026620-T6-29_315	CCGTATGATATGAGGATGCATAAATAACCT
1A-0026620-T6-29_378	GCACAAAAAGAAACTGAGAATTGAGCAA
1A-0026620-T6-29_441	TATGTAAATCAGCAAAGTAACAAACAGCG
1A-0026620-T6-29_504	TGGCCAATTGTGATATTTATACTAAACGCA
1A-0026620-T6-29_567	AGCATCATACGCTTCTAACACAAATAAGG
1A-0026620-T6-29_630	CACTAGAAACATGCAAATCGAACAATGTA
1A-0026620-T6-29_819	GAGACCTCAACACAACACATATTATTTTCT
1A-0026620-T6-29_882	CTATTCAATCAAATCAACCTAACATAGCCC
1A-0026620-T6-29_1071	CACCCCTCCATAGCAACATAAATATCCGCT
1A-0026620-T6-29_1134	TGAGGATTGCATAAATAATCTTAGTATATC
1A-0026620-T6-29_1197	AAACTGAGATTTGACAAAGTACGAAACA
1A-0026620-T6-29_1260	GCAAAGTGACAGAAAGGAACTTTTAGCA
1A-0026620-T6-29_1323	ATACTTATACTAGATATCCACAACAGACAG
1A-0026620-T6-29_1386	CAGGCTGATAACAGAAATAAGGATGAAG
1A-0026620-T6-29_1449	AACATGCAAATGGACCTGGGCTGTGCTTT
1A-0026620-T6-29_1512	ACTTCAGAAACAGAAACAAGCTTTCTGA
1A-0026620-T6-29_1701	GCCAGGGGGTTGGCATCTCTGCCTGCTG
1A-0026620-T6-29_1764	CAGTGGCAAAGCAAAAGAGGAATTAAAA
1A-0026620-T6-29_1827	TTACGGTCGGATCTAGACATGCGTGGTCG
1A-0026620-T6-29_1890	CGATCTGTGGATTCCGCGCGGAAACGAG
1B-0108930-T6-29_0	CGTCGACCTAAAATAAATCACAGCCAAGC
1B-0108930-T6-29_58	AAAAGCTATTATCACCAGCTCAGGGGTGC
1B-0108930-T6-29_116	GGGCGGCCACACTTGGTGCGGCAGACCA
1B-0108930-T6-29_174	GATGAAACGAGGAGTGGAAGGAGGCTC
1B-0108930-T6-29_348	TGGAGCGCGGCGGCACGAGCGGAGCGG
1B-0108930-T6-29_1211	GAGCGAGACCGAGACGGAGGGTGGGGA
1B-0108930-T6-29_1274	TCCGTTGGTGAGCTGTTCACCCTTTTTTAG
1D-0193790-T6-29-2_64	ATGACATGTACTTCATATTAGCTTATGGTAA
1D-0193790-T6-29-1_65	AGCTAAGGTCGGATCTACACATGCGTGGG
1D-0193790-T6-29-1_130	GATTTGTGGATTCCGCGCGGAAACGAGG
1D-0193790-T6-29-2_256	TTTAACATACTCCATAACAACATAAATATCC
1D-0193790-T6-29-1_260	CGCACGCACGCAAGGGAGGGCGCGGGA
1D-0193790-T6-29-2_320	ATATGAGGATGCATAAATAATCTCAGTATC
1D-0193790-T6-29-2_384	AACTGAGAATTGAGCAAAGGTGCAAAAC
1D-0193790-T6-29-2_448	GCAAAGTAACAAACAGTGACTTTTAATGT
1D-0193790-T6-29-2_512	TATTTATACTAGACACATCAGCCAACAGAA
1D-0193790-T6-29-2_576	ATAACACAAATAAGGATGAATAGTTTGAG
1D-0193790-T6-29-2_768	AATCACTAATTTCAGCTCGTTAGACTTAAC
1D-0193790-T6-29-2_832	ACAATCTTTTCTTAGGTGGCAATTTTTTTG
1D-0193790-T6-29-1_898	TGCGGCAAATCGGTTGAGCGAGACCGAG
1D-0193790-T6-29-1_960	CCGTGGGTGCGTGCGACGCGGACTCCTC
1D-0193790-T6-29-2_960	TAAAAGTCACACCCTCACTCTTAACCTGTA
1D-0193790-T6-29-1_1022	GCCGATTTTGATCTACACATCAACTGCCAC
1D-0193790-T6-29-2_1024	CACGAACACTCACGAGCTTAACAAGCCG
1D-0193790-T6-29-2_1088	ATATCCGCTGGGGTATCTATGACCTCATAA
1D-0193790-T6-29-2_1152	GCATCTCAATTTCTCTTTTTGCTGTAAATTA
1D-0193790-T6-29-2_1216	AACAACAAAAGTGGGAAAAGCACGAGTA
1D-0193790-T6-29-2_1280	ACATGACAAATTCAAGAAACTACGGATGG
1D-0193790-T6-29-2_1344	CCCGTAGAAATCTTGCAAGTTGATTGCTA
1D-0193790-T6-29-2_1408	GTGTTATACAGTTTCTATTTTCTACCTTAGA
1D-0193790-T6-29-2_1472	TTCTCAATGGGCGCAAATCTACGTACACTC
1D-0193790-T6-29-1_0	CGTCGGCCTAAATCACAGGGGCAAAAAG
1D-0193790-T6-29-2_0	AATGATATTATGATAGAAGCAGACAAAAA
3A-0649780-T6-30_128	AAGCAGGACAGATCCAGCTGGTCCGTGT
3A-0649780-T6-30_192	TAAAAGGCAGGCAGAAATGCTTAATTCTG
3A-0649780-T6-30_320	TAAGCTCGGGAGCTCACGGATCTGCAGG
3A-0649780-T6-30_384	ATGAAACGAAAACTCACGGTCCGCCCAA
3A-0649780-T6-30_896	TGAGCGCCACGGGTTCAACAGTGTCGGT
3A-0649780-T6-30_960	AGCAAGAGAATATCTGACACCGTAGAGA
3B-0775050-T6-30_0	CTTCCACCTTAATCGCAATCACACCAAACT
3B-0775050-T6-30_128	TTAAGTAGGACAGATCCAGCTGGCCCGTG
3B-0775050-T6-30_192	TGGGGATAAAAGGCAGGCAGAAATGCTT
3B-0775050-T6-30_256	GCTCTTCCTGCCCAATTCAACCTGAGACC
3B-0775050-T6-30_320	GCTCGGGAGCTCACGGATCTGCAGGCTA
3B-0775050-T6-30_384	AAACGAAAACTCAGGGTCCACCCATAAA
3B-0775050-T6-30_896	TCAACAGTGTCGGTACTCGCTTCAGCTGC
3B-0775050-T6-30_960	TCTGACACAGCAGAGACCAAATTACTACT
3B-0775050-T6-30_1024	GGCAACCTGCAAATTTCCTCCTGCACCCG
3B-0775050-T6-30_1088	GAAGTATGTATATATCTGCCGACAAATTCA
3D-0889780-T6-30_0	CTTCCACCTTAATCGCAATCACACCAAACC
3D-0889780-T6-30_192	TAGAAGGCAGGCAGAAATGCTTAATTCCG
3D-0889780-T6-30_320	TAAGCTCGGGAGCTCACGGATCTGCAGG
3D-0889780-T6-30_384	ATGAAACAAAAACTCACGGTCCACCTCAA
3D-0889780-T6-30_896	AACAGTGTCGGTACTCGCTTCACCTGCGA
3D-0889780-T6-30_1088	GAAAGAAGGAGCCAACATATCATCTTGAG
3D-0889780-T6-30_1280	GGGATACCAAAGTCCCTCTAACCATCCAA
4A-0952320-T6-31_0	GGCCGCCGGCGATCTCTCAACTAAACTTC
4A-2108990-T6-31_0	GGCCGCCGGCGATCTCTCAGCTAAACTCT
4A-0952320-T6-31_64	GTGTGCTTTGTAAGGTGCTTCTAGTACGTA
4A-2108990-T6-31_64	TGTGTGCTTTGTTAGGTGTTTCTGCTACGT
4A-0952320-T6-31_128	AAAGGTACATTATTTCACTTCTGCTTCGGT
4A-2108990-T6-31_128	GTAAGGAGATATTGTAGTATGCCAATTCAA
4A-0952320-T6-31_192	TCCGACATTGCACGTAGGTACAGCCGTAC
4A-2108990-T6-31_192	GGCATCGCTTACAAGTACAACGGATTGTA
4A-0952320-T6-31_256	TGTATAAAATAGAATACGAGAATGTAAAAT
4A-2108990-T6-31_256	GGTCAAATGTCTTGCCTTGAGAGCTACAG
4A-0952320-T6-31_320	TGGAATGCATTTCAATTATCACAAGGATTG
4A-2108990-T6-31_320	AAATGCCAACTTTAGTTTTCAGGTTTGAC
4A-0952320-T6-31_384	TTTGACTTTGAAGTTTCATATAGATAAAAT
4A-2108990-T6-31_384	AAATACTGAAACCAGATCATGGTTATGCT
4A-0952320-T6-31_448	CTCAAATGGAATATAAACATAAGCTGGATA
4A-2108990-T6-31_448	CAAGCAAGCTTTGGGAGAAAAGGTCAAG
4A-0952320-T6-31_512	TTCGAATAGTAGTAGGCTACTACCATTAGT
4A-2108990-T6-31_512	AGCATGGAGTTAATTTGTCTCTCCTCCGTA
4A-0952320-T6-31_576	AAAAAATGAGAAGATCCGAATGTGCCTG
4A-2108990-T6-31_576	CGCAGGTTTCAGTAGGCCAAGAGGAAAG
4A-0952320-T6-31_640	AGCATAGATGACCATGACATGGTGTTGTT
4A-2108990-T6-31_640	CAGTGCTAGGTCCTCAGACATAGCCACAT
4A-0952320-T6-31_704	CAAAAAATTAATTACATTAGAGTGACGGA
4A-2108990-T6-31_704	ACAAATACAATGTTTGAAATTGTTCTTCAA
4A-0952320-T6-31_768	GATGGATGATCAGCACGAGAAGTTGTTAG
4A-2108990-T6-31_768	CTTCAGAACAAAATAGAATGCAGCAGCCA
4A-0952320-T6-31_832	AGTTGGAGGTCATCATGTTTGTTGATTGT
4A-2108990-T6-31_832	TACAAACAAATAAACATACATATTAAGGGT
4A-2108990-T6-31_896	CAAAAATGTAGAATTGTGATGTCATACAAT
4A-2108990-T6-31_960	TGAATAGAAATCGCTTGGTTCTAGCACAG
4A-2108990-T6-31_1024	ATTGTGTTAAACATGTTTTGTACTTTGTGT
4A-2108990-T6-31_1088	GGGATATGTATTAAAAGTTGTTTGGTTGTA
4A-2108990-T6-31_1152	AGGTGCATGAAATAGTTGTCACGGAAACA
4A-2108990-T6-31_1216	TACAAATAGCATGCAGCAGCCATATAAACA
4A-2108990-T6-31_1280	TTTTTGTAACAAACTACTCCTAAACATATG
4A-2108990-T6-31_1344	AAGCCATGCTCCGTACTATTTAAATTGTTG
4A-2108990-T6-31_1408	TTTGCCAAATTGCAGTCGGTTCGGCAAAT
4A-2108990-T6-31_1536	TATGTGAAGATCCGAATATGCGGTGGAAC
4A-2108990-T6-31_1580	CTAGTTTGTAGTACTAATTTGCAGTATACA
4B-1034400-T6-31_0	GGCCGCCGGCGATCTCTCAACTAAACTTC
4B-1034400-T6-31_64	CTTCTAGTACGTATATATAGGCATAAATAAT
4B-1034400-T6-31_128	CTACGTATATAATATCATATGTACGGCATCG
4B-1034400-T6-31_192	GGTTCGTACAAAAATATACGCGGAGTCAA
4B-1034400-T6-31_256	ACATTTCTGTTATTATTAGAAGGATTAAGG
4B-1034400-T6-31_320	AAATTTCAGTACTATAGAATTGTGGAAATA
4B-1034400-T6-31_448	ATACGGTGAGTTGTATCTTATGTTCTCACTA
4B-1034400-T6-31_512	GGGGTTAATTTGTTCTCACCTCTCTATAAC
4B-1034400-T6-31_576	CCGATTTCATTAAGTCAGGAAAGCAACAA
4B-1034400-T6-31_640	AATGCTAGGTCCTGCAGACATAAGCATAC
4B-1034400-T6-31_768	GTATCTAGAACTAAAATACatccatttctctgac
4B-1034400-T6-31_832	ACAGTTTCAGAACAAAATAACATATGCAA
4B-1034400-T6-31_896	TAAAAAGGGAATGTTTAGTTCTCATTGTT
4B-1034400-T6-31_960	ATTTGTATTAGAAGTTGTTTGGTTGTAACC
4B-1034400-T6-31_1024	CAACCCTTAAAATCAGCTATAGCGAAGCT
4B-1034400-T6-31_1088	GCGAGCAATTGTACATTGATTTCATTTAGC

4B-1034400-T6-31_1152	TTTTATTGGTTGTAAGACAGAAAAAATGA
4B-1034400-T6-31_1216	TAGTCACAGAAACATAGGAGAATCCCAA
4B-1034400-T6-31_1280	TGCAGCAACCATGCGTTTAGAACACTGG
4B-1034400-T6-31_1344	TTTTTAACAAACTACTAACATATGCAGATT
4B-1034400-T6-31_1408	TACTCCGTACTATTTAAATTGTTGACTTAG
4B-1034400-T6-31_1580	GGAACCTTGCCAACTTGTCACTAGTTTTT
4A-0989060-T6-32_512	CCGCCGGCGATCTCTCAACTAAACTTCTG
4A-0989060-T6-32_576	GTGCTTTGTAAGGTGCTTCTAGTACGTAT
4A-0989060-T6-32_640	AGGTACATTATTTCACTTCTGCTTCGGTAC
4A-0989060-T6-32_704	CGACATTGCACGTAGGTACAGCCGTACAT
4A-0989060-T6-32_768	TATAAAATAGAATACGAGAATGTAAAATG
4A-0989060-T6-32_832	GAATGCATTTCAATTATCACAAGGATTGC
4A-0989060-T6-32_896	TGACTTTGAAGTTTCATATAGATAAAATTG
4A-0989060-T6-32_960	CAAATGGAATATAAACATAAGCTGGATAT
4A-0989060-T6-32_1024	CGAATAGTAGTAGGCTACTACCATTAGTTG
4A-0989060-T6-32_1088	AAAATGAGAAGATCCGAATGTGCCTGAG
4A-0989060-T6-32_1152	CATAGATGACCATGACATGGTGTTGTTTA
4A-0989060-T6-32_1216	AAAAATTAATTACATTAGAGTGACGGACA
4A-0989060-T6-32_1280	TGGATGATCAGCACGAGAAGTTGTTAGG
4A-0989060-T6-32_1344	TTGGAGGTCATCATGTTTGTTGATTGTGTA
7A-1759120-T6-33_0	TGACAACGTGACATTCTTGTTCTATAGGC
7A-1759120-T6-33_64	AATCCACTTAGAATGGTGGGGCGACGAG
7A-1759120-T6-33_128	TCACATAGGGGCTTGCAGTGTCGAGTCT
7A-1759120-T6-33_320	GCTTATCTTGGTCTCTGACCAAATAAGCC
7A-1759120-T6-33_384	CGGTGCTGCCTAGTCCCAACTGTTGTGGG
7A-1759120-T6-33_448	TCCACATTTGTTGCTCTGGGGGCTGAAAG
7A-1759120-T6-33_512	CATGTGCTCTAGAGGTTGTGGGTGTCGGT
7A-1759120-T6-33_576	TTGCATGATTGGCTCACCGGTTAGCAATG
7A-1759120-T6-33_640	GATGACGAGGGGTGTATCGACTGGTGAT
7A-1759120-T6-33_768	CCTAGGTACCTCGACATGGCACGAGCGA
7A-1759120-T6-33_832	TGGGTATCATGCCTCTCTCTGAAACGGCG
7A-1759120-T6-33_896	AACCCAAGTCCATCTTGTTGGTTTCGCCG
7A-1759120-T6-33_960	GAATCGTGGAGTTTAGTACCTTTAGGCAA
7A-1759120-T6-33_1152	GCGACAGTTTGGTCGAGGTTGTGCAATG
7A-1759120-T6-33_1216	CTTCTTCAGTGGCCACTGGTGTTTCTTGC
7A-1759120-T6-33_1280	GCAAGTTGATGGAGTCGAGTACTGCATAT
7A-1759120-T6-33_1344	TGATTTTTTTTCTTTAATCTACTTTGTGAGT
7A-1759120-T6-33_1408	GTGGCATTATATATAAAGCGGGATAAAGG
7B-1884660-T6-33_64	ACGACGCGACATTCTTGTTCTATAAGCCG
7B-1884660-T6-33_128	TCCACTTAGAATGGTGGGGACGATGAGA
7B-1884660-T6-33_320	AGGGGGCATGTCCTTTGGCTTATCTTGGT
7B-1884660-T6-33_384	TGCTTGTGGGTCAACATCGGTGCTGCCTA
7B-1884660-T6-33_576	GGATCCTGGATGGCACAGTTGCATGATTG
7B-1884660-T6-33_640	ACTCCTCTTGATTGTGTGGATGACGAGGA
7B-1884660-T6-33_768	GAGCGAGATGCTGAGCCTCGAAGCAGTC
7B-1884660-T6-33_832	GACGGCGTGTAGCCCCCTCCCCATACCAG
7B-1884660-T6-33_896	TTCGGCAGCAGCAATGTTGTGGTATTGTC
7B-1884660-T6-33_960	TCAGGCAAGCTGGACTCGTAGTTCGAAG
7B-1884660-T6-33_1152	TGCAACGATGTCTGGAGCTTTCTTAACGA
7B-1884660-T6-33_1216	TTTTTTGCATGGGTTGGGCTAATCTTTGTG
7B-1884660-T6-33_1280	CTGCATATCTTGTGCATTGCTTTTGTGTTG
7B-1884660-T6-33_1344	ACTTTGCGAGAGTTTTTTTTCTCATCACC
7B-1884660-T6-33_1408	TAAAGCGGGATAAAGGTTTTTTTCCTTTG
7B-1884660-T6-33_1472	TCCCAAAAATGAGGAGTCGTTGAAGACT
7D-1997730-T6-33_0	TAACGAGCGACTAGGGCCGTTGATCTGC
7D-1997730-T6-33_64	TCGAGACTTGTGGGCTAGCCATCACCTGG
7D-1997730-T6-33_128	GTTTTTTGTGGCGCTGAGCGGGCACGTG
7D-1997730-T6-33_192	CGCCCCCGTTCAGCTTCGTCGATGCAGGG
7D-1997730-T6-33_256	TATGCCCTGGCCAGGCGTCTTTTGTGCTT
7D-1997730-T6-33_320	TACGGGCAGGGGGATGGCCTAGCCCTGG
7D-1997730-T6-33_448	GGTGTCGGTCCTGGATCCTAGGGTGGCG
7D-1997730-T6-33_512	TAGTAGTGTGGACGTGTTGGTTGCTACTC
7D-1997730-T6-33_576	TGGTGATCCTGGCAAGGTCGTTGTAGTCG
7D-1997730-T6-33_640	TTCTCGGGGTGGGTTTGATGGACTAGGTA
7D-1997730-T6-33_704	TGCAGGCGGCGGTGACGCATGTGGGTGT
7D-1997730-T6-33_768	CCGTGCCAGAGCTCCCAGTGGAAACCCA
7D-1997730-T6-33_832	CCATTGTCAACCTCTTGGGGTGTCATCGT
7D-1997730-T6-33_896	TATATAGGCAAGCTTGACTTGTAGCTCGA
7D-1997730-T6-33_960	GCCTCCTACCTTTTACATGTGACTGCATCG
7D-1997730-T6-33_1024	ACCAGCGCTCCTCAAACCAGAGCGAGAG
7D-1997730-T6-33_1088	TCGTGCAACGATGTACGGAGGTTTCTCAA
7D-1997730-T6-33_1152	GTATTTCTTGCTAGGATTTGGCTAATCTTT
7D-1997730-T6-33_1216	GTAGTGCATATCTTGTGCATTGCTTTCTTT
7D-1997730-T6-33_1280	ACTTTGCAAGAGTGTTTTTTCTTCCATCAC
7D-1997730-T6-33_1344	actcaactttgtactaagtttagaaaaagtATTACCA
7D-1997730-T6-33_1408	ACATTTTTTTTCTTCGAATATTGCCTTTTAT
7D-1997730-T6-33_1580	TACCTCCCCTTCATCGTCCCGTCTCAAAAC
6A-1564640-T6-34_576	TGACCTCTTTCTTGCTTTCTTTTTTAACAT
6A-1564640-T6-34_640	AACATGGTGACCTCATTTCAGACACCTAA
6A-1564640-T6-34_704	CAACCCGTGATTAGATAGCTATAGGAAAA
6A-1564640-T6-34_768	ACTTAATATTGATGCTTACAATATTCATGG
6A-1564640-T6-34_832	GTTCCCGAGTCAACTGGCTGCTAGTTTTT
6A-1564640-T6-34_896	CTGCGGGAACCACTAGCTGCTTTTTTTTT
6A-1564640-T6-34_960	AAGAACGAATATCATAAATGTTTCTTCATC
6A-1564640-T6-34_1024	CTCGGATTGGATCATGGTAACAGGCTTGG
6A-1564640-T6-34_1088	AAAATTGGCCAAGAACGGAAAACAAATT
6A-1564640-T6-34_1152	TGAGGGTTACCACTTACAAAGGTCACTCC
6A-1564640-T6-34_1344	ctttgaaacaagatcctttattaataggtttcaacaaa
6A-1564640-T6-34_1408	AAAAACAGAAACCAGAAAAATATGGAAA
6A-1564640-T6-34_1472	AAAAAACAGAAGAAAAAACGGATTCCGA
6A-1564640-T6-34_1536	GGTGGGAGGGGCACATGTTGTGTTTTCC
6A-1564640-T6-34_1580	GGTTGTATTTCTCATGAAAGCACATGCGA
6B-1635270-T6-34_192	TGCGCCGAGCCGGAAGATGAGTCGGAAG
6B-1635270-T6-34_576	CGGACGGCGCAAGCTCGCGCGACCACGG
6B-1635270-T6-34_832	ACCAACATCGTGACCTCTTCTTGCTCTCTT
6B-1635270-T6-34_896	CAAAGGGTACCTCTTTTAACATCGTGACC
6B-1635270-T6-34_960	CAGACCACGTGAAACATGTGGTAAGCAA
6D-1725630-T6-34_576	CATCGTGACCTCTTTCTTGCTTTCTTTTTA
6D-1725630-T6-34_640	TAATCAAGGGATACCTCTTTTAACACGGT
6D-1725630-T6-34_768	ACCAGAGTTCAAGTCTTAGACTTAATATTG
6D-1725630-T6-34_832	TTTCTCTGGCTGCTAGAAAGTTCCTAAGT
6D-1725630-T6-34_896	AGCTGCTATTTTTTTCTATGAAAACAACCT
6D-1725630-T6-34_960	TAAATGTTTCTCCATCTGGCGCACAGTCTT
6D-1725630-T6-34_1024	TGGTAATAGGCTTGGGCGCCGCGAAAGC
6D-1725630-T6-34_1088	GCGCAAAAAAAATTTGTTCGCTCAAAAAC
6D-1725630-T6-34_1344	tattaataggttttagtaaactttttCCCATGAAAA
6D-1725630-T6-34_1408	ATGGAAACAAAAAAACTAATCCAAAAAAT
6D-1725630-T6-34_1472	ACCAGAGTCCGAAAGCAAGGTTACGGTT
6D-1725630-T6-34_1536	AGCGCATGTTGTGTTTTTCCTTTTTGGGG
6D-1725630-T6-34_1580	TTCTCGTGAAAGCACATGTGATGGTTTTA
3A-0638220-T6-35_0	TGTGAAGGTGAAAAAAATGCTATAAGCTG
3A-0638220-T6-35_128	CAACCGGTGCGCCAAAGTCTCTTTCCCAT
3A-0638220-T6-35_192	ACCCTTGCATCACACGAAGGTTACCTGCT
3A-0638220-T6-35_384	TGCGCATGCAGCCGCAGCATCTGCACTTT
3A-0638220-T6-35_448	GCGTACGTACGTGGCCATGTTCATGCATG
3A-0638220-T6-35_576	CAAGTGCTAGGGTAGTGGCATTTTAACCT
3A-0638220-T6-35_640	TGATTCGCGTGTCTAATCCGATGTTTCTTC
3A-0638220-T6-35_832	GTCCAGAGGAGGAACATGCTTCACACTC
3A-0638220-T6-35_1024	AGACGTCGTAGTAAAAGTTTGGCATGGC
3A-0638220-T6-35_1088	GACGGGAACATGCATAGTTCATTAGCAG
3A-0638220-T6-35_1152	GATCAGGTTCACACGATCACATGGTTAAT
3A-0638220-T6-35_1216	TCCATATTGTTTTTCTTCTTCCCAGTGGCG
3B-0789720-T6-35_0	TTCTGCTCGCGTAATTTTGTGTTACTTGGC
3B-0789720-T6-35_192	CCACTAACTAACCAACGTTTCCTGTACATC
3B-0789720-T6-35_384	GGAGCAAACCAGATCTTGTATGTACATGC
3B-0789720-T6-35_448	TTGCAGTTTGGTCTGAAGATACCGATTTC
3B-0789720-T6-35_512	TCATGCATGTGAAGCCGAGAACCTTAGTA
3B-0789720-T6-35_576	CTTATCATTTGGTGTTGGACTGGTAGTGCT
3B-0789720-T6-35_640	TTTTAACCTCGTTAGATTAATCTGTTTAACT
3B-0789720-T6-35_1024	CGCGACCTTGACATCCTGTCCCTGCCACA
3B-0789720-T6-35_1152	ATCCTGATTGAATCGTGATATATGCCGATC
3D-0873340-T6-35_0	ATGCCACTCCAGTGAAGTCTTAAACTAGT
3D-0873340-T6-35_64	AGTGGGTAGCGTGACTCGCCAATGAGCA
3D-0873340-T6-35_256	CCTCCCTATCATGAGAGCTCTTATACAACG
3D-0873340-T6-35_320	GCCGGCCTAGCTACTCGGGGTCGCTCGCT
3D-0873340-T6-35_384	catagaccagttgaccattgacagttgacTTTTAGA
3D-0873340-T6-35_448	GTGAGTTTGAAAAAAGTTGATTTTTTTGA
3D-0873340-T6-35_512	TGAAAAAAAATTACTAACTCGAAAAAAGT
3D-0873340-T6-35_576	GGTTTGCCTATATGAAAAAAGTTTGCGGC
3D-0873340-T6-35_640	AACATGAGAAAAAGTTCAGAAATTCAAG
3D-0873340-T6-35_704	AAGTTTGTGGCTTCAAAAGAATGTTAACG
3D-0873340-T6-35_768	AAATTTGGAAAAATGTTCGAGAATTTGAA
3D-0873340-T6-35_832	AAAAGCAATGAAGGAAAAAAGGAAAAA
3D-0873340-T6-35_896	AAATAAAAACGGTTGAAATAAACCACTGG
3D-0873340-T6-35_960	GGTAGAGAAAAAAAAGAGCCTACATGGG
3D-0873340-T6-35_1024	TTAATATTGTTTCTACTGCTGTAAGCGCTG
3D-0873340-T6-35_1088	TTGAATCCAAATGACTGAAATGGCTTGGC
3D-0873340-T6-35_1152	CAATTTTCATTTATTGATCGGTGTGGCAAC

3D-0873340-T6-35_1216	TTTTTTTCTAAAACAAGGTAAAATATTTGC
3D-0873340-T6-35_1280	AAAGAGACCCAAATGAGTTCATTACACTC
3D-0873340-T6-35_1580	CGCATGACCCTACCTGTCCTCTTTGCCTGA
4A-0961700-T6-36_128	GACGAAGGGGCATGGGTATTTATAGATGG
4A-0961700-T6-36_320	TACGTACGTGTAGTTCCATGCACCACACAT
4A-0961700-T6-36_448	AAATCACAGCTACTACCTTTTTGGCGAAA
4A-0961700-T6-36_576	TGCGAGGAAATTAAATCTAGCACAGTTGT
4A-0961700-T6-36_768	TGTAAGCTAGGGCAGTCACATATGTACAG
4A-0961700-T6-36_832	GAAAAAAGAAAAGTCCATGGAAAAATCT
4A-0961700-T6-36_896	AAGAAGGATCCATAGACTAGTAGGCGGT
4A-0961700-T6-36_960	CTGGACGATATGTAGGAAACCAAAAACT
4A-0961700-T6-36_1216	CGTAGACTGATCTGAAGCAAGTTTCCATG
4A-0961700-T6-36_1280	AAACCTTTTGAAAATGTTAAACAAACATA
4A-0961700-T6-36_1344	CTAAACTCTTCGGTTATGATAATTAAGCCT
4A-0961700-T6-36_1408	TGTTTCAGCGCATATATTCCAATGTTTGTT
6B-0550820-T6-36B_64	TAGCCACAGGTATAGCAAAAATCACGCAG
6B-0550820-T6-36B_1280	CCCCTGATCGCCCGGGCTAGGAAACCCTA
U-0550820-T6-36A_128	ctctcctccctGTTGTTCCTTCTCCTTGTTGCT
U-0550820-T6-36A_1344	AATGAAGAAGAAGGAAGAGTTGTTCTTG
7A-1797770-T6-37_128	TGAAGCCAAAGCTTGAGAAATTCTCCAGA
7A-1797770-T6-37_192	ACAAACTAGCAGCAGGTTTGACCATTACT
7A-1797770-T6-37_256	AAGAAAAGTATGACCAATGAAGCCTAAA
7A-1797770-T6-37_320	AGAAATTCTGCATGACTGGACAAACTAGC
7A-1797770-T6-37_1280	CCTCGGCTCCATTGCATATTTTGCACAAAC
7A-1797770-T6-37_1344	CTTGGTTGGTATTGAGTTATGAGCCATCCT
7A-1797770-T6-37_1408	GTCCCCCATAAGCCGCACCATTGTTGCTTT
7B-1866300-T6-37_0	TGCTGCAAGCTCTAGTTAGCTTCAGTGCT
7B-1866300-T6-37_64	TGAATTTGTTAGGACAGAGCCATGCGTAT
7B-1866300-T6-37_128	TGAAGCCAAAGCTTGAGAAATTCTCCAGA
7B-1866300-T6-37_192	GGACAAACTAGCAGCAGGTTTGACCATTA
7B-1866300-T6-37_256	ATAGATAGCAGATGAGAAGAAAAGTATGG
7B-1866300-T6-37_448	GTATTTGGATGCTTCTTGCCGTGTTCGACT
7B-1866300-T6-37_512	ACATTTCACATGGACGCACGTACATTATAT
7B-1866300-T6-37_576	CAACGTATGTGTGTACCAATGCTCCGGAG
7B-1866300-T6-37_768	CAAACTGTGGTACTCAGTATGTGATGACC
7B-1866300-T6-37_960	GCTCGTGACAAAACCAGAGTCGCTATGCG
7B-1866300-T6-37_1088	TTTTTCGTCTGTCCACTTCGTCCATATTATT
7D-1985660-T6-37_384	CAACGACGTCGCCACACACGCCTGCAGT
7D-1985660-T6-37_448	AGCCTCTATACGTCGCACCGCGAATCAATA
7D-1985660-T6-37_512	AAGGTGTACTGCTTAATACATCACATTGTC
7D-1985660-T6-37_576	ACTATGAGCGTTTCATTGATATTTGTGTAC
7D-1985660-T6-37_640	CTTAGATTGGACAGCACCCTGACGCAACA
7D-1985660-T6-37_704	TCACGACATTGACATCGGCGTGCCAAAGG
7D-1985660-T6-37_768	TACGCGGCCATCATGTGTACAGCTAGCTT
7D-1985660-T6-37_832	TCCTGCATGTTGATCCGTGGTGTTTGTTTT
7D-1985660-T6-37_896	TGCATCATGTCATGATGTCAAAGGCCTTTT
7D-1985660-T6-37_960	CCACGCGAGGCGAGTTCTCGACCCGAAG
7D-1985660-T6-37_1088	GTTGGTTCGCTGCCGCAAGCGATGCTGA
7D-1985660-T6-37_1280	CACCGGTCGAGCGTTGGGAACAGCCTGA
7D-1985660-T6-37_1408	GGAGGAGCTGCAACGTTCTTCCCCCGCG
7D-1985660-T6-37_1580	CACTGGGGCCTTATATAGAGGAGATCAAA
3A-0690110-T6-38_51	caactatatcgatcctttcatctggcaccggCTTCAC
3A-0690110-T6-38_102	CGCGGCGGTGACCTCAACTGCGGCCGGT
3A-0690110-T6-38_153	GAACCATGAGTTTTTGGCGAGCTGCAAC
3A-0690110-T6-38_201	CCCGGGCCTATGGCGAGCCGTGGTTGCA
3A-0690110-T6-38_651	GGGTGGAGGGGGGTCGTTGGGCTGTTG
3A-0690110-T6-38_713	CGGTAGACGCGAGAGGATGGAGTTTGGG
3A-0690110-T6-38_775	TCTCAGTTACAGGACCACCAAATCCAACG
3A-0690110-T6-38_837	GTGCTAGGCGACGGTCCGCCGGCGCCTC
3A-0690110-T6-38_899	TCGCCCTTGCAAAAAAAAAAGAGAAATC
3A-0690110-T6-38_961	ACATGATGCCAGGAGTGACCCAGAGAAA
3A-0690110-T6-38_1333	TTACACCATCTAGATCCGCCCCTGGGAGG
3A-0690110-T6-38_1395	GGCACGCGCTGCAATTTTTGTTTGCTAGG
3A-0690110-T6-38_1457	CTTGAATTTTGTCAAACATGATGTAATGAT
3B-0798870-T6-38_128	AACCATCAGTACGAGAAACAAATTTGAG
3B-0798870-T6-38_192	AATTCGAGATATCATATGACCCGAAGTCT
3B-0798870-T6-38_256	GTGCCCAATAGCAGTATAATTATACGGGT
3B-0798870-T6-38_576	GGGGGATTTTCTTTTTTCCGCCGAGATCC
3B-0798870-T6-38_640	TTTTTTGACGTCACCGGAGGGCAGCTTTG
3B-0798870-T6-38_704	GTCACTCCTTGGCATCATGTTTTAAACTAG
3B-0798870-T6-38_768	CATTTCTGCAAACCGCCTTGAGGGGTTGA
3B-0798870-T6-38_832	AGACCGGGTTGTCCTCATCCTCTCGGCAT
3B-0798870-T6-38_896	CTAGCGCTCTTCTCCCCCCAGCGCCTCCCT
3B-0798870-T6-38_960	GCTACTCCTGTTATGAAGGGGGCACCATT
3B-0798870-T6-38_1024	ATGAAGTCGCTGCTGCAAAGGGGCGACA
3B-0798870-T6-38_1088	GGGGCTGCGTTGCAGCAAGCCACCAGTG
3B-0798870-T6-38_1280	TCATGGCAGTCGAGGTTGTTGTAGGGAG
3B-0798870-T6-38_1536	GCGTCGCGAGGGTCGTTGAAGCTTCGTC
3D-0913190-T6-38_128	TAACCATCAGTACGAGAAACAAATTTGAG
3D-0913190-T6-38_192	AAATTCAAGATATCATATAACCCGAAGTCC
3D-0913190-T6-38_576	ATTTGGGGGATTTTATTTTTCCCTAGATCC
3D-0913190-T6-38_640	TGATTAAACAAAAACAAACTATGTACTGTT
3D-0913190-T6-38_704	ACTAGGAAAATGTTTTAATTTAAACGGAT
3D-0913190-T6-38_768	ACACGTGTCTGGTGGGACCGATGCACTG
3D-0913190-T6-38_832	TTGTCCTCATTCTGTGGGCATTCCCGATCT
3D-0913190-T6-38_896	CTTCTCCCCCCGAGCGCCTCCCCGTTGTC
3D-0913190-T6-38_1088	GTTGCAGCGAGCCACCGATGTTGCAGCC
3D-0913190-T6-38_1152	ATGTTGCAATCGGGAGGCTGGGGAGAGG
3D-0913190-T6-38_1344	CGTTGTCGCAACAGAGGAGGTCGTCGGC
3D-0913190-T6-38_1408	AGATGGGGGCTCGCCGGCGCTGCGATGA
3D-0913190-T6-38_1536	GGGTCGTTGAAGCTTCGTCGAGGTTGCAT
4A-1025320-T6-39_0	TTGTGTATTGAGAATGCAATTCAGTTTGCC
4A-1025320-T6-39_62	GACAGAGGTTGTTTACTATTTGTTATTCAA
4A-1025320-T6-39_930	GTAGGTTTTGATGCATAGAATGATTTGGTT
4A-1025320-T6-39_992	ATACGAAGGAGTTTGAGAAGTTGGGGAA
4A-1025320-T6-39_1054	GCTGAAGTTACAAAGGTTGCCACCCCCAA
4A-1025320-T6-39_1116	CCATTTTTCATCTCGTGGAAACATGTTCAG
4A-1025320-T6-39_1302	GTTGAGAAGCAGAATTTTTAGAGTTGAGA
4A-1025320-T6-39_1364	TCAGAAAATGGTTAAAACATGAAATTTAG
4A-1025320-T6-39_1426	TCGATTCAATAGTCTATGTGATCACTTAAA
4A-1025320-T6-39_1488	ACGCCCTTTAGAATTTACTTTGCCCTAAGC
4A-1025320-T6-39_2294	TGCCTGTCCCCTGGGGTTTAATTCAGATC
4A-1025320-T6-39_2356	CTGCGAGTTAATAGCACATAGTACCACATG
4A-1025320-T6-39_2418	TATATATGGTGTCATACATAGATTGCACAGT
4A-1025320-T6-39_2480	CTGTTGAAAAAGGGCATTTGTTGTTGTAT
4A-1025320-T6-39_2542	TATCGATGGTAGAAAGATGGTCATTGCCC
4A-1025320-T6-39_2604	TAGAGGATCCTGCATGCGTGTGTCTATGC
4A-1025320-T6-39_2666	GTATTGACTTTACACTTGAGAGATGGTAA
4A-1025320-T6-39_2728	AATCTGCTTCTGTTCTATTACTTTTTTTTGA
4A-1025320-T6-39_2790	TAGTATTCTTTCTCATAATTTATCTTGTGAT
4A-1025320-T6-39_2852	GAGAAGAAGGAGAAGGACAAATGATATA
4A-1025320-T6-39_2914	CAACTTTATGACTCTTGCTAACACGTTTTA
4A-1025320-T6-39_2976	GAGATCCTGATCACACTAGTTAGATTGTTA
4A-1025320-T6-39_3038	GCATGCTTGATATAGCTACAGTTGCCTATG
4A-1025320-T6-39_3100	ATAGGTTCCCTTTATTAACTCTTCATTTCTT
4A-1025320-T6-39_3115	TAACTCTTCATTTCTTAACCTGTGGTAGAT
4B-1064640-T6-39_0	CAAGAATTGTGGACAAGCGTTGCGTCTTT
4B-1064640-T6-39_64	ACCTTAGCACAAATGTTAATCCATTAATCT
4B-1064640-T6-39_128	AAACTATTACACCGTACGAATTGTAACCAT
4B-1064640-T6-39_192	CATGCATTTCTGCATCCTGAAATTAACCAT
4B-1064640-T6-39_256	CTCACGAGCAAAAAAACAAAGAAACGG
4B-1064640-T6-39_320	GTGTGGCCAGCAACATATTAAATTATTAAT
4B-1064640-T6-39_384	TATTTTTGTCTACTCCAATGGCAGATCATAT
4B-1064640-T6-39_448	CTAGGCCCTCAATTAGTATTAGCATTGTGA
4B-1064640-T6-39_512	TTAAACCATATTGCAATCTCAAAGGAGCG
4B-1064640-T6-39_640	GAGAACACGCTACCATACGTATGTCCTGAT
4B-1064640-T6-39_704	ACAACTTTTATTCACACTCTGAAGCTTCAT
4B-1064640-T6-39_768	TGGCAGCTTTCTATTCACTGTGCAATCTAT
4B-1064640-T6-39_832	ACACATCTTGGTACGCATGTGGTACTATGT
4D-1137010-T6-39_0	CAAGAATTGTGGACAAGCGTTGCGTCTTT
4D-1137010-T6-39_64	ACCTTAACACAAATGTTAATCCATTAATCT
4D-1137010-T6-39_128	AACCTATGGTTACACCGTACGAATTGTAA
4D-1137010-T6-39_192	AAGCATGCATTTCTGCATCCTGAAATCAA
4D-1137010-T6-39_256	GATCTCACGAGCAAAAAAACAAAGGAAC
4D-1137010-T6-39_320	GTTGCCTACTGGTGGCCAGCAACATATTA
4D-1137010-T6-39_384	TCTAAGCCATATTTTTGTCTACTCCAATGA
4D-1137010-T6-39_448	GAAAGAATACTAGGCCCTCAATTAGTTATT
4D-1137010-T6-39_512	GAAGCAGATTAAACCACATTGTAATCTCA
4D-1137010-T6-39_576	AGTCAATACTTCCTTTGAAGCTAGAAACT
4D-1137010-T6-39_640	ATCCTCTAGAGAACACACTACCATACGTAT
4D-1137010-T6-39_704	TCGATAACGCAACTTTTATTCACGCTCTGA
4D-1137010-T6-39_768	ACAGAAAGACAAAAGAAAATGGCAGGTT
4D-1137010-T6-39_832	TATATAGTCAAACAAGGAAACACATCTTG
4D-1137010-T6-39_1408	GGAGGAGATGACAGCAAGGGGGAATTCT
4D-1137010-T6-39_1580	GGCTTTTTGCGGATAGACCCCTTCGGCCG
1B-0153690-T6-40_320	gcggttgggccaaatttttgacgccagcgacaaaaaa
1B-0153690-T6-40_384	cgactcgagatgctcTAAGCACCATTCCTTGAA
1B-0153690-T6-40_448	TACTAAATAAACTTATGTTTTATTTGTTAAG

										1B-0153690-T6-40_512	AACAACATCGTTCCTTTTTTCTTTTTGCTA
										1B-0153690-T6-40_576	CTGGGCATAATTCCTTGGAAACGGTCTTA
										1B-0153690-T6-40_640	TAAAGTTATGTTATCTTTGGTAAGCCAAAA
										1B-0153690-T6-40_704	ATTATTATAGACAGTGTTTTTCAAACACTAT
										1B-0153690-T6-40_768	CATCACATCAATACGGGCAAGTAAATCAT
										1B-0153690-T6-40_1152	AAAAAAAGCAATTATCTAGTGTGTACAAA
										1B-0153690-T6-40_1280	AAAGGCAAAGGGAAAATTTGCAACTCAC
										1B-0153690-T6-40_1472	TTCCCCCCGCTTTCCATTCCTCCGTCCCTT
										1D-0253170-T6-40_0	GTAAGCTACCTACAAAAATTTGCAATCAA
										1D-0253170-T6-40_64	AAAGCATATTCTAAATGCATGACTTCTTGT
										1D-0253170-T6-40_192	CTTGGATTCATACTAGAGCCACGATGGTTT
										1D-0253170-T6-40_320	AACTTCTTCAGTGACGCATGGCAGTTTCTT
										1D-0253170-T6-40_448	CATCCCCTCCCAAGGAATGTTCGCCAGAT
										1D-0253170-T6-40_512	TTATAGCAAGTTATACAGGTGTCGATTTAT
										1D-0253170-T6-40_576	CATAGGTGTAAGTGTAAGCCCATCCAAGC
										1D-0253170-T6-40_640	CTAGGTGGACATAATCATGTTGATACCTCT
										1D-0253170-T6-40_704	ACGTGGGGTGCTTCATCCAACCTTTCATC
										1D-0253170-T6-40_768	CTTACTAAATAAACTTATGCTTTCTTTGGT
										1D-0253170-T6-40_960	TGTTTTCTTTGATAAGCCAATAAGTTATTAT
										1D-0253170-T6-40_1024	TAGGTATGTTACAGATAGTATTTGGTAGAC
										1D-0253170-T6-40_1088	AAATCACGGTGTAACATACTGTACTAGAAT
										1D-0253170-T6-40_1152	cctccgtttcaaaatagatgacccaactttatactaGA
										1D-0253170-T6-40_1216	CCATACCAATTGCCTCTCCAACCTCTCACG
										1D-0253170-T6-40_1280	AGAAAGGCAAAGGGAAAATTTGCACCTC
										1D-0253170-T6-40_1472	CCCCCGCTTTCCATTCCTCCGTCCCTTCCT
										1D-0253170-T6-40_1536	CCCTGCTGCCTGTTTTGTTTCCATCCTCCG
										4A-0942630-T6-42_0	GTGTTCTAACTTCCGAGAAGGCTAAACTT
										4A-0942630-T6-42_128	CTAGCCACCTAGCCACCGCACGATGGGCT
										7A-0801380-T6-42_128	AAATACCAAAAGTTGCCATCTTCTAAACAT
										7A-0801380-T6-42_192	TATCTCCTAAACATCATCTTTGTCAAAGAA
										7A-1850170-T6-42_192	TGAGCCAATTGAGACAGCAAACAGCAAG
										7A-0801380-T6-42_256	GCACAACATAACCAACCACGCAAACCTTC
										7A-1850170-T6-42_256	TCCCTAATTTTCTGCTCTCATTCTCCTATATT
										7A-1850170-T6-42_320	TCAAAGTTAAATGCTGATTTTTTTAGGGA
										7A-1850170-T6-42_384	TGAGCATAAGAATTAGGCCATAAATGATTT
										7A-0801380-T6-42_448	ATTTCTTCAGCGGACTCCGCGAAAGACTC
										7A-1850170-T6-42_448	AAAGCTACATTGCAATATTGGTTGTATTGA
										7A-0801380-T6-42_576	ACTGGAGGTCTTTTAAGACCATATGTTGTT
										7A-0801380-T6-42_768	GCTCTTGCATCTCGTAGATGCAAGAGAGG
										7A-0801380-T6-42_832	AGATCACGGAAGAAGGCTGCTATTTTTCT
										4A-0942630-T6-42_896	GACGCCGAGGAGGAGCAGCGTGGGGCG
										7A-0801380-T6-42_896	GAGATGAATAGTAGGTGATGAGATAGACG
										7A-0801380-T6-42_960	TTTACCAGGGAAGAGAAGGTCAATTGTA
										7A-0801380-T6-42_1024	GAGAAGGGCTCTCATCCATGGCGGTCAA
										4A-0942630-T6-42_1152	TCAGTCTTCGTGGCCGGCTTTAACGTTTA
										7A-1850170-T6-42_1152	GTCTGTCCAAACATATGAGACGGGTTTGA
										4A-0942630-T6-42_1216	gtttttaattttctttttcgtttcttttatcttACAAGAA
										7A-1850170-T6-42_1216	GGCGGCACCTGCTCTGCTCTGCTCTGTTT
										7A-0801380-T6-42_1280	CCTTAGGCGTGGAGAGCTGAGGCTCTGA
										7A-1850170-T6-42_1280	GTGTAGCAAAATTATCTATGTATTGTGATC
										4A-0942630-T6-42_1344	CCCTTTTCTTGCTAGCTTTAGTTTGGTGGG
										7A-0801380-T6-42_1344	GCCGACGACAACGAGTGAAACCGGCGG
										7A-1850170-T6-42_1344	CGAGATCTCCCTTTTCTTGCTAGCTTTAGT
										7A-0801380-T6-42_1408	AGATCGACCGAGATCTCCTTTTTCTTGCTA
										7A-1850170-T6-42_1408	CAACAAACAAACATCGTCCAATCCTGTGG
										7A-0801380-T6-42_1472	ACCGGAGGATACCTCCAACAAACAACATC
										7A-0801380-T6-42_1536	TGGATAACCTGCCGCTCCCGGAAAGAAA
										4A-0942630-T6-42_1580	ACGGCTCAACTCCACGTCCTGGAAACATC
										7A-1850170-T6-42_1580	CCCGGATCAACTCCTCGTCCTGGAAGCAT
										7D-2062490-T6-42_64	GAATATAATTTTTATATTGTATAACTTTAATA
										7D-2062490-T6-42_128	GATCAAAGTCGACGAGCTTACCATTCGGC
										7D-2062490-T6-42_192	CTCCACCTCCGTCAGCTCCGACAACTCTT
										7D-2062490-T6-42_256	ACGGGAATTGAGCAGAGAGGGTGAGGA
										7D-2062490-T6-42_320	CCAAATGCGCGGGCTCCGCCATGATTTTG
										7D-2062490-T6-42_384	GTGGAGTGGGGGCAAATAATAGGAGCGG
										7D-2062490-T6-42_512	CACTCAGCACTATGTAGGTCCGCCTCTGG
										7D-2062490-T6-42_576	AACTCCCGCAAAGCCCCTTGGTTTGCGTC
										7D-2062490-T6-42_640	AGGACAAATAAGAGTCCATGTTGAATCTG
										7D-2062490-T6-42_704	GGTCTGGGTTGAATGGAGTGTTGTATGCA
										7D-2062490-T6-42_832	GTTTCGCCCGAAAACGGGGAGCGCCGCT
										7D-2062490-T6-42_960	GGTCCATGTAAGATCGGAGTTAGGTTCGG
										7D-2062490-T6-42_1024	CTGCAGAAGAGCCAGCTTCTTCAGCGTG
										7D-2062490-T6-42_1152	CCCGCTACAGCAGTGTGGCTCCGGTTGG
										7D-2062490-T6-42_1216	TTGATTAGTTTAAGAGGTGAGGCACTATC
										7D-2062490-T6-42_1280	TGATCGGATCAGCACTGTTGTGCCTAACTT
										7D-2062490-T6-42_1344	TTAGTTTGGTGGGTaaagaaagaaagaaaga
										7D-2062490-T6-42_1408	ATCATCCAATCCTGTGGATTCCCATACCAC
										7D-2062490-T6-42_1580	GCCACCTTTTTGCCCGCTATAAATACTTGG
										4A-0982100-T6-43_0	TGCTCGATCCCGCCGTGCGTCTCCTCTTTA
										4A-0982100-T6-43_64	GCTAACTGCTTTACTAGCACTGATTGCCG
										4A-0982100-T6-43_128	TGGCGGGATTATATAGAGTTTGGAGTTGA
										4A-0982100-T6-43_448	acttaagataatattatcttatcaaccattgtagatgcc
										4A-0982100-T6-43_512	CGGGTGATACCATCACGTGTATACTCCCAT
										4A-0982100-T6-43_768	ATGTAATTTGACTTTGAACTTTGATCTGAT
										4A-0982100-T6-43_832	CATCCACTTCTTCTTTTTTTAAAATTTCTGA
										4A-0982100-T6-43_896	TCATGGGAGCATACGGTATCACCTGATATT
										4A-0982100-T6-43_960	CCTCATATGTCCGCAGACGTATTCGAGCA
										4A-0982100-T6-43_1024	TCGACGACGTTGCAAGCACAATCCTCAAA
										4A-0982100-T6-43_1088	CATGTGGAATTCATCTACGTACTAGATCAA
										4A-0982100-T6-43_1216	GGGCAGCATCTCCGGCTTATCTTCCTTGT
										4A-0982100-T6-43_1344	CAAGGATGGCGGCTTGCTCTGCCTCGAA
										4A-0982100-T6-43_1408	AAAATCCACCTCTGCCATGCCAAAGACAT
										4A-0982100-T6-43_1536	cacccgctcctcctcctccATCACCGCAAGCTCG
										4A-0982100-T6-43_1580	GTGCCTCCTTCGAGGCGAGCATCTTGGCT
										4B-1090980-T6-43_0	CTCGACTTGGATCTCCGCCCGGTGCTCAA
										4B-1090980-T6-43_64	ACCATGGCaggcgacgagattggattggaagtgg
										4B-1090980-T6-43_192	GCAGGATTCCGCGCGCCATCATGAATGCG
										4B-1090980-T6-43_256	ATGAGTTTTGGCGCAAGCCCCGTTCGTAA
										4B-1090980-T6-43_320	TTTGCCCCACATATGAGCTGGGTTTTGGG
										4B-1090980-T6-43_384	AGGGTTCGGTTGGGTGGCAAATTCGTGA
										4B-1090980-T6-43_448	GGGGCCCGGATGTAGATGCTCTAATAAGC
										4B-1090980-T6-43_640	gagtaatttggaacggagggagtaCTACCTATTG
										4B-1090980-T6-43_704	ATATTTTTATTCTAATATAACATAGTATAATT
										4B-1090980-T6-43_768	CATAGCTGCAGAAATAGGCCCCATTTTCTA
										4B-1090980-T6-43_1088	AGAAAATTTCTAAGGATTCTAATCCTCCAA
										4B-1090980-T6-43_1152	AACAACACAAGTTTGTGCTTCTTCACCTC
										4B-1090980-T6-43_1216	CTCATCTTATTTTATTTGCTTCCTACTATGTA
										4B-1090980-T6-43_1280	CATTTAACAAACCTATATTTTTCTCTTGGTT
										4B-1090980-T6-43_1344	ATAAGTCTAAACATTTGGCCTCTCCTATTA
										4B-1090980-T6-43_1408	CTAGAGAATCGAGTTAGTCACCCTCTCATG
										4D-1168460-T6-43_0	CACTGGCGCATCATACGACTGCCCATACG
										4D-1168460-T6-43_256	TCATGTAGGCACAAAATAAGTATCAAAAT
										4D-1168460-T6-43_448	AGAAGTCAAGCGCCAGGAGGAGGATGC
										4D-1168460-T6-43_512	AGGTCCCTGACGGCTTTGGCATGGCGGA
										4D-1168460-T6-43_640	CCAACCGACACCGTATCTACCTCACGGAC
										4D-1168460-T6-43_768	GCACGGCAGTCATCTACATCTCCAACAAC
										4D-1168460-T6-43_832	CGTTTGATCTAGTACGTAGATGAATCCCAC
										4D-1168460-T6-43_896	TACATTTGAGGATTGTGCTTGCAACGTTG
										4D-1168460-T6-43_960	GCGCCCGGATACGTCTGTGGACATATGAG
										4D-1168460-T6-43_1152	AAAAATCAGATCAAAATTCAAATTCAAAG
										4D-1168460-T6-43_1216	ATAGTTTCAACAAAGAAAAAAAAACAAG
										4D-1168460-T6-43_1280	TTTTCTTTTTTGTTTCTCTATGTGTACTTTG
										4D-1168460-T6-43_1344	TACACATATGTGTTGTCAACATTCACATTTT
										4D-1168460-T6-43_1408	TTTCAAATTGATATCACGGGAGTATACACG

4D-1168460-T6-43_1472	TCTTAGGTAATTAACCCAGGTAGTCAGGT
4D-1168460-T6-43_1536	CTCCAGCTTCCATCAACTCCAAACTCTATA
4D-1168460-T6-43_1580	ATACACATCCTCAAACCACACTACTCACC
2A-0299310-T6-44_64	GTCAACCTCACCGGCGACAACAACGACA
2A-0299310-T6-44_128	CATCGGCGGAACAACCCTTATCTAGTTTC
2A-0299310-T6-44_256	TTATATTATTTGCAATGATTATTTGTCTTTT
2A-0299310-T6-44_320	GTTTAGGACGCAGCGGGCTGTTTGGGCG
2A-0299310-T6-44_448	ACACATGGGTGGAATGCTTCCCGTGATCG
2A-0299310-T6-44_512	TAGCCGTCTCCGACCGATCTCCAGAGAAG
2A-0299310-T6-44_704	CGCCTAGCTCTCCCTTTCTTTTCCCCCTCA
2A-0299310-T6-44_768	TGTGCACCTACAGCACAAAGATTCCTCCC
2A-0299310-T6-44_832	GCTTGGCTGCGTGCCAGGGTCAGTGGAA
2A-0299310-T6-44_896	CGGCGGTTAAAGGCGCCCAACGACCGTG
2A-0299310-T6-44_960	CCCATCGCCGGCACTTCCACTGGCCAGG
2A-0299310-T6-44_1024	TCCATTCGTATGCGTCACGAATACAGTCC
2A-0299310-T6-44_1088	cccCTTTTACTTCCAGCCGGCCGCTCAAAG
2A-0299310-T6-44_1152	CACCGAAGACACCAGCAATAAGACTCCT
2A-0299310-T6-44_1216	AACACTGCACTAAAACGACCAGAAAGCA
2A-0299310-T6-44_1280	CCATCGGCCAAACAAACCTTCCTTGACAG
2A-0299310-T6-44_1344	ATCAACGACGCCCGCAGCCATCACCGGA
2A-0299310-T6-44_1408	AAGTTTCAATCCGGGATGGAAAAATCTCT
2A-0299310-T6-44_1580	AGCCCTCCTCACCCCGACGCCTCCTATATT
2B-0391920-T6-44_192	GCCATTGGAAGCCCCATGGTTTTCAGGA
2B-0391920-T6-44_256	GAAAGCTTTGCATGTCCGGGCCTACACTC
2B-0391920-T6-44_384	ACCACTCCATAGCCGTCTCCATATCGTTGC
2B-0391920-T6-44_576	CCGGCGCATCATCGCCTAGCTCCGCCTTG
2B-0391920-T6-44_640	ACGTTTGGTGTGCACCCACACTAGAAAG
2B-0391920-T6-44_704	TTTGTACTGTCGATCTTATCAACTTTGGAT
2B-0391920-T6-44_768	CGCCAAGGTCAGTGGAACTATCTCACGTC
2B-0391920-T6-44_960	TTCCATTCATGTGCGGCAGGAATATGTCA
2B-0391920-T6-44_1024	ACCGGTTGCCCAAAGCCTCTCGATGGTCA
2B-0391920-T6-44_1088	AGCAGACTCCTATAAAAGACTGACCTGAC
2B-0391920-T6-44_1152	GACCAGAAAAGCGCATCACCTCCCCCGG
2B-0391920-T6-44_1216	CCTTCCTTCCCTATACACATAACAATTGAG
2B-0391920-T6-44_1280	TCCAGTGATCGGCGACGAGGCACGCCAG
2B-0391920-T6-44_1344	CCAGGTTTCAGACAGCAGGGCCGAAGG
2B-0391920-T6-44_1408	CGTGGATCCTGGATGGAAACATCTCCCAA
2B-0391920-T6-44_1580	CCTCCTCGCCCCGACGCCTCCTATATTTAA
2D-0546500-T6-44_256	TAGATCAGATCTTTTCGCAAAAAAACAAA
2D-0546500-T6-44_384	TCATAGCCGTCTCCAACCGATCTTCAGAAT
2D-0546500-T6-44_576	GCATCATCTCCTAGCTCTGCCTTCCTTTTC
2D-0546500-T6-44_640	TGGTGTGCACTATGCACCCACGGTACAAA
2D-0546500-T6-44_704	ATTCTTTCTACTATCGATCTTATCAACTTTG
2D-0546500-T6-44_768	CGTGCGCCAAGGTCAGTGGAACTATCTCA
2D-0546500-T6-44_832	GCAGCGAAAAGAGCCCAGCGAACATGCG
2D-0546500-T6-44_1024	CTTTTTACCTCGCACCGGTCGCCCAAGGC
2D-0546500-T6-44_1088	CACCGAAAACATGAGCAAGCAGACTCCT
2D-0546500-T6-44_1152	ACACTGCACTAAAACCGACCAGAAAGCG
2D-0546500-T6-44_1216	TGCCCTCCCTATACACGTACCCACGCTGGA
2D-0546500-T6-44_1280	TCCGCCTTCGTCCATCCAGTGATCGGCGA
2D-0546500-T6-44_1344	GACCATGGATGAAAACAAGTTTCAATTTC
2D-0546500-T6-44_1408	TGCAGAGAATCCAGTTTCCGGATACCATG
2D-0546500-T6-44_1580	ACTCTCCTCACCCCAGCGCCTCCTATATTA
4A-1003790-T6-45_0	GGTTTTAGTCGGAGGGGTCGAAAGAATG
4A-1003790-T6-45_64	GCACTGGATTGATCAGGGTATGAACTGGA
4A-1003790-T6-45_128	ATACATCATATAGTAGATCGCGTAAAGAGG
4A-1003790-T6-45_192	ATAACTACAAAAGTACAAGTCCAAACGAA
4A-1003790-T6-45_256	ACTGTCAATCTATATATGTCATGTGGAATG
4A-1003790-T6-45_320	TTTTCTTCGATATGCAATCACACTTTATCCT
4A-1003790-T6-45_448	GTCCGTCTCTCCTTCCACTTTGCACTTTTC
4A-1003790-T6-45_640	CCGCAACCAATAACTCGCTGTCTCCAGTG
4A-1003790-T6-45_704	CACACCTTCCCGAGATCTCATCTCGGATCG
4A-1003790-T6-45_896	GCACCAATATCAAACACAAGGTAGTAGTA
4A-1003790-T6-45_960	AGAAGGCATCTCCTCAACGGCATGAATTG
4A-1003790-T6-45_1152	CTTGAGATATTTACGACTAATTATATTTCCA
4A-1003790-T6-45_1216	GGATAAGTTTTAGAACTATCACAATTTTGG
4A-1003790-T6-45_1280	GAACCGCATAGGAATCTACATGAAGTACA
4A-1003790-T6-45_1344	CTCTTTGACAATATCGAGTCCCGGATGAG
4A-1003790-T6-45_1408	ACTAAAACAATATAATGATTGTAGGGTCAG
4A-1003790-T6-45_1472	TTCATTTTATCCATTTTGAACCTGGCAACG
4A-1003790-T6-45_1536	CAATCATGTATCCGAAACAAGGGGGCATG
4A-1003790-T6-45_1580	AAAAACATTCACCGTCTTGACCCGTTGGT
4B-1054990-T6-45_128	CGCTAATGTGGTCCAATCAAGACTTACCC
4B-1054990-T6-45_192	CGACCTTATTTCTTAACGAATATATGGTTG
4B-1054990-T6-45_384	AAGTGACGACACTCTTGCTCATGTGTCGA
4B-1054990-T6-45_448	AAATAAATTGCAATTGTCTTCTTCTTTGTA
4B-1054990-T6-45_512	GCCAACTCTGCATATATGGCACGGTAAGG
4B-1054990-T6-45_704	GCCATATATTATAGTATATTACGTGTCACCG
4B-1054990-T6-45_1088	TATGCGCGTGTATATGAGCACTTGTGTGTG
4B-1054990-T6-45_1152	ATCATTAACCCTTAAAAGCATAAATGCCCC
4B-1054990-T6-45_1344	TGATTGCATGTCGAAGAAAAAGTGGTACG
4B-1054990-T6-45_1408	CATATATAGCTAATGTATATATTGGCAGTCG
4B-1054990-T6-45_1472	TATTTCTTTTGGACTTTGTATACTTTTGCAG
4B-1054990-T6-45_1536	AAGCTCCTGTTTACGCGATCTACTATATGA
4B-1054990-T6-45_1580	GATATCTCTCAATTCATTTTCCAGTTCATAC
4D-1124750-T6-45_0	TTGATGCTAAATGTGGTTCAGACAAGGAT
4D-1124750-T6-45_64	TTCAAAATAAACCAACATGCCCCCATGTTT
4D-1124750-T6-45_128	ACTAAAGTTAGACGCGTTGCCAGGTTCGA
4D-1124750-T6-45_192	CCCTTATCTTAGCGCTGACCCTACAATCAT
4D-1124750-T6-45_384	ATCAAGACTTACCCCAAAATTGTGATAGTT
4D-1124750-T6-45_448	ACGAATATACGGTTGGAAATATAATCAGTC
4D-1124750-T6-45_512	GCAAGTCGATCGAGTTGACACCGCGTGA
4D-1124750-T6-45_640	TGCTCATGTGTCGACACAATTCATGCCGTT
4D-1124750-T6-45_896	TACTTTTTTGCTTGAACGATCCGAGATGA
4D-1124750-T6-45_1088	gagagagagCTAGCTAAGTTCATCCACTACT
4D-1124750-T6-45_1152	CTATCATTAACCCTTAAAAGCATATACTATG
4D-1124750-T6-45_1216	ATGCAGAGTGGAGGGAGAGACGGACAG
4D-1124750-T6-45_1344	AAGTGGTGATTGCATGTCGAAGAAAAAG
4D-1124750-T6-45_1408	CCACATGACATATATAGCTAGATGAATAGA
4D-1124750-T6-45_1472	ATGTATCTTTATTTCGTTTGAACTTTGTACT
4D-1124750-T6-45_1536	TCTTACGAAGCTCCTCCTCTTTACGCGATC
4D-1124750-T6-45_1580	TGTTGACATTATCTCTCAATTCATTTTCCAG
2A-0325680-T6-46_128	CTCTGCTGCAGACATCATGTCGCGGTTAAT
2A-0325680-T6-46_256	GTAGCACAGGTTGTCAGCGTTGTAAGCG
2A-0325680-T6-46_512	GTTTCTTTTCCACAAAAAGAAATTGCTGG
2A-0325680-T6-46_896	gagacggggggagtaTAAATTTTTAAAAATAT
2A-0325680-T6-46_960	AAAATATACATGAATTTTAAAATATGCTCAT
2A-0325680-T6-46_1024	CCCGAATTTATATATTTGTGTATTTCAAATT
2A-0325680-T6-46_1088	AAAAAGTGTTCATGTTTTTTTTGAAATATT
2A-0325680-T6-46_1152	ATAAAATATATAAAAACAATATAAATAAAG
2A-0325680-T6-46_1344	CATTGTATAATGAAGTTATATCACACTTAA
2A-0325680-T6-46_1472	CGGGTTGGGTTTGTGCGTGTGGTGTGTG
2A-0325680-T6-46_1536	CAGTTGACGTTGGCGAGAAGATGGCTTC
2A-0325680-T6-46_1580	AATATTATCCCCGTTATCGTCGTTTCAACA
2B-0397670-T6-46_128	AGCTCTGCTGCAGACATCATGTCGCTATTA
2B-0397670-T6-46_256	GCGTAGCCCAGGTTGTCAGCCTTGTAAGC
2B-0397670-T6-46_448	GATTCTCCTATGAAGATAGCTAGCATAGTT
2B-0397670-T6-46_576	ATCTAATTCCTATGTCATTGATTTTAGTCGG
2B-0397670-T6-46_640	TTTCCTTTTTTATACTTGATTGGTATAATGA
2B-0397670-T6-46_704	ATAAGTTTTCCACGTAAGGATTGCCGACG
2B-0397670-T6-46_1152	TATTGGAGATGCTCTTACTAAGGTTCGGT
2B-0397670-T6-46_1216	TCATCAATGATGTGGAGTAATATTATCCCC
2B-0397670-T6-46_1408	TCTGACTATTTCTTCGTTCGACGATGTGTG
2B-0397670-T6-46_1472	GTCGGGTCTCTCCAATTCTTGTTAGCTGG
2B-0397670-T6-46_1536	TTATTCGCGACGACGATATGATCCTTAGTT
2D-0534750-T6-46_0	GGCTAGGCTGTCGTGATGCACGCGACTG
2D-0534750-T6-46_64	GAGCTTCTTCGCAGCTAGCCACGTTTGTT
2D-0534750-T6-46_128	GGAGCCAGCGACGTTATGTCAGTGTCAG
2D-0534750-T6-46_192	ATCAGACGATCTGTTCAGATGAACGACGA
2D-0534750-T6-46_256	CACGGCGTAGCACAGGGGGTCCGCGTTG
2D-0534750-T6-46_320	GCGAAGGAGATTTTTTTCCTGCTTTACTCT
2D-0534750-T6-46_384	ATCCGTCTACGGACAGGGACCAATCCGCG

										2D-0534750-T6-46_896	TCTCCTTGAGCGCCTCCTTTCTTTCCTCGA
										1A-0073380-T6-47_128	GAGAAACGAGGGAGGTTTCAACGCGAG
										1A-0073380-T6-47_320	ACCGTCTTTCTCGCTGAAGACCGGCAATC
										1A-0073380-T6-47_384	TTTGACGGAACAGCACCTAATACTTTGCA
										1A-0073380-T6-47_640	AAAAGCCTGTAAATTGTTTGTACGTGTAG
										1B-0167090-T6-47_320	tatgaaaatataataatacatacattattgcctcaagg
										1B-0167090-T6-47_384	TTCCACTGACAGAATATTCTGCCTCTATGA
										1B-0167090-T6-47_512	aaagttatactaaagttgatacacttattttggataga
										1B-0167090-T6-47_576	ATATTCTGTATTTTTCCGGATTATTACAACA
										1B-0167090-T6-47_640	AATTGCAATTGGCAAATGAGCACTAGTATA
										1B-0167090-T6-47_704	GCGTCACCAATTAATATCTTCGACCGACAA
										1B-0167090-T6-47_832	TCCGGTGTTTGCCTGACACCGAGTGGACG
										1B-0167090-T6-47_896	ACTACCTTTGGGTTAGCAATAGGCTATAG
										1B-0167090-T6-47_960	TCACATCGCCCACGCAAATGCCTGATTGTT
										1B-0167090-T6-47_1152	TTCCGGACGGGGGGAGTAGTATACAAAA
										1B-0167090-T6-47_1216	ATGTATGTGCAAAGATTGCCAGTCTTTGG
										1B-0167090-T6-47_1408	taGCAGTGAGCAAACAATACTATACAGTAC
										1B-0167090-T6-47_1472	ACTAATAAACCGTTCTTCTTCTCTCGTAAA
										1D-0263760-T6-47_128	AAAAGAAACAAGCGAGGTTTCAACGCGA
										1D-0263760-T6-47_192	GTGGAAACTTGCGAGAGAAGAAGAAAC
										1D-0263760-T6-47_384	ACATACAGCACCTAATACTTTCTTTTTTGA
										1A-0057560-T6-48_0	GGCCCGAGAATCCCGAGCGGTGCTAAATT
										1A-0057560-T6-48_704	TGGACTGGGCTCATCTCATCGGCCTTCAG
										1A-0057560-T6-48_768	CGAGTTATTGTGCTTGTGCCGATTACGAAT
										1A-0057560-T6-48_832	AGGGTTTCATATTTTTAAAAGGCCTATGTT
										1A-0057560-T6-48_896	GAATATAAATTTACTCCTATTCTTTTGCCGG
										1A-0057560-T6-48_960	GATTCGACCATTTGAATCTGAAAAGGGG
										1A-0057560-T6-48_1088	CTTTTTCTTCTTTTAGGGAAGCAGGGGTT
										1A-0057560-T6-48_1152	TTTTGAGTAAAAGTATAGATTTGCTCCTTG
										1A-0057560-T6-48_1216	GAAACAAAATCATGGTTCTCGAAGCACGT
										1A-0057560-T6-48_1280	AAATTCTCTTTTTTCAAAATCTAGGGAAAA
										1A-0057560-T6-48_1472	AGAGGTAACCATTGGTTGGTCTCATACAA
										1A-0057560-T6-48_1536	AAATGCATCTATCCCAATATCCAATCATGCA
										1A-0057560-T6-48_1580	TCAATATGATAACTTACATGAGAAAAGATT
										1B-0150920-T6-48_0	GGCCTGAGAATCCCGAGCGGTGCTAAATT
										1B-0150920-T6-48_1238	GCTGAGAGGCAGGGGACCTCGTATGTTT
										1B-0150920-T6-48_1295	TTTTCATCATATTTTAAGAGAATAAACTTA
										1B-0150920-T6-48_1352	CGATATAGAGTTTTTTGTCCTCACAGATTC
										1B-0150920-T6-48_1409	CAAAGAAAAATGAATACTCAAAAAAAATC
										1B-0150920-T6-48_1466	CGGGTCCGGTTTTCACCGTTTTTGGGGAA
										1D-0264750-T6-48_568	CGATTTTTTTGAGTATTCATTTTTTTGGTT
										1D-0264750-T6-48_632	GGACAAAAACCTCTCTACCCTTATGACAC
										1D-0264750-T6-48_824	AAACCCTCGGAAGCACGCCTAACCTCAGA
										1D-0264750-T6-48_888	TTTTGAGAAATGCCTAACCTCAGATGAAA
										1D-0264750-T6-48_1580	TGCGAAGATAGCAAGGCATCCACCGCATC
										7A-2089960-T6-49_0	GCTTGTCTTTTTTTTATATGAAATTAGCTC
										7A-2089960-T6-49_64	GGACCTCTCAAGGAGTACACAGTTATCCC
										7A-2089960-T6-49_128	GAAATGATATTTTAGTTTCAAAAATTATTTA
										7A-2089960-T6-49_192	AATTTGGCATCAAAACCGCCTGATAGTTCA
										7A-2089960-T6-49_256	CACCGCACTGACCACCCCCCACCAGGGCA
										7A-2089960-T6-49_320	CACCTCACAACATTGTGTCATTAACTTGCG
										7A-2089960-T6-49_384	AATAAGGGAAATAAAACTTAGAAAAGTAG
										7A-2089960-T6-49_448	AACCATAGAAAAAGGTTTTTACAGGGTTT
										7A-2089960-T6-49_768	ATGCAAGGATTTAAACGACGATGATTGTG
										7A-2089960-T6-49_832	GAGACTTCCCCGCTGTCATCAACGAATGT
										7A-2089960-T6-49_896	AAGGTCAAGCCGGCGGAGCGGCGACAA
										7A-2089960-T6-49_960	TTTGATGTATTTCTTATTATGTTCGACATAC
										7A-2089960-T6-49_1024	GAACTTTTTGGGATAAAAAAAGATGCCTG
										7A-2089960-T6-49_1088	GCAGTGAAGCAATCTCCCCGCACGATCCA
										7A-2089960-T6-49_1152	CTCTGATTATTGGCGACCGTCCGTCGGGC
										7A-2089960-T6-49_1216	TCGCCTCACCAGATAAATTCCCTCCGCCAC
										7A-2089960-T6-49_1280	CGTCCGCCCCGGCCAGAGTCCTCCCCCCG
										7A-2089960-T6-49_1472	CCGCGGCGTCCATCGCCCCCAACCCCCAG
										7A-2089960-T6-49_1536	AGCTTGTTAATTAGGAGTACCAATTTGCTC
										7A-2089960-T6-49_1580	CTTAGAAAAAGCTCAGAATTTTCGGTGCC
										7B-1878150-T6-49_64	TTGATGTCATGTGATTCAGATCCATGCAAG
										7B-1878150-T6-49_128	GTGCACAAAGGCTTCCGCTATCATCGACA
										7B-1878150-T6-49_256	TATTTTTTATTATGTTTGAGGCACTTTGTAC
										7B-1878150-T6-49_320	TTGGGATACAAGAGATGCCTGAAACCTGA
										7B-1878150-T6-49_1024	TGGAAGTTTTTCCAAAAAAAAAAGATTTG
										7B-1878150-T6-49_1088	ACAGGGGAGGGGACCCAAATCCTACGAG
										7B-1878150-T6-49_1152	GTGGCTCAGATTCCGCACCCCGTCCCACT
										7B-1878150-T6-49_1216	CCTCCGCCAGCGCCGcctccctccctccctcccc
										7B-1878150-T6-49_1408	CGCGCCGTCTAGCTACCTCCCGCCGCGGC
										7B-1878150-T6-49_1472	CGGAGGAACAATCGTAGGCGAAGCAGCT
										7B-1878150-T6-49_1536	TTATTGTTAAACGAATCTCTGTTGTTCGTT
										7B-1878150-T6-49_1580	TTCTTCTTTTATAGAAAAAGCTCTGAATTT
										7D-2002830-T6-49_0	GAACATAGTGATGCCTTGTTTTTTCAGCG
										7D-2002830-T6-49_64	AGCCCAGCCAGCCAGTGACCCAGAAATC
										7D-2002830-T6-49_128	CGCTAATCGCCGACCTAGTTTTCTCCATGG
										7D-2002830-T6-49_192	TACATACATAAGTGGTATTATTCATCGTCCT
										7D-2002830-T6-49_256	TCGGCGGCGCACGGTATCGCGACGTGCC
										7D-2002830-T6-49_384	tcaatacacttattttgaaacagaaaaaataTTATTT
										7D-2002830-T6-49_768	CCACCGACAACCGAGGTCGGCGGCGGAG
										7D-2002830-T6-49_832	CAATAGTTAATTGATCCATCCATATATTCAA
										7D-2002830-T6-49_896	TGTTCATATATATTCCGCAAAAAAAAAGTG
										7D-2002830-T6-49_960	TGATTTTTTTTGGCATCCAGCGCAGCACC
										7D-2002830-T6-49_1024	TACAACCAGTACAATACAAGAGACGATAA
										7D-2002830-T6-49_1088	AAATAAATAAAAAGGAGGGGGCGAAAAG
										7D-2002830-T6-49_1152	CAGGTGGCTGATATTCCTATCCCACCCGCG
										7D-2002830-T6-49_1216	CGGAAAGCCCAGATAAATTCGCccgcccgcc
										7D-2002830-T6-49_1280	ctcgctcccgagcctccgccgccgcaccggcggccgT
										7D-2002830-T6-49_1344	CTCGCCTGGTAAGCCCGGTTTCTCCGCTT
										7D-2002830-T6-49_1408	TCGGCTCGCTTCGGATCAGCCGCGCCGCC
										7D-2002830-T6-49_1472	CCCCTCCGCAGCCGGTCAACGGGCGGGC
										7D-2002830-T6-49_1536	GCTTATTTTTATTATTGTCAGCAACCTCTCC
										7D-2002830-T6-49_1580	GTGACCCGGACGGGCGACTTTCCCGGCG
										7A-1797770-T6-50_128	GAACGGAGGGAGTACATGCGAACTAATTA
										7A-1797770-T6-50_192	TATACTGAACAAATGCATGGAGTTGAAGT
										7A-1797770-T6-50_256	AGCACTAATATCCGCCTCCCCCTTTGTGTT
										7A-1797770-T6-50_320	ATCCTTGGCCATGCTGTTTCCATTCGCATC
										7A-1797770-T6-50_384	GTGCATGATTGAAAACACATTCTGCCACTA
										7A-1763720-T6-50_576	gcactaatgtgcttcacgtttgaccctttttctactagt
										7A-1797770-T6-50_576	CAGGGGAAAAAGGATGTGAGCCAATTGA
										7A-1763720-T6-50_640	GAACTCGAACGAGTTTTGCTCATGCATCCT
										7A-1797770-T6-50_640	AAACTCGCCTCCCGTGGCAAATCCTTTGC
										7A-1763720-T6-50_704	CTCCTATGAAATTCAAGACCCCTAAGGCT
										7A-1797770-T6-50_704	GACATGACACAAATCTGACGACAGGGTGT
										7A-1763720-T6-50_768	GGAATGGATTATCCTTCATTGTCACCTTTC
										7A-1797770-T6-50_768	CCTTAGATTGGTCTCATTTTTATTCACAAG
										7A-1763720-T6-50_832	GAATAAACTTTATTCGTGAGATGTATGGTT
										7A-1797770-T6-50_832	GCTTCAAAACAAACACCACAGATCAACAT
										7A-1763720-T6-50_1024	TATTCTATGATTTGTGATTCTAACATCGGA
										7A-1797770-T6-50_1024	GACCCTACTGGGTCAGGGTGCTGTCCAAT
										7A-1763720-T6-50_1088	GTTTGCAAATATCCAGCAAACCATCTAAAA
										7A-1797770-T6-50_1088	TGTGTACTCAAATATCAATGAAACGTTCAT
										7A-1763720-T6-50_1152	GAAATTTTTGTTGGACTACCCCAGATAAC
										7A-1797770-T6-50_1152	TAATATGATAATGTGATGTATTAAGCAGCA
										7A-1763720-T6-50_1216	CCCGACCCATCTCCCCTCAAAGAATGGCG
										7A-1797770-T6-50_1216	GCCTTTTATTGATTCGTGGTGCGACGTGA
										7A-1763720-T6-50_1408	GGTGGCAAAGGCACGGTGGGGGCGGGG
										7A-1763720-T6-50_1472	TCCCTACCGGCTCTTTTTCCTCTGAAAAAA
										7A-1763720-T6-50_1536	TGGTCCATTTTTTAAAATTATTTGAGGGAT
										7B-1878160-T6-50_128	gttttctactagtgTATGGGCAATGGTGCAACT
										7B-1878160-T6-50_192	CTTATGATGAGATCGATCTTGTTTATCATAT
										7B-1878160-T6-50_256	TTTCTGAGTCTGGCTTCATTTCTTTTTGTCA
										7B-1878160-T6-50_320	TATTCTCAATATGACACCTTATTAAATTGCT
										7B-1878160-T6-50_384	TGGCGGCATAGAACCTTGACTAACATGCC
										7B-1878160-T6-50_448	GCCTCATCATTTCCACGAATTCTCTCACAA
										7B-1878160-T6-50_512	CTTTTCACGGAGAGGTGGTAAATGATCTA
										7B-1878160-T6-50_576	AATAATCTATAAAATTACCTAGAAAGTGAC
										7B-1878160-T6-50_704	AACACTTGGTCCGTCGTGGCAGATTCACC

7B-1878160-T6-50_960	TCGGAGCTCGGATCCAATTCAAAATCGCG
7B-1878160-T6-50_1024	TGTGTTTAGCCTCTAGTTCTTAGGTCTTAG
7B-1878160-T6-50_1088	TTCGTCAAACCCACTAAAAGATTTGTATCA
7B-1878160-T6-50_1152	TGGGCTACCCCAGATAACAAATGAAAATT
7B-1878160-T6-50_1216	CTCCACTCAAAGAATGGCGCTCAAGCTCC
7B-1878160-T6-50_1408	CACAGGCACGGTGGCTGCGGGAGCATGG
7B-1878160-T6-50_1472	ACCGGCTCTTTTTCCTCTGAAAAAAGGCG
7B-1878160-T6-50_1536	CATATTTTTTGTTAATCATTTGAGGGATTCT
2A-0301300-T6-51_64	CAGTGCATTGGAGAGGCATTTGGAGGTT
2A-0301300-T6-51_256	ATTCGAGGGCTTGTCAAAGTATGATTTCT
2A-0301300-T6-51_320	GCTTCCGCTACATAACAGTCGACTGACACT
2A-0301300-T6-51_384	TTTGCTTTTATTTTTATGATGACTAGGAGT
2A-0301300-T6-51_448	CGCACTTAGGTTTGTCTTGAGAAATTTTGT
2A-0301300-T6-51_512	ACATATTCAGTTAGTATTGATGATAGTATG
2A-0301300-T6-51_576	ACCTTTATGTTTGTTTCCTTGTTGAGTCTG
2A-0301300-T6-51_768	TCGCCACAAGAAGTACACGCATATCCAGG
2A-0301300-T6-51_832	CACAATTTGACATGATCCTCACTGATACGG
2A-0301300-T6-51_1024	CTATTGTTGAGAGATCAGGCAGGCAGGA
2A-0301300-T6-51_1088	ATAAGGGAAAAAGAGCTTTTCCATTGCCA
2A-0301300-T6-51_1152	TAGATCCCGTATGAACTTCATTCGGTCAAT
2A-0301300-T6-51_1216	ATCGGTGGAAGCAGAAGCTGTTAGCATCT
2A-0301300-T6-51_1280	TATATGTTCTCTGTTCTGGTTTCTGCCTTTC
2A-0301300-T6-51_1344	TTTAGATCTTTTTTTTGAGGTGTTTTTAGA
2A-0301300-T6-51_1408	ACTCCTAACTTTCTCCAGGCCTTCTGACAG
2A-0301300-T6-51_1472	AGTAGACACAAGAGCCCAGACCACAGGT
2B-0389520-T6-51_470	GTGTGTCAGTAATGGGCCGAGCTGTCAG
2B-0389520-T6-51_532	TTTCTCAAAAAATAAAAGAGTTGGGAAA
2B-0389520-T6-51_594	CTCTGAACTTCTGAAGTCTGAAGCACTGT
2B-0389520-T6-51_656	ACAACTTCAGCTTCCACCGATTTATTATTT
2B-0389520-T6-51_718	GAATGAAGTTCATACGGGATCTCCATCCA
2B-0389520-T6-51_780	AACGGAAAGCTCTGTTCCCTTATTCCTCTC
2B-0389520-T6-51_966	TCTTGCCTTCAGGGGTGATGATGCTCTCT
2B-0389520-T6-51_1028	ATATATCAGTTAGCGGAGAATTGTGCCTAA
2B-0389520-T6-51_1214	CGGTAAAAGGGCTGTTCATCCAAGTGAG
2B-0389520-T6-51_1276	TGCCAGACTGAACAAGGAAACAAACAGA
2B-0389520-T6-51_1338	ATACTAGCATACTATCATCAATACTAATTGA
2B-0389520-T6-51_1400	GCACATGCTACAAAGTTTCTCAAGACAAA
2B-0389520-T6-51_1462	CTCGAAAGCACTCCTGGTTATCATAAAAG
2B-0389520-T6-51_1524	TTTACAATAGCAGTCAGTCAACTGTTATGT
2D-0533310-T6-51_0	GAGGGTAATTTGCAAGTTGCAGTTGAGG
2D-0533310-T6-51_62	GTGTGTGCAATGCAATCTGCAGAAGAATG
2D-0533310-T6-51_124	AGCAGGTACACCTGTGCCATGGTGAATAC
2D-0533310-T6-51_186	ACATACAACAGTAGCTAGGATGTGCATAA
2D-0533310-T6-51_248	ATTCTACTCCATTACAGTTGGACATAATTCT
2D-0533310-T6-51_310	TAGCTTAATCTAGTGTTGATATCAATATATC
2D-0533310-T6-51_496	GCGATCTGTACTACTCTGTTAGTTACCCGT
2D-0533310-T6-51_992	GCGTTGGACTCCCCGTCTCGTCCTATCTGA
2D-0533310-T6-51_1116	GCTCTTGTGTCTACTGGGCCTTCGCTCATT
2D-0533310-T6-51_1178	CCACTATTAGCTAGGAGTTGGAACTTCTC
2D-0533310-T6-51_1240	CGAAAACAAAAAGAGAGTTGGGAAAGG
2D-0533310-T6-51_1302	TGAACTCTGAAGTTTGAAGCATTGTGCCA
2D-0533310-T6-51_1364	CTTCAGTTTCCACCGATTTATTATTTGAGA
2D-0533310-T6-51_1426	GAAGTTCATACGGGATCTCGATCCACGAT
2D-0533310-T6-51_1488	GAAAGCTCTTTTTCCCTTATTCCCTTCCTCT
2D-0533310-T6-51_1736	AGTTACAGCATACCAGTTAGCACTCTCGC
2D-0533310-T6-51_1922	ATGACCAAGCTATGATATCTTGCGGCGGT
2D-0533310-T6-51_1984	AAATCAGAAGCTCAATTTGGACAAGTGCC
2D-0533310-T6-51_2046	CGTGGTTGAAAAAAAAGCTACCTCCATAC
2D-0533310-T6-51_2108	TAAAAATTCGACTCGCCATTGAATGGCAC
2D-0533310-T6-51_2170	CGTGCTCTAAACAGGTAAGTACAATAGAG
2D-0533310-T6-51_2232	AGCAAAGACAACATGTGTGCAGTTCAATG
2D-0533310-T6-51_2294	ATGTAAGCTAGAACCGACAATCCATGGTT
2D-0533310-T6-51_2356	CCCTCGAATATGGACTTTCCTAGAACTTCG
5A-1191460-T6-52_594	GAGCTGAAGGACATTATTACTACACTAGG
5A-1191460-T6-52_655	CTCCTTAAAGAAAATCTGAGACAAACACT
5A-1191460-T6-52_1101	ATTAGCAACACCCCGTCGACGCCAACCTT
5A-1191460-T6-52_1161	GGAGAAGACGGTGTGTATGGTGGGGGTG
5A-1191460-T6-52_1221	CCTCTCACACAAGGCGTTGTGGCACTAGT
5A-1191460-T6-52_1281	TCCTTTAAATCAAAGATGCCCTAAAAGTTG
5A-1191460-T6-52_1341	TTTGAGTTTCTGCAAAGCATACAACTTGG
5A-1191460-T6-52_1401	TGGTCTCAGCAAATAATAAAGTGAAGTAC
5A-1191460-T6-52_1580	CACTTCGCCTACACCAAAACCCACCCTTGT
5B-1342560-T6-52_186	GATATTTGCATGTGGTTGCCTCGGCCGCCT
5B-1342560-T6-52_372	TATAAACTGTAAAAAAAGTGCAGCAAGCT
5B-1342560-T6-52_496	GTAAAAGCAGCAGCAAGGAAGTCGTTGA
5B-1342560-T6-52_558	AAATCATCCCGGAGAAGAAAACGCCGAA
5B-1342560-T6-52_620	CGGTCGAATTTAGACGGATCCACTGGAAA
5B-1342560-T6-52_744	GGATAGCATGCCCCAAACCAGACATGAAG
5B-1342560-T6-52_1229	GCAAGGCGTTGCGGCACTGGTTGTGCTA
5B-1342560-T6-52_1288	AATCAAAGATGCCCTAAAAGTTGGACGTG
5B-1342560-T6-52_1347	TTTCTACAAACCATACAACTTGGCATGAGA
5B-1342560-T6-52_1406	TCAGCAAATAATAAAAGTGAAGTACGGAC
5B-1342560-T6-52_1580	CCACTTCGCCTACACCAAAACTTCCCTTGT
5D-1414720-T6-52_448	CTCCATGGGGCTCGGAGGGTAAGGAGAG
5D-1414720-T6-52_576	atgctgctggcttgctggatgcttgatTAGCCAATT
5D-1414720-T6-52_640	TTCTGAAACAGAGATTAGTGAGAGTAGCT
5D-1414720-T6-52_704	ATGCTTAATAACGTCCACTAACCAAAAATT
5D-1414720-T6-52_768	TGAGTACCAGCTAATTTTTATACTCCTACA
5D-1414720-T6-52_896	TCAGCAACAGCTAGGAGTACAAAACAATA
5D-1414720-T6-52_1024	GCTAGTGCCCTTTTCAGCCATACGCATGTC
5D-1414720-T6-52_1152	GGTACCATGCCTTAAATCTGAGTAGGTGG
5D-1414720-T6-52_1216	GTTAGTCTGTGAATCATGCAGATTTTGTCG
5D-1414720-T6-52_1280	TATATGCTAGACTCCTAGTGATCCCAAAAT
5D-1414720-T6-52_1344	CACACACGTGCATAGGAATTTGACCGGAG
5D-1414720-T6-52_1408	TACTCAAAATTTTTTTGCCAGTTTTTCGTT
5D-1414720-T6-52_1472	AAAGTTCTGGAGCAAAATATTCGTTATTTT
5D-1414720-T6-52_1536	AAGCACTACTATTTTTTAGAAAATCGCAAG
7A-1769490-T6-53_0	TTTACTGGAAGTACAAAGCACATCAAGCA
7A-1769490-T6-53_192	AGAACATCTTATATTTGTGAACGGATGGA
7A-1769490-T6-53_256	CGAGACAAAATATCATTGTTATCCTGTTATT
7A-1769490-T6-53_320	AAGGCGACTAGGGTGAAGCCTTTCTCCC
7A-1769490-T6-53_384	TTCTGTCTACCGCCGGTGGTGAACCTAGT
7A-1769490-T6-53_448	TCAAAAAATTACACAACTAATATTAAATTT
7A-1769490-T6-53_512	CTAAAATTGCATTAAATCGTCATGTATTAG
7A-1769490-T6-53_704	ATGAGTGTATGTGTGTGTATATAGGGCTTC
7A-1769490-T6-53_768	TTCGTTTCTGCCCAAACGTCTCAAAATATT
7A-1769490-T6-53_832	TGAAAAAACTTTTTTTAGGGGGTTTCTAA
7A-1769490-T6-53_896	CTAAAAAGATACTGCAACTTCTTCCATATG
7A-1769490-T6-53_960	CCAAACGGAAACCAAAACGAGGCGGTTG
7A-1769490-T6-53_1024	AGGCGCGGAGGCGGTTAATCAACCCGGC
7A-1769490-T6-53_1088	GTCTCTCTCGGCTTTCCGGCACGGCCCCG
7A-1769490-T6-53_1152	CGCCCGATCCGTCTTGGACTCCTGGGTGA
7A-1769490-T6-53_1216	GTGCCGCCTCCGTCACCATCCATccgccccg
7A-1769490-T6-53_1280	gctctccccgtcctgcgctcgcaccgTTTTCCAGC
7A-1769490-T6-53_1344	ACCCGCCCCCCTCCCCTCACCTGCCACTG
7A-1769490-T6-53_1472	AGTCCAGTCCAGGGATATTAAATCTCTAGA
7B-1907150-T6-53_64	TTCGTCAATCTCAAGATATGATGCCGGCTT
7B-1907150-T6-53_128	CGTGCATGCATGTATTCATACAAATGAGG
7B-1907150-T6-53_896	ttattttgggactgagggagtaCATTTATTGGGTA
7B-1907150-T6-53_960	AGGGTCCTCCCTGTAGAGAAGAAGATATA
7B-1907150-T6-53_1024	CCAAACGGAAACTAAAAGGACGAGGCGG
7B-1907150-T6-53_1088	GGCGGGCGAGGCGGTTAATCAACCCGGG
7B-1907150-T6-53_1152	tcccCATAAGCCGTCTCCCTCGGCTTTCCGG
7B-1907150-T6-53_1216	GTTCATCACGCCTCTCCCTCCCCATCCCTC
7B-1907150-T6-53_1280	GTGGACTGGGCACTGCTGGTAGGCTCCC
7B-1907150-T6-53_1344	GCCTGCCCGCACCGTTTCCTGCTCCGCTC
7B-1907150-T6-53_1472	AGGTGGGCTGGCCAGTTAATGGCGGGCG
7D-1963900-T6-53_0	ATGTAGCAAAGCCGCTGTCCGTGTCCTGC
7D-1963900-T6-53_64	TGCCTCTGCACCCTCGTCTTCTCCTCCTCC
7D-1963900-T6-53_128	AGGTAGGTGGGTTTGCCAGTTAATGGCG
7D-1963900-T6-53_192	TGAACCTACCACCATATATACATCACCAAC
7D-1963900-T6-53_256	CTCTTCTTGGCTCTCTTGCCGCCGTGTCG
7D-1963900-T6-53_320	GCGCAAAGAAGCGGGAGCGGAAGAGCC
7D-1963900-T6-53_576	TAAGCAAGCACACAAtcctcctcctcctcat
7D-1963900-T6-53_640	TCGCCGTCGCGCTCGGCCCTCGTGACCGG
7D-1963900-T6-53_704	ATGCGCCGGTGAATTTTTTGTCCGTTACC
7D-1963900-T6-53_768	CTGATCAACCTCTCCCCCCGTAATTCGTGG
7D-1963900-T6-53_832	GTCGCTGGACTTGGACTGGAACAGAACT
7D-1963900-T6-53_896	CGATCCGGCCTTTCTGGTAGTAGTAACGT
7D-1963900-T6-53_960	TCTGATGACGACGGCCGGCGTGCACCGA
7D-1963900-T6-53_1024	TGAACCAACTTCACCGTCGCCGGGCTACT
7D-1963900-T6-53_1088	CATATCCAAACTTTGTTTATACTTGCAGTT
7D-1963900-T6-53_1152	GATCACGCAAAGTCACTAATTCTTTTCTCA
7D-1963900-T6-53_1216	TCTTTCCCTTCTTGTCTTGTCCTTGCTTTTT

										7D-1963900-T6-53_1280	TTTTTGCCATATCTGTTCGTTCACAGAAAA
										7D-1963900-T6-53_1344	TTTCACAATTATTTACGTAATCATTTTTTAT
										6A-1555330-T6-54_128	CCTTGAGCTGATGCTCGTCTTCTCATCACT
										6A-1555330-T6-54_192	TACTTACTATGGAAATATCTGCATATTGTCA
										6A-1555330-T6-54_256	GTGGTAGCTTCTTTTTGTCCTGTTGTTAAT
										6A-1555330-T6-54_320	ACATGTATTTAGTGTAAACGGGAAATGTTT
										6A-1555330-T6-54_512	CCAATCATTGGACTGTCAAACATTGTCAG
										6A-1555330-T6-54_576	TATCATCTCCACGATGAGTATACCTCAATTA
										6A-1555330-T6-54_640	TGCCAAGGAACAATGTAGCCTTTCTTTCTT
										6A-1555330-T6-54_704	TTATATTTATGAGCATGCACTTTCAGTTCA
										6A-1555330-T6-54_768	AAGTAATGCCGAAAATGCTGCACCGTTGG
										6A-1555330-T6-54_1152	ACTTGAGGGTGTGAGTTCTCCAGAAGAC
										6A-1555330-T6-54_1344	TGCCAGCTTGTACTCACCACCAAAGCAAA
										6A-1555330-T6-54_1536	GCTACTGCGGTGGTTGCTATGTTCTCCAGT
										6A-1555330-T6-54_1580	GTTGGAGGTACAGTCTCAACCTGGCAAC
										6B-1671990-T6-54_0	TTCCTGTTGCAGAATTGGTCAGAACAAGA
										6B-1671990-T6-54_64	ATGTCACTCATCGAAACCTATTAAAGGAGT
										6B-1671990-T6-54_128	GCGGCAGCAATATGCATCAGAAATACAGC
										6B-1671990-T6-54_192	AAGGTGGCTAGCAATGCCCAGGGCTGCT
										6B-1671990-T6-54_256	AAATTGCGAGATATTGTTTTTGCTCGACG
										6B-1671990-T6-54_320	CTCCGCACCGGaaccaaaccaaaccaaaccaa
										6B-1671990-T6-54_384	CACCACTGACGCGCGCGCAAACAGCTCG
										6B-1671990-T6-54_442	TAAAAAATACTGGGGCGGCAACTCAACA
										6B-1671990-T6-54_1076	GCCCGCCTATAGCCCTTTGTTTGTTTTGTT
										6B-1671990-T6-54_1140	ACGACTCGTTCGGATTTGCTGGAATTTGG
										6B-1671990-T6-54_1268	GGTCGTTGGTGCCCGTCGCTCTCGCCGCT
										6B-1671990-T6-54_1332	CCCGCCGGTCTTCCAGTTTCTCACATATGC
										6B-1671990-T6-54_1396	TTTGGGCCCGGCCTGTAGACAGAAATCA
										6B-1671990-T6-54_1460	CAAACATCCGTTGACTTTTATGGGTATGA
										6B-1671990-T6-54_1524	TTGTTTACAGGAAAGGAGTCGGTCAAAA
										6B-1671990-T6-54_1580	CATTCCGTTCAAATAAACCACATTTATTTCT
										6D-1735700-T6-54_0	CGCGAACAGGAACACAGGAACTGAAAA
										6D-1735700-T6-54_192	ATATGCAACTGTAACTGTTTGACCTGATGT
										6D-1735700-T6-54_576	GACCTCAAAGTTGATATCCTCAATAGCTTC
										6D-1735700-T6-54_704	GCATCAATGCAGACTCGAGTTTACTGAAC
										6D-1735700-T6-54_768	ATTGCTAGTAAAAAAATCACAGTATGAAG
										6D-1735700-T6-54_832	ATGCTCAAAATTCTCTAAGAATATATAATTG
										6D-1735700-T6-54_1024	TCTTAAAACTGAATATCAAGGGAAAGATA
										6D-1735700-T6-54_1088	TAGGATAAAATTCATCTCCTGAAACCAAA
										6D-1735700-T6-54_1152	GTCCCTAGTGAAAACTGATAACTTGGATT
										6D-1735700-T6-54_1216	GGCTTCCCTCTTTTTACCCCATTTATGACA
										6D-1735700-T6-54_1280	CTCCATATTGAAAAAATAAAAAATACAGTG
										6D-1735700-T6-54_1536	CGGCAGAAGCGCCATCCGCCGCATGGAG
										6D-1735700-T6-54_1580	TGGCTCTCTCCATTCTGCTCCATTCCTGTT
										4A-1021690-T6-55_124	GTGCAACGCACGGGCATCTTGCTAGTCCA
										4A-1021690-T6-55_186	TTTTTTAAAAATAGCCTTGTGCCAAACACT
										4A-1021690-T6-55_744	ACAGACTGATACAGAACTACGTTAAATGG
										4A-1021690-T6-55_806	TACGGAAGGTTGGCGGGTTCTCCTTGAA
										4A-1021690-T6-55_868	GTTAGCACGTGAAGCAAGCTAAGGTTAGT
										4A-1021690-T6-55_930	GGTTAACGACTGAAGGAGTACTTCCTAGA
										4A-1021690-T6-55_992	AAAAGTCTGCTCACAGGACAATCATAAAT
										4A-1021690-T6-55_1054	AACGCATATACCTAGATTTTACCCTTATGA
										4A-1021690-T6-55_1364	CTCGAAAAAAAAATGTGAGAACTTAGATT
										4A-1021690-T6-55_1426	GTCCATTGGGAGAAGAGTCTTCCCGTTGT
										4A-1021690-T6-55_1488	CTTGAGTTAAGCTCGACCGATGACTCAAC
										4A-1021690-T6-55_1612	GTGTCCACAAACAAACTAAAGCACTCCAC
										4A-1021690-T6-55_1674	TTGCTTGAAGCAACTACTCCATCTCCATGG
										4A-1021690-T6-55_1736	AAAGGAGGAGGTAAATACAGTAGATCTTT
										4A-1021690-T6-55_1798	TTATTTTTATGATTTGATTGATGCCGGACGT
										4B-1050740-T6-55_0	AATCATCACGTGCACATTCTCATATGCGCC
										4B-1050740-T6-55_192	TTTTTCGTGTCGGGTTGCACGGTAGTGGG
										4B-1050740-T6-55_256	TAGTACCGCTTACAAACAGTAGTACCGCC
										4B-1050740-T6-55_384	CAGTTCGAGCGGCAGTACCACTTTTGCGG
										4B-1050740-T6-55_512	GCACTACCGCCTTGTGGTCTCGTGCCATT
										4B-1050740-T6-55_576	TACCGCTTAGTGCGGGATGAGAACAAGG
										4B-1050740-T6-55_1152	ATCAAACATGACCGTAATCAAACGATAAA
										4B-1050740-T6-55_1216	CTGATGACCCAACCTGATCTCTAACTGAA
										4B-1050740-T6-55_1344	CGTGTCCACAAACAAACTCCAGCACTCCA
										4B-1050740-T6-55_1408	TGCTTGAAGCAACTTTACTCCATGGCGAC
										4B-1050740-T6-55_1472	GAGACAGATACAGTAGATCTTTATCGCAG
										4B-1050740-T6-55_1536	GACGTGCAAAAAGGCAGTTAATGTTGGAT
										4B-1050740-T6-55_1580	TAAATTCGTTCCAACAAAAGCCAGTCCTG
										4D-1144210-T6-55_448	GTGTGGGCGTTAGACTTTTCGTTCATGTT
										4D-1144210-T6-55_512	TGTCCATGGATGCTTGTGGTACTGCATTG
										4D-1144210-T6-55_576	GTTTGCGAGACAGTGTTGGAAATACCCTA
										4D-1144210-T6-55_640	ATTTCTCCACCTCACAATGATAAGTGTGTG
										4D-1144210-T6-55_704	CAGTGAAATGAAAGAAGAGTCTGCTCAC
										4D-1144210-T6-55_768	CCCACCTTTATGAACACATACATACACGCA
										4D-1144210-T6-55_832	CCTTATGGGCATCTTTGAAATACTGAACCG
										4D-1144210-T6-55_896	ATGCATTCATAGCCAACGTGACGTCTTCTC
										4D-1144210-T6-55_960	AAATCCAAACAATACGAGCATCAATACCTG
										4D-1144210-T6-55_1024	ACGAACCAAACCATGTGAGCCACGGCCA
										4D-1144210-T6-55_1088	TTTTGCGAATACATTCACAGAATAGTCTGC
										4D-1144210-T6-55_1152	CGACTCTCGAGTTAAGCGCGACGGATGA
										4D-1144210-T6-55_1344	GTGCGATTTCCTCCGAGGCGTTGCTTGAA
										4D-1144210-T6-55_1408	TCCGAGTTTCATTCAAAAGGAGGAGATAG
										4D-1144210-T6-55_1472	CACAATTTTCAATTTCATTTTTTGATTTGAT
										4A-1013050-T6-56_118	CCTTTGCGTGAACGTACAATTGATAGCGC
										4A-1013050-T6-56_295	GCTGCTTGGATGATAAGCCAAGATTGGTG
										4A-1013050-T6-56_354	ATTCTTCTATTGGACTGTCTGGAGCAAATA
										4A-1013050-T6-56_413	GGTTCTTTGTTTACTGAGAGGTAAAAATAT
										4A-1013050-T6-56_472	CTCCTGAAATGTAGCGTGTCGTGTAGGAG
										4A-1013050-T6-56_529	GTTAATTAGGACGTCTCGTCGCAAAATAC
										4A-1013050-T6-56_877	ATTTGGGGCCCTGATTTCTTAAAAAAATC
										4A-1013050-T6-56_941	ACCCTCACTTAATCATATAATTTAAATGTGT
										4A-1013050-T6-56_1005	TTTTAATGCTCAATGAGTAAAGTGATGCTA
										4A-1013050-T6-56_1197	GCTAAGCCCGCGCAGCGGGCGGCGGGG
										4A-1013050-T6-56_1261	AGTTGTCGAACGGCAGAATTTTTTTCCGG
										4A-1013050-T6-56_1325	ACGCGACACCCAATGGGCCCTTATCTGCC
										4A-1013050-T6-56_1389	ATGGGCCTAGCTGGGCTCCTTGCTACGAG
										4A-1013050-T6-56_1453	AAGACGGACTCAAGAGGTGAGATGGAC
										4A-1013050-T6-56_1517	CAGATGCGTCTACAAGCGTCGTCACCTTG
										4A-1013050-T6-56_1580	CCTTATTACTCCATGGGAAACAATAGATTC
										4B-1069640-T6-56_192	aaggAAAAAATCCTCCTTAGCACTGTCTGG
										4B-1069640-T6-56_256	ACATATTAGACCATGACCTTACCTCAGGC
										4B-1069640-T6-56_448	CTAGCACGGCAGGCTGCCTGACCATCGG
										4B-1069640-T6-56_640	ACCCGGGAGGCGACTTCTTTTCGGTGTCT
										4B-1069640-T6-56_832	ttaagacacttattttgggacggagggagtaCAATT
										4B-1069640-T6-56_896	TGAGGGTATAATTTGCTGTTATATGAATTCT
										4B-1069640-T6-56_1024	AGTTTTCCCCCCTCAAACTGGCCCTCACC
										4B-1069640-T6-56_1088	ATTCATGTATTTTGTGATGGCACGTCCTAA
										4B-1069640-T6-56_1152	TCCTACACGACACGCTACATTTCAGGAGC
										4B-1069640-T6-56_1216	CTCTGGGTAAACAAAGAACCACCCATCTC
										4B-1069640-T6-56_1280	TCCAGAGCAGCCAGACAGTCCAATAGAA
										4B-1069640-T6-56_1344	ACCAATCTTGGCCCATCATCCAAGCCGCT
										4B-1069640-T6-56_1472	AGACTACCCTGGCACCCATTGTTTGATCC
										4D-1144210-T6-56_448	GTGTGGGCGTTAGACTTTTCGTTCATGTT
										4D-1144210-T6-56_512	TGTCCATGGATGCTTGTGGTACTGCATTG
										4D-1144210-T6-56_576	GTTTGCGAGACAGTGTTGGAAATACCCTA
										4D-1144210-T6-56_640	ATTTCTCCACCTCACAATGATAAGTGTGTG
										4D-1144210-T6-56_704	CAGTGAAATGAAAGAAGAGTCTGCTCAC
										4D-1144210-T6-56_768	CCCACCTTTATGAACACATACATACACGCA
										4D-1144210-T6-56_832	CCTTATGGGCATCTTTGAAATACTGAACCG
										4D-1144210-T6-56_896	ATGCATTCATAGCCAACGTGACGTCTTCTC

4D-1144210-T6-56_960	AAATCCAAACAATACGAGCATCAATACCT
4D-1144210-T6-56_1024	ACGAACCAAACCATGTGAGCCACGGCCA
4D-1144210-T6-56_1088	TTTTGCGAATACATTCACAGAATAGTCTG
4D-1144210-T6-56_1152	CGACTCTCGAGTTAAGCGCGACGGATGA
4D-1144210-T6-56_1344	GTGCGATTTCCTCCGAGGCGTTGCTTGAA
4D-1144210-T6-56_1408	TCCGAGTTTCATTCAAAAGGAGGAGATA
4D-1144210-T6-56_1472	CACAATTTTCAATTTCATTTTTTGATTTGA
5A-1279000-T6-57_0	TTCTACAACAAGCCACTTATGTGACTTGTT
5A-1279000-T6-57_64	AATTTGCGATCTTAACCAGAATTTGTGTGT
5A-1279000-T6-57_128	TTATATTTTATTAAGGGTTAATGTTTGTGG
5A-1279000-T6-57_192	CACCACATGGCCACGAGGTCTCACACGG
5A-1279000-T6-57_256	GTGACGCTCGTGCATGAAATCTCTTCCTC
5A-1279000-T6-57_320	TCTCTCTTGCCTTCCTTTGTGTTGTCGACG
5A-1279000-T6-57_512	CCAGAAATCTATGTCCCACGACGACCCAT
5A-1279000-T6-57_576	GACGCCTGTCTCCGCCGGTGCTACAACTT
5A-1279000-T6-57_640	ACGTCGAGGTTTGCTACAACCAGCGACG
5A-1279000-T6-57_704	CGATGACGCCGACAATGATTTGTTGTAGA
5A-1279000-T6-57_768	ATTCATGGCGAATGATGCGACCTGTGATG
5A-1279000-T6-57_832	AGTTGTTACTACCGCCGACGACAATTGCT
5A-1279000-T6-57_896	GCGAAAATCAGGGAAACATTGTGACCGG
5A-1279000-T6-57_960	TTCAACTAGCTACGGAAAAGCTTCAAACA
5A-1279000-T6-57_1024	AGGAGCGCCGACCAATCGCTGGTGTGGT
5A-1279000-T6-57_1088	CTGTCGCGGGGCAGGTTGCCTGCGACGC
5A-1279000-T6-57_1152	GGAAGATATGACAGAAAAAAAGAGGGG
5A-1279000-T6-57_1216	GATCTTGTATCACGCGGTGAGTGAGGCCA
5A-1279000-T6-57_1280	CACTTCTCTTTGTTAAAACCGATAGATATA
5A-1279000-T6-57_1344	GATCATGCTCCAAAAAATTGACATGTGAA
5A-1279000-T6-57_1408	ATTGCAAAGACTGTCATTTATGATATCTTA
5A-1279000-T6-57_1472	AGACATCGCCTGATCGCCACAATAGGAGA
5B-1380480-T6-57_126	TTTTTGGACCCTTTTATAGTAGTACGCTTG
5B-1380480-T6-57_189	GCTAGTCCTACTCTCCTATTGTGTCGATCA
5B-1380480-T6-57_252	ACACGAGTATATAAAGATATCATAAATTATA
5B-1380480-T6-57_315	ACTGTTTTTATGCTATTCACATGTCTGTAA
5B-1380480-T6-57_376	TGTATGACAATTCGGTGTAAAAATATATCT
5B-1380480-T6-57_740	TTTTCCCTTTTTTCTGTCACATCTTCCTTTT
5B-1380480-T6-57_935	AAGCTTTTTTCGTAGCTGGTTGAAGGATT
5B-1380480-T6-57_1000	CACAATGTTTCCTTGATTTCCGTGAACGA
5B-1380480-T6-57_1065	GTCATCGCCGATAGTAACAAGTGTCAAAG
5B-1380480-T6-57_1260	AATCATAGCAAAATCTACAACAAATCATCG
5B-1380480-T6-57_1325	GGTTGTAGAATTGTGGTCGTTGGTTGTAG
5B-1380480-T6-57_1390	GGATGTAGCTCCGACTGGTGAAGGAGCT
5B-1380480-T6-57_1580	GCCATGGCCCCATTCCAACAGCCGATTGG
5D-1479950-T6-57_0	AATCTCTCTCTCACCTTCCTTTGTGTCGTC
5D-1479950-T6-57_64	TCATGCAGTAAGCTCACACATCGTGGCTC
5D-1479950-T6-57_256	CCGTGATGCCTGTCTCTCCGTCGATGCTA
5D-1479950-T6-57_320	AGCTACATCCCGACGAACCTAGTGTTGCA
5D-1479950-T6-57_384	ATTCTACAACCATTCATGGCGATGTTGCG
5D-1479950-T6-57_448	TTTGCTATGATTGGCAACGGCAGGTGCTA
5D-1479950-T6-57_512	CGCCCCCGACAATTTTGTCGCAACCGTCT
5D-1479950-T6-57_576	CAACCATTCATGATGATGACCGGAGCCAT
5D-1479950-T6-57_768	GCTGCTCACCACCGCAAGCAAGAGCACC
5D-1479950-T6-57_832	GTGGGAAGGGGGTGCTTGCTGATTTAAG
5D-1479950-T6-57_960	GAGGCCACCGGCCCAAACCCTGGCCCGC
5D-1479950-T6-57_1024	TTTTTACACCAAATTGTCATACAAGAGTCA
5D-1479950-T6-57_1088	GTGAATAGCATAATAAAATGCATTGCAAA
5D-1479950-T6-57_1152	GAAATTTCGGGGACAACCAATAGAAATC
6D-1695080-T6-58_64	CACCACTCCAACACGAACGCCACAATATA
6D-1695080-T6-58_128	CCAACAAAACAGCGTCTTGACCAAGCGC
6D-1695080-T6-58_192	ACTCTTGATTGCCTGAAAGAGTTGACAGC
6D-1695080-T6-58_256	GACAAATGGGTGGCGACCACTCTTTTTTT
6D-1695080-T6-58_384	GAGGAGTACAACGCCCGTAGGCAGCTAG
2A-0299340-T6-59_256	ACAAAATAAAAGCAAAAAAACAAACAATA
2A-0299340-T6-59_320	AACCAGATAAAACCATTGGAAAAACAAA
2A-0299340-T6-59_512	TTTGATTTAGATTTCGCCTACAAGTTCAAG
2A-0299340-T6-59_576	ATAATATGAACCGTCAGACATATTTTTATA
2A-0299340-T6-59_640	ATAAAACACATTTGATTTAATGAAAGAGG
2A-0299340-T6-59_704	GGAGAGTTGCCTCCTCTCCCGAATCTCAT
2A-0299340-T6-59_768	GTTATGTATCTTGATCAGGTCAGTGATCAC
2A-0299340-T6-59_832	TTAGTCCTTTCCGACGAGAATCCGCGTGA
2A-0299340-T6-59_1024	CACCGAAGCCGACCCTTCTTTCAGCTACG
2A-0299340-T6-59_1088	AGATCGATCTCGCCCGGATCTTCGTTGAC
2A-0299340-T6-59_1216	TTTATCGACGATGCGTGCCGAGTAGGAAC
2A-0299340-T6-59_1280	CTCCTGCACCGTTGTTTGCACACAAGCTC
2A-0299340-T6-59_1408	GCTCCCAAGCTTTTTGGTCGAAGCTAGAG
2A-0299340-T6-59_1472	GTACGCGCAATTATCCATTCTAACCGAAGA
2A-0299340-T6-59_1536	CTTGGAATCACTCACAGCTCTCTCAAGAG
2B-0399330-T6-59_124	ACAGCCTCACGGGGAGCAGCCACCGATT
2B-0399330-T6-59_186	CCTCTTCCGTCGCCTTTCCATCATTTTCTG
2B-0399330-T6-59_248	GTCGAAGCTAGAGGGCCCAGCAGGTGAG
2B-0399330-T6-59_310	ATTCTAACCGAAGAATCAAGAATGTCCTT
2B-0399330-T6-59_372	CAGCTCTCTCGAGAGAGCTCGCTCGGTG
2B-0399330-T6-59_434	ACGAACACCGTCCTCTGTCCGGCGGTCCG
2B-0399330-T6-59_744	TCGGCATACAAGCAAACTGACGGGAGTT
2B-0399330-T6-59_868	GAGAGATTTTTATCTAAAAAATAACAGAG
2B-0399330-T6-59_1054	CCCAAGTGCATGTTATATAACCGGTGCTTC
2B-0399330-T6-59_1302	tgTCTACCCCACCAGCGACCATTTAGTGCC
2B-0399330-T6-59_1364	CTGTATGCCTGTGTATTGCCATATGAGATT
2B-0399330-T6-59_1426	TCCTTTGCACAAGAAAGTTGCACAATTGA
2B-0399330-T6-59_1488	TCTAAAATTTCTGAATATAATGTAGTTTCAT
2B-0399330-T6-59_1550	CAAATTGAAATTTACAGGTATATAATGTAG
2B-0399330-T6-59_1612	GATACAAATTTAAATTTACTGGTTATGAAC
2B-0399330-T6-59_1674	GAGTAATTGAAGAGGGAAGAGGACCATA
2B-0399330-T6-59_1798	ATTTTAGTATGTCTGCAACAACATATTATTC
2B-0399330-T6-59_1860	GTCGAAATTGTGGACCACCCTGTTTTCATA
2B-0399330-T6-59_1922	AAATCACACGGATTCTCGTCTGAAAGGAC
2B-0399330-T6-59_1984	AACTAGTAGTAGTGATCACTGATCTGATCA
2B-0399330-T6-59_2108	TTAAATCAAAGGTGTTTTATTGTTTGTTTG
2B-0399330-T6-59_2294	AACTCTCTTATCCCGGTCTATTTTGAACCT
2B-0399330-T6-59_2331	ATTTAGTTGGTATTCCTTTTTCTTTATTTTT
2D-0553420-T6-59_124	AGGAGTCATCGGACCGCCGGACAGAGGA
2D-0553420-T6-59_186	GTACTACTACTAGCTCCGAGTGAGCTCGA
2D-0553420-T6-59_248	CCATGGAGCAAGGAGGACATTCTTGATTC
2D-0553420-T6-59_434	TCCGCACCGCAGCAATCGGTGGCTGCTCC
2D-0553420-T6-59_558	ACGCATCGTCGCTAAAATATCTGCTGATCA
2D-0553420-T6-59_620	TCTAGACCAAGGGGCTGAAATTGGAGAG
2D-0553420-T6-59_682	CCGGGCGAGATCGATCTACGGTTTGCTTG
2D-0553420-T6-59_868	CGACTCGAAAAGCAAGAAATGAAGCGGG
2D-0553420-T6-59_930	CCTCCCGCAACCGTTCGAACCGGACCGG
2A-0292820-T6-60_0	CGGGATCTTCTTCCTAACTCTGCGGATTAG
2A-0292820-T6-60_64	AGAAAACTGCAACTTCCCTTTTCTTAAAAT
2A-0292820-T6-60_128	GACCATGGTAAAAACCGCTGAGGACAAA
2A-0292820-T6-60_192	TTAAAGTTGGGAGACCAAATCTAAACTTT
2A-0292820-T6-60_256	ATCATCAGTAGTCACAAGACTTGCGTCGA
2A-0292820-T6-60_448	TGCGCAAAACCCCGTCGATGAACCAGAG
2A-0292820-T6-60_512	CTAGACCCGGACGCGCTTCTCCTTGGGTT
2A-0292820-T6-60_576	GGCAACGCCCTGCGGTGAACATCCAACT
2B-0436110-T6-60_128	GATACTGACGCGCCGTGCGGATTGGATG
2B-0436110-T6-60_192	CAGCTATAGTACACTGACTGGTAGATGTT
2B-0436110-T6-60_256	TTGATATTCATTCAGTTGGCGCCGGCTGA
2B-0436110-T6-60_320	CCTGTCCTCCTATCCTAAACTAATCCAGCG
2B-0436110-T6-60_384	ATGAAATGGAAATCTCTGTCCGAGCTTAC
2B-0436110-T6-60_512	ATGCGCTCCCTCTGTAAACCAGCGCAGAA
2B-0436110-T6-60_576	AAACTGAGGTTCCAACGCTTATCCGACTG
2B-0436110-T6-60_640	CCTCGTGTACATACATAGATAAATGCATCT
2B-0436110-T6-60_704	CCGCTTTCGCTGGCTGGCCCGAGCACGA
2B-0436110-T6-60_768	ACTCTGGGGCGCGGCAGGCGCTATCTCC
2B-0436110-T6-60_832	GGCTCCACTTGGATAGGTACTACACATAT
2B-0436110-T6-60_1024	ACGTACTGACAGCGAGCGATCCCCCTAAT
2B-0436110-T6-60_1088	AAGATTCCTCCAAGTCCCACGACTTTGAC
2B-0436110-T6-60_1280	GGCCATGCTTGACTGGAGATCAAATGCTC
2B-0436110-T6-60_1408	GGCCAGCGTGCGAAAGCACGTAAGCTAC
2D-0517040-T6-60_128	ACTTTGATACTGACGCACACCGTGCGGAT
2D-0517040-T6-60_192	ATACTATTCCAGCTATACATACTACTACCAG
2D-0517040-T6-60_256	ATTTCGAGTTAGATGTGAATATTGATTATT
2D-0517040-T6-60_320	GTTCAAGTGGCCGTTGTTTAGCCGTACGT
2D-0517040-T6-60_384	CGAGCCTGTCCGCCTAAACTAATCGGGCG

2D-0517040-T6-60_768	TTAGTCAGACTAAAATAAGTCCCTTAGATC
2D-0517040-T6-60_832	GAATGCAGACAGCTTTCGGGGAGTAGTG
2D-0517040-T6-60_896	GTGAATCGGGAATGCACAGCTAAGACCG
2D-0517040-T6-60_960	CGGCACAAAATTTGGCCTGGTTTTCTTCA
2D-0517040-T6-60_1024	CAACGCTTATCCGATTCCAGCTGTATAACA
2D-0517040-T6-60_1088	CCTCGTGTACATACATATACATAGATAAATG
2D-0517040-T6-60_1152	TCAGTTCAAGGCCGCTTTCGCTGGCTGG
2D-0517040-T6-60_1280	TCGATCACACGTCCAGCTCCACTTGGATG
2D-0517040-T6-60_1408	CAGCACGCGGGCAGCATGTGAAAGCAG
2D-0517040-T6-60_1472	ATTGATCCCCTAATAATCGATGCGTATAAAT
1A-0081220-T6-61_248	AAAGGATCACAGCAATTAGGAGCTTCTTC
1A-0081220-T6-61_372	AGAAGCGGATGAGATGTCCATGGTTACTT
1A-0081220-T6-61_434	CCATCTAAAAACAACAACTGAGTCAATAC
1A-0081220-T6-61_496	CAAGATGCTATAATAAGAAGAAAAAAAAT
1A-0081220-T6-61_558	ATGAAGTGACCACATGTGAAAAAAATACT
1A-0081220-T6-61_620	GAATATTCATAAAATAATAGTCAATAAAGA
1A-0081220-T6-61_682	CAGTCATTAAGATCGAAATCCACAGAGGA
1A-0081220-T6-61_806	GTCAGCGAAGGCAAAGTCAATATTCAGTA
1A-0081220-T6-61_868	CTAGCCAAACAACATATGCCTACGAACTA
1A-0081220-T6-61_930	CCTTTATGTGAACAACACGGAGCAGAGTA
1A-0081220-T6-61_1054	TCGCAGAAGAAAATCACTGTGCGGGCAC
1A-0081220-T6-61_1116	TGCAGATTCGATCACACCTAAAAAACCCT
1A-0081220-T6-61_1178	CGGCAAGACCACACAAACATCAGCAGTA
1A-0081220-T6-61_1240	TACCTCGGCCCCCAAACCACAGAAGAATC
1A-0081220-T6-61_1302	GGCAGAAAAAAAAGATGCCTCTTCTACCC
1A-0081220-T6-61_1426	ACAAATTGGAGTTTGGCACCACGGGAGG
1A-0081220-T6-61_1612	ATGGAATCGCGTCGCAAGGACCCGACGC
1A-0081220-T6-61_1798	CCGCGTCCACAATCACCGGTGACTGAAAC
1A-0081220-T6-61_1922	CTCTCGCCATGGAGGGTCCATTGGTGGGA
1B-0162340-T6-61_64	GTCCATGGTTACTTCAGGAACATGGGGCG
1B-0162340-T6-61_128	CTGAGTCAATACACGAGTACAATAATGATA
1B-0162340-T6-61_192	AAAATTATGTGCGTAGTTCCTTACTTCTAG
1B-0162340-T6-61_256	AAGTGCCCACATATGAAAAATACTATCACA
1B-0162340-T6-61_320	TTGGTATGCAGAAATAATAGTCAAATAAGA
1B-0162340-T6-61_384	GTCATTAAGATTGAAATCCACAGGATATG
1B-0162340-T6-61_448	AGCATATTTTAGCATGCAGTTCATAACAAT
1B-0162340-T6-61_512	GATGACATCAGCTATGAAAAGGTCAGCCA
1B-0162340-T6-61_576	GGCAGGAATCTGGAGCTTGCTAGCCAAA
1B-0162340-T6-61_640	AGATAAAACTAACTTTCCACCTTTATGTGA
1B-0162340-T6-61_704	GCAAATTCAAAAAAGAATTAACAGAAAG
1B-0162340-T6-61_768	CAAAACTTTGAACAAAATCACTGTACGGG
1B-0162340-T6-61_832	CCACGAATTCGATCCACCTAAAACCTCAA
1B-0162340-T6-61_896	ACAGACATCAACACTACTAATCTGCAAAC
1B-0162340-T6-61_960	CCGGAGATCAATCAGATACCCGGCATTCA
1B-0162340-T6-61_1152	TAGGGGCAGCTGGGATGGATAAGTACTC
1B-0162340-T6-61_1216	TGATTAATCCGCCACGGCACGCCGCTGGC
1B-0162340-T6-61_1408	CATCCTTCGAATTTTCTTCCCGCAATCTCT
1B-0162340-T6-61_1472	CGTCCACAATCACCGCTAACTGAAACCCA
1D-0248740-T6-61_0	CTGAAAACAACAACTGAGTCAATACACAA
1D-0248740-T6-61_64	GCTATAATAATAAGAAGAAAATTCTTGTGT
1D-0248740-T6-61_128	CCCACATGTGAAAAAAATACTATCACAAG
1D-0248740-T6-61_192	AAAATAATAGTCAATTAAGATCACGATCCA
1D-0248740-T6-61_256	TTGAAATCCACAAAGGATAACATATCTACC
1D-0248740-T6-61_384	TCAATGTTCAGTAGGTTTGCAAAACATGG
1D-0248740-T6-61_448	CCTACGAACTAACGACAGTGATTTGAGAT
1D-0248740-T6-61_512	AGCAGAGTAACTCAAGTGGCCACACCTA
1D-0248740-T6-61_640	CAGAACAAAATCACTGTGCGGGCACTGC
1D-0248740-T6-61_704	ATTCGATCACACCTTAAAAAAACCTGAAC
1D-0248740-T6-61_768	GACTACACAGACATGAACACTACTAATCT
1D-0248740-T6-61_832	AAATTACGGGAGATCAATCAGATACGCGG
1D-0248740-T6-61_896	CCAATATTCACCAATCTGGTAGTAGACACA
1D-0248740-T6-61_960	GATGCTGCACAGAATGAGGGGAAAAGCG
1D-0248740-T6-61_1024	CTAGTAGTGGTAGCTGCGATGGATAAGTA
1D-0248740-T6-61_1216	AAATAAACATGCGCCGAGACAGCGACCC
1D-0248740-T6-61_1344	CCCGGCCGCGTCCTACCTCCCCTCCTCTCC
1D-0248740-T6-61_1536	GACGGAGCGGAGTAGAAAATCAATCCAC
2A-0366380-T6-62_0	ACATGCATGGTAATAATTGGTTTGTCCTCA
2A-0366380-T6-62_192	ATGATATGTGTCGTGTTGTGGTGTGGTGA
2A-0366380-T6-62_256	TTGTTGTAATAAGTGTTTTTGACACTCTAT
2A-0366380-T6-62_320	TGTAACACCGTACCGATGCTTTCCTAATTG
2A-0366380-T6-62_384	CTTCTTTTTTAAGAAAATAATCATCTCGAT
2A-0366380-T6-62_512	GTTGCCAAATGATTGGAGTGCGGGATTTT
2A-0366380-T6-62_576	TTGTGTGCCAGCCATTTTAATGAATGGAA
2A-0366380-T6-62_704	TCCTTGTTGCTCCAAGTAGACCATTTAGG
2A-0366380-T6-62_768	AAAGAGGAGCAATGCTGTAGGGATGCAA
2A-0366380-T6-62_960	GTATTTGGCTAGTTTCCACCGGCGGGATC
2A-0366380-T6-62_1152	TTACTATTTAAAAAATTCCGAAAAAAATCA
2A-0366380-T6-62_1216	TTATCATTACAGTCATACAAAATTTTTTATC
2A-0366380-T6-62_1344	ATAACAACATGAAAGGCATTTCTTCGCAA
2B-0480120-T6-62_0	CTATCAAAGCGGAGTTAATTTGATTCATTC
2B-0480120-T6-62_64	AGTCGCAAACCTTCTGAAGGCCGAGGTC
2B-0480120-T6-62_128	CTTCTCACTGTTTTTCCCGCTAGGATTGAA
2B-0480120-T6-62_192	ATTAACGACGCATTCGTAAAAAGTTCACC
2B-0480120-T6-62_320	ACATGCCGGACATGTCGTTGCCTAAGTGG
2B-0480120-T6-62_512	TTGGGCTGAGGTGGATAGTGCCTTTCACG
2B-0480120-T6-62_640	GAATGATAACTAGATTTATTCGGTGGCTAG
2B-0480120-T6-62_704	TAAAAATAATACACTATAGATCTTGAATTT
2B-0480120-T6-62_896	CCGTTGGATACTCGTAAAAATTAAGCTAG
2B-0480120-T6-62_1088	ATTCGTATGTAGTTCATATTAAAATCTCTAA
2B-0480120-T6-62_1152	TGAATCTACACTCTAAAATATGTCTATATAC
2B-0480120-T6-62_1280	GTGTATATTAAAATCTCTCAGAAAGAATTA
2B-0480120-T6-62_1580	CCCTCGCCTCAATCACTCACTCTCAGAGC
2D-0593010-T6-62_0	CCATTGTAGAATCTCCTTGTATGTTGATGA
2D-0593010-T6-62_128	CTGAAGACCGAGGTCTTCCCCATCCATTG
2D-0593010-T6-62_192	CCCCCGCTATGATTGGAAACCTTCTCGTG
2D-0593010-T6-62_256	TTCGCAAAAGGTTCGCCTGCAGTCGCTCA
2D-0593010-T6-62_384	TTTTGTTGCCTAAGTGGGCGAGTGCCAAG
2D-0593010-T6-62_448	TGGCGATAGTGAGACCGCTGGTGGTAGG
2D-0593010-T6-62_640	ATCCACAACTGGCACGACAACTGGACTTG
2D-0593010-T6-62_704	CACCATTGCATTTTGGAAAAACAGGACG
2D-0593010-T6-62_768	TCGGTGGCTAGATTAAGCGGACCTGAGC
2D-0593010-T6-62_960	GATAGTATTTGGATGCTCTCGCGCCTTTCT
2D-0593010-T6-62_1024	ACTTGTGATGACACATACTCCCTTCATTCT
2D-0593010-T6-62_1280	GTATATAGTATATATTAAAATCTTTATAAAAT
3A-0646970-T6-63_0	GCGGCGGAAAGGGCAAGGAACACATGA
3A-0646970-T6-63_64	AAGTTGTTATCGGTTCTAAATGCAATGTTT
3A-0646970-T6-63_128	CAGAGATTGCTCAAGTAATGAAGTATTCA
3A-0646970-T6-63_192	CAGAAATGGAGCGCTACGAGAGCTCATG
3A-0646970-T6-63_320	TGTTGCGTGTTTGTTGTCTTCGAGATGCA
3A-0646970-T6-63_512	GGCCTGAAAGAGCAGGCAGTGCGAGAC
3A-0646970-T6-63_576	CGCTGGTTGTCCCCAAATCCGTCACGGTT
3A-0646970-T6-63_640	TAATTTCAAAAGGTAGATCTGAACTCCAA
3A-0646970-T6-63_704	TGGTCATACATGGTATGGATTTGTGGCGA
3A-0646970-T6-63_768	ATCTTCTATGTCCTCGGGAAGAAGTCGTA
3A-0646970-T6-63_832	CAATCTAAAAAGTGTGCGTGCTTGATTGC
3A-0646970-T6-63_896	TGTGGATTTACAATTCATTTAAAGTTAGAG
3A-0646970-T6-63_960	ATGTTTCGGTGACTGTTCACACCTTGGTC
3A-0646970-T6-63_1024	TTAGTAATATAAAATTATACATCTTTACCAA
3A-0646970-T6-63_1088	ATGCTATAACCCACATTGTGCTTTTTCATG
3A-0646970-T6-63_1152	TCCATGAAAATAGTCTCTTTCAGATGGGC
3A-0646970-T6-63_1216	TCAATCTTGTATAATGTAGTTGCAATTTTTC
3A-0646970-T6-63_1280	TTCAGAACAAACAACTGGGACAACAACA
3A-0646970-T6-63_1344	AGAGGACAAGAAGGCGAAAAAATAATTT
3A-0646970-T6-63_1408	GAGAATACTTTCAAAACAACAACTACAAG
3B-0712080-T6-63-1_64	GCTATAACCCACAATGTGCTTTGTCATGCA
3B-0712080-T6-63-2_64	TGACTTTTACAAAAAAGACCCAAAACCAT
3B-0712080-T6-63-1_128	GAAAATAGTCTCTTTCAAGTGGGTTCATT
3B-0712080-T6-63-2_128	CACCCGAACAACCTCACCGGTATCGGGG
3B-0712080-T6-63-1_192	CTAGTATAATGTAGCGTTGCGAGTGCTCA
3B-0712080-T6-63-2_192	CATGTTGCGGGCTCCCTGATAAACAAATG
3B-0712080-T6-63-1_256	GGGCTCTGTAGGCCAGTCCCCCAAATGAT
3B-0712080-T6-63-2_256	GATCTAGCCACCCTTCAGCCCGCAGATGC
3B-0712080-T6-63-1_320	GGTTTGACGCGTATTTATTGTCTTCGAGAT
3B-0712080-T6-63-2_320	ACAATTGGCGCCAAGGCCTCCCACAAAC
3B-0712080-T6-63-1_384	TTGCCCCAGATCCAGCTCTTTCACATCGCA
3B-0712080-T6-63-2_384	CCATCTCTTACCACTTCATGCCCACCGCCA
3B-0712080-T6-63-2_448	CTCCTTCTCCAAAATCGCTATCACCACGAG
3B-0712080-T6-63-2_512	GCCACACTGAGGAAGAAAAGCATACCATA
3B-0712080-T6-63-1_576	CGCGGCGACACTGGTTTTCTCCAAATCGG
3B-0712080-T6-63-2_576	CGCAAGGCGGGCCTCCAAGGGAGGTTG
3B-0712080-T6-63-1_640	CTCCGGGGCTGATTTTAAAAAGTAGATCT
3B-0712080-T6-63-2_640	CCAAAATTGCCTCATTCTGAAGCATGAGG
3B-0712080-T6-63-1_704	TGTTTTTGTTGGTCATACATGGTATGAATT
3B-0712080-T6-63-2_704	CATCAACAAAGATTGCGAGTAAAAATGTG
3B-0712080-T6-63-1_768	AAGGAGTTAATCTTCTCTGTCCTCAGGAA
3B-0712080-T6-63-2_768	atggagatAACCAAAGATGAAGATAAGGAA
3B-0712080-T6-63-1_832	CAATTTTCAATCTAAAAAATGTGCATGCTT
3B-0712080-T6-63-2_832	TAAGAATATCTAGAGAAAAAACGAGAAAA

3B-0712080-T6-63-1_896	AGGATTACTCCATATACTAATCAAACAAAA
3B-0712080-T6-63-2_896	CCAATAAAGAGAAAAGTAAGGCGACAAA
3B-0712080-T6-63-1_960	CAATCAGATTGTTCGCTTATGGAGCATTC
3B-0712080-T6-63-2_960	TTCACATCCCACTTTCATGGATCAGTCGCC
3B-0712080-T6-63-1_1024	CAAAATAATATGCATCGTTCTAGTCATGAT
3B-0712080-T6-63-2_1024	GAATTGGAGACCACATCACCTCCACACCG
3B-0712080-T6-63-1_1088	TGCGTTCATTCCTCCAAGGACAAATGTCA
3B-0712080-T6-63-2_1088	TGACACAAAATCAAAGGCTATATTAACATG
3B-0712080-T6-63-1_1152	ATTCTATTTCAGACTTCCTGATTCTTTCATG
3B-0712080-T6-63-2_1152	CCAGCCAGCGAAGCCGACAGAGAGAAG
3B-0712080-T6-63-1_1216	TATCTCAGAATACCTAATCAAATACACAAA
3B-0712080-T6-63-2_1216	AACGCTTGAGAAGGAGGAGAGAGAATG
3B-0712080-T6-63-1_1280	TGTCCTCAACCTAGGTATAAAAAAACCCC
3B-0712080-T6-63-2_1280	GGTTCTAAATGAAACGTTTAATTATGTGA
3B-0712080-T6-63-1_1344	ATTAATCACACAGAGGACAAGAAGGCGA
3B-0712080-T6-63-2_1344	GTATTCATACGAGGGTATTGTCCCTAGATA
3B-0712080-T6-63-1_1408	TCAAACACCGAGAATGCTTTCAAAACAA
3B-0712080-T6-63-2_1408	GCTTAGGCAAACAACAGCATGGTGGGAC
3B-0712080-T6-63-2_1472	AACGATGGTCTTGTGTTGTTCGAAGTCCA
3B-0712080-T6-63-2_1580	GCCCCCAGATTTAGCTCTTCCACACCGCA
3B-0712080-T6-63-1_0	GTAATGTATAAAACTATTTTTTTTACCAAA
3B-0712080-T6-63-2_0	ATTAATTAAACATAGCAAGTACACAACATG
3D-0848520-T6-63-1_0	GGAGAGAGAGAAAGAAACGGGGAGAAT
3D-0848520-T6-63-1_64	GTTTAATCATATGAAAAAGAGAACAAACC
3D-0848520-T6-63-2_124	GAAATCCCGGACTGCATGTTCTGATCAGA
3D-0848520-T6-63-1_128	TCACACAAGGGCACTGTCCTTAAAGACA
3D-0848520-T6-63-2_186	AAGCTAGTTATCGACCTATGCACCTACACG
3D-0848520-T6-63-1_192	CCGCGTGGTGGGCCCCGTCCGGCTTGTG
3D-0848520-T6-63-2_248	GAGTCTCTAAGGATATTTGATTGATGTGCA
3D-0848520-T6-63-1_256	GTGTTCAAAGTCCGGGTGCGGCTTGTCG
3D-0848520-T6-63-2_310	GATTGCCTTTTTTCTACTACAACTCTTAATT
3D-0848520-T6-63-1_320	ACTGATACAAAAGAGTAAGGCACCCGCA
3D-0848520-T6-63-2_372	GAAATCCGGCTGTTGTAAATTAATGGCAA
3D-0848520-T6-63-2_434	AAAATCTTATATTATGAGACAGAGGGAGT
3D-0848520-T6-63-2_496	TTCTGAAATAAATGGCAGTTGTAAATTCA
3D-0848520-T6-63-1_512	AGCAGCGCGAGAGGCCGCCAAGGCCCT
3D-0848520-T6-63-2_558	TGGCATCAATTACTCATTTTATCTTGCATG
3D-0848520-T6-63-1_576	TCACGGTTCGCATACAGTCCACCTCTCCC
3D-0848520-T6-63-2_620	GAAATTATTCTTAACTAGGAGCCTTGTTCT
3D-0848520-T6-63-1_640	GCAAAATCACCGGGCTTTCATGAAAATTG
3D-0848520-T6-63-2_682	ACATCTATCTCTAAATTGGGATTTTCACTTT
3D-0848520-T6-63-1_704	CTTATGGAATTTCTAGATGTTGCCAAAGG
3D-0848520-T6-63-2_736	TAGGGTACATATATACAATTATTTACCTATC
3D-0848520-T6-63-1_768	ATTTCATCGGTATCTTATCTTTCGGTATAAT
3D-0848520-T6-63-1_832	GAGTACCATTACTTTGCTATGTCACATGGT
3D-0848520-T6-63-1_896	ATCACTCCATATGCTAACCAAACAAAATTT
3D-0848520-T6-63-1_960	TTAGATTGTTCGCTTATGGAGCATTCTTATT
3D-0848520-T6-63-1_1024	AATAACACGCCTCGTTCTGCTCATGCTTGA
3D-0848520-T6-63-1_1088	TTCATTCCCCAAGGACAAATGGCACAAAT
3D-0848520-T6-63-1_1152	ATTTCATACTTCCTGATTTTTTCATGCTTCA
3D-0848520-T6-63-2_1185	AATCTACCTGGTGTAAAGTGCAATTTACTC
3D-0848520-T6-63-1_1216	CGTTTTACACACTAGTCTTATATTATGGGA
3D-0848520-T6-63-2_1251	AAGGGAGCCTCCACTTAGTAAATATAAAA
3D-0848520-T6-63-1_1280	CCCAGAATACCTAACCAAATACTCAAATAT
3D-0848520-T6-63-2_1317	TAAAGAGGCTTCCCATCATTGATCAGAAG
3D-0848520-T6-63-1_1344	ATAGAAAATAATCACACAGAGGACAAGA
3D-0848520-T6-63-2_1383	CAAACAAGTGGGACAGCTGGGTTGCGAT
3D-0848520-T6-63-1_1408	GGCCTAATCAAACACCGAGAATACTTTCA
3D-0848520-T6-63-2_1449	ATGTGGCAATTTTATTTTTTTTGGATTACG
3D-0848520-T6-63-1_1472	AGTCTTCTCCTAGTAGAAAAATAACCACA
3D-0848520-T6-63-2_1515	ATTCTAAAATCATATCTAGATGGTGTGATT
4B-1039150-T6-64_320	GATACAAATCTTTTGCCTTCGTTTCAAAAA
4B-1039150-T6-64_448	AGAGATTAGGTAATACATTTGTACGTCAA
4B-1039150-T6-64_576	GCCGGCCAGCCATACACAGATATACACTT
4B-1039150-T6-64_640	TACGTACGTGCTTATGGTTGTAGTATATCT
4B-1039150-T6-64_704	TGGCAATGTTCATGCTGCCTCCGGAGAAA
4B-1039150-T6-64_896	AACCTAAATGAGTCCATATATCAACTCCAC
4B-1039150-T6-64_960	ATATAGGGAGTTACTTTCAAGGAAGGATT
4B-1039150-T6-64_1024	AGTTAAAAAAAGGTGTGGCTAGGTCTTAA
4B-1039150-T6-64_1088	GAAGAAAATAGTTAAAAGGAAAATTTTG
4B-1039150-T6-64_1152	TGACATTTAGCAATCCTGTAAAAAAGACT
4B-1039150-T6-64_1216	ATATGTGACAACACAGTGACAAGCAACG
4B-1039150-T6-64_1408	TCCATTTTCGTGATGTCACTTTTTGACCCC
4B-1039150-T6-64_1580	cattgcacggcTTGGCTTAGCTTACCTGCCTA
4D-1125130-T6-64_704	TTCAAGAGCCCGTGGCAACGCACGGACA
4D-1125130-T6-64_768	TTGATCGAGATAAGTGGTTGGTGTGGCA
4D-1125130-T6-64_960	GTGATTAGCCAACAAAATCGGCAACCTAA
4D-1125130-T6-64_1024	CTTTTCGTCGGTCCTACATTGGTATACAGG
4D-1125130-T6-64_1088	CTGGTTGAAAGCTGATGATTATTAGTTAA
4D-1125130-T6-64_1152	CAAGTGAAATAACATATAAAGAAGAAGA
4D-1125130-T6-64_1216	GTACAATATTATGAACAGTCGACTGAGATT
4D-1125130-T6-64_1344	TGAAAATTTACTCTCTCTGCGCCTCTCTTG
4D-1125130-T6-64_1408	TTCCATTTTCGTAATGTCACTTTTTGACCC
4D-1125130-T6-64_1580	GCATTGCACGGCTTGGCTTAGCTTACCTA
4A-1010510-T6-65_64	CGAGAAATGAGAACATCAATCCCTATCTA
4A-1010510-T6-65_256	TGAAACAATTTACAGCAAGGGAAAACCA
4A-1010510-T6-65_320	AATCAGAAACTTATGCATGAACAACAAGA
4A-1010510-T6-65_1088	CTTCAGAATTCAGAAGAGCACTGGCCACT
4A-1010510-T6-65_1152	ACCCTTCCGTATGCTTTTGCATGTTACACG
4A-1010510-T6-65_1216	CGATCAAGTTTTTTCTTTTTCTGCTGTACT
4A-1010510-T6-65_1280	TGAGGAACATCGCTCCCAATTTCATTCAA
4A-1010510-T6-65_1344	AGGCTTCTTTACTGGACATCGACAAGATA
4A-1010510-T6-65_1408	TGATCAAGTCGACAAGGAAAAAAAATACA
4A-1010510-T6-65_1580	GCAGCAGAAGTACAGGATCAGACTGGAG
4B-1052590-T6-65_0	TATCACCGACAAGGTTGCTTGTGCCTATGT
4B-1052590-T6-65_63	AACATCAATCCCTATGCCGAAAGAGATAAT
4B-1052590-T6-65_126	ACATGAACTCTCCATGTTTGTTTCTGATGG
4B-1052590-T6-65_189	TACACAACAGTACTGATACACAAACGCAT
4B-1052590-T6-65_252	TAAGCTCTGGACTGAATGAAACAAGTTAC
4B-1052590-T6-65_315	CTTCAGAACCGAAGGAAGAAATCAGAAC
4B-1052590-T6-65_378	CCCAACACCACTGATTTTATTTATAAAGCG
4B-1052590-T6-65_441	TTTCGTAACCATGAAGAATTAATGGAACG
4B-1052590-T6-65_819	CAATGAAACTGCGTTTCAGTTCTCTCTGT
4B-1052590-T6-65_1463	AAACTTCAGTATTCAGAGGAGCACTGTCC
4B-1052590-T6-65_1522	TCTATTTTTAGCCTGCCCTATGCTTGTGCTT
4B-1052590-T6-65_1580	CATAGTCTGCCTAGTTTTGGCATTTCTCTC
4D-1125130-T6-65_704	TTCAAGAGCCCGTGGCAACGCACGGACA
4D-1125130-T6-65_768	TTGATCGAGATAAGTGGTTGGTGTGGCA
4D-1125130-T6-65_960	GTGATTAGCCAACAAAATCGGCAACCTAA
4D-1125130-T6-65_1024	CTTTTCGTCGGTCCTACATTGGTATACAGG
4D-1125130-T6-65_1088	CTGGTTGAAAGCTGATGATTATTAGTTAA
4D-1125130-T6-65_1152	CAAGTGAAATAACATATAAAGAAGAAGA
4D-1125130-T6-65_1216	GTACAATATTATGAACAGTCGACTGAGATT
4D-1125130-T6-65_1344	TGAAAATTTACTCTCTCTGCGCCTCTCTTG
4D-1125130-T6-65_1408	TTCCATTTTCGTAATGTCACTTTTTGACCC
4D-1125130-T6-65_1580	GCATTGCACGGCTTGGCTTAGCTTACCTA
4A-1009640-T6-66_0	TACCTTTAGTAAGATGATCTTTCACTGCAC
4A-1009640-T6-66_63	AAATTAATTCGTGACAGTAGTAAAATTACT
4A-1009640-T6-66_126	ACGAATTAATTTCTTGCATCCATGCTCATC
4A-1009640-T6-66_189	TTTACTACTGTCACAAATTACTGCACGGA
4A-1009640-T6-66_252	CGTGACAGTAGTAAAATTACTATGTTACAT
4A-1009640-T6-66_311	CCGCGGCCCATTGTTGATCCGAATCTCTAT
4A-1009640-T6-66_1250	GCCCGGCCGTCACGGCGGCCGGTCGCTT
4A-1009640-T6-66_1382	acGGCACGAGCTGATTCCTTCCTCCATGT
4A-1009640-T6-66_1580	GAGCTGCCGCTTCTCCTTGTGCTCTCATC
4B-1034040-T6-66_0	GCATGCACTATCTCGTAACCATTTTTTTTA
4B-1034040-T6-66_64	TGTTAGAATGGCCCAAACTACATTAGAAA
4B-1034040-T6-66_128	AAGATGATCTTTCACTGCACGCACACAAT
4B-1034040-T6-66_192	TGACAGTTGTAAAAATTACTACGTTGCATA
4B-1034040-T6-66_256	ATGCTTTAAGGTATATATTATTTGCAGCGT
4B-1034040-T6-66_320	GGTATCAAGTTAGTTGTTCCCCTCCGATAC
4B-1034040-T6-66_512	GATCCGAATCTCTATCCTGTTACTTGCATCT
4B-1034040-T6-66_576	TTGGCATGCAACGATTGTGGTTCGATGGG
4B-1034040-T6-66_640	TTGCGACAAAAATGTTCTTGCGTTTCAAA
4B-1034040-T6-66_704	AGCAAACAAAAGCATGCATTTGCCGTACG
4B-1034040-T6-66_1216	GCCCGTAACGCCCGGCCATGTCGCGTTG

										4B-1034040-T6-66_1344	GCACACTGACCACACGCACACATAACTGC
										4B-1034040-T6-66_1408	GCTGATTTATCTCTCTTTTTTTCAGAGGGA
										4B-1034040-T6-66_1580	TGCCGCTTCTCCTTGTGCTCTCATCCCTCC
										4D-1115020-T6-66-2_0	CGCAGCGTATATCAGAGGTGGAACGAAG
										4D-1115020-T6-66-1_64	CGAGAAATGAGAACATCAATCCCTATGTA
										4D-1115020-T6-66-1_128	GAAGTTAGAACTAGAAACGAACTCTCCAT
										4D-1115020-T6-66-2_128	GTATATATGCTGAACTTACAGAGCCCAACA
										4D-1115020-T6-66-1_192	acacacacacacacacacatggacatgcatgacc
										4D-1115020-T6-66-2_192	TGTCCTGGTGTCCCATGAGAGAGCACATG
										4D-1115020-T6-66-1_256	CTCTGAACTGATCAACTGAATGAAACAAT
										4D-1115020-T6-66-1_320	TCCGGAACTGAAGGAAGGAATCAGAAAC
										4D-1115020-T6-66-2_512	AACCTGACCTTGTATCCATGGTGGTCTGT
										4D-1115020-T6-66-2_640	CGGTGCGGGGATGGGAGTGTCTGTCTGT
										4D-1115020-T6-66-2_704	AACAAGCACATACAAACGCATGAACAAG
										4D-1115020-T6-66-1_768	CAGCGACAGGTGACAGAAGGAACAATCA
										4D-1115020-T6-66-2_768	GTTCCGAATTAAATGGAATTGTTTTATTTA
										4D-1115020-T6-66-1_832	ATTGCAGAAAACACATATGGGCCAGAGT
										4D-1115020-T6-66-2_832	CAGAACCGAAGGAAGGAATTCGAAACTT
										4D-1115020-T6-66-2_896	CCAACAGGACCGATGTATCATTCAGAACA
										4D-1115020-T6-66-1_1024	GCACACGTTCGGTTAATATGCAATCGAAC
										4D-1115020-T6-66-1_1088	ACATTCAGGCTTGATCAAATGCATGTGTC
										4D-1115020-T6-66-2_1088	AATTATGAATCTGCGTCGAGGATTGCACC
										4D-1115020-T6-66-1_1152	AGGAGACTGACCACTGCAAAAGAAACTT
										4D-1115020-T6-66-1_1216	ACCAGCTTATGAGAGCATCACACCCTAGC
										4D-1115020-T6-66-2_1216	TTTTGCAGAACACAACCGGTGTTGAAAG
										4D-1115020-T6-66-1_1280	ACAGCATACTCATCTTTCCAGTATGTATGT
										4D-1115020-T6-66-2_1280	GGCTATGAGCACTCCCTGGTAGTTTATCA
										4D-1115020-T6-66-1_1344	CGACAATACAATTTCTTAAACCGTAGTTAT
										4D-1115020-T6-66-2_1344	CCCTGCCTTGTGTGTGCATGCAGTTATACA
										4D-1115020-T6-66-2_1408	ATCAGAGTCATAAAGGGGATAAAATATTG
										4D-1115020-T6-66-1_1472	CCGTCTGAACATGATCTGCGGCAGCAGA
										4D-1115020-T6-66-1_1536	ATCGCGAAAACAGATCGTTTGATCAACTA
										4D-1115020-T6-66-1_1580	CTAATTAACTAGTACTAGTAGCGTTTGGA
										4D-1115020-T6-66-2_1580	TGCAGATCATGCTCCGTCGGCGGTGCATA
										7A-1793720-T6-67_256	TCGCCCGTCCATCTCTCCCCCTCTCTGACT
										7A-1793720-T6-67_320	CGTGAGTTATAATTTCTTTGTTAAAACTTT
										7A-1793720-T6-67_384	AGAAAATGCAATTTCTTGGAGAGAGAAA
										7A-1793720-T6-67_448	TATCGTAAGGATGCACGTAGCTTCGTCAG
										7A-1793720-T6-67_512	CATTCTAGTGCAAGAGGGGTGACATAAAT
										7A-1793720-T6-67_576	CAGTGATAAAACTTCGCTGCACATTATCA
										7A-1793720-T6-67_768	tAGATTAAGGCTTTCATGTTGTTGTCGAAG
										7A-1793720-T6-67_896	GGGCTAGGGAGGGTGGAGATTGATTATA
										7A-1793720-T6-67_960	TTGAGATGATTGTGGAGGAGAAGATATCA
										7A-1793720-T6-67_1024	TTGACAATTTGTTGCTAATGGCCAACAAT
										7A-1793720-T6-67_1088	AGATGTGATCCACACATCTCACAAGAGGG
										7A-1793720-T6-67_1152	CACAGAAGTACACTTTTTTGCCATATATAT
										7A-1793720-T6-67_1280	CGAGAACAAAGTTAATCATGTTCACCAAT
										7A-1793720-T6-67_1344	AAATAGAAATATGCCTGCCGAACTCTAAG
										7A-1793720-T6-67_1472	TGACATTAATTGGTTGCTAACAAAGTGCC
										7A-1793720-T6-67_1536	TTGCTTCGAGGGTATACTGTGGATTAGAC
										7A-1793720-T6-67_1580	CAACCGTAGCCTATAACCTAAGGTAACTA
										7B-1880190-T6-67_256	GGCTTCGCCCGTCCATCTCTCTCCCTCTCC
										7B-1880190-T6-67_320	CACGTGAGTTAATTTCTTTGTCAAAACTTT
										7B-1880190-T6-67_384	GAAAATGCAATTTCTTGGGGAGAGAAAA
										7B-1880190-T6-67_448	TACCACAAGGATGCACATAACTCCGTCAC
										7B-1880190-T6-67_512	ACATTCTAGTGCAAGAGGGGTGATACAAG
										7B-1880190-T6-67_576	TCTTCGTGCTGCAGTGATAAAACTTCGCT
										7B-1880190-T6-67_768	CTTGCGACAAATGCACGCTCTTGCTTGAG
										7B-1880190-T6-67_832	GTTTCATGCCTCCTCTCTCTCTCTCACTAG
										7B-1880190-T6-67_960	TGTAGCGAGAAGCGTGTAGTGGTATGTAG
										7B-1880190-T6-67_1280	CAGCCCCTAGATCGTGGGTAGGAGAACC
										7B-1880190-T6-67_1344	ATCATATCACCCTCTTCACGACATTTGACG
										7B-1880190-T6-67_1408	CGACGACATATGTAATATTCAATCTTCAAG
										7B-1880190-T6-67_1472	AGGGCGAATTATCAAAATGCGATATGTAT
										4D-1147220-T6-67_0	ACACAGTGGATGCTTGCCTGAGAGGtcga
										4D-1147220-T6-67_192	GTCAGTCAGTCCGTCAGATGTGGCGATGT
										4D-1147220-T6-67_256	AACCAGCTTTTTCTCAACAACAACAAAAA
										7D-1986040-T6-67_256	TGGCTTCGCCCGTCCATCTCTCCCCCTCTC
										4D-1147220-T6-67_320	GGCTCGTGCCGTGCGTGCGGTTATGTGTG
										7D-1986040-T6-67_320	ACACACGTGAGTTAATTTCTTTGTTAAAA
										7D-1986040-T6-67_384	GAGAAAATGCAATTTCTTGGGGAGAGAA
										7D-1986040-T6-67_448	TTTACCGCAAGGATGCACATAGCTCCGTC
										7D-1986040-T6-67_512	TACACAATCTAGTGCAAGAGGGGTGACA
										7D-1986040-T6-67_576	TTTTCTTCGTGTTGCCGTAATAAAACTTCG
										7D-1986040-T6-67_768	ATGCACGCTCTTGCTTGATGCCTCGTCGC
										7D-1986040-T6-67_832	tcctctctctatctctctctctAGATTAAGGCTTTC
										4D-1147220-T6-67_896	TCTCGACCAGCTTTTCTGACCGGCCGGAA
										4D-1147220-T6-67_960	TACGGCAAAGGCACGCTTTAATTTGCTGG
										7D-1986040-T6-67_960	GAGAAGCGTGTAGTGGTATGTAGGGCTA
										4D-1147220-T6-67_1024	AGCATTTGAAACGCAAGAATGTTTTTGTC
										7D-1986040-T6-67_1024	GGGTAGGAGAACCATGGCTCAAAGTTGG
										4D-1147220-T6-67_1088	TTCACCCATCGAACCACAATTGTTGCATG
										7D-1986040-T6-67_1088	TCAGACATTTGACGCAAACAAATTTGACA
										7D-1986040-T6-67_1152	ATTCAATCTTCAAGATGAGATAAAGATGC
										7D-1986040-T6-67_1216	ACGCCATATGTATCACGTAACGTCGGAGA
										4D-1147220-T6-67_1280	TAAAGATCATGATGAAGCTTCAGCGCGAC
										7D-1986040-T6-67_1280	AGTTCTGACCTTTGTTTTATTAAAGAAAAT
										4D-1147220-T6-67_1344	GCACCGGTTTTGAGTATCGGAGGGGAAC
										7D-1986040-T6-67_1344	CTCTTGAGTTTCACGACGAGCACACGAGA
										4D-1147220-T6-67_1408	GTTCTCTTTTTTCACGCCGCAAATAATACC
										7D-1986040-T6-67_1408	TGGAATCCCTCAAACACCACTTTGAAATA
										4D-1147220-T6-67_1472	ACTCTAGATATGCAACGTAGTAATTTTTAC
										4D-1147220-T6-67_1536	ATGTAATAAATTGTGTGCGTGCAGTGAAA
										7D-1986040-T6-67_1536	AACTATGACATGTATGTGGCTTGTTGACAT
										4D-1147220-T6-67_1580	TGTATTACTCAAACTACGGATGCATATCAT
										7D-1986040-T6-67_1580	CAAAGTGCCATGTATGTTGGGCGTACTGT
										2B-0397780-T6-68_128	AAACGATCCTCTTATTATGGCCTAGTTGTT
										2B-0283710-T6-68B_128	AAACGATCCTCTTATTATGGCCTAGTTGTT
										2B-0397780-T6-68_192	GGCTCCAACGCCATGACAAACCCTAGATT
										2B-0283710-T6-68B_192	GGCTCCAACGCCATGACAAACCCTAGATT
										2B-0397780-T6-68_384	AGGGGACAAAACCCTAGAGCAACAAGGA
										2B-0283710-T6-68B_384	AGGGGACAAAACCCTAGAGCAACAAGGA
										2B-0397780-T6-68_448	CCCTATCAATGGAATGCATTAATGAGGATT
										2B-0283710-T6-68B_448	CCCTATCAATGGAATGCATTAATGAGGATT
										2B-0397780-T6-68_512	CATAGTTGGAATGCATTAATGAGGGGGCG
										2B-0283710-T6-68B_512	CATAGTTGGAATGCATTAATGAGGGGGCG
										2B-0397780-T6-68_704	ctccccccctcccccctccctcccttcctgtcctcctccT
										2B-0283710-T6-68B_704	ctccccccctcccccctccctcccttcctgtcctcctccT
										2B-0397780-T6-68_896	GCACCGGCCGGCGGCGTAAGTCCCTCCC
										2B-0283710-T6-68B_896	GCACCGGCCGGCGGCGTAAGTCCCTCCC
										2B-0397780-T6-68_960	CCTTCGCCCTAGATGGATACACAGATCTCT
										2B-0283710-T6-68B_960	CCTTCGCCCTAGATGGATACACAGATCTCT
										2B-0397780-T6-68_1024	GAATGAATCCCATCCCATCCCATCCCATCT
										2B-0283710-T6-68B_1024	GAATGAATCCCATCCCATCCCATCCCATCT
										2B-0397780-T6-68_1088	TGTATCTGTCTGTATTTATTTACTCTCAACA
										2B-0283710-T6-68B_1088	TGTATCTGTCTGTATTTATTTACTCTCAACA
										2B-0397780-T6-68_1152	CAAatacatacatacatagatggatgcatacataca
										2B-0283710-T6-68B_1152	CAAatacatacatacatagatggatgcatacataca
										2B-0397780-T6-68_1216	TCCCGTATATATTCATACATTCATCTCCCGT
										2B-0283710-T6-68B_1216	TCCCGTATATATTCATACATTCATCTCCCGT
										2B-0397780-T6-68_1280	catacatacatacatacatacatacGGCATCCTGT
										2B-0283710-T6-68B_1280	catacatacatacatacatacatacGGCATCCTGT
										2B-0397780-T6-68_1344	TTTCATATATACATACATACTGAATTTTGTA
										2B-0283710-T6-68B_1344	TTTCATATATACATACATACTGAATTTTGTA
										2B-0397780-T6-68_1408	GATGATGGTGCTCACATGTCTTCTGATGC
										2B-0283710-T6-68B_1408	GATGATGGTGCTCACATGTCTTCTGATGC
										2B-0397780-T6-68_1472	GCTCCTCATGTTCAATAACATTTATTTCAG
										2B-0283710-T6-68B_1472	GCTCCTCATGTTCAATAACATTTATTTCAG
										2B-0397780-T6-68_1536	CTTCCAATCCACCCCTGCTTCCACTCTGCT
										2B-0283710-T6-68B_1536	CTTCCAATCCACCCCTGCTTCCACTCTGCT
										2B-0397780-T6-68_1580	TCATTTTCTCTTATTTAAGCTAAGCTAAGAT
										2B-0283710-T6-68B_1580	TCATTTTCTCTTATTTAAGCTAAGCTAAGAT
										2D-0521450-T6-68_0	GTGACCCCCTGTCAAGACAGCACAACGG
										2D-0521450-T6-68_128	AAAGTGAGGGTTAGACCCATGTAACGGA
										2D-0521450-T6-68_192	AAGACGGCTTCGTCGAAACGATCCTCTTT
										2D-0521450-T6-68_256	AGCCGACAAGAATGCTCCATGTCCATCGC

2D-0521450-T6-68_320	GTGTGTGGAAACCGGCAAGCTACGACGA
2D-0521450-T6-68_384	GCCGTTGCGGGCGGCGACTGGAGACAA
2D-0521450-T6-68_448	GACAAAACCCTAGAGTGAGTGGGGGGTT
2D-0521450-T6-68_512	atgaatggaatgcattaatgaGGATATATGTCAT
2D-0521450-T6-68_576	AACGAGGGAGGCCCGGCCCAGCCCAAG
2D-0521450-T6-68_704	cgcctgccgtgccacccccttccctccccctcccc
2D-0521450-T6-68_896	CTCGACAACAGCACCGGCCGGCGGCGTA
2D-0521450-T6-68_960	ACTCGACCtagatagatagatagattagatagata
2D-0521450-T6-68_1024	GAGTATTCGTTATCAAAGGAATGAATGTC
2D-0521450-T6-68_1088	ctatctgtatctgtatctgtctgtctgtATTTACTCTG
2D-0521450-T6-68_1152	TCTGCTTACTCATACTATTTGTTTACATCAC
2D-0521450-T6-68_1216	GCTATTTTCTTTCTTGTTatacatacatagatag
2D-0521450-T6-68_1280	CTATTTCCTTGTCTGTCTCGACTCTGAAGA
2D-0521450-T6-68_1344	catacTGAATTTTGTAACAATTTCGTTTTCC
2D-0521450-T6-68_1408	TCTTCTGATGCCACAACAAAGGACTATAT
2D-0521450-T6-68_1472	TATTAATTTCAGCATTCGACCTGGTTGCCA
2D-0521450-T6-68_1536	ACCCCTGCTTCGACTCTGCTCTTTTTCTTT
2D-0521450-T6-68_1580	TCTTATTTAAGCTAAGCTAAGATTCTGAGT
U-0283710-T6-68_0	TAGGGGAAATTGATTGATATGTAGTAGAG
U-0283710-T6-68_62	CTAGTGTAATGAGATGGATGCAAACTCAG
U-0283710-T6-68_124	GGAAAAAAAAAAGAGAGAAGCAGAGTC
U-0283710-T6-68_186	TGACTATGAGGTCCAATGCTGAAATTAATA
U-0283710-T6-68_248	CACTGAAGAAATCACATATAGTCCTTTGTT
U-0283710-T6-68_310	AATTGTTACAAAATTCAGTATGTATCCTTA
U-0283710-T6-68_372	gtatgtatgtatgtatgtatgtattgtatgcatctatcta
U-0283710-T6-68_496	ATTTTGTTGAGAGTAAATACAAATACAGA
U-0283710-T6-68_558	CCTAATACTAGATGGGGATGGGATCGACA
U-0283710-T6-68_620	AGCTGAGATGGGGCCGAGAGATCTATCTA
U-0283710-T6-68_672	TGTTGGGGCGAGGAGAAGAGGGGAGCG
U-0283710-T6-68_1292	TTTGTCATGGCGTTGGAGCCTTCATCTCG
U-0283710-T6-68_1351	CTAGGCCATAATAAGAGGATCGTTTCGAT
U-0283710-T6-68_1528	GTACCGGCTTCTCTCAAGGGCGTTCCAAT
U-0283710-T6-68_434	cagaagaagaagagagcagaTGATGTAAACAA
U-0283710-T6-68_1233	GTCGGAGCTTGCTGGCTTCCACACACACA
3A-PTO1-T6-71_0	TTTGTTTACTGCATCACAGTAGAGGCATG
3A-PTO1-T6-71_64	TGTCATGCAGTACAGAGAACCTCGAGTTA
3A-PTO1-T6-71_128	TATTCTAAAAGGCTAGCTATATTATATTTGC
3A-PTO1-T6-71_320	taCATATGAAGCAGAGTTTGTTACAGATG
3A-PTO1-T6-71_384	TATTTCTTACATATTCTCGTTGTTTTCTTCG
3A-PTO1-T6-71_448	GCCTATGAGTAGATGGAAGATGCATTTATT
3A-PTO1-T6-71_512	CAACAATTAGTTTATCATACAAACTTAAAA
3A-PTO1-T6-71_576	ACTAGTCCTCATTTCCCAAGAATAAAAGT
3A-PTO1-T6-71_640	AGTTTTAACATTTCCATGTAACAGTCATTT
3A-PTO1-T6-71_704	GAAGTTGATCTTACTTCCTAGCCATAAAAT
3A-PTO1-T6-71_768	TCAATGGTGATGGATTTGTTTGAGATCAA
3A-PTO1-T6-71_832	TCCTCTGTATTTCAGTGCAGATACTCATGT
3A-PTO1-T6-71_960	AAGGTTGTACAGAGGCTGCGTCAATTAAT
3A-PTO1-T6-71_1024	AAAATGGCGATGATGCACTTTTGATTTTG
3A-PTO1-T6-71_1088	AAGTTCACAATGCAATGCAGACCTAGACG
3A-PTO1-T6-71_1152	TTAGAGCAACTCTAATCCATCCCTAATAAT
3A-PTO1-T6-71_1216	GGAGTTAAGCCCATCTAACCCATCCCAAA
3A-PTO1-T6-71_1280	CCAAAACACACCCTTCCCGAGGCTGATG
3A-PTO1-T6-71_1344	CTGTAGCCGAGGCTGGCGCCAGGTCCAT
3A-PTO1-T6-71_1536	tctacaacatcaaattagttccattagatacatcacaa
3A-PTO1-T6-71_1580	ttttCCTACTATATATTTATATGATCTTCAGCG
3B-PTO1-T6-71_0	TGAGCTGCCTGCTATTGTATCTGCATTTGG
3B-PTO1-T6-71_64	ACGATCTTCTAGTTAAAACCTGGTTTATTT
3B-PTO1-T6-71_128	AATCAGTGCAACTATAGAAGAACACAATA
3B-PTO1-T6-71_192	TGAAGGCCATTGTTGACAAACCTACTGTA
3B-PTO1-T6-71_256	CATCATAAACTCTGAATTAGTCTGCATGTC
3B-PTO1-T6-71_320	CGGTTGCTTAGTACATACATATGAAGCAG
3B-PTO1-T6-71_384	CATGTGGTTGAGAGATATTTCTTACATATT
3B-PTO1-T6-71_448	AATTTGAACATGAGTTCCTATGAGTAGAT
3B-PTO1-T6-71_512	ACTTCACAACTGATACAACAATTAGTTTAC
3B-PTO1-T6-71_576	AATTTTATTGCAGGAACAACTAGTCCTCAT
3B-PTO1-T6-71_640	ACAAACTTTGACAGAGATAGTTTTAACAT
3B-PTO1-T6-71_704	AAAAGAAAATTTGTCACTTGAAGTTGATC
3B-PTO1-T6-71_768	AGTCTCTTAACAATTTTAGGATCAATGGTG
3B-PTO1-T6-71_832	TAATTGTCCACATACCACATGTCCTCTGCA
3B-PTO1-T6-71_960	caatattgtataaaggttgtacagaggctgcgtcaatt
3B-PTO1-T6-71_1024	TTTGCACATGAGAAAATGGTGATGATGCA
3B-PTO1-T6-71_1088	ACGATGCCCAAAGACAATACGATCAACTC
3B-PTO1-T6-71_1152	AAAACTGCAATTTTGGGGAGTTGAGCCC
3B-PTO1-T6-71_1216	AGATTTTTGGGAGCTCCCAAAACACACC
3B-PTO1-T6-71_1280	GCCACCATTGGTACCTCTGTAGCCGAGG
3B-PTO1-T6-71_1344	CCATCGGTAACCACATGTGTACTATTTATA
3B-PTO1-T6-71_1536	Catactatatatatttgatgttttagatgtttatatatttt
3D-PTO1-T6-71_0	AAGTATGCCTGAGCTGCCTGCTATTGTATC
3D-PTO1-T6-71_64	AGGGGAAGCAACTTCTATGATCTTCTAGT
3D-PTO1-T6-71_128	TAGTATCACATAATAGAGATAATGCATCTC
3D-PTO1-T6-71_192	GTATTGTGTATTCTAAAAGGCTAGATATTA
3D-PTO1-T6-71_320	AAATAGAAGACGTTTTTTGATACGAAGG
3D-PTO1-T6-71_384	GTACAATTTAAGCAGAAGTTGTTAAGGAT
3D-PTO1-T6-71_448	TGGTTGAGAGATATTTCTTACACTTTTTTA
3D-PTO1-T6-71_512	TTCAACATAAGTGGTCATGAGTATATGGA
3D-PTO1-T6-71_576	CTGCAAGGTCAACATTCCTTTAGTTTATCG
3D-PTO1-T6-71_640	ACTGGTCCAGCACATTAGTAATTTCTGCTG
3D-PTO1-T6-71_704	CAAATAAGTTTATATGTGAAAAAAAACCC
3D-PTO1-T6-71_768	TGAAGTTGATCTTGCTTACCTCCTAGAGTA
3D-PTO1-T6-71_832	TTTAGAATTAGTAGTGATGAATTTTGTTTT
3D-PTO1-T6-71_896	TTGATACTTCAGGTTAGAATACTTAATGCC
3D-PTO1-T6-71_960	GACTTAACTAGTGTTGCATAGGACAAGAT
3D-PTO1-T6-71_1216	ATTAAATGGGACCCGGTTGACATCCCTAT
3D-PTO1-T6-71_1280	CCGGCAAAAAAATCCGGCTGATTGCATCT
3D-PTO1-T6-71_1344	TAACAGCGCCAATTGAGTCCGGACCGAG
3D-PTO1-T6-71_1408	CAGTTTAACCTTTTTTTTTGCGAATACGGT
3D-PTO1-T6-71_1472	CATGCATTGCCAGTTTGGCACACCTGAGG
3D-PTO1-T6-71_1536	GGATCTGACAGGCTTGGTTTACAGCGAC
3D-PTO1-T6-71_1580	TTGCACTTGCGCCTCTAGCTGGTGAGAAA
7A-TaVP1A-T6-72_0	TTAATATCTAATTTCTCCATAAGGCCTTATT
7A-TaVP1A-T6-72_64	AGGGCCCAGAATACCAAGGAATCACTATC
7A-TaVP1A-T6-72_192	CAAGGGTGCTCGCGATGCTACTGCTGTCG
7A-TaVP1A-T6-72_256	AATAGGGAGGGGGAAGGGGATTAGTGA
7A-TaVP1A-T6-72_320	AATCCCCTCCAAAGGAGGCATAAAGaaga
7A-TaVP1A-T6-72_384	taaataaaataataacataaGAAGGTAGGGAA
7A-TaVP1A-T6-72_448	CAAAAAGGGGCGCTAGTTAACCATCTTTA
7A-TaVP1A-T6-72_512	CACACTTTTTCTTTGCAGGAAAACTTGCA
7A-TaVP1A-T6-72_576	TGGAATTCCACCGAGCCTCTAACCCAAAA
7A-TaVP1A-T6-72_640	GGACGGAGGCTTTAGCCATTTTTGGAATC
7A-TaVP1A-T6-72_704	AATTCACCCGTCCAAATCTAGAAGACCAG
7A-TaVP1A-T6-72_768	AAAACCACGAGGTGAAAAATGCAATACA
7A-TaVP1A-T6-72_832	TTTTGCTACGAGTATGCACACCTCTGGTTA
7A-TaVP1A-T6-72_896	TCAACTTTTTATCTTCATTTATATGAAAAAG
7A-TaVP1A-T6-72_960	GAAGTAGCTTATCGGGCCTAACCAAAAG
7A-TaVP1A-T6-72_1024	TGGCAATAAAAACTTCTTACCAGTACCATC
7A-TaVP1A-T6-72_1216	TACCACCAGAAATGCATTGTTAAGCCGGG
7A-TaVP1A-T6-72_1280	CCGTTCGGGTTAGCCGTGGTTTCGCTCCG
7A-TaVP1A-T6-72_1344	CAGGCGCAAAAAACAAAAAAGAGAATCG
7A-TaVP1A-T6-72_1580	GGCATCCGGAGCACGGAGACACACGCAG
7B-TaVP1B-T6-72_0	AAGGAATCACCGCCGGCCCCATGCCAAC
7B-TaVP1B-T6-72_64	ACCCAACCGACGTGTTGTAGAAAACTCTG
7B-TaVP1B-T6-72_128	TCGTGATGCTACTACTGTCGTATGTGGTTT
7B-TaVP1B-T6-72_192	GAGGGGGGAGGGTATTAGTTGGGAGTT
7B-TaVP1B-T6-72_256	CTCCAAACGAGGCATaaagaagaaacaaaa
7B-TaVP1B-T6-72_320	ataaaataataacataaaaaggttaaaaaaaGGA
7B-TaVP1B-T6-72_384	GGGGTGCTAGCTAAGCATCCTTATTTCACA
7B-TaVP1B-T6-72_448	TTACATGTTGAAGCTCAATTGAAAAATAG
7B-TaVP1B-T6-72_512	AATTATGGAGACGTCTATATACAGGGAGA
7B-TaVP1B-T6-72_576	GCCTTACCTGCACCTCCCCCCTGCCGAAA
7B-TaVP1B-T6-72_640	CGCTCCTCCCCTAATAGGTCATTTGAAAAT
7B-TaVP1B-T6-72_704	TGTACATCCACGTGTTGTATCAACTCTGTT
7B-TaVP1B-T6-72_768	CTATGATCAAGTACTCCTCTAGCCCATTTC
7B-TaVP1B-T6-72_832	ACTCCGCTAGCAACAAGTAATGGATCAGT
7B-TaVP1B-T6-72_896	CATCATCATCGGCATCTTAACCATCTCATC
7B-TaVP1B-T6-72_960	CCCAGTGACAAGGCGTCGTGCTGTAACTG
7B-TaVP1B-T6-72_1152	GGAGTAGATAGATGCCCCCCGGTCGACC
7B-TaVP1B-T6-72_1216	GTGCCATCCGTGGTTACCAGAAATGCATT
7B-TaVP1B-T6-72_1280	AGAAGTGCTAATCCGTTCGTGTTGGCCGT
7B-TaVP1B-T6-72_1472	CAGAAAACTTACAACGGGACAGAGGGA
7D-TaVP1D-T6-72_0	ATGGGAATATCCCCATAGGGCCCAAATAC
7D-TaVP1D-T6-72_64	CCCCCACAATCCCCACCAACCCCTACAAA
7D-TaVP1D-T6-72_128	ACTCGTGGAACGGGATAAGACAAGGGCG
7D-TaVP1D-T6-72_192	AGCAAAGGGATTTACGAGGAGGAGAGG
7D-TaVP1D-T6-72_256	TGGGTGGAGGGGAGGGATTACCAAACC
7D-TaVP1D-T6-72_320	aaatgtagagaagaagataaaaaacttaaataaaat
7D-TaVP1D-T6-72_384	AAGCAACCCGTAATTGGCCGTACCAAAAG
7D-TaVP1D-T6-72_448	AGCATGGAGAAACCTTTAAACTTGCACA
7D-TaVP1D-T6-72_512	GGAATTCTACCGGGCCTCTAACACAAAAG

										7D-TaVP1D-T6-72_576	GAGGCTTTAGCCATTTTTGGAATTCCTCG
										7D-TaVP1D-T6-72_640	CCCATCCAAATCCGGAAGCCCGGCTTACA
										7D-TaVP1D-T6-72_704	GAGGTGAAAAATGTAATATAACTGCACTT
										7D-TaVP1D-T6-72_896	tccggacggagggagtaCTTCTGTAGCCCATTT
										7D-TaVP1D-T6-72_960	ACTCCGCCAGCAAGGAGTGGATCAGTTG
										7D-TaVP1D-T6-72_1024	TCTTAACGAGCGAGCCTAGCCTCATCAAT
										7D-TaVP1D-T6-72_1088	GTGACTAAGTGCCGTGCTGTCAAGTGCGT
										7D-TaVP1D-T6-72_1216	CCTGCAAGTAGCCGTGGCGTGCCATCCGT
										7D-TaVP1D-T6-72_1280	GTAAGATAACCATGCCCAATTTGCCGACT
										7D-TaVP1D-T6-72_1344	TCTTTTCACTGTAATCACAGATTCTCTCTCT
										7D-TaVP1D-T6-72_1408	CTCGCAAATACCACCGGATAAAAAGATAC
										7D-TaVP1D-T6-72_1580	CGCGGCCGCTATATAAAGCGGCAGGCAT
										6A-TaNA-T6-73_256	AGACTTTTATAGGCTGTTGGGTACAAAGG
										6A-TaNA-T6-73_320	TGCCTGCGTTTTCACGGGAAAAAAAAAC
										6A-TaNA-T6-73_384	CAGCACAGTGAAAAGCTGCAGCGCCATG
										6A-TaNA-T6-73_448	CATTCGTAAATACTTGTCATTAAGATGAAT
										6A-TaNA-T6-73_512	AAGAAAAAGTACATGTTAAGGACCCACAT
										6A-TaNA-T6-73_576	AATCATAGAGAGGGCCATGTGAATTGGTT
										6A-TaNA-T6-73_640	TCTGTGCATTTGTAAAACTTTTCTCTTTCC
										6A-TaNA-T6-73_704	TGTTTTAGTCAAATTCTATATTTTTCTTCCC
										6A-TaNA-T6-73_768	TGTACTGTATAATCTTAGAGACTCATGTCG
										6A-TaNA-T6-73_832	ATATCCATAAAAAATTGTGTTCGTCCAGTTT
										6D-TaNA-T6-73A_64	TTGTGCGTGTAAGAGATGTGCGTTCTGCC
										6D-TaNA-T6-73B_256	CTCTCGAAATCATTTTAATAGTTGTATATCT
										6D-TaNA-T6-73B_320	CTATTAGAGGTATGCAAACAACTTGGGCT
										6D-TaNA-T6-73B_384	AGAACCCTGTATTTTGAGGCAGACTTAGA
										6D-TaNA-T6-73A_448	AGTATAATAAAAACTGTTTTCTCAACTCAT
										6D-TaNA-T6-73B_640	GCGCGCTCTTTCGGCGCCGACCTCGCCTC
										6D-TaNA-T6-73B_704	ttccacGGAAAGTTATTATCTTCTTAAGTTAT
										6D-TaNA-T6-73B_768	TCCATTTAGAAACAATTATACAATGCATAT
										6D-TaNA-T6-73B_896	AATGAGTAAACAAACGCACTAAAGCGCTT
										6D-TaNA-T6-73B_960	TTTATAATTCGGAACTAAGGAAGTATTTGT
										6D-TaNA-T6-73A_1024	ACTGTATCACAATCCCCTATTACTGCCATG
										6D-TaNA-T6-73B_1024	AAGAAGTTGATGATAGCTTCGCACACTAT
										6D-TaNA-T6-73A_1152	AGAAATAGAGCAACCAATTATCGGCGACA
										6D-TaNA-T6-73A_1344	TAATAGGAACACATACGTAATTTGGTTATA
										6D-TaNA-T6-73A_1408	CAGAATAAAAGAATAGGTTTCTTGCTATC
										6D-TaNA-T6-73A_1472	CTTGTAAATTAAGCTAAGGGGTAGATTCCT
										6D-TaNA-T6-73B_1472	atttattttgagacggatggagtaTTGCACTTCAA
										6D-TaNA-T6-73B_1536	ACTCCAAGTACAAATACAAGTAGTACTTA

Trait&gene no.	Gene	Homoeologues	IWGSC gene ID (refseq1.1)	WGIN Promotome ID	Orientation	ATG	Target Sequence	MYbaits	MYbaits coverage (%)	MYbaits ID	MYbaits sequence
T7-1	FT1 = VRN3	ABD	TraesCS7A02G115400	7A-1825000-T7-1	forward	71,669,854	TTCACATCACCTCAATTATTGGTAGTCGTACGACCTAAATTGTGTGGC	18	80.9	7A-1825000-T7-1_0	TTCACATCACCTCAATTATTGG
T7-1	FT1 = VRN3		TraesCS7B02G013100	7B-1958810-T7-1	forward	9,702,478	TTCACATCACCTCAATTATTGATAGTTGTACGACCTAAATTGTGTGGC	16	73.4	7A-1825000-T7-1_64	GTTTTTGTGCATTAATATGTCC
T7-1	FT1 = VRN3		TraesCS7D02G111600	7D-2022890-T7-1	reverse	68,417,532	TCCCCCTTCCTTCTTAGTATAGATCGACCGGCCTGCTAGCTAGGATAC	16	72.2	7A-1825000-T7-1_128	CCTGGTGAATCAAGTCATGCA
T7-10	FT11	ABD	TraesCS4A02G392900LC	4A-0943490-T7-10	reverse	550,957,583	GATTGGGAAGAAAGGGACACCACTGGAGCTTGGTGAGCTAGGATA	10	54.1	7A-1825000-T7-1_192	TATGGATAGTATATGCGTTGTA
T7-10	FT11		TraesCS4B02G073800	4B-1108830-T7-10	forward	69,163,023	CTAGAGCATCATTTGTAATAGACTCGGAGGATTAACAAGATAAAAGT	8	49.9	7A-1825000-T7-1_256	TAAATGTTGGTGTCGTCGTGT
T7-10	FT11		TraesCS4D02G072300	4D-1182900-T7-10	forward	47438815	CTAGACTAGAGCATCATTTGTAATAGACTCGGAGGATTAACAAGATA	10	54.1	7A-1825000-T7-1_320	TAACTTATAGCTTCATTGTTCT
T7-11	WCO1/Hd1	ABD	TraesCS7A02G211300	7A-1814120-T7-11	forward	174,203,443	CCTTTCGAACAGATAGAAACCAATTATTCTGGGAAGATATCTATCTAT	10	57.4	7A-1825000-T7-1_384	TTTAAACACGTGTTTATTAATG
T7-11	WCO1/Hd1		TraesCS7B02G118300	7B-1952050-T7-11	forward	137,793,989	AGACTTACATGCAAGTTTAACTTTTGTTCAGAAAATTTGTGTCAAGA	17	80.5	7A-1825000-T7-1_448	TGCAAACATTTTTGTTGGCAG
T7-11	WCO1/Hd1		TraesCS7D02G212900	7D-2057910-T7-11	reverse	171,308,881	CCTGAAACTAGTTTTAGCTGTTCCTGTGCTTGGATGGTTCGGGTTCA	17	82.6	7A-1825000-T7-1_512	CAAAAAAAAAAGGCAGAAAT
T7-12	CO-2	ABD	TraesCS6A02G289400	6A-1519320-T7-12	reverse	521,453,784	ATAAATCTTGCAACTTGTAGGTGGAGGGGTCTTACCGAAAACTCTG	19	86.8	7A-1825000-T7-1_576	TGCTTTCGCTTGCCATCCCCT
T7-12	CO-2		TraesCS6B02G319500	6B-1608580-T7-12	reverse	567,399,418	ATAAATCTTGCAACTTGTAGGCGGAGAGGTCTTACCGAAGGTTGAG	20	90.6	7A-1825000-T7-1_640	TTCAATATTATTTTCGCAGCTC
T7-12	CO-2		TraesCS6D02G269500	6D-1702700-T7-12	forward	379,572,213	ACGTAGTCCATAGTCATGCCTTAGAAAAACGGTTACTGTTTTCTGTTA	18	84	7A-1825000-T7-1_704	GTAAAAGATGCGCTCAAAGG
T7-13	CO-3	ABD	TraesCS5A02G166100	5A-1245850-T7-13	reverse	355,072,685	CGCCGCCCGCGAGGTCGATCGGTTCAGTTCACCCAGCGCCCAGGC	3	17.9	7A-1825000-T7-1_768	AAAAATCAGAGAGTTTATTTC
T7-13	CO-3		TraesCS5B02G163500	5B-1333300-T7-13	reverse	302,754,855	CGCCGCGCGCGAGGTCGATTGGTCCAGTTCACCCACCGCTCAGGCG	14	65.9	7A-1825000-T7-1_896	CAGCGTCCCGTGTAAGCTAAC
T7-13	CO-3		TraesCS5D02G170700	5D-1424230-T7-13	reverse	267,763,959	CGCCGCGCGCGAGGTCGATCGGTCCAGTTCACCCACCGCTCAGGCG	8	40	7A-1825000-T7-1_1216	ccgcggcgtgaccgccggtcggccCA
T7-14	CO-4	ABD	TraesCS2A02G353900	2A-0317750-T7-14	forward	594,580,295	AGAGCATAAATTATGGCCACTGGAGGAGGAGTACGTCGGAGACAGT	9	43.8	7A-1825000-T7-1_1280	GCAGGCAGCCAGGCACGGCC
T7-14	CO-4		TraesCS2B02G372000	2B-0432900-T7-14	forward	530,306,534	TCCCCCCCCCCCGTGCCCCGACGTCACACTACCACATGACTGACATT	4	21.6	7A-1825000-T7-1_1344	ATTACGGCAGACAGATGCATC
T7-14	CO-4		TraesCS2D02G351900	2D-0531670-T7-14	forward	450,234,865	AGGAGGTCGATGTTGTGATGAAGAAGGCTACGCCGGCCGAGCTGA	2	10.8	7A-1825000-T7-1_1580	GCCACCGGCCGTGGGGCAAC
T7-15	CO-5	ABD	TraesCS6A02G218900	6A-1518320-T7-15	forward	405,166,850	CGGAGATGTCTACGTTCCGGAGCTGGATGTGAAGCAGGAGGTGAA	10	50.8	7B-1958810-T7-1_0	TTCACATCACCTCAATTATTGA
T7-15	CO-5		TraesCS6B02G248400	6B-1600890-T7-15	forward	445,851,986	GCCCTGCCAAGCAAATGGACAGTTCTTCCACTCCCAGTGCATACAAT	2	14.1	7B-1958810-T7-1_64	TGTTTTGTGCATTAATCTGTCC
T7-15	CO-5		TraesCS6D02G301700LC	6D-1719340-T7-15	forward	284,848,003	GAAGAGGCGTGGATTTTACAATTCACCCATGTGGAGCGCGGGAAGA	6	29.2	7B-1958810-T7-1_128	CCCTGTTGATCAAGTCATGCAT
T7-16	CO-6	ABD	TraesCS7A02G497200	7A-1758420-T7-16	forward	686,936,985	TTATAATACCATGGTAAATTTTGACTCACGCAAAAAAAGTAGTTGAAA	13	58.8	7B-1958810-T7-1_192	CATGGATAGTATACGTGTTGTA
T7-16	CO-6		TraesCS7B02G400600	7B-1898970-T7-16	reverse	667,071,548	AACTAGCTAGCACCCCTGCACGTCCTCTTAGCTCCGACAGGCAATGG	15	72.9	7B-1958810-T7-1_256	TAAATGTTGGTGTCGTCGTGT
T7-16	CO-6		TraesCS7D02G484400	7D-1985690-T7-16	reverse	594,730,835	AACTAACTAGCACCCCTGCACGTCCTCGATCGGTCCTCCTAGCTCCGA	18	76.5	7B-1958810-T7-1_320	GTAACTCATAGCTTCATTTTTC
T7-17	CO-7	ABD	TraesCS6A02G150900	6A-1541240-T7-17	reverse	134,949,373	CACACCTCACACCACACTACCACAGCACCACCAGCAGCAACACTTG	9	55.8	7B-1958810-T7-1_384	TTTTAAACATGTGTTTATTAAT
T7-17	CO-7		TraesCS6B02G179000	6B-1655930-T7-17	reverse	198,986,228	CGCACCTCACACCACAGCACCACCAGCAGCAACACTTGGAAGCAAA	9	52.5	7B-1958810-T7-1_448	ATGCAAATGTTTTTGCTGGCA
T7-17	CO-7		TraesCS6D02G140900	6D-1748960-T7-17	reverse	110,444,621	CGCACCTCACACCACAGCACCCACCAGCAGCAACACTTGGAAGCGA	10	60.7	7B-1958810-T7-1_576	TGCTTTCGCTTGCCATCCCATC
T7-18	CO-8	ABD	TraesCS7A02G178400LC	7A-1833330-T7-18	forward	85,159,675	TAGTACTTTGATGAATTGTTGTCATTTTCCTTCCAAGACAAAAAGAAA	18	83.1	7B-1958810-T7-1_640	TTCAATATGATTTTTGCAGCTC
T7-18	CO-8		TraesCS7B02G032800	7B-1938900-T7-18	reverse	32,664,641	GCCCGTGGCTTCTGCTGGCTCTGCCACTGCGCTGAGGAGGATTTCT	23	95.3	7B-1958810-T7-1_704	TAGTGAAAAGATGCGCTCAAA
T7-18	CO-8		TraesCS7D02G131700	7D-2015990-T7-18	reverse	83,473,993	GCTGAGGAGGATTTCTTGGGTTGATCGGTCAACGGAGCAACCACC	7	36.2	7B-1958810-T7-1_768	TCAAAAACAATCAGAGGGTTT
T7-19	CO9	ABD	TraesCS1A02G220300	1A-0012040-T7-19	forward	389,129,419	TTAAAAGCATTCCTGGCTAGAATATGTGCCACATAGTTCGCCTCCCTG	19	84.7	7B-1958810-T7-1_832	GCCGGGACCGGCACTGTATAT
T7-19	CO9		TraesCS1B02G233600	1B-0099330-T7-19	forward	419,402,399	CCTTCTCCTGATCTACAGTCGGACTTGGCAAATCCGATCCCTCAAAC	1	7.1	7B-1958810-T7-1_896	ACGGCCCGCGTCCCGTGTAAG
T7-19	CO9		TraesCS1D02G221800	1D-0204060-T7-19	forward	309,584,165	CTTTCAACTATCTCCAAATTCTTCCTCGGAATTCTCCGAAACGAATGA	3	21.2	7B-1958810-T7-1_1280	CACGGACAGAAACCACCATTA
T7-2	FT2	ABD	TraesCS3A02G143100	3A-0686640-T7-2	forward	124172881	CCACTCAAAATTTAAATTGCGATAAAAGATTGATTGTTTGTGTTGTAA	12	99.1	7B-1958810-T7-1_1580	ATCATCACCACTTCCTCAATTC
T7-2	FT2		TraesCS3B02G162000	3B-0786660-T7-2	reverse	158,719,064	CTTCTTCCTCCTGTCACCACTGGCGCCCTTCTCCGCCGGCCGCCGGC	19	86.8	7D-2022890-T7-1_0	TCCCCCTTCCTTCTTAGTATAG
T7-2	FT2		TraesCS3D02G144500	3D-0917880-T7-2	reverse	107,681,960	CTTCTTCCTCCTGTCACCGCTGGTCCTTCTCCGCCGGCCGCCGGCAC	18	83.1	7D-2022890-T7-1_256	TCGTCGCCTCCCTCTGTAGAAT
T7-20	GID1	ABD	TraesCS1A02G255100	1A-0037970-T7-20	forward	447,005,332	GGTCGCTGCTGGAGCCACCGGAGCCGCCCGCCCTGCGCCCTTCGC	5	32.2	7D-2022890-T7-1_320	CACAGCCACAGGCAGCAGCG
T7-20	GID1		TraesCS1B02G265900	1B-0129000-T7-20	forward	466,936,456	TCATCTGCGGCAAGCTCCGGGGTTTTTCGCGGGTGATCTTCGAGGT	7	39.5	7D-2022890-T7-1_704	CATTGGAGGGCAGGGTTATT
T7-20	GID1		TraesCS1D02G254500	1D-0205760-T7-20	forward	346,662,682	TCACACCACAACGGCAGCCATTGGCGTTAACTAATTGCGTCCGTCCA	15	76.2	7D-2022890-T7-1_896	GACGATAATGCTCCACCTGTC
T7-21	GID2	ABD	TraesCS3A02G056000	3A-0681860-T7-21	reverse	32,253,037	GGCGGGGCGCCTGTACACACGACTGTCAGGATCACGATGGATCTGA	5	25.4	7D-2022890-T7-1_960	TTCTTTTTAATATACCCTCCACT
T7-21	GID2		TraesCS3B02G068800	3B-0725290-T7-21	reverse	40866842	GGCGGGGCACCTGTACACACGACTGTCAGGATCCCGATGGATCTGA	17	89.6	7D-2022890-T7-1_1024	TATATCTTTCCCTTCGGATTCTT
T7-21	GID2		TraesCS3D02G056100	3D-0898770-T7-21	reverse	23,174,585	GGCGGGGCACCTGTACACACGACTGTCAGGATCCCGATGGATCTGA	16	74.1	7D-2022890-T7-1_1088	AAATTAATAATACAAATGCTAT
T7-22	GA2ox3	ABD	TraesCS3A02G294000	3A-0644720-T7-22	reverse	526865600	GGCCAATATATGGTATGTGCGATCGAGGGAGGTGAGGAGCGGAGCA	13	67.5	7D-2022890-T7-1_1152	GTCAGTTTTAGTTGCGATGCA
T7-22	GA2ox3		TraesCS3B02G328700	3B-0772640-T7-22	reverse	530611426	GGCTGTGCTGAGCTACCAAATATGGTATGTGCGATCGAGGGAGGTG	11	57.9	7D-2022890-T7-1_1216	CTTCTAGAAACTGCAACGCAT
T7-22	GA2ox3		TraesCS3D02G293800	3D-0870310-T7-22	reverse	404,442,213	GGCTGGGCTGCCAAATATGGTACGTATGCGCGATCGAGAGAGGCGA	15	71.8	7D-2022890-T7-1_1280	TCACGACGAACGTTCGCCAGC
T7-23	GA2ox4	ABD	TraesCS1A02G334400	1A-0006730-T7-23	reverse	521,878,514	GGCGCGCGCGTATATATAGTGGTGGCGGGGGGGAGGAGGTCGCCG	2	10.8	7D-2022890-T7-1_1344	TGATATAGGATGTTCTCAGCTT
T7-23	GA2ox4		TraesCS1B02G347600	1B-0138950-T7-23	reverse	576,688,562	TGCCAAGAAACAGAGCAGAGCTGGGCCAAGCAAATAAGCAGAGCA	3	17.9	7D-2022890-T7-1_1408	GTACAAAAAGAATGTTTTTGA
T7-23	GA2ox4		TraesCS1D02G336900	1D-0204240-T7-23	reverse	426,870,633	TGCCAAGAAACAGAGCAGAGCTGGGCCAAGCAAATAAGCAGAGCA	4	17.9	7D-2022890-T7-1_1472	AATATGAATTAGATATTTTTGC
T7-24	GA2ox5	ABD	TraesCS3A02G133400	3A-0701660-T7-24	forward	110,332,677	GGCCCACCAAACCGGCTGCCACTGAAGGTAACAAGGCCCGCGGGG	18	77.6	7D-2022890-T7-1_1536	TTAACATTGGAAAATTAATGTG
T7-24	GA2ox5		TraesCS3B02G166100	3B-0782440-T7-24	reverse	166503519	GGTCGGTCGGCTTCTTCTTCCTTGGTCTCGAGGAAACAGAGGAGAA	21	91.1	7D-2022890-T7-1_1580	TCACCAGGGAAAAAGGCATG
T7-24	GA2ox5		TraesCS3D02G149600	3D-0928740-T7-24	forward	114,335,660	GCGGCAGCGGCGGCAGGCAGCAGCAGCCGTCGCGGCGGGGAGCA	13	71.8	3A-0686640-T7-2_0	CCACTCAAAATTTAAATTGCGA
T7-25	GA3ox1	ABD	TraesCS2A02G540400	2A-0318130-T7-25	reverse	752,337,915	GGCGAGCGATGCGCGCGGAACCACAATAATCACTAGCTAGATAGAG	14	68	3A-0686640-T7-2_124	AGCGACAACTAATATGGATCG
T7-25	GA3ox1		TraesCS2B02G570900	2B-0442410-T7-25	reverse	761,329,103	GGCGAGCGATGCGGAACCACAACAATAATCACTAGAGTAGCGAGTA	6	25.9	3A-0686640-T7-2_186	CAAATCCTTTCCTCCCACCTAA
T7-25	GA3ox1		TraesCS2D02G542100	2D-0552010-T7-25	reverse	620,686,402	GGCGAGCGATGCGCGTGGAACCACAAGAATCACTAGCTAGATCGA	7	32.9	3A-0686640-T7-2_248	TCCCAGTAAAACAGGTAGCG
T7-26	GA3ox2	ABD	TraesCS3A02G122600	3A-0692510-T7-26	forward	97,973,005	TAGGTTTAACATATTGGACAAAACCTTTATAAATAACACCCCCACACG	16	80	3A-0686640-T7-2_310	AGGGGACGCATAGAAAACAT
T7-26	GA3ox2		TraesCS3B02G141800	3B-0715770-T7-26	forward	128948333	TCGGAATGATTTAAAGCAATAGTCTATAATTTGACATAAAAGCCATAA	14	65.9	3A-0686640-T7-2_434	CATTTGACCCACCCACTCCCC
T7-26	GA3ox2		TraesCS3D02G124500	3D-0902420-T7-26	forward	82411917	TAAAAGCAATAATAATAAGGCATACCAACGAACAATCATGTCTTAAAA	16	76.7	3A-0686640-T7-2_496	GGACTCCGGACAGAGCAATG
T7-27	GA20ox1	A, BD	TraesCS4A02G319100	4A-0938430-T7-27	reverse	608044864	GATCGACTGGACCAAGAACCGGAGTATGCTAGGCCAGAGAAGAAA	13	55.1	3A-0686640-T7-2_558	CGTCGATCGACAGCAACCATC
T7-27	GA20ox1		TraesCS5B02G560300	5B-1314550-T7-27	forward	706,852,578	ACAGAACCTGCATATGTTTTACTGCACAGAACCTGCATATTTTTATTA	20	88.5	3A-0686640-T7-2_620	CTCGGCAGCTGCTCGTGCTCC
T7-27	GA20ox1		TraesCS5D02G566200	5D-1414740-T7-27	forward	564,586,950	AATAAGAGATTCAACAGGTGTGCTGTCCAAATGAACAGGGATGACA	19	80.6	3A-0686640-T7-2_682	cgagcgagcgagcgagctagTCCAG
T7-28	GA20ox3	ABD	TraesCS3A02G399800	3A-0639020-T7-28	forward	646684230	CTTCGCTGGCTCCCTCTTCTCATGCACTGTGGATAGCCCGCCGAGAC	16	76.7	3A-0686640-T7-2_744	GGCCGTCGTGGTGCAATAGCA
T7-28	GA20ox3		TraesCS3B02G432800	3B-0741640-T7-28	forward	671844055	TTTGTTCGAATACAGGAACATTTTTATAATGCGTAAATTTTTTATTTGA	14	75.8	3A-0686640-T7-2_801	TAGAGCTGCTAGCACGGGCG
T7-28	GA20ox3		TraesCS3D02G394000	3D-0843900-T7-28	forward	509,076,947	AATCTTCAAGCGCATTGTATCCATCTGGATCTTGTGTAGATCGACGAC	1	7.1	3B-0786660-T7-2_64	AATGAAGGCGGCCGGCTCTAT
T7-29	TaCPS	ABD	TraesCS7A02G552700	7A-1758330-T7-29	reverse	725,556,827	CCTGGCCGCGGCAATGCACGTTCCTCAATCGGCAATGAACACGACT	11	57.9	3B-0786660-T7-2_128	TGTTGCTGCTGCACCACGACG
T7-29	TaCPS		TraesCS7B02G476400	7B-1885950-T7-29	forward	732,567,163	ACATTTGAGATTATGCAGGATTATACACGAAAGATCTTTTTTTGGCTC	10	46.4	3B-0786660-T7-2_192	TCTCTGGAATAGCTCGCTCGC
T7-29	TaCPS		TraesCS7D02G539200	7D-1962550-T7-29	reverse	629,534,978	CCTCACTTGGTTGACCAGTGGCTGCCCAAACTCCGCCCCAGGAATC	17	76	3B-0786660-T7-2_256	CGAGGTGTCCTGCGTGCGTCA
T7-3	FT3 = Ppd-2	ABD	TraesCS1A02G338600	1A-0043540-T7-3	reverse	528,066,476	ATACTGGAGGACTACCAGTTAACAGCCTTAGCCTTTGTCTGTTATTGT	25	99.1	3B-0786660-T7-2_448	GAGAAGTGCCCGTGCTGGCG
T7-3	FT3 = Ppd-2		TraesCS1B02G351100	1B-0103850-T7-3	reverse	581,414,952	ATACTGGAGGAGGACTACCAATTAATAGCCTTGTCCTTGTCTGCTATT	20	99.2	3B-0786660-T7-2_512	TCTATATCACCTCGATGTTTTCT
T7-3	FT3 = Ppd-2		TraesCS1D02G340800	1D-0201670-T7-3	reverse	430,469,335	ATACTAGAGGAGGACTACCAATTAACAGCCTTAGCCTTGTCTGTTATT	24	99.1	3B-0786660-T7-2_576	GAGTTAATAAAGATGCTTTTTA
T7-30	TaKS	ABD	TraesCS2A02G425400	2A-0312750-T7-30	reverse	679,267,800	GGGTTGGGAGGGATCTACGATCGCCCAGGGCCGCGGCCGCCGTCG	15	65.2	3B-0786660-T7-2_640	ACACAATTGTGCACTCATCCCT
T7-30	TaKS		TraesCS2B02G445700	2B-0416140-T7-30	reverse	638,759,820	GGGTTGGGAGGGATCTACGATCGCCCAGGGCCGCGGCCGCCGTCG	6	32.5	3B-0786660-T7-2_704	AGGTATCGATCCCACAAGAAA
T7-30	TaKS		TraesCS2D02G423300	2D-0518570-T7-30	reverse	536,511,156	GGGTTGGGAGGGATCTACGATCGCGCAGGGCCGCGGCCGCCGTCG	1	7.1	3B-0786660-T7-2_768	TGATTAGCTAGCACACGGTTG
T7-31	TaKO	ABD	TraesCS7A02G362300	7A-1763880-T7-31	reverse	536,777,086	GGGGGTCGTTCGGGCGTGGGGTGGGAGAGGAGAAAAGGTGCCTG	9	43.8	3B-0786660-T7-2_896	GAGGAACGAATTTGAGTGGA
T7-31	TaKO		TraesCS7B02G265800	7B-1873370-T7-31	reverse	488,374,797	ATGGGCGCGGAGCGGAAAAGGGATGCCGGCCGTCGCTCTATTTGT	16	65.6	3B-0786660-T7-2_960	TCTTTTACAAAAGAATAGAAAT
T7-31	TaKO		TraesCS7D02G360700	7D-2113450-T7-31	forward	463,698,654	ATTTGTTCTGACTTCGTGTGTTGTTGTGTTGCGGATAGGACACAACC	20	85.2	3B-0786660-T7-2_1152	TCATTCTTGACACGAGCTTTC
T7-32	TaKAO	AD	TraesCS7A02G029600	7A-1851630-T7-32	forward	12,095,995	AAGAAGTTAAGCAAAAAAAAAACAAGAACAAAACGTTTCATGGAG	14	61.9	3B-0786660-T7-2_1216	GCTCGTTTGCCCGCGGGTTCT
T7-32	TaKAO	no B	TraesCS7D02G026000	7D-2055390-T7-32	forward	12,458,865	AGCGTCAGTACAAAGACCACACGAGGCGCTAGGCCGCTAGCAAAG	13	68.7	3B-0786660-T7-2_1280	CCCCCTTCACTCACTTGATGCT
T7-33 & T9-23	Rht1	ABD	TraesCS4A02G271000	4A-0978250-T7-33	reverse	582,479,578	GATCTCGCCTCACCGCGAGCGAGCTGCCTCGCTTCTCTACCTATCTC	19	76.9	3B-0786660-T7-2_1344	GATGTTGTGTGTTTCGAATCTT
T7-33 & T9-23	Rht1		TraesCS4B02G043100	4B-1095100-T7-33	forward	30861382	CTCCGCGATAAGGTTTGCTAGGCCTCGTGAACCTACCAGGATCGGCT	16	70.1	3B-0786660-T7-2_1472	TGAAGACGTCACGGGGCTGA
T7-33 & T9-23	Rht1		TraesCS4D02G040400	4D-1165870-T7-33	forward	18,781,062	TTTTAATGACATGTGTAGCATAAGTACATGATCTAAAAGACTTGAGTC	14	59	3B-0786660-T7-2_1536	CCTTCACCTCTCCATTCCTACC
T7-34	Bri1	ABD	TraesCS3A02G245000	3A-0616220-T7-34	forward	458,681,484	TTATCCCAATCTTTTTGCGGTCATGCAGCACATCCTCCAAGCTGCCAA	2	10.8	3B-0786660-T7-2_1580	ACCCCCCCTCAGTGTGAAAAA
T7-34	Bri1		TraesCS3B02G275000	3B-0824910-T7-34	forward	443,806,079	CCGCCAAGTCCGCTGGCGGTGGCGGTGGCGGGCAGGGGTTCGCG	1	7.1	3D-0917880-T7-2_128	GTTGTTGCTGCTGCACCTGCA
T7-34	Bri1		TraesCS3D02G246500	3D-0867790-T7-34	reverse	344339522	CCCCTCCACCCTCTGCCAAGATCCCAACTCCAGGCTCCGCGTGCTCT	1	7.1	3D-0917880-T7-2_192	ACAGATCTCTGGGCTAGCTAG
T7-35	TaGI	ABD	TraesCS3A02G116300	3A-0706870-T7-35	forward	84,190,136	GATGTTTTCTTTATTCCATCGAGATTTTATGTCTTTGTTACTTCTTATTT	13	62.1	3D-0917880-T7-2_256	GATCACGAGGTGTCCTGCGTG
T7-35	TaGI		TraesCS3B02G135400	3B-0833960-T7-35	forward	117,927,557	TCCATCAAAAGAAAAGCGGTTGGTAGATGAAGTATTAGGTAACATAT	14	71.3	3D-0917880-T7-2_448	GAGGTGCCCGTGCTGGCGGT
T7-35	TaGI		TraesCS3D02G118200	3D-0931440-T7-35	forward	71,969,562	TGGAGAATTGAAGCCTCCAACAACTGGTTAGTAACTAGATGTTTTCT	12	58.4	3D-0917880-T7-2_512	TATATCACCTCGATGTTTTCTA
T7-36	Ppd-1	AD, U	TraesCS2A02G081900							3D-0917880-T7-2_576	GTTAATAAAAATACTTTTTATA
T7-36	Ppd-1		TraesCS2D02G079600	2D-0605730-T7-36	reverse	33,955,668	GTTGGGGTCCTTTGGTGGTTGACGGTGAGGGAGGAGGAGGCGCG	5	22.4	3D-0917880-T7-2_640	ATAATTGTGCACTCATCCCTAA
T7-36	Ppd-1		TraesCSU02G196100	U-0489460-T7-36	reverse	293,692,277	TTGGGGTCGTTTGGTGGTTGACGGCGAGGGAGGAGGCGCGCATAA	2	10.8	3D-0917880-T7-2_704	GTATCGAGCCCACAAGGAAC
T7-37	Vrn1	AABD	TraesCS5A02G286800	5A-1202840-T7-37	forward	494,863,148	GTCGGCCATTCATCAAGTCGGTGTTGAAGCAAGTATCGTGCTCATTC	11	54.6	3D-0917880-T7-2_768	ATTAGCTAGCACATGGTTGAG
T7-37	Vrn1		TraesCS5A02G391700	5A-1486810-T7-37	reverse	587,423,240	CTCCGCTCGAGAACCGGGCCAACCCTACGCCCCTACCCTCCAACACC	8	43.3	3D-0917880-T7-2_896	AACGAATTTGAGTGGAGTCG
T7-37	Vrn1		TraesCS5B02G396600	5B-1325650-T7-37	reverse	573,815,903	CTCCGCTGGAGAACGGGGCCAACCCTACACCCCAAGCCTCCAACAC	8	43.3	3D-0917880-T7-2_960	TTACAAAAGAACAGAAATAAT
T7-37	Vrn1		TraesCS5D02G401500	5D-1432830-T7-37	reverse	467,184,278	CTCCGCTCGAGAACCGGGCCAACCCTACGCCCCTACCCTCCAACACC	7	35.9	3D-0917880-T7-2_1152	TCTTGACACGAGCTTTCCTTTA
T7-38	Vrn2 = ZCCT1/ZCCT2	D1	TraesCS4D02G364400	4D-1117600A-T7-38	reverse	509,340,956	ACTGCTAGCTAGCTCCAAGGGTTGCTTGCTTGTTTGTTTTTTCCTGG	10	50.8	3D-0917880-T7-2_1216	GTTTGCCTGCGGGTTCTAGGA
T7-38	Vrn2 = ZCCT1/ZCCT2	D2	TraesCS4D02G364500	4D-1117610B-T7-38	reverse	509,284,203	ACTGCAGCTAGCTCCAACGTTTGCTTGCTTGCTTGTTCGTTTCTGGT	16	82.6	3D-0917880-T7-2_1280	CTTCACTCACTTGCTGCTCCG
T7-39	Tb1	ABD	TraesCS4A02G271300	4A-0986290-T7-39	reverse	582840989	GCTTTAGTGATGGGACTCCAAGACCCCTGAGACCTCCCGTCTCTAGG	15	71.8	3D-0917880-T7-2_1344	TTGTGTGTTTCGAACCTCTGG
T7-39	Tb1		TraesCS4B02G042700	4B-1076210-T7-39	forward	30,362,277	TATAAAGAGGTCTCACTCTTCTTACTGTAGCAGTATGGGACTAAACTT	14	69.2	3D-0917880-T7-2_1472	AGACGTCACGAGGCTGATGCT
T7-39	Tb1		TraesCS4D02G040100	4D-1159720-T7-39	forward	18,464,255	AAATGACTAAGGATGGGAATGGGTCTGTCCGCTCTCGCCCGGACCT	13	63.2	3D-0917880-T7-2_1536	CTCTCCATTCGTATCCCCTTGG
T7-4	FT4	ABD	TraesCS2A02G132300	2A-0358780-T7-4	reverse	79,333,386	AATATTTTGGAGTCTCAGAGCAAGTGTATACACTATCAAACTACAAAA	18	84.2	3D-0917880-T7-2_1580	CTCAGTGTGAAAAAATTGTCT
T7-4	FT4		TraesCS2B02G154800	2B-0485540-T7-4	reverse	122,756,877	AATATTTTGGAGTCTCAGAGCAAGTGTATACACTATTGAACTACAAAA	20	85.2	1A-0043540-T7-3_0	ATACTGGAGGACTACCAGTTA
T7-4	FT4		TraesCS2D02G134200	2D-0590500-T7-4	reverse	79,068,117	AATATTTTGGAGTCTCAGAGCAAGTGTATACACTATTGAACTACAAAA	19	81.4	1A-0043540-T7-3_128	GTTGTCTTATTCACCTTTCAAA
T7-40	ERS	ABD	TraesCS4A02G274300	4A-0949140-T7-40	forward	584,272,892	ATTTGATTTCGCGTTGCTCTCCCCGTGCGAGCTGCATCCTTTTTGCG	10	52.9	1A-0043540-T7-3_192	CGTCATTGATATTTGTCGATTA
T7-40	ERS		TraesCS4B02G039300	4B-1102590-T7-40	reverse	28,072,756	CGCCGAAGGTTCAGCACCGCATCAGTTGTCACAACATTCCCTGCGG	8	53.2	1A-0043540-T7-3_256	CATGCATGCTAAAGCCATCCTA
T7-40	ERS		TraesCS4D02G326400	4D-1163300-T7-40	reverse	16,235,046	CGCCAAAGCTTCAGCACTGCATCAGTTGTCACAACATTCCCTACAGA	11	54.6	1A-0043540-T7-3_320	GTAAACGATAGTTAGCGCATT
T7-41	Shaggy-like kinase 5	ABD	TraesCS3A02G164200	3A-0682250-T7-41	reverse	168839112	GGTCTGTAGCTCACGGTTCTTGTAGCGCTTATCCTGAAGGACCTTCT	17	53.1	1A-0043540-T7-3_384	TGAACCTAACAATGTAGTGAC
T7-41	Shaggy-like kinase 5		TraesCS3B02G201800	3B-0768030-T7-41	forward	236,123,894	CTCTCCACTCACTGCCACCCATTACCGTTCACCAGCCCGCGTCGAGT	10	52.9	1A-0043540-T7-3_448	CAACTGCATGCTTACTGGTATT
T7-41	Shaggy-like kinase 5		TraesCS3D02G177600	3D-0917540-T7-41	forward	159,425,667	GCACCCGTCACGCGTCCCTCTCACGTCCCCTCGCTGTCCCGCGGGC	11	51.3	1A-0043540-T7-3_512	GTTTTCTTGTTGGCATGTAATG
T7-42	ELF3	ABD	TraesCS1A02G443200	1A-0034450-T7-42	reverse	591,615,395	CGCGGCCAGCTAGACAACTAGCTAGGACGCCATCATCGCTCGGCCG	15	66.4	1A-0043540-T7-3_576	ATATTCCTTATAGCTTAGGGTC
T7-42	ELF3		TraesCS1B02G477400	1B-0087580-T7-42	reverse	685,649,253	CGCCGCCAGCCAGCTAGGCCGCCATCATGCGCGCTCACTCGGCCGG	15	76.2	1A-0043540-T7-3_640	AGGAATGGAAAACATATGCTT
T7-42	ELF3		TraesCS1D02G451200	1D-0221450-T7-42	reverse	493,488,588	CGCGGCCAGCTAGCTAGGACGCCATCATGCGCGCTCGCTCGGCCGG	5	28.1	1A-0043540-T7-3_704	ACTGTTGTTTACAAAGTGAATT
T7-43	PhyA	ABD	TraesCS4A02G262900	4A-0987490-T7-43	forward	575,027,251	TCCATTGCCGCCGGCGCCGGGCAGCAGGTACCCTCTCCTCTCCTCTC	21	92.2	1A-0043540-T7-3_768	GGAACAGGGCCATCACTCTTC
T7-43	PhyA		TraesCS4B02G052000	4B-1091340-T7-43	reverse	40,783,947	CACCTGCCTCGAGTACTCGAACCGCTCTCAGCAGCGGTATCTGCTGT	18	86.4	1A-0043540-T7-3_832	CACTGTTATGTTCTGTGCAACT
T7-43	PhyA		TraesCS4D02G052200	4D-1181480-T7-43	reverse	28,460,436	CTCCTGCCTCGAGTACTCGAACCGCTCTCAGCAGCGGTATCTGCTGC	22	94.8	1A-0043540-T7-3_896	TTAATCATTGCATCTCTTCACT
T7-44	PhyB	AB	TraesCS4A02G122500	4A-1008180-T7-44	forward	151946937	CCACATGTTGGAGATATCAGAGTTTTAAAATAAATTCAGGATAAATG	7	32	1A-0043540-T7-3_960	TGGCTTTATTTTTAACCGTGCC
T7-44	PhyB	no D	TraesCS4B02G182400	4B-1061080-T7-44	forward	399,940,838	AGTTGGTTCGCCGAGCGCACATGTGTTGATGGTACTCCCTCCTTTTC	4	24.9	1A-0043540-T7-3_1024	GTATTTCTTTTGCCAGTTTTCT
T7-45	PhyC	ABD	TraesCS5A02G391300	5A-1238510B-T7-45	forward	586,595,241	TATGCCCTTCATCGCACACGCAACTAAAACCTGAATCGTGTTTGATG	4	28.2	1A-0043540-T7-3_1088	AGTTTTATTCGATAGGCTCGC
T7-45	PhyC		TraesCS5B02G396200	5B-2102190-T7-45	forward	573,216,965	ACATTGAGAGGGTCCTAAGAATATATCTCCATCATCGGAGCAACAAA	6	35.8	1A-0043540-T7-3_1152	ACAACCCATCTAGCTATTTGCT
T7-45	PhyC		TraesCS5D02G401200	5D-1414250A-T7-45	reverse	466,231,744	TGGCTTATGCTGGATTTGTTGTGAATTGCCGTCGCAATTGAAACCCT	13	72	1A-0043540-T7-3_1216	ATCAGCTAAGTAGAAGTATAC
T7-46	MOC1 (MONOCULM 1)	ABD	TraesCS7A02G382800	7A-1781730-T7-46	forward	557,553,815	ATTGACTGGAGTGTATGGATTACAAGAGATTGCAAACCCTTGATATG	5	32	1A-0043540-T7-3_1280	CCTACAGGACTATAGCACGATT
T7-46	MOC1 (MONOCULM 1)		TraesCS7B02G285500	7B-1867270-T7-46	forward	519,784,422	ATCCAATAACTCATCCATGTAAGTATTATGGCAAATATCGAGCAATTTT	11	54.6	1A-0043540-T7-3_1344	TTTTGTAGAAACTTTACAAAG
T7-46	MOC1 (MONOCULM 1)		TraesCS7D02G379200	7D-2074540-T7-46	forward	491,246,692	ACTTGATTGACTGGAGTGTATGGATTACAAGAGATCGCGAACCATTG	6	35.8	1A-0043540-T7-3_1408	CTGCCCTGGGATAGCAAAGTA

T7-47	FIZZY-RELATED 4		TraesCS3A02G527600	3A-2083710-T7-47	reverse	741,974,617	GGTGACCGGACCGATCTGCGTCGTAGCCTAGCCTCTCGCGCTCCGC	14	68	1A-0043540-T7-3_1472	TTCCTTCTTCACATgggtggggt
T7-47	FIZZY-RELATED 4	ABD	TraesCS3B02G595600	3B-0757990-T7-47	forward	818,749,122	GCCGGAAAGTGTACTTTTGACTCCGCTTAGAGATATCATTAACTGAT	12	65.4	1A-0043540-T7-3_1536	CCTCAGGCTGTCCTCCATATCA
T7-47	FIZZY-RELATED 4		TraesCS3D02G533000	3D-0851700-T7-47	reverse	608,778,288	GGTGACCGGACCGATCTGCGTCGTAGCCTAGCCTCTCCCGCTCCGC	13	67.5	1A-0043540-T7-3_1580	CACATTACATGCTAGAAAAAC
T7-48	LAX	ABD	TraesCS3A02G350600	3A-0617410-T7-48	forward	599326045	CATTAGTATCATATGCATAATACTATGATACTCCCCATTACAACTAGCCT	20	91.8	1B-0103850-T7-3_0	ATACTGGAGGAGGACTACCAA
T7-48	LAX		TraesCS3B02G383000	3B-2099820B-T7-48	reverse	602,782,590	GGCAAGCTAGGAAGGATGCCTGGCTAGGTGTTGTCTAAACCACTGT	3	17.9	1B-0103850-T7-3_63	TCTAGAGACCTGAAACAAACT
T7-48	LAX		TraesCS3D02G344600	3D-0882640-T7-48	forward	456534317	GCATTTGGTGTGCTCCAATCTCGATTTGCTGTTGTCCGGTACCCCACT	12	55.1	1B-0103850-T7-3_126	CGATGAGGGTTGTCTTATTCA
T7-49	MAX3 = HTD1	ABD	TraesCS2A02G414600	2A-0283820-T7-49	reverse	671,707,431	GCTTCGGCGTTGGTCTCTTTCGGTCGTTGGTTAGGTTCCTTGCGGTT	19	83.5	1B-0103850-T7-3_252	CGTAGATATTTCGACATGCATG
T7-49	MAX3 = HTD1		TraesCS2B02G433800	2B-0383450-T7-49	reverse	622,917,435	GCTTTGGCGTTGGTCTCTTTCGGTCGCTGGTTACTTGGTTCCTTGTG	17	79.3	1B-0103850-T7-3_315	ACAAGCTTGTGAATTGTGGG
T7-49	MAX3 = HTD1		TraesCS2D02G411900	2D-0546590-T7-49	reverse	526,519,506	GCTTTGGCGTTGGTCTCTTTCGGTTGTTGGTTACTTGGTTCCTTGTG	9	47.1	1B-0103850-T7-3_378	ATGGGTACCATATTTCTGAAC
T7-5	FT5	B, U	TraesCS4B02G380600	4B-1054780-T7-5	reverse	660,684,880	ATACTGGACCCTTCTCTGTTATTCTGTTAACCTACCTCTCTATGGCCCT	20	87.3	1B-0103850-T7-3_441	ACGTACTTAGGTTCTTCATTTC
T7-5	FT5		TraesCSU02G130900	U-1126650C-T7-5	reverse	110,701,425	ATACTGGACCCTTTTCTATTATTCTGTTAACCTACCTCTCTACAGACCT	21	92.2	1B-0103850-T7-3_504	TTTTGAGATATCTGCTGATGTT
T7-50	IPA1 = TaSPL17	ABD	TraesCS7A02G246500	7A-1805200-T7-50	reverse	225,636,127	TGCGGCGCCTGTCGTCGGCCCTAGCTAGCTCGTGGTGCGCTGGCGA	7	36.2	1B-0103850-T7-3_567	GATGTTTTCCCTTCTGAAAACT
T7-50	IPA1 = TaSPL17		TraesCS7B02G144900	7B-1949880-T7-50	forward	187,777,497	TTATTTTCGTTAGCAGTAGCGCCTTACACAGAAACGCGCTGCTACAA	10	57.4	1B-0103850-T7-3_630	AGGGATTGCATAAAATGGACT
T7-50	IPA1 = TaSPL17		TraesCS7D02G245200	7D-2034840-T7-50	reverse	213,790,938	TGCGGCGCCTGTCGTCGGTCCTAGCTAGCTGGTGGTGCGGTGAGCT	7	42.8	1B-0103850-T7-3_693	AGCATTACTGTTGTTTACAAA
T7-51	STM = WRS1	ABD	TraesCS5A02G405900	5A-1248610-T7-51	forward	597499163	GCGGAGCCATCATATGAACAACCTCTATGGTGCATAAGAAGCACACT	15	79.5	1B-0103850-T7-3_756	CTATGTTGGAACAGGGTCATC
T7-51	STM = WRS1		TraesCS5B02G410600	5B-1294870-T7-51	forward	586,275,553	AGGGACACATCAAGGATCGGAAATCCTTATGTGTCCTACAAAATGG	9	60.2	1B-0103850-T7-3_819	AGCCATTGTTATGTTCTTTGTT
T7-51	STM = WRS1		TraesCS5D02G415900	5D-1436310-T7-51	forward	478,449,130	AGGGAGCCATTAGTTACTGGGTGCAACGAATGAGAGATGGTAGCA	14	69.2	1B-0103850-T7-3_882	AGCACTAGCCTAATCATTGCAT
T7-52	FEA2 = CLAVATA2	AB	TraesCS6A02G214600	6A-1515830-T7-52	forward	393,542,146	TGCCCTTTCGCCTCCCTGCACGGCCAAGTCCGGGCGCGCCTTGGGC	6	32.5	1B-0103850-T7-3_945	GCTTTTGTGTTGGCTTTAGTTT
T7-52	FEA2 = CLAVATA2	no D	TraesCS6B02G244700	6B-1594430-T7-52	reverse	436,807,841	GGTGGTTCGGTTGTGGTGGCTAGGGTTTTGCGGGGTTTGAGCGCG	4	24.9	1B-0103850-T7-3_1008	GTGTTTTAGGTTTTTTTTGCCA
T7-53 & T8-27	VRT2	ABD	TraesCS7A02G175200	7A-1847780-T7-53	forward	128,826,522	AGCATGAATTCCTGCCGTCTCCTTTGGGCGGCGATGTTAGGAGATCT	14	69.2	1B-0103850-T7-3_1134	TTGGGCCAACGCAACAACCCA
T7-53 & T8-27	VRT2		TraesCS7B02G080300	7B-1937780-T7-53	forward	90,189,613	GCGGTGATAGTCGTTTGGTGATCTAGAGACCTTGATGTAATTTGCAC	11	51.3	1B-0103850-T7-3_1197	TACCTGCAGAATTATCAGCTAA
T7-53 & T8-27	VRT2		TraesCS7D02G176700	7D-2038090-T7-53	forward	128,912,416	GCAGGAGGAGGATGAGCCGGAGCCCGGCACCGTCGTCGTGGACAT	12	58.4	1B-0103850-T7-3_1260	CATCCACTTGCTGACTTGCATC
T7-54	TOE1		TraesCS1A02G058400	1A-0073500-T7-54	reverse	38,733,045	CGCCGAGATCACCACTTCCAAACTTCCAATCAATCAACGAGAACACC	19	83.5	1B-0103850-T7-3_1321	TTGTATGACATATGGAAAGTTT
T7-54	TOE1	ABD	TraesCS1B02G076300	1B-0159520-T7-54	forward	59,193,372	TTTAGCAGGGCCAGCCTTTCTAAAGGTCTGATAGGTAAATGTCTTTT	17	77.2	1D-0201670-T7-3_0	ATACTAGAGGAGGACTACCAA
T7-54	TOE1		TraesCS1D02G362500	1D-0202460-T7-54	reverse	444,846,959	ACCCACCTCTTCTTCCCTCTCTCCAACAGATGTGAATGTGATCGATCG	13	68.7	1D-0201670-T7-3_128	ATGAGGGTTGTCTTATTCACCT
T7-55	ETTIN = ARF3	ABD	TraesCS3A02G292400	3A-0637570-T7-55	reverse	522,277,032	GGCGCCCGCCCGCAGCCCAGCCGTCCCGTCCGGAAAAGAGGGGG	2	14.1	1D-0201670-T7-3_192	ATACTCGTTACATCATTGATATT
T7-55	ETTIN = ARF3		TraesCS3B02G327000	3D-0844620-T7-55	reverse	401899417	TAATGCCCCCGAGGACGTCGCTCTGCAGCCATCTCCTTGTTGTTCTA	5	25.4	1D-0201670-T7-3_256	GATATTTCGACATGCATGCTTA
T7-55	ETTIN = ARF3		TraesCS3D02G292100	3B-0742350-T7-55	reverse	527,660,917	AGCTCGACGTCCACCACGCGGCACGCCACATGCGGCGGCAGGGCC	6	25.6	1D-0201670-T7-3_320	CTTGTGAATTGTGGGCGATAG
T7-56	FZP = ERFL1	ABD	TraesCS3A02G328000	3A-0627440A-T7-56	forward	573,112,450	CATCGACGACCCTGTTGTTGGCCAAATGCTTGTTCTAACGAACACCA	18	74.4	1D-0201670-T7-3_384	ACCATATTTCTGAACCTAACAA
T7-56	FZP = ERFL1		TraesCS3B02G357500	3B-0769340-T7-56	forward	567806997	TGTTCTAACGAACCCTGACACAGTGTCCTGATCAATGGAGAGAGCA	19	81.4	1D-0201670-T7-3_448	TTTTTCATTTCAACTGCATGCT
T7-56	FZP = ERFL1		TraesCS3D02G321500	3D-0892640-T7-56	forward	434,210,882	CATTGCACGGGAGCATTAGAGATGGAGAGAGGCGTCGTGCAGGTG	20	77.1	1D-0201670-T7-3_512	TGCTGATATTGTTTTATTGTTG
T7-57	BRI1-like protein 2	ABD	TraesCS3A02G099400	3A-0671630-T7-57	forward	64,281,433	GCGCCGGCCTCAAGACGCTCAACGTATCCTACAACGCCCTCGCCGGCGCGATCCCGGAGTCGATCGGCGACGTCG			1D-0201670-T7-3_576	TCTGAAAACTCATACTCCTTAT
T7-57	BRI1-like protein 2		TraesCS3B02G115800	3B-0782160-T7-57	forward	82,623,078	AGAACCTCCGGGTGACATGGGTGGACGCATGTCCTCCGGTGTGCCT	9	43.1	1D-0201670-T7-3_640	AATGGGCTAGCAGGAATGGA
T7-57	BRI1-like protein 2		TraesCS3D02G099700	3D-0902910-T7-57	forward	51,682,428	GCCCCGGCCTCAAGACGCTCAACGTGTCGTACAACGCCCTCGCCGGCGCGATTCCAGAGTCGATCGGCGACATCG			1D-0201670-T7-3_704	CTGGTGACATTACTGTTGTTTC
T7-58	FCA	ABD	TraesCS5A02G162800	5A-1242240-T7-58	forward	348,177,724	CCAACGAGTGCCTGTCCCTGGGCGTGGCGAAATCCTGCCCACGGCA	10	50.8	1D-0201670-T7-3_768	TACTCTATGTTGGAACAGAGC
T7-58	FCA		TraesCS5B02G160200	5B-1296540-T7-58	forward	295,206,901	ATATTGTTGTTCTATTTTGTAAGGTCCTCAAGAATAAAAAATAAAATG	2	14.1	1D-0201670-T7-3_832	CTTCTTGAAGCCATTGTTATGT
T7-58	FCA		TraesCS5D02G167800	5D-1445360-T7-58	forward	262,669,690	TGTGAGCAAATTCCAAGCAATGCCTTCATCAGGCTAACGACATAAAG	19	78.1	1D-0201670-T7-3_896	GTAGCACTAGCTTAATCATTGC
T7-6	FT6	ABD	TraesCS6A02G160200	6A-1547550-T7-6	forward	153,520,904	GTCAGCGGCCCAACGGCAGGGGGCCGTACGTCAGGAGATCCGGCT	12	57.2	1D-0201670-T7-3_960	TGCTTTTGTTGTTGGCTTTATT
T7-6	FT6		TraesCS6B02G191200	6B-1652570-T7-6	reverse	225,311,941	TTCTAGTGTATATATGATGTTGTAGTATAGTATACGCAGATGTATGTGT	12	58.4	1D-0201670-T7-3_1024	TCATGTATTGTAGTATTTCTTTT
T7-6	FT6		TraesCS6D02G152500	6D-1735170-T7-6	reverse	126,617,075	TTCTAGTGTATATATATGATGTGTATAGTATACGCAGATGTATGCGTGTT	12	60.5	1D-0201670-T7-3_1152	ATTGGGCCAACCCAACAACTC
T7-7	FT7	ABD	TraesCS5A02G154600	5A-1192330-T7-7	reverse	331889869	CGCCGGCTCGATCGGAAGACTTGTCACCAGCTAGCTAGCTAATTTTC	22	91.5	1D-0201670-T7-3_1216	TACCTGCAGAAGTATCAGCTA
T7-7	FT7		TraesCS5B02G152800	5B-1312440-T7-7	reverse	282,012,192	CGCCGGCTCGATCGGAAGACTTGGCACCAGCTAGCTAGCTAATTTTC	20	90.6	1D-0201670-T7-3_1280	GTCCACTTGCATCCCTACAGG
T7-7	FT7		TraesCS5D02G159500	5D-1447710-T7-7	reverse	249,087,580	CGTCGGCTCGATCGGAAGACTTGTCACCAGCTAGCTAGCTGATGTTC	21	91.1	1D-0201670-T7-3_1344	GGCATATGGAAAGTTTTGTAG
T7-9	FT10	ABD	TraesCS5A02G297300	5A-1227510-T7-9	reverse	505,023,557	AACTCGGTATTATTATTGAACGGAAGGCTATGCCAGAGGATTTTACTC	21	94.4	1D-0201670-T7-3_1408	AGGATTCTATTTACTGCCCTGC
T7-9	FT10		TraesCS5B02G296600	5B-1319240-T7-9	reverse	479,732,830	AACTTGGTTTTATTATCGTAGGAAGGCTACACAAGAGGATTTTACTG	15	71.8	1D-0201670-T7-3_1472	TATAATCCACTTGTTCCTTCTTC
T7-9	FT10		TraesCS5D02G304400	5D-1460980-T7-9	reverse	399,620,231	AATTAATCCTCATGGCAGCTGACTTTACGAAAGGATCCAACACATCA	17	80.5	1D-0201670-T7-3_1536	TTACCTCAGGCTGTCCTCCATA
										1D-0201670-T7-3_1580	CATCACGTTACATGCTAGAAAA
										2A-0358780-T7-4_0	AATATTTTGGAGTCTCAGAGC
										2A-0358780-T7-4_64	GGGTTTGGGGACTATTTATAG
										2A-0358780-T7-4_128	TGATTCGCGTTCTAATAATTAG
										2A-0358780-T7-4_256	CGCGATAAGAGCATATGTAAC
										2A-0358780-T7-4_320	GGAATTATATATCTTTTGTTCTT
										2A-0358780-T7-4_384	TTACATGGTATCATTGGCAATT
										2A-0358780-T7-4_448	TTGATATAGTACTTCTACGCAA
										2A-0358780-T7-4_512	tatatatatatatatatatatatat
										2A-0358780-T7-4_576	CTGTTATACTCATTAAATTTAA
										2A-0358780-T7-4_640	TGGTGTTTAGGCCTTTCAATTT
										2A-0358780-T7-4_704	AATATTGCTTTTATCTATGCCTT
										2A-0358780-T7-4_768	TATCATTTTCTTCTTAACTGTAT
										2A-0358780-T7-4_832	AGTTCATTAATTTGATGGATCT
										2A-0358780-T7-4_1024	ACAAAACTAAAAAATATGGCA
										2A-0358780-T7-4_1088	CATTCTCTAAGAAAGCAACGA
										2A-0358780-T7-4_1216	ACATATGGCAATAAACTTACG
										2A-0358780-T7-4_1280	TCTTCTTCTTTTTGGCAGGGA
										2A-0358780-T7-4_1344	TATTTCAAATGGAAAACAATAT
										2A-0358780-T7-4_1408	GCTTCTGCGCAAATTAAGTTCT
										2A-0358780-T7-4_1472	GTTAAGTAGCACATGCCCTTG
										2B-0485540-T7-4_64	GGGTTTGGGGACTATTTATAG
										2B-0485540-T7-4_128	TCGCGTTCTAATAATTAGAATA
										2B-0485540-T7-4_256	ATAAGAGCATATGTAACAAAT
										2B-0485540-T7-4_320	TTATATATCTTTTGTTCTTCTAT
										2B-0485540-T7-4_384	ATGGTATCATTGGCAATTGGC
										2B-0485540-T7-4_448	TGGTATAGTACTTCTATGCAAG
										2B-0485540-T7-4_512	ATATTATTGAGTGCTGTTATACT
										2B-0485540-T7-4_576	ACAAAACATATGTGGTATTTAG
										2B-0485540-T7-4_640	CTTTCGGTAGTAGTGTTTGTCA
										2B-0485540-T7-4_704	TAAATCAACATTTGTAAAAAAA
										2B-0485540-T7-4_768	AGTGATAATATAATGCTTAACA
										2B-0485540-T7-4_960	CTGATAGACGGCAACCCGGA
										2B-0485540-T7-4_1024	GTACATGAAAACTCCTGCATA
										2B-0485540-T7-4_1088	CCGGCATAATTCAACATGTTTG
										2B-0485540-T7-4_1152	CTTCTGCATCCATACAAATTATA
										2B-0485540-T7-4_1216	ATATATTCGTGGTTTTCAATCAT
										2B-0485540-T7-4_1280	TTTCTTAGAGAATTCTAATCCG
										2B-0485540-T7-4_1344	AACATTACCTTTTTTGTGAAAA
										2B-0485540-T7-4_1408	ACCCTTACAAAAAATTTAAAAAT
										2B-0485540-T7-4_1472	GCATCCACCGATGGGCAACGT
										2D-0590500-T7-4_128	TCGCGTTCTAATAATTAGAATA
										2D-0590500-T7-4_256	ATAAGAGCATATGTAACAAGT
										2D-0590500-T7-4_320	TTATATATCTTTTGTTCTTCTAT
										2D-0590500-T7-4_384	ATGGTATCATTGGCAATTGAC
										2D-0590500-T7-4_448	TTGATATAGTACTTATACGTAA
										2D-0590500-T7-4_512	TATATTATTGAGTGCTGTTATA
										2D-0590500-T7-4_576	GGACAAAACATATGTGATATTT
										2D-0590500-T7-4_640	TGCTCTCGGTGGTAGTGTTTG
										2D-0590500-T7-4_704	ATAGTTCATTAATTTGTTGGAT
										2D-0590500-T7-4_896	TTTGTCCCACAAAACTAAAAA
										2D-0590500-T7-4_960	GTATTCCATCCTCTAAAAGCAA
										2D-0590500-T7-4_1024	AAAAAATGTGACCACCTGAGT
										2D-0590500-T7-4_1088	CGTTCCGATACATAAGGCAATA
										2D-0590500-T7-4_1152	ATGCGTTCTTCTTCTTTTTGCG
										2D-0590500-T7-4_1216	AATTCTATTTCAAATAGAAAAA
										2D-0590500-T7-4_1280	TTAAAGGCTTCTGTGCAAAGT
										2D-0590500-T7-4_1344	CCATATGTTAAGTAGCACATGC
										2D-0590500-T7-4_1408	TCATTAGGGGCGTAAAAAAAT
										2D-0590500-T7-4_1472	CGTTGCCACCTCTGGGCCTAT
										4B-1126650B-T7-5_0	CAAGTAATTTGGATTACTTAAA
										4B-1054780-T7-5_64	GTGCATGAACTAGTTTTATGGT
										4B-1126650B-T7-5_64	ACAGACTCGAGTAAAGCCTTC
										4B-1054780-T7-5_192	CGAGCTTCATGGAGTAGTCAG
										4B-1126650B-T7-5_192	GCAGCCAACTTTCGAATCTAC
										4B-1054780-T7-5_256	CAATTATCGGGGTGTACTGGA
										4B-1126650B-T7-5_256	CCATAGGTAGTTTGGACAATG
										4B-1054780-T7-5_320	CTGTTCCTTTGGACACAATCTG
										4B-1126650B-T7-5_320	CCACCAAAGCAGATTGTGTGG
										4B-1054780-T7-5_384	AGTGGTACTGGACATATATATG
										4B-1126650B-T7-5_384	GGTTTCGTAAAGTTGTTACATA
										4B-1054780-T7-5_448	CTTTTCTAGTGATGTTAAGTAT
										4B-1126650B-T7-5_448	AGTGCATGAAGAGAGTAATCA
										4B-1054780-T7-5_512	GCATTTAATGCATTTGCATAGT
										4B-1126650B-T7-5_512	AAGAAATATGTTCTCATCTTTT
										4B-1126650B-T7-5_576	TGCATTTGGACATGGATTAATA
										4B-1126650B-T7-5_640	ACACTCTACTTTTTGAACATAC
										4B-1054780-T7-5_704	GTGAATTGCTTCAAAGGGGCT
										4B-1126650B-T7-5_704	AAACTTGCATCTTTGTGCATAG
										4B-1054780-T7-5_768	CACTCTTCCGTTACCACCAAA
										4B-1126650B-T7-5_768	TGATAGCCTGCTGCATTGTTTA
										4B-1126650B-T7-5_832	TTATACACTTATACAACATGTTA
										4B-1126650B-T7-5_896	CTTTGCTTAAATTTGCGCATAA
										4B-1054780-T7-5_960	CGGACGGAGGGAGTATTAATT
										4B-1126650B-T7-5_960	GTTCCATCCAATATAGAGCATC
										4B-1054780-T7-5_1024	CCTGCGTTGTTGGCTTGGTTT
										4B-1126650B-T7-5_1024	AAATGATGTTAATGTGGCCAG
										4B-1054780-T7-5_1088	AGCAGTGCTCTATAAGAGCCT
										4B-1126650B-T7-5_1088	TATTCCCACTAGTCCATTCCTG
										4B-1054780-T7-5_1152	AACAACCCATCCGGTTATTTCT
										4B-1126650B-T7-5_1152	ATGAGTTTTCCGACAGGGTAA
										4B-1054780-T7-5_1216	CACTAATTATTAGATAAGAAAA
										4B-1126650B-T7-5_1216	AGCAGATGTCTTAGAAATTAC
										4B-1054780-T7-5_1280	CACTTGAACATATTCTGATTTG
										4B-1126650B-T7-5_1280	GAACCCCAAACACGTTAGGTA
										4B-1054780-T7-5_1344	TTCTATTACAGTAGAAAGACA
										4B-1126650B-T7-5_1344	GTTTCGAAACAGATTGTGTCC
										4B-1054780-T7-5_1408	TTTTTGGGTATAATACACATAT

4B-1126650B-T7-5_1408	GATTGTTCCAGTACACCCCAG
4B-1054780-T7-5_1472	CACATAGTTCTACACTTCATAG
4B-1126650B-T7-5_1472	CAACAGCCTGAGAGCCTGACT
4B-1054780-T7-5_1536	CGAAGTCGCAAAGGGGTTCA
4B-1054780-T7-5_1580	GCTTTGGTGGGTCAGTCCACT
U-1126650C-T7-5_0	ATACTGGACCCTTTTCTATTAT
U-1126650C-T7-5_64	ATGCACGAACTAGTTTTATGG
U-1126650C-T7-5_192	GGAGCTTTATGGAGTAGTCAG
U-1126650C-T7-5_256	TGATTATTAGCATTGCTGGGG
U-1126650C-T7-5_320	ATCAGTTGTTCCTTTAGACAC
U-1126650C-T7-5_384	ACGTTGTGGTACTGGACATAT
U-1126650C-T7-5_512	AATGCATTTGCATAGTGTTAA
U-1126650C-T7-5_640	TTGCATTCACTTAATTGCCCTC
U-1126650C-T7-5_704	CCCTTCAAAGGGGGCCTGCC
U-1126650C-T7-5_768	TCTTCTGTTACCACCAAAAAA
U-1126650C-T7-5_832	CTGTCTCTTCACTGTATTACTT
U-1126650C-T7-5_896	GATGTTTGCTCCTGTGTTGTT
U-1126650C-T7-5_1024	GCCAACCCAACAACCCATCCA
U-1126650C-T7-5_1088	GAGTAAATCACTAATTATTAGA
U-1126650C-T7-5_1152	TTTCCACACTCAACATATTCTG
U-1126650C-T7-5_1216	TTACAGTACAAAGACAAATGA
U-1126650C-T7-5_1280	GGGTATAATACACATTGATTAC
U-1126650C-T7-5_1344	AGTAGTTCTACACTTCGTAGT
U-1126650C-T7-5_1408	AAGTCACAAAGGGGTTCATAT
U-1126650C-T7-5_1472	TCATCCCTAGTACCAATACGTG
U-1126650C-T7-5_448	TCTAGTGATGTACAGTAATTTT
6A-1547550-T7-6_64	CTCTCTGCTCCCATTAATATAG
6A-1547550-T7-6_128	TTCCAGTGCATGGGTGCTTAA
6A-1547550-T7-6_704	AAGCATTGGGAGGGGACAAA
6A-1547550-T7-6_768	AGATACTCTAGGTGAGATTTA
6A-1547550-T7-6_832	TGTGCAAGTTTTATGCCTGGC
6A-1547550-T7-6_896	TTTGTATCTATATGTGTGACCA
6A-1547550-T7-6_960	CGTATTTTCAATAAATGTTGAG
6A-1547550-T7-6_1152	taaaatcaaattaaataaaaaataac
6A-1547550-T7-6_1216	AAAAAAATTCACATTTTTTGA
6A-1547550-T7-6_1280	TTTGGATCCTCCACGTGTTTA
6A-1547550-T7-6_1344	CATCAAATTGTAGATGTGTATA
6A-1547550-T7-6_1536	TGATATGCATGGCGGTTTCTC
6A-1547550-T7-6_1580	TAAATATGACGCCTAAGTACCT
6B-1652570-T7-6_0	TTCTAGTGTATATATGATGTTG
6B-1652570-T7-6_192	GTTCCTTGTATGCCCTTGATAG
6B-1652570-T7-6_384	GACATTTGTATGATATGACGA
6B-1652570-T7-6_448	GTGAGCTCAGGTGGACAGTA
6B-1652570-T7-6_640	CGTCCGTGGAGGACCGACCA
6B-1652570-T7-6_704	AGCTCTGAACTGTCATCGAGT
6B-1652570-T7-6_768	CCTCAACATTTACTGAAAGTA
6B-1652570-T7-6_832	ATCGTCACACATAGATGCAAA
6B-1652570-T7-6_896	AAAAAAACAATATATTGCTCG
6B-1652570-T7-6_960	GGTACGCGTAGACAACCCCG
6B-1652570-T7-6_1024	TATAATTAACTATGAAAAACAC
6B-1652570-T7-6_1088	TGAAAGGTGTTTGATAGGCTA
6B-1652570-T7-6_1536	GAATCTGGGCACCGAATCGCT
6D-1735170-T7-6_0	TTCTAGTGTATATATATGATGTG
6D-1735170-T7-6_64	GTGTAATGGCTTGGTATAAAG
6D-1735170-T7-6_192	ATCGTTCGTTGTATGCGCTTG
6D-1735170-T7-6_256	GATTCCAGGCATGTCTTTCTA
6D-1735170-T7-6_320	ACAATTATTGGAGGGCATATA
6D-1735170-T7-6_384	ACATTTGTATGATATGACGGA
6D-1735170-T7-6_448	TGAACAGTAAAATAAAATAAA
6D-1735170-T7-6_512	AAATTCATAttgttttttttttcattta
6D-1735170-T7-6_576	CGGGATTAGAACAGATGCGT
6D-1735170-T7-6_640	CTTGGCATTATTTCTCCAGCTC
6D-1735170-T7-6_704	AAACCGGCTTACTTTCCTCAA
6D-1735170-T7-6_768	GGTGGGGGAGAGAGCGTGT
6D-1735170-T7-6_1088	aaCACCATATACTATTAACCATG
6D-1735170-T7-6_1152	TGGGGGAGAGAGCGTGTACT
6D-1735170-T7-6_1216	gatgggaggtgagggagagagaggg
6D-1735170-T7-6_1536	AGCTAAATTACTCCCACTATGA
6D-1735170-T7-6_1580	gtgtgtgGAATAGGACGGGGGA
5A-1192330-T7-7_64	GCTAGCTAGCTAATTAAGCTC
5A-1192330-T7-7_128	GCTTGAAATGGATACGATACG
5A-1192330-T7-7_256	GAGGTAACCATGCCAAATCCA
5A-1192330-T7-7_320	AGTGCTGACGCCTTAGAAAG
5A-1192330-T7-7_512	TTCTTCACGTAAAGTGGATGC
5A-1192330-T7-7_576	AAGAACATGAGTTCAACAAGT
5A-1192330-T7-7_640	TTTCAAGTATCATCCAGGTATG
5A-1192330-T7-7_704	GCCTAGCTATATCTTTTTAGCT
5A-1192330-T7-7_768	CCCTACGATAAAACAAGATAG
5A-1192330-T7-7_832	ATACATATATCGGGAATGAGTT
5A-1192330-T7-7_896	CAATACAATTTTATACTCCATG
5A-1192330-T7-7_960	TGACACATTGAGTTAGTAGTT
5A-1192330-T7-7_1024	TAAATTTATAAATTTTTGCTCTA
5A-1192330-T7-7_1088	TGCTAGTTAAATGGAATAGTG
5A-1192330-T7-7_1152	TGGAACGGAGGTAATAGTAGT
5A-1192330-T7-7_1216	ATTCGCTCCGCTGGAAAATGT
5A-1192330-T7-7_1280	TTTCAGACTATTCTTCTTACTG
5A-1192330-T7-7_1344	TCCATTGATATCAATTTTGTGT
5A-1192330-T7-7_1408	GCGTGGATTTGTTGGATGATC
5A-1192330-T7-7_1472	AACTAAATGAACCTTTTGTGA
5A-1192330-T7-7_1536	TATCACATGCAAGGTTTAAACT
5A-1192330-T7-7_1580	AGACCTCGCCGAAATATTCAA
5B-1312440-T7-7_0	CGCCGGCTCGATCGGAAGACT
5B-1312440-T7-7_64	GCTAGCTAGCTAATTAAGCTC
5B-1312440-T7-7_128	TTATATGCTTGAAGTGGATACG
5B-1312440-T7-7_192	GATCCATCTTAATCCTCTGGGC
5B-1312440-T7-7_384	ATATACTTACTTAGTTTTCAGG
5B-1312440-T7-7_576	TGTATCTAGACAAATCCAAGA
5B-1312440-T7-7_704	AAAGCAGATATGGTGGTGTAC
5B-1312440-T7-7_768	ACAAAGTTTATATCCCCATATAT
5B-1312440-T7-7_896	TGAATGGTAGAGCCTGTTTGA
5B-1312440-T7-7_960	CACAGAAAATCTTATGGCTCC
5B-1312440-T7-7_1024	TCGAAGTGACTTCTCCTGAAG
5B-1312440-T7-7_1088	TGTTTTGTTTTGAGTTTATATT
5B-1312440-T7-7_1152	TTGTTAATGCAAGAAAATAGT
5B-1312440-T7-7_1216	CAGCGGTACATCCTCATTATTC
5B-1312440-T7-7_1280	AAATGTATGTACATCTCTTTTT
5B-1312440-T7-7_1344	ACAAATCTAAGACAAGTTTCT
5B-1312440-T7-7_1408	TTCAGAGGGTTGTGTGTGCCT
5B-1312440-T7-7_1472	ATTTTCAGTTAACACAATTTAT
5B-1312440-T7-7_1536	TAAAGAACTTTGGAAGGTGG
5B-1312440-T7-7_1580	ACATAACCCGTATATAATATTG
5D-1447710-T7-7_0	CGTCGGCTCGATCGGAAGACT
5D-1447710-T7-7_64	GCTAGCTAACTAATTAAGCTC
5D-1447710-T7-7_128	TTATGTGCTTGAAGTGGATAC
5D-1447710-T7-7_192	GATCCATCTTAATCCTCTGGGC
5D-1447710-T7-7_384	ATATACTTACTTAGTTTTCAGG
5D-1447710-T7-7_448	TTCCCTTCTGCTTTCTCTCTTT
5D-1447710-T7-7_512	TTGGATTCTTCACGTAAAGTT
5D-1447710-T7-7_704	TTTCTAATGACTACTATGCTAA
5D-1447710-T7-7_832	GTCCAAATAATGCAGCCTACG
5D-1447710-T7-7_896	TTAGCAGCGTCCGATACATATA
5D-1447710-T7-7_960	AGAACTTTGACAATATAATTCT
5D-1447710-T7-7_1024	ATTAAGTACAACCCCTGACAC
5D-1447710-T7-7_1088	TATAAAATTTAGCTCTAATTTCT
5D-1447710-T7-7_1152	TTAAATGGAATAGTGGTTCTG
5D-1447710-T7-7_1216	GGAGGTAATAGTAGTTATTCT
5D-1447710-T7-7_1280	TCCCGTGGAAAATGTAAAGCG
5D-1447710-T7-7_1344	ACTATTCTTCTTACTGTTAACA
5D-1447710-T7-7_1408	GATATCAATTTTGTGTCACATA
5D-1447710-T7-7_1472	ATTTGTTGGATGATCAAAATA
5D-1447710-T7-7_1536	GAACCTTTTGTGAACCATGCT
5D-1447710-T7-7_1580	AAATGTGATGTCTATCACATGC
5A-1227510-T7-9_0	AACTCGGTATTATTATTGAACG
5A-1227510-T7-9_128	GTAGTTCGTCGTAAATTTATAG
5A-1227510-T7-9_192	ACTATGAGCAAATGGAATCTA
5A-1227510-T7-9_256	CATGGAAATGTTGAGCACCTA
5A-1227510-T7-9_448	ACGAGTCATTTCATGTTCAAC
5A-1227510-T7-9_512	TTTGATCAACATCAGAATGCA
5A-1227510-T7-9_640	GTATTCGCAGGTCCTATCTTAC
5A-1227510-T7-9_704	GCCAGAGAGGTTCACAAGAA
5A-1227510-T7-9_768	GCTGTTCTTGTTTACTTGAGA
5A-1227510-T7-9_832	TTTTGGGACTATGACATCGTAT
5A-1227510-T7-9_896	TGAGGTAGCCGGTTTGACTAT
5A-1227510-T7-9_960	TTCGTCTTTTTTCCATTTTGAT
5A-1227510-T7-9_1024	TGCACACTGTACAAAATACAG
5A-1227510-T7-9_1088	CCTGGTTCAAGTTGCAGAATC
5A-1227510-T7-9_1152	CCATAGCATTTGAAATCTGGC

										5A-1227510-T7-9_1216	GCATTTGGAGATAGGTGCTAT
										5A-1227510-T7-9_1344	AGAATCCATGCAGATTTTGTAT
										5A-1227510-T7-9_1408	TTCTGCATAAACTTCGGAATA
										5A-1227510-T7-9_1472	TCAGCATGTGTGCAAGATAAC
										5A-1227510-T7-9_1536	TATCTCCAAAATTCCTACAATC
										5A-1227510-T7-9_1580	AAGAAAGCATGAGGTAAAAT
										5B-1319230-T7-9_0	CACTACTTTGTGTTTTGAAGT
										5B-1319230-T7-9_64	AAGGTGGTAAAACATCTTGG
										5B-1319230-T7-9_128	TTAATTTCCGTGCTACCAAAC
										5B-1319240-T7-9_128	TAGTTCGTCGTAAATTTATAGG
										5B-1319230-T7-9_192	CTAAACAAAGAGACTTAGGTT
										5B-1319240-T7-9_192	CTATGAGCAAATGGAATCTAA
										5B-1319240-T7-9_256	TGCATGGAAATGTTGAGCACC
										5B-1319230-T7-9_384	TTGAACTATAAACATAAAAAC
										5B-1319240-T7-9_448	CAATGAGTCATTTCATGTTCA
										5B-1319240-T7-9_512	ATTTCGATCAACATCAGAATG
										5B-1319230-T7-9_576	GGGATATTTCTATCTTAGCATT
										5B-1319240-T7-9_576	TTTTCATTGTTCTTGAAAGCA
										5B-1319230-T7-9_640	CCAGAGCACAACTCTACAGA
										5B-1319240-T7-9_640	GTATTCACAGGTCCTATCTTAC
										5B-1319230-T7-9_768	GTAGTTTTGTCTATTCAGTTAT
										5B-1319230-T7-9_832	CATGAGAGAGCTTCAAACTC
										5B-1319240-T7-9_832	ACTTCCTTTTGGGACTATGAC
										5B-1319230-T7-9_896	ATTCTTGTATAGCACTGTACAA
										5B-1319240-T7-9_896	ATATCACGAGGTAGCCGGCTT
										5B-1319230-T7-9_960	CATGTACTATACTTGATCATTC
										5B-1319240-T7-9_960	TATATATTTATCTTTTTTTATTTT
										5B-1319230-T7-9_1088	AAGTTAGGTATCCTTCTTTAGT
										5B-1319230-T7-9_1152	GTTGCTTTGAGTAAGGAGCTT
										5B-1319240-T7-9_1152	GATTCTCCATATCATTTGAAAT
										5B-1319230-T7-9_1216	TGATTATTCTAGACACCATCCC
										5B-1319240-T7-9_1216	TTGGAGATAGGCGCTATCACC
										5B-1319230-T7-9_1280	AAGGTCATTTTTCATATTATCT
										5B-1319230-T7-9_1344	GGTATTGTGTTACTTACAAGT
										5B-1319240-T7-9_1408	TTTAATTCCGCATAAACTTCGG
										5B-1319240-T7-9_1472	TCTAGTTAGCATACGTGTCAA
										5B-1319230-T7-9_1536	TCCAACACATCACCAATTACCT
										5B-1319240-T7-9_1536	TTTGCAGAAGACAAGTTATAT
										5B-1319240-T7-9_1580	TTCATACAAAGAAAGACCTTA
										5D-1460980-T7-9_64	TGCCAAAGGGTCCCTGCCCCG
										5D-1460980-T7-9_128	GATTTTACTGACACTTCGATCG
										5D-1460980-T7-9_192	GAAAATTTTCTGTGTGGAAGA
										5D-1460980-T7-9_256	TTTATGTTCACAACATTCCATT
										5D-1460980-T7-9_320	ATTATTTTCCTTATAGTTCTTCT
										5D-1460980-T7-9_512	CCTTGCCTGGGCTAGGACAAA
										5D-1460980-T7-9_640	GGGGAGGTAGAAACAGGTCA
										5D-1460980-T7-9_704	CTTCACCCTTCACCCTCGAGT
										5D-1460980-T7-9_896	TATTGTGATTACTTTCAGCGAA
										5D-1460980-T7-9_960	AGGCAAGCTTAGTCTTTCTATA
										5D-1460980-T7-9_1024	CTAATTCTTCTAATGTGCAAAA
										5D-1460980-T7-9_1088	GTATTCATGTTTGATTACCTCT
										5D-1460980-T7-9_1152	CTGGAAGAAACAGGTACTGG
										5D-1460980-T7-9_1216	TTGTCAGAATCAAGCCATATT
										5D-1460980-T7-9_1408	AATCAGCAGCGTGACGAAGA
										5D-1460980-T7-9_1472	ACAACTGGCTGCTACATTTTG
										5D-1460980-T7-9_1536	TGTGCCAAAGCTTTTCTTTCA
										4A-0943490-T7-10_64	TGGCTTGCTGTTATGGCTTGAT
										4A-0943490-T7-10_256	GGGTTAGTGCGGGCGCATCG
										4A-0943490-T7-10_320	CTCTAAGTCTAGACCAGTTGG
										4A-0943490-T7-10_512	GAGGTAGCCTTCATTTTGTCA
										4A-0943490-T7-10_704	AGGATACAATTATGTTGAAATT
										4A-0943490-T7-10_832	CCACATAAAATTGTTTTCTTGC
										4A-0943490-T7-10_896	TCTCTATAACATGGATTTGTTC
										4A-0943490-T7-10_960	CCCCAAATCTTAGTGACTTGC
										4A-0943490-T7-10_1024	CGTACAGCTGCTGACATGAGA
										4A-0943490-T7-10_1088	CATTTTCTAGAAGCAATATATG
										4A-0943490-T7-10_1580	gcaaaggcgggaggtctatcctcctt
										4B-1108830-T7-10_0	CTAGAGCATCATTTGTAATAGA
										4B-1108830-T7-10_512	CGCAGCGCCATGCCACCGGT
										4B-1108830-T7-10_704	GAAATAGCAAGTCGCTAAGAT
										4B-1108830-T7-10_768	TCATTGAACAAATCCATGTCAT
										4B-1108830-T7-10_896	GGACTAGTGAGACTTGGTAG
										4B-1108830-T7-10_1088	TTTGAAAAAGAGACTGCCATA
										4B-1108830-T7-10_1280	GCTTCATGGGGCCTAGGTTAA
										4B-1108830-T7-10_1408	AAAGAGAGTAAATGATCGATG
										4B-1108830-T7-10_1472	CTATTGTCACCACTCAAACATA
										4D-1182900-T7-10_0	CTAGACTAGAGCATCATTTGTA
										4D-1182900-T7-10_512	CAGCGCCATGCCACCGGGTG
										4D-1182900-T7-10_576	CACTATTCCCATATATTGCTTCT
										4D-1182900-T7-10_704	AAATAGCAAGTCGCTAAGATT
										4D-1182900-T7-10_768	GATTGAACAAATCCATGTTATA
										4D-1182900-T7-10_832	AGTTGCAAGAAAACAAATTTA
										4D-1182900-T7-10_896	GACTAGTGAGACTTGGTAGA
										4D-1182900-T7-10_1088	TTGAAAAAGAGACTGCCATAT
										4D-1182900-T7-10_1280	CTTCATGGGGCCTAGGTTAAA
										4D-1182900-T7-10_1344	TAATCCAACTGGTCTAGACTTA
										4D-1182900-T7-10_1472	CTATTGTCACCACTCAAACATA
										7A-1814120-T7-11_128	GGTCCTGTGCACTACAATTTG
										7A-1814120-T7-11_320	TGCAGGTAAACCGTAACTCCT
										7A-1814120-T7-11_384	AACTTGGAAGGAAGCCAAAA
										7A-1814120-T7-11_512	AGGGACCAAATTTTGGCGAG
										7A-1814120-T7-11_576	ACCAGGTGTGGAGACAACCC
										7A-1814120-T7-11_896	TTTTGCTAAAGGGTGCAACTG
										7A-1814120-T7-11_960	CCATAATGGCTTCTAGTTTCCT
										7A-1814120-T7-11_1152	GAACACACGGATCATCCTGAC
										7A-1814120-T7-11_1216	GCAAACATGCAAATTTCTTTC
										7A-1814120-T7-11_1536	AACACATACGAAAGAGCCATG
										7B-1952050-T7-11_0	AGACTTACATGCAAGTTTAAC
										7B-1952050-T7-11_64	AGTATCTATTTCACAGAAGAAT
										7B-1952050-T7-11_128	AATAAAGGAAAATACACAAAG
										7B-1952050-T7-11_192	TTGACACACTACTTAATTCAA
										7B-1952050-T7-11_256	CTACAAAGAAGTTTTCTATGG
										7B-1952050-T7-11_448	ATAAAAGCTCATGAAAATAAG
										7B-1952050-T7-11_512	AGTAATTCATGGTTTGACAGG
										7B-1952050-T7-11_576	ACAGGAGATGGATTTGCCTTG
										7B-1952050-T7-11_640	TGTGAATTCATTGCTGATTAG
										7B-1952050-T7-11_704	ACTTCTCCACTACACATCATAT
										7B-1952050-T7-11_896	TAACAGAAGACAGGCCCCCTT
										7B-1952050-T7-11_1024	GTTTAGTCCAATCCAGGCAAA
										7B-1952050-T7-11_1088	GAGAGTTCTCTTGATCCAAGT
										7B-1952050-T7-11_1152	GCATCTGAGTCCAAATGTAAA
										7B-1952050-T7-11_1216	TGTAAAAGATTTAAGTCTTCTA
										7B-1952050-T7-11_1472	TCACCCCACTGACACCCTACT
										7B-1952050-T7-11_1536	TCCATACTCACATAGGCAGTG
										7D-2057910-T7-11_0	CCTGAAACTAGTTTTAGCTGT
										7D-2057910-T7-11_64	TTCCTCGTTTTGCATGGGTTTT
										7D-2057910-T7-11_256	GCGGTGTTTGGTGGCTTTGG
										7D-2057910-T7-11_320	ATAAAACTATAATATGACATGC
										7D-2057910-T7-11_384	ATGTTAGATAAAAGGAACTCA
										7D-2057910-T7-11_448	TTAATTATTCATATTTGACTTAA
										7D-2057910-T7-11_512	CATGAACACAAGCAGTTGATT
										7D-2057910-T7-11_704	GAGACTTAAAATAGTACGGTC
										7D-2057910-T7-11_768	ACAAATCCAGACAGCTACAGT
										7D-2057910-T7-11_960	TCTTCACAAAACTTGGAAGGA
										7D-2057910-T7-11_1024	ATTTTCTTTATACTTTAGCATTT
										7D-2057910-T7-11_1088	GAAAGCACGAGGGACCAAAT
										7D-2057910-T7-11_1152	CGGTATACAACCAGGTGTGGA
										7D-2057910-T7-11_1344	ATTGCTTTAGTTGTCACCATCT
										7D-2057910-T7-11_1408	GGCAACTCATGAGTCACCACC

7D-2057910-T7-11_1536	ACTGGTGGGAATGAGATTCTT
7D-2057910-T7-11_1580	GTTTTGAATCATACATACCAAG
6A-1519320-T7-12_0	ATAAATCTTGCAACTTGTAGG
6A-1519320-T7-12_64	CTATTCAGGAGAATACATATCT
6A-1519320-T7-12_256	CATCTTGTGGTTGCACGCGGA
6A-1519320-T7-12_320	TACATCATTTTTTGTCAATTATT
6A-1519320-T7-12_384	TTTCACGTAATTTGGAATCATC
6A-1519320-T7-12_448	GACTTACTAATGTATAAGGGG
6A-1519320-T7-12_512	GAATGTGTATTCATCTTTTCCT
6A-1519320-T7-12_576	AAATCGTACAGATTTTGTATAA
6A-1519320-T7-12_768	GCCATTCGTTCGTACTCCCCTT
6A-1519320-T7-12_832	GAAGATACACGAGAAAAGTTT
6A-1519320-T7-12_896	TAGATCTGCAAATATCTAAGGT
6A-1519320-T7-12_960	TTTCTTCGCTGCAGTTATTCAT
6A-1519320-T7-12_1088	TGAAATGACTTGTTCTTATGG
6A-1519320-T7-12_1152	TTACAGACAAACTTAGAGCTT
6A-1519320-T7-12_1216	TCATAGTTTGATGTTGGGTGG
6A-1519320-T7-12_1280	GTTAGAAAAAAGGCTTGAGC
6A-1519320-T7-12_1472	TCTTGGCTGCTGCATAATAAGT
6A-1519320-T7-12_1536	ATACTATAAATAATTGAGCAAA
6A-1519320-T7-12_1580	AACAGACTCAAGCCTGTCAAC
6B-1608580-T7-12_0	ATAAATCTTGCAACTTGTAGG
6B-1608580-T7-12_192	TGCATCTTGTGGTTGCACGTG
6B-1608580-T7-12_256	GCTACATCATTTTCTGTCAATT
6B-1608580-T7-12_320	ACTTTCACGTAAGTTGGAATC
6B-1608580-T7-12_384	GAGACTTACTAATGTATAAGG
6B-1608580-T7-12_448	ATTATGTATATTCATCTTTTCCT
6B-1608580-T7-12_512	CAAATCGTACAGATTTCATATA
6B-1608580-T7-12_704	CGATTCGTTGGTACTCCCCTTA
6B-1608580-T7-12_768	AAGATACATAAGAAAAGTTTT
6B-1608580-T7-12_832	TAGATCTGCAAATATCTAAGTT
6B-1608580-T7-12_896	TTCCTTTGCTGCAATTATTCAT
6B-1608580-T7-12_960	TCGACTATCTGTGACTGGAAG
6B-1608580-T7-12_1024	TGAAATGACTCGTTCTTATGG
6B-1608580-T7-12_1088	TTACAGACAAACTTAGAGCTT
6B-1608580-T7-12_1152	TTCATAGTTTGATGTTGGGTG
6B-1608580-T7-12_1280	AATCTGAGTACATAATCATCTT
6B-1608580-T7-12_1344	CATATATGAACATAGTGAATAC
6B-1608580-T7-12_1408	CAGACTCAAGCCTGTCAGCAA
6B-1608580-T7-12_1472	TTCCAGAAAAGCTTACAGGA
6B-1608580-T7-12_1536	GTAGCACTCTGATAATCTTTCC
6B-1608580-T7-12_1580	CTGCTAACAGAAAACAGTAAC
6D-1702700-T7-12_64	CTACATGTAGGGAAAGATTAT
6D-1702700-T7-12_128	AAGATAGTATAGTCCTGTAAG
6D-1702700-T7-12_192	CCAGTATAAGTTTGTTGACAG
6D-1702700-T7-12_384	CCATCCTATCTTAAAATAGTAA
6D-1702700-T7-12_512	GCCTTTTTTATAACAACAAGCT
6D-1702700-T7-12_640	GCTAATTAACTGGACTTCCAG
6D-1702700-T7-12_704	CAAACCAAAACCTATGAATAA
6D-1702700-T7-12_768	TAGACTGAACATCACCTTAGA
6D-1702700-T7-12_960	ACCCCTTTTGCAAAAGATGTG
6D-1702700-T7-12_1024	GTTATACCACATTGCCTTCTAA
6D-1702700-T7-12_1088	ACAGGCAGTTCCTTTTATACA
6D-1702700-T7-12_1152	ATTACGAATTCATTAGGAAAA
6D-1702700-T7-12_1216	CAACATAAAGTGTAACCCCTT
6D-1702700-T7-12_1280	CTCCTGTTCAATTAGATGATTC
6D-1702700-T7-12_1472	GACACCACCTGTCACTCAGTG
6D-1702700-T7-12_1536	CAACACTGACTGCATCCGCTC
6D-1702700-T7-12_1580	GGATCAAAAGCCACGGGAGA
5A-1245850-T7-13_0	CGCCGCCCGCGAGGTCGATC
5A-1245850-T7-13_384	TATGCCCGGTCTCCCATGGCG
5A-1245850-T7-13_448	TGTGCAAGCAATGCCATGTCA
5B-1333300-T7-13_384	TCTATGCCGGTCTCCCATGGC
5B-1333300-T7-13_448	TTGTGCCATGTCAGTCTTTGTA
5B-1333300-T7-13_512	ATATGCAATGCAATTATAGATC
5B-1333300-T7-13_576	AAATGGTTTGTGTGTTTGAAG
5B-1333300-T7-13_640	TCATAGTTTTTTCGAAAAAGG
5B-1333300-T7-13_704	TCGACACAACCAATCACACAA
5B-1333300-T7-13_768	GAGGACAAGTACAACCCCCA
5B-1333300-T7-13_832	AGTCAATGGAGCCCTCAACCG
5B-1333300-T7-13_896	GTGCCAAAGCAATGCCATGTC
5B-1333300-T7-13_960	CAAAAGGATATGCAACGCAAT
5B-1333300-T7-13_1024	AAGAAGAGGTATGCGTGTTTG
5B-1333300-T7-13_1088	CGTTCACATTTTTTTAAAGAG
5B-1333300-T7-13_1152	CTCGACAGAacccaccacacaac
5B-1333300-T7-13_1216	AGAACAACTACAACCCCCACC
5B-1333300-T7-13_1344	GCCAAAGCGGTGTCTTCAGG
5B-1333300-T7-13_1472	GACGACGTCCATGGATGCCG
5D-1424230-T7-13_384	TATGCCCGGTCTCCCATGGCG
5D-1424230-T7-13_448	TTGTGCACGCAATGCCATGTC
5D-1424230-T7-13_512	CCAAAAAGATATGCAATGCAA
5D-1424230-T7-13_576	TGTGTTTGAAGATGAGCATTC
5D-1424230-T7-13_640	TTTCAAAAAAGGCTTCTGCTC
5D-1424230-T7-13_704	cacaacaccatacaTGCACCCGA
5D-1424230-T7-13_832	AATTATTATTTTCAAAAATAAT
5D-1424230-T7-13_1280	GAGAATAGTTTAACATTTTTGT
2A-0317750-T7-14_256	GCACAACAACGACCAATCCCA
2A-0317750-T7-14_384	TTTGATTTAGCTAACGCCTACG
2A-0317750-T7-14_448	TTTAGTGTGGGTTTGGTTACG
2A-0317750-T7-14_512	CTACCTACCGTCGATTTTCAG
2A-0317750-T7-14_832	GGGAGACCGGCGGTGGGGG
2A-0317750-T7-14_896	GCGGCCGAGGAAGAAGAAC
2A-0317750-T7-14_960	AATCTAGGCCGTTCATTTCTAT
2A-0317750-T7-14_1024	ACGAGATTTCAGTCGACTGG
2A-0317750-T7-14_1088	AGGAATTTTTCGAGGACGGC
2B-0432900-T7-14_832	AGTTTTATTAAATGTGTTTAAT
2B-0432900-T7-14_960	GTCAGGCCAGCGTTGGGTGC
2B-0432900-T7-14_1024	CAATTCAAACGGACAAAATCG
2B-0432900-T7-14_1088	GGAATTTTTAGAGGACGGCTT
2D-0531670-T7-14_960	AGCGAGGTATATGCAGCCTAC
2D-0531670-T7-14_1024	ATTTCGAGAAAACAAAGGCG
6A-1518320-T7-15_256	CACCTTTGGCCGCCACCTCCC
6A-1518320-T7-15_320	CTAGAAGACGGCCATGACATC
6A-1518320-T7-15_384	TTCAACTTAAGTTCTATGTGCG
6A-1518320-T7-15_448	ATTCCCGTTTCAAGCTATTTTA
6A-1518320-T7-15_512	ATTTGGGATGCGACCGCGCTC
6A-1518320-T7-15_576	TCCGTTTTACCGACCCAAATA
6A-1518320-T7-15_1152	CCATGACTAACGGCCTCACGC
6A-1518320-T7-15_1216	AAAAGGTTTTAGGCCGCCGA
6A-1518320-T7-15_1344	GCAGGCGACAGCAATGGCAA
6A-1518320-T7-15_1580	GCCCACCCGCGCCCGCGCCC
6B-1600890-T7-15_1344	CAAACTTTCTCCAGCGCAAAA
6B-1600890-T7-15_1536	TAACGGCCTCACGCCCGTGTG
6D-1719340-T7-15_832	GTTTTGTCTTCAGCTCAACCC
6D-1719340-T7-15_896	GCAAACGGACGCGCACACGT
6D-1719340-T7-15_960	TTTCCACTTGCCCCATTTAATC
6D-1719340-T7-15_1024	CGGCCTCGTCCTTTTTCACGA
6D-1719340-T7-15_1088	GAACGCATTGCTCCCTCCCTC
6D-1719340-T7-15_1280	TCTTGATCACACCTCCGGCGG
7A-1758420-T7-16_0	TTATAATACCATGGTAAATTTT
7A-1758420-T7-16_320	AGGCTGGTTACATATGTTTTCT
7A-1758420-T7-16_384	CATGATTAGGTAGAAAGAAGT
7A-1758420-T7-16_448	AGCACAACCCTTTTTTTTTGG
7A-1758420-T7-16_512	ATTTATTCAAACTTCATGACCG
7A-1758420-T7-16_576	TAGTGATGGACATGAGTAAGA
7A-1758420-T7-16_640	GCGGGGCCACAATACTGAAG
7A-1758420-T7-16_704	CAATGGTAGTTCGGTGACCCA
7A-1758420-T7-16_768	TTACCCCGCAAAAAAAAAAAT
7A-1758420-T7-16_832	AAATCTGTACAATACGTGTCAT
7A-1758420-T7-16_896	GCTTTTGCAACGTATTTGTACT
7A-1758420-T7-16_960	ACAAAAAGGCTAATGCTTTGT
7A-1758420-T7-16_1088	TCGATACTTGTGTGTACTACGT
7A-1758420-T7-16_1280	GCTGTTTGCCTCCCCGGCAAT
7B-1898970-T7-16_0	AACTAGCTAGCACCCCTGCAC
7B-1898970-T7-16_320	CGGGGGAGGCAACCGATACC
7B-1898970-T7-16_576	CGTGCCGACGTGGTATATGCC
7B-1898970-T7-16_640	CATGGCACATGATGCATGATT
7B-1898970-T7-16_704	ATCTGTATCATGGCACCCAATG

7B-1898970-T7-16_768	ATTCCATGATACCCGCAATCAA
7B-1898970-T7-16_832	TCGACAGGCCACGAAAAAGA
7B-1898970-T7-16_896	AAAGACATTTTGCAACACTATT
7B-1898970-T7-16_960	AATTATTTTTACATACATGTTCA
7B-1898970-T7-16_1024	TTTCACCCAAAAACACTTGCT
7B-1898970-T7-16_1088	TGGTCATACAAAAATAAATAA
7B-1898970-T7-16_1152	TACAAATAGCTTGTTCCTCAAA
7B-1898970-T7-16_1216	CTAGATTACTCCTTATAGTACC
7B-1898970-T7-16_1344	TTGCTGGTCCTCGGGGCCACC
7B-1898970-T7-16_1580	GCAACCGCCGGAAAATGGCT
7D-1985690-T7-16_0	AACTAACTAGCACCCCTGCAC
7D-1985690-T7-16_320	cttggctTTTCGGGGGAGGCAA
7D-1985690-T7-16_640	GATTCCGACTGGTGGGCCGG
7D-1985690-T7-16_704	AATTTCCTTTTGACCTCCATCT
7D-1985690-T7-16_768	CTCTCGAGAAAAAGGAAAAG
7D-1985690-T7-16_832	AGCCTAGTGGCTAAAAGTTTG
7D-1985690-T7-16_896	TGTACTGTACTAAATTTTCTTT
7D-1985690-T7-16_960	TTACAGGTTCAAATTGTACTTC
7D-1985690-T7-16_1024	AATTGGGTCCCTAGCTACTCC
7D-1985690-T7-16_1088	GGTGTGGCGAAACCTCTAGG
7D-1985690-T7-16_1152	GCATCTTGGCCACAAGGAAG
7D-1985690-T7-16_1216	GACGACCGACAATCGATCACA
7D-1985690-T7-16_1280	GCACCTTCCCAATATAGAACT
7D-1985690-T7-16_1344	AAGTAGAGGATGAATCCGAG
7D-1985690-T7-16_1408	GAGTCCTAAGCAGATAGTGTG
7D-1985690-T7-16_1472	ATGTTATTCCCACTGCAAATAA
7D-1985690-T7-16_1536	ATTTTCTTACATGGGCCATAAA
7D-1985690-T7-16_1580	AGTATTTACATGTTCATTTCTC
6A-1541240-T7-17_0	CAcacctcacaccacactaccacag
6A-1541240-T7-17_384	GGCAAGGCAAAGAAGACGC
6A-1541240-T7-17_512	GGTTAGTGCTAATTAATTCTCC
6A-1541240-T7-17_640	TTGCTAAAAGATCCATAGCAC
6A-1541240-T7-17_704	TGGAATTATTAAGCAAGCAGC
6A-1541240-T7-17_960	ACCTTTCCGGCTTAATGCATG
6A-1541240-T7-17_1088	CCAAATACATACGTACGTATAG
6A-1541240-T7-17_1344	TGTGAGCTTGATGAGTCTTGA
6A-1541240-T7-17_1536	TGAGCGTGTCTGGCCAAGGC
6A-1541240-T7-17_1580	GCACAACATCAAACACATAAC
6B-1655930-T7-17_384	CGATGCCGATCATTTTTATGTC
6B-1655930-T7-17_512	TAATACTCCGCTTATCGTCTTA
6B-1655930-T7-17_640	GCACCACCACCATCATGCGCC
6B-1655930-T7-17_704	CAACCCAAACTTGATGGAAA
6B-1655930-T7-17_960	GGCCCGAAATGTCAGAAAAG
6B-1655930-T7-17_1024	ACTCCCAGCCGGCTGAGCTC
6B-1655930-T7-17_1344	ATGACGAACCGGCTGCCGTC
6B-1655930-T7-17_1536	GCGCTACACATCTTGCCATCG
6B-1655930-T7-17_1580	CAGTGTGCAATGCCTAAGAAC
6D-1748960-T7-17_0	CGCACCTCACACCACAGCACC
6D-1748960-T7-17_384	GCAAAGACGCGACAACCGCT
6D-1748960-T7-17_448	CTTGTTCTGGACGGATGATCA
6D-1748960-T7-17_512	GCTAATTAGTACACCGCCTATC
6D-1748960-T7-17_640	TTCACACCACTACCACCATCAT
6D-1748960-T7-17_704	AGTAAGCAACCCAAACTTGAT
6D-1748960-T7-17_960	GCCTACCTTTCCCGCTTAATGA
6D-1748960-T7-17_1088	GACCAAATACATACGTATAGTT
6D-1748960-T7-17_1344	TGTGAGCTTGATGAGCCTTGA
6D-1748960-T7-17_1536	TGAGCTTGTTTGGCCAAGGC
7A-1833330-T7-18_0	TAGTACTTTGATGAATTGTTGT
7A-1833330-T7-18_128	TTTTGTGGAGCAGTAGGCTAT
7A-1833330-T7-18_192	ACATAGTCATCCTTTGTCACCC
7A-1833330-T7-18_512	ATATGCATGATACTACTATATGA
7A-1833330-T7-18_576	ACGAGCGTGCCTAGACTCAG
7A-1833330-T7-18_640	GTAATGATACTAGCAATCATTA
7A-1833330-T7-18_704	CTCTAAACCCTAATTAAATATA
7A-1833330-T7-18_768	CATTGGCTCTATTATCTTCGCC
7A-1833330-T7-18_832	CGAGCTATCTTAGACTAAATTA
7A-1833330-T7-18_896	CTATTTATGCGCATGCAAATAT
7A-1833330-T7-18_960	CAACCATGCATGAAAAATGAT
7A-1833330-T7-18_1024	ataatcaaataataatcaaatatagta
7A-1833330-T7-18_1088	CCAGATTAAAAAAATATAAGC
7A-1833330-T7-18_1152	TATGGAATACAGTTACTGCTAA
7A-1833330-T7-18_1216	TTCTAAAGGGTGCCTAGGTTC
7A-1833330-T7-18_1280	CCGTTTCAAAAAAACGGTGCC
7A-1833330-T7-18_1408	AGTGTGCATATTTCTTGGTGTT
7A-1833330-T7-18_1472	GTGTTATTTTTCAAAGTTTCTC
7A-1833330-T7-18_1536	ACCAATAAAAATTAAACACAC
7A-1833330-T7-18_1580	TTTTACTATTTTATTTGAATTTA
7B-1938900-T7-18_0	GCCCGTGGCTTCTGCTGGCTC
7B-1938900-T7-18_64	GGAAAATCACCGAGTGTGTG
7B-1938900-T7-18_192	GGGCGTCTACTGGCCGCGCA
7B-1938900-T7-18_384	TCCTTCTCGAACCTTCCACAC
7B-1938900-T7-18_448	GTCGACGCCCTCGCCGGCTCG
7B-1938900-T7-18_512	GTGATCTCCGGGGCATAGATA
7B-1938900-T7-18_576	AGTGGTGTTGTCTCATCGGAA
7B-1938900-T7-18_640	GACCAGGTAGTTCTTGAAATA
7B-1938900-T7-18_704	TTGTGGATGTTGTTACCTCTAC
7B-1938900-T7-18_768	GGATGTCTCTACTGATTATGTG
7B-1938900-T7-18_832	CTCCCTACTACTCCGCTAGCTT
7B-1938900-T7-18_896	ATATGAAAAGAGTAAATGAGA
7B-1938900-T7-18_960	TCCAATTTAAAGTTCTAATGCA
7B-1938900-T7-18_1024	AAAATGGGTGTTTTAGTACCT
7B-1938900-T7-18_1088	AATCTAGGAATAACTTGGATG
7B-1938900-T7-18_1152	GTTTATCAATGAAAAGGGGG
7B-1938900-T7-18_1216	GAGATGGATGATTCTACTATAA
7B-1938900-T7-18_1280	ACATAGACCTATTACCAGGAG
7B-1938900-T7-18_1344	TCCCATTCCCCTAACAGAAGT
7B-1938900-T7-18_1408	TTGTGGAAATGCAGAGGGGC
7B-1938900-T7-18_1472	ATCATTTCCGAAATTTTATTTG
7B-1938900-T7-18_1536	CAATGTTGATTCCTATTATGTG
7B-1938900-T7-18_1580	ATAGAGGTAGAGCCGGTGGT
7D-2015990-T7-18_0	GCTGAGGAGGATTTCTTGGG
7D-2015990-T7-18_448	CCTCGGCCGGCCTCCAGTGG
7D-2015990-T7-18_512	CCCTTGTTTCGATTAGTTGGA
7D-2015990-T7-18_576	ATACCTGATTCGTCTGTGTTTC
7D-2015990-T7-18_768	CCCTCGTCTTCGTGTATGTGTA
7D-2015990-T7-18_832	GTCTGCTGATGGAACAAGTCC
7D-2015990-T7-18_896	GTCTCTGTTTGTGATGTCTTTT
7D-2015990-T7-18_960	AGTCAATGTCTGGGCTCCGGG
1A-0012040-T7-19_0	TTAAAAGCATTCCTGGCTAGA
1A-0012040-T7-19_64	TTCATTTTGCATCACCTCCACC
1A-0012040-T7-19_128	ATCTACTAAGCAATAACAATCT
1A-0012040-T7-19_192	CATCATCCGGATACTTATTAGC
1A-0012040-T7-19_256	CCGGATTCCACATCAAAAGTA
1A-0012040-T7-19_320	ATCTCTTCTTCAGCTCCAGCTC
1A-0012040-T7-19_384	TCACCAAACTAGTTCTGAACT
1A-0012040-T7-19_448	CCAGATATACCACCCCCAACA
1A-0012040-T7-19_512	TGCCTCCTCTCCTGAGTATTTC
1A-0012040-T7-19_576	TTGTATTGTTTCCCATAGTCCT
1A-0012040-T7-19_640	CAAATGCTTTGTATCCTCACAC
1A-0012040-T7-19_704	TCGGTGACCTAATATGCCTTTA
1A-0012040-T7-19_768	GAAAGTTCAATAATCCCACAC
1A-0012040-T7-19_896	CATCTGTGCAGGTCTGTGAAA
1A-0012040-T7-19_1024	CTTTCCGTTTCGTTCGCACGC
1A-0012040-T7-19_1088	GCAGCCAAAAATCTTGAATTT
1A-0012040-T7-19_1152	CATTAGCATTTTCAGCTAAATT
1A-0012040-T7-19_1216	AGTTTCTATTATTACTTCACCC
1A-0012040-T7-19_1472	CTCGCTTCATCTCCTCcacacac
1B-0099330-T7-19_1216	CTTCACCCTTTCAACAAGACC
1D-0204060-T7-19_0	CTTTCAACTATCTCCAAATTCT
1D-0204060-T7-19_1216	TGCTCTTACTATAATTATACCTC

										1D-0204060-T7-19_1472	ACTCGCTTCATCTCCTCCACAC
										1A-0037970-T7-20_264	AGTTTTTTGTTAAATACTCCGC
										1A-0037970-T7-20_462	ACAAGCGTCCACGATTTGGTG
										1A-0037970-T7-20_528	CTAAAAGAGAGCGGCCAAAA
										1A-0037970-T7-20_594	GCCACGCGCATCCATCCCACG
										1A-0037970-T7-20_1150	CCCCATTCGTCCCCCTCTGCTT
										1A-0037970-T7-20_1213	cgagccgagccgagccgagccTCGT
										1B-0129000-T7-20_256	GGTTTACTCGTGACCAGCCTC
										1B-0129000-T7-20_448	AGTCCGTGCCAGCTAGCCGTG
										1B-0129000-T7-20_512	TAATGTCAGGGTTTTGCTTAAT
										1B-0129000-T7-20_704	GAAAGCGGCCAAAAACGCAA
										1B-0129000-T7-20_768	GCACCCCGAGACCGTCCAAAA
										1B-0129000-T7-20_1088	AGCTTATGTTTTTGCCCATTGT
										1B-0129000-T7-20_1152	CAAAACAATAAACAAAACAAA
										1D-0205760-T7-20_0	TCACACCACAACGGCAGCCAT
										1D-0205760-T7-20_64	ATGGGGGACGACCTGCATTAG
										1D-0205760-T7-20_256	GAGGAGTAGAATATTGGACGT
										1D-0205760-T7-20_320	TTAATTAGTACGAGTATATATAT
										1D-0205760-T7-20_384	CTTGGGCAGATGAAAAATCAA
										1D-0205760-T7-20_448	TTATTGTACGCTAATAATAAAC
										1D-0205760-T7-20_512	ACTTTGGCAAAATGTCAAGG
										1D-0205760-T7-20_704	CGCCACAAACGGGGCCTGGA
										1D-0205760-T7-20_768	CAAGGCTGGGCCTGTCAGTTT
										1D-0205760-T7-20_832	ATAGTACTAAAACGCAGTGTG
										1D-0205760-T7-20_960	TGTACAAGCGTCCACGATTTG
										1D-0205760-T7-20_1024	TCTAAAAGAAAGCGGCCAAA
										1D-0205760-T7-20_1088	TCCATCCATCCCCCGAAACCGT
										1D-0205760-T7-20_1344	ctcccTGTGCTTCACGCCACAG
										1D-0205760-T7-20_1536	GAGGACTCGGCGGGCGGGAT
										3A-0681860-T7-21_64	AATCTGATCTTGACGAGGATA
										3A-0681860-T7-21_128	TTACTTACTGGAAATTAAAGA
										3A-0681860-T7-21_192	GGTACAAGGATAGCCTTTATAT
										3A-0681860-T7-21_320	CTCTCTTCTTAAATAATATCTCA
										3A-0681860-T7-21_384	TCTTTCTCTCAACTTCATTGCT
										3B-0725290-T7-21_64	TATCTGATCTTGACGAGGATTA
										3B-0725290-T7-21_128	GAAATGTAACAAGATAAGTAG
										3B-0725290-T7-21_320	GGTGCACGATGACCTCCCATA
										3B-0725290-T7-21_448	CATCGACGGCTTCTTTTTTATT
										3B-0725290-T7-21_512	TAAAAATACTTCTTGGGCTGAT
										3B-0725290-T7-21_576	CTAAAATCATATTTATCGGAAA
										3B-0725290-T7-21_640	AAACAAACACAATGGTGTTTG
										3B-0725290-T7-21_704	TTATATTTTAGAACACGGTCAT
										3B-0725290-T7-21_768	AGGCTCCAACGCTCTATTAGT
										3B-0725290-T7-21_832	TGGCGATGGGCAGGGTCGGC
										3B-0725290-T7-21_896	TCTGTGTATACACAACTAGTTA
										3B-0725290-T7-21_960	GCTAGTTCACGAGAAGTTATA
										3B-0725290-T7-21_1024	TTTGGGATTCTTTGAATTCATC
										3B-0725290-T7-21_1088	ATGAAATAGAGCAAAAACAAT
										3B-0725290-T7-21_1152	TACGAATCATTGTTTTTCTCAA
										3B-0725290-T7-21_1216	ATCGAATGAAGAGAAACAAAT
										3B-0725290-T7-21_1266	TACTTCATCCCTAAACGTTCGC
										3D-0898770-T7-21_64	AATCTGATCTTGACGAGGATA
										3D-0898770-T7-21_128	TTACTGGAAATTAAAGAACTG
										3D-0898770-T7-21_192	CTTAGCTTACCAATGATACAAG
										3D-0898770-T7-21_256	GAGAAATGGGAAATGTTTTAC
										3D-0898770-T7-21_320	CGTAGGATGCAGCCAGCACC
										3D-0898770-T7-21_384	TCTCGCGTCTGTTCTCCATGG
										3D-0898770-T7-21_832	TGGAGACCAGCGATAGCTCC
										3D-0898770-T7-21_896	CAAAAATCTGATCTTTACGAG
										3D-0898770-T7-21_960	TGAAATGCAACAAGATAAGTA
										3D-0898770-T7-21_1024	GGATAGCCTCTATATAGGGTCT
										3D-0898770-T7-21_1088	TATTAGATAATAAATTCTTAGTA
										3D-0898770-T7-21_1152	AACTGACTGCATCGACGGCTT
										3D-0898770-T7-21_1216	TCTCCCAACTAAAAATACTTCT
										3D-0898770-T7-21_1280	TATCAAGAGCTAAAACACATAT
										3D-0898770-T7-21_1344	TGTGTCTGACAGATTGAAAGC
										3D-0898770-T7-21_1396	AACACAATGGTTCACTTCTTC
										3A-0644720-T7-22_0	GGCCAATATATGGTATGTGCG
										3A-0644720-T7-22_448	CCCGCCTACGGCACCCATGCC
										3A-0644720-T7-22_512	ACCAAATCTCCTTGCGGTAAA
										3A-0644720-T7-22_704	AGCATCATGCATGCGTCGTTC
										3A-0644720-T7-22_768	ATAGAGTCAATCCGTGGGCGA
										3A-0644720-T7-22_896	AGGGGCGCGGCATTGGGAAC
										3A-0644720-T7-22_1088	ACCATCTTCTCACCATCATAAA
										3A-0644720-T7-22_1152	TCGTACCGAGCTTTCCCATAGT
										3A-0644720-T7-22_1216	GCTAGATATATGTAGATAAAAT
										3A-0644720-T7-22_1280	TCACCTTCGCTACGTCTGCAG
										3A-0644720-T7-22_1472	ATGTCCATCTCTAGCGCATGCC
										3A-0644720-T7-22_1536	GAGAGCTGGGCCTGAACTCAT
										3A-0644720-T7-22_1580	TACGTACCCCCAGACGGAGAT
										3B-0772640-T7-22_0	GGCTGTGCTGAGCTACCAAAT
										3B-0772640-T7-22_448	TCAGACGTGTCCCAAGGAGAT
										3B-0772640-T7-22_512	CCCTGCCGTATCTCATCTCACG
										3B-0772640-T7-22_704	CGCGTTAGTTAGGCCAGCATC
										3B-0772640-T7-22_768	TACTAGATCGGTAGATCCCATT
										3B-0772640-T7-22_1088	ATCGGAAGCACAGATGGCGA
										3B-0772640-T7-22_1152	TGCACGTCGACCATCTTCTCAT
										3B-0772640-T7-22_1216	CTGGTCAATGCATCGTACCGA
										3B-0772640-T7-22_1280	TGTACTCCCGCTGCTAGATATA
										3B-0772640-T7-22_1344	AGGTTGATAAGTTCACATTCG
										3B-0772640-T7-22_1472	ATGCAAATTAGTCCTAAACTG
										3D-0870310-T7-22_0	GGCTGGGCTGCCAAATATGGT
										3D-0870310-T7-22_448	GTACGGCACCCATGCCGCCAG
										3D-0870310-T7-22_512	ATCTCCTTGCGGTAAAATCTCG
										3D-0870310-T7-22_576	TCGATCGTTGCTACTGCTCCAT
										3D-0870310-T7-22_640	AATGCGCTCGATTAGATCACG
										3D-0870310-T7-22_704	ATGCATGCGTCGTTTATTCCCT
										3D-0870310-T7-22_768	TCAATCTGTGGGCGAGTAAAG
										3D-0870310-T7-22_896	TTGGGAACTAGGAAATCCGC
										3D-0870310-T7-22_1088	ACATTGGGTCACCTGCACGTC
										3D-0870310-T7-22_1152	ACTGCTACTGCTGGTCAATGC
										3D-0870310-T7-22_1216	TTAAAGATGCTGTACTCCTGCT
										3D-0870310-T7-22_1280	TATTTTCTGCAGCAGGTTGATA
										3D-0870310-T7-22_1472	AGGAAAACTGTCGTTGACCA
										3D-0870310-T7-22_1536	CGATCACTCACAGGAGCCGAT
										3D-0870310-T7-22_1580	TATGTGGATAACTACGTGCCC
										1A-0006730-T7-23_384	GACCGACGGGCGGTGTTCCT
										1A-0006730-T7-23_448	AGCCTGTTGCCAAGGGCttcct
										1A-0006730-T7-23_512	TCCAACCCATATTCTCAAACTG
										1A-0006730-T7-23_576	TCGGGTTTGTATCAGCCTGCC
										1B-0138950-T7-23_0	TGCCAAGAAAcagagcagagctg
										1B-0138950-T7-23_576	TGGACCGACGGACGGTATTC
										1B-0138950-T7-23_1152	CTGGGCCCGGCCCGGCCCGA
										1B-0138950-T7-23_1216	TCCAATCACAGGTTGGTTCGC
										1B-0138950-T7-23_1280	ATCCCCGAAAATTCGCCCGGG
										1B-0138950-T7-23_1344	TATTTGGTGTTTATTAGGATGT
										1B-0138950-T7-23_1408	gggtgcctcttggtgggtttattgtggt
										1B-0138950-T7-23_1472	aaataatttctctttatccatgcagttca
										1B-0138950-T7-23_1536	atatatatgggctttgtcaccatcacga
										1B-0138950-T7-23_1580	gaagttttcaaacattcaaatgcccga
										1D-0204240-T7-23_0	TGCCAAGAAAcagagcagagctg
										1D-0204240-T7-23_64	GAACCGAACAAGCAGGGCCG

1D-0204240-T7-23_576	ACCGACGGACGGTGTTCCTGT
1D-0204240-T7-23_640	GCCTGTCGTCAAGGGCttcctat
1D-0204240-T7-23_704	CAACCCGAACCCTCAAACCGC
1D-0204240-T7-23_768	TGTCATCCAACGCAAGTTTGT
3A-0701660-T7-24_0	GGCCCACCAAACCGGCTGCC
3A-0701660-T7-24_64	GACAGTAGGCCACCCTCCTCC
3A-0701660-T7-24_128	TGTTGGCCCTCACCGAGGAG
3A-0701660-T7-24_256	CTGCAGCCGGCAAAACGAGC
3A-0701660-T7-24_320	ACCATTGCGGCTGATGCTTATT
3A-0701660-T7-24_512	GTAGTGATACTTTTTGGTTGA
3A-0701660-T7-24_576	TTGCTAAAGGTTTATATTTATG
3A-0701660-T7-24_640	GTTCGGGATTCATGTCGTTGG
3A-0701660-T7-24_704	GATCAGGTAGGTCTGGCCACC
3A-0701660-T7-24_768	TTACTCCTCTATTTTTTAAAGG
3A-0701660-T7-24_832	ATCATCCTATCCTCTCAGCACT
3A-0701660-T7-24_1024	ACGCAGCCCATTTCTATTTTTG
3A-0701660-T7-24_1088	AAGCAAGCGCTGCAAAAGTG
3A-0701660-T7-24_1152	CGGCATGCAATTCGCGGAATT
3A-0701660-T7-24_1216	GGTATCTTGCCTGGCTACACA
3A-0701660-T7-24_1280	CCACATGGAGGTAACTTGCAA
3A-0701660-T7-24_1344	GCTGAAAATGACCTTCATCTT
3A-0701660-T7-24_1408	AAGTGTAACATGATGGAGTTT
3A-0701660-T7-24_1472	GGCCCAGATCAATCGAATGCA
3A-0701660-T7-24_1536	GCTGTACTTGCACCAACCCAG
3B-0782440-T7-24_0	GGTCGGTCGGCTTCTTCTTCC
3B-0782440-T7-24_192	CGCGCTCTGCCTGTGCACCTG
3B-0782440-T7-24_256	ATGGCACGGATCGCTAGTGTC
3B-0782440-T7-24_320	TGTCCGTGCCGGTTTAATCCC
3B-0782440-T7-24_384	TACGTCAGTGATGACACATGG
3B-0782440-T7-24_448	GCTGGCGACACAGAAAATCTT
3B-0782440-T7-24_512	AGAGCCTTCAGCCTTCAGAG
3B-0782440-T7-24_576	GATATTGCTTACAAGTTACCAC
3B-0782440-T7-24_640	AATGCTCTTGAATAGCCAGGC
3B-0782440-T7-24_704	ATGGACCAATAATGCCGTGGA
3B-0782440-T7-24_896	ATCCTCGCCAGGTGCAAGAGA
3B-0782440-T7-24_960	CGAACCCAAGAACAATAATCT
3B-0782440-T7-24_1088	CCAATAGCTCTTTTTAATCACG
3B-0782440-T7-24_1152	CAGATCCACAACCCTCTATTCA
3B-0782440-T7-24_1216	CTATTATCCGTGTTTTGAAACG
3B-0782440-T7-24_1280	ATGAATGAAATCAGAATGCTG
3B-0782440-T7-24_1344	GTAAGATCCAAGTTACACTTA
3B-0782440-T7-24_1408	ACCTTGGTCATCTTTTGACAA
3B-0782440-T7-24_1472	ATAATTTTTCTGTTTTTTTGGA
3B-0782440-T7-24_1536	TTGAAACCATTTCCTTTAAAGA
3B-0782440-T7-24_1580	CAAGGGGTTCATGCTAATAAT
3D-0928740-T7-24_126	aagcagcggcggcgATGGGGAG
3D-0928740-T7-24_189	GAGAAAGAGCACAGGGACCC
3D-0928740-T7-24_252	TAGGACCGACATGTGGGCTCC
3D-0928740-T7-24_441	AAAACGAAAGCCTGTAATGTG
3D-0928740-T7-24_630	CGGACTTATTATCCTGGTGGT
3D-0928740-T7-24_693	GAGTACATTGTACCACCACGC
3D-0928740-T7-24_756	CAGTGTTTTTAAGGCCCGTAG
3D-0928740-T7-24_819	AAAAAGACCCGTATACGTCGT
3D-0928740-T7-24_882	TGATTGTCAAAAGATGACCGA
3D-0928740-T7-24_945	TCTGACCGAGCTAAGTTTTCT
3D-0928740-T7-24_1008	GCCTACGTACAGAAGTACTCA
3D-0928740-T7-24_1071	TGCCGCGATGAGCAAAACGG
3D-0928740-T7-24_1134	CGATCTAACGGCCCATATCAA
2A-0318130-T7-25_128	GCGTAGCGGCAGAGCCATAC
2A-0318130-T7-25_256	CGATGGACCGATCAACAGCTG
2A-0318130-T7-25_320	AAAAAAGGAGTATCCTTATAC
2A-0318130-T7-25_384	atatatatatactttattatgtaaataaa
2A-0318130-T7-25_448	TAAACATCCTTTGGATAATCAT
2A-0318130-T7-25_512	GACATTTCCACATTAACTTACA
2A-0318130-T7-25_768	AGGCCTGAGCCCAGCCTGAA
2A-0318130-T7-25_832	gataacataacaaatataattatgaca
2A-0318130-T7-25_896	ATTGTTGTACATACGTGTTGA
2A-0318130-T7-25_960	AAAATAAATACTAGCAAGCAA
2A-0318130-T7-25_1024	TTCATGATTGTAAAAAGATTA
2A-0318130-T7-25_1088	ATGGAAAATAAATAATCACTC
2A-0318130-T7-25_1152	ATAACCGAAAGGCATATCTTCT
2A-0318130-T7-25_1344	TACACGAGTAATACAAAATTC
2A-0318130-T7-25_1408	TAGATCGATCTACCCACGCCCT
2A-0318130-T7-25_1472	TCTCAATGGGTAAATCGCTTCT
2A-0318130-T7-25_1536	AGCTTCCAATGCTTCTTGACA
2A-0318130-T7-25_1580	AGAAAAAATGATAGCCCCCTT
2B-0442410-T7-25_0	GGCGAGCGATGCGGAACCAC
2B-0442410-T7-25_64	GGAAAGGAAAGTGTGAGGA
2B-0442410-T7-25_128	CTTGCAGCGTAGGAGTAAAG
2B-0442410-T7-25_192	TTTCACGTAGGAGTAATCTGT
2B-0442410-T7-25_256	GTCGATGGACCGATCAACAG
2B-0442410-T7-25_320	AACACGTCTAGAGACAGAAA
2B-0442410-T7-25_1216	gataatatgtggggttggagagaagct
2B-0442410-T7-25_1408	gccccatatcggtcatggaaaactcag
2D-0552010-T7-25_128	GCGTAGCGGCAGAGCCATAC
2D-0552010-T7-25_192	AGGAGAGTAATCTTCTGTACT
2D-0552010-T7-25_256	GGACCAATCAACAGCTGTGCC
2D-0552010-T7-25_448	tatatatatatatatgtatgtataAT
2D-0552010-T7-25_512	ATAAATACTATAAAAGGTGGAT
2D-0552010-T7-25_576	ATATTTCCTTTTTCAAAATATA
2D-0552010-T7-25_640	CTAAAAATAGACGCGATATAC
3A-0692510-T7-26_0	TAGGTTTAACATATTGGACAA
3A-0692510-T7-26_64	GAAACTATGTTTGCTTCCCTCT
3A-0692510-T7-26_128	CCAAAACTTTCTTTTTAAAAC
3A-0692510-T7-26_256	CCCTTTAGCATGCTTGTAGCT
3A-0692510-T7-26_320	GCCTTATATGCAACGTCAAAAT
3A-0692510-T7-26_512	AATAGTCCACTATATTAGATGA
3A-0692510-T7-26_576	ATGGATCACCTACACACACAA
3A-0692510-T7-26_640	TCAATGAGATTTAGTTTTATGG
3A-0692510-T7-26_704	CATGGTTTATTTTATAACTCTAT
3A-0692510-T7-26_768	CTTTTGCCACGCACAGTTGCA
3A-0692510-T7-26_832	AAATTTAATTATCCGTGGAGAT
3A-0692510-T7-26_960	ATGATTGATATTGAACCACTG
3A-0692510-T7-26_1088	ATGCTAGGTACATCCATTTGAG
3A-0692510-T7-26_1152	ACAGACCTTCTACCCGTGGAC
3A-0692510-T7-26_1344	GCGCTCCACATCCCTCCAAAA
3A-0692510-T7-26_1408	ACAGGCAATGCATATTCATCTT
3B-0715770-T7-26_192	AATTGGTTCGGGTGCAACTAT
3B-0715770-T7-26_256	CATCAAATAACCTCCAAAACT
3B-0715770-T7-26_320	TGTGATGCACACAGAGTAGTT
3B-0715770-T7-26_384	ATGCCCCTTTTAGCATGCTTGT
3B-0715770-T7-26_448	TACTTTCGGCCTTATATGCAAC
3B-0715770-T7-26_512	ACAGGGGTTGCTACCGCACC
3B-0715770-T7-26_576	CTCTGGATTAGTTTGAATTGCT
3B-0715770-T7-26_704	GGATCACCTACACACACAAAC
3B-0715770-T7-26_768	AATGAGATTTGGTTTTATGAA
3B-0715770-T7-26_832	TGGTTTATTTGAATATAATTAG
3B-0715770-T7-26_896	TGAGGTGTGCTTTTTATTATGT
3B-0715770-T7-26_1024	GATTTACGTCGGACATGATTG
3B-0715770-T7-26_1088	AATTAGCTAGTACTCCTCCGAg
3B-0715770-T7-26_1152	atatAGAACGAACATATCTTCTA
3B-0715770-T7-26_1344	GGTGGCCTTGTGCGCTCCACA
3B-0715770-T7-26_1408	TCTTCATCCCAACAGGCAATG
3D-0902420-T7-26_128	CATGCACAACCCCGATGACAA
3D-0902420-T7-26_192	GATTTTCACTAGATCAGCCTTA
3D-0902420-T7-26_256	AAAACGGAGGACTTGCGAGG
3D-0902420-T7-26_320	GAAAGTAATGGTCATATGCAA
3D-0902420-T7-26_384	GCCTCCTCCACACGGGAGAA
3D-0902420-T7-26_448	CAGCATTCATGTTTTTCTTTAC
3D-0902420-T7-26_576	ctctctctctcATATCAACAGTAGT
3D-0902420-T7-26_704	GAAATTGGTTGCAAAACTTTC
3D-0902420-T7-26_768	AATTGAAAATTAAAATACACAT
3D-0902420-T7-26_832	GCATAAGAGAATGAATAGACT
3D-0902420-T7-26_896	TATAGTTGCATGTTGAGAGAA
3D-0902420-T7-26_1024	TAAAATTTACGTCGAGCATGAT
3D-0902420-T7-26_1088	CAATTAGCTAGTACTCCCTTCG

3D-0902420-T7-26_1152	ATATAGAATGAACGGATCTTCT
3D-0902420-T7-26_1344	GTGGCCTTGTGCGCTCCACAT
3D-0902420-T7-26_1408	TTCATCCCAACAGGCAAATGC
4A-0938430-T7-27_94	ggaggaggaggaggaTGGTGT
4A-0938430-T7-27_692	CTCTCTCTCtgtgtgtgtgtgtgt
4A-0938430-T7-27_756	TTTGGTGAGAGGGAGGCGC
4A-0938430-T7-27_884	CACAGTGAAACATGTGGGGG
4A-0938430-T7-27_948	TCCACTATTTTCGCGCAGCGA
4A-0938430-T7-27_1012	TACCTGGTTGTATGGATCAGT
4A-0938430-T7-27_1076	GTCCTGTATTTTTTGCACATCT
4A-0938430-T7-27_1140	AATAGCTCTAGGGAAAATAAT
4A-0938430-T7-27_1204	GGCCGTTTAACATTACGTCCT
4A-0938430-T7-27_1268	GGACCAGTCCACAGACACTG
4A-0938430-T7-27_1332	ATGCCTTTGCATGTCGCGGTT
4A-0938430-T7-27_1396	TGGCTAGACGGAGGTCCAGA
4A-0938430-T7-27_1460	TTTGCCAGAATTTGAATGTTCT
4A-0938430-T7-27_1524	GTGGGGACTCCCGCAAAGAA
4A-0938430-T7-27_1580	CCCCAACGTTGCAACTTTATTG
5B-1314550-T7-27_0	ACAGAACCTGCATATGTTTTA
5B-1314550-T7-27_64	GGTGATGAGCAAGAAGTCATA
5B-1314550-T7-27_192	CATATATGTACGAGCACCGATT
5B-1314550-T7-27_256	TTACCTTTTGTGCAGACGTTAT
5B-1314550-T7-27_320	CAGAGGGTTATGCTTCTCCTA
5B-1314550-T7-27_384	TGACAATGCTGTACACTAGGC
5B-1314550-T7-27_448	ATCCCAATAAAGTTCCCCAGC
5B-1314550-T7-27_512	ATCTTTAACACGGACCCTCAA
5B-1314550-T7-27_576	CTTCTCCGTACGTCCAAATTC
5B-1314550-T7-27_640	TGGAGGAGTCCGGACATCTGT
5B-1314550-T7-27_704	GTCTCTACTCAACCGTGACAT
5B-1314550-T7-27_768	GGCTACAACATCAGTGTCTGC
5B-1314550-T7-27_960	CATCTCTTTTTATTCATTTTGGT
5B-1314550-T7-27_1024	TCATATTAATTTTTATAGGAAA
5B-1314550-T7-27_1088	GACATTTATGTTTAATTTGCAT
5B-1314550-T7-27_1152	GCAGGTAAAATGGACATAGG
5B-1314550-T7-27_1216	ATAGTGAAAACAATGTAGAAA
5B-1314550-T7-27_1280	TGTGCGAAAATAGCACCACCC
5B-1314550-T7-27_1344	TGGCACAGCAGAGAGCAAAA
5B-1314550-T7-27_1536	ctcctcctcctcctcctcctcctcct
5B-1314550-T7-27_1580	GAGTCGGCACTGCACTCTCTC
5D-1414740-T7-27_0	AATAAGAGATTCAACAGGTGT
5D-1414740-T7-27_65	GATCACCACTTGTTTAGCCATA
5D-1414740-T7-27_130	AAAGAAAAAAATAAACAGATTA
5D-1414740-T7-27_195	CTCGGCGTTCCACGACTAGTG
5D-1414740-T7-27_260	AATGTTGTTTTGTTACGCTAAA
5D-1414740-T7-27_325	TTTCGTGACGTTGGAAGAGT
5D-1414740-T7-27_390	ATCGTCCATAAATTCTTTGTGG
5D-1414740-T7-27_455	CATTTCTGGACGTCCCAATTC
5D-1414740-T7-27_520	CCGGACATCCATCTAGCGAAC
5D-1414740-T7-27_585	CCGTGACATGCAAAGGCATAG
5D-1414740-T7-27_650	GTGTCCGGACCAACCTGAGA
5D-1414740-T7-27_715	TAACGTTGAATGTCCGATTAA
5D-1414740-T7-27_780	TTTCCCTAGAGCTATTAAAATG
5D-1414740-T7-27_845	ATTAGCATGTGCAAAAAATAG
5D-1414740-T7-27_910	GAGCCAGGTAAAATGGACATA
5D-1414740-T7-27_1040	GTGCCAAAATAGCACTACACT
5D-1414740-T7-27_1090	ATGAATGTGGCAGAGAGCAAT
5D-1414740-T7-27_1530	GGCAGACACCCAACTTGTCA
5D-1414740-T7-27_1580	TCCAGTGAGCTCGAGTCGGC
3A-0639020-T7-28_0	CTTCGCTGGCTCCCTCTTCTCA
3A-0639020-T7-28_64	TCTTGAATAATCACCGAACTA
3A-0639020-T7-28_192	TTTCCAGCTCGATCAAGCTTCT
3A-0639020-T7-28_384	CAAGTAATTCCGAACGGAGA
3A-0639020-T7-28_448	AATCCACCTTTTGGTACTAGTA
3A-0639020-T7-28_512	TTGCCTCTATGCGGAGCGATT
3A-0639020-T7-28_576	CCTGTGAGCGATCACTGCTTC
3A-0639020-T7-28_640	GGGAGCATGCTTTTCGCTTAA
3A-0639020-T7-28_704	TATGAAACCGAGGTAGTACAT
3A-0639020-T7-28_1024	AGTTCATTTAAAATCTCTAAAA
3A-0639020-T7-28_1088	AAACATGTGTGTCTTTGGTAG
3A-0639020-T7-28_1216	ATGAGGGTTTCATTGTTGGCG
3A-0639020-T7-28_1280	ATATATTGTATCCCCCTAAAAA
3A-0639020-T7-28_1344	TATCTTTGTATGTCAAAAGAAA
3A-0639020-T7-28_1408	TTTCCATGGATATTGGATAGAG
3A-0639020-T7-28_1472	CGCCCTCCCGGGACACGTGC
3B-0741640-T7-28_0	TTTGTTCGAATACAGGAACAT
3B-0741640-T7-28_64	GGGATATATAGAAAAAAAAAC
3B-0741640-T7-28_128	GTAAACCACTTGCCTAGGGG
3B-0741640-T7-28_320	GTTCACTCTTATGTATTCAATA
3B-0741640-T7-28_448	TCCAGCTCGATCAAGCTTTTC
3B-0741640-T7-28_512	TGCAAACAGTAGCAAAAGCT
3B-0741640-T7-28_704	GGTCTGGCCCATTTATCAGAT
3B-0741640-T7-28_768	CAACACAAGTGATGGGAGCAT
3B-0741640-T7-28_832	TCAGTGTCACTTAATATAAAAC
3B-0741640-T7-28_896	TAGGATTTAGCCAAGTAAAAA
3B-0741640-T7-28_1088	GGCGTCTAAGGTTTTCTATGC
3B-0741640-T7-28_1344	AGACTTAGTTTTAGAAACGGG
3B-0741640-T7-28_1408	TTCCATGGATATTGGATAGAG
3B-0741640-T7-28_1580	TCCACCCGCAAAGCACCAAC
3D-0843900-T7-28_1152	ttattgctctgatctgaccagaaccaaa
3D-0843900-T7-28_1536	GGCCAGAACCAAAGCCTTGT
7A-1758330-T7-29_0	CCTGGCCGCGGCAATGCACGT
7A-1758330-T7-29_64	gatgagtgttaagggtgatgattggttg
7A-1758330-T7-29_128	GGTGAGGGGTGGCAGCTAAC
7A-1758330-T7-29_320	GGCTTTGTGAACGGGTGGTG
7A-1758330-T7-29_384	AAGATGCCGCCCCGCAACCA
7A-1758330-T7-29_512	GCGCGTCGACGATCTGCGCG
7A-1758330-T7-29_640	ATGGTTATGCTGCGTGCGGGG
7A-1758330-T7-29_768	CGGGGCCGTGCTCTGGGCCG
7A-1758330-T7-29_832	AGTGCCGCTCGGAGGGGGA
7A-1758330-T7-29_896	GGCGGGGGATCGATGGCTGG
7A-1758330-T7-29_1088	TCCGGTCCGGCGGGCCGCCG
7A-1758330-T7-29_1152	CCTAATAATTTTCTGAAAGCTG
7A-1758330-T7-29_1216	TTGCTACGTGTCGACCGGCGG
7A-1758330-T7-29_1280	GTTTTCTCGTGACTCAATGTTG
7B-1885950-T7-29_448	AGATCTGTTCCGCCCAAATGA
7B-1885950-T7-29_576	tcagtatttatcctacatgacacttttaa
7B-1885950-T7-29_640	TAATTTAGACGAATGAATACC
7B-1885950-T7-29_704	CACGTggcgggcggcggcgggcgg
7B-1885950-T7-29_768	CCACGGAACGAGGCGGAAAA
7B-1885950-T7-29_832	CCCCGCTGACGGATAAGGCAT
7B-1885950-T7-29_896	TGGGAAAGACCAGAGCTTTC
7B-1885950-T7-29_1088	GCCACGCCCAAGGTACCAAC
7B-1885950-T7-29_1152	TCGTTCCGTCTCGTCTCGTCAT
7B-1885950-T7-29_1216	ATTACCCCGCAGGCGACGCG
7B-1885950-T7-29_1280	AAAACGAGGAATCCGCCGAA
7B-1885950-T7-29_1472	GAATCATTCGCTTTCTCCCACC
7B-1885950-T7-29_1536	GCACCGCACACACGCATCCCT
7B-1885950-T7-29_1580	TCCGTTGGCTGCCGCCCCCTC
7D-1962550-T7-29_128	CTGCAGGTACATCGAAACACA
7D-1962550-T7-29_256	CTTTACGTGATTGTAAAATTAA
7D-1962550-T7-29_320	GGCTAGGATTTTATTAGCTGAT
7D-1962550-T7-29_384	AAGAACAAAAGTATGTCGAG
7D-1962550-T7-29_448	TTTTCTATAGTTCATGCTTTGG
7D-1962550-T7-29_512	TTTAGGTTTGGATTCCTTCTCT
7D-1962550-T7-29_576	ATTCTTTTCTCACCTACTAGTT
7D-1962550-T7-29_640	TGTATACCTTTTTATCATGTTTC
7D-1962550-T7-29_704	AATGGCATGCCGTGTCGTTTT
7D-1962550-T7-29_768	CTCGACTACTAATTAATGTGCT
7D-1962550-T7-29_832	GATAGCAAAACAACAGACAG
7D-1962550-T7-29_896	TATACGGCGCTTGCCCGGTGC

7D-1962550-T7-29_1088	TGGCATCTCCTTTGCCGCTGA
7D-1962550-T7-29_1152	AACCTCACGACAAGGGCCTAT
7D-1962550-T7-29_1216	GGTGCATCCACATAATTAATCG
7D-1962550-T7-29_1408	GGCCGCGGCAATGCACGTTC
7D-1962550-T7-29_1472	GACGAGCCGACAAGtgttgagg
7D-1962550-T7-29_1536	AAAGAAGGGGTGAGGGGGC
7D-1962550-T7-29_1580	AGAATGCACAATGGATGTATG
2A-0312750-T7-30_256	CCCGCAGATAAGTTCCTCTTTA
2A-0312750-T7-30_320	TTTACGAAGGGAATATTACTT
2A-0312750-T7-30_448	CACCAACGAATGGGATCTCGT
2A-0312750-T7-30_896	TTATTATTTTTGTTGTTATTTAT
2A-0312750-T7-30_960	AAGAAAAGACGGCCATAATCT
2A-0312750-T7-30_1024	CTCAGGGCCGGCAGACGCGC
2A-0312750-T7-30_1088	AGAGCGCGATTTAATTGTGGA
2A-0312750-T7-30_1152	ATTTACAGAGCAATTTCGGTG
2A-0312750-T7-30_1216	AGCATCACATCTTCGAGGGCT
2A-0312750-T7-30_1280	TATGCTACTAATTTATATTACCA
2A-0312750-T7-30_1344	GGAGGCATGTTCAGGGTGTA
2A-0312750-T7-30_1408	ATGAGAGGTCGTGTCCGTCCT
2A-0312750-T7-30_1472	GGCGTGCACTGGTAAGGGTT
2A-0312750-T7-30_1536	GACGTCTGTAATGACGCCCCC
2A-0312750-T7-30_1580	GCACATATTTTATCTTCGGTTT
2B-0416140-T7-30_384	ATTTATTTTTTGGACGAGTAAT
2B-0416140-T7-30_448	AGTGCAATCTCTAAAAAAAAC
2B-0416140-T7-30_512	GCAATCCAAACAATTCAAAAA
2B-0416140-T7-30_640	GGGATCCCGTTGTCGTTCTTG
2B-0416140-T7-30_1216	TACCTCTGTTGTTCTTTGTAAG
2B-0416140-T7-30_1280	CAACTTTTACGAATTGGAATA
2D-0518570-T7-30_64	ATCGCCGGAATAGGAAAGCA
7A-1763880-T7-31_192	GCCGACGTGGGTGGATTTGG
7A-1763880-T7-31_512	CCATTGTCCACCTAACCAACT
7A-1763880-T7-31_576	CCTGGCGACCTTTTTTTTGGC
7A-1763880-T7-31_704	CGAACGCACTCGTAGCCGTTG
7A-1763880-T7-31_768	GTCTTCTTTAGGAATAAATTG
7A-1763880-T7-31_832	GCCATTGAATCGTTAGTAAATA
7A-1763880-T7-31_896	TAAGCCCCCCTGGGAACAGA
7A-1763880-T7-31_960	GTCTATGTCAGGTCGCGTCAT
7A-1763880-T7-31_1024	CAAACGGAGTGTACCGCGTG
7B-1873370-T7-31_64	ACGTGGCCAATTCAGCTGCGA
7B-1873370-T7-31_704	AAGAGTAACTTTCCAAGATTT
7B-1873370-T7-31_768	ACTCCCAATCCTGGTCGTTGG
7B-1873370-T7-31_832	CGTTTGGTGCACCCGAAAAG
7B-1873370-T7-31_896	CCGGCTAGCCTAAAAAATAGT
7B-1873370-T7-31_960	ACGTTCGTAAATTAAAAAACT
7B-1873370-T7-31_1024	AGCTTAGAACAATGTCTGTGA
7B-1873370-T7-31_1088	TATATTCTCAAATACGAAAAAT
7B-1873370-T7-31_1152	TTGAGAAAATACTCGGAAATA
7B-1873370-T7-31_1216	ATGTTCGGAAACAAAATATATT
7B-1873370-T7-31_1280	AAACAGATTTTTTTGGCAAAT
7B-1873370-T7-31_1344	AATCTTCAGGAGTTTGGAAAA
7B-1873370-T7-31_1408	GGAAATAAAATATATTCTCAAA
7B-1873370-T7-31_1472	TGATTTATTTATATATTTTTGCA
7B-1873370-T7-31_1536	TGAATTCTTTTCAGGAGTTTG
7B-1873370-T7-31_1580	AAAATTCAGAAATAAAATACAT
7D-2113450-T7-31_64	ACGATGGTTGTGGTGGCGGT
7D-2113450-T7-31_128	GTAAGTGCCTCACTTTCAGAT
7D-2113450-T7-31_256	GTAGGGTGAAACATATTTGTC
7D-2113450-T7-31_320	ATTTTTTAAATTGATGAACACT
7D-2113450-T7-31_448	GGAGTTCATGAACTTTTTTCA
7D-2113450-T7-31_512	GGACATTTTTGCAAACTCCTG
7D-2113450-T7-31_576	CCAAAAAATAGTTCGTGAACA
7D-2113450-T7-31_640	TTATGTCGGAACATTTTTTAAG
7D-2113450-T7-31_704	TGAGAATGTTTTTATTTCCAAG
7D-2113450-T7-31_768	TTTTCCGAATTCCTGAAAAAAT
7D-2113450-T7-31_832	ACTCCTGAAAAATTGCCAAAT
7D-2113450-T7-31_896	AACAGTTTGCGAACATTTTTC
7D-2113450-T7-31_960	ATGAATATTTTCTATATTTGCG
7D-2113450-T7-31_1024	TTTTTCGTATTTGAGAATATTT
7D-2113450-T7-31_1088	CATGCACATTTATTCAAATCCT
7D-2113450-T7-31_1152	GGAGCGACATGAGAGATGTA
7D-2113450-T7-31_1216	TTCGATTTGGACCGGGAATGA
7D-2113450-T7-31_1280	GATTTAGCTAACCTCTACAATC
7D-2113450-T7-31_1344	ATGGACCTTTTCCTAAATAAAA
7D-2113450-T7-31_1408	ATTATATAAGTCCCTAAAAAAA
7A-1851630-T7-32_509	AGAGGTCCTGATCTGCAGGC
7A-1851630-T7-32_572	AGGAGATGTGTGCTTAGGTG
7A-1851630-T7-32_635	AACGTGACAGTCTTCATCGGC
7A-1851630-T7-32_824	AGTGTAGAAATAATATTCTCCC
7A-1851630-T7-32_950	TCAACCGGCTATTGTGGTCTT
7A-1851630-T7-32_1013	GGCGACAATTTGCTTCGCAAA
7A-1851630-T7-32_1076	AGACGCTGCTCCTATAAGCTC
7A-1851630-T7-32_1139	GGCGGCATTGAGCTCCGCCAT
7A-1851630-T7-32_1202	CCTCAAGGATTTGAATGTAATT
7A-1851630-T7-32_1265	ACACGTGATGCTTTATAAATG
7A-1851630-T7-32_1328	CTAAGAAAATCAGGGAGGTT
7A-1851630-T7-32_1391	TAATAACGGTGAAAAAGAAA
7A-1851630-T7-32_1454	ATGCTAAAAAAAGATTAGCAC
7A-1851630-T7-32_1517	TATTTTAACCACGTGCACACG
7D-2055390-T7-32_320	CAAGTCCGGCAGGCCGGACT
7D-2055390-T7-32_448	CATGCGCTTTGGTGCgggccgg
7D-2055390-T7-32_512	GATTCCAAGCTGGACCCGGA
7D-2055390-T7-32_576	CATTTTGTTTCTGGTTTTAACT
7D-2055390-T7-32_640	CATGCAAGATACCCTCTCCTAG
7D-2055390-T7-32_704	aGTGACCAATTTTTTTTGTTAT
7D-2055390-T7-32_832	TGTTGTTGGTGAAAGGATGG
7D-2055390-T7-32_896	CACTAAGATGCTCCTCCTTTTG
7D-2055390-T7-32_960	TAATATAAGATGTTATTACGAC
7D-2055390-T7-32_1152	TGCTTGTTCGCATCTTCCCTCT
7D-2055390-T7-32_1216	TGATGTATCTGTTTTTGTAATCT
7D-2055390-T7-32_1344	AAAACCCCCTTTCAGTTTGCT
7D-2055390-T7-32_1408	TTTTGGCTGCAATTGCTGTCG
7D-2055390-T7-32_1472	TGGTCATCAGTCTGATATGAA
7D-2055390-T7-32_1536	AATATCCACCACTGTCTACTCT
4A-0978250-T7-33_128	CGGGGGGTGGGGGAGGAAG
4A-0978250-T7-33_192	cgagcggcagcgggagcgagTAGT
4A-0978250-T7-33_384	CACCAGACAAACTTCGCCATC
4A-0978250-T7-33_512	GCCGTGTGGCCCTCTCTGTGG
4A-0978250-T7-33_576	TATTGCACTGTTGAGGGCCTC
4A-0978250-T7-33_640	GTGCTGGTCGTTTAGTTTGTA
4A-0978250-T7-33_704	TCATTCCAAGGAATTCTAGTGT
4A-0978250-T7-33_768	TGCAACCAAACCAAAAACCGT
4A-0978250-T7-33_832	GTGTTTCTAGAATCATGTGAAT
4A-0978250-T7-33_896	CCAACATTTTGACCTATCTACA
4A-0978250-T7-33_960	CGGTTATGATACGGGTCAAGC
4A-0978250-T7-33_1024	ACAAAACGATGAATAGAAGG
4A-0978250-T7-33_1088	AATATTCACCACCAATATCCAC
4A-0978250-T7-33_1152	GCAAACTAGAGAAAGCAAAT
4A-0978250-T7-33_1216	CCATGGGATGCTCCGCGTCAG
4A-0978250-T7-33_1280	ATTGACCATATCTTGTTAAATA
4A-0978250-T7-33_1344	CAGATCATTGATCAATGTGATC
4A-0978250-T7-33_1408	AGGACAAATTATCATCGACTC
4A-0978250-T7-33_1472	AAGACACACTATCTCTTGGTGT
4A-0978250-T7-33_1536	TTGATTAAAATTAATATTGAAA
4A-0978250-T7-33_1580	CTAGTACCACAACATGTATAAT
4B-1095100-T7-33_128	GTCTGGTGGCCCACGGCAACT
4B-1095100-T7-33_320	TGGCTTTTGACAAATGTGTAC
4B-1095100-T7-33_384	GAGAAGATAAGACAATTATAT
4B-1095100-T7-33_448	GTTAATTTTAATCATCCCACAT
4B-1095100-T7-33_512	GAGATAGCGTGTCATTTTTAAT
4B-1095100-T7-33_576	ATGATAATTTTTCCTCCAAATA

										4B-1095100-T7-33_640	TTGATCAATGATATGCATGTGA
										4B-1095100-T7-33_704	CAAGGTATGATCAATGACAAA
										4B-1095100-T7-33_768	AGGAGCATCCCACGGTCATTA
										4B-1095100-T7-33_832	CTTTCTCTTCTTTGCTTGTTGG
										4B-1095100-T7-33_896	TTGATGGTGAACATTGCTGAC
										4B-1095100-T7-33_960	TCGTCGTGCTCTTACTCGAAT
										4B-1095100-T7-33_1024	TATCAGAACCGTGTGTCGCAT
										4B-1095100-T7-33_1088	TTGAAATGTTGGTTGTTATATC
										4B-1095100-T7-33_1216	CACGGCCGCAGCGCACGGAG
										4B-1095100-T7-33_1280	CCGCAGCGTTTTTTTCTCTAA
										4B-1095100-T7-33_1472	CCCCCCCTCCCATCCATCGCCC
										4D-1165870-T7-33_0	TTTTAATGACATGTGTAGCATA
										4D-1165870-T7-33_59	TTCCTCCAAATAGAATACATTT
										4D-1165870-T7-33_118	TCAATAATCTGCATGTGAAAA
										4D-1165870-T7-33_177	ACAAGGTATGAACAATGACAA
										4D-1165870-T7-33_235	CTGACATGGAGCATCCCACGG
										4D-1165870-T7-33_662	CCCCCGCTTTTCCCCTTTTGTT
										4D-1165870-T7-33_728	CAACCCTATGGCTTGCCCCGT
										4D-1165870-T7-33_794	AAAATCGAAGGTAGGAGAAA
										4D-1165870-T7-33_860	CGATTCACATGATTCTAGAAA
										4D-1165870-T7-33_926	CACGGTTTTTGGTTTCGTTGC
										4D-1165870-T7-33_992	GCTAGATTCCTTATGGAATGA
										4D-1165870-T7-33_1058	ACAAACTAAACGGTCAACATG
										4D-1165870-T7-33_1124	CCCTCAACAGTGCAATACACA
										4D-1165870-T7-33_1190	CCACAGAGAGGGCCACACGG
										4D-1165870-T7-33_1454	ACCCGATGCCGTCTACAACTA
										3A-0616220-T7-34_832	TGTTGGGATAGCACCAGACAG
										3A-0616220-T7-34_896	CCAAGCTCCTTGGGTATCTCG
										3B-0824910-T7-34_1280	ATGATATTTCTGGATTTGTCGT
										3D-0867790-T7-34_704	ACATGGGGAGCACAGAGTAC
										3A-0706870-T7-35_0	GATGTTTTCTTTATTCCATCGA
										3A-0706870-T7-35_64	TTCCAAGTGTGGAACACCTTT
										3A-0706870-T7-35_384	TTCATAGGTAAGATCCTATCAA
										3A-0706870-T7-35_576	CTTCGGGCAGCTGAAGATTAT
										3A-0706870-T7-35_640	TTCAGTGCCGTCAATCGTGAT
										3A-0706870-T7-35_704	ATGTTTATTAATCTTGGTAACT
										3A-0706870-T7-35_896	GAGTTGAAGTGCAACATGAA
										3A-0706870-T7-35_960	TAGTTTCTGCATGTGGTTAATT
										3A-0706870-T7-35_1024	GTTAGTCTTTTCTGGGGCTTA
										3A-0706870-T7-35_1088	CACTGATTTAGGCATTGTTAGT
										3A-0706870-T7-35_1152	CAGACAGCTAATCCTTATAGTT
										3A-0706870-T7-35_1216	GTGGAATGCTTCTATCAATTCG
										3A-0706870-T7-35_1280	ATTTCACACCAAAGCTGCGCA
										3A-0706870-T7-35_1408	CACTTTGAACTATTCACAGCT
										3B-0833960-T7-35_0	TCCATCAAAAGAAAAGCGGT
										3B-2081760B-T7-35_0	TTTTGATTATGCGGGTTTTCCA
										3B-0833960-T7-35_64	ACATTATTACTACAGGCATATG
										3B-0833960-T7-35_192	TAGATGGGACACTGAGTTATG
										3B-0833960-T7-35_256	CCAAAGTTCTGAGGTGAGGC
										3B-2081760B-T7-35_320	CGAACCATACGCTCGCTTGTT
										3B-2081760B-T7-35_384	CTCCTCCATTTCTAAGAAAAA
										3B-0833960-T7-35_576	TTTTGCTAACTGCACCTTTGG
										3B-0833960-T7-35_640	CTTTTTATCTTTTTCTTATGAAA
										3B-2081760B-T7-35_640	TTTCAGTGCCGTTAATCGTGAT
										3B-2081760B-T7-35_704	CATGTTTATTAATCTTGGTAAC
										3B-0833960-T7-35_832	CTAATGGGAGTGTGTGCTAAT
										3B-0833960-T7-35_896	TATGCTAATGGGAGTGTATAAT
										3B-2081760B-T7-35_896	GGAGTTGAAGTGCAACATGA
										3B-0833960-T7-35_960	TATATGCTGTTACTGTTGTAAAT
										3B-2081760B-T7-35_960	ATAGTTTCTGCATGTGGTTAAT
										3B-0833960-T7-35_1088	AGCTGGTGGAGAATTGAAGC
										3B-2081760B-T7-35_1088	CACTGATTTAGGCGTTGTTAGT
										3B-0833960-T7-35_1152	AATGAGAAGATGCAGTAACA
										3B-2081760B-T7-35_1152	CAGACAGCTAATCCTTATAGTT
										3B-0833960-T7-35_1216	TTCTTATTTTGATTATGCGGGT
										3B-2081760B-T7-35_1216	GTGGAATGCTTCTATCAATTCG
										3B-2081760B-T7-35_1280	ATTTCACACCAAAGCTGCGCA
										3B-2081760B-T7-35_1408	CACTTTGAACTATTCACAGCT
										3B-0833960-T7-35_1536	CCCTCTCGAACCATACGCTCG
										3B-0833960-T7-35_1580	TCAATTGTCATACCAGGCCGG
										3D-0931440-T7-35_0	TGGAGAATTGAAGCCTCCAA
										3D-0931440-T7-35_64	TATGTCTTTGTTACTTCTTATTT
										3D-0931440-T7-35_128	TGTAACAGATGCTAATTCTTG
										3D-0931440-T7-35_640	ACTGGCATGCGGGTATAACAT
										3D-0931440-T7-35_704	TCACGATACAAATTTTCCATAT
										3D-0931440-T7-35_960	TGGGTGGCTATGTATCATCATA
										3D-0931440-T7-35_1024	TTATTACCTGTTTTTTCTTTAGT
										3D-0931440-T7-35_1088	TTCTAGGCTCTGTTGTTAAGTT
										3D-0931440-T7-35_1152	TCATCCTTACAGTTAATTTCCA
										3D-0931440-T7-35_1216	CTTCTATCAATTCGTCAATCAC
										3D-0931440-T7-35_1280	ATTTCACACCAAAGCTGCGCA
										3D-0931440-T7-35_1408	CACTTTGAACTATTCACAGCT
										2D-0605730-T7-36_126	CTCGGTCGCATCCGGGAGTC
										2D-0495280B-T7-36_128	GCGCTCGTCCACGGCGGGCC
										2D-0605730-T7-36_189	caaatcaaatcaaaaacaaaacgca
										2D-0605730-T7-36_882	GGCCGCTGGCGGAGGCAGC
										2D-0495280B-T7-36_896	CCGACACGTGGACGGACGGG
										2D-0605730-T7-36_945	GACGGACGGGTGCCCTCGCC
										2D-0605730-T7-36_1071	TTTTGCTCATGTGACCAAATA
										2D-0495280B-T7-36_1088	ctgtttctttttactccgcatatgagattt
										2D-0605730-T7-36_1134	tttttactccgcatatgagatttgtctaa
										2D-0495280B-T7-36_1216	gactttagcattgtagatgttgatgtgtt
										2D-0605730-T7-36_1260	tagcattgtagatgttgatgtgttttagt
										2D-0495280B-T7-36_1280	ctttatacgcaaagtaaaaaagaaga
										2D-0605730-T7-36_1323	atacgcaaagtaaaaaagaagaaac
										2D-0495280B-T7-36_1344	CTTTGAGCAAAATCTGTGCGA
										2D-0605730-T7-36_1386	TGAGCAAAATCTGTGCGACAG
										2D-0495280B-T7-36_1408	GAAACTTGAAAAAAAAACTA
										2D-0605730-T7-36_1449	AACTTGAAAAAAAAACTAAAA
										2D-0495280B-T7-36_1472	TTCAACCTATTCTAGGTAGTTG
										2D-0605730-T7-36_1512	TCAACCTATTCTAGGTAGTTGT
										2D-0495280B-T7-36_1536	ATTTTTTTTAAAAGCTGGTATA
										2D-0605730-T7-36_1575	ATTTTTTTTAAAAGCTGGTATA
										2D-0495280B-T7-36_1580	TCTATTCTAGTCAAATATTTAA
										2D-0605730-T7-36_1619	TCTATTCTAGTCAAATATTTAA
										U-0489460-T7-36_63	GCTGCCGTGAGTAATAATCGA
										U-0489460-T7-36_126	CGGGCCACCGAATCAAATCAA
										5A-1202840-T7-37_0	GTCGGCCATTCATCAAGTCGG
										5A-1202840-T7-37_64	AATTCAAAGGTTTGGAGCGA
										5A-1202840-T7-37_128	AACTCATAATTTAGCCAAGTA
										5A-1202840-T7-37_192	CCTCAATTGCGTTTGATAGCT
										5A-1486810-T7-37_384	GATTTTGCTTTCCCCAAATCAT
										5A-1202840-T7-37_448	CTTCTTTATTCTACACTCTTCC
										5A-1486810-T7-37_448	CAGAATTTTTCCTTTCATAATAT
										5A-1202840-T7-37_512	TTGCAATGTCTTTTCCCTAATG
										5A-1486810-T7-37_512	tttttcctttagcatttccccttttttcctttt
										5A-1202840-T7-37_576	ATAGCATGCCGATCGGCAAGC
										5A-1202840-T7-37_704	AGTTCACGAGAGGTAAACCA
										5A-1486810-T7-37_704	TCGTTTTGGGCCGTCTCGCTT
										5A-1486810-T7-37_768	cggcgggcCGCCCGGGTCGTTC
										5A-1202840-T7-37_832	AAGCCCCCGGCCGGCCGGCT
										5A-1486810-T7-37_832	ATGAAGCGTTGCtgcagtgcagt
										5A-1202840-T7-37_896	CCCAGGCAAAAATTAACGCA
										5A-1202840-T7-37_960	ATACACGACCGGGTCGGGTTT
										5A-1486810-T7-37_1024	CGTGGGGTTGTCCGACTCCAT
										5A-1486810-T7-37_1088	ATATGGAGAAAAAAAACGCT
										5A-1486810-T7-37_1152	CCAGCTTCTCGATATATAGGG
										5A-1486810-T7-37_1216	AAACTGCTGTTGGGCCTAATA
										5A-1486810-T7-37_1280	ATTTTATTGTTTATGCTGACAA

5A-1486810-T7-37_1344	TGCAACCATTTGACGTAACCA
5A-1486810-T7-37_1408	CGCAATCATATCATGCACAtctc
5A-1486810-T7-37_1580	AGATACTATATCTAGACAAATG
5B-1325650-T7-37_384	CTTTCCCCAAATCATTTTCTCT
5B-1325650-T7-37_448	TGTCCTTTTTATACTACTATTCT
5B-1325650-T7-37_512	tcctttagcattttcctttttaatttttgc
5B-1325650-T7-37_704	CTTGTTTTGGGCCGTCTAGCT
5B-1325650-T7-37_768	ACACCGCGTGCTCGTGCCACG
5B-1325650-T7-37_832	GAGCTGGACTTTTGACAGGA
5B-1325650-T7-37_896	CATTTTTCTACTTTTGGCCGAC
5B-1325650-T7-37_1024	ACTGTTGTCAAGCGTCTCATC
5B-1325650-T7-37_1088	TGATGTTGAAACGGTGCATGT
5B-1325650-T7-37_1472	GACGGCGGTTGTTGTTCTGGT
5D-1432830-T7-37_384	AATCATTTTCTCTCGCCACAAA
5D-1432830-T7-37_448	ACTATTCTACTGTACCCTATGC
5D-1432830-T7-37_704	CGCTTGTCCCGTTTGGGAGGT
5D-1432830-T7-37_768	GTCGTTCGCGCCGCGTCCATC
5D-1432830-T7-37_832	GCAGTGGGCTGCGTGGGTTG
5D-1432830-T7-37_1398	tatatatatatTTCTTGCGAGTTCT
5D-1432830-T7-37_1459	TGCAGTGACCAGCTTCTCGGT
5D-1432830-T7-37_1520	GTTTCTACACTAAAACTGCTGT
4B-1045410-T7-38_512	TTCAATGTCCACCACCATCATG
4B-1045410-T7-38_576	ATCCAGCTGGTCAGATCTGGA
4B-1045410-T7-38_768	CTCTAGCCATGGTGGCGAGTG
4B-1045410-T7-38_832	TCCCGGTTGCTGTAAATCGGA
4B-1045410-T7-38_896	AGATGGGGGGGTGGGGGT
4B-1045410-T7-38_1088	TTGGGGGAGAAGAGTCCTCG
4B-1045410-T7-38_1152	ATGGATGGCCCATGACAAAAG
4B-1045410-T7-38_1216	TAGTATTGACATGGTTTCCATT
4B-1045410-T7-38_1408	GAAAAAAATCAACCATGGATC
4B-1045410-T7-38_1472	TTGATTTGATCCATTGTATTTT
4B-1045410-T7-38_1536	CTACTGCTTATATGCGGTGCAT
4B-1045410-T7-38_1580	CCCTAGCTGCTAGTACAACTG
4D-1117610B-T7-38_0	ACTGCAGCTAGCTCCAACGTT
4D-1117600A-T7-38_128	AGGTACTTGTAGTAGTAGCTA
4D-1117600A-T7-38_192	ACTGAGCTCTCAGGCCAGAA
4D-1117610B-T7-38_192	CAGACCAGACAAAATATTCGA
4D-1117600A-T7-38_384	GTCCACACAAGGCCACTGCTA
4D-1117610B-T7-38_384	TGTCCACGCAAGGCCACTGCT
4D-1117600A-T7-38_448	ATGCTAACACATAGGTCCGAG
4D-1117610B-T7-38_448	TGCTAACACAAAAGCCCGAG
4D-1117600A-T7-38_512	AAAAGTACTACCAGCTGTGAA
4D-1117610B-T7-38_512	GAAAAGTACTAGTTGTGGAA
4D-1117610B-T7-38_576	TGTCGATATATCTATTTTTTTAC
4D-1117610B-T7-38_640	AGGCCACGTGTTACTCATCAT
4D-1117600A-T7-38_704	AGCCTCTCGCTGTTGCGGGAG
4D-1117610B-T7-38_704	CTCCTAATCATAAAAATATGAA
4D-1117600A-T7-38_768	TCCCTTTTTTTTCGTCTGGCAT
4D-1117600A-T7-38_832	ACCCATATGTCCCGATCTACAG
4D-1117610B-T7-38_896	ACTGCCATCTTTGTGCCTCCAT
4D-1117600A-T7-38_960	CGGGGCGGACTCCGGCCAGA
4D-1117600A-T7-38_1024	TTCGGCGAGGTGCTGGCTCT
4D-1117610B-T7-38_1088	ATCCACGGGAAACCTAAACCG
4D-1117610B-T7-38_1152	CACCCTCCATCGGTGGTGAG
4D-1117610B-T7-38_1216	TGGGGAAGCGCCGCCGGCAT
4D-1117610B-T7-38_1280	ACAAAATTTTCACACTGTTGT
4D-1117610B-T7-38_1408	GCTGGATTGGTCCGGGCCGC
4D-1117610B-T7-38_1472	CCACTCGAGCCCTCATCTCCT
4D-1117610B-T7-38_1580	CGGCGGCGGCGAGAAAGGA
4A-0986290-T7-39_128	TCAGATGTGCCCGGCCATGCA
4A-0986290-T7-39_384	TAGCGAGAGAGTGCTTGTGG
4A-0986290-T7-39_448	CCCTACTTGAGATTGAGAGGG
4A-0986290-T7-39_512	TGAGTGTGGTGTGTGAATGAG
4A-0986290-T7-39_704	AGCCCCTTCTTCAGGAAACGA
4A-0986290-T7-39_768	CCTACCCGGTATACATTGAAA
4A-0986290-T7-39_960	AATGGAACCAAAGGACCATTT
4A-0986290-T7-39_1024	TTGGGATAAGTACTGCACTCT
4A-0986290-T7-39_1216	TGTCTTTTGGGTCGGCTTCGT
4A-0986290-T7-39_1280	GAGTTTCTAAAAAAACATAGC
4A-0986290-T7-39_1344	TTGAACTATGGGTTCAAAATT
4A-0986290-T7-39_1408	AGAAATGTAGATGAAGCGTTT
4A-0986290-T7-39_1472	AAAAATCCTAGCACTAAATGA
4A-0986290-T7-39_1536	TTTCATGCGCATGTTTTAATCC
4A-0986290-T7-39_1580	TGTGTAGTCAAATTACATGTCT
4B-1076210-T7-39_128	CCCATCCATAATCCATCAGTAT
4B-1076210-T7-39_192	TTTGTAACTCTAGAACAAAGG
4B-1076210-T7-39_256	ACCACCTTCTCTAACCGCAATT
4B-1076210-T7-39_512	GGCGGCGCGGCGGGCGGGC
4B-1076210-T7-39_576	CGGCTAGTCGGCTTGGCTATG
4B-1076210-T7-39_640	GGCCGGGGATTTTCCTCCTTT
4B-1076210-T7-39_704	GCTACTACAACCTTGGGCAAC
4B-1076210-T7-39_768	TTAGCCGCAGATCGAAAGGTA
4B-1076210-T7-39_832	GAGTGTAGTACTTATCCCAACT
4B-1076210-T7-39_896	GCTACCAAATAGTTCTTTGGTT
4B-1076210-T7-39_1088	CTGCCAATAAATGCCCTAGGT
4B-1076210-T7-39_1216	AGACCCTCCCTCTCTAACTATA
4B-1076210-T7-39_1408	GAGCTAGGTACCAGTGCCCCT
4B-1076210-T7-39_1472	CTCCTAGTCCTAGACCTCTTTC
4D-1159720-T7-39_0	AAATGACTAAGGATGGGAATG
4D-1159720-T7-39_66	CTTCGTTTGGGGTGGGGACAT
4D-1159720-T7-39_132	AGGTAACATGGCCCATTTTAAT
4D-1159720-T7-39_198	CATTTAGTGGTAGGATGTTTC
4D-1159720-T7-39_264	TCATATACGCAGCTTCATCTAC
4D-1159720-T7-39_330	AAGGCAATTTTGAACCAATAC
4D-1159720-T7-39_462	AATATCCCAATGGGTAACATCG
4D-1159720-T7-39_528	AAGATCCTAGTAACTTCCTTTA
4D-1159720-T7-39_594	TTTTGAATATGAGAGTGTAGT
4D-1159720-T7-39_660	GTAGCTACCAAATGGTCCTTG
4D-1159720-T7-39_726	GGTAGGCGAATCCTGTGCAG
4D-1159720-T7-39_858	GTTCAATGTATACCGGGTAGG
4D-1159720-T7-39_1450	AACTCCCATGGGAGATTTATCT
4A-0949140-T7-40_0	ATTTGATTTCGCGTTGCTCTCC
4A-0949140-T7-40_64	CGCTGGAAATTGCCAATCTAA
4A-0949140-T7-40_576	CCGCGCCCGTAGTACACCGTA
4A-0949140-T7-40_1088	CTCCTCCCCGCTCCGCCAAGC
4A-0949140-T7-40_1152	CTTGCCGTGGTCAAGGGGTG
4A-0949140-T7-40_1280	TGTGCCGTGCTGCGTGTTGAT
4A-0949140-T7-40_1344	GCTGAAATGCGACGGGTGTG
4A-0949140-T7-40_1472	TTGAGCGCTCTACTGCTATGCT
4A-0949140-T7-40_1536	GTTATTATTACACAGTTCCTGT
4A-0949140-T7-40_1580	GCTGTGGTGTTGTTGCAGTTC
4B-1102590-T7-40_0	CGCCGAAGGTTCAGCACCGC
4B-1102590-T7-40_64	TAGTTACCATGCTCAACCCACT
4B-1102590-T7-40_320	AATAATAACATAAATAACAAAA
4B-1102590-T7-40_448	CACGCTTGCCTTCCACTGGCA
4B-1102590-T7-40_640	ACCGGAAGGTGCCGCCTCTA
4B-1102590-T7-40_1152	GGCGGCCGCGGGACGCAGC
4B-1102590-T7-40_1344	GAAACAATGCCTTTGAAACGG
4B-1102590-T7-40_1580	AGAATACCCATAGCGCCGCCC
4D-1163300-T7-40_0	CGCCAAAGCTTCAGCACTGC
4D-1163300-T7-40_64	CAGTCATGCTCAACCCACTCAT
4D-1163300-T7-40_128	ACCACTTTAAAATAACAGGAA
4D-1163300-T7-40_192	TATTCGTCGTGACACATATAGC
4D-1163300-T7-40_256	CATCTGCAAGTGGTTCTTAGC
4D-1163300-T7-40_320	GCAAGATTTCCTGAATTTCAG
4D-1163300-T7-40_384	TCCCCTTTCCCCACTCGACATC
4D-1163300-T7-40_512	GGCAGGGATGCGTCCCCGCG
4D-1163300-T7-40_1024	AGCAGGTCGGCAGCGAGCGA
4D-1163300-T7-40_1216	GATCGGAGCATGGAAGAAAC
4D-1163300-T7-40_1280	GGATTATCTAAGTGCGAGAAA

										3A-0682250-T7-41_122	CTCCATGTACACCCAACATTAG
										3A-0682250-T7-41_183	GATATCCTAAATGTATAGATAG
										3A-0682250-T7-41_366	AGCTCACGGTCTGCAGGTACA
										3A-0682250-T7-41_427	CTATTCAGCTCAACAAACTATT
										3A-0682250-T7-41_488	GGCATTTAGGACATTTAGCAC
										3A-0682250-T7-41_671	AGTCTCTGTTCCCTTGCCATTA
										3A-0682250-T7-41_732	CAATCGTTAGGATTAAGTCGT
										3A-0682250-T7-41_915	GCCATCTGTTACGGAAGCCAA
										3A-0682250-T7-41_976	AGGTGTTTTGGTGTATCTGCA
										3A-0682250-T7-41_1037	ATTCAGAATAAACACGCCAGA
										3A-0682250-T7-41_1098	CATAGCCAATTTGAGCGATGA
										3A-0682250-T7-41_1159	CCATGTTCTCGGGGAAAGCAG
										3A-0682250-T7-41_1220	TGCATCTCATCTCTTGTGCATC
										3A-0682250-T7-41_1281	CAAACAATCACTACTACTGGG
										3A-0682250-T7-41_1342	GCAGAAATATTTTAGCATTCGT
										3A-0682250-T7-41_1525	GGAAAAGGCGCGGATCCTGG
										3A-0682250-T7-41_1708	AAGGGACGCGCCGCGATTCA
										3B-0768030-T7-41_640	GGCCTTGGCACTGGCACCGG
										3B-0768030-T7-41_768	CAGGGCGGAGCGAGCGGAG
										3B-0768030-T7-41_960	TGGCGTCTTCCTCCTTCTCGG
										3B-0768030-T7-41_1024	TCTGCCAGGATCTGCGCCTTT
										3B-0768030-T7-41_1216	TAAAATATTTCTGCTATTTACT
										3B-0768030-T7-41_1280	TAGTATTGATTGTTTGAGAGG
										3B-0768030-T7-41_1344	GCAGAAGAGATGAGATGCCC
										3B-0768030-T7-41_1408	CGTTATTTTAGGTCCACTTTCT
										3B-0768030-T7-41_1536	TTCGTGTTTAACCGTCGTGAAT
										3B-0768030-T7-41_1580	AATTTTGTTGCTACTTCATGTG
										3D-0917540-T7-41_512	aaaggGGGAAGCCAACCCAGC
										3D-0917540-T7-41_576	ATCTCTCTGCGAGGAGGAGG
										3D-0917540-T7-41_768	TCCGGCGACCATGATCTGGCT
										3D-0917540-T7-41_832	GCCATTCTGCGATCTCTGCCG
										3D-0917540-T7-41_896	GCGTTGGTTATTAGGAGAGAT
										3D-0917540-T7-41_960	CAAAATATTTCTGGTATTTACT
										3D-0917540-T7-41_1216	agcatgctaatagcatattccgaggtc
										3D-0917540-T7-41_1280	AATTGCCCTAGACGCACAAGA
										3D-0917540-T7-41_1344	GACTAGGGTAGGTTTGTTATG
										3D-0917540-T7-41_1408	GGTCCAGTTAGGCGGCCCAAT
										3D-0917540-T7-41_1472	GTGTCAAATAAACGTTTCGTC
										3D-0917540-T7-41_1536	CATTCTGAAATAGTTCTCTTGT
										1A-0034450-T7-42_320	AGGTCCGTCTGGTGGAGGAG
										1A-0034450-T7-42_384	tgtgtgtgtgtgtgCTGGGGCTGG
										1A-0034450-T7-42_448	ATGAAAAACAAAACAAACATA
										1A-0034450-T7-42_576	GGCTTGAGCGTCAGGGCGTC
										1A-0034450-T7-42_640	TTCTTTTCCCTGCATCTGTGTC
										1A-0034450-T7-42_704	aaacaaacaaacaaacGACGATAT
										1A-0034450-T7-42_768	CGGAGGCAAATGCCAAGCGC
										1A-0034450-T7-42_832	AGCTTTATCCAATATGACCCTT
										1A-0034450-T7-42_896	AGCTCTTCTAAGAACAAGAAA
										1A-0034450-T7-42_960	TTCACCAATGAGGAAGGGGT
										1A-0034450-T7-42_1024	GTGTGTCTGTGTGTTTTGCTA
										1A-0034450-T7-42_1216	TGCTTCTCTCCCGGCCTCTCCA
										1A-0034450-T7-42_1280	CTTAGTATATATTTTTTTAACAT
										1A-0034450-T7-42_1344	AGCAGTGGTACATAGCATCAA
										1A-0034450-T7-42_1408	TGCTAAGAGGCTACCAATTGG
										1A-0034450-T7-42_1472	GAAGCAAGCCAACGTCGGGG
										1B-0087580-T7-42_0	CGCCGCCAGCCAGCTAGGCC
										1B-0087580-T7-42_256	gaggaggaagaaagggagaggtgg
										1B-0087580-T7-42_320	CGCCCACGCCCACCACAAACA
										1B-0087580-T7-42_384	ATGACATGGGATCCTCATCCA
										1B-0087580-T7-42_448	ACTACTACTCCCACATAGTACA
										1B-0087580-T7-42_576	CGTCAGGGCATCGCCTTTCTT
										1B-0087580-T7-42_640	CCCACACCTGTGTCGCCATAC
										1B-0087580-T7-42_704	ACATACATCAACACTCGATTTT
										1B-0087580-T7-42_768	AGGTCATCGAATTTGTCATCA
										1B-0087580-T7-42_960	GGTTTCATGTACTATTCACTTG
										1B-0087580-T7-42_1024	TACTATATCCTTTTTATGCATGT
										1B-0087580-T7-42_1216	CATTGCTAGGTCGCTTTGACG
										1B-0087580-T7-42_1280	GGAGTGCCCGGATGAACACA
										1B-0087580-T7-42_1344	CATGAGTACATGACTCAGACT
										1B-0087580-T7-42_1536	TTGCATTGTCTCCCATGGGGC
										1D-0221450-T7-42_642	GTGTgctggggctggggctggggct
										1D-0221450-T7-42_704	CACCAATTGTATCCTTTTTATG
										1D-0221450-T7-42_952	CGATCCATTTGGCCACCTTTGT
										1D-0221450-T7-42_1403	CCTCCGTCATGGAGGGCAATG
										1D-0221450-T7-42_1462	GGGGAAAAGGAGGAAGTGG
										1D-0221450-T7-42_1521	TATAACCTGATTTTCCATAGTA
										4A-0987490-T7-43_64	GCCTGCGTGTATGTATGCTTG
										4A-0987490-T7-43_192	TTTGATCCTGGGAAGCTAGAG
										4A-0987490-T7-43_256	ATACTACTACACAGCATGAATT
										4A-0987490-T7-43_320	GTAAGTGTGGGGATTCAGAAT
										4A-0987490-T7-43_384	CTGCTCGGCTGATTTGGGAG
										4A-0987490-T7-43_448	TCCCATGTGCCATGTCAGTGT
										4A-0987490-T7-43_512	ATCGCTTTGCTGAGACGGAG
										4A-0987490-T7-43_576	TCTCGATGATAGTAGCGTGCT
										4A-0987490-T7-43_640	GTTCTCTTGTAGCTGTAGGTTA
										4A-0987490-T7-43_704	TGATGTACTAGCTAGAAAACC
										4A-0987490-T7-43_768	GGAATACATATGAAATAGAAC
										4A-0987490-T7-43_832	TGGCCGTGCCTTGCTAGAGTT
										4A-0987490-T7-43_896	AAATAATACAAGTACACTCTTC
										4A-0987490-T7-43_1024	AGGCCAGAACTAACCTTCCCA
										4A-0987490-T7-43_1152	GGCCACTAAAGTAGTGGCCTA
										4A-0987490-T7-43_1216	CCATATATTTTGTTTGGAAAAA
										4A-0987490-T7-43_1280	CCAGCTAAAATCACAGCCGAC
										4A-0987490-T7-43_1344	ACCATTGAAGATATACAAATTT
										4A-0987490-T7-43_1408	TTGGGTTTGCAGGAGATTTTG
										4A-0987490-T7-43_1472	CACGCTTCTTAAACCAGGAAA
										4A-0987490-T7-43_1536	TATGATCGTCTCTGTGTTTTTT
										4B-1091340-T7-43_0	CACCTGCCTCGAGTACTCGAA
										4B-1091340-T7-43_192	TTTGATGACAAGTATTTCCGG
										4B-1091340-T7-43_256	AGCTTGCAAAAAAACAGAGA
										4B-1091340-T7-43_320	TGTTTCTTTTCCTGGTTTCAGA
										4B-1091340-T7-43_512	ACGGAGGGAGTACTAGTAAA
										4B-1091340-T7-43_640	GATTTAGCTGGGTCAGGTTCA
										4B-1091340-T7-43_704	ACAAACTGTATGGCTTACTAA
										4B-1091340-T7-43_768	TTATCTCATCTGGAGAAAAAG
										4B-1091340-T7-43_832	TGTGTACTTGTATTATTTCTCTT
										4B-1091340-T7-43_1024	TTCCAGTAATATGCCTTTCTGG
										4B-1091340-T7-43_1152	GCTTGTAAAATGCACCAAGGT
										4B-1091340-T7-43_1216	CAGAATTGTGGATTTTGAGTT
										4B-1091340-T7-43_1280	AAAGCTTAAAAGGTTCAGAA
										4B-1091340-T7-43_1344	CTAAACCTACAAGACAACAGG
										4B-1091340-T7-43_1408	TACTACCATCGAGATATCTATC
										4B-1091340-T7-43_1472	CAGCAAAGAGCCCTCCGGGA
										4B-1091340-T7-43_1536	TGGCACATGGACAGAAACCA
										4B-1091340-T7-43_1580	CTACAGATGGAACGAACACCA
										4D-1181480-T7-43_64	CATAAATAAATGAGAAGCGGC
										4D-1181480-T7-43_128	AACAGCTTGCAAAAAAAAAA
										4D-1181480-T7-43_192	GAAGTGCTACTAAATATGTGTT
										4D-1181480-T7-43_256	TTTTAATCGAACACGCACCTA
										4D-1181480-T7-43_320	AGAAGCAAAAGAACTGTACA
										4D-1181480-T7-43_384	CTAATTAGTAGTAAAACATAAA
										4D-1181480-T7-43_448	GATCATAATGGCTAAATATATT
										4D-1181480-T7-43_512	AAGCGTGAACAAATTTGACA
										4D-1181480-T7-43_576	AACCCAAAAAGGTACTAGTAA
										4D-1181480-T7-43_704	GTGATTTAGCTGGGTCAGGAT
										4D-1181480-T7-43_768	ACAAATTGTATGGCTTAATAAA
										4D-1181480-T7-43_896	GTACTTGTATTATTTCTCTTGAT
										4D-1181480-T7-43_1024	TATACCCCCATGCCAAAATTCA
										4D-1181480-T7-43_1088	ACTTCCAGTAAATATGCCTTCC
										4D-1181480-T7-43_1152	CTGTAGAGGTGTGGATTTTGA

										4D-1181480-T7-43_1216	CTGAAAGCTTAAAAAGTTCAG
										4D-1181480-T7-43_1280	AAACCCTACAAGACAACAGG
										4D-1181480-T7-43_1344	ATATGTATCACCGCAAACAAC
										4D-1181480-T7-43_1408	CCCGGGGATCGACGAATCGC
										4D-1181480-T7-43_1472	GGACGGAAATCAAGAACGAC
										4D-1181480-T7-43_1536	AACGCCTCCCTCGGTCCCAA
										4D-1181480-T7-43_1580	GCTTTCACTGGTCAACTAGAA
										4A-1008180-T7-44_0	ATTAGATTTGCTAGAAAAGC
										4A-1008180-T7-44_62	ATCCTGATGCATATGTTTATTTT
										4A-1008180-T7-44_124	AGTACAAACCCATGTCATCTT
										4A-1008180-T7-44_248	GCATCAGGATTTGAATCCTGA
										4A-1008180-T7-44_310	TATTGGTTCGCTGAGCGCAC
										4A-1008180-T7-44_496	TTTTGATGACAAGTATTTCCG
										4A-1008180-T7-44_558	ATAACCAGTGAGAATATATTTA
										4B-1061080-T7-44_192	catgcatgGCCAATAAATCACTA
										4B-1061080-T7-44_320	AAACGAAAGTTTTGAAATAAG
										4B-1061080-T7-44_384	ATCTGTACTGCTCGCAAAAAA
										4B-1061080-T7-44_512	tttgggacggagggagtaCAAGGA
										4B-1061080-T7-44_576	TATATCTAACTTCACACCAGTT
										5A-1238490A-T7-45_64	ACACGTTACAGTATGGTTTTA
										5A-1238490A-T7-45_192	TCTCAGCTTGTGTGAATTATAC
										5A-1238510B-T7-45_192	GATGGGCAAAAGGCTCAACA
										5A-1238490A-T7-45_256	AGCATCAAATTATTCGACTGC
										5A-1238490A-T7-45_320	GACCAATTCCTTGGCTGTTGT
										5A-1238510B-T7-45_320	TGGGGCTACCCCTGTAACCGT
										5A-1238490A-T7-45_384	GAATTCTTCTCTTATCTAACAC
										5A-1238490A-T7-45_448	TTCTGTCGCTTTCTTTGTGGA
										5A-1238490A-T7-45_512	GGTTAATCTGCAACCCAGGGA
										5A-1238490A-T7-45_576	GAGCAAAGTGAGACGAGCAG
										5A-1238490A-T7-45_640	CTCCTACTCGTGATGCAGCTA
										5A-1238490A-T7-45_768	CCGACGGAAGCTGGGCGACG
										5A-1238490A-T7-45_896	AGAGATGGAGACGCACGCAC
										5A-1238510B-T7-45_960	TCTGGGCCACACGCGTCCAAT
										5A-1238490A-T7-45_1088	GCAAAATTCTGCAGAGCTGAT
										5A-1238510B-T7-45_1216	TCAcccgcccgcccgccgccgccctg
										5A-1238490A-T7-45_1280	AAGAACGATGTCCCTAGCTTG
										5A-1238490A-T7-45_1344	CAATGGTTTGGGTTCGCGGC
										5A-1238490A-T7-45_1472	CAACCGTGCGAGACGCCATC
										5A-1238490A-T7-45_1536	TTTTGACGAAAAGCATTGAGC
										5A-1238490A-T7-45_1580	AGGTTACAAAGCTCAAGATGA
										5B-2102190-T7-45_512	TACATAGCAGTTATAGCATATT
										5B-2102190-T7-45_640	ACCGTGTCCCCTTGCTGTTGC
										5B-2102190-T7-45_768	cacgcacgcacgcacgccgcCTCTG
										5B-2102190-T7-45_832	GATGGCAGTTTAGGGTGATCT
										5B-2102190-T7-45_896	CGGGGCACCCCGCGCACGAT
										5B-2102190-T7-45_1024	CAAGCCAACCATTCTTCAATC
										5D-1414270B-T7-45_0	CGCTCGATCCAATCACACTCG
										5D-1414250A-T7-45_64	ACACGTTACAGTATGGTTTTA
										5D-1414270B-T7-45_192	CCGATCCAGGACGACGGTTT
										5D-1414250A-T7-45_256	AGCATTAAATTATTGGACTGC
										5D-1414250A-T7-45_320	GGCCAGTTCCTTGGCTGTTAT
										5D-1414270B-T7-45_320	ACTCCCCGGACGATCCTGGCC
										5D-1414250A-T7-45_384	GAATTCTTCTCTTATCTAGGAG
										5D-1414250A-T7-45_448	TGGACAAAGTCGCACTGCTA
										5D-1414250A-T7-45_512	AGGGATGAAACTGAGAGCCA
										5D-1414250A-T7-45_640	TACTTGTGATGCAGCTACAGA
										5D-1414250A-T7-45_896	CCCGAGCCGAGGGGCGGAG
										5D-1414270B-T7-45_960	CAAATTTGTCAAATTTGACGC
										5D-1414270B-T7-45_1024	GACAAGCCAGCCATTCTTCAA
										5D-1414250A-T7-45_1088	CTAGAGAATCGGGATAAGATA
										5D-1414250A-T7-45_1152	CAACCTCCGCCTCCATCGCTC
										5D-1414250A-T7-45_1280	TCTTCGCTGCATGTGTTGTTG
										5D-1414250A-T7-45_1408	CGTGCAAGACGCCATCAAGAT
										5D-1414250A-T7-45_1472	AGGAAAAGCAATGAGCGTTG
										5D-1414250A-T7-45_1536	GCACACAACCGGTTCCACTTC
										7A-1781730-T7-46_0	Attgactggagtgtatggattacaaga
										7A-1781730-T7-46_64	atagagtgcgccgaacccctgatgatt
										7A-1781730-T7-46_128	cgacaattactggtaatgtcGGCATT
										7A-1781730-T7-46_384	TCTTTAGGCTAGGCCTGTGAC
										7A-1781730-T7-46_448	CGGCCAAGCACGCGTTAGCCT
										7A-1781730-T7-46_896	GATAGATGCAAGTTTTACTTGT
										7A-1781730-T7-46_1024	CCCTACTACACGCAGGTGTCC
										7A-1781730-T7-46_1216	CAGTCCAGGAGATAAAAGAC
										7B-1867270-T7-46_0	ATCCAATAACTCATCCATGTAA
										7B-1867270-T7-46_192	GCTGGACCAAGAGATTATCTG
										7B-1867270-T7-46_256	CATTTTTCTGGCCCAGCCACT
										7B-1867270-T7-46_320	ATATTGTCTCTAGCAATACGAG
										7B-1867270-T7-46_384	CACAGATAAAACCAAATGAAA
										7B-1867270-T7-46_448	GAGTGTAGAACGGCACCGCC
										7B-1867270-T7-46_512	TCTTCCCCCGACCTCAACCAA
										7B-1867270-T7-46_832	TATTTATTTCATATTTTTTGAGT
										7B-1867270-T7-46_896	TCAGATGTGATAGACGCAAGT
										7B-1867270-T7-46_1024	AGCAATAGCCCCCTACTACAC
										7B-1867270-T7-46_1088	AATTAATAAGTATTTCTAGATG
										7D-2074540-T7-46_64	cttatatagagtgcgccggacccctga
										7D-2074540-T7-46_256	AGATGAACCGGTCGAGTGGA
										7D-2074540-T7-46_384	TAGGGTCTGTAGGCCAGGCAT
										7D-2074540-T7-46_448	ACGGGGCGGCCAAGCACGCG
										7D-2074540-T7-46_832	ATATTTGTTAGATAACTTTTAA
										7D-2074540-T7-46_896	ATATGATAGACTGTATATAGAA
										7D-2074540-T7-46_1024	CACGCAGGTGTCCATTCATCT
										3A-2083710-T7-47_320	CACCGGATGCAATCTTTGCAC
										3A-2083710-T7-47_512	CGCGACGTCCCCTCTCCCGCC
										3A-2083710-T7-47_704	CCTCTGTCCCCGCATGGTCCA
										3A-2083710-T7-47_768	TATGTTGGTTGTTTGAGTATAG
										3A-2083710-T7-47_832	TCTTCACGGAAACGGTAAATT
										3A-2083710-T7-47_896	TTGATATGCCCATATTTATACG
										3A-2083710-T7-47_1024	CATCACACACGCACGGACAAT
										3A-2083710-T7-47_1088	TTGTAATCTTTCTACATCAAAC
										3A-2083710-T7-47_1152	GGTTCTTGTGAAATTTGAAAA
										3A-2083710-T7-47_1216	GGCGGCCCCTCTCTGCCGCAA
										3A-2083710-T7-47_1408	CTCTGGCCCCGCATGATCTAA
										3A-2083710-T7-47_1472	ATGTTGGTTGTTTGAGTATAGT
										3A-2083710-T7-47_1536	CTTCACGGAAAATGCTAAATT
										3A-2083710-T7-47_1580	CTTCTCCATGCTCAACACAATT
										3B-0757990-T7-47_0	GCCGGAAAGTGTACTTTTGAC
										3B-0757990-T7-47_64	TCGATTGTGTCGGGCATGATG
										3B-0757990-T7-47_128	GAAGAATTAATATAGTCCATGT
										3B-0757990-T7-47_192	ACATAAGGAATGTCTGGTCGG
										3B-0757990-T7-47_384	GTCGGAGAGGTGTTATCACCC
										3B-0757990-T7-47_448	AATTCATCACAGCCTAGCTCTT
										3B-0757990-T7-47_640	CTGCTTGTCAGCGCCAATGAC
										3B-0757990-T7-47_704	CTCCGCCTAGAGATATCATTAA
										3B-0757990-T7-47_768	GAAGACAAATTTGAATGTTTG
										3B-0757990-T7-47_832	GTGCGTAGATACCATTATTTTT
										3B-0757990-T7-47_896	GTACCAATACAAAGGTGTCCG
										3B-0757990-T7-47_960	GCGTCGCGTGTGTGGAGGGG
										3B-0757990-T7-47_1152	GCGCGCACCGTGCGCGCGCA
										3B-0757990-T7-47_1216	ATTTTGTCAAAACCAAGCCCT
										3D-0851700-T7-47_320	CCGTGCGGATATGCTCGTCGC
										3D-0851700-T7-47_384	AGCGATCTTGTGTTCCTTCCA
										3D-0851700-T7-47_512	GAGCCGACGGTGCGCGCGCG

										3D-0851700-T7-47_704	TAGTGGGATTTTTGGCGCGA
										3D-0851700-T7-47_768	TGGGACTCCTTTGTAACTGTA
										3D-0851700-T7-47_832	AAAAATAACGGTATCGATGCA
										3D-0851700-T7-47_1024	TGTCGTTCGCACTGACAAGCA
										3D-0851700-T7-47_1088	CATGTGTGTTCCTTCCAACAC
										3D-0851700-T7-47_1152	GATTTGATCCTTTAGTGATCTT
										3D-0851700-T7-47_1344	TCTCCCGCCAACCTTCCATCCG
										3D-0851700-T7-47_1472	CATTCCTTATGTTGGTTGTTTG
										3D-0851700-T7-47_1536	TATTAATTCTTCACGGAAACA
										3D-0851700-T7-47_1580	GGTTTGTCTTCATCATGCCCG
										3A-0617410-T7-48_0	CATTAGTATCATATGCATAATA
										3A-0617410-T7-48_64	GGGGCTTGTACTAACTCTTTT
										3A-0617410-T7-48_128	AAAAACACACACACACCACA
										3A-0617410-T7-48_256	TCTGGAAGAGCTCGGATTCTA
										3A-0617410-T7-48_320	AAAACAACTCCGCTGGGAAT
										3A-0617410-T7-48_384	TGAGCTTTGTTCACGTTGGAA
										3A-0617410-T7-48_448	GTGAGTATGCAAGAGGAAGT
										3A-0617410-T7-48_576	TGGCGGCTAGGCTGGTGCTG
										3A-0617410-T7-48_640	AGAACACTTGTGCGTGGAAT
										3A-0617410-T7-48_704	TAAAAAAAGTGGATTATGGCT
										3A-0617410-T7-48_832	GGGCAACCAAGCCTGGTCAG
										3A-0617410-T7-48_896	ACCCTTAAGATACAGGTTAGT
										3A-0617410-T7-48_960	AGACATACAAATATTAATTATT
										3A-0617410-T7-48_1024	GGTTGGATGGGTTGGAGGGT
										3A-0617410-T7-48_1088	TGACGCTTTGGTATTTCACTAA
										3A-0617410-T7-48_1152	TAATGAGGTGCATGTGATGAT
										3A-0617410-T7-48_1216	AGGCGGTGTGAGTGTGTATG
										3A-0617410-T7-48_1280	TTTCTAGAAAAAAAAATTGTAT
										3A-0617410-T7-48_1344	CACTATTCCTATATTAAATAATG
										3A-0617410-T7-48_1536	AGCTCCCTTGTCCCGTGTGGA
										3B-0757990A-T7-48_0	GCCGGAAAGTGTACTTTTGA
										3B-2099820B-T7-48_0	GGCAAGCTAGGAAGGATGCC
										3B-0757990A-T7-48_64	TCGATTGTGTCGGGCATGATG
										3B-2099820B-T7-48_64	CTAGCGGTAGCTATGTGTTTG
										3B-0757990A-T7-48_128	GAAGAATTAATATAGTCCATGT
										3B-0757990A-T7-48_192	ACATAAGGAATGTCTGGTCGG
										3B-2099820B-T7-48_192	GCTGATAGTTGTCTGATTCCTG
										3B-0757990A-T7-48_384	GTCGGAGAGGTGTTATCACC
										3B-0757990A-T7-48_448	AATTCATCACAGCCTAGCTCTT
										3B-0757990A-T7-48_640	CTGCTTGTCAGCGCCAATGAC
										3B-0757990A-T7-48_704	CTCCGCCTAGAGATATCATTAA
										3B-0757990A-T7-48_768	GAAGACAAATTTGAATGTTTG
										3B-0757990A-T7-48_832	GTGCGTAGATACCATTATTTTT
										3B-0757990A-T7-48_896	GTACCAATACAAAGGTGTCCG
										3B-0757990A-T7-48_960	GCGTCGCGTGTGTGGAGGGG
										3B-0757990A-T7-48_1152	GCGCGCACCGTGCGCGCGCA
										3B-0757990A-T7-48_1216	ATTTTGTCAAAACCAAGCCCT
										3D-0882640-T7-48_640	tccagggggcaatttttaggcgtcctg
										3D-0882640-T7-48_704	TGCTAGTGCCAATGCCCCGCC
										3D-0882640-T7-48_832	AATGGGCAACCAAGCCTGGT
										3D-0882640-T7-48_896	AACCCTTAAGATACATGTTAGT
										3D-0882640-T7-48_960	GAGGCATGCAGATATTAATTAT
										3D-0882640-T7-48_1024	TGGTTGGATGGGTTGGAGGG
										3D-0882640-T7-48_1088	TTTGACGCTTTGATGTTTCATT
										3D-0882640-T7-48_1152	GATAATGAGGCACATGTGCTG
										3D-0882640-T7-48_1216	GTAGGCGCGGTGTGAGTGCG
										3D-0882640-T7-48_1280	GTTTCTAGAAAAACAAATAGT
										3D-0882640-T7-48_1344	TCACTATTCCTATATTAAATAAT
										3D-0882640-T7-48_1408	AGGCGATTTTTTACTTCTCCA
										3D-0882640-T7-48_1580	AAATACAATAGCACCCTCCAC
										2A-0283820-T7-49_0	GCTTCGGCGTTGGTCTCTTTC
										2A-0283820-T7-49_64	CCCGCTGGTACTTCCGCAGCT
										2A-0283820-T7-49_128	ATTTTTGTCTCGTCCAATATGT
										2A-0283820-T7-49_320	TTCGTTCGCACAGTCACCAAA
										2A-0283820-T7-49_384	CTGCTTATTCTACATTGGGACA
										2A-0283820-T7-49_448	TAAGCACAGAATTTTTCGACG
										2A-0283820-T7-49_512	CAATGGGCTGCTTAAGATCCG
										2A-0283820-T7-49_576	TGAGCAAACTGAACACACAC
										2A-0283820-T7-49_640	GGCCTCTGAATAGATGGTTAG
										2A-0283820-T7-49_832	tacaaagttgggtcatctattttggaac
										2A-0283820-T7-49_896	CGTCGACGGACCAGACTGAG
										2A-0283820-T7-49_960	ATTGCTCCACGGGTCGGACG
										2A-0283820-T7-49_1024	ACTTACTAATAAACAATGCTGG
										2A-0283820-T7-49_1088	GCAAGCAGGCAACATAATTAG
										2A-0283820-T7-49_1152	GTCCAACAAATGAGATGCAG
										2A-0283820-T7-49_1216	AAGGCAGGCACCGTTCCTTTT
										2A-0283820-T7-49_1408	TCTATCCATCACGCTGCACTTA
										2A-0283820-T7-49_1472	TGAAAGAAAAGAAAAAAGTA
										2A-0283820-T7-49_1536	AGAATGGATATTGCATCCGTG
										2A-0283820-T7-49_1580	ATATAAAGCGGAAGCTGCGAA
										2B-0383450-T7-49_64	ATAACGCTCGCTGGTACTTCC
										2B-0383450-T7-49_128	GACTTCATTTTTGTCTCGTCTA
										2B-0383450-T7-49_192	TCGACGTTCAATAGTACTATAA
										2B-0383450-T7-49_384	GTGGTGCCTCTGCTTATTCTC
										2B-0383450-T7-49_448	AATCCTGATAAGCACAGAATT
										2B-0383450-T7-49_640	GCCACGTGGCCTCGGAACAG
										2B-0383450-T7-49_832	GAGCAGACCACATGCAATGC
										2B-0383450-T7-49_896	AAGGTAACAAAGACTACAAG
										2B-0383450-T7-49_960	ACACAAAATAAACAATGCTGG
										2B-0383450-T7-49_1024	CAAACAGGCAACATAATGGG
										2B-0383450-T7-49_1088	TTCGTTTTCCCAGCATATCCCT
										2B-0383450-T7-49_1152	GCGGTAATTAGATACTGAAAA
										2B-0383450-T7-49_1344	CCATCCACTTGGTTTCTGCAG
										2B-0383450-T7-49_1408	CTTTAAATTTTACAAGAACCTG
										2B-0383450-T7-49_1472	CCTAGAAAGAGTTCCAAGTAG
										2B-0383450-T7-49_1536	TATAAAGTGAAAGCTGCGAAT
										2B-0383450-T7-49_1580	CCAACAGTACATCACTGCAAG
										2D-0546590-T7-49_64	ATAACGCCCGCTGGTACTTTC
										2D-0546590-T7-49_128	TACTTCATTTTTGTCTGGTCTG
										2D-0546590-T7-49_192	TCGACGTTCAATAGTACTACA
										2D-0546590-T7-49_448	GCGATGTGTTTTTGTGCGTCT
										2D-0546590-T7-49_512	TTTTTTTAACGCGCGTCTGTC
										2D-0546590-T7-49_704	GTGGTGCCTCTGCTTATTCTAT
										2D-0546590-T7-49_768	AAGGTCGCAGTAATCCTGATA
										2D-0546590-T7-49_960	CGATTCCCTGCGCCACGTGGC
										2D-0546590-T7-49_1024	TGATGCACACTTGAGAAGTG
										2D-0546590-T7-49_1216	CACGGGTCGGAAGGTAACAA
										7A-1805200-T7-50_128	GGGAAAGAGGGAGAGTGGC
										7A-1805200-T7-50_192	GCCCTTCAAATTAAAACCCCC
										7A-1805200-T7-50_256	AGCCTAGTGTGGGTCTCTCCC
										7A-1805200-T7-50_640	TATTCATGCCATCTTTGGCATT
										7A-1805200-T7-50_832	GCTAGCGTCTCCCCACAGTAG
										7A-1805200-T7-50_896	ccttcctTTAACACCGGGTCTGT
										7A-1805200-T7-50_960	AGGTATTAATGTCGCTGTTCAT
										7A-1805200-T7-50_1152	GGTTTCCGCCCAGCTTTACCT
										7B-1949880-T7-50_256	ggctttttcctagtagtgCGAGCAC
										7B-1949880-T7-50_384	TGTTTTGTGTGCTTGCAAAGT
										7B-1949880-T7-50_448	GAATCAACGTCATTCCATGAT
										7B-1949880-T7-50_512	AAATACAGATTTAAAAAAAC
										7B-1949880-T7-50_576	TTCTCCAAGAGAATGTAGAGG
										7B-1949880-T7-50_640	ACTTTTGCACGTAGGAAATGG
										7B-1949880-T7-50_832	CGCGGCGAGTGGGTAAACTT
										7B-1949880-T7-50_896	ACCTACCAATTCTGCCACTCA
										7B-1949880-T7-50_1216	ATGAATAGGCAGCGGGGCGC
										7B-1949880-T7-50_1344	ATGGCAAGGGGCGCATCCCT
										7B-1949880-T7-50_1472	GTCAGGAGGGAGAGAGAGG
										7D-2034840-T7-50_128	GAGAGAGGGGAAAGAGGGA
										7D-2034840-T7-50_192	AGGCTTCACCCTTCAAATTAA
										7D-2034840-T7-50_256	GGGtctctctctctctctctctctctct

										7D-2034840-T7-50_768	ccctcccctcccctctctcccctccTTT
										7D-2034840-T7-50_1024	CCCTTTTGTGGTAAAAAGAAT
										7D-2034840-T7-50_1152	GTGGAGAGTGGTCCTCTACAT
										7D-2034840-T7-50_1216	TCCGCAATGATTTAATTTTTTT
										7D-2034840-T7-50_1344	ATGGATGCCATTCCATGATAAA
										7D-2034840-T7-50_1408	AATCATTTTTTTGGGAGTTTA
										5A-1248610-T7-51_0	GCGGAGCCATCATATGAACAA
										5A-1248610-T7-51_64	CTGCAAGGCAAAGCGCAAAC
										5A-1248610-T7-51_128	GTCCTGCTTGTCATTGACTATG
										5A-1248610-T7-51_320	CTTGCTTGGCCGCCGAGTCAT
										5A-1248610-T7-51_384	AGGGACACATCAAGGATGTG
										5A-1248610-T7-51_512	GAGGCCGTGGGCAGTAAGAA
										5A-1248610-T7-51_576	GGGCGGCAAGAAGGAAAAT
										5A-1248610-T7-51_704	TTATGTGACTCGGGGTCGTAT
										5A-1248610-T7-51_832	ATCCTTAGCCCATCATAGTTCA
										5A-1248610-T7-51_896	GATGCTAGACTTACGACAGGA
										5A-1248610-T7-51_960	TAAGGAGAAAAGGTCCCATC
										5A-1248610-T7-51_1024	AATCAATTTGTAGAAGCTTTT
										5A-1248610-T7-51_1216	TGTTTATACGTGCATATGTATG
										5A-1248610-T7-51_1280	TTTGCTAAAGTTGCTGTGAGA
										5A-1248610-T7-51_1536	TTCCGGcctcctcctcctcctttcttc
										5A-1248610-T7-51_1580	cAAGTTTCGTCCTCCACCACTA
										5B-1294870-T7-51_0	AGGGACACATCAAGGATCGG
										5B-1294870-T7-51_128	GAGGCCATGGGCAGCGAGAA
										5B-1294870-T7-51_320	TTATGTGACTCGGGGTCGTATA
										5B-1294870-T7-51_384	ACATATCAACTGTTATTATGATT
										5B-1294870-T7-51_640	GCACGGATGGGAACATGGGA
										5B-1294870-T7-51_768	AAGGGGGTTGGTTGGTGAGA
										5B-1294870-T7-51_960	TTTTTTAAAGGTGTTCATAAG
										5B-1294870-T7-51_1280	TTTGCTAAAGTTGCTCTGAGA
										5B-1294870-T7-51_1536	CGGcctcctcctcctcccctcctttct
										5B-1294870-T7-51_1580	CCCCAAGTTTCGTCCTCCACC
										5D-1436310-T7-51_256	ctcctttccaaaagaaaaaaGCAG
										5D-1436310-T7-51_320	CGCACCATATAATATGAAGGCT
										5D-1436310-T7-51_384	CATAGTTTTATTACATGTTGGT
										5D-1436310-T7-51_576	ACGCGTGCACGCATCTTGCTT
										5D-1436310-T7-51_768	TTGGCAGTGATAGTGGACGC
										5D-1436310-T7-51_832	AAACATATTAGATTATGGGCG
										5D-1436310-T7-51_960	GCGGCTCTCTCCGTAAATTATG
										5D-1436310-T7-51_1024	ATTATAGGCAAAAATTAACATA
										5D-1436310-T7-51_1088	GTTAGATAGACAGTGGTATCC
										5D-1436310-T7-51_1152	TATTTTTTTATAAAGATGCTAG
										5D-1436310-T7-51_1216	GGAAGAGAGGTCCCATCAGA
										5D-1436310-T7-51_1280	TATAGCATTTTTGTTTTGTGGA
										5D-1436310-T7-51_1408	TCATAGGGATATGGTGTGGGT
										5D-1436310-T7-51_1472	AGTGTTTGCGCTTATACTATGT
										6A-1515830-T7-52_576	tgggaagaggcacagggcctttgccta
										6A-1515830-T7-52_640	CCGTCGATGCCTTTCCCGTGA
										6A-1515830-T7-52_704	CCACTGTAGCCGTTCTCTGCC
										6A-1515830-T7-52_768	GTAGCCATCGTCCCCGCCCCA
										6A-1515830-T7-52_832	CCGAGCGACAATCGCCTTGCG
										6A-1515830-T7-52_1344	GGCCCGTTTGGCCAGGTCCA
										6A-1515830-T7-52_1536	TTGACTTCTTCCCCGTCTTCAA
										6B-1594430-T7-52_64	GCTGCCTGGGTGGGCAGTGG
										6B-1594430-T7-52_256	GGCGATTGTCGGGGAGTTTC
										6B-1594430-T7-52_1280	TTGTGGGTTTTAAAAAAATACC
										6B-1594430-T7-52_1408	CTTTTTTTTAAATCCTCTCAGT
										6B-1594430-T7-52_1472	TACTAAACAGCAAGTCTTCTTT
										7A-1847780-T7-53_0	AGCATGAATTCCTGCCGTCTC
										7A-1847780-T7-53_64	GACGAGATCTAGTGTTAGACA
										7A-1847780-T7-53_128	ATGGTGTTGGCTCTTAGAGGC
										7A-1847780-T7-53_192	GCTCTAGTACAGAAACAACGA
										7A-1847780-T7-53_256	TTGGTGGTTTAGAGACCTTGA
										7A-1847780-T7-53_320	TTTTATAAAAGGTTTAGATATT
										7A-1847780-T7-53_384	TCAGGAAAGTATACTTTGCCT
										7A-1847780-T7-53_448	GCTTTCAAAAAATTAATAATCG
										7A-1847780-T7-53_576	CCCTCCCTCCGTCTTTGGACT
										7A-1847780-T7-53_768	CCACGAATCCATCGGTCACCC
										7A-1847780-T7-53_832	CAATCGGCACCACCCAACAG
										7A-1847780-T7-53_896	CCCCAACCAGCCGAGTGATTA
										7A-1847780-T7-53_1216	GCTCCTGGGGGAGGAAAAG
										7A-1847780-T7-53_1536	GTTTATATACTAGGCGCACAC
										7B-1937780-T7-53_64	ATACTTCCGATGCACTTTCATA
										7B-1937780-T7-53_128	CCAGCCTATGCATAATGCCTGA
										7B-1937780-T7-53_192	TGCTCATCAGGATATAATAAGT
										7B-1937780-T7-53_256	CCAGTTCCATCAATCGCTTTCA
										7B-1937780-T7-53_320	TGCCTAGCACGCAAAGACGAT
										7B-1937780-T7-53_384	AGAGAAACTTGTTTTTTTTTG
										7B-1937780-T7-53_448	TTCGAGTTTCGACTGTAGGGC
										7B-1937780-T7-53_704	CTCCTGTTCCCTCCACGACGC
										7B-1937780-T7-53_768	GAGATTAGCAGTAAGATTAAT
										7B-1937780-T7-53_832	TAACCACCCGCAACTGTTCAC
										7B-1937780-T7-53_1152	GCCAGTACTGTTCCGATCCGC
										7B-1937780-T7-53_1580	CGCAGAGGGTAAAAATCTAG
										7D-2038090-T7-53_128	AGGCGAATGCAAAGTGGCTA
										7D-2038090-T7-53_192	GATGCTCTTAGCACCGCATTGT
										7D-2038090-T7-53_256	ATATTTCGCAAAAATGAAACTT
										7D-2038090-T7-53_320	AGACGTGTTAAGCAGGAAAT
										7D-2038090-T7-53_384	TGCCTTGAGCAACCACTCTTT
										7D-2038090-T7-53_448	TACTCGTTTTCATAGAAAAGC
										7D-2038090-T7-53_576	CGACTGTAGGGCACAGCTGT
										7D-2038090-T7-53_768	ACCCCTCCTGTTCCCTCCACG
										7D-2038090-T7-53_832	CAGGCAAAAGATTAGCAGTA
										7D-2038090-T7-53_896	ATCAAGCTAACCACCCGCAAC
										7D-2038090-T7-53_1152	CACCCTTCCTTTCCTAATCGGG
										7D-2038090-T7-53_1216	CCGGGGGAGGAAAAGGACC
										7D-2038090-T7-53_1580	CTAGCCAGCCGGTGCTCCttcg
										1A-0073500-T7-54_0	CGCCGAGATCACCACTTCCAA
										1A-0073500-T7-54_256	CCTATTCCTACGGCCGCTAGCT
										1A-0073500-T7-54_512	GGTGGCAGCCGGTTATCCTTA
										1A-0073500-T7-54_576	ATCTCAAGTCTCCTACTAGCTT
										1A-0073500-T7-54_640	AAAGAAAGTCAATTATTTCAA
										1A-0073500-T7-54_704	AATTTCGATGATGTTTTCAAG
										1A-0073500-T7-54_768	ACATTTCTACGATTCTATTGAA
										1A-0073500-T7-54_832	ATTAGATGAGAAGAATACAGA
										1A-0073500-T7-54_896	GCGACGAGTTTTTTTGATCG
										1A-0073500-T7-54_1024	CGTCTACGTTCCTATCCACAGT
										1A-0073500-T7-54_1088	TGGATAACGAGCTAGCTCAGA
										1A-0073500-T7-54_1152	AAAAGAGACATTTGCCGATCT
										1A-0073500-T7-54_1216	GTAACGGACCAATCTTTCCAG
										1A-0073500-T7-54_1280	TCTTATCCTCACTTAGAAGATG
										1A-0073500-T7-54_1344	AGGAGCTAGCTCGGATTGATT
										1A-0073500-T7-54_1408	TATTGATAAGACCTTTAGAAA
										1A-0073500-T7-54_1472	TCTTTGCGGCTCATTAGCTTAG
										1A-0073500-T7-54_1536	TTGAAGATGGAAGAATATTAA
										1A-0073500-T7-54_1580	GTGGATAACGAGCTACCTTGG
										1B-0159520-T7-54_0	TTTAGCAGGGCCAGCCTTTCT
										1B-0159520-T7-54_64	AAAGCTTAATTGATTTCGGATA
										1B-0159520-T7-54_128	ATGCGCCTCTTAATGTTCTTCC
										1B-0159520-T7-54_192	CAAGCACGCTAAGCTAATGAG
										1B-0159520-T7-54_256	GGTCGGGCTTTCTAAAGGCCT
										1B-0159520-T7-54_320	ATTGAGTTCGAATAAATTGAT
										1B-0159520-T7-54_384	CTTAATGTTCTTCCATCTTCTA
										1B-0159520-T7-54_448	GACAAGCTGATGAGCTGGAA
										1B-0159520-T7-54_512	GCTTTCTAAATGCCATAGCGA
										1B-0159520-T7-54_704	GATGAGCTGGAAAGATTGTC
										1B-0159520-T7-54_768	GGCGAATTGCGCAGACAACC
										1B-0159520-T7-54_832	GCAGGGAATGCCTCTGTATTC
										1B-0159520-T7-54_896	TCCTAGGAGTGTTCAATAGAA
										1B-0159520-T7-54_960	TGTCCTCAAAATTCTTGAAAA

1B-0159520-T7-54_1024	GAAGCAGTAGATAGTTTGAA
1B-0159520-T7-54_1280	GGCCGTTTCCCACCGCCGCTC
1B-0159520-T7-54_1580	AAAAGTAGCAGAGCAAGCGA
1D-0202460-T7-54_0	ACCCACCTCTTCTTCCCTCTCT
1D-0202460-T7-54_320	TGTCCGGTGGCGGAACTCGG
1D-0202460-T7-54_384	TACAGTACGTGACCGGAGCG
1D-0202460-T7-54_576	TGACATGTCTCGTGCGCTTCA
1D-0202460-T7-54_768	TGTCGTGCCACACACCGTCCA
1D-0202460-T7-54_832	CAATGAACTGCAGCAAAATCT
1D-0202460-T7-54_896	GATGTTTTGGCAGGAGTAGG
1D-0202460-T7-54_960	CAAACATggttgtggcgcagttggc
1D-0202460-T7-54_1024	cctccatcaccacaACAACCGGG
1D-0202460-T7-54_1088	GTCGCATGCATCTGATTTGCA
1D-0202460-T7-54_1280	acgtgataaccaactacactacgaaa
1D-0202460-T7-54_1344	TTACGTGCTATGCATTTCACAT
1D-0202460-T7-54_1408	ACTAGCTGACATGGCAGGAG
1D-0202460-T7-54_1472	CACACAACCTGAGGGACATA
1D-0202460-T7-54_1536	CCATCAGGAGAGTTGGGCAC
3A-0637570-T7-55_960	AGCCGTCACCAACGGTAGCCT
3A-0637570-T7-55_1088	AAGTCAGACGCCCGCCTTGTG
3A-0637570-T7-55_1408	CTGACTGAGTGGCTGACTGG
3B-0742350-T7-55_819	TGCCCTGCCACCGTACCCCGT
3B-0742350-T7-55_882	CGTCACGCCACGCCTTTTTTT
3B-0742350-T7-55_945	AACAAATTGAGACGCCCGCCT
3B-0742350-T7-55_1575	GATCAGGCACGGCAGGCTAG
3B-0742350-T7-55_1638	GTTTGTTTGCTTTTTGTTCATT
3B-0742350-T7-55_1701	GCACCCAACCGGCGGACCCA
3D-0844620-T7-55_512	CCCGTCAGCCGTCACCAACGG
3D-0844620-T7-55_576	AACGGTGCTGAACGAAAACG
3D-0844620-T7-55_1280	TTCGATTTAAGAGCAACGCGC
3D-0844620-T7-55_1344	AACTGGCTGCCCGTTCATCGT
3D-0844620-T7-55_1408	CATCCGCGCAGGAAACAGTTT
3A-0627440A-T7-56_0	CATCGACGACCCTGTTGTTGG
3A-0627440A-T7-56_64	GAATGGAGAGAGCACTAGTA
3A-0627440A-T7-56_128	TATTGCTTAAAACAAAAGTGC
3A-0627440A-T7-56_192	GATAAAACACATATCGACGTT
3A-0627440A-T7-56_256	ATACAATGTGCCCCAACTTAG
3A-0627440A-T7-56_320	AAGCAACTATTTACAAATTAA
3A-0627440A-T7-56_384	GTGATAACATAAAACACATGT
3A-0627440A-T7-56_448	TTTGCATACAATGCACTTCAA
3A-0627440A-T7-56_512	ATTTAAGGTGTCACGGCCGGT
3A-0627440A-T7-56_576	GAACTTCATACTTATGTCATCA
3A-0627440A-T7-56_640	ACATGATTGTTGAGAACATTT
3A-0627440A-T7-56_704	GGTGAGGCTTAGTTTGTCAAG
3A-0627440A-T7-56_768	TGCATCTTTGTATTTCTAGAAG
3A-0627440A-T7-56_832	AATTGTTTTTAAACATGAACCA
3A-0627440A-T7-56_896	GGAAAGAAAGGGAAAAAAAT
3A-0627440A-T7-56_1152	CAGGTCACGTCTTGGTATAAC
3A-0617190B-T7-56_1216	CGTCCGGCGCCGCATGCCCCA
3A-0627440A-T7-56_1216	ACTAAATTGAACTCTTGATTCG
3A-0617190B-T7-56_1280	GCGGGGCGGTCGTATTGGGT
3A-0627440A-T7-56_1280	GGGAGTATTTTTTGCAAGAA
3A-0617190B-T7-56_1472	aacccaccccaaccccccgaacccca
3A-0617190B-T7-56_1536	TGCGGCGCCGGTCTATAATAAT
3B-0769340-T7-56_0	TGTTCTAACGAACCCTGACACA
3B-0769340-T7-56_64	AAGTTTTAATTTCATTTTTCTT
3B-0769340-T7-56_128	ACTCATAGACAATCAATAATAT
3B-0769340-T7-56_192	TGACCTTAAGCGACAACGGC
3B-0769340-T7-56_256	AAACATATCATATAATTAACATT
3B-0769340-T7-56_320	TCGTAGACAATCAATATTATCC
3B-0769340-T7-56_384	ATTTATTTTTTGACCCGACATC
3B-0769340-T7-56_512	GCCATTGGGCTACTGCCCTGT
3B-0769340-T7-56_576	GAGTTGTATACAATGTACTCCT
3B-0769340-T7-56_640	TTTAAGGTTACAGGCCTAGTG
3B-0769340-T7-56_704	AGCTTCATACTTATGTCATCAG
3B-0769340-T7-56_768	CATGATTGTTGAGAACATTTT
3B-0769340-T7-56_832	GATGAAAGGTTACAACATTGG
3B-0769340-T7-56_896	ATGATAATTGCACATCTTTGTT
3B-0769340-T7-56_960	ATCTTCAAAAAATATATTTTTA
3B-0769340-T7-56_1088	ATGTCTAGCTACATACGAATTA
3B-0769340-T7-56_1152	TTGACAAGACATGATAAAGTA
3B-0769340-T7-56_1216	TTGAAATGTTATCTAAACAAAT
3B-0769340-T7-56_1280	AGAATGGAAATGACCAGCAA
3D-0892640-T7-56_0	CATTGCACGGGAGCATTAGAG
3D-0892640-T7-56_63	AGGGTCATAACCCTAGGGCTC
3D-0892640-T7-56_126	TTCTTCAACCCCTCTCCCCCTC
3D-0892640-T7-56_189	CTCATTGCCGGGCATGTTTGT
3D-0892640-T7-56_252	CGATGAATGGAAAGAGCACTA
3D-0892640-T7-56_315	ATTATTTGAAACAGAAGTACTA
3D-0892640-T7-56_378	GATAAAACACATATCAACTTTG
3D-0892640-T7-56_441	ACAATGTACCCCAAATTAGAA
3D-0892640-T7-56_504	ATTAACATTAAAGCAACTATTT
3D-0892640-T7-56_567	GGACAACCTCGAGTTGTATAC
3D-0892640-T7-56_630	CAAAAAAAAAAGCTTCATACT
3D-0892640-T7-56_693	TACAACGATGCAAGCAAAAGT
3D-0892640-T7-56_756	AACAACATTGGCTCAGTTTGT
3D-0892640-T7-56_819	AATTACGCATCTTGTATTTCTA
3D-0892640-T7-56_882	AATAGAGGGTGTATATTTTAG
3D-0892640-T7-56_945	TTTAAACGTGAATCATCACATT
3D-0892640-T7-56_1008	AAAAACATCCAGAGACTCATC
3D-0892640-T7-56_1071	TAATTAAATTGAACTGTTGATT
3D-0892640-T7-56_1134	AATGGGGAGTATTTTTGCAAG
3D-0892640-T7-56_1190	GGCAAACTAGGGGTACGGCA
3B-0782160-T7-57_567	GAGGCGTCGTAAACGCTTTTT
3B-0782160-T7-57_630	TATACTCTGAGTAACATCTCGC
3B-0782160-T7-57_693	TTTTTCCCTCAATCGCTATCTCT
3B-0782160-T7-57_756	CTCAACTCATATTGGTTGCTCT
3B-0782160-T7-57_819	GATCATTAAGATTGTGAACTAT
3B-0782160-T7-57_882	AGCTCGCTCTCTTTATCTCTCG
3B-0782160-T7-57_1236	ATACTCTCGGTTCTAACATAGT
3B-0782160-T7-57_1294	GTGCTAGCGTTGGATGACCGG
3B-0782160-T7-57_1468	AGCGAATCCTATCATCATCAAC
5A-1242240-T7-58_64	TTTTGTATTCTACTTTCTTTACC
5A-1242240-T7-58_128	TAATTTGTTTAATTGCAGATTT
5A-1242240-T7-58_256	ATGGCACGAATGTTGATTCTC
5A-1242240-T7-58_320	GAAATTGGAGACAACATCCAT
5A-1242240-T7-58_384	TGTGTCTTCACTCTTTACACTA
5A-1242240-T7-58_640	GCATGGCCGCCCGACTCTATG
5A-1242240-T7-58_1216	AGAGTATCATTTTTTAGATGAT
5A-1242240-T7-58_1280	TTTAAGAGAAGGATCACGATT
5A-1242240-T7-58_1344	GCATATGCATCATCTAGTTTCC
5A-1242240-T7-58_1408	TTTTCTTTTGAGATGGTAGTTT
5B-1296540-T7-58_576	AGTTTTAGGCCCGCTGATGTT
5B-1296540-T7-58_896	GTACCCGTATTAAGGGTTGTT
5B-1296540-T7-58_1024	TTTTTTTTTGCGGGAAAACAC
5B-1296540-T7-58_1152	ttggaacggagggagtaTATGCTAG
5B-1296540-T7-58_1408	aatgatctaaaactacgcgaatgactt
5D-1445360-T7-58_128	CACCACCCAATCAAAAGGTAT
5D-1445360-T7-58_192	ATGCACACGGTCTTGACGCGA
5D-1445360-T7-58_256	AGTGTCGCTCGCCACAGCATT
5D-1445360-T7-58_320	TCCCAAGTCTGGGATGCTTCG
5D-1445360-T7-58_384	TGCATCAGACGACACCATCAT
5D-1445360-T7-58_448	ATATCAAGGGAAAGGACATGA
5D-1445360-T7-58_512	AGCCCACAAAGGAACCCAAC
5D-1445360-T7-58_576	AGTCGACACAGCCGCCATATC
5D-1445360-T7-58_640	TGGGCGAAATGCAACTGCTG
5D-1445360-T7-58_704	GGGCGTGTTCCCCATGTTGAA
5D-1445360-T7-58_768	AGGCCGCCGTGCAGCCAAGA
5D-1445360-T7-58_832	ACATCCACGATCTGGCGCTGC
5D-1445360-T7-58_1024	CGGATCGAGAGGCCTCCCGA
5D-1445360-T7-58_1088	CTAGTAGACAAACGATATCTC
5D-1445360-T7-58_1152	AGTATGAATCTTTGAATAAGA
5D-1445360-T7-58_1216	ATGTAAAATATCATCAAGCGTT
5D-1445360-T7-58_1280	GATGGTTGGATCACGTTACTG
5D-1445360-T7-58_1344	CACGATTAATTATGAATGGTAG
5D-1445360-T7-58_1408	TAGTTTCCATTTCCCCTAAAAA

Trait&gene no.	Gene	Homoeologues	IWGSC gene ID (refseq1.1)	WGIN Promotome ID	Orientation	ATG	Target Sequence	MYbaits	MYbaits coverage (%)
T8-1	Myb-TF	ABD	TraesCS3A02G187800	3A-0698810-T8-1	reverse	225,895,116	TCTTGGTCTCTCTCTCTCTCTGTCAAGGTTCAAGGAGGAGGATTGG	21	91.1
T8-1	Myb-TF		TraesCS3B02G217100	3B-0714880-T8-1	reverse	258,721,797	TCTTGGTCTCTCTCTCTCTCTTTCTAAGGTTGAAGGAGTCTTAGGGA	16	73.4
T8-1	Myb-TF		TraesCS3D02G191400	3D-0901830-T8-1	reverse	180,471,277	TCTTGGTCTCTCTCTCTCTAAGGTTCAAGGAGGATTGGAGAGGGG	21	87.8
T8-10	AGL29	ABD	TraesCS2A02G261200	2A-0267480-T8-10-1	reverse	411,950,117	ATCCTGATTTCGCGATCTAGCTGTCGGCTCTTGTGTCTATGGCGCGC	17	72.7
T8-10	AGL29		TraesCS2B02G281000	2B-0405830-T8-10	forward	387,698,566	TGGCCCCTCCGACGTGAGGCGGAGGGGGTGATAACTCTGACTAGC	16	76.7
T8-10	AGL29		TraesCS2D02G262700	2D-0506180-T8-10-2	forward	318,911,020	AACCACGGCCCAACGGTCGAACAACGACACCACCCAACGCGACCC	8	40
T8-11	SOC1	ABD	TraesCS1A02G199600	1A-0024490-T8-11	reverse	358,771,740	CCTTCCTGCCGCTGGCGGAGCTCGATCGATCCCCTCTCCTCCGACG	3	17.8
T8-11	SOC1		TraesCS1B02G214500	1B-0109140-T8-11	reverse	389,224,699	CCTTCCTGCCGCTGGCGGAGCTCGATCGATCCCCTCTCCTCCGCCG	6	35.8
T8-11	SOC1		TraesCS1D02G203300	1D-0189260-T8-11	reverse	287,467,974	CCTTCCTGCCGCTGGCGGAGCTCGATCGATCCCCTCTCCTCCGCCG	2	14.1
T8-12	LFY	ABD	TraesCS2A02G443100	2A-0322780-T8-12	forward	693,231,203	ATCAGAAAGAGTGTTTTTCTCGGACTAAGACATCAAGTAAATCAAA	18	77.6
T8-12	LFY		TraesCS2B02G464200	2B-0418280-T8-12	forward	658,198,819	TTCCTCTTCAGTGACTAATTCAGAATCTAGTCAAAAATCAAGGCTCG	17	73.9
T8-12	LFY		TraesCS2D02G442200	2D-0536540-T8-12	forward	552,567,149	TAAATCAAACTCCATAAGGAGGGCCTATTTCTTTTTTTCTATATCAGC	16	70.1
T8-13	PIF	ABD	TraesCS5A02G376500	5A-1218580-T8-13	reverse	574,198,820	CTTCGCCCCTAGCGGGTAGTAGCCCACCCCCTGCGCGAAATGCTGC	6	34.6
T8-13	PIF		TraesCS5B02G380200	5B-1295610-T8-13	reverse	558,122,336	GGCTCAGATCATCACAAGCATCGACCGGCGATTCACTTCACCGCAG	12	61.6
T8-13	PIF		TraesCS5D02G386500	5D-1455110-T8-13	reverse	456,343,206	GGCTCAAATCATCACAAGCATCGACCGGCGATTCACTTCACCGCAG	10	47.1
T8-14	SUT1	ABD	TraesCS4A02G016400	4A-1020440-T8-14	forward	11,185,434	TTCCATCTGTGAACCTTTATTCTCCATGCTTATCATTTCTGAAGTTTG	19	86.8
T8-14	SUT1		TraesCS4B02G287800	4B-1056170-T8-14	reverse	571,473,792	CGTCGCAGGGGGGCCGCCTGGACGATCACCACCTCGTTCGTTCGC	15	68.5
T8-14	SUT1		TraesCS4D02G286500	4D-1131760-T8-14	reverse	457,506,141	GGCAGAGAGGCCGCCCTGGCGATCACCACCTCGTCCGTTCGCCTA	17	80.5
T8-15	TaSnRK1	ABD	TraesCS1A02G350500	1A-0026620-T6-29-2	reverse	535,316,931	ATAATGATATTATGATAGAAGCAGACAAAAACAAAGTATCCACAGTC	23	50.5
T8-15	TaSnRK1		TraesCS1B02G364800	1B-0108930-T6-29	reverse	595,184,659	CGTCGACCTAAAATAAATCACAGCCAAGCGATTCAGCGGCAAAGC	7	35.1
T8-15	TaSnRK1		TraesCS1D02G353300	1D-0193790-T6-29-1	reverse	439,028,615	CGTCGGCCTAAATCACAGGGGCAAAAAGGAATTAAAAGCTATTATC	7	36.1
T8-16	SnRK	ABD	TraesCS3A02G282800	3A-0649780-T6-30	reverse	511,078,383	CTTCCACCTTAATCGTAATCACACCAAAGTACACTTCAGGAAACGA	6	32.5
T8-16	SnRK		TraesCS3B02G316500	3B-0775050-T6-30	reverse	508,255,985	CTTCCACCTTAATCGCAATCACACCAAACTACACTTCAGGAAATGA	9	43.8
T8-16	SnRK		TraesCS3D02G282800	3D-0889780-T6-30	reverse	390,669,972	CTTCCACCTTAATCGCAATCACACCAAACCACACTTCAGGAAACGA	7	46.1
T8-17	VRS1	BD	TraesCS2B02G405700	2B-0439760-T1-5	forward	573,974,813	ATGTTGCCACCCATTCCTCCTACCATGTCCGCCGCCCATCATGTTACT	12	57.4
T8-17	VRS1		TraesCS2D02G385500	2D-0323970-T8-17	forward	490,117,422	CGTACCCTATCATAATCTCTTCGTTTGTTCTCCGCTATTAGTTACTTTG	2	14.1
T8-18	VRS2	ABD	TraesCS5A02G319200	5A-1225120-T8-18	reverse	530,302,699	GCGCCCAAATCCATAGGCCGCCGTGCCGATCTCCCTCTATTTCACTC	2	14.1
T8-18	VRS2		TraesCS5B02G319600	5B-2096450-T8-18	reverse	504,307,399	GCGCCCAAATCCATAGGCCACGGTGCCGATCTCCCTCTATTTCACTG	5	24.2
T8-18	VRS2		TraesCS5D02G325400	5D-1396820-T8-18	reverse	417,041,160	GCGCCCAAATCCATAGGCCGCCGTGCCGATCTCCCTCTATTTCACTC	6	32.5
T8-19	VRS4	ABD	TraesCS3A02G093200	3A-0686290-T8-19	forward	59,532,166	TTTTTGTTTTGTTTTAAGCATAGCTTGAGTGATTAAGATCATGTACTC	14	75.8
T8-19	VRS4		TraesCS3B02G108500	3B-0813300-T8-19	forward	74,294,161	ATAGCTTGAGAGATTAAGATGTACTGTCGACAATAGTAATTGAGTG	13	68.7
T8-19	VRS4		TraesCS3D02G093500	3D-0903560-T8-19	forward	47,493,516	TAATTGCTTTTTTTGTTTTGTTTTAAGCATAGCTTGAGTGATTAAGAT	18	80.9
T8-2	bHLH-TF	ABD	TraesCS2A02G212200	2A-0342330-T8-2	reverse	197,089,756	TGCTCGCTGGCCGGCCGCTAGGAGGGTCGATCGAGGAAAAAGATC	12	58.4
T8-2	bHLH-TF		TraesCS2B02G237300	2B-0488170-T8-2	reverse	237,982,210	TGCTCGCTGGCCGCTAGGAGGGTCGATCGAGGAAAACGATCAATC	10	50.8
T8-2	bHLH-TF		TraesCS2D02G218100	2D-0589930-T8-2	reverse	181,324,289	TGCTCGCTGGCCGCTAGGAGGGTCGATCGAGGAAAAAGATCAATC	14	75.8
T8-21	Q	BD	TraesCS5B02G486900	5B-1364080-T8-21	reverse	658,095,066	CTGGCAAAAAGCACCACCACCACCCACAGGATATCAACCATCGGAC	13	59.8
T8-21	Q	no A	TraesCS5D02G486600	5D-1440480-T8-21	reverse	521,716,353	CTCCGATCCGAGGCCCGCCGGGCGCCTCCGAAACTCGCTTGACCC	18	74.4
T8-22	TPS1	ABD	TraesCS1A02G339300	1A-0025400-T8-22	reverse	529,249,639	GGACCGCCGCGACGACTCCCCCGCCGGCTGATTCGGCCTGCAGCA	11	56.7
T8-22	TPS1		TraesCS1B02G351600	1B-0123110-T8-22	reverse	582,099,119	GCAGCAGGGCCGTCATGCCTCGAACTCGCACTCTTGTTTGAGAGC	15	65.8
T8-22	TPS1		TraesCS1D02G341100	1D-0192320-T8-22	reverse	430,982,287	GAACTTCCCAGACGACTCCCCCGCCGGATGATTCGTCCTGCAGCAT	18	54.8
T8-23	MADS6	BD	TraesCS6B02G286400	6B-1607090-T8-23	forward	516,075,219	TTTGTATCTCGTGCTACTCGTGCTTCGATGATCTAGACCTTGTGTGAT	12	51.8
T8-23	MADS6	no A	TraesCS6D02G240200	6D-1683650-T8-23	forward	341,645,548	CCAGCACGAGGTGTCCGCCGTAGCAGAGCTGGGATTGGACGAGG	3	14.6
T8-24	LRR PK	ABD	TraesCS2A02G058300	2A-0356340-T8-24	reverse	24,411,845	GTATACATGATTTATGGCAAGAAAGATTTAATTGCACCAAACTGAA	19	81.4
T8-24	LRR PK		TraesCS2B02G071100	2B-0495770-T8-24	forward	37,677,018	TTGTCCAATCAAGTGATAGATCCACTCCTTCTCCTTCTTTGAACCAA	19	50.7
T8-24	LRR PK		TraesCS2D02G057300	2D-0600290-T8-24	reverse	22,820,352	GTTATTTTCCTTGAGATTTATGGCCTCCAGAGAAACCAAGTTGCTG	17	79.3
T8-25	FKBP19-like	ABD	TraesCS2A02G053200	2A-0347840-T8-25	forward	21,261,151	AAAGGCGTTTTTTTTCGTTACGAGAGCCACGGCCGTACCTCCTACA	19	81.4
T8-25	FKBP19-like		TraesCS2B02G067100	2B-0474560-T8-25	forward	33,848,418	TCCTGTGAAATTTTCATAAAATTCAATCATCAAATGGTCAAGCGGTG	16	73.4
T8-25	FKBP19-like		TraesCS2D02G053400	2D-0604370-T8-25	forward	20,778,121	CTTATTTACTTTATATTTTGTATGTAATAAACTAATATTCGATGTGGTC	22	92.7
T8-26	UDP-glucosyltransferase	AD	TraesCS6A02G018200	6A-1562640-T8-26	reverse	8,869,059	TCCCACGTGCTAGCTTTTCGTGCCGGGTCGGCAAGGGCTTTGGCC	15	71.8
T8-26	UDP-glucosyltransferase	no B	TraesCS6D02G021100	6D-1728140-T8-26	reverse	8,348,928	TCCCACGTGCTAGCTTTTCGCGCCTGGTCGGCAAGGGCTTTGGCC	12	58.4
T8-27	TaSVP	ABD	TraesCS7A02G175200	7A-1847780-T7-53	forward	128,826,522	AGCATGAATTCCTGCCGTCTCCTTTGGGCGGCGATGTTAGGAGATC	15	69.2
T8-27	TaSVP		TraesCS7B02G080300	7B-1937780-T7-53	forward	90,189,613	GCGGTGATAGTCGTTTGGTGATCTAGAGACCTTGATGTAATTTGCA	12	51.3
T8-27	TaSVP		TraesCS7D02G176700	7D-2038090-T7-53	forward	128,912,416	GCAGGAGGAGGATGAGCCGGAGCCCGGCACCGTCGTCGTGGACA	13	58.4
T8-3	PHD-TF	ABD	TraesCS5A02G233600	5A-1230310-T8-3	forward	448,836,171	ATTCCTCAGTGAGCTAGTATGCCCAAAGTGTGTAGGAGGCAGCTGC	16	52.1
T8-3	PHD-TF		TraesCS5B02G232100	5B-1351010-T8-3	forward	409,059,711	CAGGTAATACCATCTTTTTATCTTCATTCTATTCCTCGGTGAGCTAGT	17	77
T8-3	PHD-TF		TraesCS5D02G240500	5D-2092170-T8-3	forward	348,746,111	CTTGCTTGCAGCTAGGCAGGTAATACCATCTTTTTATCTTCATTCTAT	14	69.2
T8-4	Myb-TF	ABD	TraesCS3A02G251200	3A-0633670-T8-4	reverse	470,830,017	CTCCTCTCTCCTCACTCAACTACTACACAGTTCACACTCACCGCAGG	14	65.9
T8-4	Myb-TF		TraesCS3B02G280700	3B-0729150-T8-4	reverse	451,361,866	CTCCTCTCACTCAACTACTCACACTCACTGCAGGCTGCACCGGTTAT	13	72
T8-4	Myb-TF		TraesCS3D02G251600	3D-0854320-T8-4	reverse	352,368,195	CTCCTCTCCTCTCACTCAACTACTACTTCACACTGACGGCAGGCTGC	14	72.5
T8-5	Myb-TF	ABD	TraesCS4A02G113000	4A-1023210-T8-5	reverse	137,524,833	CCCGGCCGGCACCACCCTCCTCCCCCTCCCCTTCTACAACAAGCAG	6	60.1
T8-5	Myb-TF		TraesCS4B02G191200	4B-1064780-T8-5	forward	413,499,439	GCCGTTAGTAGCACCCGACATGGCCACGCCGATCTTGGACGCCATC	9	50.4
T8-5	Myb-TF		TraesCS4D02G192400	4D-1127960-T8-5	forward	335,348,469	TCGTCGCCGTCAGAGACAGCAAGCGCGCTCCCGTTGCCGTTGCCAT	9	47.1
T8-6	Myb-TF	ABD	TraesCS2A02G331200	2A-0305670-T8-6	forward	564,218,234	GAAGGTTGCCCGGAGAGGATAGACACCTTCAACCAGATAGCACAC	6	25.9
T8-6	Myb-TF		TraesCS2B02G351000	2B-0379970-T8-6	reverse	498,311,009	GTCGGCGCGTCCGCCTCCGAGGCCGCCCTGCTCGCGCGCGGGATC	5	25.4
T8-6	Myb-TF		TraesCS2D02G331700	2D-0529420-T8-6	reverse	424,015,517	GTCGGCGCCTCCGCCTCCCGGGCCGCCCTGCGCGCGCACGGGGTC	9	40.5
T8-7	bHLH protein	ABD	TraesCS6A02G071200	6A-1566480-T8-7	reverse	39,049,342	GGCTCAGCTCAGCCCAGCTCAGCACCAGTGGTCAGCTTGTCCTAGC	14	79.6
T8-7	bHLH protein		TraesCS6B02G095600	6B-1659330-T8-7	reverse	71,708,086	GGCTCAGCTCAGCCCAGCTAAGCACCAGTGGTCAGCTTGTCCTAGC	13	70.8
T8-7	bHLH protein		TraesCS6D02G069300	6D-2140990B-T8-7	reverse	34,736,391	GGCTCAGCTCAGCCCAGCAAAGCACCAGTGGTCAGCTTCCTAGGA	15	72.9
T8-8	AGLG1	ABD	TraesCS5A02G391800	5A-1229620-T8-8	reverse	588,023,759	CTCCGACCTCCGACCTCTGATCTCCTCCTCCCGAACCTCTCGCCCTC	8	38.8
T8-8	AGLG1		TraesCS5B02G396700	5B-1326650-T8-8	reverse	574,061,208	CTCCGAGACCACTGATCTCTTCCCCCTGAACCTCTCACCCTCCTCAC	9	42.6
T8-8	AGLG1		TraesCS5D02G401700	5D-1405530-T8-8	reverse	467,424,628	CTCCGAGACCTGTGATCTCTTCCCCCTGAACCTCTCACCCTCCCAAC	7	39.5
T8-9	AGL10	ABD	TraesCS2A02G174300	2A-0364910-T8-9	reverse	132,876,424	CTCCGGCCTGCCTCCAACCCGCCTCCCCGGCTCCCTCACTCGGCG	11	51.3
T8-9	AGL10		TraesCS2B02G200800	2B-0494980-T8-9	reverse	180,042,222	GGAATCTTGGGCTGTCGCTGGTGTAGGTGACGGTGAGCTTGTTGT	14	72.5
T8-9	AGL10		TraesCS2D02G181400	2D-0567240-T8-9	reverse	126,149,540	CTCCGCCCTGCCTCCAACCCGCCTCCCCGGCTCCCTCACTCGGCG	12	61.6

MYbaits ID	MYbaits sequence
3A-0698810-T8-1_0	TCTTGGTCTCTCTCTCTCTCTGTCAAGGTTCAAGGAGGAGGATTGGAGGGGGAGGATGCCTTAAAAGGCGAGGAGCGGGAGGGTGAGTGGTAACGGAAGAAGAGAGGAGAGCGAAAGG
3A-0698810-T8-1_192	gaggCCGCCTCCTCTCTTCTCTTGTTCTTGCTTCGCCACCCGCAACTAAATGCCAAGCAAGAAACCAATTAATGCCTCCTAACTTTAACTTTATATCTCTCGGCTAAGCTAATACTAATT
3A-0698810-T8-1_256	CCAATTAATGCCTCCTAACTTTAACTTTATATCTCTCGGCTAAGCTAATACTAATTGGTTTTTCTTTTCTTTTCTTTTTCGGTTGTGTAACCTCGGTTTGGTTGGCTTTGGGGGGCTTCG
3A-0698810-T8-1_320	TTTTCTTTTCTTTTTCGGTTGTGTAACCTCGGTTTGGTTGGCTTTGGGGGGCTTCGCTTACTTTGAGACTCATGGCAAAAGGGTTGGAAAAGAAGGGCagagagagagagagagagag
3A-0698810-T8-1_448	agagagagagagagagagagagaTCGGTGATTTTATGAATGATTCTTTTTTGGGTTCCAGAGGCCACTCGTGTGGGTTTGGGGATCCAATCCCAACCACTACCAGGGGCAGGGGCCG
3A-0698810-T8-1_640	CATACCCAACCCACATTTCTTTCTTCACCACATTACTATGGGATGTGTATCTATATCCATCTCATGCATTTACTACTTCTGCCCCCGTTATTTATGTCAAATATACGCCAATTTTGAGCG
3A-0698810-T8-1_704	TGCATTTACTACTTCTGCCCCCGTTATTTATGTCAAATATACGCCAATTTTGAGCGCAAGCAGGCTTACACATCCTGTGTTTATGTTTGGGGATATGGCTGGCAGTTGCTATCCCAAGGC
3A-0698810-T8-1_768	CTTACACATCCTGTGTTTATGTTTGGGGATATGGCTGGCAGTTGCTATCCCAAGGCTGCAATTTGGTTACTAGTAATTACTAGTAGATGGAGATTTGAATATATTTTCAGCGCTGCAGA
3A-0698810-T8-1_832	TGGTTACTAGTAATTACTAGTAGATGGAGATTTGAATATATTTTCAGCGCTGCAGAGTCAAATATTTGGAACACCTAAGCAAAGCAAGCAAGCTACCAAGTTTTTGTAACTCACTCATCT
3A-0698810-T8-1_896	TTTGGAACACCTAAGCAAAGCAAGCAAGCTACCAAGTTTTTGTAACTCACTCATCTACACCTTCCCCCACAAAAGAAAATATCTACACATTTACGTAAGACCAGGGGATTACCTTCCATT
3A-0698810-T8-1_960	CCCCACAAAAGAAAATATCTACACATTTACGTAAGACCAGGGGATTACCTTCCATTCAGATAAGTATAGTAACCGTGGTCAAAGCCTCGACCGAGGTTCAACAATCTATAATGTCCGCCC
3A-0698810-T8-1_1024	TATAGTAACCGTGGTCAAAGCCTCGACCGAGGTTCAACAATCTATAATGTCCGCCCCCACGAACCACGACGGGAAGGGTAGCAAAAAATGGTTCTGGATATTTTAGAAAAAAAGTAACAC
3A-0698810-T8-1_1088	CACGACGGGAAGGGTAGCAAAAAATGGTTCTGGATATTTTAGAAAAAAAGTAACACAATTTAGTAGGCGACGAATCAGATTTCAGATGGCGCAAGCGACAAGACCTGATTGGTCACATAT
3A-0698810-T8-1_1152	AGGCGACGAATCAGATTTCAGATGGCGCAAGCGACAAGACCTGATTGGTCACATATGCCACTAATAGGTTTAAGTAATCGCAGAGTAACAAACTAATGAAAACGGCAATATATCATGCTA
3A-0698810-T8-1_1216	TAGGTTTAAGTAATCGCAGAGTAACAAACTAATGAAAACGGCAATATATCATGCTAGGTTCAACAATGTCTGTAATGTATGTTGCATGGTGGGTTGGGTCGCAGAAATGGTTCAAACAAT
3A-0698810-T8-1_1280	AATGTCTGTAATGTATGTTGCATGGTGGGTTGGGTCGCAGAAATGGTTCAAACAATTTGGAAGTAAGACAATTTAATAGGCGACCGGCCAGATTTCATATGACGCAGGTGACAAATTTTT
3A-0698810-T8-1_1344	AAGACAATTTAATAGGCGACCGGCCAGATTTCATATGACGCAGGTGACAAATTTTTTGTCACAAATAACGGTACGAGGTTTATGTAGTCAACAATAAGCACTGCCGAGGACAACGGGAT
3A-0698810-T8-1_1408	AATAACGGTACGAGGTTTATGTAGTCAACAATAAGCACTGCCGAGGACAACGGGATAACTACTTCATTACTTGGTTGATAACGGTAACCTAACATGTCGTGCTAGATAAAAAGTCGGGCG
3A-0698810-T8-1_1472	CATTACTTGGTTGATAACGGTAACCTAACATGTCGTGCTAGATAAAAAGTCGGGCGGATAAGACCAATAGCAATGACTTTAGAAGTTTCCAAATAAGTGATTTCATTATTTTTATAGTTG
3A-0698810-T8-1_1536	CAATAGCAATGACTTTAGAAGTTTCCAAATAAGTGATTTCATTATTTTTATAGTTGTAGGCGATGACGCGTGACAAGACCTTGGTTGCAATACGAGGTTTTCGTAATCACACCATTAACA
3A-0698810-T8-1_1580	TTTTTATAGTTGTAGGCGATGACGCGTGACAAGACCTTGGTTGCAATACGAGGTTTTCGTAATCACACCATTAACACACTAGCGAAAATGACAAGCTAATGAAGTTAGTTTCGCTAACCG
3B-0714880-T8-1_0	TCTTGGtctctctctctctttctAAGGTTGAAGGAGTCTTAGGGAGGAGGGCTATTTGCCTTAAAAGGCGAGGAGCGGGAGGGTGAGTGGTAACGGAAGAAGAGGAGGAGAGCGAAAGG
3B-0714880-T8-1_192	aagaggaggCCGCCTCCTCTCTTCTCTTGTTCTTGCTTCGCCACCCGCAACAAAATGCCAAACAAGAAACCAATTAATGCCTCCTAACTTTAACTTTATATCTCTCGGCTAATTGGTTT
3B-0714880-T8-1_256	AAGAAACCAATTAATGCCTCCTAACTTTAACTTTATATCTCTCGGCTAATTGGTTTTGTGCAGTAGTATTGTTCGGTGCTTGGGTCGGATACTCGGTTTGGTTGGGTTTGGGGGTTCCC
3B-0714880-T8-1_384	AGACTCATGGCAAAAGGGTTGGAAAAGAAGGGCagagagagagagagagagagagagagagagaTGGTTTTATGATTCTTTTTTGGGTTCCAGAGGCCACTCGTGTGGGTTTGGGGA
3B-0714880-T8-1_576	ACGCCCTTACTCTCTACGCGCACCCCGTAGCATACCCACATTTCTGCATCGTATCTATATCCATCTCATGCATTTACTACTTCTGCTCCCGTTATTTATGTCAAATATACGCGAATACTG
3B-0714880-T8-1_640	CTCATGCATTTACTACTTCTGCTCCCGTTATTTATGTCAAATATACGCGAATACTGAGCGCAAGCAGGTTTTCCATATCCCGTGTTTATGTTTGGGGATATGGCTAACAATCGCGATCCC
3B-0714880-T8-1_704	CAGGTTTTCCATATCCCGTGTTTATGTTTGGGGATATGGCTAACAATCGCGATCCCAACCCTGCACTTTTGATTACTACGTACTACTCCTCTGTTCCTAAATACTTGTCTTTCTAGAGA
3B-0714880-T8-1_896	TGTTGAGTCCATTTGAAATGAAATGTCTAGAAAGACAAGTATGTGGAACGGAGAGAGTAGTTGCTAGTATATGAAGATTTGAATATCTTTTCAGTGCTGCAGAGTCAAATATTTGAAAC
3B-0714880-T8-1_960	CTAGTATATGAAGATTTGAATATCTTTTCAGTGCTGCAGAGTCAAATATTTGAAACagcaaagcaaagcaagcaagcaagcTACAATTTTTTTGTAACTGAGACATTAACACTTTACGT
3B-0714880-T8-1_1024	aaagcaagcaagcaagcTACAATTTTTTTGTAACTGAGACATTAACACTTTACGTAAGACCAGGCGATTAACTGGCATACAGATAACTATAGTAACCGTAGTCAAAGTGTCGAGCTAGG
3B-0714880-T8-1_1088	GCGATTAACTGGCATACAGATAACTATAGTAACCGTAGTCAAAGTGTCGAGCTAGGTTCAACAATGTATACTGTCTGCCTCCACGAACCACTGCGGGAAGGTAGGAAAAAATGGTTCTA
3B-0714880-T8-1_1152	TGTATACTGTCTGCCTCCACGAACCACTGCGGGAAGGTAGGAAAAAATGGTTCTAAATATTTTAGAAGAAAAGAAACACATTTTAGTAGGCGAGCAATCAAATTTCAGATGGCGCAGGC
3B-0714880-T8-1_1216	AGAAGAAAAGAAACACATTTTAGTAGGCGAGCAATCAAATTTCAGATGGCGCAGGCGACAAAACCTGATTGATCATATATGCCACTAATAGGTTTCAGTAATCGCGGAATAACGAACTAA
3B-0714880-T8-1_1280	CTGATTGATCATATATGCCACTAATAGGTTTCAGTAATCGCGGAATAACGAACTAACCAAGAACAACAAGCTATCGTGCCAGGTTCAACAATGTCTATAATACCCGTTGCATGGTGGGTTG
3B-0714880-T8-1_1344	ACAAGCTATCGTGCCAGGTTCAACAATGTCTATAATACCCGTTGCATGGTGGGTTGGGTCGTAGAAGTGGTTCATACAATTTGGAAGGATGAAAGACTATTTAGTAGGCGACCGACTAGA
3B-0714880-T8-1_1408	AAGTGGTTCATACAATTTGGAAGGATGAAAGACTATTTAGTAGGCGACCGACTAGATATCATATGACGCAAGCGACAAAACACAATTCATCATAAATAACGGTAGGAGGTTTACGTAGTC
3B-0714880-T8-1_1472	GACGCAAGCGACAAAACACAATTCATCATAAATAACGGTAGGAGGTTTACGTAGTCAACAATAAGCACTATCGAGGACAATAAGCACTAAGAGGTTTTTTTAGTGCTGGGCCTAGCAAAA
3D-0901830-T8-1_128	TTGATGCCGCTGCGGGGGAGTCCCTCTCTAATATaggaggtggaggcggggggaggaggCCGCCTCCTCTCTTCTCTTGTTCTTGCTTCGCCACCCGCAACAAAATGCCAAACAAGAA
3D-0901830-T8-1_192	CCTCCTCTCTTCTCTTGTTCTTGCTTCACCACCCGCAACAAAATGCCAAACAAGAAACCAATTAATGCCTCCTAACTTTAACTTTATATCTCTCGGCTAAGCTTACCACTAATTGGTTTT
3D-0901830-T8-1_256	ATGCCTCCTAACTTTAACTTTATATCTCTCGGCTAAGCTTACCACTAATTGGTTTTCCTTTTCTTTTCTTTTCTTTTACGGTGGTAACCTTGTTTTGTACAGCAGTTGTATTGTTGGGT
3D-0901830-T8-1_320	TTTTCTTTTACGGTGGTAACCTTGTTTTGTACAGCAGTTGTATTGTTGGGTGCTTGGGTCGGATACTCGGTTTGGTTGGGTTTGGGGGTTCCCTTACTTTGAGACTCATGGCAA
3D-0901830-T8-1_384	CGGATACTCGGTTTGGTTGGGTTTGGGGGCTTCCCTTACTTTGAGACTCATGGCAAAAGGGTTGGAAAAGAAGGGCagagagagagagagggagagagagaTGGTTTTATGATTCTTTTTT
3D-0901830-T8-1_576	TGTCCCTTGCCACCTTGACAAACCCACCGGAACGCCCCTTCCCCCTATGCGCACCCCGTAGCATACCCACATTTCTTCATCACATTACTATGCGTCGTATCTATATCCATCTCATGCATT
3D-0901830-T8-1_640	ACCCACATTTCTTCATCACATTACTATGCGTCGTATCTATATCCATCTCATGCATTTACTACTTCTGCTCCCCTTATTTATGTCAAATATACGCCAATATTGAGCGCAAGCAGGGTTACA
3D-0901830-T8-1_704	CTGCTCCCCTTATTTATGTCAAATATACGCCAATATTGAGCGCAAGCAGGGTTACACATCCTGTGTTTATGTTTGGGGATATGGCTGACAATTGCTATATATCCCAAGGCTGCACTTTTG
3D-0901830-T8-1_768	GTTTATGTTTGGGGATATGGCTGACAATTGCTATATATCCCAAGGCTGCACTTTTGCTTACTACTATTAGTAGTTATTAGTAGATGAATATTTCAATATCTTTTCAGTGCTGCCGAGTCA
3D-0901830-T8-1_832	TATTAGTAGTTATTAGTAGATGAATATTTCAATATCTTTTCAGTGCTGCCGAGTCAAATATTTGAAACACCTAAGCAAAGCAAGCAAGCTACCAAGTTTTTGTAACTCAGTCATCTACAC
3D-0901830-T8-1_896	AAACACCTAAGCAAAGCAAGCAAGCTACCAAGTTTTTGTAACTCAGTCATCTACACCTTCCCCCACACAAAAAAAATCTACACATTTATGTAAGACCAGGGGATTGCTTGCCATTCAGAT
3D-0901830-T8-1_960	ACACAAAAAAAATCTACACATTTATGTAAGACCAGGGGATTGCTTGCCATTCAGATAAGTATAGTAACCGTGGTCAAAGTATCGAGCGAGGTTCAACAATCTATAATGTCCGCCCCCACG
3D-0901830-T8-1_1088	CGGGAAGGATAGCAAAAAATGGTTCTGGATATTTTAGAAAAAAAGAAACACAATTTAGTAGGCGAGGAATCGGATTTCAGATGGCGCAAGCGACAAAACCTGATTGGTCACATATGCCAC
3D-0901830-T8-1_1152	AGGAATCGGATTTCAGATGGCGCAAGCGACAAAACCTGATTGGTCACATATGCCACTAATAGGTTTAAATAATCGCAGAGTAACGAACTAACGAGAACGACAATATATCATGCTAGGTTC
3D-0901830-T8-1_1216	TTAAATAATCGCAGAGTAACGAACTAACGAGAACGACAATATATCATGCTAGGTTCAACAATGTATGTAATGCCCGTTGCATGGTGGGTTGGGTCGTAGAAATGGTTCAAACAATTTGGA
3D-0901830-T8-1_1280	ATGTAATGCCCGTTGCATGGTGGGTTGGGTCGTAGAAATGGTTCAAACAATTTGGAAGTAAGACAATTTAGTAGGGGACCGACCAGATTTCATATCGTCATAAATAACGGTAGGAGGTTT
3D-0901830-T8-1_1344	AATTTAGTAGGGGACCGACCAGATTTCATATCGTCATAAATAACGGTAGGAGGTTTATGTAGTCAACAATAAGCACTGTCGAGGACAACAAGATAACTACTTCATTACTCAGTTGATAAC
3D-0901830-T8-1_1408	AACAATAAGCACTGTCGAGGACAACAAGATAACTACTTCATTACTCAGTTGATAACGGTAACCTGACATGTCGTGCTAGATAAAAATTCGAGGATAAGACCAATAGCAATGACTTTAGAA
3D-0901830-T8-1_1472	GACATGTCGTGCTAGATAAAAATTCGAGGATAAGACCAATAGCAATGACTTTAGAAGTTTCCAAATATTTGATTTCATTATTTTTGTAGTTGTAGGCGATGACGCACGACAAAACCTTGT
3D-0901830-T8-1_1536	ATATTTGATTTCATTATTTTTGTAGTTGTAGGCGATGACGCACGACAAAACCTTGTTTGCAATATGAGGTTTTCGTAATCACACCATTAACATACCAACAAAAATGACAAGCAACTGAAG
3D-0901830-T8-1_1580	ACAAAACCTTGTTTGCAATATGAGGTTTTCGTAATCACACCATTAACATACCAACAAAAATGACAAGCAACTGAAGTTAGTATCGCTAACCGGGCACATAGAGCAAGACACAAATTTTCA
2A-0342330-T8-2_64	CTCCCGGTCGCCCACCGAACGAGACCAGAGGGCAGCGCCGTGCAGTGTCACTCACTCTCACTCACCCTCCCTCCTCTGCTGCTGCCGGTGCGGTGCCTTGTGGGCATCTTGCGCCTCGGG
2A-0342330-T8-2_256	GGGCTCCAACGCAACGCAAGTGAGATGAGACGGTGTGctccctccctccctcccctcccctccctccTCTGTGTCCTTGGAGAGAGCCGAGTTAGTTGCGGCGGCACCTGATGCGGTT
2A-0342330-T8-2_384	TGTTGACTGACTGCACCGAGGTAGCGAGCGAGGAGCTCCCGGTGTTCTTTCTCCTCCCTCTTCACGGTTACTACTACTGAAAAAGGTGGTAAAGTTTCAGGTGTGATTGGGTCATGGCGCTC
2A-0342330-T8-2_448	GTTACTACTACTGAAAAAGGTGGTAAAGTTTCAGGTGTGATTGGGTCATGGCGCTCATCGGCATTATGATTTCATGGCTGTATGCATCGCTTGATGGGTACTCCTCCTTTTCGAAAAAAA
2A-0342330-T8-2_512	TATGATTTCATGGCTGTATGCATCGCTTGATGGGTACTCCTCCTTTTCGAAAAAAAACGGTATTATGATTTCGAAAATGGGTGGTACACGTACAGCAGACGATGGTACAGCGAGATCT
2A-0342330-T8-2_576	TTATGATTTCGAAAATGGGTGGTACACGTACAGCAGACGATGGTACAGCGAGATCTCTGAAGCACATCCTATGATTTCTTCTTTCTTCCGGTACAAGATGCTGCGGCGCAAGATCGAGCAGT
2A-0342330-T8-2_768	TAGTGCTACGTACGCTGGATCGATGGATGGATGGACCAGCGTGTGTAGATCAGTGCAGGATCATGGGAATTCTCAAAAGGCAGAGAGGTACATGCAGATATATCTCGCGTACTACACCAA
2A-0342330-T8-2_832	GGGAATTCTCAAAAGGCAGAGAGGTACATGCAGATATATCTCGCGTACTACACCAATGATCCGGCAAGGTCTCTGAAACTTTATATCTTTCCCGATTTCTTTCTTTCTGGCACAGTAGCT
2A-0342330-T8-2_896	CAAGGTCTCTGAAACTTTATATCTTTCCCGATTTCTTTCTTTCTGGCACAGTAGCTGCATTGCATTGCATCTCAATCTCAACGAGTACACCGGACGCTCTGAGCTGATGCTTGCGTTGGT
2A-0342330-T8-2_960	TTGCATCTCAATCTCAACGAGTACACCGGACGCTCTGAGCTGATGCTTGCGTTGGTTGGAGTTCCTCTGCCCCTCAGTTTGCTCCTAGTTATTGTGTTGCGTCAGTGCAGCGAATGTATT
2A-0342330-T8-2_1024	CTCTGCCCCTCAGTTTGCTCCTAGTTATTGTGTTGCGTCAGTGCAGCGAATGTATTGCTGCGTCGATCTGTTCTGTGGTCCGAAGAATCCATCAAGACCGTTCTGTTAGGTTGATCTCTC
2A-0342330-T8-2_1472	CATAGATAGTTCAATCTACTCCGAAACCCTAGCCTAGTCGATCCCATGGTTTTAACAATGGTATCAGATGCTGTCAAGGGCTTGATTTTGGGTAGAAACAGAGAACAGGGACCCTCCCCT
2A-0342330-T8-2_1536	CAGATGCTGTCAAGGGCTTGATTTTGGGTAGAAACAGAGAACAGGGACCCTCCCCTTACCAGATCGGATCGAATCCGAACCCTAAACAGGGAAAAAAAGTTCTTGAATCACTTCACAG
2B-0488170-T8-2_64	CGGTCGCCCGCCCACCCAACGAGAGGAGagggcagcgccgtgcagtgcagtgcagtgcagtgTCACTCACTCTCACTCACCCTCCCTCtggtggtggtggcggtgcggtgccggtggcgg
2B-0488170-T8-2_320	TGTGCTCCCTCCCTCCCTCCTCGTGTCCGTGGTGGTGAGAGAGCCAGGGGAGTTAGTTGCGGCGGCACCCGACCTGATGCGGTTGGTTGCGCTGTTGACTGTCTTCCCCTCTTCACGGTT
2B-0488170-T8-2_384	GCACCCGACCTGATGCGGTTGGTTGCGCTGTTGACTGTCTTCCCCTCTTCACGGTTACTAATGGAAAAGGCGGAAAAGTTTCAGGTGTGATTGGGTCATGGCGCTCATCGGAATTATGAT
2B-0488170-T8-2_448	AAAAGGCGGAAAAGTTTCAGGTGTGATTGGGTCATGGCGCTCATCGGAATTATGATTTCATGGATGGGTGGTACAGGTACAGCAACGATGCTAGTACAGCGAGATCTCTGAAGCACATC
2B-0488170-T8-2_512	TGGGTGGTACAGGTACAGCAACGATGCTAGTACAGCGAGATCTCTGAAGCACATCATATGATTTCTTGCCGGTGCAAGATGCTGCGGCGATAGTGTTCGGCGCAAGATCGAGCAGTGCG
2B-0488170-T8-2_576	TCTTGCCGGTGCAAGATGCTGCGGCGATAGTGTTCGGCGCAAGATCGAGCAGTGCGTTATTTGATCGGAGGGGGCTCTCCAGTCTCTCTCTGCAACTTTTGGGTAATCTGGTAACCGAAA
2B-0488170-T8-2_768	AATTCTCAAAAGGCAGATAGGTACATGCAGATATATCTCGTGTACTACACGAATGATACGGTAAGATCTCTGGTCTCGATTTCTTTCTCCCACAGTAGCTGCCTTGCATTGCATCTCAAT
2B-0488170-T8-2_832	GATCTCTGGTCTCGATTTCTTTCTCCCACAGTAGCTGCCTTGCATTGCATCTCAATCTCAACGAGTACACCGGACACTCTGAGCTGATGCTTATCGTTAGAGGATGATGAGAAAAAAATG
2B-0488170-T8-2_1088	GGATTAGAGATTAATCAGAAATCGGCCATGATTTTTTTTTCCGATAAACTGGCAGGAGCGCTGCCTTTCAATTAGATGAAGAGTGAGTGTTACAAATAGTTTACACAAGGCTTTTTCCTA
2B-0488170-T8-2_1152	CTTTCAATTAGATGAAGAGTGAGTGTTACAAATAGTTTACACAAGGCTTTTTCCTATAAGATTCGGTTGGCGGGGAGCCTCCCGAACATCATCGGCATGCGGATTACTCGCGAATGAGA
2B-0488170-T8-2_1280	GGATCTGTGCAAGCGAAAAATATCTGTCAAAGCTAGTTGGGTGACTTCTTGGTAGGTCAACCTCTTGCCGATGAAAACTCGACGGTTCCTCTCCTTCCAGATGTTTCAGAGCGTGTAGAT
2D-0589930-T8-2_64	CGGTCGCCCACCCACCCAACGAGAGGAGagggcagcgccgtgcagtgcagtgcagtgtcactcactctcactcaccctccctccctccctctggtggtggtgctggtgccggtggc
2D-0589930-T8-2_256	GGAATCTCGCGGGGAAAGCAACCGCCAAGGAAGGGCTCCAACGCAAACGCAAGTGAGATGAGACTCCCTCCCTCCCTCCTCGTGTCCGGGGTGGTGAGAGAGCCGAGTTAGTTGCGGC

Trait&gene no.	gene	Homoeologues	IWGSC gene ID (refseq1.1)	WGIN Promotome ID	Orientation	ATG	Target Sequence	MYbaits	MYbaits coverage (%)	MYbaits ID	MYbaits sequence
T9-1	Asn/Gln_amidotransferase	ABD	TraesCS2A02G262700	2A-0323960-T9-1	forward	414,989,701	CTTGTTGTATGGACTCCCCACCTGAATATATTTCAAAA	15	72.9	2A-0323960-T9-1_64	ATTGCGTGAAGAGAGGAAATCACGCCACAGACCGG
T9-1	Asn/Gln_amidotransferase		TraesCS2B02G279400	2B-0399390-T9-1	reverse	385,568,255	TGTGGCCAATGTGCCTCTCGTCTACTGCAATTCACAAT	12	64.9	2A-0323960-T9-1_128	TGGTGAGCCCATACACCAAGATTATCAAGGACAGCTT
T9-1	Asn/Gln_amidotransferase		TraesCS2D02G261300	2D-0551270-T9-1	reverse	317,947,763	TGTGGCCAATGTGCCTCTTGTCTACAGCAATTCACAAT	20	84	2A-0323960-T9-1_192	GATTTTGGAAAACTTGTTCCTGGCATCCCATATTTCC
T9-10	PIN2	ABD	TraesCS7A02G492400	7A-1788290-T9-10	reverse	681,014,965	CGCGCCCGACCCGACGAACTAGCTAGATCCCAACCTA	20	94.4	2A-0323960-T9-1_256	TGAACTGACGATGGCAACATAGCATGGATCGACGTGC
T9-10	PIN2		TraesCS7B02G398100	7B-1897450-T9-10	forward	664,106,998	TGCTACGTTATGTGCCGAGTCCATGCCATGTCTACTCTG	18	89.6	2A-0323960-T9-1_448	TTTTCGAAACAGAGAAAAGGTACAATATTGTTCCCTA
T9-10	PIN2		TraesCS7D02G478800	7D-1969450-T9-10	forward	589,512,708	AAAGTCCATGATTTTGTTTTTTTTGTGCAACTCGCAAT	17	79.3	2A-0323960-T9-1_512	TGCTGTGCAGAAAAAAGTTTTAAATGTGAACATCAAT
T9-11	BONZAI-3	ABD	TraesCS1A02G236100	1A-0017770-T9-11-1	reverse	418,057,131	CCCTGATCTCGGCCCTGGAGCCGCCGGGGAAGTTTGA	15	82.8	2A-0323960-T9-1_576	GTTTGAGCTCCGGACGTCTCCAGTTTGCCATGTATACT
T9-11	BONZAI-3		TraesCS1B02G247700	1B-0111050-T9-11-1	forward	438,176,247	TAAACATCAATATGTGTGCAACTACACTATATTATAAGC	12	58.4	2A-0323960-T9-1_640	AGTCTTTGCCAGTTTTTTATTATTTTGAGCTAAAAAAA
T9-11	BONZAI-3		TraesCS1D02G236200	1D-0205980-T9-11-1	forward	325,666,658	TTTCCGGCTTATAGGGCTCAATTCAAAAATCTCACCAA	19	73.6	2A-0323960-T9-1_768	GGCTGGGCTGGGCCTACAGGGAGAGAGAAATGAAT
T9-12	AUX1	ABD	TraesCS1A02G278400	1A-0030760-T9-12	reverse	474,229,570	TTATAACGACCGCTCTAGCATACGACTAATTTCTAGCA	21	87.8	2A-0323960-T9-1_960	CTGTCTACCGGCGGGCCTCCCCGCTGTCGCAGCTGCC
T9-12	AUX1		TraesCS1B02G287300	1B-0120460-T9-12	reverse	499,471,768	CCTCCTTCCCTTGCTTCTCTCTACGCTACGCACGCACGC	18	79.8	2A-0323960-T9-1_1024	cttccacttccccttcccTAGCAATGGACTCACTCGTATGCC
T9-12	AUX1		TraesCS1D02G277600	1D-0188170-T9-12	reverse	374,474,336	CCTCCCTTTTTTTTCTCTCTACGCACGCGACAGGAAGG	17	72.7	2A-0323960-T9-1_1280	CTTCTATTTAGGAACGGAGGGAGTACTACTCAGCAAA
T9-13	QWRF (IPR007573	ABD	TraesCS5A02G222400	5A-1228910-T9-13	forward	438,468,041	ATGCTTGATTAGTACCCGCTGCAATTTTTTACTGCCCTT	14	75.8	2A-0323960-T9-1_1344	GGTTGAGTATGTCTGACTTCATGGTGGTTTTGTGTTTG
T9-13	QWRF (IPR007573		TraesCS5B02G221400	5B-1339030-T9-13	forward	395,917,417	CCGTCTGGTGCCCGCTCGACCCGGAGCCTCCCCGAGG	14	69.2	2A-0323960-T9-1_1408	AGTTACCAGCAATCAAGTCTGGTACTAGAAAACAGTA
T9-13	QWRF (IPR007573		TraesCS5D02G230200	5D-1426330-T9-13	forward	337,297,315	CAGGAGGCTGCTCGACCCGGAGCTGCCTCGAGGAGC	15	76.1	2A-0323960-T9-1_1472	AGCAAGCATGGCTTGATTCATCTTTGGTGAGAATCGC
T9-14	LAX3	AB	TraesCS4B02G111100	4B-1097390-T9-14	forward	124,741,518	AGATGCATAAGTTGAAGCCAGAGATGACAACTTCAAA	12	58.4	2A-0323960-T9-1_1536	ATGTGTAGCCACCTAAACTCAGTGAAGACAATGCCTG
T9-14	LAX3	no D	TraesCS4A02G207600	4A-0993610-T9-14	forward	500901359	GTCAACACGGGTATCTCCAAAATTGTCATATCTTTTGC	15	50	2B-0399390-T9-1_64	CAATAGGAAGAGAACAAAAAACTTGAATACAGACAG
T9-15	MIP1	ABD	TraesCS7A02G415300	7A-1781320-T9-15	reverse	606,564,465	GGCTTCCCGCTCCGCCGGGGACTCCGCTGTTATTGGC	15	69.6	2B-0399390-T9-1_192	CCTCTCACGGCAATTCTCACCAAAGATGAATCAAGCC
T9-15	MIP1		TraesCS7B02G315200	7B-1869170-T9-15	reverse	562,747,849	GGCTTCCCGCTCCGCCGGGGACTCCGTTGTTCTTGTCC	10	60.7	2B-0399390-T9-1_256	AATCAATCCTACTGTTTCCCAGTACCAGACTTGATTGC
T9-15	MIP1		TraesCS7D02G408500	7D-2008610-T9-15	reverse	526,243,845	GGCTTCCCGCTCCGCCGGTGACTCCGTTCTTCTTGGCC	9	48.8	2B-0399390-T9-1_320	CCTTTTACCAAAGACAAAACCACCATGAAGTCAGAAA
T9-16	RSL4	ABD	TraesCS5A02G401300	5A-1193610-T9-16	forward	594,132,650	TGGGCATTAGCGGGCGCGAGACACTTGACTAATGTAA	15	69.6	2B-0399390-T9-1_832	ACCGAGGTAGTAGAAAACTACGCCAGTAACCGATGA
T9-16	RSL4		TraesCS5B02G406100	5B-1365700-T9-16	forward	582,151,083	ATCTAGACCCACGTCTGAAACCGCTCCTACTAAAGGA	11	51.3	2B-0399390-T9-1_1024	AAGGGAGAAAGGAGAGGCAGTGGAGAGGGCTTGT
T9-16	RSL4		TraesCS5D02G411600	5D-1454260-T9-16	forward	475,000,589	CGTCCATAGCACGACGCCAACACTCCTCCGTGTTTGCA	5	28.7	2B-0399390-T9-1_1088	TCAATCACCACCAGGGGGCGAGCTGGGCCCCGAGAA
T9-17	N1QSF8	AD	TraesCS5A02G106400	5A-1271440-T9-17A	forward	175,832,605	TGAAACGGACTCTCCTATCCAAACTAAACAAGCAACT	8	40	2B-0399390-T9-1_1216	AGAAGCTGAGGCCATTAATCCCTGTCTGCATTGTCTC
T9-17	N1QSF8	no B	TraesCS5D02G120500	5D-1490950-T9-17	reverse	170,381,063	GACAGTTGGCCAGGTTTGATGGCCTCAAAAGGCAGA	13	60.9	2B-0399390-T9-1_1280	CTAGGCTCTAACTGGCAAAGACTTTTTTTTAGCTCAA
T9-18	RHD6	ABD	TraesCS6A02G276100	6A-1517200-T9-18	forward	502,929,063	TTGATATATTTCTCTTCATTTTTAATCAAACTTAAAAGG	7	32.9	2B-0399390-T9-1_1344	AATATACTTGACACGACAGTGCAAGTATACATGGCAA
T9-18	RHD6		TraesCS6B02G303600	6B-1587290-T9-18	forward	544,092,118	GGAAAATCTGATGCAATTAATTTGCTTACATATGCCAC	17	89.9	2B-0399390-T9-1_1408	TGAACTCTGCCATATCAACTTATTCACATTGATGTTCAC
T9-18	RHD6		TraesCS6D02G256400	6D-1692430-T9-18	forward	362,859,527	TTTGGTAACCGGGTGAGATGATTAAATACAACTTCGA	12	61.6	2B-0399390-T9-1_1580	TCTTCACCTGCCCAGCGGCTCCGGGATTCGCCCTCGG
T9-19	Root phototropism protein 3	ABD	TraesCS4A02G048200	4A-1016270-T9-19	forward	38,838,107	ATCTCCCCGCTTTCGCTTTCCCTGTTGCATATCGCTCCG	14	78.6	2D-0551270-T9-1_64	CAAGAGGAAGAGAACAAAAAACTTGAATACAGCCAG
T9-19	Root phototropism protein 3		TraesCS4B02G256600	4B-1034980-T9-19	reverse	522,336,529	CTTGTAAGAAACAGAGGTTCAAAGCACGAACAAGCC	13	72	2D-0551270-T9-1_128	ACAGTTTAGCTGGCATTGTTGTCACTACGTTTAGGTG
T9-19	Root phototropism protein 3		TraesCS4D02G256700	4D-1135060-T9-19	reverse	426,522,114	CTTGACAGAAACAGAGGTTCAAAGGACGAACAAGTA	11	64.5	2D-0551270-T9-1_256	AAAAATCAATCCTACTGTTTTCTAGTACCAGACTTGAT
T9-2	ARF7	AD	TraesCS7A02G160800	7A-1839790-T9-2	forward	116,872,033	CTGTGAACACCCAGCGTTCGCCCGATTAGTGCTACTAG	6	32.3	2D-0551270-T9-1_320	GAACCTTTTACCAAAGACAAAACCACCATGAAGTCAA
T9-2	ARF7	no B	TraesCS7D02G161900	7D-2038600-T9-2	forward	112,046,247	AAATTTTGCCATGGCAGGTGGGCCGGGGCCAGCTCG	6	42.4	2D-0551270-T9-1_384	ATCGTCGCAATATTTGCTGAGTAGTACTCACGAGATGA
T9-20	SHR	ABD	TraesCS2A02G192600	2A-0000960-T9-20	forward	160,686,645	GGTTAGCATCACACCGTGGGCCTGTTGGGCCCCTTTG	9	43.3	2D-0551270-T9-1_448	AACTACGCCAGTAACCAATGAGCTATTACAAAGTTGA
T9-20	SHR		TraesCS2B02G214600	2B-0469480-T9-20	forward	199,530,429	TTTGAGATGGGTTTAGATAAGCTAAAATTATAGAATAT	18	84.2	2D-0551270-T9-1_512	ggaggggaggggagggggaaggagtgggctgaggggaggggccgg
T9-20	SHR		TraesCS2D02G194800	2D-0567160-T9-20	reverse	140,551,033	GTAAGCTACGGCGAGGGGGCAACATGAGGGTTACCG	7	42.8	2D-0551270-T9-1_704	CAGACTAATCATCAATCGCCAGGGGGCGAGCTGGAC
T9-21	Auxin resp factor 6	ABD	TraesCS6A02G138600	6A-1555510-T9-21	forward	109,983,416	TACTTGGCCATATCGCCATGAAAAAGTTTCAGCTTTTT	12	55.1	2D-0551270-T9-1_768	CTTTGATGACAATGGCCCGCCCAATTCTCTCTCCCTGC
T9-21	Auxin resp factor 6		TraesCS6B02G167100	6B-1664750-T9-21	forward	175,338,221	CGAGTAGAATACTTGCCATATCGCTGCGGAAAAAGTT	11	51.3	2D-0551270-T9-1_832	GATGCTGAGGCCATTAATCCCTGCCCTCGCTCTAGGC
T9-21	Auxin resp factor 6		TraesCS6D02G127600	6D-1725840-T9-21	forward	91,517,263	TTTATATTTAGGCTTGTAAAACTATGCTTTATATTTGGT	11	54.6	2D-0551270-T9-1_896	TCAAAAAAAAAAAGAACTGGCAAAGACTTGAAATGT
T9-22	SCR	ABD	TraesCS4A02G191300	4A-0942540-T9-22	forward	470,688,350	GACCTCATGGAGGGAAAAGTGAAAAGGATGTTAAAA	15	68.9	2D-0551270-T9-1_960	GGCAAAGGAACTGGAGACGTCGGGAGCTCAAACTG
T9-22	SCR		TraesCS4B02G124000	4B-1096070-T9-22	forward	148920366	TTGAACCAAAGATCTTCGTGAAAGAGGTACCAATGAA	19	81.4	2D-0551270-T9-1_1024	ATGTTGTTCACATTTAAAATCTTTTTTCTGCACAGTATT
T9-22	SCR		TraesCS4D02G122000	4D-1166110-T9-22	forward	105135086	GGCCGGAAGGCCCTGGCAAGGCCGCAAGGCTGCATG	9	40.5	2D-0551270-T9-1_1088	GCTAGAGAAAAATAGTGTGCCTTTTAAAAATAAAATA
T9-23	Rht1	ABD	TraesCS4A02G271000	4A-0978250-T7-33	reverse	582,479,578	GATCTCGCCTCACCGCGAGCGAGCTGCCTCGCTTCTCT	19	76.9	2D-0551270-T9-1_1152	GGGGTTAATATAAAAGGCTCACCAACTTTGGTGGAAA
T9-23	Rht1		TraesCS4B02G043100	4B-1095100-T7-33	forward	30861382	CTCCGCGATAAGGTTTGCTAGGCCTCGTGAACCTACCA	16	70.1	2D-0551270-T9-1_1216	ATATAGTCCATTATCCTCTTCTTATACTATTTGAAAGCA
T9-23	Rht1		TraesCS4D02G040400	4D-1165870-T7-33	forward	18,781,062	TTTTAATGACATGTGTAGCATAAGTACATGATCTAAAA	14	59	2D-0551270-T9-1_1280	CTTCAGGGAGGGACACCCCACGCAGACCAATAAATG
T9-24	LBD29	ABD	TraesCS4A02G312500	4A-0971050-T9-24	reverse	603,635,540	TGCTCCTGCTACCTGATCGATGCTGGTAGTGTACTCTTG	23	93.2	2D-0551270-T9-1_1344	TATCTAAATAAAGGCTTGATACTGCAAAACCTGTGCTA
T9-24	LBD29		TraesCS4B02G001200	4B-1102010-T9-24	reverse	806039	CGATCCTCCCCATTTATAAAGGCTACTCTAGCACTAGCA	11	61.2	2D-0551270-T9-1_1408	CAGACCATGGCTTGAGCTCCTGAGGAAATAACAGGA
T9-24	LBD29		TraesCS4D02G001700	4D-1176380-T9-24	forward	1205829	CAACTAAGAGCTGCATCAGTAATCAATGAAACATATCT	26	100	2D-0551270-T9-1_1472	CCGACTTAAAAGAGAATAGTTTGATCTTTCCACCAGT
T9-25	Expansin-B7	A, BD	TraesCS4B02G375600	4B-1070490-T9-25	forward	659,520,013	GACCGCCACCGACCTAAACTTGCACAGATACACACAC	11	61.2	2D-0551270-T9-1_1536	AATCTAGACTATCAGCAGCAAGGACATGTAAGATCAT
T9-25	Expansin-B7		TraesCS4D02G361700	4D-1114210-T9-25	reverse	508,432,960	GGCTAGCAAGTTGCTCCAGTACCACCAGATGATTGAA	5	25.4	2D-0551270-T9-1_1580	AGGTAAGCATGACGCCTTGGTCCACACTTAAGTACTA
T9-25	Expansin-B7		TraesCS5A02G537300	5A-1186910-T9-25	reverse	693,675,058	CGCTAGCAAGTTACTCCAGTAGGAGATGATTGAATTG	8	40	7A-1839790-T9-2_0	CTGTGAACACCCAGCGTTCGCCCGATTAGTGCTACTA
T9-26	LBD16	ABD	TraesCS6A02G398400	6A-1526230-T9-26	reverse	609,107,591	CGATTGTTCCTAGCGCCGACGGAGCTTCTCGGTCGAG	11	57.9	7A-1839790-T9-2_126	CCGCGTATGGCATATGGTGCGACCCGAGCCGGCCAG
T9-26	LBD16		TraesCS6B02G438700	6B-1604630-T9-26	forward	704,751,310	AAAGTGGTTCAAATCTGCCTCTCTTTGAATCCTGCTCC	4	18.4	7A-1839790-T9-2_252	CGCGGGCCTGTCCCCTCCGTTAACCTTCCCAACGCCT
T9-26	LBD16		TraesCS6D02G382600	6D-1700030-T9-26	reverse	462,273,070	CGATTGTTCCTAGCGCCGACGTAGCTTGTCGATCGAG	14	69.2	7A-1839790-T9-2_315	CCCGCTGTGTTACCGCATCTCTGACCGACGCCGTTAG
T9-27	TF_GRAS	ABD	TraesCS4A02G039000	4A-1019660-T9-27	forward	31,413,603	CGTTCGGCCCTTGGTTTCCCAAGTAGGACCATTTTTGT	17	82.6	7A-1839790-T9-2_685	acgccaccggcaccgcgtcgccgtcgtctctccccgcagtccgcaccg
T9-27	TF_GRAS		TraesCS4B02G266700	4B-1056990-T9-27	reverse	538,999,544	ATTTTCCAACCTTGTGGAGGTGATCTATTTGCCGGAAT	21	94.8	7A-1839790-T9-2_1580	GGAAAGGCGCCCGTGCGCCCTCCGAGCTACTGGGCC
T9-27	TF_GRAS		TraesCS4D02G266400	4D-1151360-T9-27	reverse	437,029,388	ATTTTCCAACCTTGTGGATGTGATCTATTTGCTGGAAT	17	76	7D-2038600-T9-2_195	GAGGGATCCCTTCCGCTCCAAACGTTGCACTGCCTCG
T9-28	LBD14	ABD	TraesCS4A02G415400	4A-0949990-T9-28	reverse	685381598	TGCTTATAAGGAGAGGAGCGGAGGAGGAGGTGCATG	8	40	7D-2038600-T9-2_390	CCGTTAACCTTCCCGATCAACGCCGCCGTGCCCGCGG
T9-28	LBD14		TraesCS4B02G316200	4B-1045830-T9-28	reverse	605,693,239	TGCTTATAAGGAGACGAAGAGGAAGGGGGAAAGAG	14	69.2	7D-2038600-T9-2_585	GTCGTGGGCGCCCCGCAGAGTAGCTTGCCGCGCCCG
T9-28	LBD14		TraesCS4D02G312800	4D-1141810-T9-28	reverse	478999212	CAACCGACCGGTGATGATCGCTGGATCGCTCGGTGAT	17	77.2	7D-2038600-T9-2_780	CACCGCGTCGCCGTCGTCTCCGCACCCCCGCCACGCG
T9-29	TF_GRAS	ABD	TraesCS5A02G144000	5A-1252880-T9-29	reverse	319,754,543	CTAGTAACGCCAAGGGGAAGCGGCCGGCGCGCGCAT	4	38	7D-2038600-T9-2_843	CGCAACTAGCCCAGGCGACCCGCGGGTGATTTTTCTT
T9-29	TF_GRAS		TraesCS5B02G143100	5B-1324590-T9-29	reverse	270,014,076	CTAGTAACGCCAAGGGGAAGCGGCCGGCGCGCGCAT	24	92.5	7D-2038600-T9-2_1190	CCACTGGCCTTCCCCCTCCCCCTCGCTTCCCATCTCGG
T9-29	TF_GRAS		TraesCS5D02G148000	5D-1428120-T9-29	forward	234,821,214	ATTATTATTATTTTATGATGGCCTTTTCATTTATTCATGC	20	85.2	7D-2038600-T9-2_1580	CGCGCGGGCGCCTTCCGAGCCAGTGGCAGCGCCTAG
T9-3	Fucosyltransferase	ABD	TraesCS6A02G320100	6A-1527380-T9-3	forward	555,314,174	GCTCTTGAACTGCTCCCAGAAATCGTCATTGTCCGGGT	10	47.5	6A-1527380-T9-3_384	CGCGGCCTTGTCGGAGGCGGGGTAGGTGCCGAGCC
T9-3	Fucosyltransferase		TraesCS6B02G350900	6B-1620700-T9-3	forward	616,323,627	TGATGATTGTTACTTTATGGGCGATTTGGAGTGCAAGA	9	47.1	6A-1527380-T9-3_576	AACACGGTGTGGGGAGCGGCGTGGGAAGCGTCGGT
T9-3	Fucosyltransferase		TraesCS6D02G299600	6D-1697180-T9-3	forward	409,866,925	GAGTTGTTCTAGTATGTCTTTATAACAGCCGTTTGGTT	21	87.8	6A-1527380-T9-3_768	acggattggggccggcggaggagcggtgggggagggggcgAATC
T9-30	GNOM/ Fewer Roots	ABD	TraesCS5A02G361300	5A-1212300-T9-30	forward	562,536,087	ATTGAAACTCCTCGAAACGGGAATTTGCGGCGGTGAA	20	85.2	6A-1527380-T9-3_960	GCGTGATGGGTGGGCGGGAGGAAGAAGGGAGAGA
T9-30	GNOM/ Fewer Roots		TraesCS5B02G363900	5B-1348970-T9-30	forward	542,928,236	AACAGGAATTTGCGGCAGCGGAAAAATTGGAGGAA	23	66.5	6A-1527380-T9-3_1024	GCGCGGCTTGCAAAGATACATCTCCTGTAGGTTGTAG
T9-30	GNOM/ Fewer Roots		TraesCS5D02G370200	5D-1443170-T9-30	forward	445,198,999	CGACAAGGTGCCAAAACAGGTCTCCAGATTCGATCTC	18	77.6	6A-1527380-T9-3_1088	TGTTGTATTTTATATCTTCAGGGACTGAGAGGTAATTT
T9-31	LOB domain (IPR004883)	ABD	TraesCS4A02G236200	4A-0988850-T9-31	forward	544953084	ACACGGCACAGGTGCCAAGGGAAAACTCGAGAAATG	21	88.9	6A-1527380-T9-3_1152	TTGGAGCTGAAGATGAAAAATTAGGTATTTCTACGCC
T9-31	LOB domain (IPR004883)		TraesCS4B02G078800	4B-1089110-T9-31	reverse	75,779,728	CTCTCACCTAACGGATCAACCAACGGCGGGGCACTCG	19	86.8	6A-1527380-T9-3_1216	CAGAGCAGTTCGCTGCTTTACTGTCCCGGGTGGTCTG
T9-31	LOB domain (IPR004883)		TraesCS4D02G077600	4D-1167910-T9-31	reverse	51616874	CTCGACGGATCAACCAACGGCAGGGCACTCGGTCGAT	10	47.5	6A-1527380-T9-3_1280	AGAGTTGAGATCCCATTTTCGGCCTGTGGTGGGGGG
T9-32	PLETHORA related	ABD	TraesCS6A02G229500	6A-1522720-T9-32	reverse	432,257,969	TATGGGACGGGCGATTGATAGAACAGCCCGCGGGGC	13	71.3	6A-1527380-T9-3_1344	AGCGCAGCGCGGTGCCGGTGCCGGTGCCAGCTACGG
T9-32	PLETHORA related		TraesCS6B02G252000	6B-1602470-T9-32	forward	452,171,125	CAACCCCCTTCCCCATTAAGTATCTGGGGTTGCCCCTC	3	14.6	6A-1527380-T9-3_1408	CAAAGCCGGGCAAGGGGACAGAACAGAAAAGGAA
T9-32	PLETHORA related		TraesCS6D02G205300	6D-1700490-T9-32	forward	291,231,800	AACCTAGGACAAAAGCTTTGCCTCATCGCATTAGTTGA	18	80.9	6B-1620700-T9-3_640	CATTATGATTCAGTAATAAAGCTTTGTGAGATTCTTAA
T9-33	IAA4-like	ABD	TraesCS1A02G082700	1A-0078610-T9-33	reverse	65,800,185	CAGACAAGAGTAGAGAGAGAGAGAGAGAAAGGAG	4	20.9	6B-1620700-T9-3_832	GTACCATGTTTGGCAACCTCGTGTAAACTATAGTTGCC
T9-33	IAA4-like		TraesCS1B02G100300	1B-0168230-T9-33	reverse	108,303,286	CAGACGAGAGTAGAGAGAGAGAGGGGAGAGGTGG	4	21.6	6B-1620700-T9-3_960	CAGGCGTCCGGACACCGGAGTTACGCTTCACAGAGC
T9-33	IAA4-like		TraesCS1D02G084100	1D-0254920-T9-33	reverse	67,001,104	CAGACGAGAGAGTGGGGGAGCGGTGGCAGAAGAGA	9	45.9	6B-1620700-T9-3_1024	GCCCCGGGTGGTGGTTTGTGCTTGTTCTTTCTTGATT
T9-34	MYB36	ABD	TraesCS1A02G379700	1A-0018880-T9-34	reverse	551,593,136	CTATCTACCTACTACACACGTCGCAGCACGGCGGCCGG	18	79.8	6B-1620700-T9-3_1088	TCGGCCTGTGGTGGGGGGCAATATCGTACTTTTTCCC
T9-34	MYB36		TraesCS1B02G265400	1B-0093380-T9-34	reverse	466,076,703	TGAAGTTGTGTGTGTGTGTGTGTGTGTGTGTAGTGCTG	17	80.5	6B-1620700-T9-3_1152	TACGGGGTTGTTACGGCGACGTAGTTTGTGATTCGTC
T9-34	MYB36		TraesCS1D02G387100	1D-0199480-T9-34	reverse	459,880,642	CTATCTATCTACTACACACGTCGCAGCACGGCGGCCGG	13	67.5	6B-1620700-T9-3_1216	GTGGGGGGCAATCCCTCGACTAATGGCATCTCTCCAA
T9-35	ZF-HD homeobox protein	AD	TraesCS5A02G142700	5A-2078230-T9-35	reverse	316,351,399	GGACGACGCCTTCTTGCCGGCCGGCGGCCGTCGTCGG	10	57.4	6B-1620700-T9-3_1280	GAAAGCTCGTACGTGTAGCATTGCTGTTTTCCCACTCC
T9-35	ZF-HD homeobox protein	no B	TraesCS5D02G149400	5D-1440510-T9-35	forward	238,383,001	CAAATTACCAATTACTACTCAGCTATCTAAAAAGATATA	11	61.2	6B-1620700-T9-3_1408	GCAAGGGACAGAAATAGAAAAGGAACGAGATATTCA
T9-36	UCC1	ABD	TraesCS7A02G307900	7A-1752710-T9-36	reverse	435,827,403	GGATGAATGCCAATCGAGGGACTAGTAGTTGAGAACA	6	29.2	6D-1697180-T9-3_128	ACTCCTTTATTTCAAAAAAATTAATATATGATAGAATAA
T9-36	UCC1		TraesCS7B02G208300	7B-1867430-T9-36	reverse	380,888,385	CTCTGCCGATCGCGGCTGGAACCTGGAAGAGGCAAG	8	46.6	6D-1697180-T9-3_192	AGCTGCAATTGTGGGTTCACAAAAAAAATTACAATTG
T9-36	UCC1		TraesCS7D02G304800	7D-2007220-T9-36	reverse	386,563,339	GGATGAATGCCGAGCGACTAGTAGCTCTAGCACTTGA	11	64.5	6D-1697180-T9-3_384	ACTTTTGTTTGCATCAATGATGAATGCCACTAGAGAGT
T9-37	LRR kinase (M8C3Z3)	ABD	TraesCS3A02G135400	3A-0699520-T9-37	forward	112952865	CCGCCGGCGCATAGCTCGCCGGAACCAGCCAGCGCAC	15	72.9	6D-1697180-T9-3_448	AAGGAGATGACGAAGAATAAAGTAAGATGAGGAATA
T9-37	LRR kinase (M8C3Z3)		TraesCS3B02G169200	3B-0810080-T9-37	forward	169622854	TCTGTAACATCTCTCTGCCTTTCATTCCCTAAGACACAT	10	50.8	6D-1697180-T9-3_512	CTAATCAAACATTCACAAAAATATCCGTGGTCCTCATC
T9-37	LRR kinase (M8C3Z3)		TraesCS3D02G151400	3D-0913830-T9-37	forward	116,036,657	TCTTCTTTTGAAATTGCACTAGATTATGGAGAAAAACA	8	67.8	6D-1697180-T9-3_576	GTCATAACAATCGGTGGGTAAGATAGAATCATGTACAT
T9-38	SGN1	ABD	TraesCS6A02G024300	6A-1555880-T9-38	reverse	12,119,995	CCTCCTCGCTACCCTTAGCTAGGTAGCTTGGATTGGCG	11	53.4	6D-1697180-T9-3_640	AATAAAGTATAGTAGTTTGCCTTAGTATAGTGTTATTTC
T9-38	SGN1		TraesCS6B02G033300	6B-1657400-T9-38	reverse	19,777,736	CCTCCTCGCTACGCTTAGGTAAGTAGCTTGGATTGACG	9	40.5	6D-1697180-T9-3_704	ATAACCCCCTTTTTTGTAAAAAAAAAGAGTTTAGTTAG
T9-38	SGN1		TraesCS6D02G027700	6D-1737520-T9-38	reverse	10,514,925	CCTCCTCGCTACCCTTAGCTAGGTAGCTTGGATTGCTG	12	55.1	6D-1697180-T9-3_768	TACTAAAATGCTTTGATCTGTTTATTGTTTTGATCCAAA
T9-39	LRR kinase (M8C3Z3)	AD	TraesCS3A02G167100	3A-0675860-T9-39	forward	172629785	GTGCATTTTGAAGGCTGGCTAGTGTGCCGGCCATTGG	11	61.2	6D-1697180-T9-3_832	ATAATATCTTGTGTGTTTAAATATTTGTCATAAAAGGAA
T9-39	LRR kinase (M8C3Z3)	no B	TraesCS3D02G173200	3D-0928760-T9-39	forward	153,848,263	ACCCATCCCCGGACGAAATTTGCGCCGATTTTGGGGT	9	50.4	6D-1697180-T9-3_896	TATTTGTAGCATATGTTATGACAACACGTATAGGAAAG
T9-4	TAA1	AD	TraesCS1A02G113400	1A-0066620-T9-4	forward	116,694,157	TAAAAGTCATAGAGCGATTATGCCACTCGTTTTGCATG	11	50.1	6D-1697180-T9-3_960	ACATGTTGATTCAAATGTTCAACTACACATTTGAAACA
T9-4	TAA1	no B	TraesCS1D02G114800	1D-0262410-T9-4	forward	110,732,052	TGAACAATTTTCGAAAGAAAAAAAAAGGAAAACAGA	7	38.4	6D-1697180-T9-3_1024	TAATATGTATATACGCTACGCTGAAGAAAACCGGGGG
T9-40	SGN3/GSO1	ABD	TraesCS2A02G230100	2A-0332190-T9-40	forward	262,573,402	ATTTAGGCATCATTCTATTCTCATGAATTTTAGAGCAAG	15	69.6	6D-1697180-T9-3_1088	ATTCTAGATTGTATCCTTCCCCACTGCATGAGGAGACA
T9-40	SGN3/GSO1		TraesCS2B02G251700	2B-0493430-T9-40-1	forward	265,215,873	TCCACGCCCGAACCTCCATATAATTCATGTGACATCTTA	10	54.1	6D-1697180-T9-3_1216	GAGGTGCATGATCTCTTGCAACTTGCAAGCAACTGCA
T9-40	SGN3/GSO1		TraesCS2D02G233800	2D-0580850-T9-40	forward	209,507,156	ATTCCGCCGGCCTTGGGAGGGGATCATAAATCAATCAT	18	80.9	6D-1697180-T9-3_1280	ACTTCCGTAATACTGATTCTTAAAAAAGTTTAAAAAAT
T9-41	ARR3	ABD	TraesCS2A02G309700	2A-0289660-T9-41	reverse	532,867,753	CTCCCTCTCCTCTCCTCTCCTCTCCTCTCCTCTCTACCAG	14	71.3	6D-1697180-T9-3_1344	TTCTTTTGTATTGTTCATTGAAAAGACAATCTTTCGGT
T9-41	ARR3		TraesCS2B02G326800	2B-0435700-T9-41	reverse	467,872,969	CTCCCTCTCCTCTCCTCTCTACCACACAAGCAACCACTC	15	75.1	6D-1697180-T9-3_1408	TACAACCCACTTTGTTATAATATTCTGTTACTGGCTTAC
T9-41	ARR3		TraesCS2D02G308000	2D-0539450-T9-41	reverse	394,800,555	CTCCCTCTCCTCTCCTCTCCTCTCTACCAGACAAACAAC	7	42.8	6D-1697180-T9-3_1472	CCTAATGTCAGTTTCCATGTCCCAATCCTTTGTAGGGA
T9-42	PER64	ABD	TraesCS2A02G467000	2A-0304560-T9-42	reverse	711,510,257	TAAGTGTGCTGGCTAGTCTGACCAGCTGCTGCTGCATG	14	65.9	6D-1697180-T9-3_1536	CGCTATGAGAATAATGATAACAATGTAATTCAACTTAG
T9-42	PER64		TraesCS2B02G489800	2B-0393600-T9-42	reverse	687,228,387	TAAGTGTGCTGGCTAGTCTGAGCAGCTGCTGCTGCATG	8	43.3	6D-1697180-T9-3_1580	CAAAAAATTTTTTGAACTACAATATGCAGCAGCAAAC
T9-42	PER64		TraesCS2D02G467000	2D-0518660-T9-42	reverse	572,263,703	TAAGTGTGCTGGCTTGTCTGACCAGCTGCTGCTGCTGG	12	56.9	1A-0066620-T9-4_512	GATTTGAACCCTAATAGGTTGGGGATACCACTGTCTAC
T9-43	ARR3	ABD	TraesCS2A02G264800	2A-0277490-T9-43	forward	420,225,711	TACGTGGCACAGAAAATCAGCCAGATTGTGTCAAGAT	19	81.4	1A-0066620-T9-4_576	TAAAATTGTCATCCTTACCGGCCACGCCGGCCCACGC
T9-43	ARR3		TraesCS2B02G277300	2B-0426180-T9-43	reverse	383,058,413	TGGGAGGGGGGAGAATCTCGAGAGTGTGCGAGGAT	25	96.2	1A-0066620-T9-4_640	ATTGCATGCACGTATGGCCCGCAACCGGCCACTAACT
T9-43	ARR3		TraesCS2D02G259200	2D-0507440-T9-43	reverse	315,116,398	CGATTTTACACCGGAGCTCTCGCTCTCGCTCTCCAAAC	18	74.4	1A-0066620-T9-4_1024	TTGGAAGAGCGGCGACGCTTGTGGCCAAGCGGAGC
T9-44	RBOHF	ABD	TraesCS3A02G280200	3A-0653240-T9-44	reverse	509,428,095	CGCACACGCGGCCTCACGAGGCAGTCCGGCCCAATGC	15	69.6	1A-0066620-T9-4_1088	ATCTGCAGGATGGGGGCGAGGGGAGAAAGGGGAG
T9-44	RBOHF		TraesCS3B02G314000	3B-0738650-T9-44	reverse	504,986,637	GATCAACGCGGCGTACTCCTCCGCTTGCTCCTTAAGCC	24	57.5	1A-0066620-T9-4_1152	GCAGGGCTTGTTGTGAGAGAAAAGCATGGTCGAACG
T9-44	RBOHF		TraesCS3D02G279900	3D-0884390-T9-44	reverse	388,337,155	GATGATCTGCCAAAGAAAGGGAACGCAACATCAGTA	27	65.6	1A-0066620-T9-4_1216	TGTTTTATCATTTTCTTATCCAATAAAGATTTTCCATATA
T9-45	CLAVATA3/ESR	B,B,D	TraesCS5B02G480900	5B-1784910-T9-45	forward	202,059,614	GAGATAGGGTCGAAGGAGGTTGGATGCGGTTGGTGT	21	87.8	1A-0066620-T9-4_1280	AAAAATGGCTTTGCTAAAAAAATCTCTCTAAAATGTGT
T9-45	CLAVATA3/ESR		TraesCS1B02G314700	1B-1904800-T9-45	reverse	439,259,440	CACCGCCCGGAGCGCCGCGACTGCCCCGGCAAAAAA	22	94.8	1A-0066620-T9-4_1344	TTTATCCAAACAATCACGTAAGCAATCTTCCTAGTTGG
T9-45	CLAVATA3/ESR		TraesCS7D02G330200	7D-1988810-T9-45	reverse	421,856,379	GAACAAACAGAGAACCTTACTTGAAAAATTAAGCCGC	20	90.6	1A-0066620-T9-4_1536	AGCGCCTCTCGCCTGCCATTATTTTTACCGGCCCCTCA
T9-46	SHY2/IAA3	ABD	TraesCS5A02G597900LC	5A-1193820-T9-46	reverse	622,558,528	GGACGGACGTTCGAGCCGGCGAACAAGGTCGCTGGC	15	66.4	1A-0066620-T9-4_1580	TATTTAGCCATCTAGAATCATCCCACATCTTCTTCCATAT
T9-46	SHY2/IAA3		TraesCS5B02G446100	5B-1289280-T9-46	reverse	617,712,368	GGATGGATGGATGAAGAAAACGAGAAGTGCGTGAGC	15	61.9	1D-0262410-T9-4_256	CATAGGATTTTCCCGAACTAACCACTAACCCCTAATTG
T9-46	SHY2/IAA3		TraesCS5D02G323500	5D-1425820-T9-46	reverse	415,622,615	CACTCTCAAATTACGCAATGTACTACTCCTACTATACGA	10	54.1	1D-0262410-T9-4_832	CAGGCAGGGCAGCGCTCCGCATGCTGGGCGCTGGGT

T9-47	RALF	AD	TraesCS6A02G315100	6A-1517770-T9-47	reverse	551,655,896	TGTCCCTGCTTCAAGTGGCGCCTGCACTGTGGCCTCTG	2	10.8	1D-0262410-T9-4_896	CGTTCATGGCTTGCTGTGAGAGAAAAGCATGGTCGA
T9-47	RALF	no B	TraesCS6D02G294400	6D-1686320-T9-47	reverse	405,120,500	CGTCCCTGCTTCGAGTGGCGCTTGCAGTGTGGCCTCT	2	10.8	1D-0262410-T9-4_960	ACGTCATTTATCATTTTCTTATCCAATGAAGATTTTCCA
T9-48	MP	ABD	TraesCS2A02G491000	2A-0284740-T9-48	forward	724,297,632	CCATGGCATCTGGGTTACGAAGCAGACGCCAGGTACC	17	76	1D-0262410-T9-4_1344	ATTGCCTAAGCGTTCAAATAAACGCTCTTGCATTGTAG
T9-48	MP		TraesCS2B02G519200	2B-0400900-T9-48	forward	714,128,098	CGTGCGCCGGACGGCGGCTAGACGTGGTGAGGTACG	18	86.4	1D-0262410-T9-4_1536	GAATGACCATCTGGCGCCTCGCGCCTGCCATTATTTT
T9-48	MP		TraesCS2D02G491200	2D-0537490-T9-48	forward	589,973,755	CACACACACACACACACACACACACACACACACAC	17	79.3	1D-0262410-T9-4_1580	CCCTCACCTTCCTATTTAGCCATCTAGAATCATCTCATA
T9-49	Aux/IAA	ABD	TraesCS5A02G382600	5A-1220530-T9-49	reverse	580,466,170	AATCTAGTTCCAAGTCCAACCCACTGGTGCTGTAAAA	32	76.4	2A-0319940-T9-5_0	GGGGATATTGGGTGCACGAATGTGGGTATCGATAAAT
T9-49	Aux/IAA		TraesCS5B02G386800	5B-1334340-T9-49	reverse	565,931,391	CGGCTCCGGTGGAGCAGCAGCTGGTCGGCTAGCTAG	17	72.7	2A-0319940-T9-5_64	GTATATGGATAACAAAGCTATATATTCAGGAAAGTAAT
T9-49	Aux/IAA		TraesCS5D02G392000	5D-1454670-T9-49	reverse	460,396,047	CGGCTCCGGTGGAGCAGTTGGTCGGCTAGCTAGCCTG	16	70.1	2A-0319940-T9-5_128	ACAAATACCCGTGCGTTGCTACGTGATGGAACAATAT
T9-5	SAUR6-like	ABD	TraesCS2A02G386400	2A-0319940-T9-5	forward	632,506,993	GGGGATATTGGGTGCACGAATGTGGGTATCGATAAAT	15	72.9	2A-0319940-T9-5_192	GTAGATTATGGACAGTTTAAAAAAGAATAGCTAAAAA
T9-5	SAUR6-like		TraesCS2B02G403800	2B-0441960-T9-5	forward	572,156,348	AAATAATTTGATAAAAGCGTATGTGGATAACAAACCTA	19	84.7	2A-0319940-T9-5_256	ACAGGTTGGAAAAAATCCCTACACCTAATGAGTTGCA
T9-5	SAUR6-like		TraesCS2D02G383200	2D-1482860-T9-5	forward	487,493,419	AGCGTATGTCGATAACAAACCTATATATTTAGGAAAGT	17	77.2	2A-0319940-T9-5_320	TGCACAAAAAAATCTCTATTTAAATGGATGTGATTAAT
T9-50	SnRK2	ABD	TraesCS5A02G069500	5A-1264760-T9-50	forward	78,157,672	TTTGTCTCAGTCTCTCTTTCCTCTTGTTCTTTTGCTTTTT	13	68.7	2A-0319940-T9-5_384	ATTGATTACATTAATGGCTTGATTTTCAAGTTTACTCGA
T9-50	SnRK2		TraesCS5B02G075800	5B-1388810-T9-50	forward	90,507,424	TCTATCTTTGCTTTCGCTAAAAAAAAACTTTTATCTTTG	16	73.4	2A-0319940-T9-5_640	AGGTCTTATATTTAGGAACGGAGGGAGTATTAATAGT
T9-50	SnRK2		TraesCS5D02G081700	5D-1473890-T9-50	forward	82,187,115	GGAGAAGTTGTCCGCCGATCTGCACAGCAACGGTGA	14	69.2	2A-0319940-T9-5_704	TTTTCAAATTATTTTCACATTAATAGCTGCGTGTGAATG
T9-51	Apyrase	ABD	TraesCS2A02G102100	2A-0353830-T9-51	reverse	55,177,095	CTTGTCGGCGGGCAGGAGGAGGAGGTCGGGCGAGG	13	54.4	2A-0319940-T9-5_960	ACAAATATTTAGGAACGGATGGAGCAGCTTGCAAAA
T9-51	Apyrase		TraesCS2B02G119200	2B-0454840-T9-51	reverse	85,325,041	CTTGTCGGCGGGCAGGAGGAGGAGGTCGGGCGAGG	11	53.4	2A-0319940-T9-5_1024	ATAATAATAATAACACTCCTTCCTTCCCATAATATAATGG
T9-51	Apyrase		TraesCS2D02G101500	2D-0593940-T9-51	reverse	54,677,408	CTTGTCGGCGGGCAGGAGAAGGAGGTCGGGCGAGG	14	61.4	2A-0319940-T9-5_1152	GCGGATCTTAATGTAAGATCTCTCGGATATAGCATCGA
T9-52	ACR4/CRINKLY4	ABD	TraesCS7A02G301300	7A-1775070-T9-52	forward	419,565,121	AGACGCCACGCTTGTTTGGGTAACAAAGCCAGATTGA	4	21.6	2A-0319940-T9-5_1280	CGCCCGTGCCCTCGGAAGCACACCATCCATTAGCGAA
T9-52	ACR4/CRINKLY4		TraesCS7B02G201600	7B-1908900-T9-52	forward	368,621,788	AGGTGCGTCGCCAGTGGGCGTTCCTAATGCCGGAGCA	4	21.6	2A-0319940-T9-5_1344	AGGTAAAATCACGTCAAAATAAGAAAAGGAAACATG
T9-52	ACR4/CRINKLY4		TraesCS7D02G296700	7D-1985450-T9-52	forward	373,583,447	AACGTTTGTCTCATTAAGTCTTCATCCCATAAATTGGTG	1	7.1	2A-0319940-T9-5_1408	ATCTTCTTCACAAGCCCAGCGCCGAGCCAAGCCAGG
T9-53	Cellulose synth-like D1	ABD	TraesCS1A02G196100	1A-0034150-T9-53	forward	354,295,106	CGGCAAGTACAAGGTGAGGAAGGGCGAGGTCGCCAT	9	42.6	2A-0319940-T9-5_1472	ACGAGCTGCATGAAATTTTAGTAGGATCTCAAAAAAG
T9-53	Cellulose synth-like D1		TraesCS1B02G210700	1B-0094860-T9-53	forward	383,792,487	TGCGGGTGTTATCTTGTATTTCTGTTGGCACACCTAGT	15	78.4	2B-0441960-T9-5_0	AAATAATTTGATAAAAGCGTATGTGGATAACAAACCTA
T9-53	Cellulose synth-like D1		TraesCS1D02G199500	1D-0223780-T9-53	forward	282,281,612	CTGCAGACGTCGCATATGAGGCTGCATTTCAAGAACC	14	71.3	2B-0441960-T9-5_64	ATTGTGACTTTTTTTCTGGATAAATAAGCGCTACTGGA
T9-54	ALF4	ABD	TraesCS7A02G141600	7A-1818490-T9-54	reverse	93,064,447	GGAAGCTGCCGTGGGTGGGTTGGAAGGTGCAGTGG	15	65.2	2B-0441960-T9-5_128	ATGAAACAATATTTGAAAGAGTGGTGGTATGCAAAAG
T9-54	ALF4		TraesCS7B02G044400	7B-1937420-T9-54	forward	43,949,556	CACTTCAAAGGATCACTATTAGGAGCCAAGTTCTTTAA	13	65.4	2B-0441960-T9-5_192	AATAAATAAAAAAGTATAAGGGAAATAGGGTGAGAA
T9-54	ALF4		TraesCS7D02G142900	7D-2047690-T9-54	reverse	91,054,353	GGAGCTGCCGTGGTTGGAAGGGGCAGTGGAGGTGC	13	60.9	2B-0441960-T9-5_256	AATGACCCGGTTTCCAAACTAATTTCACTCGAAAAAA
T9-55	BHLH_TF_PTF1-like	BD	TraesCS7B02G084800LC	7B-1927230-T9-55	reverse	52,927,262	GGGTGCAGTTCACCGGCGCCGGCGCGCGCGGGGCGT	12	53.9	2B-0441960-T9-5_448	CTATATTAAAATCTCTAAAATATTTATATTTAAAAATAAA
T9-55	BHLH_TF_PTF1-like	no A	TraesCS7D02G149400	7D-2067140-T9-55	reverse	98,385,574	CGGTCTCGCGCGCGGGTGCCGGCCGGCCGGGCTGTG	1	7.1	2B-0441960-T9-5_512	ATTAATGACTTGATATTCAAATTTAATCGGCTCTTGGA
T9-56	ARABIDILLO-1	ABD	TraesCS1A02G217900	1A-0007840-T9-56	forward	385,887,871	GCAGCGAGTGATAGTCTCTTGTAATTGTCTTATGTCTT	5	28.7	2B-0441960-T9-5_576	TACATGCAAACAACGACAAGGTACACATACCCTTTTA
T9-56	ARABIDILLO-1		TraesCS1B02G231300	1B-0112750-T9-56	forward	415,221,038	CTGAAAAAAGGAAAAGGAATAGGGCAATATTAACAA	9	40.5	2B-0441960-T9-5_640	TTGATTTGTAAATTAGTTCGGCTCTTGGTTACCAAATT
T9-56	ARABIDILLO-1		TraesCS1D02G219600	1D-0205430-T9-56	forward	307,340,122	CGCCCCCGCACGAGCACGTCCGGGCGAGCGAGCGCG	2	10.8	2B-0441960-T9-5_704	ATGACGACGACGTATGCATTTCCTAGAGTTAGGTAAG
T9-57	Ethylene resp TF1	ABD	TraesCS6A02G343300	6A-1534140-T9-57	reverse	577,725,199	GATTGCCGGCGGTACCTCGTCCTAGTCGTCGCCGATTG	7	39.2	2B-0441960-T9-5_896	TACGTCTACATACGTTCATATATAGTCCGTATTGAAAAT
T9-57	Ethylene resp TF1		TraesCS6B02G375400	6B-1582630-T9-57	reverse	649,834,441	GATTGCCGGCGGTGCCTCGTCCTAGTCGTCGCCGGTG	15	71.8	2B-0441960-T9-5_960	CAAGGAAGTACAGTGTGATAAAACAAACACTCAAAC
T9-57	Ethylene resp TF1		TraesCS6D02G324200	6D-1712310-T9-57	reverse	430,847,473	GATTGCCGGCGGTGCCTCGTCCTAGTCGTCGCCGGTG	15	72.9	2B-0441960-T9-5_1024	TAAACTAGCTAGCTGTGGTCCTTTTTGATGATTTTCGT
T9-58	NRT1.1 (CHL1)	ABD	TraesCS1A02G210900	1A-0010190-T6-5	forward	373,766,720	TGAGCGCGCTTGAGAAAGAAACTCGAAGATGTAAACGAACAGTCAAAGCTCAATTAGCTAAACC			2B-0441960-T9-5_1088	TCCCACGGCTTACACTCCGTCGACATGCAAAGGCTCT
T9-58	NRT1.1 (CHL1)		TraesCS1B02G225000	1B-0090300-T9-58	reverse	403,582,209	CTCTGCTTCAAGATCAAGCCTCTCACGTGCTCGTGATG	12	58.4	2B-0441960-T9-5_1152	ATGACTCTATCTTGTAACTGTGGCCCAAAGCAGCCTA
T9-58	NRT1.1 (CHL1)		TraesCS1D02G214200		forward	299,576,089	GATCGCCGGGTAGGATTATGCTATGCGATGCTACTTGG	12	57.4	2B-0441960-T9-5_1216	TCTCCAGAGTCCAGCGGATATCATGGACCACGCCCGT
T9-59	Ethylene resp TF1	ABD	TraesCS7A02G158000	7A-1835500-T9-59	forward	112,263,640	ATGTGGCTTGGTTGGGAGCTCCGTCTTTAGATGTTAG	15	69.6	2B-0441960-T9-5_1280	GAACAAAAACGCCGCCTCGCCACCGAAAACAAGGTA
T9-59	Ethylene resp TF1		TraesCS7B02G062200	7B-1958510-T9-59	forward	65,660,513	AATCCTACACCTACCAATCCCTTCGAGTGAAGCCATTG	5	28.7	2B-0441960-T9-5_1408	CAGCGCCGAACGGAGCCAAGAACCTTCCTTTCATCA
T9-59	Ethylene resp TF1		TraesCS7D02G158500	7D-2046950-T9-59	forward	106,915,666	TGATAAAACACTTCCGCTTACGGTCTACGGGGGTACG	4	20.5	2B-0441960-T9-5_1472	AAACACCATGAAATTTTAGGATCTCAAAAAAGCCGCG
T9-6	DAO1	ABD	TraesCS2A02G327900	2A-0311890-T9-6	forward	559,620,859	GATAGATTGAATGAAAAGAATAGCTTCATGTTATTTAC	8	46.6	2D-1482860-T9-5_0	AGCGTATGTCGATAACAAACCTATATATTTAGGAAAGT
T9-6	DAO1		TraesCS2D02G335000	2D-0514640-T9-6	reverse	427,958,391	CTGTGCTGCTTTACTAGTTGGGCGGAGTCACGCGACG	15	71.8	2D-1482860-T9-5_64	CTGGATAAATACTCCTGCGTGTTGCTACAGGATGAAA
T9-60	OsNAC5(NAC_TF)	ABD	TraesCS4A02G219700	4A-0998160-T9-60	reverse	521,919,907	TCACGAACGGGGCTTGAGCTCGGAGGGCGGGCGCTC	7	36.2	2D-1482860-T9-5_128	AAAGCGGTAGATTATGGACAATTTTGAAAAGAATAAA
T9-60	OsNAC5(NAC_TF)		TraesCS4B02G098200	4B-1078970-T9-60	reverse	103,056,405	TCGCGTCCGGTGAACGGGGGGTTGAGCTCGGAGGGA	10	54.4	2D-1482860-T9-5_192	AGGGAAATAGGTTGAGAAAAAATCCCCACATCTGAC
T9-60	OsNAC5(NAC_TF)		TraesCS4D02G094400	4D-1171560-T9-60	reverse	69680159	TCACGAACGGGGGCTTGAGGTCGGGGGGCAGCGGG	13	67.5	2D-1482860-T9-5_256	CTAATTTCACACGAAAAAAATCCCTATTTAAATGAATG
T9-61	OsNAC9(NAC_TF)	ABD	TraesCS5A02G468300	5A-1185940-T9-61	forward	645726231	GCAGAAATTCGACAGTGCGGGGATGTTTTGTACGATT	12	54	2D-1482860-T9-5_448	AATATTTATATTTAGAAACAGAGGGAGTATTTGACAAA
T9-61	OsNAC9(NAC_TF)		TraesCS5B02G480900	5D-1467920-T9-61	forward	519,277,229	ATACGGCCGCAACTTCAATGCCGTATTTCAATGCCGAA	8	40	2D-1482860-T9-5_512	AAATTTATTCGGCTCTTGGACATTTAATTATTTTTGCAC
T9-61	OsNAC9(NAC_TF)		TraesCS5D02G481200	5B-1354040-T9-61	forward	653,432,486	GAGATAGGGTCGAAGGAGGTTGGATGCGGTTGGTGT	11	45.3	2D-1482860-T9-5_576	ATGTACACATACCCTTTTAACAGTAAAGATTAATTACAT
T9-63	TaZAT8	AD	TraesCS5A02G477600	5A-1235830-T6-1	forward	651,635,549	TACGACTGTAAATGCACATTGACGATCGGTCGAGCGCGTGGTGCATGTACCTCCACGGAGAAATT			2D-1482860-T9-5_640	CGGCTCTTGGTTTCCAAATTATTTTCACAATAATAGCC
T9-63	TaZAT8	no B	TraesCS5D02G491100	5D-1443700-T6-1	forward	525,250,314	TAGATTCATCATGCAGTTCATATGAAGAACTTGATGACAACTTTGAGGAGATTTTTTTAAAAGGG			2D-1482860-T9-5_704	TTTCCTAGAGATAGGTAAGATGTAGATGCTCTGACGA
T9-64	MAPK4	ABD	TraesCS7A02G422500	7A-1792950-T6-3	reverse	612,993,956	GAGGCTCTCTGTCTATTTTTTATTTCGGGGAAGGGAGGGAGGGAGGGAGGAAGGAAGGAACGT			2D-1482860-T9-5_960	TATTTAGAAACGAAGGGAGTATAATAAAACAAACACT
T9-64	MAPK4		TraesCS7B02G322900	7B-A1859240-T6-3	reverse	573,263,406	AGAGTTACCAGTCTATTAATTGAGAACAAAGGAAGCATAACAAGGAAAAAGTTTACTAGTTTAG			2D-1482860-T9-5_1024	GAGTATAAACTAGCTAGCTGTGGTCCTTCTTGATGATT
T9-64	MAPK4		TraesCS7D02G414700	7D-1974260-T6-3	reverse	532,901,470	ATTCTACCAAGAAGAAGGAAACACGGGTTAACATTCTATACTTGATAATAATGGAAGCATAGCAAT			2D-1482860-T9-5_1088	GGACATCCCACGGCCTAGACTCCGTCAACATGCAAGT
T9-65	TaMPK4	BD	TraesCS2B02G117900	2B-0448350-T9-65	forward	81,755,633	CCTAAGATCTAAACCTAACTGCTCTAGGGGGAAGGCA	20	88.5	2D-1482860-T9-5_1152	AGTACGTGAATCTATTTGTACTGATTGTGGCAGAAAG
T9-65	TaMPK4	no A	TraesCS2D02G100200	2D-0595260-T9-65	forward	52,227,288	GTTCGATGGGATCCCACCCCAGCGTATGTTCGCCAGAT	21	82.4	2D-1482860-T9-5_1344	ACGTAAAATCCCAGACAAATACCCCACACGAAATCTC
T9-66	Heme oxygenase	ABD	TraesCS7A02G375400	7A-1787570-T9-66	reverse	548,052,773	GGGTGCGGAGGCGCTCTTATAGCGCGCTAGGGTTCCG	4	21.6	2D-1482860-T9-5_1408	AACGCCGAACCGAGCCAGGAACCTTCCTTTCATCAAA
T9-66	Heme oxygenase		TraesCS7B02G277000	7B-1862220-T9-66	reverse	506,563,827	GGGTGCGGAGGTGCTCTTATAGCGCGCTAAGGTTCGT	11	53.4	2D-1482860-T9-5_1472	ACATCAAAATTTTAGTAGGATCTCAAAAACGCCGCGC
T9-66	Heme oxygenase		TraesCS7D02G371900	7D-2007060-T9-66	reverse	480,904,399	GGGTGCGGAGGCGCTCTTATAGCGCGCTAGGGTTCCG	19	80.2	2A-0311890-T9-6_192	TTTCACTCATTTGATATAACTGATTCCAGTTGTTTGAAC
T9-67	OsRPK1	ABD	TraesCS1A02G304200	1A-0021070-T9-67	reverse	497,509,046	TCTCTATCCACGGGCCAGTCCACGACGGACGGAGCAG	14	75.8	2A-0311890-T9-6_256	TTTTAATAATTTCCAAAAGCAAAGTTCGCCCATTTGAT
T9-67	OsRPK1		TraesCS1B02G314700	1B-0088950-T9-67	reverse	539,551,602	GTTGACGCTGGATAGCCGCCTGCAAAAACAAACCAG	17	75.4	2A-0311890-T9-6_576	ATAATATTACTTGGTTGTGAAAACATATTTCACTTAGTT
T9-67	OsRPK1		TraesCS1D02G303700	1D-0221570-T9-67	reverse	401,671,728	TCTATCCACGCGCCAGTCCACGGAGGACGGGGCAAC	18*	68.8	2A-0311890-T9-6_768	GAATTAAAAAAAAGTATTTCACTTACTTCAGAAACCT
T9-7	IPR011050	ABD	TraesCS2A02G423900	2A-0312050-T9-7	reverse	678,372,123	GGTGGCGTTGGAGGGGGGGCTGAAGGCGTCGGCGA	29	75.6	2D-0514640-T9-6_192	TGGTGAGACAATCAGCCGACACATATCCGTGTACGTC
T9-7	IPR011050		TraesCS2B02G443500	2B-0403410-T9-7	reverse	636,481,845	CAGACACCACCATAACACATTGGCACATTGCGCAGTT	29	67.7	2D-0514640-T9-6_256	TTACAGCACGATTGTCTCAATTTTGAAGAAACCATTG
T9-7	IPR011050		TraesCS2D02G421700	2D-0535750-T9-7	reverse	535,109,676	GCTTCCCCCCTTCTCTCTTGGGCCCGTCCTTGTGCTGCT	17	83.8	2D-0514640-T9-6_576	GTCTATATACATTCATTGGGCTGACAAGTATTTCCGGA
T9-8	IAA14	AD	TraesCS5A02G378300	5A-1197870-T9-8	forward	575,657,412	TCTGGTCCTATTTGGGCATGTATAATGGTTCTATCTTA	13	62.1	2A-0312050-T9-7_744	TAATTAAACTGCCATACGGATGTTGTAATTCAGCCATA
T9-8	IAA14	B in T9-49	TraesCS5D02G388300	5D-1448500-T9-8	forward	457,775,852	TAGGCGGACGGGGAGTGGAAAGTGGCGGAGTGGGG	5	45.7	2A-0312050-T9-7_806	TGCTCCCACGAAAAGATGTTCCAACGGAACCACGGA
T9-9	DUF547	ABD	TraesCS7A02G249900	7A-1846790-T9-9	forward	232,585,741	GTGTCCACATCCCAGCTATCCAGCAGCATCTACTGTAG	13	65.4	2A-0312050-T9-7_868	CCGAGAACCGCTTTCGTTTTAACTCGAAAAACAAAGT
T9-9	DUF547		TraesCS7B02G141300	7B-1948130-T9-9	reverse	179,258,690	GGCTAATGCTGAGTTCTCCTTCTCCTTGGCTGCTGCTT	12	61.6	2A-0312050-T9-7_1116	ATGCAACAAGAAGAAGGGCGGCCCCGACAAGAAGA
T9-9	DUF547		TraesCS7D02G248500	7D-2018470-T9-9	forward	220,274,988	CCTTTGCTTCTTTGGCCTCCGTCCGTGGGTCAGCCCAC	14	79.1	2A-0312050-T9-7_1178	GCGAGCACCTCCATGCTTCTTGCTTGGGCCCGGCCTT
										2A-0312050-T9-7_1302	TCCTCTGTCGGTGTCCGCGTGGCACCGGCGCCGTGG
										2A-0312050-T9-7_1364	AGACGGATGCACGCATATATAGGTGAGGTGTGGTGCC
										2A-0312050-T9-7_1550	AACAGCAATGGGGCCATCGTCGTGGCAGCAGTAGGC
										2A-0312050-T9-7_1612	TCTTCTGCGCTCTTTCCTGAGACTGTCACGGTGCACCT
										2A-0312050-T9-7_1674	AAAATGATACTACTACGTGCAAATTTTGCAATGCCAAC
										2A-0312050-T9-7_1736	AGATCCGTGCGGCAGCGACCAGTTTATCACCGCGTGC
										2A-0312050-T9-7_1798	TACAAACGGTAACTCTTTTTTTTTTTGAGGATTACAAA
										2A-0312050-T9-7_1860	GGTGGTGAATAAAGTAGTCCGTGCGTGTGTGACTGCC
										2A-0312050-T9-7_1922	GGTGGTGTGTGTGCGCGCGTGCGCCCCTGTTTAATCG
										2A-0312050-T9-7_1984	AAGCCCGTTTGGTGTGCTCGCATTATTCTTtcctcctcttc
										2A-0312050-T9-7_2046	ctcctTTGCTTGGATCCCCAAGCTTTCTCTTCCTTGCTTT
										2A-0312050-T9-7_2108	CTTTGTTCCCTCCCTCCCTCCCTCCTGGCCCATGCATG
										2A-0312050-T9-7_2170	AGTAATCCCTAGTGCCCGTTTATGCACTAGTAGTGTAC
										2A-0312050-T9-7_2232	ACTTGTTGAGCACAATAAGAACGCTTTTACTATTGTAT
										2A-0312050-T9-7_2666	GGTTTTAAAAAAGGATCAAAATACGAAATTTAGCCTC
										2A-0312050-T9-7_2728	TACAAGATGAGACTCGGAGACCAGGTGCCACGTACT
										2A-0312050-T9-7_2772	TGGAACTACTCTAGCGGCAGCAAGACGCTAGAATTCA
										2B-0403410-T9-7_305	AACGAAAATCCACCACGTCAGACGTGACAATAATCGG
										2B-0403410-T9-7_427	GACATTTGTCGGGTGGGTGGTTGGGAGGTGGGGCA
										2B-0403410-T9-7_488	CCCAACACGGGAAGAGAAGGGAGAATCATTTGTCGA
										2B-0403410-T9-7_549	CCTAACATTCTTGACGAAACTGCATTTGTGAGCTGAC
										2B-0403410-T9-7_610	AGAGGTAGATTCAGGCGCACGGCGTGCGTGTCCCTG
										2B-0403410-T9-7_671	ACGCAAACGCAGAGACGGAAACACAACGGGAAGAT
										2B-0403410-T9-7_732	GTTTTATTTTTTATAAATAAAAAAAGGTTCCTTTTGGG
										2B-0403410-T9-7_793	CCTCTGCAAAAGCGAATGAATGAGAAATCTCCCAGAA
										2B-0403410-T9-7_854	TGTTCCTCCGCGCTTCTACTGTAGTAATTTGTAATTAAA
										2B-0403410-T9-7_915	AATCAAGGAAACGAACGTTAACAACAACTGCTCCCAC
										2B-0403410-T9-7_976	GGAAAGGCGATGAATGTAATCACATGATTCCAAGAAC
										2B-0403410-T9-7_1037	AAAGTGATTAGAAGCAGAGAGCTTGAAGGAACTAAT
										2B-0403410-T9-7_1281	ACCAACAAGAAGTAGCGCATGCATGAGGCAGCCGCG
										2B-0403410-T9-7_1342	CTCAGGCTTCCCCCCTTCTTTCTTGGGCCCGCCCTTGT
										2B-0403410-T9-7_1525	CGAGGGGGCGGTGTAGGATACACGCATATATAGGTGT
										2B-0403410-T9-7_1708	GGTCAAGTAACTGAAGGAAGTGGGAGAGGAGAGGA
										2B-0403410-T9-7_1830	GCAACAACAACAATGGGGCCATCGTCGTGCCAGTATT
										2B-0403410-T9-7_1891	AAATACAAAGCCTTCTGGGGACTGTCACAGTGCACCC
										2B-0403410-T9-7_1952	AAAATGGTACTAATACTATGAGAGTACGTACGTGAGA
										2B-0403410-T9-7_2013	AGGGGCAATCTAGTACAGATCCGACCGGCAGTGACA
										2B-0403410-T9-7_2074	GAGGTGCCGACCAGAGACCATCATTTTACCAACGGC
										2B-0403410-T9-7_2135	GCTGAATAAAGTAGTCTTCCATTCAAAAAATAAAGTA
										2B-0403410-T9-7_2318	GTGCTCTCATTATTCTTTCCTCCTCTCCTCTTCCTCATC
										2B-0403410-T9-7_2379	CCCAAGCTTTTTTTTTCCTCCCTTGCTTTTCCTTGGGT
										2B-0403410-T9-7_2440	CTCCTGGCCCATGCATGCTTGAGAGTTGAGAAGTTGA
										2B-0403410-T9-7_2501	TCTGTAAAAATAAATAAATAATCCCTACTCCTTTTATGC
										2B-0403410-T9-7_2562	GTGACTTGTTGAGCATAAGAACGCTCTCCTACACACG
										2B-0403410-T9-7_2921	GAATTATGTTTGAATTTTCTAAATGTTAAATTATGAACA
										2D-0535750-T9-7_0	GCTTCCCCCCTTCTCTCTTGGGCCCGTCCTTGTGCTGC
										2D-0535750-T9-7_192	CCGGGCGAGGGGGCGGTGTAGGATACGCGCATATAT
										2D-0535750-T9-7_320	TCGCGCGATGGTTACAAGGTTGGTGGTGGAAGGGGT
										2D-0535750-T9-7_512	TTCCTGGGGTTTGGCAACAGAAATACAAAACCTTCTG
										2D-0535750-T9-7_576	ACCCCGTTTGTTCTCAAGACTGATCACGAAAATGATA
										2D-0535750-T9-7_640	TCAACAGACAGACAGGGGCAATCTAGTGCAGATCCG
										2D-0535750-T9-7_704	CGTGCATGGATCGAGGTGCCGACCAGACTTTACAAA
										2D-0535750-T9-7_768	GTGAATAAAGTAGTCCGGTCCGTGTGGGAAGTGACT
										2D-0535750-T9-7_896	GTTTAATCGGGTCCAAGGAGAGCCGATGAAAAGCCC
										2D-0535750-T9-7_960	CCTCTCCTCTTCCCCATCATGTCATGTCCCTTCTTCTTT
										2D-0535750-T9-7_1024	CTTGCTTTTCCGTGGGTGCTCTCCTTTTCCCTTTGTTC
										2D-0535750-T9-7_1088	ACAAGTTGAGATGCTGTAATCCCTATTGCTCGTTTCTT
										2D-0535750-T9-7_1152	CCCTAAAGCTACTGGTTTATGGACTACTGACTCTACAA
										2D-0535750-T9-7_1216	AGGACGATCTCCTACATTCGTACATTACATATCGGTTG
										2D-0535750-T9-7_1280	CGCAGAGTCAGACTCAGACACCTGCATATAGTAGCTG
										2D-0535750-T9-7_1344	GATTTGTTGGGAGGTTCTGCCAACTTTTGGCAGAAG
										2D-0535750-T9-7_1408	TTTCCCCGAACCAACCAGAATTACTTGGCCTTTACACT
										2D-0535750-T9-7_1580	TCGCGGACTATGTTACAGCGAAGCGAGAATAAGAGG
										5A-1197870-T9-8_256	agactatcttatcaaccattgtacatacccTATGTAGAATCAATT
										5A-1197870-T9-8_320	CACCGCATAGCCAGCCGAATAACGGTTCTATACAAGA
										5A-1197870-T9-8_384	AAGTACAGTAGATTCGAAAAAGATTACACACCTACAG
										5A-1197870-T9-8_448	CTGCGGATGGCCCCCACGTGATCGGATATCCATGCAC
										5A-1197870-T9-8_704	ACGTCGGGGTCGGGCTAGGGACCAAAAACAGCGGC
										5A-1197870-T9-8_768	TGAAACGTACGCGTGCCGTGTGGGCACTCCGATCCGT
										5A-1197870-T9-8_960	CCACTATAAATAACATACCATTGTAGTATATAGATGTATT
										5A-1197870-T9-8_1024	GCTTCATATGTAGTCTATGGTCCCCCGCAAATTTTTTA
										5A-1197870-T9-8_1088	TCTCTACAAAGATTTATATTTAGGAACGGGGAGAGTAT

										5A-1197870-T9-8_1152	tgatgtatagtagatgttgacatgtttttTGACCAGGAACAAAT
										5A-1197870-T9-8_1216	ACGGAAGGGGGTAGTATAGATATTGGCTACTACTAAC
										5A-1197870-T9-8_1280	AAGTCCAAAAAAGAATGTTGAGATGGTTCGCTGCCA
										5A-1197870-T9-8_1344	CTTGTGTTTGCGTGTGCGGGTTGACGGTACGATACGT
										5A-1197870-T9-8_1408	TAGCACTCCATTGGAAGGAATGGAGCGTGTACGTACC
										5A-1197870-T9-8_1472	CCAAATTGCATACGTATCCTGTTCATTCCGATATCATAT
										5D-1448500-T9-8_384	GTGAGCGGGCGGGCCGCCAAAACGTTTGAGGGTGG
										5D-1448500-T9-8_448	CAAGTACGTAGTAATATGAAATTTATATGTCAGGTCAG
										5D-1448500-T9-8_512	CAGCATGTGCCACTTTTTTTGGGGGGCGATTCGCATG
										5D-1448500-T9-8_640	TGGGAATGGAGCGTGTACGTACCATCTAACCTACTTT
										5D-1448500-T9-8_704	TATCCTGTTCATTCCGATATCATATAGGGATCTCTATTAT
										7A-1846790-T9-9_0	GTGTCCACATCCCAGCTATCCAGCAGCATCTACTGTAG
										7A-1846790-T9-9_64	TCTTTCCATGAGCAACCGCCGGAAAGCCAACAATCA
										7A-1846790-T9-9_128	GTGTACTAGCAGTAGCATAGTTCACCTAGGGCTACAT
										7A-1846790-T9-9_576	ccccccgcccacgcccacgcccacCTAATCATGCACACTCTT
										7A-1846790-T9-9_640	ATTTTTCCCCTGAGATTTTTTCTGTCTGGATCATGGTT
										7A-1846790-T9-9_704	CATAAACTAAGAAAGATGATGATTACAATTTTTTCCTT
										7A-1846790-T9-9_768	TGAATTTCGAAATATATACTACATCACACTAGTGAGAT
										7A-1846790-T9-9_832	AGCTGCTGTGCCGTCACAAAAACCTCTCCCTACAGAC
										7A-1846790-T9-9_1024	CTCCTCGCTCCCTTTTAACCTCTGCTTTTCCCTTGAGT
										7A-1846790-T9-9_1152	CCTTTGGCTAATCCTTAGCTGCTCCTCTCGTTGAGTCT
										7A-1846790-T9-9_1344	CTGCATTTTCCTTCCAATTTTTCTTCCCTTCCCTTTGCC
										7A-1846790-T9-9_1408	GGTAGAGAAGGTTTTCTCATCACCCGGACCAGGCTG
										7A-1846790-T9-9_1472	GTTTCCATTGGCGGCTTGTAGTACACAGCAGTAACTC
										7B-1948130-T9-9_128	CTGGAGAGAAGGACAAAGTCATCTAATCACTCGGCT
										7B-1948130-T9-9_192	AGTTACTGCTGTGTACTACAAGCTGCCAATAGAAACA
										7B-1948130-T9-9_256	CCTGGTCCGGGTGATGACTGATGAGAAACCTTCTCTA
										7B-1948130-T9-9_320	CAATAAACAGAGAAGACAAAGGGAAGGGAAGAAAA
										7B-1948130-T9-9_448	ACGCACTAATGCTGGCAGTTGTATATAGGCATAGGGA
										7B-1948130-T9-9_640	ATACGGTGCGGTGAAAGGGTGGTAAAACTCCCGGAA
										7B-1948130-T9-9_832	GGACGCAATGATAGGATTAAGATGTCTATAGTGAGAG
										7B-1948130-T9-9_896	GATGGACAGTGACCGGTACCCAATCTCACTAGTGTGA
										7B-1948130-T9-9_960	CCTAATTTTTAGAAAAACAACAGTAATCATTGCCTTTC
										7B-1948130-T9-9_1344	GTTCCGTTTGCAGCTTACAGGAGCCGTTTGGTGTACC
										7B-1948130-T9-9_1408	CACAAAGATTTGGTCTATGCATGGAGCCCTAGGCACA
										7B-1948130-T9-9_1472	TGATTTTGGTCTCCAGTAGTTACCCATGCAGCAACGA
										7D-2018470-T9-9_64	GGGGGAGGGAGACACACTGATAGGCGCCACTGCTC
										7D-2018470-T9-9_256	TCGTCCTTTGGCTAATCCTTGGCTGCTCCTCTCATTGA
										7D-2018470-T9-9_384	TCATTTCTGTTGAGCTTAGCTCCATTGGCTCTTGCTTG
										7D-2018470-T9-9_512	ctggagctgggagggagatagagAAGGTTTCTCATCAGTCAT
										7D-2018470-T9-9_576	AATTGGCAGCTTGTAGTACACAGCAGTAACTCATGAT
										7D-2018470-T9-9_832	CGCCTGCAACACCGCCACACCCGAAGGTTAGTATGCA
										7D-2018470-T9-9_896	TCATTAAATTTGTTCTTCTAGCAGTCCTCCAGGGTAAG
										7D-2018470-T9-9_960	GGTAAAAAGGTATAAAGAGCAGTTTCGACAGAGCAT
										7D-2018470-T9-9_1024	TTGATCAATATTTACGTATTTTCTGCCAATCTTACATCT
										7D-2018470-T9-9_1152	ACCAATGTAGACAGTTGTACTTGCACATCATTTTCCAG
										7D-2018470-T9-9_1216	GCAGTCTTGCAAGTCCTTTTGACCAACAGAACATTTG
										7D-2018470-T9-9_1280	cttttTCAAATGACAGAACATTATGTCTTTCAATAAAAAG
										7D-2018470-T9-9_1344	CTTCTCTCTTTTTTGGACAAACAGTTACTTTAAATGAA
										7D-2018470-T9-9_1472	ATTCTCGTATAACAAACCTGTGGGTAATCTAGGCTCCC
										7D-2018470-T9-9_1536	TTTTCTATGTTGTACTATTGTACATAAAAAAAGTTCAG
										7A-1788290-T9-10_0	CGCGCCCGACCCGACGAACTAGCTAGATCCCAACCTA
										7A-1788290-T9-10_64	CTGGGGATCGATGGATCGATCGGGGTGACCTTGAGT
										7A-1788290-T9-10_128	TATAGGAGAAGATCGTCGTTGAAATGGTTGTTAATTA
										7A-1788290-T9-10_256	GGGGACAAGGCGCGTACACTTGCACGCATTCATTCA
										7A-1788290-T9-10_320	TCTTCTAGCCGGCGGTGATCAAGTGTAAACACTTCTA
										7A-1788290-T9-10_384	AGACATGAACACCAGGCTGGCTGGTTGGAAGAACCA
										7A-1788290-T9-10_576	TTGTGAGATGTCGAGGGAGGTGGGCGCTCGAGCGG
										7A-1788290-T9-10_704	CGCGTCGGTTCATGGCTCTGGACGGCGGTGAGCTAG
										7A-1788290-T9-10_768	GCAAGGTTGTTATTTTTCAGGTTTCCTTTAGGATCTTG
										7A-1788290-T9-10_832	ATGTCACTGCTTCTACAAGCTCTTGAATTTTAAAACGT
										7A-1788290-T9-10_896	GGCGGCCTCGAGCTATGTTCACAGCGTGCCATCGGC
										7A-1788290-T9-10_960	CACAAGATTTTGAATGTAATTTTCTTTTTCTATAAGGAT
										7A-1788290-T9-10_1024	AATATATAGTACTACTAGGCCTTTTCACGAAAAAAATA
										7A-1788290-T9-10_1088	CTCTGCATAGATCTACCGTCCACCGCTACTTTGAGATT
										7A-1788290-T9-10_1152	AAACACCGAAACATTTTGATACATGTTGGAACAAGGA
										7A-1788290-T9-10_1280	CTACCAGCCAAGCAGCTGCATGTGGGTATGTGTGAAT
										7A-1788290-T9-10_1344	AGACAAGACTGGAGCCTCTAGATACCTCTTGCCAAAG
										7A-1788290-T9-10_1408	CATAGCAAGGTAGCTAGATGTCAAGACACGACATCAC
										7A-1788290-T9-10_1472	AGGTACGTAGGGCCCGTGAGCCAGCCAAGTCATGTA
										7A-1788290-T9-10_1580	GTTTAGCCGAGTGTGGAAAAGGCGTGGCTTGGCTTG
										7B-1897450-T9-10_0	TGCTACGTTATGTGCCGAGTCCATGCCATGTCTACTCT
										7B-1897450-T9-10_64	CTTGTCTCCGGCATCCATCAGATCCATCACTTGATCCA
										7B-1897450-T9-10_192	AATCCGATGCGTGCAAGCGTACGCACCTTGTCCCCTG
										7B-1897450-T9-10_256	TCAACATGTATCAATATGTTTCATTGTATATCGGTAAAT
										7B-1897450-T9-10_320	TCTTTTTTGGGCTTTCAAACAAGAGGATGACCACAGT
										7B-1897450-T9-10_384	GACAAATGTTGGCAAGAAAAAAACATGAATCTCAAA
										7B-1897450-T9-10_576	CTGGGCCTGCCCCTTGGTAGAGAAAACTTTTTAAAAC
										7B-1897450-T9-10_640	GTAGTCATCAATGCTGACCCAGAGTTGTCAGCGGCCA
										7B-1897450-T9-10_704	AGAAAACAAAACACTGATAAGGGTCTGTAGTAAGTCT
										7B-1897450-T9-10_832	ATGCCCTCTGCTGGCGCAAAGCACACCAACAAAACA
										7B-1897450-T9-10_896	ACCTTGGGGCAACACCTTCATCTCGCCACCTCAAACC
										7B-1897450-T9-10_1024	AGCCATGAATTGGCGCGCTCTTCTCTGGTGGTCTAGT
										7B-1897450-T9-10_1088	ACTGGATTCAACAACACCACCGTCGCACACTGCTTGA
										7B-1897450-T9-10_1280	CCCTAGCCTCGAGAAAAACCAGCTTGGTTCTTCCAAC
										7B-1897450-T9-10_1344	GTCTCCTACACAACCTAGCTAGTAGTAGAAGTGTTTAT
										7B-1897450-T9-10_1472	TTGGAATCTTTCCCCGCACACATGCACCATGCTTAGCA
										7B-1897450-T9-10_1536	ACCACTTACTTAAATTAAATTAACAACCATTTCAACGA
										7B-1897450-T9-10_1580	CCTATATAGCCACCCTTCTACTTCTCTTCCCACACTCTT
										7D-1969450-T9-10_192	CCTTGATCACCGCAAGAAACCAAAACCAGTCCGATG
										7D-1969450-T9-10_256	GACATTACTAGTTTCTTTTTCCTTGTTCAACATGTATCT
										7D-1969450-T9-10_320	ATCGGTAAATTCAATCTGAAATATGTGCACTCTTATCTG
										7D-1969450-T9-10_384	GAAACAAGAGAAAACCACGATGAAACGTTACGAAAC
										7D-1969450-T9-10_448	AAAAAAACGCCGGGTAACTTGTTTCCTTTTTTTGCGA
										7D-1969450-T9-10_512	CACATTCCTTACAAACACACCCTTACAGAAAAAAAAT
										7D-1969450-T9-10_640	TTGTAGAAGCAGTGACATGGAAGTCATCAATGCAAA
										7D-1969450-T9-10_704	GACCTGAGAAAACAAAACACCGATAAGGAACCGTAG
										7D-1969450-T9-10_768	AATCCAGGCGGATGCACTAGAAACCTAAACGGACCT
										7D-1969450-T9-10_832	TTCCACAAGCCCTCCTCCGGCGCAAAGCACACCAACA
										7D-1969450-T9-10_896	gggAACATTATTCCAACACCGCCATCTCTCCGCCTCAA
										7D-1969450-T9-10_1024	CTTCTCTGGTGATCTAGTCATGCCGGGGCACCGATGA
										7D-1969450-T9-10_1088	TCTCACGCACTTGACGTAGACCCCGGCTCGAGCGCC
										7D-1969450-T9-10_1280	CGGCTAGGGTTTTGCGGTTCGCATTGCCGCTAACCTC
										7D-1969450-T9-10_1344	AGTCTTCATGTCTCCTACACAACCTTCCTAGCTAGTAG
										7D-1969450-T9-10_1536	CCCAGTGTAGGGCACGTACCACTACTCTCTTAATTAAC
										7D-1969450-T9-10_1580	TTGAACGATCTTCTCCTAGCTATATAGCCACCCTTCTAC
										1A-0017770-T9-11-1_64	TGGACTTGATTGAGAGGCGGAGAGAGAGAGCTTGT
										1A-0017770-T9-11-1_256	TTGCCTGATGATTTTCGAAAGGAAAAAAGGTCCTATAT
										1A-0017770-T9-11-1_384	AAACGGGACCTCATACTACGCGGTAGCTGATTGATTG
										1A-0017770-T9-11-1_512	CGTGCACATTCTCGCTAATTCCCCCGCACAAAATGTAA
										1A-0017770-T9-11-1_640	CTAAACACTCTTATATTAGTTTACGGAGGGAGTAGCA
										1A-0017770-T9-11-1_704	ATCATCCATACAAATGGCCCATCGCGCATCGATTACCA
										1A-0017770-T9-11-1_768	CGTGGGATTTCTCTTGTCTCGAAGCGACACTTCACCA
										1A-0017770-T9-11-1_896	TTGTGTAGGACACCATTAGTAGGTTAACTTGTACCTTA
										1A-0017770-T9-11-1_960	GGCATGTCAAATGAAAGGTAAGTACTGTCAAATGAAA
										1A-0017770-T9-11-1_1024	AACTATTATTATTATACAATAAAAATTCCCATAGGATTT
										1A-0017770-T9-11-1_1088	ACAGCCGTAATTTGGCCAATAGAGAGCAACTAATCTT
										1A-0017770-T9-11-1_1152	TACAACAAATGGACATATGAAGGTTCTGGTAGCCTTA
										1A-0017770-T9-11-1_1280	CATCGTGCAAAAAAATATGCACTTGACAGCTCCCTTG
										1A-0017770-T9-11-1_1472	TAGTGTATTCAGCAGACTTGGAACAAATTGTTTGCAA
										1A-0017770-T9-11-1_1536	TAGGCAAGGAATGATGGCTGACAATATTAATACTCGTA
										1B-0111050-T9-11-1_128	ATCAACGTATGTGCAACTAGTCTACATTACAAGCCAAC
										1B-0111050-T9-11-1_320	ACTGAATAAGGCGAGGCCAAATCGGTGTAGCTAACA
										1B-0111050-T9-11-1_640	CGTCGCTCCGCCGAACAGAGCGTCGGCGAGGATCTC
										1B-0111050-T9-11-1_832	GTCTAGCGCGGCCGCTCGGTTCGACTAAATCGTGCAC
										1B-0111050-T9-11-1_896	TAATTGTCCCAACACGGGCGAAACATGACCTAGGAAC
										1B-0111050-T9-11-1_960	TGACGTGTCGCTTTGAGAAAAGAGAAACCCGCGTAT
										1B-0111050-T9-11-1_1024	TTCGGTGTGCGATGGGCCATTTGTACGATGGTAATAAT
										1B-0111050-T9-11-1_1088	CCTCTGTAAGTTTTTGATGGTCAAGAAATTCCTGATTA

1B-0111050-T9-11-1_1152	AGAATGTGCACGGCTATACGGCATTGGTTAAAAAAA
1B-0111050-T9-11-1_1216	CGTGTGCTCAGGTCGTTTCATCTGGTCAATCAATCAG
1B-0111050-T9-11-1_1280	ATAAGGTCCCGTTTGCTCCACGAAATATCGCGGCGAT
1B-0111050-T9-11-1_1472	TTCGACCAGCGTCGTGCCCTCCGTGCCCCTCTCGACT
1B-0111050-T9-11-1_1536	AACGCCTACAAATATCCATCCCTCCGCCGAGAGCCCG
1D-0205980-T9-11-1_448	CCGTCCGTCCAGCCGCGGAGGGATCGggagggggagg
1D-0205980-T9-11-1_512	TGGATACTTACAGAGGAACTGGAGCTCGCGGGGGCG
1D-0205980-T9-11-1_576	AGAACAGCAGCCAAGCAAATACTTTGACTTTGTTGGG
1D-0205980-T9-11-1_640	TTGGGGTGGACGTCAATCTTCTGCCATGGTCTTTGAG
1D-0205980-T9-11-1_704	CCTCAAGGCGGAAGTCCGGCTAGACAGCAAACGCAG
1D-0205980-T9-11-1_768	GTGCAAGCAATTTATCATTGACGGACGGAAACGTGG
1D-0205980-T9-11-1_832	AGGAAGGAGTCAAGGTCACGACGCGCACGGGCGAC
1D-0205980-T9-11-1_896	CGTGCATGGCTTTGGTGGCATGCCCATTTCGAGGAAA
1D-0205980-T9-11-1_960	CGCTGGTTCGCCGTGGTTTTGAGGGTGCGTTTGGTT
1D-0205980-T9-11-1_1024	TGTCTTAAACCTGCTTGGTGATTATTTGGACCATCAAA
1D-0205980-T9-11-1_1088	GTGCGGGGGATCAGCGAGAATGTACACGACTACACA
1D-0205980-T9-11-1_1152	CGGTAATGTCAAAATAAAATAAAAATGAAACGCCATC
1D-0205980-T9-11-1_1216	GGCTGATCATTCAGCCCTCTCGTAAGGTCAAAATAAA
1D-0205980-T9-11-1_1280	TTCGGAACCGCGTATGAGGCCCCCTTGTTCCACTCAC
1D-0205980-T9-11-1_1344	TTCTCAGCATTTTCCCGTGCACTGCGGTGCCGCGTGA
1D-0205980-T9-11-1_1408	TTTTCCTTTTCGACGAAAATCATCACCAGCCAACTCAC
1D-0205980-T9-11-1_1472	ACAGGGCAAGGCACAGGCGCGTGGGCCTCTTGGAC
1D-0205980-T9-11-1_1536	GGCTCCCGACTCCTGTAGGCTGCGTATGCGAAGCGCC
1D-0205980-T9-11-1_1580	TACGCTTGCTCCGCTGGAAGCCCAAGGCACGGGATT
1A-0017770-T9-11-2_0	AGGATCACTCTGCACCAAGCAGAGTTATAGCTGAGG
1A-0017770-T9-11-2_64	AACTAAACAGAATTAAGCACCATAATTTTCTGTTACAC
1A-0017770-T9-11-2_128	TACAATGAAAATTTTGACAGTAGGATGCATGTTTCTAG
1A-0017770-T9-11-2_192	CAAACATAATGATTGAATATGAATAGATTGGCAATGCA
1A-0017770-T9-11-2_256	CAAAATAAACTGAAAAAGACAGCAGTATATCTTGAAA
1A-0017770-T9-11-2_320	GTTTCATGTTTCAGCTATTCGAAATGTAAGCCTTCTTG
1A-0017770-T9-11-2_384	TGCATTGTTGTTCTTTACATATAACTTATAGGGCCAAA
1A-0017770-T9-11-2_448	TGTATCACAACTTTGATCATAGAACTACAAGGAGAAT
1A-0017770-T9-11-2_512	GTTCATGTACACTTATTTTGGCCCTAGAAAAGGTGAA
1A-0017770-T9-11-2_576	GGCATGTGGATACAGAATCACAATTCTGCACCTGTTC
1A-0017770-T9-11-2_640	TTTTAAACTTGACATTTTTTAGCAAAAAGCCAAAGGA
1A-0017770-T9-11-2_704	AGTTCCAACAGAGTTTAACTTCACTTTTTAAGTTAGG
1A-0017770-T9-11-2_896	ATGTCAATCAAGGCAGATCATATTGACATCCACTATCC
1A-0017770-T9-11-2_960	TTACTATTGGCTAGTTGAGAACATATTAGCACGCAGC
1A-0017770-T9-11-2_1024	TTATCGCTGGCTGGAACATATCCAAGCATATGGTATCC
1A-0017770-T9-11-2_1216	CATTTCTTGTACCCATTGTCACAAATCATGATACATAGG
1A-0017770-T9-11-2_1280	TCCTTAGATCCTTGAGAATTCGAAGACATGGTATTATA
1A-0017770-T9-11-2_1344	AATAGATTGAGTAAATCTTCATAAAATAAAATAAATTG
1A-0017770-T9-11-2_1472	CGATTGGTGTCCACCACCCACTCCAACCTAAAGTATA
1A-0017770-T9-11-2_1536	AAATACTACAGCGTCAAAAATATTAATCAATATTAAAA
1B-0111050-T9-11-2_64	TTACAGTTTTTATTTACAAAATGGCATACGAGTTCTTG
1B-0111050-T9-11-2_128	CCTGACGGAAGTGACGAAGTAATGTTTTTCAGTTTCC
1B-0111050-T9-11-2_192	ATATAATTTGATGGACTGGTCTCTGGAAAACATATTTT
1B-0111050-T9-11-2_384	GGACGATGAACTTTATGTCCTACATACAACCAAACAA
1B-0111050-T9-11-2_448	TCAATCTATTTCAATTAGGAGTTGTTCAGACAATATAA
1B-0111050-T9-11-2_512	CCAAGAATGTGCTTGCAGTGGGTTGTGCGCCACCTA
1B-0111050-T9-11-2_576	GGAAATGTGCGCCACCTATAGAGTTCATCTGTTAGAA
1B-0111050-T9-11-2_640	AAGGATACCATATACTTGGATATGTTCCAGCCAGCAAT
1B-0111050-T9-11-2_704	GGTGCTGCATGCTAATATGTTCTCAACTAGCTGATAGT
1B-0111050-T9-11-2_768	ATAGTGGATGTCAATATAATCTGCCTTGATTGACATGT
1B-0111050-T9-11-2_960	CTAACTTAAAAAGTGAAGTTAAACTCTGTTGGAACTG
1B-0111050-T9-11-2_1024	CCTTTGGCTTTTTGCTAAAAAATGTCAAGTTTAAAAC
1B-0111050-T9-11-2_1088	AACAGGTGCAAATTTGTGATTCTGTATCCACATGTCAT
1B-0111050-T9-11-2_1152	TCACCTTTTCTAGAGCCAAAAAAAGTGTGCATGAACA
1B-0111050-T9-11-2_1216	TTCTCCTTGTAGTTTTATGATCAAAGTTGTGATACGTA
1B-0111050-T9-11-2_1280	TGGCCCTATAAGTTATATGTAAAGAACAACAATTCAAG
1B-0111050-T9-11-2_1344	AGATGGTTACATTTTGAATAGCTGAAACATGAAACCA
1B-0111050-T9-11-2_1408	TCAAGATATGTTGCTCTCTTTTTCAGTTTATTTTGATAT
1B-0111050-T9-11-2_1472	ATTGCCAATCTATGCATATTCAATCATTGTGCTTAAGTA
1B-0111050-T9-11-2_1536	GAAAACATGCATCCTACTGTCAAAGTTTTCATTGTGG
1B-0111050-T9-11-2_1580	AATCTTTTACAGCAAAGTGTGTAACCGAAAATTATGG
1D-0205980-T9-11-2_0	ACTCGGATGCACTGTGTTCCCTACGTTTTTTTTGTTTT
1D-0205980-T9-11-2_64	ATTCGTTACACTTTTTATTTACAAAACGGCATACGAGT
1D-0205980-T9-11-2_128	TAAGATCCTGACGGAATTGACGAAGTAATGTTTTTCA
1D-0205980-T9-11-2_192	GATCACATATAATTTGATGGACTGGTCTCTGGAATTAT
1D-0205980-T9-11-2_384	ACACTGGACGATGAACTTTATGTCCTACATACTACCAA
1D-0205980-T9-11-2_512	AAGGATCTAAGGATGTGCTTGCAGTGAGTTGTGCGC
1D-0205980-T9-11-2_576	TGTGACAATGGGTACAGGAAATGTGTGGTCTCTTGTT
1D-0205980-T9-11-2_640	CATTTATAATGTTTCCAATGCATGTTCAAGGATACCATA
1D-0205980-T9-11-2_704	TAATGTAGAACTAGTTTGGTGTATGTCGGTGCTGCATG
1D-0205980-T9-11-2_832	CTTCCTGCATTGCAGCTATCCTTTTCAGCTTCCAAGTT
1D-0205980-T9-11-2_896	TAAAAACTACCATGCATTTCGATCTGTACATATATTTATA
1D-0205980-T9-11-2_960	CTGTACCCTAACTTAAAAAGTGAAGTTAAACCCTGTT
1D-0205980-T9-11-2_1024	TGGGGCTTTTTGCTAAAAAATGTCAAGTTTAAAACAA
1D-0205980-T9-11-2_1088	CAGGTGCAGAATTGTGATTCTGTATCCACATGTCATTT
1D-0205980-T9-11-2_1152	CCTTTTCTAGAGCCAAAATAAGTGTACATGAACAATAT
1D-0205980-T9-11-2_1216	TCCTTGTTCTATGATCAAAGTTGTGATACATAAGGAAA
1D-0205980-T9-11-2_1280	TATAAGTTATATGTAAAGAACAACAATGCAAGCTGAA
1D-0205980-T9-11-2_1344	AGATGGCTTACATTTTGAATAGCTGAAACATGAAACC
1D-0205980-T9-11-2_1408	TTCAAGATATGTTGCTGTTTTTGTCAGTTTATTTTGATA
1D-0205980-T9-11-2_1472	CATTGCCAATCTATTCATATTCAATCATTGTGTTTAAGT
1D-0205980-T9-11-2_1536	AGAAACATGCATCGTACTGTCAAAACTTTCATTGTAGT
1D-0205980-T9-11-2_1580	AATCTTTTACAACAAAGTGTGTAACAGAAAATTATGG
1A-0030760-T9-12_0	TTATAACGACCGCTCTAGCATACGACTAATTTCTAGCA
1A-0030760-T9-12_64	TAAAGCAGAATATAAACAACGACTATCATGAGTATACT
1A-0030760-T9-12_128	CATCGAATAGGGTACAGGTAATGTGTGGAATTTTATAG
1A-0030760-T9-12_192	GGCAGGCTTTCGCATCAAATGTATTCCATCTTGGACG
1A-0030760-T9-12_256	GAAATCTACAAAATATAAACAACAACGATCACACGGT
1A-0030760-T9-12_320	taggtaatagtaaataaatacaataaataGGATATACACCATAT
1A-0030760-T9-12_384	TTTGCCTTGATTCATGTTTTTTATATGTTGGCACATTTT
1A-0030760-T9-12_448	CCTATCTTTCATACGCTTATATCCATCTATATCTCTGCCA
1A-0030760-T9-12_512	TAAAATCTTTATATAATATGTTGAAGGTTATACGGGTAG
1A-0030760-T9-12_576	TATTAAAGTTCCGTTGACATTTAAGGTCTTTCTTTGGA
1A-0030760-T9-12_640	AAAGATAGCTTGTTTGCTAGAGGATGGTTTGGTAGTG
1A-0030760-T9-12_704	AAACATTAGATCATGTATTTTTCCATTGCTCTTTGCCTA
1A-0030760-T9-12_768	TGTGCTCTCGGGATAGATATGAATGTAGGATATGTACA
1A-0030760-T9-12_832	TTCCTAGAAAAGCTAGTGGGGAATATAGCAGTAGGA
1A-0030760-T9-12_896	AATCTAGAAATAAACCATGTTTCGAGAATATTTATCCC
1A-0030760-T9-12_960	CTGACTATTGGTTAGAATTCTGAAAATTATTATAGTAA
1A-0030760-T9-12_1024	AGGGGCAAGGCTTCTGCTGCAAGTGGCAGCTGAGA
1A-0030760-T9-12_1088	ATGGTCCTTCCCATTGCTGGTTAAGTATGATTGGACTT
1A-0030760-T9-12_1408	GTTGGATGTGTGCTCATGGTATTTTTATCCATTTGATG
1A-0030760-T9-12_1472	CTTTGTCAGAAGAAAACTTCGAACGATTTGCTTTATA
1A-0030760-T9-12_1536	AAATATAAACAAAAATGATCACACGGAAAAATTATAG
1A-0030760-T9-12_1580	AATAGATAATAGTAGTAAATACCACAATGAATAGAATA
1B-0120460-T9-12_0	CCTCCTTCCCTTGCTTCTCTCTACGCTACGCACGCACG
1B-0120460-T9-12_64	GTAAACACACGCAAGCAATGGCTGCAGGGAACAGA
1B-0120460-T9-12_448	CACACGCCACCCACATTTATAACGACCGCTCTAGCATA
1B-0120460-T9-12_512	CTTAAAAAAATATTCTAAAGCAGAATGTAAACCGTGATT
1B-0120460-T9-12_576	TTTACATTGAACAGGTACAGGTAGTGTTTGAAAAAAT
1B-0120460-T9-12_640	ATTGCTTTTGCATCAAATGCATTCCATCTTAGGACGAT
1B-0120460-T9-12_704	GAAATCTAGACAAAATATAAACGAAAATAATCATGCG
1B-0120460-T9-12_768	AATAGACAATAGTAAGTAAATACAATAAATAGGATATG
1B-0120460-T9-12_832	GTTTTGCCTTGATTCATGTGTTTTATATTTTGGCACATT

										1B-0120460-T9-12_896	tctctatctttcatacgcttatctctatctttatatatatTGGTAACTT
										1B-0120460-T9-12_960	ATATAATATGTTTAAGGTGCATGTAGATTGTCCTTATAA
										1B-0120460-T9-12_1024	TTGAAATTTAAGGTCTTTCTTTGGGTTACTTTCAGGC
										1B-0120460-T9-12_1152	CTGCTCGGCCGGCGGGTGATAGAGATACGACTAGCG
										1B-0120460-T9-12_1216	GAGGGCTAACAAGGCGGATCGCAAGGAAGAAAAGA
										1B-0120460-T9-12_1280	CACTTTGTGATCGACTGCACTACTGAGCTGTGTGATA
										1B-0120460-T9-12_1344	ACGAATGTCTGCTTCTCTCGGCTCCTAAACCTGTTGTG
										1B-0120460-T9-12_1408	AATTGTGTTATTTTTAGACTCCGCATGCGGCTATAGAG
										1B-0120460-T9-12_1472	TGGTTTGGTTAAGGTCACACAAGGGTCACTAACGAA
										1B-0120460-T9-12_1536	TTCTGCTGGGCATCGGTCAGAGTGGAGGATCAGGTA
										1B-0120460-T9-12_1580	GGAATTTCTTGGGAGGGAGGATCTTGATCGGCTCCT
										1D-0188170-T9-12_64	CTGCAGGGAACAGAGGAACACACGCACCGGCCGGT
										1D-0188170-T9-12_384	CTCGCTAGGTGTCGAGTGAGGAGGGTGGGAGGCGA
										1D-0188170-T9-12_448	GCTCTAGCATACGACTAATTTCTAGGAATACAATATTC
										1D-0188170-T9-12_512	ATAAACAACGACTATCATGAGTATACAAACATTAGAGA
										1D-0188170-T9-12_576	GTACAGGTAATGTGTGAAATTTTATAGAACTTGATTAT
										1D-0188170-T9-12_640	GCATCAATTGTATTCCATCTTGTTGGGACGATTTATGT
										1D-0188170-T9-12_704	TAGAGAAAATATAAACAACAATGACTATGCGGGGAAA
										1D-0188170-T9-12_768	caatagtaaataaatacaataaataGGATATACACCATGGAA
										1D-0188170-T9-12_832	ATTCATGTTTTTTATATATTGGCACATTTtatttctactatga
										1D-0188170-T9-12_896	taccccaatctctatctatatctctatcatttatatACTGGCAACT
										1D-0188170-T9-12_960	TATAATATGTTGAAGGCTGTACAGGCAGATTGTCGTTA
										1D-0188170-T9-12_1024	CATTGGCTTATGTTCAGGCATAGACGCATAGTATACTC
										1D-0188170-T9-12_1088	ATGGTCTGGTAGTGATATAAGTGCATATTCTGTGTAAC
										1D-0188170-T9-12_1152	TCTTTGGCTAGTTATGGTTGAGGTGCGGTCAAGTGTG
										1D-0188170-T9-12_1216	ATGTACATGACTTTTGTGGTTGGAGATTTGGTTTTCCT
										1D-0188170-T9-12_1280	AGGAGTTGTTGCGGTGTTGTGAGCTCTATGGAAATCT
										1D-0188170-T9-12_1344	TATCCTTGCGATCCTTCAAGCAATTACACTATCTAATTA
										1D-0188170-T9-12_1408	CAGAAACGAGAACAATGAGGGATGCAAGTCAGAGG
										1D-0188170-T9-12_1472	AGATCTTCAGTGAGCATGGAGGCTGGGCTCCAATGG
										1D-0188170-T9-12_1536	CCACTTGTTCTCAAGATGAAAACTTAGAATCTAGCCTT
										1D-0188170-T9-12_1580	TTTGGTCCGATGACGATGGCGCTTGAGCGTTGCTCCT
										5A-1228910-T9-13_0	ATGCTTGATTAGTACCCGCTGCAATTTTTTACTGCCCT
										5A-1228910-T9-13_64	ACACTTGTCTCCTAGTAGCAGCAATCCAGCCACACTT
										5A-1228910-T9-13_128	TGCTCATGCAATTTGTGCTTGATTCACTTTCGGTGGG
										5A-1228910-T9-13_192	TTATCCAATGTAGATGCAGCAATGAAAGCAACGTGTC
										5A-1228910-T9-13_256	GTCGATTCAGTCGAGGAAAATTCCCGGCAATTTCAAG
										5A-1228910-T9-13_320	GCCCTGTCCTGCGGCTTTCTTTTTCTGTTGTTTGCATC
										5A-1228910-T9-13_384	TCCTGGTTCATAAGTGGCACCTTAATAAAAGGTCTCTT
										5A-1228910-T9-13_576	GCCTCATGTTACATGGCAACAGCAATTTGCACAAAAC
										5A-1228910-T9-13_768	GTTTTGTACTGGAAGTGGGTATGTTATTCATTTACCTG
										5A-1228910-T9-13_832	GCTTAAACATGTTGACTCTGTATTTTTCTTGGTTTTTG
										5A-1228910-T9-13_1024	ATGAGAGACGTGTTGATGTGGTTGGTTCTAGAAATTA
										5A-1228910-T9-13_1216	TACTGCGACAAGGCGATGTACATCTCCTAGTCTTGGC
										5A-1228910-T9-13_1344	AGGTGTCAACACCCTCCACCCCAACATCAAGGTCCAT
										5A-1228910-T9-13_1536	TGGTCTCTAGCCCATCTTTAGATCATATCAAGGGACAG
										5B-1339030-T9-13_128	GCCTGCCTGTGTCTGGTCGGTTTAGGAGCTGGGGGT
										5B-1339030-T9-13_320	GTGCGATCGAGGGTTGGTTCAATCTGTCAATGGAGAT
										5B-1339030-T9-13_512	TGTGTTCCCCGCTGCGGATTCTGTGGGCGTCAATCCT
										5B-1339030-T9-13_576	TTCACTTGGTCCTCGTTCTAGCACAGCTCCCAAGTCT
										5B-1339030-T9-13_640	TTAGTACCCGCTGCAATTTTTTACTGCTGTTAATTAATC
										5B-1339030-T9-13_704	TCTCCTAGTAGCAGCAATCCAGCCACACTTACCTCCTA
										5B-1339030-T9-13_768	GCAATTTGTTCTTGATTCACTTTTGGTGGGTGAATTCT
										5B-1339030-T9-13_832	ATGTAGAGCCAGCAATGAAAACAACTTGTCATACTTC
										5B-1339030-T9-13_896	TCGAGGAAAATTCCCGGCAATTTCAAGTGAATCCGAT
										5B-1339030-T9-13_960	GCGGCTTTCTTTTTCTGTCGTTTGCATCGAAGATTACA
										5B-1339030-T9-13_1024	TAAGTGGCACCTTAATAAAAGGTCTCTTGTTTCCAATG
										5B-1339030-T9-13_1216	ACATGGCAACAGCAATTTGCACAAAACATCTTCCATT
										5B-1339030-T9-13_1408	GGAAGTGGGTATGTTATTCAGTTACCTGCAGTTTCAG
										5B-1339030-T9-13_1472	GTTGACACTGTATTTTTCTTGGTTTTTGTGTGTCTGGA
										5D-1426330-T9-13_128	TCCGGAGCCGGGGGTCTAGGGTTCGTTGCTGTTGTG
										5D-1426330-T9-13_192	CGGTTTTGTGGTGTGCCGCTGTTCAATTCGAGGAGG
										5D-1426330-T9-13_256	CCAGGTGTTTGTTTGTTTGTTTGTTATTGTGGGAGAG
										5D-1426330-T9-13_320	TGGGTTCAATCTGTCAATGGAGACGACGGACGATGT
										5D-1426330-T9-13_512	GCGGATTCCGTGGGCGTCAGTCCTGAGAAATACTATG
										5D-1426330-T9-13_576	CGTTCTAGCACGGCTGGGGAAATTAATTTGTTATTAA
										5D-1426330-T9-13_704	AGGTGACTTGACACATCTTGTCTCCTAGTACTAGCAG
										5D-1426330-T9-13_768	TTTGTGCTTGATTCACTTTTGGTGGGTGAACTCTTCG
										5D-1426330-T9-13_832	AGAGCCAACAATGAAAGCAACGTGTCATACTTCTTAC
										5D-1426330-T9-13_896	CGAGGAAAATTCCCGGCAATTTCAAGTGAATCAGATT
										5D-1426330-T9-13_960	CGGCTCTGTTTTTCTGTCGTTTGTATCGAAGATTACAA
										5D-1426330-T9-13_1024	AAGTGGCACCTTAATAAAAGGTCTCTTGTTTCCAATG
										5D-1426330-T9-13_1152	CCCTCAGAATCTCCGCAAATAGTTTTGTTTATTGGCTT
										5D-1426330-T9-13_1216	CATGGCAACAGCAATTTGCACAAAACATCTTCTATTTT
										5D-1426330-T9-13_1408	GAAGCGGGTATGTTATTCGGTTACCTGCAGTTTCAGG
										5D-1426330-T9-13_1472	GTTGGCGCTGTATTTTTCTTGGTTTTTGTGTGTCTGGA
										4A-0993610-T9-14_189	AGTAGGATGACAAAGATGTGGTTCAGCAACTTGTGCT
										4A-0993610-T9-14_252	GTGTTCATCAACCTTATGTCCTCACCTATTGGGTAGCC
										4A-0993610-T9-14_315	TGAGGATAGTTCAACTAGTGCGGATTTTACCTGCAAT
										4A-0993610-T9-14_378	TTGAGATGCATAAGTTGAAGCCAGAGATGACAACTT
										4A-0993610-T9-14_441	AAGTTCTGTCACGGACTTTTGCCCCTCGCTTGTCCTTG
										4A-0993610-T9-14_504	GTTCAATGCATCAACAAAGTCCGGTGGTTCGCTAAAA
										4A-0993610-T9-14_567	AATTATTTCTGAAGTGATCATTTTTGCTGCTAAAATTT
										4A-0993610-T9-14_630	CGTAGAGAAAAATGGATGGCAACATATGGCGTCCTG
										4A-0993610-T9-14_693	AACAGTTAATATTATAGTACCATAGATCCCCTATCACTA
										4A-0993610-T9-14_882	GCTGCTGCTCTCTCTACTGATATTCTTGTCGATCTTAGG
										4A-0993610-T9-14_945	ATTTTTGTCATATAAACATTAGACACTGACACAAGGAA
										4A-0993610-T9-14_1008	TTAATTATTAGTTTTCCGTGTGAACAAAATAAAATACAT
										4A-0993610-T9-14_1071	ACAACATTCACCTTAAAGCCTAGAAAGTACTCCTAACT
										4A-0993610-T9-14_1134	CTAAAGTGGACATCATATTTGTGCGACGAAATTAAAC
										4A-0993610-T9-14_1323	TTTAGAGATCTTTGCTACTAGTGCATTTTGTATCATACA
										4A-0993610-T9-14_1386	CTATTCGCTGAACAGCCGCTGTGTGTGTAGTGTACCA
										4A-0993610-T9-14_1575	CTTTATGATTCATTCGATCTCGCCAGCTGCTTGCTTGC
										4B-1097390-T9-14_0	AGATGCATAAGTTGAAGCCAGAGATGACAACTTCAAA
										4B-1097390-T9-14_64	TTTTGTCACGGACTTTTGCCGCTCGCTTGTCTTGGTTT
										4B-1097390-T9-14_128	AATGCATCAACAAAGTCCGGTGGTTTGCTAAAACCAT
										4B-1097390-T9-14_192	TTTCTGAAGTCGTCATTTTGCTGCTAAAAATTCTCAAA
										4B-1097390-T9-14_704	CACCAAAGTGCTCCTAATCCGCTTCCTACCATAGATCC
										4B-1097390-T9-14_896	CCACACTTGTGCCAACTAATTAGTTGCGCTGCTGCTCT
										4B-1097390-T9-14_960	GCTTGCATATGAAGGTGTAGTGGATTTTTTGTCATATA
										4B-1097390-T9-14_1024	CTAATCTCTTAATTTTGCCAACTTAATTAATTAATTTTCT
										4B-1097390-T9-14_1088	CCGGGTTAATTGCCAAGCTATTACATCATTCACCTTAA
										4B-1097390-T9-14_1152	GTTACGAACTAATCAAATTAACTAAAGTGGACATCATA
										4B-1097390-T9-14_1344	ATCACAGTCGGACCCATGTTTAGAGATCTTTGCTAGTA
										4B-1097390-T9-14_1408	CCATCTCTCTCTCTTACTATTCGCTGAACAGCCGCTGT
										4B-1097390-T9-14_1580	AGCGTTTTCTTTATGATTCATTCGATCTCGCCAGCTGC
										7A-1781320-T9-15_192	CGACTGCAGCAGCGAGTATTAACTgaggaggaggcaagg
										7A-1781320-T9-15_384	CTGGGCTGGCCCGACTGCCAGACCAGACCTCACCTG
										7A-1781320-T9-15_448	GCGAGAGGCAAGGCGGCGTGATCCGCGGACCTGCG
										7A-1781320-T9-15_704	GCCCCACGCCCACGGCCAGGCGGATGCGATGCGGA
										7A-1781320-T9-15_768	TGACTGTCCGTCACACCACCCACACACGGATCCACAT
										7A-1781320-T9-15_832	TCAAAACTTTTTTTTTGCAATCTTTTTTTTTTTGCAATC
										7A-1781320-T9-15_896	GAACCGTCGCTTGCCCGGCCCGGCCCGGCCCGGCCT
										7A-1781320-T9-15_960	AAAAAAAATCCTGAACGCCGCTAGCCCGCTACGTACC
										7A-1781320-T9-15_1024	CCGGCCAGACCATGGAGCACGACCGAGCCATGGCAA
										7A-1781320-T9-15_1088	CTACTCCACTAGCCGCTGTAGTAGGAGTACTGCGTCCT
										7A-1781320-T9-15_1152	TCGGTTGCTTCATTCAATCGCTGCTGGTGATGGAAGG
										7A-1781320-T9-15_1216	GGGAGGGATGGATGGTGACAGTTTACACTGGCCCCG
										7A-1781320-T9-15_1280	TGTGGGTGCGTGCTGCTTGCCCAGAAAGCAGCACCA
										7A-1781320-T9-15_1344	GTCCCATGAATCAACAATACTCCTACTACAGCGCAATG
										7A-1781320-T9-15_1580	CCTAGGAGCTTGTGCAAAGGTTGTTGGTTAAACTAAA
										7B-1869170-T9-15_0	GGCTTCCCGCTCCGCCGGGGACTCCGTTGTTCTTGTC
										7B-1869170-T9-15_192	CCGGCGCGACTGCGACTGCAGCAGCGAGTATTAACT
										7B-1869170-T9-15_640	GATAAAGTGTGGTAATGCTAATCAAACAGCCCCCGAA
										7B-1869170-T9-15_768	CCGCGTCTCTTCTCTTCTCCGCGCCAGCCGAGGCTAG
										7B-1869170-T9-15_1024	CCCACGCCCACAGAGGTGCCAAGCGGATGCAATGCG
										7B-1869170-T9-15_1088	GTCGCTCTGACTGTCTGTCACCACCCCAACACGGATC
										7B-1869170-T9-15_1216	CCACCGCCGTTTGCTGCTGGCGGTGACGGACACACG
										7B-1869170-T9-15_1280	GCTGAGCATGCCCGAGCCATGGCAATGAATACCGGT

7B-1869170-T9-15_1472	AATGCGACCGACTGGAGGGATGGATGGTGACAGTTT
7B-1869170-T9-15_1536	ACAACATGTGGGGCTGGGGCTGGGGCTGTGGGTGC
7D-2008610-T9-15_0	GGCTTCCCGCTCCGCCGGTGACTCCGTTCTTCTTGGC
7D-2008610-T9-15_63	CCGCTGATGACTCCACCGGAACGACTTGGTCGTTAAT
7D-2008610-T9-15_378	CGACAACAGGAGCGCGCGAGCGAGCGAGCCACCTC
7D-2008610-T9-15_567	CCGAGGctacactactactacgtctactggtactacCAGGCCA
7D-2008610-T9-15_819	CACGCCGTCCCACGCCCACGGCCAAGGGGATGCGAT
7D-2008610-T9-15_882	GTCGCTCTGACTGTCACCACCCCAACACAGATCCACA
7D-2008610-T9-15_945	TGAACCCTTCCTTGCCCTCGCCCAACACAACAAAATG
7D-2008610-T9-15_1008	CCACCGCCGTTTGCTGCTGCCGGTGCTGAGCATGCCC
7D-2008610-T9-15_1071	CGGCCAAATACACCTACACCTACACCTACTATGTGCCC
7D-2008610-T9-15_1260	GTTTACACTGACCCCGCAGTAAAGACAACATGTGGgg
7D-2008610-T9-15_1449	GACAGCGTTCTGGGCCTGGGCTTCAACAAGGATAAT
5A-1193610-T9-16_0	TGGGCATTAGCGGGCGCGAGACACTTGACTAATGTA
5A-1193610-T9-16_64	CGTTGTGGGCGTATGCCTAAACCTTCTCTCTTGATTAA
5A-1193610-T9-16_128	CCCTTGGTTTTGGTCTTGTCCTCCCAGTTTGCTCTTTG
5A-1193610-T9-16_192	TGTAATTTTAGTTTAGTACTCGACTGATGCGGCTTTTG
5A-1193610-T9-16_256	GTATCCTATTTCATTCAAATAATGGAGCAGGCCGGCGT
5A-1193610-T9-16_320	AAAAGTGCATACAACATGTAATCATAGATACTTACACG
5A-1193610-T9-16_384	CCTACGACCAATTATTCCAAAAGAAAATCAAACCATAT
5A-1193610-T9-16_576	CGCCTTGCTAGCTGCTAAAATTTTCAGAAGAAAAACC
5A-1193610-T9-16_640	CCAAGCCTTGCAAGCTCATTAGCAGAAATATCATTCG
5A-1193610-T9-16_768	AGTTGTAACACAAATTAAAACTCGCTTTGAAGATTTC
5A-1193610-T9-16_832	TGGACAGGATAAATCAAAAGGTAACCATACAGGTCAT
5A-1193610-T9-16_1280	AAATAGTCCCTTGGATCCAAGCACCCTAAAAGAGACT
5A-1193610-T9-16_1344	GAATGTTCTCAGTATCTTACCAATCATTTCCCATTTTTC
5A-1193610-T9-16_1408	CTACCACTAGTGTTTTCTCTTGAGACTTCTGACATTTT
5A-1193610-T9-16_1472	TTGCACCTAGCACCAGCACTAGCAGCGCCTGAGCTGT
5B-1365700-T9-16_512	ACTGTCATTTCCTTTGAACTCCGTATCCTGTTTCAATC
5B-1365700-T9-16_576	GGCGGTCTCCCGTGACTCTAAAAAAAAGTGCATACA
5B-1365700-T9-16_832	CAATGCTGAATATCATGCATAACGCGCCTTGCTAGCTG
5B-1365700-T9-16_896	AAGCGGACGAATTGTGGAGCTCCACGATTGTCTAATT
5B-1365700-T9-16_960	ATTTAGCACCCCAAGTCTTGCAAGCTCATTAGCAGAT
5B-1365700-T9-16_1024	CGGATTATTGATAGTGTATCACTTTAGAGAGGCTCAA
5B-1365700-T9-16_1088	GGCCGGCGAGCTATATGACAGTTGTAACACAAATTAA
5B-1365700-T9-16_1152	AACGCATTTTTTTTCTTTTTTCTTGGACAGGATAAATC
5B-1365700-T9-16_1344	ATCTTACCAATCATTTCCCATTTTCCTCTTCGAGATGGT
5B-1365700-T9-16_1408	GTGTTCTTTCTTGAGACTTTTGATATTTTGCTGACCCA
5B-1365700-T9-16_1472	GCACTAGCAGCCTTAGCTGTGCAAGGAACAATTTTGA
5B-1365700-T9-16_1536	ATAAGCACGTACGTACACATCGTCAGCCACTTGCGTA
5D-1454260-T9-16_1024	CCTGGTGATTACTTTATTTCTTCAACTCACGGTTTTGT
5D-1454260-T9-16_1216	TTTCATCAGATCTAAGTGAACTTGTTCGACAGATTACG
5D-1454260-T9-16_1344	GTCACAAGAATGTTCTCAGTATCTTACCAATCATTTCC
5D-1454260-T9-16_1408	TTGTATACTACTACTAGTGTTCTTTCTTGAGGCTTCTTA
5D-1454260-T9-16_1472	CCCCAGCTTGCACCTGGCACTAGCACTAGCAGCCTGA
5A-1271440-T9-17A_192	GTGTAGCACAAGGAATTCTGAAGATGGGCTTGACTT
5A-1271430-T9-17B_192	GAAAAGGCATGTGGTAGATGTTGATGTATACAACGAG
5A-1271440-T9-17A_256	TTTTCCAAAATAAATAATTTGCTCATTGTTGACCGCTT
5A-1271430-T9-17B_384	GCATGTAGGGCTTTTGTAACTGGAGTGTGTGTTACAG
5A-1271430-T9-17B_512	ATGTCTGCAATCCGTATCCGTGCTGAAAATTTGTGTGC
5A-1271430-T9-17B_576	GCCCGTGGTGAATATTGGAAAATTGTGTGCCACGAAA
5A-1271430-T9-17B_640	ATGTTCTAATTAAGAATTATGTGTCGAACATTATGGCAT
5A-1271430-T9-17B_704	ACTGATTCTCATGCAATCGTTGTTTCTCCTTAAACGCC
5A-1271430-T9-17B_768	CTCTTGGTAAAATGATTCTTTTGATCTCCTTTATTGATG
5A-1271440-T9-17A_832	TTATCTGTTTGAGGTCGCGGGTTGGAACGTGTTATTC
5A-1271430-T9-17B_832	GCTAAGTGCCTCTCAAAACTAAGTAAACATGAACTAT
5A-1271430-T9-17B_896	TTTTCTGAGAAATAACCCTCTCAACCTTCATACACTGG
5A-1271430-T9-17B_960	TCTACTGAAGACAAATATTTATAGCAAAATATTTAAACT
5A-1271430-T9-17B_1024	AAATACAAACGCAAAATCTAGTCTAATAATAAGTTTAC
5A-1271430-T9-17B_1152	CAGACCAAAGCTCCAAGCACGAATGAAAATCGAGAG
5A-1271430-T9-17B_1216	ACAACCAACACATCTCGTTGACACACTACCGTCATGC
5A-1271440-T9-17A_1280	TTCCTTGTCTCGTTTGACCCGGTTACGTTCTGGCCCCG
5A-1271430-T9-17B_1280	TGTGCGCGTGCCGTGTGTTTGTTTTCAGGATCTCAATA
5A-1271440-T9-17A_1344	ACGTTGGGAAATTAATGGTGAATCCAAGGTGATCAAT
5A-1271440-T9-17A_1408	AATTCGAACGAGAATTTTCCTATATGCTGTGATTTTTCT
5A-1271430-T9-17B_1408	GTAGTGTAACCAATAGAGCATGCGAAGGAAATGAAG
5A-1271440-T9-17A_1472	GGACTAGTATAAAACAGGAGCGAAAAAACCCCATCA
5A-1271440-T9-17A_1536	TGTGTAAGATACAGTAGCGCCACATTTCAAATTTTCAA
5A-1271430-T9-17B_1580	AGCCAGTCGTCTCCCCATGAACAACTTGACCTCTTCTT
5D-1490950-T9-17_64	ACAAAATCTGCTCCTAATGCAAGAATCTTGCTGGAATA
5D-1490950-T9-17_128	GAAATGTGGCGCTACTGTATCTTATCTTACACAATAATT
5D-1490950-T9-17_192	AATAAAGATTCTGATGGTTTTGGGGGGGCACGAGA
5D-1490950-T9-17_256	TCCTGTTTTATACTATAGTATAATATATAAGGGGATAAA
5D-1490950-T9-17_832	GCGGAACCCTGCGAATGTGATGATCGGTCGGGGGTG
5D-1490950-T9-17_896	CTCACGCCGTACGCCACTTTTTATTACTACTCATAAATT
5D-1490950-T9-17_960	ATACCATGAAAGTCATACCTTCGCTGAAACTTTTTATG
5D-1490950-T9-17_1024	TTCCAAAACAAATTCAAACTTTTTCTCCTTTTACTTTT
5D-1490950-T9-17_1088	ATCAGGTGCAtatgtctatatttatacctatatttatacttaAACC
5D-1490950-T9-17_1152	TCTGTCCGCACATGATTATTTTTGGTCCGACAAGCCG
5D-1490950-T9-17_1344	CCACTCTTCCGCCTGCAACCAACTCATCTAGCGAGGC
5D-1490950-T9-17_1408	GAACCCAAAACCTGTCGACTAGTACACCTACTCGAGC
5D-1490950-T9-17_1472	TTAATTAAGATAATACATAGTCTCATCTCGCTCTCTCAC
5D-1490950-T9-17_1536	TTTTTGTGTTTCTTGAGTGTCAGGAAGGCATCTAGGA
5D-1490950-T9-17_1580	ATAGAAAACAAAGGAGAAAAGGGAGGCTAATTAACT
6A-1517200-T9-18_64	CTTTATTTTTTTAAGTAGAGCAACAAAGCACATCCAA
6A-1517200-T9-18_128	GGTGAGCATCAATTTATGACTAGTCATACCGGTGTAA
6A-1517200-T9-18_192	TTAAAAGATGGATGTTAATAGCTTTCTTGACACCGATG
6A-1517200-T9-18_256	TTATTTTATTTTTTGCTTCTTGACATCGATGCACTGCTT
6A-1517200-T9-18_320	CACTTGAAGGAACGACTAGCAAAGGTTCTACGTGTT
6A-1517200-T9-18_384	ATATCAAGGCATTAGTACATTCTAGGAAATGTAGAAAA
6A-1517200-T9-18_1580	CAGAGCTCGATCGAGGTGCAACACTCTGCTAGATTAC
6B-1587290-T9-18_0	GGAAAATCTGATGCAATTAATTTGCTTACATATGCCAC
6B-1587290-T9-18_64	ATGAATTTATATTTTAGGGGCGAAAATAGCAAAGCGT
6B-1587290-T9-18_128	TCTGGATCGTTAATGGAAGATCCTATGTTCTAATGCTC
6B-1587290-T9-18_192	TGACTGCTAGATCAACGGTGGGGCGAGCTACTTAGG
6B-1587290-T9-18_256	TTTAGATAGGTTTACATTTCTATGGTGTTGTTTTCCCTT
6B-1587290-T9-18_320	ACTTTGCTCGCTGCATTGCAGATGTAATAATTCCATTG
6B-1587290-T9-18_384	ACACCTGTAGAGCTGACCATCAGAGACCGCGCTAGG
6B-1587290-T9-18_448	TAACTTCTTGTTGGCACTTTATATTTTGTGGGGTTTATG
6B-1587290-T9-18_512	GTATACATTTTCGATTAAATACTAGGTTAGCATCTATTT
6B-1587290-T9-18_576	GTAAGAAAAATATCAATAAAAGGCCAAAAAAATTAAA
6B-1587290-T9-18_640	AAAAAGAAAGATGAATGCTAATAGCCTCGTGACACCG
6B-1587290-T9-18_704	CTTGAAGGAACGAACGATTAGAAAAGGTTCTACGTGT
6B-1587290-T9-18_768	GGATATCAAGGCATTGTGGTACCATTGTAGGAAATGT
6B-1587290-T9-18_832	CAAGCCCGCAATAAAGAGAGGGCAAGGCAAATCAG
6B-1587290-T9-18_1024	CTCTTCAATTTCAGGTGGATCCGCACAACCTCCCTTCC
6B-1587290-T9-18_1152	CACAGCTACAGCAAGGATCGGCAGCTCGATCACCTAT
6B-1587290-T9-18_1340	AGCACACAACTCGCCGCCGCTTGCCAACCACTTGTGG
6D-1692430-T9-18_0	TTTGGTAACCGGGTGAGATGATTAAATACAACTTCGA
6D-1692430-T9-18_64	CTCACTTTGGCTATGGAGGAACAACATCTTTTTTCATA
6D-1692430-T9-18_128	TTTTATATTCATGTAAATTTTAGCTCCGTACTTGGTGTA
6D-1692430-T9-18_192	CCGTCAAAGACCTGTTTACAAGGAATGTGGGGTCAG
6D-1692430-T9-18_256	TGTTCTCTTCTAGACAGTTTGCTTTCCTCTTACTTGTTG
6D-1692430-T9-18_320	TTGTCTTTTTGGTTTTGTAGTACTCCCTTCGGTCCTTTT
6D-1692430-T9-18_576	ggagtaAGTTGGTTGTATACATTTTAGATGATAGGCTAG
6D-1692430-T9-18_768	ATGAATGTAATATTATAGACTAGTACCATATGCATGATA
6D-1692430-T9-18_832	AGGAACATACTAATGTTGATACTCCCTAATAACTTCTTG
6D-1692430-T9-18_896	CTCCTGCATTTTATTTTAATTTTTTGCTTCTTGACATCG
6D-1692430-T9-18_960	GCATGGCCGCAAATAAAGAGAAGGCAAGGCAACTCG
6D-1692430-T9-18_1216	CTTCAGTTCTGTTGGATCCGCACTGCGCACACAATCCC
6D-1692430-T9-18_1580	CTCGATCGAGGTGCAACACTCTGCTAGATTACATCACA
4A-1016270-T9-19_0	ATCTCCCCGCTTTCGCTTTCCCTGTTGCATATCGCTCCG
4A-1016270-T9-19_124	CACGCCGGGCCGCctcgctcctcgctcgctcctcgGGGCGTG
4A-1016270-T9-19_186	CCGCCTgcaagcaagcaggcaagctagcttgcaagGTCACCA
4A-1016270-T9-19_248	CGCCACGCCCTCCTCCGCCTTTCTCCCGCTCCCACGCT
4A-1016270-T9-19_310	GCGGCGAGCAGTTCTTGGGGCCGGCTCCGTCCGTCT
4A-1016270-T9-19_372	CCGTCCTCGCACTGTTCCTCTTTCTCCGGTCATAGCCC
4A-1016270-T9-19_558	TGCAATTCCTGTGGGCTCAACGGGTTTTTAATCAATC

4A-1016270-T9-19_744	CCCCGTTGCCTCCCCATTTGCACTAGAGAAAGATGCG
4A-1016270-T9-19_806	AATCTCGGCTAACTCTGTTTTGCCGTTGTTTGTGCAGA
4A-1016270-T9-19_868	ATGTGGACCGGTTGTTTATGCTAGGCGCGGGCGTTG
4A-1016270-T9-19_930	TTTCTCTCCTTCTCGGTTTACCGGTGCTGTGGCGGGG
4A-1016270-T9-19_1116	ACTCCCCCTGAGGCGCTTGTGCTCCTCGCCGCCCGCA
4A-1016270-T9-19_1302	TACCCAAGTTGCTTCTGTTCCTTGTACTTGCTGTTTCTT
4A-1016270-T9-19_1364	TGATGTTCCTTCCCCGGACAAGTAGTATGATGGGTTA
4A-1016270-T9-19_1424	TCTTCCTCCTCCTGCGGTGGTGGTCTGCTCTGCTCTGC
4B-1034980-T9-19_0	CTTGTAAGAAACAGAGGTTCAAAGCACGAACAAGCC
4B-1034980-T9-19_64	AGACCACCACCGCaggaggaggaggaggaggaagaagaAG
4B-1034980-T9-19_128	ACTACTTGTCCACGGGCGGAAGGAACAGCAACGGCT
4B-1034980-T9-19_448	AAGCTGACACAGCCCCACCTCCTCCTCCCCTGCCGCT
4B-1034980-T9-19_512	TCTTGGTGGATGAGATCTTCCGCAATAAATTTCCTTTC
4B-1034980-T9-19_576	AAAGAAAGAAAGAATCACGCAGTATCTTGTGTCCAA
4B-1034980-T9-19_640	TTCCTCCTCCCAGACCCTACAAATCTCTGCAGAAACA
4B-1034980-T9-19_832	GCGGGGAGGAATCTTGTAACAAGATCTCGCAGCCTT
4B-1034980-T9-19_1024	GGGAGGCGTCGAGATTTATAGCGAGCCACGGGGATG
4B-1034980-T9-19_1152	AAGAGGAACAGTGCGACGGGCGGAGGCAGACCCA
4B-1034980-T9-19_1216	CCACCCCAGCAGCGGACGGAGCCAGCCCCAAGAACT
4B-1034980-T9-19_1408	TTGGGAGCGGGAGAAAGGCGTGGCGATTAACCTCG
4B-1034980-T9-19_1580	CCACAGCAGAGAGACAGCACTACCACCACCCCTCTCT
4D-1135060-T9-19_0	CTTGACAGAAACAGAGGTTCAAAGGACGAACAAGT
4D-1135060-T9-19_64	AGCCTCTCACTCCCCCAGGCCAATGCGCTGACCACCA
4D-1135060-T9-19_128	gaagaagaagaCTTGTGTGCTATGCTAACCCATCATACTT
4D-1135060-T9-19_192	TCCAGGACCAGACGGCAAAGAAACAGCAAGTACAA
4D-1135060-T9-19_256	CGGTGGGGTGGTGGGCGGCGGAAGGGGGGGAGGG
4D-1135060-T9-19_320	GCGCTCGTACAACGGAAACAATagcagcagcagcagcag
4D-1135060-T9-19_512	CCCACCTCCTCCTCCTCCCCTGCCGCTATCTATTTCTTA
4D-1135060-T9-19_640	GAGAAAGAATCACGCAGGCAGGATCTCGTGTCCAAA
4D-1135060-T9-19_704	ACATTCCTCCTCCCAGACCCTACAAATCTCTGCAGAAA
4D-1135060-T9-19_896	GGAGGAATCTTGTGACAAGATCTCGCAGCCTTGTGC
4D-1135060-T9-19_1088	GTCGAGATTTATAGCGAGGCGAGCCACGGGGATGGC
4D-1135060-T9-19_1216	TGACCGGAGAAAGAGGAACAGTGCGACGGGCGGA
4D-1135060-T9-19_1408	GCGCGCGCGCATGTGCCCGTGTCTCCCTGTCGACCCG
4D-1135060-T9-19_1472	CCgcgtgcgtgccggtgcgcgtgcgtgcgtgcttgtgtTGGGAGT
2A-0000960-T9-20_0	GGTTAGCATCACACCGTGGGCCTGTTGGGCCCCTTTG
2A-0000960-T9-20_64	CTAGCCACCCCTGGTACACAGGACCCCATCACTCTCT
2A-0000960-T9-20_320	ttcaccgagcttcttgcctacatgtatgtaattgggtcggctgtcctaac
2A-0000960-T9-20_896	AGTTGACTTATCACGCAAGCATACATTGTAACGAACT
2A-0000960-T9-20_960	ATAGTTTTCTACATAAAATAGCTGGCGTGGTTTTAGAT
2A-0000960-T9-20_1024	CTATTTCCGAATCGGATCGGAAGGAGTACAATTGAGA
2A-0000960-T9-20_1088	ACCCATGCATCCTTTTGCATGTACATTGTCCCAACCGA
2A-0000960-T9-20_1152	GTTGGTTGGCAAAAGCCAACAGAAAGCCCTAACAAC
2A-0000960-T9-20_1280	GCTGCCCCCCGGCCAGTGGTAGATTAAACTCGGGAG
2A-0000960-T9-20_1536	CACCTCACCGCATAGCCTGGCCTAGCTAACTAACAATT
2B-0469480-T9-20_0	TTTGAGATGGGTTTAGATAAGCTAAAATTATAGAATAT
2B-0469480-T9-20_64	GGGTAGCTCAAAGCAAGAACTTGCAAGCTTCTGTTA
2B-0469480-T9-20_128	ATAGTTAATTATAAAGTTTAAGCTATGTGGTACTTCCC
2B-0469480-T9-20_320	AATTTACGGGCAATTTGGCACGAAATACGAAGGAGA
2B-0469480-T9-20_384	TAACTATGAGGTGCATGTTCATATGGATGCACAACACA
2B-0469480-T9-20_448	TTAGGGTTCATGTAGATTCTTGTTTTAGGAACCGATTA
2B-0469480-T9-20_512	TTTTTGGTGTTAGATTTTGTGAAAGGGTTCTTGTAGTT
2B-0469480-T9-20_576	GCCCTCCACCACCCACCCGCATTGGTCAAGTCGTTGT
2B-0469480-T9-20_640	GAGCACTGCCATCGCTGCCTCCGGCTTGGTCTCGGA
2B-0469480-T9-20_704	TAGAAATATCGACTTGGGCCTCCTTGGTTTGTATAGTA
2B-0469480-T9-20_832	TTGGTTTCCTATGTATCCTTCACATTTTAAAAGGAAAA
2B-0469480-T9-20_896	AAACAGAATTCCTATCATATGAATCAAATGACATATCGT
2B-0469480-T9-20_960	CCTTTCCTATTACCATCTCAAAAAAAAACTTTCCTACG
2B-0469480-T9-20_1024	CAACAGCTATTGCGATCGAGATTGCCCATGTGGAATG
2B-0469480-T9-20_1088	CTTCTGCATGTGCACATTGTCACAACGGAAACGTGAC
2B-0469480-T9-20_1152	TTCGTTAGCAGAAGCCAACAGAAAGCCCTACAACAC
2B-0469480-T9-20_1280	CCCCCCGGCCAGTGGTAGATTAAACTCGAGAGAAGA
2B-0469480-T9-20_1536	cacaccgcaTAGCCTAGCTAACTAACAATTCGCATAGGC
2D-0567160-T9-20_64	CGGCGAGGGCGACTACGTAGGGATCAGCTGGCCAG
2D-0567160-T9-20_448	TTTGCCTTTGCTTTTTCTCAATCTCGATCTTACGGCCTA
2D-0567160-T9-20_512	GTTAGGGCTTTCTGTTGGCTTTTGCCAACGAACCCTG
2D-0567160-T9-20_640	AATTGTACTCCTTCCGATCGGAAATAGTTGGCGTGGT
2D-0567160-T9-20_768	AATATATACAAGAGATGTCTTGCTTAACAAGCCAACTA
2D-0567160-T9-20_832	CTATATCAGAGATCGTAGGAAGATCAATTGGAAATAGT
2D-0567160-T9-20_960	CTGTCACCTCATACTCGTAGTATACCTACCCACTTCCG
6A-1555510-T9-21_0	TACTTGGCCATATCGCCATGAAAAAGTTTCAGCTTTTT
6A-1555510-T9-21_64	TAGAAGTCCATATAGAGTCCGTCTAGAACCATGAACC
6A-1555510-T9-21_128	TTATACGGATATTAAACAAGTTTCTGTGAAAGTTTGCA
6A-1555510-T9-21_192	GGGCCAGGTTTTTTTTTTGAGGGGTAAGAGCCGGGG
6A-1555510-T9-21_256	CACTGTTGAGTGAATTTAAATTAAGTCCATATAGATGC
6A-1555510-T9-21_448	CGGGACGGAGAAAGCCCACTAGCTGACCACGACCG
6A-1555510-T9-21_576	TTCCGTCATCTCCTTATCAACAGATCGAGCGGCACAA
6A-1555510-T9-21_640	ACATTGACGTTTGTTCGTGGACCAATTTGTCTGATATT
6A-1555510-T9-21_704	CTGTCTGCGGTAGAAACTAAATTCAGCAAATGGTAAA
6A-1555510-T9-21_768	CTGACATCATATTTTTAAACATGGCAAATCTTGACGTC
6A-1555510-T9-21_832	GCATGATAATTTACTTCAGTGGTACTAGAAGTGTACCT
6A-1555510-T9-21_896	CTTGCATGAATTACACGGGGTGCGGTGGAGAAGAAA
6A-1555510-T9-21_1088	AAGCTGCAGCAAGAAGAACAGCACCGAGAACAAGA
6B-1664750-T9-21_0	CGAGTAGAATACTTGCCATATCGCTGCGGAAAAAGTT
6B-1664750-T9-21_64	AAATAAAAGTTTTAGTCCATAGCGTGCGTCTAGAACC
6B-1664750-T9-21_128	TGTATATTTATACGGATATCAAACAAGTTTCTGTGGAA
6B-1664750-T9-21_192	AAGAACCGGGGCCAGCAGAGGTCCAGCACTCCAGT
6B-1664750-T9-21_384	GTCAAAAAGGCAGTATCACGGGGGGATCTACACGGG
6B-1664750-T9-21_448	CTGAAAAATACATGTATACACTACTACTAGGAGAAGTT
6B-1664750-T9-21_512	GTTTCGATATTCCATACTACCTGAGCATTCCGTCATCTC
6B-1664750-T9-21_576	AGGGTTTTTTCAATCGTACAGCAGCCATGGAAAACA
6B-1664750-T9-21_640	TCTGATACTCGTGAAACTTCAAAGTGAAATCTGTCTG
6B-1664750-T9-21_704	AAAAGATAAATAtaaaattaatataaagttaagtcatttattttga
6B-1664750-T9-21_768	CACTACAAATCTGACATCATATTTTCAAACACGGCAAA
6B-1664750-T9-21_832	CTGCATGATAATTTACTTCAGTGTTAGAAGTGTACCTT
6B-1664750-T9-21_896	TGCATGAATTACACGGGGGCGGTGGAGAAGAAATGT
6B-1664750-T9-21_1088	GCTGCAGCAAGAAGAACGGCACCGAGAAGAAGAAG
6D-1725840-T9-21_64	ACTATATATCTGCTTGTGAAATATGCAAAGTGTTTATTT
6D-1725840-T9-21_192	GACAAAAAACAAACAAATACGCCGTCCGTTTGGGTC
6D-1725840-T9-21_256	AGTGGCTCGAAGAGTACTACTCCAGTACACAGTTGA
6D-1725840-T9-21_448	GCAGTATCACGGGGGATCTACACGGGACGGAGAAA
6D-1725840-T9-21_576	ATATTCCATACTACCTGAGCATTCCGTCATCTCCTTATC
6D-1725840-T9-21_640	ACATTGACGTTTGTTCGTCGACCAATTTGTCTGATATT
6D-1725840-T9-21_704	TGCTGTAGAAAGTAAATAAATTCAGCTAAGGGTAAAA
6D-1725840-T9-21_768	ATCATATTTTTAGACATGACAAATTTAACGTCCGAGAT
6D-1725840-T9-21_832	TAAAAACACTCTTATATTATGGGACAGAGGGAGTAGG
6D-1725840-T9-21_896	CTGAATACTTGCATGAATTACACGGGGTGCGGTGGA
6D-1725840-T9-21_960	GCATGCACGTTGCACAGAGAAAAAGTTGGTTTTTAA
6D-1725840-T9-21_1088	AAGGCGCAGAAGCTGCAGCAAGAAGAACAGCACCG
4A-0942540-T9-22_64	AATTGGTGAACTCCATTAAATATAGCCATGACTATGCA
4A-0942540-T9-22_128	CAGATGCTGAGAATCAACAGAACTTCTACCAAGAAAA
4A-0942540-T9-22_192	TGAACTAATAATCACAGAGTGAATGGCTTCAATATGCC
4A-0942540-T9-22_256	TAGAGGATGCACATGTGAATTCTCATAGTAGTGGGAA
4A-0942540-T9-22_320	ATATTTGATTTGTTTAATCAAGGACAAGGGCATCTGAT
4A-0942540-T9-22_384	TGAAAAAGGTAAACTTAATTGGCCAGAATGGTAAAGT
4A-0942540-T9-22_448	CTATTAAGTTGAACATTAGTAACTACCACTGTGGAGA
4A-0942540-T9-22_512	GCTTTCCTTTTTCCTTTGTCTTAGAATTGATCAAATTGA
4A-0942540-T9-22_576	CAGTTGCACTAAGTAAAATCTAGTGATGCTTTAGCACT
4A-0942540-T9-22_1088	ATCAGCACAGCAATTTAAGGTGATGCTGCTGAACTAG
4A-0942540-T9-22_1152	TGGCGATGACATTTTGGTTGATTTCTTAGTTCCCCATG
4A-0942540-T9-22_1344	AGTTGGTCATGATGATAAAAACTTTTAGCTCGCTCCA
4A-0942540-T9-22_1408	CTCAGATAGAGCACTTGCAGTAGAGTAGGAGTAGTTT
4A-0942540-T9-22_1472	CATGGAAGAGCATAAAAAGTTGGTCATAATGACGAA
4A-0942540-T9-22_1580	GTACCATACTAACATTGGTGATTGATGCCTGATGCCAA
4B-1096070-T9-22_0	TTGAACCAAAGATCTTCGTGAAAGAGGTACCAATGAA
4B-1096070-T9-22_64	CATAAGTCATAAGTGCATGATTGAGTGCAACGTCAGG
4B-1096070-T9-22_128	CATGAACAATGTTTCCTCTTTCCAATTTTCACACTTTCA
4B-1096070-T9-22_192	TAGGATTTAAATATTTATGTATCTATAATAACCATTTGGT
4B-1096070-T9-22_256	TATGGGCCATTTTCCAGTGTCCTAGTTTGAATCGATTT

										4B-1096070-T9-22_320	TTCAAAGGAATTCGATGGGAATTTTGGGATTTGAATC
										4B-1096070-T9-22_384	CCATTGAAATTTTTACTAACAATTTGTCTGCACTTAATT
										4B-1096070-T9-22_448	GATCTCTTGTGTGATATCAAACACACTGTTCCAAATTC
										4B-1096070-T9-22_512	CAAAGGGCATGCCGCCTCAATAGTATGTCTTCCTATCC
										4B-1096070-T9-22_576	ATTAAGCACCTTAAGTTTCTTTAAACTCATTTTGCATA
										4B-1096070-T9-22_640	ATAAGAAGACTCCTGGGCTCATTCAGTTTGCAAGATT
										4B-1096070-T9-22_704	GTTGAGATTTTTCTCCTATGTACGACATCCATTTATAGA
										4B-1096070-T9-22_768	CAGTGTCCATATTGACCTAATTCATAAGGATTCTTAGC
										4B-1096070-T9-22_832	CTAAGGATTAGAGCGGAGAAGACATCCCAGCCCTTT
										4B-1096070-T9-22_896	TTTTATAAACCGAATCAGCCAAAATAATTATACAAAGT
										4B-1096070-T9-22_960	CTACTAATATCTGCAAAATATTTAGATTAGTATGACTGA
										4B-1096070-T9-22_1024	CTCCTGCTACTTTATAATCTACAAAAAAAATGTCAAAG
										4B-1096070-T9-22_1280	CGATTGTTCCACACTTGTACATTTTCCCTGGGGTTCTC
										4B-1096070-T9-22_1408	AGAGGAGTACATCAGACCGGGAAATAAATATGAGAC
										4D-1166110-T9-22_832	ATTACCCTACCCAAACGGACAGAATCCGGGCAAAAC
										4D-1166110-T9-22_896	GTGGTCGTGCGGTGGAGTTGGCCTTATTTTTCATGTT
										4D-1166110-T9-22_960	TCAGCCCAAAAAATTCTACAAAGTGCCTACGTAATATT
										4D-1166110-T9-22_1024	AATATTTAGATTAGTAGGATTGAAAATAATTATTGGATT
										4D-1166110-T9-22_1152	AAATGCTTTTATATTAGTTTACAGAGGGAGTACAAAA
										4D-1166110-T9-22_1216	AATGTTCCACACTTGTACCCTATCTTGTAACTTATGTAT
										4D-1166110-T9-22_1280	GTAACCTATTTTACGGTGCATGTTCCACACTTGTACATT
										4D-1166110-T9-22_1344	GGCGTTCCTTTGTCCCAGGAACCCTCTCGGGTTCACT
										4D-1166110-T9-22_1408	AGGAGTACATCAGACCGAGAAATAAATAAATGAGACA
										4A-0971050-T9-24_64	ATCAATCCTTCCCATTTATAAAGGCTACTCTAGCACTAG
										4A-0971050-T9-24_128	TTGGTTTGATGAATAATTAATCAGGCACGGTCTCCCTA
										4A-0971050-T9-24_192	ATCCAAAAGCCTGGTTCCCAGTTCTATACCAATTTGAC
										4A-0971050-T9-24_256	ACAAAGGAGGTAACAATAGCCCCAACCCATTTGTTTG
										4A-0971050-T9-24_320	TGTTGGATGAGTTTATGATGAGTTGTTCAATTTTTTCC
										4A-0971050-T9-24_384	TAGTACCACCCTTACAGGGAACAAAATGTCATAACTT
										4A-0971050-T9-24_448	CAGTGAGCAAAAGGTTGAGCTTCGGGAAAGCTGGT
										4A-0971050-T9-24_512	GTTGTTCAACAATATATGTACTCATTCTCAAGCCCATGT
										4A-0971050-T9-24_576	GATTAACTTCACTTAATCCTATGTGTCAAATCTCTGATG
										4A-0971050-T9-24_640	CCTACTGCCTAGCACCTATATATACCACTATTAGCCGTA
										4A-0971050-T9-24_704	TGTTTTTCTGCTTGATAATTTAGTAATTGTAATGGAGG
										4A-0971050-T9-24_768	CTTGATTAGTTGATTTCATCATTTTTGTGTATATCAAGC
										4A-0971050-T9-24_832	TTGACCTTGTACTATTTCGTTTTTTGCATTATGCAAGTT
										4A-0971050-T9-24_896	GATCACCCCGAGTGCATCTTTGCTTCTAAAAAGAATT
										4A-0971050-T9-24_960	TTAGTCATCTTCCCAGGAGAAGAGAACATTATGTATGT
										4A-0971050-T9-24_1024	ATGTTCCCAGGCTGTTATGCATACTTGTTTGTCGCGTA
										4A-0971050-T9-24_1088	CGACACATAGAAGACATATGCAGCTGTGTTCAGTTTAT
										4A-0971050-T9-24_1152	AAAAGTAAAATTAGCATATAAGCACAAATTAAAGGTG
										4A-0971050-T9-24_1216	GTTAAAGCCCAGTCTGACATCCTACAAAGTAGCTAGA
										4A-0971050-T9-24_1280	TGTTGTTAATTTAATTTCTTTTGAGACGAAAAGATTAT
										4A-0971050-T9-24_1344	AGTGGAATGGGCCGCCATAATTGACACAGAACAGGG
										4A-0971050-T9-24_1408	TGAGATGGGCCGCTATTTATTGGTAGGTGCGTGCTCG
										4A-0971050-T9-24_1580	CACTGCTGGGGATTTtcttcctcctcctcctGACAACTC
										4B-1102010-T9-24_0	CGATCCTCCCCATTTATAAAGGCTACTCTAGCACTAGC
										4B-1102010-T9-24_64	GGTTTGTTGAATAATTAATCTGACATGGTCTCCCTAAT
										4B-1102010-T9-24_128	AGCTTGGTTCCCAGTTCTATACCAATTTGACATTCTTT
										4B-1102010-T9-24_192	GTAACAATAGCCCAACCCATTTGTGTTTGCTTGAGAG
										4B-1102010-T9-24_256	GAGTTTATGAATTGTTTGACTTTTCCTTTCTTTTTAACA
										4B-1102010-T9-24_320	AATCAATCAATAAATTGAAAGGCAGTGAGCAAAAGG
										4B-1102010-T9-24_384	ACCAAAGAAAAACAGGGTAACTTTTCCTGTGTTTTTC
										4B-1102010-T9-24_448	AGGTCATTTAGCTAGGGGGCTGTCTGAGTGTTGATTT
										4B-1102010-T9-24_512	GCCTTTCCTTCTTTGTCATTATTGATTTGTTGGTGAAC
										4B-1102010-T9-24_576	CTGCATCTGTTTCTGCCACACAAAAAAACCGTAGGTG
										4B-1102010-T9-24_704	TGCAAAACGATCTTATATTATGGGATGGAGGGAGTAG
										4B-1102010-T9-24_768	TATATTTTGATATAGAGGAGTACTATTTAGTTGCTCACG
										4B-1102010-T9-24_832	TCTTCTTGAATAGTTATAACTTAAAATAGAACTTTAAAA
										4B-1102010-T9-24_896	TGCTTCTAAAAAGAATTAGTTAATGGAGAGTTAATATG
										4B-1102010-T9-24_960	TTCCCAGGCTGTTATGCATACTTGTTTGCCGCATAACC
										4B-1102010-T9-24_1024	AGAAGACATATGCAGCTGTGTTCAGTTTATTTCAAAA
										4B-1102010-T9-24_1088	TTAGCATATAACCACAAAGGTGTTGCTTTCCAGAGTAT
										4B-1102010-T9-24_1152	GTAAAGTAGAACTAATGATGATGCGATGTTCTTAATGT
										4B-1102010-T9-24_1216	TTGGACTGATCTGGTTGCTGTGAGGTAATATTATGTCA
										4B-1102010-T9-24_1280	TTTGCTAAAGAAGAATGTGATGTTTGATTACAGGACT
										4B-1102010-T9-24_1344	TTTTTGGGCACTGGACTGGAAGTTATTACAGTAATTG
										4B-1102010-T9-24_1408	CACATAAATGTGGGGAAGAAAACCTAGTGAAGTGGG
										4B-1102010-T9-24_1472	AAAGTGGAGGCAGCCTAACAGAGCTCAGCAGCGTGT
										4B-1102010-T9-24_1536	ATGGTTTGTGTTGCCTTTGTTAATAGTGTCATCCATGC
										4D-1176380-T9-24_0	CAACTAAGAGCTGCATCAGTAATCAATGAAACATATCT
										4D-1176380-T9-24_64	CAGCTGGGACTTCATTTCGCCTCATTCATTTAGTGCAA
										4D-1176380-T9-24_128	AGCATGGATGGCACCTATTAACAAAGGCACCAAAAAT
										4D-1176380-T9-24_192	CACGCTGCTGAGCTATGTAAGGTTGCCTCCACTTTTCT
										4D-1176380-T9-24_256	CACTCAGTTTTTCTTCCTTGCAGACGGCGTACGATGG
										4D-1176380-T9-24_320	TACAAACAATCGATCATCTCATTCTTCTTCAGGAAAAA
										4D-1176380-T9-24_384	GAAAATGTGAGAAAACCAATACTGCTACTTCTTTGAC
										4D-1176380-T9-24_448	ATCAGTCAAACCATAGAATCAAAAGTGGAAAATAAAT
										4D-1176380-T9-24_512	GTTCTACTTTAATTTGTAGGATGCCAGAGTGGGACTA
										4D-1176380-T9-24_576	TTTCCTTTCTATATATACTCTGGAAAGCAACACCTTTTG
										4D-1176380-T9-24_640	TATTATTAATTTTTGAAATCAACTGAACACAGCTGCAT
										4D-1176380-T9-24_704	GTGCAGTATGGTGGTTACGCGGCAAACAGGTATGCG
										4D-1176380-T9-24_768	TCAATAATACAAACTGTTTGTTTTCCTGAAAGATGACC
										4D-1176380-T9-24_832	ACTTGTCTATTAACTACTAATTCCTTTTAGAAGCAAAG
										4D-1176380-T9-24_896	AAAATACTATTTTTTTTAAGTTATAATAACTATTTGAGA
										4D-1176380-T9-24_960	GCAAAAGGTAAGCAACTAAATAGTACTCTACATGGTC
										4D-1176380-T9-24_1024	TATATGATAGATTTTTTTTCCCTGTTGCAAACAGTAAC
										4D-1176380-T9-24_1088	TAGCACAGATGCAGGGAAAATACATCAAAAGAGGCA
										4D-1176380-T9-24_1152	ACCAAGAAAGAAAGGCGCACGGTTGATATACGTATAT
										4D-1176380-T9-24_1216	CATTCTACCAGCTTTCCCCAAGCTCAACCTTTTGCTCA
										4D-1176380-T9-24_1280	AAGTTACGGCATTTTGTCACCCGTAAGGGCAGTACTA
										4D-1176380-T9-24_1344	AAAAAAGGAAAAGTTGGACAGCTCATAAACTCATCC
										4D-1176380-T9-24_1408	AAGCAAACACAAATGGGGTGGGCCATTGTTACCTCC
										4D-1176380-T9-24_1472	TGTCAAATTGGTATATAATTGGGAACCAAGCTTTTGG
										4D-1176380-T9-24_1536	GAGACATGTCAGATTATAATTATTCAACAAACCCAAG
										4D-1176380-T9-24_1580	CAGCACGTACCCCACCGCTAGTGCTAGAGTAGCCTCT
										5A-1186910-T9-25_64	GGCACGGGCCGTATTTATAGCCGGGTGGGGCGGCAA
										5A-1186910-T9-25_256	GGGGTCACGAGCGCTCCTCACTATGCCTCCTCTCATA
										5A-1186910-T9-25_320	CACTATAACTAAATAACTATGGTTGGTACCACACGCGC
										5A-1186910-T9-25_512	AGATATGGATGAGACATGTTTTACCATGCAAAAGAGA
										5A-1186910-T9-25_576	ACGTATGTACGTGTCAGTCACGTATTATATGATGGGTA
										5A-1186910-T9-25_640	GCATTCAATCCATGCAGACAGACACACCGTACGCTGT
										5A-1186910-T9-25_704	GCTTATTTCTTGCATGTGCATATTCCGCTACTTAGTTAG
										5A-1186910-T9-25_768	TATGGCCCCTTAAACATCTACACGCCCGCGTGTGTGTG
										4B-1070490-T9-25_128	TGGCGCGCCAACCCCTGCTGGCTGCTTGACTGTCGTG
										4B-1070490-T9-25_192	AGATTTTCCCCTGATGCTTTTCACTTGTACCATGTTCTA
										4B-1070490-T9-25_256	CTGTGTATCAGTGTATAAAGTACTCTGAACTAAAACTT
										4B-1070490-T9-25_640	GATTGATTTGATACTCTTTGATATCACATATTGTTCTTT
										4B-1070490-T9-25_832	TTGGGCCCGGGACTTGCTCCAATCCTGACTCCGCCCG
										4B-1070490-T9-25_896	AGGTAATAGTATTGTTTAGTGGAGGACTAGGCTCGCT
										4B-1070490-T9-25_960	GTTTTGGAAGGAGAAAATAAAAGAGGAGCCAGAAA
										4B-1070490-T9-25_1152	TGCGTATGATACGTGACACACAACACACGTACGTGGA
										4B-1070490-T9-25_1216	CGGGACGGGACGTGCCATAGTTAGTAGTAGTAAATAG
										4B-1070490-T9-25_1408	GAAGCCAACGATGGATGGGTTGGAATGTAGGAGAG
										4B-1070490-T9-25_1580	TCTGCGGGCGCTTCCTTCCGTTACCGGCTATAAATACG
										4D-1114210-T9-25_64	CTGGGCGCTCGCCGTATTTATAGCCGGGCGGGACGG
										4D-1114210-T9-25_256	CTATGCCTCCTCTCCTACATTCCCATCCATCCATGATCC
										4D-1114210-T9-25_320	AGTAGGAGTAATACAAAACGTACACTATATACTGTATT
										4D-1114210-T9-25_384	CTACCACGTTGCTGCATGCGCTACGCTCGCTCCACGC
										4D-1114210-T9-25_448	GGATGCTGCCACACCGACAACCGGCCGTAGCATCCAT
										6A-1526230-T9-26_0	CGATTGTTCCTAGCGCCGACGGAGCTTCTCGGTCGAG
										6A-1526230-T9-26_64	TCGTTCGCCGGCCGGTACCGGTGCAGGTTGAGGTTG
										6A-1526230-T9-26_192	AGTAGGGCAGCGTTTGTCCGGTTTGAGGGGAGCAA
										6A-1526230-T9-26_384	AGACAAGAATTTTAGAACGGAAGGAGTACTAATTTAC
										6A-1526230-T9-26_448	ATGTTCATGGCGCTGCTCAACTGGACGGGCATGTGGA
										6A-1526230-T9-26_512	GAAATGAAAATGCTTCTCATCCCATCTTTGTTTGTTTG
										6A-1526230-T9-26_576	GGGAGAGGGTTGCAGGAGAGGCTGAAACGTACGTA
										6A-1526230-T9-26_640	GTGGCCGGTCAACATCGCATCTGAAAAATGAATTGAG

										6A-1526230-T9-26_832	GCATACAAATACTGCTACGTACAAACTGATAGTGACA
										6A-1526230-T9-26_896	TTTTTTTTCTTCCAATCAGTGTATAGCACTTGCAGTAA
										6A-1526230-T9-26_1472	CGAACCCGTGCCGAGGTGGAGATGCTTCTTTTTAA
										6A-1526230-T9-26_1536	TATAAATGAAATATCGAGGGCGAGACAGTGAAAAATA
										6B-1604630-T9-26_1152	CAGTGGCTGATTGATCCCTTTGTACGTACGTTTCAGC
										6B-1604630-T9-26_1216	TGTACCGGAATGTGCGTGACAAACAAACAAAGACGG
										6B-1604630-T9-26_1280	CCATGCACGCACGCGTTGCGTCTTTACACTCCATCGT
										6B-1604630-T9-26_1344	CAGCTAGCGCCATGCACATTTGCATTTTTTAAGTGGG
										6B-1604630-T9-26_1472	CCGGACCAACGCTGCCCTACTTGTCTCTCCTCTCCAC
										6D-1700030-T9-26_0	CGATTGTTCCTAGCGCCGACGTAGCTTGTCGATCGAG
										6D-1700030-T9-26_64	gctagctgctaCTTGGTTCGCCGGCCGGTACCGGTGCAA
										6D-1700030-T9-26_256	CAAACGTGTAAGTGCTGTGTTGAATACAAGGGCCCG
										6D-1700030-T9-26_320	TTACAACTAATTAATAAGCACTAAAAAAATGCGAATGT
										6D-1700030-T9-26_448	CATCTCGTCCCATCTTTGTTTGTTTGTCACGCACATTC
										6D-1700030-T9-26_512	CTGAAACGTACGTACAAAGGGATCAATCAGCCAGGT
										6D-1700030-T9-26_704	GGATTTATCCCGgcgtgcgtgcgtgcgtgcgtgcATACAAATA
										6D-1700030-T9-26_768	CCGCTCCTCCCACTGTCCACAACATATTTTTTCTTCCA
										6D-1700030-T9-26_832	AATAGTGGCAGTGAGACTGATATTAACTGTGCAAACC
										6D-1700030-T9-26_896	AGCTGTGCCAAAGTGCTTTTACTAATGTAGTATAAATG
										6D-1700030-T9-26_1024	CGGTTTCAGTCTCAAGATTTTGAGGCCGACCGGCCG
										6D-1700030-T9-26_1216	ACACCTGCACACAAGCGTTGCACGCACGCACCACAC
										6D-1700030-T9-26_1280	GTGCTGCGATTCAGATGATATCACACGAATGGGCGCG
										6D-1700030-T9-26_1344	GAAAACTATAAACCGCACGCTCGCCGTTTGCATCATC
										6D-1700030-T9-26_1408	AAGTCCAATCAACGACTCAGGTGAAGTGAACGCCGT
										6D-1700030-T9-26_1472	GGCTACATGCACCGGGGAGCGGCGGAACCGCCGTTT
										4A-1019660-T9-27_64	GTTCAACCACCCACCGACCTTTTTTTTTAAAACTCCAT
										4A-1019660-T9-27_128	CATTTACAAGCATTGGTCCTTTTTCTAAATTTCCTGTTA
										4A-1019660-T9-27_192	AGCAGGTATATTTGTGGGGTTGATTGTAGCTGATAGG
										4A-1019660-T9-27_256	TTGCTCCATTTGGGTTAGAACCATAATTATGCGGAGA
										4A-1019660-T9-27_320	GTTGCTTCTGTCAGTGCTCACATCACAGCAGCTGCTG
										4A-1019660-T9-27_512	AACTCACTCTCAGTCGTCGTTCTTTTGCAATAAAGCC
										4A-1019660-T9-27_576	TAAGTCAGGGCACAAGCATAACAAACCATGATTACAT
										4A-1019660-T9-27_640	TCCATATGGCCTAAATAACTGCTGCAAAACCAATTTGT
										4A-1019660-T9-27_832	GATTCTGCCATAACACCAAATTCTCTTACCAGCTCTCT
										4A-1019660-T9-27_896	CTCCTAGTATGTGAGCAACCTTCACTGCTTGATTGGC
										4A-1019660-T9-27_1088	CCCCCTACAGAGTTATTTCCTTTTAAGGAGCTGTTGC
										4A-1019660-T9-27_1152	CAAATCCATTTATCCATCAAGCAAATGTTCATCACACA
										4A-1019660-T9-27_1216	TTGAAAATCCATTTTTTAGCAGGCTTATTTGATTCCAG
										4A-1019660-T9-27_1280	TGGGTTTTCTAGCTTCGCTTGTTTCCAGCATAAGTATG
										4A-1019660-T9-27_1408	ATTCCAGTCCTGGGTTTGCTAGCTTCGCTTACTTGTTT
										4A-1019660-T9-27_1472	GTTTCAATTTATTCATCCGGCTCTCTAAAATGATTTTTA
										4A-1019660-T9-27_1536	atatttctatttaacttatcgtatatttGTCTTATGCAGTTGATGT
										4A-1019660-T9-27_1580	GTCATCGTCGCACTGAACATTTATTGATAAATTAGTCA
										4B-1056990-T9-27_0	ATTTTCCAACCTTGTGGAGGTGATCTATTTGCCGGAAT
										4B-1056990-T9-27_64	ATCAACAGCAGTTAGTTTTGACTACTTTATCAATAATT
										4B-1056990-T9-27_128	CATAGGAGATAGGACaaatatacgataagttaaatagaaatat
										4B-1056990-T9-27_192	aactaaaaatCATTTGAGAGAGCCGGATGAACAAATTG
										4B-1056990-T9-27_320	GGACTGGAATAATCTCTCTGCTGGATCTTAAGTCGCC
										4B-1056990-T9-27_384	GTTAAAACACAGAAATAGCCATGCAGAATTGGAAAG
										4B-1056990-T9-27_512	CCCAGGACTGGGATAATCTCTCTGCTGGATCTTAAGT
										4B-1056990-T9-27_576	GGATGTTAAAACACAGAAATAGCCTAGCAGATTTGGA
										4B-1056990-T9-27_640	CTGAATTATTTGTTCACCTAGAGGTAAGTGTTCTGCAT
										4B-1056990-T9-27_704	GAAGGCAAAAGTTATATCCACTAAGACTAGGTCAAAT
										4B-1056990-T9-27_768	CCAAATCATATGGAACGAAAAACATGATTGTGGATGA
										4B-1056990-T9-27_832	GTGTGTACATGTAAACACTATACTTATACTTTTAATTTT
										4B-1056990-T9-27_896	GGTTTATCTACACCCAAATCATATGGAACGAAAAACA
										4B-1056990-T9-27_960	TAAATCCACAGGTGTGTGTACATGTAAACACTATACTT
										4B-1056990-T9-27_1024	cacccacccacccacccacATGTACTTCTAGAGAAAGCATA
										4B-1056990-T9-27_1088	GCAGCGCATACATCTCGTAACTGACACATGGAAAATA
										4B-1056990-T9-27_1152	AGAGAAAACAATGCATTAAGACTAAAGAAGTAAAGA
										4B-1056990-T9-27_1216	CTATAGTTATCTACAAGAATAATCCAGGACAACTTAAA
										4B-1056990-T9-27_1280	ATGCTAACAACTATTGCTCAAATGGCAAGTAAAAGGG
										4B-1056990-T9-27_1344	TAAAGTTAACCTATGGCATGCCTGCAATCCCACTAAGT
										4B-1056990-T9-27_1408	CAACATTCCTGGAGAATCATCGTTTCTCTACATGTATA
										4B-1056990-T9-27_1472	CTAGAACATATCATCGCATCGCATAATTAGATAATGTAT
										4B-1056990-T9-27_1580	AAAAATAGTTCACTGTAGCATCGCTAACGCCAACTAG
										4D-1151360-T9-27_0	ATTTTCCAACCTTGTGGATGTGATCTATTTGCTGGAAT
										4D-1151360-T9-27_64	ATTAACAGCAGTTAGTTTTGACTTCTTTATCAATAAAT
										4D-1151360-T9-27_128	CATAGGACAAATATACGATAAGTTAAAAGAGAAATATA
										4D-1151360-T9-27_320	GACTCGAACGGATCTTAATTTGGGATGTTAAAACACA
										4D-1151360-T9-27_384	TTTATGCATGGACTGCCCCTGTCTGAATCATCTGTTCA
										4D-1151360-T9-27_448	TGAAGCGCCACTTTTTCTAAAGGAAGGCAAAAGTTAT
										4D-1151360-T9-27_512	AAATTGTAGGGTTTATCTACACTGAAATTATATGGAAC
										4D-1151360-T9-27_576	ACATAAATGTAAATCCACATGTGTGTACATGTAAACAC
										4D-1151360-T9-27_640	TTCCCCCCATGTACTTCTAGAGAAAGCATATCGAACTG
										4D-1151360-T9-27_704	TATATCTCGTAACTGGCACATGGAAAATAATGTCAAAT
										4D-1151360-T9-27_1024	ataagactatcttatcaaccactgtacatgccATAAAGAAGTGA
										4D-1151360-T9-27_1088	TAGATATATTAATATATTATCTACAAGAGTAATTAGGAC
										4D-1151360-T9-27_1152	TAGACCATGCTAAGAACTATTGCTCAAATGGCAAGTA
										4D-1151360-T9-27_1216	AATAAAGTTAACCTATGGCATGCCTGCAATCCCACTAG
										4D-1151360-T9-27_1280	CTTTACATTCCTTGAGAATCATCGTTTCTCTGCATGTAT
										4D-1151360-T9-27_1344	AACTGGAACACATCATCGCGTCACATAACTAGATGAT
										4D-1151360-T9-27_1472	CATCGCTAACGCCAACTAGTTTATTTCCATACCTCAGA
										4D-1151360-T9-27_1536	TATACCGGATACAAACTGGAAACTCCTGCATGTTATGT
										4D-1151360-T9-27_1580	TACATGGAATCAATCACCATAATCAATATAGCATGTGA
										4A-0949990-T9-28_128	GCCTCTCCGGCCCGCTAAGTTCTGCCCCCCATTTTCT
										4A-0949990-T9-28_192	ATACACCACTCTGCCAATCCCAAGAGAAGGACAAACC
										4A-0949990-T9-28_384	AACCCGGGGATAATACATATACAAGCATTTCATGCATC
										4A-0949990-T9-28_448	TGTGTTATTTTCCGGTCCTTTCTTTTCCTCAGGAGACA
										4A-0949990-T9-28_512	CTTTTTCCTTGGGATCAGAGACAACTTTGATAAATTAT
										4A-0949990-T9-28_576	GCAAATTTATATTCGTGGGCATCAGCAGGCATGTATGT
										4A-0949990-T9-28_640	CAGTGGGCTAATATACCAATCTATCTATGCCACTTAAAT
										4A-0949990-T9-28_768	CAACAGAACTTCTGCGACCCTGCCCTGGCCTCTGCAT
										4A-0949990-T9-28_896	CCTATGCCAATACAGTAATGCACCTTCACTAGCTAGGT
										4B-1045830-T9-28_0	TGCTTATAAGGAGACGAAGAGGAAGGGGGAAAGAG
										4B-1045830-T9-28_128	GCTCTCGCCTCTTCCGCTAAGGTCTGCCCTCCCATTTT
										4B-1045830-T9-28_192	TCTGCCAATCCCAAGAGAAGGACAAAGCACAATGGG
										4B-1045830-T9-28_384	TAATACATATACAAGCATTTCATACATCACCTGGCCAG
										4B-1045830-T9-28_448	TCAGCTCCTTTCTTTTCCTCGGGAGACAATATGTGATG
										4B-1045830-T9-28_512	GGGATCAGAGACAACTTTGATAAATTAGCAGGGAAA
										4B-1045830-T9-28_576	ATTCGTGCACATCAGCAGGCATGTATGTCCAATCATAT
										4B-1045830-T9-28_640	ATATACCACTTAAATATGTTTTGGAAAGGAAGCCCCAA
										4B-1045830-T9-28_704	TCTAAACTAAGTGGAACCTGCAGGAAAGATTGGAAC
										4B-1045830-T9-28_896	TTCACTAGCTAGGTGGTGCTAACCCATGCTTATCACTA
										4B-1045830-T9-28_1024	CTTGAATCCCAACGTGTCCAGTACCTACAAAGATACA
										4B-1045830-T9-28_1088	ACATCGTCCGTCTCATAAACAAATGCAATTTGGGGGA
										4B-1045830-T9-28_1152	CGGCATTTGATTCCTCGGTGTGACAGTCATCGGATCC
										4B-1045830-T9-28_1216	GCATCTCCGGTGTGCATGTTGCCAAATTTCTCCTCTGT
										4B-1045830-T9-28_1280	TATAGCCGCTTGATAGCTATAACATGTTGCCGTGTCAT
										4D-1141810-T9-28_0	CAACCGACCGGTGATGATCGCTGGATCGCTCGGTGAT
										4D-1141810-T9-28_64	TGTCGCGGTGATCGCTGCTGCTGCTGAGGATGGTTG
										4D-1141810-T9-28_128	CTTATAAGGAGAGGAAGAGGAAGGGAGGCGGAGTG
										4D-1141810-T9-28_192	AAACAATTCCGGCGTGTCTCCCCGGTGGCCAACCGC
										4D-1141810-T9-28_256	GCTCTCCTCCTCCGCTAAGGCCTGCCCTCCCATTTTTT
										4D-1141810-T9-28_320	TCTGCCAATCCCAAGAGAAGGACAAAGCACAATGGG
										4D-1141810-T9-28_512	TAATACATATACAAACATTTCATACATCACCTGGCCAGA
										4D-1141810-T9-28_576	TCAGCTCCTTTCTTTTCCTCCGGAGACAATATGTGATG
										4D-1141810-T9-28_640	GGGATCAGAGACAACTTTGATAAATTAGCAAGGGAA
										4D-1141810-T9-28_704	TATTCGTGCACATCAGCAGGCATGTATGTCCAATCATA
										4D-1141810-T9-28_768	AATATACCAATCTATGCCACTTAACTATGTTTTGAAAAG
										4D-1141810-T9-28_1024	AGTAATGCACCTTCACTAGCTAGGTGGTGATAACCCAT
										4D-1141810-T9-28_1216	TGGACCAGCCAACATCATCCGTCTCACAAACAAATGC
										4D-1141810-T9-28_1280	ATATTCTGTGCAGCATTTGATTCCTCAGTGCATCAGAC
										4D-1141810-T9-28_1344	TGTTGCCAATTTCTCCTCTGTGCGTAAGCTTGGCCTCT
										4D-1141810-T9-28_1408	GAAAAAATCAGTTAACCAAACCAATACTAGACCTAGC
										4D-1141810-T9-28_1472	GCACCCTTGATCCTTTTCCCTCGCTCTAAACCTGTGTC
										5A-1252880-T9-29_189	ACGAGAGGGGAACAAGAGCGTGAGCACAACTGGTT
										5A-1252880-T9-29_252	TTTATGGTTCATGACTCGTCGGCGTCGTACTTCCTACT
										5A-1252880-T9-29_315	TGCCACTGGGCTCTCTGTTTTTGCTTTCTTTTATCCTTT

										5A-1252880-T9-29_378	TTGCTCCTGGCAATGCTTCCCTTCATCCACTGTTAATG
										5B-1324590-T9-29_128	GCTGTCGAGGCGAGTCGGGGTGGGGAGAGGAGAG
										5B-1324590-T9-29_192	GGAACAAGAGCGTGAGCACAACTGGTTAGGGGAAC
										5B-1324590-T9-29_256	CATGACTCGTCGTCGTCGGCGTCCTACTCCTACCGGC
										5B-1324590-T9-29_320	GCTATGTGTTTTCGCTTTCTTTTACTTTCCCTCCTTGTG
										5B-1324590-T9-29_384	CACTGCTAATGATAATGTCCCAACAGCATATGACAATG
										5B-1324590-T9-29_448	TAGTACCTGCATCCTCCGTTCTTGTTTATTAGTTGGGT
										5B-1324590-T9-29_512	AGTGACACAACATATAAAAGGAAGAAAAAAAAAGAA
										5B-1324590-T9-29_576	CATGTAAGATCTCACAAATATAGCTTAGGTAAGTCTTA
										5B-1324590-T9-29_640	GTTAGTTCTCTCATATTTTTGGTCAAATTTTCATCATAA
										5B-1324590-T9-29_704	TATATATTGTAAAAATAGGGACCGAATAACTGGAGAA
										5B-1324590-T9-29_768	GGGGTGACTCGGGAGGCGATCAAACCCTGGCCGTCT
										5B-1324590-T9-29_832	AGGTGGTTGTGCCGTCTTTTTTGGCCTTTTACTGGGC
										5B-1324590-T9-29_896	AAAACAGCCCAAAGAAAAAACACATGGCAAAAATGC
										5B-1324590-T9-29_960	GCATATAAATTTTGACATGATGGCAAAAAATAGTGTAA
										5B-1324590-T9-29_1024	ATACCTGTTGTGATGCACGACAAGAGATTTTGCCATG
										5B-1324590-T9-29_1088	CTTTGTGAAAGGCAGAAAAATCCCATGTCCATAAATG
										5B-1324590-T9-29_1152	TACAAGGTATGTTAAAAAATGCCATGCTAAGTCCAATA
										5B-1324590-T9-29_1216	ATTGCCATGGTGTAAAAAAAACTACCATGCTCTAAGTA
										5B-1324590-T9-29_1280	GAAAAATTCCATCATGAAATAATAATAATGCCATGTAC
										5B-1324590-T9-29_1344	ACACTAAAACTACCATAGTACTAGCAAGATAAAAAATG
										5B-1324590-T9-29_1408	GTCAAATTTTGTGGAAAAAAAATCTTTTGAAAAAAG
										5B-1324590-T9-29_1472	AAGCCATGTCGAATTTTTTTAAGAAGTTTACAAAAAA
										5B-1324590-T9-29_1536	GCTAAGTCCAATAAATTTTCGAAGTTCCATGATAATAA
										5B-1324590-T9-29_1580	CATGATTTAAAAATAATAAAAACGGCCATGCTCTCAAC
										5D-1428120-T9-29_0	ATTATTATTATTTTATGATGGCCTTTTCATTTATTCATGC
										5D-1428120-T9-29_64	ATGGCAGTTTTTTTATTTTTAAAGCATGGAAATTTTATT
										5D-1428120-T9-29_128	GACTTAGCATGTCATTTTTTTAATATACCATGGTAATTT
										5D-1428120-T9-29_192	ACATGGCATTTTTCTGTTTTTCCCAAAACACATTTTGC
										5D-1428120-T9-29_256	AAATTTGACATGTTTATATGACTATGCTGGAATGGCAC
										5D-1428120-T9-29_320	TTTTAGTGTATGTAGCATGCAATTTTTTATTATTTAGTA
										5D-1428120-T9-29_384	TGGAATTTTTCTTTTTTCCAGGGCATGACATTTTTATAT
										5D-1428120-T9-29_448	CATGGCAATTTTATTATTTGGAACATGGCAAATTTATT
										5D-1428120-T9-29_512	ACCTTGTAAATTCTTTTCTAAACACTTTTTTTTACATTC
										5D-1428120-T9-29_576	CGCAAAGTACAATTTTTTTCTAGTAATGCTTGGCAAAA
										5D-1428120-T9-29_640	TTTTTTTTGCATGCATCATGTCAATTTTTACACTATTTTT
										5D-1428120-T9-29_704	ATTTTTTGCCATGTGTTTTTTCTTTGGGCTGTTTTTTTA
										5D-1428120-T9-29_832	CCGAGTTATTGGGGGTCCTATTTTTACAATATATAATTT
										5D-1428120-T9-29_896	GATGAAAAATTCACCAAAAATACGAGGGAAGCAATA
										5D-1428120-T9-29_960	GACTTAACAAAGAAATAAATTTTCTTTCTTTTTTCTTTT
										5D-1428120-T9-29_1024	CTAGCCCCACCCAACTAATAAACCGGAAGGGAGGAT
										5D-1428120-T9-29_1088	TTCCCCTGCATTGTCATATGGTGTTGGGACATTATCATT
										5D-1428120-T9-29_1280	ACAAAAAAATCATAAAAGAGAATGAAAATCAGTTTAG
										5D-1428120-T9-29_1344	CGTGTCTTAGACAGAACCTAGAAAGAAAGCGAAAAC
										5D-1428120-T9-29_1408	CCTCTCACAGCAGGAAGTACGAGTCATGAACCATAAA
										5A-1212300-T9-30_0	ATTGAAACTCCTCGAAACGGGAATTTGCGGCGGTGA
										5A-1212300-T9-30_64	TTTCTTGATTTATGAAATTTATAGGCGAAAGAATGGA
										5A-1212300-T9-30_128	CAACCTAGTGGTGCGGGTCAGCCCCTGGTCCCGTCG
										5A-1212300-T9-30_384	TAATAGCATGAAACAATCAAAATTTATGTATACATTCGA
										5A-1212300-T9-30_448	AATCAAGTGCCCGATCAGGGGTAGTTTTGACTGATCT
										5A-1212300-T9-30_512	GACATTTTTGGAACCAAACATACAATTTTTGAGGATT
										5A-1212300-T9-30_640	ATCATGCGCGGACCCACATGCTTCGTCAATGCAACCC
										5A-1212300-T9-30_704	TCCTACTCTTATGTTTTATATGTTAAACTTTGTTTTGCAT
										5A-1212300-T9-30_768	AAATAATGAATGTAATGGTTATAAAACTGTATGCAGCT
										5A-1212300-T9-30_832	TGCACACTCTCCATCTCACTCACGATGACAAGCAAGA
										5A-1212300-T9-30_896	TGTTCATTCATGAAATCTTGTTCCATTTTTGCGGTTCAT
										5A-1212300-T9-30_960	ATTTAGTACTAAAAATGGTCATTTATATTGTCAAGAGC
										5A-1212300-T9-30_1024	AACATAGGCTATTGATATGTGGTAGAAAAACACCTTC
										5A-1212300-T9-30_1088	AACCAAACAACGATATATTGAAGGCGTGCTCACTGTC
										5A-1212300-T9-30_1152	TAAATATCGAGTCAAATCGCAATTCAAACTCTATCCCA
										5A-1212300-T9-30_1216	CAATCACATACATAAACTCGATCTACAATGGAATGGTA
										5A-1212300-T9-30_1280	TCAACACATATGATGTTCGTGCTAAACCTTCTTCTCGT
										5A-1212300-T9-30_1344	AAATCAATTCAGAAGACCTCTTTGATTCGACGGAATT
										5A-1212300-T9-30_1408	ACATGAAGAAATTTGAAAAAAAAAGAGGAATGGCCT
										5A-1212300-T9-30_1472	ATAAAGTTCAAAATTTCAAACCAAACAAAGGCAAAA
										5B-1348970-T9-30_0	AACAGGAATTTGCGGCAGCGGAAAAATTGGAGGAA
										5B-1348970-T9-30_124	GGTGTGGGTCAGCCTCTGGTCGTGTTGCCTGGTTGC
										5B-1348970-T9-30_186	ATGTCCCAACACTTTCTGGAATCTTCATGACGTGAAAT
										5B-1348970-T9-30_248	TTTGACCTTCGTAGATATGGATTTTCTGAAGAAAAAA
										5B-1348970-T9-30_372	ATAGCATGAAGCAATCAAAAATTATACATACATTCGAG
										5B-1348970-T9-30_434	TATACCTCTATACCATTGTATACTTTTGATTGTTTCATGC
										5B-1348970-T9-30_496	TAAAAAGTATTGTTTTTCAAGTAACATTGAAAATTTGT
										5B-1348970-T9-30_558	TTTGCAGCTCATTCTACATAAGTACAAGAAACCTGAA
										5B-1348970-T9-30_620	CATGAACTAAAAATGGACGTCCATGATGTCAAGAGAC
										5B-1348970-T9-30_682	AACCTAGGCTATCGCTATGTGGTGGAAAAACACCTTC
										5B-1348970-T9-30_744	TAACCAAACAACGATATATTGAAGGAGAGCTCGTTGT
										5B-1348970-T9-30_806	CGAGTCAAACAGTGATTCAAATCCTATCCCACAACAA
										5B-1348970-T9-30_868	ACATATATAACTCGATCTACAGTGGAATGGTAAAGGCT
										5B-1348970-T9-30_930	ACACATATGATGTTCGCGCTAAACCTTCTTCTCGTAGT
										5B-1348970-T9-30_992	TACTAGAATCAATTCCGAAGGCCTCTTTGATTCAACGT
										5B-1348970-T9-30_1054	ACCCCCCGAACATGAAGCAGAGAATATTTTATAAAGT
										5B-1348970-T9-30_1426	GGCGGGCGTGCGCTACGGCGGGGACGACGGCGGC
										5B-1348970-T9-30_1984	CGCCATCGTGAACGAGCAGGTGGAATTGCCTCTCAG
										5B-1348970-T9-30_2046	TATATTGCTTGCACACATGATCATGGAGACAATGCTGC
										5B-1348970-T9-30_2108	GTGATAGTTTAACTTGCAGTGCTCTTTTAGGTTTGTGT
										5B-1348970-T9-30_2170	GTGTGTGTGTATTTGTATGGAGGGTTAATTGGAGTCT
										5B-1348970-T9-30_2232	GCAATTTGACTGTGTGCACATGGAAACCGACTCCTTT
										5B-1348970-T9-30_2294	TACACTGTATCATGCTAGTTTGGAACAAAATGCATTCT
										5D-1443170-T9-30_0	CGACAAGGTGCCAAAACAGGTCTCCAGATTCGATCT
										5D-1443170-T9-30_64	AAAAAATGGAGAAAGAAGATACGAAGGGTTTCTTGA
										5D-1443170-T9-30_128	ACGAAAGACGGTGCACAGGGTGCCCACACAACCTAG
										5D-1443170-T9-30_448	TCGAGACGTATGTAGGGGTCATGCATATCAATCAAGT
										5D-1443170-T9-30_512	TCTTGAATGTTTTTTTCCGAGACTTGACTAAGACATTG
										5D-1443170-T9-30_704	ATTTCTCATGTGGATTCCTTCATCAAGTCCTACTCTTAT
										5D-1443170-T9-30_768	CATATTCATTCAAACAATAAACTAAGGAAATAATGAAC
										5D-1443170-T9-30_832	CTTAGAATACCCATAGGCGGGATTCTATAGGCACACT
										5D-1443170-T9-30_896	GAATTGTTTTTCAAGCAACATTGAAAATTTGTTCATTC
										5D-1443170-T9-30_960	TCATTCTACATAAGTACAAGAAACCTGAAACTTAGTAT
										5D-1443170-T9-30_1024	AATGGTCATCTATGATGTCAAGAGCCAATTGTCGGTAT
										5D-1443170-T9-30_1088	GATATGTGGTAGAAGAACACCTTCGCCGCAATGATTT
										5D-1443170-T9-30_1152	TATATTGAAGGCGTGCTCGCTGTCTTCTTCTGTCATAC
										5D-1443170-T9-30_1216	AAACCGCGATTCAAACCCTATCCCACGACAAACCACA
										5D-1443170-T9-30_1280	AACTCGATCTATAGTGGAATGGTAAAGGCTCCGGGGA
										5D-1443170-T9-30_1344	GTTCGTGATAAACCTTCTTCTGGGCGCAAATGTATGC
										5D-1443170-T9-30_1408	TTCAATATGAGGAAATTTCATAAAAAAAGGAATGGCC
										5D-1443170-T9-30_1472	TATAAAGTTCAAAAATTTCAAACCAAACAAAGGCAAA
										4A-0988850-T9-31_0	ACACGGCACAGGTGCCAAGGGAAAACTCGAGAAAT
										4A-0988850-T9-31_64	atgcatgcGGAGGGGCTTGTTATCCTCGGATTGAGATC
										4A-0988850-T9-31_128	TGTGAATGACACGTGGGGCTCGGTCCACATAAACGG
										4A-0988850-T9-31_192	TATCCTCACTGCTTGCACCATAGGTAAACATCGCAACT
										4A-0988850-T9-31_256	TCCATACCCGGCCCATCCAAACAACCAAGGTTATTTC
										4A-0988850-T9-31_320	GTCTGTAGCCTTTCAAAAAAAAAATTTACTCGACCCT
										4A-0988850-T9-31_384	AATGTAAGGTCAAGAAAAGGATCCGACCGCTTATGC
										4A-0988850-T9-31_448	TTTTTAGAGCCATGATTCGAGCCTTGGTCGATAGCCC
										4A-0988850-T9-31_512	TAGAGAGTGCATGTCTATTTTTCAACATTTTGCAACTA
										4A-0988850-T9-31_576	GTGTCAAGACTGTTTAATATCCAAGACAACAATGCCTA
										4A-0988850-T9-31_640	CGGCACATCGATTAATGCATAAAAATCTTATACTCCCT
										4A-0988850-T9-31_704	TTAGTACTTCCTCCATCCGGAAAAACTTGTCCGAGAA
										4A-0988850-T9-31_768	TTTAGCCAGATGGAGGACTTAAGATATTTAGTTAAATT
										4A-0988850-T9-31_832	TACATACAATAATTACTATTCAAAAAAAGTAAAGACAA
										4A-0988850-T9-31_896	AGCCACGTCTGTTTGAGCGAATTAATATGCATCAAGTT
										4A-0988850-T9-31_960	AGTAGGGTGGGTCATCGTCCTAGCACTCGATGACAAA
										4A-0988850-T9-31_1024	TAGTCGTATAGGCTGTTAAAGGTTTCTACAGGCTCGT
										4A-0988850-T9-31_1088	CCTCCCCCCGATTTCAATGGAGGTGGGTGGGCTCGTG
										4A-0988850-T9-31_1152	CCACAACACCTTGTAAACCCCTCGTCCCTGTAACATCT
										4A-0988850-T9-31_1344	ACCAACCAAATATTTCTCCTCCTCGAGCCCGCGGCCC
										4A-0988850-T9-31_1536	GACACCGACAGACGGACGTCCAGTTTAGCAGCAAGA
										4B-1089110-T9-31_0	CTCTCACCTAACGGATCAACCAACGGCGGGGCACTC
										4B-1089110-T9-31_192	AGTGGTAGGGAGTAGCAGAGGAGAGCGGACACTGC

										4B-1089110-T9-31_384	CCGCGATGGCCTCGGTCCGCACTAGTCTTTGCTTCCA
										4B-1089110-T9-31_448	TTGCTAGCCCTGTGCCCACATGCTCAAACTGTAGTCCT
										4B-1089110-T9-31_512	TGACCCACCCTACTTCACTGCGCTAAACGTGTATTTGT
										4B-1089110-T9-31_576	TTCGCTCAGAAAGATGTGGCCAAAACTTGAATTGGA
										4B-1089110-T9-31_640	TAAATAGTATAATTATTGTTTGTATTTCTGAGCCAAGGA
										4B-1089110-T9-31_768	ATTGTCTAAAATCTCTTCGAGGTCTTATAAAAATGAAC
										4B-1089110-T9-31_832	TACTAAAGTTATACTCCCTCCGTCTCGATTTCAGTTCTC
										4B-1089110-T9-31_896	CTACTAGGTTATTGACAATTAATACGATTGGAGGGAGT
										4B-1089110-T9-31_960	TACCGCTCATAATTTGCCTACATAGTCTACCTACGCATT
										4B-1089110-T9-31_1024	GACACCCAATTTTGTTCTAGAGTATTTCTTAGTTGCAA
										4B-1089110-T9-31_1088	CTAGCACGGTTGTAAAGACTTCGTGGAGTGGGGCTA
										4B-1089110-T9-31_1280	AGATACATCCGTTTTCGCGACAAGTAATTCCGAACGG
										4B-1089110-T9-31_1344	TGTCTCTACGCAGGGGTCGAGTTTTTTTTCTTTGAA
										4B-1089110-T9-31_1408	AGAAATTTGGGGAAATCTGTAGAAATAACCTTGGTTG
										4B-1089110-T9-31_1472	GAGCACCCTCGAATATGCCAGGGTTTGCGATGTTTAC
										4B-1089110-T9-31_1536	AGGGCGTATCATTTCTCGAGTTTTTGGCACTGCCGT
										4B-1089110-T9-31_1580	CTCTCAAATCGGTTTGATCAATTGACGCCGAATTACGA
										4D-1167910-T9-31_0	CTCGACGGATCAACCAACGGCAGGGCACTCGGTCGA
										4D-1167910-T9-31_64	GACAGGCGACCACCGTATCTGCCGCCGTACGCCGGA
										4D-1167910-T9-31_448	GCCCACCGCTTTGCTCGCCCTGTGCCGACGTGCCCAA
										4D-1167910-T9-31_512	GGACGATGACCCATTTTGTGCTTCCCAAAATTTCCAC
										4D-1167910-T9-31_832	gactttagtcaactctgatatgcaaagtaaatataaacggagggagt
										4D-1167910-T9-31_896	GCAGTTTGCCCATTGAAACAGGAAAATTATCTAAATT
										4D-1167910-T9-31_960	TTAGAAATTATTGATCAATGTGTCCCCCGTAGTTTGCC
										4D-1167910-T9-31_1024	CTCGGATATCGAACGGTCTTATATCCAATTTGATTACC
										4D-1167910-T9-31_1088	GAAAAATAGAAATGCACTGCCATAATCTATGGAATAA
										4D-1167910-T9-31_1152	ATGATTTTTCGTAGAATTTTTTTAAACTCTATAGTAAT
										4D-1167910-T9-31_1216	CATGGGACGGCCGAGCACCTTCGATTATGCAAAGATT
										6A-1522720-T9-32_0	TATGGGACGGGCGATTGATAGAACAGCCCGCGGGG
										6A-1522720-T9-32_192	GTGGCCAGAAAAATATCCTGGCCACATGAGAGAAGG
										6A-1522720-T9-32_256	TATATGAGAACGTAACAGTGGTGGTAGTTGTGTTTTT
										6A-1522720-T9-32_320	TTCCATGAAAATAAATTGTGTGTAGATCTACGGGTCAT
										6A-1522720-T9-32_512	TGCGTGGATTCATAGGGATGAgtgcatgtgcatgtgtatgag
										6A-1522720-T9-32_576	AAGAAATGACAAATAACAATAAGAAAAATGTTACTAC
										6A-1522720-T9-32_640	aagaagtggaaaaggaaTCAAAACGTAAGGTCAGCTAGG
										6A-1522720-T9-32_704	TTCATTTGTTTCAGAATACAACGCGTACATCTATTGAT
										6A-1522720-T9-32_896	TCCCTTCTTCCTCGATTGTCTGGTGTTTAGCTAACTTC
										6A-1522720-T9-32_1088	CCATTCATTGACTTTGATGAGGCTTCTACCTAGACCAG
										6A-1522720-T9-32_1152	GACGGTATGAAGTTACACTTCACTGTATATCCTTTGTA
										6A-1522720-T9-32_1216	GTTAGTCGTTGAACCCTTGAATCGATCCGAAGTGTGT
										6A-1522720-T9-32_1280	ACATGATGAGAAACCTAAACCTTGCCGTCCCAAGAAG
										6A-1522720-T9-32_1472	GCGGGCAGAGGTGAAGCCCGAAGGGGACGAGATCT
										6A-1522720-T9-32_1580	GAAGCACATGCAAACATCTCCAAGAACACACCTACCA
										6B-1602470-T9-32_1408	TGAGGCAAAAGCTTTTTTGCCCGTTTCAAAAAAAAA
										6B-1602470-T9-32_1472	CTTCTCTCATGTGGCCAGGATAATTTTCTGGCCACTCC
										6B-1602470-T9-32_1536	GTAGCTAGTGTTCTAGGTGCCTCTGAAAAAGGTATAC
										6D-1700490-T9-32_0	AACCTAGGACAAAAGCTTTGCCTCATCGCATTAGTTG
										6D-1700490-T9-32_64	ACAACCATTACATTTGTGTGACAAATATAAGTCTATATT
										6D-1700490-T9-32_128	TAGGTGTGTTCTTGGAGGTGTACACATGTGCTTCACC
										6D-1700490-T9-32_192	ATGTCTTCTTCTTCTCCTCGGCGGCACCTCCCGACTTT
										6D-1700490-T9-32_256	GTGGTGAAGAGGGGCGCAGAGGTGGCTCTCAGGTA
										6D-1700490-T9-32_448	TTGGTGTCAGACACTTTAGATCGATTCAAGGGTTCAA
										6D-1700490-T9-32_512	ATGCAAAAATATAAATGATGTACAGTGAAGTGTCACTT
										6D-1700490-T9-32_576	AAATGCTCGCTGATCTAGCCAGAGGCCTCATCAAAGC
										6D-1700490-T9-32_704	CCCAAAGAGGGCCTCCTATCCCCTCGCCCTGGCACGA
										6D-1700490-T9-32_768	GCTCTTAAAGCATGGAAGTTAGCTGAACACCAGACCA
										6D-1700490-T9-32_832	CAACCATCGCCAGCCATGCCACGCCACCGTAAACAAC
										6D-1700490-T9-32_896	AGGCTCGTCTTAATATTGTGATTTCACAATCATTTAAG
										6D-1700490-T9-32_960	GCCCAATTTTGATCAACAGATGTATGTGTTGTATTCTG
										6D-1700490-T9-32_1024	CTTAACAAATCGTAGCTGATCTTACTTTTTGATTCCTTT
										6D-1700490-T9-32_1280	TCTCTTTCCACTGAACGCACATCAACGGAAGGGCTGA
										6D-1700490-T9-32_1344	ACTAGTAGATCTACACATGATTTATTTTCCATGGAAAA
										6D-1700490-T9-32_1408	AAAACACAACTACCACCACGGTTACGTTCTCATATAAA
										6D-1700490-T9-32_1472	CCTTCTCTCATGTGGCCAGGATATTTTTCTGGCCACTG
										1A-0078610-T9-33_0	CAGACAagagtagagagagagagagagaaaggaggagagCG
										1A-0078610-T9-33_315	TTGGTCCTCATGATCCAGATCACCAACTACACTCATGG
										1A-0078610-T9-33_378	GATTTTTTCGACCATTGGCGATTGGGGGTGGAGGGT
										1A-0078610-T9-33_567	ATTCCTTACAGCTAGACGTTATACATGTATGGCGTCTA
										1A-0078610-T9-33_630	TCAGACATTGCACACAACTGGATGAAATGTTTTAAAA
										1B-0168230-T9-33_0	Cagacgagagtagagagagagaggggagaggtggcagaagagaa
										1B-0168230-T9-33_256	CCCCGTTGCCGTTGCCATTCGTCAACTCATCACCCTGC
										1B-0168230-T9-33_320	CTCGTCCCTTTCTGACAGCACCAACTACACTCATGGA
										1B-0168230-T9-33_384	GTTTCGACCATTGGCGATTTGGGGTGGAGGGTAATG
										1B-0168230-T9-33_512	CATGGATCACACGTACCTGCACTATTTGAATAGGTCCA
										1D-0254920-T9-33_0	CAGACGAGAGAGTGGGGGAGCGGTGGCAGAAGAG
										1D-0254920-T9-33_256	CCGTTGCCATTCGTCAACTCATCACCCTGCAGGCTCCA
										1D-0254920-T9-33_384	GATCATTGGCGATTTGGAGTGGAGGGTAATGAAACA
										1D-0254920-T9-33_448	GAAGAGTGGAAACTTAAGAGACGAGGCAACCAAGT
										1D-0254920-T9-33_512	GGATCAAACGTACCTGCACTATTTGGATATAGGTCTCA
										1D-0254920-T9-33_1408	TCGCCTGGCTCGTGGCGGCGGCTAGGGTAGGGGAA
										1D-0254920-T9-33_1472	CCTTACATGCTGCACGATTCTCTCTTCGTCTCAAAATA
										1D-0254920-T9-33_1536	AATGAGGAAGTAGTATATAATAACCACATACGCATCTG
										1D-0254920-T9-33_1580	AGTTTTCTTATATAAGTATATATTTATTGTAACAACAAC
										1A-0018880-T9-34_64	AGCTAGTTCCGACAGAGTCAATCAGCCAGTCTCGAA
										1A-0018880-T9-34_128	AGCTAGCTAGCGACGGAACAGGCAGAATGTTTCCCT
										1A-0018880-T9-34_384	TCTTGACCAAAAAGATCAACCTCGCCGTGGTTTCACA
										1A-0018880-T9-34_448	CGCGCGGTCAATAGTCAACACCTTTACTGCCTTTTATT
										1A-0018880-T9-34_512	TTGCCTTTGGAGCCAAGCACGTAGAAGAAGAAGCGG
										1A-0018880-T9-34_576	GGCATTGCACTCCAAGTCTCCGATGACCCCTTTTCGG
										1A-0018880-T9-34_640	CAAACGCTAGCGATTCAGCGGAGACGAAGCCGGGC
										1A-0018880-T9-34_704	AGgacggagacgacgacgacgacgacgGATCGGGCCAGGG
										1A-0018880-T9-34_896	GCATGATCGTGGTCACCACGGTTAGCTTGGCCGATGC
										1A-0018880-T9-34_960	GTGGGATGCGAGAGCCGTGCATGCATGGATATGGAT
										1A-0018880-T9-34_1152	GTGTGGTGGTTAGGCATAGTATAAATATGGTTTCTCTT
										1A-0018880-T9-34_1216	GTTATTCTTTGGAGATGACACTTGACGTGGAGTTTAG
										1A-0018880-T9-34_1280	TCAAGTTTATTTTAGAGGGGACGACGACGAGTCAAG
										1A-0018880-T9-34_1344	CAAGTGTATATAGAATCAACATTTTGACTTGGAAGGA
										1A-0018880-T9-34_1408	ACAAGCTTTTCTTATTTGAAAACGGAGATGAAGTCAG
										1A-0018880-T9-34_1472	CTAACAACTCACACGGAGGCACAGAAAACACATGGT
										1A-0018880-T9-34_1536	AACCTGTCTGGCATCAACCGCTAAACACCACTATCCA
										1A-0018880-T9-34_1580	TAGCGGCGAAGCGAGCCTCAAGAATCTTAGGCTATTT
										1B-0093380-T9-34_0	TGAAGTtgtgtgtgtgtgtgtgtgtgtgtagtgctgtgtTGCCTT
										1B-0093380-T9-34_64	GGTTGAGTTGGTTTGGGTGATGTGAGATGCGCGGGA
										1B-0093380-T9-34_256	GCTCATGCAGAAAGTGTGTGCTAGCTATGATTTCTGTT
										1B-0093380-T9-34_320	GCATTGAGTAACTGAGCAGAGGAGGCTAGTTTGCCA
										1B-0093380-T9-34_448	GAAACACGCCCAGGGATAAGAATGTAATGCAATCTCC
										1B-0093380-T9-34_512	TTAAGAATCCCTTGGCAAGGGGATATGCATGACTACG
										1B-0093380-T9-34_576	ATGCAACCCTCGGCTAATGAAAGGTACTCCCTCCGTT
										1B-0093380-T9-34_640	AATGATAATAACATATAAATTTGTACATCAATCGGCATG
										1B-0093380-T9-34_704	AAGTGCAAGTGTTAGCTTATGTCGTAAAATCTCATCTT
										1B-0093380-T9-34_768	TTTACTGTTGTAACATGTAGAAAATATGCGCTCAAACC
										1B-0093380-T9-34_832	TGGCAGTCCCAAATGGAGCCTAGTTCTCACTCTCTTT
										1B-0093380-T9-34_896	CCAAAAATCCAAACATTTATATCAGCTAAGATTTTCAA
										1B-0093380-T9-34_960	TGTTATCTTTGAACAAAACACTCTTGCTCCTACATGAA
										1B-0093380-T9-34_1024	TTACCATGGGAGAGAGAAGTGTCTAGCGTCACCAATA
										1B-0093380-T9-34_1088	GTCGTGGTTTTAGTTCAACCTACCAAGTACTATCATAG
										1B-0093380-T9-34_1152	GTGTGCTCTACAATCCCTATGCCGCGTAAAGAGATAA
										1B-0093380-T9-34_1344	TAACTGACAATGCAGCCGCATCCTTGTGGATGGTGTC
										1B-0093380-T9-34_1472	TTCAAACTCCTTCTCTTTTGCACACCTATGTGAGATCA
										1D-0199480-T9-34_64	AGCTAGTTCCGACAGAGTCAATCAGTCAGTCTCGAAC
										1D-0199480-T9-34_256	AGCCGGAGCGGGGCCCGGCGGTACGGTACCGcgtcct
										1D-0199480-T9-34_448	GGTTTCACACGAACCGATCATCAGCTCACCAGGTCTT
										1D-0199480-T9-34_512	AATACCTTTACACTTTTTTTACCGCTAATTATTGCCGCT
										1D-0199480-T9-34_640	ACCCCTTTTCGGGTTTGATTCTTTCGTCGAGTGTGCGT
										1D-0199480-T9-34_896	CGCATCGTTCTTCCGGACGTAGTGCAGAGGAGTCAG
										1D-0199480-T9-34_1024	tggtagggtggTCCTCCTGCCCAGGGCCAGCTCTTCTTCT
										1D-0199480-T9-34_1088	TGGGTGCATGGTGATGGTGGGAACTGTGGAAGGGT
										1D-0199480-T9-34_1280	GCATACAAATATGGTTTCTCTTTCTCTGTGCTCTGAACT

										1D-0199480-T9-34_1344	GACACTTGACATGGAGTTTAGTTTTCTTGGCCGGTGG
										1D-0199480-T9-34_1408	TAGAGGGGACGACGACGAGTCAAGTTTTTCATACTG
										1D-0199480-T9-34_1472	AGAATCAACATTTTGACCTGGAAGGAGAGGAAGTGA
										1D-0199480-T9-34_1536	GTCAAGTTTTTCTTATTTGAAAACAGAGATGAAGTCA
										1D-0199480-T9-34_1580	GGGCAAGGTTCGTCGCGCTAACAACTCACACGGAGG
										5A-2078230-T9-35_128	GAGGGAGGGGCGATGATTTTCTCCATTTCTTTTCCGC
										5A-2078230-T9-35_192	GCGTTAGGAGAAAAAAGGAGTCTCTCAAGGTTACAA
										5A-2078230-T9-35_256	AGTTTTAGATCATGCGCCGGAGCACCTGGGTAATTTG
										5A-2078230-T9-35_512	CGGTTGGCGCCATGATTCGTGTAGCCATTTGTCGTGT
										5A-2078230-T9-35_640	GACAGTTGGGCTTGGCAAGACCAATGCGTTGATCTC
										5A-2078230-T9-35_832	CTTATAGGGATGGTGACAGAGATCGCCAGAAAAATG
										5A-2078230-T9-35_896	CTTGTACTCCGTTGAATAATTTAATAAAAGAGTTTTCT
										5A-2078230-T9-35_1088	TGATGTGTGGGACACCCCATGCCATGTGGCGCTCTCA
										5A-2078230-T9-35_1216	GATTCCATGCAGGAAGTCACGTCGCCGGTGTACCCGT
										5A-2078230-T9-35_1280	ACATGTCGCACACTGGCGTACACCCACTCCAGCCGAG
										5D-1440510-T9-35_384	ATATTGCCTTGGGCATCCCCAATCTTAGGCTCTTGTCA
										5D-1440510-T9-35_512	CGGGAAGAAGGGTGTGAGAGGGGAGGGGAGGGG
										5D-1440510-T9-35_704	TGTGGACACACACACGGGCGACTGTGAGAGCGCCAC
										5D-1440510-T9-35_896	TTTGTAACCAGGGCACAAAAATAGAAAACCCTTTTAT
										5D-1440510-T9-35_960	CAAATACATAGATCAACCCAAAGCCATCTTTCTCGCGA
										5D-1440510-T9-35_1088	ACAGTGAGTTGAGAGCATCATCCGGTCTAGCATATCC
										5D-1440510-T9-35_1152	TCTTCCGTCGTCCCAATTTCAAAAGAGATCAAAGCAA
										5D-1440510-T9-35_1216	ACGTCACAATGACACCAGACAACGCCGCCGTTCTACG
										5D-1440510-T9-35_1344	GTAAACAAGAAATGAATCCAGACCACACACGATACCA
										5D-1440510-T9-35_1408	TTAACCAGGTGCTCCGACGCATGATCCTAAAACAAGA
										5D-1440510-T9-35_1472	TGCGAGACTCCTTTTTCTCCTAACGCAATTACACGCAA
										7A-1752710-T9-36_0	GGATGAATGCCAATCGAGGGACTAGTAGTTGAGAAC
										7A-1752710-T9-36_192	AGCATTTGACTCACGCGTTGTTGTACGGGTCGATCTG
										7A-1752710-T9-36_256	GGCTCGTTGAATAATCAAGGATGAGAAGCTGCTAGC
										7A-1752710-T9-36_320	TACTGCataataataataataataataataataGGATGCTTCA
										7A-1752710-T9-36_384	AAATTACTATTTCACATCTAGATGTGAAATACTTATTTC
										7A-1752710-T9-36_448	GTGTGCCCCAGCCCTTCCTGCGTCGTTGGCCATTCCG
										7B-1867430-T9-36_0	CTCTGCCGATCGCGGCTGGAACCTGGAAGAGGCAAG
										7B-1867430-T9-36_66	TATACCATGCAATGGCTAGCTACCAGCTTAGCTTAGGT
										7B-1867430-T9-36_264	CAAGAGGATCAGGGAGATTAATTAAGTGGACTAGCG
										7B-1867430-T9-36_462	GAAAGACTGGATGTGTAGGTGGGTATGTACGGCATG
										7B-1867430-T9-36_528	AGCTAGAGAGGTCGATCGCATTAAAAAAAAAAGCTA
										7B-1867430-T9-36_588	GATCTTCTATATCTAAATATCTAGCCTTCACTAAGTTATT
										7B-1867430-T9-36_1046	GGCCATGGCGTGGGCTGACACATCTGGACCGGGGTG
										7B-1867430-T9-36_1166	GGGTTGCAGGCGGCTGCTCTGCCGACCGGCAGCGTG
										7D-2007220-T9-36_64	TTGAGATGATCGAGGGATGTGTCGCAGCTCTGGACTG
										7D-2007220-T9-36_256	TTGAATAATCAAGGATGGGAAGCTGCTAGCCTACTGC
										7D-2007220-T9-36_576	TGATATGTCGGATCTTTAATGGATGGCTGAAAAATGG
										7D-2007220-T9-36_640	TGAAGTCAGTGAAGCAAGATCCTACATTTTTATGTTCT
										7D-2007220-T9-36_704	TTTGCCAACGGTTGCACAATATGTGTTTTTTCTCTATTT
										7D-2007220-T9-36_768	TTTCATTAGTCATGGGAGAAGGTTTAGACATATTGGG
										7D-2007220-T9-36_960	GCATGGGCGCGAGACCCGAAGGGTATCTTCTCTGTG
										7D-2007220-T9-36_1088	GACATTATGAGGGTGCCCTGTTCCCCCAAAGGAACG
										7D-2007220-T9-36_1152	TCATTGGCAACGATGCAAAATAAATTCAACCGGCACA
										7D-2007220-T9-36_1280	GGAGGCGATGCGGGAGTCTTGGACATTACCGAAGCT
										7D-2007220-T9-36_1472	ACGCCGATTTCTGAATAGCTACATAGAGTCCTTGTTGT
										3A-0699520-T9-37_0	CCGCCGGCGCATAGCTCGCCGGAACCAGCCAGCGCA
										3A-0699520-T9-37_384	CTCACAATGCCATCACAAGAGAGATCGATGGATGATC
										3A-0699520-T9-37_576	TCCAATGCTCTGACGGCAACGTGGAGATTCTGTAAGT
										3A-0699520-T9-37_640	TCTTTCCAAATGTTGACGCAATGCCTCCACATGATTAT
										3A-0699520-T9-37_704	ACATTGTTCAAATCATATTTGTGCCAAATTTATGTCAG
										3A-0699520-T9-37_768	TTAAGATGTTTGCAAATCATTTGTGCCAATACGATTAG
										3A-0699520-T9-37_832	ATGTACTTGAGTTCTTATTTTCTTCAACATATAGGAATT
										3A-0699520-T9-37_896	CCTCGCACCTGAGATTGGAAGCCTACAACGCCTAAGA
										3A-0699520-T9-37_960	TCTGGATTATAAGAGGTTAATTTAGAATCCCATTGTTT
										3A-0699520-T9-37_1088	GCCTTACTGGTTTCGAAAAATCATGCAAAAAAGCATG
										3A-0699520-T9-37_1152	CACCTAGAGACTTTCATTTAAAGTTATGAGTTTCTGTA
										3A-0699520-T9-37_1216	AAGAACCACATCGGGTACTATTCACTTATCTCTTGTTT
										3A-0699520-T9-37_1280	AGTTTTTGATGAAAAATTCTAGATGCACCATTGCACCT
										3A-0699520-T9-37_1344	TTCTTTTGTTTGATCAATATTATTTGAAATCCATGTGTT
										3A-0699520-T9-37_1536	GGACGATGCCACTACTCAAACAGCTGTAAGGCCAATT
										3B-0810080-T9-37_0	TCTGTAACATCTCTCTGCCTTTCATTCCCTAAGACACAT
										3B-0810080-T9-37_192	GAGTCGAGTCTTTCAAGAGAGGAGCTTGGCTGGGC
										3B-0810080-T9-37_320	TATTAGCTTCTGTGAACAACTCTGTATCTGGGTTTCGT
										3B-0810080-T9-37_384	AACTCTTCTGAATGCCTGAACCTTTATATAACTTGTCT
										3B-0810080-T9-37_448	AACACTTAATTGCTCCTGCGTGTTTATTTTTATTTTTTG
										3B-0810080-T9-37_512	TTGGTTTTTATTTCTACTAGTACGATGGGCCCTTTTAG
										3B-0810080-T9-37_576	TTGGATGCGATGGTGTGTTGAATTCTAGAGAATTGAA
										3B-0810080-T9-37_640	AAAAACAGAAAACACACAATTTTAAAGAATTCCAGA
										3B-0810080-T9-37_704	AAAAACATCTATGGAAATCTTCTTTTGAAAATGCACTA
										3B-0810080-T9-37_768	AATATGGAAAAAGCCACATGGTACATACTCCTCCAAA
										3B-0810080-T9-37_832	ATATCTCTAAATTTCTGGtactccctcttttttttaactctgcata
										3B-0810080-T9-37_896	caaacttcctgaagtatgatcaagctcgtagaaaaaaatattaacat
										3B-0810080-T9-37_960	tacaatatgtaaatatattccatgatgattctaatagtattgatttgata
										3B-0810080-T9-37_1024	atttttccgtacaaatttagttaaattttaaaaagtttgacttaaaaata
										3B-0810080-T9-37_1088	ggactaaaaagacacggagggagtaTAGAATTCTTTTGAAG
										3D-0913830-T9-37_0	TCTTCTTTTGAAATTGCACTAGATTATGGAGAAAAACA
										3D-0913830-T9-37_65	ACATGGAGCATACTCCCCCAAAAATTATTTAAAATTCT
										3D-0913830-T9-37_325	ATCTGAGACTTAGCAAAATTGAAAAATATTAGAATATC
										3D-0913830-T9-37_390	CATGATGATTCTAGTAGTATTTATTTTATATTGTAAAAA
										3D-0913830-T9-37_455	GTTAAATTCTAAAAAGACACGGAAGGGGTATAGAATT
										3D-0913830-T9-37_520	CCATGCTCTATTTCTGCGGTTTGCCGCGCGCGGCGGC
										3D-0913830-T9-37_780	CACCCATGGCCGCCGGCGAATAGCTCGCCGGAACCA
										3D-0913830-T9-37_910	AGCAAGGCCCCACCGGAGCTAGCTAGCGCCTCCGGG
										6A-1555880-T9-38_0	CCTCCTCGCTACCCTTAGCTAGGTAGCTTGGATTGGC
										6A-1555880-T9-38_192	ACCGGTCGGTCTTGGCGGAGCGTATTATTTTTCTTCG
										6A-1555880-T9-38_256	TAGGATCCGTCTGCTTCACGTGAGAGATGACAGGTTG
										6A-1555880-T9-38_320	CAGCTCGTAGTGGACGATCCGGATAGCTGCCATTCAA
										6A-1555880-T9-38_384	GGAAAGGCCTTCAGCGTGCGAGTAGTCTTGTTTGTGT
										6A-1555880-T9-38_448	TAAAAAGACCATTCTTTCTTCTTCTCTCATTTCGTCAAG
										6A-1555880-T9-38_512	TAGTGGCTCCAATCATATTTACTCAGAACCACCGCATA
										6A-1555880-T9-38_1152	TTTCTTTTTTGTTTTCCGATCTAAGATCGGTTTGTGTC
										6A-1555880-T9-38_1280	CACATCTGTCTGTTGTTGATCTCTGCCGGTTGTCACCT
										6A-1555880-T9-38_1472	TTGTTCTGCACCTCCCGTGAATCAAAGCTTCCACAGG
										6A-1555880-T9-38_1536	GCTTCACGTATCTGCGCCACCGACAGCTGCTTGTCAC
										6A-1555880-T9-38_1580	AGGCGATCCGGCACTTCGTTCATCATCTGCGTGCGAT
										6B-1657400-T9-38_0	CCTCCTCGCTACGCTTAGGTAAGTAGCTTGGATTGAC
										6B-1657400-T9-38_64	GAAaggggtaagagctagaggtataggaggaggtggatatagag
										6B-1657400-T9-38_192	CAACGGCCCAAACGAGGACGGGACGACTGGTCGGT
										6B-1657400-T9-38_256	GTATCTATGAATAGGAGCCGTCTGCCTCACGTGAGAG
										6B-1657400-T9-38_320	TAGTTCACCAGCTCTAGTGGTGGACGATCCAGATGGC
										6B-1657400-T9-38_384	ACGTGTCTTGCGGAAAGGCCTTCAGCATGACTAGTCA
										6B-1657400-T9-38_448	GTGTTTACACCCATAATAGGATTTTAAAAAGACCATCC
										6B-1657400-T9-38_768	CTAAGGTGGGCATATCACTGTCCCTCTAACCATCTAAC
										6B-1657400-T9-38_832	TTCTTTAAACAATGATATTTAACGGCTCTTGTCATATTT
										6B-1657400-T9-38_896	GGGTAACATGTGCCCACCGTCTTTACCAAAAAAAAAC
										6B-1657400-T9-38_960	CAAGTAAGTAATAAATCGCACTGATTCTTGTGTTATCT
										6B-1657400-T9-38_1009	TGTTTTTCATTGACGGGCCCACCTATTAGGCTAACTTG
										6D-1737520-T9-38_0	CCTCCTCGCTACCCTTAGCTAGGTAGCTTGGATTGCTG
										6D-1737520-T9-38_192	GGGGACGGGACGACTGGTGGGTCTTGGCGGAGCGT
										6D-1737520-T9-38_256	TTTCTCGTCGGATTGGATCCGTCTGCTTCACGTGAGA
										6D-1737520-T9-38_704	TCAATCTGATACGTCGGACCTTTAATGGATGGCTAGTA
										6D-1737520-T9-38_768	ACTGAAGCTGCATCCCAAATCAGCAGCTCTAGTGGTG
										6D-1737520-T9-38_832	TCCAATGGCTGGGAACGTGTCTTTCGGAAAGGCCTT
										6D-1737520-T9-38_896	ATGGACTTTTTTGTGTGTGTTTTCTACCGCCATAATAA
										6D-1737520-T9-38_960	TTCTCTCATTTTGTCACAGTATTTTGCTTTAAATCGTGA
										6D-1737520-T9-38_1024	ATCAGAACCATCACATATTGTACAGGGTAACATGTGG
										6D-1737520-T9-38_1088	ACCACCGACTCTAGTCTCTACCGCAAGTGAGTGACAA
										6D-1737520-T9-38_1152	TCTAACAACGCTTTTGACTGATGGGCCCACCTATTATG
										6D-1737520-T9-38_1216	GACCGGTGACTAGTCTCTcacacacacgctctctctctcaca
										6D-1737520-T9-38_1408	CTCCCTCTTCCTGCAATCTTCCGATCTAGATCTAAGAG
										6D-1737520-T9-38_1472	ATCTATGGTATTTTTAATAGTAGACAAGCAAAGATGGA
										6D-1737520-T9-38_1536	GAAAGATAGATAACAAGAGAGTAATGTGATGATGTGA
										3A-0675860-T9-39_384	TAATGTGTCGGCAGCTGATTTTGTTGCCGGCGTGAAG

										3A-0675860-T9-39_448	TTATTGTTGGCCGGTATGAACCTCGGCCGCCCATTGC
										3A-0675860-T9-39_512	AAATGAACGGGGTCATGGGTTGGATTTGCTTTGCCCT
										3A-0675860-T9-39_576	CCCTCTGGCTGTCCGTGCACAGTTAGTTGTCGTTCAG
										3A-0675860-T9-39_768	CCAGCGAGGACCCATTTTCGCGGAGGCTGACAGCGA
										3A-0675860-T9-39_960	CGGGTGCGTGCGTGATCCCACCGCCCTTTAGTGATTG
										3A-0675860-T9-39_1088	ATCGAGATACTGGTGTAGCTACGTGCTACTAGAAGGC
										3A-0675860-T9-39_1216	AGTCTTAGCAAGCATGAAAAAGGGAAAACTGAAAAC
										3A-0675860-T9-39_1280	ACTGGGAAACAGTGGGAGAATTGATTGGGGATATGG
										3A-0675860-T9-39_1472	CACATGTGTACAATCGTCAACAAATCCAAAGCATCCTC
										3A-0675860-T9-39_1536	AGCGTTCGCCTCTGTTTCTTCCTAAGCTCAGAGATCCT
										3D-0928760-T9-39_512	GCGGGCGTCTGTTTGGGGTCGCGCGGTGGAGTTGG
										3D-0928760-T9-39_576	CTCGGCCTGTCCGTGCACAGTTAGTTACTTTATAGTGT
										3D-0928760-T9-39_640	CACGAGACATGTGGTCTGGTAGTTCCGCGTTTGCCAG
										3D-0928760-T9-39_832	ACATTTCAACGATTTCAGTTCGTAATCCATGGCAAAAT
										3D-0928760-T9-39_896	TAGGGCGACTGCCTCTTCCACGCCTACCTAGCCCGCC
										3D-0928760-T9-39_1088	GATACTGGTATAGCTACGTGCTACTAGAAGGCAGCAA
										3D-0928760-T9-39_1216	AAGCATGAAAAAGGGAAAACTGAAAACGAATTGACA
										3D-0928760-T9-39_1280	CTGGTAAACAGTGGGAGAATTAATTGGGGATAGGCAT
										3D-0928760-T9-39_1472	AACATGAGTACAATCGCCAACAAATCCAAAGCATCCT
										2A-0332190-T9-40_0	ATTTAGGCATCATTCTATTCTCATGAATTTTAGAGCAAG
										2A-0332190-T9-40_64	TTTAGTTGATGTGCACGAACTCTTGCGATTGTTCATTAT
										2A-0332190-T9-40_128	CTCTAGGGACCGTGGATGTAACTGAATTATGGATGTC
										2A-0332190-T9-40_192	TGTCATCTTAGTCATTGGCCATGTGAGTCGCATGCCGT
										2A-0332190-T9-40_256	AAAACCGAGCAGTAGAGCGGTGCGAGTGGGGGATG
										2A-0332190-T9-40_320	TGTTTAAATTGGATACATTGATGAGAAAACCTCTAAAT
										2A-0332190-T9-40_384	TTGACTGAGGGTGGAAATGAAATTCATTCTATTTTTGT
										2A-0332190-T9-40_448	TGTTGACAAAGTTTATAAATACATTTAGTATTCACCTGT
										2A-0332190-T9-40_512	AGTATGAATTAAAACCGTACGATGTATTTATGGAAAAG
										2A-0332190-T9-40_576	CACGGTCCATGTTTACATATAGTGTAGCGCACAACATG
										2A-0332190-T9-40_768	ACCATTCCATTGGAGCCCGGACAGCGACAAAACCTCT
										2A-0332190-T9-40_832	GCCCGACAAGTTAAAATCCACGGCCATGGCGACGGC
										2A-0332190-T9-40_1216	gttgtcccTAAAAGGGACGCCAACCACTCTCTTTCTACG
										2A-0332190-T9-40_1280	TTCCTTGGCTTGGCCCCAGTCTACTCTGCTCTACTCAC
										2A-0332190-T9-40_1344	CCTCTCTCTCTCTCCCTCCAACCAAATTGCAATTGAGC
										2A-0332190-T9-40_1580	CATATCCACCGCCGCCGCCCGGATGCATACGTGCGTG
										2B-0493430-T9-40-1_64	CGTATCAACTTGGGTAGATGAACCCCTGAAAAACCGA
										2B-0493430-T9-40-1_128	GTAAAACATGGCATATCATGTTAATTCAGTGTTTAAATT
										2B-0493430-T9-40-1_192	AATTTGGTATGTTTTCATTCGTGCCATCCTTGACTGAG
										2B-0493430-T9-40-1_256	TAATGCCTAAAATCCTGCAATATATTAGCTGTTGACAA
										2B-0493430-T9-40-1_320	CTGTGTAATTCTTTGGCAGTAATATATACGTATGAATTA
										2B-0493430-T9-40-1_448	AGCGCAACGAGACTCAAAAAAAAAAAATAGCGTAGCG
										2B-0493430-T9-40-1_640	GCTGCTAACTCCATCCGCTGCACGTCAACCTTAACCAT
										2B-0493430-T9-40-1_767	CGTACCAACAAGTTAAAATCCACGGCTATGGCGGATC
										2B-0493430-T9-40-1_1383	AGCTCCTCCATTAGTCCATCATTCACCGAGCAGAGCA
										2B-0493430-T9-40-1_1580	ATGCACATATCCACCGCCGCCGCCCGGATGCATATGTG
										2B-0493430-T9-40-1_0	TCCACGCCCGAACCTCCATATAATTCATGTGACATCTTA
										2D-0580850-T9-40_0	ATTCCGCCGGCCTTGGGAGGGGATCATAAATCAATCA
										2D-0580850-T9-40_64	CTCTAAAAACTACCTATTGACTTGTATTTCAAAAAATA
										2D-0580850-T9-40_128	TGCAACAACTATAGATGGTCATGGACGAAATTGTGAA
										2D-0580850-T9-40_320	ATACAAATATATTTGATTTAGGCACCATCATATTCTCATA
										2D-0580850-T9-40_384	CGAGTTACCTGTTTTTTTAGTTGATGTGCACGAACTCT
										2D-0580850-T9-40_448	TTTGAACATGTGTGGCTCTAGGCACCGTGGATGTAAC
										2D-0580850-T9-40_512	TTCATATAATTCATATGTCATCTTAGTCATTGGTCATGTG
										2D-0580850-T9-40_576	TAGAGATACCCCTAAAAAACCAAGCAGAAGAGCGGT
										2D-0580850-T9-40_640	ATCATGTTAATTCGGTGTTTAAATTGGGTACATTGATG
										2D-0580850-T9-40_704	TCATTCATGCCATCCTTGACTAGGTGGAAATGAAATTC
										2D-0580850-T9-40_768	GCAATATATTGCAGGATTCAAAGTTTATAAATACATTTA
										2D-0580850-T9-40_832	AGTACTAAATATATATGAATTAAAACCGTACGATGTATT
										2D-0580850-T9-40_896	GAGCCTTCACCTTGTCGCACGACGCACGGTCCATGTT
										2D-0580850-T9-40_1088	GCTGCACGTCAACCTTAACCATTCCATTGAAGCCCCG
										2D-0580850-T9-40_1216	ATCCACGGCCATGGCGGATCCTGAACCGCATCGCCCA
										2D-0580850-T9-40_1280	TACGAACTCCATCCTCCTCTCTCTCTCTGCCTTTCCT
										2D-0580850-T9-40_1344	TACTCACTCCGGTCCCAACTGCCAAATCCCCTCGCCC
										2D-0580850-T9-40_1408	TTGGAGAGCTCTGATCTCCTCCATTAGTCCATTTCCCG
										2A-0289660-T9-41_0	CTCcctctcctctcctctcctctcctctcctctcTACCAGACAAAC
										2A-0289660-T9-41_64	TCCTCGGCCCTCCTACTAGTGGTAAGAAAGATAGTAG
										2A-0289660-T9-41_256	AGGCCAGTGATGAAGCCTCCTCCCCTCCCCTCCTCTT
										2A-0289660-T9-41_512	ATCGCCACCGGCGGTACGACGGCGATCCGCGCGTCG
										2A-0289660-T9-41_704	CCGAGGGGCTAAACAAAGTCAGCACGTGGATTCGGC
										2A-0289660-T9-41_832	AACTAAATCATGCTCTCGTGGATGCGTATCCTGCGGAT
										2A-0289660-T9-41_896	CAGTGCTGGCCGCGGATATGATGATGGTATGGTGTGC
										2A-0289660-T9-41_960	ACCAGATATGACACTGCATCTTTGCTTTGCTCCGCCCT
										2A-0289660-T9-41_1024	AGTGGATCAAGTATGGATCATTCGCAGCAACAACAAC
										2A-0289660-T9-41_1088	ATCGACTCTTCGACTTTGAAGTGAATTTCAAGAGAAC
										2A-0289660-T9-41_1152	GACAAGCAGAGATGGCAATCTATTTACATGAAGAAA
										2A-0289660-T9-41_1216	TATTAGCAAAATGAATAAAACACAATAGGTATTTCGTC
										2A-0289660-T9-41_1280	ATGTGTCTTCGTTGCTCCGGGTTTCTCACATCAAAAC
										2A-0289660-T9-41_1408	TACATGGGGCGGACCTGAAGAGGGGGTTGTGGAAG
										2A-0289660-T9-41_1536	GATCTCGACAATGTTGAAGGTGGTTTTACCCAGGTTT
										2A-0289660-T9-41_1580	CCGATGCGTAATAACCCTATGTTGTGCCTACCTATCTTT
										2B-0435700-T9-41_0	CTCCCTCTCCTCTCCTCTCTACCACACAAGCAACCACT
										2B-0435700-T9-41_192	GCATTTGATGACCCGGTGCACCTGCAGGCGAGTGAT
										2B-0435700-T9-41_384	TTCCCCTCCCCCTCCCCTAGATTTTGCTGTCGGGGAAT
										2B-0435700-T9-41_576	CAATTCTCATTATCTTAGCTTTGGTGATTATTATGTTCG
										2B-0435700-T9-41_640	CCTGCACGGGGTCCGATCGCGTCCGATCCTTTCTGGG
										2B-0435700-T9-41_832	TGCCAACTAAATCATGCTCTCGTGGATGCGTATCTTGC
										2B-0435700-T9-41_960	AGATAAGACACTGCATCTTTGCTTTGCTCGCCCTGTCT
										2B-0435700-T9-41_1024	GAACAAGTATGGATCATGTTTATGTCTCAGGCTAAAC
										2B-0435700-T9-41_1088	caataacaacaaACAGTTAGCTGCAGGCTAGGCATCTCC
										2B-0435700-T9-41_1152	AATTTCAAGAAAACTTCCAGCATCTAGCGGGATTTAAT
										2B-0435700-T9-41_1216	TTACATAAAAAAACTTCCTCGATTGATTCTTAAACTTT
										2B-0435700-T9-41_1280	ATAGGTATTTCCTCACCTGCAAATGACTACAACAACAC
										2B-0435700-T9-41_1344	CTGACATCAAAACCATTTGCTTCGAATGGGTAATTAG
										2B-0435700-T9-41_1472	GATGGACCTGAAGAGGGGGTTGTGGAAGATCCTTGG
										2B-0435700-T9-41_1536	ATGCATGTCGATGATTAATGCACTGATGGAGAGGGTC
										2B-0435700-T9-41_1580	TAAGTAGAGGAAATGGTGGTGCCCGAATAGACCTCG
										2D-0539450-T9-41_0	CTCCCTCTCCTCTCCTCTCCTCTCTACCAGACAAACAA
										2D-0539450-T9-41_128	AGTGAGGTGAGGTCAGAGGCGGCACCGAGAATGGG
										2D-0539450-T9-41_192	AGAGTACGCGCATTTGATTTGAGGACCCGGCGCACC
										2D-0539450-T9-41_512	GTGGTACGACGGCGATCCGCGCGTCGTGGCGCCACG
										2D-0539450-T9-41_576	CTAGTAAAACAATTCTCATTATCTTGGCGTTGGTGATT
										2D-0539450-T9-41_768	GGAGATGTTTATTCGCATGTCTGTGCCAACTAAATCAT
										2D-0539450-T9-41_832	TTATGTATATGTGTTTCCGCTGCTTGAGTGCTGGCCGC
										2D-0539450-T9-41_960	TCCAGCTAAATATATAGCAAGTGGAACAAGTATGGAT
										2A-0304560-T9-42_192	CAAGTGGTATGTATACAAGAGAGGCTGGGTGCAATG
										2A-0304560-T9-42_256	TGGACACAATGCGTGGGTACATCACAGTAGTCAAAG
										2A-0304560-T9-42_320	TTACAACCACGAAAAAAAACAGGTACATTAACAGGT
										2A-0304560-T9-42_384	GACTTTTACATTTGTCACCACGACCGCGCTCACTATCA
										2A-0304560-T9-42_576	TGGATGAGGCTCCGTCATCTTATACCTCGAGTTGGTC
										2A-0304560-T9-42_640	GCGGCCCACAACGTCCCTTcctccccccacccccccacccca
										2A-0304560-T9-42_704	cctaccacaaccacaaccGCACACACAGTTGAACTTGCCAT
										2A-0304560-T9-42_896	TTCCTTCTCCCCTGAATTGACTCTCTCAATTCTTAACAA
										2A-0304560-T9-42_960	ATAAGATGTTTGAAACATACTCCCTTCGTTCGGAAATA
										2A-0304560-T9-42_1024	AACGGAGGGAGTATAATGTAACGATTACGTTCAGAAC
										2A-0304560-T9-42_1088	TTTCTCTAAGAAAAAACAAAATTTGACATCGAAAGAC
										2A-0304560-T9-42_1152	CACCCCAATATCTTGACTAAAACGTCCCAAGACATCAT
										2A-0304560-T9-42_1216	GTCTAATAAGAGCATCTACAACCAGATATATCTCATCCA
										2A-0304560-T9-42_1280	CGAGCGCATTCGTGGGCACTGACCGGTCATCTCTCGT
										2A-0304560-T9-42_1408	GCTTAGCCGACATAATTTCGCATACTAACTTAGCGGAC
										2A-0304560-T9-42_1472	AGTTCATCACCGGACATTGCAAATACATATTAAAAACT
										2B-0393600-T9-42_128	TGTGAGTGCAACGCATGCATGTGATGTATGCCCAGCC
										2B-0393600-T9-42_192	CAAGTGGTATCTCGTATGTATACAAGAGAGGCTGGGT
										2B-0393600-T9-42_320	TTGCAACAGCTACATTTACAACCACGCAAAAAAAAAA
										2B-0393600-T9-42_384	ATTCAAAGGTGAAATCTGAAGGATTTTTACATTTGTCA
										2B-0393600-T9-42_512	GTCGATTTTGTGGTTGTTGGATGAGGCTCCGTCATCTT
										2B-0393600-T9-42_576	TCCCTCCTCTATCCCTGCAGCACGCAACGTCCCTCCAT
										2B-0393600-T9-42_640	caccctcccaccacaaccacaaccacacacACAGTCGAACTTG
										2B-0393600-T9-42_832	CGCATCTTCCTTCTCCCCTGAATTGACTCTCCCAATTCT

										2B-0393600-T9-42_896	TCGTAAGATGTTTGAAACATACAATGTAACGATTACGT
										2B-0393600-T9-42_960	AAGAGCATCTACAACCGGATATATCTCATCCAGCCCTC
										2D-0518660-T9-42_177	ATCTGGGCTGTGTGGTACTCCAAGTGGTAGGATCTGG
										2D-0518660-T9-42_236	CTGGGGCGCGCAATTTCTTTTTCTTCGGATTTTATCTT
										2D-0518660-T9-42_295	ATATGGATACAACGCGTCGGTACATAACAGTAGTCAA
										2D-0518660-T9-42_354	AGCTACATTTACGAGTAAAAGTGAAATCTGAAGGATT
										2D-0518660-T9-42_413	CGCTCACTATCATTGTCAATTGCCATTCTTACAGTACG
										2D-0518660-T9-42_470	CGCCTGAAAATTGAATTGCGCAAAGTCGATTTTGTGG
										2D-0518660-T9-42_997	ACCGGCAGCCGGGTGTTGCGCATCTTCCTTCTCCCCC
										2D-0518660-T9-42_1062	TACCTGTGGCTAATTAGCTTTTCATAAGATGTTTGAAA
										2D-0518660-T9-42_1127	CACCAAAATCTTCTCTACATATCGACATTTCTCTAAGA
										2D-0518660-T9-42_1192	TCATAAGATGTTTGAAACATACAATGCACCCCAATATC
										2D-0518660-T9-42_1387	CTGCTGCCACAGTTGTCTTCCTTATGTCCGCTAAATTT
										2D-0518660-T9-42_1517	ACATGGTCAGCTGACACAAAATGGTCAAAGTTCATCA
										2A-0277490-T9-43_128	CAAGTGCTAACGATTGCTATGCTGCTTCATCAAATATT
										2A-0277490-T9-43_192	AATATTACAAGTTAATACTGATTCAACTTTTAGTTTATA
										2A-0277490-T9-43_256	GATGATAACATATTCTGCATTTTACTTTAAGCTAGCAAT
										2A-0277490-T9-43_320	ACCATATAGCATGCTAACTATCCTACGGGCCGGGTGAT
										2A-0277490-T9-43_384	AGGATATCTCATGGCCGGATATTTCAAAAATCCCATGT
										2A-0277490-T9-43_448	ACTCTAATGAGACAAAGATCAAAGGGGGGCAATTCA
										2A-0277490-T9-43_640	TATGTGACAGAGGGAGTACTTTTTATATGGACATGATA
										2A-0277490-T9-43_704	CAGAATACACTTTCTATGGCCAAATATGAGGTATACAT
										2A-0277490-T9-43_768	ATGCGTGACAACTATTTATATAAGGCATGCCATACTTTT
										2A-0277490-T9-43_832	ACAACCGGGTGACCACCAACCAAACATGGTCACCAA
										2A-0277490-T9-43_896	AAACGGTTTAGAGAGCAGTTTATGTCATATTTTTGCGT
										2A-0277490-T9-43_960	TAGTGTTACAAGTTGCAAAACTAATATAACAACAAATA
										2A-0277490-T9-43_1024	AACACACTATGTTCCGAAGAAGCATAAAATTATACAAA
										2A-0277490-T9-43_1152	AGACAATCAATACGAACCTATTATGTAACCTCCGCGCC
										2A-0277490-T9-43_1216	CAATATATCCAGTCAATACTAATCCAGAATATAGAACG
										2A-0277490-T9-43_1280	TCATGTGCACATCCCCCGCTGTCTATCGTATCTGACCG
										2A-0277490-T9-43_1344	GTATTTGATGCCAAGGATTAATTGCTTCCAAAACTAAA
										2A-0277490-T9-43_1408	TTTATTTCTTCTAACACCCTGATGAAAAAAAAGGCATT
										2A-0277490-T9-43_1472	TACAGACTCAATCTGGTTGAAAGTGTGTACAACACGC
										2B-0426180-T9-43_64	CAGATAGCTACAATGAACGTCAGTTTCCATCAAGGCC
										2B-0426180-T9-43_128	GCCCACTTGCCTATGCATGCCAACAAATTCAAAATATG
										2B-0426180-T9-43_192	GTGTTGTACACACTTTCAACCAGATTGAGCTTGTGCT
										2B-0426180-T9-43_256	GCCTTTTTGTTCAGGCTATTAGAAGAAGAAGAAAATA
										2B-0426180-T9-43_320	GTTTTGGAAGCAATTAATCGTAGGCGTCAAATACGTG
										2B-0426180-T9-43_384	CGGATACGATAGACAGCGGGGGATGTGTACATAAATT
										2B-0426180-T9-43_448	CTATATTCTGGATTTGTATTAGCTGGATATAATGATTTG
										2B-0426180-T9-43_512	CCGAGGTTACATAATAAGTTCGTGTTGATTGTCTCGTA
										2B-0426180-T9-43_576	TTAATTTTTGCGGTTGGTCATTATGATTTTCTTTCGTTT
										2B-0426180-T9-43_640	AATTTTATGCTTCTTCGGAACATAGTGAGTTGCGCTCA
										2B-0426180-T9-43_704	TATTAGTTTTGCAACTTGTAACACCGAACACTCTAATC
										2B-0426180-T9-43_768	GACATAAACTGCTCTCTAAACCGTCTTCACTTCAATTT
										2B-0426180-T9-43_832	TGTTTGGTTGGTGGTCACCCGGTTGTGAAGTTTTCAA
										2B-0426180-T9-43_896	GCCTTATATAAACAGTTATCACACATGGCCATGAGGTA
										2B-0426180-T9-43_960	TATTTGGCCATAGAAAGTGTATTCTACCTCACAACCTA
										2B-0426180-T9-43_1024	TATGAAAGTAATAAAGAAACATCAAACACAACAGTAA
										2B-0426180-T9-43_1088	TCATGAGAAGATATGAATTGCCCCCCTTTGATCTTTGT
										2B-0426180-T9-43_1152	CAGTGGCATATGCATGGGTTTTTTGAAATATCCAGCCA
										2B-0426180-T9-43_1216	CAAATATGCATCACCCGGCCCGTAGGATAGTTAGCAT
										2B-0426180-T9-43_1280	TATCCATTGCTAGCCTAAAGTAAAATGCAGAATATGTT
										2B-0426180-T9-43_1344	TCTAAACTAAACTGAAAGTTGAATCAGCATTAACTTTT
										2B-0426180-T9-43_1408	CCTATGAATATTTGATGAAGCAGCATCTGAATCGTTAG
										2B-0426180-T9-43_1472	AAATTGCATACATATTCATTCTCAATCGCTAGCACTTG
										2B-0426180-T9-43_1536	TTGATGAAGGAGCATCTCAATTGTAACAGACATATCT
										2B-0426180-T9-43_1580	GAAAGAAAAAATATGCGATGGTACTAATTGGACATGTT
										2D-0507440-T9-43_128	AGGAGAAGCATTCGGGAGGGAGGAGAATCTCGAGA
										2D-0507440-T9-43_192	TCACGTCACCTACAGATAGCTACAATGGACGTTAGTTT
										2D-0507440-T9-43_576	TCGGGCTTCCCATGGCTAGGACTACTTGCACACACTT
										2D-0507440-T9-43_640	TTTCAACCAGATTAAGTTTGTATGATTGGAGTCAGAG
										2D-0507440-T9-43_704	GGGTGTTAAAAGAAATAAAATATGCGGTCGTATTAAC
										2D-0507440-T9-43_768	AATCGTTGGCGTCAAATATGTGTTTATCTTATCAAGGT
										2D-0507440-T9-43_832	GCGGGGATGTGCACACAAATTACGAATTTAATTGTAT
										2D-0507440-T9-43_896	TATTAGCTGGATATATTGATTTGTCTAGATATGCATGCA
										2D-0507440-T9-43_960	GGTTCGTATTGATTGTCTCGTAAAAGCGCGTGGTTGG
										2D-0507440-T9-43_1024	GTCATTATAATTTTCGTTCGTTTTATCTTTAGAATTTAG
										2D-0507440-T9-43_1088	GGAACATAGTGTGTTGCGCTCATGGGTCAAGTTCGTT
										2D-0507440-T9-43_1152	TGTAACACTCTAACCAACATTGACTTATCGTGCAAAAA
										2D-0507440-T9-43_1216	TCTTCATTTCAATTTTCTATACATGTCTAACTTGGTGAT
										2D-0507440-T9-43_1280	TGTGAAGTTTTCAACAACAAGAATAGGTAAAAGTATG
										2D-0507440-T9-43_1344	TATGGTCATGAGGTATTTTCTTGTGAAAAATGTATGCC
										2D-0507440-T9-43_1408	CTGCCGCACAACCTAGTAGAATATACGCATATCACGTC
										2D-0507440-T9-43_1472	ATGAAAGTAATAAAGAAACATCAAACACAACAGTAAT
										2D-0507440-T9-43_1536	TGTGAGAAGATATGAATTGCCCCCCTTTGATCTGTGTC
										3A-0653240-T9-44_128	AGGAAGCAACGGGAGAGGGAGAGCAATATATGCGC
										3A-0653240-T9-44_384	CACCTAACTTATAAGTCGGATGATTTATTGAAATCAAA
										3A-0653240-T9-44_448	GGGGGAGATGCGCTGGAGAGAATGACACGGGCGGT
										3A-0653240-T9-44_512	AGAGTCCACACTCCACGGACTTGCCGAGCTCTTCTTT
										3A-0653240-T9-44_576	TACACTTCTCACTCTTCTGGCTTCAATGAGTAGCGCAT
										3A-0653240-T9-44_640	CGTCAGCTCTATTAAGATACAAGAATCCAAGAATGCA
										3A-0653240-T9-44_704	GACCGGTGGATTTTGCAGTGCATTCTGTTGACACTCG
										3A-0653240-T9-44_768	ATAACAGTGGAGGAAGCGTGGAAGGCAGTACGCGC
										3A-0653240-T9-44_832	CGTATTTAACAAGATGATGATTCTGTCCTTGTAGCGGA
										3A-0653240-T9-44_1024	ACCAATGCTCTGATGGGCTTGTATCTTTTTCTGGAAGA
										3A-0653240-T9-44_1088	ACACCTACCAACCAGCCGTATGAAGATCATGTAGTAA
										3A-0653240-T9-44_1152	AGTATATCTCACGTTGCTTTGGAAATTGCTTACAATATA
										3A-0653240-T9-44_1216	ATTTTGCGAGAAGAAAGATACTAAGATTAGACTGTGT
										3A-0653240-T9-44_1408	ttcatatattatatctaagatatCTCAAACCTATCTACATGAAA
										3A-0653240-T9-44_1472	TAGTTTCTAGTATATTTTCAAAATTACTTCATATTTGTAT
										3B-0738650-T9-44_122	AAACGGAAAGCTGATGACGCCGCCCCGGTGAAAGG
										3B-0738650-T9-44_183	AAAAAACTACGTGAATCACCACACCTTTCAGCCTGAG
										3B-0738650-T9-44_244	GGGCAATCACGGAAATGCCCCTCACCTACTGCCTGG
										3B-0738650-T9-44_427	GGTTTAGCTCACCGACTCCGATTAGATAGATGACCAC
										3B-0738650-T9-44_488	TTCTTAATAATCCtctaattctaatctaatctaatctaattaaAAC
										3B-0738650-T9-44_549	GGATTATAGTAAGTACTGTACTGGTTATTAAGAAGGA
										3B-0738650-T9-44_732	CGTCGTCAAACCAAAGCCTAAATGATCAGATTTGGTG
										3B-0738650-T9-44_915	TCCGCATTCTTATCCACCCTGGTTGAAATTGAAAGGA
										3B-0738650-T9-44_976	GCTAACGGACAGTAAAATCGAATTACAGGCTTGATAA
										3B-0738650-T9-44_1159	CAGCGCGAACTCCTTGGATTCCGTCATCCCTGCGGCA
										3B-0738650-T9-44_1891	GTCCCCGCGGAGCCCCATCGCGGGTGGCCGCCGCCC
										3B-0738650-T9-44_1952	GTCCCCAACGCACACGCGGCCTCACGAGGCAGTCCG
										3B-0738650-T9-44_2135	CAGAAGCACAGCAATAATAATTTACAAAAGTGACTGA
										3B-0738650-T9-44_2257	TTCACTTTTCGGGTTCATCTCGTTCATATGGCAGGCAA
										3B-0738650-T9-44_2318	ACTTGCGGAGCTCTTCTTTTTGAAATAGTAGTAGTAGA
										3B-0738650-T9-44_2379	CTCTTCTGGCTTCAATGAGTAGTGCATTATCTTTGAGG
										3B-0738650-T9-44_2440	CTATTAAGATAGAAGAATCCAGGAGTGCAAGACCACA
										3B-0738650-T9-44_2623	ATTAACAAGATGATTCTGTCCTTGTAGCGGAGTCAAC
										3B-0738650-T9-44_2684	AAAACTGTTGTTGTGATCTTTCCTAACGTTGATGATGT
										3B-0738650-T9-44_2745	TCGATGTTCTTACGTGTAATCGAAAGTAAGATATTAGA
										3B-0738650-T9-44_3050	ACCAGGACGAAGGTAGTATCTTTTTATGAAAGAAATT
										3B-0738650-T9-44_3111	ACCTACCAACCAGGCGTATGAAGTTCATGTAAGCAAT
										3B-0738650-T9-44_3355	CGGAAGGAGTATTTCTGTAAGATTAGAATGTGTGAAA
										3B-0738650-T9-44_3416	TGATAGCTGACATAAAAATGCATATAAGTACATTTTATT
										3B-0738650-T9-44_3477	ATTAATTTGTAAGATATTTTTACATAGAGTATTTACATT
										3B-0738650-T9-44_3512	CATTCCAGCCCACTTTGTGGTTGGAGGTGGAAGAGG
										3D-0884390-T9-44_0	GATGATCTGCCAAAGAAAGGGAACGCAACATCAGTA
										3D-0884390-T9-44_62	ACATCTTATACTTCTTTACGGAGGGAGTAACATTTTCC
										3D-0884390-T9-44_124	CCGCCCCAGTGAAAGGCACCCTGTACTGTACGCGCCT
										3D-0884390-T9-44_372	CAGGTGGGATGGGAATTGCCGAGTTGGTGAGACGC
										3D-0884390-T9-44_434	CTCCGATTAGATTGATGACCACAGCTTCTTACGTACGT
										3D-0884390-T9-44_496	TTCCTCTAATTCTAATCTAATCTAATTAAAACCACTCCT
										3D-0884390-T9-44_558	AAGAAAGAATTGTCCTTGGTCCTCTGGAAGCACGGA
										3D-0884390-T9-44_682	CAGCTCGGTCGTCGACCAAATATTTGAGCCGTCAAAC
										3D-0884390-T9-44_868	CCCTGATTGAAATTGAAAGCAAGCAAAATACTGTGAC
										3D-0884390-T9-44_930	GAATTACAGACTTGATAATAACTGTAATGGGAGATTG
										3D-0884390-T9-44_1116	CGTGTCGAACAGCTCCAGGGCGAACTCCTTGGACTC
										3D-0884390-T9-44_1178	CCGATGTTTGAATTCCTTGGATACAGTTCGAGCAGAG

3D-0884390-T9-44_2046	GCCGAGGAAGCAACGGGAGAGGGAGAGAGCAATAT
3D-0884390-T9-44_2108	CAGCGATAATAATTTACAAAAGTGACTGAAGTACTCC
3D-0884390-T9-44_2232	TCATCTCGTTCATATGGCGGGCAAGGGGATCGACAGT
3D-0884390-T9-44_2294	TGAGCTCTTCTTTTTGAAATAGTAGAAGAAGAGATTG
3D-0884390-T9-44_2418	GAATCCAAGAATGCAAGACCACAGTTTTCTAGCTTCA
3D-0884390-T9-44_2542	TGGAAGGCAGTACGCGCGCGTATTAGGGGTCTAGTT
3D-0884390-T9-44_2604	TCTGTCCTTGTAGCGGAGTCAACTGTTGTTGTGATTAT
3D-0884390-T9-44_2790	GGCTTGTTTCTTTTTCTGGAAGAAATTGCATCGTATTC
3D-0884390-T9-44_2852	GCCGTATGAAGATCATGTAAGTAATTAAGCATCAAAC
3D-0884390-T9-44_2914	GATGCGGAAATTGCTTACGATATACGTATTTCTGTAAG
3D-0884390-T9-44_2976	TTTTGATTAATGATATGATAGCTGACATAAAAGTGCAT
3D-0884390-T9-44_3100	CACTTTGTGGTTGGAGGTGGAAGAGGAGGAGATAC
3D-0884390-T9-44_3162	AAGATACAGGGTTGCTGGGCCTCTCCTATTCTATAGG
3D-0884390-T9-44_3224	ACCTGTGAAACCAAGAATCTGTGGAGGGGGGTCCCT
3D-0884390-T9-44_3348	TCACAACATCTGGATCATTCACAGTCGGTTCAATGTAC
7A-1784910-T9-45_64	GAACTATGGAACGAGATAAAGCTAACTAAATGGGACT
7A-1784910-T9-45_128	ATTCCTTTAAAAAAAAGGACCGTCTATACCAGCTGCCT
7A-1784910-T9-45_192	CCGTTGGATCACCATCCAACAACCACTATCCTCCCACA
7A-1784910-T9-45_256	TTTCATCTTCTTCCTCTGAAAAACCCTCGGCCTACCCC
7A-1784910-T9-45_448	CCTGCTTCTTCGCATCTTCCTGGGACGCGCAGCATCG
7A-1784910-T9-45_512	GCCTTTGAGCAAAGTAGAAATTTACCGACGCTTGAAA
7A-1784910-T9-45_576	AACGCGAAGAAACTGGCAGGCACAAATGATTTTCAT
7A-1784910-T9-45_640	ACTATGCTCGTTTTGTTTAAATGACCGGTACATACGTG
7A-1784910-T9-45_704	GTCCATGCATGCACGATGTCATGTCGCCGAACAGATA
7A-1784910-T9-45_768	GCCCTCTCAATAAACTAGTTGGAAGTTTTGCTTTTGG
7A-1784910-T9-45_832	CAGATGCCTGTCTTGGACAGACTAGAACAGTTTCTCT
7A-1784910-T9-45_896	AACAAGAACCAGCAAAGGTTTGGGGGAGCTCCTATT
7A-1784910-T9-45_960	GCGCGCCCGACTGGCCCGACCCATCACTCCGGGGGT
7A-1784910-T9-45_1024	ACTCAACCAGTGCAATAGCGGCTCGACATGTCCTGTA
7A-1784910-T9-45_1088	ATTTATATAAAAATATGAATATTTCTTAAATTAAAAAAAT
7A-1784910-T9-45_1152	ACAATAATTTGGATAAAGGCCATTTGTAAAACAATGC
7A-1784910-T9-45_1216	CTTTTTTAGGGGAAATTGCAGCCATTTATTATCTAAGC
7A-1784910-T9-45_1280	TACAAAGCGGATAAATAGTAAACAGAGTCCTACATAA
7A-1784910-T9-45_1344	TTTATGCGTGTCTTTTTCACATCGAGATCTTCAAAATT
7A-1784910-T9-45_1536	AAAAATATGTGTTTCCCTTTTGTGAGAGGCATGGTCG
7A-1784910-T9-45_1580	CGTGGAATGCATTTTTTCTCTTTTGCGAGAGGCCAGA
7B-1904800-T9-45_0	AGTGTACGTACGTGCCCGCGTACGTACGTCCAGTTGA
7B-1904800-T9-45_128	GACGTGGCCGTCTGCAGGTGTGCTGTCATCGGTGTG
7B-1904800-T9-45_256	AGCGATACATACCGTGGCTGCAAGGGATGCGTGGCT
7B-1904800-T9-45_320	TCAGGTAACTAAAAAAACCATCAAACAATTTTCTAGG
7B-1904800-T9-45_384	ATCCTCCCACACATGTCCATCTTCAAGGTTTCAACCAC
7B-1904800-T9-45_448	TCGGCCTACCCCGTTCCATCCACCTGTTACACGTCGCA
7B-1904800-T9-45_640	GCGCATCATCATTTCCATCTTGGATTAGGTCTTGGCTT
7B-1904800-T9-45_704	GACGCTCGGAAAAAATAAATTCAGGCAAACAGGCAT
7B-1904800-T9-45_768	GCAGGCACAAAAGACTTTCATGAAAGATCGACTCAC
7B-1904800-T9-45_832	TGTTTAAGTGACCGGTATATCCGGTTTGTACGTACATA
7B-1904800-T9-45_896	TTGCGTCCATGCATGCACGATGCCTGTCGCCAAACAG
7B-1904800-T9-45_960	AGTGCACTCTCAATAAACTAGTTGGAAATTTTGCTTTT
7B-1904800-T9-45_1024	ATGCACATGCCTGTCTTGGACAGACTAAAATGAAAAA
7B-1904800-T9-45_1088	GGGGAGCTCCTATTGGATGCACTCGCGTCAAACGATA
7B-1904800-T9-45_1152	GGGCCACCATCACTCCGGGCGTACTGTACTGATTCGA
7B-1904800-T9-45_1216	TATAGCGACTCAACATGTCCTGTAGCTTCATGACTTAA
7B-1904800-T9-45_1280	TGATTATTTATAAAATTTTCAACCTTTTAGATAAAAATA
7B-1904800-T9-45_1344	TTTCTTAAGAACAAAAACATTATTAAAGATTTAATATTT
7B-1904800-T9-45_1408	ATATTTATTGGAGAAGGCACACTTTTTTTATTTTGAGG
7B-1904800-T9-45_1472	CAAATTTTGAAACATTTTGTTGAGATTTGCTGATAATT
7B-1904800-T9-45_1536	AAGCTTATAAATTTTTTTTGAAATTCCTTAAAAAAATAA
7B-1904800-T9-45_1580	GGAAAGAAGAAAAGAAATACATGAAAAAACAAAAA
7D-1988810-T9-45_0	GAACAAACAGAGAACCTTACTTGAAAAATTAAGCCG
7D-1988810-T9-45_192	GTTAACTAATCGTGGCTTAAATACTCAGCAAAACATCC
7D-1988810-T9-45_256	CGTAGGCGGATCGAAAATGCGACGATCTTTTAGAGA
7D-1988810-T9-45_448	TCTGTCGAACCCTTGTTTGCTTATGGAGGCCAAGGCC
7D-1988810-T9-45_576	GAATGACTCTGATTCGGATAGTCTGACTGTGTACTACG
7D-1988810-T9-45_640	TGGATCTTGGAGTAGTGCTTTTTAAATACGGTAGACA
7D-1988810-T9-45_704	ACGCACCTCTATATACACATACATATGTACACTCTACCC
7D-1988810-T9-45_768	CCGGAGAATTGCATGGTTCTGGTTGAACAGAGTGCA
7D-1988810-T9-45_896	GGGCCTATTTGTGTGGTGCTAATATGTACGGTAACAA
7D-1988810-T9-45_960	TTATTTAACCTTGGTTGTAGTGGGATAAGGAGTGTCTT
7D-1988810-T9-45_1024	GCAAAAAAGTCTTATATTGTAGGATGGAGGGAGTACA
7D-1988810-T9-45_1088	CAAAGCAAGTCCAATTAACAAAGATTAAACCAAAAAT
7D-1988810-T9-45_1152	TTGTAATTTCATAAATACATGTCGTCAAAGCATGTATCG
7D-1988810-T9-45_1216	AAAATATTTTGAAGTTCCTATAAGCAAGGTAAAATAAT
7D-1988810-T9-45_1280	TTATATATTATTTTTACGTTCTTTTAGTAACATATACACA
7D-1988810-T9-45_1344	ATATTGGGCACCGCCAGAAATAAGATGTATTTACAGA
7D-1988810-T9-45_1408	GTTTTCTTACCTTGATAATTGGAAGACGGCCGTAGAA
7D-1988810-T9-45_1472	TCATAGTTTAGAAAGGAAAAAAGTATTTACTAATGGA
7D-1988810-T9-45_1536	ATGTATAATTTCATCATAATTGCTAAACAATTTTGGGAA
7D-1988810-T9-45_1580	TAATTTTGGGAAATTTGTAAGAATAGGTGCTCAACTTA
5A-1193820-T9-46_128	ATATATAGCGCGGGAGGGACGGGGCCGGGTTGGTGA
5A-1193820-T9-46_384	GTCTGGCGGGGCCGTGCCACACGTACGTACGTGTCG
5A-1193820-T9-46_448	CTGGTCTTCACGGTCGCGGCGAGCCACGGTCATGCAT
5A-1193820-T9-46_512	TTTTTCAAAAAAAAAAACTGTACTAGTACGGGTTACT
5A-1193820-T9-46_576	CTCCCTCCGTTTCAAATTACTCGTCACAGATACGGATG
5A-1193820-T9-46_640	AGGGAGTACGTAGTACGTACTTTGTTGGCCAGTCGAT
5A-1193820-T9-46_704	TTCTTCACATTTTTTCTGCGCGGGTAAAATATGTACAG
5A-1193820-T9-46_768	CACAATCAACAAATGTTGAGCTCAACCGCATTAGGAG
5A-1193820-T9-46_832	CACCTTCCGCACGAGCAAAAATAGCTGAACAATGGCT
5A-1193820-T9-46_896	AGTAATACTAGCTAGTTCTACGGAGTTTTGGGAGTGC
5A-1193820-T9-46_960	TTTGCCTGGTTCAAACGGTTGATGGAGAGACAGCATT
5A-1193820-T9-46_1024	CGACGTAGATGCCACGACCCACTTGCTTGGCCGGCC
5A-1193820-T9-46_1408	CATTCACGACCGCGCCCAACCGGCCAGCTAGCTAGC
5A-1193820-T9-46_1472	GATCTGATTCCCTGCCACTGCCTAGCTTGCTTGCATGA
5A-1193820-T9-46_1536	TTGTCGCGCAGGGAAATTATGTACTGTATGTGCGCTA
5B-1289280-T9-46_128	CATATATAGCGGCCgggttggtgatgggtggctggctggagtgg
5B-1289280-T9-46_384	GTGGGACCGCGCCGCACGGACGTGTCGCGCGCGGC
5B-1289280-T9-46_448	CATGCATGTCGCCCTCTTCGTACGTACGTACCATGCCC
5B-1289280-T9-46_512	TTCAAAAAAAAAAGTTTCAATACTAGTACGGGTTACC
5B-1289280-T9-46_576	TAGTACGTACTTTGTTGGCCAGTCGATCACCTTTTTCC
5B-1289280-T9-46_640	TGCATGCGAGGACGCGCACGCTGCATGTATACTATATA
5B-1289280-T9-46_704	GGGTATGGCTAGGTCTCGACTCTCAGCCGAGACTTAA
5B-1289280-T9-46_768	ATACGTACACACAGAAGATTCCGGAACCCACTTGCTT
5B-1289280-T9-46_832	GCAAACGACAACGCGAGAGCACGGTCCAATCCTGCC
5B-1289280-T9-46_1216	GATCGCATTCACGCCCGCGCGCCCAACCGGCCAGCTA
5B-1289280-T9-46_1280	TCTGATTCCCTGGAGCGAGTGGCTTGCTTGCATGATT
5B-1289280-T9-46_1344	TCGCGCAGGGAAATTATGTACTAGTATGCGCGTTACG
5B-1289280-T9-46_1408	TTGCTTCCTATGTAAAGTAATGATTTAAAAATTCTTATAT
5B-1289280-T9-46_1472	TTGCTTACCACATGTGTCATGTGGTAAAAATTCTTATAT
5B-1289280-T9-46_1536	TATATAAATTTTTAGGCCGCGAAATACATATGGTTCCTC
5B-1289280-T9-46_1580	GGGTAGTTTTATAGTCTGATATAAAAAAGAGTGCATTA
5D-1425820-T9-46_128	CCGTATATATACACCGGCCGTTGGTACTACGATACTAC
5D-1425820-T9-46_384	TGGCTGCCTCCGTGGTCACCACTAAACAAATCAGCTC
5D-1425820-T9-46_448	TTGTTCGATAGTTCGATGCAGCAGCTATCAATTGCACG
5D-1425820-T9-46_512	AATGCCAAGTCGAACATTTGCAGCTTCCACATGCACT
5D-1425820-T9-46_576	CGCCACAACTTGCGCACACCGATCATTATATACTCAGA
5D-1425820-T9-46_640	CAGTATACACTATTCAACACGTTAACCGTACGTATCGG
5D-1425820-T9-46_832	TGCACAGGAGATCCGGCCGGCGACGGGCAGGGGAC
5D-1425820-T9-46_896	ATGCATGTTCAACAATCTCTTCTCCCCCCGCCGGCCGC
5D-1425820-T9-46_1152	CCCATCACGGAGCACGAGCTTCGTGCTACGCGCGCCA
5D-1425820-T9-46_1472	CGTGCAGGGAATTGTGGGCGGGGCTAGGGGAATTA
6A-1517770-T9-47_256	GTGGAGGCGAACGATGCTGGCTGTTTCACAACCACC
6A-1517770-T9-47_320	AAGGTTGACGGACACACAAGACTGTTTAGATAGTTTT
6A-1517770-T9-47_1536	TAAGGTGCCCAAACACCGAAGAGTAATAAGTTCGACA
6D-1686320-T9-47_256	GTGGAGGCGAACGATGCTGGCTGCTTCACAACCACC
6D-1686320-T9-47_320	AAAATGTGGTTGACAGACACGTAAGACTGTTTAGATA
2A-0284740-T9-48_192	TATAGGTCAGAATATTAATTTTGTCCGGATTGGGAGG
2A-0284740-T9-48_256	GGGGAATCATGGCTCCGCCCCTCGAAAGGCCGGCAT
2A-0284740-T9-48_320	ggaaggggaagggagggaggtgaggACTGAGAAGAAGCGC
2A-0284740-T9-48_384	GGCGTATCATTGCCCTCTACTCTACTCTACTCTACTGCC

2A-0284740-T9-48_448	CTACTCTGCGTACGCCGTACCACCACCTCACCGCGCA
2A-0284740-T9-48_512	GGCGCCTCCTCCGCGCGTGCTCCCGGGCCTAGCACA
2A-0284740-T9-48_576	TATCAGAGGCAGAGCGCcccctcccctcccctcccctcccc
2A-0284740-T9-48_640	cgcctgctcgctcgcccccctcccctcctccTTGCTCTTCGTACT
2A-0284740-T9-48_704	GGGGGTCTTCTTCTCCTTCATCCCCGGAGTCATTGCT
2A-0284740-T9-48_768	AGGTTGCTAGGTACGTACGTACGTTCTTGGCTTCCTCT
2A-0284740-T9-48_832	TGCTTACCCGATCACCATTTTTAATCACCACTGCTTGCT
2A-0284740-T9-48_896	TTAGCTTACAGTGCCGCCGGCGGTTTCTGGCTCTGCT
2A-0284740-T9-48_960	TGCCCTTCTCGACACGGCCCGGGGTGAACTGACTCC
2A-0284740-T9-48_1024	TCCCTTCTTGTGAGACGCCTCGCCTGGGCACCGGACT
2A-0284740-T9-48_1088	agagagagagagagagagagagGGGCGTTCGTGCTACCCC
2A-0284740-T9-48_1152	GAGCTCACGGGACGGGGGGCGGCGGCCGAGCATTC
2A-0284740-T9-48_1344	GCCATGCGGACCCAGCCCGAGCTCTCTCTCTCTAGCT
2A-0284740-T9-48_1408	TTTTTTGGGGGCCTTCTTGTTCCCCTTTTGCCTACTTA
2A-0284740-T9-48_1472	CACACTGCACTGTTACTGATATGCATCCTCTGCTTCTCT
2A-0284740-T9-48_1536	GGCACTCTGCTTTCCGAGAAGCTCTCGCTGACTGACC
2A-0284740-T9-48_1580	AATGGCGAAGCACGGTTAAGCCGCTCCCTCAGCATTC
2B-0400900-T9-48_0	CGTGCGCCGGACGGCGGCTAGACGTGGTGAGGTAC
2B-0400900-T9-48_64	CGTGTGCCGCTAGCTCGGCCTCCCATCCATCTGCAAG
2B-0400900-T9-48_192	CCACGCCAGCCTCCCTCACCTCTGCTCAGCTGCCCGG
2B-0400900-T9-48_256	AAAAAGAGGCAAAGCAAAGCAAGGGCGAGAGAGA
2B-0400900-T9-48_320	tGGGGGTTTGGGTTGGGTTGGGGCACGGAGCGCCT
2B-0400900-T9-48_384	GATGGCTTCCGCCCTCGAAAGGTCGGCATCTTTATCC
2B-0400900-T9-48_448	GCGAGAAGAAGCAGCAAAAGGCGCACACCGTATGTA
2B-0400900-T9-48_512	TCTACTACTGCCCCGCCCAGGCCCATCCTACATCCTACT
2B-0400900-T9-48_576	ACCACCGCGTCCGCGGCCCGGGAGGTAGGGCTCCTC
2B-0400900-T9-48_640	TGCCTAGCACCAGTGCACCACACCCTCCACTCCACcgc
2B-0400900-T9-48_832	CAAGGTAGGTTCTCCACCTCTCCTTCTCCCCCCCATTT
2B-0400900-T9-48_896	ACCGGTTAGTGCTAGCTTCCAATGCCGCCGGCGACCT
2B-0400900-T9-48_1024	ACTCTTGTGAAATGCCTCACACCCCACGCCTCGCCTC
2B-0400900-T9-48_1152	GCCATGCTCCAGGCCGCCGCGAGCTCGCTTCCCAGG
2B-0400900-T9-48_1344	GGGCAGGGGCGGGTTTTCAGCCAGCCATGCGGACC
2B-0400900-T9-48_1408	TACTGCTACTAGCTCGCCATTCCGAACGCATTTTTTTG
2B-0400900-T9-48_1472	ctgcctgcttgcttgcttgcttgctCACACACACTGAACTGACA
2B-0400900-T9-48_1536	CGACGACCTGCAGGCACTCTGATCTCCGAGAATCTGT
2B-0400900-T9-48_1580	TGATTGATCAATGGCGAAGTCTGGCTATTAAGCTGCT
2D-0537490-T9-48_128	TGTGATCTGTGATGGACGTACAACGAAATGGAGTAGT
2D-0537490-T9-48_192	agcagtgcagtgcagCAGGTACGTTGGCGCGTGCGTACT
2D-0537490-T9-48_384	AAGCCAAAGGTGCTCGAATGAGGAATCAACGAAACC
2D-0537490-T9-48_448	GGGAGGGAGAAGAAGCGCGCGCAGCAAAAGGC
2D-0537490-T9-48_512	ACTGCCCACTCTACCGCCCCGCCCAGGCCCATcctacgc
2D-0537490-T9-48_576	taccgcctccacaccactacCACCACCGCGGCCGGGGCTCC
2D-0537490-T9-48_640	CCTGCCTAGCACGCCTCCACTCCACCGCTTTGCCTTG
2D-0537490-T9-48_832	GTGCCCGCCGGAGAAGGTAGGTTCTCGACCTCCTGC
2D-0537490-T9-48_896	TACTAGTGGGTAGTGGCTTCGAATGGAATGCCGCCGG
2D-0537490-T9-48_960	CAGTCGGAGGTGCGGCGCCAGCCCCAGCCGGCGGC
2D-0537490-T9-48_1024	CTGCACCTGCACCTGCATGGGCTTTCGTCCTCTTCTCC
2D-0537490-T9-48_1088	CCCTCGCCTGCTTCAGGGACTAGTTCTAGCGATCCCAT
2D-0537490-T9-48_1152	TTCGACCCCACGCCATGCTCGACCTCGCAGGAGAGG
2D-0537490-T9-48_1344	AGTGAGGGAGAGGCGGGTTTTTAGCCAGCCATAGCC
2D-0537490-T9-48_1408	AGCTTGCTACTACTAGCTCGCCATTCCGAACGCATTTT
2D-0537490-T9-48_1472	TACATAACCCTGCCCCGCCTGCTTGCTTGCTTGCTCAC
2D-0537490-T9-48_1536	TTGCGACGACCTGCAGGCACTCTGATTCCCGAGAATC
2D-0537490-T9-48_1580	GATTCATCAATGCCGAAGTCTGGCTGAGCTGCTCGTC
5A-1220530-T9-49_124	CCGTTTAACTAATCTATGTACGCCTGTGGCAACTTTAT
5A-1220530-T9-49_186	TATGGTGTATAATGAAAACGGCACCAACTTTAGCCAA
5A-1220530-T9-49_248	GATTATCCACAGCCTCAATCATCTATCTTCTACAGATTA
5A-1220530-T9-49_310	GAGAATGAAACTGCTAATAACGCTTCCTTTTTGCAGT
5A-1220530-T9-49_558	ATATTTTGCCCTACTTAAAGCGTTTGCCCATTTGCATGT
5A-1220530-T9-49_620	AGTATATAACGAATCCAAACTCGAGAAAACTAGTTTAT
5A-1220530-T9-49_682	GCACATGCTCGAGCTCTACATATGTCTAACTATGTCTAT
5A-1220530-T9-49_744	ACCAGAATGAAAAGAAGAAGGAAAGGAGATAATTAA
5A-1220530-T9-49_806	TAGAGAAAACTTACACATGTTCAAACTCTACATATGTC
5A-1220530-T9-49_868	ATCTTTTGCTAAACAACCAGAATGGAAAGAAAAGGA
5A-1220530-T9-49_930	GAAGCTAGAAAAATCAAATTAACAAAACCAACAACCT
5A-1220530-T9-49_1364	TTCTCTCTCCGTGTATGCAAGATGGCTTGTGATTAGGA
5A-1220530-T9-49_1426	GGAAGCAAGAACTGGGTGGGGGTGCGTATATATAGA
5A-1220530-T9-49_1488	AAGTGGTGAATGCTCTGCTGGTAGGGTGGTAGAGAG
5A-1220530-T9-49_1674	GTTAGCACGGTGTGGAGGTCTACCAACTTGGATCCAG
5A-1220530-T9-49_1860	CCAGCTATCCATTGCCACAGTGTTTAGCTCTTTGTGGC
5A-1220530-T9-49_1922	AAGGATTATTTTTGGATCCAGGATCAATTGCAAAATTG
5A-1220530-T9-49_1984	AAGGCTCAAATTAATACGAGTTGTTGCAGTTATCACAT
5A-1220530-T9-49_2046	AAGGTACTACTATTTCTTGCTTTGCTTGAACTTGTTTTT
5A-1220530-T9-49_2108	CGGGTGAAACCCTTTCATCTAATTTTTGTTGGTTGTGT
5A-1220530-T9-49_2170	TCTAGAGCATCAATGGGAGTGATGCTAAAGTGATGGC
5A-1220530-T9-49_2232	GGAAAATTACAATTTTGTGTCACTACCTGGTAGATAGA
5A-1220530-T9-49_2294	TGTCCGTTTTACAATTTTGCGTCACTACCTGGTAGATG
5A-1220530-T9-49_2356	CCTGTCCGTTTATATCTTTTATAAAGTTTTCTGTCGGCT
5A-1220530-T9-49_2418	TTGTAAAAAAAAGTTGTGCTGAAAGGAAATAAAGAA
5A-1220530-T9-49_2480	AGATAGATGATGAATCAAGTGGAACACATGAATTTATT
5A-1220530-T9-49_2542	AGACAAAGGACAAGTAACCAAAACAAGGGCCAAGA
5A-1220530-T9-49_2604	AACTTTCAAATCCTTGAGCTTTACCATCAATGTCATCA
5A-1220530-T9-49_2666	TAAAATTATTCTATTTTTTTAAATCATGGAGCAATGATG
5A-1220530-T9-49_2728	TTTAGCATGCCCCTCGAGGCGGTAACACGCCCACTAC
5A-1220530-T9-49_2790	TAACACAAACGGGCCATTTTAGGCATAACATGGCCGC
5A-1220530-T9-49_2837	TGTGTGAAATATGTGCGGTACCACGTTTACTTTGTGTG
5B-1334340-T9-49_128	AAGATGGCTTGTGATTAGGAGATGGTGGTGGTGGAA
5B-1334340-T9-49_192	GGTGCGTATATATagagggaagagaaagagagaggagagaga
5B-1334340-T9-49_384	GTCTGCTCAACGACTGATGCAAGCATAGGTGCTGCCG
5B-1334340-T9-49_448	CTACCAACTTGGGATCCAGTGGTATGAAATGTCAACA
5B-1334340-T9-49_640	CTCTTTGTGGCCTATGAGTTTTAGTCGATGCATTTAAG
5B-1334340-T9-49_704	CAAAATTGCATGAGTTCATGTCTCATGATTTGAAGGCT
5B-1334340-T9-49_768	TACGGAAACAACTCAGATTTATTATCAAAGTTCACGGA
5B-1334340-T9-49_896	ACATCGATATCCAGCGGACAGTGCCCAATGTATGCACA
5B-1334340-T9-49_960	TCTATGAGTCGTTCATATTTTATCGTGTGTATAGCGCAG
5B-1334340-T9-49_1024	TTTGTTTATTAAGGTACTACTATTTCTTGCTTTGCTTGA
5B-1334340-T9-49_1088	CTAAAATCGGGTGAAACCCTTTCATCTAATTTTTGTTG
5B-1334340-T9-49_1152	ATTTATCTAGAGCATCAATGAGAGTGATACTAAAGTGA
5B-1334340-T9-49_1216	TAAGGAAAATTATAATTTTGTCTCACTACCTGGTATATA
5B-1334340-T9-49_1280	CTATCCGTTTATATCTTTTATAAAGTTGTCGGTCGGCTG
5B-1334340-T9-49_1344	TGAAAGAAAAAAATTGCTGAAAGGAAATGAAGAAATT
5B-1334340-T9-49_1408	GATGATGAATCAAGTGGAACACATGAATTTATTTCTCA
5B-1334340-T9-49_1472	AGGCCAGGTAACCAAAACAGCGGTCAAGAAGAAAA
5B-1334340-T9-49_1536	ACTTTCAAATCCTTGAGCTTTACCATGAAGGTCATCAA
5B-1334340-T9-49_1580	AATTAGTTTGAGAACCCTAAAATTATTCTATTTATTAAT
5D-1454670-T9-49_128	ATGACTTGTGATTAGGAGATGGTGGTGGTGGAAGAG
5D-1454670-T9-49_192	GCGTATATATagagggaagagaaagagagaggagagaggagg
5D-1454670-T9-49_256	GCTGGTAGGGTGGTAGAGAGAGAGAGAAGGCTGTG
5D-1454670-T9-49_448	CCTACCAACTTGGATCCAGTGCTATAAAATGTCAACAG
5D-1454670-T9-49_640	CTCTTTGTGGCCTGTGAGTTTTAGTCGACGCATTTAAG
5D-1454670-T9-49_704	CAAAATTGCATGAGTTCATGTCTCACGATTTGAAGGC
5D-1454670-T9-49_768	TCACATATATTTTTCTTACTTAGTTTCTTAAGGTACTACT
5D-1454670-T9-49_832	TTTTTTCAGGCACTGATTATGAAAATCGGGTGAAACC
5D-1454670-T9-49_896	TGTGTGCTTGAACTTGTGTATTTATTTGTCTAGAGCAT
5D-1454670-T9-49_960	CAAGAAATAATTTATCCAGAAAATGAGGAAAATTACA
5D-1454670-T9-49_1024	GACATTGTTCCTTGGTATTTATACCCTGGCCGTTTATAT
5D-1454670-T9-49_1088	AATTCGGATCTTCTTAGACTATTTCTATGAAAAAAGAT
5D-1454670-T9-49_1152	TAAATGTGTGTTACTATCTGATAAATAAATAGATAGATG
5D-1454670-T9-49_1216	TATTTCTCAAAAAGACAACCAACATGTTTAGGCAAAG
5D-1454670-T9-49_1280	AGAAAAGAGATTGTGCACCATCTACAATTCACTCAAC
5D-1454670-T9-49_1344	TGTCATCAACAAAGGGATTAGTTCGAGAACCCTAAAA
5D-1454670-T9-49_1408	ATGATGATAACGGTTGGATATATGGCTTTTTTCAGCAT
5D-1454670-T9-49_1472	ACTTTTCACAACTAACTCTGCGCTAGACAACACGCAC
5A-1264760-T9-50_0	TTTGTCTCAGTCTCtctttcctcttgttcttttgcttttttttcttttctttt
5A-1264760-T9-50_64	TTTGGCTCAATCTCAGGAGCAATCTCTTATATAGGTAC
5A-1264760-T9-50_320	GCATATCTCTCTGGTCTCTGGAGCCCATTTGAGCTTGG
5A-1264760-T9-50_384	AGCCCATTTAAGCAGAATGGGACTTTCGGCTCTGGAG
5A-1264760-T9-50_448	TATCTTTGCTGCTTAGAAGGCCAGGTTAGATGAGCAG
5A-1264760-T9-50_512	ATGGAACCGTGCAGCCATATACTGTGTGGTACTGTGGT

										5A-1264760-T9-50_640	TTCAAAAAGCTACATTATGGGACGGAGGGAGTGGTT
										5A-1264760-T9-50_832	TACCAGGGAAGTACGATGCTAGGAAATGGATGTAACA
										5A-1264760-T9-50_896	GCAAAAGCTTTTCCTTTCATTAACTAAGAAGAAGATA
										5A-1264760-T9-50_960	TACATGATACTAGTAGCAAAAATCAGCTTTTAGTCATT
										5A-1264760-T9-50_1024	TACTAGAAAAAAGCAAATCACCTTTGATATTTATGATG
										5A-1264760-T9-50_1216	TTATATTAGTTTACAGAGGGAGTACCAGTAATTTGATC
										5A-1264760-T9-50_1408	TTCCTGGACCGCTTCAATGATATAGGTGGGTCCCCCA
										5A-1264760-T9-50_1472	AAGGGAAAAAAAAGGTGAGCCTCGGATAAGCACGCC
										5B-1388810-T9-50_0	TCTATCTTTGCTTTCGCTAAAAAAAAACTTTTATCTTTG
										5B-1388810-T9-50_64	GCAGTAGCCGTCTGGCTCTATTGCATTTTCCATGGCA
										5B-1388810-T9-50_128	ATTAGTCCCCATATATATGGAAACTATATGATTGGTTTC
										5B-1388810-T9-50_192	CTACGCTGGTGCAGTAGACACAGGGAAGTACGATGC
										5B-1388810-T9-50_256	CCTGTCAAAATGCACGTCCTGTTTTAAAAGTACTGAA
										5B-1388810-T9-50_320	AAATGATTACCAACATTTTTTTGGTGGGAGTTACCTAC
										5B-1388810-T9-50_384	AGTCACTGGTAACCCTTTTGCTAAAAAAAGCAAATCA
										5B-1388810-T9-50_448	CCAATACCGAGAAAACCTGAGAATCGTGATGTGTGAG
										5B-1388810-T9-50_576	TGGTTTGCACTTTTTTCTAGCTACATTGTCTACATGTTA
										5B-1388810-T9-50_640	GCCATATGGTCATTTTATTACCCACACTTTATATTGCAG
										5B-1388810-T9-50_704	CGAAGTTAGTCATGAACTAGAAAGACCCCGTAATTTG
										5B-1388810-T9-50_768	AAGTGACCGATCACTGCACGACGTCTAATCAAGTGGT
										5B-1388810-T9-50_1152	TATGCATGGATGGCTCAGATCTGGTTGTCGTCCCAAA
										5B-1388810-T9-50_1344	CACTCCAGTTACCTTGACGATTCAAAGCCGCGAGTGG
										5B-1388810-T9-50_1408	GTGGGCCCGCTAGTCAGGTGAGCCTAGGATAGAATG
										5B-1388810-T9-50_1472	AGAATGTCAAAAAAAAAGGTGAGCCTAGGATAAGCA
										5D-1473890-T9-50_128	AGTGTTCAATCTGCAGTCTGACTGAGTCGCTGTCTGA
										5D-1473890-T9-50_192	TACTCTTTTTTGGGGAATTTTTTATGTGTCTTTCGGGT
										5D-1473890-T9-50_256	AAACATACATTTTTTGGTATGTTTGTCTCAGTCTCTCTT
										5D-1473890-T9-50_384	CAGCTGAACTTAAAAGCCCCCAGCTGTTGGGAGGCC
										5D-1473890-T9-50_448	TCTCTCTCTGGTCTCTGGAGCCCATTTGAGCTCGGGA
										5D-1473890-T9-50_512	TTTGCTTTCGCTAAAAAAACAAATCTCTTATCTTTGCT
										5D-1473890-T9-50_576	AGTAGCCGTCTGGCTCTAATCCATCTTACATGGAACCG
										5D-1473890-T9-50_768	TTGCATGTTGCTCGTACTGTAAAGCTACAGGTTGCGA
										5D-1473890-T9-50_896	GCCTCCGTGCTAAACACTGCAATCTCGCTCTACCAGA
										5D-1473890-T9-50_960	ACCAACTTTTTTTAAACGGAGGCAAAAGCTTTTCCTT
										5D-1473890-T9-50_1024	GCTCGGTTACCTATACATTGATAATACATGATACTAGTA
										5D-1473890-T9-50_1088	TGCGTCTGTCACTGATAATCCTTTTACTAAAAAAGCAA
										5D-1473890-T9-50_1152	AGAAAAAACATTAGGCGATGCACCACGAAAACCTGA
										5D-1473890-T9-50_1408	TCCTGGACCGCTTCAATGATATAGGTGGGCCCGCTAG
										5D-1473890-T9-50_1472	AGAAGAAAAATGGCGAGCCTCGGATAAGCACGCCC
										2A-0353830-T9-51_640	TGACGTGGTGATGGATTCAGCGATCCAGTTAAACTTT
										2A-0353830-T9-51_704	ACCCATAGTCACGGCAGTACAAACAATAAAATAAAAG
										2A-0353830-T9-51_768	AATATAAGCACTGAAGCTAGTCAAATGAGTGCTGCCG
										2A-0353830-T9-51_832	ATCTTGTTGTGGCAACAGTGAGGACGTCATCATGTTA
										2A-0353830-T9-51_896	CAACCATCATCGATGAAGAGAAGCTCAGAAATATATA
										2A-0353830-T9-51_1152	AACCGAGACAAAGAGACACCTAAAACGGAGCAGGA
										2A-0353830-T9-51_1216	CTGCACCTCCATTGCATAAAGGCCACAAAAACTGAGA
										2A-0353830-T9-51_1280	AGGAATCTCTGATCATCCGAGTAGAAGCACATGCAAA
										2A-0353830-T9-51_1344	GTAGCAATCCATTTTATCCTTAGAGAAGCCCACTCAGT
										2A-0353830-T9-51_1408	ACTAGAGTGACCTGCAACTTTTTTACTTGAATTGGAA
										2A-0353830-T9-51_1472	CATAAACAAAAACATTACTCTCTCATGTACATATTAATT
										2A-0353830-T9-51_1536	CAAAACAATTCCCATAACGATGAAACTTTTCAAAGGC
										2A-0353830-T9-51_1580	TATACCTATTAATAAAGGAAGGACTATTTTATTCCCAG
										2B-0454840-T9-51_576	GCCGGACCGTGATTGGTTGGTGTAGGCAGGTAATTG
										2B-0454840-T9-51_640	TCGACGTGTTGATGCATTCAGCGTCCAGTTAAACTTT
										2B-0454840-T9-51_704	GACTCATCCATAGTCACGGCAGTACAAACAATAAAAT
										2B-0454840-T9-51_768	CGACGAATATAAGCATTGAAGCGAGTCAAAGGAGTG
										2B-0454840-T9-51_1088	AAGGAGAACCTCATTCCATCTTCAAGAAGTCGCTGAC
										2B-0454840-T9-51_1216	GTCCACCGCACCTCCATTGCATAAGGCCACAAAAAAT
										2B-0454840-T9-51_1344	GTGCTCTAGTAGCAATCCATTTTACCCTTGGGGAAGT
										2B-0454840-T9-51_1408	AATTCTTTTACTAGAGTGGCCTACAACTTTTTTGATTG
										2B-0454840-T9-51_1472	GTTTTGGATCATAAATAAATAAATTACTCTCTCCTATAC
										2B-0454840-T9-51_1536	AAGGGAATACAAAACAATTCTCATAACGATGAATTTTT
										2B-0454840-T9-51_1580	GCAACTTTCCATACCTATTAATAAAGGAATGACTATTTA
										2D-0593940-T9-51_576	GCAGGTAATTGACCGTGTACGCGCCAACCAGAGAGC
										2D-0593940-T9-51_640	CCAGTTAAACATAAAAAAAAAACTTTCAATGGATTCAT
										2D-0593940-T9-51_704	TAAAATAAAAGTAACAAGTCCATAGACCATTACCGACT
										2D-0593940-T9-51_768	GAGTACAGCCATCGTCATCCCTTACCGATGCCGGACA
										2D-0593940-T9-51_832	TCATCATCGATGAAGAGAAGCTCAAAAGTATCCAGCC
										2D-0593940-T9-51_1024	ACCTCATTCCATCTTCAAGAAGCCGTTGACGCAAGTA
										2D-0593940-T9-51_1088	AGACACCTAAAACGGAGTAGGACCCATCCCACTGCC
										2D-0593940-T9-51_1152	GCATAAGGCCACAAAAAATGAGACAAAATGTGGCAA
										2D-0593940-T9-51_1280	ATTTTACCATTGGGGAAGCCCACTCAGTGCACACATT
										2D-0593940-T9-51_1344	TACTTGAATTGAAAAAACACAGCAAATACGGTGTTTT
										2D-0593940-T9-51_1408	TATACATATTAATTGTCACTGATTTAGTACAAGGGAATA
										2D-0593940-T9-51_1472	TTTTCAGAGGCATCTTTCTATACCTATTAATAAAGGAA
										2D-0593940-T9-51_1536	GTTTTACAGAAAATCCCTTGATCTTTCTGATATTCAAC
										2D-0593940-T9-51_1580	TCCAGGCTTAAATCGGATTTTCGCTTTTGCAGAAAAA
										7A-1775070-T9-52_0	AGACGCCACGCTTGTTTGGGTAACAAAGCCAGATTG
										7A-1775070-T9-52_1024	CGCAATCATTTTTGAGATTAGTTTGTACACGACATTAT
										7A-1775070-T9-52_1088	ACCCTTTCCGGAGTATTGACCTTTGTTTAACAGTCGTT
										7A-1775070-T9-52_1152	CTTTGTTTAACAGTCGTGGCACACGCCCCCTCACACC
										7B-1908900-T9-52_960	AAACACAGACAAAATCACCGTGCATTTGGGTCGACC
										7B-1908900-T9-52_1024	TACTCTCGTGTTGTTACTAAAAAAATATACTGGATTTTT
										7B-1908900-T9-52_1088	CTGATGCAGCCTAATAAACATCCAAAACTGATTTCACC
										7B-1908900-T9-52_1580	TCGACGGGTCGGTCGGGATCGGGGTCAACCAAGAA
										7D-1985450-T9-52_1088	GCTTACCCTTTTGCCCTTGCCCTGATGCAGCACAATAA
										1A-0034150-T9-53_896	ATCATTGACTGATGGACAAGGTAAAACCAAGTTGCAG
										1A-0034150-T9-53_960	AAAAGAATTAGTAAAAGCAGGTATCAGGTGTGGATG
										1A-0034150-T9-53_1152	CGATTCCACTCCGGTTGGCTGATTGGATGGAACCATT
										1A-0034150-T9-53_1216	ACCTCCCCAGTACCTTTCTTCCCATTCCATCGCCATTG
										1A-0034150-T9-53_1280	AGGTAACCTTCCTTTACTCTCGTTCTACATTCCCAATC
										1A-0034150-T9-53_1344	ATGTAATCTACTGATTCATCATTTTCCTTTCCTTACCAA
										1A-0034150-T9-53_1472	TTGCGTCACCCAGGCTGCGTTTATTGCTTGTTATGATC
										1A-0034150-T9-53_1536	TGCTGTGCTTTCCAAATATGTCTTGGCCTACAGCGAGA
										1A-0034150-T9-53_1580	TCAATGTTTTCCTTTGTTTCGGCATATTATTCATCCTGC
										1B-0094860-T9-53_0	TGCGGGTGTTATCTTGTATTTCTGTTGGCACACCTAGT
										1B-0094860-T9-53_64	TTGACCCGAAAAATCAATGAAGAGCTAGATCACTTCC
										1B-0094860-T9-53_128	GGAGGACGAGGGTGTCCCAACAGTATACGAAGACCA
										1B-0094860-T9-53_320	ATCTTTTCTCAGAGTATCCGTCCGTTGGTTGCTGAAGT
										1B-0094860-T9-53_448	CGCGTACGTACGACAAATGCTCAAATCTGAAGCAAGT
										1B-0094860-T9-53_512	AAAAAAAAAGTCATGAGAGGCATGAAATTGCATGATT
										1B-0094860-T9-53_576	CTATTTATACATATCTTCTTTTCTACATATTTGTCCATGA
										1B-0094860-T9-53_832	TAGGTGAGAAAGACATCATTGACTGATGGACAAGGT
										1B-0094860-T9-53_896	AGTGAAAATTCAAAAAAAAAATAAGGATAGTAGTGG
										1B-0094860-T9-53_960	GCAGGTATCAGGTGTGGATGATTAAGTTATAACTAAG
										1B-0094860-T9-53_1152	GTTGGCTGATTGGATGGAACCATTCAAATACAGCCAT
										1B-0094860-T9-53_1280	TTTGCTCTTGCTCTACATTCCCAATTCGCCTTCCTGATT
										1B-0094860-T9-53_1344	GATTCATCGTTTTCCTTTCCTTACAAAGCAAAATACAC
										1B-0094860-T9-53_1536	CCAAATATGTCTTGGCAAAAATAAATCTACAGCGAGA
										1B-0094860-T9-53_1580	CAATGTTTTCCTTTGTTTCGGCATATTATTCATCCTGCA
										1D-0223780-T9-53_64	TACTGTTTCAAGCGGCAACCAAGCACGCCCCTAGTG
										1D-0223780-T9-53_128	ACCCGAAAAATCAATGAAGAGCTATATCACTTTCCCA
										1D-0223780-T9-53_192	AGACGAGGGTGTGCCCATATATTACGATTTGGGAAAT
										1D-0223780-T9-53_384	TCAGAGTATCCGTGCGTTGGTTGCTGAAGTGGCCTCG
										1D-0223780-T9-53_448	ACCCAAACCCGTGTGCAGGTGTATGGGCATGGGAGA
										1D-0223780-T9-53_512	GCTCAAGTCTGGCACAGGAAAAATAATGTAGACACC
										1D-0223780-T9-53_576	TTGCATGATTTGCATGTAGAGCGTATAAAAATGAATCT
										1D-0223780-T9-53_832	AGCAAAAAAGACAAAAACAAATTACACGGTCAGTGT
										1D-0223780-T9-53_896	TGACAAGGTAAAACCAAGTTGCAGTAATAAGGATGG
										1D-0223780-T9-53_960	AAAGCAGGTATCAGGTGTGGATGATTAAGTTATACTG
										1D-0223780-T9-53_1152	AGTTGGCTGATTGGATGGAACCATTCAAATACAGCCA
										1D-0223780-T9-53_1280	TTTGCTCTTGCTCTACATTCCCAATTCGCCTTCCTGATT
										1D-0223780-T9-53_1344	GATTCATCGTTTTCCTTTCCTTACAAAGCAAAATACTCT
										1D-0223780-T9-53_1536	CAAATATGTCTTGGCAAAAATAAACCTACAGCGAGAC
										1D-0223780-T9-53_1580	AATGTTTTCCTTTGTTTCGTCATATTATTCGTCCTGCAG
										7A-1818490-T9-54_0	GGAAGCTGCCGTGGGTGGGTTGGAAGGTGCAGTGG
										7A-1818490-T9-54_256	AACCCGAGTCGAGCAAACGCAGAGCTCACGGCCACG
										7A-1818490-T9-54_320	ACGGCCCCATCGATCTCAACTCTCAAGCAACCTAGAA
										7A-1818490-T9-54_896	ATACATACAGTCTTGGGTTTTACCTACGAGAGCTTGAA

										7A-1818490-T9-54_960	TTTTATAATTTGTTAAGTACTGTAATTGTTTTTTACGAA
										7A-1818490-T9-54_1024	TATATAACTGTGGTTGGAAGTTTATGAATATTGTTTGA
										7A-1818490-T9-54_1088	GTATCTCTGCATCTTTTTGCTACCCAAACACTGCTCTC
										7A-1818490-T9-54_1152	ATATGAATATTTTTTTTGGCCAAAAATAGCCTTGCGTG
										7A-1818490-T9-54_1216	CATATATGAATAGGGAAACACTTCTTCTAGATCGACTG
										7A-1818490-T9-54_1280	CTCGGACACAAAAATCTCTCTTCTACCTTTTTTCTCAC
										7A-1818490-T9-54_1344	GCGTGATGACGTGAACCAAAGGCCAATGCTAGGAAA
										7A-1818490-T9-54_1408	GTATTGTGAGTGTGATTTATATGGCTTATAGTAGTTGTT
										7A-1818490-T9-54_1472	AGTGAGACAGAAAATGTTGTACTCAATGCTCTGAAAT
										7A-1818490-T9-54_1536	ATTTGTTGCCTGCTCAAGTTCTATGTTTCAGTTTGCAG
										7A-1818490-T9-54_1580	GCTCATAGGCTGAGGTGAGCCGAAAATTAGAGATCTT
										7B-1937420-T9-54_0	CACTTCAAAGGATCACTATTAGGAGCCAAGTTCTTTA
										7B-1937420-T9-54_128	AGAACTTGAGCTGGTAACAAATTCAGCGCGTCATTTC
										7B-1937420-T9-54_192	TTCAGAGCATTCGATACAACAATTTTTGTCTCACCATAT
										7B-1937420-T9-54_384	AAGGTAGAAGAGAGATTTTTGTGTCCAAGGGGAGA
										7B-1937420-T9-54_448	TTCCCTATTCATATATGATAGGTTCTGTTCGATTAGTGC
										7B-1937420-T9-54_512	AAAAAGAATATTCATATCTTTTCTTCTCATTTAAGCATG
										7B-1937420-T9-54_576	AAAAAGATGAACGGATACAACATTGCAGCTTGCATGT
										7B-1937420-T9-54_640	TCCAACCACAGTTATATAGCCCCAAAACCATCACGTAA
										7B-1937420-T9-54_704	TAACAAAATACGTATTAAAACTATGGTTAAACCCTAGC
										7B-1937420-T9-54_1216	GTCTTCCCCGTCAGGGTCTTCACGAAGATCTGCATCTT
										7B-1937420-T9-54_1280	GGTGCGTCCTGGTTAAGTCGACGGACGGAGCTTCGC
										7B-1937420-T9-54_1344	CTGGGTTCTAGCTTGTTTGGCTGCTTCAGATCGGTGG
										7B-1937420-T9-54_1580	TTGGACGCGAGGCAGCCCAAGTTGAGGAATAGGCCT
										7D-2047690-T9-54_0	GGAGCTGCCGTGGTTGGAAGGGGCAGTGGAGGTGC
										7D-2047690-T9-54_256	CGAGTCGAGCAAACGCAGAGCTCACGGCCACGTACG
										7D-2047690-T9-54_320	CCCCATCGATCTCAAGCCAACCAACAAGCTAGAGCCT
										7D-2047690-T9-54_896	GCTTGCTACTAGATATATGCCTGGGTTTTACCTACGAG
										7D-2047690-T9-54_960	GCTGTAGTTTTAATATTTTGTTAAGTAATGGTTTTTTTG
										7D-2047690-T9-54_1024	GGGCTATGTAAACTGTGGTTGGAAGTTTATGAATATT
										7D-2047690-T9-54_1088	ATGCTGTATCTGTGCATCTTTTTGCTACCCAATCCACT
										7D-2047690-T9-54_1152	GCAAAGGTATGAATATTTTTTTGTCCAAAAATAGCCT
										7D-2047690-T9-54_1344	AAGGCCGATGTACTGAAACTGAAGATTCAGAACGAA
										7D-2047690-T9-54_1408	GTCCAGCATAATTGATGTTGATGTTTGCGCCCTTGATT
										7D-2047690-T9-54_1472	TGTATTGTGAGTGTGATTTATATGGTGAGACAAAAATT
										7D-2047690-T9-54_1536	CAAAGTCTGGACTTTGCTCTGAATTTGTTACCTGCTC
										7D-2047690-T9-54_1580	ATGTTTCAGTTTGCAGAGCTATGCATGCTCATAGGCT
										7B-1927230-T9-55_768	TTTAGCGCGACTGTTGAAGATGCTCTAAGTGGCGGCT
										7B-1927230-T9-55_832	CCCGCGAGCCCGTAAATCCGTCTCCTTTTGCCCTTGA
										7B-1927230-T9-55_960	GTACCGCTCGCTCGCTAGCGACGCACATGGTGGGAC
										7B-1927230-T9-55_1024	TCGTACCATGCATCGTCTTATGGTCCAGTGCCCCTAAT
										7B-1927230-T9-55_1088	TCCAAATGGCTTCCTTTGCTAGGATATTGGTTGGCTTC
										7B-1927230-T9-55_1216	ACCTTCTGAAACCATATCAAAACCCTCTTATAAAAGTG
										7B-1927230-T9-55_1280	CAAGCTCAAGTCTTTCTCGTCTTCAAGCAATTAAGAA
										7B-1927230-T9-55_1344	AATGAGTAAAGAGTTTTACTGGCTTGTGAAGATGTAA
										7B-1927230-T9-55_1408	CTTAATTAAGAGAATAGTTGCATTCGAATGATTGCACT
										7B-1927230-T9-55_1472	AGGTGCAGAGCAAGCGAAAATGCATTCTCTCGTGAAT
										7B-1927230-T9-55_1536	ATATGAACATAACCAAGGGGTTTGGGTAGAAGAAA
										7B-1927230-T9-55_1580	CTCAAGTGTCTAATTAAGACCCACTCGCACCGCCTAA
										7D-2067140-T9-55_128	CTTTATAATCGCGTGCTGCGTGCCAATTTTCAAATGGA
										1A-0007840-T9-56_64	TACTCTCGAAAAAACAATGTAACCGTAGGCGAAACAG
										1A-0007840-T9-56_128	TTTTTACTTACCTCTTGTCCTCTTTTAATTCTACTCCCTT
										1A-0007840-T9-56_192	AAAAGGGTTTGTCTAGGACACATCTAGATGTGTCGTA
										1A-0007840-T9-56_1024	TATATTGTGCTTTATCCAAACTGTAATTTAAATTTAGAG
										1A-0007840-T9-56_1152	gcggGTATTTTCTTTTTCTTTTTCTTTTTCAAACAGGCG
										1B-0112750-T9-56_0	CTGAAAAAAGGAAAAGGAATAGGGCAATATTAACAA
										1B-0112750-T9-56_64	ATGAATATCTTATACTTTTCAAAAATGTTGAAGTGGTAT
										1B-0112750-T9-56_128	AATTGATCTAACACAAATTCATGTGTAGCTGAATCTGT
										1B-0112750-T9-56_192	AATGACACAGCAGAAAACAACAGTACGCATGCCTGAT
										1B-0112750-T9-56_256	GAACATCGTAACTGAAATTGAAGAAAAAACCTGAAA
										1B-0112750-T9-56_320	ACACCAGTCTAAGATCCGTGGCAATTTGAGCATTGCA
										1B-0112750-T9-56_1024	GGACTATTCACCAACTGCTACATTAGAAGTAGAGATTT
										1B-0112750-T9-56_1088	TATGGTGGTGCCAGCGCTCTCTACTCCACCATGGCGT
										1B-0112750-T9-56_1152	TTTTCTTTTCTTTTTCTTTTTCGATTTCGAACAGGTGT
										1D-0205430-T9-56_1088	GATTCGTGCAAAAGGCGCTGGACGATGGACGATGGA
										1D-0205430-T9-56_1152	TTTTCGTAGACTTTTCGTTTCTTTTTCGAACAGGTGTG
										6A-1534140-T9-57_256	CGGCGGAGTCTTGCGCGGTGGCCCCTGGGAGATGT
										6A-1534140-T9-57_320	AAGAGAGGGATATGCGTTTGACTTCCTTTTTCTTTTG
										6A-1534140-T9-57_512	TCCCACATATGCAGGTAACCCATGGCCCAGGTGTCGG
										6A-1534140-T9-57_571	AGTCGATAACCAACATGGACATGGTCTTTTTTTCAGAT
										6A-1534140-T9-57_1266	AGAGAGGGATATGCGTTTGACTTCCTTTTTCTTTTGA
										6A-1534140-T9-57_1455	CCCCCCACATATGCAGGTAACCCATGGCCCAGGTGTC
										6A-1534140-T9-57_1518	TCGATAACCAACATGGACATGGTCTTTTTTTCAGATCG
										6B-1582630-T9-57_256	TCCAGTCGGAGGACTCTTGCGCGGTAGCCCCTGGGA
										6B-1582630-T9-57_320	GGAGGAATGAACGGTGGATGGATGCACGTTTGACTT
										6B-1582630-T9-57_384	ATTAGCTTAAAGATTGTTGTACTACACCATGTAGTACG
										6B-1582630-T9-57_576	GGCGGCCACCCGAGGCCCATCTCTCACAGAAGCAGC
										6B-1582630-T9-57_640	CTCTAGAAAAAAAACGTAGTCAATAATCAAGGGTGTG
										6B-1582630-T9-57_832	CCGTCGAACTCTACTACCGATTCGTCGACGATGCCGC
										6B-1582630-T9-57_896	TTTCGAACTTCCCCCTAGGGGATGAACGGATTACAAA
										6B-1582630-T9-57_960	TTGGAGGCAAGCAAACATTCATTAGCTTAAGATCATT
										6B-1582630-T9-57_1088	AGGCCCCTCCCACATACGCAGCTAGCCCATGACCCAG
										6B-1582630-T9-57_1152	GTAGTCGGTAACCAACGAGGACTTGGTCTTGTTTTCT
										6B-1582630-T9-57_1216	TCGTGATGATTTCAGATGATTTCTTTTCGCCCCGACTAT
										6B-1582630-T9-57_1408	TTGGGCTAGCGTGGCATTTGTTGAGCCATCCTTGTAA
										6B-1582630-T9-57_1472	GACGATGACGTATGCAAAATAAGTATGATGTGGATGT
										6B-1582630-T9-57_1536	GGACTCCTATTTAGATTTAGTAGATAATGGAAACTGAA
										6B-1582630-T9-57_1580	TGGGAGTAATGACTATAGTCAATGTATTAAATTTGGGA
										6D-1712310-T9-57_256	GTCGGCGGAGTCTTGCGCGGCAGCCCCTGGGAGATG
										6D-1712310-T9-57_320	GAATAAACAGATGGATGCACGTTTGACTTTCTTTTTC
										6D-1712310-T9-57_384	TTTGCACTACATCCATGTAGTACGAGTGTACTACTACT
										6D-1712310-T9-57_448	CTGATGGGAGGTGGCTGGAGAGCTTAAAGAGTGTTG
										6D-1712310-T9-57_512	ACTACTGTTCGAACTCTCTCTCAGGGCCGACGCTAAG
										6D-1712310-T9-57_704	ACTACTATTCGTACTCTCTCTCGGGGCCGGCTCTGAG
										6D-1712310-T9-57_768	CCCCTCTCACACAGAAGCAGCTAGCCCATGGCCCGC
										6D-1712310-T9-57_832	CGTAGTCGGTAATTAAGGGTGTGGTCTTGTTTTCTTTT
										6D-1712310-T9-57_1024	ACCGCTTCATCGACGATGTCGCACAGGTTGGAGGCC
										6D-1712310-T9-57_1088	CTAGGGGATGAACGCATTACAAATGGATGTATGTTTG
										6D-1712310-T9-57_1152	ACATTCATTAGCTTAAGATTGATGTACTACACACCTGA
										6D-1712310-T9-57_1280	CGCAGCTAGCCCATGACCCAGCCGCCGGTAGCCTTGT
										6D-1712310-T9-57_1344	GAGAGCTTGGTCTTGTTTTCTTTCAGATCGAGTTCAC
										6D-1712310-T9-57_1408	GGATTTCTTTTCGCCCCGACTATCAATCGCGGGTCAG
										6D-1712310-T9-57_1580	AGCAGTTGGGCTAGTGTGGCATTTGTTGAGCCATCCT
										1B-0090300-T9-58_0	CTCTGCTTCAAGATCAAGCCTCTCACGTGCTCGTGAT
										1B-0090300-T9-58_192	TGCATCTCACCTTTGCTTTCGTTGCTGAGCTAGGTGTG
										1B-0090300-T9-58_256	TCTCCTTCTGCCAAAAAATAGTCACTGTATCTCCTGTCT
										1B-0090300-T9-58_384	AAACAAAAAGGCTATTGGTTGAGCAGGCGGCCGGG
										1B-0090300-T9-58_512	gtttgaccaactttatagaaaaaatatatcaacattcacGAAATTA
										1B-0090300-T9-58_576	TGTAATATTGTAAATGTTGATTTTTTTTAAAATATAAAA
										1B-0090300-T9-58_640	TTCAAACAAATTTTATAAGAAACAGAGGGAGTATTTT
										1B-0090300-T9-58_704	ACCGCGGGCTCTATATAGTATTTTCTTCGATTGAGTTT
										1B-0090300-T9-58_768	TTGATTTAGCTAATAAAACATATATAATATGCCATAAAA
										1B-0090300-T9-58_832	TGACAAATCTATAGTCAAACGCCAACTTTATCCGGTAG
										1B-0090300-T9-58_896	TATAGTTAGCTATTGGCACTTACTACTTTACCATACATG
										1B-0090300-T9-58_960	TTTGTAGCTCGTGCTGCAGTGGGCTACAAATTTACTC
										1B-0090300-T9-58_1024	TCATATCTAATAAGTTGTGGGAAGAAAGGTTTTCCTCT
										1B-0090300-T9-58_1408	ATTTGGTCTTCATTCGTCGGCGTCCGTGTGTCTACATG
										1D-0198100-T9-58_0	AGTCAAAGCTCAGTTAGCTAAACCAAAAATGGGTGG
										1D-0198100-T9-58_64	TCGAATATGTGAACAAGCATAAGCATGTAGTAGTCAAT
										1D-0198100-T9-58_192	ATGCATGGATCAGCCAACAGTTAATTTTTTTTTCGCAT
										1D-0198100-T9-58_256	TCATCCATTAACCTAGCTTAGGTAATTAATTACGTCTAG
										1D-0198100-T9-58_576	TCTTTAAATCAATAAGTCCCTGGAAATAAACACCCTCA
										1D-0198100-T9-58_704	CATCAGGATCTCTTTTCTCCATGTGTAATTACCCCACG
										1D-0198100-T9-58_768	TAGTGGGTAGCTGAGATCCTTCCTTGAAAGCCGAACA
										1D-0198100-T9-58_1152	CCCTCAAACCATCGAACCACATGTTGGTTCGCACTGT
										1D-0198100-T9-58_1216	AACATGTTCGTCTAAAATATGAACCTTAAATGGCCTGA
										1D-0198100-T9-58_1280	ATCCTCCACTAAATTTGATAGACGGGCCAGCTGTCAA
										1D-0198100-T9-58_1344	CTTTGGTAGAGCACGACGGAAGGAACTCAGGGAGG

1D-0198100-T9-58_1536	ATGGCACGCCTGAGGacctccacagccaccaacaccaccacc
7A-1835500-T9-59_192	ccttccagatgcagaggccgggggtcatcctcctttctaaaaaaag
7A-1835500-T9-59_256	AAATCCTAATAATTTGAAACTATCCAATGAAAAAAGAG
7A-1835500-T9-59_320	ATATAATCCTTCTCCTGAACTTTCCAAAGCTCTTTTGCA
7A-1835500-T9-59_384	AAAAACATTTGAACTTAATTAAATATCACGTAATATAAA
7A-1835500-T9-59_448	GCACATACTTAAGCATAGTACTAAACATATACTTTTAAT
7A-1835500-T9-59_512	ATACATATACTCTCATTAATTCTTCTTCAACTCGATTCCG
7A-1835500-T9-59_576	GAAGTGTGCATGCTAGTCGTATTTCTTGTCAGTTTCAT
7A-1835500-T9-59_640	CTCGGCATTCTTGCCCTTATATGTAGTCTAGGCAACCT
7A-1835500-T9-59_704	CCAGCCCCTCCATGGGAAGGCAACATGTGGGAGCTAT
7A-1835500-T9-59_768	TCGAAGCAGCGGTTGCCGGGAGGGCTTCACGGAGA
7A-1835500-T9-59_832	CTCACAGAGAGGTGGTAGTTACAGGAAGGCGGCGA
7A-1835500-T9-59_896	AAGGTAAGGTGGGCTACGGATGGGGGTGACAACAA
7A-1835500-T9-59_1152	AGAGAGTATGTTTTGCCTAGTATTAATATGCAATACAA
7A-1835500-T9-59_1344	TAAAAAAATTAGCAGAAGAAATGCCCTTTTTTTAGAG
7A-1835500-T9-59_1408	GGACCGGAGCAAGCTCTCCGGCGCAAACCCAATCAA
7A-1835500-T9-59_1580	CACCGCAGCGACGCAAAGCGCGCAACCCCATCATCA
7B-1958510-T9-59_1152	CGGACATGCATCTCCTCCTCGGGGCCATGTAGGATAA
7B-1958510-T9-59_1280	ATATATGTTGTCGGATGGGTTAGTATGGGTCGAGTTTG
7B-1958510-T9-59_1344	CGGAACTATGTATCTGCTCCATATTTGGACTGTATGGG
7B-1958510-T9-59_1408	TTACCACGAGTTCCTAGGACCAGAGCAAACTCTCAGA
7B-1958510-T9-59_1580	TATGCGCGCCACTCACCCCGCAGCAACGCAAAGCGC
7D-2046950-T9-59_576	GGCAGAGGGtctttttcttttctttttgctttgtcttttctatttattttta
7D-2046950-T9-59_1216	CGTCCTAACCTCCCGTATCCACCCCATATTTGAACTGTA
7D-2046950-T9-59_1280	GCAGTTACCATGGGTAAAGTTTATAAAAAGAAAAACT
7D-2046950-T9-59_1536	CTCACCCCGCGCCGCAGCAACGCAAAGCGCGCAGCC
7D-2046950-T9-59_1580	TCATcacacacacacacaccaacaccacacCAACCGACCCC
4A-0998160-T9-60_0	TCACGAACGGGGCTTGAGCTCGGAGGGCGGGCGCT
4A-0998160-T9-60_192	CGATGTGACGCACGTGCAAGTTGGGGAGGGGAGGA
4A-0998160-T9-60_512	CGGCCCTTGGGATAATTGTACGGGTCGTCGAGATTCC
4A-0998160-T9-60_576	CTCGTAGGGTAAAAAAATATAAAAAATAGTGTTTCGTT
4A-0998160-T9-60_1152	GTTACTCCCACTATGACCAGCCTTAAGGTAGATTAAAA
4A-0998160-T9-60_1216	ATCTGTACTGTCACGTACTCCTAGGCGACTAAGTGCCC
4A-0998160-T9-60_1280	TTTTGGGCAAGTTTAATCTTTCTCCTTGTTTGTGCAAT
4A-0998160-T9-60_1344	TCAGCGACAAGATCGAAAGATATAAAAAAAAGTTAAA
4B-1078970-T9-60_491	GGACGGGGAGGGCAAGATGTGACGCACGTGCAAGT
4B-1078970-T9-60_751	GCCCAGGGTTAGGAAGGGAGGCATGTTGCGCGGGG
4B-1078970-T9-60_816	TTGTACGGGTCGTCGAGATTCCCATCGTCCACTTGGT
4B-1078970-T9-60_1011	TGTATTGTCACGTACTCCTAGGCGACTAAGTGCCCGG
4B-1078970-T9-60_1076	GGGCAAGTTTAATCTTTCTGCTTTTTTGTGCAATGGTA
4B-1078970-T9-60_1141	CCTCAGCGAGAAGATCGAAAGATATAAAGTAAATTAA
4B-1078970-T9-60_1206	CTGACGATAATCGAAAGTACTCCACAAAGCACGTCAA
4B-1078970-T9-60_1336	TCTACCCTGCCAGCCGCTCTGGTTGCCGATGGCGGAG
4B-1078970-T9-60_1401	GAGTAGTTAAATCAGTGTTTTTTTAGTCCCCGCACACG
4B-1078970-T9-60_1580	CGCGAGATTTGGTGTCATGTGATTCGGATCTATTTAAG
4D-1171560-T9-60_64	GGTTCGACTGATCGGCGGCGGCGGCGGCTCGAGCTT
4D-1171560-T9-60_448	CATGTTGCGCGGGGCCCACCGGATGCTTGCGGCCCT
4D-1171560-T9-60_512	CCCATAGTCCACTTGGTCCGTCTCTCGGCTCGTATAGG
4D-1171560-T9-60_640	TGGTCAGCTTTTAGGGTAGGTTAAAATCGTCTTCTGTT
4D-1171560-T9-60_704	ACCTACTCCTAGGCGACTAAGTGCCCGGTAGGTGTAG
4D-1171560-T9-60_768	TTGATCTTTCTCCTTTTTTTCTGCAATGGTAAATCTAAT
4D-1171560-T9-60_832	CGATAAGATATAAAAAATTTAACATTGTACTCCTTCCGT
4D-1171560-T9-60_896	AGCTTCGTCTCGAGTCAAACTTTTTAAACATCAACCAA
4D-1171560-T9-60_960	AAAATCTCTATTACTTAAAAAGAACTTGCATTGACATA
4D-1171560-T9-60_1152	TTAGTCCTCACAGACGCGGCGCTCGGGCAGATGACA
4D-1171560-T9-60_1344	TTAAGGGTCCAGCGACAACAACTGTGGCTCAAGGGC
4D-1171560-T9-60_1536	TGTACTTTTGATGAACTTTTATAACAAATGTAGACATTT
4D-1171560-T9-60_1580	AAAAAATATAACAAAAATCACATCGAGATCCTTAGATC
5A-1185940-T9-61_0	GCAGAAATTCGACAGTGCGGGGATGTTTTGTACGATT
5A-1185940-T9-61_62	CGTTGGCACACCGTGGATGAGAAACAATTAATACGAT
5A-1185940-T9-61_124	ATGGTTTTTTGGGTACTATGTTAAGTTGTGCTTGAAAT
5A-1185940-T9-61_186	TTCAATTTGGTACCACTGTTTGTACTATCCGGCCCGCC
5A-1185940-T9-61_248	ACTGTCTAGATGTGCCCTAAGTCTTATGTTTATTTTTAT
5A-1185940-T9-61_310	AAAAATCACACGCGGTAGATGCATTATAAGAAAAGGG
5A-1185940-T9-61_372	AATGACTCGTGCGTCCACTGCCTTGGTACTTGTCGCAT
5A-1185940-T9-61_496	TGTGTTGCGGGATTTGGATCCCCACCTCCCGTGCTCTT
5A-1185940-T9-61_558	CCTACATGTTACTCAGATCACCGTTGCCAGCAAACATT
5A-1185940-T9-61_744	GCACCGACGTCCATAGCCATCGCCTCCTTGAGTTTCA
5A-1185940-T9-61_806	TAGCTCTGTTGCATAGATTTTACGCAACTTGACCTTTG
5A-1185940-T9-61_868	CCGCAACACGACTTATGTCGCCAAATGTTTTCGCAAC
5A-1185940-T9-61_930	TGACACAATTTTGGTTCCGTAGGATATATCCCATAAAA
5B-1354040-T9-61_0	GAGATAGGGTCGAAGGAGGTTGGATGCGGTTGGTGT
5B-1354040-T9-61_63	ATGGCGGGGGCAGGTGGCGAGGTGGTAGCAGACGG
5B-1354040-T9-61_540	TGGCCAAGGTTGTTGGATGCTTGCGTGATAGCGGTCC
5B-1354040-T9-61_604	GTTTGCTTTTGATTTACGGCAAAAATAACGCCCGAAC
5B-1354040-T9-61_668	TTCGATGGCATAATGCACCGGTATTGTGCGGTACGGAT
5B-1354040-T9-61_732	TAAGAGATGCCCTAGGTTTATTGCAAATAATATTACTT
5B-1354040-T9-61_924	CAATTTTATCCATCTCTTTGATAAGTATTTCGGGACGG
5B-1354040-T9-61_988	TGTACTGCAGAGTGGAATCAGTAAAAGGTGTAATAGG
5B-1354040-T9-61_1052	TCTTTCCTAAAAGAGCAGAAAATCTTACGCGGTAGAT
5B-1354040-T9-61_1116	TCCCAATTTTGGTTTCGTAGGAGATCCCATAAAACAG
5B-1354040-T9-61_1564	CCTCCCCTATTTATCCCCTCCCCCATTcaccaccaccatcac
5D-1467920-T9-61_0	ATACGGCCGCAACTTCAATGCCGTATTTCAATGCCGAA
5D-1467920-T9-61_128	GCTCCGGGTCGGCATGAATGCGGCACTGAACGCTCT
5D-1467920-T9-61_192	GGAGGAAGGGGGATGGCTCGAGTGAGAAAAGGGTT
5D-1467920-T9-61_256	TAGCAGTGGTCCGGTCGCCTGCGAAGCCAGCTCCCC
5D-1467920-T9-61_320	TAACGGTCGAACCGCCAGACTGCGTTGGATGGCATAA
5D-1467920-T9-61_384	GTCCGCGTTAGAGATGCCGTAGGTTTATTTTGAAAAA
5D-1467920-T9-61_768	GTCTATACTGATGTTAAAAAAATTGCCCGATAAGTAAA
5D-1467920-T9-61_832	CCACATTTATTTTCTCACAATTTTGGTTTCGTACGATAT
5D-1467920-T9-61_1550	ATTTATcccctcccccatcccccattcaccaccaccatcaccacacca
2B-0448350-T9-65_64	AGATTAGCGCTAAGAAGCAACCCTAGTTTAAGTACTA
2B-0448350-T9-65_128	AGCAGACGTTCACCAGATAACATTTTCGATGAAAAAC
2B-0448350-T9-65_192	AAAAACATGATGCAAGACATCATGTTGGTTTCTTGCA
2B-0448350-T9-65_256	AGGAATTTCAAAGGAAAGTAGGATATCAGCAACTTAA
2B-0448350-T9-65_320	TTATAGGGATGACATGCTGTAAAATTATCGTTTCCCTT
2B-0448350-T9-65_384	GTTTATACACTCGTGTAACACCTTAGCTGAAAACCACC
2B-0448350-T9-65_448	cTTAGGTTCTAAATGTATACGTAGATAGCACATCGGTAT
2B-0448350-T9-65_512	ATATATCATATTGTCTATTCCGCTTTGTCCAAAAACGTT
2B-0448350-T9-65_576	TACAAGTGTAGCTGAATATCAATATACACAAAAATGAC
2B-0448350-T9-65_640	GGAGGCAACAATGTGGTAGTGTGGTACTAAGGGCAA
2B-0448350-T9-65_704	TCGCCATAATAACCGTTATTATGGATTTTTTGCTTTAAA
2B-0448350-T9-65_832	CTCGTCTTTGCTTAAAGCTTAACTCGCATTCTGAGATC
2B-0448350-T9-65_896	ATGATTATGGAGTGAACAAAGATTAGATATTTGTGTGT
2B-0448350-T9-65_960	GAAGCACAGTACATATGCAACATGGAGGCTGCCAATA
2B-0448350-T9-65_1024	TTGGCTACAGCTACTTCACTAGTGTAGGTAGCTCCTTC
2B-0448350-T9-65_1088	CGCGCTAACTCAATTATTATTTTTAATCGGGAATAACC
2B-0448350-T9-65_1152	GAAATACATCTATCCATAACGAGAGAACGGAGACCAA
2B-0448350-T9-65_1280	CAACAGTGACACCAAATTCAATCTGGTTTCGGTCAGT
2B-0448350-T9-65_1344	AAGGTTCCATAGCCCCTCGCGAGTCAAACATCACCGT
2B-0448350-T9-65_1536	GTTACCCCTCTCACTCGCAGCAACCGCACTAGCGCGC
2D-0595260-T9-65_0	GTTCGATGGGATCCCACCCCAGCGTATGTTCGCCAGA
2D-0595260-T9-65_64	GTGTAGACACAAAGAGAAAAAACATGAGGTTAGACA
2D-0595260-T9-65_128	TGGAATGCTCTATTCCATAGGAATTCCAAAGGAAGGT
2D-0595260-T9-65_192	GTAACAAAGGAAAATTACTTATAGGGATGACATACTGT
2D-0595260-T9-65_256	ACATAGCATATAGCTAACAGTTATACACTCGTGTAACA
2D-0595260-T9-65_320	GCCCCACCTTAGGTTCTAAGTGTCATATATATATCTTAC
2D-0595260-T9-65_384	ACTGTCCATTCCGCTTTGTCCAAAAACGTTTACCTTGA
2D-0595260-T9-65_448	AGCTGAATATACACAAAATGAAGATGCGGTACTAAGT
2D-0595260-T9-65_512	GTAGGGTGGTACTAAGGGCAACTCTAGCAGAGTCGT
2D-0595260-T9-65_576	TTATTATGGCTTTTTTGCTTTTAGAGGAGTGACCACCG
2D-0595260-T9-65_640	CTGCCCTCCATATCTGGCCTCCCAGTTGTCATACATGG
2D-0595260-T9-65_704	ACTTCATATGGCAGTTACATGTAGATTGCATGTGACAT
2D-0595260-T9-65_768	CTCATGTAGGATGGTAGTGAGGTTGGGAGATTAAGG
2D-0595260-T9-65_832	TCCACTCGTACCTCTTCTTTGTTTAAAGCTTAACTCACA
2D-0595260-T9-65_896	AATGTACCATTGACGACTGACTCGCGGTTATGGAGTG
2D-0595260-T9-65_960	CATAATCTCCATATCTATATGAAGCACAGTACATATCCA
2D-0595260-T9-65_1024	TCTACTAGAGTTGCTCTATGTTGACTACAGTTACTTCA
2D-0595260-T9-65_1088	GAACAAACTAGTGTCACACGCGCGCTTACTCAATTCTT

										2D-0595260-T9-65_1152	CAACTGAAATAACGGAAAAAATCTATCCATAACCAGA
										2D-0595260-T9-65_1216	TTCAACTCACCACGCGCTTAACTCAGTTCAACGCTCC
										2D-0595260-T9-65_1280	GCAAACCAACGGTGACACCAAATTCACTCTGGTTTCG
										7A-1787570-T9-66_448	GTGCGGGTGAGGctctctctctctctctctctctctctct
										7A-1787570-T9-66_512	ACGATGGGGTTCTGGTCTCCTCCCCTCTTTCCTTTTAA
										7A-1787570-T9-66_576	AAGTTTGACATGTGGGGTAGAGGTGTCAGTGGTAGG
										7A-1787570-T9-66_768	GTCTCGTCCATTTCTTTAGGGCATGTACAACAGAAATG
										7A-1787570-T9-66_832	CTTTTATGACGTGGAAAAAGAGGACGAAGAGAGAGA
										7B-1862220-T9-66_256	GGAAGACGATGGGGTTCTGGTCTCCTCTTTCCTTTTC
										7B-1862220-T9-66_320	GTTTGACATGTGGGGTCGGGAGATGTGGGGTAGAG
										7B-1862220-T9-66_512	CGTGACGGCGAATGGGGCGACGGTCTCGTCCAGTTC
										7B-1862220-T9-66_576	ATATTTTTTCCATGGCAATTTTTTTCGAAAAATCTATTA
										7B-1862220-T9-66_640	TTTTTTTAGATGAAAGCTCGTCACGGGTGGTCATGGT
										7B-1862220-T9-66_1024	CATGCCCTAACATGTCCCGCACATCTTCGTGTTCGCTA
										7B-1862220-T9-66_1344	CAACACTATTTTTTATAAGAGTAGAGAAACTTGAGGT
										7B-1862220-T9-66_1408	AAACCAAGTTTCTGAACCACGAGGCAAATGTGCGGT
										7B-1862220-T9-66_1472	TGCCCCTGCAAATTTCTCATTGCTAATGGACTATGGGG
										7B-1862220-T9-66_1536	CACTGTCCTAATCATTGTTAGCACGGACCATTTGCCAG
										7B-1862220-T9-66_1580	TTTTCTTTGCCAATGCTAGCAACCCCTATCACATATAGA
										7D-2007060-T9-66_256	CCGGCTTGGGGAAGACGATGGGGTTCTGGTCTCCTT
										7D-2007060-T9-66_320	GGGGAATCGAAGTTTGACATgtggggtcgggtggcgtgggg
										7D-2007060-T9-66_512	GTTCATTTCTTTATCCCCGGACCAAGAAAAAAATAAGA
										7D-2007060-T9-66_576	TTTTGCCGACCTCTAGGACCTCTTGAGTAAGATTCAC
										7D-2007060-T9-66_640	AAACTATCTTTTGCTGGAATTATTGTTTGAAACCTAAC
										7D-2007060-T9-66_704	AATGTTTGTATGACATCATATATTTTAAAAAAATTTAGA
										7D-2007060-T9-66_768	ATTTTTATAGAAAACGAAACCCATAGTACCAATTATTA
										7D-2007060-T9-66_832	CAACCCTACCACAATTCTAAAGAAAAGACAAAACACA
										7D-2007060-T9-66_896	GGTCTGTTTTCCTCATGACAGACACATACACGTGCCA
										7D-2007060-T9-66_960	AAACTAGCAGCTCCGGCACAAAGTCATCTTCATTTCC
										7D-2007060-T9-66_1088	CCGCATTCCAAAAAGACGCCACGCTTGTCACAAATTG
										7D-2007060-T9-66_1152	CTGCCACTGATGACAAAAGCTCGCAATTTTCCTCAGG
										7D-2007060-T9-66_1216	CCATTAAAACACAGATCGTTTCTGCACTTCCAGATAG
										7D-2007060-T9-66_1280	TATTGGCCTGGTTACGATTATCTTGAGCCCACATAGTA
										7D-2007060-T9-66_1344	ATGCCCACGCCTACCAATTAAACTGTAAATGATTTTCC
										7D-2007060-T9-66_1408	AAAAATGTATGGTTAATGGATTCCATCTCACAACAAAA
										7D-2007060-T9-66_1472	GCCTTTTACTCAGATTATCTCTAGTGAGCAGTTTGTTG
										7D-2007060-T9-66_1536	GATCCTAGAAGGGACATGCACTCTCCACATATCAGGG
										7D-2007060-T9-66_1580	CAGGCATAATCCCTCTAAAGTTAACAAAAGCATACATG
										1A-0021070-T9-67_64	ACTTGCCGGAGCCTCGCCCGGAAGCACGCACGTTCG
										1A-0021070-T9-67_128	CTTGCGCTCTGTGTATCGTCTACCTCGCTCCTCTTGGT
										1A-0021070-T9-67_256	ATAAATCCCACCAACCCAACGCCAGCGGCCTGCCTTG
										1A-0021070-T9-67_448	CCGAGCGGCCGAGGAAAGAGAGAGCCGACTGCGCA
										1A-0021070-T9-67_512	ACTTACGCATATACAAATCCGAGCTGGAATATTCAAAT
										1A-0021070-T9-67_640	CACGGGACGACGACCTGCTGGTGGCCTGTAGGCTGT
										1A-0021070-T9-67_832	GTCTTGATCGGTTCGCGGAGATTAGTTTATACGCCAG
										1A-0021070-T9-67_1088	TAAGATACTAATTTATAATAGTATGCATTACAGAAGTAG
										1A-0021070-T9-67_1152	CTGCTCATTACGACCAGCATGATGCAACAGGGACGAG
										1A-0021070-T9-67_1216	ATCTATCCATCCATTGTCCGTCCGGCAGTCCTCTGGCC
										1A-0021070-T9-67_1280	AGCCCGTGGATTGGCGATTTTTAATGCGAGCGTCGCG
										1A-0021070-T9-67_1344	GCGACTTTCTCTATCCAAGCCTCGGTCTCGGATCGATT
										1A-0021070-T9-67_1408	CTGGATCGAACGTGCGTCTTCTTCTACGTGTGCATGTA
										1A-0021070-T9-67_1580	CTCCTGGTCAGAGAACAACAGCCAGCCCGGCCTGGC
										1B-0088950-T9-67_0	GTTGACGCTGGATAGCCGCCTGCAAAAACAAACCAG
										1B-0088950-T9-67_62	ACACACTTGGCTGTTGAAGGAGCTGCGGGCCGGGG
										1B-0088950-T9-67_248	GCGACTGCCCCGGCAAAAAAACCACACACAACAAAC
										1B-0088950-T9-67_434	GCGTCGCCGCGTCCATTCTCTATCCACGGGCCAGTCC
										1B-0088950-T9-67_558	AGCGAGCAGAGCGCCGCCGCGCTTCTTGCCCTTTGC
										1B-0088950-T9-67_744	GCTTGCTTCTTCCCCGACGCAGCGCGGAGGAGGGAA
										1B-0088950-T9-67_930	GACTGCGCATTTCCACCATCTTTTTATGCATATTCAAAT
										1B-0088950-T9-67_992	CTCGGGACAAGAACAAAATTTAAATTCCCATCAGAAG
										1B-0088950-T9-67_1054	CCCATGGCCGTGGGACGACGACCTGCTGGTGGCCTG
										1B-0088950-T9-67_1240	TCGGCTTAAAAATCCAACGACCAGCCTTTGAGTGCGT
										1B-0088950-T9-67_1302	GCCGAGGAAAACATCCCCGGGTGGTTGACAATTGAA
										1B-0088950-T9-67_1364	GGAAGGGGCATGCATCATGATGCAACTGGGATGAGG
										1B-0088950-T9-67_1426	TGGATCGATCTATCTATCCATCGTCCGTCCGGCAGTCC
										1B-0088950-T9-67_1612	AGTTGAGGCCGGGCTCTGCAACTGGATCGAATGTGC
										1B-0088950-T9-67_1674	ATGTATTTCTCTCACCATCCAGGAGGCAGAGAGGCGT
										1B-0088950-T9-67_1922	CGCCTTCCGCAGCTCCAGCCTCCCGCCTCCTTGGCGG
										1B-0088950-T9-67_2030	ACGCAGGCAGGCACAGGCCACGGAGAATTAATCCTC
										1D-0221570-T9-67_0	TCTATCCACGCGCCAGTCCACGGAGGACGGGGCAAC
										1D-0221570-T9-67_128	GCTTTGCCTATCGTCTGCCTGCCTGGCTCCTCTGGTCG
										1D-0221570-T9-67_256	CAACCCAACGCCAGCAGCAGCCTGCTTGCTTCTTCCC
										1D-0221570-T9-67_448	GGAAAGAGAGACGGAGCCGACCGCGCATTTCCACC
										1D-0221570-T9-67_512	ATATACAAATCCGAGCTGGAATATTCAAATCCTCGACG
										1D-0221570-T9-67_640	CGACGACCTGCTGGTGGCCTGTAGCCATCGCCGCCG
										1D-0221570-T9-67_832	TTTGATTGCGTCGTCTTCGGTTCGCGGAGATTAGTTTA
										1D-0221570-T9-67_896	GTTGACAATTAAAAGGGCATGCATCTGATGCAACTGG
										1D-0221570-T9-67_960	GGTGGCTGAATGGATCTATCTATCCATcgtccgtccgtccgt
										1D-0221570-T9-67_1024	GTTTATGTTTGTTGTTTAGCTCGTGGGTTGCCGATTTT
										1D-0221570-T9-67_1088	GTGGCTGATGGCGACGGCGACGGCGACTTTATGTAT
										1D-0221570-T9-67_1152	CAGTTGAGGCCGGGCACTGCAACTGGATCGAATGTG
										1D-0221570-T9-67_1216	TATCATCACATCGCAGAGGCAGAGGCCAGAGAGGCG
										1D-0221570-T9-67_1344	GAGAATAACAGCCAGCCTAGCCTGCACGCGTGCTCC
										1D-0221570-T9-67_1408	CCCCGTCGAGGCCTCGCTCTCTCCCCTAGTGGCTAGC
										1D-0221570-T9-67_1580	CACGGGGGCACGTCGGCCCTCGTCACCACGGACGCA
										2A-0272020-T9-68_0	AACTTACATATGGTTTGAGCGATAATTAATATGAGACA
										2A-0272020-T9-68_64	GGCAGTTAATGTGAGACGGAGAAAGTACTACCAAAG
										2A-0272020-T9-68_128	CGGTTATTGCTCCTATGACAAAGATAGAAAAGTCGGT
										2A-0272020-T9-68_192	TCTCATCACAAAACCAATACGCACCAGAAAAACAGTC
										2A-0272020-T9-68_256	TCGTGCCTACGTTTTCTCTTGCGGAAAACAGAAAGAT
										2A-0272020-T9-68_448	CAGTACAGCAGCATCCAGAATCCAAATCTGGCGGTAC
										2A-0272020-T9-68_512	CTGAACCCCTCTACCATCCACCGCCGCTGCCTACGAG
										2A-0272020-T9-68_576	ACGTCCGTCCGGTCGATATCGTCGTCGATCCCCGCAC
										2A-0272020-T9-68_640	GCGGACGCGTGGACGGGCGTACGGCCGGGTCGCTC
										2A-0272020-T9-68_832	CTGCcatgcacgcacgcacgcacggcacgacgTTGGTGGTGC
										2A-0272020-T9-68_960	GGAGGAGGGAGGGGCTCCTCGTATCGAAGGGGCAA
										2A-0272020-T9-68_1152	TGCCGCCCGGAAAGACCAAATCAAATCCTCAGTTGCT
										2A-0272020-T9-68_1216	AACTAAAAAAAAAGTGATGCAGTTTGTGCGTGCCCCA
										2A-0272020-T9-68_1280	GCCCACACCCCAACGGAAGGAGAGAAAAGAAAAAG
										2A-0272020-T9-68_1580	CCGCGCGTCGCGGCCAACCCAACCAACACCCAAACG
										6A-1504830-T9-69_0	AAAAGTCATATTTCTGATTACATCAATTGATATTTGTAA
										6A-1504830-T9-69_64	TAACAAGCACCTAAAAAGGCAGCATGAGTGATATTAG
										6A-1504830-T9-69_128	CTCACGTCTAGTGTCGTCAAAGAGCACATGGAGGTGT
										6A-1504830-T9-69_320	CGCTCGTCATGTATCTTAACACGACGACTTTTCAATCG
										6A-1504830-T9-69_384	CCTATGACGGAAAGACGATGACGACGATGGCCTCCG
										6A-1504830-T9-69_448	TGGTGATTCTATGGACTTGAATGTAGTTTCTGTTAATT
										6A-1504830-T9-69_512	CAGATGATGCATAGATTAAAATTTTCAAAGAAAAAAC
										6A-1504830-T9-69_768	CCTATGTTAAGCACCTTGCATTGTACAAAGCCTTACAA
										6A-1504830-T9-69_832	AAGAACAAGACGTTAGAACAACGCAGAGTAGCTGCA
										6A-1504830-T9-69_960	GTACTCGATTTGGGATCTAATGACCGGCACGATCATC
										6A-1504830-T9-69_1024	AAAAATTCCCACAGTATAATAATCCCAGCAAATCCAGG
										6A-1504830-T9-69_1088	TAGCATCACGAGACTGTCGACCGGGCGGACACACGT
										6A-1504830-T9-69_1280	GCGCCGTGGACCGCGCCTTTATTTATACCGGTGCCGT
										6A-1504830-T9-69_1536	ctgactgattgattgaAGGGGTGTTGAGGCCGTGGCAGG
										6B-1600320-T9-69_384	ACATGTATGATATCATAATTTCATACTTAATATGTATATTT
										6B-1600320-T9-69_448	AACATAAATAAAAAATACTCCTAAAAGGTCACATTTCT
										6B-1600320-T9-69_512	AATAAATAAAATGGATAAAACAATTAAGTAGCACTTAA
										6B-1600320-T9-69_576	CATTGAGTCATCCATGACTAACAGCTCTCACGTCTAGC
										6B-1600320-T9-69_640	TGTTTGATATGACTAATGTACCAAGAACAAAAAGGCG
										6B-1600320-T9-69_704	CAATGTTTGTGATTGTCGTGCGGTGATGAATAGATAGA
										6B-1600320-T9-69_768	CTAATAACGGTTGGGCTTAGTATTGAGTTAGGAGCAT
										6B-1600320-T9-69_832	CCAACCAAAGAACAAACGTTAGAACAATGCAGAGTA
										6B-1600320-T9-69_960	ACAACAGTACTTGATTTGGGATCTAATGACCAGCACG
										6B-1600320-T9-69_1024	TACTGCTACAAATCTTATAATAAACCCCAGCAAATCCA
										6B-1600320-T9-69_1088	TGTAGCATCACGAGACTGTCGACCGGGGCGGGGCGG
										6B-1600320-T9-69_1280	GCCCGTCACCGTCACGCACCAGCGGCGTGGACCGCG
										6B-1600320-T9-69_1344	AGCAACCGGCTGTTACACACGTAGTGGTAACAGAAGA
										6D-1702590-T9-69_128	GTTACAGAGGGGGTACTTGATATGTGTATATTTGATAT
										6D-1702590-T9-69_192	ATAAAAGATACTCTAAAAAAGCCACATTCCTAATTACA

6D-1702590-T9-69_256	AAAATCGATAAAACAATTAACAAGCACTTAAAAAGGA
6D-1702590-T9-69_320	CATTCATGACTAACGGATCTCACGTTTAGTGTCGTCAG
6D-1702590-T9-69_448	CTTTGTTTCTCTGTGTTCGTGTGTCTACATGTTGGATT
6D-1702590-T9-69_640	TTGTGATTGTCGCTAGGTGGTTCTATGAACCTGAATGT
6D-1702590-T9-69_704	GTACTGCCTCGAGAGGCGATGAATATACCAATTTTTTT
6D-1702590-T9-69_768	ACGGTTGGGCTTATATTGATTAGGATCATCTTACAGCT
6D-1702590-T9-69_832	GAACAATACGTTAGAACAATGCAGAGTAGCTGCAACA
6D-1702590-T9-69_960	ACAACAGTACCTGATTTGGGATCTAATGACCAGCACG
6D-1702590-T9-69_1024	TACTACTGCAAATCTTATAATAAACCCCAGCAAATCCA
6D-1702590-T9-69_1088	TCTGTAGCATCACGAGACTGTCGACCGGACGGACACA
6D-1702590-T9-69_1344	TTACACACGTAGTAGTAACAGAAGATACGGATTGGGA
6D-1702590-T9-69_1536	CCGTGCGACCGCCATTGATTGGCTCACTGATTGATTG
3A-0621140-T9-70_0	GCATGTAATAATTTTCATATTCAATAAAAATATCAAAGT
3A-0621140-T9-70_64	ataaatgataatatatatttataataaaatagttatgtattataAAAG
3A-0621140-T9-70_128	GCAGTTTGTTTTGTTTATAATGCACAATTATAATTTGCA
3A-0621140-T9-70_192	TAACAGTGCAAAATGGTCAGTAGGGTGTCGAGCCTG
3A-0621140-T9-70_320	ATTTTGACCCTGAAATCTTCAAAACCGGACAATTTTC
3A-0621140-T9-70_384	TAGTTTTAGGAGAAGATGTCGTCTTCCTCTTTCGCTCT
3A-0621140-T9-70_448	ATGGTCGTCGATGTTCCCCACCAAGGTGCTACATAGT
3A-0621140-T9-70_640	TTCAAGCGTCTCCGCCACGCCGCCCTCGCCTACTCTG
3A-0621140-T9-70_768	GCTCTGAGGATCCCCCAACGCCTCTGACGCCTCCCCT
3A-0621140-T9-70_832	CTGAAGCCTCCCCAACAAGCTCAGCGACCCTGAGTTT
3A-0621140-T9-70_896	GTTGCCGCAGCCATCATTGGCACCGCTGTCGTCCTTCT
3A-0621140-T9-70_1088	ATCCAATGAAAACAATGCCACGTCATCCCCGAAAACC
3A-0621140-T9-70_1152	GGGCAAATTTGTTCGGTTTTGAAGATTTCAGGGTCAA
3A-0621140-T9-70_1216	AAATTCATCCATTGAGGCACATTTTTGGGTGAAAATG
3A-0621140-T9-70_1408	GAGAAAAAATAATTTTGATGGGATTCACAAAATAACA
3A-0621140-T9-70_1472	ATGGCTAACAGTGGGTCCCACGTTATATGACGCATCTA
3B-0821790-T9-70_960	tccaggactctctcaCTCTTCAAGGGAAAACCAATGCAGT
3B-0821790-T9-70_1024	GAGAAACGACGTCGAGCTCGGTGACCACAAGCAAC
3B-0821790-T9-70_1088	AATACATCATTATGTAGCCAATACACCGATCAAACAAG
3B-0821790-T9-70_1152	AACAATACCCAGATCCACCATCTTCAAAATCACCATCT
3B-0821790-T9-70_1216	ATACATTCCACGAGCAACAAAAAGAACAAAAGTCGG
3B-0821790-T9-70_1280	ATGACTCGGATCTAACCGCTCATTAGTGGGTCTCACG
3B-0821790-T9-70_1344	AGAAATCAGACACATATGCCCGCCCCTAAAACAATCG
3B-0821790-T9-70_1408	AACCAAGCATTCCACATTCATCATCTGAATTACCCAAA
3B-0821790-T9-70_1472	AGTTGGGAAATATTCCACAAAAAATACCAAGATAGTT
3D-0886810-T9-70_0	TAGTAATGGTAGGGTAGGTATGATTTTATTTCAAGTTT
3D-0886810-T9-70_64	ATGTGGTTATATAATGATATATTTAGGATGGGCCTTCAG
3D-0886810-T9-70_128	CTGAAAATTTATTTGTATTAATTCCCTTGTTTCCATTTT
3D-0886810-T9-70_192	GGTGTTATGCTTTCAAGGTCCATAATCCTTGTGATGTT
3D-0886810-T9-70_256	CGGTTGTCATTCCCATGTCTGTGCCATTCACGTTTGTA
3D-0886810-T9-70_320	ATTTACTCGGTGGTTACTAGAGAACATATTTCATGTGC
3D-0886810-T9-70_768	ACAATAACTACGGGGTTGGTCAAATATTGAAAGAAAT
3D-0886810-T9-70_832	ACGCATAGGAAGCTGTTGCTGATAATCCAATCTTTAAT
3D-0886810-T9-70_896	GTTATCAATGAAGAAAATGTTGTTTCAAGTGACTGAA
3D-0886810-T9-70_960	TGTGGCGTGTGAAATATTGAACGTCAAGACGACCAG
3D-0886810-T9-70_1472	ACATCCCTTGCATTTGATAATGAAACAATCAGAATTTG
4A-0975020-T9-71_0	TCTTACTGTGGCTGGTTTTCCTTGAACAATGTTTTCAT
4A-0975020-T9-71_64	ACCTTTGTATCTTAGTCCTGCGCCAGATTTTTATGCTC
4A-0975020-T9-71_128	GTCCTTCGGTGTGATATTCCAGTCTGTGATTATTATGCA
4A-0975020-T9-71_320	CACCTGAACCCACACCTGCACCAGAGGACAAGTAAA
4A-0975020-T9-71_448	GCCCTTGCTCCTGGGCAGTAAAGCAGGGATGACTACT
4A-0975020-T9-71_512	TGTTACTTAAGTGCGTTCCTTGTGAAAGGAAATTAATT
4A-0975020-T9-71_576	AGATTGTTTGGCCAACTTTTGCTGAATGCTCATTAATT
4A-0975020-T9-71_960	CATGGATGAGGTTAGCAAGTTTTAGTCCCACTACTTTT
4A-0975020-T9-71_1024	TTTGTGCTTCCCTAGGTTCACGCCAAGCTTCGCTTCCT
4A-0975020-T9-71_1088	CTTGAAACTGAATTTCAAACTTTCAATAGGATACTTTG
4A-0975020-T9-71_1152	CGTCTTCTCTGCCACCGCTCTGACCGGCTGGGGTCTA
4A-0975020-T9-71_1216	TCCACCTCCCAGTGATAGAAGGTAGGGCAATTTCTTT
4A-0975020-T9-71_1280	ATGATCGAGCAGGTCTCGGGAAAATGAAATGGGCCA
4B-1092060-T9-71_320	TGCTTGGTGTTTTCTTTTTGGGGGAGCTGGCCGGCC
4B-1092060-T9-71_768	CGTGGACAGACCGGGGGGTCAGCAATAGCATGGAG
4B-1092060-T9-71_832	TCAGGACTTAGGGCGCTGGAATAACAGGGACTTTTG
4B-1092060-T9-71_896	GAAAACAAAACTTGAGAAGTTACGCGCTGACCCAAG
4B-1092060-T9-71_1088	GAGAAAAAAGAACATGATAAAGGCATTACAAAATCC
4B-1092060-T9-71_1152	GAGCTGAAATCATTGGTACAGGAGTTCTATGAAACTT
4B-1092060-T9-71_1216	TGGAAGCGGTCCTTAATTGTGTGCCTaccaaagttactga
3B-0725620-T9-72_64	CACAACAGCTGGTGTCCCTATCGTGTTCAGCGAAATG
3B-0725620-T9-72_128	TTTTTACATTGATTTTCATTGGATTTTACATTTGGCAAT
3B-0725620-T9-72_192	TATTCAAATTGGATGTCTATATTGCAAAATCGTTTTTG
3B-0725620-T9-72_256	ATTATTTATCACAAGATTTTGCATCTGAAGAAATTCCCT
3B-0725620-T9-72_320	TAATCTTCTTGACATGGACTAGGAGGGCTTTGGTTAT
3B-0725620-T9-72_384	TACACTACCAAAGAGAGAGAGAAAAAATCCTATGATG
3B-0725620-T9-72_448	TCATCGGTATTGAGGAACCAATGTCAAGTTATGGCACT
3B-0725620-T9-72_512	GGCATCTATCCGTTGGATATTTGTACCGACATCAATG
3B-0725620-T9-72_640	CATCCATTATTTCTTCATCTCTTGTATGGATTGGTCGCT
3B-0725620-T9-72_704	CTCAACATTGCTCAATAATTGTTGAAGAGCCAAGGAA
3B-0725620-T9-72_768	GTAAGTGATTCAATTTGCACCACTGCTGGACAGAGAG
3B-0725620-T9-72_832	CAAATACAATGAAATTCCAAATAATAAGAATTCAAAGA
3B-0725620-T9-72_896	GACACCAATAATGATGCATCAAGTGCTATGGATGAAAT
3B-0725620-T9-72_960	GAAGAGGTCCGATGGTAGGAAGAAAGGCAAGGACA
3B-0725620-T9-72_1024	AAATAGTCATTAGACAATATCATCGGAATAAGGAGATG
3B-0725620-T9-72_1152	GCAACCGAGGAGAGGAGGACCAATGTTGAGGAGAT
3B-0725620-T9-72_1216	GCATGAAAAATGAGCAACAACTCATGTTTATTGACGC
3B-0725620-T9-72_1280	ATACTTAGAGCTTTGTCGTGGTCATGGGTTGGCGGCA
U-1002520-T9-72_0	TCTGTTTATTATACTGCTAACCCAGCTTTGAACAGTCTA
U-1002520-T9-72_64	GTGGTTATTGGAAGATGATAGGGGGATGGCACCAA
U-1002520-T9-72_128	TCCATCCTTGTAGTAACAAACGTACACTTAGCTCGGCC
U-1002520-T9-72_192	TGGATAACTAGCGTGCATTTGGAGTGCTTGGCAAGGT
U-1002520-T9-72_256	GGCCTGTTGGGCGCTAACTTTTTCGTTCTCTTCCTAGC
U-1002520-T9-72_320	CATTAAGAACATGTATAAATGAAACTAGTATTATCATTT
U-1002520-T9-72_384	AATACTAGTAGCACTGCCATGCATGCTGCCACTTTTTT
U-1002520-T9-72_512	AATTCAGTCGGATGCAGAGTTATTGCGAACATTGTTG
U-1002520-T9-72_640	TGCTTACCCGCTTTAGTGTCTTGGCTGCCGTTTTTAGC
U-1002520-T9-72_704	TGCATGGTGGACCTGCGCCGTGCAAATGCACATAAAT
U-1002520-T9-72_768	AGCCGTCGTTGCTTTTGCTAAGATGATGCAGTGAATTT
U-1002520-T9-72_832	GGCATAATTAACAACGTCTGTTAGAAATTCATCAACAC
U-1002520-T9-72_896	GTGGGCAAACCCAAGCCAGTACCTCCTTCATGTGGCA
U-1002520-T9-72_960	ATCAGTCACCACTGATGCAACCTATACAATTTTGAAGG
U-1002520-T9-72_1024	CTGCTTTGCAATTGTATGCACGTCCTTGCACAGTTATAT
U-1002520-T9-72_1088	TCTGGGAAAAGGTCTTGTCCAGTTTAGGTCAATATATA
U-1002520-T9-72_1152	CGCAAGCTTGGTGGCTAGAGCATGCATGGAATGGTG
U-1002520-T9-72_1344	TATTCTTTCATTTTGTGTCAGCATCTCAGCGCGTTAAGT
U-1002520-T9-72_1408	AATTATACTGATATTTAGACTAGCTAGCTTTGAACAGC
U-1002520-T9-72_1472	GGATCGTTTGTTGAAAGATCAGCTTTAACAGCCTAAC
U-1002520-T9-72_1536	AGTTTGAATAAGAGATCTCTTTGAATAACGTGGCCGC
U-1002520-T9-72_1580	ATCCATGTGacaacaacaacaacaatagcaacaataacaacaa
U-1002520-T9-72_448	CACTCACCGGTTTTGCAGCTGCTCCACGAAATTCAGT
U-1002520-T9-72_576	ATAAGAGCACTTCCACTATAGAACAAAGCATAGAAGG
3A-0687070-T9-73_0	GGTCCACAGCCCTCCGGTCGGCCGACCTACCCCTCAA
3A-0687070-T9-73_64	TGAGGGAAAACAAACGATAGATGATGCACTTGGTCG
3A-0687070-T9-73_192	GGGTAGAGGTGACATGTATACGGGGGACAGCCGCAG
3A-0687070-T9-73_384	GAAGCAGAATATATGGTACATTCATTAAACTTGAGTAG
3A-0687070-T9-73_448	GCTAGCTTATGTTCTTGATAAGCTACCTGGAATATATTT
3A-0687070-T9-73_640	CACGTCAAGAACAGGACATTAACTGATGGCGTTGGT
3A-0687070-T9-73_832	GGGGAATGGGATTCACTCGCCATACAGTTTTCGACTT
3A-0687070-T9-73_896	TATCTTTGCGTACCTTTTGCAGCATTGTTTTTCCACGA
3A-0687070-T9-73_960	ACCAATGTTGCCTTTGTCGAGAACAAAATTGATACTCT
3A-0687070-T9-73_1152	CATAGATAGTGCTTACGTGATTGCATATTGATTTGGCT
3A-0687070-T9-73_1216	CTACTGCCCCAAAAGCTAAAATCTGTCACTGATTTATC
3A-0687070-T9-73_1280	TAAAGTTTCTTCCCGGATTAGCTATGCAAAAACATGCA
3A-0687070-T9-73_1344	CATCGTGATCAGTGCATGTACTACGTAGGGATTTATAC
3A-0687070-T9-73_1580	GCATGTGTGCACGCATATTCATTTTTACGCTGCATGAA
3B-0785120-T9-73_0	GGTCCACAGCCCCTCCGGTCGGCCGACCTACCCCTCA
3B-0785120-T9-73_64	ATTGAGGGAAAACAAACGATAGATGATGCAATTGGT
3B-0785120-T9-73_128	CAGGAGATGGAAGATGCAAACTAATGAATAAGTAGCA
3B-0785120-T9-73_448	GAATATATGGTACGTTCATTAAACTTGAGTAGTGCGCG

										3B-0785120-T9-73_512	TTATGTTCTTGATAAGCTACCTGGAATATATTTGTTGGA
										3B-0785120-T9-73_704	AAAAACAGGACATTAACTGATGGCGTTGGTTAATTGC
										3B-0785120-T9-73_832	AGGAATCCCACCCACCTTTTTCCTGAAAGGAAAACAA
										3B-0785120-T9-73_896	TAGGATTCACTCGCCATACAGTTTTCGACTTTACTTAA
										3B-0785120-T9-73_960	TGCGCACCTCTTGCAGCATTGTTTTTTCCACGAAAGG
										3B-0785120-T9-73_1024	TGTTGCCTTTGCCGAGAACAAAATTGATGCTCTCTC
										3B-0785120-T9-73_1088	GCATGGTGGAATTTACTTTTGCCTGCTAGCTTTGTCCC
										3B-0785120-T9-73_1280	GCCCCCAAAGCTAAAATCTCTCACTGATTTATCTGCTG
										3B-0785120-T9-73_1580	GTAACAAAGACTAGTGTCATATACAAAAATATGCACGA
										5A-1196250-T9-74_64	CATATGGGACGATGGTCCGACCGTGTTATCCATTTGTT
										5A-1196250-T9-74_128	ATTAAAAACGATAGAATTACAATAAAGAGttacaaaataa
										5A-1196250-T9-74_256	ttaatcaatgtttcgccaaaatattgtcactcattttcaatctctgaaatt
										5A-1196250-T9-74_448	TTATATTACCCCCAATGTTATGTCCTTGTGAAGCTATAG
										5A-1196250-T9-74_512	TGAAATTTAGCTCTCAACTTTAGTATATGCTGAGCATG
										5A-1196250-T9-74_576	GTACTTAATTTGGTCTGTGTGCTAGTCCTAACAAGCTG
										5A-1196250-T9-74_704	TCGCGCTGGGCTCAACCCTAGTGAGGAGAGGACGTT
										5A-1196250-T9-74_768	CAAATTTGAACTACATGTACATTTAAAAATGCCTCAAA
										5A-1196250-T9-74_832	GTTTAGTATATGTTTAGGTCATATATGCAAGTAACAACT
										5A-1196250-T9-74_896	CACATGTGCCTCGAGGCCGTCTTCAATTTCTAAACAA
										5A-1196250-T9-74_960	TTTGAAATTTTAACCACACACTCTACATGTGATATAATG
										5A-1196250-T9-74_1024	CAAACAAAATTATTCTTTTTAAACTAAAAATCATTTTA
										5A-1196250-T9-74_1088	TGAATTTTCAGATATACATATTTGAAAGTACCAAAATTT
										5A-1196250-T9-74_1152	TAAGTATATTATTTATGATCTTTCTTCCCATTTTGGAAAT
										5A-1196250-T9-74_1216	AATTTATCTACACGTGGAGAAACATTTTTTGTTCGTGC
										5A-1196250-T9-74_1280	tctcatgatacttatatatattagtacttcatctatatatagccatatat
										5A-1196250-T9-74_1580	GCTTGGAACATATCCCTAGCACCAACGTACGCACACT
										5B-1319010-T9-74_256	GTTGACACACTTATTTAGCCACTACTGGAATCGCCCTA
										5B-1319010-T9-74_320	TGGGACTTTTTGCGTGCATTTTGAAATTAATGTCATTT
										5B-1319010-T9-74_384	GAAGTAGGAATATAAGTATCATGAGATAAATATATAAT
										5B-1319010-T9-74_448	AATGTTTCTCCACGTGTAGATAAAATTTTGAATTTTGT
										5B-1319010-T9-74_512	CCCATGCCACGTCCTTGTTTCACCATGCTCGGCTGAG
										5B-1319010-T9-74_576	TGATGAGAGAACAAACTTTTCTGGATTGAATTTCGAA
										5B-1319010-T9-74_640	TTCGAGCTAACATCAACTCTGTTGTGTTTTTAATATATT
										5B-1319010-T9-74_704	AATACATGAAAAATAAACAATATCTAGTTATGAAAGGT
										5B-1319010-T9-74_768	AAATGAGCATATTTATTTTCTTTGCAGTTGGAACAACC
										5B-1319010-T9-74_832	CTTGCATCAAAATAAGTTAGATCGAGTGTCTTTGAATA
										5B-1319010-T9-74_896	GCCAAGTGCCGAGAATCAGAATCTCCATGAAAAAAAT
										5B-1319010-T9-74_960	AGTAGGATGTCCTTGAATTGAGATTATGGCAATCCCA
										5B-1319010-T9-74_1024	CTACTGAGAAACTGCTTATAGGTAGGGTAAAATCAGT
										5B-1319010-T9-74_1216	GCCCCACACAGGTAATATGGCCACCGACCTGGCCCAC
										5B-1319010-T9-74_1280	TGCAAGGAGCGTATGGGCGCCCTACATTGGTAAAATA
										5B-1319010-T9-74_1344	TTGTTTTATTGTGTTATTTCTTTATGGTATTGCCTGAGA
										5B-1319010-T9-74_1408	TATTTGGAATGTTTAAGCATTTTATGAATAAATTTGTAT
										5B-1319010-T9-74_1472	AATGCAAGTACATGTAAACATTTTAAAAATGGGAAGA
										5B-1319010-T9-74_1536	AATTTAGGTCTTATAAAAGAAAATTTGGGTCTTTCAAA
										5B-1319010-T9-74_1580	TATGTGAAAATTCAAACACATGAGCTTTATTTGCAATA
										5D-1441250-T9-74_854	AGGGGAGGGGGCGATTCTTTCCTGGTCCCTTGGTTC
										5D-1441250-T9-74_920	TTCCGTACCTGGATTTAGTCCTTCTTGTAGCTTTCATAG
										5D-1441250-T9-74_986	TGTGTATTCTTGTTATTGTACTATTACACAAAATATATTA
										5D-1441250-T9-74_1052	TTAAAAAAAATACTTGCCCCACTTCCGGATTCGAAATT
										5D-1441250-T9-74_1118	TTGCAAAAACTCTTCCTTTTAGGTAAAACTCAGCCGA
										5D-1441250-T9-74_1184	AACAATGTCGCGAGATGCATTTACTACGAGATTCAAA
										5D-1441250-T9-74_1250	TGTTCGTGCACAAAAAAAGTGAGATTATGTATTTATCT
										5D-1441250-T9-74_1316	AACATATAGCCATATATGGAGAAATGACATTAATTTCA
										6B-1646200-T9-75_192	GTGACACGTCCTTTTTTTGTAAAGGGGAGCAATAGGA
										6B-1646200-T9-75_256	TCTCTCTCTCTCTCTAGCTTGCACATAGAGAGACTTAG
										6B-1646200-T9-75_320	AGTGAGAAAGAAAATGAGCACAGGGACACAGCATA
										6B-1646200-T9-75_512	ATTTAAAATGTGCTGTGAGAAGTCTCAAGAAAAACGT
										6B-1646200-T9-75_640	CTTCCCCATTGATCAAAACCATGATATATACCTGTCTAG
										6B-1646200-T9-75_704	ACTACCCTATACTAAAAGTGCTTCTCATTTCAAAGCAA
										6B-1646200-T9-75_1024	GGAGGATATGATTGGCCCTTGTCTAGTGTCATGGCAA
										6B-1646200-T9-75_1216	cctccctacatctctctctcctcctcctctccTTTTTTGTGAGTAA
										6B-1646200-T9-75_1280	ATAGAGGTTCTTGAGTCACTTATTTGTAGTTCTTTGGC
										6B-1646200-T9-75_1344	ACTCTTGGAGGGTTAACTCCTTCTAGATTTGAGCGGA
										6B-1646200-T9-75_1408	TCGCAGGATCAAAGATGTTGTCATGACAGTAGACAAG
										6B-1646200-T9-75_1580	CTTAGGAAGAGGTTCTGAATATTGGATGACAAAACAA
										6D-1734740-T9-75_320	tctctAGCTTGCACATAGAGGGACTTAGAAGGATGTCT
										6D-1734740-T9-75_512	CAGCATGTGCCCCCAGCGCCCCACTCCCCAACTAGTG
										6D-1734740-T9-75_704	TGATCAAAACCATGATATATACCTGTCTAGCATATATGT
										6D-1734740-T9-75_768	TGTTAAAAGTGCTTCTCATTTCAAAGCAAATATGCCGA
										6D-1734740-T9-75_1088	ATTGGCCCTTGTCTAGTGTCATGGCAACATCTTCTATC
										6D-1734740-T9-75_1152	GATATGTTCCGCTCAAATCTAACTTCAAATGTGTCATTT
										6D-1734740-T9-75_1280	cctACATCTCACTctcctcctcctcctcctcctcctcctTTTTTGT
										6D-1734740-T9-75_1408	cttggagggttaactccttctagacgattagtgttgctcgaaacccttc
										6D-1734740-T9-75_1472	caagaagtttccgaatgttggagaccgcctacttcatgtgaaaatcca
										6D-1734740-T9-75_1536	ATGGACTTTGTGTCCTAGACCAAGGAGGCCTATGTGG
										6D-1734740-T9-75_1580	TTGGTGGGGGTCTTTTGTGACCTTAAAACTTTCTGTT
										7A-1776850-T9-76_128	CCATGCCATGCTAGATCAGCCCCAACATGTAAGCTGG
										7A-1776850-T9-76_192	AATTTGCTTGCTCACTTTCTAGCTAGTTTCTCAATTAA
										7A-1776850-T9-76_640	TGTGGTCACTTGGTAGAAGTACTCCACAAGTTAGTTA
										7A-1776850-T9-76_1280	CAGTCGGTACGTACTGTCCGTGCTGCCGCGTTTTTGG
										7A-1776850-T9-76_1344	CTGCGCTACTTAAGTCTTGTGATTCGTTTGTGTGTAAG
										7A-1776850-T9-76_1408	ctagaggaagagaggtagaaacaaggagagaggagagaagagG
										7A-1776850-T9-76_1472	TGAGCTAGCTAGCTATAGGATCTGCAAACACTGTGAG
										7A-1776850-T9-76_1536	CTAGCTTTTTGTTGCCCCCGGCTAGTTAAGCCAAAAG
										7A-1776850-T9-76_1580	TATAGCATGTATGTTTGTTCTTTATTTTGCTCGAACCAT
										7B-1886500-T9-76_128	ATCACGAACAGGAGGGGGGGTCGGGTCCCTTTTATA
										7B-1886500-T9-76_192	GTATGATCACTTTCACAGAGAGGTAGCTAGAGAGAG
										7B-1886500-T9-76_384	CTATGTGGCAATCATTTCGTACGTCTACTGTAGCGTGC
										7B-1886500-T9-76_448	GCTGTAGCACACAGATGACCGGTGGTACGTACGTTTG
										7B-1886500-T9-76_512	CTCATCGTCCATAGTTTGTGGCTGAGGCGCTGAGCCA
										7B-1886500-T9-76_576	AAGCGGGGCGAAAGCCTTTTTCGGCATAAAATTGCG
										7B-1886500-T9-76_640	TACGTAATTAGGTCTTCTTAGATCTATTCGGTTGTTCG
										7B-1886500-T9-76_832	AGCATGCTCATCCCATGGAGGCGACACACCCGATCAT
										7B-1886500-T9-76_896	TTGATGCCTATTTGGTGGAGTTTCCCGAGTTTCCACGT
										7B-1886500-T9-76_960	TTTGTAATCGTCAAACAAGATTTTAATTGGTTATCAGT
										7B-1886500-T9-76_1024	TTTCTAAATTTAGATTCAATCGACTGGTAACTAAAAAT
										7B-1886500-T9-76_1152	TGGTCTAGTTAATGAAATCGAGGAGAAGTAGTTCAAA
										7B-1886500-T9-76_1216	ATATAAATATCATCCACCTTGACGTAACCTTGGTATCGG
										7B-1886500-T9-76_1408	TTTCCTATTCTTAAGCAAGCCCTCGAGAGGATTAGTG
										7B-1886500-T9-76_1472	TGTACGCCCCGCATTCAAGAAAATGGGAACCAATAGA
										7B-1886500-T9-76_1536	AGGGAAAAACCTATGCTCATGTGTATTGAAATGTGTA
										7B-1886500-T9-76_1580	CTCTAAAGCTGGTGCCATACTAAGTCAATTTCCATAAA
										7D-1994810-T9-76_128	ATCACGAACAGGAGGGGGAGTCGGGTCCCTTTTATA
										7D-1994810-T9-76_192	GGCCTTGTGTGATCACTTTCACAGAGAGGTAGCTAGA
										7D-1994810-T9-76_384	GTGCGTATGATCCTATGTGGCAATCATTTCGTACGTCT
										7D-1994810-T9-76_448	AAGGGATATTTTGCTGTAGCACACAAGCGACCTGTGG
										7D-1994810-T9-76_512	TGTACTACTAGTTAGCTCATCATCCATAGTTTGTGGCT
										7D-1994810-T9-76_576	CATCAAACCTTCGTTTCGGTATGTAATTAGGTCTTCTT
										7D-1994810-T9-76_768	CGTGACCTCAAGAAGCACAAGCATGCTCATCCCATGG
										7D-1994810-T9-76_832	AGATGTAGGAATCTAGGAATTGATGCCTATTTGGTGG
										7D-1994810-T9-76_896	GTAGGGCATAGTTCTTGGTTTGTAAGCGTCAAACAAG
										7D-1994810-T9-76_960	AATATGAACTTTTTCAGATTTCTAAATTTAAATTCAATC
										7D-1994810-T9-76_1088	AGCGGGCCCATTGATATGTGGTCTAGTTAATGAAATC
										7D-1994810-T9-76_1152	ACTTTTCAAAATAACAAAATATAAATATCATCCACCTTG
										7D-1994810-T9-76_1344	CAAGTCTTTTTTCTTATTCTTAAGCAAGCCCTCGAGAG
										7D-1994810-T9-76_1408	ACTAAACTTTGTACGCCCCGCATTCAAGAAAATGGGA
										7D-1994810-T9-76_1472	ATATTTGTGGGGAAAAAACCTATGCTCATTTGTATTGA
										7D-1994810-T9-76_1536	GTGACATACTAAGTCAATTTCCATAAACTTGTGCCCCA
										7D-1994810-T9-76_1580	TGCATTACTTAATTAAGGGTTTCTTTCGGGGCACATTA
										7A-2081800-T9-77_128	ATTATAATTGTGGGCTTGAGCTGGTTCATGTGTGTGA
										7A-2081800-T9-77_192	TTGAAACTGGACAATTGTTTTAGTTTTCGGATCACATT
										7A-2081800-T9-77_320	TAGTGTGATTTTAGTGTTTTTAGACTTGGTTGAATGTC
										7A-2081800-T9-77_384	TGATGTTGGAATTTTTGAACTGCTGATGCATCTTCTTT
										7A-2081800-T9-77_448	GTGCAATCACGATTCACGGTGATGTGGAATCACATAAT
										7A-2081800-T9-77_512	AGTGCATTATAGTGACAACACTAATTTCCTTTTCGGAG
										7A-2081800-T9-77_576	TGCAATGAATCTACCAGAACAGTTGCAGCTAGATCATA
										7A-2081800-T9-77_640	AAAGCAGGTTTATGCCACTCCCAATGCATGCTCTTAAT
										7A-2081800-T9-77_704	ACGTAGTTTTTCTCTCATGAAAAGTCGCCCAGGAAGG
										7A-2081800-T9-77_768	AGTACAGCTGTAAAGATACCTCCTCCTAGTATCTGGAT
										7A-2081800-T9-77_832	CCTTCCTTGTAAATGATGCAACTGTGATTGAATGGAG
										7A-2081800-T9-77_896	AAAGCTTAGAGAGAATGTTATTTGACGACTCGACTCC
										7A-2081800-T9-77_960	TTAATTCGCTAGTTTCGACGATTGAACAGAAAATGCT
										7A-2081800-T9-77_1024	TTATTTCACTAGAACAGTTTTTTCATTTCTGTTGTTTTT
										7A-2081800-T9-77_1088	TGCCTTATTAGTGAGAAAATACCGTAGAACAATTGTTC
										7A-2081800-T9-77_1152	AAGGGCTGATTTGAGGCCTCTCTACAAAAAAATTAAG
										7A-2081800-T9-77_1216	TTTTTTTTAGAGAGAAGAAGGCCTTTTCCCAAGCTAA
										7A-2081800-T9-77_1408	CGACCCGACCCCTCGGAGCGCCCTGACCGTCAGATC
										7A-2081800-T9-77_1472	CCGAAACTACCCACGCACGCCTTCCCTGATTCGTTCG
										7B-1951720-T9-77_128	TTTTGATGATGAGCACTGGGAAGTGCTGCTATCTAGT
										7B-1951720-T9-77_192	GAATCTCCAGGCACAAATTGCTGCTAGTTTTTATGGC
										7B-1951720-T9-77_256	TTTCTTTTTATTATGTTTGAATTGTCATATTTCTGCAATC
										7B-1951720-T9-77_320	ATAATGTCATCTGAAGAACTGCAATTAAGTAGGTGCAT
										7B-1951720-T9-77_384	TGGAGGAGCTTGTGAAGACGCCAAAACCACCTGCAA
										7B-1951720-T9-77_448	TCATATCCACACTGTAGGTTTATGCTACTCCCAAAGCA
										7B-1951720-T9-77_512	TGCCCAAACAAACGTCGTTTTTTCTCATGAAAAATCG
										7B-1951720-T9-77_576	AGTACAATTGTAAAGATACCTCCTTCTAGTATCTGGAT
										7B-1951720-T9-77_640	CATTCCTTGTAAATGATGCAATTGTGATTGAATAAAGG
										7B-1951720-T9-77_704	CAAAGTTTAGAGAGAATATTATTTGACAACTCGACTC
										7B-1951720-T9-77_768	CTTAATTCGCTAGTTTCGAAGTTCGAACGGAAAATGC
										7B-1951720-T9-77_832	TTTTTCACTAGAACAGTTTTTTCATTCTGTTGTTTTTCT
										7B-1951720-T9-77_896	CCTTATTAGTGAGAAATACCGTAGTACAATTGTTCTAC
										7B-1951720-T9-77_960	TGATTTGAGGTTTCTTTGCAAAAAAAATTAAGGGCAC
										7B-1951720-T9-77_1024	TTTTAGAGAAAGAAGGCCTTTTTCCAAGCTAAGCCTG
										7B-1951720-T9-77_1088	TCTGAGTATGACGGGCACAAACGGCCTTTTTCCAAG
										7B-1951720-T9-77_1280	CCCCGTCCGGACTCCGGACCGGAACGGAACGACCCA
										7B-1951720-T9-77_1472	GCCCCCCTCTCTCCCGCGCTATCAAACAACAAGTGAA
										7D-2036600-T9-77_126	AGTTTTTCTTCTTTTTGTGATTAATTTCAATTAAGAAG
										7D-2036600-T9-77_189	TAGAATTTTTGAAAGCCTAATACTTATCTCTTTATTGTA
										7D-2036600-T9-77_378	TTGTGGAGGCCATCCTTGTTAAACCATGCGCCGGTTT
										7D-2036600-T9-77_441	TCCTCAGAAATTACTTTTTCAGACACGATGGGACAATA
										7D-2036600-T9-77_504	TTAGTTTTTCATATTGAGCACTTAAGCTATATTCATGTT
										7D-2036600-T9-77_693	CCTATTTCCATCATAGTACCATGTTTGCTATTTTTTTGTT
										7D-2036600-T9-77_756	CTTCAGTTTCCCTTATATTTTAGTACATATATCTCTGTTT
										7D-2036600-T9-77_819	TACAGAACTTTTGGCGTGATAAATTTGGTGCACTGCC
										7D-2036600-T9-77_882	GTTTACCCTCATGTACTAGGTATAAGATCATCTATAAGA
										7D-2036600-T9-77_1455	TCTCCATCATTATACATAAATGAGCGTTGTGGTAAACA
										7D-2036600-T9-77_1518	CTACCTTCATGTGAAGGATACTTAAATAAAAGCTAATG
										7D-2036600-T9-77_1580	CATTGGTATATATATATGATATAAAACACATCCGTACAAT

Trait&gene no.	Gene	Homoeologues	IWGSC gene ID (refseq1.1)	WGIN Promotome ID	Orientation	ATG	Target Sequence	MYbaits	MYbaits coverage (%)	MYbaits ID	MYbaits sequence
T10-1	spo11-1	ABD	TraesCS4A02G105600	4A-1021240-T10-1	forward	119,781,719	GGTGGACAGGGTGGGCCGCGGCCCACCCTGGGATTTCGGACTAGGTTATTCAATGATGGAG	11	54.6	4A-1021240-T10-1_0	[illegible]
T10-1	spo11-1		TraesCS4B02G198400	4B-1052400-T10-1	reverse	427,274,027	GTGGAGAGATCTGGAGGCGGAATCACCGGGGAGGAGGGGTGGGGGCACTACGGCGGAG	10	46.4	4A-1021240-T10-1_640	[illegible]
T10-1	spo11-1		TraesCS4D02G199200	4D-1134610-T10-1	reverse	346,133,617	GTGGAGAGATCTGAAGGCGGAATCACCGGGGACGAGGGGTGGGGACACTACGGCGGAG	17	77.2	4A-1021240-T10-1_704	[illegible]
T10-10	COM1	ABD	TraesCS3A02G124100	3A-0676090-T10-10	forward	100,188,168	AAATCGAGGGGGTGGAGTGTTTCATATTCAGGTGGTTTGTGTAACAGCCCAAATAAAAAACT	19	84.7	4A-1021240-T10-1_768	[illegible]
T10-10	COM1		TraesCS3B02G143800	3B-0772450-T10-10	reverse	132,530,541	TTCGGCAACCAGGGGCAGATCGATGGAGCGCCGGGTGGGCGCCCGAATTCGTGGAAGCTAG	17	72.7	4A-1021240-T10-1_832	[illegible]
T10-10	COM1		TraesCS3D02G125600	3D-0922690-T10-10	forward	84,013,989	CCTGTAGTCGAAGAAGGCCAACGTCCTAGCATCGCCCTATGGCTCATAGTGGTCCTCGTGGAT	12	55.1	4A-1021240-T10-1_896	[illegible]
T10-11	HOP2	BD	TraesCS4B02G045000	4B-1052400-T10-11	reverse	32,420,653	GTGGAGAGATCTGGAGGCGGAATCACCGGGGAGGAGGGGTGGGGGCACTACGGCGGAG	10	46.4	4A-1021240-T10-1_1024	[illegible]
T10-11	HOP2		TraesCS4D02G044700	4D-1168630-T10-11	reverse	20,435,782	TTGTATTTTGAGGTGTGAGGGCCTCAAAATTAGCTTTCCCGTGGCACAAGATGTGCAAACAA	16	85.4	4A-1021240-T10-1_1216	[illegible]
T10-12	MND1	AB	TraesCS5A02G174300	5A-1199900-T10-12	reverse	367,598,324	GGCAAGCTATGGAGCACCGCCGAGCCGGTTGAGAGGGAGGACGGGGCGGCAGCCGGTCG	11	53.4	4A-1021240-T10-1_1280	[illegible]
T10-12	MND1	no D	TraesCS5B02G171700	5B-1295520-T10-12	reverse	316,368,401	TGCAGGCTATAGAGCACCGCCGAGCCGGTTGAGAGGGAGGGCGGGGCGGCAGCCGGTCGC	11	53.4	4A-1021240-T10-1_1344	[illegible]
T10-13	DFO	ABD	TraesCS5A02G152300	5A-1209920-T10-13	reverse	329,251,473	ACGCTTTTTCGTTGTGGTGGTTTGATTTGATTTGAAACTCTCAGGTCGAAGTTGGCAGGTACA	12	57.2	4A-1021240-T10-1_1408	[illegible]
T10-13	DFO		TraesCS5B02G150900	5B-1289680-T10-13	reverse	278,475,913	ACGCTGTGTGGTGGTAGTTTGATTTGATTTGAAACTCTGAGGTCGAAGTTGGCCGTACAGTAT	14	64.7	4D-1134610-T10-1_192	[illegible]
T10-13	DFO		TraesCS5D02G157500	5D-2100590-T10-13	reverse	245,205,744	CGCCGCTTTTTCGTTGTGGTGGTTTGATTTGATTTGAAACTCTCAGGTCGAAGTTGGCAGGTA	11	53.4	4D-1134610-T10-1_256	[illegible]
T10-14	PHS1	ABD	TraesCS7A02G233700	7A-1833370-T10-14	reverse	205,469,977	GGCGACGACGATGACGCCGAGAATGCGGTACGGAGCGTTCGCCGTTTTGGGGGCGTTGGG	12	51.8	4D-1134610-T10-1_384	[illegible]
T10-14	PHS1		TraesCS7B02G132000	7B-1930490-T10-14	reverse	159,520,722	GGCGACGACGATGACGCCGAAAATGCGGCCGTTTTGGGGGCGTTGGGGTTTTGAGAGCAG	5	28.7	4D-1134610-T10-1_448	[illegible]
T10-14	PHS1		TraesCS7D02G233900	7D-2020240-T10-14	reverse	195,634,156	GGCGACGACGATGACGCCGAGAATGCGGTACGGAGCGTTTGCCGTTTTGGGGGCGTTGGG	16	68.2	4D-1134610-T10-1_512	[illegible]
T10-15	RAD51	B no AD	TraesCS4B02G229600LC	4B-1078310-T10-15	forward	245,594,997	AACCTTTCACGTATACATATGTTAAATATGATTTCGATGTCTACATTAAAGGGCCCCCACGTTAT	21	87.8	4D-1134610-T10-1_576	[illegible]
T10-16	RAD51B	BD	TraesCS1B02G075900	1B-0173560-T10-16	forward	58,705,113	ACCACTGTGATGGATGAAAATGAACCATCATCAGCTCAAGCACAAAAGAAGAGAGAGAAAG	8	36.7	4D-1134610-T10-1_640	[illegible]
T10-16	RAD51B		TraesCS1D02G059600	1D-0240760-T10-16	reverse	39,872,665	CTCGGCCGCCGGTAGGACTCCGGCCACCGGGGGAGAGGAGGGGAATCCGGAAATTTTAGTT	6	29.2	4D-1134610-T10-1_704	[illegible]
T10-17	RAD51C	AB	TraesCS2A02G208200	2A-0365750-T10-17	reverse	189,035,609	ACGGAGGGGCGGCGCGGGCAGACGGCGTTGACGGAGGTGCGGCGCTGGCCGGCGGCGCG	1	7.1	4D-1134610-T10-1_768	[illegible]
T10-17	RAD51C	no D	TraesCS2B02G234700	2B-0448650-T10-17	reverse	233,554,430	GTCGCATTCGCGGAGCACGGCGTAGGCCTCCTCGTCGGTGCGGTTCCTGGCGACCCCCTTGC	6	25.3	4D-1134610-T10-1_832	[illegible]
T10-18	RAD51D	AD	TraesCS7A02G455900	7A-1794510-T10-18	reverse	652,293,412	TGGGGCTCTCCCTTCCTGTCCAAAGCGGAACAGTAGGCAAGCAGTGCAGCAGGCCAACGAC	3	14.6	4D-1134610-T10-1_896	[illegible]
T10-18	RAD51D	no B	TraesCS7D02G444800	7D-2002290-T10-18	reverse	566,021,320	GTGGGCTCTCCCTCCCTGTCCAGAGCCGAGCAGTAGGCAAGCCGTGCAGCAGGCCAACGAC	12	68.5	4D-1134610-T10-1_960	[illegible]
T10-19	RPA1a	A no BD	TraesCS6A02G335700	6A-1520570-T10-19	reverse	568,497,563	CGCCTCGCGGTGGACCGGATTCGCGGGGGCTTCGCGCTCTGCGGCGGGATGGGGCGGAGG	13	57.6	4D-1134610-T10-1_1024	[illegible]
T10-2	spo11-2	AD	TraesCS7A02G300300	7A-1793920-T10-2	forward	416,424,022	TCATTATTGTGCAATTTTATAGCTTTGTATTTATCTCCAAACTATCTGTCTACTTTGGCCAAGTTT	10	47.5	4D-1134610-T10-1_1088	[illegible]
T10-2	spo11-2	no B	TraesCS7D02G296000	7D-1986190-T10-2	forward	371,787,908	ACAGGTTTTGAAAAAAAATGTTGTTGTTCAAAATGTTTTTCTTGATTTTGAACAAAAATTTCA	13	72	4D-1134610-T10-1_1344	[illegible]
T10-20	DMC1	ABD	TraesCS5A02G133000	5A-1244210-T10-20	reverse	299,601,571	CCCGCGTGCGCGCCTGCGTCACAGGAGAAGGTGTAAGGAGCGAGCACTGGGACGCACGAG	17	70.6	4D-1134610-T10-1_1472	[illegible]
T10-20	DMC1		TraesCS5B02G131900	5B-1334750-T10-20	forward	246,136,992	TTCTTACGTATGCGCTTAACTGATATGCTTAAAGTAAATCCTTATGCTAAGTATATGAAGGATAT	13	58.8	4D-1134610-T10-1_1536	[illegible]
T10-20	DMC1		TraesCS5D02G141200	5D-1449300-T10-20	reverse	224,950,703	CCCGCGTGTCCGCCTGCGTCAGAGGAGAAGGTGTCAGGAGCGAGCACTGGGACACGCGAG	18	76.5	7A-1793920-T10-2_320	[illegible]
T10-21	XRCC3	ABD	TraesCS6A02G199100	6A-1532030-T10-21	reverse	313,298,658	CGCCGGCAATCTCGCTGCCAAGGGATTTGGGGATTTAGCAATTTGGGGTTCGAATTGGCGCT	18	83.1	7A-1793920-T10-2_384	[illegible]
T10-21	XRCC3		TraesCS6B02G229400	6B-1609120-T10-21	forward	367,349,236	TGCCCCCCTCCACCATATTCCACCTCGGTCATATCATCGCGGAGTTTAGGCGAAGCCCTGCGCC	12	58.4	7A-1793920-T10-2_448	[illegible]
T10-21	XRCC3		TraesCS6D02G182800	6D-1712870-T10-21	reverse	217,759,620	AAGAAGAGGGAGGNNNNNNNNNNNNNNNNNNNNNNNNNNNNNNNNNNNNNNNNNNNNNNNNN	11	56.4	7A-1793920-T10-2_512	[illegible]
T10-22	XRCC2	ABD	TraesCS3A02G381500	3A-0655460-T10-22	forward	631,719,410	TGTTTCTAATTTGGACCTTTTGGAATAAAAGAAATGTTAGGGTCTTTCGCTACAAGTCCGCAC	15	71.8	7A-1793920-T10-2_576	[illegible]
T10-22	XRCC2		TraesCS3B02G414200	3B-0791120-T10-22	forward	650,918,903	TCCAGTACTACCACTCAAACAGTATGTAACAAATTATATAATGATACAATGCAGCCAACCAAAT	14	62.6	7A-1793920-T10-2_768	[illegible]
T10-22	XRCC2		TraesCS3D02G374700	3D-0883140-T10-22	forward	488,109,343	TGCAGAAATGGCATTTGGTGCGCGGGCTACTACTAGCTCCATTTGTCTACAAAAAATTAAAAC	22	89.4	7A-1793920-T10-2_832	[illegible]
T10-23	MCM8	A no BD	TraesCS1A02G288700	1A-0036950-T10-23	forward	486,042,256	AAGTTGCCCAGAAGCACGCCAATTATCTCCTGCGTAATTTGGGCCGGAGAATCTACTTCGAGA	6	35.6	7A-1793920-T10-2_896	[illegible]
T10-24	FIDGETIN	A no BD	TraesCS3A02G151300	3A-0681930-T10-24	reverse	140,056,408	GCCGGCCGGCCGGCTGAGCCTGAACTCCCTCCGACGGCGATGCGAACCAACGTTGAGCTAG	6	32.5	7A-1793920-T10-2_960	[illegible]
T10-25	FANCM	ABD	TraesCS4A02G217700	4A-0969220-T10-25	forward	517,400,440	ACACACATTTTCTACATGCTATAGGCTACAACACCTGCACGATCTTCGCCCACCTGGAAGGCG	19	84.1	7A-1793920-T10-2_1472	[illegible]
T10-25	FANCM		TraesCS4B02G096400	4B-1105240-T10-25	forward	100,313,808	TTATAACCTCTGCTTTCCTCTGCGAATGACCTATCCTTTACCTTTATGTATTTACTTTTTATGCAAG	15	63.8	7D-1986190-T10-2_128	[illegible]
T10-25	FANCM		TraesCS4D02G092800	4D-1162360-T10-25	forward	67,790,802	CACATGGACTACGTCTAGTTGGATGGGTTGTGACTTAACATGGAAGATAAACTCTCTTCATCCT	2	10.8	7D-1986190-T10-2_192	[illegible]
T10-26	RECQ4a	ABD	TraesCS2A02G304900	2A-0297450-T10-26	reverse	524,336,996	TCGCTGCTATGGTAATTAGGGCATGAAGTGCTATTGAATGTTTGGTGATGAGATCTCACAAAG	15	69.5	7D-1986190-T10-2_256	[illegible]
T10-26	RECQ4a		TraesCS2B02G321700	2B-0402520-T10-26	forward	458,857,418	GATCTGTTTATTTAGATGTGCACTGCAGTGTATAACCACAGAAAATATCAGTATTAATACTGGT	16	80.1	7D-1986190-T10-2_448	[illegible]
T10-26	RECQ4a		TraesCS2D02G303500	2D-0530420-T10-26	reverse	387,741,641	AGAGTGTGATCTCTGCCGCTCATCGTCTTGGGTTGGCCTCGCCATATAATCCCCAAGTATCTGG	16	78.8	7D-1986190-T10-2_512	[illegible]
T10-28	MSH4	A no BD	TraesCS2A02G171900	2A-0356710-T10-28	reverse	127,217,440	CTCCTGCGCCGAACCCTGGAAGGATGCAACCACGGCTGCTAGCTGGTGTGTGCGTGCGGACG	3	17.9	7D-1986190-T10-2_640	[illegible]
T10-29	MER3	BD	TraesCS7B02G103700	7B-1943100-T10-29	reverse	119,211,146	GGCTGCAGCTCGGACCGCGGAAGAGGGGGAGGAATTAGTTGGCACCCACCCAATGTGGAT	15	58.6	7D-1986190-T10-2_704	[illegible]
T10-29	MER3	no A	TraesCS7D02G199800	7D-2033840-T10-29	reverse	156,800,534	GGCTGCAGCTCGGGTCGCGGAAGAGCGGGGAGGAATTAGTTGGGCACCGGCCCAATGTGG	10	44.2	7D-1986190-T10-2_768	[illegible]
T10-3	MTOPVIB	ABD	TraesCS7A02G410300	7A-1765990-T10-3	reverse	596,737,216	GGAGGCGGAGGGGGAAGACGCCGCCTGCTCTGGGGGTGTAGAGTAGGGGTTTGTGAATTG	19	84.7	7D-1986190-T10-2_896	[illegible]
T10-3	MTOPVIB		TraesCS7B02G309700	7B-1904090-T10-3	reverse	553,643,828	GGAGGCGGGGGAGGGAGACGCCGCCTGCTCTGGGGTAGACCAGGGGTTTGTGAATTCTGG	19	79.8	7D-1986190-T10-2_1088	[illegible]
T10-3	MTOPVIB		TraesCS7D02G403500	7D-1971220-T10-3	reverse	520,419,251	GGAGGCGGAGGGGGAAGACGCCGCCTGCTCTGGGGGTGTAGAGTAGGGGTTTGTGAATTG	14	69.2	7D-1986190-T10-2_1152	[illegible]
T10-30	MLH1	A no BD	TraesCS3A02G500600	3A-0628520-T10-30	reverse	724,882,305	GCCCGCCGCGCCGCGCCGCGTGTGGGGACCGGGCCGAGGAGGTGGGGAATGGGGAGGGG	6	32.5	7D-1986190-T10-2_1280	[illegible]
T10-31	MLH3	ABD	TraesCS5A02G352800	5A-1199250-T10-31	reverse	555,560,801	CGACGTGTTCTGGTAAAAAAAATGAACTTAGATCAGCCAACGGTAAAGACTGAAATGTATCG	16	68.8	7D-1986190-T10-2_1472	[illegible]
T10-31	MLH3		TraesCS5B02G355000	5B-1334260-T10-31	reverse	534,341,820	CGACGTGTTCTGGTTAAAAAAAGGGAGCTTAGATCAGCCAACAGTAAAGACTGAAATGTATC	10	43.1	7A-1765990-T10-3_0	[illegible]
T10-31	MLH3		TraesCS5D02G359800	5D-1437570-T10-31	reverse	439,670,898	GGCAACGAGAGTGAGGTGGTGGCATCGATAATGGGGATATGGAAGTGTGGTGGGCCTGGTA	12	51.8	7A-1765990-T10-3_192	[illegible]
T10-32	HEI10	B, BD	TraesCS6B02G220900	6B-1664420-T10-32	forward	315,651,369	ATGGTAGGTTCTGAAGAGTATAGGGTCATCGTGTATCGTGTCTGCTTTGTTCTTGCATTGTTTG	19	81.4	7A-1765990-T10-3_256	[illegible]
T10-32	HEI10		TraesCS6D02G182400	6D-1733950-T10-32	reverse	212,803,976	TCATCGCTTGGATTTATGTCAGTAGGCTTCATATGGCTAAGAAATAATTAAAAAAAGGATTAGA	18	87.4	7A-1765990-T10-3_320	[illegible]
T10-32	HEI10		TraesCS5B02G165300	U-2113760A-T10-32	reverse	72,996,785	TTTGTATTCTTTGTTCAGAAGAACCCTGCAGGTCGAAACACAGATAAATGACTTGCACTAGAT	13	65.8	7A-1765990-T10-3_384	[illegible]
T10-34	ZIP4	ABD	TraesCS3A02G401700	3A-0645950-T10-34	reverse	647,482,859	GGCGACGCGGGCTAGGGTTCCGGCGGCCGGGATCGGGCGGATTTGGGCGGGAAGCGGCG	20	87.8	7A-1765990-T10-3_448	[illegible]
T10-34	ZIP4		TraesCS3B02G434600	3B-0809860-T10-34	reverse	672,873,938	GGCGGCCGGGGCTAGGGTTCCGGCGGCCGGGATTGGGCGGATTTGGGCGGGAAGCGGCG	21	85.6	7A-1765990-T10-3_512	[illegible]
T10-34	ZIP4		TraesCS3D02G396500	3D-0884100-T10-34	reverse	511,350,682	GGCGGCGCGGGCTAGGGTTCCGGCGGCCGGGATCGGGCGGATTTGGGCGGGAAGCGGCG	4	20.5	7A-1765990-T10-3_576	[illegible]
T10-35	TOP3a	BD	TraesCS4B02G280400	4B-1063200-T10-35	reverse	563,215,576	CGCGGGAGGAGGGGGGAGGGGGCTCCGCCGCTCGGGGGTGGGGGGAATGGGGAAAGGG	12	51.8	7A-1765990-T10-3_704	[illegible]
T10-35	TOP3a	no A	TraesCS4D02G381900LC	4D-1122780-T10-35	reverse	450,208,662	GAGGTGGCCCGTGACGGAGGTGACGAGCATGTGGCAGGCCTGGGCGCCGATGGCGTAGTT	1	7.1	7A-1765990-T10-3_768	[illegible]
T10-36	SMC1	AD	TraesCS7A02G295800	7A-1751550-T10-36	forward	388,766,735	GCAGATGCAGCAACTCTTCAAGATGAGTGATCTGGGCAAGCTGAGCTACTACTTGGGCATAG	7	42.8	7A-1765990-T10-3_832	[illegible]
T10-36	SMC1	no B	TraesCS7D02G289400	7D-2059370-T10-36	reverse	332,265,922	GTCTTCCCCGCCTCCTTAGTTCCCCTTGCCCTTCCTAACTGAACTAGGGTTTTCGTCGAAGGGA	10	44.2	7A-1765990-T10-3_896	[illegible]
T10-37	SMC3	ABD	TraesCS1A02G428700	1A-0049590-T10-37	reverse	582,420,850	GCCACCCTTTTTGGCTACTTGATCACCTGAGGATAACCACAATGTCAACTTATATGTTAAAAGT	15	76.2	7A-1765990-T10-3_960	[illegible]
T10-37	SMC3		TraesCS1B02G463400	1B-0119800-T10-37	reverse	675,570,457	GGCTAAGACAGGGAGAGATCACACCACCATTAATTCCTGCAAACAGAGGAAGGTCACTCGT	12	57.2	7A-1765990-T10-3_1024	[illegible]
T10-37	SMC3		TraesCS1D02G438000	1D-0184520-T10-37	reverse	485,115,526	GGCTAAGACGGGGAGAGATCACACCACCATTAATTCCTGCAAACAGAGGAAGATCACTCATG	12	58.4	7A-1765990-T10-3_1088	[illegible]
T10-38	REC8	ABD	TraesCS5A02G303200	5A-1214560-T10-38	reverse	512,423,049	GGCTGCGGCCTGGTGGGGAGGGAGGGAGGGTGGGGTTCGACGGGGGGGCGGGCTAGGGT	8	40	7A-1765990-T10-3_1152	[illegible]
T10-38	REC8		TraesCS5B02G303500	5B-1363750-T10-38	reverse	487,833,456	GGCTGCGGCCTGGTGGGGAGGGAGGGAGNNNNNNNNNNNNNNNNNNNNNNNNNNNNNNN	9	47.1	7A-1765990-T10-3_1216	[illegible]
T10-38	REC8		TraesCS5D02G310000	5D-2138150C-T10-38	reverse	406,829,439	CGCTGCGGCCTGNNNNNNNNNNNNNNNNNNNNNNNNNNNNNNNNNNNNNNNNNNNNNNNN	11	51.8	7A-1765990-T10-3_1408	[illegible]
T10-39	STAG3	no A	TraesCS4D02G147700	4D-1177350-T10-39	forward	139,204,183	CGGAGGGCCATGGCGGCGACCTGCACACCTCATGTGGAGTACGAAGAGGAGGGATAGTCCT	20	66.6	7A-1765990-T10-3_1472	[illegible]
T10-39	STAG3	B, D	TraesCS5B02G165300	U-1083530-T10-39	forward	272,172,403	TCAGCTGGAGCCATGAGTCTGAAGAAGAAGGAGTTCCGCAGTTTGCACCAGGGAGGACGC	all baits failed stringency test		7B-1904090-T10-3_0	[illegible]
T10-4	PRD1	ABD	TraesCS2A02G275100	2A-0313060-T10-4	forward	451,815,266	TCTTCTAGTCATTTTACATCATCTGGTGTTATAGATGCAGTTGGTAGTATATATTGCTCCCCATAT	22	94.8	7B-1904090-T10-3_192	[illegible]
T10-4	PRD1		TraesCS2B02G292500	2B-0417700-T10-4	forward	407,226,551	AAGAGTAGAACATCATCGTAACTGAAAATAAAGCAGAATAACATCACCAAGCTTCAGGCGTG	12	59.3	7B-1904090-T10-3_256	[illegible]
T10-4	PRD1		TraesCS2D02G274100	2D-0545450-T10-4	forward	342,101,342	GGATATCGGTAACTGGTACCATATCGGTCAGGTGCTGAGTAGGGATAAATAAGCGATTTTTCC	13	70.8	7B-1904090-T10-3_320	[illegible]
T10-40	ZYP1	ABD	TraesCS2A02G340400	2A-0316910-T10-40	forward	575,598,944	TATAGTCTCCTTCGGTTCATTATTTCGAACAAGGGGATGCAAAATTACCTTCAATAAAAGAAA	8	43.3	7B-1904090-T10-3_384	[illegible]
T10-40	ZYP1		TraesCS2B02G338300	2B-0426540-T10-40	forward	483,449,878	GCTGCTATAGAGAATCCTAACCCATCATCTGCAGTACTGTACACCGTTGTAGTGCAAATAACCA	7	42.8	7B-1904090-T10-3_448	[illegible]
T10-40	ZYP1		TraesCS2D02G318100	2D-0523770-T10-40	forward	407,961,521	TAGGTAAGATGGTCTAGTGGTTATGGCAGCGCGCAGAGGTCACCAGTTCGAATCTTGCACGG	1	7.1	7B-1904090-T10-3_512	[illegible]
T10-41	ASY1	ABD	TraesCS5A02G286500	5A-1234690-T10-41	forward	494,388,234	AGACCCTTTCGCAACCACCTACTCGTGCATCGCCCCGTATAAAATCAAGGAGTACAGAAAACA	11	51.3	7B-1904090-T10-3_576	[illegible]
T10-41	ASY1		TraesCS5B02G285900	5B-1328460-T10-41	forward	471,306,622	TGCCATGAAACCCTTCCTCTTGCTCTTCGTCGTCATCATAAGCATCTTCATCCTCTTGAATTACT	all baits failed stringency test		7B-1904090-T10-3_640	[illegible]
T10-41	ASY1		TraesCS5D02G294200	5D-1438450-T10-41	forward	391,048,886	AAATATATGATAGCATTTTTGAAAAAATATTAATAAATGAAAATTTTTGTAATATATGTTGAACA	13	65.4	7B-1904090-T10-3_704	[illegible]
T10-42	ASY3	ABD	TraesCS5A02G042200	5A-1272800-T10-42	reverse	37,980,680	TGCGGCTTCTGGGATAACAATAGACCAGATTGACTGAAGTTGAAGCTGATAAACAGGAGAA	14	60.9	7B-1904090-T10-3_768	[illegible]
T10-42	ASY3		TraesCS5B02G045200	5B-1370900-T10-42	reverse	50,516,417	TGCAGCTTCTGGGATAATGATAATAGACCAAATCAACTGAGGTTGAAGCTGATAAACAGGAG	19	86.8	7B-1904090-T10-3_1088	[illegible]
T10-42	ASY3		TraesCS5D02G050300	5D-1489900-T10-42	reverse	47,863,546	TGCAGCTTCTGGGATAATAATAGACCAAATTAACTGAAGTTGAAGCTGATAAACAGGAGAAC	17	80.5	7B-1904090-T10-3_1152	[illegible]
T10-43	p31 COMET	ABD	TraesCS3A02G198600	3A-2150170-T10-43	forward	314,624,424	TCCCGGTCGGTCGTCCGGTGGCTTGGACTTTTCCTTCAGCCCTCCTTCTCCGTATTCTCGTCCAT	7	31.8	7B-1904090-T10-3_1216	[illegible]
T10-43	p31 COMET		TraesCS3B02G227200	3B-0754730-T10-43	reverse	322,719,453	GGGGATGATCGTGGGATGCTCCGCATCATGGTGCCACTGAAACAGCCGCTTCCCCTAGTGGC	12	55.1	7B-1904090-T10-3_1280	[illegible]
T10-43	p31 COMET		TraesCS3D02G196500	3D-0928920-T10-43	forward	197,991,422	ATTGCATGTGTAGGTTGATTGCTTGACTTGTGCTAAGTTGCTAAAATCTGCCAAGACTTAAAAT	6	25.9	7B-1904090-T10-3_1472	[illegible]
T10-44	RBR	ABD	TraesCS4A02G143200	4A-1007510-T10-44	reverse	235,662,516	GGATTATACGAATTGTGCGGTCAATCACGAGGCTTCAGAGGCATCAAACGGCGTAGGCTAAG	13	60.9	7B-1904090-T10-3_1536	[illegible]
T10-44	RBR		TraesCS4B02G162200	4B-1071540-T10-44	4B	324,979,349	TGATTCAACCAAAAGACCAAGGCGATTTATTTCCGGAGCAAGTGATGGCCTGGCAACAACCA	15	78.2	7B-1904090-T10-3_1580	[illegible]
T10-44	RBR		TraesCS4D02G159600	4D-1118460-T10-44	reverse	230,493,346	GGATTATACGAATTGTGCGGTCAATCACGAGGCTTCAGAGGCATCAAACGGTGTAGACTAAG	5	28.7	7D-1971220-T10-3_192	[illegible]
T10-45	C-PH1	AB	TraesCS5A02G381700LC	5A-1257070-T10-45	forward	472,221,246	GATGTCTGTACTCAGACATGTTACGCTTCTGCATGTGCTTTGATTTGTATGCTATGATTGTTGGG	1	7.1	7D-1971220-T10-3_256	[illegible]
T10-45	C-PH1	no D	TraesCS5B02G256100	5B-1319020-T10-45	forward	439,001,870	CGGCGGTTAATTCCATTATTGGAGCCCAATCAGATTCGATGGATGCACGCGGTCCGGGATCTA	8	37.1	7D-1971220-T10-3_512	[illegible]
T10-46	PCH2	ABD	TraesCS2A02G325700	2A-0269760-T10-46	reverse	555,830,969	GGTGGCCAGGCTCCGCGAGGGAGCGGAATGGAGAAGGTTCTAGGGTTTTGGGTTTGGGGA	10	52.9	7D-1971220-T10-3_576	[illegible]
T10-46	PCH2		TraesCS2B02G356200	2B-0435980-T10-46	forward	508,241,300	TCATTTTTTTTATCAAAAACTATTAACTTGGGATCTACTCCCTTCGCCCCGTAATGTAAGACATT	8	43.3	7D-1971220-T10-3_640	[illegible]
T10-46	PCH2		TraesCS2D02G337000	2D-0513310-T10-46	forward	430,454,984	TTCAAGAATTTGAAAAAAGTTCACGCACTCAAAAAATATTTGTGGATTTTATTTTATTTTGTAT	20	88.5	7D-1971220-T10-3_704	[illegible]
T10-47	PSS1	ABD	TraesCS7A02G316600	7A-1789360-T10-47	forward	452,646,799	GCCTTGCACATGGCCATGTGATGGTGCATGTAAGGTTGGCTGCATGATTGGGGTATAGTCATC	11	54.2	7D-1971220-T10-3_768	[illegible]
T10-47	PSS1		TraesCS7B02G217600	7B-1875310-T10-47	reverse	400,943,271	CCTGGCATTCAGATAGGTGTCAATTTGGGCACTAGAAAAATTCAAGTGACACAGCGGCTAAC	3	17.9	7D-1971220-T10-3_832	[illegible]
T10-47	PSS1		TraesCS7D02G313500	7D-1971250-T10-47	reverse	397,210,262	CCTGGCGATTCAGATAGGTGTCAATTTGGGTGGTAGAAAAATTCAAGTGACACAGCGGCTAA	7	32.9	7D-1971220-T10-3_896	[illegible]
T10-48	MPA1	ABD	TraesCS5A02G168500	5A-1203580-T10-48	forward	359,019,904	CCATGGCGAACGCGCGGGGCTGGCTAGGCTAGGCTAGGGTTTTGCTGTCACCGGCGAGGGA	10	57.4	7D-1971220-T10-3_960	[illegible]
T10-48	MPA1		TraesCS5B02G165300	5B-2081210A-T10-48	forward	306,425,185	CGGTCCGTGGTCGGCGCCCCCTCTCCCCATGCGGCACCATCGTGAAGGCGTTCAACGACGGC	18	51.6	7D-1971220-T10-3_1152	[illegible]
T10-48	MPA1		TraesCS5D02G172800	5D-1395040-T10-48	forward	270,200,347	GAAGGACTGCGGAAGTCCCTGGAGCATTCACTTGCCCTTGTCCTTATTGAAGAAACCCATGG	8	43.3	7D-1971220-T10-3_1216	[illegible]
T10-5	PRD2	ABD	TraesCS7A02G276600	7A-2095770A-T10-5	forward	289,118,782	TAAGGTTAGATATGACATAATTATGTCACATTTAGATGTGTCTTAGACAGACAGATCCTTGGTT	3	18	7D-1971220-T10-3_1280	[illegible]
T10-5	PRD2		TraesCS7B02G174300	7B-1928490-T10-5	forward	245,171,947	CAAAAATACCTTGCTGCAATTTATTTATCATTATTTTATTTTGTGTTTTTGCTTATCTATCTATCAC	6	35.8	7D-1971220-T10-3_1408	[illegible]
T10-5	PRD2		TraesCS7D02G276600	7D-2018900-T10-5	forward	264,970,767	TGCAGAAAAACCTGATCCACCACTGATCCGCTGCTGTGGCTACCGTTCGTCGTCGAGCGCCTG	9	50.4	7D-1971220-T10-3_1472	[illegible]
T10-6	PRD3	ABD	TraesCS2A02G315000	2A-0267760-T10-6	forward	540,048,304	AAAAGCATGAAGCAAAAGTCCCAAAAGCTTAGAGCCGAACAGGACAAACTAAACGATAAAT	8	46.6	2A-0313060-T10-4_0	[illegible]
T10-6	PRD3		TraesCS2B02G333500	2B-0394520-T10-6	forward	476,718,280	GCCATGGAGCCGCGAGGGAACCCGGCGAGTTCCCTCCTAAATATGGTAAGCTTCCCCTGTTCC	4	24.9	2A-0313060-T10-4_128	[illegible]
T10-6	PRD3		TraesCS2D02G313300	2D-0526040-T10-6	forward	402,938,087	AGCTTAGAGCCGAGCAGGACAGACTAAACGATAAATGGACCGAGGTCCTGGGCGTCAAGCA	12	64.9	2A-0313060-T10-4_192	[illegible]
T10-7	RAD50	AB, D	TraesCS5A02G081400	5A-1272160-T10-7	reverse	104,637,605	CTTGGCCCCCAAATCCTATCCCTCCTCCAACTCCTCCCCTCCTGTCCCACCTCCTCTCTTGCTTCG	11	54.6	2A-0313060-T10-4_256	[illegible]
T10-7	RAD50		TraesCS5B02G085000	5B-1372400-T10-7	forward	107,720,185	TCTTCCTTATAAATGTGTGTGTGTACTCCCTCCGTTTCTTTTTAGTCTGCATATAATATTTGGTCG	3	17.9	2A-0313060-T10-4_320	[illegible]
T10-7	RAD50		TraesCS7D02G276600	7D-2018900-T10-7	forward	264,970,767	TGCAGAAAAACCTGATCCACCACTGATCCGCTGCTGTGGCTACCGTTCGTCGTCGAGCGCCTG	9	50.4	2A-0313060-T10-4_512	[illegible]
T10-8	MRE11	BD	TraesCS2B02G559100	2B-0436200-T10-8	reverse	753,097,180	TGCCTCTGCTGGTTCCAACTAGAGCCAAAGCACTTGTCTCCGATCAACGGCTCACTGCAACGA	18	83.1	2A-0313060-T10-4_576	[illegible]
T10-8	MRE11		TraesCS2D02G531600	2D-0514110-T10-8	reverse	617,396,265	TGCTCTGCTGGTTCAAACTTGAGCCAAAGCACTCGTCTCCGATCAACGGCTCACTGCAACGAC	17	80.5	2A-0313060-T10-4_704	[illegible]
T10-9	NBS1	ABD	TraesCS1A02G162200	1A-0032970-T10-9	forward	291,242,184	GGCAACTAATCAAGAGCGAGGGCAGTGCACATCAGAGCTACGCTGAATGTAGTGAGCCAAC	2	14.1	2A-0313060-T10-4_768	[illegible]
T10-9	NBS1		TraesCS1B02G178800	1B-2100890-T10-9	forward	324,336,564	TAGGTTAGCTGTGGCTGAATTCGCCCTCAGCCTCTTAGGGTCATGAACCTTTTCAGGCGTGCT	12	68.2	2A-0313060-T10-4_832	[illegible]
T10-9	NBS1		TraesCS1D02G159000	1D-0184340-T10-9	forward	224,394,722	TAGCAGTTGAAAAGATGGTTGCCTGGGAGCCACGTTCTGGTGCTCCAGCAAGCCCTTCATGT	15	72.9	2A-0313060-T10-4_896	[illegible]
										2A-0313060-T10-4_960	[illegible]
										2A-0313060-T10-4_1024	[illegible]
										2A-0313060-T10-4_1088	[illegible]
										2A-0313060-T10-4_1152	[illegible]
										2A-0313060-T10-4_1216	[illegible]
										2A-0313060-T10-4_1280	[illegible]
										2A-0313060-T10-4_1344	[illegible]
										2A-0313060-T10-4_1408	[illegible]
										2A-0313060-T10-4_1472	[illegible]
										2A-0313060-T10-4_1536	[illegible]
										2A-0313060-T10-4_1580	[illegible]
										2B-0417700-T10-4_0	[illegible]
										2B-0417700-T10-4_132	[illegible]
										2B-0417700-T10-4_198	[illegible]
										2B-0417700-T10-4_594	[illegible]
										2B-0417700-T10-4_660	[illegible]
										2B-0417700-T10-4_726	[illegible]
										2B-0417700-T10-4_792	[illegible]
										2B-0417700-T10-4_858	[illegible]
										2B-0417700-T10-4_924	[illegible]
										2B-0417700-T10-4_990	[illegible]
										2B-0417700-T10-4_1449	[illegible]
										2B-0417700-T10-4_1515	[illegible]
										2D-0545450-T10-4_128	[illegible]
										2D-0545450-T10-4_256	[illegible]
										2D-0545450-T10-4_576	[illegible]
										2D-0545450-T10-4_704	[illegible]
										2D-0545450-T10-4_896	[illegible]
										2D-0545450-T10-4_960	[illegible]
										2D-0545450-T10-4_1024	[illegible]
										2D-0545450-T10-4_1088	[illegible]
										2D-0545450-T10-4_1152	[illegible]
										2D-0545450-T10-4_1216	[illegible]
										2D-0545450-T10-4_1408	[illegible]
										2D-0545450-T10-4_1536	[illegible]
										2D-0545450-T10-4_1580	[illegible]
										7A-2095770A-T10-5_0	[illegible]
										7A-2095770A-T10-5_928	[illegible]
										7A-2095770A-T10-5_994	[illegible]
										7B-1928490-T10-5_384	[illegible]
										7B-1928490-T10-5_512	[illegible]
										7B-1928490-T10-5_576	[illegible]
										7B-1928490-T10-5_640	[illegible]
										7B-1928490-T10-5_832	[illegible]
										7B-1928490-T10-5_1024	[illegible]
										7D-2018900-T10-5_320	[illegible]
										7D-2018900-T10-5_384	[illegible]
										7D-2018900-T10-5_512	[illegible]
										7D-2018900-T10-5_576	[illegible]
										7D-2018900-T10-5_704	[illegible]
										7D-2018900-T10-5_768	[illegible]
										7D-2018900-T10-5_832	[illegible]
										7D-2018900-T10-5_1024	[illegible]
										7D-2018900-T10-5_1152	[illegible]
										7D-2018900-T10-5_1344	[illegible]
										7D-2018900-T10-5_1408	[illegible]

7D-2018900-T10-5_1472	AAGACGTATCACCGTATACTTCGTTCGTTCGGTTGTTGCTTTATACTTCTTTCGTCTCATAATATAATATAAGAACGTTTTTGACAACACTTGTATTGGGAGTTCATTTGGTTATATAGG
7D-2018900-T10-5_1536	TAATATAAGAACGTTTTTGACAACACTTGTATTGGGAGTTCATTTGGTTATATAGGTGGTATAGGGGAGTTTTTTTAATTACGAATTTCAGATTGCCCTCTCTTGTGCTTCCGTTTCTCA
2A-0267760-T10-6_0	AAAAGCATGAAGCAAAAGTCCCAAAAGCTTAGAGCCGAACAGGACAAACTAAACGATAAATGGACCGAGCTCCTCGGCACTGAGCAAGAGCTTGAGGAATGATTCGCAGGGAATAAC
2A-0267760-T10-6_64	CCGAGCTCCTCGGCACTGAGCAAGAGCTTGAGGAATGATTCGCAGGGAATAACCAGTACTACCCGCGAAGGAAGCTGCTCCCAGAGTTTGATGAGGAGGTTGTCGGCAACCCGCTATC
2A-0267760-T10-6_256	CCCCTGAAAAACCTCACCATTACGACAAGGATCCATTTCTGCCTTTTGAAACCATTGCAGAAGAAATTGGCATCAAGACCAGCTGCTCGTCTCGGTCCATCTATGGATCTAGGGAGCGCC
2A-0267760-T10-6_448	GGACCGTAACGACATCGGACATTCGGCAACCAACAACTCGGGTCCGAGGAGCTGCTCGCTCCATGTGCaggttatgcagcaccaattgtcataagggtttaaacccatcaatactgaaca
2A-0267760-T10-6_704	agcttgcccgagaactccatcggtaattgggaggacctcgaagatgccttacgAGCAAATTTTCGCCAAAACCGAAGGGAGGAGCTCGGACAGACAGAGGTGGGAGGATGGATGCCGACA
2A-0267760-T10-6_768	GCCAAAACCGAAGGGAGGAGCTCGGACAGACAGAGGTGGGAGGATGGATGCCGACACCGGGAATGGTGGCTGCCGGGCCGGGGTGGCGGCCGGTGCTCAGAGGTGAGGAGAGGT
2A-0267760-T10-6_896	ATTCTCGAAATGTGAGAATATTAAAGTTATAGGAATAGAATGTCATCTCCTCTTATACCCTACGGGATTAGGTGAAGTGTTTGATAGCACATGAAAAATAAAGGAATTCTAAAAAATGTT
2A-0267760-T10-6_960	GGATTAGGTGAAGTGTTTGATAGCACATGAAAAATAAAGGAATTCTAAAAAATGTTTGAGTGGATGGAAAGTTTTCATTCCTACAAATCAAACGAGCATCATAAAAAATACTCCCTTCAT
2A-0267760-T10-6_1344	TGGACTTGTATATAAAAATGGAGGAAGTACCCAAGGTTTCAAATCCTTTAAATATTCTAAATTCCTTTGTATCAAAGGAGCCTTTTTCAAGGATCCGCTGTATGCTCAGCAACGCAAACC
2A-0267760-T10-6_1408	CTTTGTATCAAAGGAGCCTTTTTCAAGGATCCGCTGTATGCTCAGCAACGCAAACCCCGTTCCGCACACCGCCGACGCTGCCTGCCTGAGCCCACATAGACCACGGCACCTGGGAAGGG
2B-0394520-T10-6_64	ACGTCCCCCTGTTCGTCTGCTTCCCCTGTTCCTCTATTCGTCCCATGGTTAGGTTTTCTTGTGCATATCACATGGCAGCCACGATCAGTAGGGTTTCTTGTGCTACTTGGTCTCAATTTC
2B-0394520-T10-6_448	ACTTCAGATAAGCACATGTATCATAACCCTGCAAAACACATAATACCAATTTGTTCAGTACAACAATTTGTTCAGTGCAACCTATAAACCCTGAAAATAAACAGCACACCTTTTTCCATA
2B-0394520-T10-6_1344	TTTTGTATGAATCCGTTTCCACCCCTACAACTTTATGTACAATGTTGGTATTAACTCTAAAAAAAAACCTAAGGAGCCTTTTTCAAGGACGTGCTGTATGCTCAGCAACGCAAACCCGTTT
2B-0394520-T10-6_1408	AACCTAAGGAGCCTTTTTCAAGGACGTGCTGTATGCTCAGCAACGCAAACCCGTTTCGCACACCGCCCACGTTGCCTGCCTGAGCCCACATGGCCGGCATCCTATAAGACCACGGCACCT
2D-0526040-T10-6_0	AGCTTAGAGCCGAGCAGGACAGACTAAACGATAAATGGACCGAGGTCCTGGGCGTCAAGCAAGAGCTTGAGGAAATATACGCAGAGAATAACCAGTACTACCCGCGAAGGAAGCTGC
2D-0526040-T10-6_192	CTCGGGGCTGAGATAGGCCAACAGGTGGATATCCTGATCCCCCGAAAAAACCTCACCGTTACGACAAGGATCTACTTCTTCCTTTGGAACCACATGATTTGCGGAAGAAATTAGCATCAA
2D-0526040-T10-6_256	CAAGGATCTACTTCTTCCTTTGGAACCACATGATTTGCGGAAGAAATTAGCATCAAGACCAGCTGCCCCGTCTTGATCCATCTATGGATCTAGGGAGCGCCCACCGGCTGGACATGCGGG
2D-0526040-T10-6_384	CTTGGTTAGCCAAACATGGCAATACACGTGATTATACATGTGATCGGACCGTAACGACATCGAACATTCAGCAAGCAATAGCTCGGGTCTGAGGAACTACTcaccccatgtgcttttcag
2D-0526040-T10-6_704	cctagaacttcatcggtaattggaagggcctcgaagatgccttccgagcaaatttccaCCAAAACCGAAGGGGGAGCTCCAGCAAACAGAGGTGGGAGGGTGGATGCTGGAACCGGGAGT
2D-0526040-T10-6_768	CCGAAGGGGGAGCTCCAGCAAACAGAGGTGGGAGGGTGGATGCTGGAACCGGGAGTGGTGGTCGGCGTTTATGGAGGGTGGCCACCGGGGCCGGGGTGGCGGCCGCGCTTAGAG
2D-0526040-T10-6_960	AATTTTATAGAAGAGAGCAAAAGATTTACAAGAAGCCATCGAAGGATGCGAACATCGATCGATGGAATACCTCCACGCACCCACTAGACTAAAACTAACTACTCACAAAACGATCTAAAC
2D-0526040-T10-6_1024	GAATACCTCCACGCACCCACTAGACTAAAACTAACTACTCACAAAACGATCTAAACCTCCTACACCGTCTTTGGCATCCCTTCACTCCCTAATCTCATCGATGATGTACATGGCCAGACC
2D-0526040-T10-6_1088	CCGTCTTTGGCATCCCTTCACTCCCTAATCTCATCGATGATGTACATGGCCAGACCCGACGGTTGCTTGAACTGTCCAGGACGACTGAAAACTCAGGCATTCTTTTGATATCACATTATA
2D-0526040-T10-6_1152	GCTTGAACTGTCCAGGACGACTGAAAACTCAGGCATTCTTTTGATATCACATTATAGGAATAGAATGTCATGTCCTCTTGTACTCTACGGGATTATGAGAAGTATTTGATAGCACATAAA
2D-0526040-T10-6_1216	ATGTCATGTCCTCTTGTACTCTACGGGATTATGAGAAGTATTTGATAGCACATAAAAAACAAAGGTATTTTAAAAAAATTGAGTGGATGAAAAGTTTCCATTCCTACAAATCAAACGAGC
2D-0526040-T10-6_1344	AAAAAACTAAGGATTCAAATCCTTTAAAAATTCGAAATTCCTTTGTATCAAAGGAGCCTTTTTCAAGGATCCGCTGTATGCTCAGCAACACAAACCCGTTTCGCACACCGCCCACGCTTC
2D-0526040-T10-6_1408	AAGGATCCGCTGTATGCTCAGCAACACAAACCCGTTTCGCACACCGCCCACGCTTCCGGACTGAGCCCACATGGCCGGCAGCCTATAAGACCACGGCACCTGGGAAGGGGCCCAAAGG
5A-1272160-T10-7_0	CTTGGCCCCCAAATCCTATCCCTCCTCCAACTCCTCCCCTCCTGTCCCACCTCCTCTCTTGCTTCGGCTCCTTCGTATTCCCTCCCACGCCGGCGCCGTCTCCGGCGTTGCTGCTTGCGT
5A-1272160-T10-7_64	CGGCTCCTTCGTATTCCCTCCCACGCCGGCGCCGTCTCCGGCGTTGCTGCTTGCGTCGGCGTGTCACAATGATCTATCCTCTGGGCGTGGGGGGCGAATCCATCTATTGCTCCGCCATGG
5A-1272160-T10-7_128	CACAATGATCTATCCTCTGGGCGTGGGGGGCGAATCCATCTATTGCTCCGCCATGGACGGGGGAGACGGGCGGGCGAAGGAAGGAGGCGGAGAAAATGGTGGGGAAGAGGCGTTCT
5A-1272160-T10-7_320	GAAACTGGACCTCCGGTGGAAAAAAAAAACAGAAAACCTCACCCTGAGCACCCGTTAGATCGGAGTTGGATGGCACAGATCTTGGTGCAGGAATCTGTAGGAACAAGTACAGGATTCT
5A-1272160-T10-7_384	GTTGGATGGCACAGATCTTGGTGCAGGAATCTGTAGGAACAAGTACAGGATTCTGTGCGAAAGAAACCCTTCCTTTTAGTTATTTTAAGCATTTCTAGCAGATCCCATATAATTCCGGCG
5A-1272160-T10-7_448	AACCCTTCCTTTTAGTTATTTTAAGCATTTCTAGCAGATCCCATATAATTCCGGCGAACTGTGTTTTTTTGTCGGTTTACGGGACGGGCCAAAAATATGGCCCGAACAGGTCCGCAAACA
5A-1272160-T10-7_512	TTTTTTGTCGGTTTACGGGACGGGCCAAAAATATGGCCCGAACAGGTCCGCAAACAAAGATCTGGCCCGTAAAAAAATTTACAAGCGGCTCGTAAACCAGCCGGCATTGCAGTATATATG
5A-1272160-T10-7_576	GCCCGTAAAAAAATTTACAAGCGGCTCGTAAACCAGCCGGCATTGCAGTATATATGCGGGCCCGGAGTCATTTTAGGGTTTCCGTTCGGCATTCCCCTTATCCATCTCCGCCGCAAATTT
5A-1272160-T10-7_640	GAGTCATTTTAGGGTTTCCGTTCGGCATTCCCCTTATCCATCTCCGCCGCAAATTTCAAAGTCCGGCGAGATCTCTACCCCTCAGGCAGAAAATTCCGACGCACTTGATCGTCAGATCTC
5A-1272160-T10-7_768	TGGCGTCCGCGGGTGCACGGCGTGGCGACGGCGGGGGCTTTGACTGGCACGATGGGGTTCGGGTGCGGTGGGTTCACCGGACGCAAGCGTCGCAACGACGACGAGGCCAATAGCT
5A-1272160-T10-7_896	TCCTGTGTCGGAGAAGCGTAGGGAGCAGGCACGCACCCGCGACGCGAAGTCTCGCAGCGCTGCGACTTGTCAGCTGCATTCGGATTTCCTTGAAGAAGAGAGGATGATGCAATACAGT
5B-1372400-T10-7_1216	AACTAAAAAAAAAAAACTAAAAGCGAGGGTTCCTTTCCCACAAAATCCTGTACTTGTTCCTACAGATTCCTGCACCAAGATCTGTGCCATCCAACTCCGATCTAACGGCTCACTGCTCAGG
5B-1372400-T10-7_1280	ATTCCTGCACCAAGATCTGTGCCATCCAACTCCGATCTAACGGCTCACTGCTCAGGGTGAGGTTTTCTGTTTTTTTTTTCCACCGGAGGTCCAGTTTCTATCCTAGTCGCCCCCGGTcca
5B-1372400-T10-7_1344	TTCTGTTTTTTTTTTCCACCGGAGGTCCAGTTTCTATCCTAGTCGCCCCCGGTccaaagtccaaaccctaacccccagaggccaaaatcccaaaattccaaatAGAACGCCTCTTCCCCA
5B-1372400-T10-7_1408	accctaacccccagaggccaaaatcccaaaattccaaatAGAACGCCTCTTCCCCACCATTTTCTCCGCCTCCTTCCTTCGCCCGCCCGTCTCCCCCGTCCATGGCGGAGCAATAGATGG
5B-1372400-T10-7_1580	GCAACGCCGGAGACGGCACCGGCGTGGGAGGGAATCCGAAGGAGCCGAAGCAAGAGAGGAGGGGGGCGAGGGGGGACAGGAGGGGAGGAGTTGGAGGAGGGATAGATTTGG
7D-2018900-T10-7_320	AACAACTTTGAGCACGTCACGTCCTAGGTGCGTTCCTATGCATATGTCATTATGTGCATGAAGCAAATAGCTAGATCCAGTATTGACATTAACTAAACTGAATTAGACTTCAAGATACTT
7D-2018900-T10-7_384	AAATAGCTAGATCCAGTATTGACATTAACTAAACTGAATTAGACTTCAAGATACTTTTTATTTTCTATTAGTGCCACATCTGTCACAATTTATATCCACAATTACTCACGCTTGTCGTCG
7D-2018900-T10-7_512	AAAAACTCAAGGTCTATCCGAAGTAAATATATTTGTCGCTGCATTACTTAGAGCCAATAATATATGTACATGTGTTTTTCAACTATTTAGAGATCTAGATAGAGATACTATGTGATAGTA
7D-2018900-T10-7_576	TGTACATGTGTTTTTCAACTATTTAGAGATCTAGATAGAGATACTATGTGATAGTATATCATTATTGCTAAAAGGTTTGCTTCCACTGAGCATGTATGAGTATGCGCAGATAATTTCACA
7D-2018900-T10-7_704	GAAGTTTGTTTAGTTTTCAGACCTAAGTTAAGTTTATTTTGATCCAAATCCAACAATAAAATTTGGATAATTTCTCAATGTTATCTACAATGGGTGGCATGTATTTATACCATGACTAAT
7D-2018900-T10-7_768	GGATAATTTCTCAATGTTATCTACAATGGGTGGCATGTATTTATACCATGACTAATCAGCTTGATCAATAATGTATTTTTGTATTTTTGCAGTTTCTTTTTTTTTTTGCGGAAGAAGGAA
7D-2018900-T10-7_832	TCAATAATGTATTTTTGTATTTTTGCAGTTTCTTTTTTTTTTTGCGGAAGAAGGAACTTTCATTAATCAAACGGGATGATTACATTTAGACTTAACTATGCTACTATCTCTTCGGGGAAG
7D-2018900-T10-7_1024	TGTCGAGTGATCTTCAGTAGACTAATGTTCCTCCTGCATATGTATTTTTGTGGTTCATATATGGTGAAAATTTTATATGTCAGCTTTCACATACACATATCTGAGTAATACCACCTCCGA
7D-2018900-T10-7_1152	AAGTATCACAGTCTTGAACTAAACCCAGTTTAAAACTGCGACACTTATTTATTTTAGATAGGAGGGGGTATCATTTTTATTTGTTTTTCAAGGATGAGTGTTGCATCTCAATAGTAGCA
7D-2018900-T10-7_1344	TATAGTTCACCACGGGAGGTAACAGTCAAATACCATATGAATTGTCAAATTCATTTTGTACCTGTTGCAATGCACGGACTCTTTTGCTAGTAATAATATAAAGCAATAAAAAAATTATACG
7D-2018900-T10-7_1408	TTGCAATGCACGGACTCTTTTGCTAGTAATAATATAAAGCAATAAAAAAATTATACGATACGATGAAGACGTATCACCGTATACTTCGTTCGTTCGGTTGTTGCTTTATACTTCTTTCGTC
7D-2018900-T10-7_1472	AAGACGTATCACCGTATACTTCGTTCGTTCGGTTGTTGCTTTATACTTCTTTCGTCTCATAATATAATATAAGAACGTTTTTGACAACACTTGTATTGGGAGTTCATTTGGTTATATAGG
7D-2018900-T10-7_1536	TAATATAAGAACGTTTTTGACAACACTTGTATTGGGAGTTCATTTGGTTATATAGGTGGTATAGGGGAGTTTTTTTAATTACGAATTTCAGATTGCCCTCTCTTGTGCTTCCGTTTCTCA
7A-1760100-T10-8_64	CGATAGCAGCAAGTCCTCTGTCTGGTCAACCAATATGCTAGTGTCGTGCTAACTAAGGCGATGCACGACCTATGGTAATTTTTCCGAGACCAAGCTCCAAATCCAAATAAAATGCATAAC
7A-1760100-T10-8_128	ACGACCTATGGTAATTTTTCCGAGACCAAGCTCCAAATCCAAATAAAATGCATAACTTCGCCTCTTTTAAGGGCACAAGACGAGATCAAATTAAACTATATCGAGTTGTACCTCTTTCAA
7A-1760100-T10-8_320	TACAGATGCATGCCAAAGTCAGCACGTGTCGCGCTGGGATAAACTGCAAGGCAACAACAACAACAGTTTCTGCCGCAAGGTATACAGATGCCAAAGTCAACACATGCCGCGTTGCGATA
7A-1760100-T10-8_448	GCAACAACAACAATAGTTTCTGCCGCAATGCATACAGATGCCAAAGTCAGCACGTGCCGCGTTGTGATAAACTGCAAGGCAACAACAACAATATTTTCTGCCGCAATGTATACAAATGCC
7A-1760100-T10-8_512	TGATAAACTGCAAGGCAACAACAACAATATTTTCTGCCGCAATGTATACAAATGCCAAAGTTCACAGCGTTTGTTGTGTTGCGACTTACTGCTGGATAACAAGACTAGTTTCTGCCTTCT
7A-1760100-T10-8_576	CAGCGTTTGTTGTGTTGCGACTTACTGCTGGATAACAAGACTAGTTTCTGCCTTCTGGCCTTTTAGGAAACCGGAATAATTCTAGGAGCATGCATATTGATATTGGATCAACAACCAACA
7A-1760100-T10-8_640	AGGAAACCGGAATAATTCTAGGAGCATGCATATTGATATTGGATCAACAACCAACACACACACACAACATATCAGAAACTAACCCTCGCTTGACAAGGTATACATAGCCCTGGTACCAAA
7A-1760100-T10-8_704	CAACATATCAGAAACTAACCCTCGCTTGACAAGGTATACATAGCCCTGGTACCAAATCCCGCGACATGAGAACAAATAAAACTAAAACCAAGTCACGCTGAATGCTGAACACCCACCTAT
7A-1760100-T10-8_768	CATGAGAACAAATAAAACTAAAACCAAGTCACGCTGAATGCTGAACACCCACCTATCCCGCTGCCATCACAATCATTTTCACATTCATCACCCCTACCACAGTTCAACCAGTCATGTGAG
7A-1760100-T10-8_832	CATCACAATCATTTTCACATTCATCACCCCTACCACAGTTCAACCAGTCATGTGAGCCAGCGTATGAACATCTATCTAGAGTTCTAAACTATCTAAACCAAATCGAGCAGGCAGTCCTGT
7A-1760100-T10-8_896	TGAACATCTATCTAGAGTTCTAAACTATCTAAACCAAATCGAGCAGGCAGTCCTGTAGGTAAAATGGTATATAAACTACTCCATTCAGATGAGGGCATTGAATAGCATGAAACAATTGGG
7A-1760100-T10-8_960	TGGTATATAAACTACTCCATTCAGATGAGGGCATTGAATAGCATGAAACAATTGGGGGAGGGGGATCTGGCgagggatgtacggacggacggacggacCTTGCCTGCCGCCGGTGAGCTT
7A-1760100-T10-8_1088	TGCCCTTGTCGACGAACTTGGCCAGGTCCAGCACCGTCTCCTTCCGCCCCGACTGCACAGCACAACAAAGACACAAACAAGAAACAACCCACTCCAATCAGATGGGATCGAAACTCCAA
7A-1760100-T10-8_1344	CgctctgctggctgctgctgtgctgtcctCCTCTCTCCTACCTTTTCTCCTTTTCAGAGTTTTTTTTCCTCTCCTCTTAATTCGTTCATTTTTTGACGGTTATGCGCTGGGCCTGTTTCA
7A-1760100-T10-8_1472	GGGCCTTGAGCGCGGCCCATCTCCGCGTGtcttccttcccttctactcttctcttctcttcCTCCTTCCAACGGAAGCCAGAGACGCACACGAACTGTCACTCACTCCCACTCGCTACTG
7A-1760100-T10-8_1536	CTTCCAACGGAAGCCAGAGACGCACACGAACTGTCACTCACTCCCACTCGCTACTGCCCGTCTGCCCCTGCTCATCCTCCCATCTCACTCGGCACACCTCCAGATCGGGAAGGCGGGCGG
2B-0436200-T10-8_0	TGCCTCTGCTGGTTCCAACTAGAGCCAAAGCACTTGTCTCCGATCAACGGCTCACTGCAACGACAAAAGAAACACAACCATGGTTAGCTGACACCGAATATGTTATATACCAACGACTAG
2B-0436200-T10-8_64	AAAAGAAACACAACCATGGTTAGCTGACACCGAATATGTTATATACCAACGACTAGAGTGGCGGGAGCACTAGTGAGGCTTGGAGGCGTTCCGTCGAACAGGTGATGGGCGCCAGAAC
2B-0436200-T10-8_256	CGCGGAGACACAGGATTCTACGGCGAGAGAAGACCAACAATCACCTTCGCTTCTCCTGCGGCCGCCTCCACCGCCGTCGATGGAAGAAGCCAACAGCGGCGAGCGACCGGGCGCGAA
2B-0436200-T10-8_384	CAGGCGCCAGTCCTCAGCTCGACCGACGGACCGCTCGCACCCTCTCCTGCACGCACCGGCGGCGGCTGCTGACGAGGGACGACCCGAGCGGCGCGGCGCCACCGGTGTCAGCGGCG
2B-0436200-T10-8_576	TCTTTCCTTCGCTACGTCGCCAGGCTAAACCGGCCCGCAAGCCCTGGGCCCTGACTGAACCCCGGCCCATGGGGCAGCTCCACCTCCACCTGGGCCGCCCAGGATAGAACCTCTCCCTC
2B-0436200-T10-8_640	GCCCATGGGGCAGCTCCACCTCCACCTGGGCCGCCCAGGATAGAACCTCTCCCTCCCCTGTCCGTCCGTAGCCCATAGCCACGAGCTGCTCCCGGCTTCCTTCCTCCCTACGCGAGAGTG
2B-0436200-T10-8_704	TCCGTAGCCCATAGCCACGAGCTGCTCCCGGCTTCCTTCCTCCCTACGCGAGAGTGTTCCTTCCAGAGCTGCGGCGGCGATCGATTTCTAGGGCAAACTCCCCGTCGGTTCGCGCGGTCG
2B-0436200-T10-8_768	AGAGCTGCGGCGGCGATCGATTTCTAGGGCAAACTCCCCGTCGGTTCGCGCGGTCGAGGCAAGAGCAGGTCGGCGGAGGAGGATCCGGCTGGTGAGATGGTAAGCTGGTGCGCGGG
2B-0436200-T10-8_1024	CGGCCGCCAAGGTTCGTTTCGATCCGCACAGCCGAAACCCCTCTTTTTCTTCTATCCCTGTGTTATGATGCATTGTCAGATCCGCTAGAGGCGCCCCCGATTTGTTTGCCTTAGGCTCTG
2B-0436200-T10-8_1088	ATGATGCATTGTCAGATCCGCTAGAGGCGCCCCCGATTTGTTTGCCTTAGGCTCTGTCGCCGGCGCGGTTTTTGGTGTAGAGATTGGTGGTACCAATGTCACTGACACAACTTGTTACTC
2B-0436200-T10-8_1152	GCGGTTTTTGGTGTAGAGATTGGTGGTACCAATGTCACTGACACAACTTGTTACTCTGGTCCTTGTCTGATTTAGTTGTTGCGTCTTGTGTTCCTGTAGAGTGGGTTTGTTTGCTGTTTT
2B-0436200-T10-8_1216	GTCTGATTTAGTTGTTGCGTCTTGTGTTCCTGTAGAGTGGGTTTGTTTGCTGTTTTCATTCTAGTCAGCAAACAAATGCCTCTGTGTAAGTTGTTTATCTAGTCTCCCCAGTGAGCAAAC
2B-0436200-T10-8_1280	TCAGCAAACAAATGCCTCTGTGTAAGTTGTTTATCTAGTCTCCCCAGTGAGCAAACAGGTGGGGTGCGAAAAGCAAAATTAAACCTGGTTCATGGTAATTCATCATGTGTCAGCCCGGTG
2B-0436200-T10-8_1344	TGCGAAAAGCAAAATTAAACCTGGTTCATGGTAATTCATCATGTGTCAGCCCGGTGTACGGACTTTTATCGCAAATCATTTGCAGGCTTGTGCTTGCTTGCACGTCTTTTTTGTTGGGCA
2B-0436200-T10-8_1408	TTTATCGCAAATCATTTGCAGGCTTGTGCTTGCTTGCACGTCTTTTTTGTTGGGCATGCTCGTCCATATATCTTGATGGGTGTGAGTATGAGGCGTCTGTTACCTTAGTATAGTTGACAA
2B-0436200-T10-8_1472	CATATATCTTGATGGGTGTGAGTATGAGGCGTCTGTTACCTTAGTATAGTTGACAAGTATTTAAGCTCGTATGAGCCGTCTCTTACCTTAGTACGTAGTTGAGAAGTATTTAAGCTTCTA
2B-0436200-T10-8_1536	GCTCGTATGAGCCGTCTCTTACCTTAGTACGTAGTTGAGAAGTATTTAAGCTTCTATGGTGGCCAGGACTATGAAACCCATGTACCTGTTTGTCTCCTGCCGTGTTCTTGCTAGCAGTCG
2B-0436200-T10-8_1580	TTTAAGCTTCTATGGTGGCCAGGACTATGAAACCCATGTACCTGTTTGTCTCCTGCCGTGTTCTTGCTAGCAGTCGTATACTGACGTGACATATGCATGTTTGCAGTTACAGGAACCTTG
2D-0514110-T10-8_0	TGCTCTGCTGGTTCAAACTTGAGCCAAAGCACTCGTCTCCGATCAACGGCTCACTGCAACGACAAGAGACACACAACCATGGTTAGCTGACAAGCGACAATACCAAGGAGGAGAGTGG
2D-0514120-T10-8_0	ATATTTTTTATGCTATCTAATGATATGCTATGTGTCAGTTTAGCACTATGGTGGTGATCATCGTTTTTCCTGCGAGGAATTTCCAACCTATTTATAATCAATCATGGCAGTGCAAAAAAC
2D-0514110-T10-8_64	AGAGACACACAACCATGGTTAGCTGACAAGCGACAATACCAAGGAGGAGAGTGGCGGGAGCACGATGGAGGCTTGGAGGCGTTCCGTCGAACAGGTGCTGGGCGCCAGAACAGCTG
2D-0514110-T10-8_256	GGAGACACCGGATTCTACGGCGAGAGAAGCACAAGAATCACCTTCGCTTCTCCTGAGGCCGCCTCCACCGCCACCGCCGTCGATGGGAGAAGCCCACCGCGGAGACCGACCTGGCGC
2D-0514110-T10-8_320	CCACCGCCACCGCCGTCGATGGGAGAAGCCCACCGCGGAGACCGACCTGGCGCGAAACAGCCGTCCGCAGGCGCTAGTCGGAGCTCGAGCGTCAGCCAGCCGACGGACCGGTCGAA
2D-0514120-T10-8_320	AGAGAATCGTATATCAAAAGGATTAAACCTATAAACATACGGACAAAGACTGGATCAAAAACGGATCCACCGAATACCAACACCGACTTAATCCCGCAAGACGCGAGCAAACACGAACCT
2D-0514110-T10-8_384	CCGCAGGCGCTAGTCGGAGCTCGAGCGTCAGCCAGCCGACGGACCGGTCGAACCCTCCCCTGCACGCACCGGCGGCGGAAGAGTCGCGGCTGCCGACGACACCGGTGTCAGCGGCG
2D-0514120-T10-8_384	ATCCACCGAATACCAACACCGACTTAATCCCGCAAGACGCGAGCAAACACGAACCTCATGGGCAGTACTGGGTTGGCCATGCAGGTTTGTGATTTCGTTCGATTTTTCCTAGAAAATAAT
2D-0514120-T10-8_448	GTACTGGGTTGGCCATGCAGGTTTGTGATTTCGTTCGATTTTTCCTAGAAAATAATCTCTCTACCAGATTTTTCCTATTAGCTTGTATATTTGTTTTACTTTTCATTGTTTATTCATGAG
2D-0514120-T10-8_512	CAGATTTTTCCTATTAGCTTGTATATTTGTTTTACTTTTCATTGTTTATTCATGAGTTGTTTTTGAAAATCAGGAACATAATTTGAACTTTTGAACATTTCCTCAAATAAAAAATGTTTT
2D-0514110-T10-8_576	CCTTTCCTTTCTGGGTCGCCAGGCTAAACAGGCCCGCAACTACCGAACACCGGCCCGGCCTGCAATACGACCCTAGCTGGGCCGCCTCGGATAGATTCTTCTTTTCCGCCGTCCGTAGCC
2D-0514120-T10-8_576	GAAAATCAGGAACATAATTTGAACTTTTGAACATTTCCTCAAATAAAAAATGTTTTCTTCAAAATTGTGAGCATTCTTTTAAAATTCTTGAAGATTTTTCATATTCATGAACTTTTTAAA
2D-0514110-T10-8_640	ATACGACCCTAGCTGGGCCGCCTCGGATAGATTCTTCTTTTCCGCCGTCCGTAGCCCATAGCCACAGCCCACAGGTCCCGACCGAGCCGCCTCCTGCTTCCTTCCTACGCGAGAGTGTTC
2D-0514120-T10-8_640	TTGTGAGCATTCTTTTAAAATTCTTGAAGATTTTTCATATTCATGAACTTTTTAAAAACTTACGGAGATTTTCTAAGTCCGTGAACATTATAAAACTCGTTAACAGTTTTTAGAATTCAT
2D-0514110-T10-8_704	CAGCCCACAGGTCCCGACCGAGCCGCCTCCTGCTTCCTTCCTACGCGAGAGTGTTCCTTCCTGAGCTTGCGGCGGCAGAGGTTCTCGATTTCTAGGGCAAACTCCTCGCTCGGGTTCCC
2D-0514120-T10-8_704	GAGATTTTCTAAGTCCGTGAACATTATAAAACTCGTTAACAGTTTTTAGAATTCATTGATGTCTTTTCATATTCATGAACAATTTTAAAATTTATGAACATTTTTTAAATTCGTACATGT
2D-0514110-T10-8_768	GCTTGCGGCGGCAGAGGTTCTCGATTTCTAGGGCAAACTCCTCGCTCGGGTTCCCCCTCGGTCCGCGCGCTCGAGGCGAGAGCAGGTCGTCGGAGGAGCATCCGGGATGGTAAGCTTC
2D-0514120-T10-8_768	TTTCATATTCATGAACAATTTTAAAATTTATGAACATTTTTTAAATTCGTACATGTTTTCTAACTTTATGAATATTATTGGAAATTCAAATAAAATAAAATTAATGAATGCTTTTTCTAA
2D-0514110-T10-8_832	GCGCGCTCGAGGCGAGAGCAGGTCGTCGGAGGAGCATCCGGGATGGTAAGCTTCGTCTGGCCCCGTGGTTTCGCTGATTCGATTCGTGTGATGCGAGTCTTGTGTTGTTGTTTGTTTTG
2D-0514120-T10-8_832	TTTATGAATATTATTGGAAATTCAAATAAAATAAAATTAATGAATGCTTTTTCTAAAGTGTGTTAATGGAATGTTGGTTCTGATACTGTCATGCAGGACCGGTGACCCAAGCAGTGTCCA
2D-0514120-T10-8_896	AATGGAATGTTGGTTCTGATACTGTCATGCAGGACCGGTGACCCAAGCAGTGTCCACTTTGAAGGAAAAACTTTAAAGCTTTCTTGGGCCTCTATTTGTAGGGAGCTTAGTAATTGTAAT
2D-0514120-T10-8_960	GAAAAACTTTAAAGCTTTCTTGGGCCTCTATTTGTAGGGAGCTTAGTAATTGTAATTAGGATTGTTTAAATTCAATGCTTTTTCCAAAACAAATGAATATTTTGGAAATTCACAAAATTA
2D-0514120-T10-8_1024	TTTAAATTCAATGCTTTTTCCAAAACAAATGAATATTTTGGAAATTCACAAAATTAAAAATTCCTATTTTAAAAGTATGCTAATGGATGTTGGTTCTGATACTGTTATGCAAGACAAATG
2D-0514110-T10-8_1088	TGTGTTATGCTCTACATCGTCAGATCCACTAGAGGCCCCCTGTTTGTTTTGCGTTAGGCTCTGTCGCCGGTGCAGTGTTTGGTGTAGAGATTGGTACAAATGTCACCGACAAGACTTGTG
2D-0514120-T10-8_1088	TATTTTAAAAGTATGCTAATGGATGTTGGTTCTGATACTGTTATGCAAGACAAATGACCCGACCAGACGGTTACAAACGCTGTCCACTTCAAAAAAAAGGGAGGGGGGGGGGGGGTTAA
2D-0514110-T10-8_1152	CGCCGGTGCAGTGTTTGGTGTAGAGATTGGTACAAATGTCACCGACAAGACTTGTGTTCCTGTAGTATACTGACAAATGCCTCTCTGTGAGTTGTTGGCTATTTTCATTCTAGTCAGCAA
2D-0514120-T10-8_1152	AGACGGTTACAAACGCTGTCCACTTCAAAAAAAAGGGAGGGGGGGGGGGGGTTAAATCTCTTCTAGCCTCTCCGAGGGAGCTTAGAAAGAAATTAATTTTTCGAAATACTAATGctccct
2D-0514110-T10-8_1216	GTATACTGACAAATGCCTCTCTGTGAGTTGTTGGCTATTTTCATTCTAGTCAGCAAACAAATGCCTCTTATCTAGTCTCCACAGTGAGCAAACAGGTGGTGTGGGAAAGCAAAATTAGCT
2D-0514120-T10-8_1216	AGCCTCTCCGAGGGAGCTTAGAAAGAAATTAATTTTTCGAAATACTAATGctccctccgttccacaatgtactgcgtataatttttttaaagtcaaactttgaccaaatttataaagaa
2D-0514110-T10-8_1280	CTCTTATCTAGTCTCCACAGTGAGCAAACAGGTGGTGTGGGAAAGCAAAATTAGCTGGTTCATGGTAATGCATCATGTGTTAGCGTGACGTACGGACTTCTATCACAAATCATTTGCAAG
2D-0514120-T10-8_1280	caatgtactgcgtataatttttttaaagtcaaactttgaccaaatttataaagaaaagtattaaccagatatatataaaaatatgaaacgacgtcttttgatgaatctaatgatcctag
2D-0514110-T10-8_1344	GTAATGCATCATGTGTTAGCGTGACGTACGGACTTCTATCACAAATCATTTGCAAGCTTGCGCTTGCTTGCACCTCTTTTTTGCTGGGCATGCTTGTCTATATATCTTGATGGGTATGAG
2D-0514110-T10-8_1408	TGCTTGCACCTCTTTTTTGCTGGGCATGCTTGTCTATATATCTTGATGGGTATGAGTATGAGGCGTCTCTTACCTTTGTACTCCCTCCGTTCGGAATTACTTGTCACAAAAATGAATGTA
2D-0514120-T10-8_1408	gtctttctccacaaacttgatcaaagttgttgagttttgacttTTCCGAAAATTTATACGCACATTTTTTTTTAATGCAAAATTTATACGCACATTATGCAACGAGGGAGTAATACTGTA
2D-0514120-T10-8_1472	TTTTTTTTTAATGCAAAATTTATACGCACATTATGCAACGAGGGAGTAATACTGTAGAGAAACTCAACATATGTACCGGTGCCGATACCGACTCGAAGCCTCCAGCACGTTCCCCTTTTT
2D-0514110-T10-8_1580	AACGGAGGGAGTAGTTGACAAGTATTTAAGCTCGTATGAGTCGTCCCTTGCCCACGGTGGCCAGGTCTATGAAACCCATGTACCTGTTTGTCTCCTCCTGTGTTCTTGCTTGCAGTCGTA
1A-0032970-T10-9_1280	CACGGGCGTTCTTCTAGTTGGTATAGAAGATATAGATGACCGCGAGTTTTCCTTTTTTTTTTCTGAGGGGTATGACGGCGACTTTTCCTTTTCCCGACGACGAGCTGCATAGAATTTATA
1A-0032970-T10-9_1408	GTGATCGGCCGAGCGCCCAAGCCCAGTTACTTAGCTAGGCTATCGGTCCATCCGGCCCAGACCAAACACAAACAGAAACTCCCTGTTACGTATTCTCTCAGCATAGACGCACTCGCGACA
1B-2100890-T10-9_0	TAGGTTAGCTGTGGCTGAATTCGCCCTCAGCCTCTTAGGGTCATGAACCTTTTCAGGCGTGCTCGTCTGGCTCTTGTCCACTGTTACACTTGTCCCAGCTTCAAGGAGACCAACTTTCTC
1B-2100890-T10-9_192	AAGCCTTTTTTTCGCCATAGGGACAACAGCCCCCTGGTTAGTTGTTCTACCATGAACTATCTCCATATCATTATTCTCTTCTGGAGTTGGGTTATCATGATTACCTCTCTCTCTTCCCAG
1B-2100890-T10-9_256	ATATCATTATTCTCTTCTGGAGTTGGGTTATCATGATTACCTCTCTCTCTTCCCAGATCTCCATCCCTCTCCCTGGGTCTTCCCATATTTCTCCTTCCCCTGCCACTGCTAGAGCCACTA
1B-2100890-T10-9_320	CCCTCTCCCTGGGTCTTCCCATATTTCTCCTTCCCCTGCCACTGCTAGAGCCACTAGAGGGATCATCAAAGTTCACCAGAAGCCAATCTCCCCACTCGCAATCCTCTACTGGGTGCGTGC
1B-2100890-T10-9_512	GAACAGGGTACTTTTTACTTTCTTTCAACGTCAGTGGGACAAACCGAACAATGGGGGTATCCAGCATGATGTGCACACGAGCTCTCAAGTATTTTGCTGGATTGATACGGCCCTCATTGA
1B-2100890-T10-9_576	CATGATGTGCACACGAGCTCTCAAGTATTTTGCTGGATTGATACGGCCCTCATTGACAACAACTTTGATGGGAGGTAAGCCCACCTTTCTAGCTATTTTCTCAGCAATCTCTGTCTTTCT
1B-2100890-T10-9_640	TTGATGGGAGGTAAGCCCACCTTTCTAGCTATTTTCTCAGCAATCTCTGTCTTTCTCATAAGACCATTCGGTATGCCCATAATTCGTGCCCAAACTGCAATACGATCTAACTTGTACTCC
1B-2100890-T10-9_704	CATTCGGTATGCCCATAATTCGTGCCCAAACTGCAATACGATCTAACTTGTACTCCTCTACATTCGTCAGTCCATCATATTCCTCCAACACCACCGCGGCATTTCTGAACAACCATGGGC
1B-2100890-T10-9_896	TGAAGATTACTCCCTGCACCGGTGCCCAGGTGTTCCTCATCGCCTTGTACAGGGCAACATGGCTGAAGGGCTTAGCAGTATGAACCCTAAATATTGCAATCCAACGTGTCTCTTCTATTA
1B-2100890-T10-9_1152	CGGCATCTTGCCACTCCCTCTTTCCGCCATGCAGTCCGTCAAGATCGACGCAGGCCAGTCCGTACGTGAGATTTAGGTGTAGTTGCGGATAGGTGGAACCCTAGCGGTGCGCCGCCAGG
1B-2100890-T10-9_1280	GAACCCTAGCTACCTAGGGGATTAAACTGCTAGCACCTAGTGGTGTTAGTTTTGTTTTTAGAGCTGACGACGACGAGCTTTCCTTTTCCCGACGACGAGCTGCATAGAATTTACACCGTC
1B-2100890-T10-9_1408	CCGAGCGCCCAAGCCCAGTTACTTAGCTAAGCTATCGGTCCATCCGGCCCAGATCGAACACAAGCAGAAACTCCCTGTTACGTATTCTCTCAGCAGAGACGTACTCGCAACACTAGGAGT
1D-0184340-T10-9_128	CGTCTACGTAAAGATGAAGTTGTGGTGCTTTCCGATGGCGATACCGTGACTTTCGGAAATGGCAACATGACCTTTAGGTTAGTATAAAAGAAAAATGGGGCAACGGTATGCTTGTGCCTT
1D-0184340-T10-9_192	ACATGACCTTTAGGTTAGTATAAAAGAAAAATGGGGCAACGGTATGCTTGTGCCTTCTTCCCTGCTCACACATCTTTTTTTAATTAGCATGCGCAACTATCTGTAAGAAATGATTGTCAA
1D-0184340-T10-9_256	CTCACACATCTTTTTTTAATTAGCATGCGCAACTATCTGTAAGAAATGATTGTCAATGCATGCAATCATTTTTAATGTTTTTTTTTGCTTATTTCATATATGTACTGGTGCTTTGGTTGA
1D-0184340-T10-9_320	ATCATTTTTAATGTTTTTTTTTGCTTATTTCATATATGTACTGGTGCTTTGGTTGAAAAGGTGGATGTATAAGTTGCACTTAGTTCTGTTGATATTCCTGACAGGTTCTCGTTTATCCCC
1D-0184340-T10-9_512	GGTTAGTGGTAACTACACTTGTATGATGTACTTGCTGTCGGTTCATTAGTAAGCATAGGTTTTGCCATGCTTGTTGCTACTTAATTTTGATGTCGGTATTATCCTTCTAAGGTGCATATG
1D-0184340-T10-9_576	CCATGCTTGTTGCTACTTAATTTTGATGTCGGTATTATCCTTCTAAGGTGCATATGTTACTCGTAAGTGGATTGATACATGTACACATGTTCTTGTGGATGAATCATCTCCATTGACACC
1D-0184340-T10-9_768	CCCTCCTGCAGCATTGCTTGGATGCTCGTATGGTATTTTTTAGCCAGAGAACCTGAATTATGCGAATAAAATTAATGCATATAGATGGACAGAGGAGGTCCTCTGGTGTGATTCTGTAAA
1D-0184340-T10-9_832	AATAAAATTAATGCATATAGATGGACAGAGGAGGTCCTCTGGTGTGATTCTGTAAATTATATAGCAATGATCAACTATTTCACTTGTGTCATTTACACTTTCTTATTCATATGTATATTT
1D-0184340-T10-9_896	CAATGATCAACTATTTCACTTGTGTCATTTACACTTTCTTATTCATATGTATATTTCAGGTTTGAGTTCTTGTTTTGATCTGTTTCAGGCGATGGCTGAAAAGAACATACACACAGAGAT
1D-0184340-T10-9_1088	ACTTGTGGTTCTCCCGTAACAACAGATGGTGATATATTCATGGTACTTGATCCAGATATATTCCAAACTTGACTCTTGATGGGATGGATATAAAGATGGTTGAGAACAAGCTCATGGAGA
1D-0184340-T10-9_1280	CATCAGGCGTTTTGCTGTTGACTAACGTTATTCAATTGCAGTATAAATTTGGAGAGAAAATACAAGAGTTACTGGAATCAACTGGAGCAAAATATCTCAGTATCGAGGAGTTTTGTGCTA
1D-0184340-T10-9_1344	AGAGTTACTGGAATCAACTGGAGCAAAATATCTCAGTATCGAGGAGTTTTGTGCTAACAGCCAGGTAAGTGTTCTAATATGTTATATTTTTTTTTCTTTTTCTTTTGAGGATACCTTTGA
1D-0184340-T10-9_1408	GTAAGTGTTCTAATATGTTATATTTTTTTTTCTTTTTCTTTTGAGGATACCTTTGAGGAATAATGCATTTTCTCTTTAAAAACATAAGGCACCCAAATTCTAGATGAAGAGGGCTTGAGC
1D-0184340-T10-9_1472	GCATTTTCTCTTTAAAAACATAAGGCACCCAAATTCTAGATGAAGAGGGCTTGAGCTGATAATAATGTCAGTTACTTATTCCGGTGCTGCCTTATGTGATTGCTTCATTGCCTCTATTTG
1D-0184340-T10-9_1536	ATGTCAGTTACTTATTCCGGTGCTGCCTTATGTGATTGCTTCATTGCCTCTATTTGCATGATGATTTATCTTGTTATTGGTGGGTGTGTACTCTTTGTTTACGGGAGGTGCAAACAGATG
3A-0676090-T10-10_0	AAATCGAGGGGGTGGAGTGTTTCATATTCAGGTGGTTTGTGTAACAGCCCAAATAAAAAACTTCCCTTAAGGACCTCTTTGTAATATTTATAGAAGTTATGGGTGTTTTGTGAAATGGTT
3A-0676090-T10-10_64	CCTTAAGGACCTCTTTGTAATATTTATAGAAGTTATGGGTGTTTTGTGAAATGGTTTTGATATTTTTTGATGTTGCCATTTGTTTGTTTCACATCAACTTGAATTCTTGTCCCCTTTCAC
3A-0676090-T10-10_128	TTTTGATGTTGCCATTTGTTTGTTTCACATCAACTTGAATTCTTGTCCCCTTTCACTAAGTCAATTCACAACTTTCCAGATCTCCAACTCAACTTTCAACAGGATTATTATAACCACGAC
3A-0676090-T10-10_192	TTCACAACTTTCCAGATCTCCAACTCAACTTTCAACAGGATTATTATAACCACGACAACCAACGTTTATCAAGTGAAATGAGCATGTTTTTTTGTGGGGTAAATGAGCATGTTTGACTAG
3A-0676090-T10-10_256	TTTATCAAGTGAAATGAGCATGTTTTTTTGTGGGGTAAATGAGCATGTTTGACTAGTGTTTATCGACAAAAATGAGAATGCGATGTATTAAGGATTATATCGATGACGATCTGATTCAGC
3A-0676090-T10-10_320	GACAAAAATGAGAATGCGATGTATTAAGGATTATATCGATGACGATCTGATTCAGCGGTGCTTACAAGGGTAGGACTAAATATGCATATGCGAACATATTGCCCTGTAGTCAAAGAAGGC
3A-0676090-T10-10_384	CAAGGGTAGGACTAAATATGCATATGCGAACATATTGCCCTGTAGTCAAAGAAGGCCAACGTCCTAGCATCGTCCTATGGCTCATAGTGGTCCTCGTGGATGACTTTCTCGTAGACATGG
3A-0676090-T10-10_576	GTGTGTGGCGGATAGTCGCAAGACACCAATGTGTGGAAGAGGTTATCACTATGGAGGCCGACGAGCAACGTGGAACCTAGACGTGCGCGCTTGAGCTGTATGGTGATGTTGTCCACGA
3A-0676090-T10-10_704	GATGTTGAACCTCTTGAGGAAGCTAATGACGACATCGTTGAAGTAAAAGACGAAGCTAGCGTCCACGATTCCTATTATCTTGGCAGCCTCCAGGACCATCCGGAGGCGTTCCTCGGTGCC
3A-0676090-T10-10_832	GGCCAGCGTTGTGGACCATCTACCAAAGGCGCCTTCTTCTCGCAAGCATCGATTCCATGTGGTGTGGTCCTGGTCACTTGATTTTGGTATTGGACGGAGCATATTTACCATTGATGTGGT
3A-0676090-T10-10_896	TGGTCCTGGTCACTTGATTTTGGTATTGGACGGAGCATATTTACCATTGATGTGGTGCGTAGGCGCGCAAGTTTTGTAGATGGACTGTAGTGAAGGGGCGTCTTATATGAGAGTGTAAGT
3A-0676090-T10-10_960	GCGCAAGTTTTGTAGATGGACTGTAGTGAAGGGGCGTCTTATATGAGAGTGTAAGTTGCAGTGCGCTGGCCATCTGATTGTGTGAGCTTTGGCAAACAAATACAAGATGACAATTTTTAG
3A-0676090-T10-10_1024	GCTGGCCATCTGATTGTGTGAGCTTTGGCAAACAAATACAAGATGACAATTTTTAGTTATTTTTGTTGACTTTTTAACAGCAGGCTTACGCGGGCGCTTTTAGTCGCTTGACATAATAGT
3A-0676090-T10-10_1088	GTTGACTTTTTAACAGCAGGCTTACGCGGGCGCTTTTAGTCGCTTGACATAATAGTTATTCCTACGCCGATCAAGTGGTACAACATAAAAGAAAAATTGAAGTTGCATAACTAATAAATT
3A-0676090-T10-10_1152	CGCCGATCAAGTGGTACAACATAAAAGAAAAATTGAAGTTGCATAACTAATAAATTGTGCAAAACCATAGTTGTAGAAATGCGTAATTGTATGCTTCACATCGATAAACTCAAGCTAAAT
3A-0676090-T10-10_1216	CCATAGTTGTAGAAATGCGTAATTGTATGCTTCACATCGATAAACTCAAGCTAAATCATGAATGCTTAGAATTATTAAATTTGGTAAACATTGTGTCAATCAAAATAAATTTCTCCAATA
3A-0676090-T10-10_1280	CTTAGAATTATTAAATTTGGTAAACATTGTGTCAATCAAAATAAATTTCTCCAATAATTTGCGTTGGCCCTCTCCAAATAGCCCACGCCCACCTCTATACTGTACTACCAGAAAAAAAGA
3A-0676090-T10-10_1344	TGGCCCTCTCCAAATAGCCCACGCCCACCTCTATACTGTACTACCAGAAAAAAAGACCACCAAAGTGCCATAAACCAAAATACATCTCCGGTCACTTTCCAAAATTCGAGGCAAAGCGCC

3A-0676090-T10-10_1408	GTGCCATAAACCAAAATACATCTCCGGTCACTTTCCAAAATTCGAGGCAAAGCGCCGGAAACAATAGTTTTGAAAAAGCGCGCTCTAAAATTTCGAGATCCCCCAAATCCAtcccctccc
3B-0772450-T10-10_128	actggggttagggttggtggcgtggtggggaggggaggggagggATTTGGGGGATCTCGAAATTTAGAGCGCGCTTTTTCAAAGCTATTGTGTCCGGTCCATTGTGTCCAGCGCGCTGTG
3B-0772450-T10-10_192	TAGAGCGCGCTTTTTCAAAGCTATTGTGTCCGGTCCATTGTGTCCAGCGCGCTGTGCCTCAAATTTTGGAAAGTGGCCAGAGAGGTGGCAGTGGCACTTTTTTTTTTTTTGAGGGAGGTG
3B-0772450-T10-10_256	TTTGGAAAGTGGCCAGAGAGGTGGCAGTGGCACTTTTTTTTTTTTTGAGGGAGGTGGCAGTGGCACTTGGGTGGTCTTTTTTCTGGGGGTTTTTTTTTAGCTGTCTGGGTTTTTGTTTT
3B-0772450-T10-10_320	ACTTGGGTGGTCTTTTTTCTGGGGGTTTTTTTTTAGCTGTCTGGGTTTTTGTTTTTTTTTGAGACACTGGTCTTTTTTCTGGTAGTGCAGAGGTGGGCGTGGGCTATTTTGGAGAGGGT
3B-0772450-T10-10_384	ACACTGGTCTTTTTTCTGGTAGTGCAGAGGTGGGCGTGGGCTATTTTGGAGAGGGTCAAAGCAAATTATTGGAGAAATTTATTTTGATTGACACAATGTTTACCAATTTTAATAATTCTA
3B-0772450-T10-10_448	ATTATTGGAGAAATTTATTTTGATTGACACAATGTTTACCAATTTTAATAATTCTATTCTAAGCATTCATGATTTAGCATGAGTTTATCGATGTGAAGCATGCAATTACGCACTTCCACA
3B-0772450-T10-10_512	ATTCATGATTTAGCATGAGTTTATCGATGTGAAGCATGCAATTACGCACTTCCACAACTATGGTTTTGCAAATTTTATTTGTTGTGCAACTTCAATTTTTCTTTTATGTTGTACCACTTG
3B-0772450-T10-10_576	TTTGCAAATTTTATTTGTTGTGCAACTTCAATTTTTCTTTTATGTTGTACCACTTGATCGGCATATGAATAACTATTATGTCTACTATATCAAGCGATTAAAAGTGCCGCGTAAGCCTGC
3B-0772450-T10-10_640	ATGAATAACTATTATGTCTACTATATCAAGCGATTAAAAGTGCCGCGTAAGCCTGCTGTTAGAAAGTCAACAAAAATGACTAAAAATTGTCATCTTGTAATTGTTTGCCAAAGCTCACAC
3B-0772450-T10-10_704	AGTCAACAAAAATGACTAAAAATTGTCATCTTGTAATTGTTTGCCAAAGCTCACACAACCGAATGACCACAACCTGCAATCTCATATAAGACGCCCTTCCACTATAATCCTTCTACAAAA
3B-0772450-T10-10_768	GACCACAACCTGCAATCTCATATAAGACGCCCTTCCACTATAATCCTTCTACAAAATCTTACGCACCTACGCACCACATCAATGGTAAATGTGCTCCCTCCAATACCAATATCAAGTGAC
3B-0772450-T10-10_960	ACGGCCCGGCACCGAGGAATGCCTCCAGATGATCCTCGAGGCTGCCAATATAATAGGAATCATGGACGCCAGTTTCGTCTTTTGCTTCAACGATGTCGTCTTTAGCTTCCTCGAGAGGTT
3B-0772450-T10-10_1024	GACGCCAGTTTCGTCTTTTGCTTCAACGATGTCGTCTTTAGCTTCCTCGAGAGGTTCAACATCCTCGTGAAGCTCGTGGACAACGTCACCATATGGCTCAAGCCCGCACGCCTAGGTTCC
3B-0772450-T10-10_1088	TCGTGAAGCTCGTGGACAACGTCACCATATGGCTCAAGCCCGCACGCCTAGGTTCCGCATTGCTCGCCGGCCTCCATAGTGATAACCCCTTCCACACATTGGTGCCTTGCGACTGTCCGC
3B-0772450-T10-10_1408	GCCGTCAACGCTGCTACTCAGCCACCAGTCAGTGAGCCGACGCATTGAGTATGAGAAAGAGGTCATTGATGTTCGCATATGCATATTTAGTCCTACCCTTGTAAGCACCGCTGAATCGGA
3B-0772450-T10-10_1472	ATTGATGTTCGCATATGCATATTTAGTCCTACCCTTGTAAGCACCGCTGAATCGGATCGTCGTGGCCATAATCCTTAATGCATCACATTCTCATTTTTGTCGATAAACACTAGTCAACAT
3B-0772450-T10-10_1536	GCCATAATCCTTAATGCATCACATTCTCATTTTTGTCGATAAACACTAGTCAACATGCTCATTTCACTTGATAAACATTGGTTGTCGTGGCTATAATCCTCTTGAAAGTTGAGTTGGAGA
3B-0772450-T10-10_1580	ACTAGTCAACATGCTCATTTCACTTGATAAACATTGGTTGTCGTGGCTATAATCCTCTTGAAAGTTGAGTTGGAGATTTGGAAAGTTGTGAATTGACTTAGTGAAATGGGACAAGAATTC
3D-0922690-T10-10_192	GATGTTATCACTATGGAGGCCGGCAGGCAACGTGGAACCTAGACGTGCGGGCTTGAGCCGTATGGTGATGTTGTCCACAAGCTTCACGAGGATGTTGAACCTCTTGAGGAAGCTAACGA
3D-0922690-T10-10_256	GTGATGTTGTCCACAAGCTTCACGAGGATGTTGAACCTCTTGAGGAAGCTAACGACAGCATCGTTGAAGCAAAAGGCGAAGCTGGCGTCCACGATTCCTATTATCTTGGCAGCCTCTAGG
3D-0922690-T10-10_448	CTCGCGAGCATCGATTCCATGTGGCATGGTCCTGGTCACTTGATTTTGGTATTGGAGAGAGCATATTTACCATTGATGTGGTGCGTAGGTGCGCAAGGTTTTGTAGATGGATTGTAGTGG
3D-0922690-T10-10_512	ATTTACCATTGATGTGGTGCGTAGGTGCGCAAGGTTTTGTAGATGGATTGTAGTGGACGGGTGTCTTATATGAGAGTGCAGGTTACAGTGCGTTGGTCATCCGATTGTCTGAACTTTGGC
3D-0922690-T10-10_576	CTTATATGAGAGTGCAGGTTACAGTGCGTTGGTCATCCGATTGTCTGAACTTTGGCAAACAAATACAAGATGACAATTTTTAGTCATTTTTGTTGACTTTCTAACAGTAGGCTTACACGG
3D-0922690-T10-10_640	ACAAGATGACAATTTTTAGTCATTTTTGTTGACTTTCTAACAGTAGGCTTACACGGGCACTTTTAGTCTTGCTTGATATAATAGACATAATAGTTATTCATACGCCGATCAAGTGGTACA
3D-0922690-T10-10_704	AGTCTTGCTTGATATAATAGACATAATAGTTATTCATACGCCGATCAAGTGGTACAACATAAAAGAAAAATTAAAGTTGCACAGCAAATAAATTATGCAAAATTAGTTGTAGAAACGCGT
3D-0922690-T10-10_768	GAAAAATTAAAGTTGCACAGCAAATAAATTATGCAAAATTAGTTGTAGAAACGCGTAATTGTATGCTTCACATCGGGAAACTCAAGCTAAACCATGAATGCTTAGAATGATAAAACATTG
3D-0922690-T10-10_832	GCTTCACATCGGGAAACTCAAGCTAAACCATGAATGCTTAGAATGATAAAACATTGTGTCAAACAAAATAAAATTGTCCAATAATTTGCTTTGGCCCTCTCTCCAAAATAGCCCACGCCC
3D-0922690-T10-10_896	AAAATAAAATTGTCCAATAATTTGCTTTGGCCCTCTCTCCAAAATAGCCCACGCCCACCTGTATACTGTACTACTACCAGAAAAAAGATCAGTGTCTCAGGGGGTGTTTGTTTCCAGGGA
3D-0922690-T10-10_1344	CTAGAAAAAGTTCTAAGACTTATGAACCAAACAGGGCCTAAAAAAACCCAGAAAAAAGACCACCCAAGTGCCATAAAACCAAAATAGGAGTACATCTCTGGTCACTTTCCAAAATTCGAG
3D-0922690-T10-10_1408	CAAGTGCCATAAAACCAAAATAGGAGTACATCTCTGGTCACTTTCCAAAATTCGAGGCAAAGCGCCGGAAACTATAGTTTTGAAAAAGCGCGCTCTAAATTTCGAGATCCCCCAAAtccc
4B-1052400-T10-11_128	GTCCGCACTCTGCCCTATGCGCCAAGCGCGCGGCCCACGAAGCCCGTACTAACTTGGCCCAGCAACGTCTCGCTGACGTTTTTTTTTCTTGTGCGCCAGATGTCTCCGTGAAGGTTCTAA
4B-1052400-T10-11_192	ACGTCTCGCTGACGTTTTTTTTTCTTGTGCGCCAGATGTCTCCGTGAAGGTTCTAAAAAAAAATGCCTCTGTGATTTTTTTTCCTCAAATGAtaatgtccacacgtgtgacacgaagcaac
4B-1052400-T10-11_1088	tgtgtgcgttatcagcgtccTTCTTTTTTAATTTATTGTCCATCTACACCCGGCGTGATTAATAAATTAAAAAAAGTAAAAGATATCAAAGAATCTGAACTTTTTAGGCATCCAGAATGC
4B-1052400-T10-11_1152	AATTAAAAAAAGTAAAAGATATCAAAGAATCTGAACTTTTTAGGCATCCAGAATGCTCATATTATATATGTAAGATTTGTGCAAATTTTTGTGGTATTTGGACATCTGAAGAGCTCGTGT
4B-1052400-T10-11_1280	AAAACAAATTTCAAGTGTGTGAGAAACTTTGAAAAAGAACACTATTCAGAGCTGATTTTTTTACGAGCTCCTCAAATGTGCAAACACCACAAAAATTTACACAGACATGAGCATCTTGGA
4B-1052400-T10-11_1344	GAGCTCCTCAAATGTGCAAACACCACAAAAATTTACACAGACATGAGCATCTTGGATGCCTAATTTCTTTTTCTTTTTTACTATTTTTCCAAAATTACTGTTCATCTATTGTAGATGAAC
4B-1052400-T10-11_1408	TTCTTTTTCTTTTTTACTATTTTTCCAAAATTACTGTTCATCTATTGTAGATGAACCTGAGCATAGTTTTGAATTAGTATCACAGGATAGACCCCTATTTTTATCCTCGTTAGAGTATCA
4B-1052400-T10-11_1472	AGTTTTGAATTAGTATCACAGGATAGACCCCTATTTTTATCCTCGTTAGAGTATCACCAACAGCTTCCCTTTCCCTAATAAAATCATATGTTGGGGGAGTTGGGAAAAAAAAACTGTCCTA
4B-1052400-T10-11_1536	TTCCCTTTCCCTAATAAAATCATATGTTGGGGGAGTTGGGAAAAAAAAACTGTCCTACCCGTTTAGACAAACATGAAGGATTTAGGGGCGCTCTTGGAGATGCCTTGAGCGCATCTTTTGC
4B-1052400-T10-11_1580	AAAACTGTCCTACCCGTTTAGACAAACATGAAGGATTTAGGGGCGCTCTTGGAGATGCCTTGAGCGCATCTTTTGCATCAAATACCATGTGTGCGTCACCTGTCACTATCATAGAGATTT
4D-1168630-T10-11_0	CGGTCGGCGAGCGAAGCCCAGgtggctgagtgctgacggtttggggggagggtgcgtggtgagtggtgaTGAGGCGGTTTTGCGTCGGTGTTTTATACCCCCGTCCCGTACGCGCGCGG
4D-1168630-T10-11_64	tggtgaTGAGGCGGTTTTGCGTCGGTGTTTTATACCCCCGTCCCGTACGCGCGCGGGGTGGTGTGGAGGTGTGAGTCGGAAGGAGAAAGACAATTAATTCGGACGCGGGAAGGATCCGA
4D-1168630-T10-11_256	GACGTGCATTCAAACGCTCTCAGTGAGTAGACGCGTGCGTGCCCACGACCACGAGCGAGCTGACACCGTTTCCGTTCGTTCGGCTGGGTCGGAAAGACCAACGACGCGGGCCGATCGG
4D-1168630-T10-11_384	ATAAGATGCCTGAGGGTCGGGTTTCGACCGGGCGACCGGAGAAGGGCCTGGGCCGGCAGCCGCCACGTCCGTCGGTACGTACGATTCTCAGGGCGTGGCGTGCGTGGCGCGTTCTTC
4D-1168630-T10-11_448	ACGTCCGTCGGTACGTACGATTCTCAGGGCGTGGCGTGCGTGGCGCGTTCTTCTTCTCCTCTTTTGACGGGCAACCGTGACTTCGTGGGTTGGGGGGTTTTGCGGGGAAAACGACGGC
4D-1168630-T10-11_576	CGGGTGGAGACTTGAGCCGGTTCGAGGGGATATCCAGACGTTTCGGCGCTCTTGTGAGAGGATGGATTTTGCAGATCATTTGCTGCTGCTGTGCTTTCGTTTCGTACGGTCCACAGGAC
4D-1168630-T10-11_704	CGACGGCTGCTCCTAGTGTCTTGATCCGTGCTGTTATGCCTGAGCGAAGAACGTTTCGCCTAGATGTCATCTTTGTCTTGTGTCTATGGCTTTACTTCGATGGCCGTTTCTCCCAACACC
4D-1168630-T10-11_768	TGTCATCTTTGTCTTGTGTCTATGGCTTTACTTCGATGGCCGTTTCTCCCAACACCACCCGAAATGTCATACTATATATTTAGGAAAGTGGTTGCAGATTGTCCTCTCATTTCATACGT
4D-1168630-T10-11_832	TGTCATACTATATATTTAGGAAAGTGGTTGCAGATTGTCCTCTCATTTCATACGTTCATGGCTTTAATATCGTTATTATCACCAGGGGCTAAAGCTTGGTAAAAGCTTCTGGTTCAGTC
4D-0975300-T10-11_896	TAACACATACATAGACGAAGCATGAAATGGATGGCCACCTTGTCTTGAGCCTCTTGTGTAATACTAGCATCCACACATTGCTGTCTTTGGTAACTGTTGTATTAGCCTCACAGACCAGGA
4D-1168630-T10-11_896	TTAATATCGTTATTATCACCAGGGGCTAAAGCTTGGTAAAAGCTTCTGGTTCAGTCTCTAGGATTGACATCACATGGTGAGCATCGTGTGAGAAGACAACTCCCTCTTTTCACGATGTCA
4D-0975300-T10-11_960	TAGCATCCACACATTGCTGTCTTTGGTAACTGTTGTATTAGCCTCACAGACCAGGATTTTTTTCCTGCGTTTGACAAGTTGAATTAATCAGTGTTTGATAAGTGAGCAAACTTGTGGGCA
4D-1168630-T10-11_960	TGACATCACATGGTGAGCATCGTGTGAGAAGACAACTCCCTCTTTTCACGATGTCATTTTCACAGTTGAAAAACCGAACCACCGTCGTTAATGCAGGAACCATCGATTGATTGATCAGTG
4D-0975300-T10-11_1024	CTGCGTTTGACAAGTTGAATTAATCAGTGTTTGATAAGTGAGCAAACTTGTGGGCAAATTGAAAGTTCATATTTAGAGATGCATCTAGTTTGTCATCTATGTTCAGTTATGATTAATTTG
4D-0975300-T10-11_1088	GTTCATATTTAGAGATGCATCTAGTTTGTCATCTATGTTCAGTTATGATTAATTTGCTTGAAATTGCACTTGATGAGAAAGCGTTTATTTCTTTGATTAGGCCTACCTGTATATGTCTGA
4D-1168630-T10-11_1088	TTCTGCTTTGCAGCGGCATTGGTCCAGCATGCAGGAACTGCATACTACGTACGATCCTAGGAAGCTTCGGTGCATGGAGAAAACGGCCAACTAGGCGTGAAACACTTGTCCCTTTTTGAA
4D-0975300-T10-11_1152	TGCACTTGATGAGAAAGCGTTTATTTCTTTGATTAGGCCTACCTGTATATGTCTGAATCTTTTGACTTGCTCAGGATGGAGCTAGATCCGCTTCCCGAGGACTAAGTCGAGCTGGTCACT
4D-1168630-T10-11_1152	CTTCGGTGCATGGAGAAAACGGCCAACTAGGCGTGAAACACTTGTCCCTTTTTGAACTTGTGAGACACTTTTCTCTTCTTCAACTTGTGAGGCACTTTTCTTTCCTTGGCAACGCACGAG
4D-0975300-T10-11_1216	ACTTGCTCAGGATGGAGCTAGATCCGCTTCCCGAGGACTAAGTCGAGCTGGTCACTTTTTTTTTCTGAAAATATAGGCCTTATTTCAAAGGAAAGAAATGATTTGGCGCGTTTTGGGAAAG
4D-1168630-T10-11_1216	ACACTTTTCTCTTCTTCAACTTGTGAGGCACTTTTCTTTCCTTGGCAACGCACGAGTCATGCATCCCTCCTCCGCCCGTCCGCTGTGGATTAGTCTCGCTCGCACAACATCACAGGTGCA
4D-0975300-T10-11_1280	TGAAAATATAGGCCTTATTTCAAAGGAAAGAAATGATTTGGCGCGTTTTGGGAAAGTTTTTTTTTTTTGCAAGAATAGCAGATAGTCTAGGCAATGGGCCTAAACACACAGTAATAATCA
4D-0975300-T10-11_1344	TTTTGCAAGAATAGCAGATAGTCTAGGCAATGGGCCTAAACACACAGTAATAATCACTAGTTGCGCTGTTTCTGGGCTGACGCTAGCGCCAGCGAAGGCGATACGGCCCACGCCCGTGCC
4D-1168630-T10-11_1408	CAAAGTGGCAGCAAGCCGGCAGGTAGGAGTGTAGGACTACTCCGGTGCGCCTGATGGCTGTTGCTGGTAATAAGAGAGGTGTTGTACCATTCGGGGCGTTGATCCGGTCCAGACCCAA
4D-0975300-T10-11_1536	TGACCCGTATCACAGTGGAGGCCAAACCCTTCTTCCCCCTTCCCGTCTTCTCCCCCACATCCACCCCACCCCATTTTCCCCTTTGCCTCGCTCAACCGCGAACAGCATCGACTCGGCGGC
4D-1168630-T10-11_1536	ACCTTCGGTCACAAGTCAAACTTCTCCAACGGAAACGGTCACAAGTCATGACAAGTCTTTCTCAGGTTTGCCTAAATTTACGCCATCTAGATAAGCTACTCGTAACACAAGCAATTAGGC
4D-1168630-T10-11_1580	GTCATGACAAGTCTTTCTCAGGTTTGCCTAAATTTACGCCATCTAGATAAGCTACTCGTAACACAAGCAATTAGGCATACACAAGTGTTTTCAAGAAGAAGAAAAATTAGGTATGTACTC
5A-1199900-T10-12_256	CTCCCACCCACGGGCCACGCGGAGCCGAGTCGGCATCATTCCATCGTCTTATTCTCTCTCTCCTCCCCGATTCCCCACCCCCACCCCCACCCATCGCACCAAATTCCTATCTAGGGTTTG
5A-1199900-T10-12_512	TGAATCGGCCGGTCGTTGCTCGTAGATTTATAAATTTGTTGGAATTTTTCTGAGGGGGAGGCTAGATTCCCCTCCTTCCGGAAGTAAATAAATAAGGATGATCGTCTAGGGTTTATTTCG
5A-1199900-T10-12_576	GATTCCCCTCCTTCCGGAAGTAAATAAATAAGGATGATCGTCTAGGGTTTATTTCGGTGGATTATATGTTCCGTAGACCTGTTAGCTTTTGTGTGACCACCATGGTGGTGTCTTGATTGG
5A-1199900-T10-12_1088	CCCAGCTAGGGTTGTTGGTAATCATTTTGTTTCATTTGCTGCTATTACTGTATTTCTTTCTTATGAATACTCTCTGAGATCATATGCCCTTACCATGGGTAGTAGCTATAAGGGCTTCGA
5A-1199900-T10-12_1152	GAATACTCTCTGAGATCATATGCCCTTACCATGGGTAGTAGCTATAAGGGCTTCGAATTTTGACAAATATTTTTGAACATTAGAGATGTTGTATCTTCTATGCACATGTGCACAATTGTG
5A-1199900-T10-12_1216	AAATATTTTTGAACATTAGAGATGTTGTATCTTCTATGCACATGTGCACAATTGTGATCAAGATTGTCTCAAATGTGATGAACTTATTCTGAGCTAAACATGTTGGTGCAAAAAACAGTT
5A-1199900-T10-12_1344	GCTCTTGACCTGAGAATTGCAATACTTACAAAGATACACTGCTTATCTGATATTTTAATCAGCATAGTATTTAAAGCTTACCTGTCAACTTAACTAATAAAATCAATTGACATTGCACAA
5A-1199900-T10-12_1408	TAGTATTTAAAGCTTACCTGTCAACTTAACTAATAAAATCAATTGACATTGCACAACACCAGTGAAACTCTGCTTTTGCCTAGCCATGCATAAATTAGTAGCTTGTCAAATATGAACTTC
5A-1199900-T10-12_1472	AAACTCTGCTTTTGCCTAGCCATGCATAAATTAGTAGCTTGTCAAATATGAACTTCAGTCCTCAGCATCTAATATGCTCCAACTGATATTATTTAACTGATAAAATCAATTTACATTGCA
5A-1199900-T10-12_1536	GCATCTAATATGCTCCAACTGATATTATTTAACTGATAAAATCAATTTACATTGCACACCACCAGTGAAACTCTGCTTTTGCCTAGCTATGCATAAATTAGTAGCTTGTCAAATATGGAC
5A-1199900-T10-12_1580	ATTTACATTGCACACCACCAGTGAAACTCTGCTTTTGCCTAGCTATGCATAAATTAGTAGCTTGTCAAATATGGACATCAGTCCTCGGCATCTGTAATATGCTCCAACTCCTATTATTTA
5B-1295520-T10-12_256	GCCTCTCCCACCCACGCGGAGTCGAGTCGGCATCATTCCATCGTCATATTCTCTCTCTCTCCTCCCCGATTCCCCACCACCACCCATCGCACCAAATTCCTTTCTAGGGTTTGGTTCCCC
5B-1295520-T10-12_512	GCCGGTCGTTGCTTGTAGATTTATAAATTTGTTGAAATTTTTCTGAGGGGGAGGCTAGATTCCCATCCTTCCGGAAGTAAATAAATAGGGATGATCGCTTGCATGCATGCGTCTAGGGTT
5B-1295520-T10-12_576	ATCCTTCCGGAAGTAAATAAATAGGGATGATCGCTTGCATGCATGCGTCTAGGGTTTATTTCGGTGGATTATGTTCCGTAGACCTGTTACCTTTTGTGTGACCACCATGGCGGTGTCTTG
5B-1295520-T10-12_1088	GCTGCCCCAGCTAGGGTTGTTGGTAATCATTTTGTTTCATTTGCTGCTATTACTGTATTTCTCTCTTATGAATACTCTCTGAGATCATATGCCCTTACCATGGGTAGTAGCTTTAAGGGC
5B-1295520-T10-12_1152	CTTATGAATACTCTCTGAGATCATATGCCCTTACCATGGGTAGTAGCTTTAAGGGCTTCGAATTCTGACAAATATTTTTGAACATTAGAAATGTTGTATCTTCTATGCACATGTGTTCAA
5B-1295520-T10-12_1216	CTGACAAATATTTTTGAACATTAGAAATGTTGTATCTTCTATGCACATGTGTTCAATTGTGATCAAGATTGTCTCAAATGTGATGAACTTATTCTGAGCTAAACATGTTGGTGCAAAAAA
5B-1295520-T10-12_1344	TCTTGGCTCTTGACCTGAGAATTGCAATACTTACAAAGATACACTGCTTATCTGATATTTTAATCATCATAGTATTTAAATCTTACCTGTCAACTTAACTAATAAAATCAATTGACATTG
5B-1295520-T10-12_1408	CATCATAGTATTTAAATCTTACCTGTCAACTTAACTAATAAAATCAATTGACATTGCACAACACCAGTGAAACTCTGCTTTTGCCTAGCCATGCATAAATTAGCAGCTTGTCAAATATGG
5B-1295520-T10-12_1472	CAGTGAAACTCTGCTTTTGCCTAGCCATGCATAAATTAGCAGCTTGTCAAATATGGACTTCAGTCCTCAGCATCTAATATGCTCCAACTGGTGTTATTTAACTAATAAAATCAATTTACA
5B-1295520-T10-12_1536	CCTCAGCATCTAATATGCTCCAACTGGTGTTATTTAACTAATAAAATCAATTTACATTGCACCAGTGAAACTCTGCTTTTGCCTAGCTATGCATAAATTAGTAGCTTGTCAAATATGGAC
5B-1295520-T10-12_1580	AATCAATTTACATTGCACCAGTGAAACTCTGCTTTTGCCTAGCTATGCATAAATTAGTAGCTTGTCAAATATGGACATCGGTCCTCGGCATCTGTAATATTCTCCAACTCCTATTATTTA
5A-1209920-T10-13_64	TATTTATTCGGCCCGTTTAAGCTGCTTTTTTGAGACGGTATGATATGCTGAATCCGGCCCGTAGGCACTTCCATGGCAAATTGTTTAGCTTTATTTTTTTCTTTTTTGCGATCACTTTAG
5A-1209920-T10-13_128	GCACTTCCATGGCAAATTGTTTAGCTTTATTTTTTTCTTTTTTGCGATCACTTTAGTTTCTACTCTTCCCCCAAAAAAGAAAACTTTACTTTCAACCCATTATAAGGGCCTACTTGATTC
5A-1209920-T10-13_192	CTTCCCCCAAAAAAGAAAACTTTACTTTCAACCCATTATAAGGGCCTACTTGATTCGCACAGTTTTTAAAAACACGAAAATAAGAAGAGCGTAGCATTTCACTGTGGTGATATATTGAAT
5A-1209920-T10-13_256	TTTAAAAACACGAAAATAAGAAGAGCGTAGCATTTCACTGTGGTGATATATTGAATCTTATAGGAACCGAAAACACTGAAATATGCAATGTAAGTTGCTTGATTGCCTAACAAGAAAAAT
5A-1209920-T10-13_320	AACCGAAAACACTGAAATATGCAATGTAAGTTGCTTGATTGCCTAACAAGAAAAATGCAGGAATTTTTTCAATAGCATTAGGTGTTTGACATAGTTGTTGTAAAAACAAAGAAATTTACA
5A-1209920-T10-13_384	TTTTTCAATAGCATTAGGTGTTTGACATAGTTGTTGTAAAAACAAAGAAATTTACAGTGGCAAGCGCAGTCAGCAATCTTCAAATCATTCAAGAAACAAAAGAAATTTGCCATGAGGTTG
5A-1209920-T10-13_448	CGCAGTCAGCAATCTTCAAATCATTCAAGAAACAAAAGAAATTTGCCATGAGGTTGGACTCAACGAAAATTTCCCATGATGCATTTGCATAATAGATGGTACCCATGTGACTGCTGGAGT
5A-1209920-T10-13_1152	ATGGGCAAACAAAGGTAACACAAGGATCTGATGAAGAAGAAGCAACAGTAGAAGATTCAGGAGTAGCAGAGGATGCATCAGCATCAGAGGAAGATGCAACAACAGAAGAATAAACAA
5A-1209920-T10-13_1216	AGCAGAGGATGCATCAGCATCAGAGGAAGATGCAACAACAGAAGAATAAACAACAACATGAAGAAGATGGGAATGGACTTCCCCCTAGTTGGGCACAGATGAACTTTCCCCTATTGAG
5A-1209920-T10-13_1344	GTTAATTTCTACTGTAATTTGTTAGTATCTAGGATGAACTGCCATTTCTTTAATAGCTGACTGAAACAATGATTTATTCAAACTTCATATGTTAGTGTCATATGTGGTAAGGTCGTCACT
5A-1209920-T10-13_1536	CAAGCCATCAAATGATAGGGGGAAATTGCTTCCCTAATGCAGCCAACCTACATGTAGGGTGCCAACCTACTCAACCACTCCCAACCGAGTCACAAATGCAAAAACCCAAAAATGATGCAA
5A-1209920-T10-13_1580	AACCTACATGTAGGGTGCCAACCTACTCAACCACTCCCAACCGAGTCACAAATGCAAAAACCCAAAAATGATGCAACCTATCAAACAGGTCCTTGAATATGGACTTTGATCATTACTTCG
5B-1289680-T10-13_0	ACGCTGTGTGGTGGTAGTTTGATTTGATTTGAAACTCTGAGGTCGAAGTTGGCCGTACAGTATTTATTAGGCCCGTTTAGGTTGCTTTCTGAGACGGTATGATATGCTGAATCCGGCCCA
5B-1289680-T10-13_64	TATTAGGCCCGTTTAGGTTGCTTTCTGAGACGGTATGATATGCTGAATCCGGCCCATGAGCACTGGCCGCACTGCCATGGCAAATTGTTTAGTTTCATTTTTTTTCTTTTTTGTGATCAC
5B-1289680-T10-13_128	GGCCGCACTGCCATGGCAAATTGTTTAGTTTCATTTTTTTTCTTTTTTGTGATCACTTTAGTTTCTACTCTTCCCCCAAAAACGAAAACTTTACTTTCAACCCATTATAAGGGCCTACTT
5B-1289680-T10-13_192	CTACTCTTCCCCCAAAAACGAAAACTTTACTTTCAACCCATTATAAGGGCCTACTTGATTCGCACGGTCTTAAAAAACACAGAAATAGGAAGAATGTAGCATTTCAATGTCGTGACATAT
5B-1289680-T10-13_256	CGGTCTTAAAAAACACAGAAATAGGAAGAATGTAGCATTTCAATGTCGTGACATATTGAATCTTATAGGAACCAAAAACACTGAAATATGCAATGTAAGTTGCTTGATTGTCCAACAAGA
5B-1289680-T10-13_320	ATAGGAACCAAAAACACTGAAATATGCAATGTAAGTTGCTTGATTGTCCAACAAGAAAAAATGCAGGAATTTTTCCAATAGCATTAGGTGTTTGACATAGTTGTTATAAAACAAAGAAATT
5B-1289680-T10-13_384	GGAATTTTTCCAATAGCATTAGGTGTTTGACATAGTTGTTATAAAACAAAGAAATTTACCGCGGCAAGCGCGGTCAGCAATCTTCAAATCATTCAAGAAACTAAAGAAATTTTCCATGAG
5B-1289680-T10-13_448	CAAGCGCGGTCAGCAATCTTCAAATCATTCAAGAAACTAAAGAAATTTTCCATGAGGTTGGACTCAATGAAAATTTCCCATGATGCATTTGCACAATAGATGGTACTCATGTGACTGCTA
5B-1289680-T10-13_1152	CTGAGGCAATATGGGCAAACAAAGGTAACACAAGGATCTGATGAAGAAGAAGCAACAACAGAAGATTCAGGAGTAGCAGAGGATGCATCAGCATCAGAGGAAGACGCAACAACAGAA
5B-1289680-T10-13_1280	AACATGAAGAAGATTGAGAATGGACTTCCCCCTAGTTGGGCACAAGTGAACTTTCCCCTACTAAGCCATATGGCTGTTAATTTGTATTGCAGTTTGTTAGTGTCTAGGATGAAGTGCCAT
5B-1289680-T10-13_1344	GCCATATGGCTGTTAATTTGTATTGCAGTTTGTTAGTGTCTAGGATGAAGTGCCATTTGTTTAATGGCTGACTAAAACAATGTTTTATTCAAACTTCACATGTTAGTGTCATATGTGGTA
5B-1289680-T10-13_1408	TGGCTGACTAAAACAATGTTTTATTCAAACTTCACATGTTAGTGTCATATGTGGTAAGGTCGCCACTAGTGAGAAGAGGTGAGCACAACATAGTCCATGCACAACCAAACACCGTGTAGT
5B-1289680-T10-13_1536	CTAGCCCAACGCAAGCCATCAAACGACCGGGGGGTTTTGCTTCCCTAATGCAGCCAACCTACATGTAGGGTGCCAACCTACTCAGCCAGGCCCAACTAAGCCACGAATGCAAAAAAACC
5B-1289680-T10-13_1580	CTAATGCAGCCAACCTACATGTAGGGTGCCAACCTACTCAGCCAGGCCCAACTAAGCCACGAATGCAAAAAAACCAAAACGATGCAACCTATCAAACAAGTCCTTGGATATGGACTTTGA
5D-2100590-T10-13_64	AGTATTTATTAGGCCCGTTTAAGTTGCTTTTTTGAGACGGTATGATCCGGCCCATAGGCACTTCCATGGCAAATTGTTTAGTTCCATTTTTTCTTTTTTGCAATCGCTTTAGTTTCAACC
5D-2100590-T10-13_128	CATGGCAAATTGTTTAGTTCCATTTTTTCTTTTTTGCAATCGCTTTAGTTTCAACCCTTCCCTCAAAAAAGAAAACTTTACTTTCAACCCATTATAAGGGCCTACTTGATTCGCACGGTT
5D-2100590-T10-13_192	AAAAAAGAAAACTTTACTTTCAACCCATTATAAGGGCCTACTTGATTCGCACGGTTTTTAAAAACACAAAAATAAAAATAGGAAGAATGTAGCATTTCAATGTCATGACATATTGAATCT
5D-2100590-T10-13_256	CACAAAAATAAAAATAGGAAGAATGTAGCATTTCAATGTCATGACATATTGAATCTTATAGGAACCAAAAACACTGAAACATGCAATGTAAGTTGCTTGATTGCCCAACAAGAAAAATGC
5D-2100590-T10-13_320	CCAAAAACACTGAAACATGCAATGTAAGTTGCTTGATTGCCCAACAAGAAAAATGCAGGAATTTTTTTCAATAGCATTAGGTGTTTGACATAGTTGTTATGAAAACAAAGAATTTACCGC
5D-2100590-T10-13_384	TTTTCAATAGCATTAGGTGTTTGACATAGTTGTTATGAAAACAAAGAATTTACCGCAGCAAGTGCGGTCGGCAATCTTCAAATCATTCAAGAAACAAAAGAAATTTGCCATGAGGTTGGA
5D-2100590-T10-13_448	CGGTCGGCAATCTTCAAATCATTCAAGAAACAAAAGAAATTTGCCATGAGGTTGGACTCAATGAAAATTTCCCATGATGCATTTGCACAATAGATGGTACTCATGTGACTGGTAGAGTGC
5D-2100590-T10-13_1152	ACCGAGGCAATATGGGCAAACAAAGGTAACACAAGGATCTGATAAAGAAGAAGCAACAAAAGAAGATTCAGGAGTAGCAGAGGATGCATCGGCATCAGAGGAAGACGCAACAGCAG
5D-2100590-T10-13_1344	GCCATATGGCTGTTAGTTTGTAATTCAATTTGTTCATAGTAGGATGAACTGCCATTTGTTTAATAGCTGACTGAAACAATGTTTTATTTCAAACTTCATATGTGGTAAGGTCATCACTAG
5D-2100590-T10-13_1536	AGCCATCAAACGACACAGGGAAATTGCTTCCCTAATGCAGCCAACCTACATGCAGGGCCAACCTACTCAGCCAGGCCAAACTGAGTCACAAATGCAAAAAAACCAAAAATGATGCAACA
5D-2100590-T10-13_1580	CCTACATGCAGGGCCAACCTACTCAGCCAGGCCAAACTGAGTCACAAATGCAAAAAAACCAAAAATGATGCAACATATCAAACACGTCCTTGAATATGGACTTTGATCATGACTTCGTGA
7A-1833370-T10-14_64	TGAGAGCGGAATTCGGTGTTGAATGGTGAAGGTGGATGTAGCAATTCGCTTGCGTTCTCAGCAGGTGTTTTTTACAAGAGTCCTGAACCAAATGAAAATTGCACAATGTGCTAAAATGGA
7A-1833370-T10-14_128	GTGTTTTTTACAAGAGTCCTGAACCAAATGAAAATTGCACAATGTGCTAAAATGGATATGAACATAGGGTTGGAACTGGTTTTTAGAGAAATTTTTGGCCTTTTTAAGAAAACAAGTATG
7A-1833370-T10-14_192	TAGGGTTGGAACTGGTTTTTAGAGAAATTTTTGGCCTTTTTAAGAAAACAAGTATGTTTGTATCCAAATCATGGAATAATACAAAGTCCTTCAGCACGCTAGTACAGATTCTGACATGGC
7A-1833370-T10-14_256	CAAATCATGGAATAATACAAAGTCCTTCAGCACGCTAGTACAGATTCTGACATGGCAGGTTCTCTAGAAACTGAAAAAGATGTTGGACTTATCAACATTTACCAGTTGCCCCAAAGCGGC
7A-1833370-T10-14_320	TAGAAACTGAAAAAGATGTTGGACTTATCAACATTTACCAGTTGCCCCAAAGCGGCACAGTAAGAATCCACAACTGATTTCAAAGACTTGGCATCGTAAAAATTAGCTGGCATGAGGATA
7A-1833370-T10-14_384	AATCCACAACTGATTTCAAAGACTTGGCATCGTAAAAATTAGCTGGCATGAGGATAACGAGTCCTTAGAAGAGTGAAGATTGGTGATAGCAGGGACATCTTTGCAAACATTCGCTCTAGA
7A-1833370-T10-14_448	TTAGAAGAGTGAAGATTGGTGATAGCAGGGACATCTTTGCAAACATTCGCTCTAGAAATATTTTCATTCTCGTCAGCATGCGTAAGAAAGTAGTCCGACCCTCCGTGCATAACAAATATA
7A-1833370-T10-14_512	CATTCTCGTCAGCATGCGTAAGAAAGTAGTCCGACCCTCCGTGCATAACAAATATAAATAAGGAAATAAAGGATTTCCATGCATTAAACCTTTTGTAGGTTTGAAATCCTCACTTTGTTG
7A-1833370-T10-14_576	AATAAAGGATTTCCATGCATTAAACCTTTTGTAGGTTTGAAATCCTCACTTTGTTGCATGTTAAATCGGACTTGGTATTTAACTGTGGATACACATTACATAGTAAAAATTGACTCAATT
7A-1833370-T10-14_640	ATCGGACTTGGTATTTAACTGTGGATACACATTACATAGTAAAAATTGACTCAATTCTTCTGGAAGCCAAGTTTCAACATAATCCCCTTTAAGAAAGACCACTCAACCATATCAACAATG
7A-1833370-T10-14_704	AGCCAAGTTTCAACATAATCCCCTTTAAGAAAGACCACTCAACCATATCAACAATGCATACCAAGTTTGATAGTATACAAACCCTCCTTGCCTTCTCTCTTGTTTTTAATTGTATGAAAG
7A-1833370-T10-14_832	AGCCAGTAAAGCATTGTTTGTAATTATTCTTCCAGTCACAAAGCACTTTGAGTTGGACTAATATTCTCTAGCAGAAAAACCCTTCAAACGAGTATCCACTTTTTTTGCAACAATCTtgtc
7B-1930490-T10-14_128	TAGAGTCCTAAACCAAATGAAAATTAGATAATGCGCTAAAACGGATATGAATATAGGGAGTAGCTCTTAGAGAAAATTTTGCGAGGGCAGCGTTTCGATGCTCAGATTCATCTGCATCCG
7B-1930490-T10-14_576	CTAGGGCGAGTGCAGCTAAGCCTGAGCTCAGAAGTAGATTACCGAAGTTTTAGTCTTTTCTTTAGAAAGTATGCTTGTCTTTAAATCATGAAATCGTTAGAAAATGCAGTGTCTAGGAGG
7B-1930490-T10-14_640	GAAAGTATGCTTGTCTTTAAATCATGAAATCGTTAGAAAATGCAGTGTCTAGGAGGCGCTTAGGCTAGGAAGTGAATCCTCTAACATGAAGAAGAGAAGTCACCTAAGCTACAATTCCCT
7B-1930490-T10-14_1344	CGCTCACGCCTACCGCCTTTTTTAACCTAATAATACAAAATCCTTGGCATGGCGCCCGGGAGCGCTGTAGTGCAAATTTTGGCATGGTAGGTTCTTTATATGGACTTAAAAAGATGTTGG
7B-1930490-T10-14_1408	CTGTAGTGCAAATTTTGGCATGGTAGGTTCTTTATATGGACTTAAAAAGATGTTGGGCTTATCAACATTTATCATTTACCCCTGAGCGGCACAATAAGAATTCGGAACTGACTTCAAAGC
7B-1930490-T10-14_1472	ACATTTATCATTTACCCCTGAGCGGCACAATAAGAATTCGGAACTGACTTCAAAGCCTTGGTATTGTAAATATTAGTTTTtgaaagtgcaactaatcctcgggtaattttgataattaat
7D-2020240-T10-14_64	TGAGAACAGAATTCGGTGTTGAATGGTGAAGGTGGATGTAGCAATAGCTTGGGTTCGCGGCAGGTGGTTTTTACAATGGCGAGTGCTACAAATAAGCCGGATGATTAGAAATTCGCCTAT
7D-2020240-T10-14_256	TGAGCGCAGCACCATTGCAGCCCTGACCGACTCCAACGTAGCACCGAGCCACTGACGGCGTCCAGTGAGCTCCACTGCAGCTTCAGCGGACCTCCAACCGCAGCACTAACGCTCCGGC
7D-2020240-T10-14_640	AGCGTCCATTGCAACTCCGACGCCGGGATGAAGCGGGGTATGCAGGAGTGATGTCGCCGCGGAGATCTCGTCGCCGGCGTTTTGCAGCACCGCCGACCTTCGTAGCAACAGCAGCTCC
7D-2020240-T10-14_768	ATGGGAAGGTCATGGACAGGGAAGGGGAGAGGCGCTCGTGGAGAGAAAGAGGGTTGGGTTGCAGGGGATGAGGAGAGAGCAGCTTGGGCTCGCTGAGAGAAAGAGAGCTAGGCT
7D-2020240-T10-14_832	GGGATGAGGAGAGAGCAGCTTGGGCTCGCTGAGAGAAAGAGAGCTAGGCTGCAGGGATGGGAGAGGAGATCTGGAGGATGAGGACGACTCGTTTTGGATAGAAGATAAGGTTGGG
7D-2020240-T10-14_896	GGAGATCTGGAGGATGAGGACGACTCGTTTTGGATAGAAGATAAGGTTGGGGGTTGCGGGATGGACGGTTCGCATGGGGCGCGTGTCAACACCAGGAGGCGGCTTGCGGAAGCAAG
7D-2020240-T10-14_960	ACGGTTCGCATGGGGCGCGTGTCAACACCAGGAGGCGGCTTGCGGAAGCAAGAAATCATCCGGTTTAATTTAAACGTTTTCCTTTTACAATAGAGTCCTAAACCAAATGAAAATTACACA
7D-2020240-T10-14_1024	TTAATTTAAACGTTTTCCTTTTACAATAGAGTCCTAAACCAAATGAAAATTACACAATGTGCTTAAATGGATATGAACATAGGGTTGGAACTAGTTTTTAGAGAAAATTTTGATCTTTTT
7D-2020240-T10-14_1088	AAATGGATATGAACATAGGGTTGGAACTAGTTTTTAGAGAAAATTTTGATCTTTTTAAGAGAAAAAGTGTGTTTGTATCCAAATCACGAAATAATACAAAGTCCTTCGTATGGCATCTGG
7D-2020240-T10-14_1152	AAGTGTGTTTGTATCCAAATCACGAAATAATACAAAGTCCTTCGTATGGCATCTGGGAACACGCTAGTGCAAATTCTGGCATGGCAGGTTCTCTAGGGACTGAAGAAGATGTTGGACTTA
7D-2020240-T10-14_1216	TAGTGCAAATTCTGGCATGGCAGGTTCTCTAGGGACTGAAGAAGATGTTGGACTTATCAACATATACAATTTGCCCCGAAACAGCACAGTAAGAATCCAAAACTGACTTCAAAGACTCGG
7D-2020240-T10-14_1280	TACAATTTGCCCCGAAACAGCACAGTAAGAATCCAAAACTGACTTCAAAGACTCGGGATCGAAAATATTAGCTCTCATGAGGATAACAAGTCATCATAAAAGAGAATTGGTGATAGCAGG
7D-2020240-T10-14_1344	ATATTAGCTCTCATGAGGATAACAAGTCATCATAAAAGAGAATTGGTGATAGCAGGGGCATGTCTGCAAACATTTGCTCTAGAAATGTTCCCATTCTCATCTGCATGCGTTAAGAGAGTA
7D-2020240-T10-14_1408	TGCAAACATTTGCTCTAGAAATGTTCCCATTCTCATCTGCATGCGTTAAGAGAGTAGTCCGACCCTCCGTACATAACAAATGTAAATAAGGAAATAAAGGATTTCCCTGCATTAAACCTC
7D-2020240-T10-14_1472	CTCCGTACATAACAAATGTAAATAAGGAAATAAAGGATTTCCCTGCATTAAACCTCTTATAGGTTTAAACTCTCACTTTGTTGCGCGTTAAATCAGATTCGGTATTTAACTGTGGATACt
4B-1078310-T10-15_64	TAGTTTCGCCCCAGGCCCCTGTATTCTCAGGACCGGCCCTGGCAAGCCCCATGCTTAGCCACCCGAGGTTTTGCCAAGGATTGCGGGTTGAGTACTAAAAAATGCAGGGTCCTTTTTGTA
4B-1078310-T10-15_256	TCATAAAACGGGCGCGACGGTGAGCCCGACAGACTCAATGTGTGCTTTATTAGTAGGTAAAGATTATTGTCTTGTTGCATACGTGAATGTACCAAAGACTACAATATTTCATATGCGCCT
4B-1078310-T10-15_320	TATTGTCTTGTTGCATACGTGAATGTACCAAAGACTACAATATTTCATATGCGCCTGGATTGTGATATTCCAACATGGACAATGTGAAGAAATGAGTGACTTGTTATCTACATGCATGCC
4B-1078310-T10-15_384	ATATTCCAACATGGACAATGTGAAGAAATGAGTGACTTGTTATCTACATGCATGCCATATCCTGTAAACTGAAAATTTTAGCAATCAGCCACCAAAATGAATGCATGGCTTAATTCTATA
4B-1078310-T10-15_448	TAAACTGAAAATTTTAGCAATCAGCCACCAAAATGAATGCATGGCTTAATTCTATAATTTCTTCAGAAAACTCTAGTAGTAGAAATAGTTTTTTGTCATGTGTTTGTCCAAGTGGCATAT
4B-1078310-T10-15_512	AGAAAACTCTAGTAGTAGAAATAGTTTTTTGTCATGTGTTTGTCCAAGTGGCATATGTCAAGGTCGTTGTAACTTGGTTTAGTGGTTTACGTGCTTTTGATCCTCGTATCTTTATTGTGA
4B-1078310-T10-15_576	CGTTGTAACTTGGTTTAGTGGTTTACGTGCTTTTGATCCTCGTATCTTTATTGTGATGTGGCAAGCTGTAATTAGTGGTTAACTAGTGCCCATTTCTTGTCAACAAATATTTTCATTAAA
4B-1078310-T10-15_640	GCTGTAATTAGTGGTTAACTAGTGCCCATTTCTTGTCAACAAATATTTTCATTAAATCTGTTATTTTCTTGCCAAGGCATCTGGAATAGCTGCATTAGATGTGAAAAAGCTCAAAGATGC
4B-1078310-T10-15_704	TTTCTTGCCAAGGCATCTGGAATAGCTGCATTAGATGTGAAAAAGCTCAAAGATGCCAGTCTCTACACAGTAGAGTCTCTGGCTTACACTCCAAGATCTTTTGCGAATCAAAGGAATCAG
4B-1078310-T10-15_768	ACACAGTAGAGTCTCTGGCTTACACTCCAAGATCTTTTGCGAATCAAAGGAATCAGTGAGGCCAAAGTTGAGAAGATAATTAAGGCAAGCAAGTAATCATGAGATCAGAATTGTATTTTG
4B-1078310-T10-15_832	AAGTTGAGAAGATAATTAAGGCAAGCAAGTAATCATGAGATCAGAATTGTATTTTGCATGTAAATTTTATAGCACACTGTAAAATATTTACTGTCTACAAATGTTTGCAGCATCCAGGCT
4B-1078310-T10-15_896	TTTTATAGCACACTGTAAAATATTTACTGTCTACAAATGTTTGCAGCATCCAGGCTTGTTCCGCTGGGATTTACAAGTGCCAACCAACTACATGCACAGAGGCTAGAGATCATTCAAGTT
4B-1078310-T10-15_960	TGGGATTTACAAGTGCCAACCAACTACATGCACAGAGGCTAGAGATCATTCAAGTTAGAACTGGATCAAGGGAGCTTGATAAGATCTTGGATGGTAATGTGCTACCAGCTTTATGCTTTT
4B-1078310-T10-15_1024	ATCAAGGGAGCTTGATAAGATCTTGGATGGTAATGTGCTACCAGCTTTATGCTTTTTCCCTTTCTCAGCATAATTTGATAAATGACTTGTGATCCATTGAGTATCAGATCTTACCTACCT
4B-1078310-T10-15_1088	TCAGCATAATTTGATAAATGACTTGTGATCCATTGAGTATCAGATCTTACCTACCTTTAACAGGGGGATAGAAACAAGATCTATCACAGAGTTATATGGTGAATTCCCCTCAGGAAAGAC
4B-1078310-T10-15_1152	GGGATAGAAACAAGATCTATCACAGAGTTATATGGTGAATTCCCCTCAGGAAAGACTTAGTTGTGCCACACTGTGTTGTTACATGCCAGATGAGACTTCATTTATTGTTTTTTTATTTGC

4B-1078310-T10-15_1216	GCCACACTGTGTTGTTACATGCCAGATGAGACTTCATTTATTGTTTTTTATTTGCATTGACACCTAAGTCTTGTTGTTAATCTCTCATATGTTAAACATGATATATTTCAGCTTCCATT
4B-1078310-T10-15_1408	CCAGATAGTAGACATGTGGCACCTTTGGTTACACTAGTTACTTTGGTTCTTAGCATGAATTTTATTAATAAGCAAGTGAATACTAGATTCTAGGTTGGTTTTAACATGAATTTGATTACT
4B-1078310-T10-15_1472	TTAATAAGCAAGTGAATACTAGATTCTAGGTTGGTTTTAACATGAATTTGATTACTAAGCAAATGAATATTGGATTCTAGGTTGGTTTTAACATGAATTTGGTTATTAAACAAGTGACTA
4B-1078310-T10-15_1536	GAATATTGGATTCTAGGTTGGTTTTAACATGAATTTGGTTATTAAACAAGTGACTACTGGACTCTGGGTTTGGATTGAACGGTGCTGATGTATTAGAGAATGTGGCTTATGCCAGGGCTT
4B-1078310-T10-15_1580	AACAAGTGACTACTGGACTCTGGGTTTGGATTGAACGGTGCTGATGTATTAGAGAATGTGGCTTATGCCAGGGCTTACAATACTGATCATCAATCCAGACTTCTACTGGATGCAGCTTCC
1B-0173560-T10-16_1024	AGAAAAGCTAAAAACTAGTAGTTGATTATTCATCCCTCTCTAGTTAACCATATCAATCCTTTTAGAGAGGATGAAGAGCCCAAAAAAATCAACCACAATTAGAAATATGAAAGGGATGAG
1B-0173560-T10-16_1088	GAGAGGATGAAGAGCCCAAAAAAATCAACCACAATTAGAAATATGAAAGGGATGAGCTTTTCATTAAAGGATAGTAGGAGAAAGGAGCTGAATTGAGAGTGATAGCTTGGCTTCAAGA
1B-0173560-T10-16_1152	TAAAGGATAGTAGGAGAAAGGAGCTGAATTGAGAGTGATAGCTTGGCTTCAAGAAATCGCAGGAAAATTAGTTAGTACGATTCAACTAATTTATGGATATGCCCCACAAAAAATACGAAA
1B-0173560-T10-16_1216	AAATTAGTTAGTACGATTCAACTAATTTATGGATATGCCCCACAAAAAATACGAAATTTATGGATAGAGGATGACACAAAAGAAACCATGTAAACTACTGAATGTTTTTTGAATCAAAGG
1B-0173560-T10-16_1280	TAGAGGATGACACAAAAGAAACCATGTAAACTACTGAATGTTTTTTGAATCAAAGGCTTTTTCATATGAATTTTAAAGTACTCCCTCGGTCCCATAATGTAAGATATTTTTTAACATTAG
1B-0173560-T10-16_1408	CAAAAAAGTCTTATATTATAGGAAGGAGGGAGTATTATAATCCTTAGAATTTTCTTCCATGTGGCTGTTTGATTTGTGAGGTTGAATCCCATATAAAAATATTTGCTACGGCAATGTTAT
1B-0173560-T10-16_1472	CTGTTTGATTTGTGAGGTTGAATCCCATATAAAAATATTTGCTACGGCAATGTTATCCTTCATTTCGGAAAAATAATCATCATTTGCTGAAAATAGCTCTTCCTCGAATAAAGCGAACGA
1B-0173560-T10-16_1536	TCGGAAAAATAATCATCATTTGCTGAAAATAGCTCTTCCTCGAATAAAGCGAACGAAGAAATCCACCGCAAAAACTCAAAAAGAAAGGAGAGCGCGGAAACAAAACTAAAATTTCCGGA
1D-0240760-T10-16_64	TTTCCGCGCTCTCCTTTCTTTTTGAGTTTTTGCGGTGGATTTCTTCGTTCGGTTTATTCGGGCAAGAGCTATTTCCAGCAAATGGTTTCTTGGGAGAGATTTCAGCCAAATGATGATTAT
7D-2020240-T10-16_64	TGAGAACAGAATTCGGTGTTGAATGGTGAAGGTGGATGTAGCAATAGCTTGGGTTCGCGGCAGGTGGTTTTTACAATGGCGAGTGCTACAAATAAGCCGGATGATTAGAAATTCGCCTAT
1D-0240760-T10-16_128	AGAGCTATTTCCAGCAAATGGTTTCTTGGGAGAGATTTCAGCCAAATGATGATTATTTTTCCGAGATGAAGGATAACATTACCCTAGCAAATATTTTTATATGGGATTCAACCTCACAAA
1D-0240760-T10-16_192	GATGAAGGATAACATTACCCTAGCAAATATTTTTATATGGGATTCAACCTCACAAATCAAACAACCACATGGAAGAAAATTCTAAGGATTATAATACTTTGGAATTCATATGGAAAAGCC
1D-0240760-T10-16_256	CCACATGGAAGAAAATTCTAAGGATTATAATACTTTGGAATTCATATGGAAAAGCCTTTGATTCAAAAAACATTCAGTAGTTTAGATGGTTTCTTTTGTGCCATCCTCTATTCATAAATT
7D-2020240-T10-16_256	TGAGCGCAGCACCATTGCAGCCCTGACCGACTCCAACGTAGCACCGAGCCACTGACGGCGTCCAGTGAGCTCCACTGCAGCTTCAGCGGACCTCCAACCGCAGCACTAACGCTCCGGC
1D-0240760-T10-16_320	AAAAAACATTCAGTAGTTTAGATGGTTTCTTTTGTGCCATCCTCTATTCATAAATTTCTTATTTTTTGTGGGGCATATCCATAAAcggaaaaactaacgcccacatgtgtgggcgtttgc
7D-2020240-T10-16_640	AGCGTCCATTGCAACTCCGACGCCGGGATGAAGCGGGGTATGCAGGAGTGATGTCGCCGCGGAGATCTCGTCGCCGGCGTTTTGCAGCACCGCCGACCTTCGTAGCAACAGCAGCTCC
7D-2020240-T10-16_768	ATGGGAAGGTCATGGACAGGGAAGGGGAGAGGCGCTCGTGGAGAGAAAGAGGGTTGGGTTGCAGGGGATGAGGAGAGAGCAGCTTGGGCTCGCTGAGAGAAAGAGAGCTAGGCT
7D-2020240-T10-16_832	GGGATGAGGAGAGAGCAGCTTGGGCTCGCTGAGAGAAAGAGAGCTAGGCTGCAGGGATGGGAGAGGAGATCTGGAGGATGAGGACGACTCGTTTTGGATAGAAGATAAGGTTGGG
7D-2020240-T10-16_896	GGAGATCTGGAGGATGAGGACGACTCGTTTTGGATAGAAGATAAGGTTGGGGGTTGCGGGATGGACGGTTCGCATGGGGCGCGTGTCAACACCAGGAGGCGGCTTGCGGAAGCAAG
7D-2020240-T10-16_960	ACGGTTCGCATGGGGCGCGTGTCAACACCAGGAGGCGGCTTGCGGAAGCAAGAAATCATCCGGTTTAATTTAAACGTTTTCCTTTTACAATAGAGTCCTAAACCAAATGAAAATTACACA
7D-2020240-T10-16_1024	TTAATTTAAACGTTTTCCTTTTACAATAGAGTCCTAAACCAAATGAAAATTACACAATGTGCTTAAATGGATATGAACATAGGGTTGGAACTAGTTTTTAGAGAAAATTTTGATCTTTTT
7D-2020240-T10-16_1088	AAATGGATATGAACATAGGGTTGGAACTAGTTTTTAGAGAAAATTTTGATCTTTTTAAGAGAAAAAGTGTGTTTGTATCCAAATCACGAAATAATACAAAGTCCTTCGTATGGCATCTGG
1D-0240760-T10-16_1152	acatgacGTAGCGTTGTCCTCCATAAATTAGTTGAATCCTACTAACTAATTTTCCTGCGATTTCTTGAAGCCAAACCATCACCCTCAATTCGGCTCCTTTCTGCCATATCCTTTTATGAA
7D-2020240-T10-16_1152	AAGTGTGTTTGTATCCAAATCACGAAATAATACAAAGTCCTTCGTATGGCATCTGGGAACACGCTAGTGCAAATTCTGGCATGGCAGGTTCTCTAGGGACTGAAGAAGATGTTGGACTTA
1D-0240760-T10-16_1216	TTGAAGCCAAACCATCACCCTCAATTCGGCTCCTTTCTGCCATATCCTTTTATGAAAACTCGTCTCTCCCATATTTCTGATTGTGGTTGATTTTCTTTGGGCTCTTCATCTTCTAtgaaa
7D-2020240-T10-16_1216	TAGTGCAAATTCTGGCATGGCAGGTTCTCTAGGGACTGAAGAAGATGTTGGACTTATCAACATATACAATTTGCCCCGAAACAGCACAGTAAGAATCCAAAACTGACTTCAAAGACTCGG
7D-2020240-T10-16_1280	TACAATTTGCCCCGAAACAGCACAGTAAGAATCCAAAACTGACTTCAAAGACTCGGGATCGAAAATATTAGCTCTCATGAGGATAACAAGTCATCATAAAAGAGAATTGGTGATAGCAGG
7D-2020240-T10-16_1344	ATATTAGCTCTCATGAGGATAACAAGTCATCATAAAAGAGAATTGGTGATAGCAGGGGCATGTCTGCAAACATTTGCTCTAGAAATGTTCCCATTCTCATCTGCATGCGTTAAGAGAGTA
7D-2020240-T10-16_1408	TGCAAACATTTGCTCTAGAAATGTTCCCATTCTCATCTGCATGCGTTAAGAGAGTAGTCCGACCCTCCGTACATAACAAATGTAAATAAGGAAATAAAGGATTTCCCTGCATTAAACCTC
7D-2020240-T10-16_1472	CTCCGTACATAACAAATGTAAATAAGGAAATAAAGGATTTCCCTGCATTAAACCTCTTATAGGTTTAAACTCTCACTTTGTTGCGCGTTAAATCAGATTCGGTATTTAACTGTGGATACt
2A-0365750-T10-17_128	GGAGTCAGGAGAAtttgttttattttattttattttattttaGAGCTTCCGGCTCTGCCGCCGGGAAAGAAGAGGGAAGCGGAGGCGGGAAGGAGAGACTTTTACAGATCTCTAGCCGC
2A-0365750-T10-17_192	GGAAAGAAGAGGGAAGCGGAGGCGGGAAGGAGAGACTTTTACAGATCTCTAGCCGCGTCCCAAGAAGGCCTTCAGACGGTTTTTTGATCGTCAACTTCAAAAAATCGGCCAGTCGCG
2B-0448650-T10-17_0	GTCGCATTCGCGGAGCACGGCGTAGGCCTCCTCGTCGGTGCGGTTCCTGGCGACCCCCTTGCTGTAGTGGTGTGCTAGATTGTCCGCCTCACTGCCGCCGGAATAGAAAGGGGACAGAA
2B-0448650-T10-17_62	TGTAGTGGTGTGCTAGATTGTCCGCCTCACTGCCGCCGGAATAGAAAGGGGACAGAAGCGGAGGCGGGAGGGAGAGAGATTTATCATTTTCGGAGAGTTTTGCAGGTAGCAAGTCCA
2B-0448650-T10-17_124	GGCGGGAGGGAGAGAGATTTATCATTTTCGGAGAGTTTTGCAGGTAGCAAGTCCAACCGAGGCCAAATTTGGTCTTACCTTTTGGCAAAGTTTAGCCCGATCAAACTGCCATTTAAAAC
2B-0448650-T10-17_186	CCAAATTTGGTCTTACCTTTTGGCAAAGTTTAGCCCGATCAAACTGCCATTTAAAACTACTAGTTAATTGTCCGTGCGTTGCAACAGAGCTAACCTTTTAGTGTGTGTCACTCTCTGAAA
2B-0448650-T10-17_248	GTTAATTGTCCGTGCGTTGCAACAGAGCTAACCTTTTAGTGTGTGTCACTCTCTGAAAACAACAATAAAAAACAGTCCACATCAAAGGAGGGCACTCGTGGTTTGTTCGATGATCTTCTG
2B-0448650-T10-17_310	CAATAAAAAACAGTCCACATCAAAGGAGGGCACTCGTGGTTTGTTCGATGATCTTCTGTGGCACCAGCCGTGCCTAGTTCTCTCTCGTGGTTCTCTGTGAGAGTCGGAGCTGCGCTGCCT
7A-1794510-T10-18_256	CGTCGTAGCTTGGAGGCGGGGGTGACGTGAGGGGAGCGGGAGGAGGGCGGCGGGCCTTCGGTCCAATggcggcggggcgcggcagaggcggcggccggcgggggcggggcaggggacgca
7A-1794510-T10-18_320	AATggcggcggggcgcggcagaggcggcggccggcgggggcggggcaggggacgcacggggacgggacggAATGGGATCGAAACATGGAGCGCCTGCCTCTGTCGGCTTAGTGGGCCTCC
7A-1794510-T10-18_448	GCCTTAGTGGGCTGTTCTAGTTGGGCTGGAACCCGGGCCGTTTTTATCATTGGACTACTCGCCTTGATATCTTCCAAACCATTATGTTGATAGGTTTCGACAAACCCTATGATACTCATG
7A-1794510-T10-18_512	TGATATCTTCCAAACCATTATGTTGATAGGTTTCGACAAACCCTATGATACTCATGGGTATCCAATCCACGCCTTCAACCCAAACCTGGCCATGCTTTTGCCAAAATAAAAACCTGACCA
7A-1794510-T10-18_576	ATCCACGCCTTCAACCCAAACCTGGCCATGCTTTTGCCAAAATAAAAACCTGACCATGCTGGCGGATGCGTCTGGACACGCAAATCTTTACCTAATAACTAGACAATTGCCCGTGCGTTG
7D-2002290-T10-18_0	GTGGGCTCTCCCTCCCTGTCCAGAGCCGAGCAGTAGGCAAGCCGTGCAGCAGGCCAACGACTCCTGCGCGAGGGGAGTGGACGAGCAGTTGCGCGAGACCGCTGtcctccacccctcctc
7D-2002290-T10-18_320	AGCTTGGAGGGAGGGGGGCGGGAGGAGGACAGCGGGGCGTCCGTCCAGTGGTGGCGCGGCGCAGCAGAGGCGGCGGCCGGCGGGGCAGGGCACACACTCACACGGGACGGAA
7D-2002290-T10-18_384	CAGAGGCGGCGGCCGGCGGGGCAGGGCACACACTCACACGGGACGGAACGCGGAACGGGATAGCGCCTGCCTCTGTCAGCTTAATGGGCCTCCCTGTGCGTGCCTTGGTGGGTTGTT
7D-2002290-T10-18_512	GGACCCAGGccgattttacaataattactcgccttgagatcttcaaaactagatccatgttaatgtGCCTAAAAAAAGATCTATATTAATGAATTTGGACAaaaaaataaaaaactgaat
7D-2002290-T10-18_576	gtGCCTAAAAAAAGATCTATATTAATGAATTTGGACAaaaaaataaaaaactgaataaaaatagcaaaaaacataaatgaaaaagcaaaataaaaatgaaaaCCATCGAAATCCGAAAAA
7D-2002290-T10-18_640	caaaaaacataaatgaaaaagcaaaataaaaatgaaaaCCATCGAAATCCGAAAAAATAGAAACCTGGAAGTGCAGTTGTGTTTTTTCAAAAAATAAATAAACTAGAAGCACAACTATGC
7D-2002290-T10-18_704	CTGGAAGTGCAGTTGTGTTTTTTCAAAAAATAAATAAACTAGAAGCACAACTATGCTTTTCTCTTTTTAGGAAGAACATTTTTGCTTTTTCGTTGAAGCATAGATACGCTTCTCCCTTTT
7D-2002290-T10-18_768	TTTTAGGAAGAACATTTTTGCTTTTTCGTTGAAGCATAGATACGCTTCTCCCTTTTCTGGAGAAGCACATGACTGTGCTTTTTTCTTTCAGATTGTGCTTCACTTTTTGGGAAGCAGAAC
7D-2002290-T10-18_832	GCACATGACTGTGCTTTTTTCTTTCAGATTGTGCTTCACTTTTTGGGAAGCAGAACATCGTGAAAAAGCATTGCTTCTAAGAACCCTTTTTTTTTTGACAAAACCTAGGGAAAATCAAAC
7D-2002290-T10-18_896	AAAGCATTGCTTCTAAGAACCCTTTTTTTTTTGACAAAACCTAGGGAAAATCAAACAAGAACCTGAAAGCCAAAAAACAAAAATCGTTATAACACGTGCAGAAAAATAATCCAAAATCAC
7D-2002290-T10-18_960	GAAAGCCAAAAAACAAAAATCGTTATAACACGTGCAGAAAAATAATCCAAAATCACAAGGGTGCGCCAGCTCATGACACCTGGTGGCTACTGGGCACACTAATTGAGGCGCTCCCAAGG
7D-2002290-T10-18_1024	GCCAGCTCATGACACCTGGTGGCTACTGGGCACACTAATTGAGGCGCTCCCAAGGCATGCAATTGACCTCCAAGGGAGCCCTCCAAATGACACTCCTTAATTAATTGCTCCCTTGTTTTG
7D-2002290-T10-18_1088	GACCTCCAAGGGAGCCCTCCAAATGACACTCCTTAATTAATTGCTCCCTTGTTTTGAAAGAGGAAATGGCCGATGCTACGATGGAATTGTTCGCAAATAAACCTGGCTTGGGTCACACAC
7D-2002290-T10-18_1152	AATGGCCGATGCTACGATGGAATTGTTCGCAAATAAACCTGGCTTGGGTCACACACCCATTCTAAACACCCTAAAACCTATGAAACCCGTCAGGATCCAATCCACGCCTTCAACCCAAAC
7D-2002290-T10-18_1216	AACACCCTAAAACCTATGAAACCCGTCAGGATCCAATCCACGCCTTCAACCCAAACATGGCTATGAGGGTCGGCACTTTCGGACACACAATATGTCGGCCTTTTTATAAAATGGTCGTGT
7D-2002290-T10-18_1280	GAGGGTCGGCACTTTCGGACACACAATATGTCGGCCTTTTTATAAAATGGTCGTGTCGTGCCAGTCGAAGGCCTCGGCTCAAGGATCGGCCCGACTTGTGACATAAAAAGGCCAACATGC
7D-2002290-T10-18_1344	TCGAAGGCCTCGGCTCAAGGATCGGCCCGACTTGTGACATAAAAAGGCCAACATGGCCTGGCACACATGTAGGTCAGCCTTCATTCATTTTCATCCTTTCGAGTCTTCTACATTTTAAGA
7D-2002290-T10-18_1408	ACATGTAGGTCAGCCTTCATTCATTTTCATCCTTTCGAGTCTTCTACATTTTAAGAATTAAGAAAATCTGAAAATTTATGGAAAAATCTAAATAAATAAGAAATACACAAATATAAAAGT
7D-2002290-T10-18_1472	AATCTGAAAATTTATGGAAAAATCTAAATAAATAAGAAATACACAAATATAAAAGTTAAGAAAAAATACAAAAAATGTAAAAGAGACGAAGTGTCATATAGATAAATATAGCTGCCTCGT
7D-2002290-T10-18_1536	AATACAAAAAATGTAAAAGAGACGAAGTGTCATATAGATAAATATAGCTGCCTCGTAAAAACCTTTTAGTTAAACTCCCCTTGAAGACGAACCCAATAAAAAAAAGAGTACAAAGCTACT
7D-2002290-T10-18_1580	TAGCTGCCTCGTAAAAACCTTTTAGTTAAACTCCCCTTGAAGACGAACCCAATAAAAAAAAGAGTACAAAGCTACTTGCTCGAAAATGAGAATTATAAAAGTAAAAAAACACAAAAGAAA
6A-1520570-T10-19_320	GCCCTGTGACTGTGAGAACAAATCACTGGCCTGAACGAGGGTTTCTCCATTCTACAGGTATTGCGAATGGGTCGGCCCATTTTCTTCTTTTCTTTCTGCTATTTTTTCATTTTCTGTTTC
6A-1520570-T10-19_384	GAATGGGTCGGCCCATTTTCTTCTTTTCTTTCTGCTATTTTTTCATTTTCTGTTTCTTATCTACTAGTTTTCTCATCCAGTTCTCCAGCTTTTTGATCCGTTCTCTATTTTCGTTTTTGC
6A-1520570-T10-19_448	TAGTTTTCTCATCCAGTTCTCCAGCTTTTTGATCCGTTCTCTATTTTCGTTTTTGCTTATTATTTACTTTTCATTTCCTGGTTAAATTTTTTGTTTCTTTTTCTTGTGCGTGACATAAAT
6A-1520570-T10-19_512	TACTTTTCATTTCCTGGTTAAATTTTTTGTTTCTTTTTCTTGTGCGTGACATAAATTTAGTCGTGCATGATCATTTTGTCAAAATCGAAAACTTAACTACCCATTTGACACTTAACTCCT
6A-1520570-T10-19_704	AAGCTAGCAGTTTCTTCTTTTTCGGTTTTTAATCTTCATTTCAGTTTTCTTCTATTTTCAAATTTATTTCCCTTTTTTCATCAAGTTTACTATCAAACTATATCTCACGTGTATGCTACC
6A-1520570-T10-19_768	TATTTCCCTTTTTTCATCAAGTTTACTATCAAACTATATCTCACGTGTATGCTACCTGTTGAACTAGTTCATAGCGGCACACGTTAAAAATTCATAAAATACTTGAAATGGGACATAGGT
6A-1520570-T10-19_832	TAGTTCATAGCGGCACACGTTAAAAATTCATAAAATACTTGAAATGGGACATAGGTCGAAGCACATTCATTGTGGAAAGTGCTGCCAGTCACGGCTTGCAATTTGTTCATGTACAGAAGT
6A-1520570-T10-19_896	ATTCATTGTGGAAAGTGCTGCCAGTCACGGCTTGCAATTTGTTCATGTACAGAAGTACACATCAGTGTATATGAGCAATACACAACCAACAATGGGTCATTGCGGTGTCATGAGATGTTT
6A-1520570-T10-19_960	GTGTATATGAGCAATACACAACCAACAATGGGTCATTGCGGTGTCATGAGATGTTTGGTGCGGATACGCATCAGTGCACTTGAATCGATTTCTCAGGGTGAAAACCTAGATTCTAGCCAT
6A-1520570-T10-19_1408	TATCCAAAAAAACAACGGGTCATTGGCCCCAAGAGTCCAATCCCAATGAAGTAACAACATAATGGCGTTGTCGTAGTCCGATCCAAGGTTGGATCCGAAACTTTCACTTAGAGCACACAT
6A-1520570-T10-19_1472	GCGTTGTCGTAGTCCGATCCAAGGTTGGATCCGAAACTTTCACTTAGAGCACACATGAGGTGGACATGATGCGGAAGCTTAAGACAAAGCCTCTGTTATCGACGCACGCATTGCATAATC
6A-1520570-T10-19_1536	CATGATGCGGAAGCTTAAGACAAAGCCTCTGTTATCGACGCACGCATTGCATAATCGCATCTGGAAGTCTTTCACTCGTCGGAATGGGCCAGATCGGGTGTGAGAAGAAGAAGGGGAAG
6A-1520570-T10-19_1580	CATTGCATAATCGCATCTGGAAGTCTTTCACTCGTCGGAATGGGCCAGATCGGGTGTGAGAAGAAGAAGGGGAAGGAGGAACTGAGATGAACCGAAGGTAAAGTACATTTAAAACATT
5A-1244210-T10-20_192	GAATGTGGATCACTGCGGCTGCGACGAGGTGGGGGTATTTGAAGGCCTCGCGCGAGGAACTTGAGAGCCCAACACTTGTTGCTGCTGCCAGCTAAGCCTTCCACGGCCCATTCGAAAT
5A-1244210-T10-20_256	GAGCCCAACACTTGTTGCTGCTGCCAGCTAAGCCTTCCACGGCCCATTCGAAATTCAAAATTTTGAACAGGACGGCGACGTGCCAATCAAGCAATCTAGTAACAACAGCTTTGATTCTTA
5A-1244210-T10-20_320	GAACAGGACGGCGACGTGCCAATCAAGCAATCTAGTAACAACAGCTTTGATTCTTAACCCGTTTGATATACCAACTCTTTTGTAAAAGGTGTTCTTGTTAGGGTGATCACCTTGCAATAA
5A-1244210-T10-20_384	GATATACCAACTCTTTTGTAAAAGGTGTTCTTGTTAGGGTGATCACCTTGCAATAAAAAAGTACTTTGATGTTAAAATAAAAGTGTATCCTCTCGTTAGGTTTACTAGCAAACATGCATG
5A-1244210-T10-20_448	TTTGATGTTAAAATAAAAGTGTATCCTCTCGTTAGGTTTACTAGCAAACATGCATGTGTGTTGCAGTGGGAGAAAAACACACGCTCCTAAACCCAATATGCAAGGTAATGGGAATGTACA
5A-1244210-T10-20_512	AGTGGGAGAAAAACACACGCTCCTAAACCCAATATGCAAGGTAATGGGAATGTACATGAAGAAAAATTGTGATTTAGCCAACCAATCTTTGCAAACGGCTAACAAACTTATGCATTGTA
5A-1244210-T10-20_576	AATTGTGATTTAGCCAACCAATCTTTGCAAACGGCTAACAAACTTATGCATTGTACAATTTGCGGGTTAGGTTAAAGACAATCACCTACTAAAAAAAGAGTTACAGACAATCATCTCTA
5A-1244210-T10-20_640	GGGTTAGGTTAAAGACAATCACCTACTAAAAAAAGAGTTACAGACAATCATCTCTAACAGAAATGAAAAAAATAAACTCAAGATAAGAATAAAAAGACATACAAAAGATAACAAAATTGG
5A-1244210-T10-20_704	GAAAAAAATAAACTCAAGATAAGAATAAAAAGACATACAAAAGATAACAAAATTGGAATTACTGAAACATAATTTCTTCTGCCAATGCAAGGTATTATTTCTCGCCTTCATTTCATTATC
5A-1244210-T10-20_768	AAACATAATTTCTTCTGCCAATGCAAGGTATTATTTCTCGCCTTCATTTCATTATCAGAAGAATGAATATTTCAGATTTAATACATCTTTCAATAGGAATAATGCGTACTATAATCAAGC
5A-1244210-T10-20_832	GAATATTTCAGATTTAATACATCTTTCAATAGGAATAATGCGTACTATAATCAAGCATGTGCTAGGTTGTCACTGACAAAGCATATTGCGGTACTATAATCAAGCATAGACACGGTTGAC
5A-1244210-T10-20_896	GGTTGTCACTGACAAAGCATATTGCGGTACTATAATCAAGCATAGACACGGTTGACTACCATCCTTAGGTTTATAGTGTGCGTATATCTCAACAAACAAACCAAATATAAGTGCAAAAGA
5A-1244210-T10-20_960	TTAGGTTTATAGTGTGCGTATATCTCAACAAACAAACCAAATATAAGTGCAAAAGATATAGATTATCAACCATAACAGAAGGTTAGTATACAATGCATATTAAGCATTATACAGAAAATA
5A-1244210-T10-20_1024	ATCAACCATAACAGAAGGTTAGTATACAATGCATATTAAGCATTATACAGAAAATAAAAATGTCATACATGCAGCATTTCTATTGAGTTACTAACATCATACAATGAAAATGTTGCACAC
5A-1244210-T10-20_1088	ATACATGCAGCATTTCTATTGAGTTACTAACATCATACAATGAAAATGTTGCACACATCGGACGTCCATGCATACATGCTTCCGTTGTGTGTGTGCTAACCACGAAATCCTCGCCGTATA
5A-1244210-T10-20_1152	TCCATGCATACATGCTTCCGTTGTGTGTGTGCTAACCACGAAATCCTCGCCGTATAGGTTTACTCTTTCCTCTTCTGTTGCGATATATGCAGCGCTCTCCATCACAATTGAATATTTATA
5A-1244210-T10-20_1472	TTCACGGCGTTTTTTTACTTTTTCATATTTTTCTTCATTGGTCATCCGTGCATGCTGCTCTGTATCATTTTGCTTCTGTTACTTGGTATCAATCTCTACTCCTAATGGAGCAGTTGGTAG
5B-1334750-T10-20_128	TAAAGGTGGAATATCAAAGAAATTTGGAGATTCAGGGGTACCAACTACGGAGTGGATCCCTAGATCGCATCCTGGGCCGGACGGAGCAGTTGTAGGAAGTCGCAGCCCGTGGGTGGGG
5B-1334750-T10-20_192	TCGCATCCTGGGCCGGACGGAGCAGTTGTAGGAAGTCGCAGCCCGTGGGTGGGGGGAGGGGGTACCAGCGCCACCAGGGAGCTGGAGCCGTCGTGGGAGGGGGGACGCCCGCAC
5B-1334750-T10-20_512	AAGCGACGCTAGGGTCGGGGGTCGGGCGTGAGCTTCAGAGCAAGGACTTGTTGAGATATACGCACACTATGAACCTAAGGATGGTAGTCAACCGTGTCTATGCTTGATTATAGTACCGCA
5B-1334750-T10-20_576	CACTATGAACCTAAGGATGGTAGTCAACCGTGTCTATGCTTGATTATAGTACCGCAATATGCTATGTCAGTGACAACCTAGCACATGCTTGATTATAGCACACATTATTCCTATTGAAAA
5B-1334750-T10-20_640	TGTCAGTGACAACCTAGCACATGCTTGATTATAGCACACATTATTCCTATTGAAAAATGTATCAAATTTGAAATATTCATTCTTCCGATAATGAAATGAAGGCGAGAAATAATACCTTGC
5B-1334750-T10-20_704	AATTTGAAATATTCATTCTTCCGATAATGAAATGAAGGCGAGAAATAATACCTTGCATTGGCAGAAGAAATTATGTTTCAGTAATTTCAATTTTGTTATCTTTTGTATGTCTTTTTATTC
5B-1334750-T10-20_768	AAGAAATTATGTTTCAGTAATTTCAATTTTGTTATCTTTTGTATGTCTTTTTATTCTTATGTTGAGTTTATTTTTTTTCATTTCTGTTAGAGATTATTGTCTTTAACCCTTTTTTATAGG
5B-1334750-T10-20_832	AGTTTATTTTTTTTCATTTCTGTTAGAGATTATTGTCTTTAACCCTTTTTTATAGGTGATTGTCTTTAACCTAACCCGCAAATTGTACAATGCATAAGTTTGTTAGCCGTTTGTAAAGAT
5B-1334750-T10-20_896	TTTAACCTAACCCGCAAATTGTACAATGCATAAGTTTGTTAGCCGTTTGTAAAGATTGGTTGGCTAAAATCACAATTCTCGTTCATGTACATTTTCATCACCATGCATGTTGGGTTTAGG
5B-1334750-T10-20_960	TAAAATCACAATTCTCGTTCATGTACATTTTCATCACCATGCATGTTGGGTTTAGGAGCGTGTGTTTTTCTCCCATTATAACGCACATGCATGTTTGCTAGTAAATCTAATGAGAGGGTA
5B-1334750-T10-20_1024	TTTTTCTCCCATTATAACGCACATGCATGTTTGCTAGTAAATCTAATGAGAGGGTACACTTTTATTTTGACATCAAAGTACTTCCTCCGTTCTTAAATATTTGTCCTTTTAGAGATTTCA
5B-1334750-T10-20_1280	AGGTGATCACCCTAACAAGAGGGGACGCCTTTTACAAAAGAGTTGGTACATCAAACGGGTTAAGAACCAAAGCGGTTGTTACTAGTAGATTGCTTGATTGGCACGTCGGCGTCCTGTTCA
5B-1334750-T10-20_1344	AACCAAAGCGGTTGTTACTAGTAGATTGCTTGATTGGCACGTCGGCGTCCTGTTCAAAGTTTTGAATTTCGAATGGGCCGTGCAAGGCTTAGCTGGCAGGAACAAGTTGCTCGCGCGAG
7D-2002290-T10-20_0	GTGGGCTCTCCCTCCCTGTCCAGAGCCGAGCAGTAGGCAAGCCGTGCAGCAGGCCAACGACTCCTGCGCGAGGGGAGTGGACGAGCAGTTGCGCGAGACCGCTGtcctccacccctcctc
5D-1449300-T10-20_192	GTGGATCACTGCGGCTGCGACGAGGTGGGGGTTATTTGAAGGCCTCGCACGAGGAACTTGTGAGCCGTAACACCAAGCCCACGCTCGTTGCTGCTGCCAGCTAAGCCTTTTACGGCCCA
5D-1449300-T10-20_320	CAAAATTTTGAACAGGACGCCGACGTGCCAATCAAGCAATCTAGTAAGAACCGCTTTGATTCTTAACCCGGTTGATATACCAACTCTTTTGTAAAAGGTGTTCCCTCTTGTTAGGGTGAT
7D-2002290-T10-20_320	AGCTTGGAGGGAGGGGGGCGGGAGGAGGACAGCGGGGCGTCCGTCCAGTGGTGGCGCGGCGCAGCAGAGGCGGCGGCCGGCGGGGCAGGGCACACACTCACACGGGACGGAA
5D-1449300-T10-20_384	AACCCGGTTGATATACCAACTCTTTTGTAAAAGGTGTTCCCTCTTGTTAGGGTGATGGCCTTGCAATAAAAAGTATTTTGATGTTAAAATAAAAGTGTACCCTCTCGTTAGGTAACTAGC
7D-2002290-T10-20_384	CAGAGGCGGCGGCCGGCGGGGCAGGGCACACACTCACACGGGACGGAACGCGGAACGGGATAGCGCCTGCCTCTGTCAGCTTAATGGGCCTCCCTGTGCGTGCCTTGGTGGGTTGTT
5D-1449300-T10-20_448	AATAAAAAGTATTTTGATGTTAAAATAAAAGTGTACCCTCTCGTTAGGTAACTAGCAAACATGCATGTGTGTTGCAATTGGAGAAAAACACACGCTCCTAAAACCCAATATGCATGGTGA
5D-1449300-T10-20_512	ATGTGTGTTGCAATTGGAGAAAAACACACGCTCCTAAAACCCAATATGCATGGTGATGGGAATGTACATGAAGGAGAATTGTGAGTTCGGCCAACCAATCTTTGCAAACGGCTAACAAAC
7D-2002290-T10-20_512	GGACCCAGGccgattttacaataattactcgccttgagatcttcaaaactagatccatgttaatgtGCCTAAAAAAAGATCTATATTAATGAATTTGGACAaaaaaataaaaaactgaat
5D-1449300-T10-20_576	TACATGAAGGAGAATTGTGAGTTCGGCCAACCAATCTTTGCAAACGGCTAACAAACTTATGCATTGTATAATTTGCGGGTTAGGTTAAAGACAATCACCTACAAAGAAAAGGGGTTAAAG
7D-2002290-T10-20_576	gtGCCTAAAAAAAGATCTATATTAATGAATTTGGACAaaaaaataaaaaactgaataaaaatagcaaaaaacataaatgaaaaagcaaaataaaaatgaaaaCCATCGAAATCCGAAAAA
5D-1449300-T10-20_640	TGTATAATTTGCGGGTTAGGTTAAAGACAATCACCTACAAAGAAAAGGGGTTAAAGACAATCATCTCTAACAGAAATGAAAAAAATAAACTCAACATAGGAATAAAAAGACATACAAAAG
7D-2002290-T10-20_640	caaaaaacataaatgaaaaagcaaaataaaaatgaaaaCCATCGAAATCCGAAAAAATAGAAACCTGGAAGTGCAGTTGTGTTTTTTCAAAAAATAAATAAACTAGAAGCACAACTATGC
5D-1449300-T10-20_704	CTCTAACAGAAATGAAAAAAATAAACTCAACATAGGAATAAAAAGACATACAAAAGATAACAAAATTGAAATTACCGAAGCATAATTTCTTCTGCCAATGCAAGGTATTATTTCTCGCCT
7D-2002290-T10-20_704	CTGGAAGTGCAGTTGTGTTTTTTCAAAAAATAAATAAACTAGAAGCACAACTATGCTTTTCTCTTTTTAGGAAGAACATTTTTGCTTTTTCGTTGAAGCATAGATACGCTTCTCCCTTTT
5D-1449300-T10-20_768	ATTGAAATTACCGAAGCATAATTTCTTCTGCCAATGCAAGGTATTATTTCTCGCCTTCATTTCATTATCAGAAGAATGAATATTTCAGATTTAATACATCTTTCAATAGGAATAGTGTGT
7D-2002290-T10-20_768	TTTTAGGAAGAACATTTTTGCTTTTTCGTTGAAGCATAGATACGCTTCTCCCTTTTCTGGAGAAGCACATGACTGTGCTTTTTTCTTTCAGATTGTGCTTCACTTTTTGGGAAGCAGAAC
5D-1449300-T10-20_832	TTATCAGAAGAATGAATATTTCAGATTTAATACATCTTTCAATAGGAATAGTGTGTACTATAATCAAGCATGTGCTAGGTTGTCACTGACAAAGCATACTGCGGTACTATAATCCAGTAT
7D-2002290-T10-20_832	GCACATGACTGTGCTTTTTTCTTTCAGATTGTGCTTCACTTTTTGGGAAGCAGAACATCGTGAAAAAGCATTGCTTCTAAGAACCCTTTTTTTTTTGACAAAACCTAGGGAAAATCAAAC
5D-1449300-T10-20_896	CAAGCATGTGCTAGGTTGTCACTGACAAAGCATACTGCGGTACTATAATCCAGTATAGACATGGTTGACTGCCATCCTTAGGTTTATAGTGTGCGTATATCTCAACAAACAAACCAAATA
7D-2002290-T10-20_896	AAAGCATTGCTTCTAAGAACCCTTTTTTTTTTGACAAAACCTAGGGAAAATCAAACAAGAACCTGAAAGCCAAAAAACAAAAATCGTTATAACACGTGCAGAAAAATAATCCAAAATCAC
5D-1449300-T10-20_960	TTGACTGCCATCCTTAGGTTTATAGTGTGCGTATATCTCAACAAACAAACCAAATATAAGTGCAAAAGATATAGATTATCAACCATAACAGAAGGTTAGTATACAATGCAGATTAAGCAT
7D-2002290-T10-20_960	GAAAGCCAAAAAACAAAAATCGTTATAACACGTGCAGAAAAATAATCCAAAATCACAAGGGTGCGCCAGCTCATGACACCTGGTGGCTACTGGGCACACTAATTGAGGCGCTCCCAAGG
5D-1449300-T10-20_1024	AAAGATATAGATTATCAACCATAACAGAAGGTTAGTATACAATGCAGATTAAGCATTATACACATAATAAAAATGTCGTATATGCAACATTTCTATTGAGTTACTAACATCATACAATAA
7D-2002290-T10-20_1024	GCCAGCTCATGACACCTGGTGGCTACTGGGCACACTAATTGAGGCGCTCCCAAGGCATGCAATTGACCTCCAAGGGAGCCCTCCAAATGACACTCCTTAATTAATTGCTCCCTTGTTTTG
5D-1449300-T10-20_1088	TAATAAAAATGTCGTATATGCAACATTTCTATTGAGTTACTAACATCATACAATAAAAATGTTGTATACATCGGCTGTCCATGCATGTATGCTTCTGTTGTGTGTGTGCTAACCACGAAA
7D-2002290-T10-20_1088	GACCTCCAAGGGAGCCCTCCAAATGACACTCCTTAATTAATTGCTCCCTTGTTTTGAAAGAGGAAATGGCCGATGCTACGATGGAATTGTTCGCAAATAAACCTGGCTTGGGTCACACAC
5D-1449300-T10-20_1152	TATACATCGGCTGTCCATGCATGTATGCTTCTGTTGTGTGTGTGCTAACCACGAAATCTCGTCGTATAGATTTACTCTTTCCTCTTCTGTTGTGATATATGCAAGGCTCTCCATCACAAT
7D-2002290-T10-20_1152	AATGGCCGATGCTACGATGGAATTGTTCGCAAATAAACCTGGCTTGGGTCACACACCCATTCTAAACACCCTAAAACCTATGAAACCCGTCAGGATCCAATCCACGCCTTCAACCCAAAC
7D-2002290-T10-20_1216	AACACCCTAAAACCTATGAAACCCGTCAGGATCCAATCCACGCCTTCAACCCAAACATGGCTATGAGGGTCGGCACTTTCGGACACACAATATGTCGGCCTTTTTATAAAATGGTCGTGT
7D-2002290-T10-20_1280	GAGGGTCGGCACTTTCGGACACACAATATGTCGGCCTTTTTATAAAATGGTCGTGTCGTGCCAGTCGAAGGCCTCGGCTCAAGGATCGGCCCGACTTGTGACATAAAAAGGCCAACATGC
7D-2002290-T10-20_1344	TCGAAGGCCTCGGCTCAAGGATCGGCCCGACTTGTGACATAAAAAGGCCAACATGGCCTGGCACACATGTAGGTCAGCCTTCATTCATTTTCATCCTTTCGAGTCTTCTACATTTTAAGA
7D-2002290-T10-20_1408	ACATGTAGGTCAGCCTTCATTCATTTTCATCCTTTCGAGTCTTCTACATTTTAAGAATTAAGAAAATCTGAAAATTTATGGAAAAATCTAAATAAATAAGAAATACACAAATATAAAAGT
5D-1449300-T10-20_1472	TTTTATTTCGTTGATAGGTTTCACGACGTCTTTTTAGTTTTTCCTATTTTTCTTCATTGGTTATCCGTGCATGCCACTCTCAATCCTTTTCCTTTCTGTTATTTGGTATCAGTGACTGTA
7D-2002290-T10-20_1472	AATCTGAAAATTTATGGAAAAATCTAAATAAATAAGAAATACACAAATATAAAAGTTAAGAAAAAATACAAAAAATGTAAAAGAGACGAAGTGTCATATAGATAAATATAGCTGCCTCGT
5D-1449300-T10-20_1536	CCGTGCATGCCACTCTCAATCCTTTTCCTTTCTGTTATTTGGTATCAGTGACTGTAGATTGCGATCCAAATTTCTTGCTTGCATGAGAGTTTTTTATATGTATGCATCCATTGTTAATAA
7D-2002290-T10-20_1536	AATACAAAAAATGTAAAAGAGACGAAGTGTCATATAGATAAATATAGCTGCCTCGTAAAAACCTTTTAGTTAAACTCCCCTTGAAGACGAACCCAATAAAAAAAAGAGTACAAAGCTACT
5D-1449300-T10-20_1580	TCAGTGACTGTAGATTGCGATCCAAATTTCTTGCTTGCATGAGAGTTTTTTATATGTATGCATCCATTGTTAATAAAAGAAACTAAATAGTTTTTATAGAGTGTCCGGTTTTTCCTAAAG
7D-2002290-T10-20_1580	TAGCTGCCTCGTAAAAACCTTTTAGTTAAACTCCCCTTGAAGACGAACCCAATAAAAAAAAGAGTACAAAGCTACTTGCTCGAAAATGAGAATTATAAAAGTAAAAAAACACAAAAGAAA
6A-1532030-T10-21_128	GCTCGCCTGCAAACGCGACTCGGGACCGAGGAAATATCCTCTGCAACGAGCCAATTCGTGCACCCAATACACCGAGCGATTCTGGGCGATGGGGAATAGATGGACTGGATGTTATCACA
6A-1532030-T10-21_256	CGGGTATATTTTACACAATGGACCTTGTCAAGTCATTGAATTGCGCAAAAGTCCTTCTGAAATTCCACTCTGCTTTGTACCAGTCTCCGTGAGATCCCCTCTCTGTACCAGGCGATCTAT
6A-1532030-T10-21_320	CCACTCTGCTTTGTACCAGTCTCCGTGAGATCCCCTCTCTGTACCAGGCGATCTATCACGGACGAGCTCTCTTCAGCAGAACTCCCATCCCGATGGCTGCATTCCCTTTTCCCTCTATCC
6A-1532030-T10-21_576	CTTTCTTTATTCAGATCGTGAGTTACATAAAAAGAGAGCTAAGAATATACTCCTGATTACTGATTACGATGGATATCATTGAACTCATATATACATAAAGTTAACTATGATCTTGTCAAA
6A-1532030-T10-21_640	TACGATGGATATCATTGAACTCATATATACATAAAGTTAACTATGATCTTGTCAAATTTTCTGGAGTTTCCTCATTTCACATTCTAATTTGATTAAACTTCTACGAGACAATTCTTTTTT
6A-1532030-T10-21_704	AGTTTCCTCATTTCACATTCTAATTTGATTAAACTTCTACGAGACAATTCTTTTTTGAGGGATCTACAACACAATTAAAGCCTGAATGGAATACTAAAACACATCTTCAAGCCTAAGCGC
6A-1532030-T10-21_832	TGGTGATTCTCAAAGCTGAGAAATCATTCAGCTAGTACCCAGGTGGATTAGGCCACAAAATAATTGTTAAGAGCTACATCACCatatatatatatatataGACACAGTCTATTTAATGTC
6A-1532030-T10-21_896	TGTTAAGAGCTACATCACCatatatatatatatataGACACAGTCTATTTAATGTCCTACCAACTGGTGGAAGCATGATTTTGCATTGGAACAGAGAACAAAATCTACACATCATTGGAG
6A-1532030-T10-21_960	TGGTGGAAGCATGATTTTGCATTGGAACAGAGAACAAAATCTACACATCATTGGAGAGAACCAGTCTTTAACTTCAATCAGGCAGAGACCAAAAACTAATACATATAAATGGAGAGAACC
6A-1532030-T10-21_1024	TCTTTAACTTCAATCAGGCAGAGACCAAAAACTAATACATATAAATGGAGAGAACCAGTCTTTAACTTCAATCAGGCAGAGACCAAAAACTAATACATATAAATGGAGAGAACCAGTCTC
6A-1532030-T10-21_1152	TCCTCAGTTTTTCCTGCAGCAGAAAATAATTTCTGGAGCTTCACAAAACTAACCTGGTTCAACAGTCTTGCGTTTTTCTTTTTGCAGGTAAGTCTCGCAGGTTTAACACCATAAAATTGT
6A-1532030-T10-21_1216	GTCTTGCGTTTTTCTTTTTGCAGGTAAGTCTCGCAGGTTTAACACCATAAAATTGTTACACAAATCCACTTGAACCATGATAAAACATAAAATCGTTCCACACATTTGGCATCGCGAACT
6A-1532030-T10-21_1280	TCCACTTGAACCATGATAAAACATAAAATCGTTCCACACATTTGGCATCGCGAACTAACTTCTTAAACACATGAGTGACCCTTTTTACTCGAAGCATCCAAGTGCATAAATGACAACCGA
6A-1532030-T10-21_1344	AAACACATGAGTGACCCTTTTTACTCGAAGCATCCAAGTGCATAAATGACAACCGAAGTAGAAGTACATATAGACTTGTACAGATTTGTTTCCTTTGTGCACCAGGTTTAGACAAAAGGA
6A-1532030-T10-21_1408	TACATATAGACTTGTACAGATTTGTTTCCTTTGTGCACCAGGTTTAGACAAAAGGAATCGAAAAAATCAACTTAGCAGCAGCGACTAGTAGATATACTTCACGAGGAAAGCATTTCGGGC
6A-1532030-T10-21_1472	AATCAACTTAGCAGCAGCGACTAGTAGATATACTTCACGAGGAAAGCATTTCGGGCGATAGAGTACAAATCAATCCTCAGGCCAACTAGCATGAACAGAAAGTCATCTTGATGACTTCAA
6A-1532030-T10-21_1536	ACAAATCAATCCTCAGGCCAACTAGCATGAACAGAAAGTCATCTTGATGACTTCAAGATAGATGGAATACATAATGCATGTTGAAATGACCACAGAGCCTCCTGCAAGAATAAACCTAGG
6A-1532030-T10-21_1580	TGATGACTTCAAGATAGATGGAATACATAATGCATGTTGAAATGACCACAGAGCCTCCTGCAAGAATAAACCTAGGGGTTAATATGCCAAGTTAGCTACAATTGACGGTAATATATATAT
6B-1609120-T10-21_384	GATAATAAAATATAAACATGTTTAACTTGAATACTATATCCGACGCTTAGGTCCACAGTTTGGAAATAATGATTAAAGAAACATCAAAATAGAGCAGGTGCTGCTGGTTGAACATGAGTA
6B-1609120-T10-21_448	AATAATGATTAAAGAAACATCAAAATAGAGCAGGTGCTGCTGGTTGAACATGAGTAATTTATAGAATATACTCACATTGTAAAGTAAAGTTAGAAGAATATCCTCTCTTGGAAGACACTT
6B-1609120-T10-21_512	AATATACTCACATTGTAAAGTAAAGTTAGAAGAATATCCTCTCTTGGAAGACACTTACGTGCCAAATCATAACTCATGGTATTGAACATGCCCTTCATCTACAAATTTCAATGTAGATGG
6B-1609120-T10-21_576	AATCATAACTCATGGTATTGAACATGCCCTTCATCTACAAATTTCAATGTAGATGGAAAAAGAAAAATAATGATAGTTCACATGAAAAAGATAAGCAGACTTGCAAATCATTTAATTCTG
6B-1609120-T10-21_640	AAATAATGATAGTTCACATGAAAAAGATAAGCAGACTTGCAAATCATTTAATTCTGGAGCATGAACCAAGCATTGCGGTCTCATTTGCTTACTGGATTTGATAAAACTTTCCAAATCGCA
6B-1609120-T10-21_704	ACCAAGCATTGCGGTCTCATTTGCTTACTGGATTTGATAAAACTTTCCAAATCGCATAAAAAATATATATTTTAATTGACCTGACAGGGAAGAGATCAAGAATCGCAACACTTCTAACTGA

6B-1609120-T10-21_768	TATATTTTAATTGACCTGACAGGGAAGAGATCAAGAATCGCAACACTTCTAACTGATGGAACCACCGGCTCTCACAGTCTTGTCTCCGTAGCCTCCACAAAATTAAAACCCAAAGAACAA
6B-1609120-T10-21_832	CCGGCTCTCACAGTCTTGTCTCCGTAGCCTCCACAAAATTAAAACCCAAAGAACAATCTAATGTCCTAACAAAACGGAGGATTTTCCTGTAGGTACACAGGGCTGCCGGAAGAACCATCT
6B-1609120-T10-21_896	CCTAACAAAACGGAGGATTTTCCTGTAGGTACACAGGGCTGCCGGAAGAACCATCTGGGCGGAAGTAGTGCTAGTGGCCGGTGATTGAATTCATACCAGACGAACCCAAGGCGGAGAG
6B-1609120-T10-21_1024	GGGCTGGAATCAACTCTGCCAGGACACGCGAAAAGCGAGATGAACAGACATAATAGTGCCCGGTCTGCTGCTGGATTGAAGTAGTCGTGGTCGCGGCCGGCAGCCCGGGTGCTTTGCT
6B-1609120-T10-21_1344	CACCGAGCAGCGAGCACAACGAGCGGACGTTCCACCTCCCTTGTGGAGCGAGAACATCTTATCTTAATGGGATGGGCTGGGATAACCGGCCCAGTTTACCAGAGGGCTTGGATTCGGC
6B-1609120-T10-21_1580	GCTGAGGAGACTCGCTTTAGGTGCGGTTATACTGTCCTTAATTGGAAAGGGGTTATGTCCGGTTAAAAAAAAAGTTAGGGAATCTGCTGGGAGGTCTCCAGTCGCGTTTGAAGGCGAG
6D-1712870-T10-21_540	ATTTAGCAATTTGGGGTTCGAATTGGCGCTCCTATTTCCCGCCTTAAGCAGCGAAATGCAGCTAAAACGCGCGACGGGCCAGACCAAATAGCCATCGCTCGCCTTCAAACGCGACTCGAG
6D-1712870-T10-21_602	TAAAACGCGCGACGGGCCAGACCAAATAGCCATCGCTCGCCTTCAAACGCGACTCGAGACCTCCTCCCCCAGCGGACTCCCTAATATTTTTTTAACCGGGCATAACCCCTTTCCATTAC
6D-1712870-T10-21_664	CCTCCCCCAGCGGACTCCCTAATATTTTTTTAACCGGGCATAACCCCTTTCCATTACTTTCATAATCAAAATATAACGGTCTGACAAACAGAAACGAAATTAGAAAGAGAAAAACGAGT
6D-1712870-T10-21_850	CCAATGACACCCTAGGAACTGACACCAACTACTTAAGGACTCTAGGAAACGGACCATACCAAAGTATTGCAACAGCAGCCACAGGCCAAAACACATCTCACGCTGGAGCAGCAGACCAA
6D-1712870-T10-21_912	AGTATTGCAACAGCAGCCACAGGCCAAAACACATCTCACGCTGGAGCAGCAGACCAACACAGTTTTGCGGAGACGCAAAAAATCGATATCCCACACAGGGGGTAGATATGTAGCACTAA
6D-1712870-T10-21_974	TTTTGCGGAGACGCAAAAAATCGATATCCCACACAGGGGGTAGATATGTAGCACTAAAGGGAGGATAGACTTGCTTCATCGCCCAGGGTCAGCCTGTGTTCAACACTTCATCGCGTTCCC
6D-1712870-T10-21_1160	CGCCTCACGATCAGCTCCCACCATTAGACCTGCCTAGTAATTTAGATATCCACAAGCAGAGCAAACCACGTCAAAAGGGATCTCATTTTTTTAAACTCAAAGGTTACCCGGTTACGGCAA
6D-1712870-T10-21_1222	AAACCACGTCAAAAGGGATCTCATTTTTTTAAACTCAAAGGTTACCCGGTTACGGCAATTTCACATAGCCCAGCACATCGGTGCTAAACCAAAAGTGTAGAATCTCTTCCCATCAGGAAG
6D-1712870-T10-21_1408	ACCCACAGTCCTCCATATAACTCTAGCTACTGGACATGTAAAGAAGAGATGCTGGGAGGTTTCCTTTTTGTCACAAAAAAAGCAAGTAGGGTTACCCAACCATTTCCTCCTACTCATAAC
6D-1712870-T10-21_1532	CTAGTCAAGACTGCATCCTGAAAAAGCTGTCAAAGGAAGATTTGTATTTTCAGGGGTATCTTGGATCTCCAAATCCATTTATAGTTACACACCATCACATTTCTCTCCAGATATTCATAT
6D-1712870-T10-21_1580	TTCAGGGGTATCTTGGATCTCCAAATCCATTTATAGTTACACACCATCACATTTCTCTCCAGATATTCATATATCGATTTGGTCGTAAATTTATTTTTCTTCCCCAAGGTCCAAGCCACC
3A-0655460-T10-22_0	TGTTTCTAATTTGGACCTTTTGGAATAAAAGAAATGTTAGGGTCTTTCGCTACAAGTCCGCACCGATGGCAATCCTCCTTGAGGGTATCAAGAAAGATACAAACGCTTTGGTTCATCAAA
3A-0655460-T10-22_65	ATGGCAATCCTCCTTGAGGGTATCAAGAAAGATACAAACGCTTTGGTTCATCAAAGATACAATCCTCCTTGAGGAAATTTATCCTAGCGCCCAGGCCTAGCGCCCGGCGTTGTAGATGCC
3A-0655460-T10-22_260	TATCAAGAAAGATACAAACGCTTTGGTTCATCGCGGGTGCGGACATTTGGGACTAATAATGCATGGGAGAGTAGTGATCTTTATCTTTTGTTGTTGGATACTTGAGTGTTGTATTTGTAA
3A-0655460-T10-22_325	GGAGAGTAGTGATCTTTATCTTTTGTTGTTGGATACTTGAGTGTTGTATTTGTAAACGAAACACTCTATTTCTCTGAATTAATGGACGAGGGAAAGCATTTGCATCCATTTTTAAAGAGT
3A-0655460-T10-22_390	CTATTTCTCTGAATTAATGGACGAGGGAAAGCATTTGCATCCATTTTTAAAGAGTTTCATGAAAGAATGACACTAGTGGTATTTCTTAATAAATGAAACACAAGCCGGCCTTGTTATTCC
3A-0655460-T10-22_520	GTTTTTGAATACACTTTTCAATTTTGTCATTTAGCTTAGAATACCAAGGATGTCATTTTTTCGTGAAACTTCATATGCCAGTCAACCATGTATGTTACACAAAAAATAACTAATTTTCCT
3A-0655460-T10-22_585	AAACTTCATATGCCAGTCAACCATGTATGTTACACAAAAAATAACTAATTTTCCTAGTATTTGTTTCTTGAATTAACTATTCATAATGGGTGCGTGGGACCATGTTTCACATTTTCGTGT
3A-0655460-T10-22_650	TCTTGAATTAACTATTCATAATGGGTGCGTGGGACCATGTTTCACATTTTCGTGTTCACCTTTTTAGACCCTAAAATTTGTGAAAAATCATGTATGTACACATATATGTCTAGTATATTG
3A-0655460-T10-22_715	AGACCCTAAAATTTGTGAAAAATCATGTATGTACACATATATGTCTAGTATATTGCATACAAATTTCCATCTACATATTTTTAATACATGTTTCATATAAAAATACTTAGATTAAAGTGG
3A-0655460-T10-22_780	TCCATCTACATATTTTTAATACATGTTTCATATAAAAATACTTAGATTAAAGTGGGATTACTTAGTTTTGGCCACAGATATGTCGTTTTAATGTAGCCTACAAGTCATCATATATTCCCA
3A-0655460-T10-22_844	GTTTTGGCCACAGATATGTCGTTTTAATGTAGCCTACAAGTCATCATATATTCCCACCACAACGATCatcatatatcatataacactcctattcatatatcgattataataACTATAAGA
3A-0655460-T10-22_1181	ATCTAACAACATTTTACAAATGACCCCTAAAAAAACATTTTCCAGATGGATAGAAAACATACCTATAGTTTTTGGACCCTTTTATCTTGCTGACATTTGCGGCATTTTAATGGCAGTTTT
3A-0655460-T10-22_1238	CATACCTATAGTTTTTGGACCCTTTTATCTTGCTGACATTTGCGGCATTTTAATGGCAGTTTTAGCTGGTGACCAACATATGACATTTTCTGAAAAGAAGGCTAAAAATTGTCATCCTTC
3A-0655460-T10-22_1295	AGTTTTAGCTGGTGACCAACATATGACATTTTCTGAAAAGAAGGCTAAAAATTGTCATCCTTCCAGAAAATGCAATGTTGCTCTGAAGAAACTGCCACCCCCGACATAACTATTACTATG
3A-0655460-T10-22_1409	ACTATGAGGAAAAACATTCAAAATGCCTTGCTCTCCCAGCAAATGTCATCCAGATAAGATTTCCGTAGTTTTTTTAACTTGTAGAAATCAGTTTTGTTTTTGAAATTTCGGGATCCAAAA
3A-0655460-T10-22_1466	GATTTCCGTAGTTTTTTTAACTTGTAGAAATCAGTTTTGTTTTTGAAATTTCGGGATCCAAAATGATGCTCTACCGGCGAGGCACGCGCCCTCCGCCGGTCTCCCGTACGATCCCCCACA
3B-0791120-T10-22_0	TCCAGTACTACCACTCAAACAGTATGTAACAAATTATATAATGATACAATGCAGCCAACCAAATAAACAAAGTGCATAGCATGGCCAAGAGCCTACACATCCTAGCTATTGACAAGAATG
3B-0791120-T10-22_64	AAACAAAGTGCATAGCATGGCCAAGAGCCTACACATCCTAGCTATTGACAAGAATGTGGGGAAAAAATGTTTGGTGCCCGGACTATGGGTAGCTCTTTTGGTCAGTAAAGTTTAAACATA
3B-0791120-T10-22_128	AAATGTTTGGTGCCCGGACTATGGGTAGCTCTTTTGGTCAGTAAAGTTTAAACATATTTTTAGCGAAAAAACAAACATGAATATGCTTCCACACGCACCCATGTGAATAGTGAACTCGAA
3B-0791120-T10-22_192	GAAAAAACAAACATGAATATGCTTCCACACGCACCCATGTGAATAGTGAACTCGAAAAAACATACTAGAAAGACCAAACAAATAAAAAATGTTTGGAGTATCAAACTCGGTCAACAATTC
3B-0791120-T10-22_256	CTAGAAAGACCAAACAAATAAAAAATGTTTGGAGTATCAAACTCGGTCAACAATTCTACTCATGTGTGAAGTTACATACTAGGGCATCGGATATGACATCACTTGGAAGTACGACTAGTG
3B-0791120-T10-22_320	TGTGAAGTTACATACTAGGGCATCGGATATGACATCACTTGGAAGTACGACTAGTGGCTGCTACTCGGCAGCCTCCACCGACAAGAGGGCACCAACGACATCACTTGGAAGCACACAACT
3B-0791120-T10-22_384	TCGGCAGCCTCCACCGACAAGAGGGCACCAACGACATCACTTGGAAGCACACAACTAGTGGCCACTACTAGGCGGCCTCCATCGACAATGCGCAATTTTTGGGTGCAACCCTCACCTCC
3B-0791120-T10-22_448	CTACTAGGCGGCCTCCATCGACAATGCGCAATTTTTGGGTGCAACCCTCACCTCCATGGAAAAGGATGGTTTGGAAAGCTTGGGCACTATGAGGTTAATTTTTTTGTTTGGTTGGCCAAA
3B-0791120-T10-22_512	GATGGTTTGGAAAGCTTGGGCACTATGAGGTTAATTTTTTTGTTTGGTTGGCCAAAATGTAAGACCCAAAATAACTAGATTAAAATGAGAAGGCCAATTTGGCTCGCGGGCTCAGCTGCA
3B-0791120-T10-22_960	TTTATGAGAGGGAGCATCTGAGCCTGGCCTCAGATGCGGATTTTCGGATTGAAATAGGCTATTTTTGTGTTGCCACATATCCGTCTTTTATGTAGCCTACAAGTCACCATATAGTTATTC
3B-0791120-T10-22_1024	TTGTGTTGCCACATATCCGTCTTTTATGTAGCCTACAAGTCACCATATAGTTATTCCCACAAAAAAGATCATCATATATTACCCCCGCCGATTCATGAGAAGTGTCATACATTATTTAAC
3B-0791120-T10-22_1088	AAGATCATCATATATTACCCCCGCCGATTCATGAGAAGTGTCATACATTATTTAACAACAATTTAGAAAACATCTATAGGCACCCAATTTTTTTAATTTGTACACATACATCAGTTCTGT
3B-0791120-T10-22_1152	AGAAAACATCTATAGGCACCCAATTTTTTTAATTTGTACACATACATCAGTTCTGTTTTTGAAATTTCGAGGATACAAAATGCTCTACCGGTGAGTATACCTGACCATCCTTCAGGCCGG
3B-0791120-T10-22_1472	CATGGGCAAGGTCGGATCGGGCTTTTTCGGGCCGGGCTGCCCATGGCCAGCTATACGCGGCGAGGCAGGCATGGTAGCGCGCCCTGCGCCAGTCCCCTGTACGACCCCCCACATCGGCT
3D-0883140-T10-22_0	TGCAGAAATGGCATTTGGTGCGCGGGCTACTACTAGCTCCATTTGTCTACAAAAAATTAAAACATATTTTTAGCGAAAATACAAAAATAAATATGGTTCCATGTGATCCATATGAATAGT
3D-0883140-T10-22_64	TATTTTTAGCGAAAATACAAAAATAAATATGGTTCCATGTGATCCATATGAATAGTAAATCTGAAAAACATAATAGAAAAAACAATCAAAGTTGGGGTATCAAACTTGGTCGACCATTCT
3D-0883140-T10-22_128	AAAACATAATAGAAAAAACAATCAAAGTTGGGGTATCAAACTTGGTCGACCATTCTATTCATGTGTAGAGTTATATATTCCGAGATGTCCAACTTCAAGAGGGCACCGAGTACGACATCA
3D-0883140-T10-22_192	GTAGAGTTATATATTCCGAGATGTCCAACTTCAAGAGGGCACCGAGTACGACATCACTTGGAAGCACGTGATCAGTGGCCACTACTCGCCGGCCTCCTACGACAAAATGACACCGACAAT
3D-0883140-T10-22_256	CACGTGATCAGTGGCCACTACTCGCCGGCCTCCTACGACAAAATGACACCGACAATGACATCACTTGGAAGCACACGATGAGCGGCCACTACTCGCCGGTCTCAGCCGATAAGGCGTAAT
3D-0883140-T10-22_320	TTGGAAGCACACGATGAGCGGCCACTACTCGCCGGTCTCAGCCGATAAGGCGTAATTCTTGGGTGTGCAACCCGCACCTCTATGGAAACGGCGGTTTGGAAAGCTCGGGCAGTCGGGA
3D-0883140-T10-22_384	GTGCAACCCGCACCTCTATGGAAACGGCGGTTTGGAAAGCTCGGGCAGTCGGGAAGGTTAATTTGCCATCCAAAATAGGATTTAAACGGCGGATAGGTTGGCTAAGCACATTTGACCAA
3D-0883140-T10-22_448	GCCATCCAAAATAGGATTTAAACGGCGGATAGGTTGGCTAAGCACATTTGACCAAATTACAACATGCCCTTTGTGCAAAAAAGAGTAATAAATCACTTCACACTTCACAAATGTCGGCGC
3D-0883140-T10-22_512	TGCCCTTTGTGCAAAAAAGAGTAATAAATCACTTCACACTTCACAAATGTCGGCGCACCTTGAGGCTATAGGGGTTGGTGAGGGAGTGGTTCCAACTTGGAGCGCTCTACCGAAGAGTA
3D-0883140-T10-22_640	CATGACTGAAAGCAACATCCTGAATAGGAGCGCCATGGCCTCCCTCACCATGTTTTAAGTTGGACCTTTTAGAATAAAAGAAATGTTAGGGTCTTTCGCCATAAGTTTGCACCGCCAGCA
3D-0883140-T10-22_704	CCTTTTAGAATAAAAGAAATGTTAGGGTCTTTCGCCATAAGTTTGCACCGCCAGCAATCCTCCTTGAGGGTATCAAGAAAGACACAAAGCTTTGGTTCATCGCGGGTGCGGACATTTGGG
3D-0883140-T10-22_768	TGAGGGTATCAAGAAAGACACAAAGCTTTGGTTCATCGCGGGTGCGGACATTTGGGACTAATAATGTCTGGGAGAGTAGTGATCTTTTTCTTTCACTGTTGGGCACTTGAGCGTCGTATT
3D-0883140-T10-22_832	TGTCTGGGAGAGTAGTGATCTTTTTCTTTCACTGTTGGGCACTTGAGCGTCGTATTTGTAAACGAAACACTATATCTCTGATTAATGGATGAGGCAAAGCATTTGCATTCATTTTTAAAA
3D-0883140-T10-22_896	AAACACTATATCTCTGATTAATGGATGAGGCAAAGCATTTGCATTCATTTTTAAAAAGTTTCATGAAAGAATGACACCAGAGGTATTCTTAATAAAGAAAACACAATCCAACCTTGTTAT
3D-0883140-T10-22_960	GAAAGAATGACACCAGAGGTATTCTTAATAAAGAAAACACAATCCAACCTTGTTATTCCGTCAAAGAGTGCTTTTGAATACACTTTTCGATTCTGTCATTTTGCTCAGAATACCAAGGAT
3D-0883140-T10-22_1024	AGAGTGCTTTTGAATACACTTTTCGATTCTGTCATTTTGCTCAGAATACCAAGGATGTCATTTTTTCTGTGAACACTTCATTTGCGAGTATGCCATTTAACCATGTATGTCACATAAAAA
3D-0883140-T10-22_1088	TTCTGTGAACACTTCATTTGCGAGTATGCCATTTAACCATGTATGTCACATAAAAAATAACTAGTTTTCCTATCTTATTTTTCCTAGTATTTGTTTTTGAATTTACTATTCATAATGGGC
3D-0883140-T10-22_1152	TTTTCCTATCTTATTTTTCCTAGTATTTGTTTTTGAATTTACTATTCATAATGGGCGCGTGGAACCATGTTTCACATTTCCGCGTCCATCATTTTAGACCCTAAAATTTGTGAAAAATTA
3D-0883140-T10-22_1216	CCATGTTTCACATTTCCGCGTCCATCATTTTAGACCCTAAAATTTGTGAAAAATTACGTATGTACTCATATATGTCTAGTATATTGCATACAAATTTCCATCCATATATGTCTAGTATTA
3D-0883140-T10-22_1280	CTCATATATGTCTAGTATATTGCATACAAATTTCCATCCATATATGTCTAGTATTACTCAAAACTGTCATCCTCTTAGAAAATGCAATGTTGGTCTGAAGAAACTGCCATCCCTGACACA
3D-0883140-T10-22_1344	TGTCATCCTCTTAGAAAATGCAATGTTGGTCTGAAGAAACTGCCATCCCTGACACAACTAAAGCTGTCAAAAAAAAACATTCAAAATGCCATGCTCTCCCAGCAAATGTCATCCAGATAA
3D-0883140-T10-22_1408	TGTCAAAAAAAAACATTCAAAATGCCATGCTCTCCCAGCAAATGTCATCCAGATAAGATTTCCGTAGTTTTTTTAACTTGTAAAAATCAGTTTTGTTTTTGAAATTTCGGGGATCCAAA
1A-0036950-T10-23_378	GCCTCTTGTAATGCCGTGTGCCTTTCAATTATGCTCTCAATGAACATGAAACTATCTGCTGTTAAATTCAATTTTTTCATTAGTTTGTTGTCAAATTCAGTTTGAACATTAAGTTAACTT
1A-0036950-T10-23_504	AATTGTTCCTGTCAATATGACTATATGAGTATACTTTCATGGTAAAAAAATGAATATATACTTTCATGTCAAAAAAGAATACAATTTCTTCAACAACAAAATACAAGTTGTGCAAAAAAT
1A-0036950-T10-23_567	TCATGTCAAAAAAGAATACAATTTCTTCAACAACAAAATACAAGTTGTGCAAAAAATACACTATGATGAGACCCTACCGCCGGGGAGTGTGGCGGTAGGTTTTCCCAGCCTACCGGCGGG
1A-0036950-T10-23_756	ATAGGGTTGCACACCCTATCACCAGGGTCACCGGCGGTAGAACTGCATTCCCTACCGTCGTTGGTTCTGGCGGTAGCATGTCTGCCAGGTCAGCGCCTAACCGTTTGTGTGACAGCAACT
1A-0036950-T10-23_819	GTTCTGGCGGTAGCATGTCTGCCAGGTCAGCGCCTAACCGTTTGTGTGACAGCAACTACTGTTTCCCTACTGCCGAATGTTCTAGCAGTAGGCTATGCAAGCCTACCGCCGTAGAGAACG
1A-0036950-T10-23_1000	GTTAAAAGGGTCAAAACACGAAATTTTGCCTCTCTTTGCTTCTCTTCCCCGTAGGTGGCAGTCCCTGGACCCACCTGTCTCCCACGGACATGGACTCTCACCCCTCTCTGAAACCGCCCC
3A-0681930-T10-24_0	GCCGGCCGGCCGGCTGAGCCTGAACTCCCTCCGACGGCGATGCGAACCAACGTTGAGCTAGCTAGCTTCTCCTCCTCCGCGGACCGCGCGCGGTGTCTGCACGAGAGTGGCTAAAAGA
3A-0681930-T10-24_64	GCTTCTCCTCCTCCGCGGACCGCGCGCGGTGTCTGCACGAGAGTGGCTAAAAGAGATTGGAGAGATCAACGGGGGACGGACGACGACGACGACAGCATCTGGCTAATAATGGATGGT
3A-0681930-T10-24_384	AAAGGAGGCGATGAGGATGAGCATGGGCGCGTGTGCATCATCGCGCTATTGTTGGAGAGGTGGCAATGGCATGCAGCATGGGGCATGGCACTATTGGATACAGGGACTCTGGCTTCCG
3A-0681930-T10-24_448	AATGGCATGCAGCATGGGGCATGGCACTATTGGATACAGGGACTCTGGCTTCCGCTGAATTATGCGCAGGTCTCAACAGGCACTCAAACTACAACGTGATTTCTTTTCAGAAGCCTTTCG
3A-0681930-T10-24_512	CGCAGGTCTCAACAGGCACTCAAACTACAACGTGATTTCTTTTCAGAAGCCTTTCGTGTTTTTTTAGGGACAGAAGCCTTTCGTGTTAGAGATATATTTAGCTTACATATATTTGGTAGT
3A-0681930-T10-24_768	TCTAACATGGTACCAGCCTAACCGATTCAACCCTAGCCATGACCCGCTGCTTCCGCTCCGGATTTTTTTTCGCTCTCTCGCCGGTCATTTAAATCGGTGTTTTTTTTGGTTTTCCGATCT
4A-0969220-T10-25_0	ACACACATTTTCTACATGCTATAGGCTACAACACCTGCACGATCTTCGCCCACCTGGAAGGCGATCATAGCGGGCAGGCAGGTTCTGGCCGCGGATTTGATCTACCGAATAGGGGACGGC
4A-0969220-T10-25_63	ATCATAGCGGGCAGGCAGGTTCTGGCCGCGGATTTGATCTACCGAATAGGGGACGGCTCAAGCATGTCCACATGGAATGACCAGTGGATTCCCAACACAGCCACCCTGAAACCTATGGG
4A-0969220-T10-25_126	ATGTCCACATGGAATGACCAGTGGATTCCCAACACAGCCACCCTGAAACCTATGGGGCATTTAGGTACTGCACCTCTAACCCACGTCTCACACTTAATTGATGAATACACAGGGTGCTGG
4A-0969220-T10-25_189	GGTACTGCACCTCTAACCCACGTCTCACACTTAATTGATGAATACACAGGGTGCTGGAACATTGATCTAGTTCGTGAGAACTTTCTTCCGCCAGATGCTGAAGCCATACTGAACATACCA
4A-0969220-T10-25_252	GATCTAGTTCGTGAGAACTTTCTTCCGCCAGATGCTGAAGCCATACTGAACATACCATCGAATACATCTGGGGGGGGGGGAAGATAGCCTTGCGTGGGCATTCGAAAAGTCTGGAAACTA
4A-0969220-T10-25_441	TACCGAAACTTCAACAACAAATATCAGTTGTGGTCCGCACTTTGGAAACCGCCTATCATTCCGAAGGTAAGGGTGTTCTGGTGGCGGGTGCTCCGTGGCATCCTGCCAGATGCCAATACC
4A-0969220-T10-25_504	AAGGTAAGGGTGTTCTGGTGGCGGGTGCTCCGTGGCATCCTGCCAGATGCCAATACCCTCAAATATAAACACATTAAACCCCCGCGCATATGTGACATATGTCAGGCGATGGAGGAGGAC
4A-0969220-T10-25_567	TATAAACACATTAAACCCCCGCGCATATGTGACATATGTCAGGCGATGGAGGAGGACTTGATGCATGCTCTCATTTCATGTACTCATGCAAAAAAATTCTGGATTACAGCAAAGGCTAGA
4A-0969220-T10-25_630	CATGCTCTCATTTCATGTACTCATGCAAAAAAATTCTGGATTACAGCAAAGGCTAGATTTGATCTTCAACTTCCACGGCTGCACCCAGAAACTTGGTCTCACGACATTATGCTTGACCCA
4A-0969220-T10-25_693	CTTCAACTTCCACGGCTGCACCCAGAAACTTGGTCTCACGACATTATGCTTGACCCAATGTTCTCTGTGGACACAAGATGCATATCACAATTATGCACGCTATTTGGTCATCACGGAACC
4A-0969220-T10-25_756	TCTGTGGACACAAGATGCATATCACAATTATGCACGCTATTTGGTCATCACGGAACCGATGGACACACAATGAAGATGGCTATGATCCGGTTCAGGCGATCAAAGGAGTGCATGAAAACC
4A-0969220-T10-25_814	ATGGACACACAATGAAGATGGCTATGATCCGGTTCAGGCGATCAAAGGAGTGCATGAAAACCTTGCCATCCTTGATATCCCTTCGCGACTTCAGGCGCACGGGGGCCAAGGCTGGAAAC
4A-0969220-T10-25_1125	GGAGCAAGAGGCGTGGCTCGGTCTCACCTAGCGTTTCTTGGTGCATGGAGTAAACCACTGCCGGGAGTCTTTGATCCTTTTGTGGCCGAGCTGCTGCCGAGAAAAGGGGTGATTTTCCC
4A-0969220-T10-25_1190	AGTCTTTGATCCTTTTGTGGCCGAGCTGCTGCCGAGAAAAGGGGTGATTTTCCCTAACTACGAGGATACTCACATGTGATCATGGAGGTCGATTGCCTCGATCTCGTTAACCTCTGAAAG
4A-0969220-T10-25_1255	ATACTCACATGTGATCATGGAGGTCGATTGCCTCGATCTCGTTAACCTCTGAAAGTCGTGCCAAGATACACGCTCCATTGCGGCGACTATCTTACAATAATTGGAGAATATTATTACCTT
4A-0969220-T10-25_1320	ATACACGCTCCATTGCGGCGACTATCTTACAATAATTGGAGAATATTATTACCTTCGTTTTCTTCTTTTTCGGTTCATCGTGTTGGTAGGGACCTGAATTTTTCGGCACACCTTTGTGCA
4A-0969220-T10-25_1450	GTACGCTGGAGGGAAAAGATATTTGGCTTAGCAGCCTACGGACAGATTGTAACCGGATGATAGTTCGTTGAATAAAGTCTCCATTCGATGCAAAAAAAAAGAAAGTCATGGGCCAGTACC
4A-0969220-T10-25_1515	CGTTGAATAAAGTCTCCATTCGATGCAAAAAAAAAGAAAGTCATGGGCCAGTACGAAACCAATAAAATCATCTAAGCAGGATGAAACGATGTAGGAGACCACGAACTATATATTTTTTTC
4A-0969220-T10-25_1580	AATCATCTAAGCAGGATGAAACGATGTAGGAGACCACGAACTATATATTTTTTTCTTAGGGAAAATGACATTTTTTTATTAACGGGAAAATGACGAACTTTTTTTTTGAGCGGTGGGAAA
4B-1105240-T10-25_130	aaagatccaggcatatatatccggttggaatataaCACCACTGGCTTTGCACGGGTTTCAATGTGCGGCTTTGATCATTTTTATATATCAAAGTATATGCGTTGGACATGTATAAATTGT
4B-1105240-T10-25_195	CGGCTTTGATCATTTTTATATATCAAAGTATATGCGTTGGACATGTATAAATTGTACTGTATTATTTTTCTTGCGAAATATTTTTATTTCATATATGTCTAAAATAATTTTGTAGACATA
4B-1105240-T10-25_260	TTTTCTTGCGAAATATTTTTATTTCATATATGTCTAAAATAATTTTGTAGACATACAATACGTCAAAAGAAAAGTAGACATACAATGAAAAATAATGACTGTCAATGTTGTGCGACGAAG
4B-1105240-T10-25_325	AAAGAAAAGTAGACATACAATGAAAAATAATGACTGTCAATGTTGTGCGACGAAGACAGAAAAAGTCGAGCGCGCTTTACATGTTGGGCACGAGGGAGTATTAGGAAGCAGGGAGTAT
4B-1105240-T10-25_390	TCGAGCGCGCTTTACATGTTGGGCACGAGGGAGTATTAGGAAGCAGGGAGTATAATTTTATAGGCGTCATTTAATATCTAATTACTTTGTTTCAAACAAAGTGCCAAATTTAGTGATTGA
4B-1105240-T10-25_455	GTCATTTAATATCTAATTACTTTGTTTCAAACAAAGTGCCAAATTTAGTGATTGATTTTTCTTTCCCGCGTCATGCACACGCCTCTAATCTTTATGTGTTGGCTGATGTACCTAGACTCT
4B-1105240-T10-25_520	CCGCGTCATGCACACGCCTCTAATCTTTATGTGTTGGCTGATGTACCTAGACTCTACATGGAATCCTAATGATCTACACTGCCAACTAAACCAGGTGGGAGAATGCTTTAAAATAAGGCT
4B-1105240-T10-25_585	CTAATGATCTACACTGCCAACTAAACCAGGTGGGAGAATGCTTTAAAATAAGGCTTTTGTCGTCAACGTAAATAAAATGTTGTGACACAGACAAGGAAACGAATTATCAAAGTACTTTTT
4B-1105240-T10-25_650	ACGTAAATAAAATGTTGTGACACAGACAAGGAAACGAATTATCAAAGTACTTTTTTATTTATTTCTTTTGTCAAAGTACTAGAGTTGTTACTTACGTTTTGGATTCAATTCTATCCATCC
4B-1105240-T10-25_715	TTTTGTCAAAGTACTAGAGTTGTTACTTACGTTTTGGATTCAATTCTATCCATCCGTTTCAAAATAGTGTCAAAAACGTTCTTATATTTTAGAACGGAGGGAGTAGTTTTTTAAAGTCGT
4B-1105240-T10-25_780	AGTGTCAAAAACGTTCTTATATTTTAGAACGGAGGGAGTAGTTTTTTAAAGTCGTCATCCAAATTTTTTATTCATATGATACGCCTAAATAAATAATGTATTCATCATATAACCAAATTG
4B-1105240-T10-25_845	TTTTATTCATATGATACGCCTAAATAAATAATGTATTCATCATATAACCAAATTGATCAGATTATCTTATACTAAAAAATAAGCACATCAAACATTGATAGTGCTATGATTTGATTGGTA
4B-1105240-T10-25_910	CTTATACTAAAAAATAAGCACATCAAACATTGATAGTGCTATGATTTGATTGGTATCATCATTTTTTTTTGATATCTCCCATGGCAACGCATGGGCATTCGGCTAGTAGCTATAGATTTC
4B-1105240-T10-25_975	TTTTTGATATCTCCCATGGCAACGCATGGGCATTCGGCTAGTAGCTATAGATTTCTCGATGTAGCAGACCATGAACTGTTTTTCTTAGGAAATTGACGAATATTTTTCTTGAATAACGGG
4B-1105240-T10-25_1040	AGACCATGAACTGTTTTTCTTAGGAAATTGACGAATATTTTTCTTGAATAACGGGAAAATGCCGAACTATTTTTTTGAGCGGTGGGAAAATGACGAACTATATCCGAGCAGCAATATCTG
4B-1105240-T10-25_1170	TGGCTCATCCCTAGTAGACAGCTCGTTTCACAATTGATCTGGCCCATAATCTCTCTCTCTTTTCTTCCTCTCCGAAAATCATCATTCCCCCGCCCAAACCCCACTCCCCTTTTTCCCTCC
4D-1162360-T10-25_1472	TCCCCCTTTTGTCTACTAGTGTCAAAAAGGTGACGAAAATATATCCTAGGAGCAATATCTGGGCCAGGCTATGCCTCAGATATTGATCTGGCCCATAATCTCTCTATCTTTCCTTCCTCT
4D-1162360-T10-25_1536	CAGGCTATGCCTCAGATATTGATCTGGCCCATAATCTCTCTATCTTTCCTTCCTCTCCGAAAATCATCATTTCCCCGCCCAAACCCCACGCCCCTTTCCCTCCCAAAAAATTCCCAATCC
2A-0297450-T10-26_128	CAGTCTCAAAATGCCTCAAATTGTGTGATAGATATAACTTCTGCACGCCTACAAAATATGATGCAAGAAACAGAGTAAGAAACAAGAATGCAAATGATGGTGAGCCCTGTATACATCACT
2A-0297450-T10-26_192	AAGAAACAGAGTAAGAAACAAGAATGCAAATGATGGTGAGCCCTGTATACATCACTAAGGATGCCTACATTAGCAACTGCGATGGTGAGATCATATCAGAAGCTCAAAACTTCACTGAAG
2A-0297450-T10-26_256	CTACATTAGCAACTGCGATGGTGAGATCATATCAGAAGCTCAAAACTTCACTGAAGACAAGCTATAAATAACTGAAAGCTTGTTTAAAATAGTAGACCTAAAGATGTTATCCATATTAGG
2A-0297450-T10-26_320	TAAATAACTGAAAGCTTGTTTAAAATAGTAGACCTAAAGATGTTATCCATATTAGGGTTTTGACCAACCATCATCAGAGCTCACCGAAAAGGCGAAAACAAACGGATAAGGCAAGACTAA
2A-0297450-T10-26_384	CAACCATCATCAGAGCTCACCGAAAAGGCGAAAACAAACGGATAAGGCAAGACTAAGTCCAGTTATCCAGTCAATCGCAGCGGTTCTAAAGCAGGCGTGTGCTGGGCGACGAAATAGA
2A-0297450-T10-26_448	ATCCAGTCAATCGCAGCGGTTCTAAAGCAGGCGTGTGCTGGGCGACGAAATAGAGTATGAGATGAGCCATACTCCATCAATTGCAGTCCTGTGGACATGATTCTGTTCAAACAATCGATA
2A-0297450-T10-26_512	AGCCATACTCCATCAATTGCAGTCCTGTGGACATGATTCTGTTCAAACAATCGATAGCACTGAAAATCAATGCATTTTTGGCCGTGCATATACCATACGCACACAGGGTTACGTTTTGGC
2A-0297450-T10-26_576	AATCAATGCATTTTTGGCCGTGCATATACCATACGCACACAGGGTTACGTTTTGGCTGGGAAGAAATTGAACAAACCCCTCCCCGCGGAGGCAGGCATTCCGTCGAACACCCTATCATCG
2A-0297450-T10-26_640	AATTGAACAAACCCCTCCCCGCGGAGGCAGGCATTCCGTCGAACACCCTATCATCGAACCGTACAGTCAGCCCCGCCGAGGACCGAAAACAACCCCTCCTCGCCGCTCCCAGCCACGAA
2A-0297450-T10-26_704	AGTCAGCCCCGCCGAGGACCGAAAACAACCCCTCCTCGCCGCTCCCAGCCACGAACACGATAAGAGATCCCGCGGCCGCGGTGCCGCGTGAACTCGCACCAGAAAGGTCCCCGGGAA
2A-0297450-T10-26_768	AGATCCCGCGGCCGCGGTGCCGCGTGAACTCGCACCAGAAAGGTCCCCGGGAAAACACGAGAAATTGCAAAGACGGAGGCGACTGGGTCGGGGACAGCGAGATCACCTGGCGCC
2A-0297450-T10-26_960	ATGGGGAAGGAAATCTGGGTGGGTTTGTGCGGAGGGAATGGCCAGCCGCCGCGCGGGATATGGTACACGGGAAATTGAAAACCTCCCCCGTGGAGGGAAACGAATTAGGGAACCGG
2A-0297450-T10-26_1152	ACAACGGTGCTCAAATTTCAACGGGTAGGTTGTGTGTGAAAGGGTATTTGCGCGTGTACAGGAGTGCGAGTGTGATTCCGTTGCCCATTTTGGTGATTTCCATCTCGTATTCCAGTGTTT
2A-0297450-T10-26_1216	TGCGAGTGTGATTCCGTTGCCCATTTTGGTGATTTCCATCTCGTATTCCAGTGTTTGGAAAATTCTGCAAAGAAAAAAGTAGCGAGCAGAAATTTCTGTATTGTAGGGTAAATTTGTAGT
2A-0297450-T10-26_1536	CAGACCTCCAAACGGACCCGTAAGGAAGTATATTCATGGAATATGCTTTTTTTACGGGCTGGTATATGTACGACCCAACAAGTTCACGAATTCGGTTGTTGCATTGTTCGAACCACCACT
2B-0402520-T10-26_0	GATCTGTTTATTTAGATGTGCACTGCAGTGTATAACCACAGAAAATATCAGTATTAATACTGGTAAACCGAGCTTCATAATATACACAAAAATAATCAGTAGGCCCAAGAACCAAACCGC
2B-0402520-T10-26_192	ATCAACTGATCCTTCATCCCTTGCAAATGATAATTCGGCTCTGGGCAAAAAGCTAGCTGCACAAGTTTTGTATTAACTTGTGAGTTAACATGCTGAAATAGCTACTACCTGAACAGCATG
2B-0402520-T10-26_256	GTTTTGTATTAACTTGTGAGTTAACATGCTGAAATAGCTACTACCTGAACAGCATGAATGATGATTCAACTAATACTTTGCTTACCTTGTAACCATGACTACAAATTTCAGAGAGTTCTT
2B-0402520-T10-26_320	TTCAACTAATACTTTGCTTACCTTGTAACCATGACTACAAATTTCAGAGAGTTCTTGTGGTAAACTATTATCATCTGAACCTGTGAAGTTACACTTGTTTCCTGATGGAGTTGACATTTG
2B-0402520-T10-26_448	CTAACCCTCTCGGTGTATTTGTTGCTTCATAATGTTCCATAACTATGCGGTCCACATCAATACTCTGGAACATAACTAGATTTGTCACCAATTATGATATGATAAGATGGTATGTTCATC
2B-0402520-T10-26_512	CTGGAACATAACTAGATTTGTCACCAATTATGATATGATAAGATGGTATGTTCATCTAATTCTGGGAAGATAATTCAGTAGATAAAATATCAAATTTATCAGATTAAAGCACCTACCGCC
2B-0402520-T10-26_576	GGAAGATAATTCAGTAGATAAAATATCAAATTTATCAGATTAAAGCACCTACCGCCAGAATCTCGTCATCATCCATGGCATCTAACTTATCATCAGCACACATGTTATCAGCAACAGGAA
2B-0402520-T10-26_640	GTCATCATCCATGGCATCTAACTTATCATCAGCACACATGTTATCAGCAACAGGAACAGTCTGGCATGACATAGCATCAGTTCTGGCAAAACTGCTCTGATATGTTGTTCCAACCACATG
2B-0402520-T10-26_832	AGTGCGGCGATGCAGACTTTCCTGAGAGGGAATTGTACTTTTCGGCACATTATCCAATTTGTTGGCATTATATGAAGAATTTGTTGCATAAATAGGAGAAAATTCCCTGCCACAATCTTT
2B-0402520-T10-26_896	GCATTATATGAAGAATTTGTTGCATAAATAGGAGAAAATTCCCTGCCACAATCTTTCACCTTTGCAGATAAACCAGGTCTTAAATAATTCTTACAGGTCACCTGGGTGTTACACACAGAA
2B-0402520-T10-26_1024	CTGTCATAGAAGTGCCATTAGATAATAACCACACAAAAATGAACAGAACACACTGAACTTTTTAGATAGTGGCAATAAGGTTTATGCTTTTTTTTTCAGCAAATTGATAAACTGGAAGTAA
2B-0402520-T10-26_1088	GATAGTGGCAATAAGGTTTATGCTTTTTTTTTCAGCAAATTGATAAACTGGAAGTAAAAGACAAGACAGGATTGAATTTACAGACATGCAGCAAGTGGGTAACCAGTCCTAACTCCAACAA
2B-0402520-T10-26_1216	GCGCCTTTAGTGGACAGCTAACAAGTTTAAAAACAAACTGTCCTAGTTTTGCACTTAGTCAAGACAAACAAGCGCCTAATAGCTTTTATAACATGGTTTTGAAAGAAAACTGCTTCTTAC
2B-0402520-T10-26_1280	CAAACAAGCGCCTAATAGCTTTTATAACATGGTTTTGAAAGAAAACTGCTTCTTACAGTAAGATTAGCAAAATCTACAGGTCAAAGTGATCGTGTAGCTATAGCACTTTTAAGCATTGTG
2B-0402520-T10-26_1344	TAGCAAAATCTACAGGTCAAAGTGATCGTGTAGCTATAGCACTTTTAAGCATTGTGTGATGATTCATAGCATGTTACAGTATTATTGCCATACCTTCTCGATCCATGGAACTCGAAGGCG
2B-0402520-T10-26_1472	CTTGAATTTTACAAGCAGCGGACCTGGGAATATGAGTTTTGTTGCTGATGCGGTGAACAACTGTCTCTAGGATAAGATCAACTAATTGCATATAACAGAACTATACCTTAAAGAAAGCAT
2B-0402520-T10-26_1536	CTCTAGGATAAGATCAACTAATTGCATATAACAGAACTATACCTTAAAGAAAGCATTGTATTGCAGTTCGCTTCTGGATTTGCCCTTTGTGTTGGTAAAGAGAACAGAAAGCTTGAACTC
2D-0530420-T10-26_64	ATCTGTTTATTTAGATGTGCACTGCAGTGTATAACCACAGAAAATATCAGTATTAATACTGGTAAACCGAGCTTCATAATATACACAAAAATAATCAGTAGGCCCAAGAACCAAACCGCT
2D-0530420-T10-26_256	TCAACTGATCCTTCATCCCTTGCAAATGATAATTCAGCTCTGGGCAAAAAGCTAGCTGCACAAGTTTGGTATTAGCTTGTGAGTTAACATGCTGAAATAGCTACTACCTGAACAGCATGA
2D-0530420-T10-26_320	TTTGGTATTAGCTTGTGAGTTAACATGCTGAAATAGCTACTACCTGAACAGCATGAATGATGATTCAACTAATACTTTGCTTACCTTGTAACCATGACTACAAATTTCAGAGAGTTCTTG
2D-0530420-T10-26_384	TCAACTAATACTTTGCTTACCTTGTAACCATGACTACAAATTTCAGAGAGTTCTTGTGGTAAACTATTATCATCTGAACCTGTGAAGTTACACTTGTTTCCTGATGGAGTTGACATTTGC
2D-0530420-T10-26_512	TAACCCTCTGGGTGTATTTGTTGCTTCATAATGTTCCATAACTATGCGGTCCACATCAATACTCTGGAACATAACTAGATTTGTCACCAATTATGATATGATAAGATGGTATGTTCATCT
2D-0530420-T10-26_576	TGGAACATAACTAGATTTGTCACCAATTATGATATGATAAGATGGTATGTTCATCTAATTCTGGGAAGATAATTCAGTAGATAAAATATCAAATTTATCGGATTAAAGCACCTACCGCCA
2D-0530420-T10-26_640	GAAGATAATTCAGTAGATAAAATATCAAATTTATCGGATTAAAGCACCTACCGCCAGAATCTCGTCATCATCCATGGCATCTAACTTATCATCAGCACACATGTTATCAGCAACAGGAAC
2D-0530420-T10-26_704	TCATCATCCATGGCATCTAACTTATCATCAGCACACATGTTATCAGCAACAGGAACAGTCTGGCATGACATAGCATCAGTTCTGGCAAAACTGCTCTGATATGTTGTTCCAACCACATGG
2D-0530420-T10-26_896	GTGCGGTGATGCAGACTTTCCTGAGAGGGAATTGTACTTTTCGGCACATTATCCGATTTGTTGGCATTATATGAAGAATTTGTTGCATAAATAGGAGAAAAATCCCTGCCACAATCTTTC
2D-0530420-T10-26_1088	TGTCATAGAAGTGTCATTAGATAATAACCACACAAAAATGAACAGAACACACTGAACTTTTTAGATAGTGGCCATAAGGTTTATGCTTTTTTTTCAGCAAAGCGATAAACTGGAAGTAAA
2D-0530420-T10-26_1152	ATAGTGGCCATAAGGTTTATGCTTTTTTTTCAGCAAAGCGATAAACTGGAAGTAAAAGACAAGCCAGGATTGAATTTACAGGCATGCAGCAAGTGGCTAACCAGTCCTAACTCCAACAAA
2D-0530420-T10-26_1216	CAGGATTGAATTTACAGGCATGCAGCAAGTGGCTAACCAGTCCTAACTCCAACAAAATGCGATGCGCCTTTAGTGGACAGCTAACAAATTTAAAAACAAACTGTCCTAGTTTTGCACTTA
2D-0530420-T10-26_1280	CGCCTTTAGTGGACAGCTAACAAATTTAAAAACAAACTGTCCTAGTTTTGCACTTAGTCAAGACAAACAAGCATCTAATAGCTTTTATAACATGGTTTTGAAAGAAAACTGCTTCTTACA
2D-0530420-T10-26_1344	AAACAAGCATCTAATAGCTTTTATAACATGGTTTTGAAAGAAAACTGCTTCTTACAGTAAGATTAGCAAAATCTACAGGTCGAAGTGATCGTGTAGCTATAGCATTTTTAAGCATTCTGT
2D-0530420-T10-26_1408	AGCAAAATCTACAGGTCGAAGTGATCGTGTAGCTATAGCATTTTTAAGCATTCTGTGATGATTCATAGCATGTTACAGTATTATTGCCATACCTTCTCGATCCATGGAACTTGAAGGCGT
2D-0530420-T10-26_1536	TTGAATTTTACAAGCAGCGGACCTGGGAATATGAGTTTTGTTGCTGATGCGGTGAACAACTGTCTCTAGGATAAGATCAACTAATTGCATATAACAGAATTATACCTTAAAGAAAGCATT
2D-0530420-T10-26_1580	TGATGCGGTGAACAACTGTCTCTAGGATAAGATCAACTAATTGCATATAACAGAATTATACCTTAAAGAAAGCATTGTATTGCAGTTCGCTTCTGGATTTGCCCTTTGTGTTGGTAAAGA
2A-0356710-T10-28_128	GCCTGAGGAAGTGAAGAAGAGGGCGAGATTTGTGAGGCTGAGCTCGTGGTCCCGGATTAAGTGTTGTTGTTTTTACTGCTGAGCCGGTATATCATCTTGAAATTTACGAAGTTACTGTAA
2A-0356710-T10-28_192	TGTTGTTTTTACTGCTGAGCCGGTATATCATCTTGAAATTTACGAAGTTACTGTAAGCATCTCATTGAAAATTTTGAAATAAATTCAGCAATAAATACGAGCACTAGGACTTTACTCTGA
2A-0356710-T10-28_320	GGATACCCTCTAACCATCCAACCACAGGTTGGTTTGCGTTCCGGATTAAGTGTTAGGGGATCTGCTACCTGAACAAAGTATTAAGGTTAAGAACAACTCCAATGAGGTGACTCATTTTGT
7B-1943100-T10-29_704	CGGCTCTTCTTCACACTTTCACGGGCAGGCCATCCAAAGCCTAACAAGCTGAGAAGCAGAGGCCCATTTACACGGTGATGGCCAGCCCGTTTCCCCTATTCTTTTCCTGACGTGCTTTCT
7B-1943100-T10-29_768	CATTTACACGGTGATGGCCAGCCCGTTTCCCCTATTCTTTTCCTGACGTGCTTTCTTTAGAAAAAATAGGTTTCCACCGGTTCATATATTTTTACAAAAAACAGGATTGTCAAATCTTAG
7B-1943100-T10-29_832	AATAGGTTTCCACCGGTTCATATATTTTTACAAAAAACAGGATTGTCAAATCTTAGTCGACTAAGACTAGTCTCGGACAATGTTATATTCGTGAGAGCTTACGCGAAGATTCGTGCAAAA
7B-1943100-T10-29_896	GACTAGTCTCGGACAATGTTATATTCGTGAGAGCTTACGCGAAGATTCGTGCAAAAAAAATTAGTTTTTTTTTCTTTTTTCTTTTCTATATGTATGTCACTTAATTAAGACTTGATTAAG
7B-1943100-T10-29_960	TTTTTTTTTCTTTTTTCTTTTCTATATGTATGTCACTTAATTAAGACTTGATTAAGTCTCAGTCGACCGAGATCTACACACTCTATAAAAAATGCAACCCAGTTCCTTGGAGTTACATGT

7B-1943100-T10-29_1024	GACCGAGATCTACACACTCTATAAAAAATGCAACCCAGTTCCTTGGAGTTACATGTTTTCAGTGGGAAGTAGAGTACCTCCTTGTAAGTGCCACAACATCACAATAACATCAACTGGTCA
7B-1943100-T10-29_1088	GGAAGTAGAGTACCTCCTTGTAAGTGCCACAACATCACAATAACATCAACTGGTCAAAGGCAAAAGGACCCAAAAACTAAGCTAACACCATCACCACATGATCACATCTTTGAGAAGGAA
7B-1943100-T10-29_1152	AGGACCCAAAAACTAAGCTAACACCATCACCACATGATCACATCTTTGAGAAGGAACTAAAGTAGAGGTCTCGCTAGGCTAATTGGTGTTCTTGAGTAGGTATCGAATCGGGGCTTGTCA
7B-1943100-T10-29_1216	GAGGTCTCGCTAGGCTAATTGGTGTTCTTGAGTAGGTATCGAATCGGGGCTTGTCAGACGGTAGATTCACCAAATGATCCTCATTAATTCGCCCATCTTCTCGTCCAAAATACATATATA
7B-1943100-T10-29_1280	ATTCACCAAATGATCCTCATTAATTCGCCCATCTTCTCGTCCAAAATACATATATATTGAGCCCATTAGAGCGAAGTGTGCAGGCTTACATAACAATCTCAACACATGAAACTCCCTTTC
7B-1943100-T10-29_1344	ATTAGAGCGAAGTGTGCAGGCTTACATAACAATCTCAACACATGAAACTCCCTTTCTAGGCAGCACCGTACCTCCCCGATGCTTCTCCAGACATCGCCCTAGAAGTACAAGTCATTTTGA
7B-1943100-T10-29_1408	ACCGTACCTCCCCGATGCTTCTCCAGACATCGCCCTAGAAGTACAAGTCATTTTGAATCTTTTTATAAGGTCCACATGGTAGTCACACTAAAAGCATTAAGAACAACACCATAAAATATC
7B-1943100-T10-29_1472	ATAAGGTCCACATGGTAGTCACACTAAAAGCATTAAGAACAACACCATAAAATATCTGTATACCACCACTCGAAAACAGAATGCAAGTTTGCGCCTAAAAGATCATAAAGGAACAACCAC
7B-1943100-T10-29_1536	ACCACTCGAAAACAGAATGCAAGTTTGCGCCTAAAAGATCATAAAGGAACAACCACACATACTACCTACAACATGGTCAACAAAAAGGTCCTCTGCCCATGGAAATTGAATTTTCTTCGC
7B-1943100-T10-29_1580	AGGAACAACCACACATACTACCTACAACATGGTCAACAAAAAGGTCCTCTGCCCATGGAAATTGAATTTTCTTCGCCGCCCACCATATCCCCTCTCCCGATGCTTCGCTAGGCGAGTTCC
7D-2033840-T10-29_64	AATCGGAGAGGTCGGGCTAGGATTGGGAGGGAGAGGAGGGAGGGCTCGTTACCTTGGGTTCGGCGAGTGGAGCGCCGGCTCGGCGCCTCCCCGTCGACGCGTGGCTCCTGGGGA[illegible]
7D-2033840-T10-29_384	AACGGGGTCGACCTCGTGGGCTCATGATCACCGGCATTTTTTTTTTGTTTGAGGAACCGGCACTTCTTCACTCTTTCACGAGCAGGCCATCCAAAGCCCAACAAGCCCAGAAGCAGAGG
7D-2033840-T10-29_448	TCTTCACTCTTTCACGAGCAGGCCATCCAAAGCCCAACAAGCCCAGAAGCAGAGGCCCATTTACAAGGTGATGGCCAGGCTGACCTGTTTTTTCTAAACACCAGCCCGTTTCCCCTTTTC
7D-2033840-T10-29_512	AAGGTGATGGCCAGGCTGACCTGTTTTTTCTAAACACCAGCCCGTTTCCCCTTTTCCTTTCCTGACGTGCCTTTTTTTTAAAAAAAACAAGGTTTTCCACTGGTTCATATATTTTTTTTA
7D-2033840-T10-29_576	ACGTGCCTTTTTTTTAAAAAAAACAAGGTTTTCCACTGGTTCATATATTTTTTTTAAATGCAACCAAATTCCTAACAATTCATCTCCTCATAAGTGCCACAGCAGCACGATAACATCAAC
7D-2033840-T10-29_640	CAAATTCCTAACAATTCATCTCCTCATAAGTGCCACAGCAGCACGATAACATCAACTTGTAAAAGCAAAAGGAGCAAAAACAAAACAAAGTTAACGCCATCAGCACGGGATCACAGATCT
7D-2033840-T10-29_704	GCAAAAGGAGCAAAAACAAAACAAAGTTAACGCCATCAGCACGGGATCACAGATCTGAGAAGGAGATAAGTAGAGGTCTCGTTAGACCAGTTGGTGTTCTTATAAGAAGGTATCTTAG[illegible]
7D-2033840-T10-29_768	GATAAGTAGAGGTCTCGTTAGACCAGTTGGTGTTCTTATAAGAAGGTATCTTAGCGGGGCTTGTCTGTAGTAGATCACCAAGCGATCCTCAACAATTCGCCTCTCTTCTTGTCCAGATAC
7D-2033840-T10-29_832	CTGTAGTAGATCACCAAGCGATCCTCAACAATTCGCCTCTCTTCTTGTCCAGATACATATGTTGGGTCCATTAGAGCGAAGTGTGCGGATTTGCATAACAACTCCACACACGCAACTCCC
7D-2033840-T10-29_896	GGTCCATTAGAGCGAAGTGTGCGGATTTGCATAACAACTCCACACACGCAACTCCCTTTCCATGCAGCAATGTACCTCCCTAATACTTCTCCAGGTCTGATGAGGACATGACTACCTTGA
3A-0628520-T10-30_512	ATCGCGCTCGGGGGAGGAAGGAAAAAGTGGTTGGTGCCAACAAAAAATTCAAGCAGCTGAGGTTTAACAAAACTGATAGTTTAATGGTTTAGTTTCTTTCTTGTAACCATTGTTTGCAAT
3A-0628520-T10-30_576	TAACAAAACTGATAGTTTAATGGTTTAGTTTCTTTCTTGTAACCATTGTTTGCAATTTATAAAGCAGCCATGAAAACGTTCCCTCAAACAAAAATTATCATTGGAAACATTTTTCAATAT
3A-0628520-T10-30_640	CAGCCATGAAAACGTTCCCTCAAACAAAAATTATCATTGGAAACATTTTTCAATATGAATAGAATGGTATACTTTTGATTATACCTTAATTTATGAGAAAGGTTCTGTTCCGACCAACCG
3A-0628520-T10-30_896	CAAAAGGAAGGCTTTCGAAGACTTGTCATACATTCCAAGATGTTGAGATCGTATTAAACGCGTCCTTCCTGTTCGTACCAGACTAGGATGAAACTATAGCCCCCCTCCCGAATACCCATA
3A-0628520-T10-30_1152	TGTTAACAAAATTTACCGATAACCCATTTATCAATAGGTTAATTAGGGTCATATCTATATATCTTAGCTACATATGCAATAATGCAGCAGCAACAACAATAAAGGAGAGGCAATAAACAT
3A-0628520-T10-30_1216	TAGCTACATATGCAATAATGCAGCAGCAACAACAATAAAGGAGAGGCAATAAACATCAATAGCATGTAGCATAGCACAAAACATATTAGCATATAACACAAACGGTTATATGCTGGTCTA
5A-1199250-T10-31_0	CGACGTGTTCTGGTAAAAAAAAATGAACTTAGATCAGCCAACGGTAAAGACTGAAATGTATCGAAGCAGGCAGTGTCCGAAAACTATTCCAGAAACTTCAGATAACTAACTAACTTGTCGA
5A-1199250-T10-31_63	AGCAGGCAGTGTCCGAAAACTATTCCAGAAACTTCAGATAACTAACTAACTTGTCGATATGCTACTTTTCATCAGGTGAGGACTGATCACGATGTGAGATTACAGATGCAGTTCTATCAT
5A-1199250-T10-31_126	ACTTTTCATCAGGTGAGGACTGATCACGATGTGAGATTACAGATGCAGTTCTATCATTGAGGCAAAATGTGCATGTAGCCAGCCAACCAACATGGTAAGGAACAGATTGTTTTTCTTTTT
5A-1199250-T10-31_189	AAAATGTGCATGTAGCCAGCCAACCAACATGGTAAGGAACAGATTGTTTTTCTTTTTCTCTTTTTTTTGGAACATAAGGGAACAGATTATCTAGGCTGCAACTCAGAACGCCACTACGCC
5A-1199250-T10-31_252	TTTTTGGAACATAAGGGAACAGATTATCTAGGCTGCAACTCAGAACGCCACTACGCCAGCCCCAACTCGCGAGCACATTGGACGAGCAAACCATCACGTCCTACTCTTACAAAAGGCGGA
5A-1199250-T10-31_315	AACTCGCGAGCACATTGGACGAGCAAACCATCACGTCCTACTCTTACAAAAGGCGGATCTAGATGGGGTGCGCCCGAGTTTTTTGGAGGTTCGTTAGGGTTTAACCAATCGTGGACGGA
5A-1199250-T10-31_378	TGGGGTGCGCCCGAGTTTTTTGGAGGTTCGTTAGGGTTTAACCAATCGTGGACGGAGAAGTGGCCGTACCTGAGAACCCAATGGCCGAAATCGTGGGCAGCGTTGGGATGGCGGGGG
5A-1199250-T10-31_441	CCGTACCTGAGAACCCAATGGCCGAAATCGTGGGCAGCGTTGGGATGGCGGGGGAGCGCCGAGGCTGGAGCGGGAACGGCGATTCCGGTGACGCGTCcgacgggggccggggtggcggcg
5A-1199250-T10-31_842	TGGCGCGTGCAGTCGGTGGTGGGAGAGAGGAGGGGGGGATTGTTAATTTTCTGTTTGGGAGGGGGGTTGGGGCGAAATTTGCAAGCCGTGTTGGGCCTTGCAGGGCCTCTTTTATCG
5A-1199250-T10-31_903	GGGGGTTGGGGCGAAATTTGCAAGCCGTGTTGGGCCTTGCAGGGCCTCTTTTATCGGGCCCAGAGGCCCTGCACGGTCTAACAAGTCCAATTTAGTGCAGGATAGGCctataatatacag
5A-1199250-T10-31_1269	TTAACTAGTATGAACTAAACAAAAGAATGATAGAACCCTAAAAAATGATAGGCCAATAGTTTGTTCCGACAAAAAAATGGATGAAATCATTTTCGATGTACTTTGCCACGCTTTGGAATTT
5A-1199250-T10-31_1330	TGTTCCGACAAAAAAATGGATGAAATCATTTTCGATGTACTTTGCCACGCTTTGGAATTTCAGATGATCCATGACCCATATATTCCAAAAAAGAATGGCAACCTGTTGCCACGATTTTGAA
5A-1199250-T10-31_1391	GATGATCCATGACCCATATATTCCAAAAAAGAATGGCAACCTGTTGCCACGATTTTGAATTTCATATGGTCCATGCCTCATATATTCCAACAGAAAATGACAATATGTTTTGTTACACAC
5A-1199250-T10-31_1452	TCATATGGTCCATGCCTCATATATTCCAACAGAAAATGACAATATGTTTTGTTACACACAATTACCTAAGGCTAGTTTTTGCAAAGCTAGATATGAACCAAACATACCTTGAACCCATGT
5A-1199250-T10-31_1513	TTACCTAAGGCTAGTTTTTGCAAAGCTAGATATGAACCAAACATACCTTGAACCCATGTTGTCGTGCCCATGCTATTCTTTTGTGTAACACTGCTTAGCTCTTCTGTTTCTAAAAAAGCT
5A-1199250-T10-31_1570	GTTGTCGTGCCCATGCTATTCTTTTGTGTAACACTGCTTAGCTCTTCTGTTTCTAAAAAAGCTAGACTAGTAGCGGAGGAGCCCCTATTGGCTTTTACATCAAGAATTTCTCCCCCCCCC
5B-1334260-T10-31_0	CGACGTGTTCTGGTTAAAAAAAGGGAGCTTAGATCAGCCAACAGTAAAGACTGAAATGTATCGAAGCAGGCTGCATCGGAAAATCATTCCAGAAACTTCAGAAAATTCACTAACTTGTC[illegible]
5B-1334260-T10-31_62	GAAGCAGGCTGCATCGGAAAATCATTCCAGAAACTTCAGAAAATTCACTAACTTGTCGATATGCTACTTTTCATCAGGTGAGGACTGATCACAATGTGAGATTACACAGGCAGTTCTATC
5B-1334260-T10-31_124	GCTACTTTTCATCAGGTGAGGACTGATCACAATGTGAGATTACACAGGCAGTTCTATCATTAAAGCAAAATGTGCATGTAGACTATTAACCAACATGGTAAGGAACAGATTGTTTTTCTT
5B-1334260-T10-31_186	AAGCAAAATGTGCATGTAGACTATTAACCAACATGGTAAGGAACAGATTGTTTTTCTTTACTTTTCTGGATCATAAGGAACAGAttgtttttcttttcttttcttcttttttGGAACAT
5B-1334260-T10-31_248	TTTCTGGATCATAAGGAACAGAttgtttttcttttcttttcttcttttttGGAACATAAGGAACAGATTATCTAGGCTGTAACTCAGAACGCCAGCCCCAGCTCGCGAGCACATTGGAC
5B-1334260-T10-31_310	AACAGATTATCTAGGCTGTAACTCAGAACGCCAGCCCCAGCTCGCGAGCACATTGGACAAGCAAGCCATCACGTCCTATTCCTACGAGAGGCGGATCTAAATGGGGCGCGCCCGAGTTT[illegible]
5B-1334260-T10-31_372	AAGCCATCACGTCCTATTCCTACGAGAGGCGGATCTAAATGGGGCGCGCCCGAGTTTTTCGGAGGTTCGTTAGGGTTTAACCAGTGGTGGACGGCGAAATGGCCGTACCTGAGAACCC[illegible]
5B-1334260-T10-31_1040	GGGTCGGCGCAGGGATGGGAGGGGGGCTCGCCGCCGGTGGGGCTggagagggagagggaagggcgcgggcagtcggtggtgggagagagaggaggggggGATTGTAAATTTTATGTTTGGGAG
5B-1334260-T10-31_1220	CGCAGGCCCTGCACGGTCTAATAAGTCCAATTTAGTGCAGAATAGGCCTATAATATGCAGTATGAGCTAAAAAAAGAATGATGGGCATGTAGTTTGTTCCGACAAAAAAAATGGATGGAA
5B-1334260-T10-31_1280	TATGAGCTAAAAAAAGAATGATGGGCATGTAGTTTGTTCCGACAAAAAAAATGGATGGAATCATTCCCGATGTACTTTGCCACGCTTTGGAATTTCATATGATCCATGACTCATATATTT
5B-1334260-T10-31_1340	TCATTCCCGATGTACTTTGCCACGCTTTGGAATTTCATATGATCCATGACTCATATATTTCAAAAGGACGCAGGGGTCTGTCCATACGTTTTGTGTGGTGTTTAAAAGAATCAACGCATA
5D-1437570-T10-31_0	GGCAACGAGAGTGAGGTGGTGGCATCGATAATGGGGATATGGAAGTGTGGTGGGCCTGGTAGGGAATGGATCAACGCCAATGGTGGATGATCGCGACAAGGAAAGTGCACACGTGG[illegible]
5D-1437570-T10-31_64	AATGGATCAACGCCAATGGTGGATGATCGCGACAAGGAAAGTGCACACGTGGCATGCCAATAAATGTGCGAACAAACAAGCAAGCTTCGCCACAACTGCAAATTGGTGGACAAGAGGA
5D-1437570-T10-31_128	TGTGCGAACAAACAAGCAAGCTTCGCCACAACTGCAAATTGGTGGACAAGAGGATGGGAGCAATGATGGAGAAGAACGGAGGTAGCCAAAGTAGGAAGGGGGTGGCCCCTTGCAGA
5D-1437570-T10-31_192	GATGGAGAAGAACGGAGGTAGCCAAAGTAGGAAGGGGGTGGCCCCTTGCAGAGAAGAACAAGGTAGACGCATAAGATGGATATAACAAAACATAATCTTATCATATATCATGAATACAA[illegible]
5D-1437570-T10-31_256	AGACGCATAAGATGGATATAACAAAACATAATCTTATCATATATCATGAATACAATGCATCAAGAGCACACAACACTACTATAAATAGTAAAAGAGAAACAAAGGAACACTAGGATGCGC
5D-1437570-T10-31_320	AGCACACAACACTACTATAAATAGTAAAAGAGAAACAAAGGAACACTAGGATGCGCATATGACCACTAGTCATTACTATAGACAATGTACAACAACATTATTTTTTTGCAACTTTTTTTC
5D-1437570-T10-31_384	ACTAGTCATTACTATAGACAATGTACAACAACATTATTTTTTTGCAACTTTTTTTCAGGTAATAATTTGTCTGCGTGAACATTCTTTTTCATGTGAGAAATCATTTAGGGAAGCAAGTCC
5D-1437570-T10-31_448	ATTTGTCTGCGTGAACATTCTTTTTCATGTGAGAAATCATTTAGGGAAGCAAGTCCTTGGAAAAGCAATAATCCAAGGGCATGTCCTTTCCTGTAAAAAAAAATTGTGGTTTAGTGGGAGT
5D-1437570-T10-31_512	GCAATAATCCAAGGGCATGTCCTTTCCTGTAAAAAAAAATTGTGGTTTAGTGGGAGTTGGCCCAAAAAAGTGCTCCAACAGTTTCCGTAAACAAAAAAAAGGGTACCCCATATAATCTTCC
5D-1437570-T10-31_640	CCTCCATGTAGGGAGAGTTTATCCTTCCTGTAAACATTCCCCTAGACTTGGTGCCAAAAAACGTCCATATCCTGTGTGGAGTTTCAGGTGCCCCTTCGTCTGTCGCTGCCGCGAACCGCG
5D-1437570-T10-31_704	CCATATCCTGTGTGGAGTTTCAGGTGCCCCTTCGTCTGTCGCTGCCGCGAACCGCGGCGGGATTGTCGTGACGCCGATGCAGATGACCACTGCCAACGTTGCCACCGATCCGCCATCATT
5D-1437570-T10-31_768	GTCGTGACGCCGATGCAGATGACCACTGCCAACGTTGCCACCGATCCGCCATCATTGTCGTCATTTGGCGGTGCGCTGCTGAGTGCCCCGCCTCCAGCAGTTGGCGTtgttggggaacgt
6B-1664420-T10-32_64	GTGCTCTGGATGCAACAAATTTATGGCGTTTTCTTTACCGCTAACAAAATTATGATGCTGGCTTGTTTGGATCTGTGTTATTTGAAATATATTCACTACGTTCACTGTTGAGAATTGAGA
6B-1664420-T10-32_128	GTTTGGATCTGTGTTATTTGAAATATATTCACTACGTTCACTGTTGAGAATTGAGATTATGAGAGTAAAACTTGACATGTAGCGAACTATTACATATTTGAAGCAAGTTGGATCATGCCA
6B-1664420-T10-32_192	GTAAAACTTGACATGTAGCGAACTATTACATATTTGAAGCAAGTTGGATCATGCCATTTAGTTGCAATAGTTAATTAGGAGTGAAATGTTATGGAATGCAGAAATAGTTCTAAATGCATA
6B-1664420-T10-32_256	CAATAGTTAATTAGGAGTGAAATGTTATGGAATGCAGAAATAGTTCTAAATGCATATTGCTTGATGCATCAATCAGAACTCTTGTCTCTGTTATGGAGTATAGAGAGAGTTTGTAGAAGT
6B-1664420-T10-32_320	TGCATCAATCAGAACTCTTGTCTCTGTTATGGAGTATAGAGAGAGTTTGTAGAAGTCTCCCATAAAGCTACCTAATTACCAGTATTTAGTGCCACTACCACTGTTATCACAGTTTATTTG
6B-1664420-T10-32_384	AAGCTACCTAATTACCAGTATTTAGTGCCACTACCACTGTTATCACAGTTTATTTGAGCACTCGTCCAAACATATTATAGCTACTTAGGTGCTCTGAGATAGCTTATCTACTTGATTAAG
6B-1664420-T10-32_448	TCCAAACATATTATAGCTACTTAGGTGCTCTGAGATAGCTTATCTACTTGATTAAGCTCAAATCAGCGGAATAGGTAACCGGGTTACAATAtactccctccgtcccaaaataagtgactc
6B-1664420-T10-32_640	AATTCTGCGTCAGGACTTCTGTATGTACCTGGTATGTTTGTAATGTTGATCATGTCTTGTGTTATGTGAGATCCTACGTTTAGGCGATTATAAGTGCAAGGTTAGTTACTGTAATATCTT
6B-1664420-T10-32_704	TGTGAGATCCTACGTTTAGGCGATTATAAGTGCAAGGTTAGTTACTGTAATATCTTGATATATTTTGCACATAACCGAATGGCAAGTTTGAATTAAAAAAATGCTTGAAGAAATGCTGAT
6B-1664420-T10-32_768	TTGCACATAACCGAATGGCAAGTTTGAATTAAAAAAATGCTTGAAGAAATGCTGATTACCCTTTTTCTTATATGCCAAAAGTTTCAATAGGAATTTATGTGATGGAATTTTGTTACATAC
6B-1664420-T10-32_832	TTCTTATATGCCAAAAGTTTCAATAGGAATTTATGTGATGGAATTTTGTTACATACCTGTCAGTGACACGGGATACAGGGTCCCCCGAGCTTGCTAAAAGAAGTGGACGGTATAGTATCA
6B-1664420-T10-32_1024	CTGAAATCATGAGTACTATACAAATTTGATACGGAATCACTTGAAGAATACTCGGAGAACATTATAGATGATTCCTCTCTGGCCTATTAAGACCTTTTATGCTTTGTTGATGGAATTCAG
6B-1664420-T10-32_1088	TAGATGATTCCTCTCTGGCCTATTAAGACCTTTTATGCTTTGTTGATGGAATTCAGTGGGCGAACTATGGCTCCTCAGGTTTCCTATTTCATTGTAAATATATACCGTTATCTCACTCTG
6B-1664420-T10-32_1152	CTATGGCTCCTCAGGTTTCCTATTTCATTGTAAATATATACCGTTATCTCACTCTGATCTGTAATGTACAGTCCAACCCAGTGTAGTCAAAAGTGAGGTAATTGTAATGGAAATAATAGA
6B-1664420-T10-32_1216	TGTACAGTCCAACCCAGTGTAGTCAAAAGTGAGGTAATTGTAATGGAAATAATAGAAATTGTGTGCTGCACTTCAAGGCCAACTTTATTTCTCATTTTCATCTTCATTGTCTTGCTTCCA
6B-1664420-T10-32_1280	GCTGCACTTCAAGGCCAACTTTATTTCTCATTTTCATCTTCATTGTCTTGCTTCCAGTTATTGTTTGCAGTGGGAAAAGTACAATTCTTGAGAAATTTTATGTATTGTGGTATCAGTGGT
6B-1664420-T10-32_1344	TTGCAGTGGGAAAAGTACAATTCTTGAGAAATTTTATGTATTGTGGTATCAGTGGTAAATATGTGATGCACAACAAAAACTGATTCATAGTTAAATTTCGGTACTCAAAACTAAAAGATC
6B-1664420-T10-32_1408	GATGCACAACAAAAACTGATTCATAGTTAAATTTCGGTACTCAAAACTAAAAGATCTGGCATTTTATTATGCAAATGCCCTCATGATTACACATTCCATGAAAACAGTGTATGCAGTTAT
6B-1664420-T10-32_1472	TATTATGCAAATGCCCTCATGATTACACATTCCATGAAAACAGTGTATGCAGTTATTTCTGCATTTATTTTGTGATTATTTGCACTAATCATGCATTTTGTGCTTCTTAGCCTCTTAGGA
6D-1733950-T10-32_0	TCATCGCTTGGATTTATGTCAGTAGGCTTCATATGGCTAAGAAATAATTAAAAAAAGGATTAGATGATATCGGCATATTAATACATGGCTATTAATTTGATCTCAAGACTAAAACAGAAT
6D-1733950-T10-32_64	TGATATCGGCATATTAATACATGGCTATTAATTTGATCTCAAGACTAAAACAGAATCCACTGTAACATGTTAAGAAGTGACATAGGTATATAAGGTTCTCATTAAGAGAAAAGAAAGAAT
6D-1733950-T10-32_128	ACATGTTAAGAAGTGACATAGGTATATAAGGTTCTCATTAAGAGAAAAGAAAGAATGTGAAATACACACGACACATGATGTTTTGTAGGCACTCCAGCATCTGTAGATATCCCAAATTGA
6D-1733950-T10-32_192	CACACGACACATGATGTTTTGTAGGCACTCCAGCATCTGTAGATATCCCAAATTGATATGGACAAGAGAAACGTAGCTTACACGAAAGATGAAACATTAAGCATGAAATAGTGAATGCTA
6D-1733950-T10-32_256	AGAGAAACGTAGCTTACACGAAAGATGAAACATTAAGCATGAAATAGTGAATGCTATATCAAGAGTTTATAACTGACCTTTTGGAAAGTACTTGGTCACAAATTGGACATGCACCATCGT
6D-1733950-T10-32_384	ATTTTCTTTGCATCCTCTGTACCTGCACAATAAGTATCAGTTAAAGTAAAAAAAAAATGTATTCTGCAATAGCAGCAGTCCTCATGAACTTTACTTATTTACAATTGAAACGATTAAACA
6D-1733950-T10-32_448	TGCAATAGCAGCAGTCCTCATGAACTTTACTTATTTACAATTGAAACGATTAAACAGAAAGAGATAGAAGATTGCAGGCATACATAAAACATGGCCACAAGTTGTTGTTATGGCTTGCCC
6D-1733950-T10-32_640	ACAGATAAATGACTTGCACTAGATGAACTGATCATAACCATCTAATGTGTCCTGCATTACAGCCCTAAATCCTAAGAGGCTAAGAAGCACAAAATGCATGATTAGTGCAAATAATCACAA
6D-1733950-T10-32_768	CAGAAATAACTGCATACACTGTTTTCATGGAATGTGTAATCATGAGGGCATTTGCATAATAAAATGCCAGATCTTTTAGTTTTGAGTACCGAAATTTAACTATGAATCAGTTTTTGTTGT
6D-1733950-T10-32_832	TGCCAGATCTTTTAGTTTTGAGTACCGAAATTTAACTATGAATCAGTTTTTGTTGTGCATCACATATTTACCACTGATACCACAATACATAAAATTTCTCAAGCATTGTAGTTTTCCCAC
6D-1733950-T10-32_1024	CAATTTCTGTTATTTCCATTACACATTACCTCGCTTTTGACTACACTGGGTTGGACTATACATCACAGATCAGAGATGAGATAAAGGTATATATTTACAATGAAATAGGAAACCTGAGGA
6D-1733950-T10-32_1152	TCGCCCACTGAATTCCATCAACAAAGAAGCATAAAAGGTCTTAATAGGCCAGAAAAGAATCATCTATAATGTTCTCCGAGTATTCTTCAAGTGATTCCGTATCAAATTTGTATAGTACTC
6D-1733950-T10-32_1216	TATAATGTTCTCCGAGTATTCTTCAAGTGATTCCGTATCAAATTTGTATAGTACTCATGAGTTCAGATTTAAACTTGTTAATGTTTAAAACCCTTCTGCTGTGCTCGGGAAAGCCCGAGC
6D-1733950-T10-32_1408	ATCACATAAATTCCTATTGAAACTTTTGGCATATAAGAAAAAGGGTAATCAGCATTTCTTCAAGCATTTTTTTAATTCAAATTTGCCATTCGGTTATGTGCAAAATATATCAAGATATTA
6D-1733950-T10-32_1472	CATTTTTTTAATTCAAATTTGCCATTCGGTTATGTGCAAAATATATCAAGATATTACAGTAACTAACCTTGCACTTATAATCGCCTAAACAGTAGGATCTCACATAACACAAGACATGAT
6D-1733950-T10-32_1536	AACCTTGCACTTATAATCGCCTAAACAGTAGGATCTCACATAACACAAGACATGATCAACATTACAAACATACCAGGTACATACAGAAGTCCTGATGCAGAATTCAGCTAGTATATTGTA
U-2113760A-T10-32_192	CATGGAATGTGTAATCATGAGGGCATTTGCATAATAAAATGCCAGATCTTTTTGTTTTGAGTACCGAAATTTAACTATGAATCAGTTTTTGTTGTGCATCACATATTTACCACTGATACC
U-2113760A-T10-32_256	CGAAATTTAACTATGAATCAGTTTTTGTTGTGCATCACATATTTACCACTGATACCACAATACATAAAATTTCTCAAGCATTGTAGTTTTCCCACTGCAAACAATAACTGGAAGCAAGAC
U-2113760A-T10-32_320	TAAAATTTCTCAAGCATTGTAGTTTTCCCACTGCAAACAATAACTGGAAGCAAGACAATGAAGATGAAAACGAGAAATAAAGTTGGCCTTGAAGTGCAGCACACAATTTCTGTTATTTCC
U-2113760A-T10-32_512	TATATTTACAATGAAATAGGAAACCTGAGGAGCCATAGTTCGCCCACTGAATTCCATCAACAAAGAAGCATAAAAGGTCTTAATAGGCCAGAGAAGAATCATCTATAATGTTCTCCGAGT
U-2113760A-T10-32_576	GAAGCATAAAAGGTCTTAATAGGCCAGAGAAGAATCATCTATAATGTTCTCCGAGTATTCTTTAAGTGATTCCGTATCAAATTTGTATAGTACTCATGAGTTCAGATTTAAACTTGTTAA
U-2113760A-T10-32_640	AGTGATTCCGTATCAAATTTGTATAGTACTCATGAGTTCAGATTTAAACTTGTTAATGTTTAAAACCCTTCTGCTGTGCTCGGGAAAGCCGGAGCACTTTTCCACACGTCTTCTGATACT
U-2113760A-T10-32_832	CCATCACATAAATTCCTATTGAAACTTTTGGCATATAAGAAAAAGGGTAATCAGCATTTCTTCAAGCATTTTTTTAATTCAAATTTGCCATTCGGTTATGTGCAAAATATATCAAGATAT
U-2113760A-T10-32_896	AGCATTTTTTTAATTCAAATTTGCCATTCGGTTATGTGCAAAATATATCAAGATATTACAGTAACTAACCTTGCACTTATAATCGCCTAAACAGTAGGATCTCATATAACACAAGACATG
U-2113760A-T10-32_960	CTAACCTTGCACTTATAATCGCCTAAACAGTAGGATCTCATATAACACAAGACATGATCAACATTACAAACATACCAGGTACATACAGAAGTCCTGATGCAGAATTCATCTAGTATATTG
U-2113760A-T10-32_1152	ATAATATGTTTGGACGAGTGCTCAAATAAACTGTGATAACAGTGGTAGTGCCACTAAATACTGCTAATTAGGTAGCTTTATGGGAGACTTCTACAAACTCTCTCTATACTCCATAACAGA
U-2113760A-T10-32_1216	TAATTAGGTAGCTTTATGGGAGACTTCTACAAACTCTCTCTATACTCCATAACAGAGACAAGAGTTCTGACTGATGCATCAAGCGATATGCATTTAGAACTATTTCTGCATTCCATAACA
U-2113760A-T10-32_1344	CTAATTAACTATTGCAACTAAATGGCATGATCCAACTTGCTTCAAATATAATAGTTCATTACATGTCAAGTTTTACTCTCATAATCTCAATTGTCAACAGTGAACATAGTGAATATATTT
U-2113760A-T10-32_1408	GTCAAGTTTTACTCTCATAATCTCAATTGTCAACAGTGAACATAGTGAATATATTTCAAATTAACACAGATCCAAACAAGCCAGCATAATAATTTTGTTAGCGGTACAGAAAACACCATA
U-2113760A-T10-32_1472	CACAGATCCAAACAAGCCAGCATAATAATTTTGTTAGCGGTACAGAAAACACCATAAATTTGTTGCATCCAGAGCACCAAAAATGCAAGAACAAAGCAGACACGATACACGATGACCCTA
U-2113760A-T10-32_128	CAAAATGCATGATTAGTGCAAATAATCACAAAATAAATGCAGAAATAACTGCATAAACTGTTTTCATGGAATGTGTAATCATGAGGGCATTTGCATAATAAAATGCCAGATCTTTTTGTT
U-2113760A-T10-32_1280	TTCTGACTGATGCATCAAGCGATATGCATTTAGAACTATTTCTGCATTCCATAACATTTCACTCCTAATTAACTATTGCAACTAAATGGCATGATCCAACTTGCTTCAAATATAATAGTT
3A-0645950-T10-34_128	AAATTTGAATTGTTTTTTACCGTGCCGGTCCTTCCTTCCCCCGTTTGTGATAGCTCTTCTGCTTGGATCAAAAAATGTTACTGCAAAGTTTACTCGGCTCACACCAGTCACTGCTAGACT
3A-0645950-T10-34_192	GGATCAAAAAATGTTACTGCAAAGTTTACTCGGCTCACACCAGTCACTGCTAGACTGTTTAATAAATGGTCTGCGAAAGCTCATTATCCATGCGATGTTGATCGCTTTACAAATTGGCAT
3A-0645950-T10-34_256	AATGGTCTGCGAAAGCTCATTATCCATGCGATGTTGATCGCTTTACAAATTGGCATGGGAACAGAGGCTCGGCCAGTAAATGTGATGAATTTATTTTACCACTTTTAGTTGATAAATGTG
3A-0645950-T10-34_320	AGGCTCGGCCAGTAAATGTGATGAATTTATTTTACCACTTTTAGTTGATAAATGTGATGGAATGAACGTGATGAAGTGGATTAATTTCGAACCAAATTGACATTGATTACCTTTTAGGAA
3A-0645950-T10-34_384	AACGTGATGAAGTGGATTAATTTCGAACCAAATTGACATTGATTACCTTTTAGGAATTCAAAAATTTCAAAATACAGACCTACAGATATCTCTTTTACTGTAGAAATCAGGCATTATCTG
3A-0645950-T10-34_448	TTTCAAAATACAGACCTACAGATATCTCTTTTACTGTAGAAATCAGGCATTATCTGGGGGAAAGATGGAAATCAAGCATTATCGGGGGGAAGAGAGGAGGATGAAACGTCTTAAAAATGA
3A-0645950-T10-34_640	aatcaactacaccacCCAGACCCGCTTGACATGGGACACGCCTAAAGTATGTTGTACAGACAGCTGGCATCAATTTCAACCTACGCGAGTTCCACAATCCTTAATCTTTTTCCCGAAAAT
3A-0645950-T10-34_704	TGGCATCAATTTCAACCTACGCGAGTTCCACAATCCTTAATCTTTTTCCCGAAAATACAATCTGTAACCTTGATGCCTTACGTGGATTATATTTAAAATAATATAAAAGTGGTAAAAGTC
3A-0645950-T10-34_768	TAACCTTGATGCCTTACGTGGATTATATTTAAAATAATATAAAAGTGGTAAAAGTCATATTGTAAGGACCATATGAAGAATGCAAGTGTCGAGCTATTCGAAATTGTGCAATGCTTTTGA
3A-0645950-T10-34_832	AGGACCATATGAAGAATGCAAGTGTCGAGCTATTCGAAATTGTGCAATGCTTTTGAAGCTTCCAACGAAAAATTATAGCCTCCTCCCTCTCATGCACTCCCTCCCACTCTCTTTTGATTT
3A-0645950-T10-34_896	ACGAAAAATTATAGCCTCCTCCCTCTCATGCACTCCCTCCCACTCTCTTTTGATTTAATCTCCAGATGTTGTCATTTTCACTTCTGCGGATTCTTTTTATCTATGCTATCAGGTGCTCGA
3A-0645950-T10-34_960	GATGTTGTCATTTTCACTTCTGCGGATTCTTTTTATCTATGCTATCAGGTGCTCGACAAAGAATCAAAGCAAGTAGTAGAGGAAGATTAATCTACGTTTCCAGGTCACATGTGAATACAT
3A-0645950-T10-34_1024	CAAAGCAAGTAGTAGAGGAAGATTAATCTACGTTTCCAGGTCACATGTGAATACATGAAACCAAACATGAGAACAACATTGTTGAAGTATTTTTGCTTTTCCTGATTCTTGCCATTTGAC
3A-0645950-T10-34_1088	ACATGAGAACAACATTGTTGAAGTATTTTTGCTTTTCCTGATTCTTGCCATTTGACATGGGTTCCCGGTTGTAAGTGCATTGTCTCTTAATCACTATTTTGGTGTTGATGGCCATAGGAT
3A-0645950-T10-34_1152	CCGGTTGTAAGTGCATTGTCTCTTAATCACTATTTTGGTGTTGATGGCCATAGGATTAGTAAGACCCAATGTCGTTTTCTAGATTATCTCATGAATCAGGACATTGGACTCTCAGTGATT
3A-0645950-T10-34_1280	ACATCGGTGACCCCGTATTTAAAAAGACGGTGATTTCCGAAGGCTCGGAAGTTTTTAGTTTATTTAGTCGTAGGAAAGTCGCTCTATAAAGAGGTTTTGGAGTGGAGAAATTCATATCAA
3A-0645950-T10-34_1344	TAGTCGTAGGAAAGTCGCTCTATAAAGAGGTTTTGGAGTGGAGAAATTCATATCAATCATATACATTGCCTTCCTCACTAGCTTTTGTCGAAATAATGGTAAAACCAAGGTTCAAAGTTC
3A-0645950-T10-34_1408	ATTGCCTTCCTCACTAGCTTTTGTCGAAATAATGGTAAAACCAAGGTTCAAAGTTCCTATGCCAGAAGGTCCGAGTGCCCGGAGTAACCCGAGAAGAAGCATGCACATTTCGCTAACCCA
3A-0645950-T10-34_1472	GAAGGTCCGAGTGCCCGGAGTAACCCGAGAAGAAGCATGCACATTTCGCTAACCCAAGTTCCCGAGGTCCTACCCCCCCCCCAGTGCTTGGAGGGAGGGAAACCTCTGTGCATACTCC[illegible]
3A-0645950-T10-34_1580	TGCATACTCCGGGTTGCTGGAGTCCCTTTACTACAGGTGCTCGATCTCCTGTGGTTGGTTCACCTTTTATGTATTCTGGATGCCCGAGCATTAATCTAGATCCATAGAGTCCTAGGGTAA
3B-0809860-T10-34_128	GAATTGTTTTTACCGTGCCGGTCCTTCCTTTCCCCCttttttttttcttttttttGTGAATTCGGTCCTTCCTTCCCTCGTGGCCCCGTTTTGTACGAACTGCTACACTGCCAAGTTCAT
3B-0809860-T10-34_192	GTCCTTCCTTCCCTCGTGGCCCCGTTTTGTACGAACTGCTACACTGCCAAGTTCATTATCCATGCGATGTTAATCATTTCTACAAATTGGCAAGTGAACAGAGGCTCGGCCAATTTGGAT
3B-0809860-T10-34_256	CGATGTTAATCATTTCTACAAATTGGCAAGTGAACAGAGGCTCGGCCAATTTGGATTGGATGGAGTATGTGTTAGCTTTTGTGTTTTTTTTAGTTTGATACGTAGTACTAAGTGTGATGA
3B-0809860-T10-34_320	GTATGTGTTAGCTTTTGTGTTTTTTTTAGTTTGATACGTAGTACTAAGTGTGATGAAATTGGCTGAATGTGTTGAAGTGGATTGATTTCGAACCAAATTAACATTGATTACCTTTTAGGA
3B-0809860-T10-34_384	GAATGTGTTGAAGTGGATTGATTTCGAACCAAATTAACATTGATTACCTTTTAGGAATTCAATGTTCAGAATGGACCTACATGATGACTGCTAAAGGAGAGAGAGGGGTACTAGGAAGAA
3B-0809860-T10-34_448	TTCAGAATGGACCTACATGATGACTGCTAAAGGAGAGAGAGGGGTACTAGGAAGAACAGAAAGCCGCTAGAAATTGGTAATCAAGCATATGCAGAAAAAaaaaaagttgtctaagcccag
3B-0809860-T10-34_640	CAGGCCCGCCATAAACTATTTGTACGAGTTTTAAATTTGGTAACAATCTTAATACTCCATCCAATCCAAATTAATTGACGCACGTTTAGTACAACTTGTTTTTTATCAGAGGGAGTACAC
3B-0809860-T10-34_704	TCCAAATTAATTGACGCACGTTTAGTACAACTTGTTTTTTATCAGAGGGAGTACACCTTATTACTCGGGTTATATTTAATAACAAAATATAAAGCAGTAAAAAAAAGTAAACTAGGAACA
3B-0809860-T10-34_768	TCGGGTTATATTTAATAACAAAATATAAAGCAGTAAAAAAAAGTAAACTAGGAACAGGCCAGCTGCACAAGAGTAAAGATCATATAAAAATGCAAGTGTTCAGCTATTCAAAACTACGCA
3B-0809860-T10-34_832	GCACAAGAGTAAAGATCATATAAAAATGCAAGTGTTCAGCTATTCAAAACTACGCAATGCTTTTGAAGCATCCATAAAAATATATTAAAAAATAAAGAAGTTTAAAGCTTTTGAAGCATC
3B-0809860-T10-34_896	GAAGCATCCATAAAAATATATTAAAAAATAAAGAAGTTTAAAGCTTTTGAAGCATCCATAAAAAAATATTAAAAAATAATGAAATTTAACTATTTGAAATGAATGGGGCGTAACGTAAAA
3B-0809860-T10-34_960	AATATTAAAAAATAATGAAATTTAACTATTTGAAATGAATGGGGCGTAACGTAAAATGTTCATGCAAGTCAAGTATTGCATATCTAGTTTTGCTGATTTCGCACGCATTCCAAAGTTAGA
3B-0809860-T10-34_1024	CAAGTCAAGTATTGCATATCTAGTTTTGCTGATTTCGCACGCATTCCAAAGTTAGAGAGCTAAGTAAAATGAAGCCTCCATATCCACTAGCTTTTGGGGCAGTTCGATCAATTGGTCGTC
3B-0809860-T10-34_1088	TAAAATGAAGCCTCCATATCCACTAGCTTTTGGGGCAGTTCGATCAATTGGTCGTCCGATCTCGACCCTTAGCAACCGTGATCTAGACCCACGCGAGGTTGGTGAGGTGGACGGCGTATG
3B-0809860-T10-34_1152	ACCCTTAGCAACCGTGATCTAGACCCACGCGAGGTTGGTGAGGTGGACGGCGTATGGCTGCGGGCGGTGGTACTCCTGAGAGAGCTTGGAGATCTTCATGGCGACGGAGGTTAGGGT[illegible]
3B-0809860-T10-34_1216	CGGTGGTACTCCTGAGAGAGCTTGGAGATCTTCATGGCGACGGAGGTTAGGGTTCCAGCGATTGGGATTGGATGGATTTGGGCCGGAAGAAGTGAGGCGAGGCGTCGAGAACGAAG[illegible]
3B-0809860-T10-34_1280	GGATTGGATGGATTTGGGCCGGAAGAAGTGAGGCGAGGCGTCGAGAACGAAGGTGAAGAGGGAAGGATTTGAGAACGGGAGGAAGAAGGGCCTGAAATTTGAATTTTCATGACTC[illegible]
3B-0809860-T10-34_1344	GGATTTGAGAACGGGAGGAAGAAGGGCCTGAAATTTGAATTTTCATGACTCTTATATTGCCGGTCCGTCCTTCCTTCGTCCCAATTAAGGAAATTGGCAAGTTGGATTTCTCTTTTTTTC
3B-0809860-T10-34_1408	CCGTCCTTCCTTCGTCCCAATTAAGGAAATTGGCAAGTTGGATTTCTCTTTTTTTCTTGACAAAACATAACTGGGGACTAGATTGTGATGGATTATTAGTGCTAGGGAAAAAGGAGATGC
3B-0809860-T10-34_1472	ACATAACTGGGGACTAGATTGTGATGGATTATTAGTGCTAGGGAAAAAGGAGATGCCCACACACGCAGGTTGTATGCATGTACACATACAGGAAGCGCAATCAGCCCATAGCTACTTGAA
3B-0809860-T10-34_1536	GCAGGTTGTATGCATGTACACATACAGGAAGCGCAATCAGCCCATAGCTACTTGAATCGCTCCACGACTTCATCTATATTGATGATCGCATTTTTTTGGAATTATTTTAGCCTTTTCGAC
3D-0884100-T10-34_64	GGGCGTGGGGAACGGGGAATTGGGGGGAGGGTTCGGGAACGGGAGGAGGAAGCGCACGAAATTTGAATTGCTTTTACCGTGCCCCCGTTTGTGACAGCTCTTCTGCTTGTATTGGAT
3D-0884100-T10-34_128	GAATTGCTTTTACCGTGCCCCCGTTTGTGACAGCTCTTCTGCTTGTATTGGATCAAATGTACTGCATGATTTGCTGCTATTTTTTAAAATAATACATTTTTTTATTAATTTTCATAAATA
3D-0884100-T10-34_1536	TTATGAAAATTAATTAAAAAATGTATTATTTAAAAAAAATAGCTGATTTGCTCGGCTCACACCGGTCGCTGCTAGACTGTTTAAATGGTCTGCGAAAGCCCATTATCCATGCGATGTTGA
3D-0884100-T10-34_1580	GATTTGCTCGGCTCACACCGGTCGCTGCTAGACTGTTTAAATGGTCTGCGAAAGCCCATTATCCATGCGATGTTGATCGCTTTACAAAATGGCATGTGAACAGAGGTTCGGCCAGTAAAT
4B-1063200-T10-35_64	gTTTCGGGCTTTCGGCCCTTTTCGAATTTTTTTGGTTCGGAGGGGATTCACCCGCTCGAGGGGGGTGGAAATTACTACTAGCAGCGTTGGGCGTTTTTCTAAGCGGGCTGGGCTGTGAA[illegible]
4B-1063200-T10-35_192	CCCATGGGGGGTTTTCATTTAGTCCAGTGAGCTTGTCTAAAAATAAATAGTCCAGTGAGCAATTTGCGTGCCTGCACGCACCACGCAATCAATCCGAATTTATTTCCCTAAAAAAATGCA
4B-1063200-T10-35_256	TGCGTGCCTGCACGCACCACGCAATCAATCCGAATTTATTTCCCTAAAAAAATGCAATCCGAATTTATTTATTTTTGAGCTGAATCCGAATTTATTTGCTTTGGTTGAGTTGCAGTCAAC
4B-1063200-T10-35_320	TTATTTATTTTTGAGCTGAATCCGAATTTATTTGCTTTGGTTGAGTTGCAGTCAACCTGAGATTTTGCTGCCCTATAAAAACAGTGTTCTCGCTATTTCTTTATTTATTGCAAGAAGTGT
4B-1063200-T10-35_384	TTGCTGCCCTATAAAAACAGTGTTCTCGCTATTTCTTTATTTATTGCAAGAAGTGTTGTCTCCAATTTAAGGATTTGTTTTCTCCACCTAAAGAGGAAACATGAGTGGTTTTTTCAGAGG
4B-1063200-T10-35_448	ATTTAAGGATTTGTTTTCTCCACCTAAAGAGGAAACATGAGTGGTTTTTTCAGAGGCATACATTCCAGAAGAGAATTTTGCTACACTTCTCATGATGCATTTGCGACAAGTACAAGAGAT
4B-1063200-T10-35_512	CCAGAAGAGAATTTTGCTACACTTCTCATGATGCATTTGCGACAAGTACAAGAGATCTTTGATATCAATTCATCGAACCTTGTAACAGCACGAAATCCGCTGCTGCTTTGGACATGCAAA
4B-1063200-T10-35_576	TCAATTCATCGAACCTTGTAACAGCACGAAATCCGCTGCTGCTTTGGACATGCAAAAAACATGTTTCAATTTTACGAATACGAACTTGGCAAACACATGATGTAAGGGTCTTCGTAAACT
4B-1063200-T10-35_640	TTCAATTTTACGAATACGAACTTGGCAAACACATGATGTAAGGGTCTTCGTAAACTGAGACTACGATCTTCTTTTATCAATTCTAATTGTGCTCAGCAGGTAAGAAGCTTGTTCCGTTTG
4B-1063200-T10-35_704	GATCTTCTTTTATCAATTCTAATTGTGCTCAGCAGGTAAGAAGCTTGTTCCGTTTGTACGCACCCTTCCACCTCCAGCTCCAGGTCTCCGCACATCAGTTTATCACCTCTGTTTCTCTGT
4B-1063200-T10-35_768	CTTCCACCTCCAGCTCCAGGTCTCCGCACATCAGTTTATCACCTCTGTTTCTCTGTTGAGATGGTGGAGTGGAGACAAATGATTTGTGTCCAAAGTGCTAAATCTTCATGGATAATCCAT
4B-1063200-T10-35_832	TGGAGTGGAGACAAATGATTTGTGTCCAAAGTGCTAAATCTTCATGGATAATCCATACAAACACGGCCACGGCAGAGAAAGTCGGTCCTCATAACTTCGAATTTTTGTCCAAAAGAACCA
4D-1122780-T10-35_1580	GTTCGGAGGGGATCCACCGCTCGAGGGGGGAAATTACGGGGAGAACTGCTACACGTACGACGAGTTTGTATGATGTTGTGTATAACAGCCCTCAGCAGCCGTTCGATCAACTGGATGGA
7A-1751550-T10-36_512	TCACCTGGAGCTCTCAGAAGTAGAAGATAGTTGCGCTTTCTTCATGTGAGGCAGAATATGTGGCCATTGCAGCAGCGACCTGCCAAGGGTTGTGGTTGAGCAGACTGTTGGGAGATATA[illegible]

7A-1751550-T10-36_640	GCTCCTGCGAGGTTCAAGCTCCAAGTGGACAACAAATCTGCTATAGCTTTAAGAAAAAATCCAGTACACCATGATCACAGCAAACACATTGACGTGAAGTTCCATTTTATGAGAGACTGC
7A-1751550-T10-36_704	TACACCATGATCACAGCAAACACATTGACGTGAAGTTCCATTTTATGAGAGACTGCATGGAAAAGGATCAAGTGAAGATCGATCACGTGGGCACGCAAGATCAACTGGCCGATGCTCTGA
7A-1751550-T10-36_896	ATTAGGGGGTGAAATGTTATGCTTAGTCTAATCTCATGCTAGCAACGTAGTTCTGTTTTTCTTTCTGTTTGTAATAATGTGTGCATGCGAGATGATGTGACCCGGACATGCACGTAGCTA
7A-1751550-T10-36_1152	GTAGCTAGCTAGGTTGTTGCAACCGATATAGAGAGGAATGGAAAACAGAAAAGCTGCGCGGTTGACGGCGGCACAAAACTCGCTCTCTATCTGTCGTTCAAGTTCATAGCTCTCGCTCTT
7A-1751550-T10-36_1216	ACGGCGGCACAAAACTCGCTCTCTATCTGTCGTTCAAGTTCATAGCTCTCGCTCTTTCTTTCTCTGCTCGATCGCGTGTGTTGGCAACGACATCCTTCACATTTTGGAGGAAAAAAACAT
7A-1751550-T10-36_1472	AGGCCTCATCATTTTTAGGCGGTGGAGATTTTTCTTTGTCACAAAGAGGGAATGTTTCCGCGGAATATTGCGTAGTCCATTGAATCGGTGGGCCTAAATGTGAGCAGAAAACGGCCCTGG
7D-2059370-T10-36_128	ACATTTAGGCCCACCGATTCAATGGACTACGCAATATTCCGCGGAAACATTCCCTCTTTGTGACAAAGAAAAATCTCCACCGCCTAAAAATGATGAGTTTAGATTACAAAATTAGATAAA
7D-2059370-T10-36_192	AAAGAAAAATCTCCACCGCCTAAAAATGATGAGTTTAGATTACAAAATTAGATAAACGTTACATCAGCCAATATGTGCTGATAAAAGACACAAGTACAAGGAGACTGAAATAACATTTAC
7D-2059370-T10-36_256	CAGCCAATATGTGCTGATAAAAGACACAAGTACAAGGAGACTGAAATAACATTTACAGGCAGTGCAACATGATAAATGCAATATTTTCAATAGAATTTTCATTATTTTCAACGGCCACAT
7D-2059370-T10-36_320	CAACATGATAAATGCAATATTTTCAATAGAATTTTCATTATTTTCAACGGCCACATGAGCAACAGGTGCCTCTTGCTGCATCTGGAGGACTACAGCTCGAGGCAAGCTAGGAAGCGGTAT
7D-2059370-T10-36_448	CTTATTCATTAACAGGCATGATTTTCACTTAGCCAATCAATTCATCGTGTGGGTAAGCCAACATAATATTAAGCATACCAACTCCAAGATTATGAGCATCATTAGGAGAAAAACATGTAA
7D-2059370-T10-36_512	AATATTAAGCATACCAACTCCAAGATTATGAGCATCATTAGGAGAAAAACATGTAAGTTAACAGGTACATTGTGACCCGTCTGGTTAACCTCTTCACTCAGTGGGTTGCCTCCAAATTGA
7D-2059370-T10-36_576	GTACATTGTGACCCGTCTGGTTAACCTCTTCACTCAGTGGGTTGCCTCCAAATTGAATATTGTTTGAAATTGTCCCATCCAACAAACAAACAGTAGTCGTAATACTATTTTTTTTCATCC
7D-2059370-T10-36_640	TGAAATTGTCCCATCCAACAAACAAACAGTAGTCGTAATACTATTTTTTTTCATCCTGAATATGAAGAGCCTCCTCTTCACCAACTTACCGCAGGTTCTCATTCGCAGTCATTATCAAGT
7D-2059370-T10-36_704	AAGAGCCTCCTCTTCACCAACTTACCGCAGGTTCTCATTCGCAGTCATTATCAAGTGTCCATTATTATTGGATTGGCTGTTGAAAAGCCCAATGAGCATCTTCATTTGTCATCCATGTCG
7D-2059370-T10-36_768	TTATTGGATTGGCTGTTGAAAAGCCCAATGAGCATCTTCATTTGTCATCCATGTCGGCATCCTCTTGGATATGGTCATGCCCATGTTGTGGCCCTAGAGGGTTCAAACCAGGAAAATCAA
7D-2059370-T10-36_896	TGGAGCATTGGGTGTGTGTCAACCCTAGGAAGGGGATTGGTCTTTATAGGGGGGCGCATGGTggggattgttgcatggaaaaaaaagttctacaaaacactcaagatatattctatggag
7D-2059370-T10-36_1024	aacgagagggagaggctacatacccttgtagaccgaaagtgtcacatgtacggctcggtcatacacttcataatgaccttggcatagccgtactcccttcgtgatccaatccaatctagc
7D-2059370-T10-36_1280	agtttctgcatggcttcaccaagcgttggggtgaagggaagtgaaaggagagggagggcggcggctagcaatgggatgggtggtgcgtcagcctctctcctcccttttatatagggggtg
7D-2059370-T10-36_1344	ctagcaatgggatgggtggtgcgtcagcctctctcctcccttttatatagggggtgaggggtgtggccagctctggccacccctccaagccaaggggtgGATGCCCTCTCCCCAGCcgca
7D-2059370-T10-36_1472	aggcggagtggcgcctggcccatctgggctggtgtgccctccctagggtccatgaggactctccgaggcaggtggacccttgaaacctttcaggactcccgatacaatgtcggcaacccc
1A-0049590-T10-37_0	GCCACCCTTTTTGGCTACTTGATCACCTGAGGATAACCACAATGTCAACTTATATGTTAAAAGTAATAATGACAGTGACAGCTCAAGAGCAAAATTGAGGATTACCATCAAGTGTGATGC
1A-0049590-T10-37_192	CCTAACAGAAAATTCTAAGTTAAGCAGGAAAAAGAACCATATGAAAGCCAAGAAGTACACTTTTGAACGAAAAGTTATCAGAAAAAACTAGCTTAGGTGCTTGAGGAAGGGAAGCTACA
1A-0049590-T10-37_384	TAAAAACATGCATAAATGGGAACGTGGGAGTGAGTAGTGAACACCTTATTTATATTTTTAATGTCTCCAACATGGCATCCAATTGGAAGCTGAAGATAAATTAGAATGAAATCTTCTGTA
1A-0049590-T10-37_448	CTCCAACATGGCATCCAATTGGAAGCTGAAGATAAATTAGAATGAAATCTTCTGTATCCAAACAAGGATATACTATAAAATAATCTGCGATGTCACTCATAACTCAAGTGATTTTGCTGG
1A-0049590-T10-37_512	AGGATATACTATAAAATAATCTGCGATGTCACTCATAACTCAAGTGATTTTGCTGGGTGTCAACGCTCCAAGCAAACAAAGCTAAGATGGATACATGCAACAGAAGAGATGATAACGTGA
1A-0049590-T10-37_576	GCTCCAAGCAAACAAAGCTAAGATGGATACATGCAACAGAAGAGATGATAACGTGAAGGCAGCATTGGTATTTGGTAATGCAGATGAAGGGTAAGAAGATAGATCAGAACTGAGCCTGA
1A-0049590-T10-37_640	TTGGTATTTGGTAATGCAGATGAAGGGTAAGAAGATAGATCAGAACTGAGCCTGAACTAAATTAACCATGGCAGCCCAATAAAAGATGTAGACATCCATTGGAAGGAAAAAATAACAGGA
1A-0049590-T10-37_832	AAAATCAGAGCTCTGTGGAACATTGACATTCGGAGCGTGCACTCTATTCAGTGGTATGAAAGTCACCCGTCCACCTTTTTCTCGAGTCAATACCTGAATTATCCTTGTCGATATATCGTC
1A-0049590-T10-37_896	ACCCGTCCACCTTTTTCTCGAGTCAATACCTGAATTATCCTTGTCGATATATCGTCATTTTCAACCACCACATGGAACAAGCTATTCACATCAGAAAATATGGGTTATTCAATAACAGAG
1A-0049590-T10-37_960	CCACCACATGGAACAAGCTATTCACATCAGAAAATATGGGTTATTCAATAACAGAGAAGATGAAATGAAAGTGAAGGATCAAGACAAAAAAGAAACAGAACAAGACCTATTCCCAGCAG
1A-0049590-T10-37_1152	ACCATGGTCCCTGATGATTCGGCTAACAGAATTCAGGCCTCTCCTGATATCCTGAGGAAGAAAACAAAATTAACACCATCCAAAGATAGCTTTAAGCCGTTCTTAAAGTAAAAAGGAATG
1A-0049590-T10-37_1216	CAAAATTAACACCATCCAAAGATAGCTTTAAGCCGTTCTTAAAGTAAAAAGGAATGCCATAGTATGACAAAATTGGCATAGCTCAATATTTAGAAGGAGAAGGTCTAGCAATTGCAAAAC
1A-0049590-T10-37_1344	GGGCTATGCTATATACTCCAAAATTCATGAAATCTGTTGCGACAATAAACACAAAGTCAGGTACGACTGATGTAAACCCATGATAAGATATTTTTGAAGTTGTTAAATGTATATAACAAT
1A-0049590-T10-37_1408	GACTGATGTAAACCCATGATAAGATATTTTTGAAGTTGTTAAATGTATATAACAATACACCTAAGCCATTTGATTTACAACTATCGTTGCTCTTCCCTCCAACTCATCGAATTATTCTGC
1A-0049590-T10-37_1472	GCCATTTGATTTACAACTATCGTTGCTCTTCCCTCCAACTCATCGAATTATTCTGCCCCTTACCAGTAACCACAATTTCATACTAACGATGAGGAAATGATACCAAGACCGTAAATGATG
1B-0119800-T10-37_128	CTAAGGTGGTGTTCTGGTTTGAAGCAGCAACCGCGACCAAAATTAATGCAGATGGATTACAGTGGTCCGGTTTTCCAGTAGAATTGAGCATAGCAACTAAGGTCATTAGTCAGGATTTCA
1B-0119800-T10-37_192	GTCCGGTTTTCCAGTAGAATTGAGCATAGCAACTAAGGTCATTAGTCAGGATTTCAGAAACCAATAGCTGAACGAAACAGTCGCAAAATGCCTGACAGACCCGTCAGACAGCGCACCGC
1B-0119800-T10-37_320	TTTCCAGGCACGGATAGATCAATCCGACAGACCAAAGGGCTGCCAGCAACTATACATGACTGCGCGGACAAATAACTCGTGCAGGGTAAGGAAGCAGCAGCCCGAAGAGCGCGTCCTA
1B-0119800-T10-37_384	CGGACAAATAACTCGTGCAGGGTAAGGAAGCAGCAGCCCGAAGAGCGCGTCCTACTGCACTACACTACGCCACACACCCGCGTGTAAACCACTCCAGGATCCAAGGAATTATCCACCGC
1B-0119800-T10-37_1024	GCCACAGCCAGGCCCAACGTGCACCCGGTTCCTCCCGTGCTCGTCTGGTCTGGGTCTGGTTCGACCATTCCGGTCCAGGCTCCCGGTTCCATTCGAATCACCTCTGTTTATTGAACTTCA
1B-0119800-T10-37_1088	CCATTCCGGTCCAGGCTCCCGGTTCCATTCGAATCACCTCTGTTTATTGAACTTCATATATCAGATCTTTTATTATTTTTTTGAAGTGAATCCACGGGTGATTCGAATCCAACCAAAGCT
1B-0119800-T10-37_1152	ATCTTTTATTATTTTTTTGAAGTGAATCCACGGGTGATTCGAATCCAACCAAAGCTGAAATAAACAGGGATGAATCTAGGATCTGAAGCAAACAACTTTTTTGTGTGAGCTTCGATAGCC
1B-0119800-T10-37_1216	CAGGGATGAATCTAGGATCTGAAGCAAACAACTTTTTTGTGTGAGCTTCGATAGCCATTTCGTTTTAAATTTAGGTATCAGATTCAACTTTGCCAGTAAAGCTGGATGTAGAAGCTAAAA
1B-0119800-T10-37_1280	TTAAATTTAGGTATCAGATTCAACTTTGCCAGTAAAGCTGGATGTAGAAGCTAAAACAAATACTAGCTATTTCGAATAAGCTTTGTCAGTGAAGCAATGTCTGGATTCGGGCCATTCTCA
1B-0119800-T10-37_1472	AAGCGAATCCATATGTAATTTGAATCCAATCAAAGCTGACACAAACAAGACTGCATCATCAACTTTGCGAGTAAAGAGAATCCATAGGTGTTTCGAATACAATCAAAGATGAAACAAACA
1B-0119800-T10-37_1536	TTGCGAGTAAAGAGAATCCATAGGTGTTTCGAATACAATCAAAGATGAAACAAACATGCCCCTCCTCTGATAAATGAAACATTGGTTCCAGATTCAAATTTGAACCGCGTGCTCGAGGAC
1B-0119800-T10-37_1580	ATGAAACAAACATGCCCCTCCTCTGATAAATGAAACATTGGTTCCAGATTCAAATTTGAACCGCGTGCTCGAGGACGAAATTGAAAGGATAAGGTAGCAGTACGATGCTGGTCCGCCGTG
1D-0184520-T10-37_128	CTAAGGTGGTGTTCTGGTTTGAAGCTGCAACCGCGACCAAAATTAATGCGGATGGATTACAGTGGTCCGGTTTTTCTGGTAGAATTGAGCATAGCAACTAAGGTTTTCCAAGTTTTCCTG
1D-0184520-T10-37_192	GTCCGGTTTTTCTGGTAGAATTGAGCATAGCAACTAAGGTTTTCCAAGTTTTCCTGGTCCGGCTCGAACGAATTAGGGGTTGTCATTAGTCAGCATCTCAGAAACCAAAAGCTGAACGAA
1D-0184520-T10-37_256	CGAACGAATTAGGGGTTGTCATTAGTCAGCATCTCAGAAACCAAAAGCTGAACGAAACAGTCGCAAAATGCCACACAGACCCGTCAGACAGCACACCGCTGCCTCCGATTTCCAGGCAC
1D-0184520-T10-37_384	AATCCAACAGATCAAAGGGCGGCCAGCAACTATACATGACTGCGCCAAGAAATAACTCGTGCAGGGTAAGGAAGCAACAACCCGAAGAGCGCATCCTACTGCACTACACTACACCACAC
1D-0184520-T10-37_448	GGTAAGGAAGCAACAACCCGAAGAGCGCATCCTACTGCACTACACTACACCACACACCCGCGTGTAAACCACTCGTCTGAGAACCCAGGAATTATCCACCGCGAAggagcccggagccgc
1D-0184520-T10-37_1024	CTCTGCTCCGAAACGAGACCAGAGGAGGAGAGGGCCCAACGTGCACCCGATTCCTCCCGTGCTGGTCTGGTCTGGTTCGACCATTCCGGTCCAGGCTCCCCGTGCCACTCGTCTCCGGC
1D-0184520-T10-37_1088	GTCTGGTCTGGTTCGACCATTCCGGTCCAGGCTCCCCGTGCCACTCGTCTCCGGCCCTCTGTTTGTTGAGATTCAGATATCAGTTTTTTCTTATTTATTTTTTAAAAGTGAAACCACAAG
1D-0184520-T10-37_1152	GTTGAGATTCAGATATCAGTTTTTTCTTATTTATTTTTTAAAAGTGAAACCACAAGTGATTCGAATCCAATCAAAGCCGAAATAAAACAGGGTCGAATCTAGATTCTAGAATCTGAAGCA
1D-0184520-T10-37_1216	ATCCAATCAAAGCCGAAATAAAACAGGGTCGAATCTAGATTCTAGAATCTGAAGCAAACAACTATTTTGTGTGAGCTTCGACAGTCATTTCATTTTAGATTTAGGTATCAGATTTAACTT
1D-0184520-T10-37_1280	TTTTGTGTGAGCTTCGACAGTCATTTCATTTTAGATTTAGGTATCAGATTTAACTTTTGCCAGTGAAACTGAATGTAGAAGTTAAAGCAAACAAGCAATATTTGGATTCAGGGCATTCTC
1D-0184520-T10-37_1344	GAAACTGAATGTAGAAGTTAAAGCAAACAAGCAATATTTGGATTCAGGGCATTCTCACTGCTATTGCATGAATCATCTCAAAGTGTACTTCAGTGACGAAACTGATTCCGTGAATTGGCT
1D-0184520-T10-37_1472	GAAGTGAATCCATATGTGATTTCAATCCAATCAAACCTAAAACAAACTAGACCGCATAAATCAACTCTGCCAGTAAAGAGAATCCATAGGTGTTTCGAATCCAATCAAAAACGAAGCAAA
5A-1214560-T10-38_64	TgcggcggcggaggtggcggcggcgaaggccgggagggcggggcggAGATGAGATTGGGGCGGGATTGGATTGGGGGGAAGTTATCTTGCTGGGATGGGGCGGCGGGAAGATGAAATGGA
5A-1214560-T10-38_128	ATTGGATTGGGGGGAAGTTATCTTGCTGGGATGGGGCGGCGGGAAGATGAAATGGAGGGCGGGATCATTTTGGGAGGAAAGTTTCAATTTTTGGCGGGATCATTTTGGGTGGAGGGT
5A-1214560-T10-38_192	TCATTTTGGGAGGAAAGTTTCAATTTTTGGCGGGATCATTTTGGGTGGAGGGTGTGGGGTGGGGGTCAATTTTTGGCGGGATTATTTTGCGGGAAGTTTCTTTTTCTTGTGCTGCTAAAC
5A-1214560-T10-38_256	GTCAATTTTTGGCGGGATTATTTTGCGGGAAGTTTCTTTTTCTTGTGCTGCTAAAGTTTCGTCTTTCCCTTAAAAAAATCTCGCCTTTTTCATCGGACGGCCACACCAATTTCCTCCTCG
5A-1214560-T10-38_320	TTCCCTTAAAAAAATCTCGCCTTTTTCATCGGACGGCCACACCAATTTCCTCCTCGCACGCCCCATTTTTTCCAGCCAAAGAAAAAATACAAAAAGAACCTTCTCCTTCCGTAAAAAGTA
5A-1214560-T10-38_384	ATTTTTTCCAGCCAAAGAAAAAATACAAAAAGAACCTTCTCCTTCCGTAAAAAGTACCCACGGGGTCAAAGCTGGCCAAACGGGCTGGGCCTGGCATTGCCGGGCCGCGAGCACGCTG
5A-1214560-T10-38_576	GCCTGAGCCTGATGGCTGGGCATGCGGGCCGGCATGGCACGCCATGTTTACCTTTTTTATTTATATTTTTTGTATATAAATGGCATAAGAGCATATCCAACGCGGGGCGCTAAGGCAAGG
5A-1214560-T10-38_640	ATTTTTTGTATATAAATGGCATAAGAGCATATCCAACGCGGGGCGCTAAGGCAAGGCGCCAGGATTGTTATCCTGGCCGATCGGCAGTTAAGCGCTCAAATCCTGGCGCTCGACGCGGCA
5A-1214560-T10-38_832	GGTTATTTCAGCGCCCGTGATTAGCATTTAGGATTGGAGCAGTAGGCGCTAAGGTTAGCTTTTAATTCTAGGACATTTATTTTATTTTCCTAAGCGCCTCTCCAGGCGCCCTGCATTGAA
5A-1214560-T10-38_960	AAGAAACAACCTAATAGGCCAAAATTGGCCCAAATAACAGCCTAACATGCCAACAATCTAGCAGATCAGCGTGCCTGGCtgttgtggaacgttgcagaaaacaaaaaaaattctacggtt
5B-1363750-T10-38_443	AGGGCGGGGGGGAGATGAGATGGGGGCGGGATTGGATTGGGGGGAAGTTATCTTGCTGGGATGGGGGGCGGCGGGAAGATCAAATGGAGGGCGGGATCATTTTGGGAGGAAAGT
5B-1363750-T10-38_507	GGGGCGGCGGGAAGATCAAATGGAGGGCGGGATCATTTTGGGAGGAAAGTTTCAATTTTGGCGGGATCATTTTGGGTGGAGGGTGGGGGGTGGGGGGTCAATTTTGGCGGGATTAT
5B-1363750-T10-38_571	GATCATTTTGGGTGGAGGGTGGGGGGTGGGGGGTCAATTTTGGCGGGATTATTTTGCGGGAAGTTTCTTTTTCTGTGCTGCTAAGGTTTCGTCTTTCCCTTCAAAAAAATATCTCGCCTT
5B-1363750-T10-38_763	GAACCGTGCTTTCTGAGCGCTCCTCTTCGGCGCCCCTTAAGCCATCTGGCGCTCGAAGGCAAGAAGTAATGGGCCGGCCCAGCATCGCTCGGTAAAAAAAAATGGGGACAGAGCGGTA
5B-1363750-T10-38_827	AGTAATGGGCCGGCCCAGCATCGCTCGGTAAAAAAAAATGGGGACAGAGCGGTAGTATTTTGCGAGTAGTGGGAATTGAACAAGCATGTGTACGCTAAAGCAGTTGCCGCAATAACCAC
5B-1363750-T10-38_891	AGTAGTGGGAATTGAACAAGCATGTGTACGCTAAAGCAGTTGCCGCAATAACCACTGCACCTCACTAGCTTAGATGTTATCAATGCGAAAGAGAACACAATTAGACCCTTGTTTCAGTCC
5B-1363750-T10-38_955	CTAGCTTAGATGTTATCAATGCGAAAGAGAACACAATTAGACCCTTGTTTCAGTCCTAGTATAATTTCTTTGAAAAAAAATTGAAATATAAAAATGATTTCATGAATTTGAAAAAAGTTCA
5B-1363750-T10-38_1275	TTTCACGGGTTTGAAAAAGTTCATCAATTTCGATAAAAAAGTTCACTAATTTGAAAAAGTTCACGAATTCAAAAACCTTATCATTTTTTAATAAAAAAGTTCATGAACTAGGTTTAAAAA
5B-1363750-T10-38_1339	GAATTCAAAAACCTTATCATTTTTTAATAAAAAAGTTCATGAACTAGGTTTAAAAAAATATATGCATTTAAGAAAAAGaaaggaaaaaggaacaagaaaaaaaagggaaaaagagatgat
5B-1363750-T10-38_1403	CATTTAAGAAAAAGaaaggaaaaaggaacaagaaaaaaaagggaaaaagagatgatgcactaaaaggataaaaaggaTCTCACAAATCAAAGCGAAGTAGTTGCGTAAATGGCTACCGCA
5B-1363750-T10-38_1467	aggataaaaaggaTCTCACAAATCAAAGCGAAGTAGTTGCGTAAATGGCTACCGCAGCCAAGTTAAATAGAGGAGGTCACGAGTTCGATTGTCACCTGTGCGCAGTATTTTGCTATTTGA
5D-2138150C-T10-38_411	AAGTTATCTTGCTGGGATGGGGGGCGGCGGGAAGATGAAATGGAGGGCGGGATCATTTTGGGAGGAAAGTTTCAATTTTGGCGGGATCATTTTGGGTGGAGGGTGGGGGGTGGGGG
5D-2138150C-T10-38_476	AAAGTTTCAATTTTGGCGGGATCATTTTGGGTGGAGGGTGGGGGGTGGGGGATCAATTTTGGCGGGATTATTTTGCGGGAAGTTTCTTTTTCTGTGCTGCTAAGGTTTCGTCTTTCCCTT
5D-2138150C-T10-38_541	GATTATTTTGCGGGAAGTTTCTTTTTCTGTGCTGCTAAGGTTTCGTCTTTCCCTTCAAAAAAAATCTCGCCTTTTTCATCGGACGGCCACACCAATTTCCTCCTCGCACGCCCCATTTTT
5D-2138150C-T10-38_606	CTCGCCTTTTTCATCGGACGGCCACACCAATTTCCTCCTCGCACGCCCCATTTTTTCCAGCCAAAGAAAAAATACAAAAAGAACCGTGCTTTCTTCTCCTTCCGACCCACGGGATCAAAG
5D-2138150C-T10-38_866	CATGCGGGTCGGCATGGCACGGCATGTTTACCTtttttatttatatatatatttgtatataaatGGCATAAGAAACAACCCAATAGGTCAAAATTGGCCCAAATAACAGCCTATCAGGCC
5D-2138150C-T10-38_931	GCATAAGAAACAACCCAATAGGTCAAAATTGGCCCAAATAACAGCCTATCAGGCCAACAACCTAGCAAACAGGCGTGCCGTCGGGCCGGTCCGCATTGCTCCTGTGCTGTACCTGGACTC
5D-2138150C-T10-38_1126	ACGATTAAGATGGGCTGGGCCGGCCCGTTTGGCCAGCTCTGCGCGGGATACTCAAAGCTCCGTTAGACATCCATCAATCCAAATTCTTTCCCGTGAAAAGGATAGTTGCAACGCACACAA
5D-2138150C-T10-38_1191	GACATCCATCAATCCAAATTCTTTCCCGTGAAAAGGATAGTTGCAACGCACACAATCATTCTTATTAAATATAGTACAAAACAAAAGTAACAAATCTCAAACCATTTTAAGATATGGAAT
5D-2138150C-T10-38_1256	TAAATATAGTACAAAACAAAAGTAACAAATCTCAAACCATTTTAAGATATGGAATTAACATATATTACCTCCATGTTGTGCCTAGGCATCCGCATCTATACAAATCTAAGACAAATAATT
5D-2138150C-T10-38_1321	TACCTCCATGTTGTGCCTAGGCATCCGCATCTATACAAATCTAAGACAAATAATTTTGGACAGGGGGAGTATGAGTAAGACGACTTCTTCCAACTCAATACCTTTAAGAAGGATTAAACG
5D-2138150C-T10-38_1386	GGAGTATGAGTAAGACGACTTCTTCCAACTCAATACCTTTAAGAAGGATTAAACGTCCCTGCGCCATCGTCCGCATGTCCGGTCGGAGACAACATGAAGAAGGATTTATATCTTGGTTGC
5D-2138150C-T10-38_1451	ATCGTCCGCATGTCCGGTCGGAGACAACATGAAGAAGGATTTATATCTTGGTTGCCGAGACTATCGACAAAGGCCATTTTGACTGAATTGGTCGGAGCGTAGACGAGCAAGCAGAGCCC
4D-1177350-T10-39_62	CCTTGGCTGGTGTCATTCATGTAGGGCACGAACCAATGTAAGATGGCGATGCAAAATCCTCCTTGGGTCGCGCACAAGAGGTGGGGCTCGATTGGCCGGCGACGGAAGTTCTTGTACCA
4D-1177350-T10-39_124	TTGGGTCGCGCACAAGAGGTGGGGCTCGATTGGCCGGCGACGGAAGTTCTTGTACCATCCCCATGGCAAAAATGGTGAAGAGAGAGTAGACGAGGTGAATGAGGTCGGCGACAGCGA
4D-1177350-T10-39_186	ATGGCAAAAATGGTGAAGAGAGAGTAGACGAGGTGAATGAGGTCGGCGACAGCGAATGGACCTTGATCGACAATGGTGAATGGCACTTGGCCGGCTCCACCACCTCCAAGATGGGCA
4D-1177350-T10-39_372	GCTCCTTCACCCCATCATCGGGCTCGCCTAACTAGCGCCTGGTATGGAACCGACGCACTGACGGCTGAGAAGGACAAATGGTGGTGGTGGAGGAGGAGGGTCACAGCGGCTGTGTCAG
4D-1177350-T10-39_434	GGCTGAGAAGGACAAATGGTGGTGGTGGAGGAGGAGGGTCACAGCGGCTGTGTCAGCGAGGGATTGGTGGAAGAGGAGGGGGTTGCAGCGGTTGCTGATTGGGGAATGGGGTTC
4D-1177350-T10-39_620	TCCACCCGCCCGATGGCTCACCTAGCCAATCCACATTGGGAACGAGCCCGCCAGTCATAGGCCTGGAAAAACAACTATGAGGTAAAAACCAGATTAACCACAAGGTGAACATCGGATGG
4D-1177350-T10-39_682	CTGGAAAAACAACTATGAGGTAAAAACCAGATTAACCACAAGGTGAACATCGGATGGAGGAGAGGAAGCACTTGACCAAAATGACGCTGATCCAACGACCGGAAGTGGACCAAATCGG
4D-1177350-T10-39_744	AGGAAGCACTTGACCAAAATGACGCTGATCCAACGACCGGAAGTGGACCAAATCGGGGGAGCCTCCTGGTGGCAAGTTGGCTAGGGTCCTTGTAGGTTTAAATGTGACATCTCGTATGC
4D-1177350-T10-39_868	AGGTGTTGTCCAATATAAATCTGCCCGGCACAAACGCACTCTGATTATCCAAGATGATCTCCCCCAAGATACACTTCAAATGATTGGCGATAACTTTAGACACTAGTTTATACACGATGT
4D-1177350-T10-39_930	CCCAAGATACACTTCAAATGATTGGCGATAACTTTAGACACTAGTTTATACACGATGTTGCAAAAGGGTAATCGGGCGTAAATCTTTCAATTTCTCTGGATTTTTAACCTTGGGGATAAG
4D-1177350-T10-39_1054	ATTATGGTATCGTTCCATCCCTCCGGTATATTGCCGCCTTGCAAGACATTTAACACATCCTGTACCACACTATCACTAACCAGATCCTAGTACTTCTTGTAAAAAATCATTGGCATGCCA
4D-1177350-T10-39_1116	TACCACACTATCACTAACCAGATCCTAGTACTTCTTGTAAAAAATCATTGGCATGCCATCTAATCCGGGTGCCTTTAAATCACCTATTCTGTTAAGGGCCAACTTGACCTCATCAGCCAT
4D-1177350-T10-39_1178	ATCCGGGTGCCTTTAAATCACCTATTCTGTTAAGGGCCAACTTGACCTCATCAGCCATGAAATCCGTCATCAGAAATACATTCATTTGTTGAGTAGCCTTTGAAGTAACAATGTTCATGA
4D-1177350-T10-39_1240	TCCGTCATCAGAAATACATTCATTTGTTGAGTAGCCTTTGAAGTAACAATGTTCATGAAACCAAAGATACCTCTACGAGGGTAAACAAAGAGTACAAATACAAGAGCCTTCAAGCcctcc
4D-1177350-T10-39_1364	cttccaccacctcaccatcctccttcctcaacctcTTAATAGTTGTATTTTCTGATTTACGAGTTTAAATGGAATGTAGAGGTTTATTTTGAAAAATGAAACCCGTAACGTTTGGAAAGG
4D-1177350-T10-39_1426	GTTTAAATGGAATGTAGAGGTTTATTTTGAAAAATGAAACCCGTAACGTTTGGAAAGGCCGATTTTTTTCCTGGTGTTTTGTCTTCAGATTTGTGCGTTCTTCCCCGTAAAAGAAACACA
4D-1177350-T10-39_1984	CTCGAGGATTTGACTCTCATCGGTAAGGGGCATGTTGCGTTATTAGCCGTTCTGCCTGGAGTGAGTTTGGTGGGTGCCGCGACTGGGATTCTGTGAACCTGCTGCTAGGGTTTACCCCTT
4D-1177350-T10-39_2046	GAGTTTGGTGGGTGCCGCGACTGGGATTCTGTGAACCTGCTGCTAGGGTTTACCCCTTCCACAATATGGAAGTCTAGCTTTCCTGCTAATGTGATTTTAGTTGGGATTTATGGCGCCGAA
4D-1177350-T10-39_2108	AATATGGAAGTCTAGCTTTCCTGCTAATGTGATTTTAGTTGGGATTTATGGCGCCGAACGCTGCTTCTGCTGTAACCAGGATAGATTAACCCTGGTGTGTTTCTGCACGGTGAATTGGCG
4D-1177350-T10-39_2170	GCTTCTGCTGTAACCAGGATAGATTAACCCTGGTGTGTTTCTGCACGGTGAATTGGCGATTGGTAGGAACAACAAGGCTTCATGTTAATTTAAGAAAATACGCCGAGATGTTCCTAGATT
4D-1177350-T10-39_2232	GTAGGAACAACAAGGCTTCATGTTAATTTAAGAAAATACGCCGAGATGTTCCTAGATTTAAAGCTTTACTTCAATGTAACAAACGATATTCCATCTTTCATCTCAGCTCGGTTTCCTTAC
2A-0316910-T10-40_0	TATAGTCTCCTTCGGTTCATTATTTCGAACAAGGGGATGCAAAATTACCTTCAATAAAAGAAAATTAGAGAAAATCGTATATGTCTTGTTTGTCTTGAAATTAAAAAAAGAATAGGTGTG
2A-0316910-T10-40_64	TTAGAGAAAATCGTATATGTCTTGTTTGTCTTGAAATTAAAAAAAGAATAGGTGTGAATGTTTCTCTAAAAATTCACGTACATGCTACTCACCCATGTATTTCGTGCTTATTGCTATCCA
2A-0316910-T10-40_512	CCGAACCCGGCGGGTGCCATGTTGAGATGGAGACAGGGGCAACCGTATCGAAGAGGAATAGGGGAAGCCTCGATGACAACGTGCGAGTAGGAGGGAGGGCGGCCATGACGCCGGC
2A-0316910-T10-40_704	ACAGACGGGCGCGGGTGGCAAGCAGAAACCCTAGCAGGGGCGCTCGGGAGTGAATGAGGTAGACCGAACACAACCGACTATAGTGGGCCTACCGACAGCGATACATCCACTTACAAA
2A-0316910-T10-40_768	CGAACACAACCGACTATAGTGGGCCTACCGACAGCGATACATCCACTTACAAATGAGCCAATCAACGCCCTCCAAAGCCCCAATGAACCAGATCCCAACATTTCGTGGGAGCTGTAAAAA
2A-0316910-T10-40_960	ACTTGATACCAGGAGTACTAGTACATCGCTGCCTGAAAAAAGATAACAGATAACAGTAGATCGCTGCCCTGGAGCGTGTGACCGTAACCGACATACGCGCATTGGTCTCGTTGCAACGAT
2A-0316910-T10-40_1024	TGCCCTGGAGCGTGTGACCGTAACCGACATACGCGCATTGGTCTCGTTGCAACGATTTTCCTGTGCTTTCCCCCACGAAGGAGTTTTTTAGTTTTTTTTTTGGTGAAGGAGTTCTTTTAG
2A-0316910-T10-40_1088	GCTTTCCCCCACGAAGGAGTTTTTTAGTTTTTTTTTTGGTGAAGGAGTTCTTTTAGTTTTTTTTTTGAGAAATGAAGGAGTTCTTTTAGTAACCCCACGAAGGAGTAGGGGACACGGAAA
2B-0426540-T10-40_0	GCTGCTATAGAGAATCCTAACCCATCATCTGCAGTACTGTACACCGTTGTAGTGCAAATAACCAGTGGATGCTTTATGGATTTGTTTAGAGATACATCTAACCCTGGGAATAGTTtcaaa
2B-0426540-T10-40_64	GTGGATGCTTTATGGATTTGTTTAGAGATACATCTAACCCTGGGAATAGTTtcaaatccagattgaaaccaggccaaaccactcgacttcccttccagtcaccaatccaatccaatccaa
2B-0426540-T10-40_128	gaaaccaggccaaaccactcgacttcccttccagtcaccaatccaatccaatccaatTATTGAGGTCTAGAATCTAAACTGAACCAAACTAGGTGTAGTTTCCATCCAAACCAATCCAGA
2B-0426540-T10-40_192	GTCTAGAATCTAAACTGAACCAAACTAGGTGTAGTTTCCATCCAAACCAATCCAGACCGTTTCATCCTTTTGGATTTAATCCATAGTTTGCAACCATGGACAAGCTGGTGTTGGTATGCA
2B-0426540-T10-40_256	TCCTTTTGGATTTAATCCATAGTTTGCAACCATGGACAAGCTGGTGTTGGTATGCAACATGAAGCTACTGATCATCTGCGTGGCCGGCCGTGCGCTTAATGGAGAGAGACACGCTGCTAG
2B-0426540-T10-40_512	CAACGGCTAGAGCGTGGAAGATGGCTTACGATGTGCTGGTCTCGTACGCTTGTTTTGGGACGCTGGTCTCGTACACCCCTAAACAGGTGGCTGATTTGAGATCTATTAgggcatgtacaa
2B-0426540-T10-40_1024	AGGTTTGAGTCCAAACTATTCCCAGGCTTAGATACATCGTTGCCTAAAAAAAAATATACTACGCCCTGGATTCTGAACGTAACCGACATAGGCGTTGCAACAGTTTTTCCGTGCTTTCCT
2B-0426540-T10-40_1088	CCTGGATTCTGAACGTAACCGACATAGGCGTTGCAACAGTTTTTCCGTGCTTTCCTCCACGAAGGAGTTATTTTTATAGTAACCCCACGAAGGAGTAGGGGACACGGAAACGCATTCCGA
2B-0426540-T10-40_1536	CGCCCGCGGTTTTATCCGTTGGTTCCTGAGGTACGAGCATTTCGACATCCCGCGACTAGTTAAATTCCACCGACCTCCAGAAATTTGGGGTGAACCATCTAGATCCTCTCCTGCTCTGGC
2D-0523770-T10-40_0	TAGGTAAGATGGTCTAGTGGTTATGGCAGCGCGCAGAGGTCACCAGTTCGAATCTTGCACGGCGTGTTTTTTTTTATTCAGCAAAAAAGAAGAAGAATGGGCCGGCCCAGTGTGGCGGC
5A-1234690-T10-41_0	AGACCCTTTCGCAACCACCTACTCGTGCATCGCCCCGTATAAAATCAAGGAGTACAGAAAACACATATTGCTTAACTTTTGGTTGTAGCTGGTCTTTGCACGTCTGCGAACAGTTGAGGA
5A-1234690-T10-41_64	ATATTGCTTAACTTTTGGTTGTAGCTGGTCTTTGCACGTCTGCGAACAGTTGAGGAAAGAAATCCTTCACAAACAAAGTCTCATATAATAAAAAATAAACCACTAGCGAAAGCTATGTGC
5A-1234690-T10-41_832	ATCGTGACTTACAATTAATCCATACAAATTGTTTACGATGAGACAAAACAACAGAAACAGTTGAGGGGGGTGCCGGTCAGCCTGGCCATTTGAGGGCCGTTTGAGAGGCCCATCTAGGT
5A-1234690-T10-41_960	GTGACCGACCAGTGACATGGCGGTCCGCCCAGACGTATAAGGCGGGTTTGAGAGATCCGCTTGTAGATGCTCTAAGCGCAACAAGGTAGTTGTTGCAAAGGTCCTCAACTAGCTATGAT
5A-1234690-T10-41_1024	AGATGCTCTAAGCGCAACAAGGTAGTTGTTGCAAAGGTCCTCAACTAGCTATGATGGTAGATCAGACTGCTATTGTTATAAATGTTTCTGCAACCCCGTTCTTGTTGCAAGGAAGGTTAC
5A-1234690-T10-41_1088	GACTGCTATTGTTATAAATGTTTCTGCAACCCCGTTCTTGTTGCAAGGAAGGTTACACACGTGGATGAGTAGATCGGATGGCCATTCTTGTGCCCATCCAACGACCAGTTACGTGGCGGA
5A-1234690-T10-41_1152	ATGAGTAGATCGGATGGCCATTCTTGTGCCCATCCAACGACCAGTTACGTGGCGGATGTTTTCTACTTATTCGCCGGCTGATGCGTAGCATGTCATACTCATCTCTTTTTATTGTAATCG
5A-1234690-T10-41_1216	ACTTATTCGCCGGCTGATGCGTAGCATGTCATACTCATCTCTTTTTATTGTAATCGAACGACGGTGTAATCCGGCGATGCTGTGAGATTCGACGCACGCTTTCTGATCGACGGATTTAAA
5A-1234690-T10-41_1280	TGTAATCCGGCGATGCTGTGAGATTCGACGCACGCTTTCTGATCGACGGATTTAAATTTTAAATTTATTTACTAGCAGATACTAGTATTGATAGTTTTGAATGACTGGCTCGCGCATCCA
5A-1234690-T10-41_1344	TTATTTACTAGCAGATACTAGTATTGATAGTTTTGAATGACTGGCTCGCGCATCCATGTCTACGATCCGCCACGGATGAAATGCCCTTAAATTCAATGCATAAAAAAACATCGAAATTTA
5A-1234690-T10-41_1408	ATCCGCCACGGATGAAATGCCCTTAAATTCAATGCATAAAAAAACATCGAAATTTAAAGCGCTCCACAATTCAAACAGAGCGAGACTTTTCCAGCTGTCACAAGTGAAAAGGTGAACCCC
5D-1438450-T10-41_128	ACATTTTTTTAAATGAGTGAATTTTTTGGAAATTACTGTCAAGATTTCCTTAATATACGCAATGAACTTTGTATATATAGAAAAAGAAATAAAAAAGATAGTAAAGAAATATTAAAGAAA
5D-1438450-T10-41_192	AACTTTGTATATATAGAAAAAGAAATAAAAAAGATAGTAAAGAAATATTAAAGAAAAATATGCACGAACACTTTTTCAATTTTACAGTTTGTTTTCAAAATCTATGATTTTTTCCCAAAT
5D-1438450-T10-41_384	TTTTCAAAATTTGTAAACTATTTTTATTTTTGAAATTCTAGTTCAGAAGTTTCTTTTTTTCCGATCAAACAGCACCAGCATGGGGTCTCGTGTTCGAGCAGAGTTTTTTTTTTCTGCTTT
5D-1438450-T10-41_768	TTCCCCTGAATGATGAAACGTAGCAATGTTATATACAATGATGATCATCGTTGGTTGCTTCACGCAATGATGGATCTGTTTGAACTTTTGTACCGAGCAGCGACGATCGGGGCAAGCTTT
5D-1438450-T10-41_832	CAATGATGGATCTGTTTGAACTTTTGTACCGAGCAGCGACGATCGGGGCAAGCTTTTTTTCTTGTTGTTGTCTTGGGTGCTGTAGCTGTAGGTACTGTTCTGTGGTCTGTGTACGCCGGCG
5D-1438450-T10-41_960	CTTGGCGTATGACGAAAGCTTTTCCGGCGGGGACCGTGGGGCGGCGTTTCCTTCCCTCGCCTGGTGCATGGGCAGCGCGGCATTGATGATGGCCGGCACACTGTCACCTGGATCGTGTT
5D-1438450-T10-41_1024	TGCATGGGCAGCGCGGCATTGATGATGGCCGGCACACTGTCACCTGGATCGTGTTGGATTAGTGCGTACACCTGTGCTACTAAAAGCCTGGTGGTGGTAGGCTTGAGTGATGGCCGTGTT
5D-1438450-T10-41_1088	CGTACACCTGTGCTACTAAAAGCCTGGTGGTGGTAGGCTTGAGTGATGGCCGTGTTGGGTCACTGTAGCAGTTCATTCCTTGTCGGCGTTCCGAGTTATTGACGCGGTGGTGGTCACGTC
5D-1438450-T10-41_1152	GTAGCAGTTCATTCCTTGTCGGCGTTCCGAGTTATTGACGCGGTGGTGGTCACGTCTCCCGGTGGTCGATCGCTGTACAGCTCCGGCCGATGAAATTTGATCCGGAGTACGTACTGGATC
5D-1438450-T10-41_1344	TCCAGCGAATCAATCATCGCTAGGCTTTGTCTTGCTTCGCATCGTTAGCTTGATAACGAAGAGATCGATAACGAAATCCAGTAGCGTTGAGCTGTCTAAATCTAAAAAGAACATTCTCTT
5D-1438450-T10-41_1408	TCGATAACGAAATCCAGTAGCGTTGAGCTGTCTAAATCTAAAAAGAACATTCTCTTTCGATGGACTCCACATGCCCACACTCGGTTCTGAGGTCTAGTAAATCTAAAACACAACTTCAAT
5D-1438450-T10-41_1472	CTCCACATGCCCACACTCGGTTCTGAGGTCTAGTAAATCTAAAACACAACTTCAATTCGTCCTTTTTTCTTTAAATTTTACTAAAACTAACACAAATTCGAAGTGCCGCTGGAGCAGCTC
5D-1438450-T10-41_1536	TTTTCTTTAAATTTTACTAAAACTAACACAAATTCGAAGTGCCGCTGGAGCAGCTCCCCAAATCTTTATTTTTTTGAGACAAACTCCCCCAAATCTGCCAAAGCCCAAACGCGCGCGCGA
5A-1272800-T10-42_0	TGCGGCTTCTGGGATAACAATAGACCAGATTGACTGAAGTTGAAGCTGATAAACAGGAGAACAAGAGAATTAGCTAAGGTTCCACCAATGTTAAAGCATCTTTTAGGATCAAATTAAGCT
5A-1272800-T10-42_63	AGAGAATTAGCTAAGGTTCCACCAATGTTAAAGCATCTTTTAGGATCAAATTAAGCTCCACAGATGTATAAATAAATACAATATCAGACTATACGCCATGCCTGAGACCAAAATTTAGAA
5A-1272800-T10-42_126	ATGTATAAATAAATACAATATCAGACTATACGCCATGCCTGAGACCAAAATTTAGAAATGAACAGTAACACAGAAGACAGAACTGTGCAAAATATTCCCGTCAAGTGTAACTTATGTCAG
5A-1272800-T10-42_189	AGTAACACAGAAGACAGAACTGTGCAAAATATTCCCGTCAAGTGTAACTTATGTCAGGTCTGAATTGCTATGCAGTACTAAAATGAGTCAAATCGAACAGGCTGCTGAGAGTAATAGCAT
5A-1272800-T10-42_252	ATTGCTATGCAGTACTAAAATGAGTCAAATCGAACAGGCTGCTGAGAGTAATAGCATAACAACCAGATCGACTTGGCCTACTCGGAGAAATACCAAGATCAGCCCCTTGTAAGCATATAT
5A-1272800-T10-42_315	CAGATCGACTTGGCCTACTCGGAGAAATACCAAGATCAGCCCCTTGTAAGCATATATTGAATTAATAGACTGAAATTAACAGGTGAAACTGTATCGCCGAGCACCGGACTGAACGCGGTC
5A-1272800-T10-42_378	AATAGACTGAAATTAACAGGTGAAACTGTATCGCCGAGCACCGGACTGAACGCGGTCCCGGCATCCAGAGATCGTCTTCCACAAGCAATACAGCTTAGGTCAAATAACTTGACTCGGGG
5A-1272800-T10-42_567	TCAGGTGTTGGATAGCGACCAATCGGAACCGAGCAAACCCCGGCGAAATCAGACGACGCGAGCAAACAAAAACGGCGGCAACAAGCTTGCAATCGAGGTGAAATTAGAACATACGGA
5A-1272800-T10-42_630	AAACAAAAACGGCGGCAACAAGCTTGCAATCGAGGTGAAATTAGAACATACGGATTAGGAAATCCACAGGCGGCGGGCACCTAATTCCAACCCGTAAACCCGAAATCCTCgcgtgcggcg
5A-1272800-T10-42_1346	GGAGGCACGCTTTCATGGCCCGCGTGTCAGTCAAAGAAAGGGAGCGCGCGTTGGCGTAGGACGGCGAGAGACGCTTTGAGTCGTCACGGAGGACCGCCATGGCCTCGCCACCAAGA
5A-1272800-T10-42_1405	GACGGCGAGAGACGCTTTGAGTCGTCACGGAGGACCGCCATGGCCTCGCCACCAAGAGCAAGCTGTCAATTGAGCCTCTTATAAACGGCACTTTATACCTTAACTTTTTATTCTATTTTT
5A-1272800-T10-42_1464	AAGCTGTCAATTGAGCCTCTTATAAACGGCACTTTATACCTTAACTTTTTATTCTATTTTTATATTTGCTCCGCTAAACAAGTTCCCCGATGAGCTTTTAAATTTAATTTTCTATAAAAA
5A-1272800-T10-42_1523	TTATATTTGCTCCGCTAAACAAGTTCCCCGATGAGCTTTTAAATTTAATTTTCTATAAAAAAAAATCACACAAATTTAGATATCGATAGCTCAATTTGATTCATACCATATTGCATTAACA
5A-1272800-T10-42_1580	AAAAAAATCACACAAATTTAGATATCGATAGCTCAATTTGATTCATACCATATTGCATTAACATGGATATTTGCTTCTATAAAGAATGGCGAAGTACCCACCTTCTCAACTGCTCGTGGG
5B-1370900-T10-42_0	TGCAGCTTCTGGGATAATGATAATAGACCAAATCAACTGAGGTTGAAGCTGATAAACAGGAGAACAAGAGAATTAGGTAAGGTTCCACCAATGTTAAAGCATCATTTAGGATCAAATTAA
5B-1370900-T10-42_64	CAAGAGAATTAGGTAAGGTTCCACCAATGTTAAAGCATCATTTAGGATCAAATTAAACTCCACGGATGTATAAATAAATAGTACAATATGAGACTATAAGCCATGTCTGCGACCAAAATT
5B-1370900-T10-42_128	GATGTATAAATAAATAGTACAATATGAGACTATAAGCCATGTCTGCGACCAAAATTAGAGATGAACAGTACCACAGAAGACAGAACTGTGCAAAATATTCTTCTCAAGTGTAACTTATGC
5B-1370900-T10-42_192	ACAGTACCACAGAAGACAGAACTGTGCAAAATATTCTTCTCAAGTGTAACTTATGCCAGGTCTGAATTGCTATGCAGTACTAAAATGAGTCAAATCGAACAGGCTGCTGAGAGTAATAGA
5B-1370900-T10-42_256	AATTGCTATGCAGTACTAAAATGAGTCAAATCGAACAGGCTGCTGAGAGTAATAGAATACCAACCAGATCGACTTAGCCTACTCTGAGAAATCTCGAGGACCAGCCCCTTCTAAGCACAT

5B-1370900-T10-42_320	CAGATCGACTTAGCCTACTCTGAGAAATCTCGAGGACCAGCCCCTTCTAAGCACATCGAATTAATAGAAGACCAAAATTAACAGGTGCAACTGTACCACCGAGCACCGGACTAAACGCGG
5B-1370900-T10-42_448	TCCAGAGACCGTCTTCCACAAGCAATACAGCTTAGGTCAAATAACTTGACTCGGGAACGCAATACCGACACATTTGTAGGACTTGCGGCAGCTAACCGCGTGTACAGAAGCATTCCAACA
5B-1370900-T10-42_512	CCGACACATTTGTAGGACTTGCGGCAGCTAACCGCGTGTACAGAAGCATTCCAACATTAAGATCCATGGAGTATCAGGTGTGTTTTTTTTTGCGGGGGGAGTATCAGGTGTTTGATAGCG
5B-1370900-T10-42_576	CATGGAGTATCAGGTGTGTTTTTTTTTGCGGGGGGAGTATCAGGTGTTTGATAGCGACGAATCGGAACCGAGCAAACCACAGCGAAATGGCAACAACAAGCGTGCAATCGAGGTGAAA
5B-1370900-T10-42_640	GAACCGAGCAAACCACAGCGAAATGGCAACAACAAGCGTGCAATCGAGGTGAAATTAGAACATGTAGATTAGGGAATCCACAGGCGGGCACCTAATTCCAACCCGTAAACCCTAATACC
5B-1370900-T10-42_704	TAGATTAGGGAATCCACAGGCGGGCACCTAATTCCAACCCGTAAACCCTAATACCTCgcgtgcggcggcagcggcggcggcggcaaggtataccaggggcggcggcgaggtggaccaggg
5B-1370900-T10-42_896	gagagagagagGGGCGAGGGGGTGGCGGTAGGGTAGGGCCGTAGGGGGGGATTTCGTGAGGGGGCGGGTCGCGGGGCGAATTTTCAGAAGGGTGTCGAAGAGAGGAAGACGGCGG
5B-1370900-T10-42_1088	TCAAAGAAATGGTGCGAGACACGTTTTAAGTCGTCACGGAGGACCGCCATGGCCTCACCACCAAGAGCAAAGCTGTCAATTTAGCCTCTTAAACGGCACTTATACCTTATATACTACTCC
5B-1370900-T10-42_1152	GAGCAAAGCTGTCAATTTAGCCTCTTAAACGGCACTTATACCTTATATACTACTCCCTCCATCAAAAAAGTCTTACATTTCGGAACGGAGGGTAGTATTTTTATATTAACTCCGCTCAAC
5B-1370900-T10-42_1216	AAAAAGTCTTACATTTCGGAACGGAGGGTAGTATTTTTATATTAACTCCGCTCAACAAGTTCCCCGCTGAACTTTTAAATTTAATTTCTATAAAAAAATCACAGAAATTTAAATATCGAT
5B-1370900-T10-42_1280	CGCTGAACTTTTAAATTTAATTTCTATAAAAAAATCACAGAAATTTAAATATCGATAGCCCAATTTAATAATTCAAACTATATTGCATTAACATGGATATTGCTTATAAAGGATGATGGA
5B-1370900-T10-42_1344	TTAATAATTCAAACTATATTGCATTAACATGGATATTGCTTATAAAGGATGATGGAGTACCTGCCTTCATGAGCTGCTCGTGGGTGGAGAGAGCCCATGGCCGTTCATCCCGCGGTGCCT
5B-1370900-T10-42_1408	CTTCATGAGCTGCTCGTGGGTGGAGAGAGCCCATGGCCGTTCATCCCGCGGTGCCTTGCCGTAGGGGCTACTCCGTGGGGCTCCAATCTGCCCGGACGGCTCGGACAGTGGCGACCCG
5B-1370900-T10-42_1536	CTATGACGGGTGGTCCGCGTGAGTGTGCCGAGGGGCCACCGTCGTTGGGCCACGTCAGGGGCGCCCTCACATGGCCTTTCTCACGGCGTTGCATGTGGGAGAACCCGGCTCGGTGGT
5B-1370900-T10-42_1580	TTGGGCCACGTCAGGGGCGCCCTCACATGGCCTTTCTCACGGCGTTGCATGTGGGAGAACCCGGCTCGGTGGTGCATGACAGTTGGACCACGGGGCGTGCCGGAGGCTCTCTAGGCA
5D-1489900-T10-42_0	TGCAGCTTCTGGGATAATAATAGACCAAATTAACTGAAGTTGAAGCTGATAAACAGGAGAACAAGAGAATTAGGTAAGGTTCCAGCAATGTAAAGCATCTTTTAGGGTCAAATTTAGCTC
5D-1489900-T10-42_64	GAGAATTAGGTAAGGTTCCAGCAATGTAAAGCATCTTTTAGGGTCAAATTTAGCTCCACAGATGTGTAAATAAATACAATATGAGACTATAAGCCATGCCTGAGACCAAATTTAGAAATG
5D-1489900-T10-42_128	TGTAAATAAATACAATATGAGACTATAAGCCATGCCTGAGACCAAATTTAGAAATGAACAGTACCACAGAAGACAGAACTGTGCAAAATATTCCCGTCAACTGTAACTTACTCCAGGGTC
5D-1489900-T10-42_192	CACAGAAGACAGAACTGTGCAAAATATTCCCGTCAACTGTAACTTACTCCAGGGTCTGAATTGCTATGCAGTACTGAAATGGGTCAAATCGAACAGGCTGCTGAGAGTAAGAGCATACCA
5D-1489900-T10-42_256	TATGCAGTACTGAAATGGGTCAAATCGAACAGGCTGCTGAGAGTAAGAGCATACCAACCAGATCCACTTAGCCTACTCGGAGAAATACCAGGACCAGTCCCTTATAAGCATATCGAATTA
5D-1489900-T10-42_320	CACTTAGCCTACTCGGAGAAATACCAGGACCAGTCCCTTATAAGCATATCGAATTAATAAAAAGACCAAAATTAACAGGTGCGACTGTATCACCGAGCACCGGACTAAACGCGGTCTCAG
5D-1489900-T10-42_448	GATCGTCTTCCAGAAGCAATACAGCTTAGGTCAAATAACTTGACTCGGGGAAAGAAATACTAACAAATTTGTAGGAATTGCGGCAGCTAACCGCGTGTACAAAAGCATTCCAGCAGTAAG
5D-1489900-T10-42_512	AAATTTGTAGGAATTGCGGCAGCTAACCGCGTGTACAAAAGCATTCCAGCAGTAAGATCCATGGAGGATCAGGTGTTTGATAGCGACCAATCGGAACCGAGCAAACCACGGCGAAATCA
5D-1489900-T10-42_576	AGGATCAGGTGTTTGATAGCGACCAATCGGAACCGAGCAAACCACGGCGAAATCAGACGACGTGAGCGAAAGGAAATGGCAACAACAAGCGCGCAATCGAGGTGAAATTAGAACATG
5D-1489900-T10-42_640	AGCGAAAGGAAATGGCAACAACAAGCGCGCAATCGAGGTGAAATTAGAACATGTAGATTAGGAAATCCACGGGCGGGCACCTAATTCCAGCCCATAAACCCTAAAGCCTCGCGTGCGG
5D-1489900-T10-42_896	AGGGGTGAGGGGGTGGCGGATTGGGTGGGGAAAATTTGGGGGAGGGGGTTCGTGGGGCGAATTTTCTGAGGGCATCCAAGACGGCGGGGATTTTGAAATTTTGGGGAACTGGTCA
5D-1489900-T10-42_1088	TGGCCTCACCACCAAGAGCAAAGCTGTCAGCCTCTTATTAAACGGCACTTATACCATAACTTTTTTATTTTTGCTCCGCTAAAAAAAGTTCCCCGCTCAAATTTTAAATTTATTTTCCTA
5D-1489900-T10-42_1152	TTATTTTTGCTCCGCTAAAAAAAGTTCCCCGCTCAAATTTTAAATTTATTTTCCTATATATATAAAAAGTTCACACAATTTTGAATATCGATAGCCCAATATTGCATTAACATGGATATTGC
5D-1489900-T10-42_1216	AAGTTCACACAATTTTGAATATCGATAGCCCAATATTGCATTAACATGGATATTGCTTCTATAGAGGATGGTGAAGTGCCCGCCTTCATGAGCTGCCTCGTCGGCGGAGAGAGCCCCATG
5D-1489900-T10-42_1280	AGGATGGTGAAGTGCCCGCCTTCATGAGCTGCCTCGTCGGCGGAGAGAGCCCCATGGCCCTTCGTCCCGCGGTGCCTTGCCATGGGGGCTACTCGATGGCGCTTCGATCTGCCCGGACA
5D-1489900-T10-42_1408	CCGGCGACCCGGCTGGGCAGTCTATGACGGGTGGACCGCGTGAATGTGCTGGGGAGGCCACCGTCACTGGGCTGCGTCGGGCGCGCCCTCACGCGGCCTTTCTCGCGACGTTGCATG
5D-1489900-T10-42_1472	CACTGGGCTGCGTCGGGCGCGCCCTCACGCGGCCTTTCTCGCGACGTTGCATGTGGGAGAGACCGGCTCGGTGGTGAATGATACGTGGACCGCGGGGCGTGCCCGGGGCCACCATCG
3A-2150170-T10-43_896	CAGTCCTTTATTCCCAATCCCTTTGTCAAGCAATCTAGAAGATGCTGCTCATCCCCTTCATGTCATGCGAAGTTAGTGACATGAAATTTTGACCAAAAATTCGGAAGTGTCCAGAAAGAT
3A-2150170-T10-43_1088	AAAATATGATACTCGTACATCAGTCACTAACGGTTCTTGTTTCAAGGGTGAATTGGATACCAGCATTTTGACAAGTTGCCAATATATtactccctccgtcccaaaataagtgtctcaact
3A-2150170-T10-43_1280	TGCATCCCAGAGTCCTATTTTGCTGGGACAACTATGTCCGCAGCGAAGGTCATAATGTTATGCACCCCGAAAATGCACGCATCAATGGCGATTGAACCTTGAACAAATCTGATGGTAATG
3A-2150170-T10-43_1344	CCCCGAAAATGCACGCATCAATGGCGATTGAACCTTGAACAAATCTGATGGTAATGTGCTGTTTAAATTTTGTGTTGGAATCATGAATAAACTAGAAGACAACATGTAGTCTGGCTTACA
3A-2150170-T10-43_1408	AAATTTTGTGTTGGAATCATGAATAAACTAGAAGACAACATGTAGTCTGGCTTACAGAGTATATCCATTTAGCAGAGCATACTAGGCAGCACAGTTATTTACATTGTTTCTATATAATTG
3A-2150170-T10-43_1472	CCATTTAGCAGAGCATACTAGGCAGCACAGTTATTTACATTGTTTCTATATAATTGTCAGATTGTTTCAAGTTTATTTTATTTTTTCCTACTTTCAGGGTATTGCAGAGTCTTGAAACTG
3A-2150170-T10-43_1536	TTTCAAGTTTATTTTATTTTTTCCTACTTTCAGGGTATTGCAGAGTCTTGAAACTGAATTTGCAGGTTTAAAGGAGGAAATGGTATGTTCAGTATGCAGTAAGCATGTTTATGTTGTCAT
3A-2150170-T10-43_1580	GTCTTGAAACTGAATTTGCAGGTTTAAAGGAGGAAATGGTATGTTCAGTATGCAGTAAGCATGTTTATGTTGTCATCTATTTTCTCAATGACCCTTTTATTAATTTATGGGTATCTCTAG
3B-0754730-T10-43_64	AGCTACTGGGATAGCACCTTGGCCCCTGATACCAGTGTGGGATGGTGGTCCGAATTGAATATTGATCAATTCAGTGCGGCGGCGCCATGTTGGGGTTCATGTTCTTGTACTGCTGGTACC
3B-0754730-T10-43_128	ATCAATTCAGTGCGGCGGCGCCATGTTGGGGTTCATGTTCTTGTACTGCTGGTACCACATTTATGCACTGCCCGTTAGGTGGAAGAAGGTGATGCTCACCGTCTCTGTGAAGGCCGTGCG
3B-0754730-T10-43_832	TTTGTCAAGCAATCCGGAAGATGCTGCTCATCCCCGTCATGTCATGCGAAGTTAGTGACATGAAATTTTGACCAAAAATTTGGAAGTGTCCAGAAAGATTGGCCACTTGGCCAGACCAGT
3B-0754730-T10-43_960	GTAGCAGCAGCCTGACTGGTTTGGTCACCGGCCTGGTTTAAAAAAAAATATGATACTCGGTTCTTGTTTCAAGGGTGAATTTGGATACCAGCATTTTGACAAGTAGCCAATATATTACGTA
3B-0754730-T10-43_1024	GTTTCAAGGGTGAATTTGGATACCAGCATTTTGACAAGTAGCCAATATATTACGTACTAAACTGTGCATCCCAGAGTCCTATTTTGCTGGGACAACTGTGTCCACAGCGAACGCATCAAT
3B-0754730-T10-43_1088	TGCATCCCAGAGTCCTATTTTGCTGGGACAACTGTGTCCACAGCGAACGCATCAATGGTGGCCGAACCTTGAAAAAATCTGATGGTAATGTGCTGTTTAAATTTTGTGTTGGAATCATAA
3B-0754730-T10-43_1152	AACCTTGAAAAAATCTGATGGTAATGTGCTGTTTAAATTTTGTGTTGGAATCATAAATAAACTACAAGACAACATGTAGTCTGGCTTACAGAGTATATCCATTTAGCAGAGCATACTAGG
3B-0754730-T10-43_1216	CAAGACAACATGTAGTCTGGCTTACAGAGTATATCCATTTAGCAGAGCATACTAGGCAGCACAGTTATTTACATTATTTCTATATAATTGTCAGAGTATTTCAAGTTTATTTTATTTTTT
3B-0754730-T10-43_1280	TTATTTACATTATTTCTATATAATTGTCAGAGTATTTCAAGTTTATTTTATTTTTTCCTATTTTCAGGGTATTGCAGAGTCTTGAAACTGAATTTGCAGGTTTAAAGGAGGAAATGGTAT
3B-0754730-T10-43_1344	CAGGGTATTGCAGAGTCTTGAAACTGAATTTGCAGGTTTAAAGGAGGAAATGGTATGTTCAGTATGCAGTAAGCATGTTTATATTGTCATCTATTTTCTCAATGACCCTTTTATTAATTT
3B-0754730-T10-43_1408	TGCAGTAAGCATGTTTATATTGTCATCTATTTTCTCAATGACCCTTTTATTAATTTATGGGTGTCTCTAGTCAGAAATGACAGCACCATTGGCAGAACTGAAACCTTCTGATCAGAGAAA
3B-0754730-T10-43_1472	CTCTAGTCAGAAATGACAGCACCATTGGCAGAACTGAAACCTTCTGATCAGAGAAAGTACAACATACGAAAAAGGGAGGTTAGATGCAGAGTCAAGAAGCAGGAAAAGTTAATGAAAG
3D-0928920-T10-43_1216	TTGACATGAAACGATCAAAAATTATAGATACGACGGAGACGTATCATTCACCTTGTGGTTTTTCACGTTTTCCTGTTGTGTTCTTCGTGTTCTTCGGGTTTCCCCCCTCCAATTTGTGAA
3D-0928920-T10-43_1280	ACGTTTTCCTGTTGTGTTCTTCGTGTTCTTCGGGTTTCCCCCCTCCAATTTGTGAAAAATCGCTAGCTAGGGCTTGGCCCTACATCATCGTGTGTGTGGGCGGTCGGGCCGTATGCCTGA
3D-0928920-T10-43_1344	AGCTAGGGCTTGGCCCTACATCATCGTGTGTGTGGGCGGTCGGGCCGTATGCCTGAGCCAGGACGTGCGGGCTGTTTGGCCGTACAACTGACAACTCGTTCGACACGGTCCATATACATC
3D-0928920-T10-43_1408	GTGCGGGCTGTTTGGCCGTACAACTGACAACTCGTTCGACACGGTCCATATACATCGCACGTTCGGTGAATTACGGCATCCAATACTTATTATCCAAATTCTCGTCTTTTAGTTTTTGAG
3D-0928920-T10-43_1472	GGTGAATTACGGCATCCAATACTTATTATCCAAATTCTCGTCTTTTAGTTTTTGAGTACAAATACGTGGGTTTCTTTTTAATTAACCGAATTCGATGACCGAATATTGAAAACCTAATTT
3D-0928920-T10-43_1536	CGTGGGTTTCTTTTTAATTAACCGAATTCGATGACCGAATATTGAAAACCTAATTTTCTGTTTATCCCCAACGCCCAGTCCGTTTCTCGTGGCCCCCACCCACCCACACGCCCCGTTTGA
4A-1007510-T10-44_128	TTGGGGATTTGGGGATCGGGATTTTAAGAAATAACTTGTTCAAAGGGGTTTATGGAGAAACTTCGGTTGTCCGTCTTGCCCCGCTTATGAACTGTGGGGCTTGCTAGATATTTGCGAATT
4A-1007510-T10-44_256	CGAAAGGGGCTGGACTGCAAAAGTGTGCGCTATTGTTTTCATTTTGGACACCCGTGCTCCCAACATATCTCTACTATTTAAAAAGAATGTAAGGTTGCGAACCAACCTCTGATTGAGATG
4A-1007510-T10-44_896	TTGCAACATAAGGGCATTGTTTTAGTTTTTATTTAAAATTAGTAAAATTGTCTGTGCGTTGCAACAGGTTAAAATATATCCCTAATATCATGGGAGCAAAAACCTGCACCCTTGTACTAA
4A-1007510-T10-44_960	CAGGTTAAAATATATCCCTAATATCATGGGAGCAAAAACCTGCACCCTTGTACTAAAGGGCATAATATATAAGATGCGAGAGTCGATCTCTACTTAGTGAGACTCATTCATCGTCAATAC
4A-1007510-T10-44_1024	ATATATAAGATGCGAGAGTCGATCTCTACTTAGTGAGACTCATTCATCGTCAATACTAATGTGTGTTTCAAATCAGTTGCTGAGAGGCACGATCTTTACATCATGATATGTTTGATAATG
4A-1007510-T10-44_1088	GTTTCAAATCAGTTGCTGAGAGGCACGATCTTTACATCATGATATGTTTGATAATGTAAAAGAGGTCAATGCTACTATTAAATACATAAACAACTTGTTTAGGATTTTTTATTATTATAG
4A-1007510-T10-44_1152	GTCAATGCTACTATTAAATACATAAACAACTTGTTTAGGATTTTTTATTATTATAGGAATTAGAAATATATGAAAAAAATGGATACATATGCAAACTATCTCCAAACATATCAATATATC
4A-1007510-T10-44_1216	AATATATGAAAAAAATGGATACATATGCAAACTATCTCCAAACATATCAATATATCACATTGAGATAAAAAAGGACTATTGTTGTGAAAAATGTTTAGAAAATTAAAAACAAATGATAGT
4A-1007510-T10-44_1280	ATAAAAAAGGACTATTGTTGTGAAAAATGTTTAGAAAATTAAAAACAAATGATAGTCACGCGTGCTTGTATAGTGAGACACATAATTCTTTTTACCACTCACCCGAGGCATTAATAGGTT
4A-1007510-T10-44_1344	CTTGTATAGTGAGACACATAATTCTTTTTACCACTCACCCGAGGCATTAATAGGTTGTAAACATTTTGGATGTACCCAACTCAAAATCTATTTCAACATTGTAAATGGTATAGTTGATTC
4A-1007510-T10-44_1472	TATCATACCACCAATAATTATATTAGCATATTTTTTAGAATAACATATTATCAAATATATGTATTGACCGAGCAAATAATAAAAATTAAGGGAAATACTTTACTCCATATATATGTCTAT
4A-1007510-T10-44_1536	TGACCGAGCAAATAATAAAAATTAAGGGAAATACTTTACTCCATATATATGTCTATATATATATGCATGCGAATTTGTGGGGGAAATGTAGTTATCAGAATAAATGCATAATAAAGATGACG
4A-1007510-T10-44_1580	ATATATGTCTATATATATATGCATGCGAATTTGTGGGGGAAATGTAGTTATCAGAATAAATGCATAATAAAGATGACGATAAGATCAGTGCTACATATTCAGAAATTAAGAAATTCCTTAAT
4B-1071540-T10-44_64	GAATTATACAGTGATGAACCTTTCTCCCATGCCATGCTTTCCAAAAGCTGTTCTTCTAATGAATTTAAATGTCTTTTCAATTCTCTTGGAAGGCTTTCTTCATGTCTCACGAAGTTCTCA
4B-1071540-T10-44_128	TTAAATGTCTTTTCAATTCTCTTGGAAGGCTTTCTTCATGTCTCACGAAGTTCTCAATTATCTTGCTCAAATCAAATGCAGTTAGACCAGTACTCTCCAAGACAGCAGGGAACATCATGA
4B-1071540-T10-44_320	ACAACAATGGAGTCAGATTATTAACATTAGTATTGAGTGATTCTGCTCTGCAAATTGCCTCCAACACCCTATAGTACAACTTGGAGGCCTCCATCTTTCTTGCTTCGGCCCATGGAATAT
4B-1071540-T10-44_448	TTTGTACAATTCAGGCCCATGGAACCAGCCCAATGGCCAGAAGGCTTAGTTGGAAAAAATTGCTTCCAAAATTATGCTGACCCTTTTTGTTACATCACTTGTCAAATCAGTATCACAAGAC
4B-1071540-T10-44_512	CCAAAATTATGCTGACCCTTTTTGTTACATCACTTGTCAAATCAGTATCACAAGACGAAAGAAATTCCTCAAGCTTAGACGAAGGCTTCTCTGGCAACAAAGATATCACCTCACGAAGCC
4B-1071540-T10-44_768	AAAGACACGCTGCAAAATGAGAGGGTCAAAATCATGTGGGGAAGGAATTCAATCTAGACTACTGACAGAATTATGAATTACTGAGGATACCTTCGGGCAGCTTAGATTAGTGAAATCTCT
4B-1071540-T10-44_832	ACAGAATTATGAATTACTGAGGATACCTTCGGGCAGCTTAGATTAGTGAAATCTCTAGAAGAGTTCTCAGCACTGATTATATTGTCATTATTGATCAAAAACCTTTTCAAATCAAGCTCC
4B-1071540-T10-44_960	GCTAGTGGTACTACTTAGCTTCTCCAACTTCTCAAAATTTGAATGAAAACACTCCTTATCAAGGAGATCTCTGAAATAAATTAGACCATCTGCCACCATAACAACACAACACTTAAGCTA
4B-1071540-T10-44_1024	AGATCTCTGAAATAAATTAGACCATCTGCCACCATAACAACACAACACTTAAGCTAATTAAACATAGCAAAATAACCGAAACTCAATAGGTCTGTGTGCACGCGAGAGAATGGATATACG
4B-1071540-T10-44_1088	TAGCAAAATAACCGAAACTCAATAGGTCTGTGTGCACGCGAGAGAATGGATATACGGGTCATGCCTGTATCTATTTTATCCAAAGTTTCTATTTTGCACTCTGAAGCCCTTACTGCTTTC
4B-1071540-T10-44_1280	TAGTTATGACATAACGAAGCAAGGAGATCAACACCCCTCACCGTTTGTTTTACTGAATTGGATTAGAACAGCAAAACAAAATTGTAACTATGTGTTGCATAATGACAACTAAATTAAGGA
4B-1071540-T10-44_1344	AGAACAGCAAAACAAAATTGTAACTATGTGTTGCATAATGACAACTAAATTAAGGAGTGTGCCTTCAAGAAATAATAACTGATATAAAGGTGGGAATATTTACTTGAGTGAGAAGAACCT
4B-1071540-T10-44_1408	TCAAGAAATAATAACTGATATAAAGGTGGGAATATTTACTTGAGTGAGAAGAACCTTCGATTGTGAAGCTCCTAAATTTAGCAGGTACATGAATTAAAAGTATGGCCTGCCACAGATTAA
4B-1071540-T10-44_1472	GAAGCTCCTAAATTTAGCAGGTACATGAATTAAAAGTATGGCCTGCCACAGATTAAACAATTTTGACAAATAAGCCCAAGAAAGGATAGAAAGGATAAGAGAAAGTGGTGGCTATTTGG
4B-1071540-T10-44_1529	CAATTTTGACAAATAAGCCCAAGAAAGGATAGAAAGGATAAGAGAAAGTGGTGGCTATTTGGGAAACTGGTCACTTGAATATAAATACCAGCTCGTATTAAAAAAAATGAACTGTACCAG
4D-1118460-T10-44_64	GCCGACGCCTCGTATTGAACCGCTTGAGAATTCACCCGATTGCGCAGCTCCGGCGAGCTAGGGTTTGGGGGTTTGGGGATCGGGATTCAGAAATACTGTAACTTGTTCAAAGGGGTTTA
4D-1118460-T10-44_256	ATTCAAAAGGGGCTGGACTGCAAAAGTGGGCGCTGTTGTTTCCATTTTGGACCCACTGCTCCCAGCATATTTCTGTCCAAAACTAGTAAAACTGCACTGCACCGTGTGTTGCAATGGGTA
4D-1118460-T10-44_320	GCATATTTCTGTCCAAAACTAGTAAAACTGCACTGCACCGTGTGTTGCAATGGGTAAAAATTATCCCTACTACTACCGAAGCAAAAACTTGCACCCTTTTGCTAAAGGGCACAATGTGTA
4D-1118460-T10-44_384	CCCTACTACTACCGAAGCAAAAACTTGCACCCTTTTGCTAAAGGGCACAATGTGTAAAATGCGAGAGTCAATCTTTATTCTTTAAAAAAACCAACTTGGACTTTATTAATTAGAAATAAT
4D-1118460-T10-44_1344	TATCGGGATCGCTGGGACTCTATGAGCATCAATTGGCCACAACATTTGTCTCACCAATTCGTAACTAGGTCAATAAGATCAGAAACTTTCGTCATGTGATTCCCTCTTCTAGGTGTAATG
5A-1257070-T10-45_1344	GCGGGATGAGGCTGATTTGCACGAGCTCCACTGGTGGTCAACTGTGGTTTTCATTTTTTTTATTTGAGGGTGTTTGTGTAAAATTTGAGGGTCTAGTTTGTAATTTTAGATTAAAACCCA
5B-1319020-T10-45_1054	GGTCCACATTTGATGACAAACTATGTATAGACAACTTGGTTGGACGTTTGCGGGTGTTTTGAATGTCCGCGGTGGAGATACACCAACCCCTTGTTGTTACCTGTGTTTTGCAACCAGACG
5B-1319020-T10-45_1116	ATGTCCGCGGTGGAGATACACCAACCCCTTGTTGTTACCTGTGTTTTGCAACCAGACGATATGACATCATCCGGAAGTTTCAGTTCGTAACCGTAGCAGTAGTGATTCTTTGATCACTGC
5B-1319020-T10-45_1178	GACATCATCCGGAAGTTTCAGTTCGTAACCGTAGCAGTAGTGATTCTTTGATCACTGCATTTTGCACCGTCGTCACTGAAGATTATATTCCGATCGTGCCGGCCACCGTCGGGTCACTCG
5B-1319020-T10-45_1240	TGCACCGTCGTCACTGAAGATTATATTCCGATCGTGCCGGCCACCGTCGGGTCACTCGGGTGGCGCGACGTGCCGTGGAGACGATGAGTAGGTCATCGTCGATCGTTTCTTTCCACAGAC
5B-1319020-T10-45_1426	GAGATTATATTCCCAGCTCTGGACGCTTTCCGTTTCACGTCGCCGTCGCCACTCCCGGCTTACGCGCGTGGCACCCTCTATAAATTCGGCACGATTTCTCTTGGTTTCCACACAAGGAGC
5B-1319020-T10-45_1488	CGCGCGTGGCACCCTCTATAAATTCGGCACGATTTCTCTTGGTTTCCACACAAGGAGCAGCAAAGCTTCCTCAAGGTCGTGTGGGTTACTGGGTTTAACTAGCTTGGCTCGGCCGCTTCC
5B-1319020-T10-45_1674	GAAAGAGCTAGCTAGTAGAGCCGGCCATGCATGGCACAGATACCTATCATACACGACTGAATTTTGTCTTGTCAAACATTTCATCGGTGCGTTTTTTTTGTCTCTCCAGGCTCAATCCGG
5B-1319020-T10-45_1844	GCCATCCTCGAGCTCCTGCCCCAGTTTGATCACCACGCATCAACGGAACAGGGTACTGATTTTGTGGCCATCTTCCGATGGAAGGCCGTCTCTCTCTAGCTCACCCACGTGCCTTGCTGC
2A-0269760-T10-46_64	AGGGGAAGGCGGAGCCGTGGAGGAATAGGAGATTTTTTTGGGGGAAGGAGGCTGTGAGAGGATTTGGGTTTCCCGCGCTTGGGGTACCTTAAGGCCTCTTTGGATTGAAGGCATAG
2A-0269760-T10-46_512	CTTCCATTTAACTTCCAATGATTTTTTCTGTAAGCTCCGGGTGGGTTTAGCGACCTGGATGGGCTCAATGGGCACCCATGTTTTTGACCAAATCCTTATCGACCCAGCAGCATGCATATG
2A-0269760-T10-46_576	TCAATGGGCACCCATGTTTTTGACCAAATCCTTATCGACCCAGCAGCATGCATATGTGGTCTTTCCTATGGTTGCTCAAAAAAAAAAAGTGGTCTTTCCTATTCGACACTCTCTGTGTTAA
2A-0269760-T10-46_640	CCTATGGTTGCTCAAAAAAAAAAAGTGGTCTTTCCTATTCGACACTCTCTGTGTTAAATAGAGAAAAATCACTAGTTAAGAGTTAACCCTAGCAAAGGTCACTCCCTACCTTCTtactccc
2A-0269760-T10-46_832	AGACAAGTAATTCCGAACGGAGGGAGTAGGTTCTTGCTGTGTCGCTTGTTATAACTTGGAAGTTTTTCCTTTTTTAGATTTGTTCATTTTAAAAAAATTATCTTCTAAActgttttcact
2A-0269760-T10-46_896	TTTCCTTTTTTAGATTTGTTCATTTTAAAAAAATTATCTTCTAAActgttttcactattggatttctcatgtcgagatcttcaaaactaCTCCCTCCGTATCATAATGTAAAATGTTTTT
2A-0269760-T10-46_1024	ACTAGTACAAAAAACGTCTTATATTAATGGACGGATGGAGTAGATCCCATGTTAATAGTTATATCACAAAATGagagagagaaaaaaaatcagaaaccccaaaaagaaatgaaaaaagaa
2A-0269760-T10-46_1088	CACAAAATGagagagagaaaaaaaatcagaaaccccaaaaagaaatgaaaaaagaagaaaaaaaacagaaactgaaaaaggaaaaaggaaaaaaacaacaaaGCCTAGAAGCACTGTAGG
2A-0269760-T10-46_1152	acagaaactgaaaaaggaaaaaggaaaaaaacaacaaaGCCTAGAAGCACTGTAGGGTTTTCAATTGTTCTTTTGGTGTGATTTTCCTTTTTATTCATGGGAAGCACGGTTGTGCTTTTC
2A-0269760-T10-46_1216	TTGTTCTTTTGGTGTGATTTTCCTTTTTATTCATGGGAAGCACGGTTGTGCTTTTCCCTTTTCGAGAAGCACAATTATGCTTATCATAAAAGCGAAATGTTATGCTTTCGACGGAAGCAA
2A-0269760-T10-46_1280	AGAAGCACAATTATGCTTATCATAAAAGCGAAATGTTATGCTTTCGACGGAAGCAAATATATGCTTCCACGAGCTCGCGGAGGTAAATTTGTGCCTCCATAAGAAGCACAATCGTGCTTC
2A-0269760-T10-46_1344	TTCCACGAGCTCGCGGAGGTAAATTTGTGCCTCCATAAGAAGCACAATCGTGCTTCCTGAATAAGGGAAGGTCAAAAAAATCTAAAACCCGAAAATGCGTACAGAAAAATAAAAAACATA
2A-0269760-T10-46_1536	AAAAAGTGACCCTTGCGGAAACCCCACAGGGGGTAACCCTTAGTTAGTTGCTCCCTAGAATTGAGTCGGTTCAGTAGTGGGTTTCAAGTTGTTCATTCGTGCAACATTGTCCCAAACCCT
2A-0269760-T10-46_1580	TAGTTGCTCCCTAGAATTGAGTCGGTTCAGTAGTGGGTTTCAAGTTGTTCATTCGTGCAACATTGTCCCAAACCCTATTTAGCACCTTCTCTGATTGTTACAATGCATTTGACAAACCAG
2B-0435980-T10-46_64	TTTTTGACACTAAACGTCTTACATTATGGGACGAAGGGAGtagttttgaagatctcgacacgagaaatccaatagtgaaaacagtttagaagataaaacattttgaataagtgaatctag
2B-0435980-T10-46_128	aaatccaatagtgaaaacagtttagaagataaaacattttgaataagtgaatctagaaaAGGAAAAACTTTCAAGTTATTACAAGTGACGCAGTAAGAACCTAAGAAGGTAGGGAGTGGC
2B-0435980-T10-46_192	AAACTTTCAAGTTATTACAAGTGACGCAGTAAGAACCTAAGAAGGTAGGGAGTGGCCTTTGCGAGGGTTAACTCTTAACTAGTGATTTTTCTCTATTTAATTAACATAGAGAGCGTCAAA
2B-0435980-T10-46_256	GGGTTAACTCTTAACTAGTGATTTTTCTCTATTTAATTAACATAGAGAGCGTCAAATACGAGAGACCACATTTGAGAGCTCCTAATCAGCGCCTTCAGAGCCGATTAGGAGAAGTGGCGC
2B-0435980-T10-46_960	AAAAGTTCACTGAATTTCAGAAAAGTTCATCGATTTTTGAAAAAAGTTCATCACATTTGGAAAAAATAATCATTTTTTGCATTGATTTGAAAAATGTTCACGCACTTAAGAGAAGATAAA
2B-0435980-T10-46_1024	AATAATCATTTTTTGCATTGATTTGAAAAATGTTCACGCACTTAAGAGAAGATAAAAATGGAAGTCAAGGAGGGAGGAAAACCAGgaagaagaacaaaaaagaaaaaagaagcagaaaat
2B-0435980-T10-46_1088	TCAAGGAGGGAGGAAAACCAGgaagaagaacaaaaaagaaaaaagaagcagaaaatgaaaaccaaaaaaagagaaaggaaagtataaaagaaTGAAAGAGTTGAATCTGAGAAGATGCCG
2B-0435980-T10-46_1152	aaaaaagagaaaggaaagtataaaagaaTGAAAGAGTTGAATCTGAGAAGATGCCGCTCCATAGCGGGGTGGGTAATACGTCGAACCTCAGCGAGCGAGGTGGTGGGTTCGATTTGCAAA
2B-0435980-T10-46_1216	CGGGGTGGGTAATACGTCGAACCTCAGCGAGCGAGGTGGTGGGTTCGATTTGCAAAGCTCAATTATTTATTGCGGATTTAAATACACCGTAATGGCTCGGCCCAGCTTGACACGCTGCAG
2B-0435980-T10-46_1344	TTGCAAAAATACAGGCGCCTGCAGCGTCAAATAGGATTCGCCACCACATTTACCTACCCCGGAAAAAAAAAAGACCACACTTGTTTTTTGAGCAACTATAGGAAAGACCACATATGCATAC
2B-0435980-T10-46_1408	AAAAAAAGACCACACTTGTTTTTTGAGCAACTATAGGAAAGACCACATATGCATACTGCTGGGTCGATGAGGATTTGGTCAAAAACATGGGTGCCCATTGAGCCCATCAAATAGAAGCCC
2B-0435980-T10-46_1472	CGATGAGGATTTGGTCAAAAACATGGGTGCCCATTGAGCCCATCAAATAGAAGCCCATCTGCTGACCCTAAAGGTACCCCAAGCGCGGGAAACCCAATTCCTCTCAACTCTCATAGTCTC
2D-0513310-T10-46_0	TTCAAGAATTTGAAAAAAGTTCACGCACTCAAAAAATATTTGTGGATTTTATTTTATTTTGTATGTGAAAAAGTCCACAGATTTTTAAAAATTCACAAATTAAAGAAAGTTCACACATTT
2D-0513310-T10-46_64	GTGAAAAAGTCCACAGATTTTTAAAAATTCACAAATTAAAGAAAGTTCACACATTTGGAAAAATAGTTTGCGAAAAGGGCAAAAGAACCTAAATAGAAAACCCCCAAACTATCAAAAG
2D-0513310-T10-46_128	TAGTTTGCGAAAAGGGCAAAAGAACCTAAATAGAAAACCCCCAAACTATCAAAAGCTTCTAGAAGCTTCCTAAAACCGGCTGGAATCTTCCAGAAGCTTCACAAAATTGGTTGGGAAAG
2D-0513310-T10-46_320	AAACAAAATTGTTGCCACGAAAGAGAGAAAATAAATAAATGGGTCGAGCGCAAGGTGGAGAGGGGTTGTACGCTGGTTTGTCAAATGGATTGTAACAATCACAGAAGGTGCTAAATAG
2D-0513310-T10-46_384	GTTGTACGCTGGTTTGTCAAATGGATTGTAACAATCACAGAAGGTGCTAAATAGGGTTTGGGACAACAGTGCACGAACGAACAACTTGAAACCCACTACTGAACCCACTCAATTCTAGGG
2D-0513310-T10-46_448	AACAGTGCACGAACGAACAACTTGAAACCCACTACTGAACCCACTCAATTCTAGGGAGCAACTAACTAAAGGTTACCCCTTGTCGGGTTCCTGCAAGGGTAACTTTTTTGGCATGAGAG
2D-0513310-T10-46_576	TGGTGCGCCCAGCCGTCGCCACATGCCGCATGCTGGGCACTTCATATGGATTTCATTTTTTGTTTTTCCGTACGCATTTTCGGGTTTTAGATGTTTTTTTAACCTTCCCTTATTTGCGA
2D-0513310-T10-46_640	TTTTCCGTACGCATTTTCGGGTTTTAGATGTTTTTTTAACCTTCCCTTATTTGCGAAGCATAATTGTGCTTCTCATGAAGGCACAAATTCACTTCCGCGAGCTCGTGAAAGCACCTATTT
2D-0513310-T10-46_704	TGTGCTTCTCATGAAGGCACAAATTCACTTCCGCGAGCTCGTGAAAGCACCTATTTGCTTCCGTCAGAAGCACAACATTTCCTTTCACGATAAACATAGTTATGCTTCTCGGAAAGGAAA
2D-0513310-T10-46_768	CAGAAGCACAACATTTCCTTTCACGATAAACATAGTTATGCTTCTCGGAAAGGAAATGAAAAATCCAACTAGGTTTATCGTGCTTCCCGTGAATAAAAAGAAAAGCACAACATGTGCTTC
2D-0513310-T10-46_832	CCAACTAGGTTTATCGTGCTTCCCGTGAATAAAAAGAAAAGCACAACATGTGCTTCTACGAGAAGCACAACTTGCACTTCAAAAGAAGATTTGAAAGCCGCACAGTGCTTCCAGGCTTCG
2D-0513310-T10-46_896	GCACAACTTGCACTTCAAAAGAAGATTTGAAAGCCGCACAGTGCTTCCAGGCTTCGTTGTTTTCTTCCTTTTTTTCAATTTCTGTTTTTTTTCTTTTTCTTCTGATTTTTTCATTTCTTT
2D-0513310-T10-46_960	TTCCTTTTTTTCAATTTCTGTTTTTTTTCTTTTTCTTCTGATTTTTTCATTTCTTTTTCTAGGTTTTCTGATTTTTTTCTCTCTCATTTTTTTATCAAAAACTATTAACATAGATCTACT
2D-0513310-T10-46_1024	TTTCTGATTTTTTTCTCTCTCATTTTTTTATCAAAAACTATTAACATAGATCTACTCTACTCCCTCCGCTCCATAATGTAAGACATTTTTTGACACTAAATGTCTTACATTGGGACGGAG
2D-0513310-T10-46_1088	TCCGCTCCATAATGTAAGACATTTTTTGACACTAAATGTCTTACATTGGGACGGAGGGAGTAGTTTTGAAGATCTCGAAATGAGAAATCCAATAGTGAAAATAGTTTAGAAGATAAAAAC
2D-0513310-T10-46_1152	TTTGAAGATCTCGAAATGAGAAATCCAATAGTGAAAATAGTTTAGAAGATAAAAACATTTTGAATAAATGAATTAAAAAAGGAAAAAACTTCCAAGTTATAACAAGTGACACAGTAAGAA
2D-0513310-T10-46_1216	TAAATGAATTAAAAAAGGAAAAAACTTCCAAGTTATAACAAGTGACACAGTAAGAACCTAAGAAGGTAGGGAGTGGCCTTTGCGAGGGTTAACTCTTAGTGATTTTTCTCTATTTAACAC
2D-0513310-T10-46_1280	GGTAGGGAGTGGCCTTTGCGAGGGTTAACTCTTAGTGATTTTTCTCTATTTAACACAGAGAGCGTCAAATACGAGAGACCACATTTACCCCGCAAAAAAAAAAACACATTTGTTTTTTGA
2D-0513310-T10-46_1344	TCAAATACGAGAGACCACATTTACCCCGCAAAAAAAAAAACACATTTGTTTTTTGAGCAACTATAGGGAAGACCGCATGCTGCTGGGTCGATAAGGATTTGGTCAAAAACATGGGTGCCC
2D-0513310-T10-46_1472	CATCCAGGTCTCTAAGCCCACCCAGAGCTTACAGAAAATTTCACTGGAAGTCAAATAGAAGCCCATCTCCTGACCCTAAAGGTACCCCAAGCGCGGGAAACCCAAATCCTCTCACAGCCT
7A-1789360-T10-47_66	CATCAGCCACCCATGAGCTAAATCAATGCGATGATGCCAAAGTAGTACTACGCGACTACAGGTTGGAACATTGATGCCGGATGATATAACCTGCTTCTCTTTTCTGGTGACGCGGGAGAT
7A-1789360-T10-47_198	CACACGCCTATCCTAAAAGTGCAAAACCTTTTTTTTCTAGTACATTTTGCTCATCTAGCATATTCTCTGTCCTAAAAAGTTCATTTTGTCACTTCTTCAAGATGGTTTTGAAGTTATCAT
7A-1789360-T10-47_264	CTGTCCTAAAAAGTTCATTTTGTCACTTCTTCAAGATGGTTTTGAAGTTATCATGACATATTAATAAAAAAACAACAGTTAAGTGCACACAATCCCAATATGTTAAAAATAATGTTTTCA
7A-1789360-T10-47_330	AAAAAACAACAGTTAAGTGCACACAATCCCAATATGTTAAAAATAATGTTTTCATTCCACAAACACACAATATGTGATCTAAATGAACTAAGAATCCAAATAAACATGAAAAAGGCCCTT
7A-1789360-T10-47_396	ACAATATGTGATCTAAATGAACTAAGAATCCAAATAAACATGAAAAAGGCCCTTCCCCCCTCCAATCATTTTCTGCCATTACGGCATATTCAACACTGTGCATCAAAATTCCTGAATGTA
7A-1789360-T10-47_852	ACATATGCGAACCGGTCTGGACATCTAAAATCCTACAAACCGTAGAAAATTTATGGGTGTTCGAAACATCTGCTATGTAGACGCCGACCGAGAGGTTCCACCCAAGGCCCTCGTTGCATG
7A-1789360-T10-47_913	CGAAACATCTGCTATGTAGACGCCGACCGAGAGGTTCCACCCAAGGCCCTCGTTGCATGTTCGGTCCTAGAATCATCATCGCACATTCATGCTACTCATTAGCCCGCTACGGGCGGCTGC
7A-1789360-T10-47_974	CGGTCCTAGAATCATCATCGCACATTCATGCTACTCATTAGCCCGCTACGGGCGGCTGCCAACCACATTCATTCCGATCAAACCGATGTGATTGGACGGCTGACACGGCTATCAAGAAGC
7A-1789360-T10-47_1035	ACCACATTCATTCCGATCAAACCGATGTGATTGGACGGCTGACACGGCTATCAAGAAGCCTAACTGGTCTGGATGGCGCAAAAAAATCAGATCGTGAAAATCTTAAGAAGCGGGATCCTC
7A-1789360-T10-47_1340	GCGCATATGTATTCATATGTCACCATTGTACACGCCCTTAAAATGGAGTGGAATGAGAAACTTTGACCAAAAGTCAAAACACTAGAACATCAGCGGCGACGCACAACTCCTGCTCAACGC
7A-1789360-T10-47_1401	TTTGACCAAAAGTCAAAACACTAGAACATCAGCGGCGACGCACAACTCCTGCTCAACGCACCCCCTCCTGCTCAGGCTGCCGCACGCACCTGCCGTGTCCACCTCGTCCGCAACCTTTGG
7B-1875310-T10-47_192	CGGCCAAAGGTTGCGGACGGGGTGGACACGGCAGAAACGAGGAGTCCCGACGAAGAAGAACCGGAGCGGCAGTGGCGGCGACGGGGGCAGCTGTGGGGCACGCGGTGGTAGGG
7B-1875310-T10-47_384	GGCGCAATCCAGGAGATGTGCGTGCGGCAGCCTGAGCAGGAGGGGCACCTGCAGCGTGCTTGGGCAGGAGGTGTGCCGTGTGCGTCGCCGGCTTGCGTGCGCTGCTGTACCCAGTGT
7B-1875310-T10-47_448	CAGGAGGTGTGCCGTGTGCGTCGCCGGCTTGCGTGCGCTGCTGTACCCAGTGTAAAGACTAAAATCGGCAGAAATCACATATTTGACCCCAAATCAAATCACAAAATGACCTGCTCACGA
7D-1971250-T10-47_192	GGACGGGGCGGACACGGCAAGGACGAGTCCCGACGAAGAAGAACCGGAGCGGCAGTGGCGGCGACGGGGGCAGCTGTGGGGCACGCGGTGGTAGGGTTTAGAGGTTTAGGGCCC
7D-1971250-T10-47_320	ATGGAACGCGTGCGGCGACGAGATGCAGAGGCGAGGGAGAGGGAGGCGGGCGCGATACAGGAGgtgtgcgtgcagcgtgcttgagcaggaggtgtgcgtcgccggcgtgcgtgtgcgcgt
7D-1971250-T10-47_384	tgtgcgtgcagcgtgcttgagcaggaggtgtgcgtcgccggcgtgcgtgtgcgcgtgcgctgcTGTACCCAGTGTAAAGGCTAAAATCACAGTTTTGACTTTTGGTCAAAGTTTAGCACC
7D-1971250-T10-47_448	GTACCCAGTGTAAAGGCTAAAATCACAGTTTTGACTTTTGGTCAAAGTTTAGCACCATTTTGAAAGATTTTCATTCCTTTGGGACGCTATCCAGGCCGGAATAGGCTTCTCGGTAGCCGT
7D-1971250-T10-47_512	AGATTTTCATTCCTTTGGGACGCTATCCAGGCCGGAATAGGCTTCTCGGTAGCCGTGCCAGCATTGACGCTGCGCTGGTCAGCCGTCCGGTCACGTCGGTTTGACCGGCATGAATGCGAT
7D-1971250-T10-47_576	TGACGCTGCGCTGGTCAGCCGTCCGGTCACGTCGGTTTGACCGGCATGAATGCGATAGGCGTCCGCCCCTTAGCATCTGCTATGAACTGCACTAACGAGTGGCACGAATGTGCAATGATG
7D-1971250-T10-47_640	GCCCCTTAGCATCTGCTATGAACTGCACTAACGAGTGGCACGAATGTGCAATGATGATTCTGGAGCGGAACGTGCCACAAGGGTTTTGGATGGGACCGTCAGTTGGTGCCTACGTGGCG
7D-1971250-T10-47_704	GCGGAACGTGCCACAAGGGTTTTGGATGGGACCGTCAGTTGGTGCCTACGTGGCGGACGTCCGGACACCCACAAACCTCCTCCAGTTTGTGGGATTTTAGACGTTCAAACCGGCAGTG
7D-1971250-T10-47_768	ACACCCACAAACCTCCTCCAGTTTGTGGGATTTTAGACGTTCAAACCGGCAGTGGCGGAGGACCTAGCAGGGCAAGGGTTGGCGGCCATCCCTGCCTTGATTTGTCCGATGCATGCAGt
5A-1203580-T10-48_0	CCATGGCGAACGCGCGGGGCTGGCTAGGCTAGGCTAGGGTTTTGCTGTCACCGGCGAGGGAGTAAGGATGGGCAGATGTGGACGGATGAAAGAAGTGGATGAGGCCGACTGTGGGC
5A-1203580-T10-48_192	GCGGCCGTCACACGGGCCTAAGGCGGACACTGAACAGACACACGGCTCGTCCATTTAATATCCGTGTGAATGCAAGTCGGACGTAAATATGGACCAGAAATGCGTCTAGACGGACGACG
5A-1203580-T10-48_256	TGTGAATGCAAGTCGGACGTAAATATGGACCAGAAATGCGTCTAGACGGACGACGAGCAGACGAATTTTTGATTTGCATCGGCAGGTTGGGCCGGCGTCCATCCATTTGCATGGGCGGG
5A-1203580-T10-48_384	CGTCCATTTTATATAATGCAAATCGAACGTAAATATGGACCGGAAATGCGTAGGGGACGAATTTTCGATTTGCATGGGCGGATTGGACCGGCGTCCATCCATTTGCATGGGCGGGTTGGG
5A-1203580-T10-48_512	CATCCACCGTTTTTGTCCGTCCAGTTGCCCTTACCCCTCCAATtcgctctctctctttctctcccctctACTTATCCCCATCGCCCGTATCCCCGGTTACTTGCTCCCTCTGGCTCCG
5A-1203580-T10-48_1088	CCCCTGCCTCTACTCATCTTTCTTGTTCATATATACTCAAGTCTCATCCCCTGCCTCTCCTCATCCTCCCAGCAAGCAATTGCTTAGCATTGTAACAGTCCTTGTTTTTTTTTTTTGGAA
5A-1203580-T10-48_1152	CCTCCCAGCAAGCAATTGCTTAGCATTGTAACAGTCCTTGTTTTTTTTTTTTGGAAAGTTTGCTAACCCTAGTTATATGTTGGTGCAGATCTTACTTTTCCTTGGGAATTTGAAAGACCT
5A-1203580-T10-48_1216	AACCCTAGTTATATGTTGGTGCAGATCTTACTTTTCCTTGGGAATTTGAAAGACCTATTTAAACTACATGTGTTGTACTTTATTGGCGTATAAACTATTTTTTTTTATTTTTTGCGTCAA
5A-1203580-T10-48_1280	TACATGTGTTGTACTTTATTGGCGTATAAACTATTTTTTTTTATTTTTTGCGTCAATATTATATGATGTCCCCTTGGATTTCTAGAATATTTGATAAAACATTGCTACATAATCATCCAA
5A-1203580-T10-48_1580	GAAACCAATTCAATACGGAGGCCTTGAATACAGACATCCAAACACAGCATTAATGTTATGATATGGTTTGCTATAGGGTTAAGGTTGAACGCCAATTGAGTTCGAGTGTTGGGATTCGGA
5B-2081210-T10-48_124	ACCCATGGCGAACACGCGGGCTGGCTAGTCTAGGCTAGTGTTTTGCTGTCACCGACGAGGGAGCGAGGATGGCCAGACGTGGACGAAAGTGAAAGAAGAAGTGGATGAGGCCGGCC
5B-2081210-T10-48_186	GCGAGGATGGCCAGACGTGGACGAAAGTGAAAGAAGAAGTGGATGAGGCCGGCCGTGGGCTCTGGAAGCTTTCATACGCTGTCATGCGGGCCCAAGGTAGATGGACGCATTGACTAC
5B-2081210-T10-48_310	TGAGGTAGTTGGGTGGCCGACACGCAGGCCTGAAGCGGACACTCAACAGACACGCGGCGCGTCTATTTCATATCCGTGTGGATGCAAATCGGACGTAAATATAGACCGAAAATGCGTCC
5B-2081210-T10-48_372	CTATTTCATATCCGTGTGGATGCAAATCGGACGTAAATATAGACCGAAAATGCGTCCAAACGGACAGACAAGCACGAATTTTCAATTTGCATCGGCTGGTTGGGCCGGCGTCCATCCATT
5B-2081210-T10-48_1116	TCTTGTTCATATACTCAAGTCTCATTCCCTGCCTCTCCTCATCTTCCCAGCAAGCAATTGCTTAGCATTGTAACAGTCCTTGTTTTTTTTGGAAAGTTTGCTAACCCTAGTTATATGTTG
5B-2081210-T10-48_1178	TAGCATTGTAACAGTCCTTGTTTTTTTTGGAAAGTTTGCTAACCCTAGTTATATGTTGGTGCAGATCTTACTTATCCTTGGGAATTTGAAAGACCTATTTAAACTATATGTGTTGTACTT
5B-2081210-T10-48_1240	AGATCTTACTTATCCTTGGGAATTTGAAAGACCTATTTAAACTATATGTGTTGTACTTTATTGGTGTATAAACTTTTTTTTGCGTCAATATTATATGATGTCCCCTTGGATTTCTAGAAT
5B-2081210-T10-48_1302	GGTGTATAAACTTTTTTTTGCGTCAATATTATATGATGTCCCCTTGGATTTCTAGAATTTTTGATAAAACATTGCTACATAATCATCCAATTATAGCAACACATTATACAACTTCTGTTT
5B-2081210-T10-48_1798	ACTTTCCCTCAACTACTCACTCGTTTCCTTCCAGTGTTCTTCTCCTGCAAATTGTAGCTAGTTTGCCTTCTAGAGTTTACAAACACCTAATTTTTCAATGTTGGAATTGCTACCGACAGT
5B-2081210-T10-48_1860	TTGCCTTCTAGAGTTTACAAACACCTAATTTTTCAATGTTGGAATTGCTACCGACAGTGTAATTTCAAGTTAAATTACTCCTTTTGTGCTGGCTTGATGGGATTCTTCATTTATTTGTGC
5B-2081210-T10-48_1922	TTTCAAGTTAAATTACTCCTTTTGTGCTGGCTTGATGGGATTCTTCATTTATTTGTGCATCTGACATAGACTTATACTTCctctgtttcaaaataattgaagtttaaggtttctcctaag
5B-2081210-T10-48_2170	ttgcatagacttggtcaaacgtaaataagtttgactgaagataatcctagaacttattattttggTTAGGAGGGAGTACTAGATTGGCAGAGCTCATATTTATGTGTTGTGGGGCCGTTC
5B-2081210-T10-48_2356	AGTATCAAAGATTGCCTTACTCCTCTTCAAAGAGAGGTTCCCCAGGGAATAAGTACTCTGGCTGCCTCGCTAATTTAAAAAAGGAGGTGGGGCTCCTCTTGTAATCTTGTGGTTGCATGA
5B-2081210-T10-48_2418	TGCCTCGCTAATTTAAAAAAGGAGGTGGGGCTCCTCTTGTAATCTTGTGGTTGCATGATAATGATATATATTTATTGCACACTGTTACCCCCTTCCAGTTTTGGGCTTTTGGCCTTGTTG
5B-2081210-T10-48_2480	GATATATATTTATTGCACACTGTTACCCCCTTCCAGTTTTGGGCTTTTGGCCTTGTTGCATGCCTTTACTTTGCGTTGCTTAGATTATGGTCTGCATTGTTGCAGGGTTCACACTGGACA
5B-2081210-T10-48_2604	CAAGGTCCAATCTTTTGTTGTCGTTACATGTTAGTTTTATCTTCCATACATGATTACTATCCTTCGTTGAACATTTACGTGTTTCCTCAAATGTTGTGTTAATTGGGCTGGCAATAAAGT
5B-2081210-T10-48_2666	TTCGTTGAACATTTACGTGTTTCCTCAAATGTTGTGTTAATTGGGCTGGCAATAAAGTGTTGCATTTTATGTAACTAAAAAGAATTTTACGAATTAAATAATATGTACTTTCTGGCAAGT
5B-2081210-T10-48_2728	CATTTTATGTAACTAAAAAGAATTTTACGAATTAAATAATATGTACTTTCTGGCAAGTGCGTTCAAATGGCACATTTCTTACTTAATTTCATGTGGCATACATGTATATTCCGTGTTCTT
5B-2081210-T10-48_2790	TCAAATGGCACATTTCTTACTTAATTTCATGTGGCATACATGTATATTCCGTGTTCTTATTATTTTCTTTTGTATTTCAGAGTTCAAAGTTTGTCAGCAAGAGGACAGCTTGTTTGATTG
5D-1395040-T10-48_64	AACGCACGGGCTGGCTAGGCTAGGCTAGGGTTTTGCTGTCACCAGCGAGGGAGCAGAGGTGGACATATGTCGACGGATGGAAGAAGAAGTGGATGAGGCCGGCCGTGGGCTCTGAA
5D-1395040-T10-48_256	CGACACACGGGTCTGAGGCGGACACTGAACAGACGCGCGGCGCGTTTATTTCATATCCATGTAAATGCAACGTAAATATGGACTGGAAATGCGTCGAGACGGACGACTAGCAGACGAAT
5D-1395040-T10-48_320	ATGCAACGTAAATATGGACTGGAAATGCGTCGAGACGGACGACTAGCAGACGAATTTTCGATTTGCATCGGCGGATGGGGCCGGCGTCCATCCATTTGCTTGGGTGGGTTGGGCCGGC
5D-1395040-T10-48_576	AGGAACAGAACCCACAACCTGATCAGGAGGTACGGATCGTTTCTCCTCCATCGCGTCGATTCCATCGATGCACAGCTTCCGTCGATCTATCTCACTACCCGTGGTGTTTAATTTGAATCG
5D-1395040-T10-48_1088	TTCCCCTGCCCCTACTCCTCTTTCTTGTTCATACACTCAAGTCTCATTCCCTGCCTCTCCTCATCTTCCCAGCAAGCAATTGCTTAGCATTGTAACAGTCCTTTTTTTTTGGAAAGTTTGC
5D-1395040-T10-48_1152	CTTCCCAGCAAGCAATTGCTTAGCATTGTAACAGTCCTTTTTTTTTGGAAAGTTTGCTAACCCTAGTTATATGTTGGTGCAGATATTATACTTTTCGTTGAGAAAATGAAAGACCTATTTA
5D-1395040-T10-48_1216	GTTATATGTTGGTGCAGATATTATACTTTTCGTTGAGAAAATGAAAGACCTATTTAAACTCTATGTGTTGTACTTTATTGGTGTAAACTATTTTTTGTTTTTTTGCGTCAATATTATATG
5D-1395040-T10-48_1280	GTGTTGTACTTTATTGGTGTAAACTATTTTTTGTTTTTTTGCGTCAATATTATATGATGTACCCTTGGATTTCTAGAATATTTGATAAAACATTGCTACATAATCATCCAATTATAGTAA
5D-1395040-T10-48_1580	GAAACCAATTCAATACGGAGGCCTCGAATACAGACATCCAAACACAGCATTAATGTTATGATATGGTTTGCTATAGGGTTAAGGTTGAACGCGAATTGAGTTCGAGTGTTGGGATTCGGA
