# Supplementary material for: Exploring the diversity of promoter and 5′UTR sequences in ancestral, historic and modern wheat
Source: Plant Biotechnol J. 2021 Sep 16;19(12):2469–87. doi: 10.1111/pbi.13672 (PMC8633512; doi:10.1111/pbi.13672)
Supplement: Supplementary file 13 — Data S3 Sequencing lengths, depths and homeologue specificity. [file PBI-19-2469-s016.pdf]

sheets 2 - 5	genes ordered by different parameters (shown in TAB)				
**trait gene**	specifying the trait category (T1 - T10), arbitrary gene no. and homoeologue				
**gene ID**	these have been updated to IWGSCrefseq1.1 gene annotation and can be directly used for searches inIGV, expression browser etc.				
**promoter**	length of sequence obtained in bp				
**5'UTR**	length of sequence obtained in bp				
**promoter + 5'UTR**	length of sequence obtained in bp				
**Exons /Introns**	length of sequence obtained in bp				
**total bp**	length of sequence obtained in bp				
**MYbaits**	number of high specificity baits				
**TARGET coverage (%)**	target coverage by MYbaits (Geneious)				
sheet 6:	coverage depths - the maximum number of aligned sequenced fragments				
**Trait**	trait category and gene number				
**gene ID**	as above, this includes the subgenome and chromosome number embedded in the ID: eg TraesCS**6A**02G189300 (T1-1) is located on Chr6A				
**CS**	observed maximum coverage depth			ABD	
**KR**	observed maximum coverage depth			AB	
**MDR031**	observed maximum coverage depth			A	
**ENT**	observed maximum coverage depth			D	
**ASP**	observed maximum coverage depth			B	
**APG**	observed maximum coverage depth			B?	
**normalised by CS depth**				expected ratio	cut-off ratio (50%)
**CS**	cells exceeding the cut-off value are coloured			1	
**KR**		A subgenome specific capture		1.5	0.7
**MDR031**		B subgenome specific capture		3	1.5
**ENT**		D subgenome specific capture		3	1.5
**ASP**				3	1.5
**APG**				1.5	0.7
**gene ID**					
expected ratios:					
KR 1.5 (6/4)					
exclude if <0.7					
MDR 3 (6/2)					
exclude if <1.5					
ENT 3 (6/2)					
exclude if <1.5					
ASP 3 (6/2)					
exclude if <1.5					
APG 1.5 (6/4)					
exclude if <0.7					

Lengths of sequence captured

Sequence lengths were determined in IGV only for CS. Sequence length averages were thus only determined for CS across all analysed genes, and not across accessions for individual genes, but generally, most accessions exhibited similar lengths to CS. Sequence lengths were determined by using the ‘Marking Tool’ in IGV across the annotated 5’UTR and CDS, and for the putative promoters starting from the 5’end of the 5’UTR.

trait gene	gene ID	promoter	5'UTR	promoter + 5'UTR	Exons / Introns	total bp	MYbaits	TARGET coverage (%)
T1-1A	TraesCS6A02G189300	1811	184	1995	266	2261	11	54.6
T1-1B	TraesCS6B02G215300	1645	167	1812	342	2154	11	54.6
T1-1D	TraesCS6D02G176900	1693	193	1886	210	2096	12	55.1
T1-10A	TraesCS2A02G295400	693	0	693	1687	2380	16	47.6
T1-10B	TraesCS2B02G311900	1209	124	1333	296	1629	9	43.8
T1-10D	TraesCS2D02G293200	962	0	962	1499	2461	18	57.7
T1-11A	TraesCS5A02G373900	1497	0	1497	0	1497	13	58.2
T1-11B	TraesCS5B02G375800	1552	297	1849	140	1989	11	54.2
T1-11D	TraesCS5D02G383300	1453	0	1453	0	1453	12	52.4
T1-12A	TraesCS1A02G088000	1779	194	1973	385	2358	10	47.5
T1-12B	TraesCS1B02G107000	1854	124	1978	407	2385	9	40
T1-12D	TraesCS1D02G089300	1714	307	2021	463	2484	11	54.6
T1-13A	TraesCS5A02G030300	2108	372	2480	0	2480	16	73.4
T1-13B	TraesCS5B02G029100	1694	363	2057	0	2057	13	57.6
T1-14A	TraesCS2A02G168600	2263	296	2559	0	2559	16	72.2
T1-14B	TraesCS2B02G194900	1785	315	2100	0	2100	15	68.5
T1-14D	TraesCS2D02G176200	1604	307	1911	0	1911	16	70.1
T1-15A	TraesCS3A02G194100	989	122	1111	168	1279	4	24.9
T1-15B	TraesCS3B02G223900	988	449	1437	117	1554	3	21.2
T1-15D	TraesCS3D02G197700	1345	421	1766	62	1828	12	64.9
T1-16A	TraesCS1A02G350200	1178	122	1300	1029	2329	22	67.8
T1-16B	TraesCS1B02G364500	1851	120	1971	0	1971	11	48
T1-16D	TraesCS1D02G353100	1509	156	1665	1099	2764	17	49.7
T1-17A	TraesCS2A02G176000	1107	434	1541	301	1842	6	35.8
T1-17B	TraesCS2B02G202300	1546	393	1939	1802	3741	22	54.8
T1-17D	TraesCS2D02G183400	1566	398	1964	1811	3775	24	62.6
T1-18B	TraesCS5B02G379300	1779	0	1779	311	2090	13	60.9
T1-18D	TraesCS5D02G385900	1978	116	2094	162	2256	11	53.4
T1-19A	TraesCS4A02G016300	1046	1094	2140	224	2364	15	69.6
T1-19B	TraesCS4D02G286600	2099	175	2274	276	2550	11	61.2
T1-19D	TraesCS4B02G287900	1721	182	1903	309	2212	14	67.9
T1-2A	TraesCS3A02G212900L	1605	157	1762	274	2036	8	42.1
T1-2B	TraesCS3B02G433000	551	296	847	142	989	5	22.1
T1-2D	TraesCS3D02G172900	1415	121	1536	343	1879	2	14.1
T1-20A	TraesCS1A02G083000	1016	336	1352	328	1680	9	43.1
T1-20B	TraesCS1B02G100400	1019	478	1497	405	1902	6	35
T1-20D	TraesCS1D02G084200	1243	321	1564	425	1989	9	49.2
T1-21A	TraesCS6A02G306200	1066	272	1338	0	1338	10	47.5
T1-21D	TraesCS6D02G285300	1403	204	1607	0	1607	8	43.3
T1-22A	TraesCS7A02G185300	520	90	610	219	829	3	14.6
T1-22B	TraesCS7B02G090500	435	94	529	83	612	2	10.8
T1-22D	TraesCS7D02G187000	1449	102	1551	21	1572	13	65.4
T1-23A	TraesCS5A02G221600	1516	267	1783	161	1944	16	74
T1-23B	TraesCS5B02G220400	1318	344	1662	199	1861	16	74.6
T1-23D	TraesCS5D02G229400	1710	258	1968	23	1991	11	50.1
T1-24A	TraesCS6A02G237700	1500	527	2027	286	2313	14	74.6
T1-24B	TraesCS6B02G266200	1005	596	1601	492	2093	8	49.9
T1-25A	TraesCS1A02G102400	1515	0	1515	158	1673	9	53.6
T1-25B	TraesCS1B02G113100	1325	60	1385	224	1609	5	28.8
T1-25D	TraesCS1D02G094200	1557	0	1557	341	1898	8	40
T1-26A	TraesCS5A02G025900	1546	381	1927	0	1927	14	69.2
T1-26B	TraesCS5B02G025100	1508	459	1967	0	1967	13	68.7
T1-26D	TraesCS5D02G033700	0	0	0	2218	2218	16	72.2
T1-27A	TraesCS6A02G378500	1606	231	1837	389	2226	12	67.1
T1-27B	TraesCS6B02G416400	1347	611	1958	378	2336	10	57.4
T1-28-2B	TraesCS2B02G566500	1726	56	1782	306	2088	16	66.8
T1-28-3A	TraesCS3A02G021900	1726	49	1775	344	2119	14	68
T1-28-3D	TraesCS3D02G024700	1287	56	1343	259	1602	7	39.5
T1-3A	TraesCS5A02G215100	814	318	1132	82	1214	6	35.7
T1-3B	TraesCS5B02G208700	1290	321	1611	0	1611	7	41.1
T1-3D	TraesCS5D02G216900	1239	333	1572	79	1651	7	35.9
T1-4A	TraesCS3A02G311100	1665	335	2000	281	2281	19	83.5
T1-4B	TraesCS3B02G161000	1173	56	1229	133	1362	4	24.9
T1-4D	TraesCS3D02G143300	1971	147	2118	236	2354	14	65.9
T1-4(B)D	TraesCS3D02G143500	940	61	1001	114	1115	4	24.9
T1-5A	TraesCS2A02G188500	1328	0	1328	0	1328	10	47.5
T1-5B	TraesCS2B02G405700	1649	0	1649	227	1876	14	65.9
T1-5D	TraesCS2D02G385500	823	160	983	477	1460	2	14.1
T1-6-1B	TraesCS1B02G479100	276	768	1044	250	1294	16	68.3
T1-6-7A	TraesCS7A02G000200	562	631	1193	291	1484	6	32.5
T1-6-7D	TraesCS7D02G000100	665	714	1379	451	1830	9	43.8
T1-7A	TraesCS4A02G012100	1560	259	1819	323	2142	20	84
T1-7B	TraesCS4B02G292300	1732	179	1911	305	2216	14	72.5
T1-7D	TraesCS4D02G290900	1730	140	1870	263	2133	17	80.5
T1-8-4A	TraesCS4A02G474000	1728	342	2070	129	2199	17	76
T1-8-7A	TraesCS7A02G017700	1687	329	2016	0	2016	16	76.7
T1-8-7D	TraesCS7D02G015000	1541	346	1887	0	1887	15	72.9
T1-9A	TraesCS7A02G260500	1637	124	1761	0	1761	12	61.6
T1-9B	TraesCS7B02G158500	1721	239	1960	0	1960	12	64.9
T1-9D	TraesCS7D02G261500	1364	553	1917	0	1917	12	64.9
T2-1A	TraesCS4A02G107400	1585	277	1862	92	1954	18	74.4
T2-1B	TraesCS4B02G197000	1604	255	1859	122	1981	17	70.6
T2-1D	TraesCS4D02G197300	1727	254	1981	48	2029	17	70.6
T2-10A	TraesCS1A02G351900	782	349	1131		2337	8	45.4
T2-10B	TraesCS1B02G371300	610	933	1543	0	1543	14	65.9
T2-10D	TraesCS1D02G358500	540	994	1534	1392	2926	18	56
T2-11A	TraesCS7A02G287500	0	2096	2096	312	2408	8	49.9
T2-12A	TraesCS7A02G441400	1750	306	2056	51	2107	11	53.4
T2-12D	TraesCS7D02G430700	1721	107	1828	0	1828	17	72.7
T2-13A	TraesCS2A02G220400	1868	105	1973	396	2369	19	84.7
T2-13B	TraesCS2B02G245900	1760	99	1859	175	2034	23	89.9
T2-13D	TraesCS2D02G226000	1831	178	2009	231	2240	24	93.6
T2-14A	TraesCS3A02G111400	1516	74	1590	205	1795	15	69.6
T2-14B	TraesCS3B02G132400	1792	155	1947	280	2227	16	70.1
T2-14D	TraesCS3D02G113600	306	70	376	253	629	1	7.1
T2-15A	TraesCS2A02G436100	588	267	855	463	1318	6	29.2
T2-15B	TraesCS2B02G457100	308	70	378	108	486	10	43.1
T2-16A	TraesCS3A02G220800	765	87	852	0	852	2	10.8
T2-16B	TraesCS3B02G251100	998	42	1040	253	1293	7	32.9
T2-16D	TraesCS3D02G231700	465	109	574	161	735	1	7.1
T2-17B	TraesCS3B02G262600	1606	60	1666	0	1666	15	66.4
T2-17D	TraesCS3D02G219900	544	0	544	270	814	2	14.1
T2-18A	TraesCS7A02G236200	1704	212	1916	183	2099	9	43.8
T2-18B	TraesCS7B02G134200	1547	120	1667	47	1714	12	64.9
T2-18D	TraesCS7D02G236300	2316	309	2625	114	2739	7	42.8
T2-19A	TraesCS6A02G178000	1757	212	1969	441	2410	10	47.5
T2-19B	TraesCS6B02G206800	1268	174	1442	471	1913	7	36.2
T2-19D	TraesCS6D02G166400	1516	182	1698	463	2161	11	51.3
T2-2A	TraesCS5A02G247200	1514	466	1980	356	2336	18	80.9
T2-2B	TraesCS5B02G244300	1704	42	1746	387	2133	14	59.3
T2-2D	TraesCS5D02G253700	1410	469	1879	432	2311	21	88.9
T2-20A	TraesCS3A02G494800	1844	10	1854	420	2274	21	95.5
T2-20B	TraesCS3B02G556500	1900	42	1942	231	2173	17	83.8
T2-20D	TraesCS3D02G501900	2082	0	2082	80	2162	19	86.8
T2-21A	TraesCS1A02G115500	2368	386	2754	0	2754	9	47.1
T2-21D	TraesCS1D02G116900	1877	189	2066	0	2066	7	39.5
T2-22A	TraesCS2A02G122200	1730	0	1730	3016	4746	45	85.3
T2-22B	TraesCS2B02G144300	596	79	675	54	729	9	38.7
T2-22D	TraesCS2D02G124900	1831	166	1997	1038	3035	21	70.7
T2-23A	TraesCS7A02G495700	2705	0	2705	0	2705	15	68.5
T2-23B	TraesCS7B02G401600	1351	629	1980	311	2291	11	57.9
T2-23D	TraesCS7D02G483000	1425	656	2081	94	2175	10	52.9
T2-24A	TraesCS2A02G235500	852	291	1143	1021	2164	13	65.4
T2-24B	TraesCS7B02G133900	1546	305	1851	168	2019	10	54.1
T2-24D	TraesCS7D02G235600	799	264	1063	214	1277	7	42.8
T2-25A	TraesCS7A02G133500	2100	68	2168	302	2470	24	99.1
T2-25B	TraesCS7B02G034600	1967	8	1975	367	2342	23	99.1
T2-25D	TraesCS7D02G133100	1854	0	1854	361	2215	25	99.5
T2-26B	TraesCS6B02G008200	1857	0	1857	351	2208	23	92.4
T2-26D	TraesCS6D02G000200	2013	0	2013	482	2495	26	100
T2-27A	TraesCS5A02G395200	1852	97	1949	46	1995	19	83.5
T2-27B	TraesCS5B02G400000	0	0	0	2160	2160	20	85.2
T2-27D	TraesCS5D02G404500	1892	141	2033	92	2125	20	80.7
T2-28B	TraesCS3B02G111100	2297	0	2297	475	2772	17	85.9
T2-28D	TraesCS3D02G096000	1667	0	1667	273	1940	11	65.3
T2-29A	TraesCS3A02G366400	0	0	0	1921	1921	11	61.4
T2-29B	TraesCS3B02G397900	1702	65	1767	157	1924	8	39.1
T2-29D	TraesCS3D02G359200	1859	63	1922	265	2187	6	39.1
T2-3A	TraesCS1A02G040600	310	298	608	280	888	2	14.1
T2-3B	TraesCS1B02G053600	1558	341	1899	318	2217	15	68.5
T2-3D	TraesCS1D02G041200	1628	299	1927	213	2140	10	47.5
T2-30D	TraesCS3D02G095700	2074	0	2074	268	2342	16	75.5
T2-31A	TraesCS2A02G159300	1812	84	1896	117	2013	14	76.3
T2-31B	TraesCS2B02G184900	2051	26	2077	119	2196	13	67.5
T2-31D	TraesCS2D02G166600	1856	37	1893	139	2032	8	43.3
T2-32B	TraesCS7B02G072000	1611	0	1611	492	2103	17	71.4
T2-32D	TraesCS7D02G168000	1789	0	1789	434	2223	12	55.9
T2-33A	TraesCS2A02G123800	959	857	1816	323	2139	15	73.7
T2-33B	TraesCS2B02G145700	1415	892	2307	331	2638	11	54.6
T2-33D	TraesCS2D02G126600	1133	842	1975	240	2215	11	63.8
T2-34B	TraesCS4B02G328100	1583	0	1583	251	1834	17	70.6
T2-34D	TraesCS4D02G325100	1749	0	1749	426	2175	19	81.4
T2-35A	TraesCS5A02G472000	1931	131	2062	113	2175	18	80.9
T2-35B	TraesCS5B02G484700	1939	103	2042	133	2175	19	81.4
T2-35D	TraesCS5D02G484500	1965	9	1974	0	1974	9	47.1
T2-36B	TraesCS6B02G003500	1901	0	1901	239	2140	17	76
T2-37A	TraesCS1A02G419600	297	1173	1470	476	1946	11	57.9
T2-37B	TraesCS1B02G449700	531	1258	1789	1201	2990	23	72.2
T2-37D	TraesCS1D02G427400	522	1292	1814	209	2023	15	76.2
T2-39A	TraesCS2A02G292900L	1597	0	1597	274	1871	13	58.8
T2-39B	TraesCS5B02G175800	1145	165	1310	151	1461	4	24.7
T2-39D	TraesCS5D02G182600	766	84	850	192	1042	6	29.2
T2-4A	TraesCS3A02G440800	1871	0	1871	1276	3147	24	78.2
T2-4B	TraesCS3B02G474900	1313	391	1704	1047	2751	19	69.6
T2-4D	TraesCS3D02G433400	1547	321	1868	1085	2953	24	80.7
T2-40A	TraesCS1A02G466500L	1790	0	1790	318	2108	16	83.3
T2-42D	TraesCS5D02G004100	1567	0	1567	213	1780	16	65.6
T2-43A	TraesCS7A02G120300	1596	144	1740	0	1740	12	55.1
T2-43B	TraesCS7B02G018600	1553	302	1855	0	1855	14	62.6
T2-43D	TraesCS7D02G117800	0	0	0	2013	2013	13	62.1
T2-44D	TraesCS5D02G004300	1065	0	1065	268	1333	11	43.5
T2-45A	TraesCS7A02G189000	2167	0	2167	0	2167	14	69.2
T2-45B	TraesCS7B02G093800	1313	285	1598	218	1816	14	65.9
T2-45D	TraesCS7D02G190100	1745	0	1745	0	1745	10	59.5
T2-46B	TraesCS7B02G746900L	2086	0	2086	100	2186	19	88
T2-47A	TraesCS6A02G307800	1346	0	1346	0	1346	13	56.2
T2-47B	TraesCS6B02G336400	2016	302	2318	265	2583	11	57.9
T2-48B	TraesCS1B02G398500	1376	556	1932	376	2308	21	91.1
T2-48B	TraesCS1B02G412400	1388	556	1944	352	2296	20	87.3
T2-48D	TraesCS1D02G000100	1030	605	1635	528	2163	18	73.2
T2-49A	TraesCS1A02G137200	1938	118	2056	229	2285	5	28.7
T2-49B	TraesCS1B02G155700	842	156	998	344	1342	6	35.8
T2-49D	TraesCS1D02G138100	2010	111	2121	293	2414	10	50.8
T2-5A	TraesCS7A02G287400	1894	441	2335	105	2440	13	58.8
T2-5B	TraesCS7B02G183300	1753	128	1881	299	2180	15	69.6
T2-5D	TraesCS7D02G284900	1446	262	1708	172	1880	9	37.2
T2-50A	TraesCS1A02G091500	1543	184	1727	142	1869	6	39.9
T2-50B	TraesCS1B02G119300	1508	366	1874	66	1940	10	57.4
T2-51A	TraesCS2A02G468800	1991	158	2149	296	2445	18	86.4
T2-51B	TraesCS2B02G491700	1878	240	2118	0	2118	17	76.4
T2-51D	TraesCS2D02G468900	3240	128	3368	66	3434	37	85.4
T2-52A	TraesCS1A02G353300	0	1970	1970	318	2288	21	88.9
T2-52B	TraesCS1B02G368500	1440	0	1440	0	1440	6	35.9
T2-52D	TraesCS1D02G300700	1596	346	1942	576	2518	11	60.1
T2-53-4A	TraesCS4A02G418200	1239	728	1967	275	2242	21	91.1
T2-53-7A	TraesCS7A02G070100	944	842	1786	230	2016	16	63.3
T2-53-7D	TraesCS7D02G064300	1315	865	2180	253	2433	21	91.1
T2-54A	TraesCS5A02G373600	0	1652	1652	458	2110	21	81.2
T2-54B	TraesCS5B02G390700	1371	16	1387	217	1604	17	70.6
T2-54D	TraesCS5D02G369800	0	1791	1791	802	2593	26	85.4
T2-55A	TraesCS5A02G549300	1905	141	2046	0	2046	15	76
T2-55B	TraesCS7B02G472300	1876	108	1984	0	1984	21	88.9
T2-55D	TraesCS5D02G535600	2022	72	2094	0	2094	22	88.2
T2-56A	TraesCS2A02G293400	1795	272	2067	1111	3178	24	71.4
T2-56B	TraesCS2B02G309500	1276	220	1496	0	1496	9	54.2
T2-56D	TraesCS2D02G290800	1561	277	1838	0	1838	15	71.8
T2-57A	TraesCS2A02G310300	1870	205	2075	394	2469	12	64.9
T2-57B	TraesCS7B02G327300	0	0	0	2314	2314	8	42.1
T2-57D	TraesCS7D02G308600	1813	148	1961	231	2192	12	63.9
T2-58A	TraesCS7A02G251400	1682	0	1682	0	1682	6	32.5
T2-58B	TraesCS7B02G139700	1958	0	1958	0	1958	21	81.2
T2-58D	TraesCS7D02G249500	810	134	944	341	1285	2	10.8
T2-59A	TraesCS1A02G247100	1700	110	1810	0	1810	9	47.1
T2-59B	TraesCS1B02G257700	1959	0	1959	183	2142	8	31.3
T2-59D	TraesCS1D02G246300	1241	128	1369	107	1476	2	10.8
T2-6A	TraesCS7A02G298600	1323	341	1664	111	1775	10	52.9
T2-6B	TraesCS7B02G188400	1058	335	1393	170	1563	10	50.8
T2-6D	TraesCS7D02G294300	642	808	1450	206	1656	6	32.5
T2-60A	TraesCS1A02G248700	315	290	605	1048	1653	2	10.8
T2-60A	TraesCS1A02G248800	1923	98	2021	160	2181	2	10.8
T2-60B	TraesCS1B02G246400	926	291	1217	1141	2358	15	52.4
T2-60D	TraesCS1D02G255800	1750	287	2037	0	2037	17	72.7
T2-61A	TraesCS6A02G175100	857	1142	1999	276	2275	12	57.2
T2-61B	TraesCS6B02G210000	763	728	1491	255	1746	9	43.8
T2-7A	TraesCS4A02G267500	510	129	639	507	1146	1	7.1
T2-7B	TraesCS4B02G047300	1707	142	1849	450	2299	11	54.6
T2-7D	TraesCS4D02G047300	1325	0	1325	265	1590	8	36.7
T2-8A	TraesCS6A02G309400	0	26	26	753	779	4	21.6
T2-8B	TraesCS6B02G339100	0	21	21	744	765	4	18.4
T2-8D	TraesCS6D02G288800	0	122	122	844	966	4	18.4
T2-9A	TraesCS3A02G036600	1556	0	1556	133	1689	12	61.6
T2-9B	TraesCS3B02G047300	2163	270	2433	243	2676	16	72.2
T2-9D	TraesCS3D02G043500	3216	1614	4830	150	4980	19	90.1
T4-1A	TraesCS7A02G264400	1986	0	1986	79	2065	18	74.4
T4-1B	TraesCS7B02G162500	0	0	0	1731	1731	11	51.3
T4-1D	TraesCS7D02G265400	0	0	0	1180	1180	9	39.8
T4-10A	TraesCS4A02G106400	1421	796	2217	222	2439	7	41.6
T4-10B	TraesCS4B02G197800	1661	104	1765	319	2084	14	65.2
T4-10D	TraesCS4D02G198600	1336	245	1581	206	1787	12	58.4
T4-11A	TraesCS7A02G311300	839	218	1057	49	1106	4	21.6
T4-11B	TraesCS7B02G009200	898	238	1136	47	1183	5	25.4
T4-11D	TraesCS7D02G106400	955	329	1284	122	1406	6	32.5
T4-12A	TraesCS7A02G373500	1104	0	1104	250	1354	13	52.2
T4-12B	TraesCS7B02G253500	1987	0	1987	401	2388	18	77.6
T4-12D	TraesCS7D02G349600	1930	0	1930	197	2127	22	91.5
T4-13A	TraesCS7A02G412400	1054	163	1217	181	1398	8	43.3
T4-13B	TraesCS7B02G311600	893	172	1065	332	1397	7	29.6
T4-13D	TraesCS7D02G405500	595	249	844	167	1011	6	25.9
T4-14A	TraesCS3A02G105400	1724	110	1834	111	1945	14	65.9
T4-14B	TraesCS3B02G158300L	1181	0	1181	0	1181	10	43.1
T4-14D	TraesCS3D02G107500	1738	109	1847	90	1937	12	58.4
T4-15-1D	TraesCS1D02G018500	734	0	734	278	1012	7	28.5
T4-15-6A	TraesCS6A02G083200	2033	152	2185	253	2438	15	76.2
T4-15-6B	TraesCS6B02G113900	2337	66	2403	384	2787	17	82.6
T4-16A	TraesCS7A02G310500	1329	433	1762	262	2024	6	36
T4-16B	TraesCS7B02G381100L	1013	0	1013	735	1748	15	72.9
T4-16D	TraesCS7D02G307000	922	0	922	234	1156	14	71.1
T4-17A	TraesCS1A02G131600	1477	0	1477	1818	3295	18	50.4
T4-17B	TraesCS1B02G154400	1494	202	1696	1804	3500	22	55.7
T4-17D	TraesCS1D02G136400	1838	102	1940	145	2085	22	89.4
T4-18-4A	TraesCS4A02G485400	1869	236	2105	75	2180	18	84.2
T4-18-7A	TraesCS7A02G009800	0	1939	1939	99	2038	24	92.5
T4-18-7D	TraesCS7D02G009400	1977	63	2040	0	2040	15	78.4
T4-19-4A	TraesCS4A02G486000	559	75	634	953	1587	4	28.2
T4-19-7A	TraesCS7A02G009100	546	72	618	859	1477	3	17.9
T4-19-7D	TraesCS7D02G008700	992	71	1063	1017	2080	8	38.8
T4-2A	TraesCS6A02G417900	0	0	0	2316	2316	19	90.6
T4-2B	TraesCS6B02G471100	1189	0	1189	983	2172	20	81.9
T4-2D	TraesCS6D02G403200	0	0	0	1748	1748	16	82.6
T4-20-4A	TraesCS4A02G485900	1885	127	2012	253	2265	14	69.2
T4-20-7A	TraesCS7A02G009200	1869	0	1869	270	2139	18	79.8
T4-20-7D	TraesCS7D02G008800	1734	190	1924	280	2204	17	82.6
T4-21A	TraesCS6A02G151700	1100	498	1598	283	1881	14	69.2
T4-21B	TraesCS6B02G179900	2221	0	2221	366	2587	5	28.7
T4-21D	TraesCS6D02G141700	467	158	625	1438	2063	9	50.4
T4-22A	TraesCS2A02G395000	1521	183	1704	29	1733	14	65.9
T4-22B	TraesCS2B02G413000	1788	0	1788	0	1788	12	61.6
T4-22D	TraesCS2D02G392800	1806	0	1806	0	1806	6	32.3
T4-23A	TraesCS4A02G216100	983	153	1136	351	1487	5	28.7
T4-23B	TraesCS4B02G100100	1609	166	1775	2337	4112	27	72.2
T4-23D	TraesCS4D02G096400	1639	165	1804	2302	4106	6	35.8
T4-24A	TraesCS4A02G097400	1705	60	1765	235	2000	14	49.6
T4-24B	TraesCS4B02G207100	1295	115	1410	202	1612	10	53.6
T4-24D	TraesCS4D02G207900	1215	96	1311	178	1489	4	17.8
T4-25A	TraesCS4A02G097500	1829	152	1981	165	2146	18	77.1
T4-25B	TraesCS4B02G207000	1618	169	1787	159	1946	13	57.6
T4-25D	TraesCS4D02G207800	1641	140	1781	175	1956	18	74.4
T4-26-4B	TraesCS4B02G322700	1868	221	2089	166	2255	12	67.1
T4-26-4D	TraesCS4D02G319100	1545	425	1970	195	2165	13	67.5
T4-26-5A	TraesCS5A02G494800	1450	427	1877	112	1989	13	68.7
T4-28-5B	TraesCS5B02G544800	1735	42	1777	273	2050	22	89.4
T4-29A	TraesCS7A02G548000	1853	74	1927	56	1983	18	77.6
T4-29B	TraesCS7B02G471500	1813	70	1883	218	2101	20	87.3
T4-29D	TraesCS7D02G534700	1563	79	1642	77	1719	14	61.1
T4-3B	TraesCS4B02G329500	1790	100	1890	102	1992	15	76.2
T4-3D	TraesCS4D02G326400	1700	140	1840	290	2130	16	73.4
T4-30-4B	TraesCS4B02G378500	0	0	0	2411	2411	15	76.2
T4-30-5A	TraesCS5A02G545100	1982	95	2077	2459	4536	33	75.3
T4-31A	TraesCS6A02G146900	1781	172	1953	449	2402	14	69.2
T4-31B	TraesCS6B02G175100	2240	147	2387	361	2748	10	56.2
T4-31D	TraesCS6D02G136200	1214	587	1801	235	2036	12	64.2
T4-32A	TraesCS7A02G508800	1675	95	1770	267	2037	7	39.5
T4-32B	TraesCS7B02G418500	2042	130	2172	131	2303	18	77.6
T4-32B(2)	TraesCS7B02G418500	1063	118	1181	218	1399	6	38.5
T4-32D	TraesCS7D02G497400	1951	154	2105	136	2241	9	53.6
T4-33-4A	TraesCS4A02G384800	1748	48	1796	310	2106	20	77.4
T4-33-7D	TraesCS7D02G080300	1236	411	1647	231	1878	14	65.9
T4-34A	TraesCS4A02G296300	1934	0	1934	260	2194	17	66.1
T4-34D	TraesCS4D02G015800	1662	44	1706	212	1918	21	91.1
T4-35A	TraesCS5A02G295900	1562	330	1892	0	1892	14	69.2
T4-35D	TraesCS1D02G296300	1246	246	1492	0	1492	13	65.4
T4-36A	TraesCS5A02G236700	1394	202	1596	0	1596	16	80.3
T4-36B	TraesCS5B02G236300	2670	109	2779	19	2798	11	63.3
T4-37A	TraesCS7A02G049400	1825	70	1895	367	2262	15	75.1
T4-37D	TraesCS7D02G044500	1968	47	2015	452	2467	16	70.1
T4-38A	TraesCS3A02G125200	1761	35	1796	144	1940	17	73.9
T4-38B	TraesCS3B02G144800	2498	0	2498	0	2498	14	69.2
T4-38D	TraesCS3D02G126200	1486	127	1613	0	1613	9	47.1
T4-39A	TraesCS2A02G595500	1647	86	1733	2439	4172	27	70.6
T4-39B	TraesCS2B02G298300	405	83	488	2502	2990	19	73.4
T4-39D	TraesCS2D02G264200	1745	82	1827	294	2121	12	68.2
T4-4A	TraesCS4A02G049500	1050	0	1050	138	1188	17	76
T4-4B	TraesCS4B02G056100	1900	0	1900	111	2011	17	77.2
T4-4D	TraesCS4D02G049300	1450	0	1450	219	1669	22	89.4
T4-40A	TraesCS1A02G186800	917	78	995	184	1179	3	17.9
T4-40B	TraesCS1B02G194700	407	128	535	249	784	4	20.9
T4-40D	TraesCS1D02G189800	1480	121	1601	234	1835	10	50.1
T4-41-4B	TraesCS4B02G346900	1916	0	1916	0	1916	7	46.1
T4-41-5A	TraesCS5A02G551600	1667	0	1667	0	1667	12	61.6
T4-42A	TraesCS4A02G017000	2033	0	2033	433	2466	15	69.6
T4-42B	TraesCS4B02G287100	1686	84	1770	370	2140	12	64.9
T4-42D	TraesCS4D02G285800	1808	89	1897	194	2091	12	68.2
T4-43A	TraesCS2A02G270800	1897	116	2013	0	2013	9	50.4
T4-43D	TraesCS2D02G270500	786	105	891	444	1435	4	23.9
T4-44A	TraesCS4A02G453200	1921	0	1921	0	1921	19	88
T4-44B	TraesCS4B02G462400	1080	0	1080	210	1290	10	54.6
T4-44D	TraesCS4D02G464000	1589	0	1589	232	1821	15	69.6
T4-45A	TraesCS7A02G537200	1573	312	1885	0	1885	21	88.9
T4-45B	TraesCS7B02G766300L	1104	0	1104	68	1172	9	40.5
T4-45D	TraesCS7D02G524300	1909	0	1909	0	1909	20	85.2
T4-46A	TraesCS4A02G234500	1815	46	1861	368	2229	17	82.6
T4-46B	TraesCS4B02G224800	1577	131	1708	723	2431	14	68.6
T4-46D	TraesCS5D02G233700	1677	83	1760	792	2552	16	76.7
T4-47A	TraesCS4A02G129000	2490	371	2861	36	2897	5	24.2
T4-47B	TraesCS4B02G148100	2117	22	2139	127	2266	20	47.8
T4-47D	TraesCS4D02G129900	1833	337	2170	135	2305	18	86.4
T4-48A	TraesCS2A02G136800	1878	50	1928	314	2242	16	76.7
T4-48B	TraesCS2B02G160700	2022	0	2022	0	2022	16	76.7
T4-48D	TraesCS2D02G139500	1309	596	1905	287	2192	19	86.8
T4-49A	TraesCS1A02G338700	1913	98	2011	261	2272	21	85.6
T4-49B	TraesCS1B02G351600	1896	0	1896	232	2128	19	69.1
T4-5A	TraesCS4A02G343100	1419	320	1739	161	1900	6	32.5
T4-5B	TraesCS4B02G340700	527	300	827	0	827	1	7.1
T4-5D	TraesCS4D02G321400	607	293	900	88	988	6	35.8
T4-50A	TraesCS5A02G261400	2074	0	2074	0	2074	16	83.3
T4-50D	TraesCS5D02G268800	2443	37	2480	11	2491	12	61.6
T4-51A	TraesCS1A02G089500	1716	128	1844	326	2170	13	63.2
T4-51B	TraesCS1B02G117400	1880	96	1976	295	2271	20	87.3
T4-51D	TraesCS1D02G098000	1731	101	1832	297	2129	12	55.1
T4-52A	TraesCS7A02G478700	1855	0	1855	0	1855	16	73.4
T4-52B	TraesCS7B02G381400	2441	35	2476	0	2476	20	84
T4-53A	TraesCS5A02G321300	1227	218	1445	130	1575	6	29
T4-53B	TraesCS5B02G321800	1643	250	1893	110	2003	11	57.9
T4-53D	TraesCS5D02G328100	1888	206	2094	96	2190	13	72
T4-54A	TraesCS7A02G176700	1251	108	1359	0	1359	10	65.7
T4-54B	TraesCS7B02G081700	1627	0	1627	0	1627	14	69.2
T4-54D	TraesCS7D02G178000	2153	0	2153	155	2308	15	72.9
T4-55A	TraesCS2A02G583400	766	268	1034	175	1209	5	32
T4-55B	TraesCS2B02G603100	1670	0	1670	285	1955	12	61.2
T4-55D	TraesCS2D02G596300	2054	0	2054	340	2394	18	83.1
T4-56A	TraesCS1A02G047000	488	775	1263	0	1263	9	47.1
T4-56B	TraesCS1B02G060700	579	393	972	0	972	4	24.9
T4-56D	TraesCS1D02G047900	1428	322	1750	0	1750	16	65.6
T4-57A	TraesCS3A02G206400	1200	622	1822	180	2002	17	77.2
T4-57B	TraesCS3B02G238500	1530	0	1530	175	1705	16	70.1
T4-57D	TraesCS3D02G209200	1315	653	1968	308	2276	20	84
T4-58A	TraesCS5A02G447200	0	861	861	240	1101	11	54.6
T4-58B	TraesCS5B02G572600	1463	341	1804	1697	3501	27	67.5
T4-58D	TraesCS5D02G564000	1911	194	2105	238	2343	15	79.5
T4-59A	TraesCS6A02G241000	317	369	686	30	716	5	25.1
T4-59B	TraesCS6B02G283600	1832	288	2120	194	2314	6	32.5
T4-59D	TraesCS6D02G223300	1606	184	1790	155	1945	8	42.1
T4-6A	TraesCS6A02G203600.3	520	0	520	1738	2258	12	71.5
T4-6B	TraesCS6B02G223700	1862	234	2096	101	2197	10	57.4
T4-6D	TraesCS6D02G186900	0	0	0	2624	2624	8	42.1
T4-60A	TraesCS6A02G089400	982	0	982	0	982	9	46.8
T4-60	TraesCS6B02G111800	1837	289	2126	308	2434	8	45.4
T4-7A	TraesCS7A02G293300	1736	182	1918	152	2070	11	51.3
T4-7B	TraesCS7B02G182300	0	0	0	2370	2370	18	79.8
T4-7D	TraesCS7D02G291200	1552	261	1813	218	2031	11	61.2
T4-8A	TraesCS2A02G414300	1323	152	1475	97	1572	11	54.6
T4-8B	TraesCS2B02G433400	394	135	529	2001	2530	25	53.7
T4-8D	TraesCS2D02G411600	796	28	824	0	824	3	17.9
T4-9A	TraesCS3A02G347800L	1919	0	1919	327	2246	19	86.8
T4-9B	TraesCS3B02G255300	1965	0	1965	91	2056	15	76.2
T4-9D	TraesCS3D02G227500	1454	116	1570	571	2141	10	60.7
T5-1A	TraesCS4A02G035400	928	151	1079	231	1310	10	46.7
T5-1B	TraesCS4B02G268100	700	239	939	155	1094	12	55.1
T5-1D	TraesCS4D02G267500	1330	0	1330	179	1509	11	56.7
T5-10-4B	TraesCS4B02G396900	936	697	1633	272	1905	14	67.5
T5-10-5A	TraesCS5A02G558200	849	389	1238	20	1258	8	43.3
T5-11A	TraesCS1A02G176700	1481	647	2128	93	2221	10	54.1
T5-11D	TraesCS1D02G162400	2004	0	2004	152	2156	12	60.5
T5-12A	TraesCS1A02G020500	836	1060	1896	229	2125	18	73.2
T5-12B	TraesCS1B02G024500	558	1046	1604	311	1915	15	68.6
T5-12D	TraesCS1D02G020000	583	1163	1746	371	2117	18	76.5
T5-13A	TraesCS7A02G178000	636	1233	1869	384	2253	12	55.1
T5-13B	TraesCS7B02G083700	555	1263	1818	243	2061	16	78.8
T5-13D	TraesCS7D02G179800	596	1292	1888	326	2214	13	62.1
T5-14A	TraesCS7A02G412400	1009	166	1175	79	1254	5	32
T5-14B	TraesCS7B02G311600	913	174	1087	232	1319	4	24.9
T5-14D	TraesCS7D02G405500	605	248	853	115	968		
T5-15A	TraesCS4A02G235600	1511	312	1823	99	1922	16	74
T5-15B	TraesCS4B02G079300	1717	346	2063	171	2234	15	72.9
T5-15D	TraesCS4D02G078100	1002	323	1325	169	1494	6	35.2
T5-16A	TraesCS5A02G481400	1940	112	2052	164	2216	15	76.2
T5-16(B)-A	TraesCS5A02G481300	1918	0	1918	0	1918	10	60.7
T5-16B	TraesCS5B02G494600	669	214	883	0	883	6	32.5
T5-16(A)-B	TraesCS5B02G494500	1834	136	1970	421	2391	15	76.2
T5-16D	TraesCS5D02G495000	2319	186	2505	280	2785	8	53.2
T5-16(B)-D	TraesCS5D02G494900	795	412	1207	0	1207	7	36.8
T5-18D	TraesCS3D02G099500	2069	0	2069	0	2069	11	60.5
T5-19A	TraesCS2A02G432400	2049	0	2049	296	2345	24	95.8
T5-19B	TraesCS2B02G453700	1938	0	1938	81	2019	16	76.7
T5-2A	TraesCS3A02G099200	1857	192	2049	0	2049	17	72.7
T5-2B	TraesCS3B02G115400	1975	0	1975	0	1975	12	57.6
T5-2D	TraesCS3D02G099500	2071	0	2071	0	2071	11	60
T5-20A	TraesCS4A02G208400	924	0	924	229	1153	8	38.8
T5-20B	TraesCS4B02G112000	1391	158	1549	245	1794	15	78.4
T5-20D	TraesCS4D02G109500	896	79	975	116	1091	8	39.9
T5-21A	TraesCS2A02G142800	2066	205	2271	0	2271	18	76.5
T5-21B	TraesCS2B02G167900	2192	189	2381	145	2526	14	59.3
T5-21D	TraesCS2D02G146100	2288	186	2474	226	2700	15	66.4
T5-22B	TraesCS3B02G055700L	1900	0	1900	401	2301	20	93.9
T5-22D	TraesCS3D02G045700	1901	0	1901	290	2191	14	69.2
T5-23A	TraesCS5A02G384100	1788	133	1921	0	1921	17	77.2
T5-23B	TraesCS5B02G388900	1623	179	1802	0	1802	11	49.3
T5-23D	TraesCS5D02G393600	1915	0	1915	0	1915	18	80.9
T5-24A	TraesCS4A02G177600	1060	207	1267	1860	3127	28	65.3
T5-24B	TraesCS4B02G140200	1828	364	2192	1811	4003	35	76.1
T5-24D	TraesCS4D02G134900	1184	210	1394	553	1947	11	53.4
T5-25A	TraesCS2A02G277100	1307	263	1570	149	1719	8	43.3
T5-25B	TraesCS2B02G294500	1614	335	1949	141	2090	16	78.8
T5-25D	TraesCS2D02G276000	988	334	1322	206	1528	5	31.8
T5-26D	TraesCS3D02G046300	871	105	976	247	1223	9	90.7
T5-27A	TraesCS7A02G235700	1839	229	2068	294	2362	18	83.1
T5-27B	TraesCS7B02G133800	850	234	1084	322	1406	5	32
T5-27D	TraesCS7D02G235700	1621	333	1954	235	2189	18	83.1
T5-28A	TraesCS3A02G021800	1776	184	1960	22	1982	16	80
T5-28B	TraesCS3B02G024000	1870	178	2048	305	2353	7	36.2
T5-28D	TraesCS3D02G025000	1556	165	1721	159	1880	8	49.9
T5-29A	TraesCS1A02G119500	1878	135	2013	239	2252	12	64.9
T5-29B	TraesCS1B02G139100	834	0	834	33	867	4	24.9
T5-29D	TraesCS1D02G121700	1647	205	1852	63	1915	12	64.9
T5-3A	TraesCS1A02G221900	1291	131	1422	2383	3805	29	66.1
T5-3B	TraesCS1B02G235100	1609	134	1743	135	1878	11	61.2
T5-3D	TraesCS1D02G223600	1041	127	1168	2320	3488	25	58
T5-30A	TraesCS1A02G119100	601	0	601	152	753	3	15.1
T5-30B	TraesCS1B02G138600	711	169	880	309	1189	5	25.4
T5-30D	TraesCS1D02G120000	874	129	1003	169	1172	5	25.4
T5-4D	TraesCS3D02G099500	2071	0	2071	0	2071	12	57.6
T5-5A	TraesCS5A02G164000	250	0	250	1135	1385	9	50.6
T5-5B	TraesCS7B02G069800	778	196	974	963	1937	4	21.6
T5-5D	TraesCS7D02G166100	1695	405	2100	81	2181	19	76.9
T5-6B	TraesCS6B02G132600	954	0	954	569	1523	3	14.6
T5-6D	TraesCS5D02G140400	1028	0	1028	367	1395	4	17.2
T5-7B	TraesCS5B02G193200	857	504	1361	325	1686	5	32
T5-7D	TraesCS5D02G200900	1854	0	1854	271	2125	9	39.3
T5-8A	TraesCS5A02G382800	1817	155	1972	264	2236	18	84.2
T5-8B	TraesCS5B02G387100	1241	250	1491	0	1491	12	58.9
T5-8D	TraesCS5D02G387100	1891	144	2035	268	2303	18	86.8
T8-1A	TraesCS3A02G187800	1833	142	1975	235	2210	21	91.1
T8-1B	TraesCS3B02G217100	1781	146	1927	216	2143	16	73.4
T8-1D	TraesCS3D02G191400	1872	131	2003	340	2343	21	87.8
T8-10A	TraesCS2A02G261200	1561	402	1963	2577	4540	35	75.2
T8-10B	TraesCS2B02G281000	1824	91	1915	0	1915	16	76.7
T8-10D	TraesCS2D02G262700	1501	132	1633	2070	3703	28	60.4
T8-11A	TraesCS1A02G199600	443	285	728	223	951	3	17.8
T8-11B	TraesCS1B02G214500	1231	0	1231	226	1457	6	35.8
T8-11D	TraesCS1D02G203300	871	361	1232	0	1232	2	14.1
T8-12A	TraesCS2A02G443100	2299	85	2384	274	2658	18	77.6
T8-12B	TraesCS2B02G464200	1883	64	1947	232	2179	17	73.9
T8-12D	TraesCS2D02G442200	2002	193	2195	224	2419	16	70.1
T8-13A	TraesCS5A02G376500	0	968	968	352	1320	6	34.6
T8-13B	TraesCS5B02G380200	1189	1121	2310	210	2520	12	61.6
T8-13D	TraesCS5D02G386500	100	1021	1121	67	1188	10	47.1
T8-14A	TraesCS4A02G016400	0	0	0	2545	2545	19	86.8
T8-14B	TraesCS4B02G287800	1669	216	1885	0	1885	15	68.5
T8-14D	TraesCS4D02G286500	1508	443	1951	139	2090	17	80.5
T8-15A	TraesCS1A02G350500	250	80	330	2157	2487	23	50.5
T8-15B	TraesCS1B02G364800	1154	116	1270	193	1463	7	35.1
T8-15D	TraesCS1D02G353300	152	292	444	2086	2530	26	60.4
T8-16	TraesCS3A02G282800	2370	623	2993	276	3269	6	32.5
T8-16B	TraesCS3B02G316500	1579	181	1760	240	2000	9	43.8
T8-16D	TraesCS3D02G282800	1987	485	2472	387	2859	7	46.1
T8-17B	TraesCS2B02G405700	1657	0	1657	619	2276	12	57.4
T8-17D	TraesCS2D02G385500	799	158	957	529	1486	2	14.1
T8-18B	TraesCS3B02G319600	1740	276	2016	0	2016	5	24.2
T8-18D	TraesCS2D02G325400	1769	226	1995	167	2162	6	32.5
T8-19A	TraesCS3A02G093200	1169	845	2014	219	2233	14	75.8
T8-19B	TraesCS3B02G108500	1196	834	2030	192	2222	13	68.7
T8-19D	TraesCS3D02G093500	869	1050	1919	169	2088	18	80.9
T8-2A	TraesCS2A02G212200	1815	145	1960	0	1960	12	58.4
T8-2B	TraesCS2B02G237300	1399	198	1597	0	1597	10	50.8
T8-2D	TraesCS2D02G218100	1734	69	1803	0	1803	14	75.8
T8-21B	TraesCS3B02G486900	1434	0	1434	0	1434	13	59.8
T8-21D	TraesCS3D02G486600	1746	123	1869	180	2049	18	74.4
T8-22A	TraesCS1A02G339300	1450	269	1719	0	1719	11	56.7
T8-22B	TraesCS1B02G351600	1858	117	1975	201	2176	15	65.8
T8-22D	TraesCS1D02G341100	1936	116	2052	1066	3118	18	54.8
T8-23B	TraesCS6B02G286400	1432	279	1711	0	1711	12	51.8
T8-23D	TraesCS6D02G240200	819	274	1093	0	1093	3	14.6
T8-24A	TraesCS2A02G058300	0	1022	1022	1033	2055	19	81.4
T8-24B	TraesCS2B02G071100	1680	128	1808	840	2648	19	50.7
T8-24D	TraesCS2D02G057300	1432	0	1432	1039	2471	17	79.3
T8-25A	TraesCS2A02G053200	1716	85	1801	215	2016	19	81.4
T8-25B	TraesCS2B02G067100	1881	0	1881	351	2232	16	73.4
T8-25D	TraesCS2D02G053400	1871	74	1945	226	2171	22	92.7
T8-26A	TraesCS6A02G018200	1984	97	2081	305	2386	15	71.8
T8-26D	TraesCS6D02G021100	1356	111	1467	348	1815	12	58.4
T8-27A	TraesCS7A02G175200	1649	284	1933	430	2363	15	69.2
T8-27B	TraesCS7B02G080300	1936	174	2110	359	2469	12	51.3
T8-27D	TraesCS7D02G176700	1901	132	2033	314	2347	13	58.4
T8-3A	TraesCS5A02G233600	2037	96	2133	256	2389	16	52.1
T8-3B	TraesCS5B02G232100	1974	7	1981	85	2066	17	77
T8-3D	TraesCS5D02G240500	1983	29	2012	182	2194	14	69.2
T8-4A	TraesCS2A02G251200	1679	206	1885	242	2127	14	65.9
T8-4B	TraesCS2B02G280700	1941	260	2201	285	2486	13	72
T8-4D	TraesCS2D02G251600	1545	260	1805	171	1976	14	72.5
T8-5A	TraesCS4A02G113000	892	99	991	0	991	6	60.1
T8-5B	TraesCS4B02G191200	1965	141	2106	163	2269	9	50.4
T8-5D	TraesCS4D02G192400	1146	113	1259	195	1454	9	47.1
T8-6A	TraesCS2A02G331200	925	0	925	0	925	6	25.9
T8-6B	TraesCS2B02G351000	899	0	899	0	899	5	25.4
T8-6D	TraesCS2D02G331700	1114	142	1256	0	1256	9	40.5
T8-7A	TraesCS6A02G071200	1000	608	1608	210	1818	14	79.6
T8-7B	TraesCS6B02G095600	1292	610	1902	188	2090	13	70.8
T8-7D	TraesCS6D02G069300	1065	578	1643	270	1913	15	72.9
T8-8A	TraesCS5A02G391800	1164	323	1487	309	1796	8	38.8
T8-8B	TraesCS5B02G396700	1493	232	1725	297	2022	9	42.6
T8-8D	TraesCS5D02G401700	1088	258	1346	393	1739	7	39.5
T8-9A	TraesCS2A02G174300	1486	198	1684	145	1829	11	51.3
T8-9B	TraesCS2B02G200800	957	239	1196	0	1196	14	72.5
T8-9D	TraesCS2D02G181400	1765	202	1967	169	2136	12	69.6
T9-1A	TraesCS2A02G262700	943	853	1796	248	2044	15	72.9
T9-1B	TraesCS2B02G279400	943	1049	1992	299	2291	12	64.9
T9-1D	TraesCS2D02G261300	1208	745	1953	223	2176	20	84
T9-10A	TraesCS7A02G492400	1336	654	1990	304	2294	20	94.4
T9-10B	TraesCS7B02G398100	1881	350	2231	376	2607	18	89.6
T9-10D	TraesCS7D02G478800	2369	104	2473	289	2762	17	79.3
T9-11A	TraesCS1A02G236100	1791	115	1906	2444	4350	35	83.8
T9-11B	TraesCS1B02G247700	1727	121	1848	2366	4214	32	71.2
T9-11D	TraesCS1D02G236200	1166	154	1320	2401	3721	42	84.2
T9-12A	TraesCS2A02G278400	2469	0	2469	0	2469	21	87.8
T9-12B	TraesCS2B02G287300	1904	0	1904	202	2106	18	79.8
T9-12D	TraesCS2D02G277600	2047	0	2047	0	2047	17	72.7
T9-13A	TraesCS5A02G222400	1108	349	1457	977	2434	14	75.8
T9-13B	TraesCS5B02G221400	155	2027	2182	81	2263	14	69.2
T9-13D	TraesCS5D02G230200	1738	387	2125	179	2304	15	76.1
T9-14A	TraesCS4A02G111100	1810	0	1810	272	2082	12	58.4
T9-14B	TraesCS4B02G207600	2212	0	2212	154	2366	15	50
T9-15A	TraesCS7A02G415300	1366	403	1769	311	2080	15	69.6
T9-15B	TraesCS7B02G315200	1932	0	1932	751	2683	10	60.7
T9-15D	TraesCS7D02G408500	1370	261	1631	806	2437	9	48.8
T9-16A	TraesCS5A02G401300	1819	0	1819	162	1981	15	69.6
T9-16B	TraesCS5B02G406100	1414	112	1526	34	1560	11	51.3
T9-16D	TraesCS5D02G411600	690	0	690	23	713	5	28.7
T9-17A	TraesCS5A02G106400	1119	726	1845	356	2201	13	60.9
T9-17D	TraesCS5D02G120500	1290	562	1852	288	2140	8	40
T9-18A	TraesCS6A02G276100	740	0	740	0	740	7	32.9
T9-18B	TraesCS6B02G303600	1683	31	1714	0	1714	17	89.9
T9-18D	TraesCS6D02G256400	1911	152	2063	285	2348	12	61.6
T9-19A	TraesCS4A02G048200	161	1385	1546	322	1868	14	78.6
T9-19B	TraesCS4B02G256600	646	1592	2238	221	2459	13	72
T9-19D	TraesCS4D02G256700	0	1993	1993	183	2176	11	64.5
T9-2A	TraesCS7A02G160800	1051	441	1492	173	1665	6	32.3
T9-2D	TraesCS7D02G161900	946	482	1428	444	1872	6	42.4
T9-20A	TraesCS2A02G192600	647	367	1014	312	1326	9	43.3
T9-20B	TraesCS2B02G214600	1961	0	1961	0	1961	18	84.2
T9-20D	TraesCS2D02G194800	1105	617	1722	0	1722	7	42.8
T9-21A	TraesCS6A02G138600	1676	0	1676	0	1676	12	55.1
T9-21B	TraesCS6B02G167100	1304	359	1663	0	1663	11	51.3
T9-21D	TraesCS6D02G127600	1195	367	1562	0	1562	11	54.6
T9-22A	TraesCS4A02G191300	0	0	0	2330	2330	15	68.9
T9-22B	TraesCS4B02G124000	1969	151	2120	186	2306	19	81.4
T9-22D	TraesCS4D02G122000	1047	150	1197	162	1359	9	40.5
T9-23A	TraesCS4A02G271000	2013	0	2013	0	2013	19	76.9
T9-23B	TraesCS4B02G043100	1744	115	1859	179	2038	16	70.1
T9-23D	TraesCS4D02G040400	1213	0	1213	63	1276	16	59
T9-24A	TraesCS4A02G312500	1923	38	1961	271	2232	23	93.2
T9-24B	TraesCS4B02G001200	1983	0	1983	297	2280	11	61.2
T9-24D	TraesCS4D02G001700	2038	49	2087	218	2305	26	100
T9-25B	TraesCS4B02G375600	1782	62	1844	272	2116	11	61.2
T9-25D	TraesCS4D02G361700	783	0	783	554	1337	5	25.4
T9-25A	TraesCS5A02G537300	1627	0	1627	164	1791	8	40
T9-26A	TraesCS6A02G398400	758	82	840	185	1025	11	57.9
T9-26B	TraesCS6B02G438700	679	91	770	282	1052	4	18.4
T9-26D	TraesCS6D02G382600	1723	93	1816	120	1936	14	69.2
T9-27A	TraesCS4A02G039000	0	1834	1834	315	2149	17	82.6
T9-27B	TraesCS4B02G266700	0	2086	2086	312	2398	21	94.8
T9-27D	TraesCS4D02G266400	0	1897	1897	267	2164	17	76
T9-28A	TraesCS4A02G415400	1117	0	1117	338	1455	8	40
T9-28B	TraesCS4B02G316200	1723	0	1723	374	2097	14	69.2
T9-28D	TraesCS4D02G312800	1767	0	1767	323	2090	17	77.2
T9-29A	TraesCS5A02G144000	471	225	696	102	798	4	38
T9-29B	TraesCS5B02G143100	2061	63	2124	117	2241	24	92.5
T9-29D	TraesCS5D02G148000	1813	231	2044	361	2405	20	85.2
T9-3A	TraesCS6A02G320100	1363	0	1363	26	1389	10	47.5
T9-3B	TraesCS6B02G350900	1313	0	1313	0	1313	9	47.1
T9-3D	TraesCS6D02G299600	1900	0	1900	208	2108	21	87.8
T9-30A	TraesCS5A02G361300	1882	397	2279	0	2279	20	85.2
T9-30B	TraesCS5B02G363900	1464	176	1640	836	2476	23	66.5
T9-30D	TraesCS5D02G370200	1936	0	1936	165	2101	18	77.6
T9-31A	TraesCS4A02G236200	1732	330	2062	53	2115	21	88.9
T9-31B	TraesCS4B02G078800	1682	294	1976	117	2093	19	86.8
T9-31D	TraesCS4D02G077600	1682	0	1682	299	1981	10	47.5
T9-32A	TraesCS6A02G229500	1822	214	2036	345	2381	13	71.3
T9-32B	TraesCS6B02G252000	696	0	696	303	999	3	14.6
T9-32D	TraesCS6D02G205300	1743	212	1955	338	2293	18	80.9
T9-33A	TraesCS1A02G082700	1186	0	1186	530	1716	4	21.6
T9-33B	TraesCS1B02G100300	948	46	994	254	1248	9	45.9
T9-33D	TraesCS1D02G084100	2048	0	2048	349	2397	4	20.9
T9-34A	TraesCS1A02G379700	1656	224	1880	58	1938	18	79.8
T9-34B	TraesCS1B02G265400	1777	82	1859	208	2067	17	80.5
T9-34D	TraesCS1D02G387100	1737	223	1960	0	1960	13	67.5
T9-35A	TraesCS4A02G142700	1586	84	1670	245	1915	10	57.4
T9-35D	TraesCS5D02G149400	1506	81	1587	270	1857	11	61.2
T9-36A	TraesCS7A02G307900	609	0	609	293	902	6	29.2
T9-36B	TraesCS7B02G208300	848	225	1073	276	1349	8	46.6
T9-36D	TraesCS7D02G304800	1918	103	2021	288	2309	11	64.5
T9-37A	TraesCS3A02G135400	0	194	194	1582	1776	15	72.9
T9-37B	TraesCS3B02G169200	1177	329	1506	0	1506	10	50.8
T9-37D	TraesCS3D02G151400	485	461	946	0	946	8	67.8
T9-38A	TraesCS6A02G024300	1710	129	1839	196	2035	11	53.4
T9-38B	TraesCS6B02G033300	990	140	1130	190	1320	9	40.5
T9-38D	TraesCS6D02G027700	1994	127	2121	63	2184	12	55.1
T9-39A	TraesCS3A02G167100	1336	269	1605	232	1837	11	61.2
T9-39D	TraesCS3D02G173200	1020	327	1347	230	1577	9	50.4
T9-4A	TraesCS1A02G113400	1310	108	1418	306	1724	11	50.1
T9-4D	TraesCS1D02G114800	1403	38	1441	298	1739	7	38.4
T9-40A	TraesCS2A02G230100	2241	0	2241	127	2368	15	69.6
T9-40B	TraesCS2B02G251700	1654	0	1654	0	1654	10	54.1
T9-40D	TraesCS2D02G233800	1665	476	2141	0	2141	18	80.9
T9-41A	TraesCS2A02G309700	2124	0	2124	283	2407	14	71.3
T9-41B	TraesCS2B02G326800	1673	374	2047	172	2219	15	75.1
T9-41D	TraesCS2D02G308000	766	422	1188	261	1449	7	42.8
T9-42A	TraesCS2A02G467000	1682	126	1808	173	1981	14	65.9
T9-42B	TraesCS2B02G489800	1334	117	1451	220	1671	8	43.3
T9-42D	TraesCS2D02G467000	1345	186	1531	421	1952	12	56.9
T9-43A	TraesCS2A02G264800	1878	170	2048	218	2266	19	81.4
T9-43B	TraesCS2B02G277300	1667	33	1700	365	2065	25	96.2
T9-43D	TraesCS2D02G259200	1613	87	1700	197	1897	18	74.4
T9-44A	TraesCS3A02G280200	2095	195	2290	185	2475	15	69.6
T9-44B	TraesCS3B02G314000	1762	242	2004	1389	3393	24	57.5
T9-44D	TraesCS3D02G279900	1461	303	1764	1750	3514	27	65.6
T9-45B	TraesCS5B02G480900	2024	84	2108	0	2108	21	87.8
T9-45B	TraesCS1B02G314700	1582	381	1883	787	2670	22	94.8
T9-45D	TraesCS7D02G330200	2046	0	2046	0	2046	20	90.6
T9-46A	TraesCS5A02G597900L	1894	0	1894	158	2052	15	66.4
T9-46B	TraesCS5B02G446100	1859	0	1859	0	1859	15	61.9
T9-46D	TraesCS5D02G323500	1864	0	1864	61	1925	10	54.1
T9-47A	TraesCS6A02G315100	705	96	801	0	891	2	10.8
T9-47D	TraesCS6D02G294400	793	0	793	0	793	2	10.8
T9-48A	TraesCS2A02G491000	798	1076	1874	57	1931	17	76
T9-48B	TraesCS2B02G519200	1387	549	1936	211	2147	18	86.4
T9-48D	TraesCS2D02G491200	444	1291	1735	288	2023	17	79.3
T9-49A	TraesCS5A02G382600	1768	258	2026	1330	3356	16	70.1
T9-49B	TraesCS5B02G386800	365	308	673	1286	1959	17	72.7
T9-49D	TraesCS5D02G392000	1461	322	1783	258	2041	32	76.4
T9-5A	TraesCS2A02G386400	1902	133	2035	228	2263	15	72.9
T9-5B	TraesCS2B02G403800	2107	0	2107	160	2267	19	84.7
T9-5D	TraesCS2D02G383200	2146	0	2146	171	2317	17	77.2
T9-50A	TraesCS5A02G069500	2178	137	2315	304	2619	13	68.7
T9-50B	TraesCS5B02G075800	2000	131	2131	6	2137	16	73.4
T9-50D	TraesCS5D02G081700	1586	237	1823	197	2020	14	69.2
T9-51A	TraesCS2A02G102100	1643	562	2205	0	2205	13	54.4
T9-51B	TraesCS2B02G119200	1623	128	1751	0	1751	11	53.4
T9-51D	TraesCS2D02G101500	1642	427	2069	0	2069	14	61.4
T9-52A	TraesCS7A02G301300	1190	429	1619	122	1741	4	21.6
T9-52B	TraesCS7B02G201600	618	508	1126	0	1126	4	21.6
T9-52D	TraesCS7D02G296700	458	374	832	0	832	1	7.1
T9-53A	TraesCS1A02G196100	1199	0	1199	416	1615	9	42.6
T9-53B	TraesCS1B02G210700	1441	494	1935	278	2213	15	78.4
T9-53D	TraesCS1D02G199500	1601	482	2083	408	2491	14	71.3
T9-54A	TraesCS7A02G141600	1940	68	2008	276	2284	15	65.2
T9-54B	TraesCS7B02G044400	1969	30	1999	424	2423	13	65.4
T9-54D	TraesCS7D02G142900	1985	35	2020	140	2160	13	60.9
T9-55B	TraesCS7B02G484800L	1573	0	1573	0	1573	12	53.8
T9-55D	TraesCS7D02G149400	533	44	577	197	774	1	7.1
T9-56A	TraesCS1A02G217900	1478	377	1855	8	1863	5	28.7
T9-56B	TraesCS1B02G231300	1662	382	2044	317	2361	9	40.5
T9-56D	TraesCS1D02G219600	1130	0	1130	368	1498	2	10.8
T9-57A	TraesCS6A02G343300	1554	105	1659	58	1717	7	39.2
T9-57B	TraesCS6B02G375400	1773	105	1878	30	1908	15	71.8
T9-57D	TraesCS6D02G324200	1728	107	1835	6	1841	15	72.9
T9-58A	TraesCS1A02G210900	1478	98	1576	0	1576	13	68.7
T9-58B	TraesCS1B02G225000	1734	113	1847	50	1897	12	61.6
T9-58D	TraesCS1D02G214200	1845	142	1987	24	2011	10	60.7
T9-59A	TraesCS7A02G158000	1694	113	1807	211	2018	15	69.6
T9-59B	TraesCS7B02G062200	678	93	771	259	1030	5	28.7
T9-59D	TraesCS7D02G158500	1954	123	2077	229	2306	4	20.5
T9-6A	TraesCS2A02G327900	1684	218	1902	34	1936	8	46.6
T9-6D	TraesCS2D02G335000	1923	247	2170	58	2228	15	71.8
T9-60A	TraesCS4A02G219700	2271	114	2385	250	2635	7	36.2
T9-60B	TraesCS4B02G098200	1325	60	1385	38	1423	10	54.4
T9-60D	TraesCS4D02G094400	1954	123	2077	229	2306	13	67.5
T9-61A	TraesCS5A02G468300	1567	0	1567	0	1567	12	54
T9-61B	TraesCS5B02G480900	1818	92	1910	227	2137	8	40
T9-61D	TraesCS5D02G481200	1249	149	1398	120	1518	11	45.3
T9-63A	TraesCS5A02G477600	1844	149	1993	312	2305	15	78.2
T9-63D	TraesCS5D02G491100	1283	135	1418	114	1532	12	61.6
T9-64A	TraesCS7A02G422500	1756	0	1756	0	1756	11	50.1
T9-64B	TraesCS7B02G322900	1415	689	2104	384	2488	9	56.2
T9-64D	TraesCS7D02G414700	327	1002	1329	303	1632	6	39.6
T9-65B	TraesCS2B02G117900	1727	88	1815	85	1900	20	88.5
T9-65D	TraesCS2D02G100200	2005	83	2088	100	2188	21	82.4
T9-66A	TraesCS7A02G375400	1316	179	1495	125	1620	4	21.6
T9-66B	TraesCS7B02G277000	1744	327	2071	171	2242	11	53.4
T9-66D	TraesCS7D02G371900	1638	305	1943	175	2118	19	80.2
T9-67A	TraesCS1A02G304200	1424	462	1886	629	2515	14	75.8
T9-67B	TraesCS1B02G314700	1583	370	1953	779	2732	17	75.4
T9-67D	TraesCS1D02G303700	1605	349	1954	578	2532	18	68.8
T9-68A	TraesCS2A02G551100	1724	100	1824	0	1824	15	76.2
T9-69A	TraesCS6A02G318400	1723	371	1994	208	2202	13	65.4
T9-69B	TraesCS6B02G348700	1332	337	1669	0	1669	13	58.8
T9-69D	TraesCS6D02G297600	1531	362	1893	236	2129	14	72.5
T9-7A	TraesCS2A02G423900	1893	287	2180	265	2445	29	75.6
T9-7B	TraesCS2B02G443500	1394	406	1800	1153	2953	29	67.7
T9-7D	TraesCS2D02G421700	1878	51	1929	129	2058	17	83.8
T9-70A	TraesCS3A02G428800	1678	151	1729	145	1874	14	72.9
T9-70B	TraesCS3B02G466400	714	156	870	323	1193	9	37.2
T9-70D	TraesCS3D02G424400	1951	152	2103	97	2200	11	51.3
T9-71A	TraesCS4A02G247200	1861	274	2135	0	2135	13	62.1
T9-72B	TraesCS4B02G375600	2001	61	2062	268	2330	18	77.6
T9-73A	TraesCS3A02G176100	1944	0	1944	193	2137	14	79.8
T9-73B	TraesCS3B02G205800	2175	177	2352	210	2562	12	64.9
T9-74A	TraesCS5A02G157300	1712	92	1804	241	2045	13	62.1
T9-74B	TraesCS5B02G156400	2825	96	2921	213	3134	20	80.7
T9-74D	TraesCS5D02G162600	841	76	917	184	1101	8	34.2
T9-75B	TraesCS6B02G214900	1999	0	1999	336	2335	11	61.2
T9-75D	TraesCS6D02G173000	1871	0	1871	0	1871	8	45.4
T9-76A	TraesCS7A02G363100	1261	145	1406	496	1902	7	38.4
T9-76B	TraesCS7B02G264800	1798	134	1932	338	2270	17	79.3
T9-76D	TraesCS7D02G359900	1905	129	2034	299	2333	17	79.3
T9-77A	TraesCS7A02G213600	1471	296	1467	265	1732	18	77.6
T9-77B	TraesCS7B02G120800	1715	0	1715	0	1715	17	70.6
T9-77D	TraesCS7D02G215600	1907	0	1907	0	1907	12	57.8
T9-8A	TraesCS5A02G378300	1233	401	1634	290	1924	13	62.1
T9-8D	TraesCS5D02G388300	773	171	944	213	1157	5	45.7
T9-9A	TraesCS7A02G249900	1143	1201	2344	63	2407	13	65.4
T9-9B	TraesCS7B02G141300	1372	442	1814	101	1915	12	61.6
T9-9D	TraesCS7D02G248500	389	471	860	1127	1987	14	79.1
T10-1A	TraesCS4A02G105600	1292	105	1397	321	1718	11	54.6
T10-1B	TraesCS4B02G198400	2066	0	2066	0	2066	10	46.4
T10-1D	TraesCS4D02G199200	1796	100	1896	116	2012	17	77.2
T10-10A	TraesCS3A02G124100	2000	0	2000	384	2384	11	54.6
T10-10B	TraesCS3B02G143800	1877	225	2102	163	2265	10	46.4
T10-10D	TraesCS3D02G125600	2315	147	2462	130	2592	17	77.2
T10-11B	TraesCS4B02G045000	921	72	993	137	1130	10	46.4
T10-11D	TraesCS4D02G044700	857	101	958	304	1262	16	85.4
T10-12A	TraesCS5A02G174300	1882	0	1882	0	1882	11	53.4
T10-12B	TraesCS5B02G171700	1872	101	1973	0	1973	11	53.4
T10-13A	TraesCS5A02G152300	1888	30	1918	308	2226	12	57.2
T10-13B	TraesCS5B02G150900	1953	31	1984	448	2432	14	64.7
T10-13D	TraesCS5D02G157500	1964	31	1995	476	2471	11	53.4
T10-14A	TraesCS7A02G233700	1110	143	1253	387	1640	12	51.8
T10-14B	TraesCS7B02G132000	1700	88	1788	479	2267	5	28.7
T10-14D	TraesCS7D02G233900	942	904	1846	373	2219	16	68.2
T10-15B	TraesCS4B02G229600L	853	0	853	1311	2164	21	87.8
T10-16B	TraesCS1B02G075900	816	29	845	301	1146	8	36.7
T10-16D	TraesCS1D02G059600	1586	69	1655	187	1842	6	29.2
T10-17B	TraesCS2B02G234700	523	115	638	217	855	6	25.3
T10-18A	TraesCS7A02G455900	745	398	1143	116	1259	3	14.6
T10-18D	TraesCS7D02G444800	1662	420	2082	0	2082	12	68.5
T10-19A	TraesCS6A02G335700	1961	158	2119	0	2119	13	57.6
T10-2A	TraesCS7A02G300300	1120	482	1602	175	1777	10	47.5
T10-2D	TraesCS7D02G296000	1911	29	1940	282	2222	13	72
T10-20A	TraesCS5A02G133000	1677	188	1865	50	1915	17	70.6
T10-20B	TraesCS5B02G131900	1377	204	1581	0	1581	13	58.8
T10-20D	TraesCS5D02G141200	1869	202	2071	37	2108	18	76.5
T10-21A	TraesCS6A02G199100	1538	190	1728	163	1891	18	83.1
T10-21B	TraesCS6B02G229400	1695	67	1762	142	1904	12	58.4
T10-21D	TraesCS6D02G182800	1369	91	1460	280	1740	11	56.4
T10-22A	TraesCS3A02G381500	2192	62	2254	137	2391	15	71.8
T10-22B	TraesCS3B02G414200	2092	14	2106	0	2106	14	62.6
T10-22D	TraesCS3D02G374700	1999	39	2038	0	2038	22	89.4
T10-23A	TraesCS1A02G288700	974	0	974	0	974	6	35.6
T10-24A	TraesCS3A02G151300	1048	0	1048	77	1125	6	32.5
T10-25A	TraesCS4A02G217700	1889	33	1922	471	2393	19	84.1
T10-25B	TraesCS4B02G096400	1492	0	1492	54	1546	15	63.8
T10-25D	TraesCS4D02G092800	448	80	528	288	816	2	10.8
T10-26A	TraesCS2A02G304900	712	1105	1817	190	2007	15	69.5
T10-26B	TraesCS2B02G321700	641	1070	1711	[illegible]	2169	16	80.1
T10-26D	TraesCS2D02G303500	0	393	393	2131	2524	16	78.8
T10-28A	TraesCS2A02G171900	588	78	666	261	927	3	17.9
T10-29B	TraesCS7B02G103700	1956	0	1956	331	2287	15	58.6
T10-29D	TraesCS7D02G199800	1039	0	1039	222	1261	10	40.4
T10-3A	TraesCS7A02G410300	1952	0	1952	336	2288	19	84.7
T10-3B	TraesCS7B02G309700	1950	0	1950	270	2220	19	79.8
T10-3D	TraesCS7D02G403500	1841	0	1841	300	2141	14	69.2
T10-30A	TraesCS3A02G500600	1567	20	1587	56	1643	6	32.5
T10-31A	TraesCS5A02G352800	1686	0	1686	262	1948	16	68.8
T10-31B	TraesCS5B02G355000	1249	594	1843	277	2120	10	43.1
T10-31D	TraesCS5D02G359800	1608	0	1608	0	1608	12	51.8
T10-32B	TraesCS6B02G220900	1156	771	1927	169	2096	19	81.4
T10-32D	TraesCS6D02G182400	0	1802	1802	965	2767	18	87.4
T10-34A	TraesCS3A02G401700	1650	97	1747	246	1993	20	87.8
T10-34B	TraesCS3B02G434600	1796	89	1885	196	2081	21	85.6
T10-34D	TraesCS3D02G396500	2174	117	2291	337	2628	4	20.5
T10-35B	TraesCS4B02G280400	1142	105	1247	148	1395	12	51.8
T10-35D	TraesCS4D02G381900LC	923	0	923	0	923	1	7.1
T10-36A	TraesCS7A02G295800	1315	226	1541	109	1650	7	42.8
T10-36D	TraesCS7D02G289400	1045	186	1231	136	1367	10	44.2
T10-37A	TraesCS1A02G428700	0	0	0	2206	2206	15	76.2
T10-37B	TraesCS1B02G463400	983	1106	2089	0	2089	12	57.2
T10-37D	TraesCS1D02G438000	947	1119	2066	0	2066	12	58.4
T10-38A	TraesCS5A02G303200	972	113	1085	155	1240	8	40
T10-38B	TraesCS5B02G303500	1465	0	1465	0	1465	9	47.1
T10-38D	TraesCS5D02G310000	1472	0	1472	0	1472	11	51.8
T10-39D	TraesCS4D02G147700	1733	187	1920	1045	2965	20	66.6
T10-4	TraesCS2A02G275100	0	0	0	1969	1969	22	94.8
T10-4	TraesCS2B02G292500	0	0	0	1881	1881	12	59.3
T10-4	TraesCS2D02G274100	0	0	0	1982	1982	13	70.8
T10-40A	TraesCS2A02G340400	1635	354	1989	0	1989	8	43.3
T10-40B	TraesCS2B02G338300	1382	469	1851	281	2132	7	42.8
T10-40D	TraesCS2D02G318100	794	0	794	0	794	1	7.1
T10-41A	TraesCS5A02G286500	1290	0	1290	87	1377	11	51.3
T10-41B	TraesCS5B02G285900	1303	0	1303	166	1469	CROSS-HYBRIDISNG BAITS F	
T10-41D	TraesCS5D02G294200	1884	35	1919	338	2257	13	65.4
T10-42A	TraesCS5A02G042200	653	747	1400	374	1774	14	60.9
T10-42B	TraesCS5B02G045200	981	860	1841	422	2263	19	86.8
T10-42D	TraesCS5D02G050300	897	963	1860	298	2158	17	80.5
T10-43A	TraesCS3A02G198600	948	0	948	315	1263	7	31.8
T10-43B	TraesCS3B02G227200	1478	0	1478	445	1923	12	55.1
T10-43D	TraesCS3D02G196500	1923	0	1923	298	2221	6	25.9
T10-44A	TraesCS4A02G143200	1877	205	2082	0	2082	13	60.9
T10-44B	TraesCS4B02G162200	0	0	0	2349	2349	15	78.2
T10-44D	TraesCS4D02G159600	602	151	753	0	753	5	28.7
T10-45A	TraesCS5A02G381700LC	887	0	887	136	1023	1	7.1
T10-45B	TraesCS5B02G256100	773	0	773	402	1175	8	37.1
T10-46A	TraesCS2A02G325700	2231	0	2231	283	2514	10	52.9
T10-46B	TraesCS2B02G356200	1768	90	1858	370	2228	8	43.3
T10-46D	TraesCS2D02G337000	1892	117	2009	369	2378	20	88.5
T10-47A	TraesCS7A02G316600	1267	261	1528	0	1528	11	54.2
T10-47B	TraesCS7B02G217600	544	524	1068	295	1363	3	17.9
T10-47D	TraesCS7D02G313500	688	480	1168	190	1358	7	32.9
T10-48A	TraesCS5A02G168500	457	1010	1467		2512	10	57.4
T10-48B	TraesCS5B02G165300	484	1357	1841	1612	3453	18	51.6
T10-48D	TraesCS5D02G172800	1943	0	1943	124	2067	8	43.3
T10-5A	TraesCS7A02G276600	1489	0	1489	229	1718	3	18
T10-5B	TraesCS7B02G174300	1537	278	1815	185	2000	6	35.8
T10-5D	TraesCS7D02G276600	1624	0	1624	363	1987	9	50.4
T10-6A	TraesCS2A02G315000	1925	105	2030	19	2049	8	46.6
T10-6B	TraesCS2B02G333500	1775	98	1873	54	1927	4	24.9
T10-6D	TraesCS2D02G313300	1651	155	1806	0	1806	12	64.9
T10-7A	TraesCS5A02G081400	805	286	1091	321	1412	11	54.6
T10-7B	TraesCS5B02G085000	465	274	739	382	1121	3	17.9
T10-7D	TraesCS7D02G276600	1612	0	1612	354	1966	9	50.4
T10-8B	TraesCS2B02G559100	1535	542	2077	297	2374	18	83.1
T10-8D	TraesCS2D02G531600	1411	544	1955	0	1955	17	80.5
T10-9A	TraesCS1A02G162200	533	174	707	0	865	2	14.1
T10-9B	TraesCS1B02G178800	1522	236	1758	0	1758	12	68.2
T10-9D	TraesCS1D02G159000	0	0	0	2108	2108	15	72.9
T6-10A	TraesCS6A02G017500	862	164	1026	305	1331	9	44.1
T6-10B	TraesCS6B02G024900	1481	209	1690	333	2023	16	76.8
T6-10D	TraesCS6D02G020700	1197	161	1358	333	1691	12	58.5
T6-11A	TraesCS4A02G376700	1594	0	1594	512	2106	18	76.5
T6-11D	TraesCS7D02G073700	2212	0	2212	0	2212	16	72.2
T6-12B	TraesCS2B02G528300	1609	633	2242	49	2291	11	57.9
T6-12D	TraesCS2D02G500600	1360	438	1798	262	2060	15	71.8
T6-13A	TraesCS6A02G298100	1830	203	2033	102	2135	7	42.6
T6-13B	TraesCS6B02G327500	1374	245	1619	99	1718	11	67.8
T6-13D	TraesCS6D02G383600LC	1777	31	1808	289	2097	15	76.2
T6-14A	TraesCS6A02G335400	1636	138	1774	270	2044	19	81.4
T6-14B	TraesCS6B02G366100	1571	131	1702	44	1746	19	81.4
T6-14D	TraesCS6D02G315300	1616	345	1961	20	1981	18	84.2
T6-15A	TraesCS6A02G031100	1745	221	1966	224	2190	19	81.4
T6-15B	TraesCS6B02G044100	1778	0	1778	216	1994	12	68.2
T6-16A	TraesCS6A02G031200	2115	96	2211	198	2409	16	73.4
T6-16B	TraesCS6B02G044000	2045	0	2045	198	2243	17	80.5
T6-16D	TraesCS6D02G035600	1915	72	1987	188	2175	16	80
T6-17A	TraesCS6A02G031000	1548	93	1641	276	1917	12	61.6
T6-17B	TraesCS6B02G044200	1995	167	2162	424	2586	17	82.6
T6-17D	TraesCS6D02G035700	2137	85	2222	375	2597	13	65.8
T6-18A	TraesCS6A02G030700	1990	0	1990	296	2286	24	95.8
T6-18B	TraesCS6B02G044500	1777	0	1777	397	2174	16	70.1
T6-18D	TraesCS6D02G035900	2681	0	2681	320	3001	18	86.4
T6-2A	TraesCS7A02G157700	2047	0	2047	351	2398	20	85.2
T6-2B	TraesCS7B02G062100	312	2068	2380	351	2731	8	40
T6-2D	TraesCS7D02G158300	628	1225	1853	391	2244	19	81.4
T6-31A	TraesCS4A02G416400	1174	43	1217	316	1533	14	47.3
T6-31A(2)	TraesCS4A02G416300	2074	0	2074	317	2391	25	99.5
T6-31B	TraesCS4B02G317200	2019	50	2069	221	2290	21	92.2
T6-4A	TraesCS7A02G301700	723	205	928	1076	2004	6	29.2
T6-4B	TraesCS7B02G201900	1717	256	1973	57	2030	13	60.9
T6-4D	TraesCS7D02G297000	667	155	822	1036	1858	12	61.6
T6-6B	TraesCS4B02G375800	492	63	555	351	906	9	43.1
T6-6D	TraesCS4D02G361500	1986	83	2069	258	2327	14	61.4
T6-6A	TraesCS5A02G537100	1335	46	1381	300	1681	12	54
T6-7A	TraesCS5A02G185700	1860	140	2000	158	2158	19	84.7
T6-7B	TraesCS5B02G183800	1695	0	1695	0	1695	12	61.2
T6-7D	TraesCS5D02G190800	2109	173	2282	0	2282	15	79.5
T6-8A	TraesCS5A02G185600	1613	129	1742	313	2055	11	56.7
T6-8B	TraesCS5B02G183700	1872	134	2006	290	2296	22	86.1
T6-8D	TraesCS5D02G190700	531	185	716	289	1005	3	17.9
T6-9A	TraesCS1A02G065700	1071	117	1188	1281	2469	15	46.9
T6-9B	TraesCS1B02G083900	985	350	1335	1302	2637	3	17.9
T6-9D	TraesCS1D02G066700	1718	207	1925	0	1925	4	21.6
	average	1416	235	1650	342	1993		
	stdev	575	327	536	496	568		
	SEM	19	11	18	16	19		

trait gene	gene ID	promoter	5'UTR	promoter + 5'UTR	Exons / Introns	total bp	MYbaits	TARGET coverage (%)
T2-11A	TraesCS7A02G287500	0	2096	2096	312	2408	8	49.9
T9-27B	TraesCS4B02G266700	0	2086	2086	312	2398	21	94.8
T6-2B	TraesCS7B02G062100	312	2068	2380	351	2731	8	40
T9-13B	TraesCS5B02G221400	155	2027	2182	81	2263	14	69.2
T9-19D	TraesCS4D02G256700	0	1993	1993	183	2176	11	64.5
T2-52A	TraesCS1A02G353300	0	1970	1970	318	2288	21	88.9
T4-18-7A	TraesCS7A02G009800	0	1939	1939	99	2038	24	92.5
T9-27D	TraesCS4D02G266400	0	1897	1897	267	2164	17	76
T9-27A	TraesCS4A02G039000	0	1834	1834	315	2149	17	82.6
T10-32D	TraesCS6D02G182400	0	1802	1802	965	2767	18	87.4
T2-54D	TraesCS2D02G369800	0	1791	1791	802	2593	26	85.4
T2-54A	TraesCS2A02G373600	0	1652	1652	458	2110	21	81.2
T2-9D	TraesCS3D02G043500	3216	1614	4830	150	4980	19	90.1
T9-19B	TraesCS4B02G256600	646	1592	2238	221	2459	13	72
T9-19A	TraesCS4A02G048200	161	1385	1546	322	1868	14	78.6
T10-48B	TraesCS5B02G165300	484	1357	1841	1612	3453	18	51.6
T2-37D	TraesCS1D02G427400	522	1292	1814	209	2023	15	76.2
T5-13D	TraesCS7D02G179800	596	1292	1888	326	2214	13	62.1
T9-48D	TraesCS2D02G491200	444	1291	1735	288	2023	17	79.3
T5-13B	TraesCS7B02G083700	555	1263	1818	243	2061	16	78.8
T2-37B	TraesCS1B02G449700	531	1258	1789	1201	2990	23	72.2
T5-13A	TraesCS7A02G178000	636	1233	1869	384	2253	12	55.1
T6-2D	TraesCS7D02G158300	628	1225	1853	391	2244	19	81.4
T9-9A	TraesCS7A02G249900	1143	1201	2344	63	2407	13	65.4
T2-37A	TraesCS1A02G419600	297	1173	1470	476	1946	11	57.9
T5-12D	TraesCS1D02G020000	583	1163	1746	371	2117	18	76.5
T2-61A	TraesCS6A02G175100	857	1142	1999	276	2275	12	57.2
T8-13B	TraesCS5B02G380200	1189	1121	2310	210	2520	12	61.6
T10-37D	TraesCS1D02G438000	947	1119	2066	0	2066	12	58.4
T10-37B	TraesCS1B02G463400	983	1106	2089	0	2089	12	57.2
T10-26A	TraesCS2A02G304900	712	1105	1817	190	2007	15	69.5
T1-19A	TraesCS4A02G016300	1046	1094	2140	224	2364	15	69.6
T9-48A	TraesCS2A02G491000	798	1076	1874	57	1931	17	76
T10-26B	TraesCS2B02G321700	641	1070	1711	2169	2169	16	80.1
T5-12A	TraesCS1A02G020500	836	1060	1896	229	2125	18	73.2
T8-19D	TraesCS3D02G093500	869	1050	1919	169	2088	18	80.9
T9-1B	TraesCS2B02G279400	943	1049	1992	299	2291	12	64.9
T5-12B	TraesCS1B02G024500	558	1046	1604	311	1915	15	68.6
T8-24A	TraesCS2A02G058300	0	1022	1022	1033	2055	19	81.4
T8-13D	TraesCS5D02G386500	100	1021	1121	67	1188	10	47.1
T10-48A	TraesCS5A02G168500	457	1010	1467		2512	10	57.4
T9-64D	TraesCS7D02G414700	327	1002	1329	303	1632	6	39.6
T2-10D	TraesCS1D02G358500	540	994	1534	1392	2926	18	56
T8-13A	TraesCS5A02G376500	0	968	968	352	1320	6	34.6
T10-42D	TraesCS5D02G050300	897	963	1860	298	2158	17	80.5
T2-10B	TraesCS1B02G371300	610	933	1543	0	1543	14	65.9
T10-14D	TraesCS7D02G233900	942	904	1846	373	2219	16	68.2
T2-33B	TraesCS2B02G145700	1415	892	2307	331	2638	11	54.6
T2-53-7D	TraesCS7D02G064300	1315	865	2180	253	2433	21	91.1
T4-58A	TraesCS5A02G447200	0	861	861	240	1101	11	54.6
T10-42B	TraesCS5B02G045200	981	860	1841	422	2263	19	86.8
T2-33A	TraesCS2A02G123800	959	857	1816	323	2139	15	73.7
T9-1A	TraesCS2A02G262700	943	853	1796	248	2044	15	72.9
T8-19A	TraesCS3A02G093200	1169	845	2014	219	2233	14	75.8
T2-33D	TraesCS2D02G126600	1133	842	1975	240	2215	11	63.8
T2-53-7A	TraesCS7A02G070100	944	842	1786	230	2016	15	69.6
T8-19B	TraesCS3B02G108500	1196	834	2030	192	2222	13	68.7
T2-6D	TraesCS7D02G294300	642	808	1450	206	1656	6	32.5
T4-10A	TraesCS4A02G106400	1421	796	2217	222	2439	7	41.6
T4-56A	TraesCS1A02G047000	488	775	1263	0	1263	9	47.1
T10-32B	TraesCS6B02G220900	1156	771	1927	169	2096	19	81.4
T1-6-1B	TraesCS1B02G479100	276	768	1044	250	1294	16	68.3
T10-42A	TraesCS5A02G042200	653	747	1400	374	1774	14	60.9
T9-1D	TraesCS2D02G261300	1208	745	1953	223	2176	20	84
T2-53-4A	TraesCS4A02G418200	1239	728	1967	275	2242	21	91.1
T2-61B	TraesCS6B02G210000	763	728	1491	255	1746	9	43.8
T9-17A	TraesCS5A02G106400	1119	726	1845	356	2201	13	60.9
T1-6-7D	TraesCS7D02G000100	665	714	1379	451	1830	9	43.8
T5-10-4B	TraesCS4B02G396900	936	697	1633	272	1905	14	67.5
T9-64B	TraesCS7B02G322900	1415	689	2104	384	2488	9	56.2
T2-23D	TraesCS7D02G483000	1425	656	2081	94	2175	10	52.9
T9-10A	TraesCS7A02G492400	1336	654	1990	304	2294	20	94.4
T4-57D	TraesCS3D02G209200	1315	653	1968	308	2276	20	84
T5-11A	TraesCS1A02G176700	1481	647	2128	93	2221	10	54.1
T6-12B	TraesCS2B02G528300	1609	633	2242	49	2291	11	57.9
T1-6-7A	TraesCS7A02G000200	562	631	1193	291	1484	6	32.5
T2-23B	TraesCS7B02G401600	1351	629	1980	311	2291	11	57.9
T8-16	TraesCS3A02G282800	2370	623	2993	276	3269	6	32.5
T4-57A	TraesCS3A02G206400	1200	622	1822	180	2002	17	77.2
T9-20D	TraesCS2D02G194800	1105	617	1722	0	1722	7	42.8
T1-27B	TraesCS6B02G416400	1347	611	1958	378	2336	10	57.4
T8-7B	TraesCS6B02G095600	1292	610	1902	188	2090	13	70.8
T8-7A	TraesCS6A02G071200	1000	608	1608	210	1818	14	79.6
T2-48D	TraesCS1D02G000100	1030	605	1635	528	2163	18	73.2
T1-24B	TraesCS6B02G266200	1005	596	1601	492	2093	8	49.9
T4-48D	TraesCS2D02G139500	1309	596	1905	287	2192	19	86.8
T10-31B	TraesCS5B02G355000	1249	594	1843	277	2120	10	43.1
T4-31D	TraesCS6D02G136200	1214	587	1801	235	2036	12	64.2
T8-7D	TraesCS6D02G069300	1065	578	1643	270	1913	15	72.9
T9-17D	TraesCS5D02G120500	1290	562	1852	288	2140	8	40
T9-51A	TraesCS2A02G102100	1643	562	2205	0	2205	13	54.4
T2-48B	TraesCS1B02G398500	1376	556	1932	376	2308	21	91.1
T2-48B	TraesCS1B02G012400	1388	556	1944	352	2296	20	87.3
T1-9D	TraesCS7D02G261500	1364	553	1917	0	1917	12	64.9
T9-48B	TraesCS2B02G519200	1387	549	1936	211	2147	18	86.4
T10-8D	TraesCS2D02G531600	1411	544	1955	0	1955	17	80.5
T10-8B	TraesCS2B02G559100	1535	542	2077	297	2374	18	83.1
T1-24A	TraesCS6A02G237700	1500	527	2027	286	2313	14	74.6
T10-47B	TraesCS7B02G217600	544	524	1068	295	1363	3	17.9
T9-52B	TraesCS7B02G201600	618	508	1126	0	1126	4	21.6
T5-7B	TraesCS5B02G193200	857	504	1361	325	1686	5	32
T4-21A	TraesCS6A02G151700	1100	498	1598	283	1881	14	69.2
T9-53B	TraesCS1B02G210700	1441	494	1935	278	2213	15	78.4
T8-16D	TraesCS3D02G282800	1987	485	2472	387	2859	7	46.1
T9-2D	TraesCS7D02G161900	946	482	1428	444	1872	6	42.4
T9-53D	TraesCS1D02G199500	1601	482	2083	408	2491	14	71.3
T10-2A	TraesCS7A02G300300	1120	482	1602	175	1777	10	47.5
T10-47D	TraesCS7D02G313500	688	480	1168	190	1358	7	32.9
T1-20B	TraesCS1B02G100400	1019	478	1497	405	1902	6	35
T9-40D	TraesCS2D02G233800	1665	476	2141	0	2141	18	80.9
T9-9D	TraesCS7D02G248500	389	471	860	1127	1987	14	79.1
T2-2D	TraesCS5D02G253700	1410	469	1879	432	2311	21	88.9
T10-40B	TraesCS2B02G338300	1382	469	1851	281	2132	7	42.8
T2-2A	TraesCS5A02G247200	1514	466	1980	356	2336	18	80.9
T9-67A	TraesCS1A02G304200	1424	462	1886	629	2515	14	75.8
T9-37D	TraesCS3D02G151400	485	461	946	0	946	8	67.8
T1-26B	TraesCS5B02G025100	1508	459	1967	0	1967	13	68.7
T1-15B	TraesCS3B02G223900	988	449	1437	117	1554	3	21.2
T8-14D	TraesCS4D02G286500	1508	443	1951	139	2090	17	80.5
T9-9B	TraesCS7B02G141300	1372	442	1814	101	1915	12	61.6
T2-5A	TraesCS7A02G287400	1894	441	2335	105	2440	13	58.8
T9-2A	TraesCS7A02G160800	1051	441	1492	173	1665	6	32.3
T6-12D	TraesCS2D02G500600	1360	438	1798	262	2060	15	71.8
T1-17A	TraesCS2A02G176000	1107	434	1541	301	1842	6	35.8
T4-16A	TraesCS7A02G310500	1329	433	1762	262	2024	6	36
T9-52A	TraesCS7A02G301300	1190	429	1619	122	1741	4	21.6
T4-26-5A	TraesCS5A02G494800	1450	427	1877	112	1989	13	68.7
T9-51D	TraesCS2D02G101500	1642	427	2069	0	2069	14	61.4
T4-26-4D	TraesCS4D02G319100	1545	425	1970	195	2165	13	67.5
T9-41D	TraesCS2D02G308000	766	422	1188	261	1449	7	42.8
T1-15D	TraesCS3D02G197700	1345	421	1766	62	1828	12	64.9
T10-18D	TraesCS7D02G444800	1662	420	2082	0	2082	12	68.5
T5-16(B)-D	TraesCS5D02G494900	795	412	1207	0	1207	7	36.8
T4-33-7D	TraesCS7D02G080300	1236	411	1647	231	1878	14	65.9
T9-7B	TraesCS2B02G443500	1394	406	1800	1153	2953	29	67.7
T5-5D	TraesCS7D02G166100	1695	405	2100	81	2181	19	76.9
T9-15A	TraesCS7A02G415300	1366	403	1769	311	2080	15	69.6
T8-10A	TraesCS2A02G261200	1561	402	1963	2577	4540	35	75.2
T9-8A	TraesCS5A02G378300	1233	401	1634	290	1924	13	62.1
T1-17D	TraesCS2D02G183400	1566	398	1964	1811	3775	24	62.6
T10-18A	TraesCS7A02G455900	745	398	1143	116	1259	3	14.6
T9-30A	TraesCS5A02G361300	1882	397	2279	0	2279	20	85.2
T1-17B	TraesCS2B02G202300	1546	393	1939	1802	3741	22	54.8
T4-56B	TraesCS1B02G060700	579	393	972	0	972	4	24.9
T10-26D	TraesCS2D02G303500	0	393	393	2131	2524	16	78.8
T2-4B	TraesCS3B02G474900	1313	391	1704	1047	2751	19	69.6
T5-10-5A	TraesCS5A02G558200	849	389	1238	20	1258	8	43.3
T9-13D	TraesCS5D02G230200	1738	387	2125	179	2304	15	76.1
T2-21A	TraesCS1A02G115500	2368	386	2754	0	2754	9	47.1
T9-56B	TraesCS1B02G231300	1662	382	2044	317	2361	9	40.5
T1-26A	TraesCS5A02G025900	1546	381	1927	0	1927	14	69.2
T9-45B	TraesCS1B02G314700	1582	381	1883	787	2670	22	94.8
T9-56A	TraesCS1A02G217900	1478	377	1855	8	1863	5	28.7
T9-41B	TraesCS2B02G326800	1673	374	2047	172	2219	15	75.1
T9-52D	TraesCS7D02G296700	458	374	832	0	832	1	7.1
T1-13A	TraesCS5A02G030300	2108	372	2480	0	2480	16	73.4
T4-47A	TraesCS3A02G129000	2490	371	2861	36	2897	5	24.2
T9-69A	TraesCS6A02G318400	1723	371	1994	208	2202	13	65.4
T9-67B	TraesCS1B02G314700	1583	370	1953	779	2732	17	75.4
T4-59A	TraesCS6A02G241000	317	369	686	30	716	5	25.1
T9-20A	TraesCS2A02G192600	647	367	1014	312	1326	9	43.3
T9-21D	TraesCS6D02G127600	1195	367	1562	0	1562	11	54.6
T2-50B	TraesCS1B02G119300	1508	366	1874	66	1940	10	57.4
T5-24B	TraesCS4B02G140200	1828	364	2192	1811	4003	35	76.1
T1-13B	TraesCS5B02G029100	1694	363	2057	0	2057	13	57.6
T9-69D	TraesCS6D02G297600	1531	362	1893	236	2129	14	72.5
T8-11D	TraesCS1D02G203300	871	361	1232	0	1232	2	14.1
T9-21B	TraesCS6B02G167100	1304	359	1663	0	1663	11	51.3
T10-40A	TraesCS2A02G340400	1635	354	1989	0	1989	8	43.3
T9-10B	TraesCS7B02G398100	1881	350	2231	376	2607	18	89.6
T6-9B	TraesCS1B02G083900	985	350	1335	1302	2637	3	17.9
T2-10A	TraesCS1A02G351900	782	349	1131		2337	8	45.4
T9-13A	TraesCS5A02G222400	1108	349	1457	977	2434	14	75.8
T9-67D	TraesCS1D02G303700	1605	349	1954	578	2532	18	68.8
T1-8-7D	TraesCS7D02G015000	1541	346	1887	0	1887	15	72.9
T2-52D	TraesCS1D02G303700	1596	346	1942	576	2518	11	60.1
T5-15B	TraesCS4B02G079300	1717	346	2063	171	2234	15	72.9
T6-14D	TraesCS6D02G315300	1616	345	1961	20	1981	18	84.2
T1-23B	TraesCS5B02G220400	1318	344	1662	199	1861	16	74.6
T1-8-4A	TraesCS4A02G474000	1728	342	2070	129	2199	17	76
T2-3B	TraesCS1B02G053600	1558	341	1899	318	2217	15	68.5
T2-6A	TraesCS7A02G298600	1323	341	1664	111	1775	10	52.9
T4-58B	TraesCS5B02G572600	1463	341	1804	1697	3501	27	67.5
T4-47D	TraesCS3D02G129900	1833	337	2170	135	2305	18	86.4
T9-69B	TraesCS6B02G348700	1332	337	1669	0	1669	13	58.8
T1-20A	TraesCS1A02G083000	1016	336	1352	328	1680	9	43.1
T1-4A	TraesCS3A02G311100	1665	335	2000	281	2281	19	83.5
T2-6B	TraesCS7B02G188400	1058	335	1393	170	1563	10	50.8
T5-25B	TraesCS2B02G294500	1614	335	1949	141	2090	16	78.8
T5-25D	TraesCS2D02G276000	988	334	1322	206	1528	5	31.8
T1-3D	TraesCS5D02G216900	1239	333	1572	79	1651	7	35.9
T5-27D	TraesCS7D02G235700	1621	333	1954	235	2189	18	83.1
T4-35A	TraesCS1A02G295900	1562	330	1892	0	1892	14	69.2
T9-31A	TraesCS4A02G236200	1732	330	2062	53	2115	21	88.9
T1-8-7A	TraesCS7A02G017700	1687	329	2016	0	2016	16	76.7
T4-11D	TraesCS7D02G106400	955	329	1284	122	1406	6	32.5
T9-37B	TraesCS3B02G169200	1177	329	1506	0	1506	10	50.8
T9-39D	TraesCS3D02G173200	1020	327	1347	230	1577	9	50.4
T9-66B	TraesCS7B02G277000	1744	327	2071	171	2242	11	53.4
T5-15D	TraesCS4D02G078100	1002	323	1325	169	1494	6	35.2
T8-8A	TraesCS5A02G391800	1164	323	1487	309	1796	8	38.8
T4-56D	TraesCS1D02G047900	1428	322	1750	0	1750	16	65.6
T9-49D	TraesCS5D02G392000	1461	322	1783	258	2041	32	76.4
T1-20D	TraesCS1D02G084200	1243	321	1564	425	1989	9	49.2
T1-3B	TraesCS5B02G208700	1290	321	1611	0	1611	7	41.1
T2-4D	TraesCS3D02G433400	1547	321	1868	1085	2953	24	80.7
T4-5A	TraesCS2A02G343100	1419	320	1739	161	1900	6	35.2
T1-3A	TraesCS5A02G215100	814	318	1132	82	1214	6	35.7
T1-14B	TraesCS2B02G194900	1785	315	2100	0	2100	15	68.5
T4-45A	TraesCS7A02G537200	1573	312	1885	0	1885	21	88.9
T5-15A	TraesCS4A02G235600	1511	312	1823	99	1922	16	73.4
T2-18D	TraesCS7D02G236300	2316	309	2625	114	2739	7	42.8
T9-49B	TraesCS5B02G386800	365	308	673	1286	1959	17	72.7
T1-12D	TraesCS1D02G089300	1714	307	2021	463	2484	11	56.1
T1-14D	TraesCS2D02G176200	1604	307	1911	0	1911	16	70.1
T2-12A	TraesCS7A02G441400	1750	306	2056	51	2107	11	53.4
T2-24B	TraesCS7B02G133900	1546	305	1851	168	2019	10	54.1
T9-66D	TraesCS7D02G371900	1638	305	1943	175	2118	19	80.2
T9-44D	TraesCS3D02G279900	1461	303	1764	1750	3514	27	65.6
T2-43B	TraesCS7B02G018600	1553	302	1855	0	1855	14	62.6
T2-47B	TraesCS6B02G336400	2016	302	2318	265	2583	11	57.9
T4-5B	TraesCS2B02G340700	527	300	827	0	827	1	7.1
T2-3D	TraesCS1D02G041200	1628	299	1927	213	2140	10	47.5
T2-3A	TraesCS1A02G040600	310	298	608	280	888	2	14.1
T1-11B	TraesCS5B02G375800	1552	297	1849	140	1989	11	54.2
T1-14A	TraesCS2A02G168600	2263	296	2559	0	2559	16	72.2
T1-2B	TraesCS3B02G433000	551	296	847	142	989	5	22.1
T9-77A	TraesCS7A02G213600	1471	296	1467	265	1732	18	77.6
T9-31B	TraesCS4B02G078800	1682	294	1976	117	2093	19	86.8
T4-5D	TraesCS2D02G321400	607	293	900	88	988	6	35.8
T8-15D	TraesCS1D02G353300	152	292	444	2086	2530	26	60.4
T2-24A	TraesCS7A02G235500	852	291	1143	1021	2164	13	65.4
T2-60B	TraesCS6B02G246400	926	291	1217	1141	2358	15	52.4
T2-60A	TraesCS5A02G248700	315	290	605	1048	1653	2	10.8
T4-60	TraesCS6B02G111800	1837	289	2126	308	2434	8	45.4
T4-59B	TraesCS6B02G283600	1832	288	2120	194	2314	6	32.5
T10-60B	TraesCS5B02G255800	1750	287	2037	0	2037	17	72.7
T9-7A	TraesCS2A02G423900	1893	287	2180	265	2445	29	75.6
T10-7A	TraesCS5A02G081400	805	286	1091	321	1412	11	54.6
T2-45B	TraesCS7B02G093800	1313	285	1598	218	1816	14	65.9
T8-11A	TraesCS1A02G199600	443	285	728	223	951	3	17.8
T8-27A	TraesCS7A02G175200	1649	284	1933	430	2363	15	69.2
T8-23B	TraesCS6B02G286400	1432	279	1711	0	1711	12	51.8
T10-5B	TraesCS7B02G174300	1537	278	1815	185	2000	6	35.8
T2-1A	TraesCS4A02G107400	1585	277	1862	92	1954	18	74.4
T2-56D	TraesCS2D02G290800	1561	277	1838	0	1838	15	71.8
T8-18B	TraesCS5B02G319600	1740	276	2016	0	2016	5	24.2
T8-23D	TraesCS6D02G240200	819	274	1093	0	1093	3	14.6
T9-71A	TraesCS4A02G247200	1861	274	2135	0	2135	13	62.1
T10-7B	TraesCS5B02G085000	465	274	739	382	1121	3	17.9
T1-21A	TraesCS6A02G306200	1066	272	1338	0	1338	10	47.5
T2-56A	TraesCS2A02G293400	1795	272	2067	1111	3178	24	71.4
T2-9B	TraesCS3B02G047300	2163	270	2433	243	2676	16	72.2
T8-22A	TraesCS1A02G339300	1450	269	1719	0	1719	11	56.7
T9-39A	TraesCS3A02G167100	1336	269	1605	232	1837	11	61.2
T4-55A	TraesCS2A02G583400	766	268	1034	175	1209	5	32
T1-23A	TraesCS5A02G221600	1516	267	1783	161	1944	16	74
T2-15A	TraesCS6A02G436100	588	267	855	463	1318	6	29.2
T2-24D	TraesCS7D02G235600	799	264	1063	214	1277	7	42.8
T5-25A	TraesCS2A02G277100	1307	263	1570	149	1719	8	43.3
T2-5D	TraesCS7D02G284900	1446	262	1708	172	1880	6	37.2
T4-7D	TraesCS7D02G291200	1552	261	1813	218	2031	11	61.2
T9-15D	TraesCS7D02G408500	1370	261	1631	806	2437	9	48.8
T10-47A	TraesCS7A02G316600	1267	261	1528	0	1528	11	54.2
T8-4B	TraesCS3B02G280700	1941	260	2201	285	2486	13	72
T8-4D	TraesCS3D02G251600	1545	260	1805	171	1976	14	72.5
T1-7A	TraesCS4A02G012100	1560	259	1819	323	2142	20	84
T1-23D	TraesCS5D02G229400	1710	258	1968	23	1991	11	50.1
T8-8D	TraesCS5D02G401700	1088	258	1346	393	1739	7	39.5
T9-49A	TraesCS5A02G382600	1768	258	2026	1330	3356	16	70.1
T6-4B	TraesCS7B02G201900	1717	256	1973	57	2030	13	60.9
T2-1B	TraesCS4B02G197000	1604	255	1859	122	1981	17	70.6
T2-1D	TraesCS4D02G197300	1727	254	1981	48	2029	17	70.6
T4-53B	TraesCS5B02G321800	1643	250	1893	110	2003	11	57.9
T5-8B	TraesCS5B02G387100	1241	250	1491	0	1491	12	58.8
T4-13D	TraesCS7D02G405500	595	249	844	167	1011	6	25.9
T5-14D	TraesCS7D02G405500	605	248	853	115	968		
T9-6D	TraesCS2D02G335000	1923	247	2170	58	2228	15	71.8
T4-35D	TraesCS1D02G296300	1246	246	1492	0	1492	13	65.4
T4-10D	TraesCS4D02G198600	1336	245	1581	206	1787	12	58.4
T6-13B	TraesCS6B02G327500	1374	245	1619	99	1718	11	67.8
T9-44B	TraesCS3B02G314000	1762	242	2004	1389	3393	24	57.5
T2-51B	TraesCS2B02G491700	1878	240	2118	0	2118	17	76
T1-9B	TraesCS7B02G158500	1721	239	1960	0	1960	12	64.9
T5-1B	TraesCS4B02G268100	700	239	939	155	1094	12	55.1
T8-9B	TraesCS2B02G200800	957	239	1196	0	1196	14	72.5
T4-11B	TraesCS7B02G009200	898	238	1136	47	1183	5	25.4
T9-50D	TraesCS5D02G081700	1586	237	1823	197	2020	14	69.2
T4-18-4A	TraesCS4A02G485400	1869	236	2105	75	2180	18	84.2
T10-9B	TraesCS1B02G178800	1522	236	1758	0	1758	12	68.2
T4-6B	TraesCS6B02G223700	1862	234	2096	101	2197	10	57.4
T5-27B	TraesCS7B02G133800	850	234	1084	322	1406	5	32
T8-8B	TraesCS5B02G396700	1493	232	1725	297	2022	9	42.6
T1-27A	TraesCS6A02G378500	1606	231	1837	389	2226	12	67.1
T9-29D	TraesCS5D02G148000	1813	231	2044	361	2405	20	85.2
T5-27A	TraesCS7A02G235700	1839	229	2068	294	2362	18	83.1
T8-18D	TraesCS5D02G325400	1769	226	1995	167	2162	6	32.5
T10-36A	TraesCS7A02G295800	1315	226	1541	109	1650	7	42.8
T9-29A	TraesCS5A02G144000	471	225	696	102	798	4	38
T9-36B	TraesCS7B02G208300	848	225	1073	276	1349	8	46.6
T10-10B	TraesCS3B02G143800	1877	225	2102	163	2265	10	46.4
T9-34A	TraesCS1A02G379700	1656	224	1880	58	1938	18	79.8
T9-34D	TraesCS1D02G387100	1737	223	1960	0	1960	13	67.5
T4-26-4B	TraesCS4B02G322700	1868	221	2089	166	2255	12	67.1
T6-15A	TraesCS6A02G031100	1745	221	1966	224	2190	19	81.4
T2-56B	TraesCS2B02G309500	1276	220	1496	0	1496	9	54.2
T4-11A	TraesCS7A02G111300	839	218	1057	49	1106	4	21.6
T4-53A	TraesCS5A02G321300	1227	218	1445	130	1575	6	29
T9-6A	TraesCS2A02G327900	1684	218	1902	34	1936	8	46.6
T8-14B	TraesCS4B02G287800	1669	216	1885	0	1885	15	68.5
T5-16B	TraesCS5B02G494600	669	214	883	0	883	6	32.5
T9-32A	TraesCS6A02G229500	1822	214	2036	345	2381	13	71.3
T2-18A	TraesCS7A02G236200	1704	212	1916	183	2099	9	43.8
T2-19A	TraesCS6A02G178000	1757	212	1969	441	2410	10	47.5
T9-32D	TraesCS6D02G205300	1743	212	1955	338	2293	18	80.9
T5-24D	TraesCS4D02G134900	1184	210	1394	553	1947	11	53.4
T6-10B	TraesCS6B02G024900	1481	209	1690	333	2023	16	76.8
T5-24A	TraesCS4A02G177600	1060	207	1267	1860	3127	28	65.3
T6-9D	TraesCS1D02G066700	1718	207	1925	0	1925	4	21.6
T4-53D	TraesCS5D02G328100	1888	206	2094	96	2190	13	72
T8-4A	TraesCS3A02G251200	1679	206	1885	242	2127	14	65.9
T2-57A	TraesCS2A02G310300	1870	205	2075	394	2469	12	64.9
T5-21A	TraesCS2A02G142800	2066	205	2271	0	2271	18	76.5
T5-29D	TraesCS3D02G121700	1647	205	1852	63	1915	12	64.9
T10-44A	TraesCS4A02G143200	1877	205	2082	0	2082	13	60.9
T6-4A	TraesCS7A02G301700	723	205	928	1076	2004	6	29.2
T1-21D	TraesCS6D02G285300	1403	204	1607	0	1607	8	43.3
T10-20B	TraesCS5B02G131900	1377	204	1581	0	1581	13	58.8
T6-13A	TraesCS6A02G298100	1830	203	2033	102	2135	7	42.6
T4-17B	TraesCS1B02G154400	1494	202	1696	1804	3500	22	55.7
T4-36A	TraesCS5A02G236700	1394	202	1596	0	1596	16	80.3
T8-9D	TraesCS2D02G181400	1765	202	1967	169	2136	12	69.6
T10-20D	TraesCS5D02G141200	1869	202	2071	37	2108	18	76.5
T8-2B	TraesCS2B02G237300	1399	198	1597	0	1597	10	50.8
T8-9A	TraesCS2A02G174300	1486	198	1684	145	1829	11	51.3
T5-5B	TraesCS7B02G069800	778	196	974	963	1937	4	21.6
T9-44A	TraesCS3A02G280200	2095	195	2290	185	2475	15	69.6
T1-12A	TraesCS1A02G088000	1779	194	1973	385	2358	10	47.5
T4-58D	TraesCS5D02G564000	1911	194	2105	238	2343	15	79.5
T9-37A	TraesCS3A02G135400	0	194	194	1582	1776	15	72.9
T1-1D	TraesCS6D02G176900	1693	193	1886	210	2096	12	55.1
T8-12D	TraesCS2D02G442200	2002	193	2195	224	2419	16	70.1
T5-2A	TraesCS3A02G099200	1857	192	2049	0	2049	17	72.7
T4-20-7D	TraesCS7D02G008800	1734	190	1924	280	2204	17	82.6
T10-21A	TraesCS6A02G199100	1538	190	1728	163	1891	18	83.1
T2-21D	TraesCS1D02G116900	1877	189	2066	0	2066	7	39.5
T5-21B	TraesCS2B02G167900	2192	189	2381	145	2526	14	59.3
T10-20A	TraesCS5A02G133000	1677	188	1865	50	1915	17	70.6
T10-39D	TraesCS4D02G147700	1733	187	1920	1045	2965	20	66.6
T5-16D	TraesCS5D02G495000	2319	186	2505	280	2785	8	53.2
T5-21D	TraesCS2D02G146100	2288	186	2474	226	2700	15	66.4
T9-42D	TraesCS2D02G467000	1345	186	1531	421	1952	12	56.9
T10-36D	TraesCS7D02G289400	1045	186	1231	136	1367	10	44.2
T6-8D	TraesCS5D02G190700	531	185	716	289	1005	3	17.9
T1-1A	TraesCS6A02G189300	1811	184	1995	266	2261	11	54.6
T2-50A	TraesCS1A02G091500	1543	184	1727	142	1869	6	39.9
T4-59D	TraesCS6D02G223300	1606	184	1790	155	1945	8	42.1
T5-28A	TraesCS3A02G021800	1776	184	1960	22	1982	16	80
T4-22A	TraesCS2A02G395000	1521	183	1704	29	1733	14	65.9
T1-19D	TraesCS4B02G287900	1721	182	1903	309	2212	14	67.9
T2-19D	TraesCS6D02G166400	1516	182	1698	463	2161	11	51.3
T4-7A	TraesCS7A02G293300	1736	182	1918	152	2070	11	51.3
T8-16B	TraesCS3B02G316500	1579	181	1760	240	2000	9	43.8
T1-7B	TraesCS4B02G292300	1732	179	1911	305	2216	14	72.5
T5-23B	TraesCS5B02G388900	1623	179	1802	0	1802	11	49.3
T9-66A	TraesCS7A02G375400	1316	179	1495	125	1620	4	21.6
T2-13D	TraesCS2D02G226000	1831	178	2009	231	2240	24	93.6
T5-28B	TraesCS3B02G024000	1870	178	2048	305	2353	7	36.2
T9-73B	TraesCS3B02G205800	2175	177	2352	210	2562	12	64.9
T9-30B	TraesCS5B02G363900	1464	176	1640	836	2476	23	66.5
T1-19B	TraesCS4D02G286600	2099	175	2274	276	2550	11	61.2
T2-19B	TraesCS6B02G206800	1268	174	1442	471	1913	7	36.2
T5-14B	TraesCS7B02G311600	913	174	1087	232	1319	4	24.9
T8-27B	TraesCS7B02G080300	1936	174	2110	359	2469	12	51.3
T10-9A	TraesCS1A02G162200	533	174	707	0	865	2	14.1
T6-7D	TraesCS5D02G190800	2109	173	2282	0	2282	15	79.5
T4-13B	TraesCS7B02G311600	893	172	1065	332	1397	7	29.6
T4-31A	TraesCS6A02G146900	1781	172	1953	449	2402	14	69.2
T9-8D	TraesCS5D02G388300	773	171	944	213	1157	5	45.7
T9-43A	TraesCS2A02G264800	1878	170	2048	218	2266	19	81.4
T4-25B	TraesCS4B02G207000	1618	169	1787	159	1946	13	57.6
T5-30B	TraesCS1B02G138600	711	169	880	309	1189	5	25.4
T1-1B	TraesCS6B02G215300	1645	167	1812	342	2154	11	54.6
T6-17B	TraesCS6B02G044200	1995	167	2162	424	2586	17	82.6
T2-22D	TraesCS2D02G124900	1831	166	1997	1038	3035	21	70.7
T4-23B	TraesCS4B02G100100	1609	166	1775	2337	4112	27	72.2
T5-14A	TraesCS7A02G412400	1009	166	1175	79	1254	5	32
T2-39B	TraesCS5B02G175800	1145	165	1310	151	1461	4	24.7
T4-23D	TraesCS4D02G096400	1639	165	1804	2302	4106	6	35.8
T5-28D	TraesCS3D02G025000	1556	165	1721	159	1880	8	49.9
T6-10A	TraesCS6A02G017500	862	164	1026	305	1331	9	44.1
T4-13A	TraesCS7A02G412400	1054	163	1217	181	1398	8	43.3
T6-10D	TraesCS6D02G020700	1197	161	1358	333	1691	12	58.5
T1-5D	TraesCS2D02G385500	823	160	983	477	1460	2	14.1
T2-51A	TraesCS2A02G468800	1991	158	2149	296	2445	18	86.4
T4-21D	TraesCS6D02G141700	467	158	625	1438	2063	9	50.4
T5-20B	TraesCS4B02G112000	1391	158	1549	245	1794	15	78.4
T8-17D	TraesCS2D02G385500	799	158	957	529	1486	2	14.1
T10-19A	TraesCS6A02G335700	1961	158	2119	0	2119	13	57.6
T1-2A	TraesCS3A02G212900L	1605	157	1762	274	2036	8	42.1
T1-16D	TraesCS1D02G353100	1509	156	1665	1099	2764	17	49.7
T2-49B	TraesCS1B02G155700	842	156	998	344	1342	6	35.8
T9-70B	TraesCS3B02G466400	714	156	870	323	1193	9	37.2
T2-14B	TraesCS3B02G132400	1792	155	1947	280	2227	16	70.1
T5-8A	TraesCS5A02G382800	1817	155	1972	264	2236	18	84.2
T10-6D	TraesCS2D02G313300	1651	155	1806	0	1806	12	64.9
T6-4D	TraesCS7D02G297000	667	155	822	1036	1858	12	61.6
T4-32D	TraesCS7D02G497400	1951	154	2105	136	2241	9	53.6
T9-11D	TraesCS1D02G236200	1166	154	1320	2401	3721	42	84.2
T4-23A	TraesCS4A02G216100	983	153	1136	351	1487	5	28.7
T4-15-6A	TraesCS6A02G083200	2033	152	2185	253	2438	15	76.2
T4-25A	TraesCS4A02G097500	1829	152	1981	165	2146	18	77.1
T4-8A	TraesCS2A02G414300	1323	152	1475	97	1572	11	54.6
T9-18D	TraesCS6D02G256400	1911	152	2063	285	2348	12	61.6
T9-70D	TraesCS3D02G424400	1951	152	2103	97	2200	11	51.3
T5-1A	TraesCS4A02G035400	928	151	1079	231	1310	10	46.7
T9-22B	TraesCS4B02G124000	1969	151	2120	186	2306	19	81.4
T9-70A	TraesCS3A02G428800	1678	151	1729	145	1874	14	72.5
T10-44D	TraesCS4D02G159600	602	151	753	0	753	5	28.7
T9-22D	TraesCS4D02G122000	1047	150	1197	162	1359	9	40.5
T9-61D	TraesCS5D02G481200	1249	149	1398	120	1518	11	45.3
T9-63A	TraesCS5A02G477600	1844	149	1993	312	2305	15	78.2
T2-57D	TraesCS2D02G308600	1813	148	1961	231	2192	12	64.9
T1-4D	TraesCS3D02G143300	1971	147	2118	236	2354	14	65.9
T4-31B	TraesCS6B02G175100	2240	147	2387	361	2748	10	56.2
T10-10D	TraesCS3D02G125600	2315	147	2462	130	2592	17	77.2
T8-1B	TraesCS3B02G217100	1781	146	1927	216	2143	16	73.4
T8-2A	TraesCS2A02G212200	1815	145	1960	0	1960	12	58.4
T9-76A	TraesCS7A02G363100	1261	145	1406	496	1902	7	38.4
T2-43A	TraesCS7A02G120300	1596	144	1740	0	1740	12	55.1
T5-8D	TraesCS2D02G387100	1891	144	2035	268	2303	18	86.8
T10-14A	TraesCS7A02G233700	1110	143	1253	387	1640	12	51.8
T2-7B	TraesCS4B02G047300	1707	142	1849	450	2299	11	54.6
T8-1A	TraesCS3A02G187800	1833	142	1975	235	2210	21	91.1
T8-6D	TraesCS2D02G331700	1114	142	1256	0	1256	9	40.5
T9-58D	TraesCS1D02G214200	1845	142	1987	24	2011	10	60.7
T2-27D	TraesCS5D02G404500	1892	141	2033	92	2125	20	80.7
T2-55A	TraesCS7A02G549300	1905	141	2046	0	2046	17	76
T8-5B	TraesCS4B02G191200	1965	141	2106	163	2269	9	50.4
T1-7D	TraesCS4D02G290900	1730	140	1870	263	2133	17	80.5
T4-25D	TraesCS4D02G207800	1641	140	1781	175	1956	18	74.4
T4-3D	TraesCS4D02G326400	1700	140	1840	290	2130	16	73.4
T9-38B	TraesCS6B02G033300	990	140	1130	190	1320	9	40.5
T6-7A	TraesCS5A02G185700	1860	140	2000	158	2158	19	84.7
T6-14A	TraesCS6A02G335400	1636	138	1774	270	2044	19	81.4
T9-50A	TraesCS5A02G069500	2178	137	2315	304	2619	13	68.7
T5-16(A)-B	TraesCS5B02G494500	1834	136	1970	421	2391	15	76.2
T4-8B	TraesCS2B02G433400	394	135	529	2001	2530	25	53.7
T5-29A	TraesCS3A02G119500	1878	135	2013	239	2252	12	64.9
T9-63D	TraesCS5D02G491100	1283	135	1418	114	1532	12	61.6
T2-58D	TraesCS7D02G249500	810	134	944	341	1285	2	10.8
T5-3B	TraesCS1B02G235100	1609	134	1743	135	1878	11	61.2
T9-76B	TraesCS7B02G264800	1798	134	1932	338	2270	17	79.3
T6-8B	TraesCS5B02G183700	1872	134	2006	290	2296	22	86.1
T5-23A	TraesCS5A02G384100	1788	133	1921	0	1921	17	77.2
T9-5A	TraesCS2A02G386400	1902	133	2035	228	2263	15	72.9
T8-10D	TraesCS2D02G262700	1501	132	1633	2070	3703	28	60.4
T8-27D	TraesCS7D02G176700	1901	132	2033	314	2347	13	58.4
T2-35A	TraesCS5A02G472000	1931	131	2062	113	2175	18	80.9
T4-46B	TraesCS5B02G224800	1577	131	1708	723	2431	14	68.6
T5-3A	TraesCS1A02G221900	1291	131	1422	2383	3805	29	66.1
T8-1D	TraesCS3D02G191400	1872	131	2003	340	2343	21	87.8
T9-50B	TraesCS5B02G075800	2000	131	2131	6	2137	16	73.4
T6-14B	TraesCS6B02G366100	1571	131	1702	44	1746	19	81.4
T4-32B	TraesCS7B02G418400	2042	130	2172	131	2303	18	77.6
T2-7A	TraesCS4A02G267000	510	129	639	507	1146	1	7.1
T5-30D	TraesCS1D02G120000	874	129	1003	169	1172	5	25.4
T9-38A	TraesCS6A02G024300	1710	129	1839	196	2035	11	53.4
T9-76D	TraesCS7D02G359900	1905	129	2034	299	2333	17	79.3
T6-8A	TraesCS5A02G185600	1613	129	1742	313	2055	11	56.7
T2-5B	TraesCS7B02G183300	1753	128	1881	299	2180	15	69.6
T2-51D	TraesCS2D02G468900	3240	128	3368	66	3434	37	85.4
T2-59D	TraesCS1D02G246300	1241	128	1369	107	1476	2	10.8
T4-40B	TraesCS1B02G194700	407	128	535	249	784	4	20.9
T4-51A	TraesCS1A02G089500	1716	128	1844	326	2170	13	63.2
T8-24B	TraesCS2B02G071100	1680	128	1808	840	2648	19	50.7
T9-51B	TraesCS2B02G119300	1623	128	1751	0	1751	15	63.4
T4-20-4A	TraesCS4A02G485900	1885	127	2012	253	2265	14	69.2
T4-38D	TraesCS1D02G126200	1486	127	1613	0	1613	9	47.1
T5-3D	TraesCS1D02G223600	1041	127	1168	2320	3488	25	58
T9-38D	TraesCS6D02G027700	1994	127	2121	63	2184	12	55.1
T9-42A	TraesCS2A02G467000	1682	126	1808	173	1981	14	65.9
T1-10B	TraesCS2B02G311900	1209	124	1333	296	1629	9	43.8
T1-12B	TraesCS1B02G107000	1854	124	1978	407	2385	9	40
T1-9A	TraesCS7A02G260500	1637	124	1761	0	1761	12	61.6
T8-21D	TraesCS2D02G486600	1746	123	1869	180	2049	18	74.4
T9-59D	TraesCS5D02G158500	1954	123	2077	229	2306	4	20.5
T9-60D	TraesCS4D02G094400	1954	123	2077	229	2306	13	67.5
T1-15A	TraesCS3A02G194100	989	122	1111	168	1279	4	24.9
T1-16A	TraesCS1A02G350200	1178	122	1300	1029	2329	22	67.8
T2-8D	TraesCS6D02G288800	0	122	122	844	966	4	18.4
T1-2D	TraesCS3D02G172900	1415	121	1536	343	1879	2	14.1
T4-40D	TraesCS1D02G189800	1480	121	1601	234	1835	10	50.1
T9-11B	TraesCS1B02G247700	1727	121	1848	2366	4214	32	71.2
T1-16B	TraesCS1B02G364500	1851	120	1971	0	1971	11	48
T2-18B	TraesCS7B02G134200	1547	120	1667	47	1714	12	64.9
T2-49A	TraesCS1A02G137200	1938	118	2056	229	2285	5	28.7
T4-32B(2)	TraesCS7B02G418500	1063	118	1181	218	1399	6	38.5
T8-22B	TraesCS1B02G351600	1858	117	1975	201	2176	15	65.8
T9-42B	TraesCS2B02G489800	1334	117	1451	220	1671	8	43.3
T10-34D	TraesCS3D02G396500	2174	117	2291	337	2628	4	20.5
T10-46D	TraesCS2D02G337000	1892	117	2009	369	2378	20	88.5
T6-9A	TraesCS1A02G065700	1071	117	1188	1281	2469	15	46.9
T1-18D	TraesCS2D02G385900	1978	116	2094	162	2256	11	53.4
T4-43A	TraesCS3A02G270800	1897	116	2013	0	2013	9	50.4
T4-9D	TraesCS2D02G227500	1454	116	1570	571	2141	10	60.7
T8-15B	TraesCS1B02G364800	1154	116	1270	193	1463	7	35.1
T8-22D	TraesCS1D02G341100	1936	116	2052	1066	3118	18	54.8
T4-24B	TraesCS4B02G207100	1295	115	1410	202	1612	10	53.6
T9-11A	TraesCS1A02G236100	1791	115	1906	2444	4350	35	83.8
T9-23B	TraesCS4B02G043100	1744	115	1859	179	2038	16	70.1
T10-17B	TraesCS2B02G234700	523	115	638	217	855	6	25.3
T9-60A	TraesCS4A02G219700	2271	114	2385	250	2635	7	36.2
T8-5D	TraesCS4D02G192400	1146	113	1259	195	1454	9	47.1
T9-58B	TraesCS1B02G225000	1734	113	1847	50	1897	12	61.6
T9-59A	TraesCS5A02G158000	1694	113	1807	211	2018	15	69.6
T10-38A	TraesCS5A02G303200	972	113	1085	155	1240	8	40
T5-16A	TraesCS5A02G481400	1940	112	2052	164	2216	15	76.2
T9-16B	TraesCS5B02G406100	1414	112	1526	34	1560	11	51.3
T2-49D	TraesCS1D02G138100	2010	111	2121	293	2414	10	50.8
T8-26D	TraesCS6D02G021100	1356	111	1467	348	1815	12	58.4
T2-59A	TraesCS1A02G247100	1700	110	1810	0	1810	9	47.1
T4-14A	TraesCS3A02G105400	1724	110	1834	111	1945	14	65.9
T2-16D	TraesCS2D02G231700	465	109	574	161	735	1	7.1
T4-14D	TraesCS3D02G107500	1738	109	1847	90	1937	12	58.4
T4-36B	TraesCS5B02G236300	2670	109	2779	19	2798	11	63.3
T2-55B	TraesCS7B02G472300	1876	108	1984	0	1984	21	88.9
T4-54A	TraesCS2A02G176700	1251	108	1359	0	1359	10	65.7
T9-4A	TraesCS1A02G113400	1310	108	1418	306	1724	11	50.1
T2-12D	TraesCS7D02G430700	1721	107	1828	0	1828	17	72.7
T9-57D	TraesCS6D02G324200	1728	107	1835	6	1841	15	72.9
T2-13A	TraesCS2A02G220400	1868	105	1973	396	2369	19	84.7
T4-43D	TraesCS3D02G270500	786	105	891	444	1335	4	23.9
T5-26D	TraesCS3D02G046300	871	105	976	247	1223	9	90.7
T9-57A	TraesCS6A02G343300	1554	105	1659	58	1717	7	39.2
T9-57B	TraesCS6B02G375400	1773	105	1878	30	1908	15	71.8
T10-1A	TraesCS4A02G105600	1292	105	1397	321	1718	11	54.6
T10-35B	TraesCS4B02G280400	1142	105	1247	148	1395	12	51.8
T10-6A	TraesCS2A02G315000	1925	105	2030	19	2049	8	46.6
T4-10B	TraesCS4B02G197800	1661	104	1765	319	2084	14	65.2
T9-10D	TraesCS7D02G478800	2369	104	2473	289	2762	17	79.3
T2-35B	TraesCS5B02G484700	1939	103	2042	133	2175	19	81.4
T9-36D	TraesCS7D02G304800	1918	103	2021	288	2309	11	64.5
T1-22D	TraesCS7D02G187000	1449	102	1551	21	1572	13	65.4
T4-17D	TraesCS1D02G136400	1838	102	1940	145	2085	22	89.4
T4-51D	TraesCS1D02G098000	1731	101	1832	297	2129	12	55.1
T10-11D	TraesCS7D02G044700	857	101	958	304	1262	16	85.4
T10-12B	TraesCS5B02G171700	1872	101	1973	0	1973	11	53.4
T4-3B	TraesCS4B02G329500	1790	100	1890	102	1992	15	76.2
T9-68A	TraesCS2A02G551100	1724	100	1824	0	1824	15	76.2
T10-1D	TraesCS4D02G199200	1796	100	1896	116	2012	17	77.2
T2-13B	TraesCS2B02G245900	1760	99	1859	175	2034	23	89.9
T8-5A	TraesCS4A02G113000	892	99	991	0	991	6	60.1
T2-60A	TraesCS5A02G395200	1923	98	2021	160	2181	2	10.8
T4-49A	TraesCS1A02G338700	1913	98	2011	261	2272	21	85.6
T9-58A	TraesCS1A02G210900	1478	98	1576	0	1576	13	68.7
T10-6B	TraesCS2B02G333500	1775	98	1873	54	1927	4	24.9
T2-27A	TraesCS5A02G395200	1852	97	1949	46	1995	19	83.5
T8-26A	TraesCS6A02G018200	1984	97	2081	305	2386	15	71.8
T10-34A	TraesCS3A02G401700	1650	97	1747	246	1993	20	87.8
T4-24D	TraesCS4D02G207900	1215	96	1311	178	1489	4	17.8
T4-51B	TraesCS1B02G117400	1880	96	1976	295	2271	20	87.3
T8-3A	TraesCS5A02G233600	2037	96	2133	256	2389	16	52.1
T9-47A	TraesCS6A02G315100	705	96	801	0	891	2	10.8
T9-74B	TraesCS5B02G156400	2825	96	2921	213	3134	20	80.7
T6-16A	TraesCS6A02G031200	2115	96	2211	198	2409	16	73.4
T4-30-5A	TraesCS5A02G545100	1982	95	2077	2459	4536	33	75.3
T4-32A	TraesCS7A02G508800	1675	95	1770	267	2037	7	39.5
T1-22B	TraesCS7B02G090500	435	94	529	83	612	2	10.8
T9-26D	TraesCS6D02G382600	1723	93	1816	120	1936	14	69.2
T9-59B	TraesCS7B02G062200	678	93	771	259	1030	5	28.7
T6-17A	TraesCS6A02G031000	1548	93	1641	276	1917	12	61.6
T9-61B	TraesCS5B02G480900	1818	92	1910	227	2137	8	40
T9-74A	TraesCS5A02G157300	1712	92	1804	241	2045	13	62.1
T8-10B	TraesCS2B02G281000	1824	91	1915	0	1915	16	76.7
T9-26B	TraesCS6B02G438700	679	91	770	282	1052	4	18.4
T10-21D	TraesCS6D02G182800	1369	91	1460	280	1740	11	56.4
T1-22A	TraesCS7A02G185300	520	90	610	219	829	3	14.6
T10-46B	TraesCS2B02G356200	1768	90	1858	370	2228	8	43.3
T4-42D	TraesCS3D02G285800	1808	89	1897	194	2091	12	68.2
T10-34B	TraesCS3B02G434600	1796	89	1885	196	2081	15	65.6
T9-65B	TraesCS2B02G117900	1727	88	1815	85	1900	20	88.5
T10-14B	TraesCS7B02G132000	1700	88	1788	479	2267	5	28.7
T2-16A	TraesCS2A02G220800	765	87	852	0	852	2	10.8
T9-43D	TraesCS2D02G259200	1613	87	1700	197	1897	18	74.4
T4-39A	TraesCS2A02G259500	1647	86	1733	2439	4172	27	70.6
T8-12A	TraesCS2A02G443100	2299	85	2384	274	2658	18	77.6
T8-25A	TraesCS2A02G053200	1716	85	1801	215	2016	19	81.4
T6-17D	TraesCS6D02G035700	2137	85	2222	375	2597	13	65.8
T2-31A	TraesCS1A02G159300	1812	84	1896	117	2013	14	76.3
T2-39D	TraesCS5D02G182600	766	84	850	192	1042	6	29.2
T4-42B	TraesCS3B02G287100	1686	84	1770	370	2140	12	64.9
T9-35A	TraesCS5A02G142700	1586	84	1670	245	1915	10	57.4
T9-45B	TraesCS5B02G480900	2024	84	2108	0	2108	21	87.8
T4-39B	TraesCS2B02G282400	405	83	488	2502	2990	19	49.2
T4-46D	TraesCS5D02G233700	1677	83	1760	792	2552	16	76.7
T9-65D	TraesCS2D02G100200	2005	83	2088	100	2188	21	82.4
T6-6D	TraesCS4D02G361500	1986	83	2069	258	2327	14	61.4
T4-39D	TraesCS2D02G264200	1745	82	1827	294	2121	12	68.2
T9-26A	TraesCS6A02G398400	758	82	840	185	1025	11	57.9
T9-34B	TraesCS1B02G265400	1777	82	1859	208	2067	17	80.5
T9-35D	TraesCS5D02G149400	1506	81	1587	270	1857	11	61.2
T8-15A	TraesCS1A02G350500	250	80	330	2157	2487	23	50.5
T10-25D	TraesCS4D02G092800	448	80	528	288	816	2	10.8
T2-22B	TraesCS2B02G144300	596	79	675	54	729	9	38.7
T4-29D	TraesCS7D02G534700	1563	79	1642	77	1719	14	61.1
T5-20D	TraesCS4D02G109500	896	79	975	116	1091	8	39.9
T4-40A	TraesCS1A02G186800	917	78	995	184	1179	3	17.9
T10-28A	TraesCS2A02G171900	588	78	666	261	927	3	17.9
T9-74D	TraesCS5D02G162600	841	76	917	184	1101	8	34.2
T4-19-4A	TraesCS4A02G486000	559	75	634	953	1587	4	28.2
T2-14A	TraesCS3A02G111400	1516	74	1590	205	1795	15	69.6
T4-29A	TraesCS7A02G548000	1853	74	1927	56	1983	18	77.6
T8-25D	TraesCS2D02G053400	1871	74	1945	226	2171	22	92.7
T2-55D	TraesCS7D02G535600	2022	72	2094	0	2094	22	88.2
T4-19-7A	TraesCS7A02G009100	546	72	618	859	1477	3	17.9
T10-11B	TraesCS4B02G045000	921	72	993	137	1130	10	46.4
T6-16D	TraesCS6D02G035600	1915	72	1987	188	2175	16	80
T4-19-7D	TraesCS7D02G008700	992	71	1063	1017	2080	8	38.8
T2-14D	TraesCS3D02G113600	306	70	376	253	629	1	7.1
T2-15B	TraesCS6B02G457100	308	70	378	108	486	10	43.1
T4-29B	TraesCS7B02G471500	1813	70	1883	218	2101	20	87.3
T4-37A	TraesCS7A02G049400	1825	70	1895	367	2262	15	75.1
T8-2D	TraesCS2D02G218100	1734	69	1803	0	1803	14	75.8
T10-16D	TraesCS1D02G059600	1586	69	1655	187	1842	6	29.2
T2-25A	TraesCS7A02G133500	2100	68	2168	302	2470	24	99.1
T9-54A	TraesCS7A02G141600	1940	68	2008	276	2284	15	65.2
T10-21B	TraesCS6B02G229400	1695	67	1762	142	1904	12	58.4
T4-15-6B	TraesCS6B02G113900	2337	66	2403	384	2787	17	82.6
T2-29B	TraesCS3B02G397900	1702	65	1767	157	1924	8	39.1
T8-12B	TraesCS2B02G464200	1883	64	1947	232	2179	17	73.9
T2-29D	TraesCS3D02G359200	1859	63	1922	265	2187	6	39.1
T4-18-7D	TraesCS7D02G009400	1977	63	2040	0	2040	15	78.4
T9-29B	TraesCS5B02G143100	2061	63	2124	117	2241	24	92.5
T6-6B	TraesCS4B02G375800	492	63	555	351	906	9	43.1
T9-25B	TraesCS4B02G376600	1782	62	1844	272	2116	11	61.2
T10-22A	TraesCS3A02G381500	2192	62	2254	137	2391	15	71.8
T1-4(B)D	TraesCS3D02G143500	940	61	1001	114	1115	4	24.9
T9-72B	TraesCS4B02G375600	2001	61	2062	268	2330	18	77.6
T1-25B	TraesCS5B02G113100	1325	60	1385	224	1609	5	28.8
T2-17B	TraesCS3B02G262600	1606	60	1666	0	1666	15	66.4
T4-24A	TraesCS4A02G097400	1705	60	1765	235	2000	10	49.6
T9-60B	TraesCS4B02G098200	1325	60	1385	38	1423	10	54.4
T1-28-2B	TraesCS2B02G566500	1726	56	1782	306	2088	16	66.8
T1-28-3D	TraesCS3D02G024700	1287	56	1343	259	1602	7	39.5
T1-4B	TraesCS3B02G161000	1173	56	1229	133	1362	4	24.9
T9-7D	TraesCS2D02G421700	1878	51	1929	129	2058	17	83.8
T4-48A	TraesCS2A02G136800	1878	50	1928	314	2242	16	76.7
T6-31B	TraesCS4B02G317200	2019	50	2069	221	2290	21	92.2
T1-28-3A	TraesCS3A02G021900	1726	49	1775	344	2119	14	68
T9-24D	TraesCS4D02G001700	2038	49	2087	218	2305	26	100
T4-33-4A	TraesCS4A02G384800	1748	48	1796	310	2106	20	77.4
T4-37D	TraesCS7D02G044500	1968	47	2015	452	2467	16	70.1
T4-46A	TraesCS5A02G226400	1815	46	1861	368	2229	17	82.6
T9-33B	TraesCS1B02G100300	948	46	994	254	1248	9	45.9
T6-6A	TraesCS4A02G537100	1335	46	1381	300	1681	12	54
T4-34D	TraesCS4D02G015800	1662	44	1706	212	1918	21	91.1
T9-55D	TraesCS7D02G149400	533	44	577	197	774	1	7.1
T6-31A	TraesCS4A02G416400	1174	43	1217	316	1533	14	47.3
T2-16B	TraesCS2B02G251100	998	42	1040	253	1293	7	32.9
T2-2B	TraesCS5B02G244300	1704	42	1746	387	2133	14	59.3
T2-20B	TraesCS3B02G556500	1900	42	1942	231	2173	17	83.8
T4-28-5B	TraesCS5B02G544800	1735	42	1777	273	2050	22	89.4
T10-22D	TraesCS3D02G374700	1999	39	2038	0	2038	22	89.4
T9-24A	TraesCS4A02G312500	1923	38	1961	271	2232	23	93.2
T9-4D	TraesCS1D02G114800	1403	38	1441	298	1739	7	38.4
T2-31D	TraesCS1D02G166600	1856	37	1893	139	2032	8	43.3
T4-50D	TraesCS5D02G268800	2443	37	2480	11	2491	12	61.6
T4-38A	TraesCS1A02G125200	1761	35	1796	144	1940	17	73.9
T4-52B	TraesCS7B02G381400	2441	35	2476	0	2476	20	84
T9-54D	TraesCS7D02G142900	1985	35	2020	140	2160	13	60.9
T10-41D	TraesCS5D02G294200	1884	35	1919	338	2257	13	65.4
T9-43B	TraesCS2B02G277300	1667	33	1700	365	2065	25	96.2
T10-25A	TraesCS4A02G217700	1889	33	1922	471	2393	19	84.1
T9-18B	TraesCS6B02G303600	1683	31	1714	0	1714	17	89.9
T10-13B	TraesCS2B02G150900	1953	31	1984	448	2432	14	64.7
T10-13D	TraesCS2D02G157500	1964	31	1995	476	2471	11	53.4
T6-13D	TraesCS6D02G383600LC	1777	31	1808	289	2097	15	76.2
T9-54B	TraesCS7B02G044400	1969	30	1999	424	2423	13	65.4
T10-13A	TraesCS5A02G152300	1888	30	1918	308	2226	12	57.2
T8-3D	TraesCS5D02G240500	1983	29	2012	182	2194	14	69.2
T10-16B	TraesCS1B02G075900	816	29	845	301	1146	8	36.7
T10-20	TraesCS7D02G296000	1911	29	1940	282	2222	13	72
T4-8D	TraesCS2D02G411600	796	28	824	0	824	3	17.9
T2-31B	TraesCS1B02G184900	2051	26	2077	119	2196	13	67.5
T2-8A	TraesCS6A02G309400	0	26	26	753	779	4	21.6
T4-47B	TraesCS3B02G148100	2117	22	2139	127	2266	20	47.8
T2-8B	TraesCS6B02G339100	0	21	21	744	765	4	18.4
T10-30A	TraesCS1A02G500600	1567	20	1587	56	1643	6	32.5
T2-54B	TraesCS2B02G390700	1371	16	1387	217	1604	17	70.6
T10-22B	TraesCS3B02G414200	2092	14	2106	0	2106	14	62.6
T2-20A	TraesCS3A02G508400	1844	10	1854	420	2274	21	95.5
T2-35D	TraesCS5D02G484500	1965	9	1974	0	1974	9	47.3
T2-25B	TraesCS7B02G034600	1967	8	1975	367	2342	23	99.1
T8-3B	TraesCS5B02G232100	1974	7	1981	85	2066	17	77
T1-10A	TraesCS2A02G295400	693	0	693	1687	2380	16	47.6
T1-10D	TraesCS2D02G293200	962	0	962	1499	2461	18	57.7
T1-11A	TraesCS5A02G373900	1497	0	1497	0	1497	13	58.2
T1-11D	TraesCS5D02G383300	1453	0	1453	0	1453	12	52.4
T1-18B	TraesCS2B02G379300	1779	0	1779	311	2090	13	60.9
T1-25A	TraesCS5A02G102400	1515	0	1515	158	1673	9	53.6
T1-25D	TraesCS5D02G094200	1557	0	1557	341	1898	8	40
T1-26D	TraesCS5D02G033700	0	0	0	2218	2218	16	72.2
T1-5A	TraesCS2A02G188500	1328	0	1328	0	1328	10	47.5
T1-5B	TraesCS2B02G405700	1649	0	1649	227	1876	14	65.9
T2-17D	TraesCS3D02G219900	544	0	544	270	814	2	14.1
T2-20D	TraesCS3D02G501900	2082	0	2082	80	2162	19	86.8
T2-22A	TraesCS2A02G122200	1730	0	1730	3016	4746	45	85.4
T2-23A	TraesCS7A02G495700	2705	0	2705	0	2705	15	68.5
T2-25D	TraesCS7D02G133100	1854	0	1854	361	2215	25	99.5
T2-26B	TraesCS6B02G008200	1857	0	1857	351	2208	23	92.4
T2-26D	TraesCS6D02G000200	2013	0	2013	482	2495	26	100
T2-27B	TraesCS5B02G400000	0	0	0	2160	2160	20	85.2
T2-28B	TraesCS3B02G111100	2297	0	2297	475	2772	17	85.9
T2-28D	TraesCS3D02G096000	1667	0	1667	273	1940	11	65.3
T2-29A	TraesCS3A02G366400	0	0	0	1921	1921	11	61.4
T2-30D	TraesCS3D02G095700	2074	0	2074	268	2342	16	75.5
T2-32B	TraesCS7B02G072000	1611	0	1611	492	2103	17	71.4
T2-32D	TraesCS7D02G168000	1789	0	1789	434	2223	12	55.9
T2-34B	TraesCS4B02G328100	1583	0	1583	251	1834	17	70.6
T2-34D	TraesCS4D02G325100	1749	0	1749	426	2175	19	81.4
T2-36B	TraesCS5B02G003500	1901	0	1901	239	2140	17	76
T2-39A	TraesCS5A02G292900L	1597	0	1597	274	1871	13	58.8
T2-4A	TraesCS3A02G440800	1871	0	1871	1276	3147	24	78.2
T2-40A	TraesCS1A02G466500L	1790	0	1790	318	2108	16	83.3
T2-42D	TraesCS5D02G004100	1567	0	1567	213	1780	16	65.6
T2-43D	TraesCS7D02G117800	0	0	0	2013	2013	13	62.1
T2-44D	TraesCS5D02G004300	1065	0	1065	268	1333	11	43.5
T2-45A	TraesCS7A02G189000	2167	0	2167	0	2167	14	69.2
T2-45D	TraesCS7D02G190100	1745	0	1745	0	1745	10	59.5
T2-46B	TraesCS7B02G746900L	2086	0	2086	100	2186	19	88
T2-47A	TraesCS6A02G307800	1346	0	1346	0	1346	13	56.2
T2-52B	TraesCS1B02G368500	1440	0	1440	0	1440	6	35.9
T2-57B	TraesCS2B02G327300	0	0	0	2314	2314	8	42.1
T2-58A	TraesCS7A02G251400	1682	0	1682	0	1682	6	32.5
T2-58B	TraesCS7B02G139700	1958	0	1958	0	1958	21	81.2
T2-59B	TraesCS1B02G257700	1959	0	1959	183	2142	8	31.3
T2-7D	TraesCS4D02G047300	1325	0	1325	265	1590	8	36.7
T2-9A	TraesCS3A02G036600	1556	0	1556	133	1689	12	61.6
T4-1A	TraesCS7A02G264400	1986	0	1986	79	2065	18	74.4
T4-1B	TraesCS7B02G162500	0	0	0	1731	1731	11	51.3
T4-1D	TraesCS7D02G265400	0	0	0	1180	1180	9	39.8
T4-12A	TraesCS7A02G373500	1104	0	1104	250	1354	13	52.2
T4-12B	TraesCS7B02G253500	1987	0	1987	401	2388	18	77.6
T4-12D	TraesCS7D02G349600	1930	0	1930	197	2127	22	91.5
T4-14B	TraesCS3B02G158300LC	1181	0	1181	0	1181	10	43.1
T4-15-1D	TraesCS1D02G018500	734	0	734	278	1012	7	28.5
T4-16B	TraesCS7B02G381100LC	1013	0	1013	735	1748	15	72.9
T4-16D	TraesCS7D02G307000	922	0	922	234	1156	14	71.1
T4-17A	TraesCS1A02G131600	1477	0	1477	1818	3295	18	50.4
T4-2A	TraesCS6A02G417900	0	0	0	2316	2316	19	90.6
T4-2B	TraesCS6B02G471100	1189	0	1189	983	2172	20	81.9
T4-2D	TraesCS6D02G403200	0	0	0	1748	1748	16	82.6
T4-20-7A	TraesCS7A02G009200	1869	0	1869	270	2139	18	79.8
T4-21B	TraesCS6B02G179900	2221	0	2221	366	2587	5	28.7
T4-22B	TraesCS2B02G413000	1788	0	1788	0	1788	12	61.6
T4-22D	TraesCS2D02G392800	1806	0	1806	0	1806	6	32.3
T4-30-4B	TraesCS4B02G378500	0	0	0	2411	2411	15	76.2
T4-34A	TraesCS4A02G296300	1934	0	1934	260	2194	17	66.1
T4-38B	TraesCS3B02G144800	2498	0	2498	0	2498	14	69.2
T4-4A	TraesCS3A02G049500	1050	0	1050	138	1188	17	76
T4-4B	TraesCS3B02G056100	1900	0	1900	111	2011	17	77.2
T4-4D	TraesCS3D02G049300	1450	0	1450	219	1669	22	89.4
T4-41-4B	TraesCS4B02G346900	1916	0	1916	0	1916	7	46.1
T4-41-5A	TraesCS5A02G515600	1667	0	1667	0	1667	12	61.6
T4-42A	TraesCS4A02G017000	2033	0	2033	433	2466	15	69.6
T4-44A	TraesCS5A02G453200	1921	0	1921	0	1921	19	88
T4-44B	TraesCS5B02G462200	1080	0	1080	210	1290	10	54.4
T4-44D	TraesCS5D02G464000	1589	0	1589	232	1821	15	69.6
T4-45B	TraesCS7B02G766300LC	1104	0	1104	68	1172	9	40.5
T4-45D	TraesCS7D02G524300	1909	0	1909	0	1909	20	85.2
T4-48B	TraesCS2B02G160700	2022	0	2022	0	2022	16	76.7
T4-49B	TraesCS1B02G351200	1896	0	1896	232	2128	16	69.1
T4-50A	TraesCS5A02G261400	2074	0	2074	0	2074	16	83.3
T4-52A	TraesCS7A02G478700	1855	0	1855	0	1855	16	73.4
T4-54B	TraesCS2B02G081700	1627	0	1627	0	1627	14	69.2
T4-54D	TraesCS2D02G178000	2153	0	2153	155	2308	15	72.9
T4-55B	TraesCS2B02G603100	1670	0	1670	285	1955	12	61.2
T4-55D	TraesCS2D02G596300	2054	0	2054	340	2394	18	83.1
T4-57B	TraesCS3B02G238500	1530	0	1530	175	1705	16	70.1
T4-6A	TraesCS6A02G203600.3	520	0	520	1738	2258	12	71.5
T4-6D	TraesCS6D02G186900	0	0	0	2624	2624	8	42.1
T4-60A	TraesCS6A02G089400	982	0	982	0	982	9	46.8
T4-7B	TraesCS7B02G182300	0	0	0	2370	2370	18	79.8
T4-9A	TraesCS2A02G347800LC	1919	0	1919	327	2246	19	86.8
T4-9B	TraesCS2B02G255300	1965	0	1965	91	2056	15	76.2
T5-1D	TraesCS4D02G267500	1330	0	1330	179	1509	11	56.7
T5-11D	TraesCS1D02G162400	2004	0	2004	152	2156	12	60.5
T5-16(B)-A	TraesCS5A02G481300	1918	0	1918	0	1918	10	60.7
T5-18D	TraesCS3D02G099500	2069	0	2069	0	2069	11	60.5
T5-19A	TraesCS2A02G432400	2049	0	2049	296	2345	24	95.8
T5-19B	TraesCS2B02G453700	1938	0	1938	81	2019	16	76.7
T5-2B	TraesCS3B02G115400	1975	0	1975	0	1975	12	57.6
T5-2D	TraesCS3D02G099500	2071	0	2071	0	2071	11	60
T5-20A	TraesCS4A02G208400	924	0	924	229	1153	8	38.8
T5-22B	TraesCS3B02G055700LC	1900	0	1900	401	2301	20	93.9
T5-22D	TraesCS3D02G045700	1901	0	1901	290	2191	14	69.2
T5-23D	TraesCS5D02G393600	1915	0	1915	0	1915	18	80.9
T5-29B	TraesCS3B02G139100	834	0	834	33	867	4	24.9
T5-30A	TraesCS1A02G119100	601	0	601	152	753	3	15.1
T5-4D	TraesCS3D02G099500	2071	0	2071	0	2071	12	57.6
T5-5A	TraesCS7A02G164000	250	0	250	1135	1385	9	50.6
T5-6B	TraesCS5B02G132600	954	0	954	569	1523	3	14.6
T5-6D	TraesCS5D02G140400	1028	0	1028	367	1395	4	17.2
T5-7D	TraesCS5D02G200900	1854	0	1854	271	2125	9	39.3
T8-11B	TraesCS1B02G214500	1231	0	1231	226	1457	6	35.8
T8-14A	TraesCS4A02G016400	0	0	0	2545	2545	19	86.8
T8-17B	TraesCS2B02G405700	1657	0	1657	619	2276	12	57.4
T8-21B	TraesCS2B02G486900	1434	0	1434	0	1434	13	59.8
T8-24D	TraesCS2D02G057300	1432	0	1432	1039	2471	17	79.3
T8-25B	TraesCS2B02G067100	1881	0	1881	351	2232	16	73.4
T8-6A	TraesCS2A02G331200	925	0	925	0	925	6	25.9
T8-6B	TraesCS2B02G351000	899	0	899	0	899	5	25.4
T9-12A	TraesCS1A02G278400	2469	0	2469	0	2469	21	87.8
T9-12B	TraesCS1B02G287300	1904	0	1904	202	2106	18	79.8
T9-12D	TraesCS1D02G277600	2047	0	2047	0	2047	17	72.7
T9-14A	TraesCS4A02G111100	1810	0	1810	272	2082	12	58.4
T9-14B	TraesCS4B02G207600	2212	0	2212	154	2366	15	50
T9-15B	TraesCS7B02G315200	1932	0	1932	751	2683	10	60.7
T9-16A	TraesCS5A02G401300	1819	0	1819	162	1981	15	69.6
T9-16D	TraesCS5D02G411600	690	0	690	23	713	5	28.7
T9-18A	TraesCS6A02G276100	740	0	740	0	740	7	32.9
T9-20B	TraesCS2B02G214600	1961	0	1961	0	1961	18	84.2
T9-21A	TraesCS6A02G138600	1676	0	1676	0	1676	12	55.1
T9-22A	TraesCS4A02G191300	0	0	0	2330	2330	15	68.9
T9-23A	TraesCS4A02G271000	2013	0	2013	0	2013	19	76.9
T9-23D	TraesCS4D02G040400	1213	0	1213	63	1276	14	59
T9-24B	TraesCS4B02G001200	1983	0	1983	297	2280	11	61.2
T9-25D	TraesCS4D02G361700	783	0	783	554	1337	5	25.4
T9-25A	TraesCS4A02G537300	1627	0	1627	164	1791	8	40
T9-28A	TraesCS4A02G415400	1117	0	1117	338	1455	8	40
T9-28B	TraesCS4B02G316200	1723	0	1723	374	2097	14	69.2
T9-28D	TraesCS4D02G312800	1767	0	1767	323	2090	17	77.2
T9-3A	TraesCS3A02G320100	1363	0	1363	26	1389	10	47.5
T9-3B	TraesCS6B02G350900	1313	0	1313	0	1313	9	47.1
T9-3D	TraesCS6D02G299600	1900	0	1900	208	2108	21	87.8
T9-30D	TraesCS5D02G370200	1936	0	1936	165	2101	18	77.6
T9-31D	TraesCS4D02G077600	1682	0	1682	299	1981	10	47.5
T9-32B	TraesCS6B02G252000	696	0	696	303	999	3	14.6
T9-33A	TraesCS1A02G082700	1186	0	1186	530	1716	4	21.6
T9-33D	TraesCS1D02G084100	2048	0	2048	349	2397	4	20.9
T9-36A	TraesCS7A02G307900	609	0	609	293	902	6	29.2
T9-40A	TraesCS2A02G230100	2241	0	2241	127	2368	15	69.6
T9-40B	TraesCS2B02G251700	1654	0	1654	0	1654	10	54.1
T9-41A	TraesCS2A02G309700	2124	0	2124	283	2407	14	71.3
T9-45D	TraesCS7D02G330200	2046	0	2046	0	2046	20	90.6
T9-46A	TraesCS5A02G597900LC	1894	0	1894	158	2052	15	66.4
T9-46B	TraesCS5B02G446100	1859	0	1859	0	1859	15	61.9
T9-46D	TraesCS5D02G323500	1864	0	1864	61	1925	10	54.1
T9-47D	TraesCS6D02G294400	793	0	793	0	793	2	10.8
T9-5B	TraesCS2B02G403800	2107	0	2107	160	2267	19	84.7
T9-5D	TraesCS2D02G383200	2146	0	2146	171	2317	17	77.2
T9-53A	TraesCS1A02G196100	1199	0	1199	416	1615	9	42.6
T9-55B	TraesCS7B02G048800LC	1573	0	1573	0	1573	12	53.9
T9-56D	TraesCS1D02G219600	1130	0	1130	368	1498	2	10.8
T9-61A	TraesCS5A02G468300	1567	0	1567	0	1567	12	54
T9-64A	TraesCS7A02G422500	1756	0	1756	0	1756	11	50.1
T9-73A	TraesCS3A02G176100	1944	0	1944	193	2137	14	79.8
T9-75B	TraesCS6B02G214900	1999	0	1999	336	2335	11	61.2
T9-75D	TraesCS6D02G173000	1871	0	1871	0	1871	8	45.4
T9-77B	TraesCS7B02G120800	1715	0	1715	0	1715	17	70.6
T9-77D	TraesCS7D02G215600	1907	0	1907	0	1907	12	57.8
T10-1B	TraesCS4B02G198400	2066	0	2066	0	2066	10	46.4
T10-10A	TraesCS3A02G124100	2000	0	2000	384	2384	11	54.6
T10-12A	TraesCS5A02G174300	1882	0	1882	0	1882	11	53.4
T10-15B	TraesCS4B02G229600LC	853	0	853	1311	2164	21	87.8
T10-23A	TraesCS1A02G288700	974	0	974	0	974	6	35.6
T10-24A	TraesCS3A02G151300	1048	0	1048	77	1125	6	32.5
T10-25B	TraesCS4B02G096400	1492	0	1492	54	1546	15	63.8
T10-29B	TraesCS7B02G103700	1956	0	1956	331	2287	15	58.6
T10-29D	TraesCS7D02G199800	1039	0	1039	222	1261	10	44.2
T10-3A	TraesCS7A02G410300	1952	0	1952	336	2288	19	84.7
T10-3B	TraesCS7B02G309700	1950	0	1950	270	2220	19	79.8
T10-3D	TraesCS7D02G403500	1841	0	1841	300	2141	14	69.2
T10-31A	TraesCS5A02G352800	1686	0	1686	262	1948	16	68.8
T10-31D	TraesCS5D02G359800	1608	0	1608	0	1608	12	51.8
T10-35D	TraesCS4D02G381900LC	923	0	923	0	923	1	7.1
T10-37A	TraesCS1A02G428700	0	0	0	2206	2206	15	76.2
T10-38B	TraesCS5B02G303500	1465	0	1465	0	1465	9	47.1
T10-38D	TraesCS5D02G310000	1472	0	1472	0	1472	11	51.8
T10-4	TraesCS2A02G275100	0	0	0	1969	1969	22	94.8
T10-4	TraesCS2B02G292500	0	0	0	1881	1881	13	59.3
T10-4	TraesCS2D02G274100	0	0	0	1982	1982	13	70.8
T10-40D	TraesCS2D02G318100	794	0	794	0	794	1	7.1
T10-41A	TraesCS5A02G286500	1290	0	1290	87	1377	11	51.3
T10-41B	TraesCS5B02G285900	1303	0	1303	166	1469	CROSS-HYBRIDISNG BAITS F	
T10-43A	TraesCS3A02G198600	948	0	948	315	1263	7	31.8
T10-43B	TraesCS3B02G227200	1478	0	1478	445	1923	12	55.1
T10-43D	TraesCS3D02G196500	1923	0	1923	298	2221	6	25.9
T10-44B	TraesCS4B02G162200	0	0	0	2349	2349	15	78.2
T10-45A	TraesCS5A02G381700LC	887	0	887	136	1023	4	18.2
T10-45B	TraesCS5B02G256100	773	0	773	402	1175	8	37.1
T10-46A	TraesCS2A02G325700	2231	0	2231	283	2514	10	52.9
T10-48D	TraesCS5D02G172800	1943	0	1943	124	2067	8	43.3
T10-5A	TraesCS7A02G276600	1489	0	1489	229	1718	3	18
T10-5D	TraesCS7D02G276600	1624	0	1624	363	1987	9	50.4
T10-7D	TraesCS7D02G276600	1612	0	1612	354	1966	9	50.4
T10-9D	TraesCS1D02G159000	0	0	0	2108	2108	15	72.9
T6-11A	TraesCS4A02G376700	1594	0	1594	512	2106	18	76.5
T6-11D	TraesCS4D02G073700	2212	0	2212	0	2212	16	72.2
T6-15B	TraesCS6B02G044100	1778	0	1778	216	1994	12	68.2
T6-16B	TraesCS6B02G044000	2045	0	2045	198	2243	17	80.5
T6-18A	TraesCS6A02G030700	1990	0	1990	296	2286	24	95.8
T6-18B	TraesCS6B02G044500	1777	0	1777	397	2174	16	70.1
T6-18D	TraesCS6D02G035900	2681	0	2681	320	3001	18	86.4
T6-2A	TraesCS7A02G157700	2047	0	2047	351	2398	20	85.2
T6-31A(2)	TraesCS4A02G416300	2074	0	2074	317	2391	25	99.5
T6-7B	TraesCS5B02G183800	1695	0	1695	0	1695	12	61.2

trait gene	promoter	5'UTR	promoter + 5'UTR	Exons / Introns	total bp	MYbaits	TARGET coverage (%)
T1-26D	0	0	0	2218	2218	16	72.2
T2-27B	0	0	0	2160	2160	20	85.2
T2-29A	0	0	0	1921	1921	11	61.4
T2-43D	0	0	0	2013	2013	13	62.1
T2-57B	0	0	0	2314	2314	8	42.1
T4-1B	0	0	0	1731	1731	11	51.3
T4-1D	0	0	0	1180	1180	9	39.8
T4-2A	0	0	0	2316	2316	19	90.6
T4-2D	0	0	0	1748	1748	16	82.6
T4-30-4B	0	0	0	2411	2411	15	76.2
T4-6D	0	0	0	2624	2624	8	42.1
T4-7B	0	0	0	2370	2370	18	79.8
T8-14A	0	0	0	2545	2545	19	86.8
T9-22A	0	0	0	2330	2330	15	68.9
T10-37A	0	0	0	2206	2206	15	76.2
T10-4	0	0	0	1969	1969	22	94.8
T10-4	0	0	0	1881	1881	12	59.3
T10-4	0	0	0	1982	1982	13	70.8
T10-44B	0	0	0	2349	2349	15	78.2
T10-9D	0	0	0	2108	2108	15	72.9
T2-8B	0	21	21	744	765	4	18.4
T2-8A	0	26	26	753	779	4	21.6
T2-8D	0	122	122	844	966	4	18.4
T9-37A	0	194	194	1582	1776	15	72.9
T5-5A	250	0	250	1135	1385	9	50.6
T8-15A	250	80	330	2157	2487	23	50.5
T2-14D	306	70	376	253	629	1	7.1
T2-15B	308	70	378	108	486	10	43.1
T10-26D	0	393	393	2131	2524	16	78.8
T8-15D	152	292	444	2086	2530	26	60.4
T4-39B	405	83	488	2502	2990	19	49.2
T4-6A	520	0	520	1738	2258	12	71.5
T10-25D	448	80	528	288	816	2	10.8
T1-22B	435	94	529	83	612	2	10.8
T4-8B	394	135	529	2001	2530	25	53.7
T4-40B	407	128	535	249	784	4	20.9
T2-17D	544	0	544	270	814	2	14.1
T6-6B	492	63	555	351	906	9	43.1
T2-16D	465	109	574	161	735	1	7.1
T9-55D	533	44	577	197	774	1	7.1
T5-30A	601	0	601	152	753	3	15.1
T2-60A	315	290	605	1048	1653	2	10.8
T2-3A	310	298	608	280	888	2	14.1
T9-36A	609	0	609	293	902	6	29.2
T1-22A	520	90	610	219	829	3	14.6
T4-19-7A	546	72	618	859	1477	3	17.9
T4-21D	467	158	625	1438	2063	9	50.4
T4-19-4A	559	75	634	953	1587	4	28.2
T10-17B	523	115	638	217	855	6	25.3
T2-7A	510	129	639	507	1146	1	7.1
T10-28A	588	78	666	261	927	3	17.9
T9-49B	365	308	673	1286	1959	17	72.7
T2-22B	596	79	675	54	729	9	38.7
T4-59A	317	369	686	30	716	5	25.1
T9-16D	690	0	690	23	713	5	28.7
T1-10A	693	0	693	1687	2380	16	47.6
T9-29A	471	225	696	102	798	4	38
T9-32B	696	0	696	303	999	3	14.6
T10-9A	533	174	707	0	865	2	14.1
T6-8D	531	185	716	289	1005	3	17.9
T8-11A	443	285	728	223	951	3	17.8
T4-15-1D	734	0	734	278	1012	7	28.5
T10-7B	465	274	739	382	1121	3	17.9
T9-18A	740	0	740	0	740	7	32.9
T10-44D	602	151	753	0	753	5	28.7
T9-26B	679	91	770	282	1052	4	18.4
T9-59B	678	93	771	259	1030	5	28.7
T10-45B	773	0	773	402	1175	8	37.1
T9-25D	783	0	783	554	1337	5	25.4
T9-47D	793	0	793	0	793	2	10.8
T10-40D	794	0	794	0	794	1	7.1
T9-47A	705	96	801	0	891	2	10.8
T6-4D	667	155	822	1036	1858	12	61.6
T4-8D	796	28	824	0	824	3	17.9
T4-5B	527	300	827	0	827	1	7.1
T9-52D	458	374	832	0	832	1	7.1
T5-29B	834	0	834	33	867	4	24.9
T9-26A	758	82	840	185	1025	11	57.9
T4-13D	595	249	844	167	1011	6	25.9
T10-16B	816	29	845	301	1146	8	36.7
T1-2B	551	296	847	142	989	5	22.1
T2-39D	766	84	850	192	1042	6	29.2
T2-16A	765	87	852	0	852	2	10.8
T5-14D	605	248	853	115	968		
T10-15B	853	0	853	1311	2164	21	87.8
T2-15A	588	267	855	463	1318	6	29.2
T9-9D	389	471	860	1127	1987	14	79.1
T4-58A	0	861	861	240	1101	11	54.6
T9-70B	714	156	870	323	1193	9	37.2
T5-30B	711	169	880	309	1189	5	25.4
T5-16B	669	214	883	0	883	6	32.5
T10-45A	887	0	887	136	1023	1	7.1
T4-43D	786	105	891	444	1435	4	23.9
T8-6B	899	0	899	0	899	5	25.4
T4-5D	607	293	900	88	988	6	35.8
T9-74D	841	76	917	184	1101	8	34.2
T4-16D	922	0	922	234	1156	14	71.1
T10-35D	923	0	923	0	923	1	7.1
T5-20A	924	0	924	229	1153	8	38.8
T8-6A	925	0	925	0	925	6	25.9
T6-4A	723	205	928	1076	2004	6	29.2
T5-1B	700	239	939	155	1094	12	55.1
T2-58D	810	134	944	341	1285	2	10.8
T9-8D	773	171	944	213	1157	5	45.7
T9-37D	485	461	946	0	946	8	67.8
T10-43A	948	0	948	315	1263	7	31.8
T5-6B	954	0	954	569	1523	3	14.6
T8-17D	799	158	957	529	1486	2	14.1
T10-11D	857	101	958	304	1262	16	85.4
T1-10D	962	0	962	1499	2461	18	57.7
T8-13A	0	968	968	352	1320	6	34.6
T4-56B	579	393	972	0	972	4	24.9
T5-5B	778	196	974	963	1937	4	21.6
T10-23A	974	0	974	0	974	6	35.6
T5-20D	896	79	975	116	1091	8	39.9
T5-26D	871	105	976	247	1223	9	90.7
T4-60A	982	0	982	0	982	9	46.8
T1-5D	823	160	983	477	1460	2	14.1
T8-5A	892	99	991	0	991	6	60.1
T10-11B	921	72	993	137	1130	10	46.4
T9-33B	948	46	994	254	1248	9	45.9
T4-40A	917	78	995	184	1179	3	17.9
T2-49B	842	156	998	344	1342	6	35.8
T1-4(B)D	940	61	1001	114	1115	4	24.9
T5-30D	874	129	1003	169	1172	5	25.4
T4-16B	1013	0	1013	735	1748	15	72.9
T9-20A	647	367	1014	312	1326	9	43.3
T8-24A	0	1022	1022	1033	2055	19	81.4
T6-10A	862	164	1026	305	1331	9	44.1
T5-6D	1028	0	1028	367	1395	4	17.2
T4-55A	766	268	1034	175	1209	5	32
T10-29D	1039	0	1039	222	1261	10	44.2
T2-16B	998	42	1040	253	1293	7	32.9
T1-6-1B	276	768	1044	250	1294	16	68.3
T10-24A	1048	0	1048	77	1125	6	32.5
T4-4A	1050	0	1050	138	1188	17	76
T4-11A	839	218	1057	49	1106	4	21.6
T2-24D	799	264	1063	214	1277	7	42.8
T4-19-7D	992	71	1063	1017	2080	8	38.8
T2-44D	1065	0	1065	268	1333	11	43.5
T4-13B	893	172	1065	332	1397	7	29.6
T10-47B	544	524	1068	295	1363	3	17.9
T9-36B	848	225	1073	276	1349	8	46.6
T5-1A	928	151	1079	231	1310	10	46.7
T4-44B	1080	0	1080	210	1290	10	54.4
T5-27B	850	234	1084	322	1406	5	32
T10-38A	972	113	1085	155	1240	8	40
T5-14B	913	174	1087	232	1319	4	24.9
T10-7A	805	286	1091	321	1412	11	54.6
T8-23D	819	274	1093	0	1093	3	14.6
T4-12A	1104	0	1104	250	1354	13	52.2
T4-45B	1104	0	1104	68	1172	9	40.5
T1-15A	989	122	1111	168	1279	4	24.9
T9-28A	1117	0	1117	338	1455	8	40
T8-13D	100	1021	1121	67	1188	10	47.1
T9-52B	618	508	1126	0	1126	4	21.6
T9-38B	990	140	1130	190	1320	9	40.5
T9-56D	1130	0	1130	368	1498	2	10.8
T2-10A	782	349	1131		2337	8	45.4
T1-3A	814	318	1132	82	1214	6	35.7
T4-11B	898	238	1136	47	1183	5	25.4
T4-23A	983	153	1136	351	1487	5	28.7
T2-24A	852	291	1143	1021	2164	13	65.4
T10-18A	745	398	1143	116	1259	3	14.6
T5-3D	1041	127	1168	2320	3488	25	58
T10-47D	688	480	1168	190	1358	7	32.9
T5-14A	1009	166	1175	79	1254	5	32
T4-14B	1181	0	1181	0	1181	10	43.1
T4-32B(2)	1063	118	1181	218	1399	6	38.5
T9-33A	1186	0	1186	530	1716	4	21.6
T9-41D	766	422	1188	261	1449	7	42.8
T6-9A	1071	117	1188	1281	2469	15	46.9
T4-2B	1189	0	1189	983	2172	20	81.9
T1-6-7A	562	631	1193	291	1484	6	32.5
T8-9B	957	239	1196	0	1196	14	72.5
T9-22D	1047	150	1197	162	1359	9	40.5
T9-53A	1199	0	1199	416	1615	9	42.6
T5-16(B)-D	795	412	1207	0	1207	7	36.8
T9-23D	1213	0	1213	63	1276	14	59
T2-60B	926	291	1217	1141	2358	15	52.4
T4-13A	1054	163	1217	181	1398	8	43.3
T6-31A	1174	43	1217	316	1533	14	47.3
T1-4B	1173	56	1229	133	1362	4	24.9
T8-11B	1231	0	1231	226	1457	6	35.8
T10-36D	1045	186	1231	136	1367	10	44.2
T8-11D	871	361	1232	0	1232	2	14.1
T5-10-5A	849	389	1238	20	1258	8	43.3
T10-35B	1142	105	1247	148	1395	12	51.8
T10-14A	1110	143	1253	387	1640	12	51.8
T8-6D	1114	142	1256	0	1256	9	40.5
T8-5D	1146	113	1259	195	1454	9	47.1
T4-56A	488	775	1263	0	1263	9	47.1
T5-24A	1060	207	1267	1860	3127	28	65.3
T8-15B	1154	116	1270	193	1463	7	35.1
T4-11D	955	329	1284	122	1406	6	32.5
T10-41A	1290	0	1290	87	1377	11	51.3
T1-16A	1178	122	1300	1029	2329	22	67.8
T10-41B	1303	0	1303	166	1469	CROSS-HYBRIDISNG BAITS F	
T2-39B	1145	165	1310	151	1461	4	24.7
T4-24D	1215	96	1311	178	1489	4	17.8
T9-3B	1313	0	1313	0	1313	9	47.1
T9-11D	1166	154	1320	2401	3721	42	84.2
T5-25D	988	334	1322	206	1528	5	31.8
T2-7D	1325	0	1325	265	1590	8	36.7
T5-15D	1002	323	1325	169	1494	6	35.2
T1-5A	1328	0	1328	0	1328	10	47.5
T9-64D	327	1002	1329	303	1632	6	39.6
T5-1D	1330	0	1330	179	1509	11	56.7
T1-10B	1209	124	1333	296	1629	9	43.8
T6-9B	985	350	1335	1302	2637	3	17.9
T1-21A	1066	272	1338	0	1338	10	47.5
T1-28-3D	1287	56	1343	259	1602	7	39.5
T2-47A	1346	0	1346	0	1346	13	56.2
T8-8D	1088	258	1346	393	1739	7	39.5
T9-39D	1020	327	1347	230	1577	9	50.4
T1-20A	1016	336	1352	328	1680	9	43.1
T6-10D	1197	161	1358	333	1691	12	58.5
T4-54A	1251	108	1359	0	1359	10	65.7
T5-7B	857	504	1361	325	1686	5	32
T9-3A	1363	0	1363	26	1389	10	47.5
T2-59D	1241	128	1369	107	1476	2	10.8
T1-6-7D	665	714	1379	451	1830	9	43.8
T6-6A	1335	46	1381	300	1681	12	54
T1-25B	1325	60	1385	224	1609	5	28.8
T9-60B	1325	60	1385	38	1423	10	54.4
T2-54B	1371	16	1387	217	1604	17	70.6
T2-6B	1058	335	1393	170	1563	10	50.8
T5-24D	1184	210	1394	553	1947	11	53.4
T10-1A	1292	105	1397	321	1718	11	54.6
T9-61D	1249	149	1398	120	1518	11	45.3
T10-42A	653	747	1400	374	1774	14	60.9
T9-76A	1261	145	1406	496	1902	7	38.4
T4-24B	1295	115	1410	202	1612	10	53.6
T9-4A	1310	108	1418	306	1724	11	50.1
T9-63D	1283	135	1418	114	1532	12	61.6
T5-3A	1291	131	1422	2383	3805	29	66.1
T9-2D	946	482	1428	444	1872	6	42.4
T8-24D	1432	0	1432	1039	2471	17	79.3
T8-21B	1434	0	1434	0	1434	13	59.8
T1-15B	988	449	1437	117	1554	3	21.2
T2-52B	1440	0	1440	0	1440	6	35.9
T9-4D	1403	38	1441	298	1739	7	38.4
T2-19B	1268	174	1442	471	1913	7	36.2
T4-53A	1227	218	1445	130	1575	6	29
T2-6D	642	808	1450	206	1656	6	32.5
T4-4D	1450	0	1450	219	1669	22	89.4
T9-42B	1334	117	1451	220	1671	8	43.3
T1-11D	1453	0	1453	0	1453	12	52.4
T9-13A	1108	349	1457	977	2434	14	75.8
T10-21D	1369	91	1460	280	1740	11	56.4
T10-38B	1465	0	1465	0	1465	9	47.1
T8-26D	1356	111	1467	348	1815	12	58.4
T9-77A	1471	296	1467	265	1732	18	77.6
T10-48A	457	1010	1467		2512	10	57.4
T2-37A	297	1173	1470	476	1946	11	57.9
T10-38D	1472	0	1472	0	1472	11	51.8
T4-8A	1323	152	1475	97	1572	11	54.6
T4-17A	1477	0	1477	1818	3295	18	50.4
T10-43B	1478	0	1478	445	1923	12	55.1
T8-8A	1164	323	1487	309	1796	8	38.8
T10-5A	1489	0	1489	229	1718	3	18
T2-61B	763	728	1491	255	1746	9	43.8
T5-8B	1241	250	1491	0	1491	12	58.9
T4-35D	1246	246	1492	0	1492	13	65.4
T9-2A	1051	441	1492	173	1665	6	32.3
T10-25B	1492	0	1492	54	1546	15	63.8
T9-66A	1316	179	1495	125	1620	4	21.6
T2-56B	1276	220	1496	0	1496	9	54.2
T1-11A	1497	0	1497	0	1497	13	58.2
T1-20B	1019	478	1497	405	1902	6	35
T9-37B	1177	329	1506	0	1506	10	50.8
T1-25A	1515	0	1515	158	1673	9	53.6
T9-16B	1414	112	1526	34	1560	11	51.3
T10-47A	1267	261	1528	0	1528	11	54.2
T4-57B	1530	0	1530	175	1705	16	70.1
T9-42D	1345	186	1531	421	1952	12	56.9
T2-10D	540	994	1534	1392	2926	18	56
T1-2D	1415	121	1536	343	1879	2	14.1
T1-17A	1107	434	1541	301	1842	6	35.8
T10-36A	1315	226	1541	109	1650	7	42.8
T2-10B	610	933	1543	0	1543	14	65.9
T9-19A	161	1385	1546	322	1868	14	78.6
T5-20B	1391	158	1549	245	1794	15	78.4
T1-22D	1449	102	1551	21	1572	13	65.4
T2-9A	1556	0	1556	133	1689	12	61.6
T1-25D	1557	0	1557	341	1898	8	40
T9-21D	1195	367	1562	0	1562	11	54.6
T1-20D	1243	321	1564	425	1989	9	49.2
T2-42D	1567	0	1567	213	1780	16	65.6
T9-61A	1567	0	1567	0	1567	12	54
T4-9D	1454	116	1570	571	2141	10	60.7
T5-25A	1307	263	1570	149	1719	8	43.3
T1-3D	1239	333	1572	79	1651	7	35.9
T9-55B	1573	0	1573	0	1573	12	53.9
T9-58A	1478	98	1576	0	1576	13	68.7
T4-10D	1336	245	1581	206	1787	12	58.4
T10-20B	1377	204	1581	0	1581	13	58.8
T2-34B	1583	0	1583	251	1834	17	70.6
T9-35D	1506	81	1587	270	1857	11	61.2
T10-30A	1567	20	1587	56	1643	6	32.5
T4-44D	1589	0	1589	232	1821	15	69.6
T2-14A	1516	74	1590	205	1795	15	69.6
T6-11A	1594	0	1594	512	2106	18	76.5
T4-36A	1394	202	1596	0	1596	16	80.3
T2-39A	1597	0	1597	274	1871	13	58.8
T8-2B	1399	198	1597	0	1597	10	50.8
T2-45B	1313	285	1598	218	1816	14	65.9
T4-21A	1100	498	1598	283	1881	14	69.2
T1-24B	1005	596	1601	492	2093	8	49.9
T4-40D	1480	121	1601	234	1835	10	50.1
T10-2A	1120	482	1602	175	1777	10	47.5
T5-12B	558	1046	1604	311	1915	15	68.6
T9-39A	1336	269	1605	232	1837	11	61.2
T1-21D	1403	204	1607	0	1607	8	43.3
T8-7A	1000	608	1608	210	1818	14	79.6
T10-31D	1608	0	1608	0	1608	12	51.8
T1-3B	1290	321	1611	0	1611	7	41.1
T2-32B	1611	0	1611	492	2103	17	71.4
T10-7D	1612	0	1612	354	1966	9	50.4
T4-38D	1486	127	1613	0	1613	9	47.1
T9-52A	1190	429	1619	122	1741	4	21.6
T6-13B	1374	245	1619	99	1718	11	67.8
T10-5D	1624	0	1624	363	1987	9	50.4
T4-54B	1627	0	1627	0	1627	14	69.2
T9-25A	1627	0	1627	164	1791	8	40
T9-15D	1370	261	1631	806	2437	9	48.8
T5-10-4B	936	697	1633	272	1905	14	67.5
T8-10D	1501	132	1633	2070	3703	28	60.4
T9-8A	1233	401	1634	290	1924	13	62.1
T2-48D	1030	605	1635	528	2163	18	73.2
T9-30B	1464	176	1640	836	2476	23	66.5
T6-17A	1548	93	1641	276	1917	12	61.6
T4-29D	1563	79	1642	77	1719	14	61.1
T8-7D	1065	578	1643	270	1913	15	72.9
T4-33-7D	1236	411	1647	231	1878	14	65.9
T1-5B	1649	0	1649	227	1876	14	65.9
T2-54A	0	1652	1652	458	2110	21	81.2
T9-40B	1654	0	1654	0	1654	10	54.1
T10-16D	1586	69	1655	187	1842	6	29.2
T8-17B	1657	0	1657	619	2276	12	57.4
T9-57A	1554	105	1659	58	1717	7	39.2
T1-23B	1318	344	1662	199	1861	16	74.6
T9-21B	1304	359	1663	0	1663	11	51.3
T2-6A	1323	341	1664	111	1775	10	52.9
T1-16D	1509	156	1665	1099	2764	17	49.7
T2-17B	1606	60	1666	0	1666	15	66.4
T2-18B	1547	120	1667	47	1714	12	64.9
T2-28D	1667	0	1667	273	1940	11	65.3
T4-41-5A	1667	0	1667	0	1667	12	61.6
T9-69B	1332	337	1669	0	1669	13	58.8
T4-55B	1670	0	1670	285	1955	12	61.2
T9-35A	1586	84	1670	245	1915	10	57.4
T9-21A	1676	0	1676	0	1676	12	55.1
T2-58A	1682	0	1682	0	1682	6	32.5
T9-31D	1682	0	1682	299	1981	10	47.5
T8-9A	1486	198	1684	145	1829	11	51.3
T10-31A	1686	0	1686	262	1948	16	68.8
T6-10B	1481	209	1690	333	2023	16	76.8
T6-7B	1695	0	1695	0	1695	12	61.2
T4-17B	1494	202	1696	1804	3500	22	55.7
T2-19D	1516	182	1698	463	2161	11	51.3
T9-43B	1667	33	1700	365	2065	25	96.2
T9-43D	1613	87	1700	197	1897	18	74.4
T6-14B	1571	131	1702	44	1746	19	81.4
T2-4B	1313	391	1704	1047	2751	19	69.6
T4-22A	1521	183	1704	29	1733	14	65.9
T4-34D	1662	44	1706	212	1918	21	91.1
T2-5D	1446	262	1708	172	1880	9	37.2
T4-46B	1577	131	1708	723	2431	14	68.6
T8-23B	1432	279	1711	0	1711	12	51.8
T10-26B	641	1070	1711	2169	2169	16	80.1
T9-18B	1683	31	1714	0	1714	17	89.9
T9-77B	1715	0	1715	0	1715	17	70.6
T8-22A	1450	269	1719	0	1719	11	56.7
T5-28D	1556	165	1721	159	1880	8	49.9
T9-20D	1105	617	1722	0	1722	7	42.8
T9-28B	1723	0	1723	374	2097	14	69.2
T8-8B	1493	232	1725	297	2022	9	42.6
T2-50A	1543	184	1727	142	1869	6	39.9
T10-21A	1538	190	1728	163	1891	18	83.1
T9-70A	1678	151	1729	145	1874	14	72.5
T2-22A	1730	0	1730	3016	4746	45	85.3
T4-39A	1647	86	1733	2439	4172	27	70.6
T9-48D	444	1291	1735	288	2023	17	79.3
T4-5A	1419	320	1739	161	1900	6	32.5
T2-43A	1596	144	1740	0	1740	12	55.1
T6-8A	1613	129	1742	313	2055	11	56.7
T5-3B	1609	134	1743	135	1878	11	61.2
T2-45D	1745	0	1745	0	1745	10	59.5
T2-2B	1704	42	1746	387	2133	14	59.3
T5-12D	583	1163	1746	371	2117	18	76.5
T10-34A	1650	97	1747	246	1993	20	87.8
T2-34D	1749	0	1749	426	2175	19	81.4
T4-56D	1428	322	1750	0	1750	16	65.6
T9-51B	1623	128	1751	0	1751	11	53.4
T9-64A	1756	0	1756	0	1756	11	50.1
T10-9B	1522	236	1758	0	1758	12	68.2
T4-46D	1677	83	1760	792	2552	16	76.7
T8-16B	1579	181	1760	240	2000	9	43.8
T1-9A	1637	124	1761	0	1761	12	61.6
T1-2A	1605	157	1762	274	2036	8	42.1
T4-16A	1329	433	1762	262	2024	6	36
T10-21B	1695	67	1762	142	1904	12	58.4
T9-44D	1461	303	1764	1750	3514	27	65.6
T4-10B	1661	104	1765	319	2084	14	65.2
T4-24A	1705	60	1765	235	2000	10	49.6
T1-15D	1345	421	1766	62	1828	12	64.9
T2-29B	1702	65	1767	157	1924	8	39.1
T9-28D	1767	0	1767	323	2090	17	77.2
T9-15A	1366	403	1769	311	2080	15	69.6
T4-32A	1675	95	1770	267	2037	7	39.5
T4-42B	1686	84	1770	370	2140	12	64.9
T6-14A	1636	138	1774	270	2044	19	81.4
T1-28-3A	1726	49	1775	344	2119	14	68
T4-23B	1609	166	1775	2337	4112	27	72.2
T4-28-5B	1735	42	1777	273	2050	22	89.4
T6-18B	1777	0	1777	397	2174	16	70.1
T6-15B	1778	0	1778	216	1994	12	68.2
T1-18B	1779	0	1779	311	2090	13	60.9
T4-25D	1641	140	1781	175	1956	18	74.4
T1-28-2B	1726	56	1782	306	2088	16	66.8
T1-23A	1516	267	1783	161	1944	16	74
T9-49D	1461	322	1783	258	2041	32	76.4
T2-53-7A	944	842	1786	230	2016	15	69.6
T4-25B	1618	169	1787	159	1946	13	57.6
T4-22B	1788	0	1788	0	1788	12	61.6
T10-14B	1700	88	1788	479	2267	5	28.7
T2-32D	1789	0	1789	434	2223	12	55.9
T2-37B	531	1258	1789	1201	2990	23	72.2
T2-40A	1790	0	1790	318	2108	16	83.3
T4-59D	1606	184	1790	155	1945	8	42.1
T2-54D	0	1791	1791	802	2593	26	85.4
T4-33-4A	1748	48	1796	310	2106	20	77.4
T4-38A	1761	35	1796	144	1940	17	73.9
T9-1A	943	853	1796	248	2044	15	72.9
T6-12D	1360	438	1798	262	2060	15	71.8
T9-7B	1394	406	1800	1153	2953	29	67.7
T4-31D	1214	587	1801	235	2036	12	64.2
T8-25A	1716	85	1801	215	2016	19	81.4
T5-23B	1623	179	1802	0	1802	11	49.3
T10-32D	0	1802	1802	965	2767	18	87.4
T8-2D	1734	69	1803	0	1803	14	75.8
T4-23D	1639	165	1804	2302	4106	6	35.8
T4-58B	1463	341	1804	1697	3501	27	67.5
T9-74A	1712	92	1804	241	2045	13	62.1
T8-4D	1545	260	1805	171	1976	14	72.5
T4-22D	1806	0	1806	0	1806	6	32.3
T10-6D	1651	155	1806	0	1806	12	64.9
T9-59A	1694	113	1807	211	2018	15	69.6
T8-24B	1680	128	1808	840	2648	19	50.7
T9-42A	1682	126	1808	173	1981	14	65.9
T6-13D	1777	31	1808	289	2097	15	76.2
T2-59A	1700	110	1810	0	1810	9	47.1
T9-14A	1810	0	1810	272	2082	12	58.4
T1-1B	1645	167	1812	342	2154	11	54.6
T4-7D	1552	261	1813	218	2031	11	61.2
T2-37D	522	1292	1814	209	2023	15	76.2
T9-9B	1372	442	1814	101	1915	12	61.6
T9-65B	1727	88	1815	85	1900	20	88.5
T10-5B	1537	278	1815	185	2000	6	35.8
T2-33A	959	857	1816	323	2139	15	73.7
T9-26D	1723	93	1816	120	1936	14	69.2
T10-26A	712	1105	1817	190	2007	15	69.5
T5-13B	555	1263	1818	243	2061	16	78.8
T1-7A	1560	259	1819	323	2142	20	84
T9-16A	1819	0	1819	162	1981	15	69.6
T4-57A	1200	622	1822	180	2002	17	77.2
T5-15A	1511	312	1823	99	1922	16	73.4
T9-50D	1586	237	1823	197	2020	14	69.2
T9-68A	1724	100	1824	0	1824	15	76.2
T4-39D	1745	82	1827	294	2121	12	68.2
T2-12D	1721	107	1828	0	1828	17	72.7
T4-51D	1731	101	1832	297	2129	12	55.1
T4-14A	1724	110	1834	111	1945	14	65.9
T9-27A	0	1834	1834	315	2149	17	82.6
T5-9D	1728	107	1835	6	1841	15	72.9
T1-27A	1606	231	1837	389	2226	12	67.1
T2-56D	1561	277	1838	0	1838	15	71.8
T9-38A	1710	129	1839	196	2035	11	53.4
T4-3D	1700	140	1840	290	2130	16	73.4
T10-3D	1841	0	1841	300	2141	14	69.2
T10-42B	981	860	1841	422	2263	19	86.8
T10-48B	484	1357	1841	1612	3453	18	51.6
T10-31B	1249	594	1843	277	2120	10	43.1
T4-51A	1716	128	1844	326	2170	13	63.2
T9-25B	1782	62	1844	272	2116	11	61.2
T9-17A	1119	726	1845	356	2201	13	60.9
T10-14D	942	904	1846	373	2219	16	68.2
T4-14D	1738	109	1847	90	1937	12	58.4
T9-58B	1734	113	1847	50	1897	12	61.6
T9-11B	1727	121	1848	2366	4214	32	71.2
T1-11B	1552	297	1849	140	1989	11	54.2
T2-7B	1707	142	1849	450	2299	11	54.6
T2-24B	1546	305	1851	168	2019	10	54.1
T10-40B	1382	469	1851	281	2132	7	42.8
T5-29D	1647	205	1852	63	1915	12	64.9
T9-17D	1290	562	1852	288	2140	8	40
T6-20	628	1225	1853	391	2244	19	81.4
T2-20A	1844	10	1854	420	2274	21	95.5
T2-25D	1854	0	1854	361	2215	25	99.5
T5-7D	1854	0	1854	271	2125	9	39.3
T2-43B	1553	302	1855	0	1855	14	62.6
T4-52A	1855	0	1855	0	1855	16	73.4
T9-56A	1478	377	1855	8	1863	5	28.7
T2-26B	1857	0	1857	351	2208	23	92.4
T10-46B	1768	90	1858	370	2228	8	43.3
T2-1B	1604	255	1859	122	1981	17	80.6
T2-13B	1760	99	1859	175	2034	23	89.9
T9-23B	1744	115	1859	179	2038	16	70.1
T9-34B	1777	82	1859	208	2067	17	80.6
T9-46B	1859	0	1859	0	1859	15	61.9
T10-42D	897	963	1860	298	2158	17	80.5
T4-46A	1815	46	1861	368	2229	17	82.6
T2-1A	1585	277	1862	92	1954	18	74.4
T9-46D	1864	0	1864	61	1925	10	54.1
T10-20A	1677	188	1865	50	1915	17	70.6
T2-4D	1547	321	1868	1085	2953	24	80.7
T4-20-7A	1869	0	1869	270	2139	18	79.8
T5-13A	636	1233	1869	384	2253	12	55.1
T10-12B	1746	123	1869	180	2049	18	74.4
T1-7D	1730	140	1870	263	2133	17	80.5
T2-4A	1871	0	1871	1276	3147	24	78.2
T9-75D	1871	0	1871	0	1871	8	45.4
T10-6B	1775	98	1873	54	1927	4	24.9
T2-50B	1508	366	1874	66	1940	10	57.4
T9-48A	798	1076	1874	57	1931	17	76
T4-26-5A	1450	427	1877	112	1989	13	68.7
T9-57B	1773	105	1878	30	1908	15	71.8
T2-2D	1410	469	1879	432	2311	21	88.9
T9-34A	1656	224	1880	58	1938	18	79.8
T2-5B	1753	128	1881	299	2180	15	69.6
T8-25B	1881	0	1881	351	2232	16	73.4
T10-12A	1882	0	1882	0	1882	11	53.4
T4-29B	1813	70	1883	218	2101	20	87.3
T9-45B	1582	301	1883	787	2670	22	94.8
T5-45A	1573	312	1885	0	1885	21	88.9
T8-14B	1669	216	1885	0	1885	16	65.9
T8-4A	1679	206	1885	242	2127	14	65.9
T10-34B	1796	89	1885	196	2081	21	85.6
T1-1D	1693	193	1886	210	2096	12	55.4
T9-67A	1424	462	1886	629	2515	14	75.8
T1-8-7D	1541	346	1887	0	1887	17	72.9
T5-13D	596	1292	1888	326	2214	13	62.1
T4-3B	1790	100	1890	102	1992	15	76.2
T4-35A	1562	330	1892	0	1892	14	69.2
T2-31D	1856	37	1893	139	2032	8	43.3
T4-53B	1643	250	1893	110	2003	11	57.9
T9-69D	1531	362	1893	236	2129	14	73.6
T9-46A	1894	0	1894	158	2052	15	66.4
T4-37A	1825	70	1895	367	2262	15	75.1
T2-31A	1812	84	1896	117	2013	14	76.3
T4-49B	1896	0	1896	230	2126	16	69.1
T5-12A	836	1060	1896	229	2125	18	73.2
T10-1D	1796	100	1896	116	2012	17	77.2
T4-42D	1808	89	1897	194	2091	12	68.2
T9-27D	0	1897	1897	267	2164	17	76
T2-3B	1558	341	1899	318	2217	15	68.5
T4-48	1900	0	1900	111	2011	17	77.2
T5-22B	1900	0	1900	401	2301	20	93.9
T9-3D	1900	0	1900	208	2108	21	87.8
T2-36B	1901	0	1901	239	2140	17	76
T5-22D	1901	0	1901	290	2191	14	69.2
T8-7B	1292	610	1902	188	2090	13	70.8
T9-6A	1684	218	1902	34	1936	8	46.6
T1-19D	1721	182	1903	309	2212	14	67.9
T9-12B	1904	0	1904	202	2106	18	79.8
T4-48D	1309	596	1905	287	2192	19	86.8
T9-11A	1791	115	1906	2444	4350	35	83.8
T9-77D	1907	0	1907	0	1907	12	57.8
T4-45D	1909	0	1909	0	1909	20	85.2
T9-61B	1818	92	1910	227	2137	8	40
T1-14D	1604	307	1911	0	1911	16	70.1
T1-7B	1732	179	1911	305	2216	14	72.5
T5-23D	1915	0	1915	0	1915	18	80.9
T8-10B	1824	91	1915	0	1915	15	76.7
T2-18A	1704	212	1916	183	2099	9	43.8
T4-41-4B	1916	0	1916	0	1916	7	46.1
T1-9D	1364	553	1917	0	1917	12	64.9
T4-7A	1736	182	1918	152	2070	11	51.3
T5-16(B)-A	1918	0	1918	0	1918	10	60.7
T10-13A	1888	30	1918	308	2226	12	57.2
T4-9A	1919	0	1919	327	2246	19	86.8
T8-19D	869	1050	1919	169	2088	18	80.9
T10-41D	1884	35	1919	338	2257	13	65.4
T10-39D	1733	187	1920	1045	2965	20	66.6
T4-44A	1921	0	1921	0	1921	19	88
T5-23A	1788	133	1921	0	1921	17	77.2
T2-29D	1859	63	1922	265	2187	6	39.1
T10-25A	1889	33	1922	471	2393	19	84.1
T10-43D	1923	0	1923	298	2221	6	25.9
T4-20-7D	1734	190	1924	280	2204	17	82.6
T6-9D	1718	207	1925	0	1925	4	21.6
T1-26A	1546	381	1927	0	1927	14	69.2
T2-3D	1628	299	1927	213	2140	10	47.5
T4-29A	1853	74	1927	56	1983	18	77.6
T8-1B	1781	146	1927	216	2143	16	73.4
T10-32B	1156	771	1927	169	2096	19	81.4
T4-48A	1878	50	1928	314	2242	16	76.7
T9-7D	1878	51	1929	129	2058	17	83.8
T4-12D	1930	0	1930	197	2127	22	91.5
T2-48B	1376	556	1932	376	2308	21	91.1
T9-15B	1932	0	1932	751	2683	10	60.7
T9-76B	1798	134	1932	338	2270	17	79.3
T8-27A	1649	284	1933	430	2363	15	69.2
T4-34A	1934	0	1934	260	2194	17	66.1
T9-53B	1441	494	1935	278	2213	15	78.4
T9-30D	1936	0	1936	165	2101	18	77.6
T9-48B	1387	549	1936	211	2147	18	86.4
T5-19B	1938	0	1938	81	2019	16	76.7
T1-17B	1546	393	1939	1802	3741	22	54.8
T4-18-7A	0	1939	1939	99	2038	24	92.5
T4-17D	1838	102	1940	145	2085	22	89.4
T10-2D	1911	29	1940	282	2222	13	72
T2-20B	1900	42	1942	231	2173	17	83.8
T2-52D	1596	346	1942	576	2518	11	60.1
T9-66D	1638	305	1943	175	2118	19	80.2
T10-48D	1943	0	1943	124	2067	8	43.3
T2-48B	1388	556	1944	352	2296	20	87.3
T9-73A	1944	0	1944	193	2137	14	79.8
T8-25D	1871	74	1945	226	2171	22	92.7
T2-14B	1792	155	1947	280	2227	16	70.1
T8-12B	1883	64	1947	232	2179	17	73.9
T2-27A	1852	97	1949	46	1995	19	83.5
T5-25B	1614	335	1949	141	2090	16	78.8
T10-3B	1950	0	1950	270	2220	19	79.8
T8-14D	1508	443	1951	139	2090	17	80.5
T10-3A	1952	0	1952	336	2288	19	84.7
T4-31A	1781	172	1953	449	2402	14	69.2
T9-1D	1208	745	1953	223	2176	20	84
T9-67B	1583	370	1953	779	2732	17	75.4
T5-27D	1621	333	1954	235	2189	18	83.1
T9-67D	1605	349	1954	578	2532	18	68.8
T9-32D	1743	212	1955	338	2293	18	80.9
T10-8D	1411	544	1955	0	1955	17	80.5
T10-29B	1956	0	1956	331	2287	15	58.6
T1-27B	1347	611	1958	378	2336	10	57.4
T2-58B	1958	0	1958	0	1958	21	81.2
T2-59B	1959	0	1959	183	2142	8	31.3
T1-9B	1721	239	1960	0	1960	12	64.9
T5-28A	1776	184	1960	22	1982	16	80
T8-2A	1815	145	1960	0	1960	12	58.4
T9-34D	1737	223	1960	0	1960	13	67.5
T2-57D	1813	148	1961	231	2192	12	64.9
T9-20B	1961	0	1961	0	1961	16	84.2
T9-24A	1923	38	1961	271	2232	23	93.2
T6-14D	1616	345	1961	20	1981	18	84.2
T8-10A	1561	402	1963	2577	4540	35	75.2
T1-17D	1566	398	1964	1811	3775	24	62.6
T4-9B	1965	0	1965	91	2056	15	76.2
T6-15A	1745	221	1966	224	2190	19	81.4
T1-26B	1508	459	1967	0	1967	13	68.7
T2-53-4A	1239	728	1967	275	2242	21	91.1
T8-9D	1765	202	1967	169	2136	12	69.6
T1-23D	1710	258	1968	23	1991	11	50.1
T4-57D	1315	653	1968	308	2276	20	84
T2-19A	1757	212	1969	441	2410	17	47.5
T2-52A	0	1970	1970	318	2288	21	88.9
T4-26-4D	1545	425	1970	195	2165	13	67.5
T5-16(A)-B	1834	136	1970	421	2391	15	76.2
T1-16B	1851	120	1971	0	1971	11	48
T5-8A	1817	155	1972	264	2236	18	84.2
T1-12A	1779	194	1973	385	2358	10	47.5
T2-13A	1868	105	1973	396	2369	19	84.7
T10-12B	1872	101	1973	0	1973	11	53.4
T6-4B	1717	256	1973	57	2030	13	60.9
T2-3D	1965	9	1974	0	1974	9	47.5
T2-25B	1967	8	1975	367	2342	23	99.1
T2-33D	1133	842	1975	240	2215	11	63.8
T5-2B	1975	0	1975	0	1975	12	57.6
T8-1A	1833	142	1975	235	2210	21	91.1
T8-22B	1858	117	1975	201	2176	15	65.8
T4-51B	1880	96	1976	295	2271	20	87.3
T9-31B	1682	294	1976	117	2093	19	86.8
T4-15B	1854	124	1978	407	2385	9	40
T2-2A	1514	466	1980	356	2336	18	80.9
T2-23B	1351	629	1980	311	2291	17	57.9
T2-1D	1727	254	1981	48	2029	17	70.6
T4-25A	1829	152	1981	165	2146	18	77.1
T8-3B	1974	7	1981	85	2066	17	77
T9-24B	1983	0	1983	297	2280	11	61.2
T2-55B	1876	108	1984	0	1984	21	88.9
T10-13B	1953	31	1984	448	2432	14	64.7
T4-1A	1986	0	1986	79	2065	18	74.4
T4-12B	1987	0	1987	401	2388	18	77.6
T9-58D	1845	142	1987	24	2011	10	60.7
T6-16D	1915	72	1987	188	2175	16	80
T10-40A	1633	354	1989	0	1989	8	43.3
T9-10A	1336	654	1990	304	2294	20	94.4
T6-18A	1990	0	1990	296	2286	24	95.8
T9-1B	943	1049	1992	299	2291	12	64.9
T9-19D	0	1993	1993	183	2176	11	64.5
T9-63A	1844	149	1993	312	2305	15	78.2
T9-69A	1723	371	1994	208	2202	13	65.4
T1-1A	1811	184	1995	266	2261	14	54.6
T8-18D	1769	226	1995	167	2162	6	32.5
T10-13D	1964	31	1995	476	2471	11	53.4
T2-22D	1831	166	1997	1038	3035	21	70.7
T2-61A	857	1142	1999	276	2275	12	57.2
T9-54B	1969	30	1999	424	2423	13	65.4
T9-75B	1999	0	1999	336	2335	11	61.2
T1-4A	1665	335	2000	281	2281	19	83.5
T10-10A	2000	0	2000	384	2384	11	54.6
T6-7A	1860	140	2000	158	2158	19	84.7
T8-1D	1872	131	2003	340	2343	21	87.8
T5-11D	2004	0	2004	152	2156	12	60.5
T9-44B	1762	242	2004	1389	3393	24	57.5
T6-8B	1872	134	2006	290	2296	22	86.1
T9-54A	1940	68	2008	276	2284	15	65.2
T2-13D	1831	178	2009	231	2240	22	93.6
T10-46D	1892	117	2009	369	2378	20	88.5
T4-49A	1913	98	2011	261	2272	21	85.6
T4-20-4A	1885	127	2012	253	2265	14	69.2
T8-3D	1983	29	2012	182	2194	14	69.2
T2-26D	2013	0	2013	482	2495	26	100
T4-43A	1897	116	2013	0	2013	10	50.4
T5-29A	1878	135	2013	239	2252	12	64.9
T9-23A	2013	0	2013	0	2013	19	76.9
T8-19A	1169	845	2014	219	2233	14	75.8
T4-37D	1968	47	2015	452	2467	16	70.1
T1-8-7A	1687	329	2016	0	2016	16	76.7
T8-18B	1740	276	2016	0	2016	5	24.2
T9-54D	1985	35	2020	140	2160	13	60.9
T1-12D	1714	307	2021	463	2484	11	54.6
T2-60A	1923	98	2021	160	2181	2	10.8
T9-36D	1918	103	2021	288	2309	11	64.5
T4-48B	2022	0	2022	0	2022	16	76.7
T9-49A	1768	258	2026	1330	3356	16	70.1
T1-24A	1500	527	2027	286	2313	14	74.6
T8-19B	1196	834	2030	192	2222	13	68.7
T10-6A	1925	105	2030	19	2049	8	46.6
T2-27D	1892	141	2033	92	2125	20	80.7
T4-42A	2033	0	2033	433	2466	15	69.6
T8-27D	1901	132	2033	314	2347	13	58.4
T6-13A	1830	203	2033	102	2135	7	42.6
T9-76D	1905	129	2034	299	2333	17	79.3
T5-8D	1891	144	2035	268	2303	18	86.8
T9-5A	1902	133	2035	228	2263	15	72.9
T9-32A	1822	214	2036	345	2381	13	71.3
T2-60D	1750	287	2037	0	2037	17	72.7
T10-22D	1999	39	2038	0	2038	22	89.4
T4-18-7D	1977	63	2040	0	2040	15	78.4
T2-35B	1939	103	2042	133	2175	19	81.4
T9-29D	1813	231	2044	361	2405	20	85.2
T9-56B	1662	382	2044	317	2361	9	40.5
T6-16B	2045	0	2045	198	2243	17	80.5
T2-55A	1905	141	2046	0	2046	17	76
T9-45D	2046	0	2046	0	2046	20	90.6
T9-12D	2047	0	2047	0	2047	17	72.7
T9-41B	1673	374	2047	172	2219	15	75.1
T6-2A	2047	0	2047	351	2398	20	85.2
T5-28B	1870	178	2048	305	2353	7	36.2
T9-33D	2048	0	2048	349	2397	4	20.9
T9-43A	1878	170	2048	218	2266	19	81.4
T5-19A	2049	0	2049	296	2345	24	95.8
T5-2A	1857	192	2049	0	2049	17	72.7
T5-16A	1940	112	2052	164	2216	15	76.2
T8-22D	1936	116	2052	1066	3118	18	54.8
T4-55D	2054	0	2054	340	2394	18	83.1
T2-12A	1750	306	2056	51	2107	11	53.4
T2-49A	1938	118	2056	229	2285	5	28.7
T1-13B	1694	363	2057	0	2057	13	57.6
T2-35A	1931	131	2062	113	2175	18	80.9
T9-31A	1732	330	2062	53	2115	21	88.9
T9-72B	2001	61	2062	268	2330	18	77.6
T5-15B	1717	346	2063	171	2234	15	72.9
T9-18D	1911	152	2063	285	2348	12	61.6
T2-21D	1877	189	2066	0	2066	7	39.5
T10-1B	2066	0	2066	0	2066	10	46.4
T10-37D	947	1119	2066	0	2066	12	58.4
T2-56A	1795	272	2067	1111	3178	24	71.4
T5-27A	1839	229	2068	294	2362	18	83.1
T5-18D	2069	0	2069	0	2069	11	60.5
T9-51D	1642	427	2069	0	2069	14	61.4
T6-31B	2019	50	2069	221	2290	21	92.2
T6-6D	1986	83	2069	258	2327	14	61.4
T1-8-4A	1728	342	2070	129	2199	17	76
T5-2D	2071	0	2071	0	2071	11	60
T5-4D	2071	0	2071	0	2071	12	57.6
T9-66B	1744	327	2071	171	2242	11	53.4
T10-20D	1869	202	2071	37	2108	18	76.5
T2-30D	2074	0	2074	268	2342	16	75.5
T4-50A	2074	0	2074	0	2074	16	83.3
T6-31A(2)	2074	0	2074	317	2391	25	99.5
T2-57A	1870	205	2075	394	2469	12	64.9
T2-31B	2051	26	2077	119	2196	13	67.5
T4-30-5A	1982	95	2077	2459	4536	33	75.3
T9-59D	1954	123	2077	229	2306	4	20.5
T9-60D	1954	123	2077	229	2306	13	67.5
T10-8B	1535	542	2077	297	2374	18	83.1
T2-23D	1425	656	2081	94	2175	10	52.9
T8-26A	1984	97	2081	305	2386	15	71.8
T2-20D	2082	0	2082	80	2162	19	86.8
T10-18D	1662	420	2082	0	2082	12	68.5
T10-44A	1877	205	2082	0	2082	13	60.9
T9-53D	1601	482	2083	408	2491	14	71.3
T2-46B	2086	0	2086	100	2186	19	88
T9-27B	0	2086	2086	312	2398	21	94.8
T9-24D	2038	49	2087	218	2305	26	100
T9-65D	2005	83	2088	100	2188	21	82.4
T4-26-4B	1868	221	2089	166	2255	12	67.1
T10-37B	983	1106	2089	0	2089	12	57.2
T1-18D	1978	116	2094	162	2256	11	53.4
T2-55D	2022	72	2094	0	2094	22	88.2
T4-53D	1888	206	2094	96	2190	13	72
T2-11A	0	2096	2096	312	2408	8	49.9
T4-6B	1862	234	2096	101	2197	10	57.4
T1-14B	1785	315	2100	0	2100	15	68.5
T5-5D	1695	405	2100	81	2181	19	76.9
T10-10B	1877	225	2102	163	2265	10	46.4
T9-70D	1951	152	2103	97	2200	11	51.3
T9-64B	1415	689	2104	384	2488	9	56.2
T4-18-4A	1869	236	2105	75	2180	18	84.2
T4-32D	1951	154	2105	136	2241	9	53.6
T4-58D	1911	194	2105	238	2343	15	79.5
T8-5B	1965	141	2106	163	2269	9	50.4
T10-22B	2092	14	2106	0	2106	14	62.6
T9-5B	2107	0	2107	160	2267	19	84.7
T9-45B	2024	84	2108	0	2108	21	87.8
T8-27B	1936	174	2110	359	2469	12	51.3
T1-4D	1971	147	2118	236	2354	14	65.9
T2-51B	1878	240	2118	0	2118	17	76
T10-19A	1961	158	2119	0	2119	13	57.6
T4-59B	1832	288	2120	194	2314	6	32.5
T9-22B	1969	151	2120	186	2306	19	81.4
T2-49D	2010	111	2121	293	2414	10	50.8
T9-38D	1994	127	2121	63	2184	12	55.1
T9-29B	2061	63	2124	117	2241	24	92.5
T9-41A	2124	0	2124	283	2407	14	71.3
T9-13D	1738	387	2125	179	2304	15	76.1
T4-60	1837	289	2126	308	2434	8	45.4
T5-11A	1481	647	2128	93	2221	10	54.1
T9-50B	2000	131	2131	6	2137	16	73.4
T8-3A	2037	96	2133	256	2389	16	52.1
T9-71A	1861	274	2135	0	2135	13	62.1
T4-47B	2117	22	2139	127	2266	20	47.8
T1-19A	1046	1094	2140	224	2364	15	69.6
T9-40D	1665	476	2141	0	2141	18	80.9
T9-5D	2146	0	2146	171	2317	17	77.2
T2-51A	1991	158	2149	296	2445	18	86.4
T4-54D	2153	0	2153	155	2308	15	72.9
T6-17B	1995	167	2162	424	2586	17	82.6
T2-45A	2167	0	2167	0	2167	14	69.2
T2-25A	2100	68	2168	302	2470	24	99.1
T4-47D	1833	337	2170	135	2305	18	86.4
T9-6D	1923	247	2170	58	2228	15	71.8
T4-32B	2042	130	2172	131	2303	18	77.6
T2-53-7D	1315	865	2180	253	2433	21	91.1
T9-7A	1893	287	2180	265	2445	29	75.6
T9-13B	155	2027	2182	81	2263	14	69.2
T4-15-6A	2033	152	2185	253	2438	15	76.2
T5-24B	1828	364	2192	1811	4003	35	76.1
T8-12D	2002	193	2195	224	2419	16	70.1
T8-4B	1941	260	2201	285	2486	13	72
T9-51A	1643	562	2205	0	2205	13	54.4
T6-16A	2115	96	2211	198	2409	16	73.4
T9-14B	2212	0	2212	154	2366	15	50
T6-11D	2212	0	2212	0	2212	16	72.2
T4-10A	1421	796	2217	222	2439	7	41.6
T4-21B	2221	0	2221	366	2587	5	28.7
T6-17D	2137	85	2222	375	2597	13	65.8
T9-10B	1881	350	2231	376	2607	18	89.6
T10-46A	2231	0	2231	283	2514	10	52.9
T9-19B	646	1592	2238	221	2459	13	72
T9-40A	2241	0	2241	127	2368	15	69.6
T6-12B	1609	633	2242	49	2291	11	57.9
T10-22A	2192	62	2254	137	2391	15	71.8
T5-21A	2066	205	2271	0	2271	18	76.5
T1-19B	2099	175	2274	276	2550	11	61.2
T9-30A	1882	397	2279	0	2279	20	85.2
T6-7D	2109	173	2282	0	2282	15	79.5
T9-44A	2095	195	2290	185	2475	15	69.6
T10-34D	2174	117	2291	337	2628	4	20.5
T2-28B	2297	0	2297	475	2772	17	85.9
T2-33B	1415	892	2307	331	2638	11	54.6
T8-13B	1189	1121	2310	210	2520	12	61.6
T9-50A	2178	137	2315	304	2619	13	68.7
T2-47B	2016	302	2318	265	2583	11	57.9
T2-5A	1894	441	2335	105	2440	13	58.8
T9-9A	1143	1201	2344	63	2407	13	65.4
T9-73B	2175	177	2352	210	2562	12	64.9
T6-2B	312	2068	2380	351	2731	8	40
T5-21B	2192	189	2381	145	2526	14	59.3
T8-12A	2299	85	2384	274	2658	18	77.6
T9-60A	2271	114	2385	250	2635	7	36.2
T4-31B	2240	147	2387	361	2748	10	56.2
T4-15-6B	2337	66	2403	384	2787	17	82.6
T2-9B	2163	270	2433	243	2676	16	72.2
T10-10D	2315	147	2462	130	2592	17	77.2
T9-12A	2469	0	2469	0	2469	21	87.8
T8-16D	1987	485	2472	387	2859	7	46.1
T9-10D	2369	104	2473	289	2762	17	79.3
T5-21D	2288	186	2474	226	2700	15	66.4
T4-52B	2441	35	2476	0	2476	20	84
T1-13A	2108	372	2480	0	2480	16	73.4
T4-50D	2443	37	2480	11	2491	12	61.6
T4-38B	2498	0	2498	0	2498	14	69.2
T5-16D	2319	186	2505	280	2785	8	53.2
T1-14A	2263	296	2559	0	2559	16	72.2
T2-18D	2316	309	2625	114	2739	7	42.8
T6-18D	2681	0	2681	320	3001	18	86.4
T2-23A	2705	0	2705	0	2705	15	68.5
T2-21A	2368	386	2754	0	2754	9	47.1
T4-36B	2670	109	2779	19	2798	11	63.3
T4-47A	2490	371	2861	36	2897	5	24.2
T9-74B	2825	96	2921	213	3134	20	80.7
T8-16	2370	623	2993	276	3269	6	32.5
T2-51D	3240	128	3368	66	3434	37	85.4
T2-9D	3216	1614	4830	150	4980	19	90.1

trait gene	gene ID	promoter	5'UTR	promoter + 5'UTR	Exons / Introns	total bp	MYbaits	TARGET coverage (%)
T2-14D	TraesCS3D02G113600	306	70	376	253	629	1	7.1
T2-16D	TraesCS3D02G231700	465	109	574	161	735	1	7.1
T2-7A	TraesCS4A02G267000	510	129	639	507	1146	1	7.1
T4-5B	TraesCS2B02G340700	527	300	827	0	827	1	7.1
T9-52D	TraesCS7D02G296700	458	374	832	0	832	1	7.1
T9-55D	TraesCS7D02G149400	533	44	577	197	774	1	7.1
T10-35D	TraesCS4D02G381900LC	923	0	923	0	923	1	7.1
T10-40D	TraesCS2D02G318100	794	0	794	0	794	1	7.1
T10-45A	TraesCS5A02G381700LC	887	0	887	136	1023	1	7.1
T1-2D	TraesCS3D02G172900	1415	121	1536	343	1879	2	14.1
T1-22B	TraesCS7B02G090500	435	94	529	83	612	2	10.8
T1-5D	TraesCS2D02G385500	823	160	983	477	1460	2	14.1
T2-16A	TraesCS3A02G220800	765	87	852	0	852	2	10.8
T2-17D	TraesCS3D02G219900	544	0	544	270	814	2	14.1
T2-3A	TraesCS1A02G040600	310	298	608	280	888	2	14.1
T2-58D	TraesCS7D02G249500	810	134	944	341	1285	2	10.8
T2-59D	TraesCS1D02G246300	1241	128	1369	107	1476	2	10.8
T2-60A	TraesCS5A02G248700	315	290	605	1048	1653	2	10.8
T2-60A	TraesCS5A02G395200	1923	98	2021	160	2181	2	10.8
T8-11D	TraesCS1D02G203300	871	361	1232	0	1232	2	14.1
T8-17D	TraesCS2D02G385500	799	158	957	529	1486	2	14.1
T9-47A	TraesCS6A02G315100	705	96	801	0	891	2	10.8
T9-47D	TraesCS6D02G294400	793	0	793	0	793	2	10.8
T9-56D	TraesCS1D02G219600	1130	0	1130	368	1498	2	10.8
T10-25D	TraesCS4D02G092800	448	80	528	288	816	2	10.8
T10-9A	TraesCS1A02G162200	533	174	707	0	865	2	14.1
T1-15B	TraesCS3B02G223900	988	449	1437	117	1554	3	21.2
T1-22A	TraesCS7A02G185300	520	90	610	219	829	3	14.6
T4-19-7A	TraesCS7A02G009100	546	72	618	859	1477	3	17.9
T4-40A	TraesCS1A02G186800	917	78	995	184	1179	3	17.9
T4-8D	TraesCS2D02G411600	796	28	824	0	824	3	17.9
T5-30A	TraesCS1A02G119100	601	0	601	152	753	3	15.1
T5-6B	TraesCS5B02G132600	954	0	954	569	1523	3	14.6
T8-11A	TraesCS1A02G199600	443	285	728	223	951	3	17.8
T8-23D	TraesCS6D02G240200	819	274	1093	0	1093	3	14.6
T9-32B	TraesCS6B02G252000	696	0	696	303	999	3	14.6
T10-18A	TraesCS7A02G455900	745	398	1143	116	1259	3	14.6
T10-28A	TraesCS2A02G171900	588	78	666	261	927	3	17.9
T10-47B	TraesCS7B02G217600	544	524	1068	295	1363	3	17.9
T10-5A	TraesCS7A02G276600	1489	0	1489	229	1718	3	18
T10-7B	TraesCS5B02G085000	465	274	739	382	1121	3	17.9
T6-8D	TraesCS5D02G190700	531	185	716	289	1005	3	17.9
T6-9B	TraesCS1B02G083900	985	350	1335	1302	2637	3	17.9
T1-15A	TraesCS3A02G194100	989	122	1111	168	1279	4	24.9
T1-4B	TraesCS3B02G161000	1173	56	1229	133	1362	4	24.9
T1-4(B)D	TraesCS3D02G143500	940	61	1001	114	1115	4	24.9
T2-39B	TraesCS5B02G175800	1145	165	1310	151	1461	4	24.7
T2-8A	TraesCS6A02G309400	0	26	26	753	779	4	21.6
T2-8B	TraesCS6B02G339100	0	21	21	744	765	4	18.4
T2-8D	TraesCS6D02G288800	0	122	122	844	966	4	18.4
T4-11A	TraesCS7A02G111300	839	218	1057	49	1106	4	21.6
T4-19-4A	TraesCS4A02G486000	559	75	634	953	1587	4	28.2
T4-24D	TraesCS4D02G207900	1215	96	1311	178	1489	4	17.8
T4-40B	TraesCS1B02G194700	407	128	535	249	784	4	20.9
T4-43D	TraesCS3D02G270500	786	105	891	444	1435	4	23.9
T4-56B	TraesCS1B02G060700	579	393	972	0	972	4	24.9
T5-14B	TraesCS7B02G311600	913	174	1087	232	1319	4	24.9
T5-29B	TraesCS3B02G139100	834	0	834	33	867	4	24.9
T5-5B	TraesCS7B02G069800	778	196	974	963	1937	4	21.6
T5-6D	TraesCS5D02G140400	1028	0	1028	367	1395	4	17.2
T9-26B	TraesCS6B02G438700	679	91	770	282	1052	4	18.4
T9-29A	TraesCS5A02G144000	471	225	696	102	798	4	38
T9-33A	TraesCS1A02G082700	1186	0	1186	530	1716	4	21.6
T9-33D	TraesCS1D02G084100	2048	0	2048	349	2397	4	20.9
T9-52A	TraesCS7A02G301300	1190	429	1619	122	1741	4	21.6
T9-52B	TraesCS7B02G201600	618	508	1126	0	1126	4	21.6
T9-59D	TraesCS7D02G158500	1954	123	2077	229	2306	4	20.5
T9-66A	TraesCS7A02G375400	1316	179	1495	125	1620	4	21.6
T10-34D	TraesCS3D02G396500	2174	117	2291	337	2628	4	20.5
T10-6B	TraesCS2B02G333500	1775	98	1873	54	1927	4	24.9
T6-9D	TraesCS1D02G066700	1718	207	1925	0	1925	4	21.6
T1-2B	TraesCS3B02G433000	551	296	847	142	989	5	22.1
T1-25B	TraesCS1B02G113100	1325	60	1385	224	1609	5	28.8
T2-49A	TraesCS1A02G137200	1938	118	2056	229	2285	5	28.7
T4-11B	TraesCS7B02G009200	898	238	1136	47	1183	5	25.4
T4-21B	TraesCS6B02G179900	2221	0	2221	366	2587	5	28.7
T4-23A	TraesCS4A02G216100	983	153	1136	351	1487	5	28.7
T4-47A	TraesCS3A02G129000	2490	371	2861	36	2897	5	24.2
T4-55A	TraesCS2A02G583400	766	268	1034	175	1209	5	32
T4-59A	TraesCS6A02G241000	317	369	686	30	716	5	25.1
T5-14A	TraesCS7A02G412400	1009	166	1175	79	1254	5	32
T5-25D	TraesCS2D02G276000	988	334	1322	206	1528	5	31.8
T5-27B	TraesCS7B02G133800	850	234	1084	322	1406	5	32
T5-30B	TraesCS1B02G138600	711	169	880	309	1189	5	25.4
T5-30D	TraesCS1D02G120000	874	129	1003	169	1172	5	25.4
T5-7B	TraesCS5B02G193200	857	504	1361	325	1686	5	32
T8-18B	TraesCS5B02G319600	1740	276	2016	0	2016	5	24.2
T8-6B	TraesCS2B02G351000	899	0	899	0	899	5	25.4
T9-16D	TraesCS5D02G411600	690	0	690	23	713	5	28.7
T9-25D	TraesCS4D02G361700	783	0	783	554	1337	5	25.4
T9-56A	TraesCS1A02G217900	1478	377	1855	8	1863	5	28.7
T9-59B	TraesCS7B02G062200	678	93	771	259	1030	5	28.7
T9-8D	TraesCS5D02G388300	773	171	944	213	1157	5	45.7
T10-14B	TraesCS7B02G132000	1700	88	1788	479	2267	5	28.7
T10-44D	TraesCS4D02G159600	602	151	753	0	753	5	28.7
T1-17A	TraesCS2A02G176000	1107	434	1541	301	1842	6	35.8
T1-20B	TraesCS1B02G100400	1019	478	1497	405	1902	6	35
T1-3A	TraesCS5A02G215100	814	318	1132	82	1214	6	35.7
T1-6-7A	TraesCS7A02G000200	562	631	1193	291	1484	6	32.5
T2-15A	TraesCS2A02G436100	588	267	855	463	1318	6	29.2
T2-29D	TraesCS3D02G359200	1859	63	1922	265	2187	6	39.1
T2-39D	TraesCS5D02G182600	766	84	850	192	1042	6	29.2
T2-49B	TraesCS1B02G155700	842	156	998	344	1342	6	35.8
T2-50A	TraesCS1A02G091500	1543	184	1727	142	1869	6	39.9
T2-52B	TraesCS1B02G368500	1440	0	1440	0	1440	6	35.9
T2-58A	TraesCS7A02G251400	1682	0	1682	0	1682	6	32.5
T2-6D	TraesCS7D02G294300	642	808	1450	206	1656	6	32.5
T4-11D	TraesCS7D02G106400	955	329	1284	122	1406	6	32.5
T4-13D	TraesCS7D02G405500	595	249	844	167	1011	6	25.9
T4-16A	TraesCS7A02G310500	1329	433	1762	262	2024	6	36
T4-22D	TraesCS2D02G392800	1806	0	1806	0	1806	6	32.3
T4-23D	TraesCS4D02G096400	1639	165	1804	2302	4106	6	35.8
T4-32B(2)	TraesCS7B02G418500	1063	118	1181	218	1399	6	38.5
T4-5A	TraesCS2A02G343100	1419	320	1739	161	1900	6	32.5
T4-5D	TraesCS2D02G321400	607	293	900	88	988	6	35.8
T4-53A	TraesCS5A02G321300	1227	218	1445	130	1575	6	29
T4-59B	TraesCS6B02G283600	1832	288	2120	194	2314	6	32.5
T5-15D	TraesCS4D02G078100	1002	323	1325	169	1494	6	35.2
T5-16B	TraesCS5B02G494600	669	214	883	0	883	6	32.5
T8-11B	TraesCS1B02G214500	1231	0	1231	226	1457	6	35.8
T8-13A	TraesCS5A02G376500	0	968	968	352	1320	6	34.6
T8-16	TraesCS3A02G282800	2370	623	2993	276	3269	6	32.5
T8-18D	TraesCS5D02G325400	1769	226	1995	167	2162	6	32.5
T8-5A	TraesCS4A02G113000	892	99	991	0	991	6	60.1
T8-6A	TraesCS2A02G331200	925	0	925	0	925	6	25.9
T9-2A	TraesCS7A02G160800	1051	441	1492	173	1665	6	32.3
T9-2D	TraesCS7D02G161900	946	482	1428	444	1872	6	42.4
T9-36A	TraesCS7A02G307900	609	0	609	293	902	6	29.2
T9-64D	TraesCS7D02G414700	327	1002	1329	303	1632	6	39.6
T10-16D	TraesCS1D02G059600	1586	69	1655	187	1842	6	29.2
T10-17B	TraesCS2B02G234700	523	115	638	217	855	6	25.3
T10-23A	TraesCS1A02G288700	974	0	974	0	974	6	35.6
T10-24A	TraesCS3A02G151300	1048	0	1048	77	1125	6	32.5
T10-30A	TraesCS3A02G500600	1567	20	1587	56	1643	6	32.5
T10-43D	TraesCS3D02G196500	1923	0	1923	298	2221	6	25.9
T10-5B	TraesCS7B02G174300	1537	278	1815	185	2000	6	35.8
T6-4A	TraesCS7A02G301700	723	205	928	1076	2004	6	29.2
T1-28-3D	TraesCS3D02G024700	1287	56	1343	259	1602	7	39.5
T1-3B	TraesCS5B02G208700	1290	321	1611	0	1611	7	41.1
T1-3D	TraesCS5D02G216900	1239	333	1572	79	1651	7	35.9
T2-16B	TraesCS3B02G251100	998	42	1040	253	1293	7	32.9
T2-18D	TraesCS7D02G236300	2316	309	2625	114	2739	7	42.8
T2-19B	TraesCS6B02G206800	1268	174	1442	471	1913	7	36.2
T2-21D	TraesCS1D02G116900	1877	189	2066	0	2066	7	39.5
T2-24D	TraesCS7D02G235600	799	264	1063	214	1277	7	42.8
T4-10A	TraesCS4A02G106400	1421	796	2217	222	2439	7	41.6
T4-13B	TraesCS7B02G311600	893	172	1065	332	1397	7	29.6
T4-15-1D	TraesCS1D02G018500	734	0	734	278	1012	7	28.5
T4-32A	TraesCS7A02G508800	1675	95	1770	267	2037	7	39.5
T4-41-4B	TraesCS4B02G346900	1916	0	1916	0	1916	7	46.1
T5-16(B)-D	TraesCS5D02G494900	795	412	1207	0	1207	7	36.8
T5-28B	TraesCS3B02G024000	1870	178	2048	305	2353	7	36.2
T8-15B	TraesCS1B02G364800	1154	116	1270	193	1463	7	35.1
T8-16D	TraesCS3D02G282800	1987	485	2472	387	2859	7	46.1
T8-8D	TraesCS5D02G401700	1088	258	1346	393	1739	7	39.5
T9-18A	TraesCS6A02G276100	740	0	740	0	740	7	32.9
T9-20D	TraesCS2D02G194800	1105	617	1722	0	1722	7	42.8
T9-4D	TraesCS1D02G114800	1403	38	1441	298	1739	7	38.4
T9-41D	TraesCS2D02G308000	766	422	1188	261	1449	7	42.8
T9-57A	TraesCS6A02G343300	1554	105	1659	58	1717	7	39.2
T9-60A	TraesCS4A02G219700	2271	114	2385	250	2635	7	36.2
T9-76A	TraesCS7A02G363100	1261	145	1406	496	1902	7	38.4
T10-36A	TraesCS7A02G295800	1315	226	1541	109	1650	7	42.8
T10-40B	TraesCS2B02G338300	1382	469	1851	281	2132	7	42.8
T10-43A	TraesCS3A02G198600	948	0	948	315	1263	7	31.8
T10-47D	TraesCS7D02G313500	688	480	1168	190	1358	7	32.9
T6-13A	TraesCS6A02G298100	1830	203	2033	102	2135	7	42.6
T1-2A	TraesCS3A02G212900LC	1605	157	1762	274	2036	8	42.1
T1-21D	TraesCS6D02G285300	1403	204	1607	0	1607	8	43.3
T1-24B	TraesCS6B02G266200	1005	596	1601	492	2093	8	49.9
T1-25D	TraesCS1D02G094200	1557	0	1557	341	1898	8	40
T2-10A	TraesCS1A02G351900	782	349	1131		2337	8	45.4
T2-11A	TraesCS7A02G287500	0	2096	2096	312	2408	8	49.9
T2-29B	TraesCS3B02G397900	1702	65	1767	157	1924	8	39.1
T2-31D	TraesCS2D02G166600	1856	37	1893	139	2032	8	43.3
T2-57B	TraesCS2B02G327300	0	0	0	2314	2314	8	42.1
T2-59B	TraesCS1B02G257700	1959	0	1959	183	2142	8	31.3
T2-7D	TraesCS4D02G047300	1325	0	1325	265	1590	8	36.7
T4-13A	TraesCS7A02G412400	1054	163	1217	181	1398	8	43.3
T4-19-7D	TraesCS7D02G008700	992	71	1063	1017	2080	8	38.8
T4-59D	TraesCS6D02G223300	1606	184	1790	155	1945	8	42.1
T4-6D	TraesCS6D02G186900	0	0	0	2624	2624	8	42.1
T4-60	TraesCS6B02G111800	1837	289	2126	308	2434	8	45.4
T5-10-5A	TraesCS5A02G558200	849	389	1238	20	1258	8	43.3
T5-16D	TraesCS5D02G495000	2319	186	2505	280	2785	8	53.2
T5-20A	TraesCS4A02G208400	924	0	924	229	1153	8	38.8
T5-20D	TraesCS4D02G109600	896	79	975	116	1091	8	39.9
T5-25A	TraesCS2A02G277100	1307	263	1570	149	1719	8	43.3
T5-28D	TraesCS3D02G025000	1556	165	1721	159	1880	8	49.9
T8-8A	TraesCS5A02G391800	1164	323	1487	309	1796	8	38.8
T9-17D	TraesCS5D02G120500	1290	562	1852	288	2140	8	40
T9-25A	TraesCS5A02G537300	1627	0	1627	164	1791	8	40
T9-28A	TraesCS4A02G415400	1117	0	1117	338	1455	8	40
T9-36B	TraesCS7B02G208300	848	225	1073	276	1349	8	46.6
T9-37D	TraesCS3D02G151400	485	461	946	0	946	8	67.8
T9-42B	TraesCS2B02G489800	1334	117	1451	220	1671	8	43.3
T9-6A	TraesCS2A02G327900	1684	218	1902	34	1936	8	46.6
T9-61B	TraesCS5B02G480900	1818	92	1910	227	2137	8	40
T9-74D	TraesCS5D02G162600	841	76	917	184	1101	8	34.2
T9-75D	TraesCS6D02G173000	1871	0	1871	0	1871	8	45.4
T10-16B	TraesCS1B02G075900	816	29	845	301	1146	8	36.7
T10-38A	TraesCS5A02G303200	972	113	1085	155	1240	8	40
T10-40A	TraesCS2A02G340400	1635	354	1989	0	1989	8	43.3
T10-45B	TraesCS5B02G256100	773	0	773	402	1175	8	37.1
T10-46B	TraesCS2B02G356200	1768	90	1858	370	2228	8	43.3
T10-48D	TraesCS5D02G172800	1943	0	1943	124	2067	8	43.3
T10-6A	TraesCS2A02G315000	1925	105	2030	19	2049	8	46.6
T6-2B	TraesCS7B02G062100	312	2068	2380	351	2731	8	40
T1-10B	TraesCS2B02G311900	1209	124	1333	296	1629	9	43.8
T1-12B	TraesCS1B02G107000	1854	124	1978	407	2385	9	40
T1-20A	TraesCS1A02G083000	1016	336	1352	328	1680	9	43.1
T1-20D	TraesCS1D02G084200	1243	321	1564	425	1989	9	49.2
T1-25A	TraesCS1A02G102400	1515	0	1515	158	1673	9	53.6
T1-6-7D	TraesCS7D02G000100	665	714	1379	451	1830	9	43.8
T2-18A	TraesCS7A02G236200	1704	212	1916	183	2099	9	43.8
T2-21A	TraesCS1A02G115500	2368	386	2754	0	2754	9	47.1
T2-22B	TraesCS2B02G144300	596	79	675	54	729	9	38.7
T2-35D	TraesCS5D02G484500	1965	9	1974	0	1974	9	47.1
T2-5D	TraesCS7D02G284900	1446	262	1708	172	1880	9	37.2
T2-56B	TraesCS2B02G309500	1276	220	1496	0	1496	9	54.2
T2-59A	TraesCS1A02G247100	1700	110	1810	0	1810	9	47.1
T2-61B	TraesCS6B02G210000	763	728	1491	255	1746	9	43.8
T4-1D	TraesCS7D02G265400	0	0	0	1180	1180	9	39.8
T4-21D	TraesCS6D02G141700	467	158	625	1438	2063	9	50.4
T4-32D	TraesCS7D02G497400	1951	154	2105	136	2241	9	53.6
T4-38D	TraesCS3D02G126200	1486	127	1613	0	1613	9	47.1
T4-43A	TraesCS3A02G270800	1897	116	2013	0	2013	9	50.4
T4-45B	TraesCS7B02G766300LC	1104	0	1104	68	1172	9	40.5
T4-56A	TraesCS1A02G047000	488	775	1263	0	1263	9	47.1
T4-60A	TraesCS6A02G089400	982	0	982	0	982	9	46.8
T5-26D	TraesCS3D02G046300	871	105	976	247	1223	9	90.7
T5-5A	TraesCS7A02G164000	250	0	250	1135	1385	9	50.6
T5-7D	TraesCS5D02G200900	1854	0	1854	271	2125	9	39.3
T8-16B	TraesCS3B02G316500	1579	181	1760	240	2000	9	43.8
T8-5B	TraesCS4B02G191200	1965	141	2106	163	2269	9	50.4
T8-5D	TraesCS4D02G192400	1146	113	1259	195	1454	9	47.1
T8-6D	TraesCS2D02G331700	1114	142	1256	0	1256	9	40.5
T8-8B	TraesCS5B02G396700	1493	232	1725	297	2022	9	42.6
T9-15D	TraesCS7D02G408500	1370	261	1631	806	2437	9	48.8
T9-20A	TraesCS2A02G192600	647	367	1014	312	1326	9	43.3
T9-22D	TraesCS4D02G122000	1047	150	1197	162	1359	9	40.5
T9-3B	TraesCS6B02G350900	1313	0	1313	0	1313	9	47.1
T9-33B	TraesCS1B02G100300	948	46	994	254	1248	9	45.9
T9-38B	TraesCS6B02G033300	990	140	1130	190	1320	9	40.5
T9-39D	TraesCS3D02G173200	1020	327	1347	230	1577	9	50.4
T9-53A	TraesCS1A02G196100	1199	0	1199	416	1615	9	42.6
T9-56B	TraesCS1B02G231300	1662	382	2044	317	2361	9	40.5
T9-64B	TraesCS7B02G322900	1415	689	2104	384	2488	9	56.2
T9-70B	TraesCS3B02G466400	714	156	870	323	1193	9	37.2
T10-38B	TraesCS5B02G303500	1465	0	1465	0	1465	9	47.1
T10-5D	TraesCS7D02G276600	1624	0	1624	363	1987	9	50.4
T10-7D	TraesCS7D02G276600	1612	0	1612	354	1966	9	50.4
T6-10A	TraesCS6A02G017500	862	164	1026	305	1331	9	44.1
T6-6B	TraesCS4B02G375800	492	63	555	351	906	9	43.1
T1-12A	TraesCS1A02G088000	1779	194	1973	385	2358	10	47.5
T1-21A	TraesCS6A02G306200	1066	272	1338	0	1338	10	47.5
T1-27B	TraesCS6B02G416400	1347	611	1958	378	2336	10	57.4
T1-5A	TraesCS2A02G188500	1328	0	1328	0	1328	10	47.5
T2-15B	TraesCS2B02G457100	308	70	378	108	486	10	43.1
T2-19A	TraesCS6A02G178000	1757	212	1969	441	2410	10	47.5
T2-23D	TraesCS7D02G483000	1425	656	2081	94	2175	10	52.9
T2-24B	TraesCS7B02G133900	1546	305	1851	168	2019	10	54.1
T2-3D	TraesCS1D02G041200	1628	299	1927	213	2140	10	47.5
T2-45D	TraesCS7D02G190100	1745	0	1745	0	1745	10	59.5
T2-49D	TraesCS1D02G138100	2010	111	2121	293	2414	10	50.8
T2-50B	TraesCS1B02G119300	1508	366	1874	66	1940	10	57.4
T2-6A	TraesCS7A02G298600	1323	341	1664	111	1775	10	52.9
T2-6B	TraesCS7B02G188400	1058	335	1393	170	1563	10	50.8
T4-14B	TraesCS3B02G158300LC	1181	0	1181	0	1181	10	43.1
T4-24A	TraesCS4A02G097400	1705	60	1765	235	2000	10	49.6
T4-24B	TraesCS4B02G207100	1295	115	1410	202	1612	10	53.6
T4-31B	TraesCS6B02G175100	2240	147	2387	361	2748	10	56.2
T4-40D	TraesCS1D02G189800	1480	121	1601	234	1835	10	50.1
T4-44B	TraesCS5B02G462200	1080	0	1080	210	1290	10	54.4
T4-54A	TraesCS7A02G176700	1251	108	1359	0	1359	10	65.7
T4-6B	TraesCS6B02G223700	1862	234	2096	101	2197	10	57.4
T4-9D	TraesCS3D02G227500	1454	116	1570	571	2141	10	60.7
T5-1A	TraesCS4A02G035400	928	151	1079	231	1310	10	46.7
T5-11A	TraesCS1A02G176700	1481	647	2128	93	2221	10	54.1
T5-16(B)-A	TraesCS5A02G6481300	1918	0	1918	0	1918	10	60.7
T8-13D	TraesCS5D02G386500	100	1021	1121	67	1188	10	47.1
T8-2B	TraesCS2B02G237300	1399	198	1597	0	1597	10	50.8
T9-15B	TraesCS7B02G315200	1932	0	1932	751	2683	10	60.7
T9-3A	TraesCS6A02G320100	1363	0	1363	26	1389	10	47.5
T9-31D	TraesCS4D02G077600	1682	0	1682	299	1981	10	47.5
T9-35A	TraesCS5A02G142700	1586	84	1670	245	1915	10	57.4
T9-37B	TraesCS3B02G169200	1177	329	1506	0	1506	10	50.8
T9-40B	TraesCS2B02G251700	1654	0	1654	0	1654	10	54.1
T9-46D	TraesCS5D02G323500	1864	0	1864	61	1925	10	54.1
T9-58D	TraesCS1D02G214200	1845	142	1987	24	2011	10	60.7
T9-60B	TraesCS4B02G098200	1325	60	1385	38	1423	10	54.4
T10-1B	TraesCS4B02G198400	2066	0	2066	0	2066	10	46.4
T10-10B	TraesCS3B02G143800	1877	225	2102	163	2265	10	46.4
T10-11B	TraesCS4B02G045000	921	72	993	137	1130	10	46.4
T10-2A	TraesCS2A02G300300	1120	482	1602	175	1777	10	47.5
T10-29D	TraesCS7D02G199800	1039	0	1039	222	1261	10	44.2
T10-31B	TraesCS5B02G355000	1249	594	1843	277	2120	10	43.1
T10-36D	TraesCS7D02G289400	1045	186	1231	136	1367	10	44.2
T10-46A	TraesCS2A02G325700	2231	0	2231	283	2514	10	52.9
T10-48A	TraesCS5A02G168500	457	1010	1467		2512	10	57.4
T1-1A	TraesCS6A02G189300	1811	184	1995	266	2261	11	54.6
T1-1B	TraesCS6B02G215300	1645	167	1812	342	2154	11	54.6
T1-11B	TraesCS5B02G375800	1552	297	1849	140	1989	11	54.2
T1-12D	TraesCS1D02G089300	1714	307	2021	463	2484	11	54.6
T1-16B	TraesCS1B02G364500	1851	120	1971	0	1971	11	48
T1-18D	TraesCS2D02G385900	1978	116	2094	162	2256	11	53.4
T1-19B	TraesCS6B02G286600	2099	175	2274	276	2550	11	61.2
T1-23D	TraesCS5D02G229400	1710	258	1968	23	1991	11	50.1
T2-12A	TraesCS5A02G441400	1750	306	2056	51	2107	11	53.4
T2-19D	TraesCS6D02G162000	1516	182	1698	463	2161	11	51.3
T2-23B	TraesCS7B02G401600	1351	629	1980	311	2291	11	57.9
T2-28D	TraesCS3D02G096000	1667	0	1667	273	1940	11	65.3
T2-29A	TraesCS3A02G366400	0	0	0	1921	1921	11	61.4
T2-33B	TraesCS2B02G145700	1415	892	2307	331	2638	11	54.6
T2-33D	TraesCS2D02G126600	1133	842	1975	240	2215	11	63.8
T2-37A	TraesCS1A02G419600	297	1173	1470	476	1946	11	57.9
T2-44D	TraesCS5D02G004300	1065	0	1065	268	1333	11	43.5
T2-47B	TraesCS6B02G336400	2016	302	2318	265	2583	11	57.9
T2-52D	TraesCS1D02G303700	1596	346	1942	576	2518	11	60.1
T2-7B	TraesCS4B02G047300	1707	142	1849	450	2299	11	54.6
T4-1B	TraesCS7B02G162500	0	0	0	1731	1731	11	51.3
T4-36B	TraesCS3B02G236300	2670	109	2779	19	2798	11	63.3
T4-53B	TraesCS5B02G321800	1643	250	1893	110	2003	11	57.9
T4-58A	TraesCS5A02G447200	0	861	861	240	1101	11	54.6
T4-7A	TraesCS7A02G293300	1736	182	1918	152	2070	11	51.3
T4-7D	TraesCS7D02G291200	1552	261	1813	218	2031	11	61.2
T4-8A	TraesCS2A02G414300	1323	152	1475	97	1572	11	54.6
T5-1D	TraesCS4D02G267500	1330	0	1330	179	1509	11	56.7
T5-18D	TraesCS3D02G099500	2069	0	2069	0	2069	11	60.5
T5-2D	TraesCS3D02G099500	2071	0	2071	0	2071	11	60
T5-23B	TraesCS3B02G388900	1623	179	1802	0	1802	11	49.3
T5-24D	TraesCS4D02G134900	1184	210	1394	553	1947	11	53.4
T5-3B	TraesCS1B02G235100	1609	134	1743	135	1878	11	61.2
T8-22A	TraesCS1A02G339300	1450	269	1719	0	1719	11	56.7
T8-9A	TraesCS2A02G174300	1486	198	1684	145	1829	11	51.3
T9-16B	TraesCS5B02G406100	1414	112	1526	34	1560	11	51.3
T9-19D	TraesCS4D02G256700	0	1993	1993	183	2176	11	64.5
T9-21B	TraesCS4B02G167100	1304	359	1663	0	1663	11	51.3
T9-21D	TraesCS4D02G127600	1195	367	1562	0	1562	11	54.6
T9-24B	TraesCS4B02G001200	1983	0	1983	297	2280	11	61.2
T9-25B	TraesCS4B02G375600	1782	62	1844	272	2116	11	61.2
T9-26A	TraesCS6A02G398400	758	82	840	185	1025	11	57.9
T9-35D	TraesCS5D02G149400	1506	81	1587	270	1857	11	61.2
T9-36D	TraesCS7D02G304800	1918	103	2021	288	2309	11	64.5
T9-38A	TraesCS6A02G024300	1710	129	1839	196	2035	11	53.4
T9-39A	TraesCS3A02G167100	1336	269	1605	232	1837	11	61.2
T9-4A	TraesCS1A02G113400	1310	108	1418	306	1724	11	50.1
T9-51B	TraesCS2B02G119200	1623	128	1751	0	1751	11	53.4
T9-61D	TraesCS5D02G481200	1249	149	1398	120	1518	11	45.3
T9-64A	TraesCS7A02G422500	1756	0	1756	0	1756	11	50.1
T9-66B	TraesCS7B02G277000	1744	327	2071	171	2242	11	53.4
T9-70D	TraesCS3D02G424400	1951	152	2103	97	2200	11	51.3
T9-75B	TraesCS6B02G214900	1999	0	1999	336	2335	11	61.2
T10-1A	TraesCS4A02G105600	1292	105	1397	321	1718	11	54.6
T10-10A	TraesCS3A02G124100	2000	0	2000	384	2384	11	54.6
T10-12A	TraesCS5A02G174300	1882	0	1882	0	1882	11	53.4
T10-12B	TraesCS5B02G171700	1872	101	1973	0	1973	11	53.4
T10-13D	TraesCS5D02G157500	1964	31	1995	476	2471	11	53.4
T10-21D	TraesCS6D02G182800	1369	91	1460	280	1740	11	56.4
T10-38D	TraesCS5D02G310000	1472	0	1472	0	1472	11	51.8
T10-41A	TraesCS5A02G286500	1290	0	1290	87	1377	11	51.3
T10-47A	TraesCS7A02G316600	1267	261	1528	0	1528	11	54.2
T10-7A	TraesCS5A02G081400	805	286	1091	321	1412	11	54.6
T6-12B	TraesCS2B02G528300	1609	633	2242	49	2291	11	57.9
T6-13B	TraesCS6B02G327500	1374	245	1619	99	1718	11	67.8
T6-8A	TraesCS6A02G185600	1613	129	1742	313	2055	11	56.7
T1-1D	TraesCS6D02G176900	1693	193	1886	210	2096	12	55.1
T1-11D	TraesCS5D02G383300	1453	0	1453	0	1453	12	52.4
T1-15D	TraesCS3D02G197700	1345	421	1766	62	1828	12	64.9
T1-27A	TraesCS6A02G378500	1606	231	1837	389	2226	12	67.1
T1-9A	TraesCS7A02G260500	1637	124	1761	0	1761	12	61.6
T1-9B	TraesCS7B02G158500	1721	239	1960	0	1960	12	64.9
T1-9D	TraesCS7D02G261500	1364	553	1917	0	1917	12	64.9
T2-18B	TraesCS7B02G134200	1547	120	1667	47	1714	12	64.9
T2-32D	TraesCS7D02G168000	1789	0	1789	434	2223	12	55.9
T2-43A	TraesCS7A02G120300	1596	144	1740	0	1740	12	55.1
T2-57A	TraesCS2A02G310300	1870	205	2075	394	2469	12	64.9
T2-57D	TraesCS2D02G308600	1813	148	1961	231	2192	12	64.9
T2-61A	TraesCS6A02G175100	857	1142	1999	276	2275	12	57.2
T2-9A	TraesCS3A02G036600	1556	0	1556	133	1689	12	61.6
T4-10D	TraesCS4D02G198600	1336	245	1581	206	1787	12	58.4
T4-14D	TraesCS3D02G107500	1738	109	1847	90	1937	12	58.4
T4-22B	TraesCS2B02G413000	1788	0	1788	0	1788	12	61.6
T4-26-4B	TraesCS4B02G322700	1868	221	2089	166	2255	12	67.1
T4-31D	TraesCS6D02G136200	1214	587	1801	235	2036	12	64.2
T4-39D	TraesCS2D02G264200	1745	82	1827	294	2121	12	68.2
T4-41-5A	TraesCS5A02G515600	1667	0	1667	0	1667	12	61.6
T4-42B	TraesCS4B02G287100	1686	84	1770	370	2140	12	64.9
T4-42D	TraesCS4D02G285800	1808	89	1897	194	2091	12	68.2
T4-50D	TraesCS5D02G268800	2443	37	2480	11	2491	12	61.6
T4-51D	TraesCS1D02G098000	1731	101	1832	297	2129	12	55.1
T4-55B	TraesCS2B02G603100	1670	0	1670	285	1955	12	61.2
T4-6A	TraesCS6A02G203600.3	520	0	520	1738	2258	12	71.5
T5-1B	TraesCS4B02G268100	700	239	939	155	1094	12	55.1
T5-11D	TraesCS1D02G162400	2004	0	2004	152	2156	12	60.5
T5-13A	TraesCS7A02G178000	636	1233	1869	384	2253	12	55.1
T5-2B	TraesCS3B02G115400	1975	0	1975	0	1975	12	57.6
T5-29A	TraesCS3A02G119500	1878	135	2013	239	2252	12	64.9
T5-29D	TraesCS3D02G121700	1647	205	1852	63	1915	12	64.9
T5-4D	TraesCS3D02G099500	2071	0	2071	0	2071	12	57.6
T5-8B	TraesCS5B02G387100	1241	250	1491	0	1491	12	58.9
T8-13B	TraesCS5B02G380200	1189	1121	2310	210	2520	12	61.6
T8-17B	TraesCS2B02G405700	1657	0	1657	619	2276	12	57.4
T8-2A	TraesCS2A02G212200	1815	145	1960	0	1960	12	58.4
T8-23B	TraesCS6B02G286400	1432	279	1711	0	1711	12	51.8
T8-26D	TraesCS3D02G011100	1356	111	1467	348	1815	12	58.4
T8-27B	TraesCS7B02G080300	1936	174	2110	359	2469	12	51.3
T8-9D	TraesCS2D02G181400	1765	202	1967	169	2136	12	69.6
T9-1B	TraesCS2B02G279400	943	1049	1992	299	2291	12	64.9
T9-14A	TraesCS4B02G111100	1810	0	1810	272	2082	12	58.4
T9-18D	TraesCS6D02G256400	1911	152	2063	285	2348	12	61.6
T9-21A	TraesCS4A02G138600	1676	0	1676	0	1676	12	55.1
T9-38D	TraesCS6D02G027700	1994	127	2121	63	2184	12	55.1
T9-42D	TraesCS2D02G467000	1345	186	1531	421	1952	12	56.9
T9-55B	TraesCS7B02G084800LC	1573	0	1573	0	1573	12	53.9
T9-58B	TraesCS1B02G225000	1734	113	1847	50	1897	12	61.6
T9-61A	TraesCS5A02G468300	1567	0	1567	0	1567	12	54
T9-63D	TraesCS5D02G491100	1283	135	1418	114	1532	12	61.6
T9-73B	TraesCS3B02G205800	2175	177	2352	210	2562	12	64.9
T9-77D	TraesCS7D02G215600	1907	0	1907	0	1907	12	57.8
T9-9B	TraesCS7B02G141300	1372	442	1814	101	1915	12	61.6
T10-13A	TraesCS5A02G152300	1888	30	1918	308	2226	12	57.2
T10-14A	TraesCS7A02G233700	1110	143	1253	387	1640	12	51.8
T10-18D	TraesCS7D02G444800	1662	420	2082	0	2082	12	68.5
T10-21B	TraesCS6B02G229400	1695	67	1762	142	1904	12	58.4
T10-31D	TraesCS5D02G359800	1608	0	1608	0	1608	12	51.8
T10-35B	TraesCS4B02G280400	1142	105	1247	148	1395	12	51.8
T10-37B	TraesCS1B02G463400	983	1106	2089	0	2089	12	57.2
T10-37D	TraesCS1D02G438000	947	1119	2066	0	2066	12	58.4
T10-4	TraesCS2B02G292500	0	0	0	1881	1881	12	59.3
T10-43B	TraesCS3B02G227200	1478	0	1478	445	1923	12	55.1
T10-6D	TraesCS2D02G313300	1651	155	1806	0	1806	12	64.9
T10-9B	TraesCS1B02G178800	1522	236	1758	0	1758	12	68.2
T6-10D	TraesCS6D02G020700	1197	161	1358	333	1691	12	58.5
T6-15B	TraesCS6B02G044100	1778	0	1778	216	1994	12	68.2
T6-17A	TraesCS6A02G031000	1548	93	1641	276	1917	12	61.6
T6-4D	TraesCS7D02G297000	667	155	822	1036	1858	12	61.6
T6-6A	TraesCS5A02G537100	1335	46	1381	300	1681	12	54
T6-7B	TraesCS5B02G183800	1695	0	1695	0	1695	12	61.2
T1-11A	TraesCS5A02G373900	1497	0	1497	0	1497	13	58.2
T1-13B	TraesCS5B02G029100	1694	363	2057	0	2057	13	57.6
T1-18B	TraesCS5B02G379300	1779	0	1779	311	2090	13	60.9
T1-22D	TraesCS7D02G187000	1449	102	1551	21	1572	13	65.4
T1-26B	TraesCS5B02G025100	1508	459	1967	0	1967	13	68.7
T2-24A	TraesCS7A02G235500	852	291	1143	1021	2164	13	65.4
T2-31B	TraesCS2B02G184900	2051	26	2077	119	2196	13	67.5
T2-39A	TraesCS5A02G292900LC	1597	0	1597	274	1871	13	58.8
T2-43D	TraesCS7D02G117800	0	0	0	2013	2013	13	62.1
T2-47A	TraesCS6A02G307800	1346	0	1346	0	1346	13	56.2
T2-5A	TraesCS7A02G287400	1894	441	2335	105	2440	13	58.8
T4-12A	TraesCS7A02G373500	1104	0	1104	250	1354	13	52.2
T4-25B	TraesCS4B02G207000	1618	169	1787	159	1946	13	57.6
T4-26-4D	TraesCS4D02G319100	1545	425	1970	195	2165	13	67.5
T4-26-5A	TraesCS5A02G494800	1450	427	1877	112	1989	13	68.7
T4-35D	TraesCS1D02G296300	1246	246	1492	0	1492	13	65.4
T4-51A	TraesCS1A02G089500	1716	128	1844	326	2170	13	63.2
T4-53D	TraesCS5D02G328100	1888	206	2094	96	2190	13	72
T5-13D	TraesCS7D02G179800	596	1292	1888	326	2214	13	62.1
T8-19B	TraesCS3B02G108500	1196	834	2030	192	2222	13	68.7
T8-21B	TraesCS5B02G486900	1434	0	1434	0	1434	13	59.8
T8-27D	TraesCS7D02G176700	1901	132	2033	314	2347	13	58.4
T8-4B	TraesCS3B02G280700	1941	260	2201	285	2486	13	72
T8-7B	TraesCS6B02G095600	1292	610	1902	188	2090	13	70.8
T9-17A	TraesCS5A02G106400	1119	726	1845	356	2201	13	60.9
T9-19B	TraesCS4B02G256600	646	1592	2238	221	2459	13	72
T9-32A	TraesCS6A02G229500	1822	214	2036	345	2381	13	71.3
T9-34D	TraesCS1D02G387100	1737	223	1960	0	1960	13	67.5
T9-50A	TraesCS5A02G069500	2178	137	2315	304	2619	13	68.7
T9-51A	TraesCS2A02G102100	1643	562	2205	0	2205	13	54.4
T9-54B	TraesCS7B02G044400	1969	30	1999	424	2423	13	65.4
T9-54D	TraesCS7D02G142900	1985	35	2020	140	2160	13	60.9
T9-58A	TraesCS1A02G210900	1478	98	1576	0	1576	13	68.7
T9-60D	TraesCS4D02G094400	1954	123	2077	229	2306	13	67.5
T9-69A	TraesCS6A02G318400	1723	371	1994	208	2202	13	65.4
T9-69B	TraesCS6B02G348700	1332	337	1669	0	1669	13	58.8
T9-71A	TraesCS4A02G247200	1861	274	2135	0	2135	13	62.1
T9-74A	TraesCS5A02G157300	1712	92	1804	241	2045	13	62.1
T9-8A	TraesCS5A02G378300	1233	401	1634	290	1924	13	62.1
T9-9A	TraesCS7A02G249900	1143	1201	2344	63	2407	13	65.4
T10-19A	TraesCS6A02G335700	1961	158	2119	0	2119	13	57.6
T10-2D	TraesCS7D02G296000	1911	29	1940	282	2222	13	72
T10-20B	TraesCS5B02G131900	1377	204	1581	0	1581	13	58.8
T10-4	TraesCS2D02G274100	0	0	0	1982	1982	13	70.8
T10-41D	TraesCS5D02G294200	1884	35	1919	338	2257	13	65.4
T10-44A	TraesCS4A02G143200	1877	205	2082	0	2082	13	60.9
T6-17D	TraesCS6D02G035700	2137	85	2222	375	2597	13	65.8
T6-4B	TraesCS7B02G201900	1717	256	1973	57	2030	13	60.9
T1-19D	TraesCS4B02G287900	1721	182	1903	309	2212	14	67.9
T1-24A	TraesCS6A02G237700	1500	527	2027	286	2313	14	74.6
T1-26A	TraesCS5A02G025900	1546	381	1927	0	1927	14	69.2
T1-28-3A	TraesCS3A02G021900	1726	49	1775	344	2119	14	68
T1-4D	TraesCS3D02G143300	1971	147	2118	236	2354	14	65.9
T1-5B	TraesCS2B02G405700	1649	0	1649	227	1876	14	65.9
T1-7B	TraesCS4B02G292300	1732	179	1911	305	2216	14	72.5
T2-10B	TraesCS1B02G371300	610	933	1543	0	1543	14	65.9
T2-2B	TraesCS5B02G244300	1704	42	1746	387	2133	14	59.3
T2-31A	TraesCS2A02G159300	1812	84	1896	117	2013	14	76.3
T2-43B	TraesCS7B02G018600	1553	302	1855	0	1855	14	62.6
T2-45A	TraesCS7A02G189000	2167	0	2167	0	2167	14	69.2
T2-45B	TraesCS7B02G093800	1313	285	1598	218	1816	14	65.9
T4-10B	TraesCS4B02G197800	1661	104	1765	319	2084	14	65.2
T4-14A	TraesCS3A02G105400	1724	110	1834	111	1945	14	65.9
T4-16D	TraesCS7D02G307000	922	0	922	234	1156	14	71.1
T4-20-4A	TraesCS4A02G485900	1885	127	2012	253	2265	14	69.2
T4-21A	TraesCS6A02G151700	1100	498	1598	283	1881	14	69.2
T4-22A	TraesCS2A02G395000	1521	183	1704	29	1733	14	65.9
T4-29D	TraesCS7D02G534700	1563	79	1642	77	1719	14	61.1
T4-31A	TraesCS6A02G146900	1781	172	1953	449	2402	14	69.2
T4-33-7D	TraesCS7D02G080300	1236	411	1647	231	1878	14	65.9
T4-35A	TraesCS1A02G295900	1562	330	1892	0	1892	14	69.2
T4-38B	TraesCS3B02G144800	2498	0	2498	0	2498	14	69.2
T4-46B	TraesCS5B02G224800	1577	131	1708	723	2431	14	68.6
T4-54B	TraesCS7B02G081700	1627	0	1627	0	1627	14	69.2
T5-10-4B	TraesCS4B02G396900	936	697	1633	272	1905	14	67.5
T5-21B	TraesCS2B02G167900	2192	189	2381	145	2526	14	59.3
T5-22D	TraesCS3D02G045700	1901	0	1901	290	2191	14	69.2
T8-19A	TraesCS3A02G093200	1169	845	2014	219	2233	14	75.8
T8-2D	TraesCS2D02G218100	1734	69	1803	0	1803	14	75.8
T8-3D	TraesCS5D02G240500	1983	29	2012	182	2194	14	69.2
T8-4A	TraesCS3A02G251200	1679	206	1885	242	2127	14	65.9
T8-4D	TraesCS3D02G251600	1545	260	1805	171	1976	14	72.5
T8-7A	TraesCS6A02G071200	1000	608	1608	210	1818	14	79.6
T8-9B	TraesCS2B02G200800	957	239	1196	0	1196	14	72.5
T9-13A	TraesCS5A02G222400	1108	349	1457	977	2434	14	75.8
T9-13B	TraesCS5B02G221400	155	2027	2182	81	2263	14	69.2
T9-19A	TraesCS4A02G048200	161	1385	1546	322	1868	14	78.6
T9-23D	TraesCS4D02G040400	1213	0	1213	63	1276	14	59
T9-26D	TraesCS6D02G382600	1723	93	1816	120	1936	14	69.2
T9-28B	TraesCS4B02G316200	1723	0	1723	374	2097	14	69.2
T9-41A	TraesCS2A02G309700	2124	0	2124	283	2407	14	71.3
T9-42A	TraesCS2A02G467000	1682	126	1808	173	1981	14	65.9
T9-50D	TraesCS5D02G081700	1586	237	1823	197	2020	14	69.2
T9-51D	TraesCS2D02G101500	1642	427	2069	0	2069	14	61.4
T9-53D	TraesCS1D02G199500	1601	482	2083	408	2491	14	71.3
T9-67A	TraesCS1A02G304200	1424	462	1886	629	2515	14	75.8
T9-69D	TraesCS6D02G297600	1531	362	1893	236	2129	14	72.5
T9-70A	TraesCS3A02G428800	1678	151	1729	145	1874	14	72.5
T9-73A	TraesCS3A02G176100	1944	0	1944	193	2137	14	79.8
T9-9D	TraesCS7D02G248500	389	471	860	1127	1987	14	79.1
T10-13B	TraesCS5B02G150900	1953	31	1984	448	2432	14	64.7
T10-22B	TraesCS3B02G414200	2092	14	2106	0	2106	14	62.6
T10-3D	TraesCS7D02G403500	1841	0	1841	300	2141	14	69.2
T10-42A	TraesCS5A02G042200	653	747	1400	374	1774	14	60.9
T6-31A	TraesCS4A02G416400	1174	43	1217	316	1533	14	47.3
T6-6D	TraesCS4D02G361500	1986	83	2069	258	2327	14	61.4
T1-14B	TraesCS2B02G194900	1785	315	2100	0	2100	15	68.5
T1-19A	TraesCS6A02G016300	1046	1094	2140	224	2364	15	69.6
T1-8-7D	TraesCS7D02G015000	1541	346	1887	0	1887	15	72.9
T2-14A	TraesCS3A02G111400	1516	74	1590	205	1795	15	69.6
T2-17B	TraesCS3B02G262600	1606	60	1666	0	1666	15	66.4
T2-23A	TraesCS7A02G495700	2705	0	2705	0	2705	15	68.5
T2-3B	TraesCS1B02G053600	1558	341	1899	318	2217	15	68.5
T2-33A	TraesCS2A02G123800	959	857	1816	323	2139	15	68.5
T2-37D	TraesCS1D02G427400	522	1292	1814	209	2023	15	76.2
T2-5B	TraesCS7B02G183300	1753	128	1881	299	2180	15	69.6
T2-53-7A	TraesCS7A02G070100	944	842	1786	230	2016	15	69.6
T2-56D	TraesCS2D02G290800	1561	277	1838	0	1838	15	71.8
T2-60B	TraesCS5B02G246400	926	291	1217	1141	2358	15	52.4
T4-15-6A	TraesCS6A02G083200	2033	152	2185	253	2438	15	76.2
T4-16B	TraesCS7B02G381100LC	1013	0	1013	735	1748	15	72.9
T4-18-7D	TraesCS7D02G009400	1977	63	2040	0	2040	15	78.4
T4-3B	TraesCS4B02G329500	1790	100	1890	102	1992	15	76.2
T4-30-4B	TraesCS4B02G378500	0	0	0	2411	2411	15	76.2
T4-37A	TraesCS7A02G049400	1825	70	1895	367	2262	15	75.1
T4-42A	TraesCS4A02G017000	2033	0	2033	433	2466	15	69.6
T4-44D	TraesCS5D02G464000	1589	0	1589	232	1821	15	69.6
T4-54D	TraesCS7D02G178000	2153	0	2153	155	2308	15	72.9
T4-58D	TraesCS5D02G564000	1911	194	2105	238	2343	15	76.2
T4-9B	TraesCS3B02G255300	1965	0	1965	91	2056	15	76.2
T5-12B	TraesCS1B02G024500	558	1046	1604	311	1915	15	68.6
T5-15B	TraesCS4B02G079300	1717	346	2063	171	2234	15	72.9
T5-16A	TraesCS5A02G481400	1940	112	2052	164	2216	15	76.2
T5-16(A)-B	TraesCS5B02G494500	1834	136	1970	421	2391	15	76.2
T5-20B	TraesCS4B02G112000	1391	158	1549	245	1794	15	78.4
T5-21D	TraesCS2D02G146100	2288	186	2474	226	2700	15	66.4
T8-14B	TraesCS4B02G287800	1669	216	1885	0	1885	15	68.5
T8-22B	TraesCS1B02G351600	1858	117	1975	201	2176	15	65.8
T8-26A	TraesCS3A02G018200	1984	97	2081	305	2386	15	71.8
T8-27A	TraesCS7A02G175200	1649	284	1933	430	2363	15	69.2
T8-7D	TraesCS6D02G069300	1065	578	1643	270	1913	15	72.9
T9-1A	TraesCS2A02G262700	943	853	1796	248	2044	15	72.9
T9-13D	TraesCS5D02G230200	1738	387	2125	179	2304	15	76.1
T9-14B	TraesCS4B02G207600	2212	0	2212	154	2366	15	50
T9-15A	TraesCS7A02G415300	1366	403	1769	311	2080	15	69.6
T9-16A	TraesCS5A02G401300	1819	0	1819	162	1981	15	69.6
T9-22A	TraesCS4A02G191300	0	0	0	2330	2330	15	68.9
T9-37A	TraesCS3A02G135400	0	194	194	1582	1776	15	72.9
T9-40A	TraesCS2A02G230100	2241	0	2241	127	2368	15	69.6
T9-41B	TraesCS2B02G326800	1673	374	2047	172	2219	15	75.1
T9-44A	TraesCS3A02G280200	2095	195	2290	185	2475	15	69.6
T9-46A	TraesCS5A02G597900LC	1894	0	1894	158	2052	15	66.4
T9-46B	TraesCS5B02G446100	1859	0	1859	0	1859	15	61.9
T9-5A	TraesCS2A02G386400	1902	133	2035	228	2263	15	72.9
T9-53B	TraesCS1B02G210700	1441	494	1935	278	2213	15	78.4
T9-54A	TraesCS7A02G141600	1940	68	2008	276	2284	15	65.2
T9-57B	TraesCS6B02G375400	1773	105	1878	0	1878	15	71.8
T9-57D	TraesCS6D02G324200	1728	107	1835	6	1841	15	72.9
T9-59A	TraesCS7A02G158000	1694	113	1807	211	2018	15	69.6
T9-6D	TraesCS2D02G335000	1923	247	2170	58	2228	15	71.8
T9-63A	TraesCS5A02G477600	1844	149	1993	312	2305	15	78.2
T9-68A	TraesCS2A02G551100	1724	100	1824	0	1824	15	76.2
T10-22A	TraesCS3A02G381500	2192	62	2254	137	2391	15	76.2
T10-25B	TraesCS4B02G096400	1492	0	1492	54	1546	15	63.8
T10-26A	TraesCS2A02G304900	712	1105	1817	190	2007	15	69.5
T10-29B	TraesCS7B02G103700	1956	0	1956	331	2287	15	58.6
T10-37A	TraesCS1A02G428700	0	0	0	2206	2206	15	76.2
T10-44B	TraesCS4B02G162200	0	0	0	2349	2349	15	78.2
T10-9D	TraesCS1D02G159000	0	0	0	2108	2108	15	72.9
T6-12D	TraesCS2D02G500600	1360	438	1798	262	2060	15	71.8
T6-13D	TraesCS6D02G383600LC	1777	31	1808	289	2097	15	76.2
T6-7D	TraesCS5D02G190800	2109	173	2282	0	2282	15	79.5
T6-9A	TraesCS1A02G065700	1071	117	1188	1281	2469	15	49.9
T1-10A	TraesCS2A02G295400	693	0	693	1687	2380	16	47.6
T1-13A	TraesCS5A02G030300	2108	372	2480	0	2480	16	73.4
T1-14A	TraesCS2A02G168600	2263	296	2559	0	2559	16	72.2
T1-14D	TraesCS2D02G176200	1604	307	1911	0	1911	16	70.1
T1-23A	TraesCS5A02G221600	1516	267	1783	161	1944	16	74
T1-23B	TraesCS5B02G220400	1318	344	1662	199	1861	16	74.6
T1-26D	TraesCS5D02G033700	0	0	0	2218	2218	16	72.2
T1-28-2B	TraesCS2B02G566500	1726	56	1782	306	2088	16	66.8
T1-6-1B	TraesCS1B02G479100	276	768	1044	250	1294	16	68.3
T1-8-7A	TraesCS7A02G017700	1687	329	2016	0	2016	16	76.7
T2-14B	TraesCS3B02G132400	1792	155	1947	280	2227	16	70.1
T2-30D	TraesCS3D02G095700	2074	0	2074	268	2342	16	75.5
T2-40A	TraesCS1A02G466500LC	1790	0	1790	318	2108	16	83.3
T2-42D	TraesCS7D02G004100	1567	0	1567	213	1780	16	65.6
T2-9B	TraesCS3B02G047300	2163	270	2433	243	2676	16	72.2
T4-2D	TraesCS6D02G403200	0	0	0	1748	1748	16	82.6
T4-3D	TraesCS4D02G326400	1700	140	1840	290	2130	16	73.4
T4-36A	TraesCS3A02G236700	1394	202	1596	0	1596	16	80.3
T4-37D	TraesCS7D02G044500	1968	47	2015	452	2467	16	70.1
T4-46D	TraesCS5D02G233700	1677	83	1760	792	2552	16	76.7
T4-48A	TraesCS2A02G136800	1878	50	1928	314	2242	16	76.7
T4-48B	TraesCS2B02G160700	2022	0	2022	0	2022	16	76.7
T4-49B	TraesCS1B02G351200	1896	0	1896	232	2128	16	69.1
T4-50A	TraesCS5A02G261400	2074	0	2074	0	2074	16	83.3
T4-52A	TraesCS7A02G478400	1855	0	1855	0	1855	16	73.4
T4-56D	TraesCS1D02G047900	1428	322	1750	0	1750	16	65.6
T4-57B	TraesCS3B02G238500	1530	0	1530	175	1705	16	70.1
T5-13B	TraesCS7B02G083700	555	1263	1818	243	2061	16	78.8
T5-15A	TraesCS4A02G235600	1511	312	1823	99	1922	16	73.4
T5-19B	TraesCS2B02G453700	1938	0	1938	81	2019	16	76.7
T5-25B	TraesCS2B02G294500	1614	335	1949	141	2090	16	78.8
T5-28A	TraesCS3A02G021800	1776	184	1960	22	1982	16	80
T8-1B	TraesCS3B02G217100	1781	146	1927	216	2143	16	73.4
T8-10B	TraesCS2B02G281000	1824	91	1915	0	1915	16	76.7
T8-12D	TraesCS2D02G442200	2002	193	2195	224	2419	16	70.1
T8-25B	TraesCS2B02G067100	1881	0	1881	351	2232	16	73.4
T8-3A	TraesCS5A02G233600	2037	96	2133	256	2389	16	52.1
T9-23B	TraesCS4B02G043100	1744	115	1859	179	2038	16	70.1
T9-49A	TraesCS5A02G382600	1768	258	2026	1330	3356	16	70.1
T9-50B	TraesCS5B02G075800	2000	131	2131	6	2137	16	73.4
T10-11D	TraesCS4D02G044700	857	101	958	304	1262	16	85.4
T10-14D	TraesCS7D02G233900	942	904	1846	373	2219	16	68.2
T10-26B	TraesCS2B02G321700	641	1070	1711	2169	2169	16	80.1
T10-26D	TraesCS2D02G303500	0	393	393	2131	2524	16	78.8
T10-31A	TraesCS5A02G352800	1686	0	1686	262	1948	16	68.8
T6-10B	TraesCS6B02G024900	1481	209	1690	333	2023	16	76.8
T6-11D	TraesCS7D02G073700	2212	0	2212	0	2212	16	72.2
T6-16A	TraesCS6A02G031200	2115	96	2211	198	2409	16	73.4
T6-16D	TraesCS6D02G035600	1915	72	1987	188	2175	16	80
T6-18B	TraesCS6B02G044500	1777	0	1777	397	2174	16	70.1
T1-16D	TraesCS1D02G353100	1509	156	1665	1099	2764	17	49.7
T1-7D	TraesCS4D02G290900	1730	140	1870	263	2133	17	80.5
T1-8-4A	TraesCS4A02G474000	1728	342	2070	129	2199	17	76
T2-1B	TraesCS4B02G197000	1604	255	1859	122	1981	17	70.6
T2-1D	TraesCS4D02G197300	1727	254	1981	48	2029	17	70.6
T2-12D	TraesCS7D02G430700	1721	107	1828	0	1828	17	72.7
T2-20B	TraesCS3B02G556500	1900	42	1942	231	2173	17	83.8
T2-28B	TraesCS3B02G111100	2297	0	2297	475	2772	17	85.9
T2-32B	TraesCS7B02G072000	1611	0	1611	492	2103	17	71.4
T2-34B	TraesCS4B02G328100	1583	0	1583	251	1834	17	70.6
T2-36B	TraesCS5B02G003500	1901	0	1901	239	2140	17	76
T2-51B	TraesCS2B02G491700	1878	240	2118	0	2118	17	76
T2-54B	TraesCS2B02G390700	1371	16	1387	217	1604	17	70.6
T2-55A	TraesCS7A02G549300	1905	141	2046	0	2046	17	76
T2-60D	TraesCS5D02G255800	1750	287	2037	0	2037	17	72.7
T4-15-6B	TraesCS6B02G113900	2337	66	2403	384	2787	17	82.6
T4-20-7D	TraesCS7D02G008800	1734	190	1924	280	2204	17	82.6
T4-34A	TraesCS4A02G296300	1934	0	1934	260	2194	17	66.1
T4-38A	TraesCS3A02G125200	1761	35	1796	144	1940	17	73.9
T4-4A	TraesCS3A02G049500	1050	0	1050	138	1188	17	76
T4-4B	TraesCS3B02G056100	1900	0	1900	111	2011	17	77.2
T4-46A	TraesCS5A02G226400	1815	46	1861	368	2229	17	82.6
T4-57A	TraesCS3A02G206400	1200	622	1822	180	2002	17	77.2
T5-2A	TraesCS3A02G099200	1857	192	2049	0	2049	17	72.7
T5-23A	TraesCS5A02G384100	1788	133	1921	0	1921	17	77.2
T8-12B	TraesCS2B02G464200	1883	64	1947	232	2179	17	73.9
T8-14D	TraesCS4D02G286500	1508	443	1951	139	2090	17	80.5
T8-24D	TraesCS2D02G057300	1432	0	1432	1039	2471	17	79.3
T8-3B	TraesCS5B02G232100	1974	7	1981	85	2066	17	77
T9-10D	TraesCS7D02G478800	2369	104	2473	289	2762	17	79.3
T9-12D	TraesCS1D02G277600	2047	0	2047	0	2047	17	72.7
T9-18B	TraesCS6B02G303600	1683	31	1714	0	1714	17	89.9
T9-27A	TraesCS4A02G039000	0	1834	1834	315	2149	17	82.6
T9-27D	TraesCS4D02G266400	0	1897	1897	267	2164	17	76
T9-28D	TraesCS4D02G312800	1767	0	1767	323	2090	17	77.2
T9-34B	TraesCS1B02G265400	1777	82	1859	208	2067	17	80.5
T9-48A	TraesCS2A02G491000	798	1076	1874	57	1931	17	76
T9-48D	TraesCS2D02G491200	444	1291	1735	288	2023	17	79.3
T9-49B	TraesCS5B02G386800	365	308	673	1286	1959	17	72.7
T9-5D	TraesCS2D02G383200	2146	0	2146	171	2317	17	77.2
T9-67B	TraesCS1B02G314700	1583	370	1953	779	2732	17	75.4
T9-7D	TraesCS2D02G421700	1878	51	1929	129	2058	17	83.8
T9-76B	TraesCS7B02G264800	1798	134	1932	338	2270	17	79.3
T9-76D	TraesCS7D02G359900	1905	129	2034	299	2333	17	79.3
T9-77B	TraesCS7B02G120800	1715	0	1715	0	1715	17	70.6
T10-1D	TraesCS4D02G199200	1796	100	1896	116	2012	17	77.2
T10-10D	TraesCS3D02G125600	2315	147	2462	130	2592	17	77.2
T10-20A	TraesCS5A02G133000	1677	188	1865	50	1915	17	70.6
T10-42D	TraesCS5D02G050300	897	963	1860	298	2158	17	80.5
T10-8D	TraesCS2D02G531600	1411	544	1955	0	1955	17	80.5
T6-16B	TraesCS6B02G044000	2045	0	2045	198	2243	17	80.5
T6-17B	TraesCS6B02G044200	1995	167	2162	424	2586	17	82.6
T1-10D	TraesCS2D02G293200	962	0	962	1499	2461	18	57.7
T2-1A	TraesCS4A02G107400	1585	277	1862	92	1954	18	74.4
T2-10D	TraesCS1D02G358500	540	994	1534	1392	2926	18	56
T2-2A	TraesCS5A02G247200	1514	466	1980	356	2336	18	80.9
T2-35A	TraesCS5A02G472000	1931	131	2062	113	2175	18	80.9
T2-48D	TraesCS1D02G000100	1030	605	1635	528	2163	18	73.2
T2-51A	TraesCS2A02G468800	1991	158	2149	296	2445	18	86.4
T4-1A	TraesCS7A02G264400	1986	0	1986	79	2065	18	74.4
T4-12B	TraesCS7B02G253500	1987	0	1987	401	2388	18	77.6
T4-17A	TraesCS1A02G131600	1477	0	1477	1818	3295	18	50.4
T4-18-4A	TraesCS4A02G485400	1869	236	2105	75	2180	18	84.2
T4-20-7A	TraesCS7A02G009200	1869	0	1869	270	2139	18	79.8
T4-25A	TraesCS4A02G097500	1829	152	1981	165	2146	18	77.1
T4-25D	TraesCS4D02G207800	1641	140	1781	175	1956	18	74.4
T4-29A	TraesCS7A02G548000	1853	74	1927	56	1983	18	77.6
T4-32B	TraesCS7B02G418400	2042	130	2172	131	2303	18	77.6
T4-47D	TraesCS3D02G129900	1833	337	2170	135	2305	18	86.4
T4-55D	TraesCS2D02G596300	2054	0	2054	340	2394	18	83.1
T4-7B	TraesCS7B02G182300	0	0	0	2370	2370	18	79.8
T5-12A	TraesCS1A02G020500	836	1060	1896	229	2125	18	73.2
T5-12D	TraesCS1D02G020000	583	1163	1746	371	2117	18	76.5
T5-21A	TraesCS2A02G142800	2066	205	2271	0	2271	18	76.5
T5-23D	TraesCS5D02G393600	1915	0	1915	0	1915	18	80.9
T5-27A	TraesCS7A02G235700	1839	229	2068	294	2362	18	83.1
T5-27D	TraesCS7D02G235700	1621	333	1954	235	2189	18	83.1
T5-8A	TraesCS5A02G382800	1817	155	1972	264	2236	18	84.2
T5-8D	TraesCS2D02G387100	1891	144	2035	268	2303	18	86.8
T8-12A	TraesCS2A02G443100	2299	85	2384	274	2658	18	77.6
T8-19D	TraesCS3D02G093500	869	1050	1919	169	2088	18	80.9
T8-21D	TraesCS5D02G486600	1746	123	1869	180	2049	18	74.4
T8-22D	TraesCS1D02G341100	1936	116	2052	1066	3118	18	54.8
T9-10B	TraesCS7B02G398100	1881	350	2231	376	2607	18	89.6
T9-12B	TraesCS1B02G287300	1904	0	1904	202	2106	18	79.8
T9-20B	TraesCS2B02G214600	1961	0	1961	0	1961	18	84.2
T9-30D	TraesCS5D02G370200	1936	0	1936	165	2101	18	77.6
T9-32D	TraesCS6D02G205300	1743	212	1955	338	2293	18	80.9
T9-34A	TraesCS1A02G379700	1656	224	1880	58	1938	18	79.8
T9-40D	TraesCS2D02G233800	1665	476	2141	0	2141	18	80.9
T9-43D	TraesCS2D02G259200	1613	87	1700	197	1897	18	74.4
T9-48B	TraesCS2B02G519200	1387	549	1936	211	2147	18	86.4
T9-67D	TraesCS1D02G303700	1605	349	1954	578	2532	18	68.8
T9-72B	TraesCS4B02G375600	2001	61	2062	268	2330	18	77.6
T9-77A	TraesCS7A02G213600	1471	296	1467	265	1732	18	77.6
T10-20D	TraesCS5D02G141200	1869	202	2071	37	2108	18	76.5
T10-21A	TraesCS6A02G199100	1538	190	1728	163	1891	18	83.1
T10-32D	TraesCS6D02G182400	0	1802	1802	965	2767	18	87.4
T10-48B	TraesCS5B02G165300	484	1357	1841	1612	3453	18	51.6
T10-8B	TraesCS2B02G559100	1535	542	2077	297	2374	18	83.1
T6-11A	TraesCS4A02G376700	1594	0	1594	512	2106	18	76.5
T6-14D	TraesCS6D02G315300	1616	345	1961	20	1981	18	84.2
T6-18D	TraesCS6D02G035900	2681	0	2681	320	3001	18	86.4
T1-4A	TraesCS3A02G311100	1665	335	2000	281	2281	19	83.5
T2-13A	TraesCS2A02G220400	1868	105	1973	396	2369	19	84.7
T2-20D	TraesCS3D02G501900	2082	0	2082	80	2162	19	86.8
T2-27A	TraesCS5A02G395200	1852	97	1949	46	1995	19	83.5
T2-34D	TraesCS4D02G325100	1749	0	1749	426	2175	19	81.4
T2-35B	TraesCS5B02G484700	1939	103	2042	133	2175	19	81.4
T2-4B	TraesCS3B02G474900	1313	391	1704	1047	2751	19	69.6
T2-46B	TraesCS7B02G746900LC	2086	0	2086	100	2186	19	88
T2-9D	TraesCS3D02G043500	3216	1614	4830	150	4980	19	90.1
T4-2A	TraesCS6A02G417900	0	0	0	2316	2316	19	90.6
T4-39B	TraesCS2B02G282400	405	83	488	2502	2990	19	49.2
T4-44A	TraesCS5A02G453200	1921	0	1921	0	1921	19	88
T4-48D	TraesCS2D02G139500	1309	596	1905	287	2192	19	86.8
T4-9A	TraesCS3A02G347800LC	1919	0	1919	327	2246	19	86.8
T5-5D	TraesCS7D02G166100	1695	405	2100	81	2181	19	76.9
T8-14A	TraesCS4A02G016400	0	0	0	2545	2545	19	86.8
T8-24A	TraesCS2A02G058000	0	1022	1022	1033	2055	19	81.4
T8-24B	TraesCS2B02G071100	1680	128	1808	840	2648	19	50.7
T8-25A	TraesCS2A02G053200	1716	85	1801	215	2016	19	81.4
T9-22B	TraesCS4B02G124000	1969	151	2120	186	2306	19	81.4
T9-23A	TraesCS4A02G271000	2013	0	2013	0	2013	19	76.9
T9-31B	TraesCS4B02G078800	1682	294	1976	117	2093	19	86.8
T9-43A	TraesCS2A02G264800	1878	170	2048	218	2266	19	81.4
T9-5B	TraesCS2B02G403800	2107	0	2107	160	2267	19	84.7
T9-66D	TraesCS7D02G371900	1638	305	1943	175	2118	19	80.2
T10-25A	TraesCS4A02G217700	1889	33	1922	471	2393	19	84.1
T10-3A	TraesCS7A02G410300	1952	0	1952	336	2288	19	84.7
T10-3B	TraesCS7B02G309700	1950	0	1950	270	2220	19	79.8
T10-32B	TraesCS6B02G220900	1156	771	1927	169	2096	19	81.4
T10-42B	TraesCS5B02G045200	981	860	1841	422	2263	19	86.8
T6-14A	TraesCS6A02G335400	1636	138	1774	270	2044	19	81.4
T6-14B	TraesCS6B02G366100	1571	131	1702	44	1746	19	81.4
T6-15A	TraesCS6A02G031100	1745	221	1966	224	2190	19	81.4
T6-2D	TraesCS7D02G158300	628	1225	1853	391	2244	19	81.4
T6-7A	TraesCS5A02G185700	1860	140	2000	158	2158	19	84.7
T1-7A	TraesCS4A02G012100	1560	259	1819	323	2142	20	84
T2-27B	TraesCS5B02G400000	0	0	0	2160	2160	20	85.2
T2-27D	TraesCS5D02G404500	1892	141	2033	92	2125	20	80.7
T2-48B	TraesCS1B02G012400	1388	556	1944	352	2296	20	87.3
T4-2B	TraesCS6B02G471100	1189	0	1189	983	2172	20	81.9
T4-29B	TraesCS7B02G471500	1813	70	1883	218	2101	20	87.3
T4-33-4A	TraesCS4A02G384800	1748	48	1796	310	2106	20	77.4
T4-45D	TraesCS7D02G524300	1909	0	1909	0	1909	20	85.2
T4-47B	TraesCS3B02G148100	2117	22	2139	127	2266	20	47.8
T4-51B	TraesCS1B02G117400	1880	96	1976	295	2271	20	87.3
T4-52B	TraesCS7B02G381400	2441	35	2476	0	2476	20	84
T4-57D	TraesCS3D02G209200	1315	653	1968	308	2276	20	84
T5-22B	TraesCS3B02G055700LC	1900	0	1900	401	2301	20	93.9
T9-1D	TraesCS2D02G261300	1208	745	1953	223	2176	20	84
T9-10A	TraesCS7A02G492400	1336	654	1990	304	2294	20	94.4
T9-29D	TraesCS5D02G148000	1813	231	2044	361	2405	20	85.2
T9-30A	TraesCS5A02G361300	1882	397	2279	0	2279	20	85.2
T9-45D	TraesCS7D02G330200	2046	0	2046	0	2046	20	90.6
T9-65B	TraesCS2B02G117900	1727	88	1815	85	1900	20	88.5
T9-74B	TraesCS5B02G156400	2825	96	2921	213	3134	20	80.7
T10-34A	TraesCS3A02G401700	1650	97	1747	246	1993	20	87.8
T10-39D	TraesCS4D02G147700	1733	187	1920	1045	2965	20	66.6
T10-46D	TraesCS2D02G337000	1892	117	2009	369	2378	20	88.5
T6-2A	TraesCS7A02G157700	2047	0	2047	351	2398	20	85.2
T2-2D	TraesCS5D02G253700	1410	469	1879	432	2311	21	88.9
T2-20A	TraesCS3A02G494800	1844	10	1854	420	2274	21	95.5
T2-22D	TraesCS2D02G124900	1831	166	1997	1038	3035	21	70.7
T2-48B	TraesCS1B02G398500	1376	556	1932	376	2308	21	91.1
T2-52A	TraesCS1A02G353300	0	1970	1970	318	2288	21	88.9
T2-53-4A	TraesCS4A02G418200	1239	728	1967	275	2242	21	91.1
T2-53-7D	TraesCS7D02G064300	1315	865	2180	253	2433	21	91.1
T2-54A	TraesCS2A02G373600	0	1652	1652	458	2110	21	81.2
T2-55B	TraesCS7B02G472300	1876	108	1984	0	1984	21	88.9
T2-58B	TraesCS7B02G139700	1958	0	1958	0	1958	21	81.2
T4-34D	TraesCS4D02G015800	1662	44	1706	212	1918	21	91.1
T4-45A	TraesCS7A02G537200	1573	312	1885	0	1885	21	88.9
T4-49A	TraesCS1A02G338700	1913	98	2011	261	2272	21	85.6
T8-1A	TraesCS3A02G187800	1833	142	1975	235	2210	21	91.1
T8-1D	TraesCS3D02G191400	1872	131	2003	340	2343	21	87.8
T9-12A	TraesCS1A02G278400	2469	0	2469	0	2469	21	87.8
T9-27B	TraesCS4B02G266700	0	2086	2086	312	2398	21	94.8
T9-3D	TraesCS6D02G299600	1900	0	1900	208	2108	21	87.8
T9-31A	TraesCS4A02G236200	1732	330	2062	53	2115	21	88.9
T9-45B	TraesCS5B02G480900	2024	84	2108	0	2108	21	87.8
T9-65D	TraesCS2D02G100200	2005	83	2088	100	2188	21	82.4
T10-15B	TraesCS4B02G229600LC	853	0	853	1311	2164	21	87.8
T10-34B	TraesCS3B02G434600	1796	89	1885	196	2081	21	85.6
T6-31B	TraesCS4B02G317200	2019	50	2069	221	2290	21	92.2
T1-16A	TraesCS1A02G350200	1178	122	1300	1029	2329	22	67.8
T1-17B	TraesCS2B02G202300	1546	393	1939	1802	3741	22	54.8
T2-55D	TraesCS7D02G535600	2022	72	2094	0	2094	22	88.2
T4-12D	TraesCS7D02G349600	1930	0	1930	197	2127	22	91.5
T4-17B	TraesCS1B02G154400	1494	202	1696	1804	3500	22	55.7
T4-17D	TraesCS1D02G136400	1838	102	1940	145	2085	22	89.4
T4-28-5B	TraesCS5B02G544800	1735	42	1777	273	2050	22	89.4
T4-4D	TraesCS3D02G049300	1450	0	1450	219	1669	22	89.4
T8-25D	TraesCS2D02G053400	1871	74	1945	226	2171	22	92.7
T9-45B	TraesCS1B02G314700	1582	381	1883	787	2670	22	94.8
T10-22D	TraesCS3D02G374700	1999	39	2038	0	2038	22	89.4
T10-4	TraesCS2A02G275100	0	0	0	1969	1969	22	94.8
T6-8B	TraesCS5B02G183700	1872	134	2006	290	2296	22	86.1
T2-13B	TraesCS2B02G245900	1760	99	1859	175	2034	23	89.9
T2-25B	TraesCS7B02G034600	1967	8	1975	367	2342	23	99.1
T2-26B	TraesCS6B02G008200	1857	0	1857	351	2208	23	92.4
T2-37B	TraesCS1B02G449700	531	1258	1789	1201	2990	23	72.2
T8-15A	TraesCS1A02G350500	250	80	330	2157	2487	23	50.5
T9-24A	TraesCS4A02G312500	1923	38	1961	271	2232	23	93.2
T9-30B	TraesCS5B02G363900	1464	176	1640	836	2476	23	66.5
T1-17D	TraesCS2D02G183400	1566	398	1964	1811	3775	24	62.6
T2-13D	TraesCS2D02G226000	1831	178	2009	231	2240	24	93.6
T2-25A	TraesCS7A02G133500	2100	68	2168	302	2470	24	99.1
T2-4A	TraesCS3A02G440800	1871	0	1871	1276	3147	24	78.2
T2-4D	TraesCS3D02G433400	1547	321	1868	1085	2953	24	80.7
T2-56A	TraesCS2A02G293400	1795	272	2067	1111	3178	24	71.4
T4-18-7A	TraesCS7A02G009800	0	1939	1939	99	2038	24	92.5
T5-19A	TraesCS2A02G432400	2049	0	2049	296	2345	24	95.8
T9-29B	TraesCS5B02G143100	2061	63	2124	117	2241	24	92.5
T9-44B	TraesCS3B02G314000	1762	242	2004	1389	3393	24	57.5
T6-18A	TraesCS6A02G030700	1990	0	1990	296	2286	24	95.8
T2-25D	TraesCS7D02G133100	1854	0	1854	361	2215	25	99.5
T4-8B	TraesCS2B02G433400	394	135	529	2001	2530	25	53.7
T5-3D	TraesCS1D02G223600	1041	127	1168	2320	3488	25	58
T9-43B	TraesCS2B02G277300	1667	33	1700	365	2065	25	96.2
T6-31A(2)	TraesCS4A02G416300	2074	0	2074	317	2391	25	99.5
T2-26D	TraesCS6D02G000200	2013	0	2013	482	2495	26	100
T2-54D	TraesCS2D02G369800	0	1791	1791	802	2593	26	85.4
T8-15D	TraesCS1D02G353300	152	292	444	2086	2530	26	60.4
T9-24D	TraesCS4D02G001700	2038	49	2087	218	2305	26	100
T4-23B	TraesCS4B02G100100	1609	166	1775	2337	4112	27	72.2
T4-39A	TraesCS2A02G259500	1647	86	1733	2439	4172	27	70.6
T4-58B	TraesCS5B02G572600	1463	341	1804	1697	3501	27	67.5
T9-44D	TraesCS3D02G279900	1461	303	1764	1750	3514	27	65.6
T5-24A	TraesCS4A02G177600	1060	207	1267	1860	3127	28	65.3
T8-10D	TraesCS2D02G262700	1501	132	1633	2070	3703	28	60.4
T5-3A	TraesCS1A02G221900	1291	131	1422	2383	3805	29	66.1
T9-7A	TraesCS2A02G423900	1893	287	2180	265	2445	29	75.6
T9-7B	TraesCS2B02G443500	1394	406	1800	1153	2953	29	67.7
T9-11B	TraesCS1B02G247700	1727	121	1848	2366	4214	32	71.2
T9-49D	TraesCS5D02G392000	1461	322	1783	258	2041	32	76.4
T4-30-5A	TraesCS5A02G545100	1982	95	2077	2459	4536	33	75.3
T5-24B	TraesCS4B02G140200	1828	364	2192	1811	4003	35	76.1
T8-10A	TraesCS2A02G261200	1561	402	1963	2577	4540	35	75.2
T9-11A	TraesCS1A02G236100	1791	115	1906	2444	4350	35	83.8
T2-51D	TraesCS2D02G468900	3240	128	3368	66	3434	37	85.4
T9-11D	TraesCS1D02G236200	1166	154	1320	2401	3721	42	84.2
T2-22A	TraesCS2A02G122200	1730	0	1730	3016	4746	45	85.3

Trait	gene ID	CS	KR	MDR031	ENT	ASP	APG	normalised by
T1-1	TraesCS6A02G189300	52	60	204	0	4	4	
T1-1	TraesCS6B02G215300	55	59	4	0	167	1	expected ratio
T1-1	TraesCS6D02G176900	54	1	36	131	1	119	KR 1.5 (6/4)
T1-10	TraesCS2A02G295400	57	57	213	0	10	25	exclude if <0.7
T1-10	TraesCS2B02G311900	49	48	0	0	130	23	MDR 3 (6/2)
T1-10	TraesCS2D02G293200	40	0	11	115	51	80	exclude if <1.5
T1-11	TraesCS5A02G373900	32	47	170	0	13	23	ENT 3 (6/2)
T1-11	TraesCS5B02G375800	50	47	7	7	90	59	exclude if <1.5
T1-11	TraesCS5D02G383300	42	2	0	104	0	4	ASP 3 (6/2)
T1-12	TraesCS1A02G088000	31	50	152	2	5	6	exclude if <1.5
T1-12	TraesCS1B02G107000	31	38	4	0	84	3	APG 1.5 (6/4)
T1-12	TraesCS1D02G089300	44	0	24	89	13	35	exclude if <0.7
T1-13	TraesCS5A02G030300	70	99	302	0	1	8	
T1-13	TraesCS5B02G029100	68	62	55	0	78	76	
T1-14	TraesCS2A02G168600	58	96	255	0	2	4	
T1-14	TraesCS2B02G194900	72	92	93	0	210	49	
T1-14	TraesCS2D02G176200	76	0	65	206	32	75	
T1-15	TraesCS3A02G194100	69	32	168	1	6	11	
T1-15	TraesCS3B02G223900	38	37	20	0	99	21	
T1-15	TraesCS3D02G197700	89	13	318	236	193	142	
T1-16	TraesCS1A02G350200	62	64	191	0	34	26	
T1-16	TraesCS1B02G364500	63	73	0	0	24	10	
T1-16	TraesCS1D02G353100	30	1	0	96	27	23	
T1-17	TraesCS2A02G176000	59	86	268	0	28	40	
T1-17	TraesCS2B02G202300	72	93	10	0	259	100	
T1-17	TraesCS2D02G183400	80	0	26	188	15	81	
T1-18	TraesCS5B02G379300	41	36	4	0	57	28	
T1-18	TraesCS5D02G385900	39	0	33	132	10	6	
T1-19	TraesCS4A02G016300	84	98	279	3	57	91	
T1-19	TraesCS4D02G286600	69	8	38	171	87	81	
T1-19	TraesCS4B02G287900	75	93	63	5	182	69	
T1-2	TraesCS3A02G212900LC	22	27	36	4	9	9	
T1-2	TraesCS3B02G433000	33	30	0	0	43	9	
T1-2	TraesCS3D02G172900	15	12	19	24	19	30	
T1-20	TraesCS1A02G083000	26	40	97	0	2	3	
T1-20	TraesCS1B02G100400	39	34	1	1	40	16	
T1-20	TraesCS1D02G084200	23	0	1	62	8	39	
T1-21	TraesCS6A02G306200	49	47	172	0	51	21	
T1-21	TraesCS6D02G285300	43	0	17	110	45	26	
T1-22	TraesCS7A02G185300	20	28	45	0	0	7	
T1-22	TraesCS7B02G090500	6	11	2	0	2	2	
T1-22	TraesCS7D02G187000	29	0	18	93	31	31	
T1-23	TraesCS5A02G221600	53	55	136	0	30	23	
T1-23	TraesCS5B02G220400	56	53	50	0	41	17	
T1-23	TraesCS5D02G229400	45	0	10	88	29	29	
T1-24	TraesCS6A02G237700	36	53	120	1	1	18	
T1-24	TraesCS6B02G266200	25	31	0	4	66	19	
T1-25	TraesCS1A02G102400	33	36	194	4	5	21	
T1-25	TraesCS1B02G113100	43	0	0	1	148	13	
T1-25	TraesCS1D02G094200	24	2	9	74	5	19	
T1-26	TraesCS5A02G025900	61	75	208	0	35	31	
T1-26	TraesCS5B02G025100	54	70	70	2	108	49	
T1-26	TraesCS5D02G033700	68	4	36	192	180	135	
T1-27	TraesCS6A02G378500	50	64	208	0	27	14	
T1-27	TraesCS6B02G416400	36	57	52	18	34	24	
T1-28	TraesCS2B02G566500	58	67	0	0	0	0	
T1-28	TraesCS3A02G021900	43	94	378	0	0	36	
T1-28	TraesCS3D02G024700	34	7	33	90	0	29	
T1-3 & T6-41	TraesCS5A02G215100	30	36	79	0	1	3	
T1-3 & T6-41	TraesCS5B02G208700	35	30	0	1	78	6	
T1-3 & T6-41	TraesCS5D02G216900	29	0	2	86	4	27	
T1-4	TraesCS3A02G311100	52	61	209	0	0	0	
T1-4	TraesCS3B02G161000	34	43	0	0	123	10	
T1-4	TraesCS3D02G143300	59	0	0	148	2	52	
T1-4B	TraesCS3D02G143500	40	0	0	77	8	56	
T1-5	TraesCS2A02G188500	37	48	136	0	1	29	
T1-5 & T8-17	TraesCS2B02G405700	43	23	0	0	97	11	
T1-5	TraesCS2D02G385500	23	0	0	31	0	15	
T1-6	TraesCS1B02G479100	65	79	0	0	139	0	
T1-6	TraesCS7A02G000200	36	30	72	0	0	15	
T1-6	TraesCS7D02G000100	59	1	43	129	1	10	
T1-7	TraesCS4A02G012100	39	41	72	0	13	7	
T1-7	TraesCS4B02G292300	39	37	0	0	55	18	
T1-7	TraesCS4D02G290900	32	0	0	95	21	37	
T1-8	TraesCS4A02G474000	63	78	36	0	126	43	
T1-8	TraesCS7A02G017700	78	36	268	0	61	58	
T1-8	TraesCS7D02G015000	79	59	48	186	91	46	
T1-9	TraesCS7A02G260500	41	46	140	2	1	31	
T1-9	TraesCS7B02G158500	44	50	21	0	107	48	
T1-9	TraesCS7D02G261500	54	0	12	145	23	53	
T2-1	TraesCS4A02G107400	82	99	300	0	26	2	
T2-1	TraesCS4B02G197000	83	90	0	0	197	0	
T2-1	TraesCS4D02G197300	65	0	0	194	4	132	
T2-10	TraesCS1A02G351900	25	27	66	0	15	26	
T2-10	TraesCS1B02G371300	51	35	27	0	44	63	
T2-10	TraesCS1D02G358500	39	2	15	92	27	25	
T2-11	TraesCS7A02G287500	50	47	177	3	16	16	
T2-12	TraesCS7A02G441400	45	47	118	0	25	26	
T2-12	TraesCS7D02G430700	44	2	12	86	9	13	
T2-13 & T3-25	TraesCS2A02G220400	59	74	254	0	0	20	
T2-13 & T3-25	TraesCS2B02G245900	59	72	95	0	108	22	
T2-13 & T3-25	TraesCS2D02G226000	74	0	62	177	0	121	
T2-14	TraesCS3A02G111400	66	65	197	0	18	14	
T2-14	TraesCS3B02G132400	71	100	1	0	83	11	
T2-14	TraesCS3D02G113600	10	0	0	42	17	11	
T2-15	TraesCS2A02G436100	22	26	56	0	1	4	
T2-15	TraesCS2B02G457100	22	27	1	0	5	17	
T2-16	TraesCS3A02G220800	25	20	35	0	0	0	
T2-16	TraesCS3B02G251100	45	50	0	0	88	0	
T2-16	TraesCS3D02G231700	45	50	0	0	88	0	
T2-17	TraesCS3B02G262600	72	81	28	0	81	44	
T2-17	TraesCS3D02G219900	22	0	1	73	0	10	
T2-18	TraesCS7A02G236200	60	68	33	0	0	10	
T2-18	TraesCS7B02G134200	48	57	14	0	45	10	
T2-18	TraesCS7D02G236300	37	27	45	90	31	54	
T2-19	TraesCS6A02G178000	40	42	20	3	2	12	
T2-19	TraesCS6B02G206800	17	15	6	7	21	10	
T2-19	TraesCS6D02G166400	42	13	24	60	12	10	
T2-2 & T3-13	TraesCS5A02G247200	37	38	116	0	4	6	
T2-2 & T3-13	TraesCS5B02G244300	35	46	7	1	39	16	
T2-2 & T3-13	TraesCS5D02G253700	42	0	3	107	8	44	
T2-20	TraesCS3A02G494800	47	63	136	0	18	26	
T2-20	TraesCS3B02G556500	57	39	15	0	102	48	
T2-20	TraesCS3D02G501900	62	5	6	170	42	72	
T2-21	TraesCS1A02G115500	54	57	122	0	36	2	
T2-21	TraesCS1D02G116900	53	0	53	177	48	56	
T2-22	TraesCS2A02G122200	52	63	233	0	100	55	
T2-22	TraesCS2B02G144300	53	62	3	0	124	31	
T2-22	TraesCS2D02G124900	56	1	17	140	88	80	
T2-23	TraesCS7A02G495700	51	69	206	0	3	21	
T2-23	TraesCS7B02G401600	60	67	113	0	135	15	
T2-23	TraesCS7D02G483000	66	1	66	159	71	25	
T2-24	TraesCS7A02G235500	39	42	84	0	0	14	
T2-24	TraesCS7B02G133900	37	37	2	0	61	8	
T2-24	TraesCS7D02G235600	26	0	14	58	8	28	
T2-25	TraesCS7A02G133500	67	81	325	0	1	68	
T2-25	TraesCS7B02G034600	69	74	0	0	230	19	
T2-25	TraesCS7D02G133100	62	0	1	188	3	35	
T2-26	TraesCS6B02G008200	0	0	0	0	66	63	
T2-26	TraesCS6D02G000200	129	0	167	221	34	168	
T2-27 & T3-27	TraesCS5A02G395200	38	52	167	2	54	40	
T2-27 & T3-27	TraesCS5B02G400000	66	81	65	0	214	19	
T2-27 & T3-27	TraesCS5D02G404500	45	1	32	150	67	129	
T2-28	TraesCS3B02G111100	63	88	0	0	152	123	
T2-28	TraesCS3D02G096000	87	0	2	159	19	95	
T2-29	TraesCS3A02G366400	69	64	202	1	95	31	
T2-29 & T3-26	TraesCS3B02G397900	68	58	3	0	147	50	
T2-29 & T3-26	TraesCS3D02G359200	50	0	128	123	138	51	
T2-3 & T3-12	TraesCS1A02G040600	13	15	38	0	1	0	
T2-3 & T3-12	TraesCS1B02G053600	30	3	1	0	30	17	
T2-3 & T3-12	TraesCS1D02G041200	34	5	2	70	20	12	
T2-30	TraesCS3D02G095700	66	0	0	186	1	63	
T2-31	TraesCS2A02G159300	79	71	221	5	5	15	
T2-31	TraesCS2B02G184900	64	64	1	19	192	58	
T2-31	TraesCS2D02G166600	39	0	86	106	0	60	
T2-32	TraesCS7B02G072000	63	79	181	1	129	39	
T2-32	TraesCS7D02G168000	59	1	15	133	44	70	
T2-33	TraesCS2A02G123800	27	39	101	0	10	20	
T2-33	TraesCS2B02G145700	27	34	0	0	44	4	
T2-33	TraesCS2D02G126600	39	0	0	97	19	23	
T2-34	TraesCS4B02G328100	87	114	0	0	101	49	
T2-34	TraesCS4D02G325100	53	0	1	131	79	72	
T2-35 & T3-23	TraesCS5A02G472000	70	85	260	0	15	4	
T2-35 7 T3-23	TraesCS5B02G484700	63	63	0	0	2	0	
T2-35 & T3-23	TraesCS5D02G484500	33	0	4	120	50	39	
T2-36	TraesCS5B02G003500	66	87	3	0	110	53	
T2-37 & T3-16	TraesCS1A02G419600	33	49	100	2	70	56	
T2-37&T3-16	TraesCS1B02G449700	45	50	34	0	94	33	
T2-37 & T3-16	TraesCS1D02G427400	44	6	69	100	77	19	
T2-39 & T3-24	TraesCS5A02G292900LC	36	32	149	0	5	7	
T2-39 & T3-24	TraesCS5B02G175800	36	30	0	0	51	5	
T2-39 & T3-24	TraesCS5D02G182600	47	1	0	116	54	23	
T2-4	TraesCS3A02G440800	56	77	272	0	79	38	
T2-4	TraesCS3B02G474900	51	60	3	1	64	46	
T2-4	TraesCS3D02G433400	51	0	8	141	134	90	
T2-40	TraesCS1A02G466500LC	88	84	325	1	19	73	
T2-42	TraesCS5D02G004100	63	0	236	170	166	134	
T2-43 & T3-30	TraesCS7A02G120300	55	60	60	0	6	12	
T2-43 & T3-30	TraesCS7B02G018600	50	57	16	8	117	25	
T2-43 & T3-30	TraesCS7D02G117800	42	0	11	93	18	13	
T2-44	TraesCS5D02G004300	62	0	171	144	213	105	
T2-45 & T3-31	TraesCS7A02G189000	79	92	44	0	3	10	
T2-45 & T3-31	TraesCS7B02G093800	32	31	17	4	48	7	
T2-45 & T3-31	TraesCS7D02G190100	54	25	5	120	193	53	
T2-46	TraesCS7B02G746900LC	44	60	12	0	28	51	
T2-47 & T3-32	TraesCS6A02G307800	56	59	191	4	3	18	
T2-47 7 T3-32	TraesCS6B02G336400	34	41	7	0	48	19	
T2-48	TraesCS1B02G398500	86	118	2	0	51	1	
T2-48	TraesCS1B02G012400	57	76	5	0	50	1	
T2-48	TraesCS1D02G000100	45	11	5	128	160	102	
T2-49	TraesCS1A02G137200	67	63	83	0	2	9	
T2-49 & T3-33-2	TraesCS1B02G155700	41	37	1	0	45	3	
T2-49 & T3-33	TraesCS1D02G138100	34	0	3	100	0	33	
T2-5 & T3-17	TraesCS7A02G287400	83	89	288	1	1	14	
T2-5 & T3-17	TraesCS7B02G183300	77	77	0	0	191	8	
T2-5 & T3-17	TraesCS7D02G284900	78	1	13	219	8	141	
T2-50 & T3-34	TraesCS1A02G091500	18	33	94	0	1	25	
T2-50 & T3-34	TraesCS1B02G119300	35	56	1	0	56	6	
T2-51 & T3-35	TraesCS2A02G468800	68	80	149	20	1	39	
T2-51 & T3-35	TraesCS2B02G491700	44	40	0	3	117	1	
T2-51	TraesCS2D02G468900	64	0	199	190	36	105	
T2-52 & T3-36	TraesCS1A02G353300	75	102	222	2	59	52	
T2-52 & T3-36	TraesCS1B02G368500	57	63	3	1	114	27	
T2-52-1 & T9-67	TraesCS1D02G303700	37	1	19	119	4	40	
T2-53 & T3-28	TraesCS4A02G418200	80	74	4	1	8	50	
T2-53 & T3-28	TraesCS7A02G070100	33	61	11	2	17	6	
T2-53 & T3-28	TraesCS7D02G064300	58	0	35	27	31	17	
T2-54 & T3-29	TraesCS2A02G373600	64	74	259	0	1	55	
T2-54 & T3-29	TraesCS2B02G390700	68	81	0	2	101	8	
T2-54 & T3-29	TraesCS2D02G369800	66	2	2	197	165	131	
T2-55	TraesCS7A02G549300	50	47	161	0	30	16	
T2-55	TraesCS7B02G472300	74	98	14	0	10	93	
T2-55	TraesCS7D02G535600	50	0	40	124	97	22	
T2-56 & T3-39	TraesCS2A02G293400	85	101	279	21	11	57	
T2-56	TraesCS2B02G309500	45	61	0	0	162	45	
T2-56 & T3-39	TraesCS2D02G290800	58	2	24	158	28	79	
T2-57 & T3-40	TraesCS2A02G310300	53	45	183	1	19	30	
T2-57-1	TraesCS2B02G327300	60	67	69	4	146	45	
T2-57 & T3-40	TraesCS2D02G308600	60	3	52	151	32	112	
T2-58 & T3-42	TraesCS7A02G251400	46	37	147	0	1	20	
T2-58 & T3-42	TraesCS7B02G139700	49	49	1	0	106	0	
T2-58 & T3-42	TraesCS7D02G249500	5	1	3	21	1	13	
T2-59 & T3-43	TraesCS1A02G247100	39	40	197	0	6	14	
T2-59 &T3-43	TraesCS1B02G257700	28	26	0	0	10	1	
T2-59 & T3-43	TraesCS1D02G246300	24	1	0	55	9	4	
T2-6	TraesCS7A02G298600	44	46	163	1	4	8	
T2-6	TraesCS7B02G188400	58	69	4	0	180	13	
T2-6	TraesCS7D02G294300	45	0	0	102	9	77	
T2-60 & T3-44	TraesCS5A02G248700	19	27	79	0	20	9	
T2-60 & T3-45	TraesCS5A02G395200	38	52	167	1	54	40	
T2-60 & T3-46	TraesCS5B02G246400	42	42	14	0	6	27	
T2-60 &T3-44	TraesCS5D02G255800	76	1	6	298	39	19	
T2-61 & T3-22	TraesCS6A02G175100	71	79	289	1	0	2	
T2-61 7 T3-22	TraesCS6B02G210000	68	78	2	0	230	6	
T2-7	TraesCS4A02G267000	13	14	33	0	10	3	
T2-7	TraesCS4B02G047300	41	18	8	0	24	4	
T2-7	TraesCS4D02G047300	42	0	3	94	10	35	
T2-8	TraesCS6A02G309400	15	23	59	0	4	16	
T2-8	TraesCS6B02G339100	13	14	0	0	26	11	
T2-8	TraesCS6D02G288800	14	0	0	41	14	15	
T2-9	TraesCS3A02G036600	40	53	16	0	10	9	
T2-9	TraesCS3B02G047300	59	68	51	0	57	3	
T2-9	TraesCS3D02G043500	70	0	90	141	28	61	
T4-1	TraesCS7A02G264400	62	77	315	0	1	102	
T4-1	TraesCS7B02G162500	87	120	11	0	280	59	
T4-1	TraesCS7D02G265400	69	8	0	181	6	44	
T4-10	TraesCS4A02G106400	21	22	81	0	3	17	
T4-10	TraesCS4B02G197800	66	61	0	0	136	34	
T4-10	TraesCS4D02G198600	53	0	1	143	22	73	
T4-11	TraesCS7A02G111300	7	30	35	0	44	27	
T4-11	TraesCS7B02G009200	48	48	12	0	19	3	
T4-11	TraesCS7D02G106400	33	0	8	89	23	11	
T4-12	TraesCS7A02G373500	39	47	13	0	0	5	
T4-12	TraesCS7B02G253500	76	78	0	1	1	3	
T4-12	TraesCS7D02G349600	71	0	2	182	1	3	
T4-13	TraesCS7A02G412400	50	65	223	19	4	57	
T4-13	TraesCS7B02G311600	52	54	0	0	113	3	
T4-13	TraesCS7D02G405500	59	9	131	165	1	117	
T4-14	TraesCS3A02G105400	58	56	236	0	34	13	
T4-14	TraesCS3B02G158300LC	42	47	30	0	58	0	
T4-14	TraesCS3D02G107500	48	0	16	118	22	11	
T4-15	TraesCS1D02G018500	42	2	0	0	9	0	
T4-15	TraesCS6A02G083200	43	58	71	0	14	5	
T4-15	TraesCS6B02G113900	54	59	50	0	56	40	
T4-16	TraesCS7A02G310500	30	34	125	2	3	40	
T4-16	TraesCS7B02G381100LC	45	51	8	0	132	19	
T4-16	TraesCS7D02G307000	57	22	58	106	76	44	
T4-17	TraesCS1A02G131600	64	69	263	8	5	20	
T4-17	TraesCS1B02G154400	60	72	38	39	157	44	
T4-17	TraesCS1D02G136400	53	11	70	149	40	123	
T4-18	TraesCS4A02G485400	68	93	0	0	167	92	
T4-18	TraesCS7A02G009800	84	106	331	0	94	69	
T4-18	TraesCS7D02G009400	89	65	3	209	204	97	
T4-19	TraesCS4A02G486000	24	16	0	0	59	16	
T4-19	TraesCS7A02G009100	29	53	142	0	37	16	
T4-19	TraesCS7D02G008700	34	16	0	80	33	20	
T4-2	TraesCS6A02G417900	160	171	418	127	317	143	
T4-2	TraesCS6B02G471100	112	42	80	69	54	22	
T4-2	TraesCS6D02G403200	63	105	134	212	123	37	
T4-20	TraesCS4A02G485900	31	46	0	0	83	18	
T4-20	TraesCS7A02G009200	72	63	238	5	87	52	
T4-20	TraesCS7D02G008800	67	0	1	100	4	21	
T4-21	TraesCS6A02G151700	58	52	223	0	33	18	
T4-21	TraesCS6B02G179900	36	38	1	0	86	8	
T4-21	TraesCS6D02G141700	24	1	1	70	9	22	
T4-22	TraesCS2A02G395000	71	84	283	12	83	51	
T4-22	TraesCS2B02G413000	78	99	1	0	134	49	
T4-22	TraesCS2D02G392800	71	0	66	201	95	96	
T4-23	TraesCS4A02G216100	63	77	261	0	82	37	
T4-23	TraesCS4B02G100100	75	81	18	0	126	18	
T4-23	TraesCS4D02G096400	71	1	52	196	50	133	
T4-24	TraesCS4A02G097400	73	97	303	0	0	0	
T4-24	TraesCS4B02G207100	72	95	0	0	132	0	
T4-24	TraesCS4D02G207900	73	0	9	230	4	136	
T4-25	TraesCS4A02G097500	77	97	303	0	0	5	
T4-25	TraesCS4B02G207000	72	95	0	0	132	0	
T4-25	TraesCS4D02G207800	73	0	9	230	5	136	
T4-26	TraesCS4B02G322700	44	54	1	19	102	36	
T4-26	TraesCS4D02G319100	35	0	41	75	41	26	
T4-26	TraesCS5A02G494800	38	42	64	0	19	12	
T4-28	TraesCS5B02G544800	66	77	52	7	146	27	
T4-29	TraesCS7A02G548000	61	79	138	0	16	8	
T4-29	TraesCS7B02G471500	78	91	0	0	95	1	
T4-29	TraesCS7D02G534700	67	4	0	183	0	71	
T4-3	TraesCS4B02G329500	33	16	1	0	50	39	
T4-3	TraesCS4D02G326400	45	17	0	95	26	31	
T4-30	TraesCS4B02G378500	61	157	19	0	98	80	
T4-30	TraesCS5A02G545100	74	82	257	3	77	94	
T4-31	TraesCS6A02G146900	52	47	164	0	3	9	
T4-31	TraesCS6B02G175100	48	37	13	0	103	17	
T4-31	TraesCS6D02G136200	54	0	10	113	3	30	
T4-32	TraesCS7A02G508800	48	42	147	0	102	28	
T4-32	TraesCS7B02G418400	46	68	18	0	93	9	
T4-32	TraesCS7B02G418500	33	37	25	0	65	31	
T4-32	TraesCS7D02G497400	40	0	22	105	101	21	
T4-33	TraesCS4A02G384800	49	67	0	44	34	1	
T4-33	TraesCS7D02G080300	54	0	0	136	72	79	
T4-34	TraesCS4A02G296300	61	81	155	0	64	4	
T4-34	TraesCS4D02G015800	67	0	22	165	26	22	
T4-35	TraesCS1A02G295900	41	53	174	0	3	4	
T4-35	TraesCS1D02G296300	36	0	0	101	25	14	
T4-36	TraesCS5A02G236700	59	53	204	0	9	0	
T4-36	TraesCS5B02G236300	54	53	4	1	22	1	
T4-37	TraesCS7A02G049400	59	17	37	27	85	24	
T4-37	TraesCS7D02G044500	45	16	69	84	56	28	
T4-38	TraesCS3A02G125200	59	60	202	0	143	2	
T4-38	TraesCS3B02G144800	54	74	10	0	150	89	
T4-38	TraesCS3D02G126200	39	37	52	126	74	52	
T4-39-1	TraesCS2A02G259500	57	60	241	0	38	57	
T4-39-1	TraesCS2B02G282400	47	53	16	0	86	24	
T4-39	TraesCS2D02G264200	46	8	15	129	63	43	
T4-4	TraesCS3A02G049500	72	65	2	2	25	105	
T4-4	TraesCS3B02G056100	52	84	10	0	2	55	
T4-4	TraesCS3D02G049300	38	3	3	3	66	8	
T4-40	TraesCS1A02G186800	22	27	2	0	0	0	
T4-40	TraesCS1B02G194700	21	36	12	0	24	11	
T4-40	TraesCS1D02G189800	76	0	49	159	6	12	
T4-41	TraesCS4B02G346900	25	34	39	0	44	16	
T4-41	TraesCS5A02G515600	42	45	90	0	4	2	
T4-42	TraesCS4A02G017000	42	45	90	0	3	2	
T4-42	TraesCS4B02G287100	33	34	0	0	27	2	
T4-42	TraesCS4D02G285800	27	0	0	71	51	31	
T4-43	TraesCS3A02G270800	24	31	26	0	3	7	
T4-43	TraesCS3D02G270500	6	0	10	15	11	15	
T4-44	TraesCS5A02G453200	48	57	224	0	46	27	
T4-44	TraesCS5B02G462200	38	53	14	3	62	39	
T4-44	TraesCS5D02G464000	46	4	97	119	46	39	
T4-45	TraesCS7A02G537200	59	60	10	0	41	8	
T4-45	TraesCS7B02G766300LC	46	32	20	0	14	11	
T4-45	TraesCS7D02G524300	69	0	46	173	55	60	
T4-46	TraesCS5A02G226400	45	53	103	0	21	28	
T4-46	TraesCS5B02G224800	58	78	6	0	135	63	
T4-46	TraesCS5D02G233700	55	1	72	141	66	56	
T4-47	TraesCS3A02G129000	31	32	90	0	2	0	
T4-47	TraesCS3B02G148100	54	68	69	9	56	4	
T4-47	TraesCS3D02G129900	47	0	56	106	56	90	
T4-48	TraesCS2A02G136800	40	50	15	0	19	3	
T4-48	TraesCS2B02G160700	31	43	104	0	3	40	
T4-48	TraesCS2D02G139500	57	1	45	146	10	13	
T4-49	TraesCS1A02G338700	69	80	285	0	38	15	
T4-49	TraesCS1B02G351200	63	67	10	0	135	35	
T4-5	TraesCS2A02G343100	48	50	160	0	13	18	
T4-5	TraesCS2B02G340700	28	33	2	0	32	3	
T4-5	TraesCS2D02G321400	35	0	2	94	10	56	
T4-50	TraesCS5A02G261400	64	63	221	0	30	11	
T4-50	TraesCS5D02G268800	56	17	25	155	30	83	
T4-51	TraesCS1A02G089500	52	59	190	0	70	21	
T4-51	TraesCS1B02G117400	57	70	70	3	111	43	
T4-51	TraesCS1D02G098000	56	1	2	118	10	51	
T4-52	TraesCS7A02G478700	66	56	222	0	10	31	
T4-52	TraesCS7B02G381400	41	60	5	4	87	69	
T4-53	TraesCS5A02G321300	40	17	53	0	52	6	
T4-53	TraesCS5B02G321800	23	37	4	0	42	26	
T4-53	TraesCS5D02G328100	54	0	9	134	36	47	
T4-54	TraesCS7A02G176700	45	44	129	0	24	50	
T4-54	TraesCS7B02G081700	48	61	2	0	313	5	expected ratio
T4-54	TraesCS7D02G178000	50	0	7	160	15	10	KR 1.5 (6/4)
T4-55	TraesCS2A02G583400	25	29	87	1	0	15	exclude if <0.7
T4-55	TraesCS2B02G603100	44	44	59	0	113	54	MDR 3 (6/2)
T4-55	TraesCS2D02G596300	61	3	37	128	39	33	exclude if <1.5
T4-56	TraesCS1A02G047000	31	47	85	0	0	0	ENT 3 (6/2)
T4-56	TraesCS1B02G060700	16	25	10	0	42	13	exclude if <1.5
T4-56	TraesCS1D02G047900	38	0	1	109	14	22	ASP 3 (6/2)
T4-57	TraesCS3A02G206400	68	73	241	0	0	8	exclude if <1.5
T4-57	TraesCS3B02G238500	52	69	3	0	163	0	APG 1.5 (6/4)
T4-57	TraesCS3D02G209200	61	0	0	156	1	128	exclude if <0.7
T4-58	TraesCS5A02G447200	56	58	221	0	1	3	
T4-58	TraesCS5B02G572600	60	64	0	0	77	44	
T4-58	TraesCS5D02G564000	32	0	2	100	2	50	
T4-59	TraesCS6A02G241000	39	39	225	0	6	42	
T4-59	TraesCS6B02G283600	26	36	0	0	120	28	
T4-59	TraesCS6D02G223300	39	0	0	110	14	19	
T4-6	TraesCS6A02G203600.3	68	74	272	22	72	62	
T4-6	TraesCS6B02G223700	56	55	46	11	117	24	
T4-6	TraesCS6D02G186900	25	0	5	56	1	29	
T4-60	TraesCS6A02G089400	31	45	166	0	2	13	
T4-60	TraesCS6B02G111800	32	29	0	0	29	9	
T4-7	TraesCS7A02G293300	38	37	37	0	4	5	
T4-7	TraesCS7B02G182300	84	93	4	8	174	16	
T4-7	TraesCS7D02G291200	22	0	0	71	2	19	
T4-8	TraesCS2A02G414300	60	67	221	0	32	31	
T4-8-1	TraesCS2B02G433400	79	87	57	0	170	42	
T4-8	TraesCS2D02G411600	59	0	13	179	36	56	
T4-9	TraesCS3A02G347800LC	72	92	244	4	170	11	
T4-9	TraesCS3B02G255300	66	84	39	1	106	16	
T4-9	TraesCS3D02G227500	76	16	82	204	43	170	
T5-1	TraesCS4A02G035400	30	33	11	0	0	14	
T5-1	TraesCS4B02G268100	55	40	3	0	44	3	
T5-1	TraesCS4D02G267500	31	0	0	68	1	50	
T5-10	TraesCS4B02G396900	50	67	12	0	122	37	
T5-10	TraesCS5A02G558200	47	56	184	0	11	12	
T5-11	TraesCS1A02G176700	52	84	227	0	17	50	
T5-11	TraesCS1D02G162400	78	5	70	184	113	107	
T5-12	TraesCS1A02G020500	59	69	53	0	1	5	
T5-12	TraesCS1B02G024500	37	40	1	0	39	15	
T5-12	TraesCS1D02G020000	37	0	2	125	23	21	
T5-13	TraesCS7A02G178000	31	35	102	0	9	29	
T5-13	TraesCS7B02G083700	64	58	9	0	166	10	
T5-13	TraesCS7D02G179800	32	0	22	86	60	48	
T5-14	TraesCS7A02G412400	50	65	223	19	3	57	
T5-14	TraesCS7B02G311600	52	54	0	0	113	3	
T5-14	TraesCS7D02G405500	59	8	131	165	1	117	
T5-15	TraesCS4A02G235600	73	79	73	0	20	19	
T5-15	TraesCS4B02G079300	61	62	20	2	44	9	
T5-15	TraesCS4D02G078100	35	0	5	105	37	39	
T5-16	TraesCS5A02G481400	56	63	194	0	27	59	
T5-16B	TraesCS5A02G6481300	38	26	65	0	1	1	
T5-16A	TraesCS5B02G494600	44	69	16	9	64	22	
T5-16B	TraesCS5B02G494500	16	5	0	0	11	1	
T5-16	TraesCS5D02G495000	46	8	56	134	55	42	
T5-16B	TraesCS5D02G494900	9	1	14	13	2	6	
T5-18	TraesCS3D02G099500	56	0	1	144	1	38	
T5-19	TraesCS2A02G432400	64	85	270	0	9	78	
T5-19	TraesCS2B02G453700	75	102	186	0	257	153	
T5-2	TraesCS3A02G099200	36	50	154	0	7	50	
T5-2	TraesCS3B02G115400	47	48	0	0	123	40	
T5-2	TraesCS3D02G099500	56	0	1	144	1	38	
T5-20	TraesCS4A02G208400	20	30	88	0	20	1	
T5-20	TraesCS4B02G112000	15	28	0	0	20	3	
T5-20	TraesCS4D02G109500	34	0	0	51	14	34	
T5-21	TraesCS2A02G142800	82	103	722	0	42	54	
T5-21	TraesCS2B02G167900	75	3	108	1	66	56	
T5-21	TraesCS2D02G146100	54	3	156	184	124	54	
T5-22	TraesCS3B02G055700LC	33	61	0	0	33	39	
T5-22	TraesCS3D02G045700	68	2	28	170	11	10	
T5-23	TraesCS5A02G384100	74	70	38	0	23	36	
T5-23	TraesCS5B02G388900	43	67	12	0	21	8	
T5-23	TraesCS5D02G393600	65	3	84	204	55	42	
T5-24	TraesCS4A02G177600	68	91	264	0	39	61	
T5-24	TraesCS4B02G140200	75	98	46	3	84	24	
T5-24	TraesCS4D02G134900	49	1	83	150	127	94	
T5-25	TraesCS2A02G277100	37	44	119	0	0	8	
T5-25	TraesCS2B02G294500	36	40	0	0	102	1	expected ratio
T5-25	TraesCS2D02G276000	41	0	13	111	12	30	KR 1.5 (6/4)
T5-26	TraesCS3D02G046300	57	23	0	118	45	25	exclude if <0.7
T5-27	TraesCS7A02G235700	70	77	231	0	6	69	MDR 3 (6/2)
T5-27	TraesCS7B02G133800	31	42	13	0	58	10	exclude if <1.5
T5-27	TraesCS7D02G235700	67	0	31	172	59	62	ENT 3 (6/2)
T5-28	TraesCS3A02G021800	45	38	129	0	25	8	exclude if <1.5
T5-28	TraesCS3B02G024000	26	10	20	0	9	9	ASP 3 (6/2)
T5-28	TraesCS3D02G025000	26	3	20	66	27	11	exclude if <1.5
T5-29	TraesCS3A02G119500	38	39	115	0	2	12	APG 1.5 (6/4)
T5-29	TraesCS3B02G139100	16	19	2	0	23	1	exclude if <0.7
T5-29	TraesCS3D02G121700	22	0	1	76	14	34	
T5-3	TraesCS1A02G221900	47	52	120	0	27	45	
T5-3	TraesCS1B02G235100	38	35	6	0	87	18	
T5-3	TraesCS1D02G223600	43	0	227	115	53	60	
T5-30	TraesCS1A02G119100	16	15	27	0	0	3	
T5-30	TraesCS1B02G138600	61	74	42	0	141	26	
T5-30	TraesCS1D02G120000	64	0	215	174	111	80	
T5-4	TraesCS3D02G099500	56	0	1	144	1	38	
T5-5	TraesCS7A02G164000	29	31	89	0	29	8	
T5-5	TraesCS7B02G069800	32	42	22	0	45	9	
T5-5	TraesCS7D02G166100	73	0	57	145	16	54	
T5-6	TraesCS5B02G132600	49	74	81	2	149	98	
T5-6	TraesCS5D02G140400	60	1	151	173	63	43	
T5-7	TraesCS5B02G193200	28	25	1	0	58	13	
T5-7	TraesCS5D02G200900	62	19	59	134	27	42	
T5-8	TraesCS5A02G382800	56	61	240	13	90	27	
T5-8	TraesCS5B02G387100	31	37	0	0	252	26	
T5-8	TraesCS2D02G387100	32	0	2	84	4	59	
T8-1	TraesCS3A02G187800	65	83	294	0	0	13	
T8-1	TraesCS3B02G217100	67	69	1	0	190	5	
T8-1	TraesCS3D02G191400	60	0	1	204	1	147	
T8-10	TraesCS2A02G261200	86	112	334	0	164	18	
T8-10	TraesCS2B02G281000	74	107	8	1	208	65	
T8-10	TraesCS2D02G262700	88	0	2	254	20	155	
T8-11	TraesCS1A02G199600	20	25	63	0	9	1	
T8-11	TraesCS1B02G214500	13	23	9	0	59	3	
T8-11	TraesCS1D02G203300	14	0	3	40	5	28	
T8-12	TraesCS2A02G443100	64	84	149	0	108	24	
T8-12	TraesCS2B02G464200	54	79	86	0	37	15	
T8-12	TraesCS2D02G442200	69	0	139	174	117	85	
T8-13	TraesCS5A02G376500	39	48	137	0	51	8	
T8-13	TraesCS5B02G380200	58	58	1	1	111	7	
T8-13	TraesCS5D02G386500	70	0	2	162	36	62	
T8-14	TraesCS4A02G016400	52	69	244	0	9	19	
T8-14	TraesCS4B02G287800	55	72	17	0	121	60	
T8-14	TraesCS4D02G286500	60	0	46	143	61	67	
T8-15	TraesCS1A02G350500	65	82	236	0	19	102	
T8-15	TraesCS1B02G364800	46	62	2	0	129	34	
T8-15	TraesCS1D02G353300	59	0	7	159	26	43	
T8-16	TraesCS3A02G282800	11	12	56	0	3	9	
T8-16	TraesCS3B02G316500	21	15	0	0	43	11	
T8-16	TraesCS3D02G282800	18	0	2	34	6	20	
T8-17	TraesCS2B02G405700	43	23	0	0	97	11	
T8-17	TraesCS2D02G385500	23	0	0	31	0	15	
T8-18	TraesCS5B02G319600	14	26	0	0	48	5	
T8-18	TraesCS5D02G325400	30	2	14	84	3	63	
T8-19	TraesCS3A02G093200	71	86	285	20	60	38	
T8-19	TraesCS3B02G108500	64	84	42	5	136	58	
T8-19	TraesCS3D02G093500	67	33	70	191	186	149	
T8-2	TraesCS2A02G212200	44	49	181	0	5	5	
T8-2	TraesCS2B02G237300	34	45	2	0	119	47	
T8-2	TraesCS2D02G218100	40	0	0	140	75	56	
T8-21	TraesCS5B02G486900	72	76	0	0	122	15	
T8-21	TraesCS5D02G486600	63	3	12	159	47	26	
T8-22	TraesCS1A02G339300	39	55	214	0	40	23	
T8-22	TraesCS1B02G351600	71	70	32	0	94	4	
T8-22	TraesCS1D02G341100	55	0	5	122	96	68	
T8-23	TraesCS6B02G286400	63	79	75	0	117	51	
T8-23	TraesCS6D02G240200	27	0	0	53	4	14	
T8-24	TraesCS2A02G058300	48	31	109	0	62	82	
T8-24	TraesCS2B02G071100	61	75	39	0	29	58	
T8-24	TraesCS2D02G057300	49	28	114	119	32	74	
T8-25	TraesCS2A02G053200	89	86	299	0	1	0	
T8-25	TraesCS2B02G067100	77	66	10	0	73	39	
T8-25	TraesCS2D02G053400	61	0	0	194	7	69	
T8-26	TraesCS6A02G018200	73	239	501	4	11	233	
T8-26	TraesCS6D02G021100	63	27	105	107	1115	74	
T8-27	TraesCS7A02G175200	51	49	84	0	5	32	
T8-27	TraesCS7B02G080300	46	55	6	2	47	16	
T8-27	TraesCS7D02G176700	38	1	2	73	5	25	
T8-3	TraesCS5A02G233600	45	56	122	0	22	65	
T8-3	TraesCS5B02G232100	54	66	103	1	134	47	
T8-3	TraesCS5D02G240500	56	1	192	174	75	79	
T8-4	TraesCS3A02G251200	35	56	202	0	2	36	
T8-4	TraesCS3B02G280700	42	49	0	0	125	22	
T8-4	TraesCS3D02G251600	47	0	0	121	49	74	
T8-5	TraesCS4A02G113000	119	171	109	1	13	9	
T8-5	TraesCS4B02G191200	41	46	7	0	86	15	
T8-5	TraesCS4D02G192400	37	1	20	118	9	49	
T8-6	TraesCS2A02G331200	31	27	97	0	1	2	
T8-6	TraesCS2B02G351000	23	22	0	0	63	0	
T8-6	TraesCS2D02G331700	33	0	5	57	20	3	
T8-7	TraesCS6A02G071200	51	70	167	0	64	47	
T8-7	TraesCS6B02G095600	56	49	11	0	105	11	
T8-7	TraesCS6D02G069300	34	0	37	112	103	58	
T8-8	TraesCS5A02G391800	23	31	105	0	0	0	
T8-8	TraesCS5B02G396700	34	38	0	0	52	21	
T8-8	TraesCS5D02G401700	13	0	0	37	10	12	
T8-9	TraesCS2A02G174300	41	56	136	0	6	18	
T8-9	TraesCS2B02G200800	26	20	0	0	46	2	
T8-9	TraesCS2D02G181400	59	22	29	171	89	106	
T9-1	TraesCS2A02G262700	54	71	263	0	49	91	
T9-1	TraesCS2B02G279400	58	53	10	0	136	28	
T9-1	TraesCS2D02G261300	56	0	0	143	0	58	
T9-10	TraesCS7A02G492400	44	53	143	0	8	16	
T9-10	TraesCS7B02G398100	40	46	0	2	79	27	
T9-10	TraesCS7D02G478800	31	0	1	86	0	12	
T9-11	TraesCS1A02G236100	111	122	683	2	133	115	
T9-11	TraesCS1B02G247700	90	134	24	0	132	67	
T9-11	TraesCS1D02G236200	90	0	0	273	234	135	
T9-12	TraesCS1A02G278400	92	114	422	0	0	157	
T9-12	TraesCS1B02G287300	65	77	1	0	148	5	
T9-12	TraesCS1D02G277600	95	0	15	208	5	75	
T9-13	TraesCS5A02G222400	70	77	265	8	102	70	
T9-13	TraesCS5B02G221400	64	75	92	9	53	45	
T9-13	TraesCS5D02G230200	56	3	58	171	168	75	
T9-14	TraesCS4B02G111100	41	40	13	0	101	25	
T9-14	TraesCS4A02G207600	57	66	45	2	19	30	
T9-15	TraesCS7A02G415300	29	22	86	0	44	12	
T9-15	TraesCS7B02G315200	29	19	0	0	21	6	
T9-15	TraesCS7D02G408500	24	0	9	64	25	30	
T9-16	TraesCS5A02G401300	34	57	151	0	18	14	
T9-16	TraesCS5B02G406100	63	57	3	0	74	18	
T9-16	TraesCS5D02G411600	32	0	17	64	10	13	
T9-17	TraesCS5A02G106400	77	95	311	2	4	17	
T9-17	TraesCS5D02G120500	40	0	4	135	7	91	
T9-18	TraesCS6A02G276100	31	32	95	0	5	10	
T9-18	TraesCS6B02G303600	60	77	5	0	167	25	
T9-18	TraesCS6D02G256400	39	0	99	129	10	20	
T9-19	TraesCS4A02G048200	33	39	126	0	12	5	
T9-19	TraesCS4B02G256600	36	48	4	0	35	8	
T9-19	TraesCS4D02G256700	27	1	12	99	59	59	
T9-2	TraesCS7A02G160800	11	10	52	0	0	3	
T9-2	TraesCS7D02G161900	10	1	4	29	1	13	
T9-20	TraesCS2A02G192600	24	19	85	0	0	8	
T9-20	TraesCS2B02G214600	54	85	0	0	121	3	
T9-20	TraesCS2D02G194800	32	0	18	79	0	44	
T9-21	TraesCS6A02G138600	61	61	205	0	34	49	
T9-21	TraesCS6B02G167100	53	60	20	0	91	15	
T9-21	TraesCS6D02G127600	77	0	5	170	32	56	
T9-22	TraesCS4A02G191300	29	44	142	0	12	36	
T9-22	TraesCS4B02G124000	59	77	5	4	160	83	
T9-22	TraesCS4D02G122000	65	3	16	146	10	45	
T9-23	TraesCS4A02G271000	57	86	221	0	8	29	
T9-23	TraesCS4B02G043100	63	86	0	0	112	6	
T9-23	TraesCS4D02G040400	58	0	27	130	59	106	
T9-24	TraesCS4A02G312500	74	105	160	0	0	20	
T9-24	TraesCS4B02G001200	96	128	23	0	218	92	
T9-24	TraesCS4D02G001700	67	0	0	209	15	23	
T9-25	TraesCS4B02G375600	47	50	0	0	46	0	
T9-25	TraesCS4D02G361700	21	0	0	52	6	41	
T9-25	TraesCS5A02G537300	26	39	105	0	5	1	
T9-26	TraesCS6A02G398400	31	31	126	0	18	6	
T9-26	TraesCS6B02G438700	38	43	0	0	35	20	
T9-26	TraesCS6D02G382600	43	2	0	94	48	43	
T9-27	TraesCS4A02G039000	84	96	229	0	42	21	
T9-27	TraesCS4B02G266700	67	88	168	1	155	104	
T9-27	TraesCS4D02G266400	64	0	171	186	75	90	
T9-28	TraesCS4A02G415400	57	73	229	0	4	44	
T9-28	TraesCS4B02G316200	58	68	8	0	128	53	
T9-28	TraesCS4D02G312800	46	0	17	149	105	49	
T9-29	TraesCS5A02G144000	35	34	64	0	1	19	
T9-29	TraesCS5B02G143100	75	104	1	0	179	12	
T9-29	TraesCS5D02G148000	85	0	1	245	5	118	
T9-3	TraesCS6A02G320100	67	53	74	0	2	1	
T9-3	TraesCS6B02G350900	45	73	9	0	91	41	
T9-3	TraesCS6D02G299600	79	4	175	236	86	107	
T9-30	TraesCS5A02G361300	57	70	228	0	40	34	
T9-30	TraesCS5B02G363900	63	69	1	1	113	22	
T9-30	TraesCS5D02G370200	60	0	61	151	57	72	
T9-31	TraesCS4A02G236200	56	54	162	0	2	14	
T9-31	TraesCS4B02G078800	57	54	3	0	106	14	
T9-31	TraesCS4D02G077600	60	0	4	130	0	52	
T9-32	TraesCS6A02G229500	61	64	235	0	30	32	
T9-32	TraesCS6B02G252000	25	45	0	0	33	0	
T9-32	TraesCS6D02G205300	47	0	0	113	7	62	
T9-33	TraesCS1A02G082700	42	33	84	0	1	15	
T9-33	TraesCS1B02G100300	28	38	5	0	42	16	
T9-33	TraesCS1D02G084100	32	0	63	111	51	15	
T9-34	TraesCS1A02G379700	69	80	289	0	30	41	
T9-34	TraesCS1B02G265400	51	50	34	0	89	31	
T9-34	TraesCS1D02G387100	65	0	14	164	15	28	
T9-35	TraesCS5A02G142700	43	46	110	0	0	16	
T9-35	TraesCS5D02G149400	21	0	4	81	2	24	
T9-36	TraesCS7A02G307900	39	34	48	0	0	17	
T9-36	TraesCS7B02G208300	33	35	5	0	39	4	
T9-36	TraesCS7D02G304800	37	0	31	117	2	61	
T9-37	TraesCS3A02G135400	77	86	275	0	15	38	
T9-37	TraesCS3B02G169200	97	81	9	8	148	38	
T9-37	TraesCS3D02G151400	48	1	47	142	49	50	
T9-38	TraesCS6A02G024300	37	56	171	0	22	18	
T9-38	TraesCS6B02G033300	39	46	0	0	41	9	
T9-38	TraesCS6D02G027700	43	0	2	96	8	15	
T9-39	TraesCS3A02G167100	41	51	166	0	1	29	
T9-39	TraesCS3D02G173200	47	0	0	128	65	54	
T9-4	TraesCS1A02G113400	50	38	94	0	0	16	
T9-4	TraesCS1D02G114800	25	0	7	73	7	34	
T9-40	TraesCS2A02G230100	72	88	259	0	1	21	
T9-40	TraesCS2B02G251700	69	69	0	0	173	31	
T9-40	TraesCS2D02G233800	54	5	2	183	25	100	
T9-41	TraesCS2A02G309700	44	60	194	36	2	28	
T9-41	TraesCS2B02G326800	52	84	1	0	111	49	
T9-41	TraesCS2D02G308000	31	0	2	95	45	19	
T9-42	TraesCS2A02G467000	37	46	128	0	31	9	
T9-42	TraesCS2B02G489800	35	36	8	1	62	11	
T9-42	TraesCS2D02G467000	37	0	56	93	79	25	
T9-43	TraesCS2A02G264800	97	119	252	0	0	42	
T9-43	TraesCS2B02G277300	83	116	5	0	235	91	
T9-43	TraesCS2D02G259200	87	0	188	244	26	94	
T9-44	TraesCS3A02G280200	70	74	236	0	13	26	
T9-44	TraesCS3B02G314000	61	71	3	0	131	20	
T9-44	TraesCS3D02G279900	87	4	18	214	134	63	
T9-45	TraesCS5B02G480900	40	48	2	24	35	10	
T9-45	TraesCS1B02G314700	36	52	2	0	66	19	
T9-45	TraesCS7D02G330200	78	1	2	236	59	51	
T9-46	TraesCS5A02G597900LC	30	41	137	0	2	4	
T9-46	TraesCS5B02G446100	33	52	0	0	44	5	
T9-46	TraesCS5D02G323500	24	0	2	67	1	24	
T9-47	TraesCS6A02G315100	17	16	84	0	16	4	
T9-47	TraesCS6D02G294400	20	0	0	51	16	15	
T9-48	TraesCS2A02G491000	24	35	59	0	0	13	
T9-48	TraesCS2B02G519200	34	34	3	0	74	13	
T9-48	TraesCS2D02G491200	31	0	2	59	27	13	
T9-49	TraesCS5A02G382600	82	82	348	0	18	25	
T9-49	TraesCS5B02G386800	96	90	48	2	96	67	
T9-49	TraesCS5D02G392000	74	0	65	213	83	73	
T9-5	TraesCS2A02G386400	64	74	270	0	10	2	
T9-5	TraesCS2B02G403800	69	78	22	0	48	107	
T9-5	TraesCS2D02G383200	73	10	13	182	21	117	
T9-50	TraesCS5A02G069500	49	42	173	0	1	3	
T9-50	TraesCS5B02G075800	74	84	0	0	47	20	
T9-50	TraesCS5D02G081700	37	1	19	116	13	39	
T9-51	TraesCS2A02G102100	60	61	250	1	50	90	
T9-51	TraesCS2B02G119200	68	71	1	0	16	12	
T9-51	TraesCS2D02G101500	48	0	0	129	41	13	
T9-52	TraesCS7A02G301300	16	15	78	0	0	2	
T9-52	TraesCS7B02G201600	29	30	0	0	107	0	
T9-52	TraesCS7D02G296700	25	0	1	60	2	35	
T9-53	TraesCS1A02G196100	41	57	185	0	28	8	
T9-53	TraesCS1B02G210700	59	85	34	4	78	33	
T9-53	TraesCS1D02G199500	56	4	111	200	96	130	
T9-54	TraesCS7A02G141600	80	86	297	0	16	45	
T9-54	TraesCS7B02G044400	80	90	16	5	3	57	
T9-54	TraesCS7D02G142900	74	0	26	200	10	71	
T9-55	TraesCS7B02G084800LC	57	67	13	0	61	23	
T9-55	TraesCS7D02G149400	10	0	1	22	0	1	
T9-56	TraesCS1A02G217900	26	16	97	0	15	13	
T9-56	TraesCS1B02G231300	114	105	1050	0	663	187	
T9-56	TraesCS1D02G219600	26	0	0	50	1	2	
T9-57	TraesCS6A02G343300	24	40	90	0	20	1	
T9-57	TraesCS6B02G375400	54	49	0	15	63	35	
T9-57	TraesCS6D02G324200	31	0	1	83	43	76	
T9-58	TraesCS1A02G210900	43	51	134	0	2	59	
T9-58	TraesCS1B02G225000	64	73	24	0	67	29	
T9-58	TraesCS1D02G214200	36	0	2	114	12	18	
T9-59	TraesCS7A02G158000	43	38	97	0	0	11	
T9-59	TraesCS7B02G062200	19	21	0	0	29	5	
T9-59	TraesCS7D02G158500	16	0	2	38	3	2	
T9-6	TraesCS2A02G327900	38	55	240	4	57	133	
T9-6	TraesCS2D02G335000	45	0	12	119	1	36	
T9-60	TraesCS4A02G219700	33	40	86	1	9	8	
T9-60	TraesCS4B02G098200	34	42	2	0	39	2	
T9-60	TraesCS4D02G094400	51	2	10	136	33	66	
T9-61	TraesCS5A02G468300	51	49	196	0	0	1	
T9-61	TraesCS5B02G480900	40	48	3	24	35	10	
T9-61	TraesCS5D02G481200	24	0	0	8	4	3	
T9-63	TraesCS5A02G477600	29	36	115	0	1	2	
T9-63	TraesCS5D02G491100	35	0	0	84	3	5	
T9-64	TraesCS7A02G422500	42	49	213	0	0	24	
T9-64	TraesCS7B02G322900	33	55	0	0	110	26	
T9-64	TraesCS7D02G414700	60	0	5	118	36	23	
T9-65	TraesCS2B02G117900	72	82	15	0	31	11	
T9-65	TraesCS2D02G100200	58	0	152	166	64	101	
T9-66	TraesCS7A02G375400	40	48	86	0	0	11	
T9-66	TraesCS7B02G277000	60	50	25	0	67	24	
T9-66	TraesCS7D02G371900	49	0	1	136	13	37	
T9-67	TraesCS1A02G304200	38	38	131	0	3	6	
T9-67	TraesCS1B02G314700	36	52	2	0	66	19	
T9-67	TraesCS1D02G303700	37	1	19	119	4	40	
T9-68	TraesCS2A02G551100	28	37	68	0	6	16	
T9-69	TraesCS6A02G318400	61	63	183	0	39	16	
T9-69	TraesCS6B02G348700	51	59	1	0	33	11	
T9-69	TraesCS6D02G297600	58	0	9	125	12	14	
T9-7	TraesCS2A02G423900	42	52	80	0	38	6	
T9-7	TraesCS2B02G443500	46	51	73	0	57	24	
T9-7	TraesCS2D02G421700	50	0	42	133	54	41	
T9-70	TraesCS3A02G428800	34	36	2	0	0	1	
T9-70	TraesCS3B02G466400	22	43	12	0	56	8	
T9-70	TraesCS3D02G424400	56	16	1	30	10	3	
T9-71	TraesCS4A02G247200	56	64	244	0	20	35	
T9-72	TraesCS4B02G375600	47	50	0	0	46	0	
T9-73	TraesCS3A02G176100	44	61	207	0	2	23	
T9-73	TraesCS3B02G205800	45	62	14	2	157	62	
T9-74	TraesCS5A02G157300	96	106	345	0	14	28	
T9-74	TraesCS5B02G156400	87	119	1	0	244	28	
T9-74	TraesCS5D02G162600	51	0	2	145	37	96	
T9-75	TraesCS6B02G214900	51	97	0	0	68	2	
T9-75	TraesCS6D02G173000	54	1	37	153	87	99	
T9-76	TraesCS7A02G363100	25	40	136	0	4	9	
T9-76	TraesCS7B02G264800	53	65	17	5	111	36	
T9-76	TraesCS7D02G359900	50	0	17	146	57	73	
T9-77	TraesCS7A02G213600	55	62	205	3	12	67	
T9-77	TraesCS7B02G120800	67	79	0	4	167	78	
T9-77	TraesCS7D02G215600	45	0	8	117	30	86	
T9-8	TraesCS5A02G378300	68	67	251	0	3	4	
T9-8	TraesCS5D02G388300	35	0	3	85	15	22	
T9-9	TraesCS7A02G249900	46	69	212	1	38	20	
T9-9	TraesCS7B02G141300	60	68	0	2	132	43	
T9-9	TraesCS7D02G248500	55	0	0	173	52	83	
T10-1	TraesCS4A02G105600	43	61	144	0	3	16	
T10-1	TraesCS4B02G198400	56	52	1	0	108	12	
T10-1	TraesCS4D02G199200	83	0	1	209	1	80	
T10-10	TraesCS3A02G124100	82	94	101	0	12	38	
T10-10	TraesCS3B02G143800	73	85	173	0	238	46	
T10-10	TraesCS3D02G125600	80	1	108	205	21	93	
T10-11	TraesCS4B02G045000	37	41	0	0	34	14	
T10-11	TraesCS4D02G044700	55	0	0	148	132	18	
T10-12	TraesCS5A02G174300	77	70	291	17	163	117	
T10-12	TraesCS5B02G171700	71	81	10	0	43	46	
T10-13	TraesCS5A02G152300	59	71	246	0	12	49	
T10-13	TraesCS5B02G150900	54	66	4	0	166	13	
T10-13	TraesCS5D02G157500	77	0	0	148	4	32	
T10-14	TraesCS7A02G233700	62	74	225	0	0	18	
T10-14	TraesCS7B02G132000	34	43	0	0	48	26	
T10-14	TraesCS7D02G233900	68	0	0	171	3	43	
T10-15	TraesCS4B02G229600LC	75	78	0	0	203	0	
T10-16	TraesCS1B02G075900	72	76	2	0	15	3	
T10-16	TraesCS1D02G059600	64	0	6	149	85	15	
T10-17	TraesCS2B02G234700	35	31	0	0	21	2	
T10-18	TraesCS7A02G455900	24	24	57	0	2	3	
T10-18	TraesCS7D02G444800	50	0	0	135	1	2	
T10-19	TraesCS6A02G335700	58	62	225	1	3	37	
T10-2	TraesCS7A02G300300	71	79	314	0	0	0	
T10-2	TraesCS7D02G296000	56	0	11	129	2	103	
T10-20	TraesCS5A02G133000	79	100	362	2	6	188	
T10-20	TraesCS5B02G131900	74	94	9	0	118	12	
T10-20	TraesCS5D02G141200	86	0	4	229	0	117	
T10-21	TraesCS6A02G199100	59	69	333	7	2	9	
T10-21	TraesCS6B02G229400	47	59	45	5	107	80	
T10-21	TraesCS6D02G182800	37	2	7	73	0	54	
T10-22	TraesCS3A02G381500	73	92	205	0	0	25	
T10-22	TraesCS3B02G414200	48	54	0	0	90	0	
T10-22	TraesCS3D02G374700	56	0	2	148	0	72	
T10-23	TraesCS1A02G288700	22	35	104	0	0	0	
T10-24	TraesCS3A02G151300	45	49	124	0	2	1	
T10-25	TraesCS4A02G217700	49	50	158	0	6	23	
T10-25	TraesCS4B02G096400	82	113	0	0	204	1	
T10-25	TraesCS4D02G092800	16	0	0	54	1	7	
T10-26	TraesCS2A02G304900	57	62	255	49	73	97	
T10-26	TraesCS2B02G321700	55	85	77	37	157	77	
T10-26	TraesCS2D02G303500	69	24	60	163	120	123	
T10-28	TraesCS2A02G171900	34	23	63	0	2	1	
T10-29	TraesCS7B02G103700	44	46	1	0	99	22	
T10-29	TraesCS7D02G199800	40	0	70	124	2	4	
T10-3	TraesCS7A02G410300	61	72	75	0	98	12	
T10-3	TraesCS7B02G309700	73	83	74	0	104	230	
T10-3	TraesCS7D02G403500	64	0	77	178	21	42	
T10-30	TraesCS3A02G500600	36	41	119	1	0	36	
T10-31	TraesCS5A02G352800	58	61	173	0	19	21	
T10-31	TraesCS5B02G355000	48	45	0	1	45	32	
T10-31	TraesCS5D02G359800	58	2	2	139	20	74	
T10-32	TraesCS6B02G220900	98	118	26	13	268	21	
T10-32	TraesCS6D02G182400	102	11	40	241	91	164	
T10-34	TraesCS3A02G401700	74	94	281	1	76	55	
T10-34	TraesCS3B02G434600	66	90	3	0	62	2	
T10-34	TraesCS3D02G396500	40	0	10	93	50	30	
T10-35	TraesCS4B02G280400	57	69	1	0	2	24	
T10-35	TraesCS4D02G381900LC	14	0	0	33	0	0	
T10-36	TraesCS7A02G295800	26	29	87	1	10	3	
T10-36	TraesCS7D02G289400	39	3	3	144	1	145	
T10-37	TraesCS1A02G428700	21	30	63	0	19	95	
T10-37	TraesCS1B02G463400	39	37	4	0	18	48	
T10-37	TraesCS1D02G438000	37	0	80	100	39	114	
T10-38	TraesCS5A02G303200	35	45	189	12	17	9	
T10-38	TraesCS5B02G303500	48	64	0	5	60	16	
T10-38	TraesCS5D02G310000	27	0	0	74	25	40	
T10-39	TraesCS4D02G147700	44	0	11	103	30	74	
T10-4	TraesCS2A02G275100	54	73	248	8	26	43	
T10-4	TraesCS2B02G292500	48	63	3	13	114	48	
T10-4	TraesCS2D02G274100	68	10	12	138	71	88	
T10-40	TraesCS2A02G340400	41	45	109	0	2	10	
T10-40	TraesCS2B02G338300	13	18	3	0	20	13	
T10-40	TraesCS2D02G318100	18	0	1	46	3	12	
T10-41	TraesCS5A02G286500	12	10	53	0	3	3	
T10-41	TraesCS5B02G285900	31	30	0	0	68	18	
T10-41	TraesCS5D02G294200	38	0	59	107	16	52	
T10-42	TraesCS5A02G042200	53	48	363	0	81	28	
T10-42	TraesCS5B02G045200	52	48	3	0	10	78	
T10-42	TraesCS5D02G050300	42	0	18	110	73	37	
T10-43	TraesCS3A02G198600	62	85	314	4	5	34	
T10-43	TraesCS3B02G227200	91	87	1	2	171	5	
T10-43	TraesCS3D02G196500	80	4	35	160	120	150	
T10-44	TraesCS4A02G143200	81	100	284	0	0	0	
T10-44	TraesCS4B02G162200	51	61	12	1	162	47	
T10-44	TraesCS4D02G159600	72	24	5	166	30	122	
T10-45	TraesCS5A02G381700LC	27	10	54	8	0	4	
T10-45	TraesCS5B02G256100	10	10	0	0	3	6	
T10-46	TraesCS2A02G325700	29	36	128	0	2	16	
T10-46	TraesCS2B02G356200	41	41	3	0	76	5	
T10-46	TraesCS2D02G337000	55	0	32	116	67	25	
T10-47	TraesCS7A02G316600	35	45	121	0	0	0	
T10-47	TraesCS7B02G217600	27	24	0	0	34	8	
T10-47	TraesCS7D02G313500	22	0	0	62	10	38	
T10-48	TraesCS5A02G168500	67	78	269	0	70	51	
T10-48	TraesCS5B02G165300	76	83	8	7	157	61	
T10-48	TraesCS5D02G172800	60	8	37	150	148	105	
T10-5	TraesCS7A02G276600	35	44	28	0	0	51	
T10-5	TraesCS7B02G174300	24	26	1	0	54	0	
T10-5	TraesCS7D02G276600	81	3	94	217	47	48	
T10-6	TraesCS2A02G315000	28	22	59	0	15	10	
T10-6	TraesCS2B02G333500	26	21	1	0	11	3	
T10-6	TraesCS2D02G313300	33	0	2	104	18	33	
T10-7	TraesCS5A02G081400	38	34	108	4	8	3	
T10-7	TraesCS5B02G085000	35	38	8	0	81	1	
T10-7	TraesCS7D02G276600	81	3	94	217	47	48	
T10-8	TraesCS2B02G559100	53	65	2	35	10	7	
T10-8	TraesCS2D02G531600	41	0	4	25	16	11	
T10-9	TraesCS1A02G162200	58	80	262	2	4	45	
T10-9	TraesCS1B02G178800	47	66	44	13	161	62	
T10-9	TraesCS1D02G159000	73	4	59	145	68	73	
T6-10	TraesCS6A02G017500	31	28	106	1	12	13	
T6-10	TraesCS6B02G024900	27	33	2	1	27	31	
T6-10	TraesCS6D02G020700	31	0	3	79	31	4	
T6-11	TraesCS4A02G376700	74	65	0	0	0	1	
T6-11	TraesCS7D02G073700	52	0	0	160	62	16	
T6-12	TraesCS2B02G528300	18	26	45	8	54	10	
T6-12	TraesCS2D02G500600	23	0	5	45	42	31	
T6-13	TraesCS6A02G298100	45	49	144	0	14	20	
T6-13	TraesCS6B02G327500	52	52	8	0	126	25	
T6-13	TraesCS6D02G383600LC	63	2	27	155	54	67	
T6-14	TraesCS6A02G335400	94	105	399	0	106	20	
T6-14	TraesCS6B02G366100	93	112	1	0	205	32	
T6-14	TraesCS6D02G315300	81	0	0	269	76	138	
T6-15	TraesCS6A02G031100	42	69	201	8	47	27	
T6-15	TraesCS6B02G044100	44	49	19	0	62	21	
T6-16	TraesCS6A02G031200	58	73	318	0	74	15	
T6-16	TraesCS6B02G044000	52	71	2	1	55	14	
T6-16	TraesCS6D02G035600	59	9	16	153	107	126	
T6-17	TraesCS6A02G031000	42	31	138	0	33	11	
T6-17	TraesCS6B02G044200	51	54	39	0	28	39	
T6-17	TraesCS6D02G035700	40	1	30	115	26	44	
T6-18	TraesCS6A02G030700	43	34	166	0	75	11	
T6-18	TraesCS6B02G044500	36	21	0	1	1	15	
T6-18	TraesCS6D02G035900	47	12	0	131	48	26	
T6-2	TraesCS7A02G157700	87	83	287	0	72	48	
T6-2	TraesCS7B02G062100	56	66	34	0	164	41	
T6-2	TraesCS7D02G158300	79	2	8	187	121	166	
T6-31	TraesCS4A02G416400	78	115	325	0	0	81	
T6-31	TraesCS4A02G416300	65	83	295	0	11	72	
T6-31	TraesCS4B02G317200	68	92	0	0	125	36	
T6-4	TraesCS7A02G301700	35	43	139	0	25	14	
T6-4	TraesCS7B02G201900	58	49	8	1	138	44	
T6-4	TraesCS7D02G297000	38	0	0	104	38	39	
T6-6	TraesCS4B02G375800	48	51	3	0	6	39	
T6-6	TraesCS4D02G361500	69	0	1	192	49	39	
T6-6	TraesCS5A02G537100	39	57	174	1	0	1	
T6-7	TraesCS5A02G185700	42	50	168	0	6	8	
T6-7	TraesCS5B02G183800	49	61	3	0	102	9	
T6-7	TraesCS5D02G190800	56	0	2	147	39	66	
T6-8	TraesCS5A02G185600	50	50	169	0	2	4	
T6-8	TraesCS5B02G183700	56	66	1	135	44		
T6-8	TraesCS5D02G190700	31	0	0	79	0	26	
T6-9	TraesCS1A02G065700	61	65	254	0	35	38	
T6-9	TraesCS1B02G083900	59	52	0	18	101	34	
T6-9	TraesCS1D02G066700	42	0	20	125	63	20	
	AVERAGE DEPTH	50	60	180	130	119	65	
	StDev	20	26	109	53	88	37	
