# Supplementary material for: Exploring the diversity of promoter and 5′UTR sequences in ancestral, historic and modern wheat
Source: Plant Biotechnol J. 2021 Sep 16;19(12):2469–87. doi: 10.1111/pbi.13672 (PMC8633512; doi:10.1111/pbi.13672)
Supplement: Supplementary file 14 — Data S4 Hexaploid haplotypes. [file PBI-19-2469-s011.pdf]

Column	header	explanation	
A	**trait gene**	gene identifier, eg T1-1 refers to Trait 1 gene 1	
B	**homoeologue**	A, B, D homoeologues	
C	**hexaploids summary**	this includes a) how many cultivars have the same SNPs as Chinese Spring - if this number is above 70 it is shown in bold (larger font) b) how many have no SNPs (only if CS has SNPs) c) how many cultivars share the same haplotype, also includes explanations for specific IGV observations, eg: NO*	example: **T1-1A** 15var=CS, 46var=W115 , 3var=W203, 2var=W292, 10var=W624. This shows that 15 varieties are the same as CS (0 SNPs), 46 var have the same haplotype as W115 (4 SNPs, column I), 3 var have the same haplotype as W203 (5 SNPs, AV, HO&MW), 2 var have the same haplotype as W292 (5 SNPs, AB&UK) and 10 var are identical to W624.
D	**shared haplotypes**	value	
E	**unique haplotypes**	value	
F	**total haplotypes**	value	
G	**KR**	the durum wheat Kronos, unexpected presence in D homoeologue shown in red	
H	**CS**	individual (unexpected) SNPs in the CS promoter and 5'UTR shown in red	
I to V	**W115 - W786**	the Watkins landraces used	
W to CL	**AB - ZE**	the 68 commercial bread wheat varieites used	
G to CL	**colour coding**	cells filled with identical colours show that these cultivars/ accessions share the same haplotype. Dark green = zero SNPs (in most cases also =CS). **Please note**: the colour coding only shows identities per single rows (=homoeologues). The same colours in different rows do NOT indicate shared haplotypes across diffenet homoeologues.	
**abbreviations used throughout:**	**vsim**	very similar to	
	**NO**	NO_gene, absence of captured sequences	

SNP calling

For this analysis allele frequency was set to 1.0 to only view high-confidence homozygous SNPs. Also, SNPs with a coverage depth of less than 10 (less than 10 aligned sequenced fragments) were disregarded. Additionally, for accessions where the complete target sequence had a coverage depth of <10, the sequence was called ‘NO_gene’, but only if the corresponding depth for CS was >30. In a very few cases with only one or two baits, where even CS had a depth of <10, the whole target sequence was marked as ‘failed’.

Lengths of sequence captured

Sequence lengths were determined in IGV only for CS. Sequence length averages were thus only determined for CS across all analysed genes, and not across accessions for individual genes, but generally, most accessions exhibited similar lengths to CS. Sequence lengths were determined by using the ‘Marking Tool’ in IGV across the annotated 5’UTR and CDS, and for the putative promoters starting from the 5’end of the 5’UTR.

trait gene	homoeologue	hexaploids summary	shared haplotypes	unique haplotypes	total haplotypes	KR	CS	W115	W141
T1-1	A	15var=CS, 46var=W115 , 3var=W203, 10var=W624, 2var=W292,	5	3	8	1		4	
	B	23var=W141, 18var=W203, 16var=AM, 7var=BR, 5var=BU, 5var=W292,	6	6	12	4		3	2
	D	**80var=CS**	1	2	3	NO			
trait gene		hexaploids summary	shared haplotypes	unique haplotypes	total haplotypes		CS	W115	W141
T1-2	A	generally low coverage **81var=CS**	1	1	2				
	B	21var=CS, 49var=W141, 4var=W160, 4var=BA	4	4	8	12			1
trait gene		hexaploids summary	shared haplotypes	unique haplotypes	total haplotypes		CS	W115	W141
T1-3	A	**78var=CS**	1	4	5				
	B	25var=CS, 50var=W141, 7var=W387	3	0	3				2
	D	61var=CS, 19var=W292	2	2	4	NO			
trait gene		hexaploids summary	shared haplotypes	unique haplotypes	total haplotypes		CS	W115	W141
T1-4	A	16var=W203, 45var=W115, 4var=W141, 8var=W624, 8var=CG	5	2	7	1		12	12
	B	5var=CS, **76var=W115**	2	2	4	2		2	
	D	**74var=CS**	1	5	6	NO			
	D 2	**81var=CS**	1	1	2	NO			
trait gene		hexaploids summary		0			CS	W115	W141
T1-5	A	3 W **NO_SNPs, 79var=CS**	2	0	2	1	1		
	B	6var=CS, 2var=W115, **72var NO_GENE**	3	1	4			5	
	D	**81var=CS**	1	1	2	NO			
trait gene		hexaploids summary	shared haplotypes	unique haplotypes	total haplotypes		CS	W115	W141
T1-6	7A	46var=W115, 28var=W160, W246=TA	3	5		6		1	4
	1B	2var=CS, 9var=W141, 61var=	4	7	11	8		8	31
	7D	4var=CS, 66var=W115, 4var=W387,	4	5	9	NO		1	
trait gene		hexaploids summary	shared haplotypes	unique haplotypes	total haplotypes		CS	W115	W141
T1-7	A	**76var=CS**	1	6	7				
	B	**77var=CS**	1	2	3	1			
	D	**80var=CS**	1	2	3	NO			
trait gene		hexaploids summary	shared haplotypes	unique haplotypes	total haplotypes		CS	W115	W141
T1-8	4A	8var=CS, 8var=W115 (del1&2), 51var=W199 (no dels), 9var=W203, PA=W387 (only del2),	5	HW (only del1)	6	9		9, del1&2	
	7A	2var =CS, 13var=W115 (=KR),	4	7	11	26		26	10
trait gene		hexaploids summary	shared haplotypes	unique haplotypes	total haplotypes		CS	W115	W141
T1-9	A	70var=CS, 9var=W115, 3var=W292	3	0	3	2		2	
	B	73var=CS, 5var=W199,	2	3	5	2			
	D	**78var=CS**, W141=AP	2	2	4	NO			1
trait gene		hexaploids summary	shared haplotypes	unique haplotypes	total haplotypes		CS	W115	W141
T1-10	A	23var **NO_SNPs**, 4var=CS, 14var=W115,	7	6	13	2		2	4
	B	6var=CS, 66var=W141, 3var=W115	3	5	8	9		2	1
	D	**76var=CS**, 3var=CE	2	3	5	NO			1
trait gene		hexaploids summary	shared haplotypes	unique haplotypes	total haplotypes		CS	W115	W141
T1-11	A	41var=CS, 13var=W292, 20var=AB,	4	3	7	12			17
	B	15var NO_SNP, 30var=W115,	5	6	11	6		5	
	D	53var=CS, 27var=AL	2	2	4	NO			
trait gene		hexaploids summary	shared haplotypes	unique haplotypes	total haplotypes		CS	W115	W141
T1-12	A	75var=CS, 4var=KR	2	3	5	1			
	B	23var=NO_SNP, 4var=W115,	5	3	8	5		4	1
	D	69var=CS, 10var=SA	2	1	3	NO			
trait gene		hexaploids summary	shared haplotypes	unique haplotypes	total haplotypes		CS	W115	W141
T1-13	A	34var=CS, 5var=W160, 42var=AB	3	1	4	19			
	B	**none=CS**, 49var=W115, 15var=W160, 9var=W246, 6var=W292	4	4	8	18		17	
trait gene		hexaploids summary	shared haplotypes	unique haplotypes	total haplotypes		CS	W115	W141
T1-14	A	4var **NO_SNP**, **76var=CS**	2	2	4	10	3		
	B	73var=CS, 8var=W115	2	1	3	1		2	
	D	**80var=CS**	1	2	3	NO			
trait gene		hexaploids summary	shared haplotypes	unique haplotypes	total haplotypes		CS	W115	W141
T1-15	A	**80var=CS**	1	2	3				
	B	3var=CS, 54var=W115, 8var=W199,	5	5	10	3		3	4
	D	30var=CS, 48var=W160	2	2	4	NO			
trait gene		hexaploids summary	shared haplotypes	unique haplotypes	total haplotypes		CS	W115	W141
T1-16	B	18var=CS, 64var= W115	2	1	3			17	
	D	**76var=CS**, 3var=W786	2	1	3	NO			
trait gene		hexaploids summary	shared haplotypes	unique haplotypes	total haplotypes		CS	W115	W141
T1-17	D	**81var=CS**	1	1	2	NO			
trait gene		hexaploids summary	shared haplotypes	unique haplotypes	total haplotypes		CS	W115	W141
T1-18	B	4var=CS, 49var=W115, 7var=W203, 11var=W209, 2var=W246, W292=W387,	7	1	8			12	
	D	**79var=CS**	1	3	4	NO			
trait gene		hexaploids summary	shared haplotypes	unique haplotypes	total haplotypes		CS	W115	W141
T1-19	A	50var=CS, 20var=W199, W387=BW, 2var=W579, 3var=AM,	5	2	7	2	4		
	B	**73var=CS**, 4var=W292	2	4	6				
	D	51var=CS, 28var=AL	2	1	3	NO			
trait gene		hexaploids summary	shared haplotypes	unique haplotypes	total haplotypes		CS	W115	W141
T1-20	A	**75var=CS**, 5var=W115(=KR)	2	1	3	1		1	
	B	60var=CS, 3var=W141, 3var=W246,	5	6	11	3			14
	D	**75var=CS**, 3var=CO	2	2	4	NO			
trait gene		hexaploids summary	shared haplotypes	unique haplotypes	total haplotypes		CS	W115	W141
T1-21	A	10var **NO_SNP**, 68var=CS,	2	3	5	3	2		
	D	**80var=CS**	1	2	3	NO			
trait gene		hexaploids summary	shared haplotypes	unique haplotypes	total haplotypes		CS	W115	W141
T1-22	A	3var=NO_SNP, 56var=CS, 16var=W115, W199=TA,	4	4	8		3	2	
	D	80var=CS	1	2	3	NO			
trait gene		hexaploids summary	shared haplotypes	unique haplotypes	total haplotypes		CS	W115	W141
T1-23	A	**70var=CS**, 7var=BA, W199=AP	3	2	5				
	B	2var **NO_SNP** (W), 23var=W115,	3	4	7	14		10	
	D	**77var=CS**	1	4	5	NO			1
trait gene		hexaploids summary	shared haplotypes	unique haplotypes	total haplotypes		CS	W115	W141
T1-24	A	39var=CS, 43var=W246	2	0	2	5			
	B	2var **NO_SNP**, 23var=CS, **47var=W115**, 6var=BR	4	5	9	1		2	
trait gene		hexaploids summary	shared haplotypes	unique haplotypes	total haplotypes		CS	W115	W141
T1-25	A	63var=CS, 7var=W115, 9var=AM	3	1	4	1		1	
	B	50var **NO_SNP**, 8var=CS, 4var=W160,	4	2	6	NO			
	D	53var=CS, 22var=W209, W387=RI,	4	2	6	NO			
trait gene		hexaploids summary	shared haplotypes	unique haplotypes	total haplotypes		CS	W115	W141
T1-26	A	26var=CS, 10var=W292, 21var=AB,	6	4	10	17		1	
	B	**none=CS**, 41var=W115, 6var=W199,	4	4	8	16		11	
	D	51var=CS, 16var=W141, 6var=EI, 4var=FI,	4	4	8	NO			1
trait gene		hexaploids summary	shared haplotypes	unique haplotypes	total haplotypes		CS	W115	W141
T1-27	A	**only SU NO_SNPs**, 50var=CS, 4var=W199,	6	7	13	5	5		
	B	33var=CS, 7var=W141, 4var=W160,	5	9	14	2			2
trait gene		hexaploids summary	shared haplotypes	unique haplotypes	total haplotypes		CS	W115	W141
T1-28	2B	**only SU=CS**, 8var=W115, W199=W387,	4	3	7	39		39	
	3A	3var **NO_SNPs**, 34var=CS, 29var=W160,	4	2	6	40			
	3D	2var=CS, 77var=W115	2	1	3	NO		1	

trait gene		hexaploidsummary	shared haplotypes	unique haplotypes	total haplotypes	KR	CS	W115
T2-1	A	11var NO_SNPs, 68var=CS	2	2	4	2	2	
	B	26var=CS, 53var=W141	2	1	3	8		
	D	**78var=CS**	1	2	3	NO		
trait gene		hexaploidsummary	shared haplotypes	unique haplotypes	total haplotypes	KR	CS	W115
T2-2	5A	56var=CS, 8var =W387, **12var=M037**	3	4	7	5		
	5B	28var=CS, 42var=W141, 4var=CR	3	5	8			
	5D	79var=CS, GR=SO,	2	1	3	NO		
trait gene		hexaploidsummary	shared haplotypes	unique haplotypes	total haplotypes	KR	CS	W115
T2-3	1A	27var=W115, 14var=AM, 6var=W203, 2var=W292	4	4	8	16		5
	1B	54var=CS, 13var **NO_GENE**, 6var=CG	3	2	5	NO		NO
	1D	12var=CS, 62var=W115, 4var=W246	3	4	7	YES vfrag vsh prom		5, **del1**
trait gene		hexaploidsummary	shared haplotypes	unique haplotypes	total haplotypes	KR	CS	W115
T2-4	A	28var=KR, 5var=CS, AP=W199, 24var=AB, 5var=AL, BW=XI, ME=RO	7	12	19		11	10
	B	4var=CS, 22var=W115, 29var=W203, 4var=W199	4	0	4	15		8
	D	73var=CS, W624=SC	2	5	7	NO		
trait gene		hexaploidsummary	shared haplotypes	unique haplotypes	total haplotypes	KR	CS	W115
T2-5	A	BW **NO_SNPs**, 73var=CS, 8var=CO	2	3	5	3	1	
	B	71var **NO_SNPs**, 5var=W141, 5var=W199(KR)	3	3	6	12	11	
	D	69var=W115, 7var=W292, 4var=W141	3	3	6	NO		1
trait gene		hexaploidsummary	shared haplotypes	unique haplotypes	total haplotypes	KR	CS	W115
T2-6	A	58var=CS, 8var=AL, 6var=KR	3	4	7	YES 6		
	B	77var **NO_SNPs**, W292=CS, [illegible]	3	2	5	2		
	D	80var=CS	1	1	2	NO		
trait gene		hexaploidsummary	shared haplotypes	unique haplotypes	total haplotypes	KR	CS	W115
T2-7	A	ALLvar=CS	1	0	1			
	B	75var=CS, 2var=W246, 3var=GT	3	2	5	>50		
	D	80var=CS	1	2	3	NO		
trait gene		hexaploidsummary	shared haplotypes	unique haplotypes	total haplotypes	KR	CS	W115
T2-8	A	60var=CS, 22var=W141	2	0	2	1		
	B	78var=CS, 4var=W160	2	0	2			
	D	81var=CS	1	1	2	NO		
trait gene		hexaploidsummary	shared haplotypes	unique haplotypes	total haplotypes	KR	CS	W115
T2-9	A	4var=CS, 38var=KR, 37var=W624	3	2	5	1		
	B	5var=CS, 18var=KR, 11var=W160, W203=SS, 12var=W292, 9var=W387, 6var=AL, 5var=AV, SU=YU	9	7	16	2		21, 2dels
	D	66var=CS, 7var=W141, 5var=FL	3	3	6	NO		
trait gene		hexaploidsummary	shared haplotypes	unique haplotypes	total haplotypes	KR	CS	W115
T2-10	A	12var=CS, 42var NO_SNPs, [illegible]	5	9	14	1 [illegible]	1, CSdel1	3 [illegible]
	B	67var=CS, 12var=AB	2	2	4	YES 6		
	D	**79var=CS**	1	3	4	NO		
trait gene		hexaploidsummary	shared haplotypes	unique haplotypes	total haplotypes	KR	CS	W115
T2-11	A	74var=CS, 6var=KR, HO=MA,	3	0	3	0_SNPs, del1		
trait gene		hexaploidsummary	shared haplotypes	unique haplotypes	total haplotypes	KR	CS	W115
T2-12	A	17var=CS, 41var=W141, 4var=W209, 5var=W387(**=Tmon**), 2var=W624, 3var=GL,	6	4	10	4		
	D	16var=W115 (NO_SNPs), 61var=CS	2	2	4	NO		
trait gene		hexaploidsummary	shared haplotypes	unique haplotypes	total haplotypes	KR	CS	W115
T2-13	A	4var NO_SNPs, 32var=KR, [illegible]	4	6	10	2	1	2
	B	73var=CS, 6var=W115, 3var=W579	3	4	7	6		
	D	3var=CS, 63var=W141, 14var=W624	3	2	5	NO		
trait gene		hexaploidsummary	shared haplotypes	unique haplotypes	total haplotypes	KR	CS	W115
T2-14	A	51var=CS, 24var=W203, 6var=CG	3	2	5	6		
	B	2var=CS(0SNPs, 1del), 3var=W115, 58var=W141, 9var=W199, 2var=BU	5	3	8	2	1del	4
	D	73var=CS, 6var=W579, 2var=W387	3	1	4	NO		
trait gene		hexaploidsummary	shared haplotypes	unique haplotypes	total haplotypes	KR	CS	W115
T2-15	A	**81var=CS**	1	1	2			
	B	27var=CS, 43var=AB, 3var=W777, [illegible]	4	5	9			
trait gene		hexaploidsummary	shared haplotypes	unique haplotypes	total haplotypes	KR	CS	W115
T2-16	A	**ALLvar=CS**	1	0	1			
	B	4var=CS, 12var=W115, 10var=W141, 5var=W199, 48var=W203, W786=AB	6	1	7	2		2
trait gene		hexaploidsummary	shared haplotypes	unique haplotypes	total haplotypes	KR	CS	W115
T2-17	B	5var=CS, 2var=W786, 68var=W115, 6var=AM	4	0	4	10		10
	D	**79var=CS,**	1	3	4	NO		1
trait gene		hexaploidsummary	shared haplotypes	unique haplotypes	total haplotypes	KR	CS	W115
T2-18	A	34var=W115(=KR), 22var=W786, [illegible]	5	2	7		1	
	B	18var **NO_SNPs**, 24var=W115, [illegible]	7	7	14	5	5	3
	D	79var=CS	1	2	3			
trait gene		hexaploidsummary	shared haplotypes	unique haplotypes	total haplotypes	KR	CS	W115
T2-19	A	24var=CS, 41var=W246, 15var=BA	3	2	5	1		
	B	4var=CS, 40var=W115, 23var=W160, W246=W579, 4var=AB	5	6	11	2		16
trait gene		hexaploidsummary	shared haplotypes	unique haplotypes	total haplotypes	KR	CS	W115
T2-20	A	8var **NO_SNPs**, 32var=W115, [illegible]	5	5	10	9	12	9
	B	47var=CS, 14var=W209, 12var=W199,	3	7	10	0 del8		
	D	**74var=CS,** W246=W292	2	5	7	YES frag [illegible]		
trait gene		hexaploidsummary	shared haplotypes	unique haplotypes	total haplotypes	KR	CS	W115
T2-21	A	2var=CS, 67var=W141, 11var=W115,	3	1	4	6		4
	D	35var=CS, 10var=W115, 10var=W246, [illegible]	6	3	9	NO		1
trait gene		hexaploidsummary	shared haplotypes	unique haplotypes	total haplotypes	KR	CS	W115
T2-22	A	14var=0SNPs, 43var=CS, 11var=KR, W160=W387, 3var=NO_gene	5	9	14	6	13	
	B	2var=0SNPs, 19var=CS, 13var=W115, 2var=W141, 9var=W209, 15var=AL, 3var=CA	7	12	19		17	8
	D	25var=CS, 55var=W115	2	2	4	NO		12
trait gene		hexaploidsummary	shared haplotypes	unique haplotypes	total haplotypes	KR	CS	W115
T2-23	B	**80var=CS**	1	2	3	4		
	D	65var=CS, 14var=W203, 2var=FL	3	0	3	NO		
trait gene		hexaploidsummary	shared haplotypes	unique haplotypes	total haplotypes	KR	CS	W115
T2-24	A	**ALLvar=CS**	1	0	1			
	B	45var=W292, 23var **NO_SNPs**, [illegible]	3	4	7	2	2	2
	D	**81var=CS**	1	1	2	NO		
trait gene		hexaploidsummary	shared haplotypes	unique haplotypes	total haplotypes	KR	CS	W115
T2-25	A	53var=W115, 22var si**m to CS**, [illegible]	3	4	7	28	18	1
	B	10var=CS, 44var=W115, 11var=W160, [illegible]	4	5	9	20		25
	D	**81var=CS**	1	1	2	NO		
trait gene		hexaploidsummary	shared haplotypes	unique haplotypes	total haplotypes	KR	CS	W115
T2-26	B	**47var including CS NO_GENE,** [illegible]	5	0	5		NO	NO
	D	70var=CS, W203=W786, W624=TA, 2var=CA,	4	5	9	NO		
trait gene		hexaploidsummary	shared haplotypes	unique haplotypes	total haplotypes	KR	CS	W115
T2-27	A	60var=CS, 7var=W292, 10var=BA, [illegible]	4	1	5	14 [illegible]		
	B	15var=CS, 2var=W115, 50var=W203 [illegible]	5	2	7	19		8
	D	43var=CS, 31var=BA, 7var=W203	3	1	4	NO		
trait gene		hexaploidsummary	shared haplotypes	unique haplotypes	total haplotypes	KR	CS	W115
T2-28	B	21var=CS, 40var=W115, 8var=W387, [illegible]	4	5	9	18		1
	D	**81var=CS**	1	1	2	NO		
trait gene		hexaploidsummary	shared haplotypes	unique haplotypes	total haplotypes	KR	CS	W115
T2-29	A	**80var=CS**	1	2	3			
	B	74var=CS, BW=AP, NA=SP	3	4	7	6		
	D	77var=CS	1	3	4	NO		
trait gene		hexaploidsummary	shared haplotypes	unique haplotypes	total haplotypes	KR	CS	W115
T2-30	D	18var=CS, 62var=W115	2	1	3			1
trait gene		hexaploidsummary	shared haplotypes	unique haplotypes	total haplotypes	KR	CS	W115
T2-31	A	59var=CS, 19var=W199, UK=W786	3	2	5	1	12	
	B	38var=W203, 26var=CS, 7var=AB	3	10	13	5		
	D	58var=CS, 14var=W115, 4var=W141, [illegible]	4	2	6	NO		1
trait gene		hexaploidsummary	shared haplotypes	unique haplotypes	total haplotypes	KR	CS	W115
T2-32	B	20var=CS, 28var=W115, 31var=W199, FL=UK	4	1	5	2	6	5
	D	5var=CS, 73var=W115, W199=BW,	3	2	5			1
trait gene		hexaploidsummary	shared haplotypes	unique haplotypes	total haplotypes	KR	CS	W115
T2-33	A	5var=0SNPs, 38var=W115, 33var=W199,	3	5	8	16	21	
	B	**76var=CS**,	1	6	7			
	D	38var=CS, 42var=W141	2	2	4	NO		
trait gene		hexaploidsummary	shared haplotypes	unique haplotypes	total haplotypes	KR	CS	W115
T2-34	B	42var=CS, 37var=W160, W246=W579,	3	2	5	4		
	D	**82var=CS**	1	0	1	NO		
trait gene		hexaploidsummary	shared haplotypes	unique haplotypes	total haplotypes	KR	CS	W115
T2-35	A	80var=CS	1	2	3	1		
	B	32var=CS, 47var=NO_Gene	2	1	3			
	D	79var=CS, 2var=NO_gene	2	1	3	NO		
trait gene		hexaploidsummary	shared haplotypes	unique haplotypes	total haplotypes	KR	CS	W115
T2-36	B	**78var=CS,** W579 & HO **NO_GENE**	2	2	4	2		
trait gene		hexaploidsummary	shared haplotypes	unique haplotypes	total haplotypes	KR	CS	W115
T2-37	A	51var=CS, others have clusters [illegible]	1		>9	5		2
	B	**None=CS**, 20var=W160, 28var=AB, [illegible]	4	10	14	15		1
	D	76var= CS, 5var=W733	2	1	3	NO		
trait gene		hexaploidsummary	shared haplotypes	unique haplotypes	total haplotypes	KR	CS	W115
T2-39	A	**80var=CS**	1	1	2	1		
	B	16var=CS, 10var=W141, 33var=AB, [illegible]	4	0	4	1		
	D	**80var=CS**	1	2	3	NO		
trait gene		hexaploidsummary	shared haplotypes	unique haplotypes	total haplotypes	KR	CS	W115
T2-40	A	42var=CS, 10var=W115, 27var=W141, [illegible]	4	1	5	0 del2	8, [illegible]	10
trait gene		hexaploidsummary	shared haplotypes	unique haplotypes	total haplotypes	KR	CS	W115
T2-42	D	35var=CS,44var=W115	2	1	3	NO		1
trait gene		hexaploidsummary	shared haplotypes	unique haplotypes	total haplotypes	KR	CS	W115
T2-43	A	6var=KR, 49var=CS, 8var=W115, W777=W786, 6var=AB, 3var=BW	6	5	11		14	8
	B	50var=CS, 26var=**KR**,	2	4	6	1		
	D	31var=CS, 15var=W160, [illegible]	5	20	25	NO		2
trait gene		hexaploidsummary	shared haplotypes	unique haplotypes	total haplotypes	KR	CS	W115
T2-44	D	**81var=CS**	1	1	2	NO		
trait gene		hexaploidsummary	shared haplotypes	unique haplotypes	total haplotypes	KR	CS	W115
T2-45	A	15var **NO_SNPs**, 31var=W141, 10var=W160, 15var=AM, 4var=W199	5	5	10		9	26
	B	**70var=CS**, 6var=W733, 4=W579,	3	1	4			
	D	**69var=CS**, 8var=W199,	2	6	8	YES prox half del1 & 3		
trait gene		hexaploidsummary	shared haplotypes	unique haplotypes	total haplotypes	KR	CS	W115
T2-46	B	6var=CS, 10var=**KR**, 23var=W203, [illegible]	5	5	10	35 [illegible]		4
trait gene		hexaploidsummary	shared haplotypes	unique haplotypes	total haplotypes	KR	CS	W115
T2-47	A	46var=CS, 22var=W115, 9var=BW,	3	5	8		2	9
	B	3var=CS, 67var=KR, W160=BW, 4var=W777, HF=VE,	5	1	6	1		
trait gene		hexaploidsummary	shared haplotypes	unique haplotypes	total haplotypes	KR	CS	W115
T2-48	B-1	14var=CS, 40var=W115, 5var=W160, 8var=W624, W777=TA, GT=SA,	6	5	11			1
	B-2	15var=CS, 39var=W141, W203=SF, 2var=W292, **NO_gene: 1) only W777 absolutely NO capture, 2)15var=NO*=very short mid promoter fragments at low coverage 3)12var=NO# =very fragmented low coverage of whole promoter**	6	3	9	>50		NO*
	D	W786=AL, 60var=W141, 19var=CS,	3	1	4	NO		
trait gene		hexaploidsummary	shared haplotypes	unique haplotypes	total haplotypes	KR	CS	W115
T2-49	A	3var=CS, 12var=KR, 66var=W141	3	1	4	12		
	B	**73var=CS**, 4var=W160, 4var=BA	3	1	4	1		
	D	**77var=CS**, 4var=W160	2	1	3	NO		
trait gene		hexaploidsummary	shared haplotypes	unique haplotypes	total haplotypes	KR	CS	W115
T2-50	A	**75var=CS**, 4var=W115	2	3	5	1		1
	B	4var=CS, 4var=KR, 58var=W115, 2var=AL, 11var=NO_gene,	5	1	6	13		1
trait gene		hexaploidsummary	shared haplotypes	unique haplotypes	total haplotypes	KR	CS	W115
T2-51	A	45var=CS, 4var=KR, 4var=W160, 3var=W246, 21var=W624(=**Tmon**)	5	2	7	6		
	B	10var=CS, 21var=AB, 28var=AM, [illegible]	4	9	13	4 [illegible]		7 [illegible]
	D	15var=CS, 42var=W160, 21var=AB	3	3	6	NO		
trait gene		hexaploidsummary	shared haplotypes	unique haplotypes	total haplotypes	KR	CS	W115
T2-52	A	SU, YU **NO_SNPs**, 4var=W141, [illegible]	4	6	10	3	8	4
	B	51var=CS, 9var=W115, 18var=W203	3	3	6	1		35
	D	**79var=CS**	1	3	4	NO		
trait gene		hexaploidsummary		0		KR	CS	W115
T2-53	4A	**63var=CS**, 4var=W209, 3var=CA,	3	10	13	11		
	7A	**73var=CS**, W203=W292, W209=GC,	3	5	8			
	7D	**64var=CS**, 8var=AV, 8var=W579	3	1	4	NO		
trait gene		hexaploidsummary	shared haplotypes	unique haplotypes	total haplotypes	KR	CS	W115
T2-54	A	**72var=CS,** 7var=W115	2	2	4			18
	B	56var=CS, 20var=W115, 5var=W199	3	1	4	5 del1		1, KRdel1
	D	**81var=CS**	1	1	2	NO		
trait gene		hexaploidsummary	shared haplotypes	unique haplotypes	total haplotypes	KR	CS	W115
T2-55	A	27var=CS, **ALL others have KR del3&4:** [illegible]	2	5	7	12 del1-4		
	B	27var=AL, 20var=W115, 13var=W141, [illegible]	5	3	8	21 del1		15, W115 [illegible]
	D	53var=CS, TA=YU	2	>10	>12	NO		
trait gene		hexaploidsummary	shared haplotypes	unique haplotypes	total haplotypes	KR	CS	W115
T2-56	A	71var=CS, 4var=W141, VE=W199, [illegible]	4	3	7	11	10	12
	B	**69var=CS**, 8var=W115,	2	6	8	11 [illegible]		12, del1
	D	55var=CS, 26var=W141	2	1	3	NO		
trait gene		hexaploidsummary	shared haplotypes	unique haplotypes	total haplotypes	KR	CS	W115
T2-57	A	61var=CS, W777=CG, 17var=W786,	3	1	4	1		
	B	81var=CS,	1	1	2			
	D	60var=CS, 4var=W292, 14var=CL,	3	1	4	NO		
trait gene		hexaploidsummary	shared haplotypes	unique haplotypes	total haplotypes	KR	CS	W115
T2-58	A	23var=KR, 77var=CS	2	1	3		9	
	B	10var=0SNPs, 55var=CS, 3var=W141, W203=W246, 6var=W777	5	4	9	4	5	
trait gene		hexaploidsummary	shared haplotypes	unique haplotypes	total haplotypes	KR	CS	W115
T2-59	A	5var=KR, 2var=CS, 3var=GA, 69var=W141	4	2	6	10		
	B	57var=W115, 14var=CS, 4var=BA, [illegible]	4	1	5			9, [illegible]
	D	**78var=CS**	1	2	3	NO		
trait gene		hexaploidsummary	shared haplotypes	unique haplotypes	total haplotypes	KR	CS	W115
T2-60	A	**60var=CS**, 9var=W292, 4var=W199, [illegible]	4	2	6	8		1
	B	27var=CS, 49var=W209	2	10	12	2		
	D	54var=CS, 16var=AB, 5var=W387, [illegible]	4	4	8	NO		1
trait gene		hexaploidsummary	shared haplotypes	unique haplotypes	total haplotypes	KR	CS	W115
T2-61	A	23var=CS, 58var=W115	2	1	3			1
	B	4var=CS, 26var=KR, 49var=W115	3	1	4	3		3

trait gene	homoeologue	hexaploid ssummary	shared haplotypes	unique haplotypes	total haplotypes	KR	CS
T4-1	A	62var=CS, 8var=KR, W141=SO, W160=SL,	4	3	7	1	3
	B	2var=CS, 2var=GT, 77var=0SNPs	3	1	4	4	3
	D	80var=CS ,	1	2	3	NO	
T4-2	A	14var=0SNPs, 23var **sim** W115, 26 **sim** W160, W777=CS, 13var=W733, ***nb: var similar to Watkins fall into two clusters, BUT every var has unique SNPs**	3	2	5	1	1
	B	65var=W115, 9var=W141, 7var =W199, BR=CS,	4	1	5	14	
	D	0var=CS, 36var=W115, 44var=W141, 3=W292	3	0	3	40	
T4-3	B	42var=CS, 40var=W141	2	0	2	20, del1-5*	
	D	80var=CS	1	2	3	50	
T4-4	A	66var=CS, 12var=KR, CE=TA, BW**vsimTmon**	3	1	4	1	
	B (paralogue)	26var=CS, 24 NO_GENE, 17var=W160, 13var=W209,	4	0	4	40	
	D (paralogue)	69var=CS, 11var=W624	2	1	3	NO	
T4-5	A	59var=CS, 19var=W246,	2	2	4		
	B	17var=CS, 61var=W160,	2	4	6		
	D	81var=CS	1	1	2	NO	
T4-6	A	79var=CS, 3var=BW	2	0	2		
	B	del1 from [-629] in 81var, only partial del in CS and SU, 3var=CS, 58var=W115, 9var=W141, 8var=W199	4	1	5		
	D	79var=CS	1	2	3	NO	
T4-7	A	55var=CS, 4var=0 SNP , 8var=W624, 8var=CO, [illegible]	5	4	9	10	5
	B	77var=CS, 4var=W199, HU=NO_GENE	3	0	3		
	D	5var=CS, 63var=W160, 7var=W115, 6var=W141,	4	1	5	NO	
T4-8	A	3var 0 SNP or del, 79var=CS (with del)	2	1	3	1	del*
	B	only SU = CS, 71var=W141, 4var=BW	2	3	5		
	D	3var=CS, 76var=W115	2	3	5	NO	
T4-9	A	82var=CS	1	0	1		
	B	2var=CS, 62var=KR, W141=W160, 4var=W199, 2var=W387, 5var=**NO_gene**,	6	3	9	5	
	D	81var=CS	1	1	2	NO	
T4-10	A	11var=0SNPs, 71var=CS	2	1	3	15	8
	B	27var=CS, 37var=W203(=KR), W141=AP, 11var=AB	4	4	8	4	
	D	81var=CS	1	1	2	NO	
T4-11	A	*48var=CS [extremely low coverage (<5) called "NO Gene"], 28var=OSNPs(=KR), 3var=W141	3	1	4		NO*
	B	6var=CS, 55var=KR, 4var=W199, 3var=W246	4	11	15	10	
	D	54var=CS, 13var=W733, 9var=CSE	3	4	7	NO	
T4-12	A	80var=CS	1	1	2		
	B	5var=CS, 16var 0 SNP , 5var=W203, 43var=AB,	4	12	16	6	7
	D	74var=CS, 3var=W786	2	2	4	NO	
T4-13	A	24var=CS, 8var=W115, 31var=W203, 16var=W777,	4	3	7	13	
	B	38var 0 SNP , 42var=W141	2	3	5		1
	D	80var=CS	1	2	3	NO	
T4-14	A	30var=CS, 44=W160,	2	4	6		
	B	62var=CS, 17=W733	2	3	5		
	D	78var=CS, W209=W292	2	2	4	NO	
T4-15	1D	68var=CS, 10var=NO_GENE	2	6	8	NO	
	6A	12var=CS, 21var**VSIM** KR, 21var**VSIM** W115, 15var**VSIM** BA, GC=HW	5	0	5	17, del1-3*	
	6B	53var=CS, 17var=W292, 10var=W203,	3	2	5		
T4-16	A	8var 0 SNP , 63var=CS, 7var=CO,	3	2	5	4	4
	B	3var=CS, 79var=0SNP	2	0	2	3	3
	D	81var=CS,	1	1	2	9	
T4-17	A	12var=CS, 7var=W209, 2var=W115, 58var=W141	4	2	6	5	
	B	81var=CS	1	2	3		
	D	80var=CS	1	2	3	NO	
T4-18	4A	9var=CS, 14var=KR, 9var=W203, 32var=AB, 2var=AM, 2var=CA,	6	9	15	53	
	7A	13var=W115, 16var=W199, 3var=W387, 32var=AB	4	18	22	35	
	7D	10var=CS, 52var=W160, 7var=CL, 3var=FI,	4	8	12	YES fr[-825]	
T4-19	4A	4var(only W)=CS, CP=W199, 8var=W203, 68var=KR **NB: a large number of KR cultivars have extremely low coverage (<10) which could be considered "NO Gene"**	4	0	4	10	
	7A	W160=W209, 46var=W115, 34var=AB,	3	0	3	11	
	7D	66var=CS, 9var=W115, 5var=W141,	3	3	6	del1-3	
T4-20	4A	4 var 0 SNP, CP=W199, 4var=W203, 16var=AB, 6var=AL, CH=CR, 4var=CG, 2var=FI, 2var=FL, 2var=HW, MW=KSI	11	26	37	17	1
	7A	35 var **vSIM** AB **(=MDR043)**, 16var**sim**W115, 6varsimW199, 18var **vsim**W292	4	2	6	40 1_del	
	7D	61var=CS, 5var=W115, 9var=CL, 3var=FI	4	4	8	NO	
T4-21	A	49var=CS, 17var=AB, 15var=BA,	3	1	4		
	B	38var=CS, 39var=0SNPs, W209=W777,	3	2	5		6
	D	78var=CS	1	2	3	NO	
T4-22	A	45var=CS, 5var=W141, 32var=AB, 4var=W141	4	0	4		
	B	35var=CS, 33var=W160, 14var=W115	3	1	4		
	D	81var=CS	1	1	2	NO	
T4-23	A	50var=CS, 27var=AL, 3var=W141	3	3	6		
	B	33var=CS, 44var=W141, 3var=W203	3	2	5	14	
	D	81var=CS	1	1	2	NO	
T4-24	A	70var=W115, 10var=W141 (=KR),	2	3	5	1	21
	B	80var 0 SNP BUT deletion, only ST = CS (0 SNP [illegible]	2	1	3	1del *	
	D	75var=CS, CH=SL,	2	6	8	NO	
T4-25	A	14var=W115, 7var=KR(0SNPs), 45var=W160, BW=AP, 9var=IQ,	5	4	9		13
	B	80var=CS	1	2	3		
	D	80var=CS	1	2	3	NO	
T4-26	5A	75var=CS, 4var=W115,	2	2	4		
	4B	76var=CS,	1	4	5	8	
	4D	80var=CS	1	2	3	NO	
T4-28	5Bonly	3var=CS, 26var=W160, 3var=W199, W387=VE, 14var=W209, CP=W733, 11var=CL, HW=SU,	8	12	20		
T4-29	A	20var=CS, 42var=W141, 4var=CH, 4var=EI,	4	9	13	17, 1_del	
	B	9var=CS, 25var=KR, 10var=W160, 7var=W203, 12var=W199, 3var=CE, W292=TA, W141=CR,	8	5	13	1	
	D	77var=W115	1	5	6	NO	
T4-30	5A	68var=CS, 10var=W203,	2	4	6	22	
	4B	14var=CS, 65var=W160, BW=SU,	3	2	5	14	
	U	TA 0 SNP , 78var=CS	1	4	5	NO	1
T4-31	A	42var=CS, 37var=AB,	2	3	5	1	
	B	42var=CS, 28var=W141, W209=VE, W246=W579, W292=VA,	4	7	11	4, del1* del2**	
	D	79var=CS, SF=VA	2	1	3	NO	
T4-32	A	16var=CS, 47var=W141, 14var=W209,	3	3	6	3	
	B	65var=CS, 6var 0 SNP, 10var=W209	3	1	4		32
	B(2)	11var 0 SNP , 55var=W115,	2	7	9		4
	D	61var=CS, 15var=W209, W786=MH,	3	3	6	NO	
T4-33	4A	49var=0SNPs(=KR), 15var=CS, VE=W160, 8var=W203, RE=BW, CE=SO,	6	3	9		7
	7D	81var=CS	1	1	2	NO	
T4-34	A	73var=CS, 5var=BA, HW=SL,	3	2	5		
	D	81var=CS	1	1	2	NO	
T4-35	A	28var=CS, 51var=W141, W115=W209	3	2	5	4	1
	D	81var=CS	1	1	2	NO	
T4-36	A	12var=CS, 26var=W160, 3var=W579, 2var=W777, 9var=AL, 3var=BR, 3var=BU, 5var=DI, XI=YU	9	10	19	7	
	B	49var=CS, 2var=W141, 4var=W160, 4var=W199, 10var=W292, 3var=HO,	6	4	10	1	
T4-37	A	45var**NO_gene** [actually vlc, vfrag], W141=CP, 10var=W203, 2var=W733, 17var=AB, SF=SP	6	1	7	NO	
	D	81var=CS	1	1	2	NO	
T4-38	A	ALLvar=CS	1	0	1		
	B	4var=CS, 65var=KR/W115, 4var=W199, 9var=W292	4	0	4	2	
	D	81var=CS	1	1	2	3	
T4-39	A	4var=0SNPs, 70var=CS, 4var=CG	4	4	8	3	3
	B	22var=CS, 52var=W160, 2var=AL	3	4	7	2	
	D	81var=CS	1	1	2	NO	
T4-40	A	13var=CS, 3var=W115, 58var=W199, BW=VE, GL=RE	5	2	7	4	
	B	50var=CS, 18var=AB, 4var=BA, 4var=FL	4	2	6	9	
	D	53var=CS, 8var=AL, 19var=AM,	3	3	6	NO	1
T4-41	5A	46var 0 SNP , 34var=CS	2	2	4	12	10
	4B	24var=CS, 57var=W203	2	1	3	10	
T4-42	A	58var=CS, 22var=W115	2	2		14	12
	B	70var=CS, 3var=W292, W387=W777, W733=GT	3	4	7	2	
	D	52var=CS, 27var=AL	2	2	4	NO	
T4-43	A	70var=CS, 5var=W292, 3var=W733,	3	2	5	1	
	B	capture failed					
	D	81var=CS **[xlow coverage]**	1	1	2		
T4-44	A	36var=CS, 29var=W199, 3var=W141	3	2	5	1	
	B	26var=CS, 29var=W115,	2	1	3	0	
	D	4var 0 SNP , 75var=CS	2	3	5	NO	1
T4-45	A	8var NO_GENE, 17var=CS, 6var=W141, 43var=AB, [illegible]	5	3	8	15	
	B	29var=CS, 13var=W115, 12var=W203, 16var=AM, [illegible]	6	2	8		
	D	76var=CS, 4var=MK	2	2	4	NO	
T4-46	A	22var=CS, 50var=W199, 5var=BA	3	4	7	4	
	B	56var=CS, 22var=W115,	2	4	6	4	
	D	80var=CS	1	2	3	NO	
T4-47	A	64var 0 SNP , 5var=BA, 8var=CL, 5var=KA,	4	1	5	1	1
	B	55var=W141, 24var=W115, BW=VE	3	2	5	1 del1	
	D	49var=CS, 21var=AB, 4var=AM, 3var=AV	4	1	5	NO	
T4-48	A	39var=W115, 2var=W199, 14var=W292, 4var=AM, 6var=AV, 6var=BA, 2var=CG	7	2	9	3	4
	B	19var=CS, 12var=W115(=0SNPs), 40var=W199, DI=RB, 2var-MK	5	4	9	12	1
	D	53var=CS, 28var=W141	2	1	3	NO	
T4-49	A	55var=NO_GENE, 5var 0 SNP , 7var=W141, [illegible]	5	1	6	19 [illegible]	20
	B	67var=CS, 10var=W579, 4var=NO_GENE	3	1	4	9	
T4-50	A	28var=CS, 24var=AV, 14var=W579, 11var=W160	4	4	8	30	
	D	80var=CS	1	2	3		
T4-51	A	81var=CS,	1	1	2		
	B	63var=CS, 10var=AM, 4var=BA, 3var=W160,	4	2	6	9	
	D	79var=CS, W733=W786	2	1	3	NO	
T4-52	A	12var=CS, 45var=W141, 16var=CH, 5var=W579,	4	4	8	8	2
	B	20var=CS, 21var 0 SNP , 39var=AB	3	3	6	13	7
T4-53	A	11var=W199, 71var=W115	2	1	3		
	B	27var=CS, 53var=W115	2	2	4		
	D	78var=CS, 3var=W199	2	1	3	NO	
T4-54	A	4var=KR=0SNPs, 56var=CS, 2var=W199, 10var=W292,	4	9	13		6
	B	81var=CS, HO = 0 SNP GENE	1	1	2		
	D	77var=CS, 4var=W624	2	1	3	NO	
T4-55	A	3var=KR(=0SNPs), 67var=CS, 7var=IQ	3	4	12		6
	B	36var=CS, 14var=W141, 8var=W209, 2var=W292, 5var=W579, 3var=KSA, 2var=AP	7	6	13	1	
	D	62var=CS, 9var=AM, 3W vsim to MDR037, 3var NO_GENE	4	3	7	NO	
T4-56	A	68var=CS, 8var 0 SNP , 3var=BW	3	4	7		1
	B	7var 0 SNP , W141=AP, 58var=W160, KSI=KTR, 10var=NO_GENE	5	3	8	3	2
	D	63var=CS, 13var=W199, SU=YU	3	3	6	NO	
T4-57	A	ALLvar & KR = CS	1	0	1		
	B	3var=CS, 2var=W141 72var=W115(=KR)	3	2	5	8	
	D	67var=W115, AB=IS, 9var=CO, SU=VA=YU	4	3	7	NO	partial coverage of del1*
T4-58	A	67var=CS, 10var=W203	2	1	3	5 [illegible]	
	B	5var=CS, 8var=W115, W141=W160, 55var=W203, CH=CL	5	3	8	>30 3_del	2
	D	30var=CS, 27var=W115, 4var=W387, 9var=0SNPs, SF=ZE,	5	7	12	NO	14
T4-59	A	52var=W246, 16var=W115, 3var=W141, 6var=BA,	4	4	8	2	
	B	11var=CS, 17var=W203, 51var=W115	3	4	7	1	1
	D	77var=CS, W199=AP	2	3	5	NO	
T4-60	A	47var=CS, 30var=AB, W141=VE	3	3	6	8	
	B	63var=CS, 11var=W579, CG=OA,	3	3	6		
	U	78var=CS	1	3	4	NO	

trait gene		hexaploids summary	shared haplotypes	unique haplotypes	total haplotypes	KR	CS	W115	W141
T5-1	A	67var=CS, 6var=W115, 4var=W199, W786=PA	4	3	7		2	1	
	B	3var **NO_SNPs**, 26var=W115, 26var=W160, 14var=W199, 8var=CE	5	3	8	20		20	
	D	**80var=CS**	1	2	3	NO			
trait gene		hexaploids summary	shared haplotypes	unique haplotypes	total haplotypes	KR	CS	W115	W141
T5-2	A	**80var=CS**	1	2	3				
	B	4var=CS, **56var=KR**, 20var=W246	3	1	4	7		7	
	D	20var=CS, 61var=W115	2	1	3	NO		1	
trait gene		hexaploids summary	shared haplotypes	unique haplotypes	total haplotypes	KR	CS	W115	W141
T5-3	A	**72var=CS**, 3var=FL, 4W=W115(=KR), W246=GT	4	0	4	3		3	
	B	2var **NO_SNPs**, 64var=W115, 4var=BA, TA=VE, AP=YU	5	7	12	YES		2	
	D	**80var=CS**	1	2	3	NO			
trait gene		hexaploids summary	shared haplotypes	unique haplotypes	total haplotypes	KR	CS	W115	W141
T5-5	A	30var=**NO_SNPs**, 30var=AB, 3var=W246	3	1	4	3	6		
	B	**80var=CS**	1	2	3				
	D	**81var=CS**	1	1	2	NO			
trait gene		hexaploids summary	shared haplotypes	unique haplotypes	total haplotypes	KR	CS	W115	W141
T5-6	B	**74var=CS,** 5var=BW,	2	2	4				
	D	**79var=CS**, W160=W209	2	1	3	NO			
trait gene		hexaploids summary	shared haplotypes	unique haplotypes	total haplotypes	KR	CS	W115	W141
T5-7	B	**79var=CS**	1	3	4	7			
	D	**81var=CS**	1	1	2	YES			
trait gene		hexaploids summary	shared haplotypes	unique haplotypes	total haplotypes	KR	CS	W115	W141
T5-8	A	28var=CS, 13var=BW, 41var **NO_SNPs**	3	0	3		2 del1*		
	B	**72var=CS**, 7var=W115,	2	1	3	2		3	
	D	**81var=CS**	1	1	2	NO			
trait gene		hexaploids summary	shared haplotypes	unique haplotypes	total haplotypes	KR	CS	W115	W141
T5-9	D	**78var=CS**, W624=CP	2	2	4	NO			
trait gene		hexaploids summary	shared haplotypes	unique haplotypes	total haplotypes	KR	CS	W115	W141
T5-10	5A	best evidence for Tm Introgresion: [illegible]	4	2	6		4, del1*		
	4B	12var=CS, 9var=W115, 19var=W203, 18var=W246, 14var=W624	5	6	11	16, 3dels		13, 1del	
	1B	55var=CS, 10var=**NO_gene**, 3var=W777, 3var=BA, 6var=CL	5	2	7	1			
trait gene		hexaploids summary	shared haplotypes	unique haplotypes	total haplotypes	KR	CS	W115	W141
T5-11	A	23var=CS, 13var=W141, 36var=W160, [illegible]	4	3	7	6 [illegible]			1, [illegible]
	D	**81var=CS**	1	1	2	NO			
trait gene		hexaploids summary	shared haplotypes	unique haplotypes	total haplotypes	KR	CS	W115	W141
T5-12	A	11var=CS, W115=W160, 31var=W141, 12var=W292, 3var=BA, 10var=**NO_gene**,	6	5	11	23		7	22
	B	22var=CS, 3var=W199, 35var=W292, [illegible]	5	6	11				9
	D	**75var=CS**, 3var=W203,	2	4	6	NO		12	
trait gene		hexaploids summary	shared haplotypes	unique haplotypes	total haplotypes	KR	CS	W115	W141
T5-13	A	39var=CS, 13var=W292, 21var=BA, [illegible]	5	4	9	3			
	B	**81var=CS**, **HO=NO_GENE**	1	1	2				
	D	**81var=CS**	1	1	2	NO			
trait gene		hexaploids summary	shared haplotypes	unique haplotypes	total haplotypes	KR	CS	W115	W141
T5-14	A	30var=CS, 7var=**KR**, 26var=W203, 10var=CL,	4	7	11	13		13	
	B	38var=NO_SNP, 7var=W141, 16var=W160, 5var=DI, 8var=BA	5	5	10		1		2
	D	**81var=CS**	1	1	2	NO			
trait gene		hexaploids summary	shared haplotypes	unique haplotypes	total haplotypes	KR	CS	W115	W141
T5-15	A	40var=**NO_SNPs**, 40var=CS	2	3	5	5	5		
	B	**81var=CS**	1	1	2	9			
	D	77var=CS, W199=CP, W733=W786,	3	1	4	NO			
trait gene		hexaploids summary	shared haplotypes	unique haplotypes	total haplotypes	KR	CS	W115	W141
T5-16	A	29var=CS, 37var=AB, 14var=W203	3	2	5	8			
	B	9var=CS, 9var=W115, 28var=W141, [illegible]	6	11	17	14		14	12
	D	**78var=CS**, FI=MW	2	2	4	NO			
trait gene		hexaploids summary	shared haplotypes	unique haplotypes	total haplotypes	KR	CS	W115	W141
T5-19	A	10var=CS, 69var=W115	2	2	4	1 del1*		1	
	B	SU=CS, **68var=W115**, 5var=W777	3	8	11	7		6	
trait gene		hexaploids summary	shared haplotypes	unique haplotypes	total haplotypes	KR	CS	W115	W141
T5-20	A	**81var=CS**	1	1	2	1			
	B	**80var=CS**	1	2	3	2			
	D	**78var=CS**	1	4	5	NO			
trait gene		hexaploids summary	shared haplotypes	unique haplotypes	total haplotypes	KR	CS	W115	W141
T5-21	A	**68var=CS**, 12var=W292	2	1	3				
	B	29var=CS, 5var=W115, **45var=NO_GENE**	3	3	6	NO		6	NO
	D	**79var=CS**, AP=YU	2	1	3	NO			
trait gene		hexaploids summary	shared haplotypes	unique haplotypes	total haplotypes	KR	CS	W115	W141
T5-22	B	5var=CS, 12var=W115, 28var=W160, 8var=AV, 4var=AB, 12var=BU, 3var=**NO_gene** (*** 2 vsh cov**)	7	5	12	8		1	
	D	29var=CS, 10var=NO_GENE, 37var=AB,	3	2	5	NO			NO
trait gene		hexaploids summary	shared haplotypes	unique haplotypes	total haplotypes	KR	CS	W115	W141
T5-23	A	29var=KR, 41var=CS,	2	12	14	1del	11		
	B	65var=CS, 4var=KR, 4var=W115, 3var=W203	4	3	7	4 [illegible]		5	
	D	**75var=CS**, 4var=W786	2	1	3	NO			1
trait gene		hexaploids summary	shared haplotypes	unique haplotypes	total haplotypes	KR	CS	W115	W141
T5-24	A	64var=CS, 9var **NO_SNPs**, 3var=CE	3	5	8	4	4		
	B	28var=CS, 28var=W160, 3var=W786,	3	1	4				
	D	17var=CS, 63var=W115,	2	2	4	NO		1	
trait gene		hexaploids summary	shared haplotypes	unique haplotypes	total haplotypes	KR	CS	W115	W141
T5-25	A	5var **NO_SNPs**, 51var=CS, 5var=W387, [illegible]	4	1	5	1	1		
	B	25var=CS, 52var=W160,	2	3	5	9			
	D	**78var=CS**, 3var=W786	2	1	3	NO			
trait gene		hexaploids summary	shared haplotypes	unique haplotypes	total haplotypes	KR	CS	W115	W141
T5-26	D	72var=CS, **9var=**NO_gene,	2	1	3	YES dist prom			NO
trait gene		hexaploids summary	shared haplotypes	unique haplotypes	total haplotypes	KR	CS	W115	W141
T5-27	A	**73var=CS**, 8var=CL	2	1	3				
	B	53var=CS, 9var **NO_SNPs**, 4var=W115, [illegible]	5	1	6	2	2	2	2
	D	**80var=CS**	1	2	3	NO			
trait gene		hexaploids summary	shared haplotypes	unique haplotypes	total haplotypes	KR	CS	W115	W141
T5-28	A	W264=CS, 22var=W115, 4var=W160, [illegible]	5	15	20	YES [illegible]		9	8
	B	**12var=CS,** 6var=W115, 18var=W141, [illegible]	4		>5			18	
	D	**81var=CS**	1	1	2	NO			
trait gene		hexaploids summary	shared haplotypes	unique haplotypes	total haplotypes	KR	CS	W115	W141
T5-29	A	**81var=CS**	1	1	2				
	B	**73var=KR**, 5var=W199, 2var=CS	3	1	4	7		7	
	D	**77var=CS**, W579=W624	2	1	3	NO			
trait gene		hexaploids summary	shared haplotypes	unique haplotypes	total haplotypes	KR	CS	W115	W141
T5-30	A	69var=CS, 12var=W115	2	1	3	3		3	
	B	**55var=CS**, 7var=CL, 4var=W777, 4var=BA, **10var NO_GENE**	5	1	6	1			
	D	**79var=CS**	1	3	4	NO			

trait gene		hexaploids summary	shared haplotypes	unique haplotypes	total haplotypes	KR	CS	W115	W141	W160	W199	W203	W209	W246	W292	W387	W579	W624	W733	W777	W786	AB	AL	AM	AV	BA	BW	BR	BU	CA	CE	CH	CL	CP	CO	CG	CR	DI	EI	FI	FL	GA	GC	GT	GL	GR	HF	HW	HO	HU	IS	IQ	JB	KSA	KSL	KSI	KTR	MA	MH	MW	MK	ME	NA	OA	PA	PI	RE	RL	RV	RI	RB	RO	SA	SC	SS	SF	SO	SL	SP	ST	SU	TA	UK	AP	VA	VE	XI	YU	ZE
T6-1	A	78var=W115(=KR), CS=W624	2	2	4	1		1													2																																																									9																
(T9-63)	D	73var=CS, 4var=CA	2	5	7	NO											1		1		NO																																																									15									del1*, NO CDS/ UTR					
trait gene		hexaploids summary	shared haplotypes	unique haplotypes	total haplotypes	KR	CS	W115	W141	W160	W199	W203	W209	W246	W292	W387	W579	W624	W733	W777	W786	AB	AL	AM	AV	BA	BW	BR	BU	CA	CE	CH	CL	CP	CO	CG	CR	DI	EI	FI	FL	GA	GC	GT	GL	GR	HF	HW	HO	HU	IS	IQ	JB	KSA	KSL	KSI	KTR	MA	MH	MW	MK	ME	NA	OA	PA	PI	RE	RL	RV	RI	RB	RO	SA	SC	SS	SF	SO	SL	SP	ST	SU	TA	UK	AP	VA	VE	XI	YU	ZE
T6-2	7A	69var=CS, 11var=BR	2	1	3	14 del4#														1																																																																									
	B	10var=CS, 2var=0SNPs, 34var=W115, 3var=W199,	4	15	19	6	7	6			6	5			7					5														7	6																		NO																	3													5		5	1	4	4					
	D	78var=CS, JB=KSL	2	1	3	NO									1																																										1																																										
trait gene		hexaploids summary	shared haplotypes	unique haplotypes	total haplotypes	KR	CS	W115	W141	W160	W199	W203	W209	W246	W292	W387	W579	W624	W733	W777	W786	AB	AL	AM	AV	BA	BW	BR	BU	CA	CE	CH	CL	CP	CO	CG	CR	DI	EI	FI	FL	GA	GC	GT	GL	GR	HF	HW	HO	HU	IS	IQ	JB	KSA	KSL	KSI	KTR	MA	MH	MW	MK	ME	NA	OA	PA	PI	RE	RL	RV	RI	RB	RO	SA	SC	SS	SF	SO	SL	SP	ST	SU	TA	UK	AP	VA	VE	XI	YU	ZE
T6-3	A	20=NO_SNPs, 6=CS, 31var=NO_GENE, 8=W160, 6=W199 (=KR)	5	4	9	5 del1*	5			8	5		NO		1	NO				NO		NO	NO		NO			NO	NO	NO		7		NO	NO			NO	NO		NO				no SNPs, del3** In1>Ex2	NO	NO			NO			NO									NO							NO	NO	NO	NO		NO	NO		NO		NO			NO			NO		NO	NO	
(T9-64)	B	52var=CS, 5var=W203 (KR), UK=W209	3	3	6	9				8			1	1																																																																			8													
	D	79var=CS,	1	3	4	YES, del5-7																										5																																																	5													
trait gene		hexaploids summary	shared haplotypes	unique haplotypes	total haplotypes	KR	CS	W115	W141	W160	W199	W203	W209	W246	W292	W387	W579	W624	W733	W777	W786	AB	AL	AM	AV	BA	BW	BR	BU	CA	CE	CH	CL	CP	CO	CG	CR	DI	EI	FI	FL	GA	GC	GT	GL	GR	HF	HW	HO	HU	IS	IQ	JB	KSA	KSL	KSI	KTR	MA	MH	MW	MK	ME	NA	OA	PA	PI	RE	RL	RV	RI	RB	RO	SA	SC	SS	SF	SO	SL	SP	ST	SU	TA	UK	AP	VA	VE	XI	YU	ZE
T6-4	A	74var=CS, 7var=CO	2	0	2	4																													3																																																												
	B	17var=NO_SNPss, W141=W579, W199=W246, 48var=W203, W292=W387, 4var=AL, 5var=CG	7	2	9	5	5		1		5	1			4								4													2																																																				1						
	D	79var=CS, W387=GC	2	1	3	NO										1																																																																		9, del1*																
trait gene		hexaploids summary	shared haplotypes	unique haplotypes	total haplotypes	KR	CS	W115	W141	W160	W199	W203	W209	W246	W292	W387	W579	W624	W733	W777	W786	AB	AL	AM	AV	BA	BW	BR	BU	CA	CE	CH	CL	CP	CO	CG	CR	DI	EI	FI	FL	GA	GC	GT	GL	GR	HF	HW	HO	HU	IS	IQ	JB	KSA	KSL	KSI	KTR	MA	MH	MW	MK	ME	NA	OA	PA	PI	RE	RL	RV	RI	RB	RO	SA	SC	SS	SF	SO	SL	SP	ST	SU	TA	UK	AP	VA	VE	XI	YU	ZE
T6-5	A	W579=CS, 38var=W115, 25var=W199, W209=GC, 2var=GR,	5	8	13	4		4	4	3	4		3		2					4			5								3														3	3			3																						3																									
	B	76var=CS, 4var=CP	2	1	3	16		1																										7																																																														
	D	21var=CS, 31var=W115, 8var=W387, 19var=AM	4	2	6	NO		1		1						1			1					1																																																																				
trait gene		hexaploids summary	shared haplotypes	unique haplotypes	total haplotypes	KR	CS	W115	W141	W160	W199	W203	W209	W246	W292	W387	W579	W624	W733	W777	W786	AB	AL	AM	AV	BA	BW	BR	BU	CA	CE	CH	CL	CP	CO	CG	CR	DI	EI	FI	FL	GA	GC	GT	GL	GR	HF	HW	HO	HU	IS	IQ	JB	KSA	KSL	KSI	KTR	MA	MH	MW	MK	ME	NA	OA	PA	PI	RE	RL	RV	RI	RB	RO	SA	SC	SS	SF	SO	SL	SP	ST	SU	TA	UK	AP	VA	VE	XI	YU	ZE
T6-6	5A	67=CS, 2=W141, 6=W199, 5=AV,	4	3	7				10, del4#		1		9								8				7																																																										1												
	4B	78var=CS, 3=W292	2	1	3										1																																																															1															
	4D	70var=CS, 4=NO_GENE, 5=CE	3	4	7	NO								1	NO												NO				1																																																				19							NO	NO	no dist						
trait gene		hexaploids summary	shared haplotypes	unique haplotypes	total haplotypes	KR	CS	W115	W141	W160	W199	W203	W209	W246	W292	W387	W579	W624	W733	W777	W786	AB	AL	AM	AV	BA	BW	BR	BU	CA	CE	CH	CL	CP	CO	CG	CR	DI	EI	FI	FL	GA	GC	GT	GL	GR	HF	HW	HO	HU	IS	IQ	JB	KSA	KSL	KSI	KTR	MA	MH	MW	MK	ME	NA	OA	PA	PI	RE	RL	RV	RI	RB	RO	SA	SC	SS	SF	SO	SL	SP	ST	SU	TA	UK	AP	VA	VE	XI	YU	ZE
T6-7	A	ALL=CS	1	0	1																																																																																													
	B	51=W141, 2=W733, 25var=CS,	3	3	6				1		1del					1			2																																																												2															
	D	80var=CS	1	2	3	NO																									2																																																		6															
trait gene		hexaploids summary	shared haplotypes	unique haplotypes	total haplotypes	KR	CS	W115	W141	W160	W199	W203	W209	W246	W292	W387	W579	W624	W733	W777	W786	AB	AL	AM	AV	BA	BW	BR	BU	CA	CE	CH	CL	CP	CO	CG	CR	DI	EI	FI	FL	GA	GC	GT	GL	GR	HF	HW	HO	HU	IS	IQ	JB	KSA	KSL	KSI	KTR	MA	MH	MW	MK	ME	NA	OA	PA	PI	RE	RL	RV	RI	RB	RO	SA	SC	SS	SF	SO	SL	SP	ST	SU	TA	UK	AP	VA	VE	XI	YU	ZE
T6-8	A	36=CS, 45=W141,	2	1	3			1	1																																																																																						
	B	61=CS, 8=W203, 4=W624	3	9	12							1				2	1	1	17	13	18													1																			15																									13								15						
	D	76=CS, 2=W115	2	4	6	NO		poss ins				del in Ex1																			2																																																																		
trait gene		hexaploids summary	shared haplotypes	unique haplotypes	total haplotypes	KR	CS	W115	W141	W160	W199	W203	W209	W246	W292	W387	W579	W624	W733	W777	W786	AB	AL	AM	AV	BA	BW	BR	BU	CA	CE	CH	CL	CP	CO	CG	CR	DI	EI	FI	FL	GA	GC	GT	GL	GR	HF	HW	HO	HU	IS	IQ	JB	KSA	KSL	KSI	KTR	MA	MH	MW	MK	ME	NA	OA	PA	PI	RE	RL	RV	RI	RB	RO	SA	SC	SS	SF	SO	SL	SP	ST	SU	TA	UK	AP	VA	VE	XI	YU	ZE
T6-9	A	34=CS, 9=W141, 35=W203, 3=AW	4	0	4	13			9			1					7																					1																																																						
	B	48=CS, 2=W199, 17=W203, 10=AM (v	4	3	7						4	3												no prom,																																16	17																															5			
	D	37=CS, 37=W141,	2	7	9	NO			8, sh																																																																																						
trait gene		hexaploids summary	shared haplotypes	unique haplotypes	total haplotypes	KR	CS	W115	W141	W160	W199	W203	W209	W246	W292	W387	W579	W624	W733	W777	W786	AB	AL	AM	AV	BA	BW	BR	BU	CA	CE	CH	CL	CP	CO	CG	CR	DI	EI	FI	FL	GA	GC	GT	GL	GR	HF	HW	HO	HU	IS	IQ	JB	KSA	KSL	KSI	KTR	MA	MH	MW	MK	ME	NA	OA	PA	PI	RE	RL	RV	RI	RB	RO	SA	SC	SS	SF	SO	SL	SP	ST	SU	TA	UK	AP	VA	VE	XI	YU	ZE
T6-10	A	UK=CS, 41var=W115, 7var=W203,	4	6	10	11		1				8										11											5																																9		9					10	3						10															
	B	4var NO_SNPs, 51var=CS, 8var=W160,	4	4	8	14	17			16								9		17		18													16																																														16													
	D	80var=CS	1	2	3	NO																									8																																																														
trait gene		hexaploids summary	shared haplotypes	unique haplotypes	total haplotypes	KR	CS	W115	W141	W160	W199	W203	W209	W246	W292	W387	W579	W624	W733	W777	W786	AB	AL	AM	AV	BA	BW	BR	BU	CA	CE	CH	CL	CP	CO	CG	CR	DI	EI	FI	FL	GA	GC	GT	GL	GR	HF	HW	HO	HU	IS	IQ	JB	KSA	KSL	KSI	KTR	MA	MH	MW	MK	ME	NA	OA	PA	PI	RE	RL	RV	RI	RB	RO	SA	SC	SS	SF	SO	SL	SP	ST	SU	TA	UK	AP	VA	VE	XI	YU	ZE
T6-11	4A	9var NO_GENE (also all Tm), 72var=CS	2	1	3			NO						3	NO			NO		NO							NO							NO																																																					NO		NO		NO	
	7D	76var=CS	1	2	3	NO											1		1																																																													25															
trait gene		hexaploids summary	shared haplotypes	unique haplotypes	total haplotypes	KR	CS	W115	W141	W160	W199	W203	W209	W246	W292	W387	W579	W624	W733	W777	W786	AB	AL	AM	AV	BA	BW	BR	BU	CA	CE	CH	CL	CP	CO	CG	CR	DI	EI	FI	FL	GA	GC	GT	GL	GR	HF	HW	HO	HU	IS	IQ	JB	KSA	KSL	KSI	KTR	MA	MH	MW	MK	ME	NA	OA	PA	PI	RE	RL	RV	RI	RB	RO	SA	SC	SS	SF	SO	SL	SP	ST	SU	TA	UK	AP	VA	VE	XI	YU	ZE
T6-12	B							10, del1*		5									9 del1*			34, 1del lowcov																																																																								
	D	17=CS, W115=W209, 63var=W160	3	1	4	NO		1		50																																																																																				
trait gene		hexaploids summary	shared haplotypes	unique haplotypes	total haplotypes	KR	CS	W115	W141	W160	W199	W203	W209	W246	W292	W387	W579	W624	W733	W777	W786	AB	AL	AM	AV	BA	BW	BR	BU	CA	CE	CH	CL	CP	CO	CG	CR	DI	EI	FI	FL	GA	GC	GT	GL	GR	HF	HW	HO	HU	IS	IQ	JB	KSA	KSL	KSI	KTR	MA	MH	MW	MK	ME	NA	OA	PA	PI	RE	RL	RV	RI	RB	RO	SA	SC	SS	SF	SO	SL	SP	ST	SU	TA	UK	AP	VA	VE	XI	YU	ZE
T6-13	A	16var=CS, 38var=W115, 5var=W141,	4	3	7	10			10											4,					10																																					9																9													10					
	B	3var NO_SNPs&NO Ins, 5var=W141,	5	6	11	8		8					3				9	2	2	5		2					1																																																						7	2													
	D	73var=CS, 5var=AL	2	2	4	NO			1														1																																																							14															
trait gene		hexaploids summary	shared haplotypes	unique haplotypes	total haplotypes	KR	CS	W115	W141	W160	W199	W203	W209	W246	W292	W387	W579	W624	W733	W777	W786	AB	AL	AM	AV	BA	BW	BR	BU	CA	CE	CH	CL	CP	CO	CG	CR	DI	EI	FI	FL	GA	GC	GT	GL	GR	HF	HW	HO	HU	IS	IQ	JB	KSA	KSL	KSI	KTR	MA	MH	MW	MK	ME	NA	OA	PA	PI	RE	RL	RV	RI	RB	RO	SA	SC	SS	SF	SO	SL	SP	ST	SU	TA	UK	AP	VA	VE	XI	YU	ZE
T6-14	A	7var=CS, 4var=W115, 11var=W141,	8	13	21			14	13	12	15			14	12	11						15			14							14							16	13						16							14		14				14								13	14							9			14					low dist						
	B	26var=CS, 56var=W203,	2	0	2							2																																																																																	
	D	80var=CS	1	2	3	NO																																																																									20															
trait gene		hexaploids summary	shared haplotypes	unique haplotypes	total haplotypes	KR	CS	W115	W141	W160	W199	W203	W209	W246	W292	W387	W579	W624	W733	W777	W786	AB	AL	AM	AV	BA	BW	BR	BU	CA	CE	CH	CL	CP	CO	CG	CR	DI	EI	FI	FL	GA	GC	GT	GL	GR	HF	HW	HO	HU	IS	IQ	JB	KSA	KSL	KSI	KTR	MA	MH	MW	MK	ME	NA	OA	PA	PI	RE	RL	RV	RI	RB	RO	SA	SC	SS	SF	SO	SL	SP	ST	SU	TA	UK	AP	VA	VE	XI	YU	ZE
T6-15	A	NB: 1) unusually high SNPs in Watkins lines 2) very different cov pattern for MDR308 with only 3 SNPs 52var=CS, 12var=W115, 5var=W141, W246=W624, AL & HF=Tm	5	9	14	30											3						M045																																																																						
	B	NB: 1) unusually high SNPs in Watkins	3	15	18	20																																																																																									
trait gene		hexaploids summary	shared haplotypes	unique haplotypes	total haplotypes	KR	CS	W115	W141	W160	W199	W203	W209	W246	W292	W387	W579	W624	W733	W777	W786	AB	AL	AM	AV	BA	BW	BR	BU	CA	CE	CH	CL	CP	CO	CG	CR	DI	EI	FI	FL	GA	GC	GT	GL	GR	HF	HW	HO	HU	IS	IQ	JB	KSA	KSL	KSI	KTR	MA	MH	MW	MK	ME	NA	OA	PA	PI	RE	RL	RV	RI	RB	RO	SA	SC	SS	SF	SO	SL	SP	ST	SU	TA	UK	AP	VA	VE	XI	YU	ZE
T6-16	A	47var=CS, 9var=W115, 6var=W141,	8	1	9	40 del1*		41	43				40	42		40	M043															del2#																																																					42							
	B	53var=CS, 20=W203, 2=W624(=KR),	4	4	8	9						10					11	9 del1*																		12	4																						9															10																		
	D	76var=CS, GR=SU	2	2	4	NO					1																				1			1												1																																																
trait gene		hexaploids summary	shared haplotypes	unique haplotypes	total haplotypes	KR	CS	W115	W141	W160	W199	W203	W209	W246	W292	W387	W579	W624	W733	W777	W786	AB	AL	AM	AV	BA	BW	BR	BU	CA	CE	CH	CL	CP	CO	CG	CR	DI	EI	FI	FL	GA	GC	GT	GL	GR	HF	HW	HO	HU	IS	IQ	JB	KSA	KSL	KSI	KTR	MA	MH	MW	MK	ME	NA	OA	PA	PI	RE	RL	RV	RI	RB	RO	SA	SC	SS	SF	SO	SL	SP	ST	SU	TA	UK	AP	VA	VE	XI	YU	ZE
T6-17	A	53var=CS, 12var=W115, 10var=W141, 2var=W209, 2var=W579(**vsim Tmon**),	5	0	5	29, del1		28, del1									Tm																																																																													
	B	42var=CS, 11var=W199, 27var=W203	3	1	4	14					20	10																																																																	1															
	D	80var=CS	1	2	3	NO																									4																																																8, ins?															
trait gene		hexaploids summary	shared haplotypes	unique haplotypes	total haplotypes	KR	CS	W115	W141	W160	W199	W203	W209	W246	W292	W387	W579	W624	W733	W777	W786	AB	AL	AM	AV	BA	BW	BR	BU	CA	CE	CH	CL	CP	CO	CG	CR	DI	EI	FI	FL	GA	GC	GT	GL	GR	HF	HW	HO	HU	IS	IQ	JB	KSA	KSL	KSI	KTR	MA	MH	MW	MK	ME	NA	OA	PA	PI	RE	RL	RV	RI	RB	RO	SA	SC	SS	SF	SO	SL	SP	ST	SU	TA	UK	AP	VA	VE	XI	YU	ZE
T6-18	A	2var=CS, 13var=W115, 11var NO_GENE, 47var=W203, W246=SU, 2var=W579, FL=RL,	7	0	7	YES, 59, del1-2*		>40, Tm_del3, del4^	NO	NO		2 M037	NO	>40, sim to W115, no del4			1 M045										NO	NO									NO				1																																									NO						NO		NO		NO		NO	
trait gene		hexaploids summary	shared haplotypes	unique haplotypes	total haplotypes	KR	CS	W115	W141	W160	W199	W203	W209	W246	W292	W387	W579	W624	W733	W777	W786	AB	AL	AM	AV	BA	BW	BR	BU	CA	CE	CH	CL	CP	CO	CG	CR	DI	EI	FI	FL	GA	GC	GT	GL	GR	HF	HW	HO	HU	IS	IQ	JB	KSA	KSL	KSI	KTR	MA	MH	MW	MK	ME	NA	OA	PA	PI	RE	RL	RV	RI	RB	RO	SA	SC	SS	SF	SO	SL	SP	ST	SU	TA	UK	AP	VA	VE	XI	YU	ZE
T6-31	A	36var=CS, 19var=W160, 20var=AB, 2var=JB	4	3	7	5, del1,		KR_INS		2, KR_INS											1	3		2																													2																																												
	A	36var=CS, 39var=W160, 3var=CL,	4	1	5	4				3																							2																				4																							4													
	B	3var=CS, 23var=W141, 26var=W160,	5	7	12	4 del3**			2	4	3		2																						4																								3					3						3				1							3	3						

trait gene		hexaploids summary	shared haplotypes	unique haplotypes	total haplotypes	KR	CS	W115	W141	W160	W199	W203	W209	W246	W292	W387	W579	W624	W733	W777	W786	AB	AL	AM	AV	BA	BW	BR	BU	CA	CE	CH	CL	CP	CO	CG	CR	DI	EI	FI	FL	GA	GC	GT	GL	GR	HF	HW	HO	HU	IS	IQ	JB	KSA	KSL	KSI	KTR	MA	MH	MW	MK	ME	NA	OA	PA	PI	RE	RL	RV	RI	RB	RO	SA	SC	SS	SF	SO	SL	SP	ST	SU	TA	UK	AP	VA	VE	XI	YU	ZE
T8-1	A	54var=CS, 16var=AL, 11var=AM	3	0	3																		2	1																																																																
	B	2var NO_SNPs, 65var=KR, 4var=GR, 3var=W199	4	5	9	6		6			1								5								5																			5			5																									4	5														
	D	52var=CS, 22var=AB, 5var=CG	3	1	4	NO																1														1																																								8														
trait gene		hexaploids summary	shared haplotypes	unique haplotypes	total haplotypes	KR	CS	W115	W141	W160	W199	W203	W209	W246	W292	W387	W579	W624	W733	W777	W786	AB	AL	AM	AV	BA	BW	BR	BU	CA	CE	CH	CL	CP	CO	CG	CR	DI	EI	FI	FL	GA	GC	GT	GL	GR	HF	HW	HO	HU	IS	IQ	JB	KSA	KSL	KSI	KTR	MA	MH	MW	MK	ME	NA	OA	PA	PI	RE	RL	RV	RI	RB	RO	SA	SC	SS	SF	SO	SL	SP	ST	SU	TA	UK	AP	VA	VE	XI	YU	ZE
T8-2	A	4var NO_SNPs, 74var=CS, 3var=FL	3	1	4	8	9																																		8																																		6														
	B	7var=CS, 4var NO_GENE, 13var=W209, 34var=W246,	8	7	15			8	7	7		NO	8	8				7		NO			10				9					9								9				NO	8																									4					10								NO						7
	D	81var=CS	1	1	2	NO																																																																					5														
trait gene		hexaploids summary	shared haplotypes	unique haplotypes	total haplotypes	KR	CS	W115	W141	W160	W199	W203	W209	W246	W292	W387	W579	W624	W733	W777	W786	AB	AL	AM	AV	BA	BW	BR	BU	CA	CE	CH	CL	CP	CO	CG	CR	DI	EI	FI	FL	GA	GC	GT	GL	GR	HF	HW	HO	HU	IS	IQ	JB	KSA	KSL	KSI	KTR	MA	MH	MW	MK	ME	NA	OA	PA	PI	RE	RL	RV	RI	RB	RO	SA	SC	SS	SF	SO	SL	SP	ST	SU	TA	UK	AP	VA	VE	XI	YU	ZE
T8-3	A	37var=CS, 18var=W115(=KR), 18var=W141,	3	1	4	3			1												2																																																								2													
	B	27var=CS, 17var=W141, 36var=W292	3	2	5	2			1						2						1																																																																						1
	D	80var=CS	1	2	3	NO					1																																																																3														
trait gene		hexaploids summary	shared haplotypes	unique haplotypes	total haplotypes	KR	CS	W115	W141	W160	W199	W203	W209	W246	W292	W387	W579	W624	W733	W777	W786	AB	AL	AM	AV	BA	BW	BR	BU	CA	CE	CH	CL	CP	CO	CG	CR	DI	EI	FI	FL	GA	GC	GT	GL	GR	HF	HW	HO	HU	IS	IQ	JB	KSA	KSL	KSI	KTR	MA	MH	MW	MK	ME	NA	OA	PA	PI	RE	RL	RV	RI	RB	RO	SA	SC	SS	SF	SO	SL	SP	ST	SU	TA	UK	AP	VA	VE	XI	YU	ZE
T8-4	A	ALL=CS	1	0	1	3																																																																																		
	B	6var=CS, 52var=w115, 4var=W579, 3var=HF	4	1	5			2									3		3																																													1																								
	D	79var=CS	1	2	3	NO																																									1																													6														
trait gene		hexaploids summary	shared haplotypes	unique haplotypes	total haplotypes	KR	CS	W115	W141	W160	W199	W203	W209	W246	W292	W387	W579	W624	W733	W777	W786	AB	AL	AM	AV	BA	BW	BR	BU	CA	CE	CH	CL	CP	CO	CG	CR	DI	EI	FI	FL	GA	GC	GT	GL	GR	HF	HW	HO	HU	IS	IQ	JB	KSA	KSL	KSI	KTR	MA	MH	MW	MK	ME	NA	OA	PA	PI	RE	RL	RV	RI	RB	RO	SA	SC	SS	SF	SO	SL	SP	ST	SU	TA	UK	AP	VA	VE	XI	YU	ZE
T8-5	A	NO_SNPs anywhere, BUT: 1) poss insertion at [-638] for 28var	2	1	3			*Ins @ [-638]?													prox heavy coverage																																																																				
	B	31var=CS, 49var=W141	2	1	3	1			6							NO SNP, insertion @ [-1126]?																																																																									
	D	81var=CS	1	1	2	NO																																																																						1														
trait gene		hexaploids summary	shared haplotypes	unique haplotypes	total haplotypes	KR	CS	W115	W141	W160	W199	W203	W209	W246	W292	W387	W579	W624	W733	W777	W786	AB	AL	AM	AV	BA	BW	BR	BU	CA	CE	CH	CL	CP	CO	CG	CR	DI	EI	FI	FL	GA	GC	GT	GL	GR	HF	HW	HO	HU	IS	IQ	JB	KSA	KSL	KSI	KTR	MA	MH	MW	MK	ME	NA	OA	PA	PI	RE	RL	RV	RI	RB	RO	SA	SC	SS	SF	SO	SL	SP	ST	SU	TA	UK	AP	VA	VE	XI	YU	ZE
T8-6	A	50var=CS, 32var NO_SNPs	2	0	2		4																																																																																	
	B	81var=CS	1	1	2																																																																							2														
	D	81var=CS	1	1	2	NO																																																																						12, 3dels														
trait gene		hexaploids summary	shared haplotypes	unique haplotypes	total haplotypes	KR	CS	W115	W141	W160	W199	W203	W209	W246	W292	W387	W579	W624	W733	W777	W786	AB	AL	AM	AV	BA	BW	BR	BU	CA	CE	CH	CL	CP	CO	CG	CR	DI	EI	FI	FL	GA	GC	GT	GL	GR	HF	HW	HO	HU	IS	IQ	JB	KSA	KSL	KSI	KTR	MA	MH	MW	MK	ME	NA	OA	PA	PI	RE	RL	RV	RI	RB	RO	SA	SC	SS	SF	SO	SL	SP	ST	SU	TA	UK	AP	VA	VE	XI	YU	ZE
T8-7	A	55var=CS, 17var=W141, BW, EI, SC, SP, VE=vsim Tm	3	5	8				1							2			4	5							61 (M031 +11)							7					65																																					50	5				59							53			
	B	77var=CS, 4var=W733	2	0	2	1													40																																																																					
	D	79var=CS	1	3	4	NO								del1																																																												7							5							
trait gene		hexaploids summary	shared haplotypes	unique haplotypes	total haplotypes	KR	CS	W115	W141	W160	W199	W203	W209	W246	W292	W387	W579	W624	W733	W777	W786	AB	AL	AM	AV	BA	BW	BR	BU	CA	CE	CH	CL	CP	CO	CG	CR	DI	EI	FI	FL	GA	GC	GT	GL	GR	HF	HW	HO	HU	IS	IQ	JB	KSA	KSL	KSI	KTR	MA	MH	MW	MK	ME	NA	OA	PA	PI	RE	RL	RV	RI	RB	RO	SA	SC	SS	SF	SO	SL	SP	ST	SU	TA	UK	AP	VA	VE	XI	YU	ZE
T8-8	A	70var NO_SNPs, 11var=CS	2	0	2		2																																																																																	
	B	74var=W141 (=KR), 4var=W115,	3	1	4	1		11	1			2																																																																												
	D	81var=CS	1	1	2	NO	1																																																																																	
trait gene		hexaploids summary	shared haplotypes	unique haplotypes	total haplotypes	KR	CS	W115	W141	W160	W199	W203	W209	W246	W292	W387	W579	W624	W733	W777	W786	AB	AL	AM	AV	BA	BW	BR	BU	CA	CE	CH	CL	CP	CO	CG	CR	DI	EI	FI	FL	GA	GC	GT	GL	GR	HF	HW	HO	HU	IS	IQ	JB	KSA	KSL	KSI	KTR	MA	MH	MW	MK	ME	NA	OA	PA	PI	RE	RL	RV	RI	RB	RO	SA	SC	SS	SF	SO	SL	SP	ST	SU	TA	UK	AP	VA	VE	XI	YU	ZE
T8-9	A	76var=CS, 5var NO_SNPs	2	1	3	6		1												5																																																																				
	B	14var=CS, 34var=W115,	4	5	9	6		2	3					3			4		5															0_SNPs,																																								5						5								
trait gene		hexaploids summary	shared haplotypes	unique haplotypes	total haplotypes	KR	CS	W115	W141	W160	W199	W203	W209	W246	W292	W387	W579	W624	W733	W777	W786	AB	AL	AM	AV	BA	BW	BR	BU	CA	CE	CH	CL	CP	CO	CG	CR	DI	EI	FI	FL	GA	GC	GT	GL	GR	HF	HW	HO	HU	IS	IQ	JB	KSA	KSL	KSI	KTR	MA	MH	MW	MK	ME	NA	OA	PA	PI	RE	RL	RV	RI	RB	RO	SA	SC	SS	SF	SO	SL	SP	ST	SU	TA	UK	AP	VA	VE	XI	YU	ZE
T8-10	A	34var=CS, 38var=W141, 5var	4	0	4	3	3		3																				2																																																				3							
	B	79var=CS,	1	3	4	10															1						11																																																	1														
	D	78var=CS, W777=BA=CA	2	1	3	NO														1																																																								6 del1*														
trait gene		hexaploids summary	shared haplotypes	unique haplotypes	total haplotypes	KR	CS	W115	W141	W160	W199	W203	W209	W246	W292	W387	W579	W624	W733	W777	W786	AB	AL	AM	AV	BA	BW	BR	BU	CA	CE	CH	CL	CP	CO	CG	CR	DI	EI	FI	FL	GA	GC	GT	GL	GR	HF	HW	HO	HU	IS	IQ	JB	KSA	KSL	KSI	KTR	MA	MH	MW	MK	ME	NA	OA	PA	PI	RE	RL	RV	RI	RB	RO	SA	SC	SS	SF	SO	SL	SP	ST	SU	TA	UK	AP	VA	VE	XI	YU	ZE
T8-11	A	66var=KR, 14var=W141, W579=CS	3	0	3	1		1	2																																																																														
	B	5var=CS, 72var=W141,	2	2	4	7		4	5																																																																													4				
	D	79var=CS,	1	3	4	NO																																									cov only from																						cov from [-260]						3														
trait gene		hexaploids summary	shared haplotypes	unique haplotypes	total haplotypes	KR	CS	W115	W141	W160	W199	W203	W209	W246	W292	W387	W579	W624	W733	W777	W786	AB	AL	AM	AV	BA	BW	BR	BU	CA	CE	CH	CL	CP	CO	CG	CR	DI	EI	FI	FL	GA	GC	GT	GL	GR	HF	HW	HO	HU	IS	IQ	JB	KSA	KSL	KSI	KTR	MA	MH	MW	MK	ME	NA	OA	PA	PI	RE	RL	RV	RI	RB	RO	SA	SC	SS	SF	SO	SL	SP	ST	SU	TA	UK	AP	VA	VE	XI	YU	ZE
T8-12	6A	36var=CS, 38var=KR, IS, SO, AP=Tm	3	5	8	3		3			3					2																									1										Tm																2																		vlc from						
	B	5var=CS, 55var=W115, 4var=W199, 2var=W203, 2var=W387, BW=SF, BU=XI, CG=IS, nb: only 13 var do NOT have del1 (210bp from [-653])	8	4	12	23, del1		21, del1			1, no_del1	21, del1				21, del1					1						30, del1		21, del1							20, del1																																								23, del1		14, del1							15, del1					
	D	79var=W115(=ENT), W786=UK	2	2	4	NO		1													1																																																							28														
trait gene		hexaploids summary	shared haplotypes	unique haplotypes	total haplotypes	KR	CS	W115	W141	W160	W199	W203	W209	W246	W292	W387	W579	W624	W733	W777	W786	AB	AL	AM	AV	BA	BW	BR	BU	CA	CE	CH	CL	CP	CO	CG	CR	DI	EI	FI	FL	GA	GC	GT	GL	GR	HF	HW	HO	HU	IS	IQ	JB	KSA	KSL	KSI	KTR	MA	MH	MW	MK	ME	NA	OA	PA	PI	RE	RL	RV	RI	RB	RO	SA	SC	SS	SF	SO	SL	SP	ST	SU	TA	UK	AP	VA	VE	XI	YU	ZE
T8-13	A	79var=CS	1	2	3																													1																	3, ins																																							
	B	4var=CS, 22var=W115, 47var=W246,	4	1	5			3, del1*							4											4																																																			5														
	D	32var=CS, 47var=W387, TA=UK	3	1	4	NO										1																																																											10						1								
trait gene		hexaploids summary	shared haplotypes	unique haplotypes	total haplotypes	KR	CS	W115	W141	W160	W199	W203	W209	W246	W292	W387	W579	W624	W733	W777	W786	AB	AL	AM	AV	BA	BW	BR	BU	CA	CE	CH	CL	CP	CO	CG	CR	DI	EI	FI	FL	GA	GC	GT	GL	GR	HF	HW	HO	HU	IS	IQ	JB	KSA	KSL	KSI	KTR	MA	MH	MW	MK	ME	NA	OA	PA	PI	RE	RL	RV	RI	RB	RO	SA	SC	SS	SF	SO	SL	SP	ST	SU	TA	UK	AP	VA	VE	XI	YU	ZE
T8-14	A	60var=CS, 3var=W199, 10var=W292, 3var=RE	4	5	9	23	22				21				21	28											29																								23		21														CS -1																								
	B	73var=CS, 2var=W292, W387=W777, CP=FL	4	2	6										7	6																		1																	6																																						
	D	53var=CS, 24var=AL, CE=IS, MH=RO,	4	1	5	NO																	5								4																												4																		24, 1 del														
trait gene		hexaploids summary	shared haplotypes	unique haplotypes	total haplotypes	KR	CS	W115	W141	W160	W199	W203	W209	W246	W292	W387	W579	W624	W733	W777	W786	AB	AL	AM	AV	BA	BW	BR	BU	CA	CE	CH	CL	CP	CO	CG	CR	DI	EI	FI	FL	GA	GC	GT	GL	GR	HF	HW	HO	HU	IS	IQ	JB	KSA	KSL	KSI	KTR	MA	MH	MW	MK	ME	NA	OA	PA	PI	RE	RL	RV	RI	RB	RO	SA	SC	SS	SF	SO	SL	SP	ST	SU	TA	UK	AP	VA	VE	XI	YU	ZE
T8-15	A	20var NO_SNPs, 5var=CS,	3	1	4	1	1		1		2																																																										1																						
	B	18var=CS, 62var=W115	2	2	4			14														14																																																															14						
	D	81var=CS	1	1	2	NO																																																																																		
trait gene		hexaploids summary	shared haplotypes	unique haplotypes	total haplotypes	KR	CS	W115	W141	W160	W199	W203	W209	W246	W292	W387	W579	W624	W733	W777	W786	AB	AL	AM	AV	BA	BW	BR	BU	CA	CE	CH	CL	CP	CO	CG	CR	DI	EI	FI	FL	GA	GC	GT	GL	GR	HF	HW	HO	HU	IS	IQ	JB	KSA	KSL	KSI	KTR	MA	MH	MW	MK	ME	NA	OA	PA	PI	RE	RL	RV	RI	RB	RO	SA	SC	SS	SF	SO	SL	SP	ST	SU	TA	UK	AP	VA	VE	XI	YU	ZE
T8-17	B	6var=CS, 4var=KR, 72var NO_GENE	3	0	3	24		24		NO		NO	NO			NO		NO	NO	NO		NO	NO	NO	NO	NO		NO	NO	NO	NO	NO	NO	NO	NO	NO	NO	NO	NO	NO	NO	NO	NO	NO	NO	NO	NO	NO	NO	NO	NO	NO	NO	NO	NO	NO	NO	NO	NO	NO	NO	NO	NO	NO	NO	NO	NO	NO	NO	NO	NO	NO	NO	NO	NO		NO	NO	NO	NO	NO		NO	NO	NO	NO	NO	NO	NO	NO
	D	76var=CS, 5var NO_GENE	2	1	3	NO																						NO									NO								NO												NO																							NO												
trait gene		hexaploids summary	shared haplotypes	unique haplotypes	total haplotypes	KR	CS	W115	W141	W160	W199	W203	W209	W246	W292	W387	W579	W624	W733	W777	W786	AB	AL	AM	AV	BA	BW	BR	BU	CA	CE	CH	CL	CP	CO	CG	CR	DI	EI	FI	FL	GA	GC	GT	GL	GR	HF	HW	HO	HU	IS	IQ	JB	KSA	KSL	KSI	KTR	MA	MH	MW	MK	ME	NA	OA	PA	PI	RE	RL	RV	RI	RB	RO	SA	SC	SS	SF	SO	SL	SP	ST	SU	TA	UK	AP	VA	VE	XI	YU	ZE
T8-18	D	24var=CS, 57var=W160 (1 SNP)	2	1	3	NO				1																																																																																	
trait gene		hexaploids summary	shared haplotypes	unique haplotypes	total haplotypes	KR	CS	W115	W141	W160	W199	W203	W209	W246	W292	W387	W579	W624	W733	W777	W786	AB	AL	AM	AV	BA	BW	BR	BU	CA	CE	CH	CL	CP	CO	CG	CR	DI	EI	FI	FL	GA	GC	GT	GL	GR	HF	HW	HO	HU	IS	IQ	JB	KSA	KSL	KSI	KTR	MA	MH	MW	MK	ME	NA	OA	PA	PI	RE	RL	RV	RI	RB	RO	SA	SC	SS	SF	SO	SL	SP	ST	SU	TA	UK	AP	VA	VE	XI	YU	ZE
T8-19	A	8var=CS, 71var=KR,	2	3	5	3		3																			1																											2																															shc						
	B	10var=CS, 11var=KR, 52var=W115,	4	1	5	8		del1							7			8																																																																							
	D	77var=CS	1	4	5	YES							1				1del		3																																																									1														
trait gene		hexaploids summary	shared haplotypes	unique haplotypes	total haplotypes	KR	CS	W115	W141	W160	W199	W203	W209	W246	W292	W387	W579	W624	W733	W777	W786	AB	AL	AM	AV	BA	BW	BR	BU	CA	CE	CH	CL	CP	CO	CG	CR	DI	EI	FI	FL	GA	GC	GT	GL	GR	HF	HW	HO	HU	IS	IQ	JB	KSA	KSL	KSI	KTR	MA	MH	MW	MK	ME	NA	OA	PA	PI	RE	RL	RV	RI	RB	RO	SA	SC	SS	SF	SO	SL	SP	ST	SU	TA	UK	AP	VA	VE	XI	YU	ZE
T8-21	B	33var=CS, 10var=W141, 14var=AM, 4var=CG	4	13	17				24					22	1							24		25		23			23							24				24							24					24										24	23	23										24	>20, ins @ [-1001]?														
	D	78var=CS, 2W NO_GENE,	2	1	3	NO					NO					1					NO																																																							7														
trait gene		hexaploids summary	shared haplotypes	unique haplotypes	total haplotypes	KR	CS	W115	W141	W160	W199	W203	W209	W246	W292	W387	W579	W624	W733	W777	W786	AB	AL	AM	AV	BA	BW	BR	BU	CA	CE	CH	CL	CP	CO	CG	CR	DI	EI	FI	FL	GA	GC	GT	GL	GR	HF	HW	HO	HU	IS	IQ	JB	KSA	KSL	KSI	KTR	MA	MH	MW	MK	ME	NA	OA	PA	PI	RE	RL	RV	RI	RB	RO	SA	SC	SS	SF	SO	SL	SP	ST	SU	TA	UK	AP	VA	VE	XI	YU	ZE
T8-22	A	3var=CS, W141=AP, W160=W733,	7	8	15	14	12		13	12	11	13										11		11		14	12									12		12																12									12					11																					
	B	23var=CS, 29var=W292, 11var=AM,	5	7	12	16		10				6		12	8	6								1					7				9	9							10									7																																							
	D	77var=CS, W733=W786	2	3	5	NO					1																																																															9														
trait gene		hexaploids summary	shared haplotypes	unique haplotypes	total haplotypes	KR	CS	W115	W141	W160	W199	W203	W209	W246	W292	W387	W579	W624	W733	W777	W786	AB	AL	AM	AV	BA	BW	BR	BU	CA	CE	CH	CL	CP	CO	CG	CR	DI	EI	FI	FL	GA	GC	GT	GL	GR	HF	HW	HO	HU	IS	IQ	JB	KSA	KSL	KSI	KTR	MA	MH	MW	MK	ME	NA	OA	PA	PI	RE	RL	RV	RI	RB	RO	SA	SC	SS	SF	SO	SL	SP	ST	SU	TA	UK	AP	VA	VE	XI	YU	ZE
T8-23	B	W199=CS, 11var=W387(=KR),	5	6	11	1		8, del1*	2					6, NO	7	1																																																				7							3						7	vlc					6	
	D	81var=CS	1	1	2	NO																																																																																		
trait gene		hexaploids summary	shared haplotypes	unique haplotypes	total haplotypes	KR	CS	W115	W141	W160	W199	W203	W209	W246	W292	W387	W579	W624	W733	W777	W786	AB	AL	AM	AV	BA	BW	BR	BU	CA	CE	CH	CL	CP	CO	CG	CR	DI	EI	FI	FL	GA	GC	GT	GL	GR	HF	HW	HO	HU	IS	IQ	JB	KSA	KSL	KSI	KTR	MA	MH	MW	MK	ME	NA	OA	PA	PI	RE	RL	RV	RI	RB	RO	SA	SC	SS	SF	SO	SL	SP	ST	SU	TA	UK	AP	VA	VE	XI	YU	ZE
T8-24	A	56var=CS, 6var=W115, 14var=AB,	5	2	7	30		3														xlc, 2												2		NO	NO																																											2								
	B	78var=CS, W579 & W786 NO_GENE	2	2	4												NO		2		NO																																																																			
	D	81var=CS,	1	1	2	YES																																																																						NO														
trait gene		hexaploids summary	shared haplotypes	unique haplotypes	total haplotypes	KR	CS	W115	W141	W160	W199	W203	W209	W246	W292	W387	W579	W624	W733	W777	W786	AB	AL	AM	AV	BA	BW	BR	BU	CA	CE	CH	CL	CP	CO	CG	CR	DI	EI	FI	FL	GA	GC	GT	GL	GR	HF	HW	HO	HU	IS	IQ	JB	KSA	KSL	KSI	KTR	MA	MH	MW	MK	ME	NA	OA	PA	PI	RE	RL	RV	RI	RB	RO	SA	SC	SS	SF	SO	SL	SP	ST	SU	TA	UK	AP	VA	VE	XI	YU	ZE
T8-25	A	11var=CS, 16var=NO_GENE, 2var=W141, 16var=W786, 4var=W203, 2var=W292, GC=SP, nb: * all unique very similar to shared	7	25*	32	14		9	12	11	9	10	9	9	9		10	10		12		NO		NO		17				11			12	11		NO	NO	NO		NO		10	10		NO				9			10, ins?		NO	NO	NO	NO		10		NO		NO	9	10	NO	8	9	NO	11	12	9	NO		10								NO				8		
	B	20var=CS, 3var=W115, 2var=W160, 2var=W199, 32var=W203, 7var=AM, GA=SO, RL=RB	8	5	13	2		1		10	10	11						8	9					1																		10																							10	7		10	10																						
	D	13var=CS, 4var=W115, 11var=W160, 33var=W246, 3var=W209, 4var=CL, 4var=GL, SO=SL,	8	7	15	NO		5		10		8	11	6				2													10		9												5			3																											35		5					4	NO						
trait gene		hexaploids summary	shared haplotypes	unique haplotypes	total haplotypes	KR	CS	W115	W141	W160	W199	W203	W209	W246	W292	W387	W579	W624	W733	W777	W786	AB	AL	AM	AV	BA	BW	BR	BU	CA	CE	CH	CL	CP	CO	CG	CR	DI	EI	FI	FL	GA	GC	GT	GL	GR	HF	HW	HO	HU	IS	IQ	JB	KSA	KSL	KSI	KTR	MA	MH	MW	MK	ME	NA	OA	PA	PI	RE	RL	RV	RI	RB	RO	SA	SC	SS	SF	SO	SL	SP	ST	SU	TA	UK	AP	VA	VE	XI	YU	ZE
T8-26	A	45var=CS, 2var=CE, nb: * all unique very similar to each other	2	33*	35	10						14					9	8	1			17	15	13		13			15		1	15						15	12	14			11	19	15	15		15	15				15		15		16		15		16	15	14			12		12		13					4	12				11				8			12		
	D	79var=CS	1	3	4	17								8																	9																																													W246 -3/														
trait gene		hexaploids summary	shared haplotypes	unique haplotypes	total haplotypes	KR	CS	W115	W141	W160	W199	W203	W209	W246	W292	W387	W579	W624	W733	W777	W786	AB	AL	AM	AV	BA	BW	BR	BU	CA	CE	CH	CL	CP	CO	CG	CR	DI	EI	FI	FL	GA	GC	GT	GL	GR	HF	HW	HO	HU	IS	IQ	JB	KSA	KSL	KSI	KTR	MA	MH	MW	MK	ME	NA	OA	PA	PI	RE	RL	RV	RI	RB	RO	SA	SC	SS	SF	SO	SL	SP	ST	SU	TA	UK	AP	VA	VE	XI	YU	ZE
T8-27	A	3var=0SNPs, AL=CS, 5var=W115, 7var=W141, 16var=W160, BW=W209, 5var=W292, 26var=AV, BR=CL, 2var=CG,	10	5	15	7	3	5	6	4			6		8										3			13			7					14																													14			11	4								2								5					
(=T7-53)	B	80var=CS, HO NO_GENE	1	2	3	7																																											NO																																											
	D	80var=CS, YU NO_GENE	1	2	3	NO																																																																																			NO	

trait gene		hexaploids summary	shared haplotypes	unique haplotypes	total haplotypes	KR	CS	W115
T9-1	A	4var **NO_SNPs**, 74var=CS, W115=OA	3	1	4	1	1	1
	B	50var=CS, 29var=AM	2	1	3	5		
	D	**80var=CS**, W141=W624	2	0	2	NO		
trait gene		hexaploids summary	shared haplotypes	unique haplotypes	total haplotypes	KR	CS	W115
T9-3	A	**76var=CS**, 3var=W115	2	2	4	4		4
	B	4var=CS, 68var=W115, 3var=BW	3	6	9	3 del1*		4 del2**, KR_del1
	D	**81var=CS**	1	1	2	NO		
trait gene		hexaploids summary	shared haplotypes	unique haplotypes	total haplotypes	KR	CS	W115
T9-4	A	70var=CS, 12var=(W115)(=KR)	2	0	2	3		3
	D	**81var=CS**	1	1	2	NO		
trait gene		hexaploids summary	shared haplotypes	unique haplotypes	total haplotypes	KR	CS	W115
T9-5	A	CL **NO_SNPs**, **77var=CS**	1	5	6	1	1	2
	B	**77var=CS,** BW=W579	2	3	5	23		
	D	**78var=CS**	1	3	4	NO		
trait gene		hexaploids summary	shared haplotypes	unique haplotypes	total haplotypes	KR	CS	W115
T9-6	A	14var=CS, 17var=W160, **28var NO_SNPs**, 5var=GT, BW=AP, 13var=AB, 2var=FL, 4var=GT,	8	12	20		9, del1*	
	D	**73var=CS,** 6var=AB	2	3	5	NO		
trait gene		hexaploids summary	shared haplotypes	unique haplotypes	total haplotypes	KR	CS	W115
T9-7	A	46var=CS, 21var=W141, 7var=BU, CR=HW	4	6	10			1
	B	64var=CS, 3var=CA, W199=W246(=KR)	3	9	12	12		
	D	**81var=CS**	1	1	2	NO		
trait gene		hexaploids summary	shared haplotypes	unique haplotypes	total haplotypes	KR	CS	W115
T9-8	A	71var=CS, IS=SO=SU= NO_SNPs, KTR=RO	3	6	9	7	10	
	D	**75var=CS**, 5var=W160	2	2	4	NO		
trait gene		hexaploids summary	shared haplotypes	unique haplotypes	total haplotypes	KR	CS	W115
T9-9	A	56var=CS, 12var=W115, 8var=CL, BW **NO_SNPs**	3	6	9	3	4	3
	B	11var=NO_SNPs, 54var=CS, 13var=W203, **HO=**	3	2	5		1	
	D	**81var=CS**		2	2	NO		
trait gene		hexaploids summary	shared haplotypes	unique haplotypes	total haplotypes	KR	CS	W115
T9-10	A	29var=CS, 40var=W115(=KR), W203=HO,	6	4	10	11		
trait gene		hexaploids summary	shared haplotypes	unique haplotypes	total haplotypes	KR	CS	W115
T9-11-1	A	W246&W579=CS, 32var=W115, 43var=W199,	5	2	7	6		4
	B	**72var=CS**, 3var=W160, 3var=BA	3	3	6	5		
	D	**81var=CS**	1	1	2	NO		
trait gene		hexaploids summary	shared haplotypes	unique haplotypes	total haplotypes	KR	CS	W115
T9-12	A	39var **NO_SNPs**, 10var=W115, 7var=W141,	4	6	10	4	3	5
	B	66var=W115, 11var=AM, 4var=BA	3	0	3	3		1
	D	**79var=CS**	1	2	3	NO		
trait gene		hexaploids summary	shared haplotypes	unique haplotypes	total haplotypes	KR	CS	W115
T9-13	A	36var=CS, 2var=W199, 4var=AL, AP=XI	4	2	6			
	B	9var=W141, 22var=W115, 15var=W209,	5	3	8	4		3
	D	**81var=CS**	1	1	2	NO		
trait gene		hexaploids summary	shared haplotypes	unique haplotypes	total haplotypes	KR	CS	W115
T9-14	A	**ALLvar=CS**	1	0	1			
	B	**78var=CS**, W777=TA	2	2	4	3		
trait gene		hexaploids summary	shared haplotypes	unique haplotypes	total haplotypes	KR	CS	W115
T9-15	A	32var **NO_SNPs**, 5var=W115(=KR), 23var=CS,	3	3	6	15	7	
	B	3var **NO_SNPs**, 37var=W141, 32var=W115(=KR),	3	8	11	1	2	
	D	79var=W115(=ENT)	1	3	4	NO		1
trait gene		hexaploids summary	shared haplotypes	unique haplotypes	total haplotypes	KR	CS	W115
T9-16	A	68var **NO_SNPs**, 8var=W115, W777=W786,	3	4	7	13	14	1
	B	**78var=CS**, 2var=W160	2	1	3	9		
	D	**81var=CS**	1	0	1	NO		
trait gene		hexaploids summary	shared haplotypes	unique haplotypes	total haplotypes	KR	CS	W115
T9-17	A	**74var=CS**, 6var=BA	2	1	3			
	D	**80var=CS**	1	2	3	NO		
trait gene		hexaploids summary	shared haplotypes	unique haplotypes	total haplotypes	KR	CS	W115
T9-18	B	**76var=CS,** 3var=W387	2	2	4	3		
	D	**76var=W115,** 4var=AL	2	3	5	NO		1
trait gene		hexaploids summary	shared haplotypes	unique haplotypes	total haplotypes	KR	CS	W115
T9-19	A	81var=CS	1	1	2	1	1	
	B	45var=CS, 26var=W203, 5var=W292,	3	5	8	5		
	D	**73var=CS,** 7var=W115	2	1	3	NO		1
trait gene		hexaploids summary	shared haplotypes	unique haplotypes	total haplotypes	KR	CS	W115
T9-20	A	60var=CS, 7var=KR, 3var=W199, 10var=BR	4	3	7	1	2	1
	B	3var=CS, 14var=AB, 3var=AV, 4var=W199,	5	4	9	3		
	D	**81var=CS**,	1	1	2	NO		
trait gene		hexaploids summary	shared haplotypes	unique haplotypes	total haplotypes	KR	CS	W115
T9-21	A	36var=CS, 45var=W141	2	0	2			
	B	20var=CS, 34var=W115, 3var=W624, 19var=AB,	4	3	7			2
	D	41var=AM, 36var=W115,	2	2	4	NO		1
trait gene		hexaploids summary	shared haplotypes	unique haplotypes	total haplotypes	KR	CS	W115
T9-22	A	13var **NO_SNPs,** 37var=CS, 27var=W160,	3	4	7		3	
	B	40var=CS, 31var=W141, 3var=HF, 3var=IS	4	2	6	6		
	D	66var=W115, 12var=W160,	2	5	7	NO		2
trait gene		hexaploids summary	shared haplotypes	unique haplotypes	total haplotypes	KR	CS	W115
T9-23	A	**78var=CS**, 3var=W199	2	1	3	1		
	B	59var=CS, 14var=AB, 6var=CL	3	3	6			
	D	62var=CS, 3var=W387, 13var=CE,	3	2	5	NO		
trait gene		hexaploids summary	shared haplotypes	unique haplotypes	total haplotypes	KR	CS	W115
T9-24	A	48var=CS, 9var=W292, 2var=W579,	4	2	6	4		
	B	26var=CS, 35var=W160, 5var=CH,	3	4	7			
	D	**77var=CS**, 3var=W246	2	2	4	NO		
trait gene		hexaploids summary	shared haplotypes	unique haplotypes	total haplotypes	KR	CS	W115
T9-25	B	12var=CS, 64var=W292, W199=W203	3	3	6	1		
	D	21var=CS, 58var=W115,	2	2	4	NO		1
trait gene		hexaploids summary	shared haplotypes	unique haplotypes	total haplotypes	KR	CS	W115
T9-26	A	11var=CS, 38var NO_SNPs, 2var=W115,	5	7	12	18	18	16
	B	5var=CS, 12var=W115, 4var=W203, 22var=W292, 8var=W624, 2var=BW, 6var=CA,	10	5	15	12		11
	D	14var=CS, 18var NO_SNPs, W246=XI,	3			NO	5	
trait gene		hexaploids summary	shared haplotypes	unique haplotypes	total haplotypes	KR	CS	W115
T9-27	A	**81var=CS**	1	1	2			
	B	5var=CS, 47var=W115, 13var=W199,	5	2	7	6		6
	D	**81var=CS**	1	1	2	NO		
trait gene		hexaploids summary	shared haplotypes	unique haplotypes	total haplotypes	KR	CS	W115
T9-28	A	**75var=CS**, 4var=W160, JB=KSL,	3	0	3	9		
	B	**48var=CS,** 23var=W160, 3var=W203	3	6	9	1		
	D	73var=CS, 4var=W246, 3var=HO	3	2	5	NO		
trait gene		hexaploids summary	shared haplotypes	unique haplotypes	total haplotypes	KR	CS	W115
T9-29	A	**74var=CS,** 8var=W209,	2	0	2			
	B	**76var=CS**, W160=SU,	2	4	6			
	D	**65var=CS,** 12var=W160,	2	5	7	NO		
trait gene		hexaploids summary	shared haplotypes	unique haplotypes	total haplotypes	KR	CS	W115
T9-30	A	69var=CS, 6var NO_SNPs, 3var=CH,	3	3	6	21	5	
	B	**77var=CS**	1	5	6			
	D	49var=CS, 4var=W141, 28var=AL,	3	1	4	NO		
trait gene		hexaploids summary	shared haplotypes	unique haplotypes	total haplotypes	KR	CS	W115
T9-31	A	37var **NO_SNPs**, 27var=W199, W209=MW	3	15	18	18	18	17
	B	**81var=CS**	1	1	2	7		
	D	**78var=W115 (1 ENT SNP)**	1	4	5	NO		1
trait gene		hexaploids summary	shared haplotypes	unique haplotypes	total haplotypes	KR	CS	W115
T9-32	6A	56var=CS, 21=W115,	2	5	7			5
	B	7var=CS, 29var=W115, 3var=KR, 37var=W203,	4	3	7	2		1
	D	**81var=CS**	1	1	2	NO		
trait gene		hexaploids summary	shared haplotypes	unique haplotypes	total haplotypes	KR	CS	W115
T9-33	A	39var CS, 38var=W141	2	1	3	YES		
	B	69var=CS, 3var=W141, 10var=AM	3	4	7	YES		
	D	**73var=CS**, 3var=W579,	2	5	7	NO		
trait gene		hexaploids summary	shared haplotypes	unique haplotypes	total haplotypes	KR	CS	W115
T9-34	A	68var=CS, 2var=W115, 2W=W209,	3	5	8			3
	B	12var=CS, 45var=W115, BW=W199,	5	2	7	7		3
	D	33var=CS, 46var NO_SNPs,	2	2	4	NO	1	
trait gene		hexaploids summary	shared haplotypes	unique haplotypes	total haplotypes	KR	CS	W115
T9-35	A	**79var=CS**	1	3	4			
	D	**79var=CS**	1	3	4	NO		
trait gene		hexaploids summary	shared haplotypes	unique haplotypes	total haplotypes	KR	CS	W115
T9-36	A	17var NO_SNPs, 65var=CS	2	0	2		2	
	B	**76var NO_SNPs**, 3var=CS,	2	4	6	2	2	
	D	68var=CS, 9var=W160	2	4	6	NO		
trait gene		hexaploids summary	shared haplotypes	unique haplotypes	total haplotypes	KR	CS	W115
T9-37	A	ALLvar=CS	1	2	3			
	B	5var=CS, 53=W115, 8=W579, 7=CH, 3=PI	5	4	9	9		10
	D	**81var=NO_SNPs**	1	1	2	NO		
trait gene		hexaploids summary	shared haplotypes	unique haplotypes	total haplotypes	KR	CS	W115
T9-38	A	0var=NO_SNPs, 8var=W115,	1	-1				47, vsim
	B	33var=CS, 21=W141, 7=AM, 3var=NO_GENE,	5	>5	>10	16		
	D	0var=NO_SNPs, 75var=CS, W579=TA	2	5	7	NO	1	
trait gene		hexaploids summary	shared haplotypes	unique haplotypes	total haplotypes	KR	CS	W115
T9-39	A	**81var=CS**	1	1	2			
	D	**80=CS**	1	2	3	NO		
trait gene		hexaploids summary	shared haplotypes	unique haplotypes	total haplotypes	KR	CS	W115
T9-40	A	9=CS, 72=W115(=KR)	2	1	3	7		7
	B	**81=CS**	1	1	2			
	D	**76=CS,** 4=W115	2	2	4	NO		1
trait gene		hexaploids summary	shared haplotypes	unique haplotypes	total haplotypes	KR	CS	W115
T9-41	A	60=CS, 17=AL,	2	5	7			
	B	78=CS,	1	2	3			
	D	63=CS, 6=AB, 9=CH	3	1	4	NO		
trait gene		hexaploids summary	shared haplotypes	unique haplotypes	total haplotypes	KR	CS	W115
T9-42	A	19var=CS, 2var=W292, 4var=W115,	4	>10	>14	14		13
	B	4var=CS, 9var=AB, 7var=W199 (=KR), 17var	4	13	17	7		NO
	D	14var=CS, 66var=W160, W115=TA,	3	1	4	NO		8
trait gene		hexaploids summary	shared haplotypes	unique haplotypes	total haplotypes	KR	CS	W115
T9-43	A	58var=CS, 6var **NO_SNPs**, 12var=W624,	4	0	4	5	4	
	B	3var=CS, **75var=W115**	2	4	6	22		2
	D	**73var=CS**, 5var=AM	2	3	5	NO		
trait gene		hexaploids summary	shared haplotypes	unique haplotypes	total haplotypes	KR	CS	W115
T9-44	A	44var=CS, 35var=W203	2	3	5			10
	B	12var=W115, 8var=CS, 12var=W292,	6	7	13	8		31
	D	**79var=CS**	1	3	4	NO		no SNPs,
trait gene		hexaploids summary	shared haplotypes	unique haplotypes	total haplotypes	KR	CS	W115
T9-46	A	**73var=CS**,	1	9	10	21, del1*	21, del1*	
	B	20var=CS, 36var=W579(var=KR), 15var=W777,	4	8	12	8		
	D	**80var=CS**	1	2	3	NO		
trait gene		hexaploids summary	shared haplotypes	unique haplotypes	total haplotypes	KR	CS	W115
T9-47	A	15var=**NO_SNPs**, 66var=W141,	2	1	3	1	3	
	D	**80var=CS**	1	2	3	NO		
trait gene		hexaploids summary	shared haplotypes	unique haplotypes	total haplotypes	KR	CS	W115
T9-48	A	18var=W115(var=KR), 33var=W160,	2	>3	>5	1		1
	B	8var=CS, 3var=W199, 10var=W115,	5	5	10	4		4
	D	19var=CS, **62var NO_GENE**	2	1	3	NO		
trait gene		hexaploids summary	shared haplotypes	unique haplotypes	total haplotypes	KR	CS	W115
T9-49	A	40var=**NO_SNPs**, 21var=W246, 13var=BW,	4	3	7		7	
	B	5var=CS, 6var=W141, 46var=W292,	6	14	20	3		
	D	**78var=W115**	1	4	5	NO		1
trait gene		hexaploids summary	shared haplotypes	unique haplotypes	total haplotypes	KR	CS	W115
T9-50	A	18var=CS, 61var=W115	2	2	4	3		3,
	B	**78varvar=CS**	1	4	5			
	D	**80var=CS**	1	2	3	NO		
trait gene		hexaploids summary	shared haplotypes	unique haplotypes	total haplotypes	KR	CS	W115
T9-51	A	**72var=CS**, 7var=W777, 2var=W246,	3	1	4	22		
	B	**82var=CS**	1	0	1			
	D	**81var=CS**	1	1	2	NO		
trait gene		hexaploids summary	shared haplotypes	unique haplotypes	total haplotypes	KR	CS	W115
T9-52	A	69var=CS, 3var=W115, 8var=CO,	3	2	5	7		1
	B	4var=NO_SNP, 4var=CS, 7var=W115,	5	2	7	5	6	1
	D	**81var=CS**	1	1	2	NO		
trait gene		hexaploids summary	shared haplotypes	unique haplotypes	total haplotypes	KR	CS	W115
T9-53	A	14var=CS, 67var=W115	2	1	3	1		1
	B	6var=CS, 33var=W115, 13var=W160,	5	8	13	2		5
	D	**81var=CS**	1	1	2	NO		
trait gene		hexaploids summary	shared haplotypes	unique haplotypes	total haplotypes	KR	CS	W115
T9-54	A	10var=NO_SNP, 62var=CS, 5var=CG, 3var=EI	4	2	6	5 del1*	6, del1*	
	B	30var=CS, 3var=W160, 44var=W209,	4	0	4	7		
	D	**78var=CS**	1	4	5	NO		
trait gene		hexaploids summary	shared haplotypes	unique haplotypes	total haplotypes	KR		W115
T9-55	B	65var=CS, 14var=W115(var=KR)	2	3	5	3		3
trait gene		hexaploids summary	shared haplotypes	unique haplotypes	total haplotypes	KR	CS	W115
T9-56	A	W579var=CS, 42var=W115(var=KR),	3	6	9	13		13
	B	6var=CS, 44var=W115(var=KR), 23var=W141,	5	2	7	1		1
trait gene		hexaploids summary	shared haplotypes	unique haplotypes	total haplotypes	KR	CS	W115
T9-57	A	**78var=KR**, 2var=KSI	2	3	5	2	1	2
	B	15var=CS, 22var=AB, 3var=BW,	3	>5	>8			
	D	5var=CS, **75var=ENT**	2	1	3	NO		1
trait gene		hexaploids summary	shared haplotypes	unique haplotypes	total haplotypes	KR	CS	W115
T6-5=	A	38var=W115, 27var=W199, CE=DI,	3	15	18			5
	B	**76var=CS,** 4var=CP	2	1	3			1
	D	21var=CS, 31var=W115, 8var=W387, 19var=AM	4	2	6	4		1
trait gene		hexaploids summary	shared haplotypes	unique haplotypes	total haplotypes	KR	CS	W115
T9-59	A	71var=CS, 10var=W141	2	1	3	40 del1-3*		
	B	9var=CS, 44var=W115, 4var=W246, 6var=SP	4	7	11	10	11	9
	D	**77var=CS**,	1	4	5	NO		
trait gene		hexaploids summary	shared haplotypes	unique haplotypes	total haplotypes	KR	CS	W115
T9-60	A	**70var=CS**, 2var=W203, 2var=BW,	3	8	11	2 del1*		
	B	29var=CS, 41var=W141, 3var=W203,	3	7	10	18		
	D	**78var=CS**, 2var=W246	2	1	3	NO		
trait gene		hexaploids summary	shared haplotypes	unique haplotypes	total haplotypes	KR	CS	W115
T9-61	A	2var=CS, 28var=W115, 49var=W160	3	2	5	1		1
	B	3var=CS, 51var=W115, 7var=AM, 4var=W292,	4	9	13	17		30
	D	55var=CS, 10var=AM, 11var=W160,	3	3	6	NO	2	
trait gene		hexaploids summary	shared haplotypes	unique haplotypes	total haplotypes	KR	CS	W115
T9-63	A	1var=CS, **79var=W115(var=KR)**	2	2	4	1		1
	D	73var=CS, 4var=CA	2	5	7	NO		
trait gene		hexaploids summary	shared haplotypes	unique haplotypes	total haplotypes	KR	CS	W115
T9-64	A	6var=CS, 20var=NO_SNP, 31var=NO_GENE,	5	3	8	5	5	
	B	72var=CS, 8var=KR, 2var=W203,	3	3	6	9		
	D	**80var=CS**	1	2	3	NO		
trait gene		hexaploids summary	shared haplotypes	unique haplotypes	total haplotypes	KR	CS	W115
T9-65	B	3var=NO_SNP, **77var=W115**,	2	3	5		3	2
	D	**80var=CS**	1	2	3	NO		
trait gene		hexaploids summary	shared haplotypes	unique haplotypes	total haplotypes	KR	CS	W115
T9-66	A	50var=CS, 24var=W141, 4var=W199,	4	1	5			no SNPs,
	B	73var=CS, 7var=W579,	2	2	4	19		
	D	30var=CS, 49var=W115	2	2	4	NO		1
trait gene		hexaploids summary	shared haplotypes	unique haplotypes	total haplotypes	KR	CS	W115
T9-67	A	56var=CS, 18var=W141, 5var=W199	3	3	6	8	3	
	B	42var=W141, 4var=CS	2	>>3	>>3	16		19

trait gene		hexaploids summary	shared haplotypes	unique haplotypes	total haplotypes	KR	CS	W115
T10-1	A	67var=CS, 10var=W115 (**NO_SNPs, only del1)**	2	5	7	8, del1&2	8, del1&2	
	B	22var=CS, 8var=W141, 44var=W203, 3var=BW	4	3	7	1		
	D	65var=CS, 15var=AV (only **1 SNP**)	2	1	3	NO		
trait gene		hexaploids summary	shared haplotypes	unique haplotypes	total haplotypes	KR	CS	W115
T10-2	A	**75var=CS,** 7var=CO (only **1 SNP**)	2	0	2	1		
	D	25var=CS, 53var=W203	3	3	6	NO		
trait gene		hexaploids summary		0		KR	CS	W115
T10-3	A	60var=CS, 9var=W115, 4var=W209	3	6	9	14		17
	B	30var=CS, 39var=W141, 3var=AB, 4var=BW,	4	3	7			
	D	68var=CS, 10var=W141	2	4	6	NO		
trait gene		hexaploids summary	shared haplotypes	unique haplotypes	total haplotypes	KR	CS	W115
T10-5	A	68var=CS, 8var=W209	2	4	6	3		
	B	78var=CS, IS=SO	2	3	5			
	D	70var=CS, 10var=W141	2	2	4	NO		
trait gene		hexaploids summary	shared haplotypes	unique haplotypes	total haplotypes	KR	CS	W115
T10-6	A	3var **NO_SNPs**, 55var=CS, 18var=W786	3	6	9	5	4	
	B	80var=CS	1	2	3	19		
	D	49var=CS, 32var **NO_SNPs** but **Ins1**	2	4	6	NO		
trait gene		hexaploids summary	shared haplotypes	unique haplotypes	total haplotypes	KR	CS	W115
T10-7	5A	19var=CS, 60var=W115(=KR)	2	3	5	7		7
	5B	**77var=CS**, BU=CG	2	3	5			
	7D	70var=W115, 10var=W141, VE=CS	3	1	4	NO		1
trait gene		hexaploids summary	shared haplotypes	unique haplotypes	total haplotypes	KR	CS	W115
T10-8	2B	64var=CS, 7var=W115, W292=W777	3	6	9			4
	2D	73var=CS, 6var **NO gene**	2	3	5	NO		2
trait gene		hexaploids summary	shared haplotypes	unique haplotypes	total haplotypes	KR	CS	W115
T10-9	A	68var=CS, 6var=KR, 8var=W199	3	0	3	7		7
	B	59var=W115, 8var=W777, 2var=CS,	4	9	13	4		7
	D	**81var=CS**	1	1	2	NO		
trait gene		hexaploids summary	shared haplotypes	unique haplotypes	total haplotypes	KR	CS	W115
T10-10	A	36var=CS, 46var=W115	2	0	2			1
	B	29var=W141 (= KR), 32var=W160	2	23	25	18		19
	D	**80var=CS**	1	2	3	NO		
trait gene		hexaploids summary	shared haplotypes	unique haplotypes	total haplotypes	KR	CS	W115
T10-11	B	48var=CS, 33var **NO gene***	2	0	2			
	D	38var=CS, 10var=W160, 29var=W246	3	2	5	NO		
trait gene		hexaploids summary	shared haplotypes	unique haplotypes	total haplotypes	KR	CS	W115
T10-12	A	**81var=CS**	1	1	2			
	B	18var=CS, 58var=W203, 5var=W209	3	1	4	1		
trait gene		hexaploids summary	shared haplotypes	unique haplotypes	total haplotypes	KR	CS	W115
T10-13	A	**80var=CS,** W209=VE	2	0	2			
	B	69var=W115, 4var=W160, W733=W786,	4	3	7	3		3
	D	71var=CS, 9var=W203	2	2	4	NO		
trait gene		hexaploids summary	shared haplotypes	unique haplotypes	total haplotypes	KR	CS	W115
T10-14	A	71var=CS, 6var **NO_SNPs**,	2	5	7		3	
	B	3var **NO_SNPs**, 16var=W160, 3var=BW	3	10	13		1	
	D	**81var=CS**	1	1	2	NO		
trait gene		hexaploids summary	shared haplotypes	unique haplotypes	total haplotypes	KR	CS	W115
T10-15	B	**81var=CS**	1	1	2	8		
trait gene		hexaploids summary	shared haplotypes	unique haplotypes	total haplotypes	KR	CS	W115
T10-16	1B	23var=CS, 45var=W115, **10var NO GENE**	3	3	6	6		7
	1D	34var=CS, 36var=W141, 7var=BW,	5	1	6	NO		
trait gene		hexaploids summary	shared haplotypes	unique haplotypes	total haplotypes	KR	CS	W115
T10-17	B	SU=CS, 68var=W115, 4var=W160,	5	2	7	10		4
trait gene		hexaploids summary	shared haplotypes	unique haplotypes	total haplotypes	KR	CS	W115
T10-18	A	3var=CS, **76var=W115**	2	3	5	4		6
	D	**78var=CS**	1	4	5	NO		
trait gene		hexaploids summary	shared haplotypes	unique haplotypes	total haplotypes	KR	CS	W115
T10-19	A	5var=CS, 3var=W115, 58var=W141,	5	3	8			20
	B	62var=W141, 18var NO_SNPs	2	3	5		2	
	D	**80var=CS**	1	2	3	NO		
trait gene		hexaploids summary	shared haplotypes	unique haplotypes	total haplotypes	KR	CS	W115
T10-20	A	**80var=CS**, W203=MK	2	0	2			
	B	**81var=CS,**	1	1	2			
	D	**81var=CS,**	1	1	2	NO		
trait gene		hexaploids summary	shared haplotypes	unique haplotypes	total haplotypes	KR	CS	W115
T10-21	A	**ALLvar=CS**	1	0	1			
	B	8var=W115, 67var=W141, 3var=CS	3	2	5			2
	D	81var=CS,	1	1	2	NO		
trait gene		hexaploids summary	shared haplotypes	unique haplotypes	total haplotypes	KR	CS	W115
T10-22	A	81var=CS, VE 1 SNP	1	1	2			
	B	77var=CS, BW=AP (1 SNP)	2	2	4			
	D	77var=CS, W209=W579 (1 SNP)	2	2	4	NO		
trait gene		hexaploids summary	shared haplotypes	unique haplotypes	total haplotypes	KR	CS	W115
T10-23	A	10var=NO_SNPs, 60var=W115, 7var=W160,	3	1	4	6	1	6 del1*
trait gene		hexaploids summary	shared haplotypes	unique haplotypes	total haplotypes	KR	CS	W115
T10-24	A	10var=CS, 59var=W115, 11var=W160,	3	0	3	1		6,
trait gene		hexaploids summary	shared haplotypes	unique haplotypes	total haplotypes	KR	CS	W115
T10-25	A	80var=CS,	1	2	3			
	B	33var=CS, 42var=W141, 3var=W203	3	4	7	8		
	D	78var=CS, 3var=CA,	2	1	3	NO		
trait gene		hexaploids summary	shared haplotypes	unique haplotypes	total haplotypes	KR	CS	W115
T10-26	A	25var NO_SNPs, 53var=W115	2	5	7	10	10	11
	B	**81var=CS**	1	1	2	1		
	D	**79var=CS**	1	3	4	NO		
trait gene		hexaploids summary	shared haplotypes	unique haplotypes	total haplotypes	KR	CS	W115
T10-28	A	63var=CS, 16var=AL,	2	2	4	15		
trait gene		hexaploids summary	shared haplotypes	unique haplotypes	total haplotypes	KR	CS	W115
T10-29	B	78var=CS, W199=SS	2	1	3	18		
	D	76var=CS, TA=AP	2	2	4	NO		
trait gene		hexaploids summary	shared haplotypes	unique haplotypes	total haplotypes	KR	CS	W115
T10-30	A	63var=CS, 6var=CA, 7var=CL, 3var=W777	4	2	6	6		7
trait gene		hexaploids summary	shared haplotypes	unique haplotypes	total haplotypes	KR	CS	W115
T10-31	A	W387=0SNPs, 25var=CS, W115=CP, 52var=W160	3	1	4	11	26	20
	B	56var=CS, 3=W115, 16=W203, 3=AL	4	2	6			32
	D	81var=CS	1	1	2	NO		
trait gene		hexaploids summary	shared haplotypes	unique haplotypes	total haplotypes	KR	CS	W115
T10-32	U	16var=CS, 55var=W115, 8var=W141	3	1	4	1		2
	B	SU=CS, 42var=W141, 21=W199(=KR), 3var=GT, RL=UK	5	2	7	2		2
	D	81var=CS	1	1	2	NO		
trait gene		hexaploids summary	shared haplotypes	unique haplotypes	total haplotypes	KR	CS	W115
T10-34	A	78var=CS, W246=SS=SU	2	1	3			
	B	71var=CS, 9var=W115	2	1	3	11		13
	D	36var=CS, 43var=AB	2	2	4	NO		
trait gene		hexaploids summary	shared haplotypes	unique haplotypes	total haplotypes	KR	CS	W115
T10-35	B	80var=CS, W292=KR	2	1	3	2		
	D			0				
trait gene		hexaploids summary	shared haplotypes	unique haplotypes	total haplotypes	KR	CS	W115
T10-36	A	74var=CS, 2var=CO, 4var=HW	3	2	5	5		
	D	**79var=CS**, CA=UK	2	1	3	NO		
trait gene		hexaploids summary	shared haplotypes	unique haplotypes	total haplotypes	KR	CS	W115
T10-37	A	68var=CS, 10var=W199,	2	3	5			
	B	4var=CS, 2var=W141, 9var=W160,	6	7	13	20		19
	D	71var=CS, 9=W141	2	2	4	NO		
trait gene		hexaploids summary	shared haplotypes	unique haplotypes	total haplotypes	KR	CS	W115
T10-38	A	45var=CS, 31=W141, 4=W624	3	2	5			
	B	51var=CS, 9=W115, 6=W786	3	7	10	16		18
	D	37var=CS, 13=AV, 24=AB, 4=BW	4	4	8	NO		
trait gene		hexaploids summary	shared haplotypes	unique haplotypes	total haplotypes	KR	CS	W115
T10-39	D	74var=CS, 5=W777	2	3	5	NO		1
trait gene		hexaploids summary	shared haplotypes	unique haplotypes	total haplotypes	KR	CS	W115
T10-40	A	57var=CS, 21var=W777	2	4	6			
	B	69var=CS, 5var=BA,	2	8	10		2	1
	D	**79var=CS**	1	3	4	NO		
trait gene		hexaploids summary	shared haplotypes	unique haplotypes	total haplotypes	KR	CS	W115
T10-41	A	67var=CS, 7var=W203, 3var=W209,	5	0	5			
	B	3var=CS, 24var=W115, 2var=W199,	4	3	7	5		1
	D	80var=CS	1	2	3	NO		
trait gene		hexaploids summary	shared haplotypes	unique haplotypes	total haplotypes	KR	CS	W115
T10-42	6A	75var=CS, 5var=W292	2	2	4	11		2
	B	**none=CS**, 58var=W115, 13var=W199,	4	3	7	4		3
	D	44var=CS, 29var=AB, AV=MH, RB=TA	4	5	9	NO		
trait gene		hexaploids summary	shared haplotypes	unique haplotypes	total haplotypes	KR	CS	W115
T10-43	A	82var=CS	1	0	1			
	B	3var=CS, 69var=W115, 9var=W387	3	1	4	4		2
	D	**81var=CS**,	1	1	2	NO		
trait gene		hexaploids summary	shared haplotypes	unique haplotypes	total haplotypes	KR	CS	W115
T10-44	A	5var=CS, 66var=W141, 5var=W115, 2var=W579, 2var=CP	5	2	7		2	14
	B	**79var=CS**	1	3	4	3		
	D	**80var=CS**	1	2	3	NO		
trait gene		hexaploids summary	shared haplotypes	unique haplotypes	total haplotypes	KR	CS	W115
T10-45	A	2var=CS, 34var=W115, W160=W387,	4	>>4	>>4	40		18
	B	53var=CS, 11var=W199, 13var=W624,	3	5	8			
trait gene		hexaploids summary	shared haplotypes	unique haplotypes	total haplotypes	KR	CS	W115
T10-46	A	30var=0SNPs, 25var=AB, 19var=CS,	3	7	10		25	
	B	67var=CS, 5var=BA, 8var=AB,	3	2	5			
	D	**80var=CS**	1	2	3	NO		
trait gene		hexaploids summary	shared haplotypes	unique haplotypes	total haplotypes	KR	CS	W115
T10-47	A	73var=CS, 3var=W115, 4var=0SNP,	3	1	4	9	5	6
	B	46var=0SNP, 15var=W579,	2	>6	>8	4	8	
	D	**79var=CS**	1	3	4	NO		
trait gene		hexaploids summary	shared haplotypes	unique haplotypes	total haplotypes	KR	CS	W115
T10-48	A	60var=CS, 20var=AB	2	1	3			
	B	W579=CS, 73var=W115(=KR),	4	4	8	5		
	D	**77var=CS**,	1	5	6	YES		

trait gene	homoeologue	no. of cultivars	cultivars
T1-6	A	1	ST
T1-6	B	1	W203
T1-8	A	1	ST
T1-9	B	1	HO
T1-13	B	1	HO
T1-20	B	10	AM BW BR FI GT GL NA RL RI SA
T1-24	B	1	W209
T1-25	B	8	W246 W579 W777 BA CA MK SS XI
T1-26	B	1	HO
T1-28	B	68	W203 W579 W624 W733 W777 AB AL AM AV BA BR BU CA CE CH CL CO CG CR DI EI FI FL GA GC GT GL GR HF HW HO HU IS IQ JB KSA KSL KSI KTR MA MH MW MK ME NA OA PA PI RE RL RV RI RB RO SA SC SS SF SO SL SP ST TA UK AP VA XI ZE
T2-3	B	13	W115 W209 AM BW BR CP FI GT GL NA RL RI SA
T2-5	B	1	HO
T2-9	A	1	AL
T2-9	B	1	RV
T2-18	B	1	HO
T2-24	B	1	HO
T2-25	B	1	HO
T2-26	B	60	CS W115 W141 W160 W203 W209 W246 W292 W387 W624 W733 W777 CS W115 W141 W160 W203 W209 W246 W292 W387 W624 W733 W777 AL AM BA BW BU CE CH CL CO CG CR FI GC GL HF IS IQ KSL KSI KTR MK NA OA PI RE RV RO SA SC SS SF SO TA UK AP ZE
T2-28	B	1	RV
T2-32	B	1	HO
T2-35	B	46	W141 W160 W246 W579 AB AM BA BR BU CH CP CG CR DI EI FI FL GT GL HF HW HO HU IS IQ JB KSA KSL KSI KTR MH MK ME NA OA PI RF RL RV RI RO SC SL ST TA ZE
T2-36	B	2	W579 HO
T2-43	B	2	AV HO
T2-48	B	1	W777
T2-50	B	11	AM BW BR FI GT GL NA PI RL RI SA
T2-55	B	13	W199 W246 W579 W733 W786 FL GT JB KSL KSI KTR ME SU
T2-58	B	1	HO
T2-60	A	3	GC GT VE
T4-1	B	1	HO
T4-4	B	22	W199 W246 W579 W733 BA BR CA CP GL HW IQ MW PA RV RB SA SP SU TA UK AP XI
T4-7	B	1	HO
T4-8	B	1	W786
T4-11	B	1	HO
T4-14	A	15	CG KSA OA RE RO
T4-15	D	10	W115 W203 W387 W624 BW CP SU TA AP VE
T4-18	A	1	ST
T4-40	B	1	W199
T4-45	A	8	W115 W160 W292 CH CL CR RV ZE
T4-45	B	1	CP
T4-49	A	54	W203 W777 AB AL AM AV BR BU CH CL CO CG CR DI EI FI FL GA GC GT GL GR HF HW HO HU IS IQ JB KSA KSL KSI KTR MA MH MW MK ME NA OA PI RF RL RV RI RB RO SA SC SL SP ST YU ZE
T4-49	B	4	W733 W786 SF VA
T4-51	B	1	PI
T4-54	B	1	HO
T4-55	D	2	BW AP
T4-56	B	10	AM BW BR FI GT GL NA RL RI SA
T4-58	D	1	W786
T4-60	U	1	UK
T5-2	B	1	RV
T5-5	B	1	HO
T5-10	B	1	HO
T5-12	A	10	BW CE EI FL GR MW SA SO AP YU
T5-12	B	10	AM BW BR FI GT GL NA RL RI SA
T5-16	D	1	W786
T5-21	B	45	W141 AB AM BR CE CH CL CG CR DI EI FI GA GC GL GR HW HO IS IQ JB KSA KSL KSI KTR MA MH MK ME NA OA PI RF RL RV RI RB SA SF SO SL ST VA XI ZE
T5-22	D	10	W141 W624 CE FL HU JB ME PI RL SO
T5-27	B	1	HO
T5-30	B	10	AM BW BR FI GT GL NA RL RI SA
T6-1	D	1	W786
T6-3	A	31	W209 W387 W777 AB AL AV BR BU CA CP CO DI EI FL GR HF HU JB ME RV RI RB RO SC SS SO SP TA VA XI YU
T6-6	D	4	W292 BW SU TA
T6-11	A	9	W115 W292 W624 W777 BW CP AP VE YU
T6-18	A	11	W141 W160 W209 BW BR CR SS TA AP VE YU
T8-2	B	4	W203 W777 GT UK
T8-17	B	72	W160 W203 W209 W387 W624 W733 W777 AB AL AM AV BA BR BU CA CE CH CL CP CO CG CR DI EI FI FL GA GC GT GL GR HF HW HO HU IS IQ JB KSA KSL KSI KTR MA MH MW MK ME NA OA PA PI RE RL RV RI RB RO SA SC SF SO SL SP ST TA UK AP VA VE XI YU ZE
T8-21	D	2	W199 W786
T8-24	A	2	CG CR
T8-24	B	2	CG CR
T8-24	D	1	SS
T8-25	A	16	AB AM CG CR DI FI GL KSA KSL KSI KTR MK NA PI RV SA
T8-27	B	1	HO
T8-27	D	1	YU
T9-9	B	1	HO
T9-25	D	1	TA
T9-26	D	1	W292
T9-33	B	1	W292
T9-38	B	2	AL CP
T9-42	B	16	W115 W160 W203 W209 W387 W733 BA BW CA CO PA SF SP TA AP VE
T9-48	D	63	W160 W203 W292 W579 W624 W733 W777 W786 AB AL AM AV BA BR BU CA CE CH CL CO CG CR DI EI FI FL GA GC GT GL GR HF HW HO HU IQ JB KSA KSL KSI KTR MA MH MW MK ME NA OA RE RL RV RI RB RO SA SC SF SL SP ST VA XI ZE
T9-50	B	1	HO
T9-59	B	1	HO
T9-64	A	31	W203 W292 W733 W786 AB AM BW BR BU CL CP CR DI FI GL GR HO IQ MK RL RV RI RB SA SC SF SL SU AP VE XI
T10-5	B	1	HO
T10-7	B	1	HO
T10-8	D	6	W160 W292 W579 W624 W733 SS
T10-11	B	33	W246 W292 W579 W624 W733 W777 W786 AB AM BA BR CA CH CG EI FL GA GL HW HO KSL MK NA PA RL SS SF SP SU VA VE XI YU
T10-16	B	10	AM BW BR FI GT GL NA RL RI SA
T10-29	B	1	HO
T10-42	B	1	HO
T10-44	B	1	CP
T6-57	B	1	HO

Hobbit							A&D homoeologues	depth (HO/CS)	1.5		depth (HO/CS)	1.5
	GENE	gene ID	chromosome	Mbp	CULTIVARS				0.36			0.43
TaMPK6(T4)	T4-11	TraesCS7B02G009200	7BS	5.52	1	HO	TraesCS7A02G111300	xlc		TraesCS7D02G106400	56/33	1.7
VRN3(T7)	T7-1	TraesCS7B02G013100	7BS	9.7	1	HO	TraesCS7A02G115400	91/55	1.7	TraesCS7D02G111600	103/82	1.3
PGM(T3)	T3-17	TraesCS7B02G183300	7BS	14.88	1	HO	TraesCS7A02G287400	103/83	1.2	TraesCS7D02G284900	95/88	1.1
SSI(T2/T3)	T2-43	TraesCS7B02G018600	7BS	15.60	2	HO	TraesCS7A02G120300	60/55	1.1	TraesCS7D02G117800	69/42	1.6
CO-8(T7)	T7-18	TraesCS7B02G032800	7BS	32.66	1	HO	TraesCS7A02G178400LC	95/71	1.3	TraesCS7D02G131700	71/48	1.5
PUL(T2)	T2-25	TraesCS7B02G034600	7BS	33.92	1	HO	TraesCS7A02G133500	115/67	1.7	TraesCS7D02G133100	93/62	1.5
TaVP1(T6)	T6-72	TraesCS7B02G042600	7BS	42.03	1	HO	TraesCS7A02G141300	54/34	1.6	TraesCS7D02G142600	65/57	1.1
ALF4 (T9)	T9-54	TraesCS7B02G044400	7BS	43.95	1	HO	TraesCS7A02G141600	106/80	1.3	TraesCS7D02G142900	118/74	1.6
BHLH-TF-PTF1(T9)	T9-55	TraesCS7B02G084800L[illegible]	7BS	52.93	1	HO	none			TraesCS7D02G149400	14/10	1.4
TabHLH1(T6)	T6-2	TraesCS7B02G062100	7BS	64.73	1	HO	TraesCS7A02G157700	108/87	1.2	TraesCS7D02G158300	103/79	1.3
Ethylene-respTF1(T9)	T9-59	TraesCS7B02G062200	7BS	65.66	1	HO	TraesCS7A02G158000	82/43	1.9	TraesCS7D02G158500	23/16	1.4
Sucr-synth(T3)	T3-5	TraesCS7B02G482200	7BS	68.35	1	HO	TraesCS7A02G557600	106/83	1.3	TraesCS7D02G552800	118/61	1.9
TaER-B1/D1(T5)	T5-5	TraesCS7B02G069800	7BS	75.16	1	HO	TraesCS7A02G164000	40/29	1.4	TraesCS7D02G166100	90/73	1.2
WTAI-CM1/2(T2)	T2-32	TraesCS7B02G072000	7BS	79.31	1	HO	none			TraesCS7D02G168000	119/59	2.0
VRT2(T7)/TaSVP(T8)	T8-27	TraesCS7B02G080300	7BS	90.19	1	HO	TraesCS7A02G175200	70/51	1.4	TraesCS7D02G176700	62/38	1.6
AK360626(T4)	T4-54	TraesCS7B02G081700	7BS	92.06	1	HO	TraesCS7A02G176700	76/45	1.7	TraesCS7D02G178000	75/50	1.5
TaFBA1(T5)	T5-13	TraesCS7B02G083700	7BS	94.31	1	HO	TraesCS7A02G178000	53/31	1.7	TraesCS7D02G179800	42/32	1.3
Trehal-6-P-phosph(T6)	T6-28	TraesCS7B02G085800	7BS	97.97	1	HO	TraesCS7A02G180800	84/68	1.2	TraesCS7D02G182600	96/76	1.3
BHLH172(T1)	T1-22	TraesCS7B02G090500	7BS	104.76	1	HO	TraesCS7A02G185300	29/20	1.5	TraesCS7D02G187000	48/29	1.7
SSIIa/SSII-3(T2)	T2-45	TraesCS7B02G093800	7BS	107.72	1	HO	TraesCS7A02G189000	108/79	1.4	TraesCS7D02G190100	80/54	1.5
MER3(T10)	T10-29	TraesCS7B02G103700	7BS	119.21	1	HO	none			TraesCS7D02G199800	70/40	1.8
WCO1/Hd1(T7)	T7-11	TraesCS7B02G118300		137.79	1	HO	TraesCS7A02G211300	67/52	1.3	TraesCS7D02G212900	85/57	1.5
PHS1(T10)	T10-14	TraesCS7B02G132000	7BS	159.52	1	HO	TraesCS7A02G233700	78/62	1.3	TraesCS7D02G233900	77/68	1.1
TaGT61-10(T5)	T5-27	TraesCS7B02G133800	7BS	162.78	1	HO	TraesCS7A02G235700	82/70	1.2	TraesCS7D02G235700	109/67	1.6
TaGT61-13(T2)	T2-24	TraesCS7B02G133900	7BS	162.93	1	HO	TraesCS7A02G235500	49/39	1.3	TraesCS7D02G235600	32/26	1.2
TaGT61-11(T2)	T2-18	TraesCS7B02G134200	7BS	163.51	1	HO	TraesCS7A02G236200	84/60	1.4	TraesCS7D02G236300	52/37	1.4
ISA1(T2/T3)	T2-58	TraesCS7B02G139700	7BS	176.01	1	HO	TraesCS7A02G251400	57/46	1.2	TraesCS7D02G249500	18/5 (xlc)	3.6
DUF547(T9)	T9-9	TraesCS7B02G141300	7BS	179.26	1	HO	TraesCS7A02G249900	84/46	1.8	TraesCS7D02G248500	74/55	1.3
TaSPL17(T7)	T7-50	TraesCS7B02G144900	7BS	187.78	1	HO	TraesCS7A02G246500	34/27	1.3	TraesCS7D02G245200	72/43	1.7
OsSPL16(T1)	T1-9	TraesCS7B02G158500	7BS	214.07	1	HO	TraesCS7A02G260500	55/41	1.3	TraesCS7D02G261500	61/54	1.1
TaCERK1(T4)	T4-1	TraesCS7B02G162500	7BS	223.18	1	HO	TraesCS7A02G264400	100/62	1.6	TraesCS7D02G265400	98/69	1.4
PRD2(T10)	T10-5	TraesCS7B02G174300	7BS	245.17	1	HO	TraesCS7A02G276600	106/35	3.0	TraesCS7D02G276600	117/81	1.4
TaBAK1-7L(T4)	T4-7	TraesCS7B02G182300	7BS	283.11	1	HO	TraesCS7A02G293300	87/63	1.4	TraesCS7D02G291200	79/66	1.2
PGM(T2/T3)	T2-5	TraesCS7B02G183300	7BS	290.02	1	HO	TraesCS7A02G287400	103/83	1.2	TraesCS7D02G284900	95/78	1.2
	T2-36		5BS	5.06	2	HO	none			none		
	T1-13	TraesCS5B02G029100	5BS	27.84	1	HO	TraesCS5A02G030300	96/70	1.4	none		
	T10-42	TraesCS5B02G045200	5BS	50.52	1	HO	TraesCS5A02G042200	57/53	1.1	TraesCS5D02G050300	65/42	1.5
	T9-50	TraesCS5B02G075800	5BS	90.51	1	HO	TraesCS5A02G069500	65/49	1.3	TraesCS5D02G081700	62/37	1.7
	T1-26	TraesCS5B02G025100	5BS				TraesCS5A02G025900	81/61	1.3	TraesCS5D02G033700	95/68	1.4
	T6-57											
	T5-10		4BL	671.63	1	HO						
	T8-17		2BL	573.97	72	HO						
	T9-43		2BL	383.06	63	HO						
	T1-28		2BL	757.48	68	HO						
	T2-35		5BL	656.23	46	HO						
	T4-49		1BL	581.60	54	HO						
	T5-21		2BS	140.85	45	HO						
	T9-43		2BL	383.06	63	HO						
	T9-64		7AL	612.99	31	HO						
	T10-11		4BS	32.42	33	HO						
	Chr7BL genes		depth (HO/CS)					depth (HO/CS)	pth (HO/CS)		depth (HO/CS)	
	T2-6	TraesCS7B02G188400	97/58	319.86			TraesCS7A02G298600	54/44	1.2	TraesCS7D02G294300	84/46	1.8
	T4-12	TraesCS7B02G253500	119/76	467.45			TraesCS7A02G373500	51/39	1.3	TraesCS7D02G349600	100/71	1.4
		TraesCS7B02G277000	75/60	506.56			TraesCS7A02G375400	58/40	1.5	TraesCS7D02G371900	69/49	1.4
	T6-53	TraesCS7B02G320100		570.45			TraesCS7A02G419500			TraesCS7D02G412400		
	T7-16	TraesCS7B02G400600		667.07			TraesCS7A02G497200			TraesCS7D02G484400		
	T2-55	TraesCS7B02G472300		728.62			TraesCS7A02G549300			TraesCS7D02G535600		

trait gene	homoeologue	no. of cultivars	cultivars			homoeologue	GENES	cultivars																																																																
T2-9	A	1	AL			A	1	AL																																																																
T10-44	B	1	CP			B	1	PI																																																															
T4-45	B	1	CP			B	1	SS																																																															
T1-13	B	1	HO			B	1	TA																																																															
T1-9	B	1	HO			B	1	UK																																																															
T10-29	B	1	HO				1	W199																																																															
T10-42	B	1	HO				1	W203																																																															
T10-5	B	1	HO			D	1	W209																																																							AP	VA						
T10-7	B	1	HO			A	1	W199																																																								XI	ZE				
T2-18	B	1	HO				1	W203																																																															
T2-24	B	1	HO				1	W209																																																														
T2-25	B	1	HO			D	1	W777																																																														
T2-32	B	1	HO			U	1	YU																																																														
T2-5	B	1	HO				2	CP																																																														
T2-58	B	1	HO				2	W292																																																														
T4-1	B	1	HO				2	W292																																																														
T4-11	B	1	HO				3	RV																																																														
T4-54	B	1	HO				3	ST																																																														
T4-7	B	1	HO				4	W786																																																														
T5-10	B	1	HO				34	HO																																																														
T5-27	B	1	HO																																																																					
T5-5	B	1	HO																																																																					
T8-27	B	1	HO																																																																					
T9-50	B	1	HO																																																																					
T9-59	B	1	HO																																																																					
T9-9	B	1	HO																																																																					
T4-51	B	1	PI																																																																					
T2-28	B	1	RV																																																																					
T2-9	B	1	RV																																																																					
T5-2	B	1	RV																																																																					
T8-24	D	1	SS																																																																					
T1-6	A	1	ST																																																																					
T1-8	A	1	ST																																																																					
T4-18	A	1	ST																																																																					
T9-25	D	1	TA																																																																					
T4-60	U	1	UK																																																																					
T4-40	B	1	W199																																																																					
T1-6	B	1	W203																																																																					
T1-24	B	1	W209																																																																					
T9-26	D	1	W292																																																																					
T9-33	B	1	W292																																																																					
T2-48	B	1	W777																																																																					
T4-58	D	1	W786																																																																					
T4-8	B	1	W786																																																																					
T5-16	D	1	W786																																																																					
T6-1	D	1	W786																																																																					
T8-27	D	1	YU																																																																					
T2-36	B	2	W579	HO																																																																				
T2-43	B	2	AV	HO																																																																				
T4-55	D	2	BW	AP																																																																				
T8-21	D	2	W199	W786																																																																				
T8-24	A	2	CG	CR																																																																				
T8-24	B	2	CG	CR																																																																				
T9-38	B	2	AL	CP																																																																				
T2-60	A	3	GC	GT	VE																																																																		
T4-49	B	4	W733	W786	SF	VA																																																																	
T6-6	D	4	W292	BW	SU	TA																																																																	
T8-2	B	4	W203	W777	GT	UK																																																																	
T10-8	D	6	W160	W292	W579	W624	W733	SS																																																												
T1-25	B	8	W246	W579	W777	BA	CA	MK	SS	XI																																																										
T4-45	A	8	W115	W160	W292	CH	CL	CR	RV	ZE																																																										
T6-11	A	9	W115	W292	W624	W777	BW	CP	AP	VE	YU																																															TA	UK						
T10-16	B	10	AM	BW	BR	FI	GT	GL	NA	RL	RI	SA																																																AP	VA	VE	XI	YU	ZE
T1-20	B	10	AM	BW	BR	FI	GT	GL	NA	RL	RI	SA																																																						
T4-15	D	10	W115	W203	W387	W624	BW	CP	SU	TA	AP	VE																																																						
T4-56	B	10	AM	BW	BR	FI	GT	GL	NA	RL	RI	SA																																																						
T5-12	A	10	BW	CE	EI	FL	GR	MW	SA	SO	AP	YU																																																						
T5-12	B	10	AM	BW	BR	FI	GT	GL	NA	RL	RI	SA																																																						
T5-22	D	10	W141	W624	CE	FL	HU	JB	ME	PI	RL	SO																																																						
T5-30	B	10	AM	BW	BR	FI	GT	GL	NA	RL	RI	SA																																																						
T2-50	B	11	AM	BW	BR	FI	GT	GL	NA	PI	RL	RI	SA																																																					
T6-18	A	11	W141	W160	W209	BW	BR	CR	SS	TA	AP	VE	YU																																																					
T2-3	B	13	W115	W209	AM	BW	BR	CP	FI	GT	GL	NA	RL	RI	SA																																																	
T2-55	B	13	W199	W246	W579	W733	W786	FL	GT	JB	KSL	KSI	KTR	ME	SU																																																	
T4-14	A	15	CG	KSA	OA	RE	RO																																																																
T8-25	A	16	AB	AM	CG	CR	DI	FI	GL	KSA	KSL	KSI	KTR	MK	NA	PI	RV	SA																																											
T9-42	B	16	W115	W160	W203	W209	W387	W733	BA	BW	CA	CO	PA	SF	SP	TA	AP	VE																																											
T4-4	B	22	W199	W246	W579	W733	BA	BR	CA	CP	GL	HW	IQ	MW	PA	RV	RB	SA	SP	SU	TA	UK	AP	XI																																		
T6-3	A	31	W209	W387	W777	AB	AL	AV	BR	BU	CA	CP	CO	DI	EI	FL	GR	HF	HU	JB	ME	RV	RI	RB	RO	SC	SS	SO	SP	TA	VA	XI	YU																																									
T9-64	A	31	W203	W292	W733	W786	AB	AM	BW	BR	BU	CL	CP	CR	DI	FI	GL	GR	HO	IQ	MK	RL	RV	RI	RB	SA	SC	SF	SL	SU	AP	VE	XI																																									
T10-11	B	33	W246	W292	W579	W624	W733	W777	W786	AB	AM	BA	BR	CA	CH	CG	EI	FL	GA	GL	HW	HO	KSL	MK	NA	PA	RL	SS	SF	SP	SU	VA	VE	XI	YU																																							
T5-21	B	45	W141	AB	AM	BR	CE	CH	CL	CG	CR	DI	EI	FI	GA	GC	GL	GR	HW	HO	IS	IQ	JB	KSA	KSL	KSI	KTR	MA	MH	MK	ME	NA	OA	PI	RE	RL	RV	RI	RB	SA	SF	SO	SL	ST	VA	XI	ZE																											
T2-35	B	46	W141	W160	W246	W579	AB	AM	BA	BR	BU	CH	CP	CG	CR	DI	EI	FI	FL	GT	GL	HF	HW	HO	HU	IS	IQ	JB	KSA	KSL	KSI	KTR	MH	MK	ME	NA	OA	PI	RE	RL	RV	RI	RO	SC	SL	ST	TA	ZE																										
T4-49	A	54	W203	W777	AB	AL	AM	AV	BR	BU	CH	CL	CO	CG	CR	DI	EI	FI	FL	GA	GC	GT	GL	GR	HF	HW	HO	HU	IS	IQ	JB	KSA	KSL	KSI	KTR	MA	MH	MW	MK	ME	NA	OA	PI	RE	RL	RV	RI	RB	RO	SA	SC	SL	SP	ST	YU	ZE																		
T2-26	B	60	CS	W115	W141	W160	W203	W209	W246	W292	W387	W624	W733	W777	CS	W115	W141	W160	W203	W209	W246	W292	W387	W624	W733	W777	AL	AM	BA	BW	BU	CE	CH	CL	CO	CG	CR	FI	GC	GL	HF	IS	IQ	KSL	KSI	KTR	MK	NA	OA	PI	RE	RV	RO	SA	SC	SS	SF	SO	TA	UK	AP	ZE												
T9-48	D	63	W160	W203	W292	W579	W624	W733	W777	W786	AB	AL	AM	AV	BA	BR	BU	CA	CE	CH	CL	CO	CG	CR	DI	EI	FI	FL	GA	GC	GT	GL	GR	HF	HW	HO	HU	IQ	JB	KSA	KSL	KSI	KTR	MA	MH	MW	MK	ME	NA	OA	RE	RL	RV	RI	RB	RO	SA	SC	SF	SL	SP	ST	VA	XI	ZE									
T1-28	B	68	W203	W579	W624	W733	W777	AB	AL	AM	AV	BA	BR	BU	CA	CE	CH	CL	CO	CG	CR	DI	EI	FI	FL	GA	GC	GT	GL	GR	HF	HW	HO	HU	IS	IQ	JB	KSA	KSL	KSI	KTR	MA	MH	MW	MK	ME	NA	OA	PA	PI	RE	RL	RV	RI	RB	RO	SA	SC	SS	SF	SO	SL	SP	ST	TA	UK								
T8-17	B	72	W160	W203	W209	W387	W624	W733	W777	AB	AL	AM	AV	BA	BR	BU	CA	CE	CH	CL	CP	CO	CG	CR	DI	EI	FI	FL	GA	GC	GT	GL	GR	HF	HW	HO	HU	IS	IQ	JB	KSA	KSL	KSI	KTR	MA	MH	MW	MK	ME	NA	OA	PA	PI	RE	RL	RV	RI	RB	RO	SA	SC	SF	SO	SL	SP	ST								

hobbit	34 genes

cultivars	genes missing
1	48
2	7
3	1
4	3
6	1
8	2
9	1
10	7
11	2
13	2
15	1
16	2
22	1
31	2
33	1
45	1
46	1
54	1
60	1
63	1
68	1
72	1

	133	15%
other shared	150	17%
unique	625	69%
	908	

5	
4	
20	
4	
6	
4	
23	
7	
4	
3	
10	
4	
16	
6	
3	
4	
3	
9	
12	
4	
1	
3	
9	
13	
1	
27	
NO	
9	
13	
1	
2	
8	
11	
3	
4	
1	
17	
10	
3	
NO	
11	
10	
7	
7	
10	
NO	
26	
3	
12	
7	
6, 1del	
8	
11	
1	
2	
3	
13 **del1-4**	
1	
**11**	
11	
1	
12	
7	
10	
4	
5	
7	
7	
6	
28	
12	
15	
20	
6	
2	
2	
9	
NO	
3	
6	
	30
13	
	20
2	
12 del1 (In4)	
11	
8	
16	
15	
15	
1	
9	
3	
16	
12	
13	
21	
9	
23	
4	
3	
24	
13	
1	
20	
	40
3	
13	
11	
19	
7	
3	
9, del1	
1	
15	
3	
19	
3	
9	
3 del1	
11	
>30	
3	
1	
2	
1	
	50
>50	
>30	
NO	
5	
2	
1	
15	
15	
20	
15	
3	
1	
7	
8	
11	
	30
25	
10	
7	
15	
6	
12	
5	
3	
23 1_del	
45	
	60
7	
3, del	
7	
17	
4	
6 1 del*	
5 KR del	
13	
6	
4	
9	
10	
6 2del	
9	
17	
6	
1	
3, del1&2	
17 1_del	
16	
9	
17	
20	
22	
7	
3	
18	
	40
1	
12	
>20	
12	
14	
14	
1	
6	
15	
12	
20	
6, del1	
4	
40 Tmdel2	
19 1_del	
	70
10, del2	
	50
15	
14	
2del	
16	
9	
25 **del1***	
25	
19	
14	
10 del1*, 2del	
12	
>30 **del1-2***	
2	
10	
6	
10	
8	
3	
22	
14	
5	
3	
14, 2del	
16	
4	
2	
7	
6	
2, del1*	
6	
	60
3, 3dels	
5	
	70
8	
9	
13	
9	
18	
2	
15, 1del	
9	
7	
12	
8	
3	
3	
8	
4	
3	
14	
4	
8	
NO	
2	
5	
17	
1	
14	
7	
20	
8	
20	
5	
7	
9	
15	
NO	
	70
**9, del1***	
	80
1	
19	
2	
6	
13	
10	
25	
7	
14	
9	
20	
1	
8, ins?	
NO	
	90
1	
	80
5	
8	
6	
10	
5	
3	
6	
1	
2	
12, 3dels	
5	
7	
5	
1	
6 del1*	
3	
23, del1	
28	
5 no_del1	
10	
24, 1 del	
	100
1	
>20, ins @ [-1001]?	
7	
	90
9	
3	
NO	
10	
35	
4	
W246 -3/ +del7#	
8	
vlc	
9	
18, del2**	
18	
21	
10	
20	
6 del1-2	
16	
20	
11	
10	
	110
8	
10	
5	
1	
3	
3	
7	
NO	
7	
3, del1*	
2	
10	
19, 1del	
4	
3	
8	
2	
3	
16	
3	
12	
4	
8, del1	
3	
4	
15	
14	
9	
10	
13	
	100
1	
8	
2	
18	
18	
3	
4	
5 del5**	
20 del1*	
20	
3	
27, del1*	
20	
1	
20	
14	
3	
6	
10	
4	
1	
7	
8	
8	
22 del2*	
	110
15	
6	
4	
6	
	120
10	
13	
3 Tm_del2	
4, ASP_del1	
12	
21	
2	
6, del2**	
9	
11, del1	
10	
4	
13 3dels	
5, del7&8##	
19	
9	
15	
	130
8	
5	
14	
6 del1&2	
14	
20	
13	
1	
19	
3	
32	
2	
22	
1	
NO	
2	
19	
29	
NO	
1	
8	
	120
5	
17	
5	
	140
6	
1	
17 1del	
21 1del	
9	
15	
4	
17	
15	
NO	
2	
5	
4	
1	
6	
8	
11	
10	
5	
6	
**15** del1-2*	
6	
8	
	130
5 del	
2	
10	
14	
5	
8 del1*	
12	
2	
5	
3	
8	
	150
5	
8	
3	
11 del1	
9	
26	
8	
14	
8	
6	
	133
5	
20	

T1	T2	T4	T5	T6	T8	T9	T10
6	2	3	2			1	8, del1&2
2		3					
				CS			
CS	CS	CS	CS		CS	CS	CS
		1		8	9		
3							
CS	2		1	CS			CS
	CS	CS	CS	5	CS	CS	
	6						
	1						
CS		CS		CS		CS	CS
11	CS		CS		CS	1	
	10, del1		1	5			
			CS				
		CS	2	CS		CS	CS
CS	CS				CS	9, del1	4
1	1						
	11		CS			CS	
		CS		CS			CS
CS	CS				CS		
			CS		4		
	3					CS	
		CS		CS		10	CS
CS	CS	5	CS		CS		
			2 del1			CS	
						4	CS
		CS		CS		1	
CS	CS	del*	CS		CS		
12					2	CS	
			CS				CS
CS		CS	4, del1	CS	1	CS	
	CS				CS		
	25		CS				
							CS
CS		CS		CS	CS	CS	
	CS	8	CS		3	3	
	1, CSdel1			17			CS
CS		CS		CS	CS	CS	
	CS	NO*	CS				CS
	CS			CS			
CS	3	CS			CS	CS	
	1		CS				CS
5	CS	7		CS			3
	1					CS	1
CS		CS			CS	7	
			CS			2	CS
	CS	1	5	CS			
CS	1					CS	CS
5	23	CS	1		CS	14	
1			CS		22		
	CS			CS			CS
CS						CS	
		CS			CS		CS
	CS		CS	CS	1		
1						CS	
CS							CS
	CS	CS	CS		CS		
		4		CS		CS	2
CS		3				1	
	CS				CS		CS
CS	2	CS	CS				
	5			CS	CS	CS	
	29					2	
CS	CS			CS			CS
2		CS	CS	1			
	5				CS	CS	
	CS						
CS	10		CS				CS
		CS	9, 2dels		CS		
					12	CS	
	CS					3	
CS			CS				CS
2		CS	4		CS		1
	CS	1				CS	CS
CS	10						
3	15		CS		CS		CS
		CS	2				
CS	CS					CS	
		6					
			CS		CS		CS
	CS	CS					10
CS			CS			CS	
5	2						
2			2		CS		CS
CS	CS	CS				CS	
	28		CS			18	CS
1					CS		
					2	5	
CS	promoter	CS				CS	CS
	NO	21	CS				
	25						CS
	CS						25
CS		CS				CS	
2		13	CS				
							CS
CS	CS						
		CS				CS	
18							
	CS						CS
		CS				CS	
						5	
	CS	CS					CS
	1						
	CS					CS	
	12					18	CS
		CS					
	CS					CS	CS
	8	1					
	3	CS					
	CS						
	7					CS	CS
		CS					
	CS						
	4	32				CS	CS
		4					
	CS						CS
	2	CS				1	
		7				CS	2
	CS	CS					CS
						CS	
	CS					2	
		CS				2	
		1					CS
						CS	
	CS	CS					
							CS
		CS				CS	
	CS						
	10, del1						
	CS	CS				1	CS
						CS	2
	CS						
	13						
		CS				CS	CS
		3					
	CS						
							CS
	CS	CS				CS	25
	9						
		1					CS
	CS	CS				CS	5
		10					8
	CS						
	3	CS					CS
	1	12				CS	
	CS					4	
	1						
	7 dist	CS					
	35					CS	
	CS						
	1						
		CS					
						CS	
	CS					21, del1	
		1					
	1	CS					
	CS					CS	
	8					3	
		CS				CS	
	CS						
	5						
		CS				CS	
	CS	1				7	
		CS				CS	
	CS	4					
		1					
		CS				CS	
	CS	20					
		CS					
						CS	
	CS						
	11	CS				6	
						CS	
	CS						
	1	CS					
		2					
		7				CS	
	CS	CS				6, del1	
	1						
	1						
	CS						
	5	CS					
		6				CS	
	CS						
		CS				CS	
		6				1	
	CS						
	1	CS				CS	
	3	1					
		2					
		CS				CS	
						11	
		partial coverage of del1*					
		CS				CS	
		2					
		14					
		CS				CS	
		1					
						2	
		CS				CS	
						CS	
						5	
						CS	
						3	
						CS	
						CS	
						3	

SNPs	incidence	%		
1	28	21.1%	28	
2	19	14.3%	38	
3	15	11.3%	45	
4	10	7.5%	40	
5	10	7.5%	50	
6	7	5.3%	42	
7	5	3.8%	35	
8	6	4.5%	48	
9	4	3.0%	36	
10	5	3.8%	50	
11	4	3.0%	44	
12	3	2.3%	36	
13	3	2.3%	39	
14	2	1.5%	28	
17	1	0.8%	17	
18	4	3.0%	72	
20	1	0.8%	20	
21	2	1.5%	42	
22	1	0.8%	22	
25	2	1.5%	50	
32	1	0.8%	32	
	133		814	
	total SNPs:	814		908 analysed pro
				0.9 SNPs per pro

	shared haplotypes										
**trait**	**1**	**2**	**3**	**4**	**5**	**6**	**7**	**8**	**9**	**10**	11
**T1**	16	21	10	14	7	3	1	0	0	0	
**T2**	27	32	42	23	10	4	1	0	2	0	
**T4**	35	40	39	29	10	6	2	2	1	0	1
**T5**	23	19	10	4	7	3	0	0	0	0	
**T6**	7	17	9	9	5	0	2	2	0	0	
**T8**	19	17	13	12	4	0	1	2	0	0	
**T9**	44	53	38	19	13	3	0	1	0	0	
**T10**	26	42	21	12	6	1	0	0	0	0	
total genes	197	241	182	122	62	20	7	7	3	0	

trait	shared haplotypes	unique haplotypes	total haplotypes	genes	total haplotypes/gene
T1	204	254	458	72	6
T2	447	446	893	133	7
T4	484	465	949	154	6
T5	193	189	382	69	6
T6	162	172	334	49	7
T8	200	158	358	63	6
T9	444	522	966	165	6
T10	263	291	554	105	5
	2397	2497	4894	810	48

	shared haplotypes										
	**1**	**2**	**3**	**4**	**5**	**6**	**7**	**8**	**9**	**10**	
%	23.4%	28.5%	21.8%	14.5%	7.4%	2.4%	0.8%	0.8%	0.4%	0.0%	
no of genes	197	240	183	122	62	20	7	7	3	0	841
%	23.4%	28.5%	21.8%	14.5%	7.4%	2.4%	0.8%	0.8%	0.4%	0.0%	

	shared haplotypes (normalised)									
**trait**	**1**	**2**	**3**	**4**	**5**	**6**	**7**	**8**	**9**	**10**
**T1**	22.2%	29.2%	13.9%	19.4%	9.7%	4.2%	1.4%	0	0	0
**T2**	20.3%	24.1%	31.6%	17.3%	7.5%	3.0%	0.8%	0	1.5%	0
**T4**	22.7%	26.0%	25.3%	18.8%	6.5%	3.9%	1.3%	1.3%	0.6%	0
**T5**	33.3%	27.5%	14.5%	5.8%	10.1%	4.3%	0	0	0	0
**T6**	14.3%	34.7%	18.4%	18.4%	10.2%	0	4.1%	4.1%	0	0
**T8**	30.2%	27.0%	20.6%	19.0%	6.3%	0	1.6%	3.2%	0	0
**T9**	26.7%	32.1%	23.0%	11.5%	7.9%	1.8%	0	0.6%	0	0
**T10**	24.8%	40.0%	20.0%	11.4%	5.7%	1.0%	0	0	0	0
