# Supplementary material for: Exploring the diversity of promoter and 5′UTR sequences in ancestral, historic and modern wheat
Source: Plant Biotechnol J. 2021 Sep 16;19(12):2469–87. doi: 10.1111/pbi.13672 (PMC8633512; doi:10.1111/pbi.13672)
Supplement: Supplementary file 16 — Data S6 Deletions, TFBS. [file PBI-19-2469-s002.pdf]

sheet2	deletions - TFBS	
**column**	**header**	**explanation**
A	trait gene	eg T1-2 = Trait 1 gene 2
B	homoeologue	A, B, D, U
C	cultivar & location	eg W786 del2 from [-845] = first found in cultivar W786, starting 845bp upstream of ATG start site
D	length (bp)	all large deletions (>120bp) are highlighted in green
E	sequence	full sequence of deletion from Chinese Spring IWGSC_RefSeq_v1.0
F	TFBS (corr)/ length	percentage of TFBS per length of deletion
F	TFBS corrected: unique & NO mismatches	number of TFBS without species duplications & no mismatches
G	TFBS name	names of TFBS with 100% match - if these are from a cereal species, this is indicated in brackets
**sheet3**	**transposable elements**	
A	trait gene	eg T1-2 = Trait 1 gene 2
B	homoeologue	A, B, D, U
C	cultivar & location	eg W786 del2 from [-845] = first found in cultivar W786, starting 845bp upstream of ATG start site
D	length (bp)	all large deletions (>120bp) are highlighted in green
E	sequence	full sequence of deletion from Chinese Spring IWGSC_RefSeq_v1.0
F	BLAST hits (IWGSC, URGI)	https://urgi.versailles.inra.fr/blast_iwgsc/blast.php, against Wheat_IWGSC_RefSeq_v1.0_all_chromosomes **SEARCH PARAMETERS**
G	identification (trepdb_complete_Rel-19)	identification of large deletions as transposable elements http://botserv2.uzh.ch/kelldata/trep-db/blast/blastTREP.html.
H	% of reference	% of identified transposable element in the deletion
I	identification (CLARITE_CLARIrepeatwheat#)	identification of large deletions as repeat elements: iwgsc_refseqv1.1_TransposableElements_2017Mar13.gff3

observed gaps in the captured sequences:
> these are noted as deletions here only if the gap (with sharply defined (cliff-edge) borders in the coverage graph (BAM files)) lies across actual defined sequence in the reference genome (IWGSC refseq 1) and not across stretches of unassigned sequence (Ns).

> They are always labelled by the first cultivar it was observed for, always going from left to right (ie CS>[control species]>Tmon accessions>Watkins lines>commercial cultivars] ,

>for example for T1-2A, the first observed gap occurred in all Tmon accessions and is thus labelled Tmdel1 (nb: if this gap were only found in one or a few of the Tmon accessions, it would be labelled with the first accession were it was observed, eg M031del1 etc) and the only deletion in T1-2B appears in ASP

> short deletions were mostly not BLASTed against IWGSC refseq1 (urgi), whereas longer ones were always BLASTed, and the number of hits and identification as a known TE and/or ClariRepeat Sequence is shown, eg for T1-2B_ASP_del there were 8,771 hits and the deletion constitutes a small part of RLG_Taes_Lila_consensus-1 (1.3%) and is also recognised as repeat region#66628_74962_Repeat_region_16 in IWGSCrefseq_v1.0

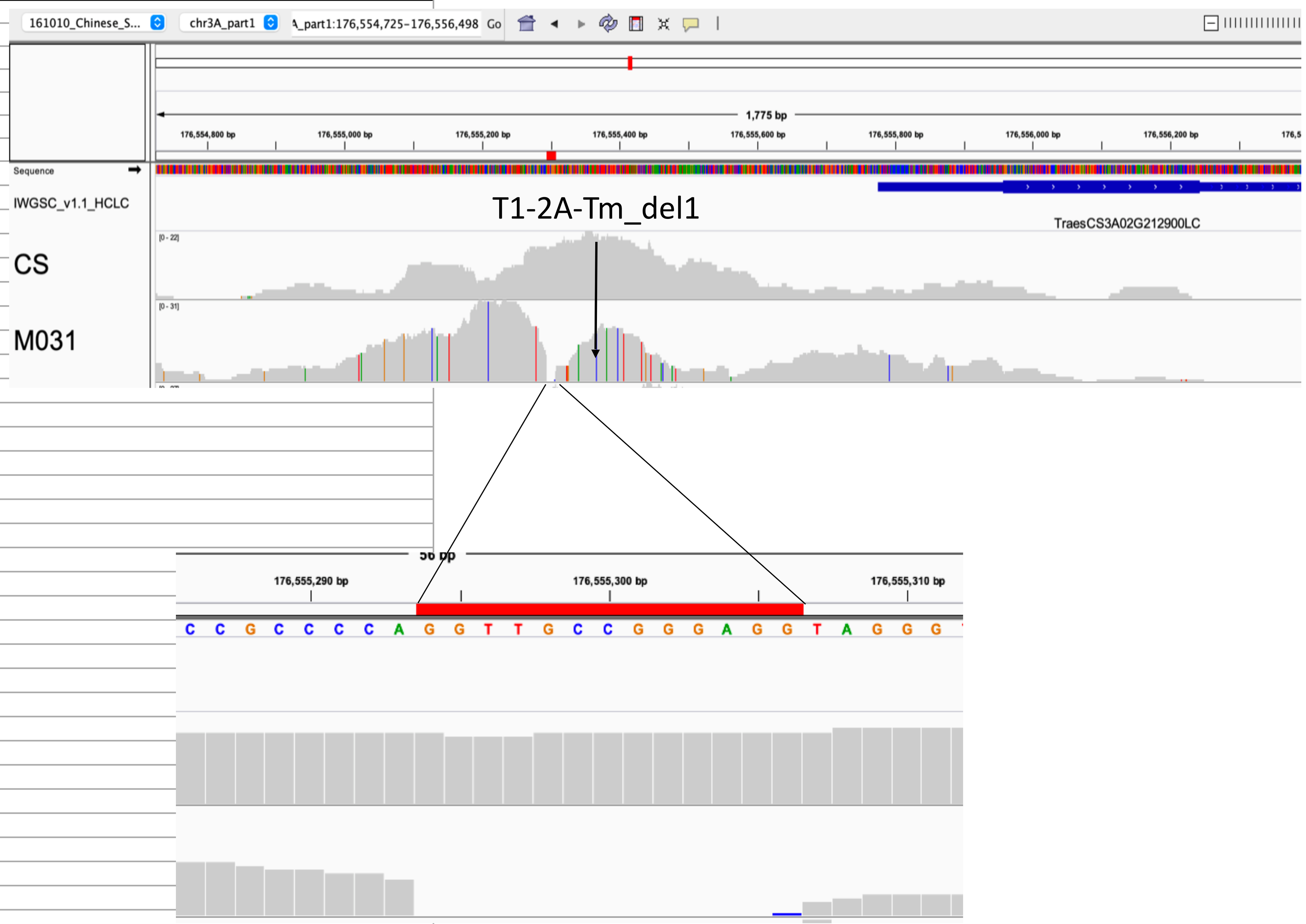

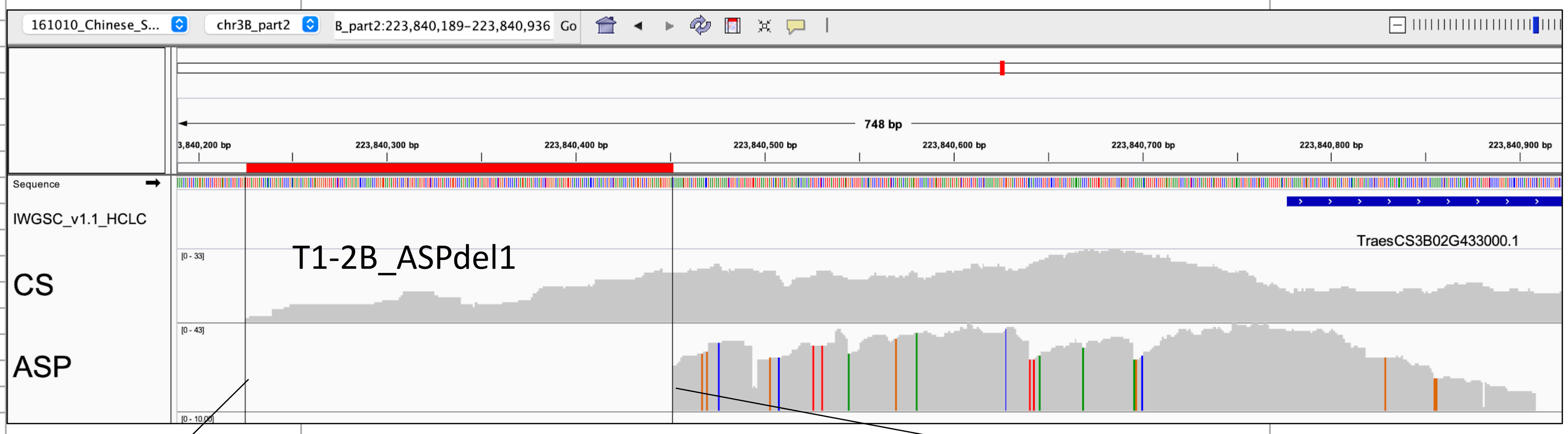

5’TTTAGTGATCACACGGAGATCCCGCAGAGCAGGACTAGGGGGTGTTATCTCCTAGGAGAGCCCCAAACCTGGGTAAGATTCGCCGGCGTGCATGTCTTCGCCTTATCCTGT TCCAGACACCGGCGATGTCTTACTGGCTCCCACTATGATTAGCCATCCTTGGGCATATGTTGCACCTACCACCCGACACGCGCCGAGAACCGATCGTGGCTCGGAGAAGGTTG GA3’

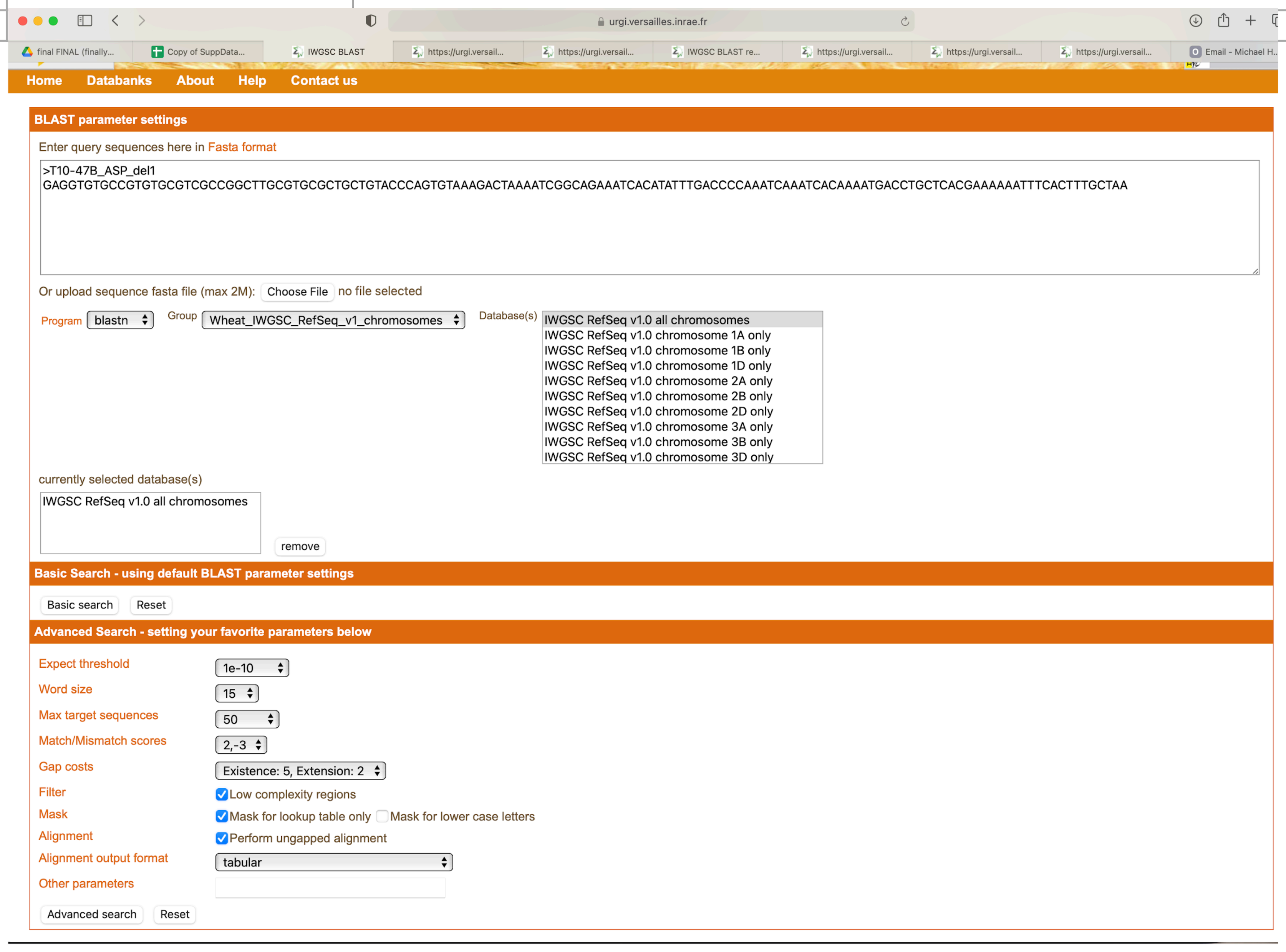

trait gene	homoeologue	cultivar & location	length (bp)	sequence	TFBS	TFBS / length	TFBS name
T1-2	A	Tmdel1 from [-655]	13	GGTTGCCGGGAGG	0	0.0%	
	B	ASP del1 from [-622]	227	GATTTAGTGATCACACGGAG	3	1.3%	AtMYB84 BS , PI, MYBa,
T1-3	A	Tm del1 from [-637]	6	CTCCTG	0	0.0%	
		Tm del2 from [-816]	293	GTAGGGTCTGTTTGGTTGGA	0	0.0%	
T1-4	D	APG del1 from [-1252]	164	TACTCCCTCCGTTCATAAATAT	2	1.2%	CArG (TBG4-b) , ARR10 BS2
T1-7	A	Tm del4 from [-704]	285	TATGGTGAGTATCATATAGTA	2	0.7%	RY4, RY (O.sat),
T1-8	A	ASPdel1 from [-860]	125	CGGATAGGTAAAAGTTCCCA	4	3.2%	LRE-TATA AA1 , GS1b AC box 2
		ASPdel2 from [-1675]	449	AGTGTCAGCTACTTTGTTCA	4	0.9%	CCA1 motif (2), AT-2b , GBF1
		W115del1 from [-474]	20	CATTTTTTCCTTCTCTGAAG		0.0%	
		W115del2 from [-1574]	29	GTTTGAGAACTGTTCGACAA	1	3.4%	AB1 heptamer
T1-11	A	M031 del1 from [-1069]	10	GAGGAAGGCA	0	0.0%	
		M049 del2 from [-1205]	50	TCATCGCAGCTGCCTTCTCA	0	0.0%	
T1-12	A	Tm del1 from [-1023]	31	AATCGGACCGTTGAGCAGC	4	12.9%	A-box, 7A element (Z.mays),
	B	KR del1 from [-546]	46	CTCCACCAGCCTGCTCACCT	5	10.9%	KN1/KIP BS (Z.mays), GA-5 ,
T1-13	A	M308 del1 from [-462]	24	AACTTTCACACAGCCCACAC	0	0.0%	
	B	APG del5 from [-1365]	116	CAAAGATCCTTGAAATTTTTT	7	6.0%	D1, WB (WRKY), GT-1-like K ,
T1-16	A	Tm del1 from [-180]	16	CCCCTCCGCCCGCAGG		0.0%	
		Tm del2 from [-388]	22	AGCGATTTCGGTTTTTATTAG	1	4.5%	LTRE (H.vulg)
T1-22	A	Tm del1 from [-373]	250	AATAAAGTGCTATAATGACCA	3	1.2%	MNF1 BS core (Z.mays), box I,
	D	ASP del1 from [-1090]	211	GTATTGAATTCATGCTCCAGT	3	1.4%	DNase I-footprint 3 (2x),
T1-23	A	APG del3 from [-1205]	173	CTGGCGGAGCTAGGAAGAA	4	2.3%	Dof1 BSopt (Z.mays), CDE,
	D	APG del 1 from [-562]	122	ACAGCTCAAAAATGCCATCG	1	0.8%	EE2,
T1-24	B	ASP del1 from [-1474]	276	AGGGCATGTACAATGGTTGA	8	2.9%	ABRE motif A (O.sat), BOX III*
T1-25	D	APG del1 from [-1647]	246	CCCCTCCGTCTCATAATATAA	3	1.2%	AT-cm , PB Element (ERE) ,
T1-28	B	KR del1 from [-1490]	283	ACTAGTATGCCTCTACTTGAC	5	1.8%	CARE2 (O.sat), CL3, ACE1
	A	Tm del3 from [-397]	84	TTGGGGATGGACGACGCCG	2	2.4%	TATA box (O.sat), Myb-2
T2-2	A	M031 del3 from [-1003]	210	GGTAGGGCAAGGAATGGTC	5	2.4%	E4-core , B2 (Z.mays,
			80	GGAAAAGAACTCTATGAGA	5	6.3%	Box-1 (H.vulg), box II EE2 , B1
		M031 del1 from [-480]	7	ATACGCG	0	0.0%	
		M031 del2 from [-927]	85	AGCTGTCTATCCAAAAAAGA	2	2.4%	E1-core , TA (O.sat)
		M031 del4 from [-1243]	7	GGCCAGT	0	0.0%	
		M037 del5 from [-543]	16	TGCACATCGCAGAGAG	0	0.0%	
		W786 del3 from [-1284]	227	TTCTCGAGATCATAACCATGG	1	0.4%	MYB 1AT BS
T2-3	B	KSI del1 from [-523]	52	GGGGCTGCTGCTGCTGCCTG	1	1.9%	CRE 45-48 (O.sat)
	D	W115 del1 from [-1708]	221	TTTGACCGTAGTCGGTCTATA	1	0.5%	E4-core
T2-4	A	ASP del1 from [-505]	152	TGCCTGAGGTGGCTCAGATG	6	3.9%	vicilin box , Myb1, TT box
T2-5	B	ASP del1 from [-344]	14	GCTAGTACATACTC	1	7.1%	GTAC-motif 1 ,
		ASP del2 from [-416]	74	GTTTTAGTATTAGATACATCT	1	1.4%	E5-core,
	D	W292 del1 from [-447]	6	ATCTAA	0	0.0%	
T2-6	A	Tm del1 from [-673]	311	AGAATCAGGGCCTCTTTGAT	3	1.0%	AuxRE, GATA (O.sat), GCAA-
T2-7	A	Tm del1 from [-246]	78	TTTAGCATTTATTATTTATATT	0	0.0%	
T2-9	B	W115 del1 from [-714]	177	CAAAAAAAAATACTAGAAAAA	4	2.3%	CCA1 BS , MYC2/MYC3/MYC4
		W199 del3 from [-801]	518	GACATCTGAGTAGCTCTCGG	2	0.4%	HSE3, HSE-2 (H.vulg)
			186	GACATCTGAGTAGCTCTCGG	3	1.6%	CCA1 BS, PY-box (O.sat),
		W115 del2 from [-1334]	112	TGCCTAGCTAGTACTCCCTTC	3	2.7%	D1 box , ERE 2, LBS
		W199 del4 from [-1377]	126	CTCCCTTCGTTCCGAATTACT	2	1.6%	TSS CC , ERE 2
T2-11	A	KR del1 from [-970]	71	ACGGGACCACCAGTCATGCT	6	8.5%	RY2, RY4, PROL box (O.sat),
		Tm del2 from [-838]	319	CGATGCGTTTGGTTGAAGGT	8	2.5%	SEF3-BE, RY4, I-box , CLE-core
T2-12	A	KR del1 from [-839]	723	GAGGTAATAGCACCAGCAGT	2	0.3%	P-box 1 (T.aes, LMWglut),
		Tm del2 from [-410]	37	CTTAAACAATTTTCTTGCTTA	3	8.1%	C2b-BS (O.sat), C2a-BS
		Tm del3 from [-839]	5	TGGTT		0.0%	
T2-13	B	ASP del1 from [-510]	184	AATCATTTCTCTTTATTTATTT	2	1.1%	Dof3 Bsopt (Z.mays), MYC2/
T2-15	A	Tm del1 from [-286]	17	TTTTTGAGCCTTGTATA	0	0.0%	
		Tm del2 from [-387]	14	TAGGTCTCGCGCTC	0	0.0%	
T2-16	A	Tmdel1 from [-23]	363	GCGCGCTGGTTGTTTGCTTT	6	1.7%	Box I, CArG box , CL3,
	B	ASP del1 from [-335]	126	CTCCCTCCGTCCGAAAATACT	4	3.2%	Box A , CCAAT box-like motif
T2-17	B	ASP del2 from [-1497]	248	CGTTTACTAGCAACTTTATAT	5	2.0%	T5-6-T5 (oat), SS-30 , CArG
		ASP del1 from [-800]	8	GTACCATG	0	0.0%	
T2-19	A	APG del 1 from [-454]	639	CAAAACTTCTGATCTATTCAT	1	0.2%	LBD-motif (Z.mays),
	B	ASP del1 from [-354]	370	ACATTTTTTCGGAAAAACTT	5	1.4%	AtMYB77 BS , dOCT (Z.mays),
	D	W115 del2 from [-904]	106	AGCCGGGCGAGCCAAAATT	9	8.5%	9/10 L , CCAAT box-like motif
		W115 del1 from [-82] in 5'UTR	83	GGCAGGGGAGGGTTCTTTC	3	3.6%	CE1-like element, TATA-box
T2-20	A	Tm del3 from [-204]	82	GACGTAGAATTGTTTATCTGA	6	7.3%	GATA, P1BS (SmPT3) , EE
		W141 del4 from [-395]	21	GTTAGCAAGCTTCCAATCTTG	2	9.5%	CCAAT box 1 , Box I
	B	KR del8 from [-1295]	228	TCAGGGATCAACGAGAATTT	2	0.9%	B1 (Z.mays, opaque2), CCAAT
T2-21	A	Tm del1 from [-792]	75	ATATAGATTATCTATTTGCTC	1	1.3%	AC-I (1),
	D	ASP del2 from [-2025]	259	CTACAACATGGGATACTATCT	1	0.4%	PLE-79
T2-24	A	Tm del1 from [-685]	459	AACTGCTGGGGAAAGGGGA	5	1.1%	L1-box , AT-cm , TERE, RIN
		Tm del2 from [-926]	19	GCCCCTCCGTTTCTAAATT	0	0.0%	
		Tm del3 from [-1050]	57	TGGCTGTCCAGATGCGTGTG	3	5.3%	Mybb, B5-like motif
	B	ASP del1 from [-750]	559	TAAAGGCATCTCCAACACCG	4	0.7%	SEF1 BS , DRE2, W-box 3
T2-25	D	APG del1 from [-321]	108	TGATGCTAGCTACGAGCTTCT	4	3.7%	CACA box , RIN BSC ["SRF"] ,
T2-26	B	ASP del1 from [-226]	472	CGGGATGACATGCTTAAATT	4	0.8%	RY4, B1 (Z.mays), CArG2,
		APG del2 from [-575]	408	GTTGTTGTGTGCCAGCACGC	2	0.5%	B1 (Z.mays, Opaque2),
		W579 del 3 from [-198]	571	AAAATAGCTCATGACACTACA	5	0.9%	RY4, B1 (Z.mays, Opaque2),
		W579 del 4 from [-1062]	508	TTTGCCATGCGGTGCTTGTT	5	1.0%	TGA2, AT-1 , NST2 BS , C3HC4/
	D	M031 del3 from [-1518]	438	TCAGTGAATTTCAGTGAAAT	5	1.1%	GATA motif, D1 box , GA-1 ,
T2-27	A	M031 del3 from [-1050]	110	GTAGCAAGTAGCATTTTTTCT	1	0.9%	EL1,
		ASP del1 from [-217]	47	TGCATGGTACGAGTTGTGGC	1	2.1%	RY,
		KR del 2 from [-990]	19	ACCATCATCCTGATACATG	2	10.5%	ATCATC motif , ANAC089 BS 2
T2-29	B	ASP del1 from [-1063]	70	ATATATGCTGCATAAAATAAA	4	5.7%	LTRE (H.vulg), AT-2b, AT-cm ,
T2-30	D	APG del1 from [-457]	106	TCTAATATAGAATCCTCTAACA	4	3.8%	SEF3 BS 2, AS1-AS2 BS I
T2-31	A	Tm del1 from [-464	32	TCTATCTATCTATCTATCTATCT	0	0.0%	
		Tm del2 from [-639]	11	CAAACACTCCA	0	0.0%	
T2-31	B	APG del1 from [-1669]	408	TTTTTTAGAAAAGAGGGTTA	7	1.7%	RY4, I-box , NON, Sph, EBS1,
			251	TTTTTTAGAAAAGAGGGTTA	5	2.0%	I-box , NON, EBS1, WUS BS,
T2-32	B	ASP del1 from [-57]	73	GTGCTGGTGGCTGTAAGCAA	1	1.4%	E-box ,
T2-33	A	APG del2 from [-1210]	516	AAAAGCACTACACTGGTGCT	11	2.1%	ABRE Bp, 9-mer box , adjb,
		APG del1 in 5'UTR from [-709]	60	CTTCCCCCGGCTTTGTGGAT	2	3.3%	Oct (d) (T.aes), CARE E
		W199 del3 from [-1390]	10	ACGGCCCGCC	0	0.0%	
T2-34	B	ASP del1 from [-79]	74	CCCAGAAAAAGTTAAGAGAG	2	2.7%	CArG box , GAMYB BS (KAR)
		ASP del2 from [-395]	76	CTACTCCCTTTGTATCAAAAT	4	5.3%	SEF3 BS 1 , AT-rich G, CCGTCG
T2-39	D	APG del1 from [-372]	42	TTTGTTTTTTTGAGGTTCAG	2	4.8%	CAT (Z.mays), UPE-box (TV2) ,
T2-40	A	CS del1 from [-1311]	115	ACTCCCTTCATTTCTAAATATT	4	3.5%	ERE-core , GT-1-like K , ERE 1,
T2-43	A	W777 del1 [-770]	33	CGAGAGCATCGAACGTCCAC	1	3.0%	GmFDL19 Motifs
	B	ASP del1 [-1997]	94	CCCGCAAAAAAAAAAACAC	3	3.2%	GT-3a Box III , MBS, RY,
T2-44	D	M031 del1 from [-127]	17	TTATTTAGTATACCAAC	2	11.8%	AC-I , PbMYB9 BS ,
		M031 del2 from [-660]	328	AGTACTCGCAAGACTTACAT	5	1.5%	AT-rich K , Site I, EM2 (CArG
T2-45	D	APG del2 from [-942]	139	AGTGGGTTGTTTGGTGGTC	3	2.2%	SEF4, CLE-core , MYB46 BS 2
		ASP del1 from [-1508]	22	AGACTAATTTCTACTCAGATG	4	18.2%	AP1, GLM1(H.vulg), E-box,
		KR del3 from [-1336]	16	GCACGGGAGATAATAA	0	0.0%	
T2-47	A	Tm del4 from [-648]	141	AAGCTTTTTAAACCAAGCCA	1	0.7%	LFY BS
	B	APG del1 from [-1017]	135	TGGAGATCCTCCGTTTCAAT	8	5.9%	Box-1 (H.vulg), GT motif , O2
T2-48	B	KR del1 in 5'UTR from [-424]	42	GGCATAGCTTCACCGGAGCT	1	2.4%	CARE A1/A2
		KR del2 from [-797]	58	AATACAAAAGGAACTGTCAT	3	5.2%	D1 (H.vulg), Bsa, GTAC-motif
T2-49	A	Tm truncation from [-1130]	925	ATAATCCAGTCGACCGCCTC	4	0.4%	GSN (H.vulg), GA-2 , LBD
	B	ASP del1 from [-249bp]	349	ACTATATCTGGCCATGGGCCA	5	1.4%	RTBP1 BS (O.sat), Box II ("CC",
T2-51	A	Tm del1 from [-729]	53	TATGGATGGATACACGCGAC	4	7.5%	Amylase-element (O.sat),
		Tm del2 from [-951]	102	ACTTTTTCCGGGAGCTGTGG	3	2.9%	GT1-bx (O.sat), ATHB6 BS ,
		Tm del 3 from [-1201]	57	TAAAAAGATATGGTGAACGA	2	3.5%	Site II element, FLS2 EIN3/
		Tm del4 from [-1339]	26	GGGATGTAAAGTGGATGCGT	8	30.8%	Dof1 Bsopt (Z.mays), "ACTTA"
	B	ASP del1 from [-1283]	286	CTCCCTCCGTCCGGGTTTATT	5	1.7%	RY4, Sph, Inr, FLS2 EIN3/EIL1
		AB del2 from [-801]	65	TGCCTACTGTAGTTTTTTTA	5	7.7%	Py box (H.vulg), H-box II , H-
		AB del3 from [-982]	84	GTTGCATGCAAAGTGAACTT	4	4.8%	RY2, PROL box (O.sat), RY,
	D	APG del1 from [-878]	40	TACTCACCTTTGTTCTCATCC	2	5.0%	PacC BS (cons), GTAC-motif 3
		APG del2 from [-1355]	146	CTTGTTTTTTTTCGATAAAG	2	1.4%	NICE element 3 , EBS1,
		W160 del5 from [-870]	151	AAGAAAAGGTACTCACCTTTG	7	4.6%	GCN4 box (O.sat), hor1-box
T2-52	B	ASP del1 from [-234]	79	CTCCCTCCGTCTCATAATATAA	4	5.1%	CCAAT box-like motif 1, PB
		ASP del2 from [-2322]	74	TATGATCCATTGACTAATCT	3	4.1%	ATMYB2 BS , GLM1 (H.vulg),
	D	APG del1 from [-1196]	107	CTACGTGTGCATGTATGTCTA	7	6.5%	RY, G-box motif (O.sat), E3-
T2-54	B	KR del1 from [-1269]	278	CCAAAATTGCTTTGAGCGTT	1	0.4%	Motif I core ().sat),
T2-55	A	KR del1 from [-89]	19	GCCGGAGGAGGAGCGTGGG	0	0.0%	
		KR del3 from [-492]	257	ACCTGAACAAACCCCCTAAA	4	1.6%	CArG Box 2, AtMYB77 BS ,
		KR del2 from [-319]	12	CAGTTTGTCATG	1	8.3%	W-box
	B	KR del1 from [-607]	55	GGTGGCGCCATGACAACAA	1	1.8%	Oct-p (T.aes)
		W115 del2 from [-315]	272	ATGTCCCTTGGTAAGTTGGT	1	0.4%	CArG1 (SVP),
T2-56	B	ASP del1 from [-719]	161	TACTCCCTCTGTTTCTAAATG	1	0.6%	FLS2 EIN3/EIL1 BS(D)
		ASP del2 from [-1053]	26	GTCTCTCTTCTTTGCTTCGAC	1	3.8%	GTGTC element
T2-58	A	Tm del1 from [-413]	208	GGATCCGGTTTGCCTGCTCC	7	3.4%	Motif I core (O.sat), CCAAT
		BW del2 from [-1015]	134	AAGCACCATCAGAAAATGTT	3	2.2%	AE box 1, Gap box 1 , Myb-2 ,
T2-59	B	W115 del2 from [-1489]	71	CTCCCTCTGTAAAGAAATATA	2	2.8%	P-box (s) (Z.mays) (2x),
		W115 del1 from [-175]	21	GGAGGCGGCGGCGGAGC	0	0.0%	
T4-2	B	W115 del1 from [+379]	64	AGATCCCTGACATGCCTCGT	6	9.4%	R1, AB1 heptamer , MYB 1AT
T4-3	B	KR del1 from [-218]	303	TGCGGGTTTTCATTTTTCTTC	4	1.3%	Box III , RIN BSC "Others" ,
		KR del2 from [-671]	127	TGTGGGAGGCGAATCCTGTA	4	3.1%	ACGT box (O.sat), vicilin box ,
		KR del3 from [-1038	80	TGCTCGCGCGTTGGGTGATG	3	3.8%	C2b-BS , B2 (Z.mays,
		KR del4 from [-1179]	38	CTGGTGAACACTTTTCTCTGA	0	0.0%	
		KR del5 from [-1387]	20	AGCAGTAGCGTCCATCCCGC	0	0.0%	
T4-4	A	M037 del1 from [-312]	12	ATTGGGCTAGGC	1	8.3%	Site II element (O.sat)
	B	W209 del1 from [-534]	243	TAAGTTCTTCAACCGCCAAT	5	2.1%	E2Fb, GA-1 SITE , B2 (Z.mays,
		W209 del2 from [-1529]	30	CAACTACGCCAGTATATACTA	2	6.7%	I-box-like element, P1BS
T4-5	B	ASP trunc from [-536]	302	TCTACTTCTAATGTAGCAGTT	4	1.3%	Oct (d) (T.aes), CCA1 motif (2)
T4-6	B	del1 from [-629] in 83var	238	ACTCCCTCCTTCCATCTATATA	2	0.8%	RY, GTAC-motif 1 (O.sat),
T4-8	A	CS del from [-582]	24	GTTTAGCTGTGTCTACTTGTG	2	8.3%	Motif IV, VRE,
T4-11	A	Tm del1 from [-350]	114	GGCCCATCCCTAAGTGCGTT	7	6.1%	Motif g , AtMYB77 BS , TATA
		Tmdel2 from [-504]	44	GTGCTGCCACCTTGTGTCCG	1	2.3%	GT-1-like K ,
		Tm del3 from [-638]	29	TGGCACTTATTAGGGTGGCT	2	6.9%	CArG box , AuxRE
T4-12	A	W246 del from [-843]	12	TTGGTTACAGTC	0	0.0%	
T4-13	A	Tm del from [-292]	214	ACTACCCCGTCACGGTTTAG	1	0.5%	L1-box ,
T4-15	A	KR del1: from [-365]	8	AAAGAGAG	0	0.0%	
		KR del2 from [-1450	40	TGCACCCCTGTCAACGGGGA	2	5.0%	B1 (Z.mays, opaque2), as-1,
		KR del3 from [-1621]	81	TCCCTCCGTTCCAAAATAGAT	5	6.2%	GCN4 box (O.sat), PBF Bsopt
		Tm del4 from [-365]	27	ATAAAAAAAAGAAAGAAAA	0	0.0%	
		Tm del5 from [-173]	132	AGGCACACCACAAGTTGGA	1	0.8%	PbMYB9 BS
		Tm del6 from [-43] in 5'UTR	58	GGAAAAGAGAAAAATCCAT	2	3.4%	adja, ANAC092-BS,
T4-16	A	Tm del from [-1199]	450	ACGCTCACAGCGTGTTTGGT	2	0.4%	ABRE2, MYB46 BS ,
T4-18	A	KR del: [-771]	152	ATTCTGTGTCGGCTACTTCCC	2	1.3%	AC-III , ACGT-motif ,
	A	M037 del1 from[-240]	371	CTGTCTTAGGCCCGACTGTA	2	0.5%	AC-III , Site 1 E2F (D1,
		Watkins del2 from [-1391]	101	GCACATAAACATGCACTCAAT	3	3.0%	RY, GAGAGA motif, Z1
T4-19	A	Tm del from [-491]	32	ATTAGCAGTAGCGCTTGAGC	0	0.0%	
T4-20	A	KR del from [-600]	32	GATGCAAAAGCCGACCGCCA	2	6.3%	PB1 (H,vulg), CARE E,
	D	W### del from [-1397]	8	ATAGTAAC	0	0.0%	
T4-22	A	W141 del from [+37]	423	GGTATCGCGGTGCGCGACGG	7	1.7%	Em1b (T.aes), AuxRE, TATA1,
T4-24	A	SS del from [-959]	29	ATTTAAGCCTCATTTGGTTTG	2	6.9%	CArG box , SMRE 1,
	B	KR del from [-1265]	169	CGAAATGGCTTCACACACGA	8	4.7%	RY, ACGT box (O.sat) , ACGT
T4-26	A	Tm del1 from [-326]	52	CGAGACCTACAGGCTACAGC	3	5.8%	MYC (H.vulg), GS1b AC box 1 ,
		Tm del2 from [-634]	57	GTCTATCTGGCCATAGTTTG	1	1.8%	OsIDD10 BS (O.sat),
	D	ENT del from [-569]	143	CTAGTTTTTAGTAGTATTTTT	2	1.4%	Box-1 (H.vulg), Inr
T4-29	A	M031 del from [-350]	27	GAAAGGAAGGATAAATGATGA	0	0.0%	
		M037 del from [-341]	12	CGAAAAGGAAAA	0	0.0%	
	B	CE del from [-294]	22	GGCATTCATGGACGGAGCCT	0	0.0%	
T4-31	B	ASP del from [-768]	512	AGGCTGGTCACAATGGGCAA	4	0.8%	ABRE Bp , G-box (O.sat), AtSGR1-RY, GbWRKY1 BS
		KR del from [-1881]	116	ACTGATCTGATCTACGCCTAC	5	4.3%	ACGT box (O.sat), AAAGAT
T4-32	D	APG del1 from [-706]	94	ACACCTTCAGCTCACTTCAGT	2	2.1%	A/T-rich BOX1 , submotif D,
	B	ASP del1 from [-208]	14	TTGGAGACTGCGGA	0	0.0%	
T4-36	A	Tm del1 from [-580]	91	CAAAATAGATGACTCAACTTG	2	2.2%	GCN4 box (O.sat), GTAC-
		Tm del2 from [-959]	16	CTAGCTGACACTAAGG	1	6.3%	DREmut1 (T.dur)
T4-37	A	KR del from [-135]	217	CGTTGGAACTTACTCCCTCC	3	1.4%	CCGTCC motif (Z.mays), Box-
		W203 del 1 from [-149]	79	CCTCCGTAAACTAATATAAAA	1	1.3%	CArG (LeACS4-a)
		W203 del2 from [-1413]	11	CGCGCGCGCGC	0	0.0%	
	D	ENT del1 from [-159]	83	ACTTTCTACATTTGAACTGTC	0	0.0%	
T4-39	D	CR del from [+685] (Intron 2)	7	TCCAAGT	2	28.6%	Inr (O.sat), DRM2 BS2
T4-41	A	Tm del1 from [-482]	189	GCGTAAAGTTTTTTGGCATCT	2	1.1%	AACA motif (O.sat), CArG
T4-43	A	Tm del1 [-438]	36	AGCGCTAACAATTGAGAAGG	1	2.8%	Box I (core) ,
		Tm del2 [-513]	89	GATAATGCAGGGGAAGAGG	4	4.5%	Em1a (T.aes), G-box (Z.mays),
		Tm del3 [-742]	119	TGCATGACGGCGACTGATGA	1	0.8%	TGTCACA motif ,
		SC del4 [-1294]	59	CAAGCGTTGAAGGAACTACT	1	1.7%	ABRE1,
T4-45	B	W115 del1 from [-816]	67	TCCAAAGGTGAGAAAGAAC	2	3.0%	C-box , NIT2 BS III
	D	W199 del [-1305]	5	AATAT	0	0.0%	
		MK del1 [-539]	30	AAGAAATCGTGTACGGATGT	3	10.0%	Mybb, TCGTGT motif , as-1,
		MK del2 [-1563]	98	TGTGCCAGTAATTCATGGTAA	5	5.1%	RY4, GA-1 SITE , GT3-bx
T4-46	D	APG del from [-700]	121	AATCCCTCCGTCCCAAAATTC	4	3.3%	CCGTCC motif (Z.mays)(2x),
		ENT del from [+9]	9	GTCCGACGA	1	11.1%	pDRE
	A	Tm del1 from [+1]	12	ATGGCTGCGTCT	1	8.3%	AGCC-box
		Tm del2 from [-186]	71	GTCCACATCGCGTGTGCTGC	3	4.2%	E3-core , TA (O.sat), Myb-2
		Tm del3 from [-604]	118	GTTTCCTACTACAAATAAACT	2	1.7%	CACA box, "-300" box (O.sat),
		W199 del1 from [-1462]	6	TATTTG	0	0.0%	
		W199 del2 from [-736]	154	ATAACCCCATCCCCTCTCTCC	1	0.6%	GARE2 (O.sat),
T4-47	B	BW del from [-1061]	31	CGAGCTCTTTGTTACTCCATC	1	3.2%	TAACAAA box (H.vulg),
T4-48	A	CG del1 from [-1368]	22	TTTTCATTCATTCAAGAAAGC	3	13.6%	Gap box 2 , DOF BS1 ,
	B	CR del1 from [-508]	16	CACATCCGACTCTTGT	3	18.8%	E5-core, Box 2, pDRE
		SS del2 from [-751]	32	TCATTTCTAATGTAATCAACG	3	9.4%	A-box , HvGAMYB BS
	D	W141 del1 from [-758]	23	TGCTTTGCTTTTGCGAAACC	1	4.3%	GT-box (Z.mays),
T4-49	A	KR del1 from [-760]	184	AAACGTGTGGCCNNNNNNN	6	3.3%	Box P, AE box 1 , E6.3 (O.sat),
T4-50	A	M043 del from [-1091]	11	TATAATTCTAT	2	18.2%	Motif h, rAT-2 ,
		W786 del2 from [-1104]	251	CCCTTTAGTTTGAAGTATAAT	3	1.2%	Inr, RIN BSC "Others" , ABRE
		W579 del3 from [-657]	16	TTTTTTTTGCGCATAT	0	0.0%	
	D	APG del1 from [-1318]	102	CTCCCTCCGATCCATATTACTT	1	1.0%	WUS BS 2 (KAN2)
		APG del2 from [-893]	12	ACGTTGAAGCTC	4	33.3%	Box I (O.sat), NIT2 BS II , CARE
T4-51	A	W786 del2 from [-1506]	57	GTTGCAGGGATACCATTTTTA	1	1.8%	CuRE,
	B	W160 del1 from [-595]	19	CAAATATCTGCTGAGTCGT	1	5.3%	CArG (LeACS4-a),
T4-53	B	W115 del3 from [-348]	6	GTGGAG	1	16.7%	GmPHD BS
		W115 del4 from [-1595	6	CCGGGG	1	16.7%	CreA BS (cons)
		W115 del5 from [-1631]	5	GCCGC	0	0.0%	
	D	W199 del1 from [-1703	19	TGCTTCTAGCGGTACATCA	2	10.5%	GT3-bx (O.sat), MYB44,
T4-54	A	Tm del1 in 5'UTR from [-131]	16	CGTTGTGGCGGCGTCT	2	12.5%	GCC-consensus, LBD motif
		Tm del2 from [-1024]	46	TGTGCGGGTGTTTATTACTTT	2	4.3%	E1-core , Pb1 (O.sat)
	D	SS del1 from [-520]	206	CAAGGGGTGCTTACTTGACT	2	1.0%	G-box , E3.1 (O.sat),
T4-55	A	M031 del1 in 5'UTR from [-226]	16	CAGGATTGAGTTTTTG	0	0.0%	
		M031 del2 from [-725]	28	ACCATTCTGAAAGCATGTTC	1	3.6%	A-box
		M037 del3 from [-88]	33	GGGACTGCAGCGGCGAGGT	0	0.0%	
		IQ del4 from [-458]	15	TTAACCGATCCCCTA	1	6.7%	MBS
T4-56	A	Tm del1 from [-97]	53	CCGCCCCGGGGACGGCGAG	2	3.8%	NIT2, TATA-box (O.sat)
		Tm del3 from [-381	9	GTGGAGGGG	0	0.0%	
		Tm del4 from [-649]	5	CACGA	0	0.0%	
T4-57	D	APG del1 from[-931]	150	GAGCACTGCTACACGGACGA	4	2.7%	RY, Box-1 (H.vulg), ABRE3, CRE
T4-58	B	W115 del1 from [-977]	76	TTGATAACCTTTTTGTAATTG	3	3.9%	D2 (H.vulg), EIN3 BS2 , Pb1
	D	SS del 1 from [-227]	14	ACCGGCCTTCTCT	0	0.0%	
		SS del2 from [-422]	39	GCCTGCATTGGATGACACGA	6	15.4%	UV LRE , R-motif (Z.mays),
T4-59	A	M043 del1 from [-80]	22	CCACCTCGCTCGGCAGCTCG	0	0.0%	
T5-1	B	ASP del1 from [-1467]	39	GTGTATTTTAGTCTAGATTAA	4	10.3%	C1-motif
T5-2	A	APG del1 from [-1153]	177	CCCTTTTATAGTGATATGAAC	6	3.4%	P-box 2 (T.aes, LMW glut)
	B	APG del1 from [-2132]	175	CCTTTTATAGTGATCTAAACG	1	0.6%	C1BS1
T5-3	B	W199 del1 from [+2073] [Intron 3]	20	CAAGATGAGACCTGTCTTCT	0	0.0%	
		BA del2 from [-560]	32	TCCGACGACCATCCGCAAAA	1	3.1%	GA-1
	D	ASP del1 from [+1620]	256	CTAACCCTAAATGAGTTTTAA	1	0.4%	9-mer box (Z.mays),
		ASP del2 from [+318]	668	CAAAATTGACAAACAGACAA	5	0.7%	S1, AT-rich K , NDE Box 3 ,
T5-5	B	ASP del1 from [-1512]	272	ATCACATCCCTAGTCTGGTAT	4	1.5%	D1 box , GA-6 , AC1, WK box
T5-8	A	CS del1 from [-407]	73	CCTCACCTACCATCCTTCCTCT	5	6.8%	H-box, MYBa, FLS2,
		M037 del2 from [-703]	160	TGCAGTAGAGTTGCTTACCC	1	0.6%	CCAAT box
		BW del3 from [-910]	13	TGCAGTAGAGCAA	0	0.0%	
	D	SS del1 from [-1192]	42	GTTTGTGGGTTTGACTGTTG	3	7.1%	WRKY1, ATCATT motif, W-box
T5-10	A	CS del1 from [-192] (UTR)	47	AGCTGCTCGCGCGCACCCTC	5	10.6%	Em2(T.aes), CTCTT motif ,
T5-11	A	KR del1 from [-827]	131	CCTTGTTCGTTTAATCCTCCT	2	1.5%	CArG Box 2 , GA-4 ,
T5-13	A	W199 del1 from [-1386]	42	GCCTTTCAACATAAACGTGG	5	11.9%	Em1a, DOF-BS1, T/G-box,
T5-14	A	Tm del1 from [-295]	215	AATACTACCCCGTCACGGTTT	1	0.5%	L1-box ,
	D	Tm del1 from [-314]	117	TTGGCAATGCGAGATGATGA	2	1.7%	G-box (O.sat), E4-core ,
T5-15	A	Tm del1 from [-430]	41	GCGGGGGGCTGGGCTAGGA	1	2.4%	Site II element ,
		Tm del2 from [-1009]	980	AAAAGCTTAATCATTTCCTTG	4	0.4%	C-box (Opaque2), N-box,
	D	SS del1 from [-697]	303	ATAGGCAAATTTGACAAATT	0	0.0%	
T5-19	A	KR del1 from [-1708]	344	AAGCATCTTAGCGGCGGACT	5	1.5%	Box C, Zc2 A/T-2 (Z.mays),
T5-21	A	ASP del1 from [-276]	49	CTTGATGGGCCTGCATGGGC	0	0.0%	
		ASP del2 from [-699]	251	CGCACCCTCAAGACGCCCGC	5	2.0%	Box C, AT-rich motif, BBS, L-
		ASP del3 from [-1275]	927	ACTATTTTTTGTGTCATGAGC	4	0.4%	GS1b AC box 1 , EM1 (CArG
T5-22	B	KR del1 from [-774]	93	TTTGGTGTCGCGAAGTGGTT	6	6.5%	"ACTTA" motif , GT-1-like K ,
		W160 del2 from [-1688]	230	CTGGGAAAAAAATTATATGA	5	2.2%	B1 (Z.mays, opaque2), GC-box
	D	AB SNP cluster from [-179]	15	CAATGGGCAA (WT)	1	6.7%	p33TCP20 BS2 ,
			15	CAATCGACGG (mt)	2	13.3%	GCCGAC motif (H.vulg),
T5-24	A	Tm del1 from [-623]	34	CTTTTTGCGGGTATGCATGCA	1	2.9%	RY,
	B	ASP del1 from [-529]	408	AATTACTACCTCCGTCCGGGT	8	2.0%	RY4, "-190 half G-box (core)",
	D	ASP del1 from [-83] (inside 5'UTR)	580	TAAAGAAAACTAATAATGCAT	8	1.4%	RY4, -190 half G-box (core),
		ASP del2 from [-1109]	282	ATCCCTTATAAAGAGGTCCAA	2	0.7%	CCA1 BS2, DOF2 domain 3 ,
T5-25	A	Tm del1 from [-751]	32	GGCCTCTATGTGAGGTGTGC	0	0.0%	
T5-27	A	Tm del1 from [-1282]	876	CAACATGGCACACGTAGGTT	7	0.8%	NDE Box 3 , GBF1 BS7 ,
	D	ASP del1 from [-731]	247	ACTACTCTCTCCGTCCGAAAA	2	0.8%	EE 1, FLS2 EIN3/EIL1 BS(P),
		ASP del 3 from [-1446]	495	TGTTTCTAAGCTGCCACCCAT	8	1.6%	MNF1 BS core (Z.mays), Site I,
			46	CGTGGGACCCCTGATACGTC	5	10.9%	ACE5, ABRE1, T/A-box ,
T5-28	A	KR del1 from [-541]	172	AACTTGGCAATAAAAAGGGA	1	0.6%	Box A,
T5-29	A	Tm del1 from [-860]	123	TTTCTTCCGTCCTAAAATTTT	4	3.3%	Box A, E5-core , LBS, CArG
	D	APG del1 from [-713]	76	CTGTCGGTTCGTTGTGTGCA	2	2.6%	DPBF-1/DPBF-2, EBS1,
T5-30	B	APG del3 from [-872]	347	GAAAATTCTAGATTGTAACTG	3	0.9%	GARE2 (O.sat), AE box 1 ,
		ASP del1 from [-188]	25	GGGTGTCTGGGTTTATATAGC	2	8.0%	Motif g, TATA1,
		ASP del2 from [-324]	17	TTTTTTTGAATATTTTT	1	5.9%	CHR,
	D	APG del 1 from [-567]	437	AGAAAATTCCAGATTGTAACT	4	0.9%	GARE2 (O.sat), box1, G-box
T6-1	A	Tm_del1 fr [-16]	13	AGCTAGCTGCCTG	1	7.7%	Motif IV
		Tm_del2 fr [-346]	17	TCCTTCAAAAAAAAAAA	0	0.0%	
		M049_del3 fr [-425]	320	CTACCGCCGCCCCGATTCGC	7	2.2%	SEF4, TAACAAA box (H.vulg),
T6-2	A	KR_del4 fr [-432]	19	ACTGCATCCCAAATTCACC	0	0.0%	
T6-3	A	APG_del1 fr [-1075]	709	ACAGTAAAAAAAATCGTGCT	3	0.4%	FLS2 EIN3/EIL1 BS(P) , CArG
		GT_del3 fr [+1071]	359	ATGACATTCTCATGGCGCAG	4	1.1%	UN Z1, NIT2 BS II , WUS BS
	B	ASP_del1 fr [-258]	33	ACTAGCAGCATGTGTTTCCA	1	3.0%	CACA box,
		ASP_del2 fr [-1482	48	AGTCAGTGGTTACGCTTAAC	0	0.0%	
		ASP_del3 fr [-2383]	436	ATCCTCCAACTTTGGGCCAC	3	0.7%	TATA box (Z.mays), Fp23/IV ,
T6-4	B	ASP del1 from [-503]	22	TAAAAAAATATATTTACATTAA	0	0.0%	
		ASP del2 from [-606	24	TACTATAATTTTTTTTTGCGAA	0	0.0%	
	D	SS del1 from [-174]	44	TGGGGAAGAAGAAAGAGAT	5	11.4%	Motif VII , GAGAG motif , Box
T6-5	A	APG del1 from [-854]	147	ATTTTTTTTATTTTTTGCGAG	3	2.0%	E2-core , TERE, GTAC-motif 2
	B	ASP del2 from [-495]	413	CCTATATATACACTACTGGAG	4	1.0%	C1 DBS (Z.mays), B1 (Z.mays,
T6-6	A	Tm_del1 fr [-158]	70	GTTAACTTTCAACTTATGAGT	2	2.9%	CARE1 (O.sat), CAT,
		Tm_del2 fr [-791	16	CGAACTTGTAGTTGGT	1	6.3%	CArG (LeACS4-a),
		Tm_del3 fr [-867]	721	GGGAGTGTCTATTTGACACT	3	0.4%	HSE3, Fp12/III , R2R3-MYB
		W141_del4 fr [-657]	86	TGATTGGATATACGTACTTGC	10	11.6%	Motif h, E-site , CCAAT box 1 ,
T6-7	A	Tm_del1 fr [-1161]	42	TTTCCCTGGCTGATCGCCGA	2	4.8%	dps1-3 (O.sat), PacC BS
T6-8	A	Tm_del1 fr [-539]	119	TCATGCCATCTACTCCCTCCG	1	0.8%	Box-1 (H.vulg)
		Tm_del2 fr [-845]	265	GCAAATTCTTGGCTCCGCCA	4	1.5%	S1, CDE, ACGT2/DRE2 , G-box
T6-12	B	W115_del1 fr [-904]	393	GGATAAAATCACCTACTACTA	3	0.8%	CCA1 BS2, E3.3(O.sat), GTAC-
	D	ENT_del1 fr [-1421]	150	CAACAGCGATCCTAGCCTTC	3	2.0%	RY, E2Fb, B4 (Opaque-2,
T6-13	A	KR del1 from [-999]	219	TAGTACTACCTCCGTCCCGGT	4	1.8%	Box A, E1-core , 3'/AT-rich J1 ,
		KR del2 from [-1398]	12	GCGGTGAATCGA	1	8.3%	GT1-bx (O.sat),
T6-15	B	ASP del1 from [-461]	45	CGGTCGAAGAAAACTATTAG	2	4.4%	rAT-3, NTL6 BS
T6-16	A	KR_del1 fr [-290	66	ACAGGTAGCTCATCTAGCAG	1	1.5%	bZIP29 BS
		CH_del2 fr [-1331]	18	GAGAAGAAGCTAGATGAA	1	5.6%	MBF1c RE
	B	KR_del1 fr [-85]	76	CAGCTGCATGCGAGCGCGG	3	3.9%	RY, TATA box (O.sat), AtSGR2-
T6-17	A	KR_del1 fr [-996]	196	TGAACCCAACCAAGGGATTC	4	2.0%	TERE, 7B element (Z.mayss),
		KR_del2 fr [-1292]	57	TACCAAATGCATTAAGCTGAA	2	3.5%	Motif IV, MYB46 BS3
	B	KR_del1 fr [-728]	45	GAACTAAAACCACGACGAGT	1	2.2%	Region C (Z.mayss),
		KR_del2 fr [-841]	49	GCCGTACACACAGTCCATTT	3	6.1%	PROL box (O.sat), SP8a, CARE
T6-18	A	KR_del1 fr [-717]	9	AAACTATCG	0	0.0%	
		KR_del2 fr [-794]	83	GCAACATGTGTGTGTACGTAT	8	9.6%	AACA box (D) (O.sat), RY2,
		W115_del4 fr [-1154]	424	GAGTCAATGAGCGTCGTATA	0	0.0%	
T6-31	A	APG del2 from [-259]	26	ATAAAATAGAATACGAGAATC	1	3.8%	DRE1
		Tm del3 from [-637	101	ACGAGCATAGATGACCATGA	0	0.0%	
	A	Tm del1 from [-1079]	13	CTGCAAACAAGGG	0	0.0%	
	B	ASP del1 from [-720]	104	ACTCCCTCCGTCCGGAATTAG	2	1.9%	Box A, Box-1 (H.vulg),
T8-1	A	Tm del1 from [-1751]	149	GAAACTCATGGGCACTAGCA	2	1.3%	Em1a (T.aes), G-box ,
	B	ASP del1 from [-787]	166	CTCCCTCTGTTCCTAAATACTT	4	2.4%	ERE-core, E motif (O.sat), Inr,
T8-2	B	APG del1 from [-422]	77	TCTTCCCCTCTTCACGGTTAC	2	2.6%	CCAAT box 1, Py box (H.vulg),
		APG del2 from [-1123]	457	TTTTTCCGATAAACTGGCAG	6	1.3%	GATA/2 , GBF1 BS2, EE (TOC1)
T8-3	A	Tm del3 from [-390]	59	ACTCCCTCTGTCCCAAAATTC	4	6.8%	E5-core , CCAAT box-like motif
		Tm del4 from [-896]	10	TTAGTAGTAC	0	0.0%	
		Tm del5 from [-1598]	13	AGTTCGTACATAC	1	7.7%	GTAC-motif (O.sat),
	B	ASP del1 from [-255]	204	TGTGCTGATCATCAATGTTTT	3	1.5%	PROL box (O.sat), E3.1 (O.sat),
	D	Tm del4 from [-807]	126	CTCTGCAGCTTTTTGCAGAG	2	1.6%	MRE-core (AtF3H), MYB46
T8-4	B	ASP del1 from [-1327	179	CCGGCAATGCTACACGTACA	5	2.8%	TGTAAAAG (2 occurences)
	D	APG del1 from [-497]	132	ATGCCTGGCTGTGAATGTGTA	2	1.5%	RY1, GC-box (T.aes),
T8-5	D	APG del1 from [-799]	14	CCGCACGAGAGATG	1	7.1%	GAGAGA motif,
T8-6	A	Tm del1 from [-401]	28	CCGGATCGACCGGAGTGGA	1	3.6%	ICEr2
		Tm del2 from [-799]	7	ACAACTG	2	28.6%	Mybb, BpMYB106 BS (petD),
T8-9	B	CP_del1 (in Exon 8) from [+17589]	36	GGAGTCGCCATGGAATCTTG	1	2.8%	S1
T8-10	B	APG_del1 from [-720]	519	AATGGATCCCTCGTGTGCCA	3	0.6%	Inr element (A.fat), TCGTGT
	D	SS_del1 from [-1127]	22	ACTCCAGACTACGACCCTAAT	0	0.0%	
T8-11	B	ASP del1 from [-687]	13	GTGGAAATGGCGG	0	0.0%	
T8-12	A	Tm_del1 from [-669]	41	TAGAAAATTCAGTCGACTGA	4	9.8%	GCN4 box (O.sat), Fp6/II ,
		Tm_del2 from [-2013]	24	TTCAAAAGAGTGTTTTTCTTG	5	20.8%	CHR, D2 (H.vulg), ACT-box
T8-13	B	W_del1 from [-1205]	12	GGGAGGGGAGAG	0	0.0%	
T8-15	B	APG del1 from [+516]	325	ATACTCCTTCATAAAAGTCAC	2	0.6%	Em2 (T.aes), Pybox (O.sat),
T8-17	B	KR del1 from [-84]	54	CGCTTTGCGGCCGGTGGTC	3	5.6%	CLE-core, GS1b AC box 1 (AC-
T8-19	B	APG del1 from [-817]	30	CAGGCTGTCCTGCCTGATCA	1	3.3%	ATMYC BS, same motif for
		APG_del2 from [-1243	176	CACATACATATGTTGGCTAAT	7	4.0%	CArG box , FLS2, EIN3 BS2 ,
		APG_del3 from [-1620]	18	TGGATGTTTTATAACTA	0	0.0%	
T8-21	B	ASP_del1 from [-889]	103	GTTTTTTTTTTTGGCCCGGA	6	5.8%	ERRE, GT-1-like K , GT3-bx
		ASP_del2 from [-1614]	14	ATTTTTTTTACGGT	0	0.0%	
T8-22	A	Tm_del1 from [-1176]	240	CTCCCTCCTTCCATCTATACA	2	0.8%	B3 (Z.mays, opaque2), GTAC-
T8-23	B	W_del1 from [-1275]	15	TAATGTCGGTTCCTT	2	13.3%	GCCGAC motif/ DRE1 (H.vulg,
T8-25	A	Tm_del1 from [-300]	129	AAATCCCCAAATTTAAAAAA	2	1.6%	Inr, AAAT-region (WRKY)
	D	W160_del1 from [-1197]	15	TTCGTCTATGCCATA	0	0.0%	
T8-26	D	SS_del7 from [-12]	38	AGCTTTTCGCGCCTGGTCGG	0	0.0%	
T8-27	B	ASP_del1 from [-681]	89	GGACGTACAGCGCACTTGCT	6	6.7%	ACGT motif , TGA1, Hex
T9-3	A	Tm_del1 fr [-158]	31	CAGCTGCTCCTGCAACCTGC	0	0.0%	
	B	KR_del1 fr [-1026]	302	CTATATCAACCATTTACTTGTT	2	0.7%	inverted telo box, telo box
		W115_del1 fr [-503	34	TAGTTTGTGATTCGTCAATAA	2	5.9%	Box II, W1-box ,
T9-4	A	Tm_del1fr [-291]	12	AGCCCGCACACA	1	8.3%	SITE II (-50) (O.sat),
		Tm_del2 fr [-696]	56	AGGAGAATGCCGGGAAACG	1	1.8%	SEF4,
	D	APG_del1 fr [-317]	313	GCATGTACAACGCCGGCGCT	2	0.6%	CL3, TERE,
		SS_del2 fr [-142]	16	TGGCGCCTCGCGCCTG	1	6.3%	E2F BS
T9-5	B	ASP_del1 fr [-673]	820	AAAAAAGTATAAGGGAAATA	0	0.0%	
		UK_del2 fr [-1184]	24	nb: 5' part of ASP_del1		0.0%	
T9-6	A	CS_del1 fr [-487]	42	CCACCTCTGTAAAAAGTACTA	2	4.8%	HvH21 BS (H.vulg), EM2
	D	APG_del1 fr [-505]	114	CTCCCTCCATCCGGAAATACT	2	1.8%	Box-1 (H.vulg), CL2,
T9-9	A	Tm_del1 fr [-1357]	101	TCCCTCTGTTCATTTTTATAAG	3	3.0%	EM2 (CArG box 2) , Inr, TERE
	B	ASP_del1 fr [-973]	120	ACAACAGTAATCATTGCCTTT	2	1.7%	DOF BS1, PRE1 (AG1)
		APG_del2 fr [-123]	122	AGCTGGAGAGAAGGACAAA	4	3.3%	GLM2 (H.vulg), GTAC-motif 3
T9-11-1	A	KR del1 from [-594]	85	CTCCCTCTGTAAACTAATATA	2	2.4%	CArG (LeACS4-a) (2x)
T9-13	A	M031_del1 fr [-132]	16	TCTTCATTTGGCCTAG	4	25.0%	CCAAAT box, Gap box 2, Gap
T9-14	A	APG_del1 fr [-424]	148	TTAACTAAAGTGGACATCATA	5	3.4%	DOF (H.vulg), RY2, AC2, SITE II
		Tm_del2 fr [-1095]	503	ATGTGGTTCAGCAACTTGTG	2	0.4%	FLS2, CArG2,
T9-15	B	ASP_del1 fr [-371]	472	CACCTCCTCGCTGCTGGGCT	7	1.5%	OCT (Z.mays), Zmhox1a BS 1
T9-16	A	Tm_del1 fr [-388]	354	GAGGGTGCTTGGATACGTTT	7	2.0%	E3-core , GA-5 , GA-6 , AT-rich
	B	APG_del1 fr [-534]	166	ATTCGTTAGCACATTGTTAAT	4	2.4%	GC-rich motif, Oct(d) (T.aes),
	D	SS_del1 fr [-499]	193	AGGAATGTATCGTCCCTGGT	2	1.0%	CARE1 (O.sat), GTAC-motif
T9-18	D	Tm_del1 fr [-833]	156	TGCTACTATGAGTCATGCATG	9	5.8%	RY4, GCN4 box (O.sat), C-box
T9-20	B	ASP_del1 fr [-942]	47	ATCGACTTGGGCCTCCTTGG	3	6.4%	GCCGAC motif (H.vulg),
	D	ENT_del1 fr [-1698]	379	AATAACTAGATCGAGAGTGT	3	0.8%	UN Z1, CCA1 BS1, ABRE
T9-21	A	Tm del1 from [-1486]	16	TTTTTTTTTGAGGGGT	1	6.3%	STRE
	B	ASP del1 from [-948]	52	GATAAATATAAAATTAATATAA	1	1.9%	as-1,
	D	APG del1 from [-859]	69	TAGACATGACAAATTTAACGT	1	1.4%	T/C-box
		APG del2 from [-1443]	116	ATATCAAAAAAAGGACGGAT	5	4.3%	Box A, Box II , CArG box , A-
T9-22	A	Tm_del1 fr [-812]	128	ACCGCCCGTCTCTTCTCTTCT	4	3.1%	R-motif (Z.mays), Inr, Gare
	B	APG_del1 fr [-377]	612	TCCTATGTACGACATCATTTA	2	0.3%	CArG (LeMAN4) , GTAC-motif
	D	Tm_del1 fr [-175]	65	TTAGAGAGAAACAAATGACA	4	6.2%	GA-1, AE box 2 , Inr, E3.2
T9-23	A	Tm del1 from [-365]	73	TTGAAGGTATTGCACTGTTG	1	1.4%	AS1-AS2 BS II (BP Y)
	B	SS del1 from [-1172]	18	GCCGCCCTATGGCTTGCC	0	0.0%	
	D	ASP del1 from [-513]	363	TGATCATCGGTAGACAAGTTA	2	0.6%	AS1-AS2 BS II (BP Y), S3,
T9-24	A	M031_del1 fr [-834]	169	CCTTGTACTATTTCGTTTTTTG	5	3.0%	ATMYB2 BS, E3.2 (O.sat), FLS2
		M037_del2 fr [-385]	250	TACCACCCTTACAGGGAACA	1	0.4%	GS1b AC box 1
	D	CG_del1 fr [-522]	1,652	TTTTCTAAAAAAGAAATTAAT	7	0.4%	Em1b (T.aes), MRECHS, MNF1
T9-25	B	ASP_del1 fr [-308]	114	CCACTCCCTTCGTCCGAAAAT	6	5.3%	Box A, Box-1 , AE box 1 , LeCp
T9-26	A	CS_del1 fr [-289]	123	TCCATCCGTATCAAAATTCTTGTCTTAAATT		0.0%	
		CS_del2 fr [-484]	13	AATGCGCAATGCG	0	0.0%	
		M031_del3 fr [-353]	23	CATCGTATTTAGATAAATCTA	1	4.3%	CARE C
	B	ASP_del1 fr [-381]	134	GCGTCTCCTGCAACGCTCTG	2	1.5%	P-box (Z.mays, zeins, T.aes),
		ASP_del2 fr [-552]	216	CTACGCGCGGCGGGCGACC	6	2.8%	Em1a (T.aes), TATA box
	D	CS_del1 fr [-992]	831	GCATTGGAGTCGAATGAAAT	2	0.2%	AT-rich K , EE2
T9-27	A	Tm del1 from [-422]	16	AATCCATTTTTTAGCA	1	6.3%	CArG (RIN-a),
	B	Tm del1 from [-1005]	252	TTAGTTTTATGCATCCCCCCA	0	0.0%	
	D	APG del1 from [-418]	42	CACCTAGAGGTAAGTGTTCT	3	7.1%	CARE A1/A2, S2, Alfin1,
		APG del2 from [-491]	14	CATGTGTGTACATG	2	14.3%	ATMYC BS (27x), CARE D,
		APG del3 from [-782]	273	AGGGCATGTACAATGGTGCT	12	4.4%	Motif I core (O.sat), Em1a
		APG del4 from [-1208]	44	TACATAAATAAAGTTAACCTA	0	0.0%	
T9-28	A	APG_del1 fr [-974]	120	CACTAGTAGAAAAATGGTCA	5	4.2%	WA (WRKY), DRE2, W-box
	B	APG_del2 fr [-1262]	647	GGTGGACCAGCCAACATCGT	4	0.6%	Fp12/III , RY-RY , AtSGR2-RY ,
T9-29	A	Tm_del1 fr [-419]	274	ATGTCCTGTTAGACTGTATTT	3	1.1%	LRE-TATA AA1, GT-3a Box , Z1
	D	APG_del1 fr [-336]	179	AAAACAGTTTGTCTAATTCA	7	3.9%	Box C, ABRE1(Z.mays)(2x),
		APG_del2 fr [-986]	132	TATCGCGGAACATGGTAAAG	3	2.3%	S1, I-box-like (Z.mays), ERE-
T9-30	B	ASP_del1 fr [-283]	90	ATGTTCGCGCTAAACCTTCTT	1	1.1%	LBD motif (Z.mays)
T9-31	A	Tm del1 from [-510]	10	GTAACATCTT	1	10.0%	ARR10 BS3
		Tm del2 from [-589]	34	CCCCCACCCCCCTCCCCCCG	4	11.8%	GS1b AC box 1, rAT-3, Box I,
		Tm del4 from [-981]	16	TTAGTACTTCCTCCAT	0	0.0%	
		Tm del6 from [-1648]	8	TGCATGCA	1	12.5%	RY,
		Tm del7 from [-1853]	7	CTGCCGC	0	0.0%	
	B	ASP del1 from [-242]	21	GAGGGGGTGATGCTTCCGG	1	4.8%	G2-like/Zn-finger TFs BS
		ASP del2 from [-479]	33	TCCTTCGTCCTCGGGTGCGAT	0	0.0%	
		ASP del3 from [-1218]	103	TCCCTCCGTTTCGAATTACTT	2	1.9%	Box-1 (H. vulg), ACGT-motif
		ASP del4 from [-1516]	462	TCATTTCTATGCATGCAAGGG	6	1.3%	RY4, GT motif , GA-2 ,
		SS del5 from [-489]	565	TCGGGTGCGATCGAGGACTA	2	0.4%	BBS, FLS2 EIN3/EIL1 BS(D)
	D	SS del1 from [-665]	81	AAACTTTGTAAACTTTGACA	6	7.4%	AT-2a , T1/T2 (2x), as-1 [2] ,
		UK del2 from [-709]	135	CATATACAATAACAAATCAAT	3	2.2%	EIRE (core), S1, TAGTCAAC
T9-32	B	ASP_del1 fr [-265]	435	CCAGACGTATCTGGACGGCC	4	0.9%	CCA1 BS2, GA-4, AACA motif
	D	APG_del1 fr [-505]	722	GGTTCAACGTCTAACAAAGG	3	0.4%	TATA box (Z.mays), HB05/
T9-33	A	Tm_del1 fr [+2]	19	ATAAGGAAGGCTGAGGTTT	1	5.3%	AC1
		Tm_del2 fr [-371]	814	GAAATAGGCTCCAGTAACAA	4	0.5%	CARE2 (O.sat) (2x), SS-30 ,
	B	ASP_del1 fr [-582]	408	GTACTCCCTCGGTAAACTAA	1	0.2%	XXIII/GATA-1 ,
T9-34	A	Tm_del1 fr [-1605]	273	TTAGGCTATTTCCCTCAACAG	3	1.1%	G-box, TERE, CArG (133R) ,
	D	SS_del1 fr [-792]	194	CGCGCACCCGGCCAGCTTTG	1	0.5%	E-box (BRI1, P1_1) (O.sat)
T9-35	A	Tm_del1 fr [-364]	152	GGGCGCATGATTGTAAACCT	3	2.0%	G-box, GS1b AC box 3 , CM4,
T9-36	A	Tm_del2 fr [-233]	373	ACAGCCCATAATTAAAGTGT	4	1.1%	AS1-AS2 BS II, Fp2/I , TCGTGT
T9-37	B	ASP_del1 fr [-926]	88	CGTCATTGAAAGAAAGAAAA	3	3.4%	GA-1, A-box , CRE 36-39
T9-38	B	ASP_del1 fr [-262]	75	TGAATAGGAGCCGTCTGCCT	3	4.0%	CCA1 BS2, G-box 5 , CRE
		ASP_del2 fr [-508]	354	CCGTATCTTGCTTTTTTTTAAT	0	0.0%	
		W246_del3 fr [-955]	20	TACCGCAAGTAAGTAATAAA	1	5.0%	DRE1
	D	APG_del1 fr [-327]	488	ACGCAGCTTCAGTGACTTGA	3	0.6%	GT motif, ACGT2/DRE2 ,
		APG_del2 fr [-1470]	496	ATTTTGCTTTAAATCGTGATA	4	0.8%	GA-5, TCGTGT motif, NAC/
T9-39	D	APG_del1 fr [-603]	18	AAATCGAGATACTGGTAT	1	5.6%	I-box-like element
T9-40	A	APG_del1 fr [-1430]	499	ATTATGGACGGACGAAGGG	2	0.4%	C-box, OsIDD10 BS (O.sat)
	B	ASP_del1 fr [-1188]	58	AGACTCAAAAAAAAAAATAG	2	3.4%	LTRE (H.vulg), ABRE2,
		APG_del2 fr [-1726]	496	TTTATTGACGGACGAAGGGT	1	0.2%	AtIDD8 BS,
T9-41	A	Tm_del1 fr [-1157]	257	CAGAGATGGCAATCTATTTAG	5	1.9%	AE box 1, EL1, PacC BS2 , CRE
T9-42	A	M031_del1 fr [-467]	103	CTCCTTCTGTTCAGAATTACT	2	1.9%	Box-1 , ICEr2
		M031_del2 fr [-644]	49	CCACAACGTCCCTTCCTCCC	1	2.0%	AACGT element
		M031_del3 fr [-974]	63	CATACTCCCTTCGTTCGGAA	1	1.6%	GAAATA element
		Tm_del4 fr [-644]	582	ACAACGTCCCTTCCTCCCCC	3	0.5%	E1-core , NIT2 BS III , RY,
T9-43	A	Tm del1 from [-1183]	75	CTCTGTCCCATAATATAGAG	4	5.3%	CDA-1 , submotif C, TGTCACA
		Tm del2 from [-1719]	341	CAGTTGCCATGTGCGCTGCA	4	1.2%	Motif I core (O.sat), GLM2
	D	APG del1 from [-313]	403	ATATACCTGTCCAAACCTCGG	3	0.7%	CDA-1 BS , dOCT (Z.mays), Inr
		SS del2 from [-678]	161	CTTTCAACCAGATTAAGTTTG	5	3.1%	CDA-1 BS, S3, WUS BS 1
T9-44	A	Tm_del1 fr [-246]	188	GGCATGTTTGGTTCCAAATA	3	1.6%	MBSII box, MYBa, R2R3-MYB
	B	ASP_del1 fr [-890]	236	ACTAACTCCGTCCGGGTTTAT	5	2.1%	Box A, ERE-like, MYB46 BS 2
		ASP_del2 fr [-1270]	149	TCCCTCCGTTTCTAAATATAA	4	2.7%	Motif VI , FLS2 EIN3/EIL1
		CP_del3 fr [-207]	143	GCAATAATAATTTACAAAAGT	1	0.7%	CArG (LeACS4-b)
	D	ASP_del1 fr [-751]	52	ATCCTTCCTAACGTTGATGAT	3	5.8%	T-box (O.sat), GARE1 (O.sat),
		APG_del2 fr [-332]	87	GGCGGGCAAGGGGATCGAC	2	2.3%	ERE-core, SP8b,
T9-45	D	ASP_del2 fr [-992]	237	TTCATTCACAATGCTATGTAC	1	0.4%	EM1 (CArG box 1),
T9-46	A	CS_del1 fr [-585]	12	GATACGGATGTA	1	8.3%	E5-core
		Tm_del2 fr [-585]	36	GATACGGATGTATCTGTGACC	3	8.3%	A-box, Inr, FLS2 EIN3/EIL1
		Tm_del3 fr [-964]	9	GGAGAGACA	1	11.1%	AuxRE,
		W777_del4 fr [-1665]	10	ACATTCACGG	0	0.0%	
		KSA_del5 fr [-52 to -467]	416	GACGGAGGTAGCGTAGCGT	5	1.2%	RY4, AuxRE (comp) , Legumin
	D	ENT_del1 fr [-776]	37	CACGTTAACGGTAGCCGGCG	3	8.1%	EIRE (core), Myba', 16 KK ,
T9-48	A	Tm_del1 fr [-8]	182	AATGGCGCCATCGCAGTTGC	0	0.0%	
		W246_del4 fr [-1609]	68	CCAGGTACCCTGGCGTCTGG	1	1.5%	Wi-box
T9-49	B	ASP_del1 fr [-770]	1,292	TTCTTACGGAAACAACTCAG	2	0.2%	box b (Z.mays), CL2
		W387_del2 fr [-766]	248	TACGGAAACAACTCAGATTTA	1	0.4%	CL2,
	D	CR_del1 fr [+169] Exon1	45	TCGGCAGCCGCGGCCTCTGC	1	2.2%	AGCC-box
T9-50	A	KR_del1 fr [-470]	69	CTGTAAACTAATATAAGAGCG	2	2.9%	CArG (LeACS4-a) (2x)
	B	ASP_del1 fr [-558]	307	AGGCCTTCTTTGGTTCATAGC	4	1.3%	Z1 (Z.mays, opaque2), CArG
	D	APG_del1 fr [-854]	110	CATCTACATTATGGGATGGAG	2	1.8%	P-box (s) (Z.mays) (H.vulg),
T9-51	A	KR_del1 fr [-1320]	42	CTACCAGACGCGTGCTTTAGT	1	2.4%	MYC2/MYC3/MYC4 BS4,
		M049_del2 fr [-542]	18	GCTAGTCAAATGAGTGCT	2	11.1%	WB (WRKY), E-box (BRI1,
T9-53	A	ASP del1 from [-399]	135	GCTGATTGGATGGAACCATT	3	2.2%	CCAAT box 1, B3 (Z.mays),
		ASP del2 from [-909]	380	GCCCACCGGGACCTCGCTGC	1	0.3%	O2 BS-1 (Z.mays, opaque2),
T9-54	A	CS_del1 fr [-1264]	8	CGTCTCGG	0	0.0%	
		APGdel2fr [-266]	160	GAGCAAACGCAGAGCTCAC	4	2.5%	Motif I core (O.sat), AB1
		APGdel3 fr [-899]	88	CATACAGTCTTGGGTTTTACC	4	4.5%	D1 box , DRE1, telo box
		APG_del4 fr [-1093]	57	TCTGCATCTTTTTGCTACCCA	2	3.5%	RY2, NICE element 1,
		APG_del5 fr [-1366]	27	CCAATGCTAGGAAACTGAAG	0	0.0%	
		APG_del6 fr [-1491]	25	TACTCAATGCTCTGAAATGAA	2	8.0%	FLS2 EIN3/EIL1 BS(P), TCAATG
		Tm_del7 fr [-211]	388	GTTCGCGCAGGAAATCGATC	7	1.8%	Motif I core (O.sat), AB1
		SO_del8 fr [-1219]	609	ATATGAATAGGGAAACACTT	9	1.5%	CARE1 (O.sat), C-rich R , GA-2,
T9-56	D	ENT_del1 fr [-581]	14	TGGACGATGGACGA	0	0.0%	
T9-59	A	KR_del1 fr [-317]	100	CGGTCCTTACATAACTTATACT	3	3.0%	EM2 (CArG box 2), Fp12/III ,
		KR_del2 fr [-738]	44	GTGACAACAAGAACAAAAA	1	2.3%	p33TCP20 BS2,
		KR_del3 fr [-1237]	18	AAGCATAGTACTAAACAT	1	5.6%	GTAC-motif (O.sat),
		W141_del4 fr [-317]	29	AAAAAAAAAATGAAACTCCA	1	3.4%	p33TCP20 BS2 ,
	B	ASP_del1 fr [-560]	678	GTGTTATCCTGTGTTTACGCT	2	0.3%	C2b-BS (O.sat), ABRE motif A
		W777_del2 fr [-145]	145	GAGTTCCTAGGACCAGAGCA	3	2.1%	TTGATC motif (H.vulg),
T9-60	A	KR_del1 fr [-689]	108	TTTTAGGCTGGTCATAGTGG	1	0.9%	PROL box (O.sat),
		Tm_del2 fr [-694]	482	GGCTGGTCATAGTGGGGAGT	1	0.2%	G-box (O.sat),
		W292_del3 fr [-773]	163	AACATAATAGTAGTATCATAG	3	1.8%	G-box (O.sat), GT-motif (LRE,
		DI_del4 fr [-936]	94	TCCGTCCCGGTGTATAAGTCA	3	3.2%	Box A, EE 1, TGTCACA motif,
		IQ_del5 fr [-867]	126	CACCTCTCATTAATTACTTGC	2	1.6%	G-box (O.sat), GT-motif (LRE)
		MW_del6 fr [-897]	118	AGAATTTTCTCGAAGAGCGC	2	1.7%	Box A, TGTCACA motif,
		SS_del7 fr [-690]	43	TAGGCTGGTCATAGTGGGGA	2	4.7%	RY, CArG (009F)
		SS_del8 fr [-897]	211	TACCTCCGTCCCGGTGTATAA	4	1.9%	Box A, E1-core , 3'/AT-rich J1 ,
	B	ASP_del1 fr [-1165]	650	AAGTAAATTAAAGACACACC	9	1.4%	ABRE-3 , A/T-rich BOX1, ABRE,
	D	ASP_del1 fr [-838]	1,229	AAATTTAACATTGTACTCCTT	3	0.2%	CArG motif (T.aes), LeHB-1 BS
T9-61	A	Tm_del1 fr [-535]	875	CGTATTAAAAGCAAAAAATCA	7	0.8%	GA-1, AE box 1 , AT-rich
		Tm_del2 fr [-1505]	7	ACCACTG		0.0%	
		Tm_del3 fr [-1731]	38	CGTCTGTCGAGAAGGACGTG	2	5.3%	Em1a (T.aes), CCAAT box-like
	B	ASP_del1 fr [-696]	239	TCCTTTGTTTCTAAGTTTTAA	0	0.0%	
		ASP_del2 fr [-1505]	41	GTGCACTAGAAGTTGCGCGG	0	0.0%	
		ASP_del3 fr [-1592]	40	GCAGGTGGCGAGGTGGTAG	0	0.0%	
T9-63	A	Tm_del1 fr [-16]	13	AGCTAGCTGCCTG	1	7.7%	Motif IV
		Tm_del2 fr [-346]	17	TCCTTCAAAAAAAAAAA	0	0.0%	
		M049_del3 fr [-425]	320	CTACCGCCGCCCCGATTCGC	8	2.5%	SEF4, TAACAAA box (H.vulg),
	B	AP_del1 fr [+110]	385	TCAAACACGCACTTTAGCCA	3	0.8%	Region C (Z.mays), GTAC-
T9-64	A	APG_del1 fr [-1075]	709	ACAGTAAAAAAAATCGTGCT	3	0.4%	FLS2 EIN3/EIL1 BS(P), CArG
		GT_del3 fr [+1071]	359	ATGACATTCTCATGGCGCAG	4	1.1%	UN Z1, NIT2 BS II , WUS BS
	B	ASP_del1 fr [-258]	33	ACTAGCAGCATGTGTTTCCA	1	3.0%	CACA box (lots of species)
		ASP_del2 fr [-1482]	48	AGTCAGTGGTTACGCTTAAC	0	0.0%	
		ASP_del3 fr [-2383]	436	ATCCTCCAACTTTGGGCCAC	3	0.7%	TATA box (Z.mays), Fp23/IV ,
T9-66	A	Tm_del1 fr [-162]	215	TACTCCCTCCGTCCGGGTGTA	3	1.4%	E1-core , 3'/AT-rich J1 , EE 1
		W115_del2 fr [-913]	152	GAAATCACATATTTGATCTGA	2	1.3%	Fp4/I , CRE 36-39 (O.sat),
T9-67	A	Tm_del1 fr [-30] (5'UTR)	183	GCAGCAACAACGGTGGGCG	5	2.7%	ACGT box (O.sat), Em2
		Tm_del2 fr [-1127]	41	CATATGATACTACTATATGATA	0	0.0%	
T10-1	B	W141 del1 from [-1065]	23	GGCGGGTGTTGCTCCCACTA	2	8.7%	Box C, E1-core ,
T10-2	A	Tm del1 from [-485]	13	GGCGGATGGCGGA	1	7.7%	CARE C
		Tm del2 from [-1120]	10	GCCCGGATGA	2	20.0%	CARE C
T10-3	A	Tm del1 from [-273]	49	CAGTGGTGCCACAAACCTTC	4	8.2%	ABRE1, STY1 BS (YUC4) ,
		Tm del2 from [-438]	74	AAATTTTTTTGGAGCAATTTA	1	1.4%	AE box 1 ,
		Tm del3 from [-635]	13	AATAATAAAATTT	1	7.7%	AtUSB1,
		Tm del4 from [-1026]	32	TTTTATGATAGCGATGGATAT	3	9.4%	GATA-box, Amylase-element
		Tm del5 from [-1245]	24	ACTGAATTTTTTTAAAAAAA	0	0.0%	
T10-6	A	Tm_del1 from [-219]	311	CTCCCTTCATTTTATATACAA	7	2.3%	RY4 (2x), AtMYB77 BS , TATA
		Tm_del2 from [-916]	55	ATTGGGAGGACCTCGAAGAT	1	1.8%	CCAAT box-like motif 1,
T10-7	D	APG_del1 from [-269]	112	TAGAAGCCGACTGATTGGGT	4	3.6%	CCAAT box 1 , S1, W3-box ,
		APG_del2 from [-811]	112	AATGTTATCTACAATGGGTG	1	0.9%	HB04/HB53 BS,
T10-10	A	APG_del1 from [-1262]	201	CCACGACAACCAACGTTTAT	1	0.5%	P1BS (SmPT5) ,
	D	APG_del1 from [-314]	392	AGGGGGTGTTTGTTTCCAGC	3	0.8%	ABRE Bp , CArG box (BNQ1),
T10-13	A	APG_del1 from [-172]	279	CGATCACTTTAGTTTCTACTC	2	0.7%	ABRE Bp, GATA/2 B,
		M031_del2 from [-58]	21	GTACAGTATTTATTCGGCCCG	0	0.0%	
	B	APG_del1 from [-172]	256	TTTTTGTGATCACTTTAGTTT	5	2.0%	ABRE Bp, 3'/AT-rich J1 , GATA/
	D	APG_del1 from [-157]	291	TTTTTTGCAATCGCTTTAGTT	3	1.0%	ABRE Bp , GATA/2 B , MYCS
T10-16	D	ASP_del1 from [-601]	774	TAAACGGAAAAACTAACGCC	6	0.8%	Motif I core, RY4 (2x), Sph,
T10-18	A	Tm_DEL	475	TTTACCTAATAACTAGACAAT	4	0.8%	EM2 (CArG box 2), MYB46 BS
		W115_del1 from [-718]	23	CTAGTAGTTAGCTTGTGACTG	1	4.3%	GCN4-box
T10-19	A	APG_del1 from [-996]	437	ATTGCGGTGTCATGAGATGT	5	1.1%	5A element W, EBS1, NST2 BS
		MDR043_del2 from [-800]	22	CTATATCTCACGTGTATGCTA	1	4.5%	G-box (O.sat)(Z.mays) , all 85
T10-20	A	APG_del1 from [-522]	34	GGCCTCGCGCGCGAGGAACTT	0	0.0%	
	B	ASP_del1 from [-436]	180	TTATTTGACATCAAAGTACT	3	1.7%	EM2 (CArG box 2) , LeHB-1 BS
T10-23	A	W115_del1 from [-212]	13	AGGGGGTGGCGCA	0	0.0%	
T10-28	A	Tm_del1 from [-197]	20	TTTTTACTGCTGAGCCGGTA	1	5.0%	VRE2,
T10-31	D	APG del1 from [-8]	70	CTGGGAAACAAAATGAGCCT	0	0.0%	
T10-32	B	W_del1 from [-728]	12	GCTTTCCCGAGC	1	8.3%	E2F BS
T10-34	A	ASP_del1 from [-459]	127	GACCTACAGATATCTCTTTTA	4	3.1%	GT-1-like K , GS1b AC box 1 ,
		ASP_del2 from [-660]	46	CCGCTTGACATGGGACACGC	4	8.7%	"ACTTA" motif , U-box , as-1 ,
	D	SS_del1 from [-210]	376	CTATTTTTTAAAATAATACATT	8	2.1%	Box II , DRE/CRT , Fp12/III ,
T10-36	A	Tm_del1 from [-216]	26	TATGAATCAAAGGAGGCCTC	1	3.8%	ATCATT element,
T10-38	B	ASP_del1 from [-657]	139	GTCTTTCCCTTCAAAAAAATA	3	2.2%	CCGTCC motif (Box A), EE
	D	APG_del1 from [-1523]	194	ATTTTGACTGAATTGGTCGG	7	3.6%	WB (WRKY), GA-5 , O2 BS-1
T10-40	B	W246_del1 from [-812]	270	GTACAATGGTTGATAAGGTA	12	4.4%	Motif I core (O.sat), Em1a
T10-41	B	AB_del1 from [-876]	29	GATCTATTTGAACTTTTGTAC	0	0.0%	
T10-44	A	Tm_del1 fr [-347]	301	GCGAACCAACCTCTGATTGA	7	2.3%	AT-rich K , B2 (Z.mays,
T10-46	A	Tm_del1 fr [-149]	348	TAAGGCCTCTTTGGATTGAA	3	0.9%	GT-3a Box III , Box-III* (O.sat),
T10-47	B	ASP_del1 fr [-452]	136	GAGGTGTGCCGTGTGCGTCC	3	2.2%	WA (WRKY1), P-box (s)
			228>150bp		1415		

trait gene	homoeologue	cultivar & location	length (bp)	sequence	BLAST hits (IWGSCrefseq_v1.0, all chromosomes)	identification (trepdb_complete_Rel-19)	% of reference	identification (CLARITE_CLARIrepeatwheat#)
T1-2	B	ASP del1 from [-622]	227	GATTTAGTGATCACACGGAG	8,771	RLG_Taes_Lila_consensus-1	1.3	#66628_74962_Repeat_region_16
T1-3	A	Tm del2 from [-816]	293	GTAGGGTCTGTTTGGTTGGA	769	XXX_Tdur_Iapetus_103H9-1		NONE
T1-4	D	APG del1 from [-1252]	164	TACTCCCTCCGTTCATAAATAT	8,674	DTT_Tspe_Thalos_consensus-1	100	#880149_880358_Repeat_region_247
T1-7	A	Tm del4 from [-704]	285	TATGGTGAGTATCATATAGTA	7,998	DTC_Taes_Jorge_3BctgI-44	5	#266716_273568_Nested_repeat_14 &
T1-8	A	ASPdel1 from [-860]	125	CGGATAGGTAAAAGTTCCCA	1	self		
		ASPdel2 from [-1675]	449	AGTGTCAGCTACTTTGTTCA	3	homoeologues		
T1-22	A	Tm del1 from [-373]	250	AATAAAGTGCTATAATGACCA	6,078	RLG_Tmon_Jeli_AF459088-1	2	#163039_176097_Repeat_region_33
	D	ASP del1 from [-1090]	211	GTATTGAATTCATGCTCCAGT	4,031	DTT_Taes_Hades_BQ243447-1		#281770_281829_Repeat_region_71
T1-23	A	APG del3 from [-1205]	173	CTGGCGGAGCTAGGAAGAA	81	RLC_Taes_Adan_3BctgD-199		none
	D	APG del 1 from [-562]	122	ACAGCTCAAAAATGCCATCG	2,409	DTM_Tmon_Rhea_AY146588-1	21.7	#615098_615648_Repeat_region_132
T1-24	B	ASP del1 from [-1474]	276	AGGGCATGTACAATGGTTGA	5,539	DTH_Tspe_Orpheus_consensus-1	100	#234756_235027_Repeat_region_55
T1-25	D	APG del1 from [-1647]	246	CCCCTCCGTCTCATAATATAA	291	DTT_Taes_Athos_BJ239223-1	[illegible].6, 3'END OF D[illegible]	#765210_765269_Repeat_region_203
T1-28	B	KR del1 from [-1490]	283	ACTAGTATGCCTCTACTTGAC	>8,800	RLC_Taes_Angela_BA1_consensus-3	3.2	#501944_510568_Repeat_region_105
	A	Tm del3 from [-397]	84	TTGGGGATGGACGACGCCG	97	DTC_Taes_Jorge_3BctgD-65		#388197_388627_Repeat_region_135
T2-2	A	M031 del3 from [-1003]	210	GGTAGGGCAAGGAATGGTC	894	DTT_Taes_Hades_BJ252139-1		#655487_655566_Repeat_region_98&99
			80	GGAAAAGAACTCTATGAGA	423	DTM_Hvul_Spring_TREP1674-1	40.8, 3' END	#655487_655566_Repeat_region_98
T2-3	D	W115 del1 from [-1708]	221	TTTGACCGTAGTCGGTCTATA	>8,800	RLG_Tdur_Erika_643D12-1	1.6	#567737_572141_Repeat_region_155
T2-4	A	ASP del1 from [-505]	152	TGCCTGAGGTGGCTCAGATG	23	DTC_Tdur_Clifford_294D11-2		none
T2-6	A	Tm del1 from [-673]	311	AGAATCAGGGCCTCTTTGAT	7,637	DTH_Taes_Xenon_consensus-1		#1004_1306_Repeat_region_1
T2-9	B	W115 del1 from [-714]	177	CAAAAAAAATACTAGAAAAA	4,153	DHH_Hvul_Mothra_NA_1H-8	10	none
		W199 del3 from [-801]	518	GACATCTGAGTAGCTCTCGG	3,731	RLG_Tmon_Latidu_115G1-1		#817414_817449_Repeat_region_195
		W199 del3 [5'only]	186	GACATCTGAGTAGCTCTCGG	3,519	RLG_Tmon_Latidu_115G1-1	3.8	none
		W115 del2 from [-1334]	112	TGCCTAGCTAGTACTCCCTTC	4,128	DTT_Tspe_Pan_consensus-1	80.5	#816988_817114_Repeat_region_194
		W199 del4 from [-1377]	126	CTCCCTTCGTTCCGAATTACT	4,695	DTT_Tspe_Pan_consensus-1	100	#816988_817114_Repeat_region_194
T2-11	A	KR del1 from [-970]	71	ACGGGACCACCAGTCATGCT	360	DTC_Taes_TAT1_RND3B-1		none
		Tm del2 from [-838]	319	CGATGCGTTTGGTTGAAGGT	629	RLC_Tmon_Ale_AY951944-1		#840407_840456_Repeat_region_202
T2-12	A	KR del1 from [-839]	723	GAGGTAATAGCACCAGCAGT	196	XXX_Taes_Tail_AB016969-1		none
T2-13	B	ASP del1 from [-510]	184	AATCATTTCTCTTTATTTATTT	2	BD		
T2-16	A	Tmdel1 from [-23]	363	GCGCGCTGGTTGTTTGCTTT	8,506	DTH_Taes_Mengano_4dss00103-1	100	none
	B	ASP del1 from [-335]	126	CTCCCTCCGTCCGAAAATACT	8,073	DTT_Tdur_Icarus_294D11-3	100	#382751_382885_Nested_repeat_17_Repeat_fr
T2-17	B	ASP del2 from [-1497]	248	CGTTTACTAGCAACTTTATAT	>8,800	RLG_Taes_Derami_AY368673-1	3.7	#333556_354740_Nested_repeat_18(repeat
T2-19	A	APG del 1 from [-454]	639	CAAAACTTCTGATCTATTCAT	>8,800	RSX_Hvul_XC_consensus-1	84.5	#845332_845963_Repeat_region_158
	B	ASP del1 from [-354]	370	ACATTTTTTCGGAAAAACTT	>8,800	RSX_Taes_XC_consensus-1	51.4	
	D	W115 del2 from [-904]	106	AGCCGGGCGAGCCAAAATT	22	DTC_Taes_Jorge_65C22-1		none
T2-20	B	KR del8 from [-1295]	228	TCAGGGATCAACGAGAATTT	3	homoeologues		
T2-21	D	ASP del2 from [-2025]	259	CTACAACATGGGATACTATCT	1,325	DTH_Bdis_BdisC_consensus-1	13.1	#257600_258202_Repeat_region_37
T2-24	A	Tm del1 from [-685]	459	AACTGCTGGGGAAAGGGGA	91	DTT_Taes_Athos_BJ312270-1		none
	B	ASP del1 from [-750]	559	TAAAGGCATCTCCAACACCG	1,538	DTC_Taes_Isaac_1020F19-1		#48507_48656_Repeat_region_13
T2-25	D	APG del1 from [-321]	108	TGATGCTAGCTACGAGCTTCT	3	homoeologues		#48658_49070_Nested_repeat_2_Repeat_frag
T2-26	B	ASP del1 from [-226]	472	CGGGATGACATGCTTAAATT	19	DTT_Taes_Athos_BJ295756-1		none
		APG del2 from [-575]	408	GTTGTTGTGTGCCAGCACGC	41	DTT_Taes_Athos_BJ295756-1		none
		W579 del 3 from [-198]	571	AAAATAGCTCATGACACTACA	42	DTT_Taes_Athos_BE516055-1		none
		W579 del 4 from [-1062]	508	TTTGCCATGCGGTGCTTGTT	150	DTT_Tmon_Athos_109N23-1		none
	D	M031 del3 from [-1518]	438	TCAGTGAATTTCAGTGAAAT	58	DTA_Taes_Talma_Lr34-1	14.6	none
T2-27	A	M031 del3 from [-1050]	110	GTAGCAAGTAGCATTTTTTCT	12	RLC_Taes_Claudia_consensus-1		#939616_939688_Repeat_region_213
T2-30	D	APG del1 from [-457]	106	TCTAATATAGAATCCTCTAACA	3	homoeologues		
T2-31	B	APG del1 from [-1669]	408	TTTTTTAGAAAAGAGGGTTA	>8,800	RLC_Tura_Angela_210J24-2	9.5	#809389_809640_Repeat_region_197 &
		APG del1[5'part]	251	TTTTTTAGAAAAGAGGGTTA	1,485	RSX_Hvul_Xanti_AY853252-1	23.8	
								#809389_809640_Repeat_region_197
T2-33	A	APG del2 from [-1210]	516	AAAAGCACTACACTGGTGCT	1,795	XXX_Taes_Ace_RND-1		#72019_72019_Repeat_region_24
T2-40	A	CS del1 from [-1311]	115	ACTCCCTTCATTTCTAAATATT	4,535	DTT_Taes_Thalos_BJ276470-1		#912131_912179_Repeat_region_242 &
T2-43	B	ASP del1 [-1997]	94	CCCGCAAAAAAAAAAACAC	1			
T2-44	A	M031 del2 from [-660]	328	AGTACTCGCAAGACTTACAT	>8,800	RLG_Tmon_Latidu_115G1-1	6.8	#254447_255811_Repeat_region_75
T2-45	D	APG del2 from [-942]	139	AGTGGGTTGTTTGGTGGTCC	216	RSX_Taes_XC_consensus-1		#690690_690810_Repeat_region_149
T2-47	A	Tm del4 from [-648]	141	AAGCTTTTTAAACCAAGCCAT	1			
	B	APG del1 from [-1017]	135	TGGAGATCCTCCGTTTCAAT	8			
T2-49	A	Tm truncation from [-1130]	925	ATAATCCAGTCGACCGCCTC	>8,800	RLG_Taes_Jeli_1020F19-1	1.3	#243712_257266_Repeat_region_74 &
T2-49	B	ASP del1 from [-249bp]	349	ACTATATCTGGCCATGGGCCA	2,058	RIX_Tmon_Paula_AY146588-1		none
T2-51	A	Tm del2 from [-951]	102	ACTTTTTCCGGGAGCTGTGG	1	self		
T2-51	B	ASP del1 from [-1283]	286	CTCCCTCCGTCCGGGTTTATT	1,240	DTT_Tdur_Athos_103H9-4		#147299_147584_Repeat_region_33
T2-51	D	APG del2 from [-1355]	146	CTTGTTTTTTTTTCGATAAAG	432	RIX_Taes_Karin_1020F19-1		none
T2-51	D	W160 del5 from [-870]	151	AAGAAAGGTACTCACCTTTG	2	AD		
T2-52	B	ASP del1 from [-234]	79	CTCCCTCCGTCTCATAATATAA	8,463	DTT_Taes_Athos_BJ295756-1	96.3	none
	D	APG del1 from [-1196]	107	CTACGTGTGCATGTATGTCTA	1	self		
T2-54	B	KR del1 from [-1269]	278	CCAAAATTGCTTTGAGCGTT	43	DTC_Taes_Isaac_1020F19-1		none
T2-55	A	KR del3 from [-492]	257	ACCTGAACAAACCCCCTAAA	52	RLG_Taes_Fatima_59e04-1		none
	B	W115 del2 from [-315]	272	ATGTCCCTTGGTAAGTTGGT	2	homoeologues		
T2-56	B	ASP del1 from [-719]	161	TACTCCCTCTGTTTCTAAATGT	8,623	DTT_Atau_Thalos_AF338431-1	100	#217383_217577_Repeat_region_62
T2-58	A	Tm del1 from [-413]	208	GGATCCGGTTTGCCTGCTCC	3,637	DTM_Tmon_Remus_AY914084-1	6.4	#472306_472360_Repeat_region_95
		BW del2 from [-1015]	134	AAGCACCATCAGAAAATGTT	113	RLG_Taes_WHAM_10k23-4		none
T2-59	B	W115 del2 from [-1489]	71	CTCCCTCTGTAAAGAAATATA	3,243	DTT_Taes_Athos_BJ312270-1	97.5	#448021_448118_Repeat_region_103
T4-3	B	KR del2 from [-671]	127	TGTGGGAGGCGAATCCTGTA	46	DTH_Taes_Kong_AC216558-1		
T4-4	B	W209 del1 from [-534]	243	TAAGTTCTTCAACCGCCAAT	2	AB		
T4-5	B	ASP trunc from [-536]	302	TCTACTTCTAATGTAGCAGTT	4,697	DTT_Taes_Athos_BJ253899-1		none
T4-6	B	del1 from [-629] in 83var	238	ACTCCCTCCTTCCATCTATATA	8,748	DTC_Atau_Jorge_D_3D-339	0.8	#276156_276397_Repeat_region_58
T4-11	A	Tm del1 from [-350]	114	GGCCCATCCCTAAGTGCGTTA	3	homoeologues		
T4-13	A	Tm del from [-292]	214	ACTACCCCGTCACGGTTTAGA	862	DTT_Hvul_SAB_consensus-1	92.5	#569461_569674_Repeat_region_118
T4-15	A	KR del3 from [-1621]	81	TCCCTCCGTTCCAAAATAGAT	8,325	DTT_Taes_Hades_BQ243447-1		#565051_565136_Repeat_region_130
		Tm del5 from [-173]	132	AGGCACACCACAAGTTGGA	3	homoeologues		
T4-16	A	Tm del from [-1199]	450	ACGCTCACAGCGTGTTTGGT	259	DTH_Taes_Centa_1ASc5692783-1		none
T4-18	A	KR del: [-771]	152	ATTCTGTGTCGGCTACTTCCC	3	homoeologues		
	A	M037 del1 from[-240]	371	CTGTCTTAGGCCCGACTGTA	3	homoeologues		
		Watkins del2 from [-1391]	101	GCACATAAACATGCACTCAAT	3	homoeologues		
T4-22	A	W141 del from [+37]	423	GGTATCGCGGTGCGCGACGC	3	homoeologues		
T4-24	B	KR del from [-1265]	169	CGAAATGGCTTCACACACGA	53	DTC_Taes_Sherlock_42j2-1		
T4-26	D	ENT del from [-569]	143	CTAGTTTTTAGTAGTATTTTTT	8,613	DTT_Taes_Icarus_BE591362-1	98.1	#581493_581629_Repeat_region_106
T4-31	B	ASP del from [-768]	512	AGGCTGGTCACAATGGGCA	8,799	DTH_Tdur_Coeus_643D12-1 & DTC_Atau_Jorge_D_3D-339	100	#736527_736774_Repeat_region_151 & #736775_737012_Repeat_region_152
		KR del from [-1881]	116	ACTGATCTGATCTACGCCTAC	3	homoeologues		
T4-32	D	APG del1 from [-706]	94	ACACCTTCAGCTCACTTCAGT	2,962	RSX_Taes_XC_consensus-1	12.2	#270235_270524_Repeat_region_69
T4-36	A	Tm del1 from [-580]	91	CAAAATAGATGACTCAACTT	1	self		
T4-37	A	KR del from [-135]	217	CGTTGGAACTTACTCCCTCC	>8,800	DTT_Taes_SBB_42j2-2	100	#16538_16619_Repeat_region_6&5
		W203 del 1 from [-149]	79	CCTCCGTAAACTAATATAAAA	8,692	DTT_Taes_Athos_42j2-5		#16418_16497_Repeat_region_5
T4-41	A	Tm del1 from [-482]	189	GCGTAAAGTTTTTTGGCATCT	129	RIX_Tdur_Morpheus_294D11-2		none
T4-43	A	Tm del3 [-742]	119	TGCATGACGGCGACTGATGA	3	homoeologues		
		SC del4 [-1294]	59	CAAGCGTTGAAGGAACTACT	110	RLG_Tura_Romani_210J24-1		
T4-45	B	MK del2 [-1563]	98	TGTGCCAGTAATTCATGGTAA	2	7D & 5B		
T4-46	D	APG del from [-700]	121	AATCCCTCCGTCCCAAAATTC	>8,800	DTT_Taes_Pan_42j2-5	99.2	#702916_703069_Repeat_region_199
		Tm del3 from [-604]	118	GTTTCCTACTACAAATAAACT	2	AD		
		W199 del2 from [-736]	154	ATAACCCCATCCCCTCTCTCC	3	homoeologues		
T4-49	A	KR del1 from [-760]	184	AAACGTGTGGCCNNNNNNN	3	homoeologues		
T4-50	A	W786 del2 from [-1104]	251	CCCTTTAGTTTGAAGTATAAT	52	DTT_Tmon_Athos_AF326781-1		
	D	APG del1 from [-1318]	102	CTCCCTCCGATCCATATTACTT	3,934	DTT_Taes_Rosana_4dss00551-1	97.1	#433855_433961_Repeat_region_84
T4-54	D	SS del1 from [-520]	206	CAAGGGGTGCTTACTTGACT	3	homoeologues		
T4-57	D	APG del1 from[-931]	150	GAGCACTGCTACACGGACGA	30	RLG_Taes_Carmilla_9p13-1		none
T5-2	A	APG del1 from [-1153]	177	CCCTTTTATAGTGATATGAAC	64	A&B seqs highly homologous		
	B	APG del1 from [-2132]	175	CCTTTTATAGTGATCTAAACG	111			
T5-3	D	ASP del1 from [+1620]	256	CTAACCCTAAATGAGTTTTAA	4	HOMOEOLOGUES & 2B		
		ASP del2 from [+318]	668	CAAAATTGACAAACAGACAA	4	HOMOEOLOGUES & 2B		
T5-5	B	ASP del1 from [-1512]	272	ATCACATCCCTAGTCTGGTAT	8,020	RLG_Taes_Laura_9p13-1	2.1	#84839_97869_Repeat_region_15
T5-8	A	M037 del2 from [-703]	160	TGCAGTAGAGTTGCTTACCC	2	AD		
T5-11	A	KR del1 from [-827]	131	CCTTGTTCGTTTAATCCTCCT	487	DTH_Hvul_unnamed_M58753-1	98.4	#282598_282728_Repeat_region_78
T5-14	A	Tm del1 from [-295]	215	AATACTACCCCGTCACGGTTT	836	DTT_Hvul_SAB_consensus-1	92.2	#569461_569674_Repeat_region_118
	D	Tm del1 from [-314]	117	TTGGCAATGCGAGATGATGA	3	homoeologues		#408130_408249_Repeat_region_91
T5-15	A	Tm del2 from [-1009]	980	AAAAGCTTAATCATTTCCTTG	224	DTT_Tmon_Fortuna_AF326781-2		none
T5-15	D	SS del1 from [-697]	303	ATAGGCAAATTTGACAAATT	5,618	XXX_Taes_unnamed_AB077381-1		none
T5-19	A	KR del1 from [-1708]	344	AAGCATCTTAGCGGCGGACT	453	DTC_Taes_Boris_consensus-1		#21632_21697_Repeat_region_13
								#21632_21697_Repeat_region_14
T5-21	A	ASP del2 from [-699]	251	CGCACCCTCAAGACGCCCGC	3	homoeologues		
		ASP del3 from [-1275]	927	ACTATTTTTTGTGTCATGAGC	3	homoeologues		
T5-22	B	KR del1 from [-774]	93	TTTGGTGTCGCGAAGTGGTT	1			
		W160 del2 from [-1688]	230	CTGGGAAAAAAATTATATGA	4,314	DTM_Hvul_Spring_TREP1674-1	100	#606624_606848_Repeat_region_187
T5-24	B	ASP del1 from [-529]	408	AATTACTACCTCCGTCCGGGT	>8,800	DTT_Taes_Polyphemus_464G14-2	100	#117917_118155_Repeat_region_20
	D	ASP del1 from [-83] (inside 5'UTR)	580	TAAAGAAAACTAATAATGCAT	3	homoeologues		
		ASP del2 from [-1109]	282	ATCCCTTATAAAGAGGTCCAA	4,066	RLC_Tspe_Sasanda_EU157184-1	2.3, 5'END	#461840_463967_Nested_repeat_22 &
T5-27	A	Tm del1 from [-1282]	876	CAACATGGCACACGTAGGTT	611	RIX_Taes_unnamed_42j2-9		#36222_54253_Nested_repeat_2
	D	ASP del1 from [-731]	247	ACTACTCTCTCCGTCCGAAAA	5524 (>8,800)	DTT_Hvul_Icarus_consensus-1	100	#604884_605014_Repeat_region_123
		ASP del 3 from [-1446]	495	TGTTTCTAAGCTGCCACCCAT	52		4.6% 5'end	#605729_605997_Repeat_region_124
T5-28	A	KR del1 from [-541]	172	AACTTGGCAATAAAAAGGGA	8,756	DTT_Taes_Icarus_42j2-4	100	#347800_347927_Repeat_region_115
T5-29	A	Tm del1 from [-860]	123	TTTCTTCCGTCCTAAAATTTTT	8,043	DTT_Taes_Pan_42j2-5	98.4	#172693_172835_Repeat_region_22
T5-30	B	APG del3 from [-872]	347	GAAAATTCTAGATTGTAACTC	8,629	RLG_Taes_Derami_A_AY188333-1	5.8	#71853_74695_Repeat_region_14
	D	APG del 1 from [-567]	437	AGAAAATTCCAGATTGTAACT	>8,800	RLG_Atau_Sabrina_B_AF497474-1	6	#138704_140117_Repeat_region_25
T6-1	A	M049_del3 fr [-425]	320	CTACCGCCGCCCCGATTCGC	25	DTM_Tdur_Gerald_103H9-1		
T6-3	A	APG_del1 fr [-1075]	709	ACAGTAAAAAAAATCGTGCT	1,082	XXX_Ogla_XAC_consensus-1	2.1% 5'end	#773102_773706_Repeat_region_131
		GT_del3 fr [+1071]	359	ATGACATTCTCATGGCGCAG	13	only 5ABD, 6ABD, 7ABD		
	B	ASP_del3 fr [-2383]	436	ATCCTCCAACTTTGGGCCAC	3	homoeologues		
T6-5	A	APG del1 from [-854]	147	ATTTTTTTTATTTTTTGCGAG	789	RSX_Taes_XC_C_42j2-1	46	#332097_332229_Repeat_region_94
	B	ASP del2 from [-495]	413	CCTATATATACACTACTGGAG	8,542	RSX_Taes_Iantha_3BctgF-279	100	includes #112259_112535_Repeat_region_32
T6-6	A	Tm_del3 fr [-867]	721	GGGAGTGTCTATTTGACACT	359	XXX_Tdur_Enuc_103H9-1		none
T6-8	A	Tm_del1 fr [-539]	119	TCATGCCATCTACTCCCTCCG	>8,800	DTT_Taes_Icarus_BJ311657-1	100	#203560_203666_Repeat_region_40
		Tm_del2 fr [-845]	265	GCAAATTCTTGGCTCCGCCA	1,237	RLC_Taes_Oref_1020F19-1		#203851_205511_Repeat_region_41
T6-12	B	W115_del1 fr [-904]	393	GGATAAAATCACCTACTACTA	41	RLG_Taes_Egug_10k23-1		
	D	ENT_del1 fr [-1421]	150	CAACAGCGATCCTAGCCTTC	3	homoeologues		
T6-13	A	KR del1 from [-999]	219	TAGTACTACCTCCGTCCCGGT	6,222	DTT_Tspe_Aison_consensus-1	99.5	#346190_346408_Repeat_region_79
T6-17	A	KR_del1 fr [-996]	196	TGAACCCAACCAAGGGATTC	3	homoeologues		
	B	KR_del1 fr [-728]	45	GAACTAAAACCACGACGAGT	316	DTT_Taes_Jason_AF325196-1		
T6-18	A	W115_del4 fr [-1154]	424	GAGTCAATGAGCGTCGTATA	8	6ABD		
T6-31	A	Tm del3 from [-637	101	ACGAGCATAGATGACCATGA	1			
	B	ASP del1 from [-720]	104	ACTCCCTCCGTCCGGAATTAC	5,898	DTT_Taes_Icarus_BE404582-1	100	#375498_375604_Repeat_region_68
T8-1	A	Tm del1 from [-1751]	149	GAAACTCATGGGCACTAGCA	362	XXX_Taes_Xenos_AM050686-1		none
	B	ASP del1 from [-787]	166	CTCCCTCTGTTCCTAAATACTT	>8,800	DTT_Aspe_Thalos_BQ159471-1	100	#766341_766524_Repeat_region_146
T8-2	B	APG del2 from [-1123]	457	TTTTTTCCGATAAACTGGCAG	70	DTT_Taes_Jason_AF325196-1		none
T8-3	A	Tm del3 from [-390]	59	ACTCCCTCTGTCCCAAAATTC	8,681	DTT_Tspe_Pan_consensus-1	47.2	#595511_595579_Repeat_region_141
	B	ASP del1 from [-255]	204	TGTGCTGATCATCAATGTTTT	3	homoeologues		
	D	Tm del4 from [-807]	126	CTCTGCAGCTTTTTGCAGAG	3	homoeologues		
T8-4	B	ASP del1 from [-1327	179	CCGGCAATGCTACACGTACA	449	RLC_Taes_Ale_42j2-4		none
	D	APG del1 from [-497]	132	ATGCCTGGCTGTGAATGTGTA	2	homoeologues		
T8-10	B	APG_del1 from [-720]	519	AATGGATCCCTCGTGTGCCAC	133	DTM_Taes_Remus_AC207950-1		
T8-12	B	KR_del1 from [-653]	210	GACGAGACTTGTCTGCTCCC	2870	DTM_Taes_Remus_AY914085-1		
T8-15	B	APG del1 from [+516]	325	ATACTCCTTCATAAAAGTCAC	413	RLG_Tmon_Sumaya_231A16-2		
T8-19	B	APG_del2 from [-1243	176	CACATACATATGTTGGCTAAT	7,805	DTT_Hvul_Hades_AV835808-1	100%	#150139_150271_Repeat_region_32
T8-21	B	ASP_del1 from [-889]	103	GTTTTTTTTTTTGGCCCGGA	1			
T8-22	A	Tm_del1 from [-1176]	240	CTCCCTCCTTCCATCTATACA	8,756	DTT_Hvul_SI_consensus-1	99.2	#910405_910646_Repeat_region_175
T8-25	A	Tm_del1 from [-300]	129	AAATCCCCAAATTTAAAAAAC	1			
T8-27	B	ASP_del1 from [-681]	89	GGACGTACAGCGCACTTGCT	101	RLG_Taes_Abiba_3BctgI-146		none
T9-3	B	KR_del1 fr [-1026]	302	CTATATCAACCATTTACTTGTT	321	RIX_Taes_unnamed_42j2-4		#831716_834127_Repeat_region_161
T9-4	D	APG_del1 fr [-317]	313	GCATGTACAACGCCGGCGCT	77	DTT_Taes_Thalos_BQ620108-1		
T9-5	B	ASP_del1 fr [-673]	820	AAAAAAGTATAAGGGAAATA	7,100	DTT_Hvul_Thalos_consensus-1	100	#43460_43662_Repeat_region_9&10&11&12
T9-6	D	APG_del1 fr [-505]	114	CTCCCTCCATCCGGAAATACT	5,678	DTT_Taes_Icarus_BJ313173-1	100	#255704_255830_Repeat_region_56
T9-9	A	Tm_del1 fr [-1357]	101	TCCCTCTGTTCATTTTTATAAG	3,311	DTT_Taes_Marta_4dss01717-1	95.3	#382465_382572_Repeat_region_74
	B	ASP_del1 fr [-973]	120	ACAACAGTAATCATTGCCTTT	1			
		APG_del2 fr [-123]	122	AGCTGGAGAGAAGGACAAA	3	homoeologues		
T9-11-1	A	KR del1 from [-594]	85	CTCCCTCTGTAAACTAATATA	>8,800	DTT_Taes_Athos_42j2-11	97.6	#910405_910646_Repeat_region_175
T9-14	A	APG_del1 fr [-424]	148	TTAACTAAAGTGGACATCATA	3	homoeologues		
		Tm_del2 fr [-1095]	503	ATGTGGTTCAGCAACTTGTG	3	homoeologues		
T9-15	B	ASP_del1 fr [-371]	472	CACCTCCTCGCTGCTGGGCT	5,177	XXX_Tdur_Iapetus_103H9-1		#189473_189728_Repeat_region_38
T9-16	A	Tm_del1 fr [-388]	354	GAGGGTGCTTGGATACGTTT	3,778	DTT_Tmon_Eos_AF326781-1		#685883_686230_Repeat_region_167
	B	APG_del1 fr [-534]	166	ATTCGTTAGCACATTGTTAAT	3	homoeologues		
	D	SS_del1 fr [-499]	193	AGGAATGTATCGTCCCTGGT	29	RLC_Tmon_Angela_AY951945-2		
T9-18	D	Tm_del1 fr [-833]	156	TGCTACTATGAGTCATGCATG	3,966	DTH_Taes_Thorne_3BctgH-222	50.9, 5' HALF	#918926_919064_Repeat_region_226
T9-20	D	ENT_del1 fr [-1698]	379	AATAACTAGATCGAGAGTGT	>8,800	RLC_Atau_Angela_AF497474-3	4.40%	#56115_63376_Repeat_region_14
T9-21	D	APG del2 from [-1443]	116	ATATCAAAAAAAGGACGGAT	151	DTC_Tmon_Pavel_AY188332-1		#292797_311630_Nested_repeat_10
T9-22	A	Tm_del1 fr [-812]	128	ACCGCCCGTCTCTTCTCTTCT	148	DTT_Tdur_Icarus_103H9-1		
	B	APG_del1 fr [-377]	612	TCCTATGTACGACATCCATTTA	>8,800	DTT_Taes_Thalos_42j2-9	[illegible] TWD 3'END OF	#45055_45253_Nested_repeat_2_Repeat_frag
T9-23	D	ASP del1 from [-513]	363	TGATCATCGGTAGACAAGTTA	29	DTH_Taes_Xenon_consensus-1		includes #381876_382082_Repeat_region_77
T9-24	A	M031_del1 fr [-834]	169	CCTTGTACTATTTCGTTTTTTC	35	RLG_Taes_Quinta_10k23-1		
		M037_del2 fr [-385]	250	TACCACCCTTACAGGGAACA	32	XXX_Taes_Xajek_RND3B-1		
	D	CG_del1 fr [-522]	1,652	TTTTCTAAAAAAGAAATTAAT	1,529	DTT_Tspe_Minimus_DQ885757-1	[illegible]0, in 5'half of d	#876301_876382_Repeat_region_209& 210
T9-25	B	ASP_del1 fr [-308]	114	CCACTCCCTTCGTCCGAAAAT	7,521	DTT_Taes_Icarus_BJ233353-1	98.2	#4939_5054_Repeat_region_1
T9-26	A	CS_del1 fr [-289]	123	TCCATCCGTATCAAAATTCTT	8,508	DTT_Tspe_Pan_consensus-1	99.2	#325736_325862_Repeat_region_102
	B	ASP_del1 fr [-381]	134	GCGTCTCCTGCAACGCTCTG	3	homoeologues		
		ASP_del2 fr [-552]	216	CTACGCGCGGCGGGCGACC	1,634	DTM_Taes_Murray_42j2-1		#159417_159552_Repeat_region_52
	D	CS_del1 fr [-992]	831	GCATTGGAGTCGAATGAAAT	25	RLG_Taes_WHAM_464G14-1		
T9-27	B	Tm del1 from [-1005]	252	TTAGTTTTATGCATCCCCCCC	2	BD		
	D	APG del3 from [-782]	273	AGGGCATGTACAATGGTGCT	5,646	DTH_Tspe_Orpheus_consensus-1	100	#481426_481696_Repeat_region_123
T9-28	A	APG_del1 fr [-974]	120	CACTAGTAGAAAAATGGTCA	8,465	DTC_Tspe_Conan_consensus-1	1.4% OF 5'END	#291257_311620_Nested_repeat_10_Repeat_Fr
	B	APG_del2 fr [-1262]	647	GGTGGACCAGCCAACATCGT	6,858	DTC_Taes_Clifford_59e04-3	5.8%. 3' end	#640005_640664_Repeat_region_136&
T9-29	A	Tm_del1 fr [-419]	274	ATGTCCTGTTAGACTGTATTTA	2,453	DTH_Taes_Coeus_3BctgB-168		#694084_696277_Repeat_region_174
	D	APG_del1 fr [-336]	179	AAAAACAGTTTGTCTAATTCA	3,782	DTC_Atau_Jorge_D_3D-339	0.8 twd middle	#709126_709313_Repeat_region_172
		APG_del2 fr [-986]	132	TATCGCGGAACATGGTAAAG	3	homoeologues		
T9-31	A	ASP del3 from [-1218]	103	TCCCTCCGTTTCGAATTACTT	8,794	DTT_Taes_Icarus_BJ233311-1	99	#397115_397261_Repeat_region_73
		ASP del4 from [-1516]	462	TCATTTCTATGCATGCAAGGG	3	homoeologues		
		SS del5 from [-489]	565	TCGGGTGCGATCGAGGACTA	3,218	DTT_Taes_Eugenia_4dls04626-1	[illegible]0%, MIDDLE OF [illegible]	#397638_397745_Repeat_region_74
	D	SS del1 from [-665]	81	AAACTTTGTAAACTTTGACA	5,309	DTT_Taes_Acotas_consensus-1	middle, 31%	middle of #686089_686350_Repeat_region_160
T9-32	B	ASP_del1 fr [-265]	435	CCAGACGTATCTGGACGGCC	77	RLG_Taes_Fatima_10k23-3		
	D	APG_del1 fr [-505]	722	GGTTCAACGTCTAACAAAGG	111	RSX_Taes_Iantha_3BctgC-83		#101410_101636_Repeat_region_36&35
T9-33	A	Tm_del2 fr [-371]	814	GAAATAGGCTCCAGTAACAA	6,997	RLC_Hvul_TAR2_AY853252-1	8.2, 3'end of	#80536_87145_Repeat_region_12
	B	ASP_del1 fr [-582]	408	GTACTCCCTCGGTAAACTAA	8,553	DTT_Taes_Athos_42j2-11	[illegible]00%, 5'end of d	#729257_729341_Repeat_region_132&
T9-34	A	Tm_del1 fr [-1605]	273	TTAGGCTATTTCCCTCAACAG	3	homoeologues		
	D	SS_del1 fr [-792]	194	CGCGCACCCGGCCAGCTTTG	3	homoeologues		
T9-35	A	Tm_del1 fr [-364]	152	GGGCGCATGATTGTAAACCT	375	RIX_Tdur_Morpheus_294D11-2		NONE
T9-36	A	Tm_del2 fr [-233]	373	ACAGCCCATAATTAAAGTGT	75	XXX_Tmon_Xenos_AY146588-1		
T9-38	B	ASP_del2 fr [-508]	354	CCGTATCTTGCTTTTTTTTAAT	8,045	RSX_Taes_Iantha_3BctgL-36	100	#321888_322186_Repeat_region_85
	D	APG_del1 fr [-327]	488	ACGCAGCTTCAGTGACTTGA	203	DTT_Taes_Athos_BJ226672-1		
		APG_del2 fr [-1470]	496	ATTTTGCTTTAAATCGTGATAT	14	XXX_Taes_Xajek_RND3B-1		
T9-40	A	APG_del1 fr [-1430]	499	ATTTATGGACGGACGAAGG	12	RLG_Taes_Cereba_consensus-1		#731457_732636_Repeat_region_141
	B	APG_del2 fr [-1726]	496	TTTATTGACGGACGAAGGGT	297	RLG_Taes_Cereba_consensus-1		#323472_324673_Repeat_region_64
T9-41	A	Tm_del1 fr [-1157]	257	CAGAGATGGCAATCTATTTA	3	homoeologues		
T9-42	A	M031_del1 fr [-467]	103	CTCCTTCTGTTCAGAATTACT	8,766	DTT_Tmon_Icarus_109N23-1	100	#69257_69377_Repeat_region_19
		M031_del3 fr [-974]	63	CATACTCCCTTCGTTCGGAAA	249	DTT_Taes_Icarus_BJ257171-1	57.3 5'part	#68806_68859_Repeat_region_18
		Tm_del4 fr [-644]	582	ACAACGTCCCTTCCTCCCCCC	178	DTT_Taes_Icarus_BQ160724-1		#68806_68859_Repeat_region_18
T9-43	A	Tm del1 from [-1183]	75	CTCTGTCCCATAATATAAGAG	2,356	DTT_Taes_Athos_BE427704-1	93.80%	#485389_485472_Repeat_region_118
T9-43		Tm del2 from [-1719]	341	CAGTTGCCATGTGCGCTGCA	3,247	DTM_Taes_Remus_AC200824-1		3'half of #483877_484933_Repeat_region_117
T9-43	D	APG del1 from [-313]	403	ATATACCTGTCCAAACCTCGG	2,635	DTX_Taes_Keres_AF325198-1		none
T9-43		SS del2 from [-678]	161	CTTTCAACCAGATTAAGTTTG	3	homoeologues		
T9-44	A	Tm_del1 fr [-246]	188	GGCATGTTTGGTTCCAAATA	1,374	DTH_Hvul_Islay_AY853252-1	79.9	#613954_614127_Repeat_region_130
	B	ASP_del1 fr [-890]	236	ACTAACTCCGTCCGGGTTTAT	8,780	DTT_Taes_Polyphemus_464G14-2	99.1	#298037_298277_Repeat_region_49
		ASP_del2 fr [-1270]	149	TCCCTCCGTTTCTAAATATAA	>8,800	DTT_Atau_Thalos_AF338431-1	94.80%	#298414_298606_Repeat_region_50
		CP_del3 fr [-207]	143	GCAATAATAATTTACAAAAGT	3	homoeologues		
T9-45	D	ASP_del2 fr [-992]	237	TTCATTCACAATGCTATGTAC	4	homoeologues & 4B		
T9-46		KSA_del5 fr [-52 to -467]	416	GACGGAGGTAGCGTAGCGT	3	homoeologues		
T9-48	A	Tm_del1 fr [-8]	182	AATGGCGCCATCGCAGTTGC	1			
		W246_del4 fr [-1609]	68	CCAGGTACCCTGGCGTCTGG	335	RLG_Taes_Laura_DQ767625-1		contains 5'half of #7942_8013_Repeat_region_3
T9-49	B	ASP_del1 fr [-770]	1,292	TTCTTACGGAAACAACTCAG	445	RLG_Taes_Laura_59e04-1		none
		W387_del2 fr [-766]	248	TACGGAAACAACTCAGATTT	54	RLX_Taes_Veju_consensus-1		
T9-50	A	KR_del1 fr [-470]	69	CTGTAAACTAATATAAGAGCG	8,756	DTT_Hvul_Athos_AJ475374-1	85.20%	#68895_81975_Repeat_region_15&
	B	ASP_del1 fr [-558]	307	AGGCCTTCTTTGGTTCATAG	3,711	DTH_Taes_Montoto_4dss00700-1	100	#867322_867629_Repeat_region_161
	D	APG_del1 fr [-854]	110	CATCTACATTATGGGATGGA	2	DA		
T9-53	A	ASP del1 from [-399]	135	GCTGATTGGATGGAACCATT	3	homoeologues		
		ASP del2 from [-909]	380	GCCCACCGGGACCTCGCTGC	288	DTX_Taes_Keres_AF325198-1		
T9-54	A	APGdel2fr [-266]	160	GAGCAAACGCAGAGCTCAC	3	homoeologues		
T9-54	A	Tm_del7 fr [-211]	388	GTTCGCGCAGGAAATCGATC	426	RIX_Taes_unnamed_42j2-7		none
T9-54	A	SO_del8 fr [-1219]	609	ATATGAATAGGGAAACACTT	3	homoeologues		
T9-59	A	KR_del1 fr [-317]	100	CGGTCCTTACATAACTTATACT	297	RLG_Tmon_Wilma_AY146588-3		none
	B	ASP_del1 fr [-560]	678	GTGTTATCCTGTGTTTACGCT	8,747	RLC_Taes_Maximus_42j2-1	3' end (1.4%)	#493916_498812_Repeat_region_116
		W777_del2 fr [-145]	145	GAGTTCCTAGGACCAGAGCA	3	homoeologues		
T9-60	A	KR_del1 fr [-689]	108	TTTTAGGCTGGTCATAGTGG	7,382	DTH_Taes_Juan2_4dss01013-1	37.9%, 3'END	#662432_662668_Repeat_region_132 & / or 131
		Tm_del2 fr [-694]	482	GGCTGGTCATAGTGGGGAGT	7,681	DTT_Taes_Aison_AC207943-1		#662432_662668_Repeat_region_132 & / or
		W292_del3 fr [-773]	163	AACATAATAGTAGTATCATAG	4,383	DTH_Taes_Juan2_4dss01013-1	63.6	#662432_662668_Repeat_region_132 & / or
		DI_del4 fr [-936]	94	TCCGTCCCGGTGTATAAGTCA	1,195	DTT_Tspe_Aison_consensus-1	43.7%, 5'PART	#662432_662668_Repeat_region_132 & / or
		IQ_del5 fr [-867]	126	CACCTCTCATTAATTACTTGC	1,357	DTT_Taes_Aison_AC200839-1	29.80%	#662432_662668_Repeat_region_132 & / or
		MW_del6 fr [-897]	118	AGAATTTTCTCGAAGAGCGC	1,384	DTT_Taes_Aison_AC200839-1	40	#662432_662668_Repeat_region_132 & / or
		SS_del7 fr [-690]	43	TAGGCTGGTCATAGTGGGGA	966	DTH_Taes_Juan2_4dss01013-1	15.1%, 3'END	#662432_662668_Repeat_region_132 & / or
		SS_del8 fr [-897]	211	TACCTCCGTCCCGGTGTATAA	3,783	DTT_Tspe_Aison_consensus-1	98.6	#662432_662668_Repeat_region_132 & / or
	B	ASP_del1 fr [-1165]	650	AAGTAAATTAAAGACACACC	1,043	RLG_Taes_Sabrina_464G14-3		#222183_222355_Repeat_region_78
	D	ASP_del1 fr [-838]	1,229	AAATTTAACATTGTACTCCTT	8,744	RSX_Taes_Iantha_3BctgL-36	[illegible]1.8, 3'END OF D[illegible]	#807152_807250_Repeat_region_201 & 200,
T9-61	A	Tm_del1 fr [-535]	875	CGTATTAAAAGCAAAAAATCA	74	RSX_Taes_Xanti_42j2-2		none
	B	ASP_del1 fr [-696]	239	TCCTTTGTTTCTAAGTTTTAA	8,388	DTT_Taes_Icarus_BJ263892-1		#669189_669461_Nested_repeat_23,
T9-63	A	M049_del3 fr [-425]	320	CTACCGCCGCCCCGATTCGC	25	DTM_Tdur_Gerald_103H9-1		
	B	AP_del1 fr [+110]	385	TCAAACACGCACTTTAGCCA	30	RLX_Taes_Ronda_42j2-2		
T9-64	A	APG_del1 fr [-1075]	709	ACAGTAAAAAAAATCGTGCT	1,082	XXX_Ogla_XAC_consensus-1	[illegible].1, 5' (3'END DE[illegible]	#773102_773706_Repeat_region_131
		GT_del3 fr [+1071]	359	ATGACATTCTCATGGCGCAG	13	XXX_Taes_unnamed_AF472572-1		
		ASP_del3 fr [-2383]	436	ATCCTCCAACTTTGGGCCAC	3	homoeologues		
T9-66	A	Tm_del1 fr [-162]	215	TACTCCCTCCGTCCGGGTGTA	5,008	DTT_Tspe_Aison_consensus-1	99.1	#856687_856905_Repeat_region_173
T9-66	A	W115_del2 fr [-913]	152	GAAATCACATATTTGATCTGA	1,098	DTT_Taes_Hades_BQ169378-1		none
T9-67	A	Tm_del1 fr [-30] (5'UTR)	183	GCAGCAACAACGGTGGGCG	3	homoeologues		
T10-6	A	Tm_del1 from [-219]	311	CTCCCTTCATTTTTATATACAA	7,318	DTT_Hvul_SD_consensus-1	99.3	[illegible]4_Repeat_region_69
T10-7	D	APG_del1 from [-269]	112	TAGAAGCCGACTGATTGGGT	17	RIX_Taes_Nicole_42j2-11		
		APG_del2 from [-811]	112	AATGTTATCTACAATGGGTGG	14	RLG_Taes_Romani_B_10k23-4		
T10-10	A	APG_del1 from [-1262]	201	CCACGACAACCAACGTTTAT	3	homoeologues		
	D	APG_del1 from [-314]	392	AGGGGGTGTTTGTTTCCAGC	6,961	XXX_Taes_Iago_3BctgI-11		#386541_386926_Repeat_region_112
T10-13	A	APG_del1 from [-172]	279	CGATCACTTTAGTTTCTACTCT	3	homoeologues		
	B	APG_del1 from [-172]	256	TTTTTGTGATCACTTTAGTTT	6	3HOMS, 7AD		
	D	APG_del1 from [-157]	291	TTTTTTGCAATCGCTTTAGTT	6	3HOMS, 7AD		
T10-16	D	ASP_del1 from [-601]	774	TAAACGGAAAAACTAACGCC	8,680	DTM_Tspe_Remus_consensus-1	93.70%	#86690_87442_Repeat_region_33
T10-18	A	Tm_del	475	TTTACCTAATAACTAGACAAT	8,699	DTT_Taes_Athos_42j2-11	[illegible]00%, MID OF DE[illegible]	#608615_608716_Repeat_region_140 &
T10-19	A	APG_del1 from [-996]	437	ATTGCGGTGTCATGAGATGT	3,215	RIX_Taes_Mara_AB012296-1		none
T10-20	B	ASP_del1 from [-436]	180	TTATTTTGACATCAAAGTACT	>8,800	DTT_Taes_Thalos_42j2-9	98.20%	#561300_561465_Repeat_region_125
T10-34	A	ASP_del1 from [-459]	127	GACCTACAGATATCTCTTTTA	1			
	D	SS_del1 from [-210]	376	CTATTTTTTAAAATAATACATT	2,779	XXX_Tdur_Enuc_103H9-1		#749521_749745_Repeat_region_165
T10-38	B	ASP_del1 from [-657]	139	GTCTTTCCCTTCAAAAAAATA	3	homoeologues		
	D	APG_del1 from [-1523]	194	ATTTGACTGAATTGGTCGGA	70	RLG_Tmon_Sabrina_AY146588-1 2		
T10-40	B	W246_del1 from [-812]	270	GTACAATGGTTGATAAGGTAC	5,618	DTH_Tspe_Orpheus_consensus-1	97.80%	#323472_324673_Repeat_region_64
T10-44	A	Tm_del1 fr [-347]	301	GCGAACCAACCTCTGATTGA	>8,800	DTC_Tmon_Enac_AY146588-2	MID 2.6%	#785331_785646_Repeat_region_140
T10-46	A	Tm_del1 fr [-149]	348	TAAGGCCTCTTTGGATTGAA	160	DTH_Taes_Xenon_464G14-1		
T10-47	B	ASP_del1 fr [-452]	136	GAGGTGTGCCGTGTGCGTCC	106	RLG_Taes_Abbie_B_3BctgB-42		none
